# Prediction of transcription factors associated with DNA demethylation during human cellular development

**DOI:** 10.1007/s10577-022-09685-6

**Published:** 2022-02-10

**Authors:** Yurina Miyajima, Shuhei Noguchi, Yuki Tanaka, Jing-Ru Li, Hajime Nishimura, Mami Kishima, Joanne Lim, Erina Furuhata, Takahiro Suzuki, Takeya Kasukawa, Harukazu Suzuki

**Affiliations:** 1 RIKEN Center for Integrative Medical Sciences, Yokohama, Kanagawa 230-0045 Japan; 2 Graduate School of Medical Life Science, Yokohama City University, Yokohama, Kanagawa 230-0045 Japan

**Keywords:** DNA demethylation, Transcription factor, IHEC, Human cellular development

## Abstract

DNA methylation of CpG dinucleotides is an important epigenetic modification involved in the regulation of mammalian gene expression, with each type of cell developing a specific methylation profile during its differentiation. Recently, it has been shown that a small subgroup of transcription factors (TFs) might promote DNA demethylation at their binding sites. We developed a bioinformatics pipeline to predict from genome-wide DNA methylation data TFs that promote DNA demethylation at their binding site. We applied the pipeline to International Human Epigenome Consortium methylome data and selected 393 candidate transcription factor binding motifs and associated 383 TFs that are likely associated with DNA demethylation. Validation of a subset of the candidate TFs using an in vitro assay suggested that 28 of 49 TFs from various TF families had DNA-demethylation-promoting activity; TF families, such as bHLH and ETS, contained both TFs with and without the activity. The identified TFs showed large demethylated/methylated CpG ratios and their demethylated CpGs showed significant bias toward hypermethylation in original cells. Furthermore, the identified TFs promoted demethylation of distinct sets of CpGs, with slight overlap of the targeted CpGs among TF family members, which was consistent with the results of a gene ontology (GO) term analysis of the identified TFs. Gene expression analysis of the identified TFs revealed that multiple TFs from various families are specifically expressed in human cells and tissues. Together, our results suggest that a large number of TFs from various TF families are associated with cell-type-specific DNA demethylation during human cellular development.

**Supplementary Information:**

The online version contains supplementary material available at 10.1007/s10577-022-09685-6.

## Introduction

DNA methylation is a stable epigenetic modification that has been widely studied in mammals. DNA methylation of CpG dinucleotides within the regulatory regions of genes (i.e., in enhancer and promoter regions) tends to be anti-correlated with downstream gene expression and is an important part of many biological processes such as genomic imprinting, X-chromosome inactivation, and transposon silencing (Smith and Meissner 2013). Aberrant DNA methylation is associated with the development of diseases such as cancers (Greenberg and Bourc’his 2019), suggesting that the dynamics of DNA methylation are strictly regulated to ensure correct gene expression.

In mammalian cells, the DNA methylation profile changes as the cells differentiate along their lineage (Zeng and Chen 2019). Initially, cells have an embryonic methylation pattern that is established by a process called de novo methylation after implantation of the embryo in the uterine wall. In this pattern, most CpG dinucleotides, except for the CpG islands in the regulatory regions of house-keeping genes, are methylated. Then, as the cells differentiate, they develop, mostly through demethylation of distinct genomic regions, a methylation pattern that is characteristic of their cell type.

De novo DNA methylation is controlled by two enzymes: DNA methyltransferase 3a and 3b (DNMT3a and DNMT3b) (Okano et al. 1998; Xie et al. 1999). In addition, methylated CpG cytosines are passed on to daughter cells during cell division via the maintenance DNA methyltransferase DNMT1 (Hermann et al. 2004; Yen et al. 1992). Active DNA demethylation involves several steps, including a series of oxidizations by ten-eleven translocation enzymes (TETs) followed by base excision repair and cell division (Ito et al. 2010, 2011; Kohli and Zhang 2013; Maiti and Drohat 2011; Tahiliani et al. 2009).

Although the enzymatic mechanisms of DNA methylation and demethylation are mostly understood, little is known about how cell-type-specific DNA methylation profiles develop. Recently, a subgroup of transcription factors (TFs) has been shown to bind to permissive heterochromatin regions and promotes histone modification and DNA demethylation at their binding sites so that the chromatin becomes accessible to other TFs and transcriptional regulators (Costa et al. 2013; de la Rica et al. 2013; Fujiki et al. 2013; Guilhamon et al. 2013; Mayran and Drouin 2018). We have demonstrated that the TF RUNT-related transcription factor 1 (RUNX1) promotes DNA demethylation by recruiting DNA demethylation machinery (e.g., TET2) to its binding sites, and that this activity likely contributes to hematopoiesis during embryonic development (Suzuki et al. 2017b). We have also developed an in vitro method for examining the DNA-demethylation-promoting activity of TFs, and by using this method, we have shown that several key TFs, involved in cellular differentiation processes, might possess DNA-demethylation-promoting activity (Suzuki et al. 2017a). Nonetheless, a systematic analysis of the TFs associated with the formation of cell-type-specific DNA methylation profiles is yet to be reported.

DNA methylation information is accumulated during cell differentiation. This means that a cell’s DNA methylation pattern can be considered a biological record of its developmental pathway. We have demonstrated enrichment of the RUNX1 binding site at DNA demethylation loci in hematopoietic stem cells compared with that in induced pluripotent stem cells and in terminally differentiated hematopoietic cells compared with that in hematopoietic stem cells (Suzuki et al. 2017b). This suggests that by exploring the TF binding motifs (TFBMs) at differentially methylated loci, we may understand which TFs contribute to the formation of cell-type-specific DNA methylation profiles.

Here, we predicted TFs associated with the formation of cell-type-specific DNA methylation profiles by using a newly developed bioinformatics pipeline. Furthermore, we examined the DNA-demethylation-promoting activities of a subset of the predicted TFs. Our results suggest that a large number of TFs from various TF families are associated with cell-type-specific DNA demethylation during human cellular development.

## Materials and methods

### Development of the bioinformatics pipeline

Whole-genome bisulfite sequencing (WGBS) data was downloaded from the website of the International Human Epigenome Consortium (IHEC; http://ihec-epigenomes.org/welcome/), and any hg38 data were liftOvered to hg19 data. The names of the datasets used and their corresponding IHEC data portal ID are presented in Supplementary Table S1. The WGBS data was computationally tiled into 200-base bins, because we have found that, on average, TFs promote DNA demethylation within the 194 bases on either side of their binding site (Suzuki et al. 2017a). For each bin whose sequence coverage was more than 100 tags per billion, the average methylation percentage was calculated by using the AverageOverBed tool (http://hgdownload.cse.ucsc.edu/admin/exe/). To further reduce individual differences, the average methylation percentages themselves were then averaged for each dataset. Next, the MethylKit tool was used to extract differentially methylated bins; pairs of bins with a > 50-point difference in methylation percentage and a q-value of < 0.0001 were extracted as differentially methylated bins. Then, the CentriMo tool was used to examine the TFBM enrichment in differentially methylated bins; target bins ± 5 bins were examined and the log-adjusted *p*-value and concentration of each TFBM were obtained. The IMAGE position weight matrix database was used to identify TFBMs (Madsen et al. 2018). Aberrant disruption of the site probability curve at the boundary of target bins and immediate neighbor bins was sometimes observed for TFBMs with CpG sequences in their consensus sequences. To determine the existence of such disruption, the ratio of the average site probability rate at the boundary (i.e., [− 100 to − 80]/[− 120 to − 100] from the center of the target bin) was calculated as the C-value, where a value greater than 1.2 was considered to empirically correlate with site probability curve disruption. RNA-seq data for averaged TF gene expression were also obtained from the IHEC website and used in this study.

### Selection of TFs associated with DNA demethylation

Four criteria were used to select TFs most likely associated with DNA demethylation during cellular differentiation. TFs fulfilling all four of the following criteria were selected: (1) CentriMo concentration > 0.12 for any cell or tissue type; (2) CentriMo log-adjusted *p*-value < e − 500 for any cell or tissue type; (3) average C-value < 1.2; and (4) average mRNA expression value was higher than that in embryonic stem cells.

### *In vitro* assay of DNA-demethylation-promoting activity

The DNA-demethylation-promoting activities of the TFs were examined by using methods we reported previously (Suzuki et al. 2017a) with slight modification. Briefly, 293 T cells (RIKEN Bio Resource Center, Tsukuba, Japan) were cultured in Dulbecco’s modified Eagle’s medium (Wako Pure Chemical Industries, Ltd, Osaka, Japan) supplemented with 10% fetal bovine serum and penicillin/streptomycin (100 U/mL, 100 µg/mL; Thermo Fisher Scientific Inc., Waltham, MA, USA). Open reading frame of target TF genes was sub-cloned into the CSII-EF-RfA-IRES2-puro vector (Suzuki et al. 2017b) by the Gateway LR recombination technique. Lentivirus vector was produced as previously described (Suzuki et al. 2012). Then, the 293 T cells were infected with the vectors at a multiplicity of infection of 1, followed by puromycin selection at 2 µg/mL for 1 week. The mRNA expression of the target TFs was confirmed by quantitative real-time polymerase chain reaction analysis. Most of the target TF genes were lowly or marginally expressed in 293 T cells. Lentivirus-infected 293 T cells showed on average 15.5-fold upregulation of target TF gene expression. Genomic DNA was isolated from the 293 T cells by using a NucleoSpin Tissue Kit (Macherey–Nagel GmbH & Co., Düren, Germany), treated with bisulfite by using an EZ DNA Methylation-Gold Kit (Zymo Research Corp., Irvine, CA, USA), and then profiled by using an Infinium MethylationEPIC Kit (Illumina Inc., San Diego, CA, USA). Data normalization and calculation of M-values, a statistical metric of log-scale methylation level, were computed using the lumi package in the Bioconductor software (Du et al. 2010). An M-value difference of ≥ 2 was considered to indicate differentially methylated CpGs. TFBM overrepresentation analysis was performed as previously described (Suzuki et al. 2017b). Briefly, sequences located ± 5 kb from the methylated or demethylated probe positions, and the same number of randomly selected probes, were extracted from the reference human genome sequence. TFBM identification was performed using the matchPWM command of the Biostrings package in the Bioconductor software and the IMAGE position weight matrix database.

## Results

### Prediction of the TFs associated with the formation of cell-type-specific DNA methylation profiles

To predict the TFs associated with the formation of cell-type-specific DNA methylation profiles in human cells, we developed a bioinformatics pipeline for the analysis of genome-wide DNA methylation data. The pipeline was designed based on the hypothesis that when a certain TF directly promotes a change in DNA methylation status at its binding site as a cell differentiates along its lineage, its binding motif might be enriched at regions of differential methylation between developmental stages.

**Fig. 1:**
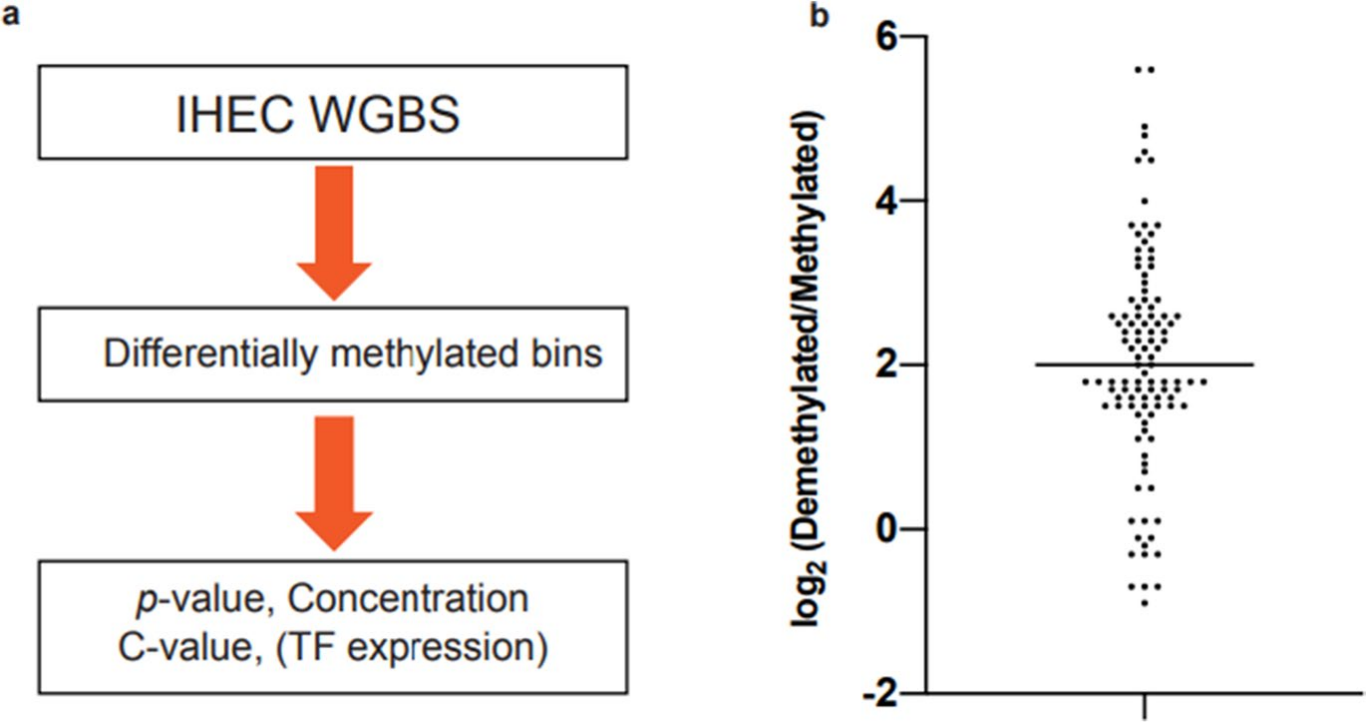
Prediction of TFs associated with DNA demethylation. **a** Overview of the bioinformatics pipeline used to predict the transcription factor binding motifs associated with the formation of cell-type-specific DNA methylation profiles. **b** Distribution of DNA-demethylated/methylated ratio. Each dot represents the ratio of demethylated to methylated bins in each IHEC dataset. The horizontal bar indicates the average ratio

The pipeline comprised of three stages: collection of genome-wide DNA methylation data, identification of differentially methylated regions, and analysis of TFBM enrichment (Fig. 1a). We obtained WGBS methylome data covering various human cells and tissues (Bujold et al. 2016) from the IHEC database. We computationally tiled the WGBS data into bins, calculated the methylation rate for each bin fulfilling our sequencing depth criteria, and used the MethylKit tool (Akalin et al. 2012) to extract bins differentially methylated compared with a reference methylation profile, which in this case was the DNA methylation profile of an embryonic stem cell because these cells possess an embryonic methylation pattern from which the cell-type-specific methylation pattern of all other cells develops.

For the enrichment analysis, we used the IMAGE TF binding position weight matrix database (Madsen et al. 2018). Using differentially methylated bins (target bins) ± 5 bins, a site probability curve was drawn for each TFBM by using the CentriMo tool (Bailey and Machanick 2012); the curve was then used to calculate local enrichment indices for each TFBM (i.e., log-adjusted *p*-value and concentration score). Disruption of the site probability curve at the boundary of target bins and immediate neighbor bins was sometimes observed for TF motif matrices with CpG sequences in their motif consensus sequences. To determine the existence of such disruption, we calculated the ratio of the average site probability rate at the boundary, which we called the C-value.

Supplementary Table S2 shows the number of bins containing methylated or demethylated DNA for each differentiated cell or tissue type compared with the reference methylation profile. The number of bins containing differentially methylated DNA varied depending on the differentiation distance: induced pluripotent stem cells; endoderm-, ectoderm-, and mesoderm-cultured cells; neuronal progenitor cells; and early neurons had relatively few bins containing differentially methylated DNA, whereas more differentiated cells and tissues had many more bins. The average number of bins containing demethylated DNA for a single cell or tissue type was 107,604 bins, which corresponded to 0.7% of the entire genomic DNA. In contrast, the average number of bins containing methylated DNA was only 19,494 (0.13%). Demethylation accounted for approximately 80% of the change in DNA methylation status (Fig. 1b), which is consistent with a previous report that the majority of the change in DNA methylation status during cellular differentiation is demethylation (Suzuki and Bird 2008). This result suggests that DNA demethylation plays an important role in the development of the DNA methylation profile during cellular differentiation. Therefore, hereafter, we focused only on DNA demethylation.

The identified regions of demethylated DNA were subjected to CentriMo analysis. For each TFBM, we determined local enrichment indices (log-adjusted *p*-value and concentration score), together with C-values. We found that many TFBMs had no local enrichment *p*-value, suggesting that the TFs containing these motifs have no association with DNA demethylation. Therefore, to select the TFs most likely associated with DNA demethylation, we selected only the TFBMs and associated TFs (TFBM-TFs) that fulfilled four criteria (see “Selection of TFs associated with DNA demethylation” in “Materials and methods”). As a result, 427 TFBM-TFs were obtained from among 393 TFBMs and 383 TFs (Supplementary Table S3). Cluster analysis of the log-adjusted *p*-values of local enrichment showed clear clustering both by lineage and TF family motif (Supplementary Fig. 1).

### In vitro analysis of DNA-demethylation-promoting activity

Enrichment of TF motifs at DNA-demethylated regions is not a direct evidence that the corresponding TFs are associated with DNA demethylation. Furthermore, TFs within a single family generally possess the same or similar binding motifs, which means that the identified TFs may not all promote DNA demethylations. Rather, their motif enrichment in demethylated regions of DNA may reflect DNA demethylation promoted by other members of their TF family. We therefore used our previously reported in vitro analysis method (Suzuki et al. 2017a) to examine the DNA-demethylation-promoting activities of the predicted TFs. The analysis system comprised of ectopic overexpression of the target TFs in human embryonic kidney cells (HEK293T cells) followed by single-base-resolution methylation array analysis and binding motif overrepresentation analysis of differentially methylated regions between target-TF-overexpressing cells and mock-infected cells. Significant enrichment of the binding motifs for target TFs in the neighborhood of demethylated CpG probes was used for judgment of DNA-demethylation-promoting activity.

**Fig. 2:**
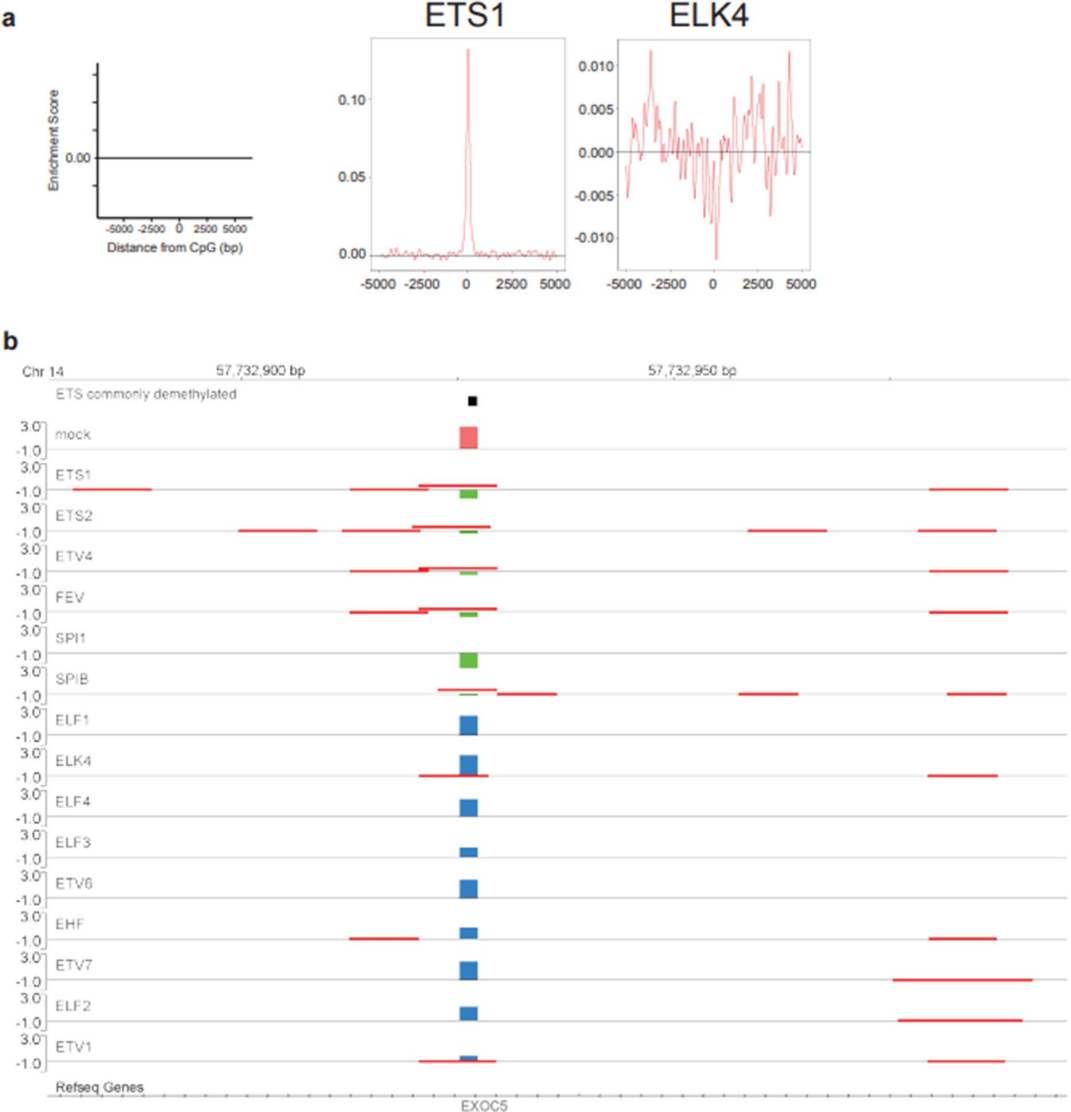
In vitro analysis of DNA-demethylation-promoting activity. **a** Examples of the binding motif overrepresentation analysis for TFs with (ETS1) and without (ELK4) DNA-demethylation-promoting activity. Distribution of TFBM enrichment score for examined TFs were drawn within ± 5000 bp of demethylated CpG probes. *X*- and *Y*-axes represent distance from probe CpG position and enrichment score, respectively. Horizontal lines are enrichment score = 0. Results of the binding motif overrepresentation analysis for all TFs with DNA-demethylation-promoting activity were shown in Supplementary Fig. 2. **b** A genomic browser screenshot showing methylation value (M-value) change of a CpG probes for ETS family TFs. This CpG probe is located on chromosome 14 as indicated. A black column represents position of the CpG probe. M-value of this CpG probe for mock, and ETS family TFs with and without DNA-demethylation-promoting activity, was represented as magenta, light green, and light blue columns, respectively. Positions of the binding motifs for each TF were shown by red lines

From 383 TFs, we examined 49 TFs from various TF families and found that 28 TFs showed significant DNA-demethylation-promoting activity (Fig. 2a, Supplementary Fig. 2, and Supplementary Table S4). Figure 2a shows examples of the binding motif overrepresentation analysis for TFs with (ETS1) and without (ELK4) the activity; ETS1 showed significant enrichment peak of the binding motif in the neighborhood of demethylated CpG probes, while ELK4 did not show the peak. We also showed an example of methylation value (M-value) change of a CpG probe for ETS family TFs (Fig. 2b); ETS family TFs with DNA-demethylation-promoting activity showed drastic decrease in M-value (demethylation), while most of TFs without the activity did not show the change.

The TFs that showed DNA-demethylation-promoting activity came from various TF families, indicating that it is not only certain TF families with this activity (Table 1). Among the TF families containing more than two of the TFs examined, the bHLH, ETS, Fork_head, and TF_bZIP families contained both TFs with DNA-demethylation-promoting activity and TFs without DNA-demethylation-promoting activity. The CTF/NF1 and zf-GATA families contained only TFs with DNA-demethylation-promoting activity, while the Homeobox family contained only TFs without DNA-demethylation-promoting activity.

**Table 1:** Summary of the results of an in vitro assay of DNA-demethylation-promoting activity of the predicted transcription factors

TF family	No. of TFs tested	Demethylation promoting activity	Activity not detected
bHLH	8	ASCL2, MYF6, MSC, MYOG, NEUROD1, TCF21	PTF1A, TFAP4
COE	1		EBF1
CTF/NFI	2	NFIB, NFIX	
ETS	15	ETS1, ETS2, ETV4, FEV, SPI1, SPIB	EHF, ELF1, ELF2, ELF3, ELF4, ELK4, ETV1, ETV6, ETV7
Fork_head	3	FOXA1, FOXA2	FOXP1
HMG	1	LEF1	
Homeobox	3		CDX2, HOXA9, HOXC9,
IRF	1		IRF8
NGFIB-like	1	NR4A1	
PAX	1	PAX8	
RUNT	1	RUNX2	
RXR-like	1		NR2F6
SF-like	1	NR5A1	
TF_bZIP	6	CEBPE, FOSB, FOSL2, NRL	NFE2, NFE2L2
THR-like	1	NR1H4	
zf-C2H2	1		IKZF3
zf-GATA	2	GATA3, GATA6	
Total number	49	28	21

**Fig. 3:**
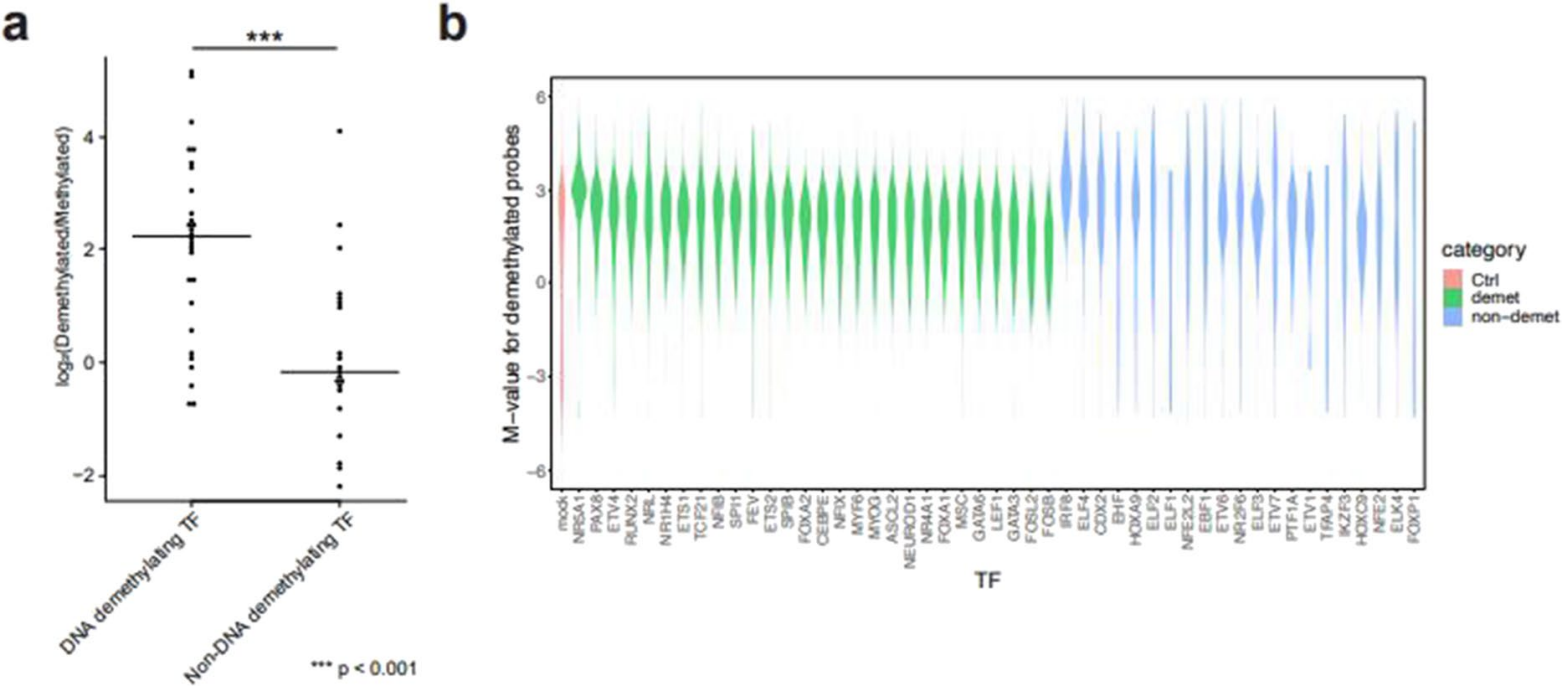
Analysis of methylation array probe. **a** Demethylated/methylated probe ratio distribution was plotted for transcription factors with or without DNA-demethylation-promoting activity. **b** Original DNA methylation level of demethylated probes for transcription factors with or without DNA-demethylation-promoting activity. Violin plots represent kernel density distribution of original M-values for demethylated probes. M-value distribution of all probes, and of demethylated probes for TFs with or without DNA-demethylation-promoting activity, is shown as magenta, light green, and light blue, respectively

TFs with DNA-demethylation-promoting activity tended to have a larger demethylated/methylated array probe ratio than TFs without such activity (Fig. 3a). Furthermore, the methylation levels of demethylated CpGs among the TFs with DNA-demethylation-promoting activity showed significant bias toward hypermethylation, whereas those among TFs without such activity tended to have a high level of variance (Fig. 3b). We also compared demethylated genomic regions with randomly selected genomic regions for target TFs with or without DNA-demethylation-promoting activity. We found a number of binding motifs tended to be higher at the demethylated genomic regions for TFs with DNA-demethylation-promoting activity (Supplementary Fig. 3). On the other hand, this difference was not observed for TFs without DNA-demethylation-promoting activity (Supplementary Fig. 4), suggesting that multiple binding motifs may facilitate DNA demethylation for TFs with the activity.

### Analysis of DNA demethylation regions promoted by identified TFs

**Fig. 4:**
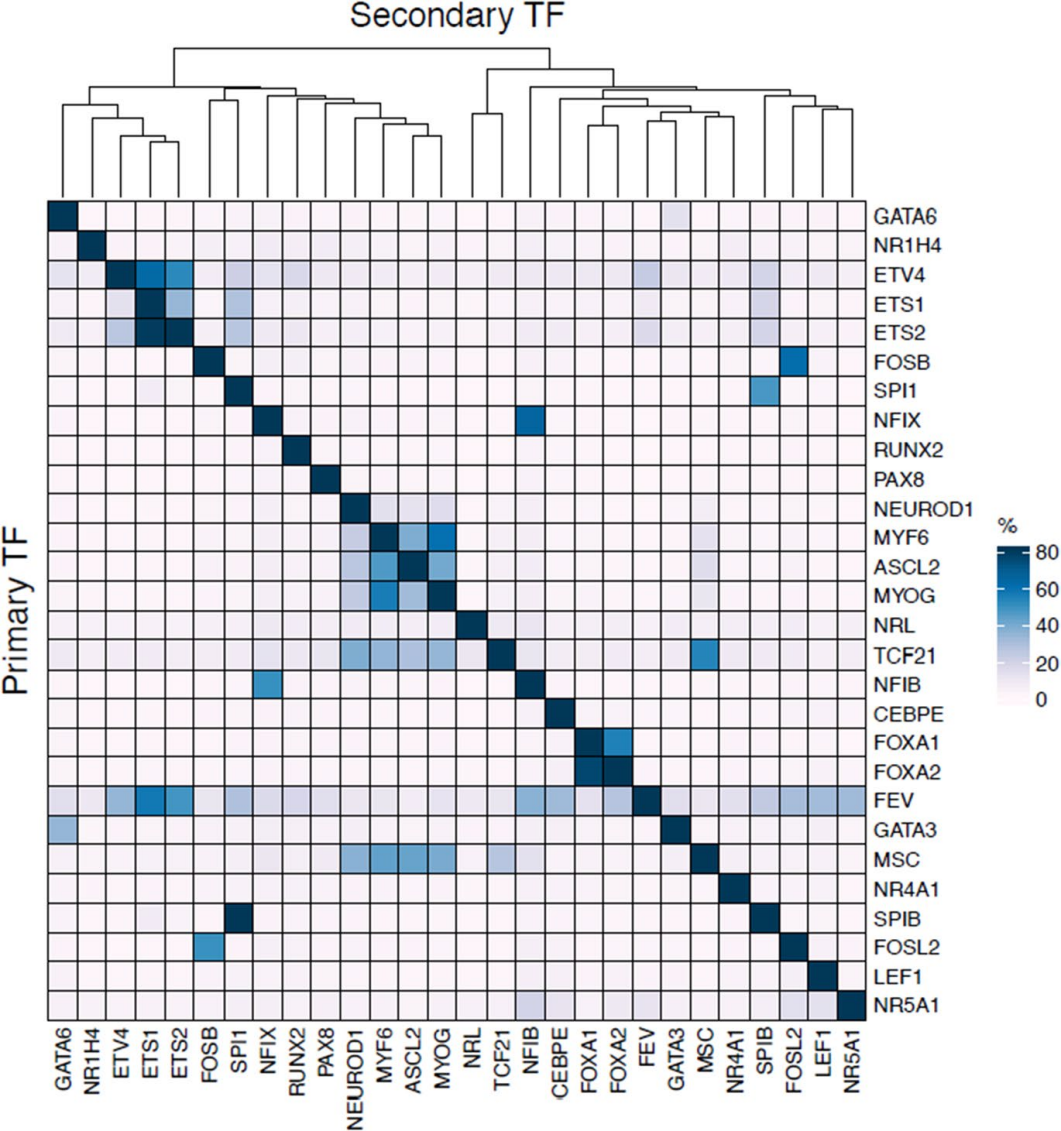
Overlap of demethylated probes among TFs with DNA-demethylation-promoting activity. Overlap percentage of demethylated probes for primary TFs (vertical) to those for secondary TFs (horizontal) is color-coded

We next examined the DNA-demethylated regions targeted by the TFs with DNA-demethylation-promoting activity. The number of demethylated DNA probes varied greatly (range 138–5930), even within a single TF family (e.g., 255–3166, 138–4376, and 313–2815 for bHLH, ETS, and TF_bZIP, respectively). Overlap analysis of the DNA-demethylated regions showed that each TF promotes demethylation of a distinct set of DNA regions, although TF families tended to have a higher percentage of shared demethylation targets (Fig. 4, Supplementary Table S5).

**Fig. 5:**
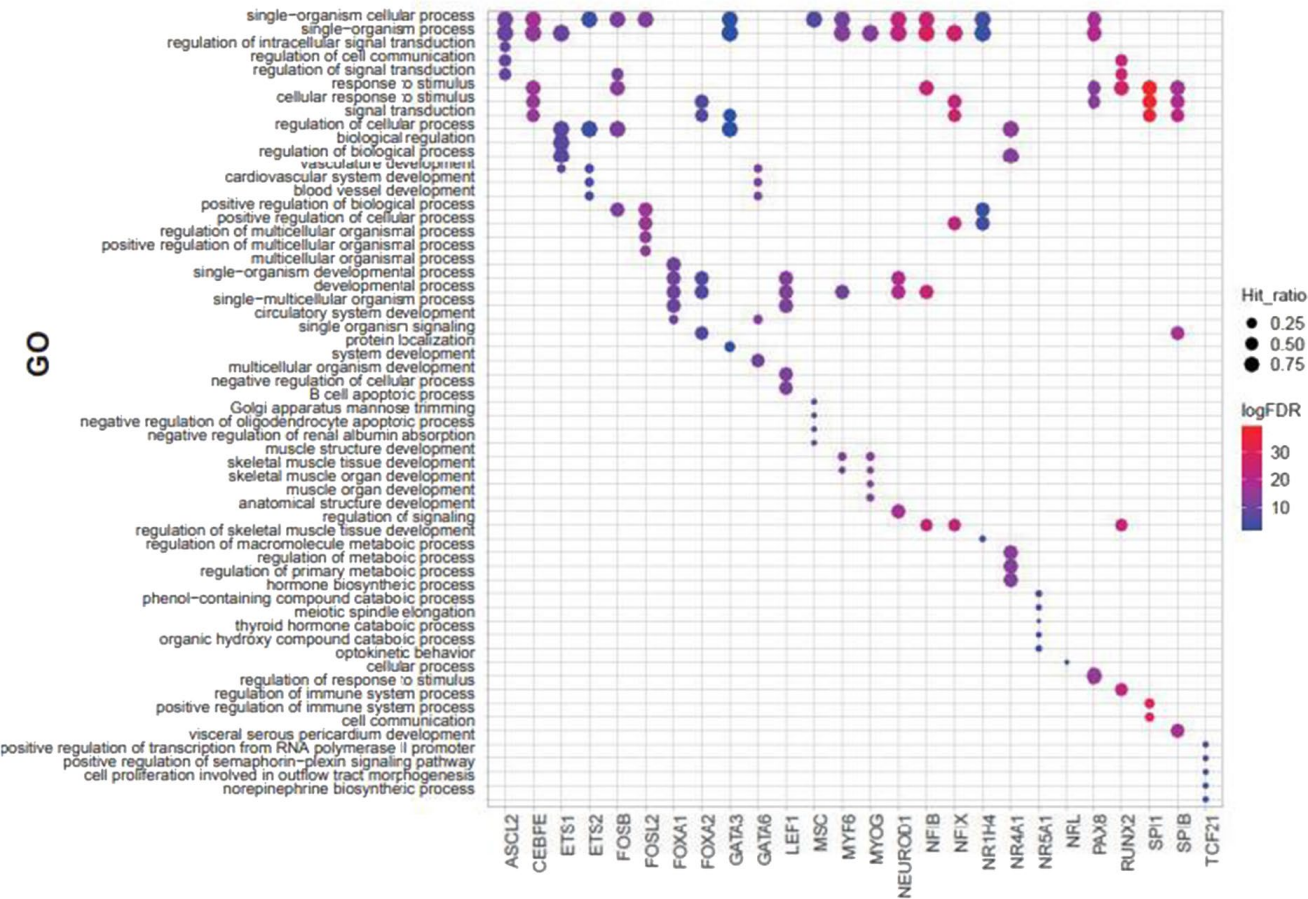
Gene Ontology (GO) analysis of demethylated probes for TFs with DNA-demethylation-promoting activity. Top 5 enriched GOs at regions demethylated by each TF overexpression computed by GREAT. The dot size represents the ratio between the number of probes that hit the GOs and that of all demethylated probes. The color represents False Discovery Rate (FDR, Benjamini–Hochberg method) of gene-based hypergeometric test. The official GO terms for “positive regulation of transcription from RNA polymerase II promoter” and “positive regulation of semaphorin-plexin signaling pathway” are “positive regulation of transcription from RNA polymerase II promoter involved in norepinephrine biosynthetic process” and “positive regulation of semaphorin-plexin signaling pathway involved in outflow tract morphogenesis,” respectively

To clarify the DNA regions targeted by the identified TFs with DNA-demethylation-promoting activity, we analyzed the category of demethylated genomic regions (Supplementary Fig. 5). FOSB, FOSL2, ETS2, SPIB, and SPI1 showed relatively higher proportion of DNA demethylation at enhancer regions, while FOXA1, FOXA2, and NR5A1 showed relatively lower proportion at enhancer regions. We next performed a gene ontology (GO) analysis of genes with demethylated CpGs in their promoter regions (Fig. 5). The enriched GOs tended to be associated with the cellular functions of the corresponding TFs. The GO terms “vasculature development,” “cardiovascular system development,” and “blood vessel development” were enriched in ETS2 and GATA6, which is consistent with previous reports that ETS2 is essential for coronary and myocardial development (Lie-Venema et al. 2003) and GATA6 is essential for cardiovascular development (Xin et al. 2006). In addition, GO terms related to muscle development, such as “muscle structure development” and “skeletal muscle tissue development,” were enriched in MYF6 and MYOG, which are well-known TFs for muscle development (Hernandez-Hernandez et al. 2017). Furthermore, the GO terms “regulation of immune system process” and “positive regulation of immune system process” were enriched in SPI1, a master regulator TF in myeloid cell development. On the other hand, other TFs did not show the GO terms associated with function of specific cells or tissues. The GO terms enriched for individual TFs were not necessarily enriched for all members of the corresponding TF family, confirming that the findings of the enrichment analysis were the result of the specific demethylation targets of the individual TFs.

### Expression profile of TFs with DNA-demethylation-promoting activity

**Fig. 6:**
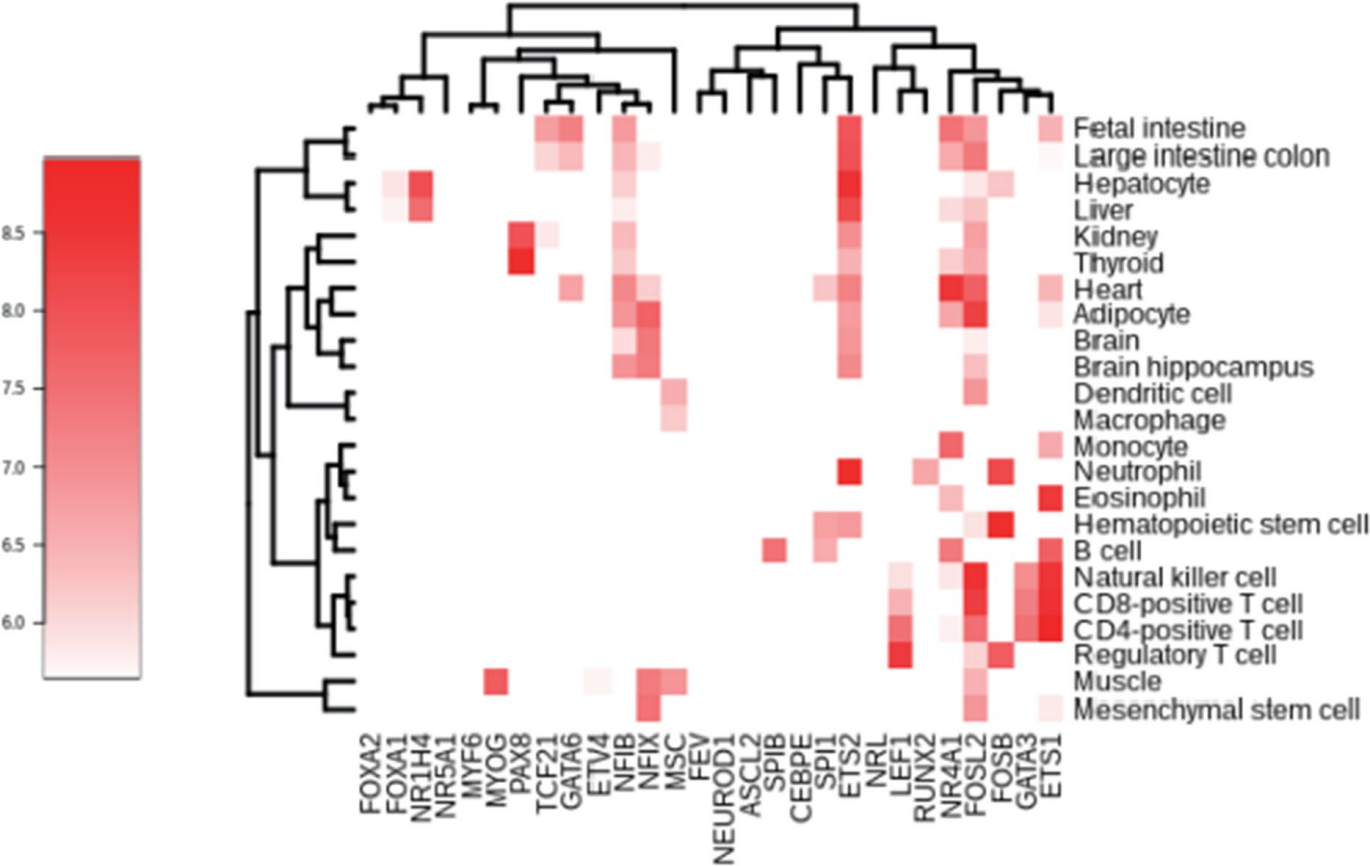
Gene expression analysis of the identified TFs with DNA-demethylation-promoting activity. Gene-expression level of the 28 identified TFs in 23 cells or tissues was subjected to cluster analysis and color-coded

Finally, we explored the gene expression of the 28 identified TFs by using a FANTOM dataset (Forrest et al. 2014) covering 23 representative human cells or tissues (Fig. 6, Supplementary Tables S6 and S7). All of the identified TFs showed cell/tissue-specific expression patterns. Multiple TFs from various TF families (2–8/family) showed high expression at more than 50 tags per million in each cell or tissue examined. In contrast, several TFs (i.e., ASCL2, CEBPE, FEV, MYF6, NR5A1, and NRL) showed low expression at less than 10 tags per million; these TFs may be expressed in other cells or tissues or they may be transiently expressed during human cellular development. For example, it is well known that ASCL2 and FEV play important roles in the development of neural and hematopoietic stem cells, respectively (Liu et al. 2019; Wang et al. 2013), and MYF6 plays an important role in the development of skeletal muscle cells (Hernandez-Hernandez et al. 2017). Taken together, these data indicate that several of the identified TFs are expressed during development of the tissues/cells examined, suggesting that those TFs play an important role in the development of the associated cell-specific DNA methylation profiles.

## Discussion

Here, we systematically analyzed IHEC methylome data to predict the TFs associated with DNA demethylation during cellular differentiation. We analyzed the enrichment of TFBMs in regions of demethylated DNA by using a novel bioinformatics pipeline that utilized IMAGE position weight matrices and the MethylKit and CentriMo informatics tools. As a result, we identified 427 TFBM-TFs from among 393 TFBMs and 383 TFs that are likely associated with the promotion of DNA demethylation at their binding sites. The predicted TFBM-TFs showed clear clustering both by lineage and TF family motif (Supplementary Fig. 1). Although we need to be careful that the IHEC methylome data has a strong bias toward hematopoietic lineage cells, we identified ETS, IRF, and RUNT motif enrichment in hematopoietic lineage cells (Supplementary Table S3), which is consistent with previous reports that TFs containing these motifs play important roles in hematopoietic cell differentiation (Ciau-Uitz et al. 2013; de Bruijn and Dzierzak 2017; Tamura et al. 2008). It is possible that these TFs regulate not only the transcription of downstream genes but also the creation of the whole epigenomic landscape, which includes DNA methylation status, to ensure that lineage-specific genes are correctly expressed.

The IMAGE position weight matrices used in the prediction of the TFBM-TFs may be sensitive enough to distinguish subtle differences among the binding motifs within a TF family. For example, the muscle tissue showed enrichment of several motifs from the bHLH TF family, but that among these motifs MYOG, MYF5, MYF6, and MYOD1 showed a relatively lower motif enrichment *p*-value compared with the others in the family (Supplementary Table S3). It is known that the genes encoding those motifs are specifically expressed in muscle tissue and that they play an important role in the development of the tissue (Hernandez-Hernandez et al. 2017). Therefore, by means of a careful one-by-one checking of local enrichment *p*-values, together with TF gene expression profiles, it may be possible to identify, or at least narrow down, the TFs associated with DNA demethylation within a target cell or tissue.

Using our previously reported in vitro assay system, we validated the DNA-demethylation-promoting activity of 49 of the 383 candidate TFs and found that 28 TFs from various TF families had DNA-demethylation-promoting activity, suggesting that a large number of TFs possess this activity. Subsequent expression analysis revealed that several of the identified TFs are co-expressed in human tissues and cells (Fig. 6). Although we do not exclude the possibility of passive DNA demethylation and we need to be careful that RNA expression does not always correlate with protein expression, our results suggest that DNA demethylation that occurs during cellular differentiation is associated with specific TFs.

Our in vitro DNA demethylation assay system has the advantage that it can be used with any TF-encoding gene. In addition, the analysis of DNA-demethylated probes facilitates examination of DNA-demethylation-promoting activity. In the present study, the TFs with DNA-demethylation-promoting activity showed a larger demethylated/methylated array probe ratio and their demethylated CpGs showed significant bias toward hypermethylation in original 293 T cells (Fig. 3a and b). Furthermore, each TF showed distinct DNA-demethylated regions but with slight overlap of these regions among members of the same TF family, which is consistent with the findings of our GO term analysis (Figs. 4 and 5), indicating that the assay was working properly despite the use of an ectopic overexpression system. Nonetheless, our approach only allows for DNA-demethylation-promoting activity to be examined in a single type of cell (HEK293T cells), which may cause the activities of some TFs to be overlooked or not detected. Recently, another method for determining the DNA-demethylation-promoting activity of TFs has been reported that uses reporter DNA fragments introduced into embryonic stem cells and in vitro differentiated neuronal progenitor cells (Vanzan et al. 2021). Although their target TFs were different from ours, FOXA1 was the overlapping TF examined and showed DNA-demethylation-promoting activity in both methods. The method by Vanzan et al. is superior to our approach in that the analysis is conducted in the type of cell in which the target TF is endogenously expressed. However, the method may be unable to identify TFs with DNA-demethylation-promoting activity if more than two TFs with similar binding motifs are simultaneously expressed by the type of cell under investigation. Thus, a complementarity approach using both our method and that of Vanzan et al. may be optimal.

Our results of various types of TFs with DNA-demethylation-promoting activity raise the question of how these TFs recruit DNA demethylation complexes such as TET proteins. Recently, Chen et al. (2018) reported that SMAD nuclear interacting protein 1 (SNIP1) physically interacts with TET2 and bridges TET2 to bind several transcription factors. We previously showed TET2 association with TF RUNX1 by co-immunoprecipitation (Suzuki et al. 2017b), although our approach did not completely exclude the possibility of SNIP1 involvement. Because *SNIP1* expression is observed in most cells and tissues in the FANTOM gene expression atlas (Forrest et al. 2014), SNIP1 may be broadly involved in recruitment of TET to TFs with DNA-demethylation-promoting activity.

Finally, the present bioinformatics pipeline and analysis results will be useful for future studies examining the TFs associated with the creation of DNA methylation profiles during human cellular development. Furthermore, we provided our analysis results as a resource for use by the scientific community; we hope that these results will be valuable for scientists interested in examining cell-specific DNA methylation profiles and TFs with DNA-demethylation-promoting activity.

## Supplementary Information

Below is the link to the electronic supplementary material.

Supplementary file1 (DOCX 519 KB)

Supplementary file2 (DOCX 146 KB)

Supplementary file3 (DOCX 120 KB)

Supplementary file4 (DOCX 80 KB)

Supplementary file5 (DOCX 51 KB)

Supplementary file6 (DOCX 38 KB)

Supplementary file7 (DOCX 32 KB)

Supplementary file8 (DOCX 1994 KB)

Supplementary file9 (DOCX 19 KB)

Supplementary file10 (DOCX 20 KB)

Supplementary file11 (DOCX 32 KB)

## Data Availability

The bioinformatics pipeline and datasets from this study are available from https://github.com/RIKEN-CFCT/methylome-pipeline. The raw data from the in vitro assay of DNA-demethylation-promoting activity is available from the Gene Expression Omnibus (accession number: GSE171773). The raw data from the in vitro assay is also available through the Figshare (https://doi.org/10.6084/m9.figshare.18376592.v1).

## Abbreviations

GO: Gene ontology
IHEC: International Human Epigenome Consortium
TET: Ten-eleven translocation enzymes
TF: Transcription factor
TFBM: TF binding motif
WGBS: Whole-genome bisulfite sequencing
