# Supplementary material for: Prediction of transcription factors associated with DNA demethylation during human cellular development
Source: Chromosome Res. 2022 Feb 10;30(1):109–21. doi: 10.1007/s10577-022-09685-6 (PMC8942926; doi:10.1007/s10577-022-09685-6)
Supplement: Supplementary file 1 — Supplementary file1 (DOCX 519 KB) [file 10577_2022_9685_MOESM1_ESM.docx]

**Figure S1**

**Color Key and Histogram**


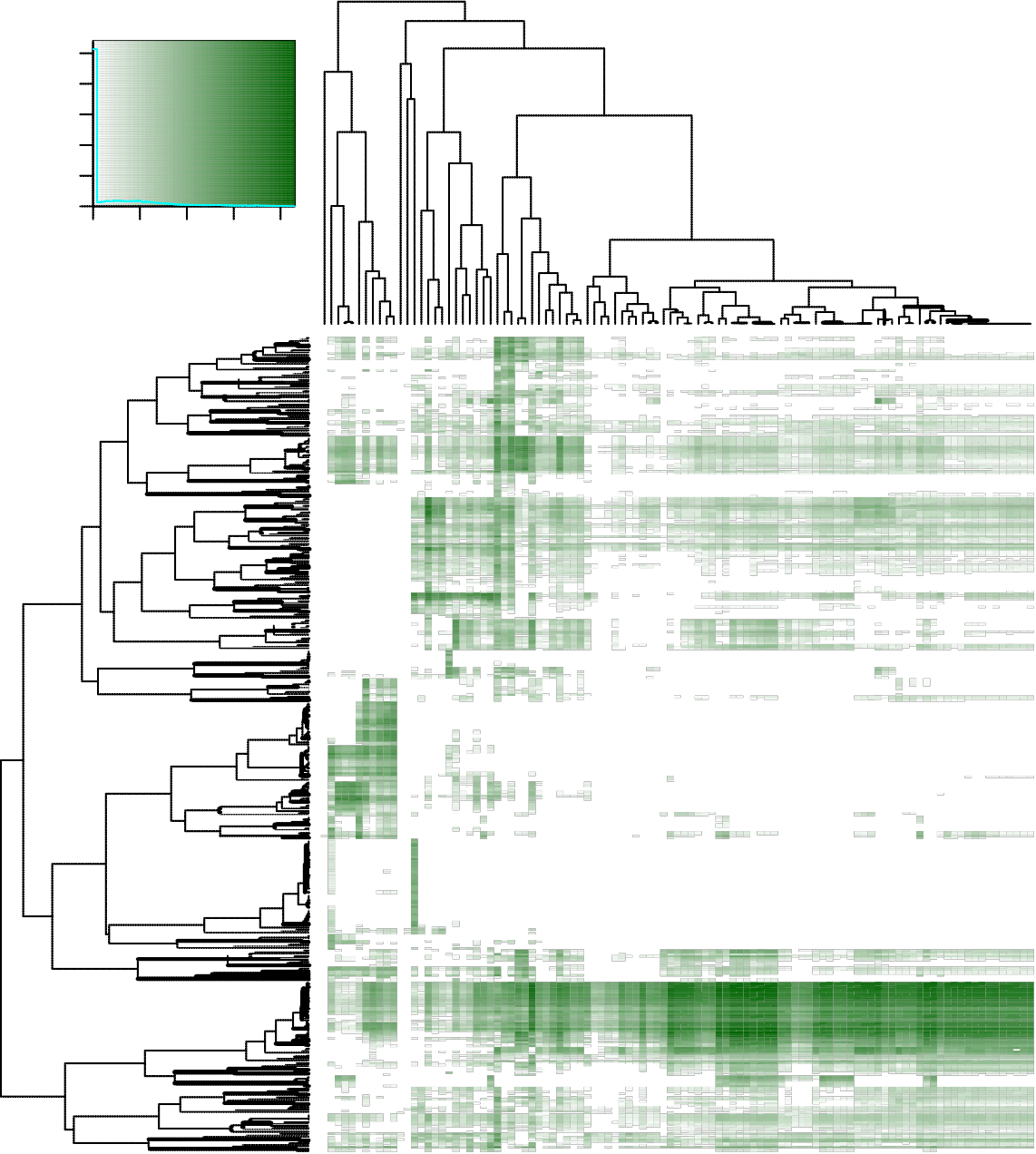

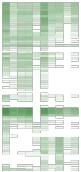


25000

2 2.5 3 3.5 4

Count

10000

0

Value

USF2_1.motif_USF2

TFEC_1.motif_TFEC ENO1_1.motif_ENO1

ATF6_1.motif_CREBZF ATF6_1.motif_ATF6

ARNTL_1.motif_ARNTL HES1_1.motif_HES6

HES1_1.motif_HES1

HES4_1.motif_HES4

ARNT_2.motif_ARNT ZBTB14_1.motif_ZBTB14

HEYL_1.motif_HEYL IRF6_1.Infer.motif_IRF6

HES7_1.motif_HES7

ZBTB7A_2.motif_ZBTB7A NFKB1_1.motif_NFKB1

NFKB2_1.motif_NFKB2

TCF3_1.motif_TCF3

ZFY_1.motif_ZFY ZFX_1.motif_ZFX TFAP2A_1.motif_TFAP2A ZFAT_2.motif_ZFAT ZNF441_1.motif_ZNF441

ZNF597_2.motif_ZNF597

TFAP2D_1.motif_TFAP2D ZIC4_1.motif_ZIC4

ZIC5_3.motif_ZIC5

PLAGL1_1.motif_PLAGL1

RELA_1.motif_RELA NFKB1_2.motif_NFKB1

ZBTB33_1.motif_ZBTB33

RELB_1.motif_RELB EBF1_1.motif_EBF2

EBF1_1.motif_EBF1

EBF1_1.motif_EBF4

ZNF329_1.motif_ZNF329

KLF12_1.motif_KLF12

ZNF606_1.motif_ZNF606

ZNF445_3.motif_ZNF445

ZFP64_1.motif_ZFP64

ZNF768_1.motif_ZNF768

ZNF550_1.motif_ZNF550

ZNF781_1.motif_ZNF781

ZNF343_1.motif_ZNF343

ZNF777_2.motif_ZNF777

ZNF142_3.motif_ZNF142

TFAP2A_2.motif_TFAP2A TFAP2E_1.motif_TFAP2E TFAP2A_3.motif_TFAP2A ZNF594_4.motif_ZNF594

MAFG_1.motif_MAFG MAFF_2.motif_MAFF MXI1_1.motif_MXD3

MXI1_1.motif_MXD1

MXI1_1.motif_MXD4

MXI1_1.motif_MXI1

MAX_2.motif_MAX MYCN_1.motif_MYCL NPAS2_1.motif_NPAS2

CREB3L3_1.motif_CREB3L3

CREB3L2_1.motif_CREB3L2

CLOCK_1.motif_SOHLH1

CLOCK_1.motif_CLOCK ARNT_1.motif_ARNT USF1_1.motif_USF1

TFEB_1.motif_TFEB TFE3_1.motif_TFE3

ATF3_1.motif_ATF3

BHLHE40_1.motif_BHLHE40

MLXIPL_1.motif_MLXIPL ZNF433_2.motif_ZNF433

MTF1_1.motif_MTF1

NR1H4_1.motif_NR1H4

RARA_1.motif_RARA NR2F1_2.motif_NR2F1

RXRA_4.motif_RXRA NR2C1_1.motif_NR2C1

RHOXF1_1.motif_RHOXF2B RHOXF1_1.motif_RHOXF1

RHOXF1_1.motif_RHOXF2

ZNF827_1.motif_ZNF827

ZNF292_1.motif_ZNF292

ZNF579_1.motif_ZNF579

ZNF100_1.motif_ZNF100

MYF6_1.motif_MYF6

MYF6_1.motif_ASCL3

MSC_1.motif_MSC TCF21_1.motif_TCF21

ASCL2_1.motif_ASCL2

MYOG_1.motif_MYOG ATOH1_1.motif_ATOH1

MYF5_1.motif_ASCL5

MYF5_1.motif_ASCL1

MYOD1_1.motif_MYOD1

TCF12_1.motif_TCF12

FERD3L_1.motif_FERD3L ATOH7_1.motif_ATOH7

NEUROD1_1.motif_NEUROD1

MAFB_1.motif_MAFB ZNF516_2.motif_ZNF516

NRL_1.motif_NRL MAF_1.motif_MAF RFX1_1.motif_RFX1

ZNF732_1.motif_ZNF732

ZFP57_1.motif_ZFP57

PTF1A_1.motif_PTF1A ZNF208_2.motif_ZNF208

ZNF208_1.motif_ZNF208

ZNF594_2.motif_ZNF594

ZNF2_1.motif_ZNF2

TFAP4_2.motif_TFAP4

LYL1_1.Infer.motif_LYL1

SMAD3_2.motif_SMAD7

SMAD3_2.motif_SMAD3

SMAD4_1.motif_SMAD6

ZNF763_2.motif_ZNF763

SMAD2_2.motif_SMAD2

RFX5_1.motif_RFX5

ZNF536_2.motif_ZNF536

ZNF547_1.motif_ZNF547

ZNF699_1.motif_ZNF699

ZNF716_1.motif_ZNF716

SMAD1_1.motif_SMAD9

MAFA_1.motif_MAFA ZNF836_2.motif_ZNF836

HIC1_1.motif_HIC1

RFX1_2.motif_RFX1

ZNF594_3.motif_ZNF594

MEIS2_1.motif_MEIS2

TGIF2LX_1.motif_TGIF2LX TAL1_1.motif_TAL1

ZBTB18_2.motif_ZBTB18

ZBTB42_1.motif_ZBTB42

NEUROD4_1.motif_NEUROD4

NFIX_1.motif_NFIX NFIB_1.motif_NFIB NFIA_1.motif_NFIA NFIX_2.motif_NFIX NFIC_1.motif_NFIC HAND1_1.motif_HAND1

TLX1_2.motif_TLX1

RFX4_2.motif_RFX4

RFX3_1.motif_RFX3

RFX2_3.motif_RFX2

RFX5_2.motif_RFX5

TEAD1_1.motif_TEAD2

TEAD3_2.motif_TEAD3

SIX1_1.motif_SIX1

JUNB_1.motif_JUNB FOS_1.motif_FOS FOSB_1.motif_FOSB JDP2_1.motif_JDP2

NFE2_2.motif_NFE2

JUN_1.motif_JUN FOSL1_1.motif_FOSL1

BACH2_1.motif_BACH2

BACH1_1.motif_BACH1

SMARCC1_1.motif_TADA2A SMARCC1_1.motif_SMARCC1

FOSL2_1.motif_FOSL2

JUND_1.motif_JUND NFE2L2_1.motif_NFE2L2

NFE2_1.motif_NFE2

PAX2_3.motif_PAX2

NKX2_8_1.motif_NKX2−8

NKX3_1_2.motif_NKX3−1

NKX2_4_2.motif_NKX2−4

NKX2_5_1.motif_NKX2−5

NKX2_2_1.motif_NKX2−2

NKX2_1_1.motif_NKX2−1

NKX2_3_1.motif_NKX2−3

NKX2_6_1.motif_NKX2−6

NKX2_5_3.motif_NKX2−5

PAX5_2.motif_PAX5

PAX1_1.motif_PAX1

PAX2_1.motif_PAX2

PAX6_1.motif_PAX6

PAX8_1.motif_PAX8

PAX8_2.motif_PAX8

KLF4_1.motif_KLF2

KLF4_1.motif_KLF17

KLF4_1.motif_KLF4

KLF1_1.motif_KLF1

KLF7_1.motif_KLF7

KLF14_1.motif_KLF14

KLF13_1.motif_KLF13

KLF13_1.motif_KLF11

KLF12_2.motif_KLF12

KLF5_1.motif_KLF5

KLF16_1.motif_KLF16

SP3_1.motif_SP3

HOXD13_1.motif_HOXD13

HOXA13_1.motif_HOXA13

HOXB13_2.motif_HOXB13

HOXC13_2.motif_HOXC13

HOXB13_1.motif_HOXB13

HOXD13_2.motif_HOXD13

HOXA11_1.motif_HOXA11

HOXD11_1.motif_HOXD11

CDX1_1.motif_CDX1

HOXC11_2.motif_HOXC11

HOXA10_1.motif_HOXA10

CDX2_1.motif_CDX2

HOXD12_1.motif_HOXD12

HOXA6_4.motif_HOXA6

ZNF560_3.motif_ZNF560

HOXD9_2.motif_HOXD9

HOXC12_2.motif_HOXC12

HOXA9_1.motif_HOXA9

HOXC10_1.motif_HOXC10

HOXD12_2.motif_HOXD12

HOXC11_1.motif_HOXC11

FOXQ1_1.motif_FOXQ1

HOXA13_2.motif_HOXA13

FOXD2_2.motif_FOXD2

FOXG1_1.motif_FOXG1

FOXD4_1.motif_FOXD4

FOXP3_1.motif_FOXP3

FOXA1_1.motif_FOXA1

FOXA2_1.motif_FOXA2

FOXC1_1.motif_FOXC1

FOXP1_1.motif_FOXP1

FOXF1_1.motif_FOXN1

FOXF1_1.motif_FOXF1

FOXF1_1.motif_FOXN4

FOXF2_1.motif_FOXF2

FOXD1_1.motif_FOXD1

FOXO3_1.motif_FOXO3

FOXK1_1.motif_FOXK1

FOXP2_1.motif_FOXP2

HOXC9_1.motif_HOXC9

HOXA6_10.motif_HOXA6

HOXA9_2.motif_HOXA9

PPARG_1.motif_PPARG RXRA_3.motif_RXRA NR2F1_1.motif_NR2F1

NR2F6_3.motif_NR2F6

NR2C2_1.motif_NR2C2

NR2F2_2.motif_NR2F2

PPARD_1.motif_PPARD HNF4G_1.motif_HNF4G HNF4A_1.motif_HNF4A HNF4A_2.motif_HNF4A RXRG_1.motif_RXRG RXRB_1.motif_RXRB NR4A2_3.motif_NR4A2

NR4A1_1.motif_NR4A1

NR4A3_1.motif_NR4A3

NR1H2_1.motif_NR1H2

LEF1_1.motif_LEF1

TCF7_1.motif_TCF7

GATA6_1.motif_MTA2

GATA6_1.motif_GATA6

GATA6_1.motif_RERE GATA5_1.motif_GATA5

GATA2_1.motif_ZGLP1

GATA2_1.motif_GATA2

HNF1B_1.motif_HNF1B HNF1A_1.motif_HNF1A GATA1_1.motif_GATAD2B GATA1_1.motif_GATA1

GATA4_1.motif_GATA4

GATA3_2.motif_GATA3

HOXA2_1.motif_HOXA2

BSX_1.motif_BSX LHX2_1.motif_LHX2

VAX1_1.motif_VAX1

NOBOX_1.motif_NOBOX HOXD3_1.motif_HOXD3

MNX1_1.motif_MNX1

NKX6_2_1.motif_NKX6−2

HOXB5_1.motif_HOXB5

BARX1_2.motif_BARX1

VAX2_1.motif_VAX2

EVX2_1.motif_EVX2

MEOX2_1.motif_MEOX2

NKX6_3_1.motif_NKX6−3

HOXA1_1.motif_HOXA1

HOXA6_7.motif_HOXA6

EN1_2.motif_EN1

ESX1_1.motif_ESX1

HOXB3_1.motif_HOXB3

HOXB2_1.motif_HOXB2

HOXD1_1.motif_HOXD1

GSX1_1.motif_GSX1

GSX2_1.motif_GSX2

LBX1_1.motif_LBX1

LMX1A_1.motif_LMX1A DLX6_1.motif_DLX6

GBX2_1.motif_GBX2

MSX1_1.motif_NKX1−2

MSX1_1.motif_MSX1

DLX2_1.motif_DLX2

DLX1_1.motif_DLX1

ALX3_1.motif_ALX3

PAX4_5.motif_PAX4

VSX1_1.motif_VSX1

OTP_1.motif_OTP PDX1_2.motif_PDX1

GBX1_1.motif_GBX1

ZFHX2_1.motif_ZFHX2

LMX1B_1.motif_LMX1B HOXA6_8.motif_HOXA6

LHX6_1.motif_LHX6

HOXA6_1.motif_HOXA6

HOXA5_2.motif_HOXA5

HOXC5_1.motif_HOXC5

HOXA6_9.motif_HOXA6

MEOX1_1.motif_MEOX1

RFX6_4.motif_RFX6

SOX6_1.motif_SOX6

SOX10_5.motif_SOX10

SOX3_1.motif_SOX3

ONECUT1_1.motif_SATB1

ONECUT1_1.motif_ONECUT1

ONECUT1_1.motif_SATB2

NR5A1_1.motif_NR5A1

ESRRG_3.motif_ESRRG HOXB7_1.motif_HOXB7

HOXB8_1.motif_HOXB8

HOXB4_1.motif_HOXB4

STAT2_1.motif_STAT2

IRF3_1.motif_IRF3

ZNF80_2.motif_ZNF80

IRF8_1.motif_IRF8

IRF4_2.motif_IRF4

IRF5_1.motif_IRF5

IRF7_1.motif_IRF7

IRF1_1.motif_IRF1

IRF9_1.motif_IRF9

MYBL1_1.motif_MYBL1

MYBL1_1.motif_DNAJC2

MYBL1_2.motif_MYBL1

MYBL1_2.motif_DNAJC2

IKZF5_1.motif_IKZF5

BARHL2_1.motif_BARHL2

BATF_1.motif_BATF BATF_1.motif_BATF2

FLI1_1.motif_FLI1

ETV4_1.motif_ETV4

ETS1_1.motif_ETS1

ERG_3.motif_ERG FEV_1.motif_FEV ETV2_1.motif_ETV2

ETV1_1.motif_ETV1

ERF_1.motif_ERF ERG_1.motif_ERG ELK4_1.motif_ELK4

ELK3_1.motif_ELK3

GABPA_1.motif_GABPA ELF2_1.motif_ELF2

ETV3_1.motif_ETV3L ETV3_1.motif_ETV3

ELK1_1.motif_ELK1

SPDEF_1.motif_SPDEF ETS2_1.motif_ETS2

EHF_1.motif_EHF ETV7_1.motif_ETV7

ETV5_1.motif_ETV5

ELF5_1.motif_ELF5

EHF_2.motif_EHF ELF3_1.motif_ELF3

ELF1_1.motif_ELF1

ELF4_1.motif_ELF4

SPIB_2.motif_SPIB SPIC_1.motif_SPIC SPI1_1.motif_SPI1

SPIB_1.motif_SPIB BCL11A_1.motif_BCL11A ETV6_1.motif_ETV6

SPI1_2.motif_SPI1

IKZF3_1.motif_IKZF3

RUNX2_1.motif_RUNX2

RUNX1_1.motif_RUNX1

CBFB_1.motif_CBFB RUNX3_1.motif_RUNX3

FLI1_2.motif_FLI1

ERG_2.motif_ERG ETS1_2.motif_ETS1

NR2C2_2.motif_NR2C2

ZNF296_2.motif_ZNF296

ZNF296_1.motif_ZNF296

ZNF721_2.motif_ZNF721

ZNF770_2.motif_ZNF770

ZNF705D_1.motif_ZNF705D

NKX3_2_1.motif_NKX3−2

ZNF681_1.motif_ZNF681

CEBPD_1.motif_CEBPD CEBPB_1.motif_CEBPB CEBPE_1.motif_CEBPE HLF_1.motif_HLF CEBPA_1.motif_CEBPA DBP_1.motif_DBP NR0B1_1.motif_NR0B2

NR0B1_1.motif_NR0B1

ZSCAN25_1.motif_ZSCAN25

ZIC5_1.motif_ZIC5

ZNF746_1.motif_ZNF746

ZNF19_1.motif_ZNF19

ZNF676_1.motif_ZNF676

ZNF579_3.motif_ZNF579

EGR2_1.motif_EGR2

EGR4_1.motif_EGR4

KLF6_1.motif_KLF6

ZIC5_2.motif_ZIC5

PLAGL2_2.motif_PLAGL2

E2F1_1.motif_E2F1

MYB_1.motif_RCOR2

MYB_1.motif_MYB MYB_1.motif_RCOR3

MYB_1.motif_SNAPC4

HIVEP2_1.motif_HIVEP2

ZNF350_1.motif_ZNF350

AHR_1.motif_AHR ZNF566_1.motif_ZNF566

ZNF91_3.motif_ZNF91

ZNF548_1.motif_ZNF548

MECP2_1.motif_MECP2

MBD2_1.motif_MBD2

ZNF335_1.motif_ZNF335

ZBTB1_1.Infer.motif_ZBTB1

PRDM15_4.motif_PRDM15

STAT3_1.motif_STAT3

STAT1_1.motif_STAT1

ZNF445_2.motif_ZNF445

CREB5_1.motif_CREB5

ATF7_1.motif_ATF7

hesc_derived_cd184positive_endoderm_cultured_cells..nih_roadmap

islet_cell..knih

hepatocyte..deep hapatocyte..crest

liver..crest fetal_intestine..nih_roadmap normal_human_colon_absorptive_epithelial_cells..crest sigmoid_colon..nih_roadmap large_intestine_colon_ascending_.right...ceehrc

large_intestine_colon..ceehrc large_intestine_colon_rectosigmoid..ceehrc

ips..knih hesc_derived_cd56positive_ectoderm_cultured_cells..nih_roadmap neurosphere_cultured_cells_cortex_derived..nih_roadmap neurosphere_cultured_cells_ganglionic_eminence_derived..nih_roadmap muscle..ceehrc

brain..ceehrc

brain_hippocampus_middle..nih_roadmap thyroid..ceehrc podocyte..knih mesangial..knih preadipocyte..knih kidney..ceehrc heart..nih_roadmap

adipocyte..knih

mesenchymal_stem_cell_of_the_bone_marrow..blueprint class_switched_memory_b_cell..blueprint plasma_cell..blueprint

b_cell..ceehrc

cd19positive_cells..ceehrc

adult_endothelial_progenitor_cell..blueprint

cd4_positive_alpha_beta_memory_t_cell..deep memory_b_cell..blueprint germinal_center_b_cell..blueprint venous_blood..blueprint

effector_memory_cd4_positive__alpha_beta_t_cell..blueprint

effector_memory_cd8_positive__alpha_beta_t_cell..blueprint

effector_memory_cd8_positive__alpha_beta_t_cell__terminally_differentiated..blueprint

endothelial_cell_of_umbilical_vein_.proliferating...blueprint endothelial_cell_of_umbilical_vein_.resting...blueprint erythroblast..blueprint cd8_positive__alpha_beta_t_cell..blueprint central_memory_cd8_positive__alpha_beta_t_cell..blueprint

cytotoxic_cd56_dim_natural_killer_cell..blueprint

naive_cd4_positive_t_cell..deep

cd34_negative__cd41_positive__cd42_positive_megakaryocyte_cell..blueprint hematopoietic_multipotent_progenitor_cell..blueprint cd38_negative_naive_b_cell..blueprint precursor_lymphocyte_of_b_lineage..blueprint cd4_positive__alpha_beta_t_cell..blueprint hematopoietic_stem_cell..ceehrc

cd4_naive..ceehrc cd4_positive_helper_t_cell..ceehrc

t_cell..ceehrc

central_memory_cd4_positive__alpha_beta_t_cell..deep central_memory_cd4_positive__alpha_beta_t_cell..blueprint regulatory_t_cell..blueprint

eosinophil..ceehrc mature_eosinophil..blueprint macrophage..deep monocyte..deep monocyte..ceehrc monocyte..blueprint

osteoclast..blueprint immature_conventional_dendritic_cell..blueprint

mature_conventional_dendritic_cell..blueprint cd14_positive__cd16_negative_classical_monocyte..blueprint conventional_dendritic_cell..blueprint

macrophage..blueprint

alternatively_activated_macrophage..blueprint inflammatory_macrophage..blueprint myeloid_cell..blueprint neutrophilic_metamyelocyte..blueprint neutrophilic_myelocyte..blueprint

mature_neutrophil..blueprint

segmented_neutrophil_of_bone_marrow..blueprint

band_form_neutrophil..blueprint cd3_negative__cd4_positive__cd8_positive__double_positive_thymocyte..blueprint cd8_positive__alpha_beta_thymocyte..blueprint cd4_positive__alpha_beta_thymocyte..blueprint

precursor_b_cell..blueprint

naive_b_cell..blueprint

b_cell_from_peripheral_blood..blueprint

natural_killer_cell_from_peripheral_blood..blueprint

cd4positive_t_cell_from_peripheral_blood..blueprint

cd8positive_t_cell_from_peripheral_blood..blueprint

megakaryocyte_from_bone_marrow..blueprint

monocyte_from_peripheral_blood..blueprint

neutrophil_from_peripheral_blood..blueprint

hematopoietic_stem_cell_from_peripheral_blood..blueprint

hematopoietic_stem_cell_from_fetal_liver..blueprint

hematopoietic_stem_cell_from_cord_blood..blueprint

multipotent_progenitor_from_cord_blood..blueprint

megakaryocyte_erythrocyte_progenitor_from_peripheral_blood..blueprint

granulocyte_macrophage_progenitor_from_peripheral_blood..blueprint

common_myeloid_progenitor_from_peripheral_blood..blueprint

hematopoietic_stem_cell_from_bone_marrow..blueprint

multipotent_progenitor_from_peripheral_blood..blueprint

immature_lymphoid_progenitor_0_from_peripheral_blood..blueprint

immature_lymphoid_progenitor_1_from_peripheral_blood..blueprint

common_lymphoid_progenitor_from_peripheral_blood..blueprint

immature_lymphoid_progenitor_2_from_peripheral_blood..blueprint

immature_lymphoid_progenitor_3_from_peripheral_blood..blueprint
