# Supplementary figures and images for: Prediction of transcription factors associated with DNA demethylation during human cellular development
Source: Chromosome Res. 2022 Feb 10;30(1):109–21. doi: 10.1007/s10577-022-09685-6 (PMC8942926; doi:10.1007/s10577-022-09685-6)

**Figure S2**


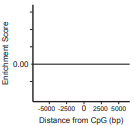


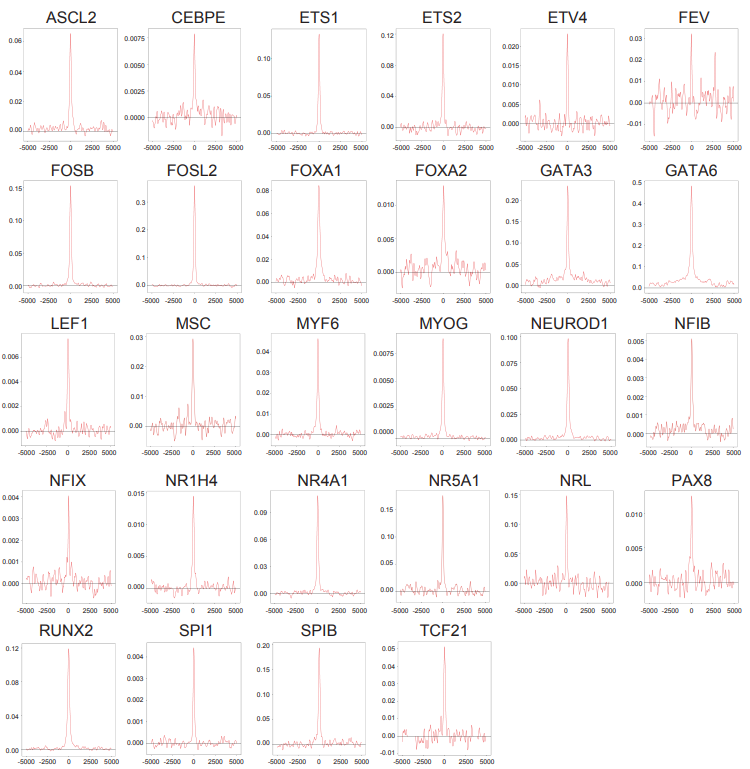

Supplement: Supplementary file 2 — Supplementary file2 (DOCX 146 KB) [file 10577_2022_9685_MOESM2_ESM.docx]

**Figure S3**

**
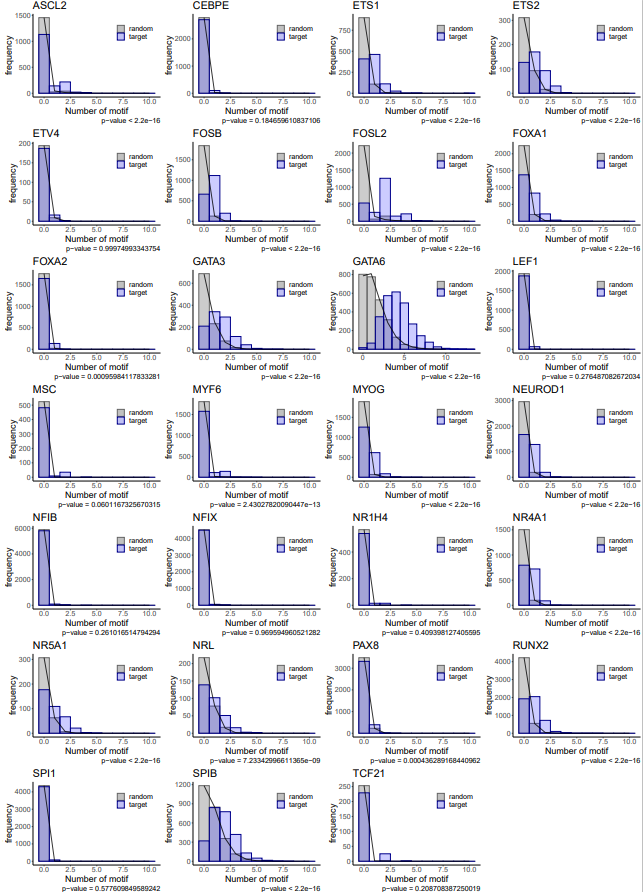
**

Supplement: Supplementary file 3 — Supplementary file3 (DOCX 120 KB) [file 10577_2022_9685_MOESM3_ESM.docx]

**Figure S4**

**
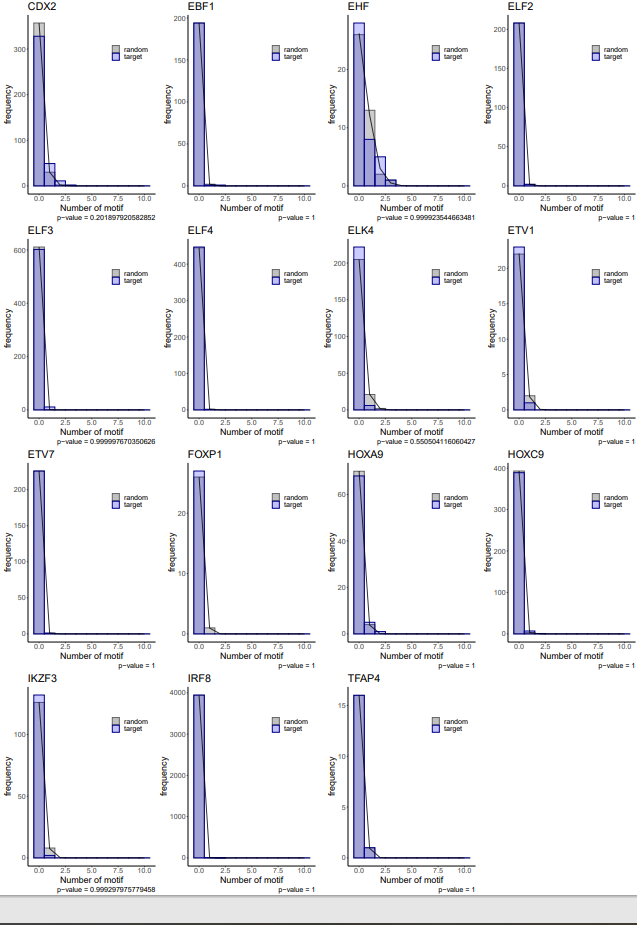
**

Supplement: Supplementary file 4 — Supplementary file4 (DOCX 80 KB) [file 10577_2022_9685_MOESM4_ESM.docx]

**Figure S5**


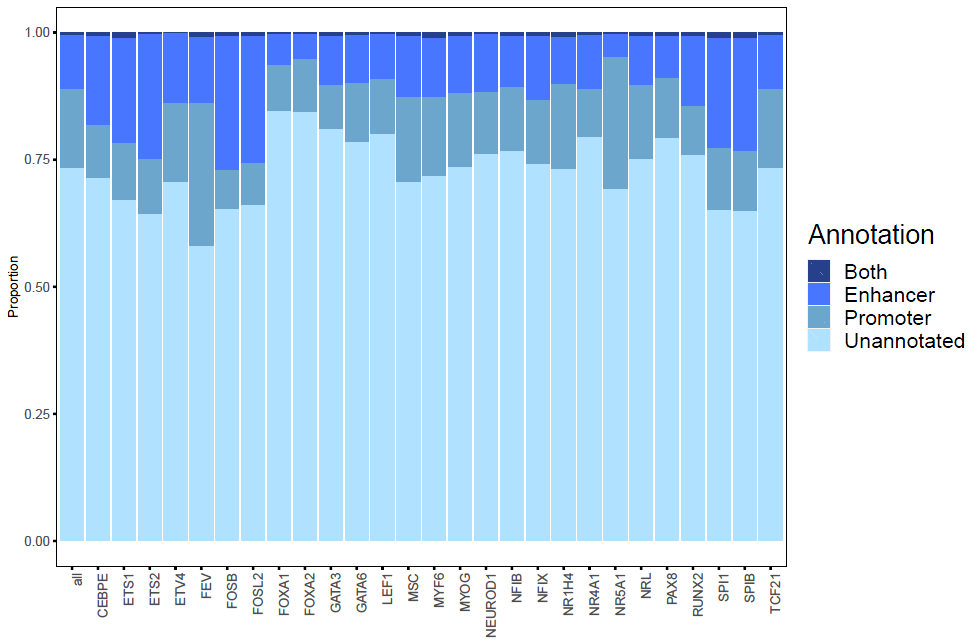

Supplement: Supplementary file 5 — Supplementary file5 (DOCX 51 KB) [file 10577_2022_9685_MOESM5_ESM.docx]
