# Supplementary material for: Prediction of transcription factors associated with DNA demethylation during human cellular development
Source: Chromosome Res. 2022 Feb 10;30(1):109–21. doi: 10.1007/s10577-022-09685-6 (PMC8942926; doi:10.1007/s10577-022-09685-6)
Supplement: Supplementary file 6 — Supplementary file6 (DOCX 38 KB) [file 10577_2022_9685_MOESM6_ESM.docx]

Name of data adipocyte..knih adult_endothelial_progenitor_cell..blueprint alternatively_activated_macrophage..blueprint band_form_neutrophil..blueprint

b_cell..ceehrc b_cell_from_peripheral_blood..blueprint brain..ceehrc brain_hippocampus_middle..nih_roadmap

cd14_positive cd16_negative_classical_monocyte..blueprint cd19positive_cells..ceehrc

cd34_negative cd41_positive cd42_positive_megakaryocyte_cell..blueprint cd38_negative_naive_b_cell..blueprint

cd3_negative cd4_positive cd8_positive double_positive_thymocyte..blueprint cd4_naive..ceehrc

cd4_positive_alpha_beta_memory_t_cell..deep cd4_positive alpha_beta_t_cell..blueprint cd4_positive alpha_beta_thymocyte..blueprint cd4_positive_helper_t_cell..ceehrc cd4positive_t_cell_from_peripheral_blood..blueprint cd8_positive alpha_beta_t_cell..blueprint cd8_positive alpha_beta_thymocyte..blueprint cd8positive_t_cell_from_peripheral_blood..blueprint

central_memory_cd4_positive alpha_beta_t_cell..blueprint central_memory_cd4_positive alpha_beta_t_cell..deep central_memory_cd8_positive alpha_beta_t_cell..blueprint class_switched_memory_b_cell..blueprint common_lymphoid_progenitor_from_peripheral_blood..blueprint common_myeloid_progenitor_from_peripheral_blood..blueprint conventional_dendritic_cell..blueprint cytotoxic_cd56_dim_natural_killer_cell..blueprint early_neuron..knih

effector_memory_cd4_positive alpha_beta_t_cell..blueprint effector_memory_cd8_positive alpha_beta_t_cell..blueprint effector_memory_cd8_positive alpha_beta_t_cell terminally_differentiated..blueprint endothelial_cell_of_umbilical_vein_(proliferating)..blueprint endothelial_cell_of_umbilical_vein_(resting)..blueprint

eosinophil..ceehrc erythroblast..blueprint

fetal_intestine..nih_roadmap germinal_center_b_cell..blueprint granulocyte_macrophage_progenitor_from_peripheral_blood..blueprint hapatocyte..crest

heart..nih_roadmap hematopoietic_multipotent_progenitor_cell..blueprint hematopoietic_stem_cell..ceehrc hematopoietic_stem_cell_from_bone_marrow..blueprint hematopoietic_stem_cell_from_cord_blood..blueprint hematopoietic_stem_cell_from_fetal_liver..blueprint hematopoietic_stem_cell_from_peripheral_blood..blueprint hepatocyte..deep hesc_derived_cd184positive_endoderm_cultured_cells..nih_roadmap hesc_derived_cd56positive_ectoderm_cultured_cells..nih_roadmap hesc_derived_cd56positive_mesoderm_cultured_cells..nih_roadmap immature_conventional_dendritic_cell..blueprint immature_lymphoid_progenitor_0_from_peripheral_blood..blueprint immature_lymphoid_progenitor_1_from_peripheral_blood..blueprint immature_lymphoid_progenitor_2_from_peripheral_blood..blueprint immature_lymphoid_progenitor_3_from_peripheral_blood..blueprint inflammatory_macrophage..blueprint

ips..ceehrc ips..knih islet_cell..knih kidney..ceehrc

large_intestine_colon_ascending_(right)..ceehrc large_intestine_colon..ceehrc large_intestine_colon_rectosigmoid..ceehrc liver..crest

macrophage..blueprint macrophage..deep mature_conventional_dendritic_cell..blueprint mature_eosinophil..blueprint mature_neutrophil..blueprint

megakaryocyte_erythrocyte_progenitor_from_peripheral_blood..blueprint megakaryocyte_from_bone_marrow..blueprint

memory_b_cell..blueprint mesangial..knih mesenchymal_stem_cell_of_the_bone_marrow..blueprint

monocyte..blueprint monocyte..ceehrc monocyte..deep

monocyte_from_peripheral_blood..blueprint multipotent_progenitor_from_cord_blood..blueprint multipotent_progenitor_from_peripheral_blood..blueprint muscle..ceehrc

myeloid_cell..blueprint naive_b_cell..blueprint naive_cd4_positive_t_cell..deep natural_killer_cell_from_peripheral_blood..blueprint neural_progenitor_cell..knih

neurosphere_cultured_cells_cortex_derived..nih_roadmap neurosphere_cultured_cells_ganglionic_eminence_derived..nih_roadmap neutrophil_from_peripheral_blood..blueprint neutrophilic_metamyelocyte..blueprint

neutrophilic_myelocyte..blueprint normal_human_colon_absorptive_epithelial_cells..crest osteoclast..blueprint

plasma_cell..blueprint podocyte..knih preadipocyte..knih precursor_b_cell..blueprint

precursor_lymphocyte_of_b_lineage..blueprint regulatory_t_cell..blueprint segmented_neutrophil_of_bone_marrow..blueprint sigmoid_colon..nih_roadmap

t_cell..ceehrc thyroid..ceehrc venous_blood..blueprint

| IHEC data portal ID | | | | | | | |
| --- | --- | --- | --- | --- | --- | --- | --- |
| 33150 | 33153 |  |  |  |  |  |  |
| 30397 | 30404 |  |  |  |  |  |  |
| 17565 | 17566 | 17574 | 17575 | 31591 | 31722 | 31723 | 31730 |
| 32278 | 32451 | 32274 |  |  |  |  |  |
| 27025 | 27569 | 27599 | 27636 | 27637 | 27812 | 27821 | 33832 |
| 32457 | 32458 | 32459 |  |  |  |  |  |
| 33837 | 33838 | 33836 | 35010 | 35011 | 34043 | 35012 | 35013 |
| 20495 | 20540 |  |  |  |  |  |  |
| 17521 | 17524 | 17528 | 17552 | 17538 | 17539 | 31254 | 31255 |
| 33839 | 33850 | 33840 | 33841 | 33842 | 33843 |  |  |
| 17562 | 31588 | 32455 |  |  |  |  |  |
| 17533 | 17544 | 17553 | 31284 | 31286 | 31703 | 31924 |  |
| 30405 | 30409 | 30406 | 30410 |  |  |  |  |
| 35072 | 35015 | 35016 | 35076 | 35077 |  |  |  |
| 32792 | 32794 | 32795 | 32796 | 32777 |  |  |  |
| 17567 | 17568 | 17571 | 31864 | 31865 | 31866 | 31867 | 31868 |
| 30407 | 30411 |  |  |  |  |  |  |
| 27040 | 27050 | 27060 | 27070 | 27081 | 27094 | 27106 | 27119 |
| 32460 | 32461 | 32462 |  |  |  |  |  |
| 17529 | 17535 | 17542 | 17576 | 31285 | 31582 | 31281 | 31731 |
| 30408 | 30412 |  |  |  |  |  |  |
| 32463 | 32464 | 32465 |  |  |  |  |  |
| 31917 | 30400 |  |  |  |  |  |  |
| 32712 | 32715 | 32718 | 32725 | 32729 | 32743 | 32753 |  |
| 17536 | 17540 | 31705 | 31706 |  |  |  |  |
| 17532 | 31704 | 31915 | 32251 |  |  |  |  |
| 32466 | 32467 | 32468 |  |  |  |  |  |
| 32470 | 32471 | 32472 |  |  |  |  |  |
| 31921 | 31922 |  |  |  |  |  |  |
| 17531 | 17543 | 17545 | 31707 | 31708 | 31283 | 30355 |  |
| 33477 | 33486 |  |  |  |  |  |  |
| 31912 | 30399 |  |  |  |  |  |  |
| 17530 | 31282 | 30401 |  |  |  |  |  |
| 31914 | 30402 |  |  |  |  |  |  |
| 17572 | 31728 | 31923 |  |  |  |  |  |
| 17573 | 31729 | 17577 | 31732 |  |  |  |  |
| 27904 | 27905 | 27906 |  |  |  |  |  |
| 17560 | 17559 | 31586 | 31724 |  |  |  |  |

| 20413 | 20532 |  |  |  |  |  |  |
| --- | --- | --- | --- | --- | --- | --- | --- |
| 17548 | 31469 | 32248 | 30072 |  |  |  |  |
| 32476 | 32477 | 32478 |  |  |  |  |  |
| 32856 | 32857 | 32866 | 32876 | 32896 | 32906 | 32916 |  |
| 20572 | 20543 | 20536 | 20645 | 20548 |  |  |  |
| 17546 | 31467 | 32246 |  |  |  |  |  |
| 33893 | 33673 |  |  |  |  |  |  |
| 32479 | 32481 |  |  |  |  |  |  |
| 32482 | 32483 | 32484 |  |  |  |  |  |
| 32485 | 32486 |  |  |  |  |  |  |
| 32487 | 32488 | 32489 |  |  |  |  |  |
| 32588 | 32599 | 32610 |  |  |  |  |  |
| 20594 | 20465 |  |  |  |  |  |  |
| 20606 | 20610 | 20617 | 20623 |  |  |  |  |
| 20400 | 20402 |  |  |  |  |  |  |
| 30464 | 30466 | 30951 |  |  |  |  |  |
| 32498 | 32499 | 32500 |  |  |  |  |  |
| 32503 | 32504 | 32505 |  |  |  |  |  |
| 32507 | 32508 | 32509 |  |  |  |  |  |
| 32510 | 32511 | 32513 |  |  |  |  |  |
| 17556 | 17558 | 17554 | 31911 | 31589 | 31584 | 31585 | 31919 |
| 27922 | 27935 |  |  |  |  |  |  |
| 33471 | 33480 |  |  |  |  |  |  |
| 33132 | 33135 | 33141 | 33144 | 33147 |  |  |  |
| 27214 | 27223 | 28080 | 28087 | 28094 | 28098 | 28105 | 28111 |
| 33894 | 33895 | 34046 | 34089 | 34056 | 34057 | 34058 | 34060 |
| 34047 | 34048 | 34049 | 34050 | 34051 | 34054 | 34055 |  |
| 34044 | 34045 | 34052 | 34053 | 34059 | 34090 | 34091 |  |
| 32886 | 32958 |  |  |  |  |  |  |
| 17541 | 17555 | 17561 | 17557 | 17578 | 31581 | 31583 | 31590 |
| 32631 | 32641 | 32651 | 32672 | 32682 | 32692 |  |  |
| 30467 | 30465 | 30952 |  |  |  |  |  |
| 31916 | 30354 |  |  |  |  |  |  |
| 17522 | 17523 | 17525 | 17526 | 17537 | 17527 | 31253 | 31188 |
| 32491 | 32492 | 32493 |  |  |  |  |  |
| 32494 | 32495 | 32496 |  |  |  |  |  |
| 17534 | 31702 | 31900 | 30403 |  |  |  |  |
| 33456 | 33465 |  |  |  |  |  |  |
| 30382 | 30383 |  |  |  |  |  |  |

| 30949 | 30950 | 30430 | 30427 | 30425 | 30429 | 30423 | 30422 |
| --- | --- | --- | --- | --- | --- | --- | --- |
| 27004 | 27022 | 27029 | 27084 | 27109 | 27122 | 27235 | 27273 |
| 32621 | 32661 | 32702 |  |  |  |  |  |
| 32524 | 32525 | 32526 |  |  |  |  |  |
| 32515 | 32516 | 32517 |  |  |  |  |  |
| 32518 | 32519 | 32520 | 32523 |  |  |  |  |
| 27132 | 27142 | 27161 | 27178 | 27188 | 27197 | 27321 | 27331 |
| 17563 | 17564 | 17569 | 17570 | 30389 | 31593 | 31592 | 31710 |
| 30476 | 32250 | 30076 |  |  |  |  |  |
| 32721 | 32732 | 32764 | 32797 | 32798 | 32799 | 32800 |  |
| 32527 | 32528 | 32529 |  |  |  |  |  |
| 33474 | 33483 |  |  |  |  |  |  |
| 20474 | 20451 |  |  |  |  |  |  |
| 20517 | 20443 |  |  |  |  |  |  |
| 32530 | 32531 | 32532 |  |  |  |  |  |
| 32283 | 32277 | 32450 |  |  |  |  |  |
| 32282 | 32276 | 32449 |  |  |  |  |  |
| 35176 | 35186 | 35196 | 35206 | 35216 | 35226 | 35236 | 35306 |
| 30953 | 30954 |  |  |  |  |  |  |
| 17549 | 17550 | 17551 | 31466 | 31471 | 31470 | 32249 | 32453 |
| 33459 | 33462 | 33159 | 33162 |  |  |  |  |
| 33156 | 33489 | 33492 |  |  |  |  |  |
| 30956 | 30955 | 31081 | 31082 | 31083 | 31084 | 31085 | 32247 |
| 17547 | 31468 |  |  |  |  |  |  |
| 31913 | 30398 |  |  |  |  |  |  |
| 32275 | 32279 | 32452 |  |  |  |  |  |
| 20609 | 20601 |  |  |  |  |  |  |
| 27011 | 27030 | 27488 | 27529 | 27568 | 27593 | 27628 | 27692 |
| 33844 | 33845 | 33846 | 33847 | 33848 | 33849 | 34092 | 34093 |
| 30474 | 30471 | 30468 | 30469 | 30472 | 30473 | 30470 | 30475 |

|  |  |  |  |  |  |  |  |  |
| --- | --- | --- | --- | --- | --- | --- | --- | --- |
|  |  |  |  |  |  |  |  |  |
| 32456 | 31925 |  |  |  |  |  |  |  |
|  |  |  |  |  |  |  |  |  |
| 33833 | 33834 | 34783 | 34784 | 34785 | 34828 | 35006 | 35007 |  |
|  |  |  |  |  |  |  |  |  |
| 35014 | 35008 | 35009 | 33835 |  |  |  |  |  |
|  |  |  |  |  |  |  |  |  |
| 31186 | 31187 | 31189 | 31252 |  |  |  |  |  |
|  |  |  |  |  |  |  |  |  |
|  |  |  |  |  |  |  |  |  |
|  |  |  |  |  |  |  |  |  |
|  |  |  |  |  |  |  |  |  |
|  |  |  |  |  |  |  |  |  |
|  |  |  |  |  |  |  |  |  |
| 31869 | 31870 | 31871 | 31725 | 31918 | 31726 | 31727 |  |  |
|  |  |  |  |  |  |  |  |  |
| 27232 | 27244 | 27255 | 27270 | 27289 | 27300 | 27311 | 27470 | 27687 |
|  |  |  |  |  |  |  |  |  |
|  |  |  |  |  |  |  |  |  |
|  |  |  |  |  |  |  |  |  |
|  |  |  |  |  |  |  |  |  |
|  |  |  |  |  |  |  |  |  |
|  |  |  |  |  |  |  |  |  |
|  |  |  |  |  |  |  |  |  |
|  |  |  |  |  |  |  |  |  |
|  |  |  |  |  |  |  |  |  |
|  |  |  |  |  |  |  |  |  |
|  |  |  |  |  |  |  |  |  |
|  |  |  |  |  |  |  |  |  |
|  |  |  |  |  |  |  |  |  |
|  |  |  |  |  |  |  |  |  |
|  |  |  |  |  |  |  |  |  |
|  |  |  |  |  |  |  |  |  |
|  |  |  |  |  |  |  |  |  |
|  |  |  |  |  |  |  |  |  |
|  |  |  |  |  |  |  |  |  |
|  |  |  |  |  |  |  |  |  |

|  |  |  |  |  |  |  |  |  |
| --- | --- | --- | --- | --- | --- | --- | --- | --- |
|  |  |  |  |  |  |  |  |  |
|  |  |  |  |  |  |  |  |  |
|  |  |  |  |  |  |  |  |  |
|  |  |  |  |  |  |  |  |  |
|  |  |  |  |  |  |  |  |  |
|  |  |  |  |  |  |  |  |  |
|  |  |  |  |  |  |  |  |  |
|  |  |  |  |  |  |  |  |  |
|  |  |  |  |  |  |  |  |  |
|  |  |  |  |  |  |  |  |  |
|  |  |  |  |  |  |  |  |  |
|  |  |  |  |  |  |  |  |  |
|  |  |  |  |  |  |  |  |  |
|  |  |  |  |  |  |  |  |  |
|  |  |  |  |  |  |  |  |  |
|  |  |  |  |  |  |  |  |  |
|  |  |  |  |  |  |  |  |  |
|  |  |  |  |  |  |  |  |  |
|  |  |  |  |  |  |  |  |  |
| 31926 |  |  |  |  |  |  |  |  |
|  |  |  |  |  |  |  |  |  |
|  |  |  |  |  |  |  |  |  |
|  |  |  |  |  |  |  |  |  |
|  |  |  |  |  |  |  |  |  |
|  |  |  |  |  |  |  |  |  |
|  |  |  |  |  |  |  |  |  |
|  |  |  |  |  |  |  |  |  |
|  |  |  |  |  |  |  |  |  |
| 31587 | 31920 | 31733 | 31909 | 31910 |  |  |  |  |
|  |  |  |  |  |  |  |  |  |
|  |  |  |  |  |  |  |  |  |
|  |  |  |  |  |  |  |  |  |
| 31190 | 31191 | 31251 | 31256 | 32291 | 32280 | 30352 | 30353 | 32284 |
|  |  |  |  |  |  |  |  |  |
|  |  |  |  |  |  |  |  |  |
|  |  |  |  |  |  |  |  |  |
|  |  |  |  |  |  |  |  |  |
|  |  |  |  |  |  |  |  |  |

| 30431 | 30428 | 30433 | 30426 | 30432 | 30434 | 30424 | 31908 |  |
| --- | --- | --- | --- | --- | --- | --- | --- | --- |
| 27443 | 27467 | 27497 | 27578 | 27612 | 27685 | 27690 | 27701 | 27794 |
|  |  |  |  |  |  |  |  |  |
|  |  |  |  |  |  |  |  |  |
|  |  |  |  |  |  |  |  |  |
|  |  |  |  |  |  |  |  |  |
| 27340 | 27350 | 27388 | 27402 | 27411 | 27419 | 27421 |  |  |
| 31711 | 32285 | 32286 | 32287 | 32288 | 32289 | 32290 | 32454 | 30394 |
|  |  |  |  |  |  |  |  |  |
|  |  |  |  |  |  |  |  |  |
|  |  |  |  |  |  |  |  |  |
|  |  |  |  |  |  |  |  |  |
|  |  |  |  |  |  |  |  |  |
|  |  |  |  |  |  |  |  |  |
|  |  |  |  |  |  |  |  |  |
|  |  |  |  |  |  |  |  |  |
|  |  |  |  |  |  |  |  |  |
| 35316 | 35326 | 35157 | 35166 |  |  |  |  |  |
|  |  |  |  |  |  |  |  |  |
| 30073 | 30074 | 30075 | 30362 | 30363 |  |  |  |  |
|  |  |  |  |  |  |  |  |  |
|  |  |  |  |  |  |  |  |  |
|  |  |  |  |  |  |  |  |  |
|  |  |  |  |  |  |  |  |  |
|  |  |  |  |  |  |  |  |  |
|  |  |  |  |  |  |  |  |  |
|  |  |  |  |  |  |  |  |  |
| 27703 | 27803 |  |  |  |  |  |  |  |
| 34893 | 35004 | 34894 | 34895 | 35005 | 35068 | 35069 | 35070 | 35071 |
| 30357 | 30359 | 30361 | 30360 | 30358 | 30356 |  |  |  |

|  |
| --- |
|  |
|  |
|  |
|  |
|  |
|  |
|  |
|  |
|  |
|  |
|  |
|  |
|  |
|  |
|  |
|  |
| 27896 |
|  |

|  |
| --- |
|  |
|  |
|  |
|  |
|  |
|  |
|  |
|  |
|  |
|  |
|  |
|  |
|  |
|  |
|  |
|  |
|  |
|  |
|  |
|  |
|  |
|  |
|  |
|  |
|  |
|  |
|  |
|  |
|  |
|  |
|  |
|  |
| 32281 |
|  |

|  |  |  |  |  |  |  |  |  |
| --- | --- | --- | --- | --- | --- | --- | --- | --- |
| 27795 | 27802 |  |  |  |  |  |  |  |
|  |  |  |  |  |  |  |  |  |
|  |  |  |  |  |  |  |  |  |
|  |  |  |  |  |  |  |  |  |
|  |  |  |  |  |  |  |  |  |
|  |  |  |  |  |  |  |  |  |
| 30387 | 30393 | 30388 | 30396 | 30395 | 30391 | 30386 | 30390 | 30392 |
|  |  |  |  |  |  |  |  |  |
|  |  |  |  |  |  |  |  |  |
|  |  |  |  |  |  |  |  |  |
|  |  |  |  |  |  |  |  |  |
|  |  |  |  |  |  |  |  |  |
|  |  |  |  |  |  |  |  |  |
|  |  |  |  |  |  |  |  |  |
|  |  |  |  |  |  |  |  |  |
|  |  |  |  |  |  |  |  |  |
|  |  |  |  |  |  |  |  |  |
|  |  |  |  |  |  |  |  |  |
|  |  |  |  |  |  |  |  |  |
|  |  |  |  |  |  |  |  |  |
|  |  |  |  |  |  |  |  |  |
|  |  |  |  |  |  |  |  |  |
|  |  |  |  |  |  |  |  |  |
|  |  |  |  |  |  |  |  |  |
|  |  |  |  |  |  |  |  |  |
|  |  |  |  |  |  |  |  |  |
|  |  |  |  |  |  |  |  |  |
|  |  |  |  |  |  |  |  |  |
|  |  |  |  |  |  |  |  |  |
