# Supplementary material for: Prediction of transcription factors associated with DNA demethylation during human cellular development
Source: Chromosome Res. 2022 Feb 10;30(1):109–21. doi: 10.1007/s10577-022-09685-6 (PMC8942926; doi:10.1007/s10577-022-09685-6)
Supplement: Supplementary file 7 — Supplementary file7 (DOCX 32 KB) [file 10577_2022_9685_MOESM7_ESM.docx]

Name of data adipocyte..knih adult_endothelial_progenitor_cell..blueprint alternatively_activated_macrophage..blueprint band_form_neutrophil..blueprint

b_cell..ceehrc b_cell_from_peripheral_blood..blueprint brain..ceehrc brain_hippocampus_middle..nih_roadmap

cd14_positive cd16_negative_classical_monocyte..blueprint cd19positive_cells..ceehrc

cd34_negative cd41_positive cd42_positive_megakaryocyte_cell..blueprint cd38_negative_naive_b_cell..blueprint

cd3_negative cd4_positive cd8_positive double_positive_thymocyte..blueprint cd4_naive..ceehrc

cd4_positive_alpha_beta_memory_t_cell..deep cd4_positive alpha_beta_t_cell..blueprint cd4_positive alpha_beta_thymocyte..blueprint cd4_positive_helper_t_cell..ceehrc cd4positive_t_cell_from_peripheral_blood..blueprint cd8_positive alpha_beta_t_cell..blueprint cd8_positive alpha_beta_thymocyte..blueprint cd8positive_t_cell_from_peripheral_blood..blueprint

central_memory_cd4_positive alpha_beta_t_cell..blueprint central_memory_cd4_positive alpha_beta_t_cell..deep central_memory_cd8_positive alpha_beta_t_cell..blueprint class_switched_memory_b_cell..blueprint common_lymphoid_progenitor_from_peripheral_blood..blueprint common_myeloid_progenitor_from_peripheral_blood..blueprint conventional_dendritic_cell..blueprint cytotoxic_cd56_dim_natural_killer_cell..blueprint early_neuron..knih

effector_memory_cd4_positive alpha_beta_t_cell..blueprint effector_memory_cd8_positive alpha_beta_t_cell..blueprint effector_memory_cd8_positive alpha_beta_t_cell terminally_differentiated..blueprint endothelial_cell_of_umbilical_vein_(proliferating)..blueprint endothelial_cell_of_umbilical_vein_(resting)..blueprint

eosinophil..ceehrc erythroblast..blueprint

fetal_intestine..nih_roadmap germinal_center_b_cell..blueprint granulocyte_macrophage_progenitor_from_peripheral_blood..blueprint hapatocyte..crest

heart..nih_roadmap hematopoietic_multipotent_progenitor_cell..blueprint hematopoietic_stem_cell..ceehrc hematopoietic_stem_cell_from_bone_marrow..blueprint hematopoietic_stem_cell_from_cord_blood..blueprint hematopoietic_stem_cell_from_fetal_liver..blueprint hematopoietic_stem_cell_from_peripheral_blood..blueprint hepatocyte..deep hesc_derived_cd184positive_endoderm_cultured_cells..nih_roadmap hesc_derived_cd56positive_ectoderm_cultured_cells..nih_roadmap hesc_derived_cd56positive_mesoderm_cultured_cells..nih_roadmap immature_conventional_dendritic_cell..blueprint immature_lymphoid_progenitor_0_from_peripheral_blood..blueprint immature_lymphoid_progenitor_1_from_peripheral_blood..blueprint immature_lymphoid_progenitor_2_from_peripheral_blood..blueprint immature_lymphoid_progenitor_3_from_peripheral_blood..blueprint inflammatory_macrophage..blueprint

ips..ceehrc ips..knih islet_cell..knih kidney..ceehrc

large_intestine_colon_ascending_(right)..ceehrc large_intestine_colon..ceehrc large_intestine_colon_rectosigmoid..ceehrc liver..crest

macrophage..blueprint macrophage..deep mature_conventional_dendritic_cell..blueprint mature_eosinophil..blueprint mature_neutrophil..blueprint

megakaryocyte_erythrocyte_progenitor_from_peripheral_blood..blueprint megakaryocyte_from_bone_marrow..blueprint

memory_b_cell..blueprint mesangial..knih mesenchymal_stem_cell_of_the_bone_marrow..blueprint

monocyte..blueprint monocyte..ceehrc monocyte..deep

monocyte_from_peripheral_blood..blueprint multipotent_progenitor_from_cord_blood..blueprint multipotent_progenitor_from_peripheral_blood..blueprint muscle..ceehrc

myeloid_cell..blueprint naive_b_cell..blueprint naive_cd4_positive_t_cell..deep natural_killer_cell_from_peripheral_blood..blueprint neural_progenitor_cell..knih

neurosphere_cultured_cells_cortex_derived..nih_roadmap neurosphere_cultured_cells_ganglionic_eminence_derived..nih_roadmap neutrophil_from_peripheral_blood..blueprint neutrophilic_metamyelocyte..blueprint

neutrophilic_myelocyte..blueprint normal_human_colon_absorptive_epithelial_cells..crest osteoclast..blueprint

plasma_cell..blueprint podocyte..knih preadipocyte..knih precursor_b_cell..blueprint

precursor_lymphocyte_of_b_lineage..blueprint regulatory_t_cell..blueprint segmented_neutrophil_of_bone_marrow..blueprint sigmoid_colon..nih_roadmap

t_cell..ceehrc thyroid..ceehrc venous_blood..blueprint

**Average**

| Methylated bins | Demethylated bins | Total number | % demethylation |
| --- | --- | --- | --- |
| 10052 | 230202 | 240254 | 95.82 |
| 12090 | 351649 | 363739 | 96.68 |
| 23923 | 40415 | 64338 | 62.82 |
| 20907 | 90696 | 111603 | 81.27 |
| 17459 | 401188 | 418647 | 95.83 |
| 25673 | 87768 | 113441 | 77.37 |
| 21168 | 63664 | 84832 | 75.05 |
| 13701 | 92780 | 106481 | 87.13 |
| 25461 | 64682 | 90143 | 71.75 |
| 11186 | 556712 | 567898 | 98.03 |
| 21585 | 23277 | 44862 | 51.89 |
| 29547 | 27114 | 56661 | 47.85 |
| 26192 | 78256 | 104448 | 74.92 |
| 26664 | 74253 | 100917 | 73.58 |
| 12995 | 83780 | 96775 | 86.57 |
| 30779 | 33155 | 63934 | 51.86 |
| 25217 | 86729 | 111946 | 77.47 |
| 22930 | 85054 | 107984 | 78.77 |
| 27304 | 74317 | 101621 | 73.13 |
| 32990 | 17189 | 50179 | 34.26 |
| 26491 | 83237 | 109728 | 75.86 |
| 26688 | 71094 | 97782 | 72.71 |
| 21700 | 68869 | 90569 | 76.04 |
| 17741 | 104233 | 121974 | 85.46 |
| 31771 | 27891 | 59662 | 46.75 |
| 19226 | 125949 | 145175 | 86.76 |
| 26193 | 73378 | 99571 | 73.69 |
| 24630 | 82138 | 106768 | 76.93 |
| 21043 | 63586 | 84629 | 75.14 |
| 25819 | 41099 | 66918 | 61.42 |
| 2451 | 2619 | 5070 | 51.66 |
| 20512 | 104070 | 124582 | 83.54 |
| 25789 | 92301 | 118090 | 78.16 |
| 23123 | 116495 | 139618 | 83.44 |
| 14889 | 41249 | 56138 | 73.48 |
| 18158 | 14528 | 32686 | 44.45 |
| 18832 | 134008 | 152840 | 87.68 |
| 21524 | 13248 | 34772 | 38.10 |

| 10193 | 93608 | 103801 | 90.18 |
| --- | --- | --- | --- |
| 20439 | 94223 | 114662 | 82.17 |
| 23801 | 83428 | 107229 | 77.80 |
| 18475 | 224195 | 242670 | 92.39 |
| 10705 | 82122 | 92827 | 88.47 |
| 27443 | 40040 | 67483 | 59.33 |
| 22837 | 78753 | 101590 | 77.52 |
| 24265 | 79790 | 104055 | 76.68 |
| 20499 | 67601 | 88100 | 76.73 |
| 20786 | 88301 | 109087 | 80.95 |
| 22145 | 112775 | 134920 | 83.59 |
| 17952 | 231724 | 249676 | 92.81 |
| 1296 | 802 | 2098 | 38.23 |
| 317 | 1706 | 2023 | 84.33 |
| 884 | 552 | 1436 | 38.44 |
| 24045 | 142961 | 167006 | 85.60 |
| 26040 | 71456 | 97496 | 73.29 |
| 26277 | 73098 | 99375 | 73.56 |
| 25951 | 73399 | 99350 | 73.88 |
| 25745 | 75201 | 100946 | 74.50 |
| 24611 | 44514 | 69125 | 64.40 |
| 1295 | 1021 | 2316 | 44.08 |
| 605 | 1357 | 1962 | 69.16 |
| 12270 | 149385 | 161655 | 92.41 |
| 9954 | 81067 | 91021 | 89.06 |
| 16660 | 177940 | 194600 | 91.44 |
| 14621 | 143185 | 157806 | 90.73 |
| 16182 | 164075 | 180257 | 91.02 |
| 20542 | 219714 | 240256 | 91.45 |
| 25425 | 35777 | 61202 | 58.46 |
| 25437 | 140955 | 166392 | 84.71 |
| 24002 | 142605 | 166607 | 85.59 |
| 21350 | 130086 | 151436 | 85.90 |
| 23928 | 74022 | 97950 | 75.57 |
| 23870 | 82199 | 106069 | 77.50 |
| 18091 | 165792 | 183883 | 90.16 |
| 22279 | 78003 | 100282 | 77.78 |
| 8825 | 53252 | 62077 | 85.78 |
| 13303 | 649189 | 662492 | 97.99 |

| 23481 | 119838 | 143319 | 83.62 |
| --- | --- | --- | --- |
| 20721 | 140126 | 160847 | 87.12 |
| 23995 | 136627 | 160622 | 85.06 |
| 21848 | 119765 | 141613 | 84.57 |
| 21828 | 66497 | 88325 | 75.29 |
| 21859 | 76830 | 98689 | 77.85 |
| 10666 | 174206 | 184872 | 94.23 |
| 31174 | 64728 | 95902 | 67.49 |
| 24362 | 76992 | 101354 | 75.96 |
| 12584 | 27473 | 40057 | 68.58 |
| 23794 | 95133 | 118927 | 79.99 |
| 1251 | 1031 | 2282 | 45.18 |
| 9770 | 111290 | 121060 | 91.93 |
| 8454 | 46557 | 55011 | 84.63 |
| 21204 | 131446 | 152650 | 86.11 |
| 20695 | 105615 | 126310 | 83.62 |
| 21523 | 101544 | 123067 | 82.51 |
| 18265 | 233585 | 251850 | 92.75 |
| 23588 | 136469 | 160057 | 85.26 |
| 11886 | 320898 | 332784 | 96.43 |
| 12078 | 106882 | 118960 | 89.85 |
| 11723 | 148064 | 159787 | 92.66 |
| 22749 | 120741 | 143490 | 84.15 |
| 31929 | 28805 | 60734 | 47.43 |
| 21902 | 76842 | 98744 | 77.82 |
| 20700 | 81651 | 102351 | 79.78 |
| 14374 | 68986 | 83360 | 82.76 |
| 20277 | 91111 | 111388 | 81.80 |
| 25345 | 89510 | 114855 | 77.93 |
| 14085 | 341647 | 355732 | 96.04 |
| **19494** | **107604** | **127098** | **77.25** |

ips (induced pluripotent stem cells)

endoderm-, ectoderm- and mesoderm-cultured cells, neuronal progenitor cells and ea

rly neuron
