# Supplementary material for: Prediction of transcription factors associated with DNA demethylation during human cellular development
Source: Chromosome Res. 2022 Feb 10;30(1):109–21. doi: 10.1007/s10577-022-09685-6 (PMC8942926; doi:10.1007/s10577-022-09685-6)
Supplement: Supplementary file 9 — Supplementary file9 (DOCX 19 KB) [file 10577_2022_9685_MOESM9_ESM.docx]

Gene Motif ID Motif source Poisson.Test.P.value

| ASCL2 | ASCL2_1.motif | IMAGE | 6.70E-224 |
| --- | --- | --- | --- |
| CDX2 | CDX2_1.motif | IMAGE | 0.031196071 |
| CEBPE | CEBPE_1.motif | IMAGE | 7.42E-23 |
| EBF1 | EBF1_1.motif | IMAGE | 0.416127744 |
| EHF | EHF_1.motif | IMAGE | 0.212465866 |
| EHF | EHF_2.motif | IMAGE | 0.269633794 |
| ELF1 | ELF1_1.motif | IMAGE | 0.264241118 |
| ELF2 | ELF2_1.motif | IMAGE | 0.412876746 |
| ELF3 | ELF3_1.motif | IMAGE | 0.182992341 |
| ELF4 | ELF4_1.motif | IMAGE | 0.60611648 |
| ELK4 | ELK4_1.motif | IMAGE | 0.996208113 |
| ETS1 | ETS1_1.motif | IMAGE | 6.50E-262 |
| ETS1 | ETS1_2.motif | IMAGE | 0.264241118 |
| ETS2 | ETS2_1.motif | IMAGE | 1.02E-67 |
| ETV1 | ETV1_1.motif | IMAGE | 0.301989849 |
| ETV4 | ETV4_1.motif | IMAGE | 0.030507638 |
| ETV6 | ETV6_1.motif | IMAGE | 0.26691022 |
| ETV7 | ETV7_1.motif | IMAGE | 0.287218606 |
| FEV | FEV_1.motif | IMAGE | 7.01E-27 |
| FOSB | FOSB_1.motif | IMAGE | 0 |
| FOSL2 | FOSL2_1.motif | IMAGE | 0 |
| FOXA1 | FOXA1_1.motif | IMAGE | 1.27E-287 |
| FOXA2 | FOXA2_1.motif | IMAGE | 3.24E-24 |
| FOXP1 | FOXP1_1.motif | IMAGE | 0.382958142 |
| GATA3 | GATA3_2.motif | IMAGE | 1.56E-271 |
| GATA6 | GATA6_1.motif | IMAGE | 0 |
| HOXA9 | HOXA9_1.motif | IMAGE | 0.076059841 |
| HOXA9 | HOXA9_2.motif | IMAGE | 0.295241472 |
| HOXC9 | HOXC9_1.motif | IMAGE | 0.056305336 |
| IKZF3 | IKZF3_1.motif | IMAGE | 0.91005878 |
| IRF8 | IRF8_1.motif | IMAGE | 0.008324115 |
| LEF1 | LEF1_1.motif | IMAGE | 7.01E-27 |
| MSC | MSC_1.motif | IMAGE | 7.68E-34 |
| MYF6 | MYF6_1.motif | IMAGE | 1.09E-189 |
| MYOG | MYOG_1.motif | IMAGE | 0 |
| NEUROD1 | NEUROD1_1.motif | IMAGE | 0 |

| NFE2 | NFE2_1.motif | IMAGE | 0.008324115 |
| --- | --- | --- | --- |
| NFE2 | NFE2_2.motif | IMAGE | 0.285666073 |
| NFE2L2 | NFE2L2_1.motif | IMAGE | 0.00102492 |
| NFIB | NFIB_1.motif | IMAGE | 4.17E-63 |
| NFIX | NFIX_1.motif | IMAGE | 1.80E-35 |
| NFIX | NFIX_2.motif | IMAGE | 0 |
| NR1H4 | NR1H4_1.motif | IMAGE | 8.58E-17 |
| NR2F6 | NR2F6_3.motif | IMAGE | 0.264241118 |
| NR4A1 | NR4A1_1.motif | IMAGE | 1.24504542751994e-318 |
| NR5A1 | NR5A1_1.motif | IMAGE | 5.71E-99 |
| NRL | NRL_1.motif | IMAGE | 9.14E-30 |
| PAX8 | PAX8_1.motif | IMAGE | 2.88E-23 |
| PAX8 | PAX8_2.motif | IMAGE | 1.01E-58 |
| PTF1A | PTF1A_1.motif | IMAGE | 0.01664823 |
| RUNX2 | RUNX2_1.motif | IMAGE | 0 |
| SPI1 | SPI1_1.motif | IMAGE | 2.63E-34 |
| SPI1 | SPI1_2.motif | IMAGE | 2.17E-76 |
| SPIB | SPIB_1.motif | IMAGE | 0 |
| SPIB | SPIB_2.motif | IMAGE | 0 |
| TCF21 | TCF21_1.motif | IMAGE | 2.50E-28 |
| TFAP4 | TFAP4_2.motif | IMAGE | 0.309053939 |

| peak.IQR.ratio Judgement | | | Demethyl | No. of Pro  Methyl |
| --- | --- | --- | --- | --- |
|  | 21.21189099 | DNA demethylating | 1526 | 140 |
| NA |  | Non-DNA demethylating | 390 | 1316 |
|  | 8.683336575 | DNA demethylating | 2815 | 1402 |
| NA |  | Non-DNA demethylating | 196 | 279 |
| NA |  | Non-DNA demethylating | 42 | 54 |
| NA |  | Non-DNA demethylating | 42 | 54 |
| NA |  | Non-DNA demethylating | 16 | 19 |
| NA |  | Non-DNA demethylating | 210 | 189 |
| NA |  | Non-DNA demethylating | 613 | 114 |
| NA |  | Non-DNA demethylating | 448 | 563 |
| NA |  | Non-DNA demethylating | 228 | 55 |
|  | 37.85491439 | DNA demethylating | 1015 | 376 |
| NA |  | Non-DNA demethylating | 1015 | 376 |
|  | 12.67881184 | DNA demethylating | 423 | 97 |
| NA |  | Non-DNA demethylating | 24 | 42 |
| NA |  | DNA demethylating | 204 | 272 |
| NA |  | Non-DNA demethylating | 228 | 297 |
| NA |  | Non-DNA demethylating | 227 | 107 |
|  | 9.680936345 | DNA demethylating | 138 | 131 |
|  | 56.37064853 | DNA demethylating | 1984 | 141 |
|  | 65.82852921 | DNA demethylating | 2449 | 206 |
|  | 24.8427066 | DNA demethylating | 2471 | 892 |
|  | 7.634397662 | DNA demethylating | 1787 | 1863 |
| NA |  | Non-DNA demethylating | 27 | 12 |
|  | 14.61944534 | DNA demethylating | 1011 | 258 |
|  | 24.24254258 | DNA demethylating | 2651 | 2432 |
| NA |  | Non-DNA demethylating | 74 | 274 |
| NA |  | Non-DNA demethylating | 74 | 274 |
| NA |  | Non-DNA demethylating | 397 | 429 |
| NA |  | Non-DNA demethylating | 134 | 595 |
| NA |  | Non-DNA demethylating | 3957 | 9617 |
|  | 10.9028019 | DNA demethylating | 1942 | 234 |
|  | 11.22095553 | DNA demethylating | 527 | 38 |
|  | 21.26413983 | DNA demethylating | 1839 | 54 |
|  | 41.73235845 | DNA demethylating | 1970 | 373 |
|  | 52.3801154 | DNA demethylating | 3166 | 602 |

| NA |  | Non-DNA demethylating | 56 | 27 |
| --- | --- | --- | --- | --- |
| NA |  | Non-DNA demethylating | 56 | 27 |
| NA |  | Non-DNA demethylating | 229 | 120 |
|  | 9.818462069 | DNA demethylating | 5930 | 1338 |
|  | 6.926406765 | DNA demethylating | 4563 | 817 |
|  | 30.38936125 | DNA demethylating | 4563 | 817 |
|  | 18.82176734 | DNA demethylating | 570 | 117 |
| NA |  | Non-DNA demethylating | 65 | 73 |
|  | 40.43236152 | DNA demethylating | 1613 | 46 |
|  | 13.43472612 | DNA demethylating | 378 | 251 |
|  | 8.324876686 | DNA demethylating | 313 | 507 |
|  | 7.896680874 | DNA demethylating | 3727 | 724 |
|  | 14.44035399 | DNA demethylating | 3727 | 724 |
| NA |  | Non-DNA demethylating | 1648 | 94 |
|  | 55.78982248 | DNA demethylating | 4828 | 747 |
|  | 28.35368949 | DNA demethylating | 4376 | 1065 |
|  | 26.28618054 | DNA demethylating | 4376 | 1065 |
|  | 24.36415603 | DNA demethylating | 2554 | 133 |
|  | 63.43052256 | DNA demethylating | 2554 | 133 |
|  | 10.0688498 | DNA demethylating | 255 | 413 |
| NA |  | Non-DNA demethylating | 17 | 16 |

be

(Demethyl/Methyl)

10.9

0.296352584

2.007845934

0.702508961

0.777777778

0.777777778

0.842105263

1.111111111

5.377192982

0.795737123

4.145454545

2.699468085

2.699468085

4.360824742

0.571428571

0.75

0.767676768

2.121495327

1.053435115

14.07092199

11.88834951

2.770179372

0.959205582

2.25

3.918604651

1.090049342

0.270072993

0.270072993

0.925407925

0.225210084

0.411458875

8.299145299

13.86842105

34.05555556

5.28150134

5.259136213

2.074074074

2.074074074

1.908333333

4.431988042

5.585067319

5.585067319

4.871794872

0.890410959

35.06521739

1.505976096

0.617357002

5.147790055

5.147790055

17.53191489

6.463186078

4.108920188

4.108920188

19.20300752

19.20300752

0.617433414

1.0625
