# Supplementary material for: Prediction of transcription factors associated with DNA demethylation during human cellular development
Source: Chromosome Res. 2022 Feb 10;30(1):109–21. doi: 10.1007/s10577-022-09685-6 (PMC8942926; doi:10.1007/s10577-022-09685-6)
Supplement: Supplementary file 10 — Supplementary file10 (DOCX 20 KB) [file 10577_2022_9685_MOESM10_ESM.docx]

ASCL2

| ASCL2 |  | CEBPE | ETS1 |  | ETS2 |  | ETV4 |  | FEV |  | FOSB |  |
| --- | --- | --- | --- | --- | --- | --- | --- | --- | --- | --- | --- | --- |
| 100 | | 1.77 |  | 1.25 |  | 0.85 |  | 0.66 |  | 0.59 |  | 3.54 |
| 0.96 | | 100 |  | 1.07 |  | 1.03 |  | 0.64 |  | 1.53 |  | 1.95 |
| 1.87 | | 2.96 |  | 100 | 32.81 | | 12.61 | |  | 7.78 |  | 1.77 |
| 3.07 | | 6.86 | 78.72 | |  | 100 | 25.06 | |  | 15.6 |  | 4.73 |
|  | 4.9 | 8.82 | 62.75 | | 51.96 | |  | 100 |  | 22.55 |  | 6.37 |
| 6.52 | | 31.16 | 57.25 | | 47.83 | | 33.33 | |  | 100 | 10.87 | |
| 2.72 | | 2.77 |  | 0.91 |  | 1.01 |  | 0.66 |  | 0.76 |  | 100 |
| 2.12 | | 3.43 |  | 0.86 |  | 0.9 |  | 0.53 |  | 1.67 | 49.45 | |
| 1.46 | | 3.44 |  | 1.3 |  | 0.77 |  | 0.77 |  | 0.69 |  | 0.85 |
| 1.01 | | 4.42 |  | 1.68 |  | 1.23 |  | 1.06 |  | 2.01 |  | 1.06 |
| 2.18 | | 4.45 |  | 2.37 |  | 2.57 |  | 2.08 |  | 1.88 |  | 3.26 |
| 1.09 | | 2.53 |  | 1.4 |  | 1.28 |  | 0.94 |  | 0.72 |  | 1.7 |
| 0.88 | | 4.58 |  | 1.29 |  | 0.98 |  | 0.88 |  | 2.16 |  | 2.94 |
| 41.94 | | 3.04 |  | 4.36 |  | 3.61 |  | 2.66 |  | 2.66 |  | 3.42 |
| 38.01 | | 1.79 |  | 1.31 |  | 0.98 |  | 0.76 |  | 0.82 |  | 2.83 |
| 30.91 | | 2.18 |  | 1.12 |  | 0.91 |  | 0.71 |  | 0.81 |  | 1.88 |
| D1 12.1 | | 1.36 |  | 1.11 |  | 0.73 |  | 0.51 |  | 0.44 |  | 2.21 |
|  | 1.7 | 1.96 |  | 0.69 |  | 0.49 |  | 0.32 |  | 0.83 |  | 1.28 |
| 1.93 | | 1.62 |  | 0.94 |  | 0.77 |  | 0.55 |  | 0.48 |  | 2.17 |
| 2.46 | | 3.68 |  | 2.63 |  | 2.81 |  | 2.63 |  | 2.28 |  | 4.74 |
| 1.67 | | 3.16 |  | 1.98 |  | 1.49 |  | 1.12 |  | 1.12 |  | 2.79 |
| 2.38 | | 11.38 |  | 3.44 |  | 2.91 |  | 2.65 |  | 11.38 |  | 3.17 |
| 5.75 | | 4.47 |  | 3.83 |  | 3.19 |  | 3.19 |  | 3.83 |  | 4.47 |
| 1.45 | | 1.61 |  | 0.78 |  | 0.51 |  | 0.48 |  | 0.51 |  | 1.26 |
| 1.12 | | 1.55 |  | 1.04 |  | 0.83 |  | 0.7 |  | 0.5 |  | 1.97 |
| 0.87 | | 1.49 |  | 6.35 |  | 2.47 |  | 0.96 |  | 0.87 |  | 1.05 |
|  | 1.1 | 1.92 |  | 7.17 |  | 3.09 |  | 1.49 |  | 1.25 |  | 1.29 |
| 27.84 | | 6.67 |  | 7.06 |  | 5.88 |  | 6.27 |  | 5.88 |  | 6.67 |

CEBPE ETS1

ETS2

ETV4

FEV FOSB FOSL2

FOXA1

FOXA2

GATA3

GATA6

LEF1

MSC MYF6

MYOG NEURO NFIB NFIX NR1H4

NR4A1

NR5A1

NRL PAX8

RUNX2

SPI1

SPIB TCF21

| FOSL2 |  | FOXA1 |  | FOXA2 |  | GATA3 |  | GATA6 |  | LEF1 |  | MSC |  | MYF6 |  |
| --- | --- | --- | --- | --- | --- | --- | --- | --- | --- | --- | --- | --- | --- | --- | --- |
| 3.41 | | 2.36 | | 1.18 | | 1.44 | |  | 1.9 |  | 1.11 | 14.48 | | 45.81 | |
| 2.98 | | 3.02 | | 2.81 | |  | 1.6 | 2.38 | |  | 3.16 |  | 0.57 | 1.17 | |
| 2.07 | | 3.15 | | 2.96 | | 2.36 | | 3.65 | |  | 2.46 |  | 2.27 | 2.36 | |
|  | 5.2 | 4.49 | |  | 5.2 | 6.15 | | 8.04 | |  | 4.49 |  | 4.49 | 4.26 | |
| 6.37 | | 9.31 | | 9.31 | | 10.29 | | 12.25 | |  | 8.33 |  | 6.86 | 6.86 | |
| 29.71 | | 12.32 | | 26.09 | | 13.77 | | 13.77 | | 30.43 | | 10.14 | | 10.87 | |
| 61.04 | | 1.06 | | 0.96 | | 1.66 | | 2.27 | |  | 2.87 |  | 0.91 | 2.62 | |
| 100 | | 1.39 | | 1.59 | | 1.55 | | 2.08 | |  | 3.96 |  | 0.78 |  | 2 |
| 1.38 | | 100 | | 54.63 | | 1.13 | | 2.19 | |  | 1.62 |  | 0.73 | 1.82 | |
| 2.18 | | 75.55 | | 100 | |  | 1.4 | 2.46 | |  | 3.58 |  | 0.84 | 1.57 | |
| 3.76 | | 2.77 | | 2.47 | | 100 | | 33.33 | |  | 5.04 |  | 2.47 | 2.67 | |
| 1.92 | | 2.04 | | 1.66 | | 12.71 | | 100 | |  | 2.53 |  | 0.75 | 1.43 | |
| 4.99 | | 2.06 | |  | 3.3 | 2.63 | | 3.45 | |  | 100 |  | 1.03 | 1.49 | |
| 3.61 | | 3.42 | | 2.85 | | 4.74 | |  | 3.8 |  | 3.8 |  | 100 | 42.88 | |
| 2.66 | | 2.45 | | 1.52 | | 1.47 | | 2.07 | |  | 1.58 | 12.29 | |  | 100 |
| 1.93 | | 2.69 | | 1.42 | | 1.73 | | 2.23 | |  | 1.42 | 10.36 | | 55.94 | |
|  | 2.4 | 1.61 | | 0.79 | | 1.58 | | 2.08 | |  | 1.07 |  | 5.94 | 12.95 | |
| 2.11 | | 0.96 | | 0.93 | | 0.86 | | 1.48 | |  | 1.67 |  | 1.13 | 1.77 | |
| 2.45 | | 1.38 | | 0.99 | | 1.31 | | 2.13 | |  | 1.31 |  | 1.18 | 1.93 | |
| 3.51 | | 2.81 | | 2.63 | | 2.11 | | 3.33 | |  | 2.46 |  | 2.28 | 3.68 | |
| 2.91 | | 2.85 | | 2.17 | | 2.23 | | 2.98 | |  | 3.72 |  | 1.55 | 2.67 | |
| 13.23 | | 3.17 | | 8.47 | |  | 4.5 | 3.97 | | 12.43 | |  | 3.44 | 3.17 | |
| 6.39 | | 5.43 | | 6.07 | | 6.39 | | 4.15 | |  | 4.15 |  | 5.43 | 6.39 | |
| 1.42 | | 1.29 | |  | 0.8 | 0.97 | | 1.23 | |  | 1.29 |  | 1.1 | 1.99 | |
| 1.86 | | 1.14 | | 0.79 | | 0.95 | | 1.41 | |  | 1.41 |  | 0.62 | 1.35 | |
| 1.12 | | 1.12 | | 0.69 | | 0.96 | | 1.69 | |  | 1.62 |  | 0.57 | 0.94 | |
| 1.53 | |  | 1.8 | 1.14 | | 1.49 | | 2.66 | |  | 2.23 |  | 0.9 | 1.21 | |
| 7.06 | | 6.67 | | 7.06 | | 7.84 | | 8.24 | |  | 4.71 | 53.33 | | 33.73 | |

| MYOG | NEUROD1 | NFIB |  | NFIX |  | NR1H4 | NR4A1 | NR5A1 |  | NRL |  |
| --- | --- | --- | --- | --- | --- | --- | --- | --- | --- | --- | --- |
| 39.91 | 25.1 |  | 6.62 |  | 5.77 | 0.92 | 1.77 | 0.59 | |  | 1.18 |
| 1.53 | 1.53 |  | 4.12 |  | 2.63 | 0.75 | 1.81 | 1.53 | |  | 0.5 |
| 2.17 | 3.45 |  | 4.04 |  | 4.24 | 1.48 | 3.15 | 1.28 | |  | 1.18 |
| 4.26 | 5.44 |  | 6.86 |  | 8.27 | 3.78 | 5.67 |  | 2.6 |  | 2.36 |
| 6.86 | 7.84 |  | 9.31 | 12.25 | | 7.35 | 8.82 |  | 4.9 |  | 4.9 |
| 11.59 | 10.14 | 35.51 | | 15.94 | | 9.42 | 13.04 | 31.16 | |  | 8.7 |
| 1.86 | 3.53 |  | 3.83 |  | 4.99 | 1.36 | 2.27 |  | 0.6 |  | 0.71 |
| 1.55 | 3.1 |  | 5.1 |  | 4.57 | 0.82 | 1.92 | 2.04 | |  | 0.82 |
| 2.14 | 2.06 |  | 2.31 |  | 2.55 | 0.65 | 1.86 | 0.49 | |  | 0.69 |
| 1.57 | 1.4 |  | 3.08 |  | 2.52 | 0.84 | 1.96 | 1.79 | |  | 1.06 |
| 3.36 | 4.95 |  | 5.04 |  | 5.93 | 1.19 | 3.56 | 1.68 | |  | 1.98 |
| 1.66 | 2.49 |  | 3.32 |  | 3.66 | 0.72 | 1.81 | 0.57 | |  | 0.49 |
| 1.44 | 1.75 |  | 5.1 |  | 3.09 | 0.72 | 3.09 | 2.42 | |  | 0.67 |
| 38.71 | 35.67 | 12.71 | | 10.25 | | 2.47 | 4.74 | 2.47 | |  | 3.23 |
| 59.92 | 22.29 |  | 5.71 |  | 4.79 | 1.14 | 2.34 | 0.65 | |  | 1.09 |
| 100 | 23.45 |  | 5.74 |  | 5.28 | 1.22 | 2.08 | 0.81 | |  | 1.02 |
| 14.59 | 100 |  | 5.21 |  | 4.93 | 0.85 | 1.33 | 0.38 | |  | 0.69 |
| 1.91 | 2.78 |  | 100 | 49.85 | | 0.52 | 1.06 | 1.21 | |  | 0.56 |
| 2.28 | 3.42 | 64.78 | |  | 100 | 0.85 | 1.34 | 0.39 | |  | 0.64 |
| 4.21 | 4.74 |  | 5.44 |  | 6.84 | 100 | 5.79 | 2.28 | |  | 2.11 |
| 2.54 | 2.6 |  | 3.91 |  | 3.78 | 2.05 | 100 | 0.81 | |  | 1.18 |
| 4.23 | 3.17 | 19.05 | |  | 4.76 | 3.44 | 3.44 | 100 | |  | 3.97 |
| 6.39 | 7.03 | 10.54 | |  | 9.27 | 3.83 | 6.07 | 4.79 | |  | 100 |
| 1.82 | 2.39 |  | 3.25 |  | 3.19 | 0.99 | 1.26 | 0.46 | |  | 0.48 |
| 1.53 | 1.59 |  | 3.09 |  | 3.15 | 0.68 | 1.47 | 0.29 | |  | 0.41 |
| 1.01 | 1.55 |  | 2.35 |  | 2.1 | 0.57 | 1.35 | 0.41 | |  | 0.32 |
| 1.17 | 1.92 |  | 2.74 |  | 2.62 | 0.78 | 1.96 | 0.78 | |  | 0.67 |
| 33.33 | 37.65 | 10.98 | | 12.16 | | 5.1 | 8.24 | 5.88 | |  | 10.2 |

| PAX8 |  | RUNX2 | SPI1 |  | SPIB |  | TCF21 |  |
| --- | --- | --- | --- | --- | --- | --- | --- | --- |
|  | 3.54 | 3.54 |  | 2.49 |  | 1.83 | 4.65 | |
|  | 2.13 | 2.66 |  | 2.31 |  | 1.74 |  | 0.6 |
|  | 2.86 | 4.93 |  | 27.39 | 18.03 | | 1.77 | |
|  | 4.49 | 9.46 |  | 25.53 | 18.68 | | 3.55 | |
|  | 8.82 | 16.67 |  | 20.59 | 18.63 | | 7.84 | |
| 13.77 | | 17.39 |  | 27.54 | 23.19 | | 10.87 | |
|  | 2.37 | 4.79 |  | 2.32 |  | 1.66 | 0.86 | |
|  | 2.16 | 3.67 |  | 2 |  | 1.59 | 0.73 | |
|  | 1.94 | 2.23 |  | 1.98 |  | 1.86 | 0.69 | |
|  | 1.68 | 2.13 |  | 1.68 |  | 1.62 | 1.01 | |
|  | 3.56 | 4.55 |  | 4.15 |  | 3.76 | 1.98 | |
|  | 1.74 | 2.57 |  | 2.79 |  | 2.57 | 0.79 | |
|  | 2.47 | 3.5 |  | 3.66 |  | 2.94 | 0.62 | |
|  | 7.78 | 5.69 |  | 4.74 |  | 4.36 | 25.81 | |
|  | 4.02 | 3.53 |  | 2.23 |  | 1.69 | 4.68 | |
|  | 3.45 | 3.76 |  | 2.23 |  | 1.52 | 4.31 | |
|  | 2.81 | 2.43 |  | 2.15 |  | 1.55 | 3.03 | |
|  | 2.04 | 2.51 |  | 1.74 |  | 1.18 | 0.47 | |
|  | 2.61 | 3.33 |  | 2.02 |  | 1.47 | 0.68 | |
|  | 6.49 | 5.79 |  | 4.39 |  | 3.51 | 2.28 | |
|  | 2.91 | 4.4 |  | 3.66 |  | 3.1 |  | 1.3 |
|  | 4.5 | 3.7 |  | 4.76 |  | 5.29 | 3.97 | |
|  | 5.75 | 6.39 |  | 4.47 |  | 5.43 | 8.31 | |
|  | 100 | 3.09 |  | 1.58 |  | 1.34 | 0.75 | |
|  | 2.38 | 100 |  | 2.05 |  | 1.47 |  | 0.5 |
|  | 1.35 | 2.26 |  | 100 | 47.05 | | 0.41 | |
|  | 1.96 | 2.78 |  | 80.62 |  | 100 | 0.78 | |
| 10.98 | | 9.41 |  | 7.06 |  | 7.84 | 100 | |
