# Supplementary material for: Prediction of transcription factors associated with DNA demethylation during human cellular development
Source: Chromosome Res. 2022 Feb 10;30(1):109–21. doi: 10.1007/s10577-022-09685-6 (PMC8942926; doi:10.1007/s10577-022-09685-6)
Supplement: Supplementary file 11 — Supplementary file11 (DOCX 32 KB) [file 10577_2022_9685_MOESM11_ESM.docx]

**Supplementary information**

**Supplementary Figures**

**Figure S1. Clustering analysis of log-adjusted *p*-values for 427 TFBMs-TFs.** Embryonic stem cell methylome data was used as a reference.

**Figure S2.** ***In vitro* analysis of DNA-demethylation-promoting activity****.** Distribution of TFBM enrichment score for identified TFs with DNA-demethylation-promoting activity was drawn within ±5000 bp of demethylated CpG probes. *X*- and *Y*-axes represent distance from probe CpG position and enrichment score, respectively. Horizontal lines are enrichment score = 0.

**Figure S3.** **Motif number distribution at** **genomic regions demethylated by TFs with DNA-demethylation-promoting activity.** Number of target TF binding motifs within a ±200 bp from demethylated (blue) and randomly selected (gray) probes was calculated and represented by histogram. *P*-value of Kolmogorov–Smirnov test between demethylated regions and randomly selected regions was shown at bottom of each histogram

**Figure S4. Motif number distribution at genomic regions demethylated by TFs without** **DNA-demethylation-promoting activity.** Same analysis for Figure S3 was performed for target TFs without DNA-demethylation-promoting activity.

**Figure S5. Category of demethylated genomic regions for TFs with DNA-demethylation-promoting activity.** Proportion of enhancer, promoter, both (enhancer/promoter), and unannotated was shown. Promoters are defined as 1 kb upstream to 200 bp downstream from GenCode v19 transcription start sites (https://www.gencodegenes.org/human/release_19.html). Enhancer annotation was from FNATOM5 permissive enhancers phase 1 and 2 (https://fantom.gsc.riken.jp/5/datafiles/latest/extra/Enhancers/).

**Supplementary Tables**

**Table S1. IHEC data portal IDs used in the present study.**

**Table S2. Number of bins containing methylated or demethylated DNA in the IHEC data.** Name of the data, used in this study, represents cell or tissue name and the IHEC data contributor.

**Table S3. Results of the transcription factor binding motif (TFBM) enrichment analysis of regions of demethylated DNA.** Selected transcription factor binding motifs likely to be involved in DNA demethylation are shown. Columns A to D show the TFBM name, consensus sequence, corresponding TF, and type, respectively. Columns E to DG show the log-adjusted *p*-value for each TFBM. The multipliers of the *p*-values are shown. Columns DK to HM show the C-values. Columns HQ to LS show the concentration scores. Column LV shows average TF gene expression, and column LW shows TF gene expression in embryonic stem cells. Columns LZ and MA show the TF family and group, respectively. TF family names were taken from AnimalTFDB 3.0 (Nucleic Acids Res. 2019, 47(D1), D33-D38). NA means that no *p*-value, C-value, or concentration score was obtained.

**Table S4. Summary of the result of our *in vitro* DNA demethylation assay.**

**Table S5. Overlap percentage of DNA demethylated regions among transcription factors with DNA-demethylation-promoting activity**

**Table S6. FANTOM datasets for TF gene expression analysis.**

**Table S7. Gene expression profiles of transcription factors with DNA-demethylation-promoting activity in 23 representative human cells or tissues.** Transcription factor expression level is shown in units of tags per million.
