# Supplementary material for: Prediction of transcription factors associated with DNA demethylation during human cellular development
Source: Chromosome Res. 2022 Feb 10;30(1):109–21. doi: 10.1007/s10577-022-09685-6 (PMC8942926; doi:10.1007/s10577-022-09685-6)
Supplement: Supplementary file 8 — Supplementary file8 (DOCX 1994 KB) [file 10577_2022_9685_MOESM8_ESM.docx]

Motif	Sequence	geneid	type	adipocyte. adult_end

AHR_1.motif CCYCVRDBKCGYGTGASA	AHR	Direct	-181.27	-262.2

| ALX3_1.moti TAATTR | ALX3 | Direct | NA | NA |
| --- | --- | --- | --- | --- |
| ARNT_1.moti TCACRYGTG | ARNT | Direct | -434.38 | -844.6 |
| ARNT_2.moti CACGCA | ARNT | Direct | -118.54 | -737.46 |
| ARNTL_1.mo GGTCACGTGACHC | ARNTL | Direct | NA | -547.96 |
| ASCL2_1.mo ARCAGCTGCY | ASCL2 | Direct | -1371.82 | -704.83 |
| ATF3_1.moti SYCACGTGACC | ATF3 | Direct | -258 | -1448.31 |
| ATF6_1.moti CCACGTCASCMC | ATF6 | Direct | -23.4 | -573.2 |
| ATF6_1.moti CCACGTCASCMC | CREBZF | Inferred | -23.4 | -573.2 |
| ATF7_1.moti ATGACGTCAT | ATF7 | Direct | -472.69 | -195.67 |
| ATOH1_1.mo RVCAKMTGGY | ATOH1 | Direct | -1716.21 | -725.91 |
| ATOH7_1.mo GBGNCAGRTGKY | ATOH7 | Direct | -180.35 | -247.94 |
| BACH1_1.mo WDNTGCTGAGTCATGST | BACH1 | Direct | -182.93 | -1145.67 |
| BACH2_1.mo SRTGAGTCAYC | BACH2 | Direct | -436.72 | -1775.47 |
| BARHL2_1.m TAAWYG | BARHL2 | Direct | NA | -116.01 |
| BARX1_2.mo CMATTA | BARX1 | Direct | NA | NA |
| BATF_1.moti TGASTCAKWYMDWRA | BATF | Direct | -143.88 | -385.17 |
| BATF_1.moti TGASTCAKWYMDWRA | BATF2 | Inferred | -143.88 | -385.17 |
| BCL11A_1.m TTTCASTTCCYCHT | BCL11A | Direct | -391.48 | -1647.46 |
| BHLHE40_1. KCACGTGM | BHLHE40 | Direct | -163.63 | -1293 |
| BSX_1.motif YVATTA | BSX | Direct | NA | NA |
| CBFB_1.moti TYTGTGGTYW | CBFB | Direct | -206.98 | -149.53 |
| CDX1_1.moti TTTWATKRC | CDX1 | Direct | NA | NA |
| CDX2_1.moti DGYMATAAAA | CDX2 | Direct | NA | NA |
| CEBPA_1.mo RTVNATTGCAHAA | CEBPA | Direct | -610.12 | NA |
| CEBPB_1.mo ATTGCRYAAY | CEBPB | Direct | -1403.13 | NA |
| CEBPD_1.moRTTRCGCAAY | CEBPD | Direct | -1510.26 | NA |
| CEBPE_1.mo TTRCGCAAYHT | CEBPE | Direct | -864.52 | NA |
| CLOCK_1.moAACACGTGT | CLOCK | Direct | -496.4 | -1378.89 |
| CLOCK_1.moAACACGTGT | SOHLH1 | Inferred | -496.4 | -1378.89 |
| CREB3L2_1. TGCCACGTGGCA | CREB3L2 | Direct | -623.92 | -2080.58 |
| CREB3L3_1. TGCCACGTGKCA | CREB3L3 | Direct | -660.92 | -2302.1 |
| CREB5_1.mo ATGACGTCAY | CREB5 | Direct | -580 | -157.76 |
| DBP_1.motif RTTACRTAAY | DBP | Direct | -745.19 | NA |
| DLX1_1.moti YAATTA | DLX1 | Direct | NA | NA |
| DLX2_1.moti YAATTA | DLX2 | Direct | NA | NA |
| DLX6_1.moti YAATTA | DLX6 | Direct | NA | NA |
| E2F1_1.motif GGCGGGARV | E2F1 | Direct | -51.77 | -248.98 |

| EBF1_1.moti TCCCHWGGGAVT | EBF1 | Direct | -2382.68 | -541.15 |
| --- | --- | --- | --- | --- |
| EBF1_1.moti TCCCHWGGGAVT | EBF2 | Inferred | -2382.68 | -541.15 |
| EBF1_1.moti TCCCHWGGGAVT | EBF4 | Inferred | -2382.68 | -541.15 |
| EGR2_1.moti MCGCCCACGC | EGR2 | Direct | -71.05 | -269.06 |
| EGR4_1.moti MCGCCCACGCA | EGR4 | Direct | -33.73 | -195.18 |
| EHF_1.motif MCTTCCTS | EHF | Direct | -292.4 | -4868.36 |
| EHF_2.motif ACCCGGAAGTA | EHF | Direct | -822.19 | -5715.58 |
| ELF1_1.motif AACCCGGAAGTG | ELF1 | Direct | -1056.07 | -7270.85 |
| ELF2_1.motif TDNCAGGAAGTRRVT | ELF2 | Direct | -1037.49 | -6885.06 |
| ELF3_1.motif ACCCGGAAGTA | ELF3 | Direct | -554.35 | -3464.14 |
| ELF4_1.motif AACCCGGAAGTR | ELF4 | Direct | -541.54 | -3446.28 |
| ELF5_1.motif AHVMGGAAGT | ELF5 | Direct | -887.78 | -5894.52 |
| ELK1_1.moti CCGGAAGT | ELK1 | Direct | -1232.45 | -6066.75 |
| ELK3_1.moti ACCGGAAGT | ELK3 | Direct | -1277.82 | -6856.54 |
| ELK4_1.moti CCGGAAGY | ELK4 | Direct | -1389.3 | -7986.18 |
| EN1_2.motif TAATTR | EN1 | Direct | NA | NA |
| ENO1_1.moti RSCAYGTGKS | ENO1 | Direct | -108.06 | -1079.66 |
| ERF_1.motif ACCGGAAGT | ERF | Direct | -1227.85 | -7153.08 |
| ERG_1.motif ACCGGAARTVM | ERG | Direct | -1354.61 | -9298.73 |
| ERG_2.motif ACCGGAWATCCGGT | ERG | Direct | -248.67 | -1221.44 |
| ERG_3.motif RCAGGAAGT | ERG | Direct | -1154.04 | -10255.8 |
| ESRRG_3.mo TGACCTTGA | ESRRG | Direct | -9.35 | -301.43 |
| ESX1_1.moti TAATTR | ESX1 | Direct | NA | NA |
| ETS1_1.moti ACMGGAAGT | ETS1 | Direct | -1307.36 | -9420.03 |
| ETS1_2.moti CCGGAAGTACWTCCGGC | ETS1 | Direct | -133.34 | -629.47 |
| ETS2_1.moti MVGGAAGTK | ETS2 | Direct | -550.3 | -3991.64 |
| ETV1_1.moti ACCGGAAGT | ETV1 | Direct | -1332.19 | -8514.8 |
| ETV2_1.moti ACCGGAAAT | ETV2 | Direct | -1118.18 | -7006.03 |
| ETV3_1.moti ACCGGAAGT | ETV3 | Direct | -701.15 | -2713.09 |
| ETV3_1.moti ACCGGAAGT | ETV3L | Inferred | -701.15 | -2713.09 |
| ETV4_1.moti ACCGGAAGT | ETV4 | Direct | -1246.2 | -9003.86 |
| ETV5_1.moti GACCGGAWGTDAY | ETV5 | Direct | -683.54 | -4379.23 |
| ETV6_1.moti CCGGAASCGGAAGT | ETV6 | Direct | -108.66 | -1397.72 |
| ETV7_1.moti KCMHCAGGAAGTRACD | ETV7 | Direct | -447.26 | -3474.26 |
| EVX2_1.moti TMATTA | EVX2 | Direct | NA | NA |
| FERD3L_1.m GYGWCAGCTGK | FERD3L | Direct | -242.26 | -359 |
| FEV_1.motif ACMGGAART | FEV | Direct | -1205 | -9467.73 |
| FLI1_1.motif ACMGGAAGT | FLI1 | Direct | -1323.26 | -9920.29 |
| FLI1_2.motif ACCGGAWATCCGG | FLI1 | Direct | -263.02 | -1391.01 |

| FOS_1.motif VTGASTCAT | FOS | Direct | -455.02 | | -2090.8 | |
| --- | --- | --- | --- | --- | --- | --- |
| FOSB_1.moti RTGAGTCAG | FOSB | Direct | -568.82 | | -1774.92 | |
| FOSL1_1.mo MTGASTCAC | FOSL1 | Direct | -566.2 | | -2315.84 | |
| FOSL2_1.mo RTGASTCAY | FOSL2 | Direct | -104.3 | | -1516.77 | |
| FOXA1_1.mo WRWGTAAACA | FOXA1 | Direct | -9.05 NA | | |  |
| FOXA2_1.mo WRWGTAAACADNNCV | FOXA2 | Direct | NA NA | | |  |
| FOXC1_1.mo CYWAWRTAAACA | FOXC1 | Direct | NA | | | -12.69 |
| FOXD1_1.mo BBWADGTAAACAWDG | FOXD1 | Direct | -28.12 | | | -7.36 |
| FOXD2_2.mo GTAAACA | FOXD2 | Direct | -159.12 | | | -25.82 |
| FOXD4_1.mo GTAAACA | FOXD4 | Direct | -157.57 | | | -32.66 |
| FOXF1_1.mo AAATAAACA | FOXF1 | Direct | NA NA | | |  |
| FOXF1_1.mo AAATAAACA | FOXN1 | Inferred | NA NA | | |  |
| FOXF1_1.mo AAATAAACA | FOXN4 | Inferred | NA NA | | |  |
| FOXF2_1.mo AAVGTAAACAA | FOXF2 | Direct | NA NA | | |  |
| FOXG1_1.mo GTAAACA | FOXG1 | Direct | -166.11 | | -44.77 | |
| FOXK1_1.mo RTAAACA | FOXK1 | Direct | -250.83 | | -142.06 | |
| FOXO3_1.mo WAKGTAAACAA | FOXO3 | Direct | -95.8 | | -77.27 | |
| FOXP1_1.mo GTAAACAAA | FOXP1 | Direct | -94.67 | | -31.16 | |
| FOXP2_1.mo GTAAACA | FOXP2 | Direct | -325.09 | | -140.42 | |
| FOXP3_1.mo RTAAACA | FOXP3 | Direct | -191.16 | | -38.36 | |
| FOXQ1_1.mo ATTGTTTATT | FOXQ1 | Direct | NA | | NA | |
| GABPA_1.moRCCGGAAGT | GABPA | Direct | -1270.51 | | -6806.19 | |
| GATA1_1.mo GBNNVSAGATAA | GATA1 | Direct | -215.93 | | -247.76 | |
| GATA1_1.mo GBNNVSAGATAA | GATAD2 | B Inferred | -215.93 | | -247.76 | |
| GATA2_1.mo AGATAAGARATAAG | GATA2 | Direct | -8.59 | | -19.81 | |
| GATA2_1.mo AGATAAGARATAAG | ZGLP1 | Inferred | -8.59 | | -19.81 | |
| GATA3_2.mo AGATAA GATA3 Direct -44.58 NA | | | | | | |
| GATA4_1.mo WGATAAS | GATA4 | Direct |  | -98.93 -148.03 | | |
| GATA5_1.mo WVANWGATAASNNRR | GATA5 | Direct |  | -21.56 NA | | |
| GATA6_1.mo GATWA | GATA6 | Direct |  | -5.54 NA | | |
| GATA6_1.mo GATWA | MTA2 | Inferred |  | -5.54 NA | | |
| GATA6_1.mo GATWA | RERE | Inferred |  | -5.54 NA | | |
| GBX1_1.moti YAATTA | GBX1 | Direct | NA | NA | | |
| GBX2_1.moti YAATTA | GBX2 | Direct | NA | NA | | |
| GSX1_1.moti YMATTA | GSX1 | Direct | NA | NA | | |
| GSX2_1.moti YMATTA | GSX2 | Direct | NA | NA | | |
| HAND1_1.moGBRTCTGGMW | HAND1 | Direct | -688.26 | | -345.22 | |
| HES1_1.moti GKCKCGTGBCK | HES1 | Direct | -159.02 | | -569.67 | |
| HES1_1.moti GKCKCGTGBCK | HES6 | Inferred | -159.02 | | -569.67 | |

| HES4_1.moti GKCRCGTGCC | HES4 | Direct | -96.79 | | -1128.1 |
| --- | --- | --- | --- | --- | --- |
| HES7_1.moti YGGCACGTGCCR | HES7 | Direct | NA | | -79.4 |
| HEYL_1.moti CHDSCGACACGTGYCVC | HEYL | Direct | -87.52 | | -506.29 |
| HIC1_1.motif GGDTGCCMAC | HIC1 | Direct | -12.2 | | -153.33 |
| HIVEP2_1.m GGYDGGGWAACCSS | HIVEP2 | Direct | -395.01 | | -765.53 |
| HLF_1.motif RTTACRYAAY | HLF | Direct | - | 475.75 NA | |
| HNF1A_1.mo KTDGTTAATNATTAAC | HNF1A | Direct | NA | NA | |
| HNF1B_1.mo RTTAATVATTAAC | HNF1B | Direct | NA | NA | |
| HNF4A_1.mo TGGACTTTGRMCY | HNF4A | Direct | -725.91 | | -460.62 |
| HNF4A_2.mo TGAMCTTTGRMCY | HNF4A | Direct | -905.75 | | -584.9 |
| HNF4G_1.moTGRACTTTGVMCY | HNF4G | Direct | -1018.91 | | -693.19 |
| HOXA1_1.mo YAATTA | HOXA1 | Direct | NA | | NA |
| HOXA10_1.m GYMATAAAA | HOXA10 | Direct | NA | | NA |
| HOXA11_1.m YMATWAAA | HOXA11 | Direct | NA | | NA |
| HOXA13_1.m TTTTATTGG | HOXA13 | Direct | NA | | NA |
| HOXA13_2.m TTTTACGAG | HOXA13 | Direct | NA | | NA |
| HOXA2_1.mo TMATTA | HOXA2 | Direct | NA | | NA |
| HOXA5_2.mo CAYTAATBRR | HOXA5 | Direct |  | -47.91 NA | |
| HOXA6_1.mo TAATKR | HOXA6 | Direct | NA | NA | |
| HOXA6_10.m GCCATAAATCA HOXA6 Direct -257.96 NA | | | | | |
| HOXA6_4.mo TTWATKR | HOXA6 | Direct | NA | NA | |
| HOXA6_7.mo CTAATKRC | HOXA6 | Direct |  | -47.89 NA | |
| HOXA6_8.mo TTAATKA | HOXA6 | Direct | NA | NA | |
| HOXA6_9.mo CNTTAATKA | HOXA6 | Direct |  | -11.97 NA | |
| HOXA9_1.mo TTTTAYKAC | HOXA9 | Direct | NA | NA | |
| HOXA9_2.mo RSYCATAAATCA | HOXA9 | Direct | - | 237.44 NA | |
| HOXB13_1.m TTTATKGG | HOXB13 | Direct | NA | NA | |
| HOXB13_2.m CTCGTAAAA | HOXB13 | Direct | NA | NA | |
| HOXB2_1.mo TMATTA | HOXB2 | Direct | NA | NA | |
| HOXB3_1.mo YMATTA | HOXB3 | Direct | NA | NA | |
| HOXB4_1.mo TGATTRATGRC HOXB4 Direct -513.58 -69.85 | | | | | |
| HOXB5_1.mo YMATTA | HOXB5 | Direct | NA | NA | |
| HOXB7_1.mo TTGATTRATK | HOXB7 | Direct |  | -55.8 NA | |
| HOXB8_1.mo TTGATTAATK | HOXB8 | Direct |  | -16.16 NA | |
| HOXC10_1.mTTTTATKRC | HOXC10 | Direct | NA | NA | |
| HOXC11_1.mTTTTACGAC | HOXC11 | Direct |  | -17.48 NA | |
| HOXC11_2.mTTTTATKRC | HOXC11 | Direct | NA | NA | |
| HOXC12_2.mRGTCGTAAAA | HOXC12 | Direct |  | -17.13 NA | |
| HOXC13_2.mCTCGTAAAA | HOXC13 | Direct | NA | NA | |

| HOXC5_1.moYTAATKA | HOXC5 | Direct | -8.47 NA | |
| --- | --- | --- | --- | --- |
| HOXC9_1.moGCCATAAATCA | HOXC9 | Direct | -284.09 NA | |
| HOXD1_1.moTAATTA | HOXD1 | Direct | NA | NA |
| HOXD11_1.mTTTTAYKA | HOXD11 | Direct | NA | NA |
| HOXD12_1.mTTTTATTAC | HOXD12 | Direct | NA | NA |
| HOXD12_2.mTTTTACGAC | HOXD12 | Direct |  | -26.36 NA |
| HOXD13_1.mCYMATAAAA | HOXD13 | Direct | NA | NA |
| HOXD13_2.mCTCGTAAAA | HOXD13 | Direct | NA | NA |
| HOXD3_1.moYAATTA | HOXD3 | Direct | NA | NA |
| HOXD9_2.moVTCGTAAAA | HOXD9 | Direct |  | -42.61 NA |

IKZF3_1.mot TAKDGGAART	IKZF3	ZifRC	-344.54	-1213.81

IKZF5_1.mot ACNGT	IKZF5	ZifRC	-684.12	-796.82

IRF1_1.motif RSTTTCMSTTTYNBTT	IRF1	Direct	-11.75 NA

| IRF3_1.motif YRGTTTCGGTTTCCBTTTC | IRF3 | Direct | -165.73 | -293.74 |
| --- | --- | --- | --- | --- |
| IRF4_2.motif CGAAACCGAAACYA | IRF4 | Direct | -295.76 | -57.12 |
| IRF5_1.motif RGTTTCGGTTTCGG | IRF5 | Direct | -178.41 | -142.22 |
| IRF6_1.Infer. GTWTCG | IRF6 | Indirect | NA | -137.2 |
| IRF7_1.motif ABTTTCRVTTTCGKA | IRF7 | Direct | -221.34 | -81.94 |
| IRF8_1.motif AGTTTCRGTTTCG | IRF8 | Direct | -450.97 | -189.19 |
| IRF9_1.motif AGTTTCGGTTTCGTT IRF9 Direct -62.48 NA | | | | |
| JDP2_1.motif ATGASTCAT | JDP2 | Direct | -330.28 | -1842.64 |
| JUN_1.motif ATGAGTCAT | JUN | Direct | -300.67 | -1622.58 |
| JUNB_1.moti TGAGTCAYY | JUNB | Direct | -422.12 | -1914.26 |
| JUND_1.moti GRTGASTCATNYYNRRA | JUND | Direct | -101.17 | -1228.7 |
| KLF1_1.moti GCCACACCCW | KLF1 | Direct | NA | -225.27 |
| KLF12_1.motCCCACTG | KLF12 | Direct | -161.6 | -230.84 |
| KLF12_2.motGRCCACGCCCW | KLF12 | Direct | NA | -365.3 |
| KLF13_1.motTGMCACGCCCCTTTTTG | KLF11 | Inferred | -11.03 | -223.34 |
| KLF13_1.motTGMCACGCCCCTTTTTG | KLF13 | Direct | -11.03 | -223.34 |
| KLF14_1.motRCCACGCCCMCY | KLF14 | Direct | NA | -158.55 |
| KLF16_1.motGMCACGCCCCC | KLF16 | Direct | NA | -282.43 |
| KLF4_1.moti GCCMCRCCCW | KLF17 | Inferred | NA | -242.85 |
| KLF4_1.moti GCCMCRCCCW | KLF2 | Inferred | NA | -242.85 |
| KLF4_1.moti GCCMCRCCCW | KLF4 | Direct | NA | -242.85 |
| KLF5_1.moti GCCCCDCCCH | KLF5 | Direct | -29.7 | -454.87 |
| KLF6_1.moti CMGCCCCC | KLF6 | Direct | -106.07 | -353.46 |
| KLF7_1.moti RCCACRCCC | KLF7 | Direct | NA | -164.86 |
| LBX1_1.moti TAATTAG | LBX1 | Direct | NA | NA |
| LEF1_1.motif AAGATCAAAGGRWW | LEF1 | Direct | -84.88 | -179.94 |

| LHX2_1.moti YRSDWBTAATTAG | LHX2 | Direct | NA | | NA | |
| --- | --- | --- | --- | --- | --- | --- |
| LHX6_1.moti TRATTA | LHX6 | Direct | NA | | NA | |
| LMX1A_1.moTAATTAA | LMX1A | Direct | NA | | NA | |
| LMX1B_1.moTTAATTR | LMX1B | Direct | NA | | NA | |
| LYL1_1.Infer CAGCTGC | LYL1 | Indirect | -815.08 | | -355.2 | |
| MAF_1.motif GTCAGCA | MAF | Direct | -912.66 | | -707.54 | |
| MAFA_1.mot TGCTGACCTGRCWVHHTYC | MAFA | Direct | -257.54 | | -447.56 | |
| MAFB_1.mot TGCTGMYGWCDGCA | MAFB | Direct | -1079.95 | | -755.77 | |
| MAFF_2.mot TGCTGASTCAGCA | MAFF | Direct | -54.28 | | -73.75 | |
| MAFG_1.mot WWNTGCTGASTCAGCA | MAFG | Direct | -202.26 | | -116.9 | |
| MAX_2.motif CACGTG | MAX | Direct | -263.18 | | -1010.22 | |
| MBD2_1.mot SSGKCCGGMGR | MBD2 | Direct | -456.36 | | -397.83 | |
| MECP2_1.moSCCGGRR | MECP2 | Direct | -1097.61 | | -587.48 | |
| MEIS2_1.motTTGACAGSTGTCA | MEIS2 | Direct | -140.27 | | -184.81 | |
| MEOX1_1.moSTAATTA MEOX1 Direct -15.68 NA | | | | | | |
| MEOX2_1.moTAATTA | MEOX2 | Direct | NA | NA | | |
| MLXIPL_1.m RTCACGTGAT | MLXIPL | Direct |  | -16.23 -1116.94 | | |
| MNX1_1.mot YAATTA | MNX1 | Direct | NA | NA | | |
| MSC_1.motif AACAGCTGTT | MSC | Direct | -665.28 | | | -359.2 |
| MSX1_1.mot CNBTAATTG | MSX1 | Direct | -24.96 NA | | |  |
| MSX1_1.mot CNBTAATTG | NKX1-2 | Inferred | -24.96 NA | | |  |
| MTF1_1.moti TTTGCACACGGCAC | MTF1 | Direct | -243.57 | | -244.67 | |
| MXI1_1.moti CACGTGG | MXD1 | Inferred | -407.83 | | -1484.88 | |
| MXI1_1.moti CACGTGG | MXD3 | Inferred | -407.83 | | -1484.88 | |
| MXI1_1.moti CACGTGG | MXD4 | Inferred | -407.83 | | -1484.88 | |
| MXI1_1.moti CACGTGG | MXI1 | Direct | -407.83 | | -1484.88 | |
| MYB_1.motif CNRVTGRCAGTTRRBGG | MYB | Direct | -602.18 | | -516.04 | |
| MYB_1.motif CNRVTGRCAGTTRRBGG | RCOR2 | Inferred | -602.18 | | -516.04 | |
| MYB_1.motif CNRVTGRCAGTTRRBGG | RCOR3 | Inferred | -602.18 | | -516.04 | |
| MYB_1.motif CNRVTGRCAGTTRRBGG | SNAPC4 | Inferred | -602.18 | | -516.04 | |
| MYBL1_1.moAACCGTTAAMC | DNAJC2 | Inferred | -730.69 | | -808.1 | |
| MYBL1_1.moAACCGTTAAMC | MYBL1 | Direct | -730.69 | | -808.1 | |
| MYBL1_2.moGDCVGTTADWACSGTTR | DNAJC2 | Inferred | -283.94 | | -360.27 | |
| MYBL1_2.moGDCVGTTADWACSGTTR | MYBL1 | Direct | -283.94 | | -360.27 | |
| MYCN_1.motCCACGTG | MYCL | Inferred | -695.85 | | -2207.27 | |
| MYF5_1.mot AACAGCTGT | ASCL1 | Inferred | -696.49 | | -356.94 | |
| MYF5_1.mot AACAGCTGT | ASCL5 | Inferred | -696.49 | | -356.94 | |
| MYF6_1.mot AACAGCTGTT | ASCL3 | Inferred | -741.4 | | -310.22 | |
| MYF6_1.mot AACAGCTGTT | MYF6 | Direct | -741.4 | | -310.22 | |

| MYOD1_1.m RRCAGSTGBYG | MYOD1 Direct | | -220.27 | -277.89 |
| --- | --- | --- | --- | --- |
| MYOG_1.mo TRACAGCTGSWG | MYOG Direct | | -924.35 | -629.93 |
| NEUROD1_1 GRCCATCTGYY | NEUROD1 Direct | | -605.62 | -557.03 |
| NEUROD4_1 RTRACATATGG | NEUROD4 Direct | | -268.64 | -100.3 |
| NFE2_1.moti RTGACTCAGCANWT | NFE2 | Direct | -470.02 | -891.23 |
| NFE2_2.moti ATGACTCATS | NFE2 | Direct | -252.38 | -1479.13 |
| NFE2L2_1.m TGCTGAGTCATBST | NFE2L2 | Direct | -361.09 | -829.07 |
| NFIA_1.motif TTGGCANRDTGCCAR | NFIA | Direct | -4247.46 | -1563.21 |
| NFIB_1.motif TTGGCANNDTGCCAR | NFIB | Direct | -4957.79 | -1864.74 |
| NFIC_1.motif TTGGC | NFIC | Direct | -1379.14 | -276.73 |
| NFIX_1.motif TTGGCANNNWGCCAR | NFIX | Direct | -5556.41 | -2002.77 |
| NFIX_2.motif TTGGCA | NFIX | Direct | -3710.31 | -1087.66 |
| NFKB1_1.mo RGGGGAWTCCCCT | NFKB1 | Direct | NA | NA |
| NFKB1_2.mo GGRAAKTCCCC | NFKB1 | Direct | -227.82 | -544.75 |
| NFKB2_1.mo GGGGAWTCCCC | NFKB2 | Direct | NA | NA |
| NKX2_1_1.m SCACTYVA | NKX2-1 | Direct | NA | NA |
| NKX2_2_1.m AASYACTTAA | NKX2-2 | Direct | NA | NA |
| NKX2_3_1.m CCACTTRA | NKX2-3 | Direct | -52.49 | -24.62 |
| NKX2_4_2.m SCACTTGARM | NKX2-4 | Direct | NA | NA |
| NKX2_5_1.m CACTTRA | NKX2-5 | Direct | NA | NA |
| NKX2_5_3.m SCACTCAAS | NKX2-5 | Direct | NA | NA |
| NKX2_6_1.m SCACTTGAR | NKX2-6 | Direct | NA | NA |
| NKX2_8_1.m SYMCTTGA | NKX2-8 | Direct | NA | -10.48 |
| NKX3_1_2.m RRSCACTTA | NKX3-1 | Direct | NA | NA |
| NKX3_2_1.m CCACTTA | NKX3-2 | Direct | -19.7 | -67.12 |
| NKX6_2_1.m YMATTA | NKX6-2 | Direct | NA | NA |
| NKX6_3_1.m TAATKA | NKX6-3 | Direct | NA | NA |
| NOBOX_1.m TAATTR | NOBOX | Direct | NA | NA |
| NPAS2_1.mo MCACGTG | NPAS2 | Direct | -711.41 | -2073.5 |
| NR0B1_1.mo GMGKGGGASR | NR0B1 | Direct | -369.64 | -681.19 |
| NR0B1_1.mo GMGKGGGASR | NR0B2 | Inferred | -369.64 | -681.19 |
| NR1H2_1.mo TAAAGGTCAAAGGTCAAC | NR1H2 | Direct | -83.69 | -132.88 |
| NR1H4_1.mo AGGTCANTGACCY | NR1H4 | Direct | -22.44 | -318.35 |
| NR2C1_1.mo TGACCTCTBRSC | NR2C1 | Direct | -162.39 | -485.4 |
| NR2C2_1.mo RGGTCARAGGTCA | NR2C2 | Direct | -396.81 | -605.93 |
| NR2C2_2.mo GMCCCGGAAGYRVW | NR2C2 | Direct | -210.39 | -1085.22 |
| NR2F1_1.mo TGACCTTTGAMCYY | NR2F1 | Direct | -566.45 | -662.9 |
| NR2F1_2.mo CNBKTGACCYYT | NR2F1 | Direct | NA | -270.19 |
| NR2F2_2.mo TGACCTTTGACCT | NR2F2 | Direct | -606.84 | -763.57 |

| NR2F6_3.mo YYMNNTGACCTTTGACCY | NR2F6 | Direct | -445.66 | -658.36 |
| --- | --- | --- | --- | --- |
| NR4A1_1.mo TGACCTTT | NR4A1 | Direct | -344.58 | -301.98 |
| NR4A2_3.mo TTAAAGGTCA | NR4A2 | Direct | -287.72 | -342.56 |
| NR4A3_1.mo STGACCTTTG | NR4A3 | Direct | -131.06 | -312.36 |
| NR5A1_1.mo TGRCCTTG | NR5A1 | Direct | -89.64 | -344.02 |
| NRL_1.motif TGCTGAC | NRL | Direct | -1029.05 | -770.12 |
| ONECUT1_1. TATYGATTTT | ONECUT | 1 Direct | NA | NA |
| ONECUT1_1. TATYGATTTT | SATB1 | Inferred | NA | NA |
| ONECUT1_1. TATYGATTTT | SATB2 | Inferred | NA | NA |
| OTP_1.motif TAATTR | OTP | Direct | NA | NA |
| PAX1_1.moti CGTCACGCWTGAVTGM | PAX1 | Direct | -36.81 | -99.95 |
| PAX2_1.moti GTCACGCWTSABTGMNY | PAX2 | Direct | -50.88 | -242.97 |
| PAX2_3.moti GTCAYKS | PAX2 | Direct | -392.93 | -733.39 |
| PAX4_5.moti CTAATTAR | PAX4 | Direct | NA | NA |
| PAX5_2.moti GTCACGCWYSRCTGMNY | PAX5 | Direct | NA | -75.35 |
| PAX6_1.moti TTCACGCWTGANTKMRY | PAX6 | Direct | -57.48 | -87.67 |
| PAX8_1.moti MYRCKYSARTBA | PAX8 | Direct | -147.49 | -414.37 |
| PAX8_2.moti GYCAYGCHTGRCTGV | PAX8 | Direct | -18.97 | -72.1 |
| PDX1_2.moti CTAATTA | PDX1 | Direct | NA | NA |
| PLAGL1_1.m CRGGGGGCCC | PLAGL1 | Direct | NA | -288.15 |
| PLAGL2_2.m GGGGC | PLAGL2 | ZifRC | -113.62 | -563.8 |
| PPARD_1.moTGACCTTTVNCCTR | PPARD | Direct | -389.56 | -750.35 |
| PPARG_1.moTGACCTTTGMCCY | PPARG | Direct | -854.92 | -819.62 |
| PRDM15_4.mTDYG | PRDM15 | ZifRC | -516.99 | -12.83 |
| PTF1A_1.mo GSRCASSTGTKSWDTTYCCY PTF1A Direct -400.7 -475.99 | | | | |
| RARA_1.moti TGACCTYTNSWTGACCT | RARA | Direct | -69.36 | -478.39 |
| RELA_1.moti GGAAAKYCCC | RELA | Direct | -236.47 | -498.63 |
| RELB_1.moti GGAAAKYCCC | RELB | Direct | -230.57 | -316.96 |
| RFX1_1.moti GTTGCYAGGBRAC | RFX1 | Direct | -524.32 | -575.81 |
| RFX1_2.moti GTTGCCATGGYRAC | RFX1 | Direct | -124.64 | -149.87 |
| RFX2_3.moti SGTTGCTARGCAACS | RFX2 | Direct | -262.99 | -154.9 |
| RFX3_1.moti CBGTTGCCATGGYAAC | RFX3 | Direct | -184.92 | -210.76 |
| RFX4_2.moti GTTGCTARGCAACG | RFX4 | Direct | -366.02 | -249.57 |
| RFX5_1.moti TCABCWGTTGCYAGGSAR | RFX5 | Direct | -263.33 | -258.88 |
| RFX5_2.moti GTTGCYATGGYAAC | RFX5 | Direct | -194.48 | -166.28 |
| RFX6_4.moti GTTGCCATGGYAAC | RFX6 | Direct | -37.32 | -43.04 |
| RHOXF1_1.mGGHTRATCC | RHOXF1 | Direct | NA | -28.74 |
| RHOXF1_1.mGGHTRATCC | RHOXF2 | Inferred | NA | -28.74 |
| RHOXF1_1.mGGHTRATCC | RHOXF2B | Inferred | NA | -28.74 |

| RUNX1_1.moAACCACA | RUNX1 | Direct | NA | -7.64 |
| --- | --- | --- | --- | --- |
| RUNX2_1.moWAACCACA | RUNX2 | Direct | NA | -6.68 |
| RUNX3_1.moWAACCRCAAACCVCA | RUNX3 | Direct | NA | NA |
| RXRA_3.moti TGACCTTTGACCY | RXRA | Direct | -682.99 | -739.98 |
| RXRA_4.moti TGACCTYTG | RXRA | Direct | -173.02 | -505.32 |
| RXRB_1.moti GGGGTCAAAGGTCA | RXRB | Direct | -312.59 | -344.55 |
| RXRG_1.mot TGACCTTTGACCCC | RXRG | Direct | -378.03 | -413.89 |
| SIX1_1.motif GKVTCADRTTWC | SIX1 | Direct | -244.59 | -172.53 |
| SMAD1_1.m GGCAGACAGGSY | SMAD9 | Inferred | -250.77 | -424.46 |
| SMAD2_2.m TGTCTGTCWSST | SMAD2 | Direct | -283.09 | -410.02 |
| SMAD3_2.m WGTCTGSC | SMAD3 | Direct | -779.42 | -533.26 |
| SMAD3_2.m WGTCTGSC | SMAD7 | Inferred | -779.42 | -533.26 |
| SMAD4_1.m YGTCTGSC | SMAD6 | Inferred | -683.81 | -491.4 |
| SMARCC1_1 TGAGTCAY SMARCC1 Direct -133.61 -1301.13 | | | | |
| SMARCC1_1 TGAGTCAY | TADA2A | Inferred | -133.61 | -1301.13 |
| SOX10_5.mo CWTTGT | SOX10 | Direct | -22.04 | -61.64 |
| SOX3_1.moti CCTTTGTYYY | SOX3 | Direct | -10.49 | -196.5 |
| SOX6_1.moti CCWTTGTY | SOX6 | Direct | NA | -335.64 |
| SP3_1.motif CCMCGCCCMC | SP3 | Direct | NA | -238.6 |
| SPDEF_1.mo AMCCGGATGT | SPDEF | Direct | -697.03 | -3859.44 |
| SPI1_1.motif AAAAAGCGGAAGTW | SPI1 | Direct | -181.25 | -588.93 |
| SPI1_2.motif RAAARAGGAAGWRRDRVT | SPI1 | Direct | -85.98 | -731.32 |
| SPIB_1.motif WGMGGAA | SPIB | Direct | -592.3 | -2322.82 |
| SPIB_2.motif AAAAGMGGAAGT | SPIB | Direct | -354.05 | -1565.38 |
| SPIC_1.motif AAAAGMGGAAGTA | SPIC | Direct | -246.36 | -1577.63 |
| STAT1_1.mo TTYCMGGRAA | STAT1 | Direct | -1208.52 | -825.75 |
| STAT2_1.mo GAAAVYGAAACTDA | STAT2 | Direct | -335.7 | -174.65 |
| STAT3_1.mo KSAYTTCCDGGAA | STAT3 | Direct | -1210.68 | -1023.55 |
| TAL1_1.moti AACAGRWGKTCDMCAGAYG TAL1 Direct -94.1 -131.93 | | | | |
| TCF12_1.mo CASCTGCTGYY | TCF12 | Direct | -504.13 | -538.3 |
| TCF21_1.mo AACAGCTGTT | TCF21 | Direct | -1246.34 | -380.19 |
| TCF3_1.moti VCASCTGY | TCF3 | Direct | NA | -11.11 |
| TCF7_1.moti AAAGATCAAAG | TCF7 | Direct | -85.95 | -60.53 |
| TEAD1_1.mo ACATTCCWSNG | TEAD2 | Inferred | -515.29 | -282.13 |
| TEAD3_2.mo RCATTCCW | TEAD3 | Direct | -305.64 | -70.66 |
| TFAP2A_1.m GCCYCAGGC | TFAP2A | Direct | -46.37 | -179.24 |
| TFAP2A_2.m GCCYSRGGGCA | TFAP2A | Direct | -569.45 | -712.59 |
| TFAP2A_3.m GCCCBVRGGGC | TFAP2A | Direct | -236.99 | -300.74 |
| TFAP2D_1.m ACGSGCCBCRGGCG | TFAP2D | Direct | NA | NA |

| TFAP2E_1.m GCCYSRGGSBA | TFAP2E | Direct | -358.6 | | -506.78 |
| --- | --- | --- | --- | --- | --- |
| TFAP4_2.mo CAMCAGCTGWB | TFAP4 | Direct | -1158.72 | | -658.1 |
| TFE3_1.motifRTCACGTG | TFE3 | Direct | -148.33 | | -1062.5 |
| TFEB_1.moti GTCACGTGRY | TFEB | Direct | -126.63 | | -898.57 |
| TFEC_1.moti RTCACRTGAB | TFEC | Direct | -50.12 | | -471.03 |
| TGIF2LX_1.mTGACAGSTGTCA | TGIF2LX | Direct | -99.93 | | -100.97 |
| TLX1_2.moti TGGCAVSDDGCCAA | TLX1 | Direct | -566.89 | | -209.82 |
| USF1_1.moti GTCACGTGRY | USF1 | Direct | -122.62 | | -887.67 |
| USF2_1.moti GTCACGTGRBS | USF2 | Direct | -28.66 | | -624.05 |
| VAX1_1.moti YTAATTA | VAX1 | Direct | NA | | NA |
| VAX2_1.moti YTAATTA | VAX2 | Direct | NA | | NA |
| VSX1_1.moti GCYAATTA | VSX1 | Direct | NA | | NA |
| ZBTB1_1.Inf CMCGCA | ZBTB1 | Indirect | -580.42 | | -133.28 |
| ZBTB14_1.m GCKNGTTCGNGWRSC | ZBTB14 | ZifRC | -254.12 | | -817.26 |
| ZBTB18_2.m ATCCAGATGTK | ZBTB18 | Direct | -123.15 | | -26.26 |
| ZBTB33_1.m RNTCKCGCGAGANC | ZBTB33 | Direct | -410.91 | | -840.87 |
| ZBTB42_1.m WTCCAGATGTK | ZBTB42 | Direct | -114.23 | | -15.47 |
| ZBTB7A_2.m GCGACCACC | ZBTB7A | Direct | NA | | -49.23 |
| ZFAT_2.moti KGGC | ZFAT | ZifRC | -44.12 | | -301.39 |
| ZFHX2_1.mo TAATTA | ZFHX2 | Direct | NA | | NA |
| ZFP57_1.mot GCCGHCBTTWNG | ZFP57 | ZifRC | -1205.01 | | -1045.72 |
| ZFP64_1.mot AANTNDBAAADGCTRATDCC ZFP64 ZifRC -199.97 -510.17 | | | | | |
| ZFX_1.motif AGGCCYAGGCCBSGS | ZFX | Direct | NA |  | -66.19 |
| ZFY_1.motif AGGCCBVRGC | ZFY | Direct | NA |  | -164.07 |
| ZIC4_1.motif GRCCCCCCGCKGYG | ZIC4 | Direct | NA |  | -254.5 |
| ZIC5_1.motif AKVCCCCCCRCCG | ZIC5 | Direct |  | -9.66 | -211.84 |
| ZIC5_2.motif GACCCCCCGCTGYG | ZIC5 | Direct |  | -62.14 | -297.6 |
| ZIC5_3.motif CCYSCDGNG | ZIC5 | Direct |  | -62.16 | -213.61 |
| ZNF100_1.m YGDTGAACDTTTNGMYAHGGZNF100 ZifRC NA -321.6 | | | | | |
| ZNF142_3.m GYTGNNYDMCGHT ZNF142 | | ZifRC | -364.57 | | -487.55 |
| ZNF19_1.mo AYGANGGTHGGGTYDNGGACZNF19 | | ZifRC | -160.11 | | -1345.02 |
| ZNF2_1.moti GDAVYCGYHGCAGTCHYCGT ZNF2 | | ZifRC | -620.22 | | -614.34 |
| ZNF208_1.m ATRCNNCTTCSDNGDNGACA ZNF208 | | ZifRC | -329.74 | | -466.24 |
| ZNF208_2.m TRCNNCTTCSDNGRYGNCTG ZNF208 | | ZifRC | -255.81 | | -473.15 |
| ZNF292_1.m GANGCG ZNF292 | | ZifRC | -18.81 | | -56.32 |
| ZNF296_1.m GGAAA ZNF296 | | ZifRC | -634.84 | | -1044.22 |
| ZNF296_2.m GGAAAT ZNF296 | | ZifRC | -716.32 | | -1818.21 |
| ZNF329_1.m GTGMGADCCGWHGVCHGGR ZNF329 | | ZifRC | -142.02 | | -477.35 |
| ZNF335_1.m GCGMTG ZNF335 | | ZifRC | -433.05 | | -321.74 |

| ZNF343_1.m GATRTGVCCGAARTGRCCTC ZNF343 | | | | ZifRC | -259.74 | | -320.9 |
| --- | --- | --- | --- | --- | --- | --- | --- |
| ZNF350_1.m GGCMMCAAABNHYTGCBBC ZNF350 | | | | Direct | -408.18 | | -663.24 |
| ZNF433_2.m GACRKBHG ZNF433 | | | | ZifRC | -1144.92 | | -1150.87 |
| ZNF441_1.m CCDHCWHTGTTGTCDDNGT ZNF441 | | | | ZifRC | NA | | -103.69 |
| ZNF445_2.m TTVCG ZNF445 | | | | ZifRC | -1957.27 | | -1404.62 |
| ZNF445_3.m TGVYG ZNF445 | | | | ZifRC | -326.2 | | -527.94 |
| ZNF516_2.m AGMAG ZNF516 | | | | ZifRC | -890.08 | | -712.96 |
| ZNF536_2.m AGCWR ZNF536 | | | | ZifRC | -453.21 | | -217.42 |
| ZNF547_1.m GTANTGCCGACNCTGGDGCCZNF547 | | | | ZifRC | -310.58 | | -559.7 |
| ZNF548_1.m GGRBTRATGDTDYTGYTRCC ZNF548 | | | | ZifRC | -100.94 | | -145.11 |
| ZNF550_1.m GGANGRHSAGWGCTANGGC ZNF550 | | | | ZifRC | -171.17 | | -600.47 |
| ZNF560_3.m TWADTDC ZNF560 ZifRC -52.31 NA | | | | | | | |
| ZNF566_1.m TAGCNRBAA | AKGANGCCGKT ZNF566 | | | ZifRC |  | -90.47 | -404.31 |
| ZNF579_1.m GDCGGGG | ZNF579 | | | ZifRC | NA |  | -233.45 |
| ZNF579_3.m GTGGCGHGG | ZNF579 | | | ZifRC | NA |  | -379.32 |
| ZNF594_2.m GCAGC | | ZNF594 | | ZifRC | -1457.14 | | -859.36 |
| ZNF594_3.m TGMGAGC | | ZNF594 | | ZifRC | -93.84 | | -202.9 |
| ZNF594_4.m TGMGAKCYGCAGC | | ZNF594 | | ZifRC | -341.27 | | -305.87 |
| ZNF597_2.m GRRNTT | | ZNF597 | | ZifRC | -38.29 | | -83.11 |
| ZNF606_1.m GNYCWGNMGAGG | | ADCCTGT ZNF606 | | ZifRC | -63.49 | | -302.68 |
| ZNF676_1.m GTGRYKRMTDYTNYTMGVD | | | ZNF676 | ZifRC | -160.23 | | -591.91 |
| ZNF681_1.m AAGGADGTMGGACAAAAMA | | | ZNF681 | ZifRC | -23.66 | | -436.44 |
| ZNF699_1.m TYNGCWGRWCCVY | | | ZNF699 | ZifRC | -286.12 | | -473.5 |
| ZNF705D_1. GAAGGAG | | | ZNF705D | ZifRC | -53.33 | | -432.93 |
| ZNF716_1.m YTGCC | | | ZNF716 | ZifRC | -250.74 | | -463.12 |
| ZNF721_2.m CGMAAAYADSAGGAWG | | | ZNF721 | ZifRC | -183.94 | | -653.47 |
| ZNF732_1.m STNYTCTAYGRCARTGDYGD | | | ZNF732 | ZifRC | -564.83 | | -521.71 |
| ZNF746_1.m TGGGGCGGG | | | ZNF746 | ZifRC | -44.29 | | -424.16 |
| ZNF763_2.m GTTGTC | | | ZNF763 | ZifRC | -317.96 | | -270 |
| ZNF768_1.m GCAATGATG | RTAGTAMTAGMZNF768 | | | ZifRC | -153.35 | | -253.46 |
| ZNF770_2.m GAGGA | ZNF770 | | | ZifRC | -196.36 | | -638.36 |
| ZNF777_2.m GCGGG | ZNF777 | | | ZifRC | -300.82 | | -618.02 |
| ZNF781_1.m GGGACYDGG | ZNF781 | | | ZifRC | -193.17 | | -554.57 |
| ZNF80_2.mo GTKGYT | ZNF80 | | | ZifRC | -20.39 | | -56.63 |
| ZNF827_1.m AGGNG | ZNF827 | | | ZifRC | NA | | -16.4 |
| ZNF836_2.m YGNGCRYKGCTGANWBT ZNF836 | | | | ZifRC | -291.16 | | -270.3 |
| ZNF91_3.mo RYADCCGCAMBRVGGGCAW ZNF91 | | | | ZifRC | -106.91 | | -289.81 |
| ZSCAN25_1. GCAGKGAAGHGGNGAKRCGAZSCAN25 | | | | ZifRC | -126.36 | | -619.23 |

alternative band_form b_cell..cee b_cell_fro brain..ceehbrain_hipp cd14_posi cd19positi

-149.35	-160.8	-175.37	-250.89	-68.52	-173.6	-159.92	-201.68

NA	NA	NA	NA	NA	NA	NA	NA

-191.84	-249.16	-1611.54	-263.94	-76.17	-177.14	-185.99	-2284.47

-408.87	-275.84	-1313.71	-247.59	-62.21	-108.61	-186.08	-2354.32

-71.55	-65.53	-918.19	-53.96 NA	NA	-41.33	-1384.5

-429.45	-764.67	-373.54	-1060.77	-2337.45	-2440.43	-497.84	-182.49

-255.47	-298.11	-1837.93	-244.38	-32.33	-130.28	-213.56	-2779.71

-70.37	-53.63	-589.39	-79.07 NA	-15.42	-56.32	-1063.5

-70.37	-53.63	-589.39	-79.07 NA	-15.42	-56.32	-1063.5

-66.8	-147	-62.09 NA	-11.78	-67.82	-86.45	-139.7

-234.64	-451.96	-233.4	-581.51	-2038.44	-2054.93	-286.62	-118.01

-96.37	-172.62	-196.32	-371.5	-666.2	-728.62	-105.79	-120.24

-274.7	-262.5	-107.29	-49.31 NA	-34.56	-274.62	-87.93

-327.83	-260.39	-285.51	-99.85 NA	-89.64	-290.05	-315.89

NA	NA	-495.79 NA	NA	NA	NA	-851.99

NA	NA	NA	NA	-27.23 NA	NA	NA

NA	NA	-434.81 NA	-8.83	-8.66	-14.14	-338.59

NA	NA	-434.81 NA	-8.83	-8.66	-14.14	-338.59

-670.19	-1189.52	-3082.25	-2104.4	-184.18	-128.2	-1473.65	-2633.66

-187.69	-271.13	-2362.69	-294.49	-53.99	-109.17	-169.41	-3429.54

NA	NA	NA	NA	-32.46 NA	NA	NA

-543.9	-1059.53	-831.47	-1444.2	-69.29	-150.31	-891.76	-584.61

NA	NA	NA	NA	NA	NA	NA	NA NA	NA	NA	NA	NA	NA	NA	NA

-52.05	-422.79	-14.24 NA	-7.19 NA	-180.23 NA

-305.67	-1480.31 NA	NA	NA	NA	-838.13 NA

-318.96	-1549.9 NA	NA	NA	NA	-874.77 NA

-184.32	-1070.83 NA	NA	NA	NA	-576.01 NA

-130.03	-237.88	-2174.83	-282.81	-156	-255.99	-145.03	-2983.31

-130.03	-237.88	-2174.83	-282.81	-156	-255.99	-145.03	-2983.31

-256.41	-399.36	-2499.84	-396.56	-195.53	-380.5	-258.65	-3462.06

-240.61	-420.16	-2982.99	-428.15	-183.42	-383.07	-284.32	-4165.55

-58.47	-171.69	-54.64 NA	NA	-52.43	-97.06	-98.39

-62.31	-351.27 NA	NA	NA	NA	-255.14 NA NA	NA	NA	NA	NA	NA	NA	NA NA	NA	NA	NA	-10.12 NA	NA	NA NA	NA	NA	NA	-22.32 NA	NA	NA

-196.84	-141.36 NA	-210.24	-76.44	-95.11	-123.53 NA

| -37.98 | -77.1 | -120.94 | -551.18 | -11.86 | -85.79 | -43.02 | -62.49 |
| --- | --- | --- | --- | --- | --- | --- | --- |
| -37.98 | -77.1 | -120.94 | -551.18 | -11.86 | -85.79 | -43.02 | -62.49 |
| -37.98 | -77.1 | -120.94 | -551.18 | -11.86 | -85.79 | -43.02 | -62.49 |
| -70.62 | -28.64 | -309.51 | -284.74 NA |  | -34.65 | -20.74 | -520.48 |
| -80.28 | -38.55 | -121.39 | -212.46 NA |  | -44.37 | -25.19 | -253.92 |
| -1161.46 | -2867.04 | -1328.66 | -3403.75 | -96.49 | -100.72 | -2078.77 | -937.6 |

-1792.02	-4172.83	-2077.43	-3966.08	-287.17	-245.07	-3107.55	-1580.61

-2710.17	-5932.5	-3086.16	-5872.57	-445.33	-344.4	-4539.32	-2343.86

-1650.39	-3707.07	-2300.95	-4505.43	-402.61	-359.65	-2887.53	-1660.48

-1219	-2837.24	-1450.68	-2549.23	-160.88	-151.11	-2125.16	-1160.45

-1733.25	-3738.05	-1538.71	-3415.08	-165.57	-149.24	-2798.95	-1139.38

-2242.09	-5341.41	-3314.49	-5111.1	-277.63	-199.78	-4149.72	-2471.58

-1891.67	-4020.93	-1382.27	-3299.94	-440.9	-392.28	-2917.67	-1079.2

-2118.37	-4585.6	-1938.84	-4060.76	-479.62	-371.94	-3289.71	-1342.41

-2443.21	-5459.04	-2110.82	-4815.66	-572.77	-468.32	-3942.16	-1583.75

NA	NA	NA	NA	-33.83 NA	NA	NA

-78.88	-104.82	-1664.9	-106.32	-48.58	-105.01	-47.86	-2549.01

-2439.85	-5317.55	-2346.25	-5098.02	-554.7	-395.24	-3919.39	-1794.23

-2826.93	-6064.7	-2972.98	-5751.78	-685.12	-417.07	-4516.99	-2201.19

-270.63	-512.82	-191.43	-370.16	-79.35	-75.99	-385.5	-177.47

-3028.65	-7208.31	-3682.91	-7459.34	-501.08	-366.31	-5289.83	-2540.06

NA	NA	-15.83 NA	NA	NA	NA	-62.03

NA	NA	NA	NA	-17.89 NA	NA	NA

-2939.62	-6579.16	-3124.29	-6408.7	-550.56	-376.42	-4800.09	-2228.21

-146.42	-271.43	-76	-240.57	-60.11	-56.12	-206.39	-60.93

-1402.69	-3258.58	-1255.96	-3136.36	-180.52	-152.63	-2550.31	-952.23

-2934.52	-6328.51	-2591.02	-6047.34	-656.38	-483.01	-4710.21	-1927.04

-2104.71	-4613.88	-2686.56	-4469.79	-471.63	-262.18	-3405.48	-2031.52

-1068.93	-2105.11	-731.03	-1808.24	-243.7	-189.85	-1548.81	-534.42

-1068.93	-2105.11	-731.03	-1808.24	-243.7	-189.85	-1548.81	-534.42

-3045.54	-6802.47	-3037.38	-6861.89	-585.81	-399.48	-5105.96	-2229.48

-1413.07	-3071.38	-827.32	-2875.54	-304.68	-250.93	-2259.97	-604.48

-1020.13	-2260.1	-849.26	-2100.49	-43.3	-20.92	-1681.52	-644.07

-890.26	-2002.79	-1271.61	-2089.56	-110.66	-96.75	-1500.82	-1009.97

NA	NA	NA	NA	-16.23 NA	NA	NA

-94.12	-214.22	-274.71	-335.11	-491.43	-581.73	-112.67	-204.18

-2597.78	-6084.54	-3566.11	-5918.97	-461.39	-256.18	-4592.68	-2516.03

-3264.67	-7328.7	-3335.18	-7336.41	-599.62	-441.07	-5418.42	-2395.36

-297.97	-569.91	-191.28	-410.57	-86.78	-78.33	-418.03	-196.11

| -245.03 | | -126.33 | | -448.35 | |  | -24.68 | NA |  |  | -68.29 | -235.37 | | -415.63 | |
| --- | --- | --- | --- | --- | --- | --- | --- | --- | --- | --- | --- | --- | --- | --- | --- |
| -173.76 | |  | -128.6 | -458.41 | |  | -53.68 |  | -29.44 | -109.79 | | -187.17 | | -355.53 | |
| -315.64 | |  | -270.5 |  | -521.4 |  | -95.03 |  | -14.16 | -137.42 | | -347.64 | | -437.13 | |
| -174.75 | |  | -50.04 | -190.28 | | NA |  | NA |  | NA |  | -120.88 | | -185.92 | |
| NA |  | NA |  | NA |  | NA |  | NA |  | NA |  | NA |  | NA |  |
| NA |  | NA |  | NA |  | NA |  | NA |  | NA |  | NA |  | NA |  |
| NA |  | NA |  | NA |  | NA |  | NA |  | NA |  | NA |  | NA |  |
| NA |  | NA |  | NA |  | NA |  | NA |  | NA |  | NA |  | NA |  |
| NA |  | NA |  |  | -8 | NA |  |  | -47.03 |  | -50.58 | NA |  | NA |  |
| NA |  | NA |  |  | -15.86 | NA |  |  | -47.55 |  | -51.72 | NA |  | NA |  |
| NA |  | NA |  | NA |  | NA |  | NA |  | NA |  | NA |  | NA |  |
| NA |  | NA |  | NA |  | NA |  | NA |  | NA |  | NA |  | NA |  |
| NA |  | NA |  | NA |  | NA |  | NA |  | NA |  | NA |  | NA |  |
| NA |  | NA |  | NA |  | NA |  | NA |  | NA |  | NA |  | NA |  |
| NA |  | NA |  |  | -11.16 | NA |  |  | -45.69 |  | -50.87 | NA |  | NA |  |
| NA |  | NA |  |  | -38.96 | NA |  |  | -89.83 | -108.73 | | NA |  |  | -27.38 |
| NA |  | NA |  |  | -47.57 | NA |  |  | -31.16 |  | -20.36 | NA |  |  | -57.16 |
| NA |  | NA |  |  | -43.27 | NA |  |  | -62.15 |  | -42.84 | NA |  |  | -49.4 |
| NA |  | NA |  |  | -68.27 |  | -26.27 |  | -99.76 |  | -142.2 |  | -6.32 |  | -54.85 |
| NA |  | NA |  |  | -11.15 | NA |  |  | -47.36 |  | -75.41 | NA |  | NA |  |
| NA |  | NA |  | NA |  | NA |  | NA |  | NA |  | NA |  | NA |  |
| -2705.62 | | -5736.53 | | -2031.33 | | -5420.41 | | -575.56 | | -487.26 | | -4244.04 | | -1439.8 | |
| NA |  |  | -41.4 | -127.89 | |  | -48.77 |  | -8.47 | NA |  |  | -26.45 | -144.49 | |
| NA |  |  | -41.4 | -127.89 | |  | -48.77 |  | -8.47 | NA |  |  | -26.45 | -144.49 | |
| NA |  | NA |  |  | -56.76 | NA |  | NA |  | NA |  | NA |  |  | -69.9 |
| NA |  | NA |  |  | -56.76 | NA |  | NA |  | NA |  | NA |  |  | -69.9 |
| NA |  | NA |  |  | -9.5 | NA |  | NA |  | NA |  | NA |  |  | -7.53 |
| NA |  |  | -29.73 |  | -58.02 |  | -20.07 | NA |  | NA |  |  | -14.39 |  | -60.24 |
| NA |  | NA |  |  | -6.88 | NA |  | NA |  | NA |  | NA |  |  | -19.65 |
| NA |  | NA |  | NA |  | NA |  | NA |  | NA |  | NA |  | NA |  |
| NA |  | NA |  | NA |  | NA |  | NA |  | NA |  | NA |  | NA |  |
| NA |  | NA |  | NA |  | NA |  | NA |  | NA |  | NA |  | NA |  |
| NA |  | NA |  | NA |  | NA |  |  | -14.07 | NA |  | NA |  | NA |  |
| NA |  | NA |  | NA |  | NA |  |  | -24.76 | NA |  | NA |  | NA |  |
| NA |  | NA |  | NA |  | NA |  |  | -22.92 | NA |  | NA |  | NA |  |
| NA |  | NA |  | NA |  | NA |  |  | -24.87 | NA |  | NA |  | NA |  |
|  | -35.75 |  | -89.04 |  | -14.57 |  | -52.69 | -364.79 | | -510.64 | |  | -42.07 |  | -16.31 |
| -145.61 | | -215.89 | | -351.84 | | -135.73 | | -113.34 | | -189.27 | | -108.84 | | -525.49 | |
| -145.61 | | -215.89 | | -351.84 | | -135.73 | | -113.34 | | -189.27 | | -108.84 | | -525.49 | |

| -361.51 | |  | -328.9 | -1027.52 | | -199.42 | | -128.09 | |  | -226.4 | -228.05 | |  | -1747 |
| --- | --- | --- | --- | --- | --- | --- | --- | --- | --- | --- | --- | --- | --- | --- | --- |
|  | -6.68 |  | -7.61 | -187.95 | | NA |  | NA |  | NA |  | NA |  |  | -304.3 |
| -196.01 | | -226.27 | |  | -498.7 | -294.86 | |  | -93.6 | -168.73 | | -165.12 | | -650.25 | |
|  | -44 |  | -92.28 |  | -8.81 |  | -43.25 |  | -275.5 | -424.39 | |  | -21.84 |  | -15.32 |
| -175.58 | | -382.89 | | -248.58 | |  | -331.7 | -163.43 | | -262.25 | | -230.57 | | -276.93 | |
|  | -71.24 | -497.36 | | NA |  | NA |  | NA |  | NA |  | -300.59 | | NA |  |
| NA |  | NA |  | NA |  | NA |  | NA |  | NA |  | NA |  | NA |  |
| NA |  | NA |  | NA |  | NA |  | NA |  | NA |  | NA |  | NA |  |
|  | -10.73 |  | -22.7 | NA |  | NA |  |  | -13.67 |  | -71.42 | NA |  | NA |  |
| NA |  |  | -8.6 |  | -14.08 | NA |  | NA |  |  | -13.18 | NA |  |  | -18.09 |
|  | -41.07 |  | -92.57 |  | -5.53 |  | -22.57 |  | -82.25 | -231.51 | |  | -34.72 | NA |  |
| NA |  | NA |  | NA |  | NA |  |  | -35.88 | NA |  | NA |  | NA |  |
| NA |  | NA |  | NA |  | NA |  | NA |  | NA |  | NA |  | NA |  |
| NA |  | NA |  | NA |  | NA |  | NA |  | NA |  | NA |  | NA |  |
| NA |  | NA |  | NA |  | NA |  | NA |  | NA |  | NA |  | NA |  |
| NA |  | NA |  |  | -17.51 | NA |  | NA |  | NA |  | NA |  |  | -14.22 |
| NA |  | NA |  | NA |  | NA |  |  | -20.69 | NA |  | NA |  | NA |  |
| NA |  | NA |  |  | -8.44 | NA |  |  | -60.1 |  | -5.96 | NA |  | NA |  |
| NA |  | NA |  | NA |  | NA |  |  | -27.43 | NA |  | NA |  | NA |  |
| NA |  | NA |  |  | -14.83 | NA |  | NA |  | NA |  | NA |  | NA |  |
| NA |  | NA |  | NA |  | NA |  | NA |  | NA |  | NA |  | NA |  |
| NA |  | NA |  | NA |  | NA |  |  | -29.12 | NA |  | NA |  | NA |  |
| NA |  | NA |  | NA |  | NA |  |  | -32.33 | NA |  | NA |  | NA |  |
| NA |  | NA |  | NA |  | NA |  |  | -60 | NA |  | NA |  | NA |  |
| NA |  | NA |  | NA |  | NA |  | NA |  | NA |  | NA |  | NA |  |
| NA |  | NA |  |  | -26.72 | NA |  | NA |  | NA |  | NA |  |  | -17.02 |
| NA |  | NA |  | NA |  | NA |  | NA |  | NA |  | NA |  | NA |  |
| NA |  | NA |  |  | -67.9 | NA |  | NA |  | NA |  | NA |  |  | -65.54 |
| NA |  | NA |  | NA |  | NA |  |  | -16.22 | NA |  | NA |  | NA |  |
| NA |  | NA |  | NA |  | NA |  |  | -17.16 | NA |  | NA |  | NA |  |
| NA |  | NA |  |  | -9.96 | NA |  |  | -48.36 |  | -58.38 | NA |  |  | -5.66 |
| NA |  | NA |  | NA |  | NA |  |  | -33.88 | NA |  | NA |  | NA |  |
| NA |  | NA |  | NA |  | NA |  | NA |  | NA |  | NA |  | NA |  |
| NA |  | NA |  | NA |  | NA |  | NA |  | NA |  | NA |  | NA |  |
| NA |  | NA |  | NA |  | NA |  | NA |  | NA |  | NA |  | NA |  |
| NA |  | NA |  |  | -50.46 | NA |  | NA |  | NA |  | NA |  |  | -62.51 |
| NA |  | NA |  | NA |  | NA |  | NA |  | NA |  | NA |  | NA |  |
| NA |  | NA |  |  | -54.93 | NA |  | NA |  | NA |  | NA |  |  | -88.08 |
| NA |  | NA |  | -100.93 | | NA |  | NA |  | NA |  | NA |  | -101.55 | |

| NA |  | NA |  | NA |  | NA |  |  | -52.18 | NA |  | NA |  | NA |  |
| --- | --- | --- | --- | --- | --- | --- | --- | --- | --- | --- | --- | --- | --- | --- | --- |
| NA |  | NA |  |  | -24.35 |  | -8.31 | NA |  | NA |  | NA |  | NA |  |
| NA |  | NA |  | NA |  | NA |  |  | -20.1 | NA |  | NA |  | NA |  |
| NA |  | NA |  | NA |  | NA |  | NA |  | NA |  | NA |  | NA |  |
| NA |  | NA |  | NA |  | NA |  | NA |  | NA |  | NA |  | NA |  |
| NA |  | NA |  |  | -61.18 | NA |  | NA |  | NA |  | NA |  |  | -93.78 |
| NA |  | NA |  | NA |  | NA |  | NA |  | NA |  | NA |  | NA |  |
| NA |  | NA |  |  | -56.23 | NA |  | NA |  | NA |  | NA |  |  | -53.2 |
| NA |  | NA |  | NA |  | NA |  |  | -36.44 | NA |  | NA |  | NA |  |
| NA |  | NA |  |  | -76.16 | NA |  |  | -7.56 | NA |  | NA |  |  | -89.76 |
| -275.76 | | -733.75 | | -911.21 | | -1037.66 | |  | -40.89 |  | -29.11 | -676.83 | | -692.38 | |
| NA |  |  | -17.29 | -932.62 | |  | -7.48 | -254.35 | |  | -76.39 |  | -7.37 | -1604.75 | |
| NA |  | NA |  | -980.46 | |  | -28.08 | NA |  | NA |  |  | -22.7 | -942.09 | |
|  | -92.19 | -161.45 | | -1918.62 | | -420.34 | | -106.53 | |  | -16.42 | -280.79 | | -2058.21 | |
| -120.64 | | -136.45 | | -1021.65 | | -245.89 | |  | -41.06 |  | -40.95 | -207.61 | | -1061.61 | |
|  | -79.76 |  | -99.56 | -995.65 | | -162.31 | |  | -22.41 |  | -19.91 | -143.38 | | -1255.63 | |
|  | -54.73 |  | -29.39 | -1307.42 | |  | -10.67 | NA |  | NA |  |  | -48.47 | -2163.95 | |
| NA |  | NA |  | -1081.67 | |  | -23.54 |  | -33.85 | NA |  |  | -30.74 | -1233.29 | |
| -257.92 | | -247.34 | | -2197.79 | | -512.85 | | -105.98 | |  | -81.12 | -468.37 | | -2303.03 | |
|  | -21.15 |  | -29.51 | -749.94 | |  | -72.13 |  | -6.03 | NA |  |  | -89.56 | -759.87 | |
| -170.98 | |  | -40.11 | -358.83 | |  | -5.84 | NA |  |  | -32.28 | -127.38 | | -301.92 | |
|  | -225.7 | -116.91 | | -313.19 | |  | -17.12 | NA |  |  | -41.73 | -211.73 | |  | -287.5 |
|  | -268.8 | -190.79 | | -465.31 | |  | -48.59 |  | -5.22 |  | -85.38 | -295.27 | | -391.42 | |
|  | -90.88 |  | -19.25 | -249.35 | | NA |  | NA |  | NA |  |  | -83.73 | -263.28 | |
| NA |  | NA |  |  | -6.03 |  | -72.42 | NA |  | NA |  | NA |  |  | -19.43 |
|  | -29.31 |  | -78.51 |  | -30.88 | -116.39 | |  | -80.93 | -134.16 | |  | -46.71 |  | -23.36 |
|  | -62.56 |  | -43.5 |  | -70.04 |  | -61.83 | NA |  | NA |  |  | -46.69 | -163.29 | |
|  | -46.17 |  | -26.15 | -196.74 | |  | -43.76 | NA |  | NA |  |  | -36.01 | -326.74 | |
|  | -46.17 |  | -26.15 | -196.74 | |  | -43.76 | NA |  | NA |  |  | -36.01 | -326.74 | |
|  | -19.65 | NA |  |  | -54.02 |  | -67.9 | NA |  | NA |  |  | -6.53 | -168.11 | |
|  | -22.49 |  | -8.74 |  | -40.01 | -254.41 | | NA |  | NA |  |  | -20.19 |  | -81.75 |
|  | -8.26 | NA |  |  | -11.23 |  | -82.73 | NA |  | NA |  |  | -5.96 |  | -41.53 |
|  | -8.26 | NA |  |  | -11.23 |  | -82.73 | NA |  | NA |  |  | -5.96 |  | -41.53 |
|  | -8.26 | NA |  |  | -11.23 |  | -82.73 | NA |  | NA |  |  | -5.96 |  | -41.53 |
|  | -35.15 |  | -19.04 |  | -18.25 | -285.25 | | NA |  | NA |  |  | -35.15 |  | -43.62 |
| -153.02 | | -158.95 | |  | -15.86 | -413.08 | | -111.84 | |  | -196.6 | -139.84 | |  | -25.22 |
| NA |  | NA |  |  | -24.45 |  | -28.2 | NA |  | NA |  | NA |  | -101.02 | |
| NA |  | NA |  | NA |  | NA |  |  | -47.88 |  | -7.2 | NA |  | NA |  |
| NA |  | NA |  | -155.59 | |  | -7.7 |  | -63.34 |  | -65.36 | NA |  | -205.33 | |

| NA |  | NA |  | NA |  | NA |  |  | -76.78 | NA |  | NA |  | NA |  |
| --- | --- | --- | --- | --- | --- | --- | --- | --- | --- | --- | --- | --- | --- | --- | --- |
| NA |  | NA |  | NA |  | NA |  |  | -12.57 | NA |  | NA |  | NA |  |
| NA |  | NA |  | NA |  | NA |  |  | -13.94 | NA |  | NA |  | NA |  |
| NA |  | NA |  | NA |  | NA |  |  | -18.06 | NA |  | NA |  | NA |  |
|  | -300.9 | -463.28 | | -115.79 | |  | -608.8 | -1758.04 | | -1922.57 | | -287.66 | |  | -52.88 |
|  | -187.9 | -364.06 | | -112.28 | |  | -203 | -259.89 | | -522.41 | | -212.35 | |  | -94.73 |
|  | -90.77 |  | -189.9 |  | -14.02 | -134.38 | | -135.29 | | -290.81 | |  | -88.01 |  | -28.57 |
| -301.39 | | -526.79 | | -214.29 | |  | -426.3 | -823.72 | | -1146.38 | | -341.48 | | -186.82 | |
| -150.42 | | -194.17 | | NA |  |  | -50.12 |  | -17.44 |  | -60.66 | -121.04 | | NA |  |
| -182.33 | |  | -260.5 | NA |  |  | -96.63 |  | -88.09 | -180.24 | | -171.71 | | NA |  |
| -194.77 | | -289.83 | | -1749.12 | | -299.94 | |  | -50.54 | -168.65 | | -174.04 | | -2287.56 | |
| -374.09 | | -289.03 | | NA |  | -169.18 | | -270.24 | | -411.07 | | -273.15 | | NA |  |
| -502.69 | | -601.22 | | NA |  | -364.03 | | -493.21 | | -639.29 | | -453.59 | | NA |  |
|  | -12.26 |  | -57.9 | -119.89 | |  | -79.79 | -175.28 | |  | -218.2 |  | -19.34 | -107.22 | |
| NA |  | NA |  | NA |  | NA |  |  | -94.66 |  | -34.87 | NA |  | NA |  |
| NA |  | NA |  | NA |  | NA |  |  | -28.67 | NA |  | NA |  | NA |  |
| -111.64 | | -162.37 | | -2407.98 | | -193.15 | | NA |  |  | -21.65 |  | -93.75 | -3402.76 | |
| NA |  | NA |  | NA |  | NA |  |  | -29.98 | NA |  | NA |  | NA |  |
| -158.73 | | -302.93 | | -332.26 | | -497.72 | | -1147.38 | | -1143.28 | | -174.04 | | -212.94 | |
| NA |  | NA |  | NA |  | NA |  |  | -81.61 |  | -34.31 | NA |  | NA |  |
| NA |  | NA |  | NA |  | NA |  |  | -81.61 |  | -34.31 | NA |  | NA |  |
|  | -142 | -201.25 | | -183.42 | | -211.77 | | -169.96 | | -302.64 | | -133.62 | | -244.97 | |
| -178.15 | | -297.08 | | -2033.37 | | -282.39 | |  | -84.83 |  | -246.3 | -177.63 | | -2786.51 | |
| -178.15 | | -297.08 | | -2033.37 | | -282.39 | |  | -84.83 |  | -246.3 | -177.63 | | -2786.51 | |
| -178.15 | | -297.08 | | -2033.37 | | -282.39 | |  | -84.83 |  | -246.3 | -177.63 | | -2786.51 | |
| -178.15 | | -297.08 | | -2033.37 | | -282.39 | |  | -84.83 |  | -246.3 | -177.63 | | -2786.51 | |
| -195.83 | | -438.61 | | -237.67 | | -428.61 | | -290.63 | | -458.55 | | -280.06 | | -232.37 | |
| -195.83 | | -438.61 | | -237.67 | | -428.61 | | -290.63 | | -458.55 | | -280.06 | | -232.37 | |
| -195.83 | | -438.61 | | -237.67 | | -428.61 | | -290.63 | | -458.55 | | -280.06 | | -232.37 | |
| -195.83 | | -438.61 | | -237.67 | | -428.61 | | -290.63 | | -458.55 | | -280.06 | | -232.37 | |
|  | -71.87 | -169.25 | | -1211.69 | |  | -169.7 | -176.68 | | -114.92 | | -145.93 | | -1795.36 | |
|  | -71.87 | -169.25 | | -1211.69 | |  | -169.7 | -176.68 | | -114.92 | | -145.93 | | -1795.36 | |
|  | -44.3 | -100.76 | | -537.22 | | -133.89 | | -103.34 | |  | -71.08 | -107.18 | | -829.66 | |
|  | -44.3 | -100.76 | | -537.22 | | -133.89 | | -103.34 | |  | -71.08 | -107.18 | | -829.66 | |
| -353.98 | | -489.55 | | -3213.14 | |  | -459.3 | -162.43 | | -364.39 | |  | -322.1 | -4575.18 | |
| -240.03 | | -406.71 | | -276.38 | |  | -694.3 | -1647.38 | | -1818.12 | | -247.23 | | -161.48 | |
| -240.03 | | -406.71 | | -276.38 | |  | -694.3 | -1647.38 | | -1818.12 | | -247.23 | | -161.48 | |
| -147.99 | | -312.89 | | -317.25 | | -513.94 | | -1034.33 | | -1085.44 | | -211.89 | | -168.48 | |
| -147.99 | | -312.89 | | -317.25 | | -513.94 | | -1034.33 | | -1085.44 | | -211.89 | | -168.48 | |

| -176.77 | | -348.19 | |  | -299.3 | -681.61 | | -1123.16 | | -1146.24 | |  | -211.9 | -173.67 | |
| --- | --- | --- | --- | --- | --- | --- | --- | --- | --- | --- | --- | --- | --- | --- | --- |
| -334.12 | | -557.66 | | -318.99 | | -931.99 | | -1944.96 | | -2221.1 | | -330.52 | | -168.51 | |
|  | -148.7 | -288.41 | | -247.97 | |  | -544.5 | -1265.57 | | -1364.21 | | -177.29 | | -164.69 | |
|  | -37.89 |  | -85.96 |  | -89.07 | -161.74 | | -397.59 | | -421.07 | |  | -54.54 |  | -45.01 |
| -240.85 | | -397.43 | | -243.64 | | -125.68 | |  | -61.33 | -170.52 | | -361.86 | | -168.11 | |
| -128.64 | |  | -26.98 | -241.33 | | NA |  | NA |  |  | -12.22 |  | -92.73 | -243.68 | |
| -279.71 | | -486.74 | | -130.61 | | -165.61 | |  | -85.21 | -266.57 | | -353.22 | | -114.28 | |
| -160.75 | | -591.18 | | NA |  | -197.14 | | -2369.1 | | -3427.17 | | -213.65 | | NA |  |
| -220.69 | | -684.68 | |  | -7.41 | -244.95 | | -2685.84 | | -3857.33 | | -267.79 | | NA |  |
|  | -7.56 |  | -49.55 | NA |  | NA |  | -482.06 | | -552.59 | |  | -6.15 | NA |  |
| -196.81 | | -673.94 | |  | -11.5 | -224.01 | | -2917.3 | | -4081.24 | | -244.42 | |  | -5.57 |
|  | -71.44 | -277.47 | |  | -24.77 |  | -115.7 | -1896.3 | | -2248.25 | | -114.28 | |  | -15.58 |
| NA |  | NA |  | -133.95 | |  | -36.44 | NA |  | NA |  | NA |  | -178.77 | |
|  | -74.13 | -137.63 | | -335.05 | | -232.78 | | NA |  |  | -17.48 | -145.98 | | -343.23 | |
| NA |  | NA |  | -133.36 | |  | -7.34 | NA |  | NA |  | NA |  | -196.24 | |
| NA |  | NA |  | NA |  | NA |  | NA |  | NA |  | NA |  | NA |  |
| NA |  | NA |  | NA |  | NA |  | NA |  | NA |  | NA |  | NA |  |
| NA |  |  | -5.65 |  | -36.88 |  | -15.88 |  | -10.05 |  | -29.33 | NA |  |  | -35.26 |
| NA |  | NA |  | NA |  | NA |  | NA |  | NA |  | NA |  | NA |  |
| NA |  | NA |  | NA |  | NA |  | NA |  | NA |  | NA |  | NA |  |
| NA |  | NA |  | NA |  | NA |  | NA |  | NA |  | NA |  | NA |  |
| NA |  | NA |  | NA |  | NA |  | NA |  | NA |  | NA |  | NA |  |
| NA |  | NA |  |  | -20.29 | NA |  | NA |  | NA |  | NA |  |  | -15.81 |
| NA |  | NA |  |  | -47.09 |  | -17.24 | NA |  | NA |  | NA |  |  | -31.21 |
|  | -23.54 |  | -63.59 | -120.18 | | -131.42 | | NA |  | NA |  |  | -56.02 |  | -117.6 |
| NA |  | NA |  | NA |  | NA |  |  | -32.21 | NA |  | NA |  | NA |  |
| NA |  | NA |  | NA |  | NA |  |  | -24.56 | NA |  | NA |  | NA |  |
| NA |  | NA |  | NA |  | NA |  |  | -38.4 | NA |  | NA |  | NA |  |
| -258.73 | | -430.88 | | -3189.38 | | -471.82 | | -157.44 | | -339.16 | | -291.37 | | -4964.9 | |
|  | -80.57 | -122.09 | |  | -353.2 | -417.99 | | -119.47 | | -259.38 | |  | -93.81 | -487.15 | |
|  | -80.57 | -122.09 | |  | -353.2 | -417.99 | | -119.47 | | -259.38 | |  | -93.81 | -487.15 | |
| NA |  | NA |  | NA |  | NA |  | NA |  | NA |  | NA |  |  | -18.98 |
| NA |  |  | -22.49 | NA |  | NA |  | NA |  | NA |  | NA |  | NA |  |
| NA |  | NA |  | NA |  | NA |  | NA |  | NA |  | NA |  |  | -24.9 |
| NA |  | NA |  |  | -26.01 |  | -22.29 | NA |  | NA |  | NA |  |  | -30.77 |
|  | -536.4 | -1083.5 | |  | -98.88 | -690.79 | | -101.41 | |  | -98.06 | -740.42 | |  | -68.34 |
| NA |  |  | -15.86 |  | -10.09 | NA |  | NA |  | NA |  | NA |  |  | -10.77 |
| NA |  | NA |  | NA |  | NA |  | NA |  | NA |  | NA |  |  | -43.58 |
| NA |  |  | -13.08 |  | -13.64 |  | -9.01 | NA |  | NA |  | NA |  |  | -24.6 |

|  | -5.2 NA |  | -42.21 | -12.53 NA | NA | NA |  | -63.51 |
| --- | --- | --- | --- | --- | --- | --- | --- | --- |
| NA | NA |  | -8.41 NA | NA | NA | NA |  | -7.27 |
| NA | NA |  | -14.78 NA | NA | NA | NA |  | -17.41 |
| NA | NA | NA | NA | NA | NA | NA |  | -10.39 |
| NA |  | -5.24 NA | NA | NA | NA | NA | NA |  |

-186.17	-384.02	-129.35	-195.68	-276.81	-506.28	-234.29	-105.02

NA	NA	NA	NA	NA	NA	NA	NA NA	NA	NA	NA	NA	NA	NA	NA NA	NA	NA	NA	NA	NA	NA	NA NA	NA	NA	NA	NA	NA	NA	NA

-64.26	-76.52	-502.92	-301.4	-28.17	-52.37	-38.86	-502.58

-27.03	-58.55	-500.79	-146.47 NA	-33.73	-38.01	-662.39

-177.83	-273.98	-420.71	-173.56	-38.39	-194.42	-209.9	-552.51

NA	NA	NA	NA	-8.42 NA	NA	NA

-39.86	-59.79	-274.4	-222.69	-32.48	-64.92	-29.47	-319.84

-7.25	-14.25	-480.36	-130.7	-41.13	-54.46	-16.24	-506.73

-27.68	-31.62	-208.07	-51.86	-112.76	-195.65	-28.46	-315.57

-27.33	-84.71	-14.18	-85.37	-69.87	-142.86	-33.52	-6.42

NA	NA	NA	NA	-18.56 NA	NA	NA

-137.15	-157.38 NA	-217.13 NA	-39.02	-109.97	-22.25

-160.88	-223.8	-71.8	-376.14	-70.14	-184.05	-152.39	-85.42

-15.55	-49.01	-35.31	-60.55 NA	-10.11	-16.98	-53.6

-13.68	-33.1	-32.34	-31.63 NA	-7.68	-10.48	-54.86

-656.19	-809.15 NA	-527.6	-279.1	-343.79	-645.35 NA

-95.6	-191.22	-150.75	-271.64	-321.96	-426.75	-102.91	-137.58

-9.33	-11.21	-19.52	-11.59 NA	NA	-8.75	-49.81

-84.8	-159.61	-463.08	-263.78 NA	-40.28	-152.42	-469.5

-23	-67.53	-215.28	-73.92	-15.48	-21.07	-40.13	-270.3

-190.1	-354.37	-211.29	-216.19	-235.77	-431.36	-226.27	-250.76

-43.82	-88.16	-32.96	-36.95	-157.31	-253.94	-43.89	-41.32

-31.71	-71.21	-72.54	-54.98	-233.57	-296.65	-54.73	-79.81

-52.52	-105.88	-64.91	-58.87	-297.98	-384.37	-67.16	-84.39

-80.43	-125.05	-74.7	-70.06	-230.18	-339.7	-93.19	-82.6

-54.9	-158.61	-34.43	-141.68	-330.92	-476.83	-104.99	-23.73

-29.14	-55.78	-50.79	-33.51	-277.31	-357.69	-41.08	-51.52

NA	-10.75	-16.79 NA	-153.1	-202.98	-5.54	-23.87

NA	NA	NA	NA	NA	NA	NA	NA NA	NA	NA	NA	NA	NA	NA	NA NA	NA	NA	NA	NA	NA	NA	NA

| -373.68 | | -906.44 | | -857.28 | | -1327.45 | | NA |  | NA |  | -729.07 | | -655.74 | |
| --- | --- | --- | --- | --- | --- | --- | --- | --- | --- | --- | --- | --- | --- | --- | --- |
| -524.42 | | -1156.09 | | -934.33 | | -1673.09 | | NA |  |  | -17.81 | -989.01 | | -720.22 | |
| -263.66 | | -505.83 | | -217.79 | | -926.87 | | NA |  |  | -7.62 | -461.12 | | -151.41 | |
|  | -8.84 |  | -11.67 |  | -17.17 |  | -18.6 | NA |  | NA |  | NA |  |  | -25.16 |
| NA |  | NA |  | NA |  | NA |  | NA |  | NA |  | NA |  |  | -5.23 |
| NA |  | NA |  | NA |  | NA |  | NA |  | NA |  | NA |  |  | -6.54 |
| NA |  | NA |  | NA |  | NA |  | NA |  | NA |  | NA |  |  | -9 |
| NA |  |  | -19.5 | -167.91 | | NA |  | NA |  | NA |  |  | -12.3 | -196.26 | |
|  | -70.14 | -167.36 | |  | -30.71 | -177.49 | |  | -350 | -537.59 | |  | -82.81 |  | -30.45 |
|  | -47.42 | -133.61 | |  | -53.73 | -138.95 | | -329.33 | | -565.11 | |  | -55.15 |  | -73.9 |
|  | -73.71 | -183.71 | |  | -24.75 | -123.78 | |  | -494.7 | -741.21 | |  | -80.42 |  | -14.73 |
|  | -73.71 | -183.71 | |  | -24.75 | -123.78 | |  | -494.7 | -741.21 | |  | -80.42 |  | -14.73 |
| -109.28 | |  | -202.3 | -111.16 | | -133.07 | |  | -366.4 | -616.15 | | -109.04 | | -116.92 | |
| -175.88 | | -115.01 | | -245.95 | |  | -8.46 | NA |  |  | -10.05 | -156.63 | | -206.23 | |
| -175.88 | | -115.01 | | -245.95 | |  | -8.46 | NA |  |  | -10.05 | -156.63 | | -206.23 | |
| NA |  |  | -5.9 |  | -22.57 | NA |  | -336.93 | | -576.65 | | NA |  |  | -11.05 |
| NA |  | NA |  |  | -30.63 | NA |  | -367.98 | | -491.05 | | NA |  |  | -37.6 |
| NA |  | NA |  | NA |  | NA |  | -402.24 | | -992.66 | | NA |  | NA |  |
|  | -9.88 | NA |  |  | -26.96 | -180.87 | | NA |  | NA |  |  | -7.11 |  | -89.12 |
| -998.47 | | -2370.58 | | -882.12 | | -1937.11 | | -329.32 | | -228.93 | | -1625.26 | | -674.04 | |
| -1303.5 | | -3177.92 | | -3734.44 | | -3783.88 | |  | -67.46 |  | -15.65 | -2791.07 | | -2933.16 | |
|  | -322.5 |  | -881.7 | -1548.34 | | -1580.13 | |  | -58.7 |  | -6.82 | -933.96 | | -1219.39 | |
| -980.09 | | -2281.63 | | -1741.39 | | -2535.9 | | -190.15 | | -174.26 | | -1833.29 | | -1355.36 | |
| -2037.87 | | -4728.33 | | -4794.97 | | -4951.99 | | -123.83 | |  | -58.23 | -4249.13 | | -3753.39 | |
| -1157.43 | | -3063.19 | | -3210.83 | | -3600.91 | | -110.15 | |  | -34.87 | -2633.67 | | -2575.29 | |
|  | -83.31 | -161.47 | | -178.19 | |  | -98.58 |  | -135.8 | -159.46 | | -145.11 | | -133.37 | |
| -112.17 | | -174.52 | | -2273.87 | | -465.02 | | -122.56 | |  | -18.95 | -349.76 | | -2161.69 | |
|  | -88.51 | -198.31 | |  | -97.35 | -109.51 | |  | -159.4 | -256.96 | | -116.43 | |  | -86.57 |
|  | -8.65 |  | -49.26 | -123.74 | |  | -64.15 |  | -96.39 | -101.04 | |  | -27.68 | -120.11 | |
| -239.19 | | -432.97 | | -280.21 | | -789.22 | | -1249.85 | | -1433.22 | | -225.97 | | -196.38 | |
| -179.93 | | -340.05 | | -306.94 | | -500.62 | | -1307.17 | | -1321.41 | | -222.97 | | -185.75 | |
| NA |  | NA |  | -224.47 | | -171.68 | | -170.47 | |  | -96.28 | NA |  | -181.62 | |
| NA |  | NA |  | -134.11 | | NA |  |  | -54.81 |  | -38.35 | NA |  | -149.53 | |
| NA |  | NA |  | NA |  | NA |  | NA |  | NA |  | NA |  |  | -5.95 |
| NA |  | NA |  | NA |  | NA |  | NA |  | NA |  | NA |  | NA |  |
| -118.24 | | -141.83 | | NA |  |  | -108.7 |  | -13.55 |  | -63.03 |  | -70.6 | NA |  |
| -191.84 | | -296.76 | |  | -25.04 | -291.66 | | -114.04 | | -301.23 | | -163.69 | |  | -22.7 |
| -234.97 | | -257.47 | | NA |  | -270.95 | |  | -57.78 | -205.24 | |  | -164.1 | NA |  |
| NA |  | NA |  | NA |  | NA |  | NA |  | NA |  | NA |  | NA |  |

| -195.59 | | -274.08 | | NA |  | -264.23 | |  | -72.31 | -206.41 | | -160.76 | | NA |  |
| --- | --- | --- | --- | --- | --- | --- | --- | --- | --- | --- | --- | --- | --- | --- | --- |
| -361.85 | | -599.43 | | -147.37 | | -723.04 | | -1486.28 | | -2018.89 | | -359.59 | |  | -84.14 |
|  | -177.3 |  | -243.9 | -1748.13 | | -206.09 | |  | -20.05 | -110.74 | | -152.79 | | -2472.67 | |
| -158.59 | | -225.38 | | -1317.21 | | -164.85 | |  | -18.79 |  | -93.52 |  | -153.6 | -1884.88 | |
| -108.58 | | -170.37 | | -733.41 | |  | -83.74 |  | -8.07 |  | -75.08 |  | -97.18 | -1042.59 | |
|  | -21.65 |  | -59.91 |  | -60.92 |  | -77.84 | -112.93 | | -151.46 | |  | -26.7 |  | -46.79 |
|  | -37.46 | -197.92 | | NA |  |  | -11.23 | -545.88 | | -814.41 | |  | -48.78 | NA |  |
| -178.49 | | -275.32 | | -1189.34 | | -199.03 | |  | -27.13 | -142.49 | | -178.33 | | -1709.16 | |
| -121.79 | | -171.14 | | -874.57 | | -115.68 | |  | -9.51 |  | -73.26 | -101.94 | | -1251.22 | |
| NA |  | NA |  | NA |  | NA |  |  | -48.05 | NA |  | NA |  | NA |  |
| NA |  | NA |  | NA |  | NA |  |  | -63.76 | NA |  | NA |  | NA |  |
| NA |  | NA |  | NA |  | NA |  |  | -10.39 | NA |  | NA |  | NA |  |
| -719.17 | | -737.57 | | NA |  | -670.41 | | -322.69 | | -553.16 | |  | -599.6 |  | -34.67 |
| -242.48 | | -298.33 | | -491.06 | | -278.12 | | -195.91 | | -347.48 | | -214.66 | | -778.52 | |
| NA |  |  | -22.47 |  | -59.5 |  | -67.97 | -186.41 | | -143.29 | |  | -6.4 |  | -33.03 |
| -148.17 | |  | -164.2 | -522.82 | | -265.99 | |  | -55.39 | -127.63 | | -127.91 | | -914.47 | |
| NA |  |  | -15.66 |  | -55.57 |  | -60.14 | -144.43 | | -117.65 | |  | -5.12 |  | -28.36 |
|  | -29.15 |  | -6.72 | -134.52 | |  | -52.24 | NA |  | NA |  | NA |  | -294.01 | |
| -102.27 | | -130.91 | |  | -8.36 | -110.24 | |  | -58.27 | -150.25 | |  | -67.6 |  | -21.67 |
| NA |  | NA |  | NA |  | NA |  |  | -6.33 | NA |  | NA |  | NA |  |
| -469.14 | | -624.51 | | -344.87 | | -417.92 | | -737.72 | | -1008.93 | | -471.21 | | -547.65 | |
|  | -5.85 |  | -41.75 |  | -41.26 |  | -12.45 |  | -71.57 |  | -76.9 |  | -15.29 |  | -51.65 |
|  | -37.48 |  | -7.27 | NA |  | NA |  | NA |  | NA |  | NA |  | NA |  |
|  | -40.36 |  | -6.07 | NA |  | NA |  | NA |  | NA |  | NA |  |  | -39.07 |
| -173.34 | | -167.54 | |  | -29.53 | -243.68 | |  | -19.13 |  | -70.63 | -121.09 | |  | -56.61 |
|  | -64.72 |  | -83.17 | NA |  | -364.12 | |  | -34.17 | -137.08 | |  | -84.8 | NA |  |
| -173.89 | | -173.27 | | NA |  | -287.95 | |  | -69.56 | -157.83 | | -163.87 | |  | -7.42 |
| -181.78 | | -237.47 | | NA |  | -251.32 | |  | -47.91 | -151.25 | |  | -156 | NA |  |
|  | -16.79 |  | -39.97 |  | -20.28 | -103.22 | |  | -5.22 |  | -28.8 |  | -17.6 |  | -79.34 |
| -231.81 | | -309.59 | | -355.09 | | -217.37 | | -238.43 | | -288.42 | | -262.83 | | -686.03 | |
|  | -84.24 | -136.25 | |  | -473.3 |  | -321.1 |  | -48.82 |  | -63.29 |  | -91.59 | -806.97 | |
| -266.12 | | -437.82 | | -174.78 | | -349.99 | | -434.03 | | -735.23 | | -298.24 | | -215.61 | |
|  | -59.31 | -152.18 | |  | -77.82 | -146.16 | |  | -204.8 |  | -349.9 | -108.86 | |  | -86.54 |
|  | -24.4 | -112.49 | | -151.24 | | -189.27 | | -231.65 | | -270.42 | |  | -86 | -177.62 | |
| -160.84 | |  | -92.15 |  | -43.13 |  | -77.4 |  | -23.6 |  | -23.88 |  | -108.3 |  | -251.4 |
|  | -93.68 | -254.17 | | -465.73 | | -238.24 | | -137.45 | | -121.49 | | -182.98 | | -521.55 | |
| -234.35 | | -633.58 | | -679.55 | | -512.36 | | -153.08 | | -108.31 | | -474.98 | | -629.09 | |
|  | -67.12 |  | -97.32 |  | -24.84 | -121.29 | |  | -98.3 | -211.12 | |  | -58.25 |  | -32.98 |
| -388.96 | | -327.62 | | NA |  | -230.69 | | -380.97 | | -549.02 | | -274.26 | |  | -6.92 |

| -83.77 | -215.52 NA |  | -152.18 | -140.82 | -269.45 | -133.14 NA |  |
| --- | --- | --- | --- | --- | --- | --- | --- |
| -159.44 | -283.49 | -35.25 | -383.69 | -207.56 | -389.11 | -211.86 | -17.46 |

-182.61	-335.77	-836.4	-231.07	-341.33	-466.08	-249.41	-1153.91

-31.46	-59.15 NA	-87.99	-39.95	-93.72	-55.13	-12.16

-524.59	-940.48	-520.06	-496.41	-466.92	-202.51	-817.8	-1232.84

NA	NA	-53.85 NA	-240.32 NA	NA	-146.71

-255.86	-463.8	-195.82	-420.98	-526.05	-782.1	-292.97	-143.26

NA	-194.67	-10.1	-163.06	-574.14	-731.69	-108.96 NA

-159.99	-259.91	-56.53	-169.08	-267.41	-484.22	-132.78	-132.98

-52.32	-103.7	-8.07	-131.07	-120.43	-181.85	-81.37 NA

-81.57	-132.78 NA	-193.68	-134.55	-285.27	-75.64	-7.9

NA	NA	NA	NA	NA	NA	NA	NA

-81.95	-262.4	-68.23	-182.39	-129.05	-160.54	-136.58	-103.53

-40.75	-21.66	-51.47	-172.29 NA	NA	-27.79	-129.42

-105.78	-83.48	-233.1	-315.5	-5.74	-18.27	-88.29	-390.83

-499.24	-728.8	-140.19	-651.19	-1311.06	-1766.15	-451.98	-86.63

-34.09	-35.45	-26.57	-42.33	-113.56	-224.07	-20.35	-63.33

-247.51	-323.64	-21.53	-241.4	-394.3	-690.3	-167.81	-24.93

NA	NA	NA	NA	-39.76 NA	NA	-10.54

-27.02	-92.96	-23.69	-124.8	-60.34	-85.06	-64.68	-25.01

-38.37	-113.23	-131.66	-318.54	-146.89	-315.17	-78.15	-185.01

NA	-40.13	-363.42	-86.5	-24.89 NA	-22.21	-482.67

-126.96	-226.19	-28.53	-147.93	-203.5	-340.92	-131.24	-51.59

-93.7	-248.66	-49.83	-396.14	-7.66	-50.69	-183.07	-77.43

-7.49	-213.85	-21.67	-180.51	-301.59	-441.72	-102.67	-19.53

-106.3	-256.31	-685.7	-251.05	-101.37	-64	-198.43	-970.19

-79.66	-206.42	-55.73	-128.72	-338.34	-517.76	-149.55	-85.33

-46.26	-52.09	-16.7	-307.05	-24.29	-102.25	-38.69	-38.83

-60.49	-135.89	-85.59	-83.21	-172.13	-387.13	-65.6	-75.7

-10.33	-57.25	-40.82	-49.06	-122.25	-193.65	-28.09	-46.4

-85.53	-197.88	-275.06	-351.3	-38.27	-62.76	-170.27	-302.12

-385.61	-300.86	-264.87	-336.47	-304.84	-386.49	-270.24	-740.29

-85.03	-116.66	-23.19	-213.11	-40.27	-135.8	-69.36	-63.93

-42.36	-116.72	-1040.77	-202.27 NA	-34.56	-103.71	-948.61

NA	NA	-11.6	-11.97 NA	NA	-40.86	-167.63

-66.05	-118.75	-52.25	-103.65	-130.79	-250.49	-72.11	-48.43

-85.88	-175.52	-123.4	-114.33	-108.2	-202.03	-102.45	-140.7

-58.49	-88.14	-126.42	-363.27	-107.41	-177.42	-51.12	-179.41

cd34_negacd38_negacd3_negat cd4_naive. cd4_positi cd4_positi cd4_positi cd4_positi

-101.39	-109.9	-198.17	-156.54	-166.19	-21.2	-217.06	-112.25

NA	NA	NA	NA	NA	NA	NA	NA

-49.47	-107.8	-219.33	-237.67	-226.33	-120.98	-272.15	-230.71

-206.52	-258.78	-83.33	-168.56	-193.1	-100.07	-133.1	-90.6

-8.7	-60.05	-16.87	-17.53	-176.29	-6.46	-18.5	-21.39

-321.61	-625.48	-1288.09	-548.66	-480.25	-139.83	-1110.11	-434.14

-79.2	-134.01	-120.11	-134.38	-489	-68.74	-157.37	-177.43

-30.42	-40.14	-32.93	-43.26	-185.9	-13.58	-49.51	-33.84

-30.42	-40.14	-32.93	-43.26	-185.9	-13.58	-49.51	-33.84

NA	NA	NA	-6.88	-5.81 NA	NA	-10.58

-172.21	-301.91	-590.54	-181.65	-308.03	-53.83	-484.44	-205.61

-43.73	-207.66	-413.53	-102.35	-277.89	-11.48	-326.21	-88.98

-49.6	-43.96	-26.41	-29.52	-132.13	-28.5	-27.18	-165.9

-71.12	-57.27	-52.84	-54.81	-297.87	-43.9	-57.54	-239.25

NA	NA	NA	NA	NA	NA	NA	NA NA	NA	NA	NA	NA	NA	NA	NA

NA	NA	NA	-13.96	-14.87	-40.34 NA	-176.7

NA	NA	NA	-13.96	-14.87	-40.34 NA	-176.7

-226.44	-315.92	-1767.63	-1681.59	-389.78	-757.81	-1885.92	-1930.1

-45.32	-127.63	-254.14	-220.19	-394.69	-100.17	-278.96	-218.07

NA	NA	NA	NA	NA	NA	NA	NA

-419.39	-476.08	-2646.25	-2060.46	-753.51	-748.66	-2561.16	-2197.24

NA	NA	NA	NA	NA	NA	NA	NA NA	NA	NA	NA	NA	-5.18 NA	NA

NA	NA	NA	NA	NA	-9.6 NA	-16.44

NA	NA	NA	NA	NA	NA	NA	NA NA	NA	NA	NA	NA	NA	NA	NA NA	NA	NA	NA	NA	NA	NA	NA

-49.66	-113.09	-210.12	-215.86	-345.33	-97.1	-249.9	-277.71

-49.66	-113.09	-210.12	-215.86	-345.33	-97.1	-249.9	-277.71

-173.49	-220.36	-311.94	-281.26	-732.6	-151.48	-338.45	-319.92

-150.97	-188.89	-339.88	-294.71	-680.42	-159.15	-372.4	-360.85

NA	NA	NA	NA	NA	-16.79 NA	-13.72

NA	NA	NA	NA	NA	-6.02 NA	NA NA	NA	NA	NA	NA	NA	NA	NA NA	NA	NA	NA	NA	NA	NA	NA NA	NA	NA	NA	NA	NA	NA	NA

-189.65	-129.19	-158.83	-110.19	-134.61	-18.64	-140.28	-41.7

| -25.21 | -258.95 | -39.91 NA | -208.27 NA |  | -16.59 NA |
| --- | --- | --- | --- | --- | --- |
| -25.21 | -258.95 | -39.91 NA | -208.27 NA |  | -16.59 NA |
| -25.21 | -258.95 | -39.91 NA | -208.27 NA |  | -16.59 NA |
| -56.92 | -32.7 | -162.38 -231.77 | -398.6 | -15.04 | -261.25 -236.02 |
| -84.99 | -44.46 | -120.51 -130.43 | -290.92 NA |  | -151.2 -101.51 |

-822.05	-739.44	-3448.88	-3117.78	-1205.79	-1039.53	-3615.21	-2861.3

-1194.97	-1080.37	-4309.34	-4167.55	-1320.77	-1820.69	-4607.31	-4071.45

-1713.76	-1691.97	-6239.49	-5866.72	-1955.66	-2468.83	-6604.46	-5644.55

-1210	-1082.52	-4666.63	-4132.15	-1577.55	-1500.68	-4811.19	-3991.57

-828.58	-740.59	-2733.22	-2679.09	-894.66	-1253.41	-2933.82	-2580.42

-1066.24	-1073.11	-3541.8	-3236.9	-1085.08	-1500.74	-3653.48	-3190.91

-1290.44	-1299.46	-5424.39	-5048.52	-1389.32	-2362.76	-5672.53	-4998

-1369.54	-1267.51	-3815.12	-3651.27	-1341.54	-1770.5	-4016.5	-3523.92

-1515.29	-1435.73	-4887.24	-4859.05	-1638.08	-2151.48	-5300.35	-4915.22

-1806.86	-1711.28	-5716.52	-5564.22	-1883.13	-2551.83	-6101.58	-5385.21

NA	NA	NA	NA	NA	NA	NA	NA

-15.31	-35.85	-43.8	-51.95	-467.59	-27.62	-53.71	-84.23

-1808.93	-1699.6	-5907.83	-5887.14	-1843.35	-2588.4	-6302.33	-5866.87

-2025.74	-2031.14	-6901.27	-6812.59	-2248.54	-3008.48	-7476.03	-6807.64

-199.97	-184.37	-402.32	-370.57	-161.72	-201.61	-406.2	-382.48

-2159.65	-2190.98	-8510.94	-7899.54	-2594.18	-3144.39	-8998.06	-7762.48

NA	NA	NA	NA	NA	NA	NA	NA NA	NA	NA	NA	NA	NA	NA	NA

-2015.57	-2045.75	-7688.4	-7483.75	-2356.48	-3128.63	-8324.15	-7388.35

-104.61	-86.18	-228.45	-215.43	-132.78	-80.67	-247.55	-197.71

-883.68	-764.09	-3376.14	-3162.42	-1044.67	-1211.67	-3577.19	-3021.77

-2037.57	-2044.2	-7045.57	-6869.47	-2229.66	-2946.24	-7591.17	-6658.15

-1487.8	-1436.37	-5472.21	-5512.09	-1593.65	-2626.16	-5826.91	-5521.58

-723.48	-730.97	-2145.14	-2222.06	-743.33	-1071.42	-2283.15	-2214.22

-723.48	-730.97	-2145.14	-2222.06	-743.33	-1071.42	-2283.15	-2214.22

-2100.43	-2131.9	-7779.67	-7469.55	-2386.11	-3147.97	-8252.79	-7235.86

-989.97	-955.7	-3222.8	-3053.41	-1099.24	-1154.11	-3404.42	-2935.91

-580.08	-556.73	-1774.14	-1576.2	-519.27	-527.92	-1739.68	-1319.31

-601.31	-593.88	-2344.49	-2284.59	-921.65	-850.99	-2611.45	-2291.06

NA	NA	NA	NA	NA	NA	NA	NA

-67.1	-187.37	-396.79	-115.62	-210.81	-14.92	-329.42	-129.39

-1741.18	-1683.22	-7204.85	-7330.24	-2102.59	-3194.1	-7937.92	-7260.13

-2253.67	-2294.51	-8454.59	-8047.11	-2586.16	-3331.23	-9014.75	-7916.86

-233.84	-220.28	-466.03	-454.32	-203.28	-227.54	-487.53	-460.52

-27.84	-7.59	-13.75	-18.95	-218.68	-47.71	-22.11	-254.63

-13.61	-9.63	-44.4	-75.32	-166.75	-79.91	-68.78	-342.64

-60.78	-35.26	-85.14	-90.95	-297.99	-83.12	-96.01	-392.23

-10.7 NA	NA	NA	-198.45	-9.41 NA	-94.69

NA	NA	NA	NA	NA	NA	NA	NA NA	NA	NA	NA	NA	NA	NA	NA NA	NA	NA	NA	NA	NA	NA	NA NA	NA	NA	NA	NA	-6.82 NA	NA

NA	NA	NA	-7.43 NA	-10.44 NA	-8.09

NA	NA	NA	-5.64 NA	-12.27 NA	-8.39

NA	NA	NA	NA	NA	NA	NA	NA NA	NA	NA	NA	NA	NA	NA	NA NA	NA	NA	NA	NA	NA	NA	NA NA	NA	NA	NA	NA	NA	NA	NA

NA	NA	NA	-7.54 NA	-13.16 NA	-12.06

NA	NA	NA	-24.24 NA	-24.94	-17.69	-39.21

NA	NA	NA	NA	NA	-31.55 NA	-10.53

NA	NA	NA	NA	NA	-16.85 NA	NA

NA	NA	-39.99	-49.67 NA	-33.98	-43.47	-61.33

NA	NA	NA	-14.33 NA	-17.34	-8.91	-11.59

NA	NA	NA	NA	NA	NA	NA	NA

-1901.46	-1896.36	-6081.26	-5793.65	-2066.7	-2445.77	-6514.14	-5530.14

-54.62 NA	-145.61	-161.44 NA	-85.33	-191.92	-163.9

-54.62 NA	-145.61	-161.44 NA	-85.33	-191.92	-163.9

NA	NA	-5.09	-38.21 NA	-33.73	-20.81	-35.36

NA	NA	-5.09	-38.21 NA	-33.73	-20.81	-35.36

-26.1 NA	-30	-52.53 NA	-49.93	-46.36	-57.59

-118.99 NA	-165.44	-192.37 NA	-108.31	-209.48	-184.49

NA	NA	NA	NA	NA	-14.65 NA	-6.68

| NA |  | NA | NA | NA |  | NA |  | -12.26 NA | NA |
| --- | --- | --- | --- | --- | --- | --- | --- | --- | --- |
| NA |  | NA | NA | NA |  | NA |  | -12.26 NA | NA |
| NA |  | NA | NA | NA |  | NA |  | -12.26 NA | NA |
| NA |  | NA | NA | NA |  | NA | NA | NA | NA |
| NA |  | NA | NA | NA |  | NA | NA | NA | NA |
| NA |  | NA | NA | NA |  | NA | NA | NA | NA |
| NA |  | NA | NA | NA |  | NA | NA | NA | NA |
| -23.99 | | -27.86 | -42.08 |  | -9.78 | -71.22 NA | | -39.54 | -14.01 |
| -71.27 | | -117.58 | -51.36 |  | -40.4 | -327.28 NA | | -58 | -14.11 |
| -71.27 | | -117.58 | -51.36 |  | -40.4 | -327.28 NA | | -58 | -14.11 |

| -175.45 | | -231.97 | |  | -87.42 |  | -86.84 | -515.19 | |  | -40.99 | -100.09 | |  | -54.59 |
| --- | --- | --- | --- | --- | --- | --- | --- | --- | --- | --- | --- | --- | --- | --- | --- |
| NA |  |  | -8.31 | NA |  | NA |  |  | -15.34 | NA |  | NA |  | NA |  |
|  | -93.73 | -171.38 | |  | -202.2 | -135.26 | | -246.99 | |  | -27.18 | -209.76 | |  | -94.33 |
|  | -26.13 |  | -57.5 | NA |  | NA |  |  | -143.3 | NA |  | NA |  | NA |  |
| -113.36 | | -120.53 | | -239.49 | | -167.16 | | -340.83 | |  | -35.37 | -240.74 | | -162.76 | |
| NA |  | NA |  | NA |  | NA |  | NA |  | NA |  | NA |  | NA |  |
| NA |  | NA |  | NA |  | NA |  | NA |  | NA |  | NA |  | NA |  |
| NA |  | NA |  | NA |  | NA |  | NA |  | NA |  | NA |  | NA |  |
| NA |  | NA |  | NA |  | NA |  |  | -132.3 | NA |  | NA |  | NA |  |
| NA |  | NA |  | NA |  | NA |  |  | -95.44 | NA |  | NA |  | NA |  |
|  | -17.11 |  | -15.2 |  | -18.17 | NA |  | -299.43 | | NA |  |  | -17.39 |  | -11.2 |
| NA |  | NA |  | NA |  | NA |  | NA |  | NA |  | NA |  | NA |  |
| NA |  | NA |  | NA |  | NA |  | NA |  | NA |  | NA |  | NA |  |
| NA |  | NA |  | NA |  | NA |  | NA |  | NA |  | NA |  | NA |  |
| NA |  | NA |  | NA |  | NA |  | NA |  | NA |  | NA |  | NA |  |
| NA |  | NA |  | NA |  | NA |  | NA |  | NA |  | NA |  | NA |  |
| NA |  | NA |  | NA |  | NA |  | NA |  | NA |  | NA |  | NA |  |
| NA |  | NA |  | NA |  | NA |  | NA |  | NA |  | NA |  | NA |  |
| NA |  | NA |  | NA |  | NA |  | NA |  | NA |  | NA |  | NA |  |
| NA |  | NA |  | NA |  |  | -6.03 | NA |  |  | -63.74 | NA |  |  | -16.05 |
| NA |  | NA |  | NA |  | NA |  | NA |  | NA |  | NA |  | NA |  |
| NA |  | NA |  | NA |  | NA |  | NA |  | NA |  | NA |  | NA |  |
| NA |  | NA |  | NA |  | NA |  | NA |  | NA |  | NA |  | NA |  |
| NA |  | NA |  | NA |  | NA |  | NA |  | NA |  | NA |  | NA |  |
| NA |  | NA |  | NA |  | NA |  | NA |  |  | -9 | NA |  | NA |  |
| NA |  | NA |  |  | -5.81 |  | -14.64 | NA |  |  | -69.8 | NA |  |  | -29.03 |
| NA |  | NA |  | NA |  | NA |  | NA |  | NA |  | NA |  | NA |  |
| NA |  | NA |  | NA |  | NA |  | NA |  |  | -19.41 | NA |  | NA |  |
| NA |  | NA |  | NA |  | NA |  | NA |  | NA |  | NA |  | NA |  |
| NA |  | NA |  | NA |  | NA |  | NA |  | NA |  | NA |  | NA |  |
| NA |  | NA |  | NA |  | NA |  | NA |  |  | -9.29 | NA |  |  | -21.81 |
| NA |  | NA |  | NA |  | NA |  | NA |  | NA |  | NA |  | NA |  |
| NA |  | NA |  | NA |  | NA |  | NA |  |  | -5.79 | NA |  | NA |  |
| NA |  | NA |  | NA |  | NA |  | NA |  | NA |  | NA |  | NA |  |
| NA |  | NA |  | NA |  | NA |  | NA |  | NA |  | NA |  | NA |  |
| NA |  | NA |  | NA |  | NA |  | NA |  |  | -58.11 | NA |  |  | -11.96 |
| NA |  | NA |  | NA |  | NA |  | NA |  | NA |  | NA |  | NA |  |
| NA |  | NA |  | NA |  |  | -6.51 | NA |  |  | -49.5 | NA |  |  | -7.15 |
| NA |  | NA |  | NA |  | NA |  | NA |  |  | -32.08 | NA |  | NA |  |

| NA |  | NA |  | NA |  | NA |  | NA |  | NA |  | NA |  | NA |  |
| --- | --- | --- | --- | --- | --- | --- | --- | --- | --- | --- | --- | --- | --- | --- | --- |
| NA |  | NA |  |  | -12.9 |  | -11.28 | NA |  |  | -69.67 | NA |  |  | -24.76 |
| NA |  | NA |  | NA |  | NA |  | NA |  | NA |  | NA |  | NA |  |
| NA |  | NA |  | NA |  | NA |  | NA |  | NA |  | NA |  | NA |  |
| NA |  | NA |  | NA |  | NA |  | NA |  | NA |  | NA |  | NA |  |
| NA |  | NA |  | NA |  |  | -15.86 | NA |  |  | -68.07 | NA |  |  | -28.29 |
| NA |  | NA |  | NA |  | NA |  | NA |  | NA |  | NA |  | NA |  |
| NA |  | NA |  | NA |  | NA |  | NA |  |  | -26.29 | NA |  | NA |  |
| NA |  | NA |  | NA |  | NA |  | NA |  | NA |  | NA |  | NA |  |
| NA |  | NA |  | NA |  |  | -15.37 | NA |  |  | -64.12 | NA |  |  | -15.57 |
| -117.48 | | -127.43 | | -1011.7 | | -893.07 | | -205.37 | | -348.77 | | -1031.41 | | -964.44 | |
| NA |  | NA |  |  | -19.28 | -312.58 | | NA |  | -238.13 | |  | -19.32 | -309.48 | |
| NA |  | NA |  |  | -66.54 | -202.94 | | NA |  | -329.76 | |  | -88.57 | -373.24 | |
|  | -6.82 |  | -53.36 | -497.99 | | -646.77 | |  | -26.2 | -573.71 | | -565.92 | | -929.85 | |
|  | -28.95 |  | -92.1 | -314.13 | | -378.19 | | -177.82 | | -405.18 | | -310.39 | | -726.73 | |
|  | -19.16 |  | -56.05 | -234.58 | |  | -298 | -104.36 | | -343.26 | | -257.56 | | -505.08 | |
| NA |  |  | -12.51 | NA |  |  | -22.97 |  | -81.07 |  | -52.26 |  | -8.34 |  | -40.97 |
| NA |  | NA |  |  | -61.72 | -174.52 | | NA |  | -324.78 | |  | -72.82 | -370.68 | |
|  | -29.14 | -136.32 | | -537.81 | | -696.72 | | -140.43 | | -676.03 | |  | -592.5 | -1134.31 | |
| NA |  |  | -9.17 | -132.94 | |  | -202.9 |  | -14.73 | -317.37 | | -147.41 | | -471.36 | |
|  | -12.4 | NA |  | NA |  | NA |  | -161.09 | |  | -36.89 | NA |  | -202.29 | |
|  | -21.61 |  | -9.6 | NA |  |  | -7.04 | -138.71 | |  | -28.68 | NA |  | -179.81 | |
|  | -33.64 |  | -17.52 |  | -27.64 |  | -57.3 |  | -222.6 |  | -77.93 |  | -40.75 | -355.35 | |
| NA |  | NA |  | NA |  | NA |  | -143.81 | |  | -12.76 | NA |  | -121.04 | |
| NA |  | NA |  |  | -14.58 |  | -30.04 | -219.02 | | NA |  |  | -22.04 |  | -13.28 |
| NA |  |  | -12.27 |  | -49.58 |  | -60.43 | -158.15 | | NA |  |  | -43.29 |  | -77.1 |
|  | -31.95 |  | -24.55 |  | -6.41 |  | -10.2 | -397.84 | | NA |  | NA |  |  | -12.16 |
|  | -27.83 |  | -22.5 |  | -30.96 |  | -32.07 | -124.61 | | NA |  |  | -29.83 |  | -27.92 |
|  | -27.83 |  | -22.5 |  | -30.96 |  | -32.07 | -124.61 | | NA |  |  | -29.83 |  | -27.92 |
|  | -18.93 |  | -6.73 |  | -7.95 |  | -14.94 | -175.95 | | NA |  |  | -9.56 | NA |  |
|  | -35.49 |  | -23.72 | -137.44 | | -102.03 | | -332.01 | | NA |  | -145.12 | |  | -64.9 |
| NA |  | NA |  |  | -18.36 |  | -27.7 | -245.46 | | NA |  |  | -21.43 |  | -8.08 |
| NA |  | NA |  |  | -18.36 |  | -27.7 | -245.46 | | NA |  |  | -21.43 |  | -8.08 |
| NA |  | NA |  |  | -18.36 |  | -27.7 | -245.46 | | NA |  |  | -21.43 |  | -8.08 |
|  | -55.77 |  | -28.16 | -180.74 | | -118.74 | |  | -282.4 | NA |  | -165.23 | |  | -67.69 |
|  | -201.2 | -148.67 | | -277.24 | | -113.23 | | -428.23 | | NA |  | -242.69 | |  | -53.23 |
| NA |  | NA |  | NA |  | NA |  | -143.73 | | NA |  | NA |  | NA |  |
| NA |  | NA |  | NA |  | NA |  | NA |  | NA |  | NA |  | NA |  |
| NA |  | NA |  | -234.36 | | -396.49 | | NA |  | -182.51 | | -376.42 | | -372.87 | |

| NA |  | NA |  | NA |  | NA |  | NA |  | NA |  | NA |  | NA |  |
| --- | --- | --- | --- | --- | --- | --- | --- | --- | --- | --- | --- | --- | --- | --- | --- |
| NA |  | NA |  | NA |  | NA |  | NA |  | NA |  | NA |  | NA |  |
| NA |  | NA |  | NA |  | NA |  | NA |  | NA |  | NA |  | NA |  |
| NA |  | NA |  | NA |  | NA |  | NA |  | NA |  | NA |  | NA |  |
| -211.03 | | -438.41 | | -584.02 | | -204.57 | | -273.95 | |  | -31.68 | -492.04 | | -148.47 | |
| -154.23 | | -116.17 | | -248.87 | | -183.82 | | -200.72 | |  | -64.14 | -258.98 | | -218.98 | |
|  | -64.08 |  | -74.34 |  | -82.28 |  | -32.22 | -222.07 | | NA |  |  | -70.43 |  | -22.46 |
| -206.02 | | -261.67 | | -544.62 | | -369.43 | | -229.32 | | -143.03 | | -591.82 | | -385.12 | |
|  | -50.69 |  | -61.94 |  | -21.55 | NA |  |  | -36.32 | NA |  |  | -17.05 |  | -6.43 |
|  | -90.03 |  | -84.07 |  | -85.64 |  | -50.65 |  | -68.47 |  | -10.94 |  | -83.42 |  | -56.07 |
|  | -53.01 | -143.51 | | -233.35 | | -206.49 | | -467.38 | |  | -68.84 | -273.93 | | -233.48 | |
| -345.01 | | -242.11 | | -161.14 | |  | -98.65 | -369.38 | |  | -34.37 |  | -128.2 |  | -30.99 |
| -386.66 | | -338.71 | | -239.01 | | -229.17 | | -178.21 | |  | -110.1 | -240.07 | | -137.33 | |
|  | -10.89 |  | -37.71 | -115.56 | |  | -61.77 |  | -72.99 |  | -14.06 | -107.99 | |  | -81.96 |
| NA |  | NA |  | NA |  | NA |  | NA |  | NA |  | NA |  | NA |  |
| NA |  | NA |  | NA |  | NA |  | NA |  | NA |  | NA |  | NA |  |
|  | -12.11 |  | -77.93 | -154.68 | | -140.38 | | -394.59 | |  | -52.69 | -172.33 | | -161.04 | |
| NA |  | NA |  | NA |  | NA |  | NA |  | NA |  | NA |  | NA |  |
| -123.26 | | -244.61 | | -704.03 | | -294.66 | | -234.57 | |  | -81.03 | -630.29 | | -279.12 | |
| NA |  | NA |  | NA |  | NA |  | NA |  | NA |  | NA |  | NA |  |
| NA |  | NA |  | NA |  | NA |  | NA |  | NA |  | NA |  | NA |  |
|  | -81.75 | -105.55 | | -195.78 | | -182.62 | | -153.79 | |  | -74.32 | -214.51 | | -141.62 | |
|  | -61.95 | -130.41 | | -174.54 | | -175.52 | | -409.49 | |  | -57.9 |  | -200.6 | -205.22 | |
|  | -61.95 | -130.41 | | -174.54 | | -175.52 | | -409.49 | |  | -57.9 |  | -200.6 | -205.22 | |
|  | -61.95 | -130.41 | | -174.54 | | -175.52 | | -409.49 | |  | -57.9 |  | -200.6 | -205.22 | |
|  | -61.95 | -130.41 | | -174.54 | | -175.52 | | -409.49 | |  | -57.9 |  | -200.6 | -205.22 | |
| -147.95 | | -134.92 | | -515.85 | | -331.26 | |  | -247.9 | -158.82 | | -471.11 | |  | -310.2 |
| -147.95 | | -134.92 | | -515.85 | | -331.26 | |  | -247.9 | -158.82 | | -471.11 | |  | -310.2 |
| -147.95 | | -134.92 | | -515.85 | | -331.26 | |  | -247.9 | -158.82 | | -471.11 | |  | -310.2 |
| -147.95 | | -134.92 | | -515.85 | | -331.26 | |  | -247.9 | -158.82 | | -471.11 | |  | -310.2 |
|  | -50.41 |  | -35.94 | -304.74 | |  | -279.2 |  | -57.61 | -244.08 | | -305.48 | | -326.34 | |
|  | -50.41 |  | -35.94 | -304.74 | |  | -279.2 |  | -57.61 | -244.08 | | -305.48 | | -326.34 | |
|  | -29.27 |  | -25.81 | -254.02 | | -214.17 | |  | -49.96 | -136.04 | | -247.45 | | -219.37 | |
|  | -29.27 |  | -25.81 | -254.02 | | -214.17 | |  | -49.96 | -136.04 | | -247.45 | | -219.37 | |
| -149.13 | |  | -238.1 | -373.12 | | -358.57 | | -884.89 | | -193.96 | | -406.85 | | -437.03 | |
| -174.98 | | -421.93 | | -909.36 | | -288.29 | | -317.66 | |  | -71.98 | -709.59 | | -206.93 | |
| -174.98 | | -421.93 | | -909.36 | | -288.29 | | -317.66 | |  | -71.98 | -709.59 | | -206.93 | |
| -118.26 | | -242.79 | | -785.79 | | -357.09 | |  | -189.2 |  | -96.53 | -695.84 | | -322.91 | |
| -118.26 | | -242.79 | | -785.79 | | -357.09 | |  | -189.2 |  | -96.53 | -695.84 | | -322.91 | |

| -112.51 | | -372.05 | | -839.42 | | -280.75 | | -328.47 | |  | -44.9 | -684.88 | | -197.96 | |
| --- | --- | --- | --- | --- | --- | --- | --- | --- | --- | --- | --- | --- | --- | --- | --- |
| -251.62 | | -533.19 | | -1095.66 | | -377.33 | | -444.88 | |  | -67.57 | -893.39 | | -302.45 | |
| -104.27 | | -267.04 | | -522.84 | | -129.95 | | -428.69 | |  | -24.41 | -414.41 | | -128.17 | |
|  | -8.65 |  | -66.09 | -164.12 | |  | -31.65 |  | -73.86 |  | -8.85 | -128.16 | |  | -48.63 |
|  | -69.54 |  | -54.76 | -149.29 | | -116.79 | | -115.24 | |  | -98.75 | -150.74 | |  | -306.1 |
|  | -8.76 | NA |  | NA |  | NA |  |  | -138.2 |  | -18.7 | NA |  |  | -151 |
| -117.22 | | -102.62 | |  | -151.6 |  | -91.99 | -160.22 | |  | -48.37 | -143.99 | | -204.43 | |
|  | -95.43 |  | -85.85 |  | -25.12 |  | -11.15 | -250.36 | | NA |  |  | -8.97 |  | -32.16 |
| -128.92 | | -106.78 | |  | -34.61 |  | -15.91 |  | -314 |  | -6.96 |  | -15.88 |  | -40.35 |
| NA |  | NA |  | NA |  | NA |  | -143.76 | | NA |  | NA |  | NA |  |
| -108.67 | |  | -99.87 |  | -30.56 |  | -16.03 | -314.75 | |  | -12.19 |  | -14.39 |  | -40.68 |
|  | -34.04 |  | -42.98 |  | -68.04 |  | -35.87 |  | -74.95 |  | -15.25 |  | -56.99 |  | -68.05 |
| NA |  | NA |  | NA |  | NA |  | -139.55 | | NA |  | NA |  | NA |  |
|  | -39.89 |  | -78.5 |  | -83.04 |  | -41.69 | -291.87 | | NA |  |  | -67.39 |  | -87.15 |
| NA |  | NA |  | NA |  | NA |  |  | -65.07 | NA |  | NA |  | NA |  |
| NA |  | NA |  | NA |  | NA |  | NA |  | NA |  | NA |  | NA |  |
| NA |  | NA |  | NA |  | NA |  | NA |  | NA |  | NA |  | NA |  |
| NA |  | NA |  |  | -7.53 |  | -12.69 |  | -9.84 | NA |  |  | -11.24 |  | -27.19 |
| NA |  | NA |  | NA |  | NA |  | NA |  | NA |  | NA |  | NA |  |
| NA |  | NA |  | NA |  | NA |  | NA |  | NA |  | NA |  | NA |  |
| NA |  | NA |  | NA |  | NA |  | NA |  | NA |  | NA |  | NA |  |
| NA |  | NA |  | NA |  | NA |  | NA |  | NA |  | NA |  | NA |  |
| NA |  | NA |  | NA |  | NA |  | NA |  | NA |  | NA |  | NA |  |
| NA |  | NA |  |  | -28.41 |  | -54.39 |  | -7.11 |  | -13.18 |  | -40.85 |  | -79.15 |
|  | -8.6 | NA |  |  | -139.9 | -135.81 | |  | -69.32 |  | -30.37 | -153.47 | | -163.03 | |
| NA |  | NA |  | NA |  | NA |  | NA |  | NA |  | NA |  | NA |  |
| NA |  | NA |  | NA |  | NA |  | NA |  | NA |  | NA |  | NA |  |
| NA |  | NA |  | NA |  | NA |  | NA |  | NA |  | NA |  | NA |  |
|  | -99.11 |  | -190.8 | -386.14 | | -353.51 | | -753.05 | | -187.72 | | -426.53 | | -429.62 | |
|  | -90.91 |  | -71.13 | -276.11 | | -195.08 | | -255.65 | |  | -13.1 |  | -281 | -153.26 | |
|  | -90.91 |  | -71.13 | -276.11 | | -195.08 | | -255.65 | |  | -13.1 |  | -281 | -153.26 | |
| NA |  | NA |  | NA |  | NA |  | NA |  | NA |  | NA |  | NA |  |
| NA |  | NA |  | NA |  | NA |  | -176.07 | | NA |  | NA |  | NA |  |
| NA |  | NA |  | NA |  | NA |  |  | -21.9 | NA |  | NA |  | NA |  |
| NA |  | NA |  | NA |  | NA |  |  | -94.4 | NA |  | NA |  | NA |  |
| -423.91 | | -366.37 | | -616.81 | | -607.43 | | -380.59 | | -267.64 | | -645.62 | | -500.11 | |
| NA |  | NA |  | NA |  | NA |  | -115.56 | | NA |  | NA |  | NA |  |
| NA |  | NA |  | NA |  | NA |  |  | -7.05 | NA |  | NA |  | NA |  |
| NA |  | NA |  | NA |  | NA |  | -123.27 | | NA |  | NA |  | NA |  |

| NA |  | NA |  |  | -9.43 | NA |  |  | -75.48 | NA |  |  | -11.89 |  | -13.4 |
| --- | --- | --- | --- | --- | --- | --- | --- | --- | --- | --- | --- | --- | --- | --- | --- |
| NA |  | NA |  | NA |  | NA |  |  | -11.86 | NA |  | NA |  | NA |  |
| NA |  | NA |  | NA |  | NA |  | NA |  | NA |  | NA |  | NA |  |
| NA |  | NA |  | NA |  | NA |  |  | -16.4 | NA |  | NA |  | NA |  |
| NA |  | NA |  | NA |  | NA |  | -163.04 | | NA |  | NA |  | NA |  |
| -157.95 | | -109.95 | | -260.47 | | -209.92 | | -169.16 | |  | -85.48 | -301.49 | |  | -276 |
| NA |  | NA |  | NA |  | NA |  | NA |  | NA |  | NA |  | NA |  |
| NA |  | NA |  | NA |  | NA |  | NA |  | NA |  | NA |  | NA |  |
| NA |  | NA |  | NA |  | NA |  | NA |  | NA |  | NA |  | NA |  |
| NA |  | NA |  | NA |  | NA |  | NA |  | NA |  | NA |  | NA |  |
|  | -28.33 | -187.58 | |  | -11.8 |  | -18.24 |  | -16.25 |  | -11.98 |  | -16.97 |  | -16.82 |
| NA |  |  | -90.4 | NA |  | NA |  |  | -13.39 | NA |  | NA |  |  | -5.28 |
|  | -81.32 |  | -98.14 |  | -71.35 |  | -82.69 |  | -79.61 |  | -48.4 |  | -99.21 | -117.84 | |
| NA |  | NA |  | NA |  | NA |  | NA |  | NA |  | NA |  | NA |  |
|  | -20.85 |  | -209.8 | NA |  | NA |  |  | -20.45 | NA |  | NA |  | NA |  |
| NA |  |  | -56.88 |  | -7.82 |  | -25.37 | NA |  |  | -18.03 |  | -18.21 |  | -35.41 |
| NA |  |  | -20.59 |  | -6.03 |  | -12.3 |  | -63.3 | NA |  |  | -9.14 |  | -23.48 |
|  | -13.41 | -112.43 | | NA |  | NA |  |  | -70.47 | NA |  | NA |  | NA |  |
| NA |  | NA |  | NA |  | NA |  | NA |  | NA |  | NA |  | NA |  |
| -138.51 | | -161.86 | |  | -83.92 | NA |  | -1276.54 | | NA |  |  | -37.06 | NA |  |
| -192.94 | | -183.43 | | -257.75 | |  | -77.43 | -664.82 | | NA |  | -208.39 | |  | -63.48 |
| NA |  | NA |  |  | -23.85 |  | -6.63 | -120.44 | | NA |  |  | -22.45 |  | -21.53 |
|  | -8.07 | NA |  |  | -13.36 | NA |  |  | -145.2 | NA |  |  | -14.16 |  | -10.09 |
| -331.56 | | -370.36 | | -419.96 | | -540.88 | |  | -68.14 | -274.65 | | -512.03 | | -221.53 | |
|  | -46.53 |  | -97.94 | -247.86 | | -143.57 | | -140.89 | |  | -26.21 | -252.15 | |  | -141.6 |
| NA |  | NA |  |  | -5.33 | NA |  | -109.95 | | NA |  | NA |  | NA |  |
|  | -47.54 |  | -79.46 | -112.14 | |  | -75.83 | -294.78 | |  | -15.01 | -101.75 | | -155.43 | |
|  | -7.83 |  | -13.38 |  | -19.68 | NA |  | -122.72 | | NA |  |  | -10.33 |  | -23.4 |
|  | -96.5 | -112.98 | |  | -191.2 | -125.46 | |  | -203.5 |  | -51.99 | -181.38 | | -146.57 | |
|  | -31.18 |  | -49.69 |  | -25.82 |  | -21.01 |  | -60.2 |  | -11.14 |  | -26.3 |  | -28.56 |
|  | -27.24 |  | -20.5 |  | -66.96 |  | -43.54 |  | -25.47 |  | -24.68 |  | -69.15 |  | -48.47 |
|  | -26.94 |  | -38.68 |  | -57.75 |  | -49.48 |  | -56.05 |  | -35.41 |  | -57.86 |  | -72.91 |
|  | -58.11 |  | -46.62 | -123.12 | |  | -91.66 |  | -66.94 |  | -60.04 | -115.97 | |  | -108.6 |
|  | -57.77 |  | -60.24 | -116.59 | |  | -70.85 | -130.32 | |  | -10.84 | -105.94 | |  | -63.53 |
|  | -8.91 |  | -15.64 |  | -61.81 |  | -58.64 |  | -31.29 |  | -35.76 |  | -62.35 |  | -86.51 |
| NA |  |  | -7.29 |  | -7.62 |  | -12.02 | NA |  |  | -16.2 |  | -9.11 |  | -22.42 |
| NA |  | NA |  | NA |  | NA |  | NA |  | NA |  | NA |  | NA |  |
| NA |  | NA |  | NA |  | NA |  | NA |  | NA |  | NA |  | NA |  |
| NA |  | NA |  | NA |  | NA |  | NA |  | NA |  | NA |  | NA |  |

| -250.38 | | -359.46 | | -2804.91 | | -2233.39 | | -759.42 | | -876.75 | | -2692.16 | | -2320.17 | |
| --- | --- | --- | --- | --- | --- | --- | --- | --- | --- | --- | --- | --- | --- | --- | --- |
| -322.85 | | -433.35 | | -3518.71 | | -2857.89 | |  | -882.7 | -1025.86 | | -3417.28 | | -2824.61 | |
| -209.94 | | -254.51 | | -1883.45 | | -1473.89 | | -522.13 | | -452.01 | | -1805.56 | | -1311.64 | |
| NA |  | NA |  | NA |  | NA |  |  | -108.1 | NA |  |  | -5.79 | NA |  |
| NA |  | NA |  | NA |  | NA |  |  | -22.37 | NA |  | NA |  | NA |  |
| NA |  | NA |  | NA |  | NA |  | NA |  | NA |  | NA |  | NA |  |
| NA |  | NA |  | NA |  | NA |  |  | -14.85 | NA |  | NA |  | NA |  |
| NA |  | NA |  |  | -17.28 |  | -29.39 | NA |  |  | -18.42 |  | -27.94 |  | -52.13 |
|  | -60.84 |  | -60.86 | -121.94 | |  | -66.83 | -271.32 | | NA |  | -138.43 | |  | -43.8 |
|  | -59.63 |  | -65.03 | -115.49 | |  | -72.2 |  | -149.3 |  | -5.33 | -131.57 | |  | -85.47 |
|  | -79.49 |  | -69.54 | -107.01 | |  | -65.84 | -160.55 | |  | -5.55 |  | -124.2 |  | -78.17 |
|  | -79.49 |  | -69.54 | -107.01 | |  | -65.84 | -160.55 | |  | -5.55 |  | -124.2 |  | -78.17 |
|  | -95.37 |  | -75.44 | -151.35 | | -108.39 | | -155.49 | |  | -30.2 | -171.31 | | -104.09 | |
|  | -10.75 |  | -9.29 | NA |  | NA |  | -140.45 | |  | -22.8 | NA |  | -152.49 | |
|  | -10.75 |  | -9.29 | NA |  | NA |  | -140.45 | |  | -22.8 | NA |  | -152.49 | |
| NA |  | NA |  |  | -40.67 |  | -62.74 | NA |  |  | -26.05 |  | -67.15 |  | -59.82 |
| NA |  | NA |  |  | -16.37 |  | -51.12 | NA |  |  | -45.01 |  | -32.41 |  | -34.07 |
| NA |  | NA |  |  | -9.12 |  | -14.37 | NA |  |  | -19.61 |  | -15.09 |  | -26.21 |
|  | -24.36 |  | -6.45 |  | -74.28 |  | -56.32 | -227.22 | | NA |  |  | -77.69 |  | -17.63 |
| -734.79 | | -665.46 | | -2340.21 | | -2456.93 | | -838.69 | | -1122.98 | | -2519.75 | | -2501.83 | |
| -644.73 | | -717.47 | | -3597.34 | | -2765.48 | | -376.75 | | -1455.6 | | -3371.57 | | -2574.38 | |
| -131.19 | | -112.51 | | -1483.26 | | -1405.59 | | -151.38 | | -418.42 | | -1532.61 | | -1247.32 | |
| -574.02 | | -532.76 | | -2647.12 | | -2206.99 | | -557.15 | | -875.95 | | -2615.01 | |  | -2062 |
| -928.34 | | -1027.29 | | -4776.54 | | -3764.77 | | -583.64 | | -1902.99 | | -4544.14 | | -3540.69 | |
| -544.09 | | -533.29 | | -3378.54 | | -2859.78 | | -389.13 | | -1349.89 | | -3285.86 | | -2707.08 | |
|  | -31.13 |  | -14.9 | -154.66 | | -104.73 | |  | -13.02 | -102.26 | | -132.18 | | -170.49 | |
| NA |  |  | -62.73 | -490.62 | | -720.93 | |  | -26.92 | -723.15 | | -596.82 | | -1100.93 | |
|  | -53.59 |  | -27.28 | -176.72 | | -140.71 | |  | -59.54 | -100.81 | | -167.93 | | -237.18 | |
| NA |  |  | -21.55 |  | -82.52 |  | -26.31 |  | -64.04 | NA |  |  | -74.69 |  | -26.97 |
| -184.67 | | -405.34 | | -853.54 | | -318.19 | |  | -433.4 |  | -51.11 | -706.35 | | -254.67 | |
|  | -144.1 | -241.91 | | -695.61 | | -346.85 | | -138.62 | | -122.32 | | -649.01 | | -297.93 | |
| NA |  | -141.84 | | -322.21 | |  | -46.34 |  | -163.9 | NA |  | -256.44 | |  | -18.62 |
| NA |  | NA |  | -124.08 | | -285.59 | | NA |  | -152.41 | | -239.91 | | -280.86 | |
| NA |  | NA |  | NA |  | NA |  | NA |  | NA |  | NA |  | NA |  |
| NA |  | NA |  | NA |  | NA |  | NA |  | NA |  | NA |  | NA |  |
| -131.07 | | -131.64 | |  | -8.29 | NA |  | -460.11 | | NA |  | NA |  | NA |  |
| -183.33 | | -195.26 | | -116.16 | |  | -15.91 | -869.53 | | NA |  |  | -83.22 |  | -12.73 |
| -223.52 | | -268.59 | |  | -96.69 | NA |  | -652.31 | | NA |  |  | -58.71 | NA |  |
| NA |  | NA |  | NA |  | NA |  |  | -36.28 | NA |  | NA |  | NA |  |

| -193.21 | | -200.47 | |  | -88 |  | -10.13 | -720.37 | | NA |  |  | -53.93 | NA |  |
| --- | --- | --- | --- | --- | --- | --- | --- | --- | --- | --- | --- | --- | --- | --- | --- |
| -270.14 | |  | -415.7 | -738.89 | | -296.91 | |  | -402.5 |  | -60.01 | -628.32 | | -246.65 | |
|  | -27.05 |  | -91.82 | -136.33 | | -132.97 | | -416.05 | |  | -60.61 | -163.21 | | -172.09 | |
|  | -26.69 |  | -69.76 | -105.99 | |  | -93.27 | -396.38 | |  | -46.59 | -120.21 | | -141.73 | |
|  | -13.58 |  | -49.38 |  | -58.11 |  | -40.83 | -227.25 | |  | -14.92 |  | -72.19 |  | -70.74 |
|  | -20.25 |  | -55.43 | -111.34 | |  | -49.38 |  | -37.93 |  | -15.59 | -107.09 | |  | -58.32 |
|  | -8.85 |  | -17.57 | NA |  | NA |  |  | -45.04 | NA |  | NA |  | NA |  |
|  | -29.81 |  | -97.87 | -132.68 | |  | -84.15 |  | -430.6 |  | -38.39 | -125.73 | | -125.92 | |
|  | -22.42 |  | -72.26 |  | -42.69 |  | -18.72 |  | -405.2 | NA |  |  | -45.44 |  | -44.12 |
| NA |  | NA |  | NA |  | NA |  | NA |  | NA |  | NA |  | NA |  |
| NA |  | NA |  | NA |  | NA |  | NA |  | NA |  | NA |  | NA |  |
| NA |  | NA |  | NA |  | NA |  | NA |  | NA |  | NA |  | NA |  |
| -433.94 | | -440.47 | | -581.19 | | -627.44 | | -212.21 | | -290.97 | | -652.41 | | -301.44 | |
| -175.67 | | -194.43 | | -198.35 | | -161.47 | | -387.39 | |  | -56.5 | -214.07 | | -136.82 | |
| NA |  |  | -36.82 |  | -147.2 |  | -22.33 |  | -42.21 | NA |  | -100.41 | |  | -16.74 |
| -104.61 | | -138.21 | | -102.88 | |  | -65.55 | -243.14 | |  | -21.74 |  | -90.46 |  | -56.73 |
| NA |  |  | -31.69 |  | -133.6 |  | -18.99 |  | -30.91 | NA |  |  | -92.03 |  | -11.83 |
|  | -16.78 |  | -21.23 |  | -54.75 |  | -29.45 | -208.65 | | NA |  |  | -53.48 |  | -27.94 |
|  | -98.73 | -103.57 | |  | -42.24 | NA |  |  | -438 | NA |  |  | -25.49 | NA |  |
| NA |  | NA |  | NA |  | NA |  | NA |  | NA |  | NA |  | NA |  |
| -386.93 | | -325.01 | | -383.68 | | -305.22 | | -413.38 | | -140.57 | | -376.36 | |  | -225.5 |
| NA |  | NA |  |  | -9.23 |  | -5.23 |  | -67.58 | NA |  |  | -11.7 |  | -15.89 |
|  | -34.04 |  | -39.21 | NA |  | NA |  |  | -510.2 | NA |  | NA |  | NA |  |
|  | -39.01 |  | -27.24 | NA |  | NA |  | -1446.1 | | NA |  | NA |  | NA |  |
| -125.05 | | -170.49 | | -167.05 | |  | -54.98 |  | -337.5 | NA |  | -137.08 | |  | -28.99 |
|  | -88.89 |  | -57.89 | -248.58 | | -115.14 | | -272.11 | | NA |  | -230.31 | |  | -59 |
| -142.11 | | -164.27 | | -218.92 | |  | -75.38 | -242.07 | |  | -6.43 | -190.76 | |  | -60.74 |
| -155.72 | | -199.71 | | -118.35 | |  | -55.05 | -209.65 | |  | -7.63 | -105.84 | |  | -22.89 |
|  | -13.94 |  | -7.62 |  | -57.1 |  | -18.33 | -184.01 | | NA |  |  | -47.69 |  | -9.04 |
| -134.56 | | -145.78 | | -227.16 | | -219.64 | | -134.61 | |  | -94.31 | -242.63 | | -127.53 | |
|  | -76.72 |  | -51.75 | -253.22 | | -206.79 | | -352.08 | |  | -14.43 | -273.78 | | -144.39 | |
|  | -238.9 | -275.54 | | -332.52 | | -248.24 | | -259.65 | | -124.54 | | -333.16 | | -200.93 | |
|  | -38.21 |  | -31.74 | -161.63 | | -117.03 | | -140.66 | |  | -36.29 |  | -170.4 |  | -98.6 |
|  | -12.22 |  | -24.92 | -212.44 | | -127.19 | | -132.43 | |  | -29.57 | -207.54 | | -141.97 | |
|  | -75.92 |  | -80.35 |  | -23.17 |  | -55.42 |  | -49.1 |  | -16.5 |  | -39.23 | NA |  |
|  | -64.35 |  | -45.57 | -282.15 | | -286.26 | |  | -61.84 | -166.66 | | -288.77 | | -344.77 | |
| -143.82 | | -118.02 | | -574.09 | | -598.08 | | -132.96 | | -346.83 | | -597.24 | | -724.94 | |
|  | -65.2 |  | -54.29 |  | -93.85 |  | -31.91 | -198.56 | |  | -7.42 |  | -85.56 |  | -35.28 |
|  | -289.1 | -280.56 | | -190.56 | | -299.39 | |  | -87.07 | -156.55 | | -231.84 | | -142.94 | |

| -54.81 | -70.7 | -167.29 | -104.42 | -149.05 | -27.88 | -161.74 | -82.21 |
| --- | --- | --- | --- | --- | --- | --- | --- |
| -133.33 | -152.36 | -378.13 | -204.73 | -332.84 | -44.93 | -325.8 | -161.11 |
| -84.68 | -116.81 | -300.73 | -240.8 | -205.7 | -130.97 | -327.31 | -226.52 |
| -13.15 | -30.45 | -95.95 | -70.81 | -78.51 | -13.87 | -100.51 | -33.96 |
| -220.62 | -202.28 | -415.95 | -563.33 | -80.59 | -598.37 | -482.85 | -536.73 |

NA	NA	NA	-44.51 NA	-6.6 NA	-54.72

-212.69	-234.52	-454.98	-307.35	-264.07	-98.67	-461.06	-308.92

NA	NA	-138.59	-48.76	-108.99 NA	-120.14	-35.56

-151.06	-136.51	-117.17	-65.8	-276.82	-9.09	-110.53	-40.38

-34.04	-47.51	-166.74	-115.81	-56.74	-34.45	-171.93	-107.25

-79.66	-56.29	-136.87	-76.19	-276.67 NA	-137.47	-53.51

NA	NA	NA	NA	NA	NA	NA	NA

-42.25	-53.15	-145.93	-145.15	-174.3	-48.46	-179.22	-122.8

-62.74	-35.39	-93.91	-43.23	-174.75 NA	-75.13	-20.04

-106.39	-91.42	-181.8	-103.58	-382.76 NA	-157.89	-49.17

-422.89	-516.99	-662.63	-400.72	-382.74	-124.86	-650.3	-343.84

-40.06	-51.38	-27.03	-11.07	-100.93 NA	-32.92	-6.62

-200.96	-279.61	-270.02	-132.33	-279.26	-40.02	-251.32	-98.03

NA	NA	NA	-16.55	-5.38 NA	NA	-14.05

-12.45	-17.67	-117.13	-82.07	-97.17 NA	-130.96	-64.29

-35.49	-35.45	-310.95	-244.7	-323.07	-26.5	-324.19	-182.49

NA	NA	-118.45	-176.83 NA	-41.42	-136.39	-151.29

-86.55	-95.95	-90.67	-21.04	-312.47 NA	-65	-17.25

-69.49	-36.56	-350.83	-239.62	-118.12 NA	-290.73	-88.04

NA	NA	-93.36	-37.6	-190.9 NA	-80.69	-29.63

-47.46	-37.47	-292.39	-302.25	-32.35	-176.67	-321.3	-295.11

-48.1	-34.55	-119.46	-89.21	-75.21	-31.87	-129.56	-79.32

-90.21	-49.22	-197.06	-84.07	-304.83 NA	-166.31	-42.71

-45.64	-54.18	-110.93	-83.4	-70.5	-20.08	-117.32	-87.45

NA	-5.1	-41.02	-57.86	-25.99	-19.34	-57.76	-80.81

-53.89	-42.49	-326.62	-256.89	-140.83	-43.23	-325.74	-216.55

-266.89	-239.61	-219.55	-167.93	-241.09	-82.26	-187.51	-77.42

-103.26	-77.68	-100.04	-11.97	-542.31 NA	-62.53	-9.43

-29.95	-37.46	-419.01	-396.21	-74.09	-634.28	-422.69	-402.34

NA	NA	NA	NA	-37.19 NA	NA	NA

-45.06	-50.69	-76.66	-51.29	-83.06	-13.81	-81.86	-47.75

-63.54	-67.33	-196.69	-150.92	-78.39	-54.51	-210.98	-134.14

-54.31	-40.36	-248.7	-178.06	-233.39 NA	-240.15	-92.46

cd4positiv cd8_positi cd8_positi cd8positiv central_m central_m central_m class_swit

-218.37	-86.13	-228.07	-262.94	-150.24	-174.62	-70.82	-164.52

NA	NA	NA	NA	NA	NA	NA	NA

-254.4	-57.3	-252.96	-226.23	-253.12	-377.89	-114.73	-638.52

-180.14	-198.3	-124.78	-216.95	-234.32	-218.07	-119.97	-868.82

-48.58	-30.63	-15.24	-54.29	-98.05	-99.38	-76.82	-806.24

-614.63	-213.8	-1106.58	-669.72	-543.75	-404.01	-322.42	-1217.92

-198.86	-88.23	-150.7	-232.22	-287.92	-376.79	-172.87	-1292.71

-74.4	-27.08	-49.29	-81.84	-121.55	-157.49	-51.01	-420.27

-74.4	-27.08	-49.29	-81.84	-121.55	-157.49	-51.01	-420.27

NA	NA	NA	NA	-6.51	-28.73 NA	-35.24

-287.92	-81.94	-498.14	-290.48	-250.37	-194.16	-130.42	-577.66

-205.52	-80.58	-328.89	-182.66	-164.51	-129.19	-110.44	-616.15

-45.77	-14.9	-22.88	-36.85	-339.97	-322.94	-123.04	-638.21

-89.01	-47.83	-63.18	-92.95	-446.66	-467.64	-185.83	-784.97

NA	NA	NA	NA	NA	NA	NA	NA NA	NA	NA	NA	NA	NA	NA	NA

NA	NA	NA	NA	-148.86	-332.17	-26.54	-79.03

NA	NA	NA	NA	-148.86	-332.17	-26.54	-79.03

-1241.56	-76.98	-1773.36	-1092.57	-813.1	-1367.49	-110.12	-500.65

-263.48	-75.63	-258.95	-257.36	-320.08	-409.2	-191.17	-1222.91

NA	NA	NA	NA	NA	NA	NA	NA

-1472.69	-384.65	-2673.01	-1724.81	-1282.69	-1623.82	-460.59	-767.48

NA	NA	NA	NA	NA	NA	NA	NA NA	NA	NA	NA	NA	NA	NA	NA NA	NA	NA	NA	NA	-6.7 NA	NA NA	NA	NA	NA	NA	NA	NA	NA NA	NA	NA	NA	NA	NA	NA	NA NA	NA	NA	NA	NA	NA	NA	NA

-243.64	-50.52	-233.93	-227.59	-289.62	-515.39	-131.64	-721.89

-243.64	-50.52	-233.93	-227.59	-289.62	-515.39	-131.64	-721.89

-343.54	-111.53	-311.08	-323.95	-436.6	-594.41	-199.01	-1251.84

-356.3	-97.92	-349.51	-331.19	-438.84	-645.46	-205.62	-1231.84

NA	NA	NA	NA	NA	-33.05 NA	-12.76

NA	NA	NA	NA	NA	NA	NA	NA NA	NA	NA	NA	NA	NA	NA	NA NA	NA	NA	NA	NA	NA	NA	NA NA	NA	NA	NA	NA	NA	NA	NA

-200.99	-125.16	-137.71	-213.2	-121.32	-40.49	-33.87	-71.07

-24.86	-6.27	-23.83	-35.36	-29.58	-7.46	-25.53	-1034.69

-24.86	-6.27	-23.83	-35.36	-29.58	-7.46	-25.53	-1034.69

-24.86	-6.27	-23.83	-35.36	-29.58	-7.46	-25.53	-1034.69

-369.16	-31.94	-259.76	-403.23	-357.3	-469.77	-45.52	-69.74

-235.43	-43.87	-157.6	-302.06	-251.21	-218.43	-36.09	-98.96

-2824.76	-374.53	-3502.06	-2769.73	-1567.68	-2017.32	-320.25	-719.09

-3333.18	-695.68	-4391.17	-3209.99	-2178.07	-2741.23	-544.69	-1024.75

-5035.73	-1025.16	-6363.45	-4741.31	-3307.66	-3937	-919.39	-1772.39

-3698.16	-550.21	-4697.37	-3586.25	-2214.84	-2887.21	-500.79	-923.82

-2170.92	-472.54	-2791.18	-2096.84	-1481.06	-1883.58	-428.29	-797.17

-2731.56	-663.76	-3532.68	-2771.58	-1909.41	-2272.18	-564.04	-1203.46

-3876.97	-717.5	-5421.78	-3674.08	-2430.79	-3317.21	-626.35	-1239.37

-3257.86	-963.31	-3894.14	-3250.34	-2349.82	-2612.21	-827.42	-1269.62

-4149.17	-1028.81	-5087.61	-4048.25	-3028.41	-3507.18	-889.42	-1501.88

-4741.66	-1246.75	-5907.17	-4695.69	-3377.91	-3861.8	-1112.92	-1730.57

NA	NA	NA	NA	NA	NA	NA	NA

-117.06	-24.91	-61.97	-111.26	-205.48	-315.08	-98.85	-1142.51

-4889.13	-1161.61	-6177.18	-4784.39	-3497.85	-4146.2	-1027.03	-1508.88

-5638.09	-1399.88	-7245.26	-5545.05	-4058.97	-4852.09	-1289.72	-2024.58

-300.15	-115.16	-399.62	-316.19	-267.87	-305.59	-103.78	-175.96

-6779.61	-1291.24	-8799.06	-6631.83	-4472.78	-5314.32	-1225.28	-2094.09

NA	NA	NA	NA	NA	NA	NA	-91.87

NA	NA	NA	NA	NA	NA	NA	NA

-6235.86	-1337.23	-8043.63	-6081.98	-4436	-5167.3	-1296.3	-2115.73

-241.72	-51.93	-235.6	-220.97	-144.48	-186.94	-43.5	-78.1

-2684.04	-444.72	-3453.32	-2714.43	-1691.63	-2145	-399.81	-725.5

-5829.81	-1437.88	-7372.57	-5688.24	-4126.74	-4761.08	-1201.54	-1984.93

-4277.14	-940.7	-5669.41	-4171.31	-3130.62	-4010.59	-818.55	-1370.31

-1811.45	-504.72	-2248.87	-1822.67	-1441.69	-1679.54	-443.71	-671.61

-1811.45	-504.72	-2248.87	-1822.67	-1441.69	-1679.54	-443.71	-671.61

-6300.49	-1373.41	-8020.56	-6229.67	-4386.45	-5078.15	-1205.41	-1978.39

-2788.96	-638.7	-3288.67	-2716.65	-1815.19	-2075.85	-616.9	-1023.31

-1420.85	-277.22	-1692.53	-1303.6	-794.1	-968.67	-201.29	-576.95

-1932.65	-401.82	-2485.36	-1929.5	-1331.38	-1683.55	-410.36	-795.46

NA	NA	NA	NA	NA	NA	NA	NA

-185.16	-63.02	-327.97	-188.31	-189.36	-165.27	-116.3	-671.94

-5689.82	-1100.59	-7542.72	-5530.55	-3997.56	-5003.16	-1077.09	-1693.72

-6793.01	-1481.62	-8762.94	-6713.31	-4716.3	-5522.38	-1359.27	-2174.16

-347.97	-131.22	-469.47	-367.97	-314.73	-388	-115.64	-208.12

| -32.2 NA | |  |  | -14.49 | -26.29 | | -399.07 | -535.05 | | -121.99 | -518.02 |
| --- | --- | --- | --- | --- | --- | --- | --- | --- | --- | --- | --- |
| -74.73 NA | |  |  | -56.31 | -46.09 | | -341.78 | -544.27 | | -110.71 | -327.6 |
| -129.93 | | -24.4 |  | -85.63 | -107.41 | | -513.22 | -686.55 | | -178.65 | -718.72 |
| NA NA | |  | NA |  | NA | | -279.21 | -354.46 | | -115.2 | -648.44 |
| NA | NA |  | NA | NA | | NA | | NA | NA | | NA |
| NA | NA |  | NA | NA | | NA | | NA | NA | | NA |
| NA | NA |  | NA | NA | | NA | | NA | NA | | NA |
| NA | NA |  | NA | NA | | NA | | NA | NA | | NA |
| NA | NA |  | NA | NA | | NA | | NA | NA | | NA |
| NA | NA |  | NA | NA | | NA | | NA | NA | | NA |
| NA | NA |  | NA | NA | | NA | | NA | NA | | NA |
| NA | NA |  | NA | NA | | NA | | NA | NA | | NA |
| NA | NA |  | NA | NA | | NA | | NA | NA | | NA |
| NA | NA |  | NA | NA | | NA | | NA | NA | | NA |
| NA | NA |  | NA | NA | | NA | | NA | NA | | NA |
| NA | NA |  |  | -7.59 NA | | NA | |  | -17.01 NA | | NA |
| NA | NA |  | NA | NA | | NA | | NA | NA | | NA |
| NA | NA |  | NA | NA | | NA | | NA | NA | | NA |
|  | -16.77 NA |  |  | -39.88 | | -11.74 NA | |  | -28.48 NA | | NA |
| NA | NA |  | NA | NA | | NA | | NA | NA | | NA |
| NA | NA |  | NA | NA | | NA | | NA | NA | | NA |

-5123.61	-1344.23	-6294.71	-5043.01	-3609.37	-4048.81	-1105.89	-1886.18

-93.5 NA	-125.45	-66.29	-40.16	-128.76 NA	NA

-93.5 NA	-125.45	-66.29	-40.16	-128.76 NA	NA NA	NA	-5.82 NA	NA	NA	NA	NA NA	NA	-5.82 NA	NA	NA	NA	NA NA	NA	-19.22 NA	NA	-10.21 NA	NA

-77.25 NA	-144.05	-40.72	-19.71	-74.18 NA	NA NA	NA	NA	NA	NA	NA	NA	NA NA	NA	NA	NA	NA	NA	NA	NA NA	NA	NA	NA	NA	NA	NA	NA NA	NA	NA	NA	NA	NA	NA	NA NA	NA	NA	NA	NA	NA	NA	NA NA	NA	NA	NA	NA	NA	NA	NA NA	NA	NA	NA	NA	NA	NA	NA NA	NA	NA	NA	NA	NA	NA	NA

-43.03	-28.85	-38.15	-37.25	-61.02	-32.22	-53.13	-159.89

-103.27	-80.92	-56.21	-117.04	-189.38	-134.06	-139.8	-1087.28

-103.27	-80.92	-56.21	-117.04	-189.38	-134.06	-139.8	-1087.28

| -175.66 | | -183.89 | |  | -92.79 | -163.24 | | -298.94 | | -240.87 | |  | -271.3 | -1566.85 | |
| --- | --- | --- | --- | --- | --- | --- | --- | --- | --- | --- | --- | --- | --- | --- | --- |
| NA |  | NA |  | NA |  | NA |  | NA |  | NA |  |  | -13.54 | -301.87 | |
| -204.26 | |  | -89.24 | -209.08 | | -218.97 | | -189.51 | | -192.74 | | -125.11 | | -651.73 | |
|  | -22.73 |  | -39.59 | NA |  |  | -35.19 |  | -56.18 |  | -19.37 |  | -79.9 | -543.61 | |
| -239.89 | |  | -75.01 | -238.25 | | -264.91 | | -250.84 | | -253.59 | | -127.63 | | -392.78 | |
| NA |  | NA |  | NA |  | NA |  | NA |  | NA |  | NA |  | NA |  |
| NA |  | NA |  | NA |  | NA |  | NA |  | NA |  | NA |  | NA |  |
| NA |  | NA |  | NA |  | NA |  | NA |  | NA |  | NA |  | NA |  |
| NA |  | NA |  | NA |  | NA |  |  | -19.45 |  | -5.4 |  | -23.47 |  | -69.32 |
| NA |  | NA |  | NA |  | NA |  |  | -5.54 |  | -6.55 |  | -11.61 |  | -43.42 |
|  | -17.89 | NA |  |  | -12.91 |  | -15.88 |  | -57.76 |  | -29.54 |  | -40.33 | -154.49 | |
| NA |  | NA |  | NA |  | NA |  | NA |  | NA |  | NA |  | NA |  |
| NA |  | NA |  | NA |  | NA |  | NA |  | NA |  | NA |  | NA |  |
| NA |  | NA |  | NA |  | NA |  | NA |  | NA |  | NA |  | NA |  |
| NA |  | NA |  | NA |  | NA |  | NA |  | NA |  | NA |  | NA |  |
| NA |  | NA |  | NA |  | NA |  | NA |  | NA |  | NA |  | NA |  |
| NA |  | NA |  | NA |  | NA |  | NA |  | NA |  | NA |  | NA |  |
| NA |  | NA |  | NA |  | NA |  | NA |  | NA |  | NA |  | NA |  |
| NA |  | NA |  | NA |  | NA |  | NA |  | NA |  | NA |  | NA |  |
| NA |  | NA |  | NA |  | NA |  | NA |  | NA |  | NA |  | NA |  |
| NA |  | NA |  | NA |  | NA |  | NA |  | NA |  | NA |  | NA |  |
| NA |  | NA |  | NA |  | NA |  | NA |  | NA |  | NA |  | NA |  |
| NA |  | NA |  | NA |  | NA |  | NA |  | NA |  | NA |  | NA |  |
| NA |  | NA |  | NA |  | NA |  | NA |  | NA |  | NA |  | NA |  |
| NA |  | NA |  | NA |  | NA |  | NA |  | NA |  | NA |  | NA |  |
| NA |  | NA |  | NA |  | NA |  | NA |  | NA |  | NA |  | NA |  |
| NA |  | NA |  | NA |  | NA |  | NA |  | NA |  | NA |  | NA |  |
| NA |  | NA |  | NA |  | NA |  | NA |  | NA |  | NA |  | NA |  |
| NA |  | NA |  | NA |  | NA |  | NA |  | NA |  | NA |  | NA |  |
| NA |  | NA |  | NA |  | NA |  | NA |  | NA |  | NA |  | NA |  |
| NA |  | NA |  | NA |  | NA |  | NA |  |  | -21.08 | NA |  | NA |  |
| NA |  | NA |  | NA |  | NA |  | NA |  | NA |  | NA |  | NA |  |
| NA |  | NA |  | NA |  | NA |  | NA |  | NA |  | NA |  | NA |  |
| NA |  | NA |  | NA |  | NA |  | NA |  | NA |  | NA |  | NA |  |
| NA |  | NA |  | NA |  | NA |  | NA |  | NA |  | NA |  | NA |  |
| NA |  | NA |  | NA |  | NA |  | NA |  | NA |  | NA |  | NA |  |
| NA |  | NA |  | NA |  | NA |  | NA |  | NA |  | NA |  | NA |  |
| NA |  | NA |  | NA |  | NA |  | NA |  | NA |  | NA |  | NA |  |
| NA |  | NA |  | NA |  | NA |  | NA |  | NA |  | NA |  | NA |  |

| NA |  | NA |  | NA |  | NA |  | NA |  | NA |  | NA |  | NA |  |
| --- | --- | --- | --- | --- | --- | --- | --- | --- | --- | --- | --- | --- | --- | --- | --- |
| NA |  | NA |  | NA |  | NA |  | NA |  | NA |  | NA |  | NA |  |
| NA |  | NA |  | NA |  | NA |  | NA |  | NA |  | NA |  | NA |  |
| NA |  | NA |  | NA |  | NA |  | NA |  | NA |  | NA |  | NA |  |
| NA |  | NA |  | NA |  | NA |  | NA |  | NA |  | NA |  | NA |  |
| NA |  | NA |  | NA |  | NA |  | NA |  |  | -11.45 | NA |  | NA |  |
| NA |  | NA |  | NA |  | NA |  | NA |  | NA |  | NA |  | NA |  |
| NA |  | NA |  | NA |  | NA |  | NA |  | NA |  | NA |  | NA |  |
| NA |  | NA |  | NA |  | NA |  | NA |  | NA |  | NA |  | NA |  |
| NA |  | NA |  | NA |  | NA |  | NA |  | NA |  | NA |  | NA |  |
| -740.74 | |  | -35.8 | -969.87 | | -658.15 | | -365.89 | | -691.28 | |  | -40.38 |  | -97.99 |
|  | -8.8 | NA |  |  | -19.92 |  | -6.76 |  | -16.9 | -400.19 | | NA |  |  | -10.2 |
|  | -14.05 | NA |  |  | -68.67 | NA |  |  | -38.68 |  | -145.6 | NA |  | NA |  |
| -295.47 | |  | -7 | -486.56 | | -226.35 | | -306.67 | | -592.37 | |  | -50.14 |  | -123.1 |
| -213.13 | |  | -46.52 | -305.34 | | -169.85 | | -463.73 | | -720.51 | | -145.84 | | -306.18 | |
| -177.52 | |  | -22.33 | -244.29 | | -136.47 | | -318.06 | | -527.51 | | -111.91 | | -257.75 | |
|  | -6.78 |  | -17.06 |  | -6.56 |  | -6.38 |  | -87.72 | -157.52 | |  | -60.87 |  | -604.4 |
|  | -27.75 | NA |  |  | -49.66 |  | -8.08 |  | -63.7 | -227.47 | | NA |  | NA |  |
| -365.94 | |  | -53.39 | -535.63 | | -281.39 | | -548.41 | | -923.58 | | -146.44 | | -396.84 | |
|  | -71.12 | NA |  | -120.37 | |  | -46.87 | -198.65 | | -400.96 | |  | -44.39 |  | -94.18 |
|  | -8.16 | NA |  | NA |  |  | -6.53 |  | -300.5 | -458.38 | | -112.28 | | -397.46 | |
|  | -10.83 | NA |  | NA |  |  | -14.65 | -284.42 | | -384.62 | | -105.49 | | -548.05 | |
|  | -68.1 |  | -12.41 |  | -31.36 |  | -56.45 | -458.88 | | -654.32 | | -155.79 | | -611.38 | |
| NA |  | NA |  | NA |  | NA |  | -238.33 | | -374.35 | |  | -91.45 | -502.77 | |
| -101.61 | | NA |  |  | -30.95 | -132.54 | |  | -54.43 |  | -82.29 | NA |  |  | -32.86 |
|  | -73.51 | NA |  |  | -53.99 |  | -74.64 |  | -76.64 | -138.15 | |  | -30.78 | -272.83 | |
|  | -48.39 |  | -20.33 |  | -10.56 |  | -69.66 |  | -85.78 |  | -81.23 |  | -40.69 | -343.48 | |
|  | -56.89 |  | -6.68 |  | -39.5 |  | -69.07 |  | -71.17 |  | -72.08 |  | -20.21 | -125.91 | |
|  | -56.89 |  | -6.68 |  | -39.5 |  | -69.07 |  | -71.17 |  | -72.08 |  | -20.21 | -125.91 | |
|  | -60.83 |  | -7.31 |  | -16.42 | -103.04 | |  | -65 |  | -62.33 |  | -8.16 |  | -88.99 |
| -246.45 | |  | -15.38 | -160.99 | | -319.46 | | -143.23 | | -181.84 | |  | -5.73 |  | -46.36 |
| -109.46 | | NA |  |  | -36.5 | -155.08 | |  | -65.14 |  | -83.22 | NA |  |  | -79.35 |
| -109.46 | | NA |  |  | -36.5 | -155.08 | |  | -65.14 |  | -83.22 | NA |  |  | -79.35 |
| -109.46 | | NA |  |  | -36.5 | -155.08 | |  | -65.14 |  | -83.22 | NA |  |  | -79.35 |
| -265.89 | |  | -20.89 | -193.22 | | -324.12 | |  | -116 | -114.42 | |  | -17.69 |  | -17.78 |
| -248.42 | |  | -87.57 | -288.95 | | -347.42 | | -166.67 | | -125.36 | |  | -68.07 | -211.94 | |
|  | -34.22 | NA |  | NA |  |  | -59.95 |  | -41.07 |  | -57.95 | NA |  |  | -150.4 |
| NA |  | NA |  | NA |  | NA |  | NA |  | NA |  | NA |  | NA |  |
| -136.32 | |  | -17.68 | -308.53 | | -121.05 | | -141.36 | | -195.85 | |  | -12.02 | NA |  |

| NA |  | NA |  | NA |  | NA |  | NA |  | NA |  | NA |  | NA |  |
| --- | --- | --- | --- | --- | --- | --- | --- | --- | --- | --- | --- | --- | --- | --- | --- |
| NA |  | NA |  | NA |  | NA |  | NA |  | NA |  | NA |  | NA |  |
| NA |  | NA |  | NA |  | NA |  | NA |  | NA |  | NA |  | NA |  |
| NA |  | NA |  | NA |  | NA |  | NA |  | NA |  | NA |  | NA |  |
| -306.75 | | -159.45 | | -510.73 | | -309.45 | | -328.75 | | -141.37 | | -222.84 | | -1074.22 | |
| -226.74 | |  | -81.41 | -252.02 | | -254.69 | | -322.83 | | -265.31 | | -147.43 | | -458.86 | |
|  | -90.19 |  | -30.15 |  | -69.91 |  | -88.4 | -169.83 | |  | -67.13 | -102.52 | | -540.35 | |
| -442.75 | | -202.73 | | -550.95 | | -396.34 | | -370.66 | | -382.85 | | -193.33 | | -459.76 | |
|  | -34.97 |  | -41.99 |  | -15.2 |  | -27.93 | -106.66 | |  | -25.96 |  | -84.26 | -457.04 | |
|  | -85.79 |  | -61.25 |  | -79.42 |  | -70.69 | -141.63 | |  | -64.21 | -101.07 | | -419.61 | |
| -268.92 | |  | -63.85 | -245.51 | | -270.52 | | -333.68 | | -439.62 | | -159.48 | | -1068.9 | |
| -118.99 | | -177.97 | | -132.49 | | -153.32 | | -127.52 | | -130.11 | |  | -93.17 | -186.63 | |
| -300.41 | | -244.33 | |  | -233.1 | -340.78 | | -204.49 | | -262.86 | | -111.53 | |  | -254.5 |
|  | -56.26 |  | -7.57 | -108.05 | |  | -61.33 |  | -93.74 |  | -92.49 |  | -44.88 | -283.95 | |
| NA |  | NA |  | NA |  | NA |  | NA |  | NA |  | NA |  | NA |  |
| NA |  | NA |  | NA |  | NA |  | NA |  | NA |  | NA |  | NA |  |
| -181.04 | |  | -38.4 | -152.24 | | -168.96 | | -250.54 | | -368.88 | | -137.75 | | -1200.87 | |
| NA |  | NA |  | NA |  | NA |  | NA |  | NA |  | NA |  | NA |  |
| -312.94 | | -112.32 | | -620.37 | | -314.28 | | -298.49 | | -220.56 | | -149.06 | |  | -591.7 |
| NA |  | NA |  | NA |  | NA |  | NA |  | NA |  | NA |  | NA |  |
| NA |  | NA |  | NA |  | NA |  | NA |  | NA |  | NA |  | NA |  |
| -203.54 | |  | -93.13 | -226.74 | | -189.62 | | -171.76 | | -177.31 | |  | -94.39 | -302.29 | |
| -245.73 | |  | -50.81 | -180.98 | | -248.09 | | -273.13 | | -481.89 | | -129.12 | | -861.12 | |
| -245.73 | |  | -50.81 | -180.98 | | -248.09 | | -273.13 | | -481.89 | | -129.12 | | -861.12 | |
| -245.73 | |  | -50.81 | -180.98 | | -248.09 | | -273.13 | | -481.89 | | -129.12 | | -861.12 | |
| -245.73 | |  | -50.81 | -180.98 | | -248.09 | | -273.13 | | -481.89 | | -129.12 | | -861.12 | |
| -332.24 | |  | -65.03 | -484.78 | | -312.36 | |  | -283 | -361.69 | |  | -97.31 | -181.81 | |
| -332.24 | |  | -65.03 | -484.78 | | -312.36 | |  | -283 | -361.69 | |  | -97.31 | -181.81 | |
| -332.24 | |  | -65.03 | -484.78 | | -312.36 | |  | -283 | -361.69 | |  | -97.31 | -181.81 | |
| -332.24 | |  | -65.03 | -484.78 | | -312.36 | |  | -283 | -361.69 | |  | -97.31 | -181.81 | |
| -167.24 | |  | -32.83 | -283.23 | |  | -157 | -143.55 | | -354.67 | |  | -44.34 |  | -90.23 |
| -167.24 | |  | -32.83 | -283.23 | |  | -157 | -143.55 | | -354.67 | |  | -44.34 |  | -90.23 |
|  | -144.6 |  | -18.08 | -239.35 | | -111.17 | |  | -95.3 | -250.86 | |  | -17.17 |  | -46.79 |
|  | -144.6 |  | -18.08 | -239.35 | | -111.17 | |  | -95.3 | -250.86 | |  | -17.17 |  | -46.79 |
| -471.43 | | -136.18 | |  | -384 |  | -466 | -580.27 | | -842.79 | |  | -275.7 | -1607.69 | |
| -370.58 | | -158.87 | | -719.29 | | -370.52 | | -329.44 | | -188.57 | | -231.88 | | -1142.64 | |
| -370.58 | | -158.87 | | -719.29 | | -370.52 | | -329.44 | | -188.57 | | -231.88 | | -1142.64 | |
| -329.45 | |  | -80.83 | -681.54 | |  | -326.7 | -281.85 | | -250.41 | | -123.35 | | -494.06 | |
| -329.45 | |  | -80.83 | -681.54 | |  | -326.7 | -281.85 | | -250.41 | | -123.35 | | -494.06 | |

| -367.56 | | -129.41 | | -700.36 | | -407.86 | | -318.69 | | -199.73 | | -209.32 | | -1069.23 | |
| --- | --- | --- | --- | --- | --- | --- | --- | --- | --- | --- | --- | --- | --- | --- | --- |
| -484.89 | |  | -188.9 | -906.69 | | -517.17 | | -450.69 | | -303.12 | | -257.01 | | -1243.92 | |
| -250.07 | |  | -104.7 |  | -425.7 |  | -252.6 | -255.29 | | -188.23 | | -155.23 | | -795.91 | |
|  | -75.69 |  | -8.35 | -112.34 | |  | -60.41 |  | -45.13 |  | -60.99 |  | -26.18 | -172.33 | |
| -114.95 | |  | -23.2 | -138.73 | | -110.33 | | -339.72 | | -372.45 | | -132.21 | | -433.15 | |
| NA |  | NA |  | NA |  | NA |  | -232.42 | | -375.12 | |  | -81.69 | -349.03 | |
| -138.53 | |  | -44.3 | -144.34 | | -124.43 | | -328.69 | | -259.74 | | -165.78 | | -607.12 | |
| -134.82 | |  | -43.96 |  | -11.75 | -112.84 | | -128.58 | |  | -38.49 | -112.87 | |  | -391.3 |
| -148.88 | |  | -54.44 |  | -17.28 | -130.83 | | -141.51 | |  | -52.17 | -138.66 | | -439.78 | |
| NA |  | NA |  | NA |  | NA |  | NA |  | NA |  |  | -9.46 | -139.98 | |
| -147.25 | |  | -56.02 |  | -18.04 | -139.04 | | -148.09 | |  | -59.03 | -133.79 | | -439.69 | |
|  | -78.86 |  | -18.92 |  | -49.3 |  | -73.74 |  | -85.05 |  | -56.02 |  | -53.46 | -162.31 | |
| NA |  | NA |  | NA |  | NA |  |  | -28.21 |  | -36.61 |  | -23.03 | -598.54 | |
|  | -103.1 |  | -28.3 |  | -80.3 | -116.91 | | -143.78 | | -215.48 | |  | -59.32 | -532.82 | |
| NA |  | NA |  | NA |  | NA |  |  | -10.38 |  | -13.45 |  | -11.15 | -638.46 | |
| NA |  | NA |  | NA |  | NA |  | NA |  | NA |  | NA |  |  | -57.3 |
| NA |  | NA |  | NA |  | NA |  | NA |  | NA |  | NA |  | NA |  |
|  | -11.36 | NA |  |  | -17.18 |  | -10.25 |  | -9.83 |  | -24.36 | NA |  |  | -20.71 |
| NA |  | NA |  | NA |  | NA |  | NA |  | NA |  | NA |  |  | -7.45 |
| NA |  | NA |  | NA |  | NA |  | NA |  | NA |  | NA |  |  | -8.64 |
| NA |  | NA |  | NA |  | NA |  | NA |  | NA |  | NA |  |  | -89.08 |
| NA |  | NA |  | NA |  | NA |  | NA |  | NA |  | NA |  |  | -77.86 |
| NA |  | NA |  | NA |  | NA |  | NA |  | NA |  | NA |  |  | -29.38 |
|  | -31.27 | NA |  |  | -40.35 |  | -24.85 |  | -25.27 |  | -71.76 | NA |  |  | -8.06 |
|  | -146.2 | NA |  | -154.01 | | -138.18 | |  | -70.65 | -142.88 | | NA |  |  | -17.93 |
| NA |  | NA |  | NA |  | NA |  | NA |  | NA |  | NA |  | NA |  |
| NA |  | NA |  | NA |  | NA |  | NA |  | NA |  | NA |  | NA |  |
| NA |  | NA |  | NA |  | NA |  | NA |  | NA |  | NA |  | NA |  |
| -443.05 | |  | -91.6 | -399.71 | | -434.05 | | -524.86 | | -817.02 | | -235.74 | | -1423.91 | |
| -302.94 | |  | -43.09 |  | -286.7 | -306.54 | | -156.04 | | -220.89 | |  | -25.88 |  | -50.61 |
| -302.94 | |  | -43.09 |  | -286.7 | -306.54 | | -156.04 | | -220.89 | |  | -25.88 |  | -50.61 |
| NA |  | NA |  | NA |  | NA |  | NA |  | NA |  | NA |  | NA |  |
| NA |  | NA |  | NA |  | NA |  |  | -6.96 | NA |  |  | -26.1 | -241.93 | |
| NA |  | NA |  | NA |  | NA |  | NA |  | NA |  | NA |  | -146.86 | |
|  | -8.42 | NA |  | NA |  |  | -9.29 |  | -16.66 |  | -25.83 | NA |  |  | -74.33 |
| -606.55 | | -277.39 | | -642.73 | | -619.72 | | -466.33 | | -411.21 | | -218.02 | | -610.35 | |
| NA |  | NA |  | NA |  | NA |  |  | -17.01 |  | -15.62 |  | -20.33 | -102.05 | |
| NA |  | NA |  | NA |  | NA |  | NA |  | NA |  | NA |  | -222.88 | |
| NA |  | NA |  | NA |  | NA |  |  | -6.59 | NA |  |  | -17.48 | -133.95 | |

| NA |  | NA |  |  | -8.67 | NA |  |  | -19.01 |  | -24.24 | NA |  |  | -23.78 |
| --- | --- | --- | --- | --- | --- | --- | --- | --- | --- | --- | --- | --- | --- | --- | --- |
| NA |  | NA |  | NA |  | NA |  | NA |  | NA |  | NA |  | NA |  |
| NA |  | NA |  | NA |  | NA |  | NA |  | NA |  | NA |  | NA |  |
| NA |  | NA |  | NA |  | NA |  | NA |  | NA |  |  | -7.86 |  | -38.08 |
| NA |  | NA |  | NA |  | NA |  | NA |  | NA |  |  | -12.37 | -166.44 | |
| -219.68 | |  | -89.66 | -277.07 | | -233.43 | | -313.21 | | -311.11 | | -144.94 | | -352.13 | |
| NA |  | NA |  | NA |  | NA |  | NA |  | NA |  | NA |  | NA |  |
| NA |  | NA |  | NA |  | NA |  | NA |  | NA |  | NA |  | NA |  |
| NA |  | NA |  | NA |  | NA |  | NA |  | NA |  | NA |  | NA |  |
| NA |  | NA |  | NA |  | NA |  | NA |  | NA |  | NA |  | NA |  |
|  | -23 |  | -23.34 |  | -15.74 |  | -21.68 |  | -45.14 |  | -18.51 |  | -27.28 | -715.04 | |
|  | -6.11 |  | -7.34 | NA |  |  | -5.68 |  | -40.14 |  | -24.84 |  | -31.78 | -904.11 | |
|  | -84.13 |  | -30.72 |  | -85.06 |  | -97.39 | -164.82 | | -155.44 | |  | -93.94 | -471.55 | |
| NA |  | NA |  | NA |  | NA |  | NA |  | NA |  | NA |  | NA |  |
| NA |  |  | -21.7 | NA |  |  | -10.42 |  | -25.73 | NA |  |  | -33.65 | -1383.65 | |
|  | -7.74 | NA |  |  | -14.97 | NA |  |  | -14.5 |  | -28.25 | NA |  | -270.67 | |
|  | -25.72 | NA |  |  | -9.99 |  | -20.45 |  | -61.74 |  | -77.91 |  | -19.15 | -206.75 | |
| NA |  |  | -20.1 | NA |  | NA |  |  | -21.04 | NA |  |  | -54 | -840.13 | |
| NA |  | NA |  | NA |  | NA |  | NA |  | NA |  | NA |  | NA |  |
|  | -88.41 |  | -62.27 |  | -62.4 | -104.42 | |  | -89.98 |  | -23.74 |  | -96.62 | -584.97 | |
| -214.69 | | -103.16 | | -249.95 | | -286.64 | | -186.05 | | -131.73 | | -102.07 | | -437.56 | |
|  | -38.94 | NA |  |  | -22.74 |  | -24.73 |  | -32.82 |  | -47.66 |  | -5.15 |  | -72.54 |
|  | -21.23 | NA |  |  | -14.75 |  | -13.12 |  | -25.18 |  | -27.89 | NA |  |  | -43.85 |
| -507.29 | | -304.32 | | -489.63 | | -498.69 | | -268.24 | | -122.56 | | -116.41 | | -153.87 | |
| -189.63 | |  | -53.45 | -253.09 | | -186.99 | | -176.79 | | -163.96 | |  | -76.62 | -312.75 | |
|  | -12.26 | NA |  | NA |  |  | -11.76 |  | -39.21 |  | -25.17 |  | -19.32 | -137.47 | |
| -136.14 | |  | -38.19 | -102.24 | | -137.87 | | -223.18 | | -294.46 | |  | -92.06 | -616.42 | |
|  | -33.81 | NA |  |  | -10.34 |  | -32.87 |  | -68.49 | -101.83 | |  | -26.59 | -363.25 | |
| -138.47 | |  | -60.82 | -187.81 | | -181.08 | | -198.65 | | -195.82 | | -101.42 | |  | -341.3 |
|  | -28.04 |  | -20.22 |  | -29.11 |  | -45.39 |  | -52.7 |  | -28.62 |  | -46.74 | -274.58 | |
|  | -44.63 |  | -17.5 |  | -54.54 |  | -46.74 |  | -44.93 |  | -57.04 |  | -27.33 |  | -59.32 |
|  | -60.11 |  | -19.83 |  | -62.53 |  | -65.44 |  | -72.37 |  | -77.43 |  | -44.26 | -154.55 | |
|  | -80.18 |  | -33.58 | -108.26 | |  | -75.67 | -113.07 | | -112.47 | |  | -59.17 | -128.65 | |
| -104.94 | |  | -39.74 | -106.08 | | -116.21 | | -129.59 | |  | -94.61 |  | -81.23 | -386.23 | |
|  | -44.46 |  | -13.47 |  | -67.99 |  | -50.64 |  | -45.83 |  | -75.25 |  | -15.13 |  | -46.66 |
|  | -13 | NA |  |  | -9.1 |  | -7.21 |  | -9.53 |  | -20.54 |  | -6.96 |  | -22.02 |
| NA |  | NA |  | NA |  | NA |  | NA |  | NA |  | NA |  |  | -52.9 |
| NA |  | NA |  | NA |  | NA |  | NA |  | NA |  | NA |  |  | -52.9 |
| NA |  | NA |  | NA |  | NA |  | NA |  | NA |  | NA |  |  | -52.9 |

| -1551.28 | | -325.67 | | -2851.31 | | -1745.39 | | -1313.45 | | -1718.89 | | -469.75 | |  | -703.6 |
| --- | --- | --- | --- | --- | --- | --- | --- | --- | --- | --- | --- | --- | --- | --- | --- |
| -2004.52 | | -392.24 | | -3600.52 | | -2226.02 | | -1608.02 | | -2083.56 | | -514.35 | |  | -706.8 |
| -1099.98 | |  | -265.2 | -1968.28 | | -1340.23 | | -802.23 | | -1002.1 | |  | -235 | -287.72 | |
|  | -12.28 | NA |  |  | -5.62 |  | -7.57 |  | -14.88 |  | -20.98 | NA |  |  | -58.37 |
| NA |  | NA |  | NA |  | NA |  | NA |  | NA |  |  | -9.5 | -280.38 | |
| NA |  | NA |  | NA |  | NA |  | NA |  | NA |  | NA |  |  | -11.4 |
| NA |  | NA |  | NA |  | NA |  | NA |  | NA |  | NA |  |  | -14.83 |
|  | -9.23 | NA |  |  | -15.9 |  | -9.57 |  | -26.53 |  | -56.66 | NA |  |  | -16.25 |
| -127.61 | |  | -30.33 | -134.17 | | -126.95 | | -125.75 | |  | -73.69 |  | -61.31 | -386.78 | |
| -131.67 | |  | -37.94 | -122.87 | |  | -136.2 | -138.35 | | -113.09 | |  | -87.08 | -362.98 | |
| -122.69 | |  | -41.83 | -120.67 | | -111.01 | | -137.46 | |  | -94.78 |  | -81.06 | -334.32 | |
| -122.69 | |  | -41.83 | -120.67 | | -111.01 | | -137.46 | |  | -94.78 |  | -81.06 | -334.32 | |
| -159.39 | |  | -61.47 | -165.93 | | -142.13 | | -155.51 | | -119.49 | |  | -78.46 | -274.22 | |
|  | -19.55 |  | -5.46 | NA |  |  | -11.85 |  | -316.1 | -405.06 | | -133.89 | | -720.63 | |
|  | -19.55 |  | -5.46 | NA |  |  | -11.85 |  | -316.1 | -405.06 | | -133.89 | | -720.63 | |
|  | -17.35 | NA |  |  | -56.91 |  | -22.96 |  | -14.2 |  | -18.8 | NA |  | NA |  |
| NA |  | NA |  |  | -28.1 | NA |  | NA |  |  | -5.63 | NA |  | NA |  |
| NA |  | NA |  |  | -13.43 | NA |  | NA |  | NA |  | NA |  | NA |  |
| -180.11 | | NA |  |  | -91.44 | -231.29 | |  | -73.67 | -107.53 | | NA |  |  | -18.98 |
| -2015.89 | | -537.27 | | -2437.13 | | -1969.93 | | -1538.76 | | -1822.25 | | -539.86 | | -822.07 | |
| -1780.27 | | -188.33 | | -3171.83 | | -1585.35 | | -909.16 | | -1371.91 | | -132.11 | | -869.04 | |
| -982.36 | |  | -16.93 | -1445.35 | | -818.74 | | -360.69 | | -631.09 | |  | -13.74 |  | -129.2 |
| -1751.12 | | -226.87 | | -2551.22 | | -1592.97 | | -896.88 | | -1268.88 | |  | -181.1 | -444.95 | |
| -2570.24 | | -279.05 | | -4304.63 | | -2233.03 | | -1253.66 | | -1963.73 | | -216.06 | | -1102.36 | |
| -2002.69 | | -161.28 | | -3096.85 | | -1738.7 | | -957.35 | | -1548.01 | |  | -123.1 | -526.87 | |
|  | -81.55 |  | -6.47 | -120.85 | |  | -55.02 |  | -71.52 | -104.63 | |  | -30.18 |  | -20.13 |
| -288.83 | | NA |  | -489.83 | | -191.21 | | -369.23 | | -720.66 | |  | -61.87 | -251.33 | |
|  | -134.3 |  | -15.28 | -149.69 | | -108.94 | | -154.98 | | -191.27 | |  | -63.68 |  | -55.96 |
|  | -29.28 | NA |  |  | -56.05 |  | -19.92 |  | -43.41 |  | -40.29 |  | -13.03 | -160.93 | |
| -465.83 | | -171.45 | | -699.93 | | -427.89 | | -441.58 | | -277.96 | | -246.52 | | -1162.77 | |
| -300.29 | |  | -95.22 | -624.39 | | -313.89 | | -245.63 | | -219.11 | | -105.55 | | -375.22 | |
|  | -98.45 |  | -45.1 | -255.41 | |  | -68.33 | -100.99 | |  | -48.23 |  | -78.18 | -958.29 | |
|  | -75.78 | NA |  | -197.64 | |  | -65.84 |  | -93.71 | -137.85 | | NA |  | NA |  |
| NA |  | NA |  | NA |  | NA |  | NA |  | NA |  | NA |  | NA |  |
| NA |  | NA |  | NA |  | NA |  | NA |  | NA |  | NA |  | NA |  |
|  | -31.62 |  | -60.33 |  | -8.18 |  | -49.34 |  | -70.82 | NA |  |  | -98.01 |  | -812.6 |
| -117.17 | |  | -79.46 | -103.61 | | -142.18 | | -184.08 | |  | -70.44 | -137.13 | | -908.55 | |
|  | -92.83 | -127.43 | |  | -80.59 | -117.67 | |  | -105.9 |  | -20.1 | -125.35 | | -813.55 | |
| NA |  |  | -10.86 | NA |  | NA |  | NA |  | NA |  |  | -8.76 | -261.87 | |

| -122.1 | -79.62 | -79.68 | -128.92 | -163.72 | -55.91 | -144.56 | -912.57 |
| --- | --- | --- | --- | --- | --- | --- | --- |
| -411.45 | -208.13 | -645.11 | -445.93 | -453.66 | -275.04 | -276.78 | -984.69 |
| -191.78 | -50.81 | -148.47 | -193.98 | -252.36 | -357.56 | -144.52 | -1111.88 |
| -141.08 | -46.18 | -112.25 | -171.75 | -236.33 | -288.44 | -167.98 | -1042.54 |
| -79.92 | -34.23 | -67.87 | -90.95 | -143.75 | -172.5 | -108.74 | -776.95 |
| -57.65 | -17.92 | -105.75 | -51.51 | -58.13 | -48.77 | -39.13 | -166.95 |

-7.56	-14.68 NA	NA	-24.29 NA	-48.98	-340.23

-176.99	-50.38	-127.75	-177.34	-244.35	-283.23	-180.41	-1178.25

-85.1	-39.84	-40.8	-93.19	-169.48	-187.99	-137.4	-1115.2

NA	NA	NA	NA	NA	NA	NA	NA NA	NA	NA	NA	NA	NA	NA	NA NA	NA	NA	NA	NA	NA	NA	NA

-646.25	-357.26	-639.33	-629.5	-417.94	-228.36	-189.17	-391.05

-248.79	-151.7	-218.23	-275.18	-289.52	-283.07	-197.5	-715.26

-24.97	-8.97	-104.49	-28.17	-35.89	-11.23	-23.67	-285.96

-126.25	-68.78	-88.8	-113.24	-156.36	-171.36	-88.68	-659.71

-19.79	-6.12	-96.32	-18.93	-24.97	-8.87	-23.41	-239.72

-80.89	-38.52	-73.89	-123.02	-132.8	-141.94	-36.94	-273.66

-55.23	-52.01	-36.75	-70.05	-88.22	-31.28	-83.22	-429.08

NA	NA	NA	NA	NA	NA	NA	NA

-314.25	-251.28	-379.45	-316.42	-378.7	-300.71	-277.46	-667.86

-14.64 NA	-6.66	-6.18	-45.74	-34.14	-23.87	-177.58

NA	-26.85 NA	NA	-7.44 NA	-70.01	-689.98

NA	-10.61 NA	NA	-11 NA	-80.69	-812.11

-194.34	-123.78	-163.71	-228.16	-128	-108.26	-124.15	-546.59

-263.84	-44.7	-269.86	-329.19	-108.98	-130.43	-25.69	-100.33

-190.84	-120.79	-209.43	-215.64	-114.08	-99.1	-71.95	-242.4

-144.96	-136.53	-122.51	-163.64	-115.53	-51.68	-121.39	-643.45

-94.11 NA	-56.58	-96	-61.88	-67.01 NA	-47.8

-241.73	-99.11	-240.28	-228.19	-184.1	-218.84	-123.33	-459.76

-271.08	-31.43	-273.22	-315.38	-215.3	-304.53	-22.83	-100.38

-283.52	-207.07	-329.59	-284.66	-331.58	-247.7	-230.94	-574.66

-158.65	-39.5	-173.67	-168.76	-152.23	-141.29	-69.21	-207.61

-142.75	-12.16	-201.67	-152.54	-143.23	-179.68	-70.01	-234.25

-69.38	-63.13	-31.71	-79.43	-54.76	-35.82	-5.62	-78.4

-198.71	-17.4	-264.77	-164.61	-142.34	-226.22	-21.19	-22.12

-403.02	-37.74	-554.55	-360.02	-316.54	-502.95	-58.18	-61.27

-96.71	-31.09	-93.96	-91.52	-122.38	-82.44	-45.17	-183.97

-196.64	-247.14	-235.69	-201.58	-204.15	-143.78	-160.09	-327.62

| -132.83 | |  | -70.95 |  | -164.3 | -133.39 | |  | -118.5 |  | -92.65 |  | -71.81 | -241.93 | |
| --- | --- | --- | --- | --- | --- | --- | --- | --- | --- | --- | --- | --- | --- | --- | --- |
| -271.82 | |  | -86.61 | -348.46 | | -291.08 | | -192.26 | | -136.67 | |  | -98.81 | -238.79 | |
| -208.13 | |  | -79.4 | -328.28 | | -210.45 | | -254.25 | | -295.49 | | -150.04 | | -458.49 | |
|  | -96.77 |  | -30.01 | -115.58 | |  | -96.75 |  | -75.78 |  | -56.87 |  | -37.87 | -171.45 | |
| -406.02 | | -162.76 | | -426.56 | |  | -392.5 | -220.36 | |  | -517.4 |  | -48.75 |  | -28.16 |
| NA |  | NA |  | NA |  | NA |  |  | -8.07 |  | -78.66 | NA |  | NA |  |
| -355.16 | | -129.54 | | -457.88 | | -344.17 | | -337.93 | | -283.67 | | -167.28 | | -469.49 | |
|  | -88.51 | NA |  | -125.43 | |  | -95.15 | -121.24 | |  | -38.43 |  | -94.33 |  | -489.4 |
| -139.96 | |  | -88.24 | -128.86 | | -136.58 | | -164.96 | | -113.88 | | -108.59 | | -430.59 | |
| -125.73 | |  | -32.18 | -189.68 | |  | -122.6 |  | -93.4 | -137.96 | |  | -64.32 |  | -97.48 |
| -189.14 | |  | -27.77 | -153.02 | | -188.28 | | -141.11 | |  | -89.89 |  | -52.24 | -223.21 | |
| NA |  | NA |  | NA |  | NA |  | NA |  | NA |  | NA |  | NA |  |
| -155.68 | |  | -38.56 | -165.79 | | -145.99 | | -158.27 | | -123.13 | |  | -73.12 | -260.16 | |
|  | -129.4 |  | -18.4 |  | -93.53 | -158.07 | |  | -67.39 |  | -87.37 |  | -9.86 |  | -94.47 |
| -236.38 | |  | -59.9 | -187.36 | |  | -281.8 | -165.87 | | -177.16 | |  | -50.53 | -284.16 | |
| -523.78 | | -317.42 | |  | -670.9 | -543.27 | | -521.81 | | -338.37 | | -327.37 | | -942.16 | |
|  | -49.78 |  | -34.82 |  | -33.85 |  | -48.49 |  | -59.66 |  | -13.72 |  | -42.17 |  | -274.7 |
| -221.09 | | -249.75 | | -282.37 | | -243.64 | | -264.98 | | -127.57 | | -243.38 | | -725.04 | |
| NA |  | NA |  | NA |  | NA |  | NA |  |  | -23.75 | NA |  | -140.56 | |
| -106.75 | |  | -12.18 | -137.28 | | -106.65 | | -107.18 | |  | -85.93 |  | -24.08 | -143.75 | |
| -309.19 | |  | -22.28 | -356.22 | | -328.61 | | -262.84 | | -316.53 | |  | -47.38 |  | -153 |
|  | -61.52 | NA |  | -117.86 | |  | -61.13 |  | -38.49 |  | -68.4 | NA |  | NA |  |
|  | -76.04 |  | -52.72 |  | -84.31 |  | -87.63 | -151.15 | |  | -64.42 |  | -134.5 | -536.27 | |
| -248.33 | |  | -11.84 | -277.11 | | -272.93 | |  | -80.24 |  | -65.21 | NA |  | NA |  |
| -130.48 | | NA |  |  | -93.24 | -139.72 | | -143.92 | |  | -68.26 |  | -86.35 | -511.37 | |
| -193.64 | |  | -19.85 |  | -279.6 | -170.24 | | -152.14 | | -213.78 | |  | -17.37 |  | -44.76 |
| -138.77 | |  | -29.77 | -120.88 | | -117.82 | |  | -99.27 | -112.91 | |  | -43.57 | -127.46 | |
| -217.48 | |  | -20.55 | -183.73 | | -286.17 | | -105.79 | | -106.67 | |  | -11.16 |  | -41.88 |
|  | -81.76 |  | -29.63 | -108.84 | |  | -75.34 | -112.75 | | -105.42 | |  | -46.72 | -196.54 | |
|  | -59.52 | NA |  |  | -50.45 |  | -51.74 |  | -55.12 |  | -74.58 |  | -23.82 |  | -69.58 |
| -283.17 | |  | -12.37 | -314.53 | |  | -255.3 | -113.12 | | -179.34 | | NA |  |  | -33.8 |
| -268.62 | | -191.05 | | -209.66 | | -296.41 | | -229.66 | | -153.66 | | -118.48 | | -515.96 | |
| -119.86 | |  | -42.39 |  | -83.19 | -114.22 | |  | -87.6 |  | -66.28 |  | -42.39 |  | -233.7 |
|  | -262.6 |  | -44.1 | -444.87 | | -275.01 | | -191.25 | | -269.38 | |  | -49.94 |  | -49.86 |
| NA |  | NA |  | NA |  |  | -13.21 |  | -15.58 | NA |  |  | -12.11 | -165.64 | |
| -101.99 | |  | -31.94 |  | -90.64 |  | -80.4 | -147.86 | | -103.16 | |  | -84.25 |  | -437.8 |
| -155.14 | |  | -72.79 | -200.66 | | -138.28 | | -116.15 | | -107.48 | |  | -68.56 | -110.41 | |
|  | -302 |  | -16.99 | -258.54 | | -307.47 | | -155.19 | | -150.83 | |  | -14.78 |  | -74.04 |

common_l common_ conventioncytotoxic_ early_neur effector_meffector_meffector_m

-184.07	-216.59	-204.45	-212.51	-28.83	-173.74	-137.63	-175.77

NA	NA	NA	NA	NA	NA	NA	NA

-190.18	-219.26	-194.57	-273.76 NA	-403.73	-387.68	-570.88

-129.24	-144.76	-290.38	-457.8 NA	-258.73	-284.89	-414.09

-22.76	-26.05	-47.42	-67.19 NA	-240.8	-300.07	-464.82

-823.74	-657	-655.72	-410.3	-27.4	-683.88	-597.8	-719.84

-176.8	-189.14	-235.88	-290.14 NA	-564.01	-604.48	-892.91

-39.32	-40.56	-71.46	-118.16 NA	-223.28	-214.68	-381.51

-39.32	-40.56	-71.46	-118.16 NA	-223.28	-214.68	-381.51

NA	NA	-80.47	-13.27 NA	-13.82	-8.26	-14.14

-488.16	-476	-391.14	-161.47 NA	-348.35	-306.25	-372.49

-186.77	-149.19	-150.34	-106.38 NA	-274.33	-237.06	-338.74

-90.27	-104.4	-414.03	-51.09 NA	-472.15	-324.66	-517.64

-101.61	-119.84	-491.72	-87.95 NA	-628.8	-477.45	-769.66

NA	NA	NA	NA	NA	NA	NA	NA NA	NA	NA	NA	-12.87 NA	NA	NA

NA	NA	-8.33 NA	NA	-212.81	-156.27	-201.04

NA	NA	-8.33 NA	NA	-212.81	-156.27	-201.04

-1843.61	-2049.91	-1573.11	-314.35 NA	-778.39	-430.32	-464.33

-197.61	-180.56	-167.31	-205.68 NA	-610.6	-626.96	-789.5

NA	NA	NA	NA	-12.86 NA	NA	NA

-1672.08	-1689.97	-904.82	-1325.91 NA	-1330.73	-1019.89	-1407.88

NA	NA	NA	NA	NA	NA	NA	NA NA	NA	NA	NA	NA	NA	NA	NA NA	NA	-76.66 NA	NA	NA	NA	NA NA	NA	-415.23 NA	NA	NA	NA	NA NA	NA	-462.55 NA	NA	NA	NA	NA NA	NA	-253.5 NA	NA	NA	NA	NA

-217.4	-230.44	-160.43	-254.8 NA	-568.34	-625.16	-785.58

-217.4	-230.44	-160.43	-254.8 NA	-568.34	-625.16	-785.58

-334.12	-343.71	-287.7	-278.44 NA	-831.73	-874.11	-1258.45

-365.18	-377.84	-280.93	-281.2 NA	-847.42	-897.28	-1233.49

NA	NA	-75.14 NA	NA	-14.47 NA	-11.58

NA	NA	-94.35 NA	NA	NA	NA	NA NA	NA	NA	NA	NA	NA	NA	NA NA	NA	NA	NA	NA	NA	NA	NA NA	NA	NA	NA	-8.44 NA	NA	NA

-204.99	-205.58	-176.91	-184.7	-48.7	-61.95	-26.2	-55.59

-52.59	-69.64	-74.57	-24.25	-11.22	-73.12	-73.09	-141.5

-52.59	-69.64	-74.57	-24.25	-11.22	-73.12	-73.09	-141.5

-52.59	-69.64	-74.57	-24.25	-11.22	-73.12	-73.09	-141.5

-154.64	-186.9	-113.15	-113.84	-17.57	-285.85	-183.37	-200.65

-122.77	-138.39	-102.38	-112.55	-24.7	-195	-147.93	-181.71

-3654.14	-4085.37	-2275.81	-1010.01 NA	-1586.16	-883.76	-1121.66

-4380.12	-4804.72	-3242.22	-1556.33 NA	-2051.72	-1170.78	-1391.03

-6279.37	-6852.57	-4821.45	-2370.25 NA	-3050.45	-1830.08	-2209.25

-5022.43	-5653.62	-3042.45	-1442.06 NA	-2200.97	-1292.79	-1539.29

-2796.28	-3027.21	-2154.69	-1027.89 NA	-1454.98	-850.53	-1008.37

-3693.55	-3959.42	-2998.87	-1427.27 NA	-1795.22	-1010.96	-1275.25

-5743.28	-6229.25	-4115.24	-1659.71 NA	-2302.69	-1278.29	-1540.73

-3876.6	-4128.63	-2942.53	-1881.84	-14.48	-2115.24	-1271.05	-1565.72

-4546.37	-4906.53	-3467.79	-2222.91 NA	-2867.63	-1797.79	-2167.1

-5512.92	-5968.81	-4010.46	-2607.09	-6.09	-3092.19	-1901.91	-2340.8

NA	NA	NA	NA	NA	NA	NA	NA

-56.56	-57.69	-58.78	-117.76 NA	-508.93	-597.45	-890.14

-5772.63	-6157.95	-4057.49	-2685.3 NA	-3158.16	-1924.5	-2266.46

-6514.31	-6923.52	-4523.81	-3164.13 NA	-3797.06	-2404.12	-2864.25

-470.52	-529.34	-362.4	-217.42 NA	-244.07	-189.48	-185.2

-8146.23	-8875.81	-5523.13	-3190.04 NA	-4243.44	-2561.45	-3120.37

NA	NA	NA	NA	NA	NA	-8.96	-32.46

NA	NA	NA	NA	NA	NA	NA	NA

-7154.03	-7761.69	-4987.94	-3213.36 NA	-4164.04	-2524.99	-3125.8

-284.37	-297.97	-211.77	-120.82 NA	-149.07	-86.6	-91.44

-3550.22	-3959.76	-2607.61	-1163.39 NA	-1533.56	-868.42	-1054.87

-6772.21	-7288.65	-4857.73	-3169.63	-10.14	-3726.06	-2252.86	-2741.23

-5122.46	-5456.8	-3420.31	-2293.53 NA	-2924.73	-1756.99	-2041.33

-2056.08	-2180.39	-1613.9	-1049.97 NA	-1314.41	-779.27	-962.1

-2056.08	-2180.39	-1613.9	-1049.97 NA	-1314.41	-779.27	-962.1

-7510.63	-8138.59	-5214.64	-3241.25 NA	-3988.94	-2393.09	-2876.84

-3244.04	-3487.56	-2270.74	-1512.89 NA	-1720.86	-1115.61	-1441.89

-2119.86	-2235.37	-1809.87	-562.07 NA	-697.02	-332.51	-424.58

-2238.6	-2461.26	-1586.19	-985.42 NA	-1455.7	-1010.09	-1289.69

NA	NA	NA	NA	NA	NA	NA	NA

-221.92	-168.29	-174.14	-137.42 NA	-319.23	-348.36	-411.07

-6840.21	-7303.07	-4497.9	-2802.35 NA	-3681.52	-2224.61	-2668.75

-8170.16	-8806.72	-5628.98	-3484.57 NA	-4358.48	-2627.94	-3212.88

-545.19	-553.24	-398.14	-253.5	-9.83	-325.02	-241.39	-268.92

-60.26	-84.25	-408.04	-15.03 NA	-595.76	-404.4	-675.02

-100.34	-120.58	-314.67	-16.41 NA	-490.48	-355.26	-532.64

-148.15	-177.35	-518.68	-60.07 NA	-768.25	-556.36	-845.39

NA	-9	-267.14	-9.44 NA	-465.15	-363.01	-655.53

NA	NA	NA	NA	NA	NA	NA	NA NA	NA	NA	NA	NA	NA	NA	NA NA	NA	NA	NA	NA	NA	NA	NA NA	NA	NA	NA	NA	NA	NA	NA NA	NA	NA	NA	NA	NA	NA	NA NA	NA	NA	NA	NA	NA	NA	NA NA	NA	NA	NA	NA	NA	NA	NA NA	NA	NA	NA	NA	NA	NA	NA NA	NA	NA	NA	NA	NA	NA	NA NA	NA	NA	NA	NA	NA	NA	NA NA	NA	NA	NA	NA	NA	NA	NA NA	-10.2 NA	NA	NA	NA	NA	NA NA	NA	NA	NA	NA	NA	NA	NA NA	NA	NA	NA	NA	NA	NA	NA

-34.16	-47.17 NA	NA	NA	NA	NA	NA NA	-9.2 NA	NA	NA	NA	NA	NA NA	NA	NA	NA	NA	NA	NA	NA

-5989.03	-6399.02	-4555.26	-2809.7	-15.19	-3250.74	-1927.34	-2349.21

-146.49	-352.95	-7.92	-14.18 NA	-27.46	-10.12	-5.27

-146.49	-352.95	-7.92	-14.18 NA	-27.46	-10.12	-5.27

NA	-20.43 NA	NA	NA	NA	NA	NA NA	-20.43 NA	NA	NA	NA	NA	NA

-20.64	-151.42 NA	NA	NA	NA	NA	NA

-153.45	-519 NA	-9.25 NA	NA	NA	NA NA	-5.58 NA	NA	NA	NA	NA	NA NA	-37.31 NA	NA	NA	NA	NA	NA NA	-37.31 NA	NA	NA	NA	NA	NA NA	-37.31 NA	NA	NA	NA	NA	NA NA	NA	NA	NA	NA	NA	NA	NA NA	NA	NA	NA	-6.76 NA	NA	NA NA	NA	NA	NA	-5.42 NA	NA	NA NA	NA	NA	NA	-5.14 NA	NA	NA

-68.38	-83.18	-47.12	-27.58 NA	-139.67	-122.26	-168.76

-65.34	-64.22	-137.42	-164.86	-7.05	-348.41	-382.14	-591.25

-65.34	-64.22	-137.42	-164.86	-7.05	-348.41	-382.14	-591.25

| -123.19 | | -114.96 | | -241.71 | | -363.29 | |  | -16.75 | -550.36 | | -620.64 | | -941.35 | |
| --- | --- | --- | --- | --- | --- | --- | --- | --- | --- | --- | --- | --- | --- | --- | --- |
| NA |  | NA |  | NA |  |  | -7.36 | NA |  |  | -30.73 |  | -42.66 | -104.87 | |
| -193.81 | | -182.46 | |  | -175.2 | -200.21 | |  | -30.93 | -302.27 | | -290.49 | | -418.08 | |
|  | -17.47 |  | -11.82 |  | -24.32 |  | -40.47 | NA |  | -145.73 | | -223.87 | | -349.74 | |
|  | -293 |  | -314.7 | -273.18 | | -175.58 | |  | -23.9 | -321.02 | | -278.52 | | -342.17 | |
| NA |  | NA |  | -100.12 | | NA |  | NA |  | NA |  | NA |  | NA |  |
| NA |  | NA |  | NA |  | NA |  | NA |  | NA |  | NA |  | NA |  |
| NA |  | NA |  | NA |  | NA |  | NA |  | NA |  | NA |  | NA |  |
| NA |  | NA |  |  | -18.93 | NA |  |  | -11.52 |  | -70.88 |  | -62.3 | -106.65 | |
| NA |  | NA |  |  | -16.24 | NA |  |  | -22.16 |  | -61.51 |  | -43.2 |  | -51.21 |
|  | -32.06 |  | -51.37 |  | -79.56 |  | -13.37 |  | -13.01 | -153.64 | | -137.93 | | -191.03 | |
| NA |  | NA |  | NA |  | NA |  |  | -6.27 | NA |  | NA |  | NA |  |
| NA |  | NA |  | NA |  | NA |  | NA |  | NA |  | NA |  | NA |  |
| NA |  | NA |  | NA |  | NA |  | NA |  | NA |  | NA |  | NA |  |
| NA |  | NA |  | NA |  | NA |  | NA |  | NA |  | NA |  | NA |  |
| NA |  | NA |  | NA |  | NA |  | NA |  | NA |  | NA |  | NA |  |
| NA |  | NA |  | NA |  | NA |  | NA |  | NA |  | NA |  | NA |  |
| NA |  | NA |  | NA |  | NA |  | NA |  | NA |  | NA |  | NA |  |
| NA |  | NA |  | NA |  | NA |  | NA |  | NA |  | NA |  | NA |  |
|  | -80.09 |  | -62.39 | NA |  | NA |  | NA |  | NA |  | NA |  | NA |  |
| NA |  | NA |  | NA |  | NA |  | NA |  | NA |  | NA |  | NA |  |
| NA |  | NA |  | NA |  | NA |  |  | -12.51 | NA |  | NA |  | NA |  |
| NA |  | NA |  | NA |  | NA |  |  | -7.57 | NA |  | NA |  | NA |  |
| NA |  | NA |  | NA |  | NA |  |  | -15.34 | NA |  | NA |  | NA |  |
| NA |  | NA |  | NA |  | NA |  | NA |  | NA |  | NA |  | NA |  |
|  | -61.48 |  | -45.48 | NA |  | NA |  | NA |  | NA |  | NA |  | NA |  |
| NA |  | NA |  | NA |  | NA |  | NA |  | NA |  | NA |  | NA |  |
| NA |  | NA |  | NA |  | NA |  | NA |  | NA |  | NA |  | NA |  |
| NA |  | NA |  | NA |  | NA |  | NA |  | NA |  | NA |  | NA |  |
| NA |  | NA |  | NA |  | NA |  | NA |  | NA |  | NA |  | NA |  |
|  | -32.86 |  | -36.73 | NA |  | NA |  | NA |  | NA |  | NA |  | NA |  |
| NA |  | NA |  | NA |  | NA |  | NA |  | NA |  | NA |  | NA |  |
| NA |  | NA |  | NA |  | NA |  | NA |  | NA |  | NA |  | NA |  |
| NA |  | NA |  | NA |  | NA |  | NA |  | NA |  | NA |  | NA |  |
| NA |  | NA |  | NA |  | NA |  | NA |  | NA |  | NA |  | NA |  |
| NA |  | NA |  | NA |  | NA |  | NA |  | NA |  | NA |  | NA |  |
| NA |  | NA |  | NA |  | NA |  | NA |  | NA |  | NA |  | NA |  |
| NA |  | NA |  | NA |  | NA |  | NA |  | NA |  | NA |  | NA |  |
| NA |  | NA |  | NA |  | NA |  | NA |  | NA |  | NA |  | NA |  |

| NA |  | NA |  | NA |  | NA |  |  | -9.92 | NA |  | NA |  | NA |  |
| --- | --- | --- | --- | --- | --- | --- | --- | --- | --- | --- | --- | --- | --- | --- | --- |
| -116.76 | |  | -94.49 | NA |  | NA |  | NA |  | NA |  | NA |  | NA |  |
| NA |  | NA |  | NA |  | NA |  | NA |  | NA |  | NA |  | NA |  |
| NA |  | NA |  | NA |  | NA |  | NA |  | NA |  | NA |  | NA |  |
| NA |  | NA |  | NA |  | NA |  | NA |  | NA |  | NA |  | NA |  |
|  | -5.31 |  | -6.88 | NA |  | NA |  | NA |  | NA |  | NA |  | NA |  |
| NA |  | NA |  | NA |  | NA |  | NA |  | NA |  | NA |  | NA |  |
| NA |  | NA |  | NA |  | NA |  | NA |  | NA |  | NA |  | NA |  |
| NA |  | NA |  | NA |  | NA |  |  | -5.97 | NA |  | NA |  | NA |  |
| NA |  | NA |  | NA |  | NA |  | NA |  | NA |  | NA |  | NA |  |
| -1173.57 | | -1350.66 | | -603.84 | | -151.01 | | NA |  | -312.74 | | -130.07 | | -137.76 | |
|  | -10.8 |  | -15.01 |  | -5.9 |  | -5.85 | NA |  | -275.69 | | -243.43 | | -320.46 | |
|  | -16.25 |  | -6.09 | NA |  | NA |  | NA |  |  | -20.19 | NA |  | NA |  |
| -395.32 | | -365.64 | |  | -227.4 |  | -65.16 | NA |  | -281.21 | | -131.35 | |  | -136.1 |
| -227.89 | | -189.77 | | -193.81 | | -182.79 | | NA |  | -496.77 | | -379.67 | | -450.06 | |
| -139.98 | | -129.75 | | -130.54 | | -131.13 | | NA |  | -381.68 | | -283.37 | | -306.04 | |
| NA |  | NA |  |  | -43.97 |  | -37.67 | NA |  | -164.01 | | -190.51 | | -274.49 | |
|  | -49.2 |  | -35.33 | NA |  | NA |  | NA |  |  | -83.57 |  | -27.98 |  | -30.63 |
| -421.51 | | -353.55 | | -422.32 | | -191.92 | | NA |  | -595.01 | | -366.52 | | -420.92 | |
|  | -59.83 |  | -43.29 |  | -46.78 |  | -30.88 | NA |  | -198.54 | | -130.53 | | -139.07 | |
|  | -17.59 |  | -31.98 | -283.92 | |  | -7.36 | NA |  | -445.98 | | -321.65 | | -535.71 | |
|  | -24.57 |  | -36.74 | -358.52 | |  | -17.66 | NA |  | -422.24 | | -314.03 | | -518.25 | |
|  | -67.14 |  | -93.49 | -448.27 | |  | -26.99 | NA |  | -647.75 | | -449.17 | | -731.25 | |
| NA |  |  | -7.51 | -161.61 | | NA |  | NA |  | -417.64 | | -313.52 | | -555.67 | |
|  | -28.43 |  | -42.82 |  | -8.78 |  | -33.29 | NA |  |  | -56.91 |  | -47.6 |  | -118 |
|  | -70.62 |  | -77.46 |  | -49.25 |  | -39.96 |  | -6.93 | -189.65 | | -206.37 | | -271.74 | |
|  | -29.15 |  | -37.08 |  | -72.52 |  | -93.2 | NA |  | -147.98 | | -167.37 | | -329.84 | |
|  | -25.25 |  | -36.13 |  | -63.24 |  | -59.85 | NA |  |  | -71.21 |  | -76.49 | -127.83 | |
|  | -25.25 |  | -36.13 |  | -63.24 |  | -59.85 | NA |  |  | -71.21 |  | -76.49 | -127.83 | |
|  | -22.56 |  | -29.66 |  | -19.51 |  | -62.96 |  | -14.43 |  | -61.14 |  | -48.99 |  | -95.19 |
| -154.33 | | -167.28 | |  | -51 |  | -61.82 |  | -28.23 | -137.73 | | -106.16 | | -137.07 | |
|  | -31.13 |  | -37.07 |  | -19.53 |  | -49.54 |  | -5.23 |  | -62.3 |  | -49.68 | -133.98 | |
|  | -31.13 |  | -37.07 |  | -19.53 |  | -49.54 |  | -5.23 |  | -62.3 |  | -49.68 | -133.98 | |
|  | -31.13 |  | -37.07 |  | -19.53 |  | -49.54 |  | -5.23 |  | -62.3 |  | -49.68 | -133.98 | |
| -197.36 | | -223.56 | |  | -68.44 |  | -92.79 |  | -49.68 |  | -95.54 |  | -68.65 | -102.85 | |
| -312.97 | | -343.84 | | -213.74 | | -193.43 | |  | -93.38 | -183.78 | | -156.42 | | -218.19 | |
| NA |  | NA |  | NA |  |  | -29.89 | NA |  |  | -38.09 |  | -30.59 | -115.99 | |
| NA |  | NA |  | NA |  | NA |  |  | -10.97 | NA |  | NA |  | NA |  |
|  | -8.18 |  | -8.33 | NA |  | NA |  |  | -13.7 | -101.02 | |  | -27.01 |  | -10.32 |

| NA |  | NA |  | NA |  | NA |  |  | -16.74 | NA |  | NA |  | NA |  |
| --- | --- | --- | --- | --- | --- | --- | --- | --- | --- | --- | --- | --- | --- | --- | --- |
| NA |  | NA |  | NA |  | NA |  | NA |  | NA |  | NA |  | NA |  |
| NA |  | NA |  | NA |  | NA |  | NA |  | NA |  | NA |  | NA |  |
| NA |  | NA |  | NA |  | NA |  | NA |  | NA |  | NA |  | NA |  |
| -351.05 | | -285.72 | |  | -449.8 | -275.48 | |  | -22.01 | -436.49 | | -412.23 | | -499.67 | |
| -245.89 | | -276.93 | | -317.08 | | -195.79 | |  | -17.65 | -470.52 | | -396.69 | | -476.06 | |
|  | -90.9 |  | -97.99 | -155.85 | |  | -88.37 |  | -16.42 | -300.36 | | -290.91 | | -408.85 | |
| -436.75 | | -458.76 | | -437.06 | | -316.72 | |  | -15.19 | -517.95 | | -449.12 | | -473.14 | |
|  | -42.02 |  | -32.64 | -142.85 | |  | -58.83 | NA |  |  | -142.1 | -137.17 | | -198.29 | |
|  | -97.27 |  | -78.7 | -197.41 | |  | -96.9 |  | -12.58 | -186.52 | | -181.83 | | -228.18 | |
| -232.47 | | -220.02 | | -209.71 | | -265.56 | | NA |  | -582.06 | | -649.88 | | -849.34 | |
| -242.46 | | -229.92 | | -326.79 | | -311.21 | |  | -94.04 |  | -56.57 |  | -53.86 |  | -40.74 |
| -358.45 | | -343.77 | | -499.29 | | -344.24 | |  | -58.5 |  | -85.76 |  | -47.75 |  | -39.13 |
|  | -59.75 |  | -51.31 |  | -33.98 |  | -57.12 | NA |  | -177.14 | | -179.55 | | -247.29 | |
| NA |  | NA |  | NA |  | NA |  |  | -19.83 | NA |  | NA |  | NA |  |
| NA |  | NA |  | NA |  | NA |  |  | -5.96 | NA |  | NA |  | NA |  |
| -128.47 | | -110.14 | |  | -93.12 | -138.22 | | NA |  | -570.78 | | -601.24 | |  | -841.4 |
| NA |  | NA |  | NA |  | NA |  | NA |  | NA |  | NA |  | NA |  |
| -381.87 | | -311.79 | | -262.19 | |  | -160.1 | NA |  | -386.12 | | -371.16 | | -394.05 | |
| NA |  | NA |  | NA |  | NA |  |  | -11.12 | NA |  | NA |  | NA |  |
| NA |  | NA |  | NA |  | NA |  |  | -11.12 | NA |  | NA |  | NA |  |
| -159.39 | | -144.45 | | -137.91 | | -158.59 | |  | -12.54 | -210.17 | | -181.95 | | -234.64 | |
| -233.95 | | -234.27 | | -213.18 | | -209.14 | | NA |  | -648.24 | |  | -717.2 | -962.68 | |
| -233.95 | | -234.27 | | -213.18 | | -209.14 | | NA |  | -648.24 | |  | -717.2 | -962.68 | |
| -233.95 | | -234.27 | | -213.18 | | -209.14 | | NA |  | -648.24 | |  | -717.2 | -962.68 | |
| -233.95 | | -234.27 | | -213.18 | | -209.14 | | NA |  | -648.24 | |  | -717.2 | -962.68 | |
| -417.31 | | -517.49 | | -312.65 | | -166.81 | |  | -8.39 | -320.26 | | -238.21 | | -314.32 | |
| -417.31 | | -517.49 | | -312.65 | | -166.81 | |  | -8.39 | -320.26 | | -238.21 | | -314.32 | |
| -417.31 | | -517.49 | | -312.65 | | -166.81 | |  | -8.39 | -320.26 | | -238.21 | | -314.32 | |
| -417.31 | | -517.49 | | -312.65 | | -166.81 | |  | -8.39 | -320.26 | | -238.21 | | -314.32 | |
| -199.61 | | -235.99 | | -115.27 | |  | -95.22 |  | -16.81 | -192.18 | | -136.96 | | -155.68 | |
| -199.61 | | -235.99 | | -115.27 | |  | -95.22 |  | -16.81 | -192.18 | | -136.96 | | -155.68 | |
| -175.33 | | -164.57 | |  | -71.6 |  | -76.76 |  | -8.42 | -112.83 | |  | -87.76 |  | -91.84 |
| -175.33 | | -164.57 | |  | -71.6 |  | -76.76 |  | -8.42 | -112.83 | |  | -87.76 |  | -91.84 |
| -401.21 | | -426.51 | | -373.79 | | -498.79 | | NA |  | -1043.53 | | -1134.14 | | -1467.44 | |
| -441.96 | | -323.76 | | -379.93 | | -240.14 | |  | -8.98 | -457.58 | | -428.26 | |  | -513.8 |
| -441.96 | | -323.76 | | -379.93 | | -240.14 | |  | -8.98 | -457.58 | | -428.26 | |  | -513.8 |
| -417.42 | | -336.12 | | -257.52 | | -168.83 | | NA |  | -327.55 | |  | -303 | -323.18 | |
| -417.42 | | -336.12 | | -257.52 | | -168.83 | | NA |  | -327.55 | |  | -303 | -323.18 | |

| -420.31 | | -286.65 | | -304.68 | | -192.89 | |  | -19.01 | -426.95 | | -417.67 | | -467.91 | |
| --- | --- | --- | --- | --- | --- | --- | --- | --- | --- | --- | --- | --- | --- | --- | --- |
| -612.91 | | -483.82 | | -513.74 | | -325.83 | |  | -30.26 | -611.93 | | -549.33 | | -652.46 | |
| -324.81 | | -263.84 | | -259.37 | |  | -152.9 | NA |  |  | -391.6 | -349.09 | | -463.98 | |
| -100.23 | |  | -95.24 |  | -66.38 |  | -33.38 | NA |  |  | -85.51 |  | -87.24 | -128.33 | |
| -251.84 | | -324.19 | | -388.55 | |  | -65 | NA |  | -486.47 | | -344.43 | | -455.55 | |
|  | -6.53 |  | -18.84 |  | -221.8 | NA |  | NA |  | -370.15 | | -286.75 | | -465.56 | |
| -287.16 | | -334.14 | | -457.04 | | -101.17 | |  | -5.64 | -484.65 | |  | -382.6 | -526.38 | |
| -320.47 | | -361.45 | | -148.82 | |  | -38.73 | NA |  | -190.97 | | -196.56 | | -277.93 | |
| -398.01 | |  | -446.9 | -192.69 | |  | -57.7 | NA |  | -231.03 | | -220.19 | | -328.94 | |
| NA |  | NA |  | NA |  | NA |  | NA |  |  | -23.84 |  | -39.59 |  | -68.22 |
| -377.94 | | -460.64 | | -185.48 | |  | -42.41 | NA |  | -245.32 | | -227.98 | | -335.75 | |
|  | -211.1 | -228.47 | |  | -82.73 |  | -27.99 | NA |  | -146.83 | | -174.39 | | -202.24 | |
| NA |  | NA |  |  | -6.96 | NA |  | NA |  |  | -30.67 |  | -25.1 |  | -68.85 |
| -153.75 | | -170.47 | | -148.14 | |  | -42.01 |  | -6.54 | -193.58 | | -155.98 | | -229.52 | |
| NA |  | NA |  | NA |  | NA |  | NA |  |  | -11.12 |  | -8.93 |  | -46.98 |
| NA |  | NA |  | NA |  | NA |  | NA |  | NA |  | NA |  | NA |  |
| NA |  | NA |  | NA |  | NA |  | NA |  | NA |  | NA |  | NA |  |
|  | -20.37 |  | -20.15 | NA |  | NA |  | NA |  |  | -24.57 |  | -30.66 |  | -39.43 |
| NA |  | NA |  | NA |  | NA |  | NA |  | NA |  | NA |  | NA |  |
| NA |  | NA |  | NA |  | NA |  | NA |  | NA |  | NA |  | NA |  |
| NA |  | NA |  | NA |  | NA |  | NA |  | NA |  | NA |  | NA |  |
| NA |  | NA |  | NA |  | NA |  | NA |  | NA |  | NA |  |  | -13.41 |
| NA |  | NA |  | NA |  | NA |  | NA |  |  | -26.87 |  | -21.8 |  | -35.3 |
|  | -31.75 |  | -39.64 | NA |  | NA |  | NA |  |  | -19.39 |  | -11.19 |  | -28.53 |
|  | -166.2 | -202.18 | |  | -66.88 |  | -26.35 | NA |  |  | -69.35 |  | -43.77 |  | -45.29 |
| NA |  | NA |  | NA |  | NA |  | NA |  | NA |  | NA |  | NA |  |
| NA |  | NA |  | NA |  | NA |  |  | -6.14 | NA |  | NA |  | NA |  |
| NA |  | NA |  | NA |  | NA |  |  | -9.9 | NA |  | NA |  | NA |  |
|  | -401.2 | -409.55 | | -288.59 | | -377.34 | | NA |  | -971.53 | | -1050.04 | | -1328.35 | |
| -318.18 | | -367.77 | | -141.49 | |  | -79.73 |  | -53.6 | -152.35 | | -102.65 | |  | -94.1 |
| -318.18 | | -367.77 | | -141.49 | |  | -79.73 |  | -53.6 | -152.35 | | -102.65 | |  | -94.1 |
| NA |  | NA |  | NA |  | NA |  |  | -13.14 | NA |  | NA |  | NA |  |
| NA |  | NA |  |  | -15.07 |  | -5.24 |  | -18.6 |  | -58.34 |  | -74.75 | -122.75 | |
| NA |  | NA |  | NA |  | NA |  |  | -17.5 |  | -28.69 |  | -31.23 |  | -60.28 |
|  | -8.58 |  | -7.4 |  | -11.95 | NA |  |  | -32.74 |  | -57.4 |  | -46.52 |  | -68.4 |
| -744.57 | |  | -753.6 | -790.51 | | -464.51 | |  | -11.25 | -443.51 | |  | -325 | -436.56 | |
| NA |  | NA |  |  | -18.24 | NA |  |  | -14.98 |  | -71.43 |  | -69.36 | -107.07 | |
| NA |  | NA |  | NA |  | NA |  |  | -27.41 | NA |  |  | -6.54 |  | -37.29 |
| NA |  | NA |  |  | -20.07 | NA |  |  | -33.62 |  | -77.43 |  | -73.43 | -116.22 | |

-7.26	-16.69	-8.49 NA	-33.49	-60.98	-36.98	-44.18

NA	NA	NA	NA	NA	NA	NA	NA NA	NA	NA	NA	NA	NA	NA	NA

NA	NA	NA	NA	NA	-23.69	-24.43	-34.49

NA	NA	-9.22 NA	NA	-28.79	-60	-100.51

-252.29	-295.37	-315.41	-191.82	-16.83	-480.62	-380.37	-434.74

NA	NA	NA	NA	NA	NA	NA	NA NA	NA	NA	NA	NA	NA	NA	NA NA	NA	NA	NA	NA	NA	NA	NA NA	NA	NA	NA	NA	NA	NA	NA

-17.75	-28.59	-63.93	-46.01	-21.66	-48.89	-56.52	-64.3

NA	NA	-39.96	-19.59	-23.56	-90.76	-89.91	-165.88

-125.86	-166.53	-225.95	-123.99	-15.67	-258.44	-226.73	-282.43

NA	NA	NA	NA	-6.39 NA	NA	NA

NA	NA	-48.52	-41.64	-33.15	-43.58	-50.39	-102.81

-7.08	-13.69	-8.27	-9.74	-33.22	-24.24	-25.38	-28.96

-9.41	-22.29	-36.65 NA	-16.35	-124.57	-92.39	-141.82

NA	NA	-39.22	-25.42	-25.94	-63.2	-89.2	-167.63

NA	NA	NA	NA	-11.96 NA	NA	NA

-155.94	-145.88	-167.55	-115.86	-56.27	-174.26	-197.34	-296.6

-287.09	-318.31	-253.32	-174.78	-57.98	-284.15	-279.83	-360.79

-49.38	-73.24	-40.68 NA	-24.09	-108.59	-88.92	-100.69

-35.97	-50.53	-46.27 NA	-55.78	-87.54	-65.42	-73.46

-485.17	-513.98	-615.86	-575.51	-46.74	-68.02	-29.85	-29.42

-205.14	-191.18	-143.17	-80.44	-8.18	-290.41	-238.53	-274.21

NA	-6.54	-20.05	-9.34	-15.62	-100.15	-88.46	-114.23

-174.26	-210.99	-156.7	-59.13	-7.93	-255.14	-201.47	-278.61

-38.17	-46.63	-45.42	-8.21	-23.52	-106.74	-106.69	-154.38

-228.47	-240.29	-264.01	-132.11	-16.14	-323.43	-283.01	-371.95

-23.7	-21.53	-56.46	-48.2	-8.44	-124.27	-119.28	-180.29

-61.6	-73.67	-46.96	-32.52	-15.44	-83.91	-56.99	-68.72

-64.29	-70.42	-69.37	-62.66	-23.56	-118.62	-103.97	-171.47

-107.57	-128.51	-91.76	-68.85	-10.98	-167.7	-130.67	-151.54

-127.35	-124.13	-111.48	-74.31	-30.87	-198.45	-171.79	-265.98

-53.67	-53.5	-34.79	-27.2	-22.73	-74.34	-53.38	-95

NA	-7.43 NA	-8.62	-17.19	-21.15	-13.54	-40.45

NA	NA	NA	NA	-20.38 NA	NA	-15.18

NA	NA	NA	NA	-20.38 NA	NA	-15.18

NA	NA	NA	NA	-20.38 NA	NA	-15.18

| -1537.67 | | -1469.83 | | -673.83 | | -1296.92 | | NA |  | -1337.42 | | -1049.82 | | -1455.92 | |
| --- | --- | --- | --- | --- | --- | --- | --- | --- | --- | --- | --- | --- | --- | --- | --- |
| -1960.96 | | -1924.32 | | -925.22 | | -1564.57 | | NA |  | -1529.06 | | -1152.75 | | -1522.67 | |
| -1112.94 | | -1015.98 | |  | -475.5 | -1001.01 | | NA |  | -728.53 | | -579.46 | | -787.34 | |
|  | -15.44 |  | -26.06 |  | -28.09 | NA |  |  | -36.56 |  | -67.7 |  | -54.77 |  | -70.19 |
| NA |  | NA |  | NA |  | NA |  |  | -18.58 |  | -14.55 |  | -39.02 |  | -84.34 |
| NA |  | NA |  | NA |  | NA |  |  | -24.97 |  | -10.64 |  | -9.23 |  | -10.2 |
| NA |  | NA |  | NA |  | NA |  |  | -38.33 |  | -12.38 | NA |  |  | -12.9 |
|  | -12.84 |  | -15.64 |  | -9.94 | NA |  | NA |  |  | -52.62 |  | -51.51 |  | -46.44 |
| -131.28 | | -160.37 | | -125.48 | |  | -67.27 |  | -21.39 | -233.28 | |  | -223 | -311.48 | |
| -109.28 | | -112.75 | |  | -95.22 | -113.66 | |  | -5.63 | -243.76 | | -234.81 | | -314.75 | |
|  | -125.5 | -146.49 | |  | -128.7 |  | -75.87 |  | -9.31 | -262.07 | | -255.39 | | -329.99 | |
|  | -125.5 | -146.49 | |  | -128.7 |  | -75.87 |  | -9.31 | -262.07 | | -255.39 | | -329.99 | |
| -134.66 | | -171.84 | | -161.85 | | -112.68 | |  | -13.62 | -257.88 | | -259.05 | | -324.12 | |
|  | -8 |  | -15.76 |  | -295.6 |  | -12.55 | NA |  | -527.83 | | -366.32 | | -660.82 | |
|  | -8 |  | -15.76 |  | -295.6 |  | -12.55 | NA |  | -527.83 | | -366.32 | | -660.82 | |
|  | -6.28 |  | -8.28 | NA |  | NA |  |  | -30.4 |  | -7.58 | NA |  | NA |  |
| NA |  | NA |  | NA |  | NA |  |  | -36.59 | NA |  | NA |  | NA |  |
| NA |  | NA |  | NA |  | NA |  |  | -61.72 | NA |  | NA |  | NA |  |
|  | -89.25 | -101.89 | |  | -27.61 |  | -31.81 |  | -27.84 |  | -81.68 |  | -45.1 |  | -77.14 |
| -2317.07 | | -2538.2 | | -1672.78 | | -1038.59 | | NA |  | -1514.73 | | -1012.37 | | -1234.94 | |
| -4018.56 | | -4144.96 | | -2804.68 | | -631.11 | | NA |  | -794.62 | | -362.61 | | -401.29 | |
| -1524.98 | | -1669.7 | | -933.61 | | -107.38 | | NA |  | -332.94 | | -118.26 | | -146.57 | |
| -2742.9 | | -3076.63 | | -1921.53 | | -610.25 | | NA |  | -798.92 | | -431.91 | | -483.81 | |
| -5340.78 | | -5713.45 | | -4254.33 | | -883.46 | | NA |  | -1094.09 | | -541.87 | | -578.14 | |
| -3865.58 | | -4146.11 | | -2511.85 | | -529.65 | | NA |  | -822.33 | | -398.95 | | -448.51 | |
| -186.25 | | -216.33 | | -128.77 | |  | -20.35 | NA |  | -102.88 | |  | -51.18 |  | -39.28 |
|  | -405.9 | -338.37 | |  | -275.9 |  | -73.71 | NA |  | -321.17 | | -148.23 | | -155.66 | |
| -192.87 | | -243.52 | |  | -128.3 |  | -37.68 | NA |  | -199.01 | | -115.57 | | -127.14 | |
|  | -45.08 |  | -35.96 |  | -21.58 |  | -9.47 | NA |  |  | -96.92 |  | -85.36 | -126.58 | |
| -495.63 | | -384.76 | | -383.12 | | -223.32 | |  | -28.15 | -568.74 | | -513.28 | | -585.28 | |
|  | -432.5 | -397.06 | | -321.94 | |  | -187.7 | NA |  | -265.34 | | -248.65 | | -266.94 | |
|  | -33.84 | NA |  | NA |  |  | -22.35 | NA |  | -175.15 | | -179.49 | | -254.94 | |
| NA |  | NA |  | NA |  | NA |  | NA |  |  | -43.82 |  | -5.71 | NA |  |
| NA |  | NA |  | NA |  | NA |  |  | -8.39 | NA |  | NA |  | NA |  |
| NA |  | NA |  | NA |  | NA |  | NA |  | NA |  | NA |  | NA |  |
|  | -29.84 |  | -24.89 |  | -140.2 |  | -93.5 |  | -31.5 | -150.78 | | -158.54 | | -300.83 | |
| -168.42 | | -184.16 | | -296.75 | | -146.78 | |  | -45.63 | -337.61 | | -357.85 | | -524.05 | |
|  | -151.1 | -141.95 | | -274.94 | | -194.47 | |  | -62.69 | -191.21 | | -194.31 | | -313.76 | |
| NA |  | NA |  | NA |  |  | -7.46 | NA |  | NA |  | NA |  |  | -9.63 |

| -150.52 | | -152.07 | | -263.96 | | -160.99 | |  | -46.89 | -266.04 | | -271.62 | | -468.76 | |
| --- | --- | --- | --- | --- | --- | --- | --- | --- | --- | --- | --- | --- | --- | --- | --- |
| -512.37 | | -503.99 | | -551.47 | | -363.03 | |  | -35.02 | -570.16 | |  | -518 | -638.84 | |
| -144.46 | | -146.88 | |  | -178.2 |  | -176.4 | NA |  | -535.34 | | -562.36 | | -779.23 | |
|  | -136.2 | -130.19 | | -169.64 | | -161.18 | | NA |  | -489.22 | | -507.44 | | -765.57 | |
|  | -67.63 |  | -72.38 | -120.46 | |  | -99.71 | NA |  | -336.97 | |  | -333 |  | -537.2 |
|  | -57.82 |  | -51.74 |  | -50.26 |  | -53.92 | NA |  |  | -81.61 |  | -96.41 | -127.13 | |
|  | -11.83 |  | -12.88 |  | -24.32 | NA |  | NA |  |  | -54.63 |  | -51.99 | -122.01 | |
| -158.19 | | -138.38 | | -179.52 | | -180.44 | | NA |  | -498.29 | |  | -517.9 | -759.48 | |
|  | -79.3 |  | -65.47 | -131.51 | | -112.19 | | NA |  |  | -386.8 | -447.11 | | -669.15 | |
| NA |  | NA |  | NA |  | NA |  |  | -9.92 | NA |  | NA |  | NA |  |
| NA |  | NA |  | NA |  | NA |  |  | -11.21 | NA |  | NA |  | NA |  |
| NA |  | NA |  | NA |  | NA |  | NA |  | NA |  | NA |  | NA |  |
| -661.65 | | -710.44 | | -648.68 | | -694.75 | |  | -57.64 |  | -185.7 | -139.86 | | -141.25 | |
| -232.64 | | -248.13 | | -299.54 | | -265.13 | |  | -22.39 | -490.99 | | -485.82 | |  | -604.6 |
|  | -36.25 |  | -15.58 |  | -21.9 |  | -14.42 | NA |  |  | -69.48 |  | -73.46 | -111.82 | |
| -144.54 | | -157.76 | | -144.37 | | -140.08 | |  | -13.61 | -229.73 | | -206.91 | | -304.16 | |
|  | -32.08 |  | -9.38 |  | -14.94 |  | -9.47 | NA |  |  | -57.72 |  | -60.85 |  | -98.1 |
|  | -18.59 |  | -14.41 |  | -17.74 | -131.39 | | NA |  | -152.86 | | -169.96 | | -248.06 | |
|  | -71.91 |  | -77.89 | -131.91 | |  | -94.95 |  | -23.69 | -175.83 | | -194.44 | | -280.66 | |
| NA |  | NA |  | NA |  | NA |  | NA |  | NA |  | NA |  | NA |  |
| -403.34 | | -401.08 | | -529.03 | | -482.75 | |  | -66.68 | -461.62 | |  | -400.9 | -500.15 | |
|  | -15.04 |  | -15.86 |  | -16.86 | NA |  | NA |  | -117.73 | | -103.78 | | -151.41 | |
| NA |  | NA |  |  | -14.69 |  | -32.34 |  | -14.8 |  | -58.52 |  | -87.28 | -220.36 | |
| NA |  | NA |  |  | -26.46 |  | -43.18 |  | -15.54 |  | -80.03 | -143.99 | | -314.83 | |
| -163.25 | | -143.17 | | -207.31 | | -206.14 | |  | -51.63 | -164.36 | | -193.39 | | -289.25 | |
| -259.28 | | -316.76 | | -114.52 | | -110.42 | |  | -38.72 | -117.21 | | -103.94 | | -135.87 | |
| -230.97 | | -219.13 | | -215.09 | |  | -196.9 |  | -59.3 | -124.59 | | -119.76 | | -182.76 | |
| -155.95 | | -121.04 | | -231.66 | | -181.74 | |  | -51.76 | -140.99 | | -154.72 | | -262.94 | |
|  | -86.59 |  | -91.79 |  | -41.29 |  | -10.47 |  | -11.91 | -104.23 | | -103.72 | | -131.48 | |
| -211.66 | | -199.38 | | -234.31 | | -253.13 | |  | -17.99 | -231.98 | | -216.51 | | -260.39 | |
| -308.09 | | -359.43 | | -134.13 | |  | -85.93 |  | -21.62 | -306.09 | | -233.65 | | -337.74 | |
| -274.36 | | -272.25 | | -368.54 | | -330.63 | |  | -24.11 | -432.44 | | -394.51 | | -478.26 | |
| -115.33 | | -151.48 | | -105.76 | |  | -59.13 |  | -9.71 | -219.73 | | -188.64 | | -247.69 | |
|  | -152.3 | -164.56 | |  | -73.05 |  | -42.85 | NA |  | -235.86 | | -201.23 | | -304.24 | |
|  | -36.37 |  | -44.11 | -115.06 | | -130.14 | |  | -23.1 |  | -11.79 |  | -6.79 |  | -6.58 |
| -349.89 | | -397.82 | | -159.89 | |  | -36.6 | NA |  | -127.81 | |  | -52.83 |  | -55.95 |
| -689.33 | | -787.55 | |  | -387.5 | -117.77 | | NA |  | -254.39 | | -118.97 | | -127.82 | |
| -115.14 | | -104.18 | | -111.29 | |  | -75.37 |  | -27.25 | -200.38 | | -179.46 | | -217.91 | |
| -189.21 | | -214.53 | | -342.15 | | -333.66 | |  | -30.6 | -276.25 | | -181.54 | | -320.36 | |

| -146.46 | | -188.47 | | -165.81 | |  | -94.93 |  | -24.2 | -184.97 | | -144.41 | |  | -213.3 |
| --- | --- | --- | --- | --- | --- | --- | --- | --- | --- | --- | --- | --- | --- | --- | --- |
| -401.22 | | -410.16 | | -264.34 | | -162.07 | |  | -35.03 | -250.84 | | -207.86 | | -274.65 | |
| -247.29 | | -277.98 | | -229.88 | | -171.16 | |  | -26.89 | -434.83 | | -383.88 | | -514.25 | |
|  | -73.56 |  | -52.01 |  | -55.41 |  | -83.79 |  | -8.35 | -109.12 | | -139.86 | | -192.11 | |
| -583.35 | | -712.99 | | -608.09 | |  | -293.7 |  | -17.08 | -257.08 | |  | -73.75 | -124.62 | |
| NA |  | NA |  | NA |  | NA |  | NA |  |  | -212.1 | -201.58 | | -253.03 | |
| -435.55 | |  | -473.5 |  | -399.6 | -228.13 | |  | -20.55 | -428.65 | | -368.99 | | -418.26 | |
| -101.96 | |  | -87.93 | NA |  | NA |  |  | -11.97 | -195.25 | | -190.82 | |  | -269.7 |
|  | -169.1 | -183.02 | | -210.55 | | -159.28 | |  | -25.99 | -262.79 | | -285.82 | | -376.26 | |
| -134.52 | | -135.91 | |  | -92.92 |  | -88.45 | NA |  | -132.44 | | -107.83 | | -127.04 | |
| -193.16 | | -232.94 | | -132.13 | |  | -67.05 |  | -22.56 | -249.12 | | -235.38 | | -270.48 | |
| NA |  | NA |  | NA |  | NA |  | NA |  | NA |  | NA |  | NA |  |
| -170.99 | | -170.83 | |  | -151.6 |  | -68.11 |  | -10.64 |  | -214.1 |  | -143.3 |  | -230.3 |
| -112.66 | | -113.49 | |  | -54.92 |  | -58.91 |  | -27.96 |  | -45.56 |  | -43.21 |  | -58.11 |
| -222.55 | | -200.22 | | -119.36 | |  | -197.9 |  | -28.06 | -169.03 | | -169.35 | | -259.74 | |
| -623.85 | | -612.88 | | -652.69 | | -481.74 | |  | -61.72 | -664.02 | | -593.94 | | -689.99 | |
|  | -21.34 |  | -23.72 |  | -41.11 |  | -65.12 |  | -39.33 | -116.45 | | -117.61 | | -203.64 | |
| -222.06 | | -192.15 | | -299.69 | | -323.78 | |  | -43.4 | -377.04 | | -338.12 | | -432.74 | |
| NA |  | NA |  | NA |  | NA |  | NA |  |  | -83.32 |  | -94.97 | -128.13 | |
|  | -98.15 | -105.41 | |  | -75.77 |  | -42.71 | NA |  | -154.45 | | -130.39 | | -168.11 | |
| -285.55 | | -294.12 | | -123.75 | | -124.48 | |  | -25.13 | -360.62 | | -293.02 | | -429.19 | |
| -110.64 | | -108.59 | |  | -26.63 | NA |  | NA |  |  | -39.86 |  | -7.94 |  | -7.54 |
| -138.12 | |  | -126.6 | -195.31 | |  | -91.11 |  | -32.88 | -253.26 | | -252.22 | | -383.52 | |
| -509.69 | |  | -579.1 | -199.98 | |  | -46.29 | NA |  | -104.83 | |  | -51.57 |  | -39.36 |
| -145.69 | | -154.34 | | -157.92 | | NA |  |  | -16.49 | -224.93 | | -213.45 | | -303.45 | |
| -303.56 | | -303.53 | | -176.92 | |  | -43.46 | NA |  | -149.69 | |  | -69.18 |  | -76.75 |
| -162.42 | | -175.33 | | -137.88 | |  | -64.1 |  | -18.53 | -148.76 | | -128.86 | | -176.08 | |
| -276.19 | | -281.21 | |  | -84.93 |  | -58.73 |  | -53.98 | -133.77 | |  | -83.86 |  | -127.7 |
|  | -71.57 |  | -68.27 | -111.05 | |  | -96.71 | NA |  |  | -186 | -208.74 | | -236.38 | |
|  | -36.88 |  | -49.14 |  | -27.99 |  | -22.12 |  | -9.78 |  | -91.72 | -102.31 | | -155.34 | |
|  | -378.6 | -426.55 | | -213.35 | |  | -39.78 | NA |  | -137.54 | |  | -78.41 |  | -91.79 |
|  | -298.4 | -293.54 | | -323.78 | | -368.61 | |  | -58.28 | -171.11 | | -150.34 | | -221.94 | |
| -170.89 | | -188.37 | | -143.26 | |  | -51.25 |  | -34.97 | -185.18 | | -170.38 | |  | -261 |
| -241.92 | | -246.66 | |  | -96.25 | -189.54 | | NA |  | -169.72 | | -110.24 | | -155.65 | |
| NA |  | NA |  | NA |  | NA |  |  | -18.87 | NA |  | NA |  |  | -13.08 |
|  | -84.28 |  | -77.71 | -107.62 | |  | -78.48 |  | -17.34 | -207.46 | | -211.63 | | -290.63 | |
| -145.85 | | -146.35 | | -128.44 | |  | -96.32 |  | -7.05 | -163.24 | | -123.06 | | -149.77 | |
| -269.25 | | -313.39 | | -101.46 | |  | -52.94 |  | -31.14 | -206.38 | | -152.01 | | -163.51 | |

endothelia endothelia eosinophil erythrobla fetal_intes germinal_cgranulocyt hapatocyt

-113.9	-55.97	-140.29	-67.83	-25.28	-153.52	-223.86	-136.85

NA	NA	NA	NA	NA	NA	NA	NA

-78.07	-45.44	-303.93	-34.84	-197.2	-383.25	-230.1	-727.5

-278.64	-117.31	-91.92	-119.36	-43.91	-543.51	-140.45	-399.97

-26.12	-6.76	-7.79	-13.17	-15.76	-274.93	-28.38	-318.1

-379.55	-168.7	-680.82	-192.47	-373.68	-1043.58	-697.19	-610.9

-133.34	-59.94	-237.88	-59.64	-108.76	-559.88	-207.28	-833.09

-59.95	-30.66	-17.65	-17.98 NA	-170.62	-39.43	-227.06

-59.95	-30.66	-17.65	-17.98 NA	-170.62	-39.43	-227.06

-43.02 NA	-166.33 NA	-65.51	-21.23	-8.65	-185.57

-284.5	-96.95	-465.03	-99.44	-173.02	-479.13	-495.86	-405.11

-58.05	-28.45	-124.76	-36.27	-31.3	-452.45	-138.53	-292.56

-247.51	-44.42	-132.16	-16.46	-33.83	-440.84	-117.39	-28.38

-364.22	-80.61	-184.58	-36.01	-148.6	-494.12	-141.69	-160.26

NA	NA	NA	NA	-28.71 NA	NA	-267.31

NA	NA	NA	NA	-36.39 NA	NA	NA

NA	NA	-72.31 NA	-140.53	-108.94	-5.39	-5.86

NA	NA	-72.31 NA	-140.53	-108.94	-5.39	-5.86

-146.34 NA	-3533.98	-43.83	-75.08	-887.2	-2032.68	-37.99

-86.28	-42.43	-202.45	-38.31	-174.3	-559.77	-171.66	-862.7

NA	NA	NA	NA	-44.13 NA	NA	NA

-47.81	-11.14	-2237.41	-179.29	-24.22	-1024.55	-1643.28	-78.69

NA	NA	NA	NA	-397.26 NA	NA	NA NA	NA	NA	NA	-1196.64 NA	NA	NA

NA	NA	-605 NA	-27.17 NA	-6.25	-155.36

NA	NA	-910.67 NA	NA	NA	NA	-727.01

NA	NA	-937.18 NA	NA	NA	NA	-739.15

NA	NA	-608.06 NA	NA	NA	NA	-395.73

-71.07	-29.55	-290.98	-39.22	-155.85	-350.63	-214.64	-927.83

-71.07	-29.55	-290.98	-39.22	-155.85	-350.63	-214.64	-927.83

-214.56	-82.5	-410.04	-114.85	-215.09	-590.5	-319.01	-1133.17

-183.82	-69.12	-485.96	-108.02	-269.59	-599.1	-364.76	-1241.74

-19.3 NA	-261.66 NA	-66.15	-11.75	-12.16	-204.48

NA	NA	-661.1 NA	-29.23 NA	NA	-139.86

NA	NA	NA	NA	NA	NA	NA	NA NA	NA	NA	NA	NA	NA	NA	NA NA	NA	NA	NA	-22.14 NA	NA	NA

-346.65	-125.4	-45.62	-158.05 NA	-72.75	-180.82	-12.59

| -73.24 | -21.44 | -15.75 | -12.34 | -29.62 | -784.55 | -59.95 | -149.81 |
| --- | --- | --- | --- | --- | --- | --- | --- |
| -73.24 | -21.44 | -15.75 | -12.34 | -29.62 | -784.55 | -59.95 | -149.81 |
| -73.24 | -21.44 | -15.75 | -12.34 | -29.62 | -784.55 | -59.95 | -149.81 |
| -58.64 | -24.29 | -75.31 | -24.57 NA |  | -74.61 | -190.3 | -77.08 |
| -101.2 | -45.89 | -37.11 | -48.14 NA |  | -83.53 | -142.63 | -49.27 |

-1060.71	-263.56	-5334.11	-302.25	-138.53	-1134.5	-4002.07	-81.16

-1524.37	-420.61	-7129.53	-442.27	-393.05	-1695.26	-4665.36	-172.2

-1882.82	-573.68	-9819.5	-660.88	-400.46	-2709.71	-6801.29	-210.16

-1610.73	-404.17	-7106.95	-401.1	-383.2	-1595.69	-5457.56	-190.85

-963.16	-319.14	-4544.31	-311.01	-360.69	-1217.35	-2977.62	-179.01

-1060.75	-359.37	-5610.71	-439.07	-223.25	-1780.37	-3861.55	-127.89

-1250.41	-378.22	-10074.7	-492.55	-464.38	-2166.76	-6093.98	-140.66

-2049.27	-659.97	-5243.53	-634.77	-414.71	-1778.11	-4043.33	-346.5

-1966.51	-641.1	-6840.69	-601.98	-303.26	-2145.27	-4823.97	-259.8

-2469.39	-844.85	-7970.07	-748.88	-433.32	-2508.66	-5755.86	-297.84

NA	NA	NA	NA	-43.2 NA	NA	NA

-35.13	-14.13	-43.05	-14.37	-9.35	-312.34	-47.36	-700.42

-2060.41	-609.34	-8298.73	-702.53	-367.19	-2444.29	-6098.41	-199.47

-2821.78	-877.05	-9205.91	-866.3	-343.33	-3040.67	-6770.63	-208.59

-408.17	-135.76	-554.73	-89.97	-39.12	-226.53	-493.41	-57.28

-2607.97	-711.74	-11913	-830.21	-377.63	-3363.17	-8730.13	-172.89

-36.07	-5.91 NA	NA	-333 NA	NA	-709.07

NA	NA	NA	NA	-33.06 NA	NA	NA

-2568.71	-768.44	-10608.4	-770.57	-346.3	-3165.76	-7485.78	-164.36

-205.98	-71.5	-317.18	-30.2	-21.79	-111.91	-295.23	-32.01

-1125.39	-266.26	-5606.1	-314.72	-149.49	-1206.33	-3902.68	-80.54

-2592.41	-794.22	-9851.66	-837.41	-386.81	-3003.45	-7137.86	-259.13

-1933.46	-523.79	-7625.22	-539.44	-327.69	-2161.63	-5327.08	-128.14

-922.92	-308.36	-2930.73	-305.77	-251.05	-1023.56	-2146.41	-228.7

-922.92	-308.36	-2930.73	-305.77	-251.05	-1023.56	-2146.41	-228.7

-2553.16	-760.41	-11161.6	-813.29	-365.62	-3190.48	-8003.96	-186.13

-1320.48	-437.57	-4372.04	-391.03	-115.52	-1437.01	-3470.15	-172.59

-377.79	-86.77	-3135.52	-214.62	-6.77	-857	-2253.46 NA

-688.04	-182.11	-3358.45	-229.71	-154.97	-1006.65	-2410.53	-107.56

NA	NA	NA	NA	-42.75 NA	NA	NA

-68.14	-32.85	-167.54	-38.58	-93.77	-486.72	-165.33	-350.51

-2192.15	-636.66	-10943.1	-630.99	-335.64	-2762.32	-7164.71	-91.69

-2727.81	-831.88	-11940.9	-897.64	-402.36	-3473.03	-8589.17	-196.42

-454.66	-163.25	-629.94	-114.19	-35.86	-267.68	-541.4	-70.91

| -317.12 | |  | -46.92 | -144.93 | |  | -8.15 | -148.88 | | -417.99 | |  | -91.33 |  | -14.38 |
| --- | --- | --- | --- | --- | --- | --- | --- | --- | --- | --- | --- | --- | --- | --- | --- |
| -233.34 | |  | -33.18 | -275.65 | | NA |  | -227.22 | | -274.56 | | -134.12 | |  | -42.43 |
| -349.22 | |  | -70.96 | -281.93 | |  | -22.08 | -205.64 | | -546.02 | | -180.79 | |  | -83.2 |
| -248.07 | |  | -49.69 |  | -17.01 | NA |  |  | -44.45 | -337.06 | |  | -14.94 |  | -10.25 |
| NA |  | NA |  | NA |  | NA |  | -2168.63 | | NA |  | NA |  | -1513.2 | |
| NA |  | NA |  | NA |  | NA |  | -1065.69 | | NA |  | NA |  | -816.99 | |
| NA |  | NA |  | NA |  | NA |  | -1556.99 | | NA |  | NA |  | -840.41 | |
| NA |  | NA |  | NA |  | NA |  | -1058.13 | | NA |  | NA |  | -499.55 | |
| NA |  | NA |  |  | -12.46 | NA |  | -1644.29 | | NA |  | NA |  |  | -957 |
| NA |  | NA |  |  | -8.69 | NA |  | -1501.86 | | NA |  | NA |  | -796.78 | |
| NA |  | NA |  | NA |  | NA |  | -484.37 | | NA |  | NA |  |  | -239.3 |
| NA |  | NA |  | NA |  | NA |  | -484.37 | | NA |  | NA |  |  | -239.3 |
| NA |  | NA |  | NA |  | NA |  | -484.37 | | NA |  | NA |  |  | -239.3 |
| NA |  | NA |  | NA |  | NA |  | -1408.06 | | NA |  | NA |  | -695.55 | |
| NA |  | NA |  |  | -15.83 | NA |  | -1489.73 | | NA |  | NA |  | -810.29 | |
|  | -19.52 | NA |  |  | -40.24 | NA |  | -1418.93 | | NA |  |  | -8.8 | -970.62 | |
| NA |  | NA |  | NA |  | NA |  | -789.75 | | NA |  | NA |  | -272.47 | |
| NA |  | NA |  | NA |  | NA |  |  | -819.5 | NA |  | NA |  | -418.59 | |
|  | -42.43 |  | -6.35 |  | -87.61 | NA |  | -1240.73 | | NA |  |  | -42.2 | -575.25 | |
|  | -6.67 | NA |  |  | -30.62 | NA |  | -1662.06 | | NA |  |  | -10.32 | -924.13 | |
| NA |  | NA |  | NA |  | NA |  | -267.75 | | NA |  | NA |  |  | -9.41 |
| -2255.77 | | -714.68 | | -8550.55 | | -833.55 | |  | -347.3 | -2796.19 | | -6276.09 | | -287.93 | |
|  | -12.61 | NA |  | -565.62 | |  | -19.87 | -1941.91 | | NA |  | -416.91 | | -323.89 | |
|  | -12.61 | NA |  | -565.62 | |  | -19.87 | -1941.91 | | NA |  | -416.91 | | -323.89 | |
| NA |  | NA |  | -128.65 | | NA |  | -974.63 | | NA |  |  | -33.2 |  | -30.03 |
| NA |  | NA |  | -128.65 | | NA |  | -974.63 | | NA |  |  | -33.2 |  | -30.03 |
| NA |  | NA |  | -353.45 | | NA |  | -2744.88 | | NA |  | -200.39 | |  | -110.6 |
|  | -32.62 | NA |  |  | -876.2 |  | -40.72 | -4751.44 | | NA |  | -608.64 | | -399.69 | |
| NA |  | NA |  |  | -98.64 | NA |  | -1518.43 | | NA |  |  | -28.26 |  | -68.78 |
| NA |  | NA |  | -126.98 | | NA |  | -1602.7 | | NA |  |  | -53.44 |  | -25.25 |
| NA |  | NA |  | -126.98 | | NA |  | -1602.7 | | NA |  |  | -53.44 |  | -25.25 |
| NA |  | NA |  | -126.98 | | NA |  | -1602.7 | | NA |  |  | -53.44 |  | -25.25 |
| NA |  | NA |  | NA |  | NA |  |  | -10.39 | NA |  | NA |  | NA |  |
| NA |  | NA |  | NA |  | NA |  |  | -19.99 | NA |  | NA |  | NA |  |
| NA |  | NA |  | NA |  | NA |  |  | -24.97 | NA |  | NA |  | NA |  |
| NA |  | NA |  | NA |  | NA |  |  | -24.7 | NA |  | NA |  | NA |  |
| -113.78 | |  | -39.58 |  | -39.01 |  | -17.06 |  | -33.06 |  | -80.3 |  | -71.36 |  | -317.9 |
| -145.24 | |  | -65.9 |  | -8.3 |  | -59.03 | NA |  | -432.94 | |  | -63.88 | -374.51 | |
| -145.24 | |  | -65.9 |  | -8.3 |  | -59.03 | NA |  | -432.94 | |  | -63.88 | -374.51 | |

| -297.69 | |  | -162.3 |  | -33.3 | -147.41 | |  | -12.63 | -638.42 | | -102.16 | | -687.44 | |
| --- | --- | --- | --- | --- | --- | --- | --- | --- | --- | --- | --- | --- | --- | --- | --- |
| NA |  |  | -5.25 | NA |  | NA |  | NA |  |  | -85.31 | NA |  |  | -80.81 |
| -121.03 | |  | -96.83 | -116.21 | |  | -83.36 |  | -21.65 | -381.36 | | -159.06 | | -290.95 | |
|  | -90.7 |  | -38.71 | NA |  |  | -28.03 | NA |  | -186.16 | | NA |  |  | -151.3 |
| -194.96 | |  | -56.68 | -322.77 | |  | -55.54 |  | -57.98 |  | -285.2 | -324.43 | | -261.12 | |
| NA |  | NA |  | -447.47 | | NA |  | NA |  | NA |  | NA |  | -160.44 | |
| NA |  | NA |  | NA |  | NA |  | -611.56 | | NA |  | NA |  |  | -26.59 |
| NA |  | NA |  | NA |  | NA |  | -827.73 | | NA |  | NA |  |  | -62.93 |
| -149.31 | |  | -38.58 | NA |  | NA |  | -4100.49 | |  | -27.38 | NA |  | -3796.7 | |
| -135.83 | |  | -43.02 |  | -5.38 | NA |  | -3977.29 | |  | -13.28 | NA |  | -3779.76 | |
| -235.02 | |  | -75.42 |  | -40.56 | NA |  | -3328.33 | |  | -81.64 |  | -33.66 | -3534.4 | |
| NA |  | NA |  | NA |  | NA |  |  | -48.42 | NA |  | NA |  | NA |  |
| NA |  | NA |  | NA |  | NA |  | -987.81 | | NA |  | NA |  | NA |  |
| NA |  | NA |  | NA |  | NA |  | -299.59 | | NA |  | NA |  | NA |  |
| NA |  | NA |  | NA |  | NA |  | -355.72 | | NA |  | NA |  | NA |  |
| NA |  | NA |  | NA |  | NA |  |  | -83.96 | NA |  | NA |  | NA |  |
| NA |  | NA |  | NA |  | NA |  |  | -32.97 | NA |  | NA |  | NA |  |
| NA |  | NA |  | NA |  | NA |  |  | -71.38 | NA |  | NA |  | NA |  |
| NA |  | NA |  | NA |  | NA |  |  | -60.2 | NA |  | NA |  | NA |  |
| NA |  | NA |  |  | -43.1 | NA |  | -462.12 | | NA |  |  | -53.03 | NA |  |
| NA |  | NA |  | NA |  | NA |  | -529.87 | | NA |  | NA |  | NA |  |
| NA |  | NA |  | NA |  | NA |  |  | -63.76 | NA |  | NA |  | NA |  |
| NA |  | NA |  | NA |  | NA |  |  | -50.59 | NA |  | NA |  | NA |  |
| NA |  | NA |  | NA |  | NA |  |  | -78.64 | NA |  | NA |  | NA |  |
| NA |  | NA |  | NA |  | NA |  | -1181.86 | | NA |  | NA |  | NA |  |
| NA |  | NA |  |  | -42.52 | NA |  | -748.47 | | NA |  |  | -34.06 | NA |  |
| NA |  | NA |  | NA |  | NA |  |  | -571.4 | NA |  | NA |  | NA |  |
| NA |  | NA |  | NA |  | NA |  | -425.39 | | NA |  | NA |  | NA |  |
| NA |  | NA |  | NA |  | NA |  |  | -21.36 | NA |  | NA |  | NA |  |
| NA |  | NA |  | NA |  | NA |  |  | -21.63 | NA |  | NA |  | NA |  |
| NA |  | NA |  |  | -63.02 | NA |  |  | -267.4 | NA |  |  | -41.02 |  | -17.76 |
| NA |  | NA |  | NA |  | NA |  |  | -29.27 | NA |  | NA |  | NA |  |
| NA |  | NA |  | NA |  | NA |  | -134.67 | | NA |  | NA |  | NA |  |
| NA |  | NA |  | NA |  | NA |  |  | -55.38 | NA |  | NA |  | NA |  |
| NA |  | NA |  | NA |  | NA |  | -906.05 | | NA |  | NA |  | NA |  |
| NA |  | NA |  |  | -16.56 | NA |  | -1052.52 | | NA |  | NA |  | NA |  |
| NA |  | NA |  | NA |  | NA |  | -905.16 | | NA |  | NA |  | NA |  |
| NA |  | NA |  |  | -18.79 | NA |  | -1062.33 | | NA |  | NA |  | NA |  |
| NA |  | NA |  | NA |  | NA |  | -386.77 | | NA |  | NA |  | NA |  |

| NA |  | NA |  | NA |  | NA |  |  | -60.11 | NA |  | NA |  | NA |  |
| --- | --- | --- | --- | --- | --- | --- | --- | --- | --- | --- | --- | --- | --- | --- | --- |
| NA |  | NA |  |  | -70.02 | NA |  | -482.39 | | NA |  |  | -80.97 | NA |  |
| NA |  | NA |  | NA |  | NA |  |  | -29.27 | NA |  | NA |  | NA |  |
| NA |  | NA |  | NA |  | NA |  | -310.79 | | NA |  | NA |  | NA |  |
| NA |  | NA |  | NA |  | NA |  | -745.74 | | NA |  | NA |  | NA |  |
| NA |  | NA |  |  | -38.59 | NA |  | -1021.63 | | NA |  |  | -5.69 | NA |  |
| NA |  | NA |  | NA |  | NA |  | -440.13 | | NA |  | NA |  | NA |  |
| NA |  | NA |  | NA |  | NA |  | -500.71 | | NA |  | NA |  | NA |  |
| NA |  | NA |  | NA |  | NA |  |  | -33.06 | NA |  | NA |  | NA |  |
| NA |  | NA |  |  | -18.2 | NA |  | -1114.47 | | NA |  | NA |  | NA |  |
| -117.42 | |  | -7.53 | -2199.99 | |  | -24.93 |  | -56.22 | -278.84 | | -1349.76 | |  | -20.72 |
| NA |  | NA |  | -587.71 | | NA |  | -501.78 | |  | -13.91 |  | -13.67 |  | -671.8 |
| NA |  | NA |  | -107.27 | | NA |  | NA |  |  | -22.24 |  | -9.68 | NA |  |
|  | -9.53 | NA |  | -735.82 | | NA |  |  | -30.92 | -247.69 | |  | -355.8 |  | -15.61 |
|  | -12.45 | NA |  | -363.45 | |  | -12.16 |  | -87.28 | -305.31 | | -180.76 | |  | -94.9 |
| NA |  | NA |  | -259.76 | | NA |  |  | -25.7 | -260.91 | | -121.03 | | -127.91 | |
| NA |  | NA |  | NA |  | NA |  | NA |  |  | -257.5 | NA |  |  | -96.59 |
| NA |  | NA |  | -168.73 | | NA |  |  | -20.63 |  | -22.09 |  | -23.73 |  | -14.59 |
|  | -16.87 | NA |  | -677.53 | | NA |  |  | -90.57 | -527.67 | | -321.92 | |  | -87.21 |
| NA |  | NA |  | -130.37 | | NA |  |  | -5.22 | -107.79 | |  | -32.65 |  | -14.57 |
|  | -243.2 |  | -40.01 |  | -74.55 | NA |  | -132.01 | | -283.06 | |  | -40.44 | NA |  |
| -271.37 | |  | -45.69 |  | -75.17 |  | -12.14 |  | -82.37 |  | -393.2 |  | -47.3 |  | -6.59 |
| -289.52 | |  | -36.06 |  | -212.4 |  | -7.75 | -174.13 | | -466.66 | | -105.83 | |  | -36.21 |
| -134.09 | |  | -23.96 |  | -14.66 | NA |  |  | -51.01 | -287.12 | |  | -7.11 | NA |  |
|  | -15.95 | NA |  | NA |  | NA |  |  | -74.67 | NA |  |  | -39.51 |  | -7.55 |
|  | -13.75 | NA |  | -107.73 | | NA |  |  | -9.02 | -126.31 | |  | -79.72 | -126.78 | |
|  | -126.5 |  | -42.9 | NA |  |  | -37.93 |  | -31.02 |  | -90.39 |  | -32.69 |  | -27.58 |
|  | -36.12 |  | -9.17 |  | -15.51 |  | -10.94 |  | -40.16 |  | -65.83 |  | -39.3 |  | -31.22 |
|  | -36.12 |  | -9.17 |  | -15.51 |  | -10.94 |  | -40.16 |  | -65.83 |  | -39.3 |  | -31.22 |
|  | -28.87 |  | -6.05 | NA |  |  | -17.39 | NA |  |  | -23.47 |  | -22.4 | NA |  |
|  | -45.4 | NA |  |  | -35.42 |  | -23.69 |  | -18.33 |  | -25.09 | -179.06 | |  | -14.17 |
|  | -35.26 | NA |  | NA |  | NA |  |  | -51.99 |  | -5.87 |  | -39.56 | NA |  |
|  | -35.26 | NA |  | NA |  | NA |  |  | -51.99 |  | -5.87 |  | -39.56 | NA |  |
|  | -35.26 | NA |  | NA |  | NA |  |  | -51.99 |  | -5.87 |  | -39.56 | NA |  |
|  | -90.93 |  | -12.47 |  | -54.9 |  | -27.12 | -102.76 | |  | -12.04 | -237.69 | |  | -19.75 |
| -231.77 | | -105.48 | | -134.91 | | -123.41 | |  | -20.6 | -155.95 | |  | -335.2 |  | -83.13 |
|  | -11.33 | NA |  | NA |  | NA |  |  | -25 |  | -23.84 | NA |  | NA |  |
| NA |  | NA |  | NA |  | NA |  |  | -55.81 | NA |  | NA |  | NA |  |
| NA |  | NA |  |  | -50.2 | NA |  | -685.99 | | NA |  |  | -9.91 | -286.68 | |

| NA |  | NA |  | NA |  | NA |  |  | -77.58 | NA |  | NA |  | NA |  |
| --- | --- | --- | --- | --- | --- | --- | --- | --- | --- | --- | --- | --- | --- | --- | --- |
| NA |  | NA |  | NA |  | NA |  |  | -77.66 | NA |  | NA |  | NA |  |
| NA |  | NA |  | NA |  | NA |  |  | -27.18 | NA |  | NA |  | NA |  |
| NA |  | NA |  | NA |  | NA |  |  | -33.8 | NA |  | NA |  | NA |  |
| -300.83 | | -106.07 | | -215.16 | | -145.16 | |  | -82.46 |  | -783.2 | -284.36 | | -322.42 | |
| -282.61 | |  | -91.01 | -314.89 | |  | -84.33 | -248.25 | | -334.43 | | -267.71 | | -600.16 | |
|  | -171.9 |  | -42.12 |  | -46.43 |  | -30.75 | NA |  | -244.69 | |  | -86.4 | -370.43 | |
| -317.28 | | -138.97 | | -579.49 | | -114.37 | | -274.86 | | -394.35 | | -457.97 | | -457.61 | |
|  | -93.75 |  | -23.19 |  | -17.44 |  | -28.06 | NA |  | -218.86 | |  | -32.74 |  | -74.08 |
| -124.05 | |  | -37.26 |  | -88.78 |  | -46.76 |  | -18.11 | -242.75 | |  | -87.38 | -128.65 | |
|  | -84.87 |  | -37.73 | -257.82 | |  | -50.75 | -114.84 | | -479.87 | | -203.99 | | -675.63 | |
| -679.72 | | -279.25 | | -116.73 | | -240.59 | | -161.14 | | -167.07 | |  | -237.3 | -243.79 | |
| -724.54 | | -311.65 | | -242.47 | | -243.38 | | -340.83 | | -295.29 | | -365.69 | | -320.81 | |
|  | -5.14 | NA |  |  | -68.16 | NA |  | -100.78 | | -158.01 | |  | -42.06 | -246.52 | |
| NA |  | NA |  | NA |  | NA |  | -111.26 | | NA |  | NA |  | NA |  |
| NA |  | NA |  | NA |  | NA |  |  | -44.49 | NA |  | NA |  | NA |  |
|  | -24.84 | NA |  | -142.31 | |  | -18.47 |  | -131 | -462.03 | |  | -98.06 | -824.73 | |
| NA |  | NA |  | NA |  | NA |  |  | -35.4 | NA |  | NA |  | NA |  |
| -142.43 | |  | -42.65 | -363.38 | |  | -77.15 | -368.23 | | -500.48 | | -319.01 | | -452.08 | |
| NA |  | NA |  | NA |  | NA |  |  | -81.48 | NA |  | NA |  | NA |  |
| NA |  | NA |  | NA |  | NA |  |  | -81.48 | NA |  | NA |  | NA |  |
| -111.09 | |  | -60.71 |  | -151.8 |  | -60.07 | -178.86 | | -199.44 | | -144.43 | | -358.58 | |
|  | -96.9 |  | -47.98 | -276.83 | |  | -57.44 | -110.94 | | -383.74 | | -221.17 | | -750.91 | |
|  | -96.9 |  | -47.98 | -276.83 | |  | -57.44 | -110.94 | | -383.74 | | -221.17 | | -750.91 | |
|  | -96.9 |  | -47.98 | -276.83 | |  | -57.44 | -110.94 | | -383.74 | | -221.17 | | -750.91 | |
|  | -96.9 |  | -47.98 | -276.83 | |  | -57.44 | -110.94 | | -383.74 | | -221.17 | | -750.91 | |
| -134.05 | |  | -37.14 |  | -670.7 |  | -67.18 | -212.63 | | -250.25 | | -518.02 | | -346.77 | |
| -134.05 | |  | -37.14 |  | -670.7 |  | -67.18 | -212.63 | | -250.25 | | -518.02 | | -346.77 | |
| -134.05 | |  | -37.14 |  | -670.7 |  | -67.18 | -212.63 | | -250.25 | | -518.02 | | -346.77 | |
| -134.05 | |  | -37.14 |  | -670.7 |  | -67.18 | -212.63 | | -250.25 | | -518.02 | | -346.77 | |
|  | -32.17 |  | -13.5 | -575.04 | |  | -16.33 | -969.03 | | -130.24 | | -243.09 | | -878.08 | |
|  | -32.17 |  | -13.5 | -575.04 | |  | -16.33 | -969.03 | | -130.24 | | -243.09 | | -878.08 | |
|  | -18.86 | NA |  | -377.17 | |  | -15.73 | -351.87 | |  | -60.71 | -165.03 | |  | -375.9 |
|  | -18.86 | NA |  | -377.17 | |  | -15.73 | -351.87 | |  | -60.71 | -165.03 | |  | -375.9 |
| -196.01 | | -106.45 | | -475.15 | |  | -117.5 | -206.14 | | -700.62 | |  | -392 | -1180.23 | |
| -179.38 | |  | -73.92 | -271.68 | | -110.07 | | -232.19 | | -846.33 | | -336.49 | | -406.49 | |
| -179.38 | |  | -73.92 | -271.68 | | -110.07 | | -232.19 | | -846.33 | | -336.49 | | -406.49 | |
| -113.65 | |  | -32.84 |  | -468.4 |  | -65.41 | -391.53 | | -459.94 | |  | -350 | -410.38 | |
| -113.65 | |  | -32.84 |  | -468.4 |  | -65.41 | -391.53 | | -459.94 | |  | -350 | -410.38 | |

| -126.03 | |  | -50.32 | -211.82 | |  | -96.14 | -157.45 | | -793.34 | | -300.19 | | -354.31 | |
| --- | --- | --- | --- | --- | --- | --- | --- | --- | --- | --- | --- | --- | --- | --- | --- |
| -361.68 | | -138.83 | | -429.98 | | -162.66 | | -329.98 | | -1006.57 | | -513.98 | | -604.25 | |
| -158.21 | |  | -73.8 | -215.01 | |  | -86.18 | -116.34 | | -567.46 | | -279.78 | | -497.32 | |
| NA |  | NA |  | -120.09 | |  | -5.58 | NA |  | -145.43 | |  | -91.13 |  | -39.9 |
| -161.65 | |  | -34.3 | -459.51 | |  | -32.64 | -149.95 | | -369.77 | | -312.31 | | -109.79 | |
| -197.32 | |  | -43.98 |  | -35.14 | NA |  | -107.78 | | -230.57 | |  | -20.72 | NA |  |
| -190.61 | |  | -48.88 | -377.99 | |  | -63.41 |  | -71.45 | -433.78 | | -316.79 | | -168.77 | |
| -783.77 | | -251.58 | | -209.67 | |  | -52.52 |  | -27.83 | -199.22 | | -327.49 | | -1667.32 | |
| -945.18 | | -302.42 | | -248.38 | |  | -73.22 |  | -35.71 |  | -257.9 | -422.91 | | -1919.25 | |
| -149.45 | |  | -53.63 | NA |  | NA |  | NA |  |  | -32.38 | NA |  | -470.47 | |
| -995.72 | |  | -336.3 | -260.71 | |  | -70.02 |  | -44.18 | -261.22 | | -428.31 | | -2120.11 | |
|  | -465.2 |  | -155.4 | -224.42 | |  | -28.22 |  | -36.63 |  | -107.7 | -210.23 | | -1260.59 | |
| NA |  | NA |  | NA |  | NA |  | NA |  | -279.93 | | NA |  | NA |  |
| -117.24 | |  | -30.78 | -108.76 | |  | -21.75 | NA |  | -370.58 | |  | -161.9 |  | -81.24 |
| NA |  | NA |  | NA |  | NA |  | NA |  | -256.23 | | NA |  | NA |  |
| NA |  | NA |  | NA |  | NA |  | NA |  |  | -7.34 | NA |  | NA |  |
| NA |  | NA |  | NA |  | NA |  | NA |  | NA |  | NA |  | NA |  |
| NA |  | NA |  |  | -42.17 | NA |  | NA |  |  | -13.76 |  | -21.61 |  | -12.55 |
| NA |  | NA |  | NA |  | NA |  | NA |  | NA |  | NA |  | NA |  |
| NA |  | NA |  | NA |  | NA |  | NA |  | NA |  | NA |  | NA |  |
| NA |  | NA |  | NA |  | NA |  | NA |  |  | -16.6 | NA |  | NA |  |
| NA |  | NA |  | NA |  | NA |  | NA |  | NA |  | NA |  | NA |  |
| NA |  | NA |  | NA |  | NA |  | NA |  | NA |  | NA |  | NA |  |
| NA |  | NA |  |  | -83.47 | NA |  | NA |  |  | -8.79 |  | -43.8 | NA |  |
| NA |  | NA |  | -292.56 | | NA |  | NA |  |  | -29.28 | -210.95 | | NA |  |
| NA |  | NA |  | NA |  | NA |  |  | -28.2 | NA |  | NA |  | NA |  |
| NA |  | NA |  | NA |  | NA |  |  | -36.49 | NA |  | NA |  | NA |  |
| NA |  | NA |  | NA |  | NA |  |  | -37.57 | NA |  | NA |  | NA |  |
| -135.68 | |  | -56.37 | -477.82 | |  | -77.83 | -241.08 | |  | -654.2 |  | -381.7 | -1249.15 | |
| -112.54 | |  | -27.97 | -236.09 | |  | -46.55 |  | -80.68 |  | -52.54 | -396.76 | |  | -141.4 |
| -112.54 | |  | -27.97 | -236.09 | |  | -46.55 |  | -80.68 |  | -52.54 | -396.76 | |  | -141.4 |
|  | -9.66 | NA |  | NA |  | NA |  | -597.18 | | NA |  | NA |  | -594.04 | |
|  | -79.89 |  | -27.06 | NA |  | NA |  |  | -71.25 |  | -76.17 | NA |  |  | -850.3 |
|  | -79.1 |  | -14.45 | NA |  | NA |  | -401.32 | |  | -17.05 | NA |  | -1225.93 | |
|  | -81.32 |  | -16.87 | NA |  | NA |  | -1407.08 | |  | -15.98 |  | -7.83 | -1767.88 | |
| -655.58 | | -264.51 | | -693.92 | | -211.13 | |  | -30.86 | -617.99 | | -720.45 | |  | -94.75 |
|  | -143.8 |  | -38.57 |  | -5.97 | NA |  | -2568.68 | |  | -29.3 | NA |  | -2844.56 | |
|  | -29.59 | NA |  | NA |  | NA |  |  | -75.49 | NA |  | NA |  | -650.28 | |
| -176.34 | |  | -57.96 | NA |  | NA |  | -1624.27 | |  | -34.58 | NA |  | -2279.77 | |

| -81.36 | -19.44 | -28.24 NA | -1528.04 NA |  | -11.48 | -1778.08 |
| --- | --- | --- | --- | --- | --- | --- |
| -65.68 | -11.75 NA | NA | -723.28 NA | NA |  | -786.21 |
| -44.86 | -8.82 NA | NA | -829.48 NA | NA |  | -906.17 |
| -84.63 | -11.61 NA | NA | -797.37 NA | NA |  | -1214.6 |

-90.72	-26.61 NA	NA	-343.37	-22.85 NA	-741.86

-308.77	-101.92	-414.91	-97.96	-378.49	-287.61	-304.7	-590.5

NA	NA	NA	NA	-17.48 NA	NA	-186.54

NA	NA	NA	NA	-17.48 NA	NA	-186.54

NA	NA	NA	NA	-17.48 NA	NA	-186.54

NA	NA	NA	NA	NA	NA	NA	NA

-44.83	-19.22	-30.26	-18.19	-10	-593.14	-24.72	-62.03

-22.59 NA	-13.35 NA	-20.94	-594.67 NA	-187.84

-169.99	-55.16	-272.41	-46.47	-164.11	-351.7	-152.4	-383.62

NA	NA	NA	NA	NA	NA	NA	NA

-60.02	-18.44 NA	-23.42 NA	-891.44 NA	-72.23

NA	NA	-46.79 NA	-37.88	-259.08	-6.65	-39.46

-46.26	-10.31	-26.9 NA	-52.98	-136.04	-21.88	-170.88

-67.39	-20.49 NA	-17.76 NA	-406.98 NA	-93.17

NA	NA	NA	NA	NA	NA	NA	NA

-150.55	-87 NA	-88.12 NA	-274.29	-136.07	-28.71

| -236.2 | | -110.69 | -153.28 -138.69 | | | -91.06 | | -280.52 | | -321.34 | -134.8 |
| --- | --- | --- | --- | --- | --- | --- | --- | --- | --- | --- | --- |
| -128.67 | | -48.23 | -51.5 NA | | | -757.39 | | -45.1 | | -54.73 | -1239.71 |
| -153.82 | | -39.26 | -43.65 NA | | | -1900.82 | | -13.24 | | -44.58 | -2088 |
| -374.6 | | -182.06 | -572.48 -205.65 | | | -283.5 | | -394.67 | | -520.21 | -216.69 |
| -116.31 | | -29.57 | -181.57 -16.51 | | | -87.09 | | -247.62 | | -175.39 | -299.89 |
| -99.26 | | -29.74 | -5.6 NA | | | -153.09 | | -29.28 | | -11.73 | -505.26 |
| -135.15 -41.03 -136.19 -30.81 NA -463.24 -187.9 -68.12 | | | | | | | | | | | |
| -51.35 | | -15.37 | -22.39 | | -7.7 | -12.51 | | -227.32 | | -37.7 | -107.55 |
| -137.02 | | -33.4 | -324.33 | | -40.78 | -171.1 | | -248.08 | | -221.7 | -323.36 |
| -80.24 | | -23.7 | -19.18 | | -20.37 | -11.31 | | -119.73 | | -18.34 | -77.21 |
| -71.03 | | -17.1 | -94.54 | | -13.64 | -243.89 | | -49.15 | | -70.99 | -124.97 |
| -75.25 | | -21.41 | -80.15 | | -9.18 | -82.67 | | -88 | | -68.43 | -123.28 |
| -96.89 | | -28.74 | -139.96 | | -33.73 | -218.81 | | -103.89 | | -108.82 | -159.02 |
| -104.87 | | -38.8 | -77.45 | | -36.72 | -51.19 | | -243.9 | | -126.56 | -157.75 |
| -44.61 | | -12.18 | -112.75 NA | |  | -144.14 | | -27.22 | | -55.75 | -103.73 |
|  | -10.66 NA | |  | -28.5 NA |  |  | -71.92 |  | -10.06 NA | | -18 |
| NA | NA | | NA | NA |  | NA |  | NA | NA | | -49.29 |
| NA | NA | | NA | NA |  | NA |  | NA | NA | | -49.29 |
| NA | NA | | NA | NA |  | NA |  | NA | NA | | -49.29 |

| NA |  | NA |  | -2145.35 | | -101.87 | | NA |  | -852.66 | | -1503.74 | | NA |  |
| --- | --- | --- | --- | --- | --- | --- | --- | --- | --- | --- | --- | --- | --- | --- | --- |
| NA |  | NA |  | -2782.1 | | -120.86 | | NA |  | -965.58 | | -1927.75 | | NA |  |
| NA |  | NA |  | -1202.45 | |  | -93.62 | NA |  | -450.55 | | -1023.28 | | NA |  |
| -150.77 | |  | -38.74 |  | -10.62 | NA |  | -1906.9 | |  | -20.63 |  | -23.6 | -2239.68 | |
| -156.43 | |  | -50.2 | NA |  | NA |  | -581.44 | |  | -31.11 | NA |  | -1591.04 | |
|  | -60.1 |  | -14.39 | NA |  | NA |  | -1613.87 | | NA |  | NA |  | -1647.75 | |
|  | -72.76 |  | -16.37 | NA |  | NA |  | -1728.02 | | NA |  | NA |  | -1932.25 | |
| NA |  | NA |  |  | -69.44 | NA |  |  | -91.83 |  | -16.44 |  | -18.49 | -102.02 | |
| -139.65 | |  | -44.61 |  | -84.83 |  | -28.46 |  | -52.61 | -185.49 | | -168.01 | | -267.54 | |
|  | -94.55 |  | -23.38 |  | -65.6 |  | -21.95 |  | -87.07 | -200.39 | | -127.06 | | -429.77 | |
| -188.95 | |  | -48.37 | -120.35 | |  | -25.43 | -102.49 | | -168.57 | | -134.56 | | -506.25 | |
| -188.95 | |  | -48.37 | -120.35 | |  | -25.43 | -102.49 | | -168.57 | | -134.56 | | -506.25 | |
| -169.47 | |  | -51.75 |  | -152 |  | -38.89 | -148.35 | | -162.16 | | -156.38 | | -457.01 | |
| -211.88 | |  | -24.4 |  | -28.02 | NA |  |  | -29.68 | -470.37 | |  | -17.87 |  | -7.37 |
| -211.88 | |  | -24.4 |  | -28.02 | NA |  |  | -29.68 | -470.37 | |  | -17.87 |  | -7.37 |
|  | -6.57 | NA |  |  | -53.31 | NA |  | -351.71 | | NA |  |  | -8.62 |  | -86.71 |
|  | -26.03 | NA |  |  | -8.4 | NA |  | -161.31 | | NA |  | NA |  |  | -23.02 |
|  | -97.02 |  | -6.89 |  | -19.89 | NA |  | -237.26 | | NA |  | NA |  |  | -60.85 |
|  | -25.35 | NA |  | NA |  |  | -13.3 | NA |  | NA |  | -110.12 | | NA |  |
| -1159.61 | | -363.62 | | -3315.11 | | -299.62 | | -196.49 | | -1041.02 | | -2352.73 | | -236.93 | |
|  | -66.63 | NA |  | -7822.44 | | -242.56 | |  | -73.75 | -1531.22 | | -4328.92 | |  | -7.49 |
|  | -11.1 | NA |  | -3112.86 | | NA |  |  | -32.07 | -375.51 | | -1734.21 | | NA |  |
|  | -466.7 | -101.43 | | -4697.52 | | -187.43 | | -179.52 | | -866.98 | | -3109.31 | |  | -81.12 |
| -225.66 | |  | -27.88 | -10949.5 | | -313.46 | | -131.73 | | -2089.1 | | -5870.26 | |  | -27.95 |
| -136.99 | | NA |  | -7480.97 | | -141.48 | |  | -106 | -1128.59 | | -4224.34 | |  | -30.27 |
| -224.17 | |  | -56.51 | -299.35 | |  | -5.25 | -157.24 | |  | -35.4 | -195.35 | | -117.76 | |
|  | -10.51 | NA |  | -784.24 | | NA |  |  | -70.77 | -393.25 | | -347.84 | |  | -32.28 |
|  | -279.5 |  | -77.9 | -280.12 | |  | -14.17 |  | -181 |  | -67.7 | -206.65 | | -227.94 | |
| NA |  | NA |  |  | -63.91 | NA |  |  | -24.42 |  | -90.47 |  | -37.89 | -158.49 | |
| -283.27 | | -118.44 | | -282.26 | | -120.48 | | -213.41 | | -868.88 | | -406.97 | | -496.94 | |
| -138.05 | |  | -43.79 | -480.94 | |  | -77.02 | -365.36 | | -425.07 | | -382.54 | | -390.37 | |
| NA |  | NA |  | NA |  | NA |  |  | -6.46 | -409.26 | | NA |  |  | -78.46 |
| NA |  | NA |  |  | -23.56 | NA |  |  | -829.9 | NA |  | NA |  | -259.94 | |
|  | -32.76 | NA |  | NA |  | NA |  | -174.28 | | NA |  | NA |  |  | -85.48 |
|  | -7.95 | NA |  | NA |  | NA |  |  | -93.26 | NA |  | NA |  |  | -19.22 |
| -229.68 | | -130.52 | | NA |  |  | -98.79 | NA |  | -323.14 | |  | -19.81 |  | -74.87 |
| -393.72 | | -194.77 | |  | -64.63 | -140.85 | |  | -40.46 |  | -459.8 | -194.54 | | -355.36 | |
| -379.62 | |  | -213.7 |  | -13.56 | -162.46 | | NA |  | -401.87 | | -141.69 | | -104.09 | |
|  | -9.1 |  | -10.16 | NA |  |  | -9.2 | NA |  |  | -24.51 | NA |  | NA |  |

| -415.03 | | -190.59 | |  | -29.7 | -155.73 | |  | -21.82 |  | -454.5 | -147.13 | |  | -263 |
| --- | --- | --- | --- | --- | --- | --- | --- | --- | --- | --- | --- | --- | --- | --- | --- |
| -484.29 | |  | -198.4 | -389.22 | | -197.07 | | -219.02 | | -810.99 | |  | -506.1 | -602.22 | |
|  | -54.01 |  | -25.28 | -213.94 | |  | -28.1 | -126.32 | | -455.19 | | -141.52 | |  | -718.5 |
|  | -54.56 |  | -19.84 |  | -181.6 |  | -33.44 |  | -81.5 | -429.36 | | -127.51 | | -591.27 | |
|  | -23.89 |  | -5.48 |  | -92.53 |  | -17.72 |  | -35.35 | -327.34 | |  | -66.55 | -388.57 | |
|  | -8.24 |  | -5.82 |  | -73.02 |  | -12.08 |  | -66.46 | -106.79 | |  | -46.57 | -176.18 | |
| -146.45 | |  | -45.77 | NA |  | NA |  | NA |  | -119.77 | |  | -10.45 | -406.59 | |
|  | -55.04 |  | -28.56 | -176.29 | |  | -35.81 |  | -82.95 | -503.06 | | -135.27 | | -632.79 | |
|  | -51.26 |  | -24.98 |  | -66.53 |  | -27.14 |  | -26.01 | -411.69 | |  | -61.09 | -499.44 | |
| NA |  | NA |  | NA |  | NA |  |  | -31.43 | NA |  | NA |  | NA |  |
| NA |  | NA |  | NA |  | NA |  |  | -44.65 | NA |  | NA |  | NA |  |
| NA |  | NA |  | NA |  | NA |  | NA |  | NA |  | NA |  | NA |  |
| -507.34 | | -237.86 | | -599.16 | | -250.18 | | -308.11 | | -523.07 | |  | -684.2 | -261.76 | |
| -267.77 | | -137.93 | | -195.14 | | -120.79 | |  | -48.68 | -327.24 | | -242.63 | | -440.39 | |
| NA |  | NA |  |  | -7.47 | NA |  | NA |  | -161.33 | |  | -15.91 |  | -28.38 |
| -187.37 | |  | -74.23 |  | -68.76 |  | -67.2 |  | -40.22 | -341.46 | | -155.71 | | -265.28 | |
| NA |  | NA |  |  | -7.43 | NA |  | NA |  | -132.41 | |  | -12.32 |  | -17.19 |
| NA |  | NA |  | NA |  |  | -18.3 | NA |  | -108.31 | |  | -17.53 |  | -5.43 |
| -200.77 | | -101.07 | |  | -8.99 |  | -84.42 |  | -9.83 | -193.85 | |  | -78.34 | -162.87 | |
| NA |  | NA |  | NA |  | NA |  |  | -68.54 | NA |  | NA |  | NA |  |
| -572.28 | | -256.06 | | -443.29 | | -229.66 | | -330.14 | | -514.44 | | -416.87 | | -866.49 | |
|  | -58 |  | -5.91 |  | -14.77 | NA |  |  | -69.34 |  | -70.77 |  | -11.43 | -267.01 | |
|  | -157.1 |  | -61.32 | NA |  |  | -61.93 | NA |  | -144.46 | | NA |  |  | -28.3 |
| -157.23 | |  | -54.82 | NA |  |  | -44.38 | NA |  | -149.67 | | NA |  |  | -60.32 |
| -126.18 | |  | -77.37 |  | -29.57 |  | -97.32 | NA |  | -259.77 | | -159.72 | |  | -97.1 |
|  | -95.53 |  | -35.03 | -118.49 | |  | -63.3 |  | -7.09 |  | -69.32 | -320.62 | |  | -52.3 |
| -178.51 | |  | -90.59 | -101.42 | |  | -121.2 |  | -14.04 |  | -166.2 | -230.06 | |  | -84.92 |
| -228.81 | | -116.91 | |  | -35.16 |  | -107.2 | NA |  | -376.22 | | -128.49 | | -105.06 | |
|  | -28.33 | NA |  |  | -32.76 | NA |  | NA |  |  | -23.21 | -107.75 | |  | -39.16 |
|  | -154.1 |  | -42.85 | -209.47 | |  | -63.44 |  | -96.28 | -352.98 | |  | -233.6 | -408.64 | |
| -118.08 | |  | -34.6 | -270.87 | |  | -45.27 |  | -79.81 |  | -57.02 | -398.35 | | -407.05 | |
| -334.04 | | -153.91 | | -291.05 | | -186.82 | |  | -141.7 | -444.86 | | -274.49 | | -447.81 | |
|  | -82.23 |  | -27.46 | -171.12 | |  | -9.26 |  | -28.83 | -120.59 | | -143.44 | |  | -212.9 |
|  | -33.81 | NA |  | -213.71 | | NA |  |  | -60.31 | -157.63 | | -169.83 | | -265.86 | |
|  | -70.45 |  | -33.65 | NA |  |  | -55.76 | NA |  |  | -93.7 |  | -32.68 |  | -17.88 |
| -251.89 | |  | -39.76 | -524.41 | |  | -7.77 | -117.55 | |  | -66.36 | -381.03 | |  | -60.67 |
| -433.42 | |  | -66.99 | -1329.77 | |  | -30.4 |  | -75.99 | -189.11 | | -735.68 | |  | -30.83 |
|  | -143.8 |  | -50.53 |  | -56.33 |  | -32.11 |  | -6.28 |  | -92.21 | -113.45 | | -180.52 | |
| -364.96 | | -207.55 | | -244.48 | | -216.02 | | -218.48 | | -254.89 | |  | -210.9 | -273.51 | |

| -149.53 | |  | -82.71 | -137.53 | |  | -52 |  | -22.88 |  | -164.7 | -194.69 | |  | -95.27 |
| --- | --- | --- | --- | --- | --- | --- | --- | --- | --- | --- | --- | --- | --- | --- | --- |
| -281.33 | |  | -88.45 |  | -346.9 |  | -80.6 | -102.42 | | -200.25 | | -449.77 | | -256.93 | |
| -191.38 | |  | -66.63 | -416.82 | |  | -47.99 | -644.77 | | -328.49 | | -267.12 | | -1460.87 | |
|  | -23.02 |  | -13.68 |  | -38.5 |  | -18.66 | NA |  |  | -96.27 |  | -59.28 |  | -71.69 |
| -341.42 | |  | -98.05 | -1410.4 | |  | -87.57 | -826.99 | | -207.81 | | -717.75 | | -1103.01 | |
| NA |  | NA |  |  | -82.1 | NA |  |  | -55.82 | NA |  | NA |  | -239.52 | |
| -322.99 | | -132.73 | | -471.52 | | -122.56 | | -319.19 | | -395.01 | | -467.86 | | -420.08 | |
| NA |  | NA |  |  | -44.84 | NA |  |  | -39.32 |  | -276.1 |  | -85.25 | -200.89 | |
| -245.04 | | -116.31 | | -105.97 | | -103.02 | |  | -57.25 | -228.75 | | -181.13 | | -381.03 | |
|  | -38.69 |  | -8.11 | -128.03 | |  | -14.21 |  | -13.14 |  | -75.51 | -125.32 | | -101.67 | |
| -176.83 | |  | -64.52 |  | -96.98 |  | -41.65 |  | -14.84 | -111.89 | | -198.99 | | -210.84 | |
| NA |  | NA |  | NA |  | NA |  | -417.79 | | NA |  | NA |  | NA |  |
|  | -79.76 |  | -10.25 | -184.89 | |  | -17.96 | NA |  | -174.77 | | -185.94 | |  | -35.31 |
|  | -74.4 |  | -36.02 |  | -18.71 |  | -55.59 |  | -11.55 |  | -54.83 | -117.15 | |  | -35.46 |
|  | -68.52 |  | -34.38 |  | -74.29 |  | -63.03 | NA |  | -150.83 | | -229.53 | |  | -90.8 |
|  | -654.2 | -312.06 | | -504.87 | | -275.64 | | -251.25 | | -722.56 | | -597.43 | | -497.69 | |
|  | -53.65 |  | -41.24 | NA |  |  | -25.14 |  | -8.76 | -124.06 | |  | -19.61 | -138.83 | |
| -276.67 | | -150.82 | |  | -80.46 | -177.37 | |  | -58.17 | -445.07 | | -187.91 | |  | -215.5 |
| NA |  | NA |  |  | -13.88 | NA |  | NA |  |  | -29.13 | NA |  |  | -45.25 |
|  | -19.76 | NA |  | -110.71 | | NA |  | NA |  |  | -82.73 | -103.89 | |  | -74.36 |
|  | -36.15 | NA |  | -287.95 | |  | -14.71 |  | -26.37 | -109.66 | | -306.62 | | -220.84 | |
| NA |  | NA |  | -297.53 | | NA |  |  | -35.96 | NA |  |  | -132.8 | NA |  |
| -224.51 | | -118.53 | |  | -63.44 |  | -73.27 |  | -14.45 | -258.65 | | -114.31 | | -276.04 | |
|  | -98.22 |  | -6.69 | -478.21 | |  | -9.82 |  | -16.01 |  | -36.06 | -585.46 | |  | -12.14 |
|  | -23.3 |  | -5.7 |  | -44.49 | NA |  |  | -19.1 | -261.68 | | -145.26 | | -332.77 | |
|  | -55.55 | NA |  |  | -499.2 |  | -7.32 |  | -64.27 |  | -86.14 | -306.31 | |  | -98.5 |
| -135.01 | |  | -44.27 | -238.66 | |  | -17.19 | -148.52 | |  | -96.09 | -176.55 | | -403.66 | |
| -130.11 | |  | -45.17 |  | -83 |  | -44.87 |  | -24.74 |  | -31.93 | -300.86 | |  | -71.28 |
|  | -53.52 |  | -34.36 | -140.25 | |  | -31.5 |  | -129 |  | -116.6 |  | -88.93 | -297.24 | |
|  | -18.17 | NA |  |  | -82.36 | NA |  |  | -46.19 |  | -40.08 |  | -43.39 | -144.82 | |
|  | -50.41 |  | -8.84 | -560.81 | |  | -20.32 |  | -30.64 |  | -57.25 | -436.25 | |  | -43.65 |
| -439.01 | | -207.36 | | -135.57 | | -187.36 | | -151.11 | | -358.78 | | -296.88 | | -375.02 | |
| -164.84 | |  | -86.89 |  | -70.04 |  | -72.71 |  | -16.05 | -106.72 | | -203.04 | | -169.53 | |
| NA |  | NA |  | -387.53 | | NA |  |  | -10.04 | -106.05 | | -257.12 | |  | -28.41 |
| NA |  | NA |  | NA |  | NA |  | NA |  |  | -88.08 | NA |  |  | -35.52 |
|  | -93.34 |  | -26.87 |  | -61.71 |  | -30.87 |  | -61 |  | -231.6 |  | -79.26 |  | -308.8 |
|  | -75.97 |  | -39.44 | -205.66 | |  | -46.08 |  | -45.39 | -109.89 | | -154.78 | | -142.26 | |
|  | -84.63 |  | -14.49 | -196.35 | |  | -18.8 |  | -42.26 |  | -62.16 | -332.31 | | -121.47 | |

heart..nih_ hematopoi hematopoi hematopoi hematopoi hematopoi hematopoi hepatocyt

-134.64	-161.01	-106.43	-206.57	-168.59	-191.16	-200.25	-48.17

NA	NA	NA	NA	NA	NA	NA	NA

-286.26	-98.97	-173.45	-210.53	-151.12	-195.23	-299.17	-545.07

-23.99	-173.38	-111.83	-133.82	-108.85	-151.9	-193.78	-176.87

-21.88	-8.4 NA	-27.51	-11.49	-11.25	-63.95	-190.6

-901.3	-642.92	-438.89	-604.93	-656.13	-539.18	-577.14	-373.2

-240.72	-93.88	-124.63	-197.8	-117.63	-152.55	-249.45	-605.65

-51.06	-32.97	-24.18	-44.41	-31.06	-33.94	-64.75	-136.59

-51.06	-32.97	-24.18	-44.41	-31.06	-33.94	-64.75	-136.59

-212.62	-5.87	-22.57	-6.52 NA	NA	-28.38	-187.51

-864.36	-340.43	-261.34	-373.74	-398.99	-333.73	-417.08	-272.84

-176.25	-105.97	-52.16	-117.63	-122.94	-94.06	-170.33	-179.01

-125.18	-34.67	-91.6	-108.42	-27.83	-56.23	-85.88	-11.92

-228.02	-55.36	-103.59	-107.26	-62.99	-73.26	-144.12	-83.33

NA	NA	NA	NA	NA	NA	NA	-425.77

NA	NA	NA	NA	NA	NA	NA	NA

-26.66 NA	-34 NA	NA	NA	NA	-33.65

-26.66 NA	-34 NA	NA	NA	NA	-33.65

-247.57	-952.69	-2750.43	-1932.69	-1922.41	-2023.61	-1526.95	-59.93

-136.22	-76.04	-112.26	-193.41	-134.67	-158.23	-268.67	-712.54

NA	NA	NA	NA	NA	NA	NA	NA

-89.35	-815.2	-1571.46	-1214.87	-1021.69	-1081.8	-676.45	-60.56

NA	NA	NA	NA	NA	NA	NA	NA NA	NA	NA	NA	NA	NA	NA	NA

NA	NA	-22.04 NA	NA	NA	NA	-308.25

NA	NA	NA	NA	NA	NA	NA	-1188.95

NA	NA	NA	NA	NA	NA	NA	-1256.16

NA	NA	NA	NA	NA	NA	NA	-768.63

-337.43	-85.93	-193.18	-213.28	-153.96	-174.26	-356.65	-803.76

-337.43	-85.93	-193.18	-213.28	-153.96	-174.26	-356.65	-803.76

-452.17	-191.79	-250.2	-322.29	-249.27	-318.7	-448.88	-940.32

-448.93	-199.75	-295.93	-365.16	-300.14	-369.53	-513.33	-1097.18

-217.43 NA	-19.46	-5.95 NA	NA	-18.75	-242.3

NA	NA	NA	NA	NA	NA	NA	-364.54

NA	NA	NA	NA	NA	NA	NA	NA NA	NA	NA	NA	NA	NA	NA	NA NA	NA	NA	NA	NA	NA	NA	NA

-42.16	-213.9	-154.94	-226.42	-275.39	-240.91	-259.59 NA

| -449.22 | |  | -89.23 |  | -11.3 |  | -75.1 |  | -63.51 |  | -76.65 | -148.92 | |  | -28.02 |
| --- | --- | --- | --- | --- | --- | --- | --- | --- | --- | --- | --- | --- | --- | --- | --- |
| -449.22 | |  | -89.23 |  | -11.3 |  | -75.1 |  | -63.51 |  | -76.65 | -148.92 | |  | -28.02 |
| -449.22 | |  | -89.23 |  | -11.3 |  | -75.1 |  | -63.51 |  | -76.65 | -148.92 | |  | -28.02 |
|  | -44.23 |  | -66.99 | -107.45 | | -176.42 | | -202.31 | | -199.87 | | -171.93 | | NA |  |
|  | -36.62 |  | -70.03 |  | -80.2 | -133.49 | | -153.08 | | -165.49 | | -115.98 | | NA |  |
|  | -588.6 | -2150.14 | | -4505.29 | | -3876.76 | | -4328.34 | | -4147.93 | | -3612.34 | |  | -40.18 |
| -1196.26 | | -2795.71 | | -5703.38 | | -4580.76 | | -4875.09 | | -4777.25 | | -4252.12 | | -172.81 | |
| -1470.33 | | -4129.05 | | -7825.5 | | -6425.71 | | -7002.06 | | -6758.75 | | -5829.2 | | -182.43 | |
| -1234.47 | | -2850.01 | | -5866.59 | | -5239.61 | | -5568.39 | | -5516.28 | | -4886.63 | | -184.88 | |
| -739.72 | | -1849.6 | | -3564.78 | | -2901.07 | | -3098.44 | | -3002.25 | | -2677.89 | | -165.09 | |
| -800.27 | | -2463.87 | | -4523.05 | | -3602.14 | | -4083.8 | | -3720.03 | | -3145.41 | | -108.83 | |
| -1158.34 | | -3528.54 | | -7649.56 | | -5854.51 | | -6246.13 | | -6084.81 | | -4830.63 | | -160.69 | |
| -1377.93 | | -2706.03 | | -4491.83 | | -3826.83 | | -4157.2 | | -3931.41 | | -3857.02 | | -255.91 | |
| -1638.82 | | -3108.05 | | -5482.63 | | -4566.69 | | -5032.63 | | -4678.17 | | -4428.24 | | -242.68 | |
| -1828.85 | | -3710.7 | | -6528.07 | | -5558.79 | | -5967.6 | | -5708.69 | | -5481.05 | | -254.83 | |
| NA |  | NA |  | NA |  | NA |  | NA |  | NA |  | NA |  | NA |  |
| -126.72 | |  | -11.76 |  | -12.76 |  | -66.09 |  | -31.15 |  | -53.28 | -176.27 | | -462.19 | |
| -1677.69 | | -3732.38 | | -7012.58 | | -5697.86 | | -6271.9 | | -5810.68 | | -5400.52 | | -216.28 | |
| -2011.26 | | -4393.79 | | -7904.88 | | -6485.37 | | -6974.56 | | -6531.96 | | -6363.74 | | -223.37 | |
|  | -269.2 | -393.77 | | -574.44 | | -502.57 | | -538.77 | | -533.45 | | -628.54 | |  | -68.79 |
| -1831.74 | | -5146.6 | | -9751.17 | | -8357.14 | | -9020.15 | | -8603.75 | | -7426.97 | | -192.31 | |
| NA |  | NA |  | NA |  | NA |  | NA |  | NA |  | NA |  | -628.28 | |
| NA |  | NA |  | NA |  | NA |  | NA |  | NA |  | NA |  | NA |  |
| -1972.41 | | -4647.59 | | -8622.31 | | -7190.23 | | -7742.21 | | -7356.37 | | -6818.89 | | -204.64 | |
| -129.73 | | -225.12 | | -296.73 | | -306.63 | |  | -332.3 |  | -332.8 | -316.82 | |  | -13.94 |
| -771.59 | | -2167.22 | | -4451.93 | | -3602.56 | | -3956.19 | | -3765.21 | | -3305.67 | |  | -41.33 |
| -1924.62 | | -4463.54 | | -8044.98 | | -6712.21 | | -7361.97 | | -6899.66 | | -6316.02 | | -245.69 | |
| -1453.85 | | -3253.97 | | -6571.93 | | -5193.01 | | -5591.08 | | -5243.34 | | -5109.26 | | -172.59 | |
| -777.06 | | -1419.93 | | -2485.49 | | -2009.99 | | -2208.68 | | -1980.61 | | -2013.68 | | -189.48 | |
| -777.06 | | -1419.93 | | -2485.49 | | -2009.99 | | -2208.68 | | -1980.61 | | -2013.68 | | -189.48 | |
| -1857.01 | | -4821.05 | | -9087.67 | | -7507.79 | | -8165.74 | | -7735.26 | | -6943.04 | | -202.37 | |
| -956.35 | | -2221.81 | | -3659.23 | | -3248.22 | | -3569.62 | | -3485.35 | | -3156.87 | |  | -83.51 |
| -171.95 | | -1411.71 | | -2520.79 | | -2215.75 | | -2511.96 | | -2438.33 | | -1928.07 | | NA |  |
| -629.41 | | -1489.05 | | -2679.03 | | -2287.64 | | -2491.49 | | -2484.65 | | -2235.1 | | -108.28 | |
| NA |  | NA |  | NA |  | NA |  | NA |  | NA |  | NA |  | NA |  |
| -209.08 | | -134.96 | |  | -62.67 | -137.97 | | -145.08 | | -125.01 | | -214.18 | |  | -243.2 |
| -1786.28 | | -4081.82 | | -8863.26 | | -6899.42 | | -7350.67 | | -7001.33 | | -6560.13 | |  | -156.8 |
| -2019.27 | | -5183.95 | | -9734.32 | | -8159.01 | | -8796.67 | | -8354.81 | | -7467.03 | | -202.56 | |
| -313.01 | | -421.26 | | -633.45 | |  | -519.2 | -607.27 | |  | -604.8 |  | -703.3 |  | -66.83 |

| -210.53 | |  | -17.3 |  | -99.53 |  | -70.84 |  | -25.68 |  | -31.11 |  | -62.7 |  | -13.35 |
| --- | --- | --- | --- | --- | --- | --- | --- | --- | --- | --- | --- | --- | --- | --- | --- |
| -249.78 | |  | -24.71 | -151.27 | | -103.26 | |  | -49.65 |  | -70.2 |  | -71.86 |  | -50.48 |
| -336.22 | |  | -55.08 | -180.92 | | -143.22 | |  | -93.96 |  | -99.55 | -159.14 | |  | -53.55 |
|  | -76.49 | NA |  |  | -10.53 |  | -5.92 | NA |  | NA |  |  | -11.27 | NA |  |
|  | -11.16 | NA |  | NA |  | NA |  | NA |  | NA |  | NA |  | -2054.11 | |
| NA |  | NA |  | NA |  | NA |  | NA |  | NA |  | NA |  | -1085.81 | |
|  | -10.4 | NA |  | NA |  | NA |  | NA |  | NA |  | NA |  | -1232.67 | |
|  | -13.58 | NA |  | NA |  | NA |  | NA |  | NA |  | NA |  | -680.64 | |
|  | -61.4 | NA |  |  | -10.63 | NA |  | NA |  | NA |  | NA |  | -1237.55 | |
|  | -54.88 | NA |  |  | -7.23 | NA |  | NA |  | NA |  | NA |  | -1021.79 | |
| NA |  | NA |  | NA |  | NA |  | NA |  | NA |  | NA |  | -435.24 | |
| NA |  | NA |  | NA |  | NA |  | NA |  | NA |  | NA |  | -435.24 | |
| NA |  | NA |  | NA |  | NA |  | NA |  | NA |  | NA |  | -435.24 | |
| NA |  | NA |  | NA |  | NA |  | NA |  | NA |  | NA |  | -1063.74 | |
|  | -70.14 | NA |  |  | -13.86 | NA |  | NA |  | NA |  | NA |  | -1054.72 | |
| -123.34 | | NA |  |  | -30.24 |  | -7.41 |  | -7.42 |  | -9.94 |  | -17.51 | -1371.16 | |
|  | -33.35 | NA |  | NA |  | NA |  | NA |  | NA |  | NA |  |  | -478.2 |
|  | -21.15 | NA |  | NA |  | NA |  | NA |  | NA |  | NA |  | -631.08 | |
| -128.74 | | NA |  |  | -74.6 |  | -33.71 |  | -43.05 |  | -47.4 |  | -49.07 | -783.76 | |
|  | -72.48 | NA |  |  | -21.65 | NA |  | NA |  |  | -6.18 |  | -10.82 | -1150.72 | |
| NA |  | NA |  | NA |  | NA |  | NA |  | NA |  | NA |  |  | -64.18 |
| -1683.95 | | -4093.27 | | -7006.12 | | -6026.04 | | -6493.48 | | -6208.13 | | -5566.1 | | -237.54 | |
| -613.22 | |  | -24.98 | -339.37 | |  | -170.5 | -142.68 | | -374.58 | |  | -134 | -362.61 | |
| -613.22 | |  | -24.98 | -339.37 | |  | -170.5 | -142.68 | | -374.58 | |  | -134 | -362.61 | |
|  | -63.02 | NA |  |  | -81.96 | NA |  | NA |  |  | -27.44 | NA |  |  | -79.87 |
|  | -63.02 | NA |  |  | -81.96 | NA |  | NA |  |  | -27.44 | NA |  |  | -79.87 |
| -560.53 | | NA |  | -163.31 | |  | -43.62 |  | -19.13 | -172.67 | |  | -10.09 | -173.32 | |
| -1260.12 | |  | -22.1 | -474.57 | |  | -241.4 | -177.24 | | -564.05 | | -116.12 | |  | -488 |
| -174.38 | | NA |  |  | -36.72 | NA |  | NA |  |  | -25.85 | NA |  | -141.03 | |
| -278.34 | | NA |  |  | -51.19 | NA |  | NA |  |  | -63.05 | NA |  |  | -73.74 |
| -278.34 | | NA |  |  | -51.19 | NA |  | NA |  |  | -63.05 | NA |  |  | -73.74 |
| -278.34 | | NA |  |  | -51.19 | NA |  | NA |  |  | -63.05 | NA |  |  | -73.74 |
| NA |  | NA |  | NA |  | NA |  | NA |  | NA |  | NA |  | NA |  |
| NA |  | NA |  | NA |  | NA |  | NA |  | NA |  | NA |  | NA |  |
| NA |  | NA |  | NA |  | NA |  | NA |  | NA |  | NA |  | NA |  |
| NA |  | NA |  | NA |  | NA |  | NA |  | NA |  | NA |  | NA |  |
| -471.54 | |  | -36.33 |  | -17.11 |  | -63.18 |  | -36.02 |  | -60.44 | -117.44 | |  | -191.7 |
| -128.01 | |  | -62.65 |  | -12.01 |  | -59.26 |  | -46.35 |  | -68.42 | -163.42 | | -157.58 | |
| -128.01 | |  | -62.65 |  | -12.01 |  | -59.26 |  | -46.35 |  | -68.42 | -163.42 | | -157.58 | |

| -147.25 | | -124.95 | |  | -47.86 | -114.64 | |  | -91.49 |  | -86.68 |  | -208.2 | -341.47 | |
| --- | --- | --- | --- | --- | --- | --- | --- | --- | --- | --- | --- | --- | --- | --- | --- |
| NA |  | NA |  | NA |  | NA |  | NA |  | NA |  | NA |  |  | -47.77 |
|  | -106.8 | -106.95 | |  | -91.64 | -185.81 | | -147.13 | | -133.75 | | -240.55 | | -196.25 | |
|  | -66.12 | NA |  | NA |  |  | -6.78 | NA |  | NA |  |  | -26.35 |  | -54.35 |
| -163.22 | | -227.58 | | -220.97 | | -301.47 | | -288.23 | | -293.29 | | -366.49 | | -132.33 | |
| NA |  | NA |  | NA |  | NA |  | NA |  | NA |  | NA |  | -390.47 | |
| NA |  | NA |  | NA |  | NA |  | NA |  | NA |  | NA |  | -189.56 | |
| NA |  | NA |  | NA |  | NA |  | NA |  | NA |  | NA |  | -230.61 | |
| -165.17 | | NA |  | NA |  | NA |  | NA |  | NA |  |  | -9.93 | -3721.35 | |
| -104.66 | | NA |  | NA |  | NA |  | NA |  | NA |  |  | -22.36 | -3841.05 | |
|  | -297.1 |  | -27.31 |  | -9.05 |  | -40.42 |  | -22.53 |  | -39.12 |  | -64.72 | -3150.44 | |
| NA |  | NA |  | NA |  | NA |  | NA |  | NA |  | NA |  | NA |  |
| NA |  | NA |  | NA |  | NA |  | NA |  | NA |  | NA |  | NA |  |
| NA |  | NA |  | NA |  | NA |  | NA |  | NA |  | NA |  | NA |  |
| NA |  | NA |  | NA |  | NA |  | NA |  | NA |  | NA |  | NA |  |
| NA |  | NA |  | NA |  | NA |  | NA |  | NA |  | NA |  | NA |  |
| NA |  | NA |  | NA |  | NA |  | NA |  | NA |  | NA |  | NA |  |
| NA |  | NA |  | NA |  | NA |  | NA |  | NA |  | NA |  | NA |  |
| NA |  | NA |  | NA |  | NA |  | NA |  | NA |  | NA |  | NA |  |
| NA |  | NA |  |  | -183 |  | -76.49 |  | -59.36 |  | -38.85 |  | -15.2 | NA |  |
| NA |  | NA |  | NA |  | NA |  | NA |  | NA |  | NA |  | NA |  |
| NA |  | NA |  | NA |  | NA |  | NA |  | NA |  | NA |  | NA |  |
| NA |  | NA |  | NA |  | NA |  | NA |  | NA |  | NA |  | NA |  |
| NA |  | NA |  | NA |  | NA |  | NA |  | NA |  | NA |  | NA |  |
| NA |  | NA |  | NA |  | NA |  | NA |  | NA |  | NA |  | NA |  |
| NA |  | NA |  | -163.15 | |  | -65.31 |  | -35.32 |  | -26.26 |  | -13.39 | NA |  |
| NA |  | NA |  | NA |  | NA |  | NA |  | NA |  | NA |  | NA |  |
| NA |  | NA |  |  | -14.37 | NA |  | NA |  | NA |  | NA |  | NA |  |
| NA |  | NA |  | NA |  | NA |  | NA |  | NA |  | NA |  | NA |  |
| NA |  | NA |  | NA |  | NA |  | NA |  | NA |  | NA |  | NA |  |
|  | -33.45 | NA |  |  | -72.95 |  | -38.18 |  | -26.73 |  | -37.25 |  | -29.08 |  | -53.9 |
| NA |  | NA |  | NA |  | NA |  | NA |  | NA |  | NA |  | NA |  |
| NA |  | NA |  |  | -15.95 | NA |  | NA |  | NA |  | NA |  | NA |  |
| NA |  | NA |  | NA |  | NA |  | NA |  | NA |  | NA |  | NA |  |
| NA |  | NA |  | NA |  | NA |  | NA |  | NA |  | NA |  | NA |  |
| NA |  | NA |  |  | -47.52 | NA |  | NA |  | NA |  | NA |  | NA |  |
| NA |  | NA |  | NA |  | NA |  | NA |  | NA |  | NA |  | NA |  |
| NA |  | NA |  |  | -44.58 | NA |  | NA |  | NA |  | NA |  | NA |  |
| NA |  | NA |  |  | -12.16 | NA |  | NA |  | NA |  | NA |  | NA |  |

| NA |  | NA |  | NA |  | NA |  | NA |  | NA |  | NA |  | NA |  |
| --- | --- | --- | --- | --- | --- | --- | --- | --- | --- | --- | --- | --- | --- | --- | --- |
| NA |  |  | -6.72 | -226.51 | | -124.48 | |  | -80.69 |  | -68.07 |  | -28.19 | NA |  |
| NA |  | NA |  | NA |  | NA |  | NA |  | NA |  | NA |  | NA |  |
| NA |  | NA |  | NA |  | NA |  | NA |  | NA |  | NA |  | NA |  |
| NA |  | NA |  | NA |  | NA |  | NA |  | NA |  | NA |  | NA |  |
| NA |  | NA |  |  | -70.4 | NA |  |  | -8.78 | NA |  | NA |  | NA |  |
| NA |  | NA |  | NA |  | NA |  | NA |  | NA |  | NA |  | NA |  |
| NA |  | NA |  |  | -12.35 | NA |  | NA |  | NA |  | NA |  | NA |  |
| NA |  | NA |  | NA |  | NA |  | NA |  | NA |  | NA |  | NA |  |
| NA |  | NA |  |  | -53.07 | NA |  | NA |  | NA |  | NA |  | NA |  |
| -233.48 | |  | -511.2 | -1676.44 | | -1255.33 | | -1292.62 | | -1277.87 | | -957.12 | |  | -41.71 |
|  | -327.4 | NA |  | -394.61 | |  | -7.8 | NA |  |  | -6.52 | -365.09 | | -525.82 | |
| NA |  | NA |  | -208.05 | |  | -15.28 | NA |  |  | -8.02 |  | -8.03 | NA |  |
|  | -68.47 |  | -97.98 | -799.81 | | -349.79 | | -332.72 | | -324.78 | | -306.43 | |  | -64.36 |
|  | -93.07 |  | -109.2 | -270.96 | | -162.77 | | -129.76 | | -111.25 | | -105.82 | | -217.64 | |
|  | -47.69 |  | -58.76 |  | -228.6 | -102.77 | |  | -75.9 |  | -72.39 |  | -91.04 |  | -156.4 |
| NA |  | NA |  | NA |  | NA |  | NA |  | NA |  | NA |  |  | -81.57 |
|  | -40.14 | NA |  | -231.84 | |  | -44.39 |  | -18.58 |  | -20.12 |  | -40.07 |  | -89.52 |
| -124.49 | | -165.53 | | -661.61 | | -351.75 | |  | -293.1 | -273.26 | | -255.44 | |  | -197 |
|  | -6.79 | NA |  | -126.76 | |  | -43.08 |  | -22.65 |  | -18.47 |  | -25.29 |  | -97.08 |
| -193.34 | |  | -7.82 |  | -52.45 |  | -23.87 |  | -12.44 | NA |  |  | -28.01 | NA |  |
| -128.49 | |  | -9.38 |  | -47.16 |  | -31.87 |  | -5.96 |  | -10.18 |  | -32.45 | NA |  |
| -197.89 | |  | -24.85 |  | -113.9 |  | -69.59 |  | -30.68 |  | -38.73 |  | -65.1 |  | -29.49 |
|  | -62.03 | NA |  |  | -11.39 | NA |  | NA |  | NA |  | NA |  | NA |  |
| NA |  | NA |  | NA |  |  | -34.88 |  | -21.35 |  | -36.71 |  | -70.14 | NA |  |
|  | -80.9 |  | -21.71 |  | -69.55 |  | -61.38 |  | -50.76 |  | -63.78 | -108.78 | |  | -32.69 |
| NA |  |  | -18.94 | NA |  |  | -30.36 |  | -18.48 |  | -23.6 |  | -55.52 | NA |  |
| NA |  |  | -33.86 |  | -15.34 |  | -40.17 |  | -28.73 |  | -55.78 |  | -42.82 |  | -8.99 |
| NA |  |  | -33.86 |  | -15.34 |  | -40.17 |  | -28.73 |  | -55.78 |  | -42.82 |  | -8.99 |
| NA |  |  | -7.27 | NA |  |  | -22.33 |  | -33.67 |  | -36.88 |  | -35.85 | NA |  |
| NA |  |  | -59.79 |  | -26.58 | -162.92 | | -188.36 | | -196.44 | | -140.77 | | NA |  |
| NA |  | NA |  | NA |  |  | -35.58 |  | -24.79 |  | -53.66 |  | -72.9 | NA |  |
| NA |  | NA |  | NA |  |  | -35.58 |  | -24.79 |  | -53.66 |  | -72.9 | NA |  |
| NA |  | NA |  | NA |  |  | -35.58 |  | -24.79 |  | -53.66 |  | -72.9 | NA |  |
|  | -27.41 |  | -74.89 |  | -64.59 | -213.36 | | -216.43 | | -238.51 | | -218.82 | | NA |  |
| -128.87 | | -266.01 | | -140.13 | | -335.89 | | -406.17 | | -376.68 | | -293.51 | | NA |  |
| NA |  | NA |  | NA |  | NA |  | NA |  | NA |  |  | -21.44 | NA |  |
| NA |  | NA |  | NA |  | NA |  | NA |  | NA |  | NA |  | NA |  |
| NA |  | NA |  |  | -47.86 | NA |  |  | -11.74 | NA |  |  | -16.05 | -424.89 | |

| NA |  | NA |  | NA |  | NA |  | NA |  | NA |  | NA |  | NA |  |
| --- | --- | --- | --- | --- | --- | --- | --- | --- | --- | --- | --- | --- | --- | --- | --- |
| NA |  | NA |  | NA |  | NA |  | NA |  | NA |  | NA |  | NA |  |
| NA |  | NA |  | NA |  | NA |  | NA |  | NA |  | NA |  | NA |  |
| NA |  | NA |  | NA |  | NA |  | NA |  | NA |  | NA |  | NA |  |
| -507.48 | | -366.58 | | -128.22 | | -248.87 | | -282.18 | | -239.06 | | -271.02 | | -150.82 | |
| -541.77 | | -202.88 | | -210.08 | | -264.57 | | -227.37 | | -278.47 | | -255.17 | | -464.01 | |
| -155.63 | |  | -95.48 |  | -24.75 |  | -87.11 |  | -84.63 |  | -109.7 | -129.51 | | -157.72 | |
| -686.92 | | -363.01 | | -334.85 | | -420.07 | | -385.11 | | -397.89 | | -408.76 | | -288.01 | |
|  | -21.88 |  | -49.14 |  | -9.25 |  | -41 |  | -13.32 |  | -30.93 |  | -52.46 |  | -24.3 |
|  | -59.91 |  | -94.72 |  | -57.41 |  | -73.13 |  | -49.76 |  | -66.73 |  | -81.42 |  | -88.31 |
| -187.18 | |  | -92.6 | -104.83 | | -218.04 | | -132.33 | | -162.95 | | -306.67 | | -499.44 | |
| -227.29 | | -375.27 | | -213.59 | | -222.77 | | -273.14 | | -233.09 | | -288.09 | |  | -81 |
| -484.21 | | -394.51 | | -344.08 | | -370.34 | | -381.13 | | -405.72 | | -464.21 | |  | -181.5 |
| -234.65 | |  | -31.01 |  | -47.75 |  | -42.66 |  | -35.09 |  | -32.32 |  | -68.25 | -174.98 | |
| NA |  | NA |  | NA |  | NA |  | NA |  | NA |  | NA |  | NA |  |
| NA |  | NA |  | NA |  | NA |  | NA |  | NA |  | NA |  | NA |  |
|  | -56.3 |  | -32.24 |  | -52.95 |  | -102.1 |  | -66.43 |  | -79.14 | -191.45 | | -657.98 | |
| NA |  | NA |  | NA |  | NA |  | NA |  | NA |  | NA |  | NA |  |
|  | -532.4 | -248.57 | | -229.94 | | -286.91 | | -302.58 | | -210.74 | |  | -312.7 | -313.73 | |
| NA |  | NA |  | NA |  | NA |  | NA |  | NA |  | NA |  | NA |  |
| NA |  | NA |  | NA |  | NA |  | NA |  | NA |  | NA |  | NA |  |
| -163.39 | | -110.34 | | -119.51 | | -152.75 | |  | -146.1 | -130.98 | |  | -161.6 | -287.05 | |
| -288.95 | |  | -86.25 | -132.01 | | -206.96 | | -144.56 | | -160.84 | | -401.05 | | -660.77 | |
| -288.95 | |  | -86.25 | -132.01 | | -206.96 | | -144.56 | | -160.84 | | -401.05 | | -660.77 | |
| -288.95 | |  | -86.25 | -132.01 | | -206.96 | | -144.56 | | -160.84 | | -401.05 | | -660.77 | |
| -288.95 | |  | -86.25 | -132.01 | | -206.96 | | -144.56 | | -160.84 | | -401.05 | | -660.77 | |
| -417.47 | | -259.78 | | -424.36 | | -400.02 | | -409.55 | | -469.14 | | -369.32 | | -226.26 | |
| -417.47 | | -259.78 | | -424.36 | | -400.02 | | -409.55 | | -469.14 | | -369.32 | | -226.26 | |
| -417.47 | | -259.78 | | -424.36 | | -400.02 | | -409.55 | | -469.14 | | -369.32 | | -226.26 | |
| -417.47 | | -259.78 | | -424.36 | | -400.02 | | -409.55 | | -469.14 | | -369.32 | | -226.26 | |
| -267.04 | |  | -91.8 |  | -397.6 | -203.18 | | -209.55 | | -251.27 | | -318.33 | | -1088.72 | |
| -267.04 | |  | -91.8 |  | -397.6 | -203.18 | | -209.55 | | -251.27 | | -318.33 | | -1088.72 | |
| -112.73 | |  | -72.54 |  | -258.8 | -139.96 | | -174.72 | |  | -139.5 | -166.32 | | -362.31 | |
| -112.73 | |  | -72.54 |  | -258.8 | -139.96 | | -174.72 | |  | -139.5 | -166.32 | | -362.31 | |
| -398.49 | | -203.46 | | -282.56 | |  | -380.7 |  | -285.8 | -343.12 | | -541.43 | | -980.21 | |
| -534.11 | | -372.93 | | -155.89 | |  | -279.6 | -353.23 | | -227.27 | | -317.48 | | -232.76 | |
| -534.11 | | -372.93 | | -155.89 | |  | -279.6 | -353.23 | | -227.27 | | -317.48 | | -232.76 | |
| -483.79 | | -263.13 | | -283.32 | |  | -296.9 |  | -298 | -237.07 | | -287.91 | | -337.85 | |
| -483.79 | | -263.13 | | -283.32 | |  | -296.9 |  | -298 | -237.07 | | -287.91 | | -337.85 | |

| -250.58 | | -282.87 | | -127.74 | | -250.41 | |  | -301.3 | -191.64 | | -285.59 | | -207.46 | |
| --- | --- | --- | --- | --- | --- | --- | --- | --- | --- | --- | --- | --- | --- | --- | --- |
| -755.61 | | -492.21 | | -258.47 | | -453.74 | | -505.01 | | -386.99 | | -450.19 | | -346.07 | |
| -432.39 | | -230.44 | | -107.34 | | -220.32 | | -254.51 | | -161.34 | | -307.19 | | -300.13 | |
| -144.07 | |  | -30.78 |  | -39.45 |  | -66.76 |  | -58.8 |  | -52.53 |  | -76.21 |  | -21.38 |
| -245.23 | |  | -88.32 | -291.43 | | -302.77 | | -113.83 | | -224.86 | | -201.73 | | -104.37 | |
| -146.65 | | NA |  |  | -32.91 |  | -9.38 | NA |  | NA |  |  | -19.77 | NA |  |
| -221.08 | |  | -122.1 | -254.09 | | -322.24 | | -145.56 | | -221.88 | | -206.62 | | -100.97 | |
| -3500.29 | |  | -35.41 |  | -89.74 | -320.25 | |  | -98.07 | -129.64 | | -535.77 | | -1393.66 | |
| -3992.69 | |  | -60.69 | -105.02 | | -401.11 | | -139.75 | |  | -189.8 | -629.58 | | -1614.98 | |
| -610.45 | | NA |  | NA |  | NA |  | NA |  | NA |  |  | -21.99 | -407.86 | |
| -4243.45 | |  | -52.32 | -111.68 | | -417.31 | | -125.77 | | -179.62 | | -663.95 | | -1770.16 | |
| -1908.16 | |  | -49.6 | -106.28 | | -201.19 | |  | -83.07 | -110.19 | |  | -245.4 | -1318.43 | |
| NA |  | NA |  | NA |  | NA |  | NA |  | NA |  |  | -6.34 | NA |  |
| -116.23 | | -109.96 | | -101.62 | | -204.35 | | -185.99 | | -188.92 | | -218.86 | | NA |  |
| NA |  | NA |  | NA |  | NA |  | NA |  | NA |  | NA |  | NA |  |
| NA |  | NA |  | NA |  | NA |  | NA |  | NA |  | NA |  | NA |  |
| NA |  | NA |  | NA |  | NA |  | NA |  | NA |  | NA |  | NA |  |
|  | -46.42 | NA |  |  | -25.12 |  | -17.31 |  | -10.47 |  | -12.36 |  | -18.85 | NA |  |
| NA |  | NA |  | NA |  | NA |  | NA |  | NA |  | NA |  | NA |  |
| NA |  | NA |  | NA |  | NA |  | NA |  | NA |  | NA |  | NA |  |
| NA |  | NA |  | NA |  | NA |  | NA |  | NA |  | NA |  | NA |  |
| NA |  | NA |  | NA |  | NA |  | NA |  | NA |  | NA |  | NA |  |
|  | -8.32 | NA |  | NA |  | NA |  | NA |  | NA |  | NA |  | NA |  |
| NA |  | NA |  |  | -56.92 |  | -37.05 |  | -19.39 |  | -22.68 |  | -18.34 | NA |  |
|  | -19.72 |  | -51.86 | -205.82 | | -178.29 | | -165.51 | | -165.81 | | -114.69 | | NA |  |
| NA |  | NA |  | NA |  | NA |  | NA |  | NA |  | NA |  | NA |  |
| NA |  | NA |  | NA |  | NA |  | NA |  | NA |  | NA |  | NA |  |
| NA |  | NA |  | NA |  | NA |  | NA |  | NA |  | NA |  | NA |  |
| -419.67 | | -150.89 | | -272.46 | | -378.42 | | -273.82 | | -333.43 | | -558.24 | | -1071.34 | |
| -169.62 | | -156.31 | | -268.63 | | -346.73 | | -404.85 | | -418.01 | |  | -349.2 |  | -54.92 |
| -169.62 | | -156.31 | | -268.63 | | -346.73 | | -404.85 | | -418.01 | |  | -349.2 |  | -54.92 |
| NA |  | NA |  | NA |  | NA |  | NA |  | NA |  | NA |  | -660.69 | |
|  | -5.17 | NA |  | NA |  | NA |  | NA |  | NA |  |  | -14.35 | -546.65 | |
|  | -19.79 | NA |  | NA |  | NA |  | NA |  | NA |  | NA |  | -1005.85 | |
| NA |  | NA |  | NA |  |  | -8.72 |  | -8.68 |  | -11.64 |  | -51.02 | -1611.41 | |
| -248.25 | | -665.78 | |  | -687.5 | -768.34 | | -803.88 | | -782.97 | | -830.05 | |  | -40.13 |
|  | -55.69 | NA |  | NA |  | NA |  | NA |  | NA |  |  | -15.13 | -2747.8 | |
| NA |  | NA |  | NA |  | NA |  | NA |  | NA |  | NA |  | -473.22 | |
|  | -60.38 | NA |  | NA |  | NA |  | NA |  | NA |  |  | -25.64 | -2053.23 | |

-41.76 NA	-6.98	-15.18	-15.15	-11.65	-33.14	-1748.57

-77.78 NA	NA	NA	NA	NA	NA	-823.25

-49.13 NA	NA	NA	NA	NA	NA	-1033.54

-28.69 NA	NA	NA	NA	NA	NA	-1063.29

-76.97 NA	NA	NA	NA	NA	NA	-547.11

-619.2	-207.27	-256.27	-274.55	-242.18	-307.96	-242.86	-509.62

NA	NA	NA	NA	NA	NA	NA	-437.26

NA	NA	NA	NA	NA	NA	NA	-437.26

NA	NA	NA	NA	NA	NA	NA	-437.26

NA	NA	NA	NA	NA	NA	NA	NA

-31.44	-26.61	-26.23	-26.36	-22.13	-33	-21.43	-37.86

-21.58	-8.71 NA	NA	NA	-7.13	-10.48	-98.66

-268.4	-90.73	-152.4	-148.95	-97.86	-142.6	-160.23	-317.24

NA	NA	NA	NA	NA	NA	NA	NA

-7.74	-13.33 NA	NA	NA	NA	-17.71	-13.82

-8.22	-6.5	-33.48	-12.32	-6.07	-14.2	-20.15	-42.28

-89.7	-9.49	-12.98	-19.45	-11.28	-20.68	-35.68	-106.89

-32.78	-5.42 NA	NA	NA	NA	-18.15	-19.4

NA	NA	NA	NA	NA	NA	NA	NA

-9.42	-213.76	-5.32	-175.48	-169.04	-192.45	-143.9 NA

| -104.81 | | -295.35 | -98.66 | | -327.81 | -350.88 | -355.93 | | -282.94 | | -11.92 | |
| --- | --- | --- | --- | --- | --- | --- | --- | --- | --- | --- | --- | --- |
| -104.54 | | -27.48 | -25.26 | | -60.44 | -56.04 | -57.24 | | -118.94 | | -1028.17 | |
| -74 | | -16.79 | -13.63 | | -48.37 | -42.2 | -50.88 | | -91.94 | | -1980.76 | |
| -79.79 | | -404.08 | -467.91 | | -465.35 | -415.48 | -460.23 | | -504.99 | | -59.05 | |
| -284.02 | | -140.17 | -115.01 | | -176.02 | -168.46 | -161.04 | | -224.58 | | -185.84 | |
| -26.8 | | -9.07 | NA | | -10.18 | -16.21 | -8 | | -31.22 | | -334.31 | |
| -132.69 | | -125.41 | -143.6 | | -202.74 | -191.57 | -196.42 | | -249.77 | | -6 | |
| -99.26 | | -25.53 | -19.12 | | -60 | -51.44 | -66.53 | | -90.44 | | -38.1 | |
| -320.68 | | -164.84 | -169.26 | | -219.03 | -168.67 | -183.42 | | -206.17 | | -216.21 | |
| -159.39 | | -37.35 | -8.47 | | -18.54 | -14.51 | -22.71 | | -43.28 | | -33.45 | |
| -172.95 | | -26.83 | -67.11 | | -68.58 | -52.25 | -59.86 | | -73.93 | | -116.61 | |
| -205.98 | | -51.85 | -44.18 | | -60.71 | -40.7 | -48.94 | | -72.72 | | -73.39 | |
| -232.68 | | -73.64 | -103.22 | | -105.76 | -101.63 | -96.67 | | -105.78 | | -151.12 | |
| -222.38 | | -66.94 | -69.6 | | -99.78 | -96.72 | -103.2 | | -144.71 | | -67.28 | |
| -199.64 | | -29.22 | -54.65 | | -49.59 | -39.86 | -47.14 | | -50.23 | | -88.63 | |
|  | -65.26 NA | |  | -11.91 NA | | NA |  | -8.21 |  | -6.36 |  | -10.9 |
| NA | NA | | NA | NA | | NA | NA |  | NA |  | NA |  |
| NA | NA | | NA | NA | | NA | NA |  | NA |  | NA |  |
| NA | NA | | NA | NA | | NA | NA |  | NA |  | NA |  |

| NA |  | -623.39 | | -1562.91 | | -1067.19 | | -862.56 | | -951.34 | | -587.51 | | NA |  |
| --- | --- | --- | --- | --- | --- | --- | --- | --- | --- | --- | --- | --- | --- | --- | --- |
| NA |  | -795.85 | | -1928.6 | | -1386.77 | | -1155.96 | | -1262.46 | |  | -784.9 | NA |  |
| NA |  | -492.66 | | -913.81 | | -732.23 | | -638.69 | | -641.61 | | -382.77 | | NA |  |
|  | -37.16 | NA |  | NA |  |  | -21.57 |  | -14.13 |  | -18.14 |  | -60.31 | -2097.99 | |
|  | -10.81 | NA |  | NA |  | NA |  | NA |  | NA |  | NA |  | -1299.82 | |
|  | -10.14 | NA |  | NA |  | NA |  | NA |  | NA |  | NA |  | -1712.76 | |
|  | -8 | NA |  | NA |  | NA |  | NA |  | NA |  | NA |  | -1980.28 | |
|  | -60.64 | NA |  |  | -26.66 |  | -6.68 |  | -10.04 |  | -13.11 |  | -18.77 | -125.75 | |
| -210.49 | | -103.74 | |  | -59.84 | -129.97 | | -125.41 | | -156.51 | | -177.57 | | -126.65 | |
| -321.77 | |  | -86.26 |  | -49.67 | -119.83 | |  | -94.2 | -122.95 | | -138.17 | | -237.06 | |
| -619.09 | | -108.22 | |  | -62.3 | -112.48 | |  | -97.44 | -114.02 | | -156.58 | | -274.61 | |
| -619.09 | | -108.22 | |  | -62.3 | -112.48 | |  | -97.44 | -114.02 | | -156.58 | | -274.61 | |
| -475.46 | | -142.34 | |  | -96.16 | -131.64 | | -122.81 | |  | -131.2 | -174.57 | | -270.85 | |
|  | -72.52 | NA |  |  | -17.73 |  | -7.99 | NA |  | NA |  |  | -24.77 | NA |  |
|  | -72.52 | NA |  |  | -17.73 |  | -7.99 | NA |  | NA |  |  | -24.77 | NA |  |
|  | -14.92 | NA |  |  | -28.32 | NA |  | NA |  | NA |  |  | -9.62 | -129.64 | |
| NA |  | NA |  |  | -31.21 | NA |  | NA |  | NA |  |  | -20.52 |  | -62.86 |
|  | -10.49 | NA |  |  | -11.22 | NA |  | NA |  | NA |  |  | -36.2 |  | -86.39 |
| NA |  |  | -32.74 |  | -10.25 | -100.48 | | -118.19 | |  | -130.8 | -124.63 | | NA |  |
| -939.89 | | -1562.54 | | -2778.53 | | -2303.99 | | -2470.43 | | -2388.79 | | -2335.35 | | -196.45 | |
| -118.64 | | -2299.13 | | -6077.13 | | -4018.57 | | -4159.16 | | -3934.68 | | -2359.32 | |  | -36.88 |
|  | -50.82 | -650.36 | | -2410.71 | | -1600.62 | | -1742.03 | | -1833.29 | | -1135.1 | | NA |  |
| -485.56 | | -1691.82 | | -3678.61 | | -2911.09 | | -3068.49 | | -3033.99 | | -2222.89 | |  | -90.77 |
| -307.33 | | -3308.28 | | -7960.78 | | -5417.22 | | -5757.91 | | -5539.56 | | -3379.14 | |  | -68.19 |
| -209.38 | | -2050.75 | | -5767.07 | | -3915.65 | | -4235.58 | | -4005.93 | | -2727.93 | |  | -60.11 |
| -453.61 | | -110.93 | | -374.54 | | -194.03 | | -185.88 | | -224.24 | | -249.66 | | -181.21 | |
|  | -98.45 | -108.46 | |  | -849.6 |  | -338 | -305.82 | | -293.48 | | -276.72 | |  | -121.9 |
| -518.45 | |  | -100.9 | -396.79 | | -249.36 | | -203.25 | | -262.86 | | -294.02 | | -233.39 | |
|  | -52.75 |  | -16.98 |  | -25.3 |  | -30.94 |  | -35.31 |  | -30.33 |  | -58.93 | -105.11 | |
| -449.51 | | -375.44 | | -186.94 | | -358.83 | | -411.46 | |  | -309.8 |  | -368.6 | -253.46 | |
| -700.07 | | -304.38 | | -350.45 | | -341.31 | | -365.57 | | -296.63 | |  | -318.6 |  | -316.3 |
| NA |  |  | -7.56 | NA |  | NA |  |  | -5.12 | NA |  |  | -14.94 |  | -18.84 |
| NA |  | NA |  |  | -21.48 | NA |  | NA |  | NA |  | NA |  | -461.23 | |
| -307.31 | | NA |  | NA |  | NA |  | NA |  | NA |  | NA |  |  | -71.96 |
|  | -232.2 | NA |  | NA |  | NA |  | NA |  | NA |  | NA |  |  | -18.72 |
|  | -37.92 | -104.08 | | NA |  |  | -45.28 |  | -48.39 |  | -62.32 |  | -75.21 | NA |  |
|  | -255.1 |  | -242.4 |  | -41.79 | -186.78 | | -212.17 | | -215.55 | | -228.59 | |  | -89.42 |
| -116.66 | | -257.11 | |  | -15.76 | -184.88 | | -162.95 | | -154.63 | | -160.98 | |  | -8.35 |
| NA |  | NA |  | NA |  | NA |  | NA |  | NA |  | NA |  | NA |  |

| -168.18 | | -226.24 | |  | -27.74 | -157.35 | |  | -185.1 | -182.02 | | -199.85 | |  | -43.79 |
| --- | --- | --- | --- | --- | --- | --- | --- | --- | --- | --- | --- | --- | --- | --- | --- |
| -840.24 | | -465.93 | | -241.34 | | -452.34 | | -466.45 | |  | -393.9 |  | -457.1 | -336.58 | |
|  | -135.2 |  | -50.34 |  | -78.68 | -142.66 | |  | -82.17 | -109.52 | | -213.48 | | -539.26 | |
| -138.31 | |  | -48.07 |  | -66.88 | -132.26 | |  | -73.79 |  | -78.33 | -177.51 | | -402.95 | |
|  | -73.9 |  | -21.26 |  | -21.03 |  | -62.79 |  | -36.64 |  | -41.03 |  | -97.38 | -230.13 | |
|  | -152 |  | -43.35 |  | -48.29 |  | -49.71 |  | -39.48 |  | -29.96 |  | -47.21 | -145.43 | |
| -729.66 | | NA |  | NA |  |  | -10.38 | NA |  | NA |  |  | -55.33 | -284.57 | |
|  | -140.7 |  | -56.87 |  | -51.64 | -141.03 | |  | -74.38 |  | -89.72 | -189.43 | | -423.79 | |
|  | -73.76 |  | -28.48 |  | -9.52 |  | -72.15 |  | -29.06 |  | -35.63 | -123.29 | | -278.74 | |
| NA |  | NA |  | NA |  | NA |  | NA |  | NA |  | NA |  | NA |  |
| NA |  | NA |  | NA |  | NA |  | NA |  | NA |  | NA |  | NA |  |
| NA |  | NA |  | NA |  | NA |  | NA |  | NA |  | NA |  | NA |  |
| -186.02 | | -539.61 | |  | -586.9 | -648.65 | | -586.26 | | -647.65 | | -605.25 | |  | -61.01 |
| -163.98 | |  | -264.1 | -124.79 | | -223.37 | | -231.66 | |  | -237.1 | -303.42 | | -184.81 | |
|  | -40.76 |  | -26.37 | NA |  |  | -8.65 |  | -14.6 |  | -10.27 |  | -14.37 |  | -8.91 |
| -177.93 | |  | -125.4 | -112.82 | | -147.73 | |  | -133.1 | -189.34 | | -266.98 | |  | -95.83 |
|  | -42.13 |  | -18.41 | NA |  |  | -5.08 |  | -13.87 | NA |  |  | -8.88 | NA |  |
| NA |  |  | -8.88 | NA |  |  | -12.51 |  | -9.41 |  | -12.66 |  | -17.52 | NA |  |
|  | -52.48 | -112.62 | | NA |  |  | -84.98 |  | -84.21 |  | -86.34 |  | -89.47 |  | -42.31 |
| NA |  | NA |  | NA |  | NA |  | NA |  | NA |  | NA |  | NA |  |
| -652.07 | | -417.79 | | -374.42 | | -397.12 | | -351.11 | | -427.48 | | -454.65 | | -591.63 | |
| -170.94 | |  | -5.92 |  | -10.77 |  | -18.66 |  | -19.14 |  | -22.45 |  | -70.5 | -170.19 | |
| NA |  | NA |  | NA |  | NA |  | NA |  | NA |  | NA |  | NA |  |
| NA |  | NA |  | NA |  | NA |  | NA |  | NA |  | NA |  | NA |  |
| NA |  | -183.75 | |  | -31.71 | -139.69 | |  | -155.7 | -158.03 | | -143.45 | | NA |  |
|  | -42.53 | -121.95 | |  | -94.94 | -276.53 | | -299.04 | | -286.68 | | -213.58 | | NA |  |
|  | -70.04 | -231.69 | |  | -91.72 | -254.84 | | -248.31 | | -263.18 | | -195.92 | |  | -7.34 |
|  | -51.19 | -204.33 | |  | -34.69 | -127.46 | | -147.29 | | -125.26 | | -171.08 | |  | -12.08 |
|  | -5.67 |  | -36.5 |  | -30.58 |  | -98.62 |  | -99.07 | -102.38 | | -104.64 | | NA |  |
| -154.67 | | -178.68 | | -192.51 | | -220.62 | | -220.22 | | -197.72 | | -251.91 | | -212.68 | |
| -101.39 | | -157.78 | | -253.08 | | -340.43 | |  | -348.5 | -389.25 | | -375.05 | | -224.85 | |
| -454.14 | | -313.83 | | -208.44 | | -273.81 | | -260.25 | | -282.87 | | -283.63 | | -286.65 | |
| -207.62 | |  | -70.32 | -103.62 | | -137.73 | |  | -132.3 | -145.19 | | -144.03 | |  | -92.02 |
| -200.28 | |  | -58.52 | -136.88 | | -150.08 | |  | -142.5 | -154.59 | |  | -203.7 | -127.37 | |
| NA |  |  | -65.49 |  | -25.24 |  | -44.79 |  | -56.54 |  | -66.87 |  | -72.58 | NA |  |
| -292.22 | | -176.42 | |  | -553.3 | -392.16 | | -385.06 | | -400.42 | | -417.16 | |  | -97.35 |
| -386.68 | | -355.79 | | -1112.7 | |  | -743.6 | -731.31 | |  | -757.6 | -806.52 | |  | -77.3 |
| -167.73 | |  | -79.72 |  | -41.09 | -113.88 | | -121.51 | | -130.98 | | -124.48 | | -107.66 | |
| -290.54 | | -274.77 | | -244.64 | | -192.82 | | -206.56 | | -165.16 | | -262.99 | |  | -92.43 |

| -188.57 | | -139.72 | |  | -92.18 | -164.02 | | -156.35 | | -170.43 | | -158.63 | |  | -45.36 |
| --- | --- | --- | --- | --- | --- | --- | --- | --- | --- | --- | --- | --- | --- | --- | --- |
|  | -247.4 | -275.48 | | -260.54 | | -396.04 | | -371.57 | | -422.73 | | -332.67 | | -121.81 | |
| -611.87 | | -187.65 | | -294.21 | | -234.15 | | -221.82 | | -265.62 | | -295.34 | | -1381.76 | |
|  | -24.68 |  | -35.96 |  | -25.26 |  | -54.49 |  | -56.87 |  | -67.67 |  | -61.73 |  | -18.55 |
| -542.65 | | -355.99 | | -832.44 | | -653.96 | | -593.32 | | -633.71 | | -797.91 | | -931.08 | |
| -214.38 | | NA |  |  | -46.38 | NA |  | NA |  | NA |  | -100.05 | | -450.68 | |
| -533.82 | |  | -366.1 |  | -338.8 | -434.92 | | -427.75 | | -416.69 | | -358.93 | | -313.95 | |
| -318.88 | | NA |  |  | -31.04 |  | -91.29 |  | -81.9 |  | -85.15 | -102.61 | |  | -83.55 |
| -216.29 | | -202.39 | |  | -84.29 | -151.72 | | -168.89 | | -170.24 | | -183.81 | | -197.07 | |
|  | -92.07 |  | -65.43 |  | -89.89 | -109.26 | |  | -96.65 | -110.56 | | -115.48 | |  | -39.44 |
| -168.57 | | -119.69 | |  | -79.76 | -200.97 | | -175.65 | | -206.05 | | -209.65 | |  | -56.18 |
| NA |  | NA |  | NA |  | NA |  | NA |  | NA |  | NA |  | NA |  |
|  | -62.07 |  | -129.9 | -165.27 | | -169.32 | | -197.22 | | -171.26 | |  | -186.5 | NA |  |
| NA |  |  | -62.15 |  | -41.37 |  | -107.3 | -125.91 | | -130.07 | | -130.47 | | NA |  |
|  | -7.99 |  | -113.1 |  | -87.15 | -201.99 | | -204.93 | | -235.34 | | -229.55 | |  | -5.48 |
| -912.93 | | -605.69 | | -373.42 | | -601.86 | | -577.37 | | -543.43 | |  | -505.2 | -300.69 | |
|  | -87.62 |  | -32.05 | NA |  |  | -25.19 |  | -20.43 |  | -24.02 |  | -51.44 |  | -28.92 |
| -264.82 | | -289.08 | |  | -85.71 | -197.25 | |  | -201.2 | -186.43 | | -177.55 | |  | -66.17 |
|  | -11.1 | NA |  |  | -9.94 | NA |  | NA |  | NA |  |  | -40.13 |  | -5.09 |
|  | -56.23 |  | -51.62 |  | -65.86 |  | -84.26 | -102.84 | | -114.78 | | -110.03 | |  | -25.17 |
| -172.14 | |  | -101.9 | -193.07 | | -251.24 | | -264.15 | | -294.84 | | -247.38 | |  | -76.21 |
|  | -8.78 |  | -7.66 | -319.13 | | -127.98 | |  | -145.4 | -201.94 | | -180.08 | | NA |  |
|  | -205.9 | -135.42 | |  | -35.46 | -131.28 | | -115.65 | | -140.98 | | -154.93 | |  | -96.23 |
|  | -22.11 | -229.25 | | -508.25 | |  | -575.6 | -604.47 | | -565.16 | | -423.65 | | NA |  |
| -236.62 | | NA |  |  | -41.03 |  | -152.8 | -131.77 | | -150.07 | | -184.25 | | -154.92 | |
|  | -82.78 | -153.37 | | -457.95 | | -306.01 | | -325.81 | | -336.78 | | -277.12 | | -118.51 | |
| -289.42 | |  | -89.93 | -122.16 | | -147.33 | | -131.46 | | -149.63 | | -166.34 | | -270.33 | |
|  | -58.02 | -128.39 | |  | -75.58 | -293.39 | | -322.83 | | -281.64 | | -259.86 | | NA |  |
| -243.02 | |  | -84.62 |  | -63.65 |  | -64.44 |  | -62.82 |  | -76.55 | -103.98 | | -214.34 | |
|  | -99.14 |  | -22.12 |  | -36.44 |  | -44.75 |  | -35.49 |  | -48.95 |  | -48.13 |  | -89.71 |
|  | -91.68 |  | -192.4 | -464.75 | | -400.26 | |  | -419.7 | -448.13 | |  | -345 |  | -13.77 |
| -146.64 | | -279.03 | | -237.64 | |  | -298.6 | -302.02 | | -292.81 | |  | -381 | -146.19 | |
| -149.27 | | -144.88 | |  | -50.69 | -190.61 | | -209.62 | | -200.05 | | -209.61 | |  | -41.93 |
|  | -18.47 |  | -93.72 | -232.68 | | -176.35 | | -161.39 | | -157.32 | |  | -105.2 |  | -28.12 |
| NA |  | NA |  | NA |  | NA |  | NA |  | NA |  |  | -16.34 | NA |  |
| -164.09 | |  | -60.18 |  | -42.68 |  | -86.17 |  | -50.49 |  | -71.29 |  | -77.07 |  | -153.6 |
|  | -111.3 | -105.42 | | -136.73 | | -135.03 | | -111.31 | | -167.06 | | -154.93 | |  | -75.35 |
|  | -81.44 | -121.57 | |  | -176.6 | -302.65 | | -306.69 | | -344.89 | | -278.72 | |  | -12.32 |

hesc_deriv hesc_deriv hesc_deriv immature_ immature_ immature_ immature_ immature_ NA	-5.66	-6.99	-229.45	-213.7	-209.06	-203.45	-217.56

NA	NA	NA	NA	NA	NA	NA	NA

NA	NA	NA	-344.42	-186.81	-192.73	-198.37	-212.72

NA	NA	NA	-148.45	-121.72	-136.41	-130.86	-132.15

NA	NA	NA	-25.76	-18.85	-24.06	-20.96	-25.64

NA	-56.01 NA	-699.92	-700.59	-819.79	-775.46	-790.43

NA	NA	NA	-378.69	-157.16	-171.84	-151.93	-171.39

NA	NA	NA	-50.83	-32.44	-42.09	-42.26	-36.47

NA	NA	NA	-50.83	-32.44	-42.09	-42.26	-36.47

NA	NA	NA	-170.97 NA	NA	NA	NA

NA	-37.09 NA	-487.04	-429.36	-506.27	-442.88	-519.1

NA	-11.11 NA	-112.42	-156.86	-209.95	-184.51	-205.68

NA	NA	-38.83	-653.99	-80.95	-92.99	-83.62	-77.43

NA	NA	-49.5	-721.21	-106.05	-118.51	-99.78	-94.43

NA	NA	NA	NA	NA	NA	NA	NA NA	NA	NA	NA	NA	NA	NA	NA NA	NA	-14.74	-160.92 NA	NA	NA	NA NA	NA	-14.74	-160.92 NA	NA	NA	NA

NA	NA	NA	-5661.62	-1753.87	-1805.48	-1806.94	-1761.91

NA	NA	NA	-236.09	-197.99	-190.18	-190.82	-192.34

NA	NA	NA	NA	NA	NA	NA	NA

NA	NA	NA	-1967.9	-1414.8	-1529.44	-1515.32	-1628.44

| NA | NA | NA | NA NA | NA | NA | NA |
| --- | --- | --- | --- | --- | --- | --- |
| NA | NA | NA | NA NA | NA | NA | NA |
| NA | NA | NA | -409.49 NA | NA | NA | NA |
| NA | NA | NA | -1178 NA | NA | NA | NA |
| NA | NA | NA | -1260.26 NA | NA | NA | NA |
| NA | NA | NA | -818.83 NA | NA | NA | NA |
| NA | NA | NA | -287.96 -192.05 | -215.97 | -205.66 | -229.34 |
| NA | NA | NA | -287.96 -192.05 | -215.97 | -205.66 | -229.34 |
| NA | NA | NA | -457.7 -300.82 | -332.41 | -309.91 | -328.36 |
| NA | NA | NA | -574.55 -341.94 | -361.46 | -354.96 | -365.42 |
| NA | NA | NA | -261.78 NA | NA | NA | NA |
| NA | NA | NA | -661.52 NA | NA | NA | NA |
| NA | NA | NA | NA NA | NA | NA | NA |
| NA | NA | NA | NA NA | NA | NA | NA |
| NA | NA | NA | NA NA | NA | NA | NA |
| NA |  | -8.64 NA | -121.64 -210.08 | -218.19 | -229.44 | -218.88 |

| NA |  |  | -37.28 | NA |  |  | -50.69 |  | -62.62 |  | -51.83 |  | -54.29 |  | -54 |
| --- | --- | --- | --- | --- | --- | --- | --- | --- | --- | --- | --- | --- | --- | --- | --- |
| NA |  |  | -37.28 | NA |  |  | -50.69 |  | -62.62 |  | -51.83 |  | -54.29 |  | -54 |
| NA |  |  | -37.28 | NA |  |  | -50.69 |  | -62.62 |  | -51.83 |  | -54.29 |  | -54 |
| NA |  |  | -9.6 | NA |  | -226.37 | | -168.14 | | -187.35 | |  | -163.3 | -187.46 | |
| NA |  |  | -7.91 | NA |  | -140.68 | | -127.33 | | -134.44 | | -119.76 | | -126.98 | |
| NA |  | NA |  |  | -6.19 | -5899.42 | | -3610.24 | | -3675.08 | | -3549.64 | | -3612.28 | |
| NA |  | NA |  |  | -8.74 | -7806.09 | | -4215.06 | | -4362.28 | | -4301.75 | | -4309.71 | |
| NA |  | NA |  |  | -9.35 | -10918.9 | | -6116.21 | | -6260.45 | | -6144.54 | | -6206.42 | |
|  | -9.67 | NA |  |  | -8.38 | -7770.46 | | -4850.5 | | -5031.97 | | -4829.01 | | -4979.1 | |
| NA |  | NA |  |  | -8.16 | -5001.75 | | -2713.2 | | -2819.69 | | -2747.71 | | -2825.88 | |
| NA |  | NA |  |  | -5.66 | -6396.12 | | -3511.59 | | -3679.34 | | -3595.34 | | -3704.07 | |
| NA |  | NA |  |  | -7.41 | -10884.9 | | -5499.76 | | -5689.48 | | -5542.36 | | -5660.37 | |
| NA |  | NA |  |  | -9.03 | -5705.54 | | -3653.66 | | -3764.73 | | -3692.21 | | -3774.32 | |
| NA |  | NA |  |  | -16.56 | -7099.5 | | -4312.46 | | -4494.22 | | -4347.89 | | -4544.74 | |
| NA |  | NA |  |  | -17.1 | -8373.03 | | -5211.86 | | -5376.45 | | -5367.3 | | -5438.28 | |
| NA |  | NA |  | NA |  | NA |  | NA |  | NA |  | NA |  | NA |  |
| NA |  | NA |  | NA |  |  | -65.98 |  | -54.41 |  | -63.85 |  | -52.26 |  | -56.04 |
| NA |  | NA |  |  | -23.7 | -8651.96 | | -5446.25 | | -5729.14 | | -5582.42 | | -5621.97 | |
| NA |  | NA |  |  | -20.45 | -9630.72 | | -6148.89 | | -6437.37 | | -6261.28 | | -6429.18 | |
| NA |  | NA |  | NA |  | -664.64 | | -432.43 | | -488.04 | | -454.87 | | -461.04 | |
| NA |  | NA |  |  | -8.71 | -12533.6 | | -7973.03 | | -8176.8 | | -7978.21 | | -8039.68 | |
| NA |  | NA |  | NA |  | NA |  | NA |  | NA |  | NA |  | NA |  |
| NA |  | NA |  | NA |  | NA |  | NA |  | NA |  | NA |  | NA |  |
| NA |  | NA |  |  | -14.62 | -10833.9 | | -6782.98 | | -7019.7 | | -6958.09 | | -7048.13 | |
| NA |  | NA |  | NA |  | -381.55 | | -282.05 | | -285.35 | | -288.82 | | -288.41 | |
| NA |  | NA |  | NA |  | -6431.44 | | -3395.16 | | -3557.01 | | -3414.81 | | -3503.39 | |
| NA |  | NA |  |  | -24.38 | -10175.7 | | -6451.55 | | -6746.46 | | -6586.67 | | -6640.09 | |
| NA |  | NA |  |  | -17.95 | -7912.3 | | -4883.77 | | -5001.1 | | -4902.51 | | -5040.38 | |
| NA |  | NA |  |  | -10.13 | -3103.41 | | -1942.76 | | -2057.89 | | -1943.43 | | -2048.66 | |
| NA |  | NA |  |  | -10.13 | -3103.41 | | -1942.76 | | -2057.89 | | -1943.43 | | -2048.66 | |
| NA |  | NA |  |  | -15.09 | -11557.1 | | -7188.71 | | -7498.04 | | -7274.95 | | -7414.49 | |
| NA |  | NA |  |  | -13.99 | -4616.33 | | -3096.85 | | -3210.15 | | -3109.6 | | -3181.31 | |
| NA |  | NA |  | NA |  | -4069.92 | | -2077.14 | | -2130.97 | | -2141.92 | | -2129.38 | |
| NA |  | NA |  | NA |  | -3640.09 | | -2234.83 | | -2288.15 | | -2234.5 | | -2256.6 | |
| NA |  | NA |  | NA |  | NA |  | NA |  | NA |  | NA |  | NA |  |
| NA |  |  | -25.65 | NA |  | -169.83 | | -186.15 | | -225.41 | | -216.43 | | -212.85 | |
| NA |  | NA |  |  | -15.05 | -11320.6 | | -6332.1 | | -6706.42 | | -6561.26 | | -6618.15 | |
| NA |  | NA |  |  | -10.74 | -12352.4 | | -7791.16 | | -8032.36 | | -7891.74 | | -8008.28 | |
| NA |  | NA |  | NA |  | -716.13 | |  | -494.4 | -534.64 | | -507.88 | | -525.79 | |

| NA |  | NA |  |  | -65.16 | -831.46 | |  | -56.92 |  | -63.85 |  | -51.9 |  | -47.12 |
| --- | --- | --- | --- | --- | --- | --- | --- | --- | --- | --- | --- | --- | --- | --- | --- |
| NA |  | NA |  |  | -46.63 | -786.96 | |  | -95.37 | -102.95 | |  | -91.57 |  | -73.85 |
| NA |  | NA |  |  | -52.8 | -991.72 | | -130.98 | | -150.72 | | -130.62 | | -125.91 | |
| NA |  | NA |  |  | -57.63 | -390.43 | | NA |  |  | -7.28 | NA |  | NA |  |
|  | -11.74 | NA |  | NA |  | NA |  | NA |  | NA |  | NA |  | NA |  |
|  | -10 | NA |  | NA |  | NA |  | NA |  | NA |  | NA |  | NA |  |
|  | -8.06 | NA |  | NA |  | NA |  | NA |  | NA |  | NA |  | NA |  |
| NA |  | NA |  | NA |  | NA |  | NA |  | NA |  | NA |  | NA |  |
|  | -7.79 | NA |  | NA |  | NA |  | NA |  | NA |  | NA |  | NA |  |
| NA |  | NA |  | NA |  | NA |  | NA |  | NA |  | NA |  | NA |  |
| NA |  | NA |  | NA |  | NA |  | NA |  | NA |  | NA |  | NA |  |
| NA |  | NA |  | NA |  | NA |  | NA |  | NA |  | NA |  | NA |  |
| NA |  | NA |  | NA |  | NA |  | NA |  | NA |  | NA |  | NA |  |
| NA |  | NA |  | NA |  | NA |  | NA |  | NA |  | NA |  | NA |  |
| NA |  | NA |  | NA |  | NA |  | NA |  | NA |  | NA |  | NA |  |
|  | -12.84 | NA |  | NA |  |  | -9.19 | NA |  | NA |  | NA |  | NA |  |
| NA |  | NA |  | NA |  | NA |  | NA |  | NA |  | NA |  | NA |  |
| NA |  | NA |  | NA |  | NA |  | NA |  | NA |  | NA |  | NA |  |
|  | -5.78 | NA |  | NA |  |  | -39.91 |  | -31.66 |  | -33.05 |  | -33.47 |  | -34.36 |
|  | -15.75 | NA |  | NA |  |  | -5.39 | NA |  | NA |  | NA |  | NA |  |
| NA |  | NA |  | NA |  | NA |  | NA |  | NA |  | NA |  | NA |  |
| NA |  | NA |  |  | -12.14 | -9154.67 | | -5779.89 | | -5985.96 | | -5871.47 | | -5964.15 | |
|  | -56.07 | NA |  |  | -38.56 | -141.13 | | -132.45 | | -136.66 | | -132.99 | | -128.22 | |
|  | -56.07 | NA |  |  | -38.56 | -141.13 | | -132.45 | | -136.66 | | -132.99 | | -128.22 | |
|  | -42.86 | NA |  |  | -8.9 | NA |  | NA |  | NA |  | NA |  | NA |  |
|  | -42.86 | NA |  |  | -8.9 | NA |  | NA |  | NA |  | NA |  | NA |  |
|  | -51.98 | NA |  |  | -15.89 |  | -7.52 |  | -17.8 |  | -18.81 |  | -15.93 |  | -16.24 |
|  | -139.7 | NA |  |  | -50.05 | -107.28 | | -152.54 | | -163.28 | | -135.35 | | -148.17 | |
|  | -54 | NA |  |  | -8.89 | NA |  | NA |  | NA |  | NA |  | NA |  |
|  | -41.6 | NA |  |  | -6 | NA |  | NA |  | NA |  | NA |  | NA |  |
|  | -41.6 | NA |  |  | -6 | NA |  | NA |  | NA |  | NA |  | NA |  |
|  | -41.6 | NA |  |  | -6 | NA |  | NA |  | NA |  | NA |  | NA |  |
| NA |  | NA |  | NA |  | NA |  | NA |  | NA |  | NA |  | NA |  |
| NA |  | NA |  | NA |  | NA |  | NA |  | NA |  | NA |  | NA |  |
| NA |  | NA |  | NA |  | NA |  | NA |  | NA |  | NA |  | NA |  |
| NA |  | NA |  | NA |  | NA |  | NA |  | NA |  | NA |  | NA |  |
| NA |  | NA |  | NA |  |  | -57.26 |  | -73.89 |  | -70.43 |  | -65.96 |  | -80.57 |
| NA |  |  | -8.7 | NA |  |  | -32.7 |  | -54.32 |  | -75.77 |  | -64.32 |  | -68.74 |
| NA |  |  | -8.7 | NA |  |  | -32.7 |  | -54.32 |  | -75.77 |  | -64.32 |  | -68.74 |

| NA | NA |  | NA |  | -90.6 | -106.78 | |  | -133 | -115.31 | | -123.34 | |
| --- | --- | --- | --- | --- | --- | --- | --- | --- | --- | --- | --- | --- | --- |
| NA | NA |  | NA | NA |  | NA |  | NA |  | NA |  | NA |  |
| NA | NA |  | NA | -187.98 | | -173.54 | | -187.08 | | -176.89 | | -189.81 | |
| NA |  | -5.62 | NA | NA |  |  | -13.39 |  | -10.87 |  | -14.74 |  | -14.76 |
| NA |  | -18.37 | NA | -429.76 | |  | -311 | -305.08 | | -311.61 | | -294.25 | |
| NA | NA |  | NA |  | -550.5 | NA |  | NA |  | NA |  | NA |  |
| NA | NA |  | NA | NA |  | NA |  | NA |  | NA |  | NA |  |
| NA | NA |  | NA | NA |  | NA |  | NA |  | NA |  | NA |  |
| NA |  | -6.69 | NA |  | -5.66 | NA |  | NA |  | NA |  | NA |  |
| NA |  | -15.22 | NA |  | -5.12 | NA |  | NA |  | NA |  | NA |  |
| NA |  | -20.44 | NA |  | -55.52 |  | -30.98 |  | -32.51 |  | -26.63 |  | -40.67 |
| NA | NA |  | NA | NA |  | NA |  | NA |  | NA |  | NA |  |
| NA | NA |  | NA | NA |  | NA |  | NA |  | NA |  | NA |  |
| NA | NA |  | NA | NA |  | NA |  | NA |  | NA |  | NA |  |
| NA | NA |  | NA | NA |  | NA |  | NA |  | NA |  | NA |  |
| NA | NA |  | NA | NA |  | NA |  | NA |  | NA |  | NA |  |
| NA | NA |  | NA | NA |  | NA |  | NA |  | NA |  | NA |  |
| NA | NA |  | NA | NA |  | NA |  | NA |  | NA |  | NA |  |
| NA | NA |  | NA | NA |  | NA |  | NA |  | NA |  | NA |  |
| NA | NA |  | NA |  | -13.28 |  | -80.3 |  | -81.22 |  | -77.85 |  | -93.22 |
| NA | NA |  | NA | NA |  | NA |  | NA |  | NA |  | NA |  |
| NA | NA |  | NA | NA |  | NA |  | NA |  | NA |  | NA |  |
| NA | NA |  | NA | NA |  | NA |  | NA |  | NA |  | NA |  |
| NA | NA |  | NA | NA |  | NA |  | NA |  | NA |  | NA |  |
| NA | NA |  | NA | NA |  | NA |  | NA |  | NA |  | NA |  |
| NA | NA |  | NA |  | -20.04 |  | -59.44 |  | -66.59 |  | -58.03 |  | -68.26 |
| NA | NA |  | NA | NA |  | NA |  | NA |  | NA |  | NA |  |
| NA | NA |  | NA | NA |  | NA |  | NA |  | NA |  | NA |  |
| NA | NA |  | NA | NA |  | NA |  | NA |  | NA |  | NA |  |
| NA | NA |  | NA | NA |  | NA |  | NA |  | NA |  | NA |  |
| NA |  | -8.09 | NA |  | -52.51 |  | -33.61 |  | -30.58 |  | -34.51 |  | -29.85 |
| NA | NA |  | NA | NA |  | NA |  | NA |  | NA |  | NA |  |
| NA | NA |  | NA | NA |  | NA |  | NA |  | NA |  | NA |  |
| NA | NA |  | NA | NA |  | NA |  | NA |  | NA |  | NA |  |
| NA | NA |  | NA | NA |  | NA |  | NA |  | NA |  | NA |  |
| NA | NA |  | NA | NA |  | NA |  | NA |  | NA |  | NA |  |
| NA | NA |  | NA | NA |  | NA |  | NA |  | NA |  | NA |  |
| NA | NA |  | NA | NA |  | NA |  | NA |  | NA |  |  | -6.92 |
| NA | NA |  | NA | NA |  | NA |  | NA |  | NA |  | NA |  |

| NA | NA |  | NA |  | NA |  | NA |  | NA |  | NA |  | NA |  |
| --- | --- | --- | --- | --- | --- | --- | --- | --- | --- | --- | --- | --- | --- | --- |
| NA | NA |  | NA |  |  | -41.65 | -125.01 | |  | -134.9 | -120.42 | | -133.39 | |
| NA | NA |  | NA |  | NA |  | NA |  | NA |  | NA |  | NA |  |
| NA | NA |  | NA |  | NA |  | NA |  | NA |  | NA |  | NA |  |
| NA | NA |  | NA |  | NA |  | NA |  | NA |  | NA |  | NA |  |
| NA | NA |  | NA |  |  | -11.96 | NA |  |  | -9.43 |  | -8.81 |  | -7.21 |
| NA | NA |  | NA |  | NA |  | NA |  | NA |  | NA |  | NA |  |
| NA | NA |  | NA |  | NA |  | NA |  | NA |  | NA |  | NA |  |
| NA | NA |  | NA |  | NA |  | NA |  | NA |  | NA |  | NA |  |
| NA | NA |  | NA |  | NA |  | NA |  | NA |  | NA |  | NA |  |
| NA | NA |  | NA |  | -2452.09 | | -1139.53 | | -1209.68 | | -1141.72 | | -1120.23 | |
| NA | NA |  | NA |  |  | -95.67 |  | -8.83 |  | -6.49 |  | -8.97 |  | -9.41 |
| NA | NA |  | NA |  | -218.55 | |  | -16.71 |  | -11.97 |  | -8.64 |  | -9.39 |
| NA | NA |  | NA |  | -1241.38 | | -366.44 | | -358.31 | | -355.58 | | -359.04 | |
| NA | NA |  | NA |  | -624.25 | | -198.33 | | -197.53 | |  | -206.6 | -232.54 | |
| NA | NA |  | NA |  |  | -389 | -128.77 | |  | -133.1 | -126.78 | | -140.08 | |
| NA | NA |  | NA |  |  | -41.53 | NA |  | NA |  | NA |  | NA |  |
| NA | NA |  | NA |  | -283.51 | |  | -42.59 |  | -40.24 |  | -32.01 |  | -46.29 |
| NA | NA |  | NA |  | -1526.68 | | -367.01 | |  | -378.6 | -343.65 | | -379.98 | |
| NA | NA |  | NA |  | -307.81 | |  | -49.4 |  | -42.44 |  | -46.93 |  | -60.64 |
| NA | NA |  |  | -59.04 | -559.14 | |  | -18.38 |  | -27.99 |  | -13.95 |  | -9.84 |
| NA | NA |  |  | -54.95 | -602.06 | |  | -29.16 |  | -28.9 |  | -21.98 |  | -19.86 |
| NA | NA |  |  | -48.15 | -885.94 | |  | -74.26 |  | -70.24 |  | -57.09 |  | -55.2 |
| NA | NA |  |  | -50.99 | -288.71 | | NA |  |  | -5.16 | NA |  | NA |  |
| NA | NA |  | NA |  |  | -42.29 |  | -39.33 |  | -29.55 |  | -25.2 |  | -35.11 |
| NA |  | -20.36 | NA |  | -100.87 | |  | -80.37 |  | -60.5 |  | -66.93 |  | -69.72 |
| NA | NA |  | NA |  |  | -40.78 |  | -29.16 |  | -23.08 |  | -26.16 |  | -35.58 |
| NA | NA |  | NA |  |  | -60.21 |  | -34.24 |  | -25.2 |  | -29.04 |  | -36.37 |
| NA | NA |  | NA |  |  | -60.21 |  | -34.24 |  | -25.2 |  | -29.04 |  | -36.37 |
| NA | NA |  | NA |  |  | -17.12 |  | -25.24 |  | -22.72 |  | -22.95 |  | -32.08 |
| NA |  | -16.34 | NA |  | -125.63 | | -171.24 | | -154.44 | | -165.96 | | -175.55 | |
| NA | NA |  | NA |  |  | -30.93 |  | -40.94 |  | -29.24 |  | -21.66 |  | -37.73 |
| NA | NA |  | NA |  |  | -30.93 |  | -40.94 |  | -29.24 |  | -21.66 |  | -37.73 |
| NA | NA |  | NA |  |  | -30.93 |  | -40.94 |  | -29.24 |  | -21.66 |  | -37.73 |
| NA |  | -8.76 | NA |  |  | -182.8 | -220.02 | | -190.53 | | -195.66 | | -214.66 | |
| NA |  | -44.37 | NA |  | -213.44 | | -307.81 | | -335.74 | | -314.74 | | -336.18 | |
| NA | NA |  | NA |  | NA |  | NA |  | NA |  | NA |  | NA |  |
| NA | NA |  | NA |  | NA |  | NA |  | NA |  | NA |  | NA |  |
| NA |  | -35.67 |  | -5.44 |  | -31.82 |  | -8.34 | NA |  |  | -6.15 | NA |  |

| NA |  | NA |  | NA |  | NA |  | NA |  | NA |  | NA |  | NA |  |
| --- | --- | --- | --- | --- | --- | --- | --- | --- | --- | --- | --- | --- | --- | --- | --- |
| NA |  | NA |  | NA |  | NA |  | NA |  | NA |  | NA |  | NA |  |
| NA |  | NA |  | NA |  | NA |  | NA |  | NA |  | NA |  | NA |  |
| NA |  | NA |  | NA |  | NA |  | NA |  | NA |  | NA |  | NA |  |
| NA |  |  | -39.41 | NA |  | -281.56 | | -332.51 | | -354.72 | | -336.66 | | -334.49 | |
|  | -5.38 |  | -44.64 | NA |  | -384.14 | | -265.87 | | -253.04 | | -239.22 | | -252.05 | |
| NA |  |  | -16.18 | NA |  |  | -76.05 |  | -82.15 |  | -95.4 | -103.63 | |  | -86.08 |
| NA |  |  | -20.78 | NA |  | -574.26 | |  | -423.6 | -453.15 | | -425.04 | | -465.82 | |
| NA |  |  | -6.39 | NA |  |  | -73.09 |  | -38.54 |  | -35.49 |  | -42.33 |  | -32.41 |
| NA |  | NA |  | NA |  | -159.73 | |  | -89.56 |  | -83.4 |  | -93.01 |  | -84.99 |
| NA |  | NA |  | NA |  | -307.32 | | -210.45 | | -212.93 | | -217.84 | | -229.35 | |
| NA |  |  | -25.32 | NA |  | -193.01 | | -232.93 | |  | -224.6 | -242.12 | | -251.36 | |
| NA |  |  | -22.17 |  | -5.72 | -384.54 | | -345.53 | | -334.52 | | -369.12 | | -367.62 | |
| NA |  |  | -7.61 | NA |  |  | -47.48 |  | -42.65 |  | -61.4 |  | -49.98 |  | -54.2 |
| NA |  | NA |  | NA |  | NA |  | NA |  | NA |  | NA |  | NA |  |
| NA |  | NA |  | NA |  | NA |  | NA |  | NA |  | NA |  | NA |  |
| NA |  | NA |  | NA |  | -149.01 | | -120.81 | | -122.98 | | -130.72 | | -118.97 | |
| NA |  | NA |  | NA |  | NA |  | NA |  | NA |  | NA |  | NA |  |
|  | -12.21 |  | -29.08 | NA |  |  | -302.1 | -335.37 | | -383.21 | | -387.95 | | -376.64 | |
| NA |  | NA |  | NA |  | NA |  | NA |  | NA |  | NA |  | NA |  |
| NA |  | NA |  | NA |  | NA |  | NA |  | NA |  | NA |  | NA |  |
| NA |  | NA |  | NA |  | -170.29 | | -140.17 | |  | -170.8 | -147.18 | | -149.59 | |
| NA |  | NA |  | NA |  | -322.93 | | -205.57 | | -223.45 | | -219.27 | | -235.39 | |
| NA |  | NA |  | NA |  | -322.93 | | -205.57 | | -223.45 | | -219.27 | | -235.39 | |
| NA |  | NA |  | NA |  | -322.93 | | -205.57 | | -223.45 | | -219.27 | | -235.39 | |
| NA |  | NA |  | NA |  | -322.93 | | -205.57 | | -223.45 | | -219.27 | | -235.39 | |
| NA |  | NA |  | NA |  | -608.97 | | -441.38 | | -445.04 | | -450.61 | | -448.08 | |
| NA |  | NA |  | NA |  | -608.97 | | -441.38 | | -445.04 | | -450.61 | | -448.08 | |
| NA |  | NA |  | NA |  | -608.97 | | -441.38 | | -445.04 | | -450.61 | | -448.08 | |
| NA |  | NA |  | NA |  | -608.97 | | -441.38 | | -445.04 | | -450.61 | | -448.08 | |
| NA |  | NA |  | NA |  | -452.46 | | -168.52 | | -190.93 | | -191.26 | | -179.84 | |
| NA |  | NA |  | NA |  | -452.46 | | -168.52 | | -190.93 | | -191.26 | | -179.84 | |
| NA |  | NA |  | NA |  | -324.84 | | -159.27 | | -136.26 | | -155.96 | | -158.89 | |
| NA |  | NA |  | NA |  | -324.84 | | -159.27 | | -136.26 | | -155.96 | | -158.89 | |
| NA |  | NA |  | NA |  | -554.85 | | -362.59 | | -394.04 | | -388.43 | | -397.94 | |
| NA |  |  | -57.08 | NA |  | -270.01 | | -374.44 | | -460.85 | | -398.16 | | -432.59 | |
| NA |  |  | -57.08 | NA |  | -270.01 | | -374.44 | | -460.85 | | -398.16 | | -432.59 | |
| NA |  |  | -20.48 | NA |  | -402.38 | | -322.25 | | -409.12 | | -376.16 | | -416.68 | |
| NA |  |  | -20.48 | NA |  | -402.38 | | -322.25 | | -409.12 | | -376.16 | | -416.68 | |

| NA |  | -30.04 | NA |  |  | -204.1 | -379.01 | | -432.67 | |  | -396.1 | -412.14 | |
| --- | --- | --- | --- | --- | --- | --- | --- | --- | --- | --- | --- | --- | --- | --- |
| NA |  | -66.52 | NA |  | -429.14 | | -564.78 | | -647.43 | | -559.67 | | -612.86 | |
| NA |  | -21.27 | NA |  | -214.11 | | -290.72 | | -344.56 | | -326.34 | | -339.75 | |
| NA | NA |  | NA |  | -105.51 | |  | -94.72 | -130.65 | | -107.42 | | -118.68 | |
| NA | NA |  |  | -20.81 | -884.72 | | -262.81 | |  | -273.3 | -236.35 | | -243.93 | |
| NA | NA |  |  | -51.6 | -423.25 | |  | -7.49 |  | -13.56 | NA |  | NA |  |
| NA | NA |  |  | -8.77 | -743.58 | | -276.87 | | -304.89 | | -276.78 | | -279.06 | |
| NA | NA |  | NA |  | -247.89 | | -318.92 | | -326.74 | | -309.91 | | -328.41 | |
| NA | NA |  | NA |  | -303.19 | | -404.03 | | -405.32 | | -385.56 | | -411.11 | |
| NA | NA |  | NA |  | NA |  | NA |  | NA |  | NA |  | NA |  |
| NA | NA |  | NA |  | -321.39 | | -393.78 | | -402.04 | | -401.25 | | -401.91 | |
| NA | NA |  | NA |  |  | -203.2 | -188.35 | | -192.67 | | -187.26 | | -214.48 | |
| NA | NA |  | NA |  | NA |  | NA |  | NA |  | NA |  | NA |  |
| NA |  | -17.47 | NA |  | -238.52 | | -156.55 | | -148.03 | | -152.59 | | -152.51 | |
| NA | NA |  | NA |  | NA |  | NA |  | NA |  | NA |  | NA |  |
| NA | NA |  | NA |  | NA |  | NA |  | NA |  | NA |  | NA |  |
| NA | NA |  | NA |  | NA |  | NA |  | NA |  | NA |  | NA |  |
| NA | NA |  | NA |  |  | -36.98 |  | -24.29 |  | -25.03 |  | -19.8 |  | -15.97 |
| NA | NA |  | NA |  | NA |  | NA |  | NA |  | NA |  | NA |  |
| NA | NA |  | NA |  | NA |  | NA |  | NA |  | NA |  | NA |  |
| NA | NA |  | NA |  | NA |  | NA |  | NA |  | NA |  | NA |  |
| NA | NA |  | NA |  | NA |  | NA |  | NA |  | NA |  | NA |  |
| NA | NA |  | NA |  | NA |  | NA |  | NA |  | NA |  | NA |  |
| NA | NA |  | NA |  |  | -78.95 |  | -31.82 |  | -33.78 |  | -30.31 |  | -48.41 |
| NA | NA |  | NA |  | -315.26 | | -177.14 | | -187.25 | |  | -181.5 | -183.67 | |
| NA | NA |  | NA |  | NA |  | NA |  | NA |  | NA |  | NA |  |
| NA | NA |  | NA |  | NA |  | NA |  | NA |  | NA |  | NA |  |
| NA | NA |  | NA |  | NA |  | NA |  | NA |  | NA |  | NA |  |
| NA | NA |  | NA |  | -547.92 | | -362.22 | | -381.89 | | -370.33 | | -398.78 | |
| NA |  | -26.24 | NA |  | -352.12 | | -317.44 | | -315.94 | | -306.93 | | -301.72 | |
| NA |  | -26.24 | NA |  | -352.12 | | -317.44 | | -315.94 | | -306.93 | | -301.72 | |
| NA |  | -10.42 | NA |  | NA |  | NA |  | NA |  | NA |  | NA |  |
| NA |  | -15.33 | NA |  | NA |  | NA |  | NA |  | NA |  | NA |  |
| NA |  | -16.09 | NA |  | NA |  | NA |  | NA |  | NA |  | NA |  |
| NA |  | -20.19 | NA |  |  | -6.78 |  | -7.25 |  | -9.69 |  | -10.82 |  | -5.4 |
| NA | NA |  | NA |  | -946.79 | | -731.52 | | -741.05 | | -733.64 | | -771.37 | |
| NA |  | -9.47 | NA |  |  | -14.4 | NA |  | NA |  | NA |  | NA |  |
| NA | NA |  | NA |  | NA |  | NA |  | NA |  | NA |  | NA |  |
| NA |  | -15.38 |  | -7.41 |  | -5.48 | NA |  | NA |  | NA |  | NA |  |

| NA |  | -18.12 | NA |  |  | -31.87 |  | -10.23 |  | -9.53 |  | -8.19 | NA |  |
| --- | --- | --- | --- | --- | --- | --- | --- | --- | --- | --- | --- | --- | --- | --- |
| NA | NA |  | NA |  | NA |  | NA |  | NA |  | NA |  | NA |  |
| NA | NA |  | NA |  | NA |  | NA |  | NA |  | NA |  | NA |  |
| NA | NA |  | NA |  | NA |  | NA |  | NA |  | NA |  | NA |  |
| NA | NA |  | NA |  | NA |  | NA |  | NA |  | NA |  | NA |  |
| NA |  | -36.78 |  | -7.57 | -474.04 | | -268.82 | | -245.95 | | -251.15 | | -254.79 | |
| NA | NA |  | NA |  | NA |  | NA |  | NA |  | NA |  | NA |  |
| NA | NA |  | NA |  | NA |  | NA |  | NA |  | NA |  | NA |  |
| NA | NA |  | NA |  | NA |  | NA |  | NA |  | NA |  | NA |  |
| NA | NA |  | NA |  | NA |  | NA |  | NA |  | NA |  | NA |  |
| NA |  | -6.13 | NA |  |  | -51.4 |  | -23.38 |  | -18.6 |  | -20.55 |  | -20.65 |
| NA |  | -7.58 | NA |  |  | -31.45 | NA |  | NA |  | NA |  | NA |  |
| NA |  | -10.05 |  | -11.2 | -410.87 | | -132.78 | | -133.09 | | -124.69 | | -140.64 | |
| NA | NA |  | NA |  | NA |  | NA |  | NA |  | NA |  | NA |  |
| NA | NA |  | NA |  | NA |  | NA |  | NA |  | NA |  | NA |  |
| NA |  | -5.49 | NA |  |  | -68.55 |  | -5.47 |  | -9.13 |  | -7.76 |  | -6.89 |
| NA |  | -9.76 |  | -5.98 |  | -59.65 |  | -18.99 |  | -13.31 |  | -8.14 |  | -15 |
| NA |  | -12.4 | NA |  | NA |  | NA |  | NA |  | NA |  | NA |  |
| NA | NA |  | NA |  | NA |  | NA |  | NA |  | NA |  | NA |  |
| NA |  | -39.27 | NA |  |  | -37 |  | -160.7 | -163.69 | | -152.21 | | -164.98 | |
| NA |  | -38.22 | NA |  | -201.81 | | -303.57 | | -311.94 | | -305.88 | | -312.29 | |
| NA |  | -12.97 | NA |  |  | -60.2 |  | -62.11 |  | -48.57 |  | -38.64 |  | -52.51 |
| NA |  | -25.93 | NA |  |  | -66.05 |  | -28.67 |  | -35 |  | -33.27 |  | -32.52 |
| NA | NA |  | NA |  | -724.76 | | -469.79 | | -506.97 | | -471.66 | | -505.33 | |
| NA |  | -17.47 | NA |  | -225.84 | | -192.78 | | -188.15 | | -177.24 | |  | -204.7 |
| NA | NA |  | NA |  |  | -15.99 |  | -7.81 |  | -11.38 |  | -11.71 |  | -8.25 |
| NA |  | -11.21 | NA |  | -273.26 | | -168.56 | | -159.12 | | -172.14 | |  | -175.3 |
| NA |  | -27.1 | NA |  |  | -65.36 |  | -40.29 |  | -34.59 |  | -45.84 |  | -41.7 |
| NA | NA |  | NA |  | -369.85 | | -206.82 | |  | -228.4 | -217.25 | | -233.51 | |
| NA |  | -20.84 | NA |  |  | -18.49 |  | -20.44 |  | -29.21 |  | -21.97 |  | -20.98 |
| NA |  | -9.15 | NA |  |  | -92.84 |  | -54.46 |  | -65.96 |  | -59.64 |  | -63.34 |
| NA |  | -9.71 | NA |  |  | -64.64 |  | -53.49 |  | -58.25 |  | -59.18 |  | -60.64 |
| NA | NA |  | NA |  |  | -137 | -100.04 | |  | -98.63 | -108.32 | | -111.04 | |
| NA |  | -14.44 | NA |  | -110.53 | | -123.87 | | -122.18 | | -126.33 | | -143.32 | |
| NA | NA |  | NA |  |  | -90.76 |  | -43.64 |  | -55.55 |  | -52.33 |  | -49.59 |
| NA |  | -9.83 | NA |  |  | -11.23 | NA |  |  | -7.61 |  | -5.68 |  | -6.89 |
| NA |  | -11.57 | NA |  | NA |  | NA |  | NA |  | NA |  | NA |  |
| NA |  | -11.57 | NA |  | NA |  | NA |  | NA |  | NA |  | NA |  |
| NA |  | -11.57 | NA |  | NA |  | NA |  | NA |  | NA |  | NA |  |

| NA |  | NA |  | NA |  | -1865.48 | | -1281.13 | | -1412.74 | | -1353.45 | | -1506.22 | |
| --- | --- | --- | --- | --- | --- | --- | --- | --- | --- | --- | --- | --- | --- | --- | --- |
| NA |  | NA |  | NA |  | -2380.24 | | -1613.69 | | -1784.14 | | -1726.95 | | -1923.51 | |
| NA |  | NA |  | NA |  | -1071.37 | | -941.17 | | -990.19 | | -1005.99 | | -1080.68 | |
| NA |  |  | -25.21 | NA |  |  | -26.34 |  | -12.76 |  | -16 |  | -11.25 |  | -8.9 |
| NA |  |  | -18.72 | NA |  | NA |  | NA |  | NA |  | NA |  | NA |  |
| NA |  |  | -16.87 | NA |  | NA |  | NA |  | NA |  | NA |  | NA |  |
| NA |  |  | -25.23 | NA |  | NA |  | NA |  | NA |  | NA |  | NA |  |
| NA |  | NA |  | NA |  |  | -66.24 | NA |  |  | -12.72 |  | -11.24 |  | -5.9 |
| NA |  |  | -28.5 | NA |  | -114.65 | | -144.45 | | -144.76 | | -141.07 | |  | -151.3 |
|  | -8.59 |  | -14 |  | -7.04 |  | -70.85 | -100.96 | | -112.95 | | -121.28 | | -120.93 | |
| NA |  |  | -9.1 |  | -6.76 | -125.64 | |  | -121 | -118.71 | | -119.59 | | -147.52 | |
| NA |  |  | -9.1 |  | -6.76 | -125.64 | |  | -121 | -118.71 | | -119.59 | | -147.52 | |
| NA |  |  | -7.79 |  | -12.14 | -172.54 | | -116.25 | | -129.59 | |  | -137.4 | -138.36 | |
| NA |  | NA |  |  | -45.78 | -428.07 | |  | -9.17 |  | -6.73 | NA |  | NA |  |
| NA |  | NA |  |  | -45.78 | -428.07 | |  | -9.17 |  | -6.73 | NA |  | NA |  |
| NA |  | -105.72 | | NA |  |  | -29.23 |  | -6.07 | NA |  |  | -7.14 | NA |  |
| NA |  | -181.22 | | NA |  |  | -10.48 | NA |  | NA |  | NA |  | NA |  |
|  | -16.33 | -191.85 | | NA |  |  | -11.04 | NA |  | NA |  | NA |  | NA |  |
| NA |  |  | -13.41 | NA |  |  | -61.29 | -108.37 | |  | -99.5 |  | -99.17 | -101.07 | |
| NA |  | NA |  |  | -11.72 | -3559.26 | | -2181.75 | | -2293.09 | | -2237.26 | | -2312.58 | |
| NA |  | NA |  | NA |  | -9036.45 | | -3827.78 | | -3902.5 | | -3946.6 | | -3814.03 | |
| NA |  | NA |  | NA |  | -4062.19 | | -1490.95 | | -1541.15 | | -1518.97 | | -1434.41 | |
|  | -7.65 | NA |  |  | -6.42 | -5407.29 | | -2755.25 | | -2742.05 | | -2636.84 | | -2724.57 | |
| NA |  | NA |  | NA |  |  | -12885 | -5085.55 | | -5290.21 | | -5209.7 | | -5141.8 | |
| NA |  | NA |  | NA |  | -8516.13 | | -3689.5 | | -3849.32 | | -3725.18 | | -3701.44 | |
| NA |  | NA |  |  | -11.19 | -433.56 | | -177.72 | | -165.43 | | -172.15 | | -174.82 | |
| NA |  | NA |  | NA |  | -1613.22 | | -345.54 | | -339.38 | | -347.93 | | -344.83 | |
| NA |  | NA |  | NA |  | -360.56 | | -206.94 | | -207.67 | | -167.74 | |  | -180.4 |
| NA |  | NA |  | NA |  |  | -40.07 |  | -39.37 |  | -54.38 |  | -40.31 |  | -42.09 |
| NA |  |  | -72.31 | NA |  | -304.36 | |  | -429.2 | -506.84 | |  | -476.2 | -480.51 | |
| NA |  |  | -22.11 | NA |  | -482.14 | |  | -378.5 | -453.14 | | -412.13 | | -438.17 | |
| NA |  | NA |  | NA |  | NA |  |  | -20.81 |  | -28.41 |  | -25.16 |  | -30.28 |
| NA |  |  | -27.35 | NA |  | NA |  | NA |  | NA |  | NA |  | NA |  |
| NA |  | NA |  |  | -10.25 | NA |  | NA |  | NA |  | NA |  | NA |  |
| NA |  | NA |  |  | -8.05 | NA |  | NA |  | NA |  | NA |  | NA |  |
| NA |  |  | -26.65 | NA |  | NA |  |  | -36.35 |  | -41.08 |  | -46.85 |  | -45.78 |
| NA |  |  | -50.08 | NA |  | -120.91 | |  | -195.8 | -185.24 | | -193.89 | | -190.03 | |
| NA |  |  | -40.04 | NA |  |  | -51.87 | -150.64 | | -154.91 | | -144.94 | | -158.69 | |
| NA |  | NA |  | NA |  | NA |  | NA |  | NA |  | NA |  | NA |  |

| NA |  |  | -40.51 | NA |  | -79.69 | -165.87 | | -160.27 | | -162.58 | | -163.69 | |
| --- | --- | --- | --- | --- | --- | --- | --- | --- | --- | --- | --- | --- | --- | --- |
| NA |  |  | -76.55 | NA | -466.89 | | -510.19 | | -547.63 | | -520.16 | | -546.58 | |
| NA |  | NA |  | NA | -273.23 | | -143.68 | | -144.23 | | -130.71 | | -148.64 | |
| NA |  | NA |  | NA | -251.75 | | -113.85 | | -134.54 | | -123.97 | |  | -133.8 |
| NA |  | NA |  | NA | -140.49 | |  | -55.72 |  | -65.73 |  | -63.71 |  | -68.62 |
| NA |  | NA |  | NA |  | -56.6 |  | -52.5 |  | -64.8 |  | -56.73 |  | -55.1 |
| NA |  | NA |  | NA | NA |  |  | -10.88 |  | -11.25 |  | -11.13 |  | -15.61 |
| NA |  | NA |  | NA | -247.83 | | -136.25 | | -152.52 | | -141.68 | | -153.01 | |
| NA |  | NA |  | NA | -111.16 | |  | -65.05 |  | -77.47 |  | -68.68 |  | -80.91 |
| NA |  | NA |  | NA | NA |  | NA |  | NA |  | NA |  | NA |  |
| NA |  | NA |  | NA | NA |  | NA |  | NA |  | NA |  | NA |  |
| NA |  | NA |  | NA | NA |  | NA |  | NA |  | NA |  | NA |  |
| NA |  |  | -11.98 | NA | -749.05 | | -629.22 | | -687.93 | | -615.14 | | -663.59 | |
| NA |  |  | -10.82 | NA | -236.55 | | -226.19 | | -226.65 | | -254.29 | | -250.91 | |
| NA |  | NA |  | NA | NA |  |  | -27.22 |  | -33.89 |  | -28.18 |  | -43.36 |
| NA |  |  | -17.27 | NA | -104.62 | | -143.31 | | -140.49 | | -148.11 | | -141.53 | |
| NA |  | NA |  | NA |  | -5.56 |  | -21.23 |  | -30.92 |  | -24.95 |  | -34.69 |
| NA |  | NA |  | NA | NA |  |  | -20.95 |  | -20.02 |  | -22.69 |  | -28.74 |
| NA |  |  | -14.07 | NA |  | -26.4 |  | -79.54 |  | -78.98 |  | -76.57 |  | -80.93 |
| NA |  | NA |  | NA | NA |  | NA |  | NA |  | NA |  | NA |  |
| NA |  |  | -35.37 | NA |  | -498.9 | -352.89 | | -381.13 | |  | -411.5 | -390.71 | |
| NA |  |  | -6.93 | NA |  | -26.6 |  | -12.01 |  | -10.73 |  | -15.79 |  | -13 |
| NA |  |  | -10.77 | NA | NA |  | NA |  | NA |  | NA |  | NA |  |
| NA |  |  | -18.88 | NA | NA |  | NA |  | NA |  | NA |  | NA |  |
| NA |  | -138.15 | | NA |  | -69.61 | -165.76 | | -169.56 | | -162.83 | | -176.38 | |
| NA |  |  | -19.32 | NA | -202.08 | | -270.72 | | -256.77 | | -260.38 | | -295.96 | |
|  | -9.64 | -124.21 | | NA | -169.25 | | -234.27 | | -229.04 | |  | -222.8 | -248.15 | |
|  | -7.1 |  | -94.11 | NA |  | -64.88 |  | -137.9 |  | -131.6 | -146.51 | | -144.92 | |
| NA |  | NA |  | NA |  | -65.8 |  | -93.48 |  | -92.62 |  | -95.55 |  | -89.4 |
| NA |  | NA |  | NA | -292.57 | | -215.16 | |  | -216.6 | -210.13 | |  | -218.6 |
| NA |  |  | -8.82 | NA | -381.44 | | -317.11 | | -331.61 | | -330.29 | |  | -321.9 |
| NA |  |  | -18.6 | NA | -341.97 | | -259.87 | | -289.56 | | -278.35 | | -287.45 | |
| NA |  |  | -8 | NA | -174.07 | | -129.43 | | -134.04 | | -143.33 | | -133.08 | |
| NA |  |  | -7.68 | NA | -214.36 | | -145.64 | | -161.64 | |  | -146 | -167.41 | |
| NA |  |  | -14.93 | NA |  | -57.12 |  | -36.6 |  | -46.8 |  | -38.82 |  | -45.78 |
| NA |  | NA |  | NA | -594.01 | | -339.87 | | -339.54 | | -340.65 | | -343.42 | |
| NA |  | NA |  | NA | -1380.47 | | -662.53 | | -674.84 | | -639.78 | |  | -652.8 |
| NA |  | NA |  | NA |  | -103.6 | -109.76 | | -116.21 | | -108.99 | | -117.77 | |
| NA |  |  | -23.57 | NA | -268.81 | | -199.47 | | -187.56 | | -208.01 | | -195.43 | |

| NA | NA |  | NA |  | -172.26 | | -165.31 | | -164.86 | | -166.56 | | -158.35 | |
| --- | --- | --- | --- | --- | --- | --- | --- | --- | --- | --- | --- | --- | --- | --- |
| NA |  | -32.4 |  | -6.06 | -407.67 | |  | -415.6 |  | -398.1 | -386.95 | | -410.48 | |
| NA |  | -26.28 | NA |  | -444.01 | | -217.42 | | -241.28 | | -239.13 | | -249.82 | |
| NA |  | -6.22 | NA |  |  | -42.25 |  | -53.38 |  | -59.32 |  | -65.86 |  | -65.28 |
| NA | NA |  | NA |  | -1559.26 | | -563.01 | | -607.05 | | -589.04 | | -615.07 | |
| NA |  | -11.14 | NA |  | NA |  | NA |  | NA |  | NA |  | NA |  |
| NA |  | -23.21 | NA |  |  | -508.7 | -436.78 | | -457.21 | | -428.56 | |  | -443.4 |
| NA |  | -17.28 | NA |  |  | -68.94 |  | -96.33 | -105.25 | | -101.14 | |  | -99.28 |
| NA |  | -17.78 | NA |  | -125.12 | |  | -164.1 | -167.85 | | -161.61 | | -172.54 | |
| NA |  | -10.12 | NA |  | -146.13 | | -127.18 | | -124.85 | | -123.42 | | -121.91 | |
| NA |  | -8.33 | NA |  | -161.38 | | -187.01 | | -200.22 | | -199.59 | | -201.09 | |
| NA | NA |  | NA |  | NA |  | NA |  | NA |  | NA |  | NA |  |
| NA |  | -24 | NA |  | -214.39 | | -176.45 | | -183.76 | | -176.52 | | -176.51 | |
| NA | NA |  | NA |  |  | -45.02 | -114.16 | | -112.74 | | -107.97 | |  | -121.2 |
| NA | NA |  | NA |  | -129.22 | | -211.75 | | -218.02 | | -205.46 | | -216.04 | |
| NA |  | -45.63 | NA |  | -554.38 | | -586.15 | | -627.02 | | -606.18 | | -627.64 | |
| NA |  | -20.04 | NA |  | NA |  |  | -29.99 |  | -26.75 |  | -20.46 |  | -36.92 |
| NA |  | -32.52 | NA |  | -100.47 | | -189.74 | | -219.07 | | -214.48 | | -202.35 | |
| NA |  | -19.58 | NA |  | NA |  | NA |  | NA |  | NA |  | NA |  |
| NA |  | -11.93 | NA |  | -140.36 | |  | -97.54 |  | -89.45 |  | -97.27 |  | -97.63 |
| NA |  | -7.84 | NA |  | -364.18 | | -255.99 | |  | -282.7 | -282.82 | | -309.39 | |
| NA | NA |  | NA |  | -359.23 | | -116.08 | | -103.44 | | -111.76 | | -105.78 | |
| NA |  | -20.54 | NA |  |  | -95.73 | -131.64 | | -150.69 | | -137.88 | | -124.56 | |
| NA | NA |  | NA |  | -632.75 | | -513.83 | | -514.55 | | -514.16 | | -499.29 | |
| NA |  | -8.85 | NA |  |  | -77.01 |  | -147.3 | -151.47 | | -148.13 | | -147.48 | |
| NA | NA |  | NA |  | -587.32 | |  | -274.9 | -282.25 | | -276.96 | | -287.95 | |
| NA |  | -8.47 | NA |  | -272.06 | | -153.17 | | -168.14 | | -150.09 | | -161.99 | |
| NA |  | -10.6 | NA |  | -176.06 | | -283.07 | | -280.41 | | -276.28 | | -281.89 | |
| NA |  | -9.84 | NA |  |  | -96.43 |  | -62.41 |  | -78.79 |  | -70.48 |  | -73.11 |
| NA |  | -8.01 | NA |  |  | -91.27 |  | -36.43 |  | -43.77 |  | -34.29 |  | -44.89 |
| NA | NA |  | NA |  | -711.91 | | -371.58 | | -377.82 | | -368.35 | |  | -376.4 |
| NA |  | -21.26 | NA |  | -255.34 | | -281.68 | | -314.15 | | -289.95 | | -304.19 | |
| NA |  | -18.26 |  | -7.53 | -123.28 | | -177.25 | |  | -187.5 | -168.59 | | -171.71 | |
| NA | NA |  | NA |  | -306.91 | | -208.49 | | -222.95 | | -217.69 | | -239.06 | |
| NA |  | -10.2 | NA |  | NA |  | NA |  | NA |  | NA |  | NA |  |
| NA |  | -19.78 | NA |  |  | -98.18 |  | -84.01 |  | -72.25 |  | -71.04 |  | -81.7 |
| NA |  | -5.5 | NA |  | -197.86 | | -126.14 | |  | -132.9 |  | -144.3 | -141.34 | |
| NA |  | -23 | NA |  |  | -298.1 |  | -286.7 | -281.67 | | -276.14 | | -284.33 | |

| inflammat | | ips..ceehrc | | ips..knih | | islet_cell.. | | kidney..ce | | large_inte | | large_inte | | large_inte | |
| --- | --- | --- | --- | --- | --- | --- | --- | --- | --- | --- | --- | --- | --- | --- | --- |
| -163.59 | |  | -14.26 |  | -51.42 |  | -29.64 |  | -101.6 |  | -56.55 |  | -25.23 |  | -57.39 |
| NA |  | NA |  | NA |  |  | -44.69 | NA |  | NA |  |  | -39.85 | NA |  |
| -189.73 | | NA |  | NA |  | -441.62 | | -268.07 | | -743.93 | |  | -480.6 | -542.45 | |
| -324.46 | |  | -12.11 |  | -50.46 |  | -49.88 |  | -25.48 | -492.58 | | -720.54 | | -607.76 | |
|  | -71.92 | NA |  | NA |  |  | -83.57 |  | -54.03 | -257.78 | |  | -64.04 |  | -84.51 |
| -524.23 | | NA |  |  | -9.47 | -1308.33 | | -621.54 | | -516.53 | | -267.36 | | -578.95 | |
| -236.35 | | NA |  | NA |  | -322.02 | | -272.85 | | -588.19 | | -269.04 | | -315.71 | |
|  | -53.9 | NA |  |  | -5.16 |  | -13.71 |  | -40.21 |  | -83.45 |  | -22.84 |  | -17.07 |
|  | -53.9 | NA |  |  | -5.16 |  | -13.71 |  | -40.21 |  | -83.45 |  | -22.84 |  | -17.07 |
|  | -59.75 | NA |  | NA |  | -112.73 | |  | -63.55 | -154.51 | | -143.71 | | -123.29 | |
| -286.18 | | NA |  | NA |  | -987.96 | | -493.51 | | -354.81 | |  | -67.96 | -225.56 | |
| -117.02 | | NA |  | NA |  |  | -583.6 |  | -131.8 | -265.04 | |  | -45.03 | -164.79 | |
| -357.59 | | NA |  | NA |  |  | -52.88 | -275.06 | | -208.16 | |  | -17.38 |  | -42.72 |
| -375.86 | | NA |  | NA |  | -159.55 | | -385.45 | | -524.13 | | -161.32 | | -260.57 | |
| NA |  | NA |  | NA |  | -207.22 | | NA |  | -923.73 | | -836.09 | | -122.06 | |
| NA |  | NA |  | NA |  | -168.66 | | NA |  |  | -30.53 |  | -96.32 |  | -45.6 |
| NA |  | NA |  | NA |  | -286.05 | | -147.51 | | -292.06 | |  | -118.1 | -144.78 | |
| NA |  | NA |  | NA |  | -286.05 | | -147.51 | | -292.06 | |  | -118.1 | -144.78 | |
| -786.57 | | NA |  | NA |  |  | -55.44 |  | -381.8 | -387.36 | | -309.81 | | -238.52 | |
| -183.46 | | NA |  | NA |  | -467.64 | | -217.45 | | -1024.46 | | -557.26 | | -623.83 | |
| NA |  | NA |  | NA |  |  | -179.4 | NA |  |  | -35.09 |  | -98.71 |  | -52.42 |
| -604.38 | | NA |  | NA |  |  | -59.53 |  | -208.3 |  | -73.77 | NA |  |  | -25.83 |
| NA |  | NA |  | NA |  | NA |  | NA |  | -1256.01 | | -1858.64 | | -1811.39 | |
| NA |  | NA |  | NA |  | NA |  |  | -51.7 | -2417.93 | | -3335.12 | | -3432.56 | |
|  | -45.86 | NA |  | NA |  | -102.39 | | NA |  |  | -75.69 |  | -47.55 |  | -70.32 |
| -299.42 | | NA |  | NA |  | NA |  | NA |  |  | -10.33 |  | -12.6 | NA |  |
| -316.25 | | NA |  | NA |  | NA |  | NA |  |  | -24.95 |  | -30.66 | NA |  |
| -183.44 | | NA |  | NA |  | NA |  | NA |  | NA |  |  | -5.64 | NA |  |
|  | -130.1 | NA |  | NA |  | -516.22 | | -248.92 | | -1014.27 | | -452.18 | | -572.38 | |
|  | -130.1 | NA |  | NA |  | -516.22 | | -248.92 | | -1014.27 | | -452.18 | | -572.38 | |
| -258.66 | |  | -9.92 |  | -7.84 | -721.38 | | -372.99 | | -1102.94 | | -481.12 | | -595.97 | |
| -234.37 | |  | -6.1 |  | -7.08 | -793.73 | | -412.62 | | -1281.91 | | -618.97 | | -751.16 | |
|  | -54 | NA |  | NA |  | -134.65 | |  | -78.84 | -152.01 | |  | -123.6 | -128.42 | |
|  | -54.02 | NA |  | NA |  |  | -60.41 | NA |  |  | -96.09 |  | -164.1 | -110.77 | |
| NA |  | NA |  | NA |  |  | -43.75 | NA |  | NA |  |  | -38.89 | NA |  |
| NA |  | NA |  | NA |  |  | -98.83 | NA |  |  | -32.63 |  | -82.39 |  | -26.15 |
| NA |  | NA |  | NA |  | -148.29 | | NA |  |  | -35.55 |  | -100.8 |  | -44.27 |
| -183.31 | |  | -52.8 | -126.91 | | NA |  |  | -32.46 | NA |  |  | -10.56 | NA |  |

-49.92 NA	-11.7	-8.74	-159.72 NA	NA	NA

-49.92 NA	-11.7	-8.74	-159.72 NA	NA	NA

-49.92 NA	-11.7	-8.74	-159.72 NA	NA	NA

-51.54	-20.42	-67.51 NA	-124	-43.15	-13.38	-44.28

-66.1	-22.48	-88.09 NA	-59.06	-37.3	-26.53	-60.43

| -1332.92 NA | NA |  | -55.72 | -778.49 | -558.34 | -252.62 | -348.22 |
| --- | --- | --- | --- | --- | --- | --- | --- |
| -2034.21 NA | NA |  | -557.55 | -1428.19 | -1783.01 | -1309.96 | -1501.84 |
| -3034.28 NA |  | -7.09 | -518.89 | -1716.17 | -1675.86 | -1121 | -1305.96 |
| -1866.48 NA | NA |  | -435.36 | -1435.16 | -1078.78 | -558.54 | -771.52 |
| -1381.5 NA | NA |  | -499.24 | -1042.97 | -1632.69 | -1188.25 | -1379.48 |
| -1930.78 NA |  | -15.91 | -338.35 | -1052.3 | -1096.56 | -790.31 | -927.15 |
| -2496.31 NA | NA |  | -600.23 | -1450.05 | -2214.69 | -1631.02 | -1857.97 |
| -2088.93 -6.79 -22.21 -606.64 -1461.26 -1027.73 -788.78 -982.63 | | | | | | | |
| -2306.8 NA | NA |  | -567.41 | -1708.73 | -1125.14 | -689.45 | -899.85 |
| -2732.04 NA |  | -29.71 | -634.52 | -1920.92 | -1298.98 | -895.09 | -1119.35 |
| NA NA | NA |  | -211.6 | NA | -51.01 | -123.08 | -75.76 |
| -62.35 NA | NA |  | -141.98 | -99.66 | -470.24 | -183.64 | -184.83 |
| -2703.48 NA | NA |  | -558.65 | -1830.36 | -1115.7 | -730.66 | -903.8 |
| -3047.28 NA |  | -5.83 | -548.38 | -2008.06 | -1259.67 | -745.38 | -924.42 |
| -288.65 NA |  | -8.68 | -74.79 | -244.58 | -100.19 | -56.71 | -70.4 |
| -3457.3 NA | NA |  | -451.8 | -2142.83 | -1562.13 | -873.63 | -1086.73 |
| NA NA | NA |  | -891.11 | -128.73 | -116.2 | -102.22 | -139.03 |
| NA NA | NA |  | -180.16 | NA | -48.76 | -126.54 | -63.16 |
| -3193.75 NA | NA |  | -524.43 | -2194.44 | -1401.96 | -806.04 | -1019.89 |
| -153.23 NA | NA |  | -32.68 | -129.83 | -56.91 | -30.71 | -35.57 |
| -1613.1 NA | NA |  | -102.18 | -876.48 | -560.38 | -376 | -475.09 |
| -3219.91 NA | NA |  | -580.46 | -2149.91 | -1277.02 | -827.72 | -1045.74 |
| -2246.27 NA | NA |  | -451.57 | -1672.31 | -1056.74 | -692.49 | -816.6 |
| -1140.87 NA | NA |  | -418.26 | -818.88 | -537.61 | -430.03 | -544.71 |
| -1140.87 NA | NA |  | -418.26 | -818.88 | -537.61 | -430.03 | -544.71 |
| -3390.04 NA | NA |  | -476.28 | -2118.21 | -1318.84 | -833.67 | -1038.23 |
| -1568.75 NA | NA |  | -133.26 | -937.62 | -295.67 | -191.78 | -265.77 |
| -1152.73 NA | NA |  | NA | -242.97 | -156.98 | -114.86 | -91.18 |
| -969.63 NA | NA |  | -197.67 | -735.88 | -567.33 | -294.2 | -386.3 |
| NA NA | NA |  | -221.01 | -17.91 | -17.56 | -69.37 | -29.4 |
| -140.66 NA | NA |  | -547.08 | -182.09 | -284.94 | -65.28 | -187.85 |
| -2850.92 NA | NA |  | -488.88 | -2022.71 | -1527.93 | -956.28 | -1122.19 |
| -3655.48 NA | NA |  | -520.01 | -2296.92 | -1442.48 | -865.34 | -1090.42 |
| -321.58 NA |  | -10.07 | -86.53 | -271.83 | -104.6 | -61.32 | -73.35 |

| -295.88 | | NA |  | NA |  | -214.1 | -462.01 | | -713.92 | -155.77 | | -266.9 |
| --- | --- | --- | --- | --- | --- | --- | --- | --- | --- | --- | --- | --- |
| -209.17 | | NA |  | NA |  | -350.2 | -445.04 | | -616.35 | -144.72 | | -246.94 |
| -374.23 | | NA |  | NA |  | -274.72 | -496.78 | | -661.02 | -121.43 | | -234.15 |
| -206.92 | | NA |  | NA |  | -36.12 | -203.85 | | -450.89 | -107.91 | | -154.32 |
| NA |  | NA |  | NA |  | -3798.31 |  | -64.07 | -2719.06 | -2185.44 | | -3208.04 |
| NA |  | NA |  | NA |  | -2021.26 |  | -26.73 | -1415.57 | -1205.07 | | -1621.96 |
| NA |  | NA |  | NA |  | -2531.17 |  | -60.43 | -1947.89 | -1651.14 | | -2331.22 |
| NA |  | NA |  | NA |  | -1355.94 |  | -33.4 | -1289.71 | -1135.62 | | -1523.99 |
| NA |  | NA |  | NA |  | -2367.08 | -151.48 | | -2274.69 | -1830.66 | | -2614.3 |
| NA |  | NA |  | NA |  | -2139.04 | -148.44 | | -2016.73 | -1655.6 | | -2388.01 |
| NA |  | NA |  | NA |  | -872.2 |  | -16.26 | -754.67 | -728.93 | | -942.54 |
| NA |  | NA |  | NA |  | -872.2 |  | -16.26 | -754.67 | -728.93 | | -942.54 |
| NA |  | NA |  | NA |  | -872.2 |  | -16.26 | -754.67 | -728.93 | | -942.54 |
| NA |  | NA |  | NA |  | -1951.31 |  | -40.52 | -1728 | -1614.98 | | -2082.98 |
| NA |  | NA |  | NA |  | -2133.13 | -160.56 | | -1949.32 | -1619.46 | | -2360.69 |
| NA |  | NA |  | NA |  | -2211.92 |  | -198.3 | -2027.78 | -1491.91 | | -2203.81 |
| NA |  | NA |  | NA |  | -1147.34 |  | -88.57 | -1105.47 | -1042.37 | | -1339.68 |
| NA |  | NA |  | NA |  | -1134.97 | -107.14 | | -1203.95 | -1151.28 | | -1356.54 |
| NA |  | NA |  | NA |  | -1804.89 | -221.34 | | -1670.06 | -1476.97 | | -2002.75 |
| NA |  | NA |  | NA |  | -2231.35 | -161.66 | | -2035.14 | -1754.3 | | -2423.24 |
| NA |  | NA |  | NA |  | -310.8 | NA |  | -479.31 | -588.57 | | -704.03 |
| -3055.67 | |  | -6.23 |  | -27.38 | -530.94 | -1852.66 | | -1168.26 | -741.22 | | -998.3 |
| NA |  | NA |  | NA |  | -913.4 |  | -19.81 | -344.89 | -321.77 | | -369.44 |
| NA |  | NA |  | NA |  | -913.4 |  | -19.81 | -344.89 | -321.77 | | -369.44 |
| NA |  | NA |  | NA |  | -265.71 | NA |  | -183.29 | -277.34 | | -205.03 |
| NA |  | NA |  | NA |  | -265.71 | NA |  | -183.29 | -277.34 | | -205.03 |
| NA |  | NA |  | NA |  | -1069.6 | NA |  | -343.61 | -549.62 | | -566.41 |
| NA |  | NA |  | NA |  | -2028.52 | NA |  | -835.54 | -1017.39 | | -1072.11 |
| NA |  | NA |  | NA |  | -666.85 | NA |  | -222.73 | -346.01 | | -298.84 |
| NA |  | NA |  | NA |  | -717.44 | NA |  | -234.78 | -362.95 | | -315.69 |
| NA |  | NA |  | NA |  | -717.44 | NA |  | -234.78 | -362.95 | | -315.69 |
| NA |  | NA |  | NA |  | -717.44 | NA |  | -234.78 | -362.95 | | -315.69 |
| NA |  | NA |  | NA |  | -119.92 | NA |  | -38.23 |  | -99.97 | -38.39 |
| NA |  | NA |  | NA |  | -149.94 | NA |  | -30.59 | -103.91 | | -49.01 |
| NA |  | NA |  | NA |  | -169.6 |  | -7.92 | -15.21 |  | -79.36 | -34.24 |
| NA |  | NA |  | NA |  | -170.66 |  | -9.29 | -34.3 | -109.07 | | -34.14 |
|  | -59.72 | NA |  | NA |  | -236.63 | -160.39 | | -64.13 | NA |  | -40.96 |
| -164.93 | |  | -12.37 |  | -22.54 | -27.39 |  | -66.51 | -63.91 |  | -10.17 | -10.98 |
| -164.93 | |  | -12.37 |  | -22.54 | -27.39 |  | -66.51 | -63.91 |  | -10.17 | -10.98 |

| -312.56 | |  | -33.5 | -124.47 | | -150.69 | | -124.36 | | -446.17 | | -282.98 | | -284.12 | |
| --- | --- | --- | --- | --- | --- | --- | --- | --- | --- | --- | --- | --- | --- | --- | --- |
| NA |  | NA |  | NA |  | NA |  | NA |  |  | -43.47 |  | -27.47 |  | -12.37 |
| -189.43 | |  | -20.76 |  | -39.59 | -130.93 | | -111.56 | | -156.44 | |  | -63.32 |  | -88.28 |
|  | -47.86 | NA |  | NA |  |  | -25.23 |  | -21.73 | NA |  | NA |  | NA |  |
| -195.29 | | NA |  | NA |  |  | -70.04 |  | -208.7 |  | -99.88 |  | -22.52 |  | -57.62 |
|  | -68.68 | NA |  | NA |  | NA |  | NA |  |  | -26.94 |  | -69.12 |  | -30.93 |
| NA |  | NA |  | NA |  | -1388.49 | | -133.87 | | -277.42 | | -422.37 | | -407.57 | |
| NA |  | NA |  | NA |  | -1822.81 | | -239.39 | | -311.56 | | -451.94 | | -474.35 | |
|  | -10.23 | NA |  | NA |  | -571.95 | | -875.05 | | -1291.03 | | -1193.05 | | -1561.29 | |
| NA |  | NA |  | NA |  | -626.72 | | -956.37 | | -1339.72 | | -1247.09 | | -1586.01 | |
|  | -47.19 | NA |  | NA |  | -688.13 | | -888.37 | | -1006.44 | | -828.09 | | -1258.82 | |
| NA |  | NA |  | NA |  |  | -236.9 |  | -16.41 |  | -30.25 |  | -93.11 |  | -51.23 |
| NA |  | NA |  | NA |  |  | -28.58 |  | -32.58 | -2010.59 | | -2845.51 | | -2947.4 | |
| NA |  | NA |  | NA |  | NA |  | NA |  | -1111.78 | | -1644.82 | | -1857.67 | |
| NA |  | NA |  | NA |  | NA |  | NA |  | -1220.37 | |  | -1612 | -2264.29 | |
| NA |  | NA |  | NA |  | NA |  | NA |  | -390.66 | | -454.54 | | -830.14 | |
| NA |  | NA |  | NA |  | -195.73 | |  | -14.2 |  | -16.11 |  | -70.42 |  | -31.1 |
| NA |  | NA |  | NA |  | -350.22 | |  | -91.11 |  | -27.82 |  | -23.99 |  | -16.3 |
| NA |  | NA |  | NA |  | -295.03 | |  | -26.48 |  | -21.95 |  | -45.85 |  | -28 |
| NA |  | NA |  | NA |  |  | -511.3 | -445.68 | | -425.82 | | -444.13 | | -473.62 | |
| NA |  | NA |  | NA |  |  | -64 |  | -15.52 | -1108.47 | | -1594.22 | | -1910.27 | |
| NA |  | NA |  | NA |  |  | -228.3 |  | -33.64 |  | -61.03 |  | -97.62 |  | -62.51 |
| NA |  | NA |  | NA |  | -304.65 | |  | -63.44 |  | -34.24 |  | -93.9 |  | -78.55 |
| NA |  | NA |  | NA |  | -348.22 | |  | -75.02 |  | -23.34 |  | -40.98 |  | -26.68 |
| NA |  | NA |  | NA |  |  | -52.79 |  | -72.75 | -2152.99 | | -2911.91 | | -3118.84 | |
| NA |  | NA |  | NA |  | -464.36 | | -496.58 | | -944.48 | | -1001.59 | | -1122.48 | |
| NA |  | NA |  | NA |  |  | -18.26 |  | -56.59 | -1161.98 | | -1388.38 | | -2204.76 | |
| NA |  | NA |  | NA |  |  | -44.72 |  | -63.81 | -1091.19 | | -1054.86 | | -1806.72 | |
| NA |  | NA |  | NA |  | -171.43 | |  | -9.1 |  | -11.22 |  | -62.31 |  | -23.6 |
| NA |  | NA |  | NA |  | -173.76 | |  | -8.81 |  | -11.42 |  | -64.16 |  | -24.21 |
| NA |  | NA |  | NA |  | -1372.58 | | -298.91 | |  | -71.6 |  | -22.83 |  | -39.8 |
| NA |  | NA |  | NA |  | -185.36 | |  | -12.76 |  | -19.27 |  | -74.37 |  | -35.04 |
| NA |  | NA |  | NA |  | -954.19 | | -180.81 | |  | -91.67 | -109.56 | |  | -72.62 |
| NA |  | NA |  | NA |  | -751.81 | |  | -92.15 |  | -22.71 |  | -47.16 |  | -24.2 |
| NA |  | NA |  | NA |  |  | -32.65 |  | -64.35 | -1918.39 | | -2594.22 | |  | -2883 |
| NA |  | NA |  | NA |  | -210.54 | | -164.37 | | -1825.63 | | -2225.89 | | -2642.64 | |
| NA |  | NA |  | NA |  |  | -11.07 |  | -35.63 | -2105.53 | | -2916.79 | | -3103.81 | |
| NA |  | NA |  | NA |  | -109.12 | |  | -115.1 | -1951.47 | | -2388.01 | | -2893.18 | |
| NA |  | NA |  | NA |  |  | -50.28 |  | -43.11 | -1037.94 | | -948.61 | | -1626.61 | |

| NA |  | NA |  | NA |  | -370.19 | |  | -94.05 |  | -15.67 |  | -28.34 |  | -22.47 |
| --- | --- | --- | --- | --- | --- | --- | --- | --- | --- | --- | --- | --- | --- | --- | --- |
| NA |  | NA |  | NA |  |  | -451.4 | -532.04 | | -500.75 | | -496.36 | | -575.97 | |
| NA |  | NA |  | NA |  | -178.41 | |  | -6.14 |  | -16.88 |  | -73.58 |  | -30.85 |
| NA |  | NA |  | NA |  | NA |  | NA |  | -1047.57 | | -1566.45 | | -1725.12 | |
| NA |  | NA |  | NA |  |  | -14.67 | NA |  | -1306.34 | | -1985.46 | | -1944.8 | |
| NA |  | NA |  | NA |  | -201.39 | |  | -159.2 | -1955.33 | | -2321.36 | | -2818.62 | |
| NA |  | NA |  | NA |  | NA |  |  | -11.17 | -1417.19 | | -1784.5 | | -2436.96 | |
| NA |  | NA |  | NA |  |  | -59.61 |  | -71.13 | -1103.63 | | -1248.93 | | -2095.5 | |
| NA |  | NA |  | NA |  | -197.76 | |  | -15.82 |  | -22.15 |  | -76.59 |  | -38.29 |
| NA |  | NA |  | NA |  | -104.24 | | -143.24 | | -2122.13 | | -2419.42 | | -3144.57 | |
| -325.59 | | NA |  | NA |  |  | -65.4 | -241.35 | | -185.14 | |  | -80.66 | -118.97 | |
| NA |  | NA |  | NA |  | -713.62 | | -329.45 | | -1016.01 | | -962.39 | | -919.42 | |
| NA |  | NA |  | NA |  | NA |  |  | -22.33 |  | -121.5 | -228.95 | |  | -54.28 |
|  | -87.6 | NA |  | NA |  |  | -20.15 | -177.33 | | -285.11 | | -357.31 | | -203.26 | |
| -123.59 | | NA |  | NA |  |  | -71.76 | -240.07 | | -320.93 | | -276.32 | | -216.41 | |
|  | -79.28 | NA |  | NA |  |  | -17.08 | -105.44 | | -131.08 | | -136.27 | |  | -94.72 |
|  | -41.24 | NA |  | NA |  | NA |  | NA |  |  | -60.66 |  | -90.93 |  | -16.69 |
| NA |  | NA |  | NA |  |  | -51.88 |  | -72.52 | -244.49 | | -284.41 | | -155.23 | |
| -246.71 | | NA |  | NA |  |  | -90.8 | -270.99 | |  | -327.3 | -319.16 | |  | -216.1 |
|  | -11.01 | NA |  | NA |  | NA |  |  | -93.35 | -158.11 | | -168.21 | |  | -75.09 |
| -194.42 | | NA |  | NA |  | -183.87 | | -378.95 | | -706.97 | | -149.52 | | -260.22 | |
| -259.82 | | NA |  | NA |  | -152.33 | | -355.66 | | -527.92 | |  | -98.56 | -159.89 | |
|  | -324.3 | NA |  | NA |  | -245.45 | | -368.04 | | -677.87 | | -169.16 | | -276.42 | |
| -111.45 | | NA |  | NA |  |  | -63.05 | -174.15 | | -451.83 | | -146.93 | | -171.58 | |
| NA |  | NA |  | NA |  | NA |  | NA |  | -792.12 | | -383.97 | | -757.79 | |
|  | -31.86 | NA |  | NA |  |  | -17.83 |  | -84.68 | NA |  | NA |  | NA |  |
|  | -69.24 |  | -10.04 |  | -28.05 | NA |  | NA |  | -923.23 | | -559.47 | | -889.89 | |
|  | -46.72 | NA |  |  | -16.02 | NA |  | NA |  |  | -348.2 | -209.05 | | -313.97 | |
|  | -46.72 | NA |  |  | -16.02 | NA |  | NA |  |  | -348.2 | -209.05 | | -313.97 | |
|  | -18.34 |  | -22.16 |  | -55.97 | NA |  | NA |  |  | -286.1 | -250.97 | | -361.31 | |
|  | -24.15 |  | -8.16 |  | -28.31 | NA |  |  | -19.84 | -167.31 | |  | -47.98 |  | -157.3 |
|  | -8.09 | NA |  |  | -5.54 | NA |  | NA |  |  | -795.2 | -426.52 | | -770.09 | |
|  | -8.09 | NA |  |  | -5.54 | NA |  | NA |  |  | -795.2 | -426.52 | | -770.09 | |
|  | -8.09 | NA |  |  | -5.54 | NA |  | NA |  |  | -795.2 | -426.52 | | -770.09 | |
|  | -42.91 |  | -18.13 |  | -16.32 | NA |  |  | -45.11 |  | -400.2 | -151.72 | | -348.57 | |
| -184.44 | |  | -31.1 |  | -95.12 | NA |  |  | -99.1 | NA |  | NA |  | NA |  |
| NA |  | NA |  |  | -5.77 | NA |  | NA |  | -870.56 | | -607.99 | | -897.81 | |
| NA |  | NA |  | NA |  | -166.25 | |  | -38.32 |  | -15.57 |  | -36.98 |  | -36.17 |
| NA |  | NA |  | NA |  |  | -171.8 |  | -91.98 | -491.38 | | -547.97 | | -446.98 | |

| NA |  | NA |  | NA |  | -213.47 | -17.62 |  | -45.59 |  | -61.73 |  | -65.38 |
| --- | --- | --- | --- | --- | --- | --- | --- | --- | --- | --- | --- | --- | --- |
| NA |  | NA |  | NA |  | -303.98 | -9.87 |  | -24.27 |  | -76.1 |  | -35.06 |
| NA |  | NA |  | NA |  | -153.68 | -5.85 |  | -30.32 |  | -96.25 |  | -46.43 |
| NA |  | NA |  | NA |  | -268.3 | -19.2 |  | -50.8 |  | -94.59 |  | -82.79 |
|  | -360.7 | NA |  | NA |  | -450.37 | -338.27 |  | -99.59 |  | -32.07 | -115.29 | |
| -262.41 | | NA |  | NA |  | -653.89 | -471.88 | -131.89 | |  | -32.59 | -104.05 | |
| -114.95 | | NA |  | NA |  | -64.58 | -206.33 | NA |  | NA |  | NA |  |
| -369.82 | | NA |  | NA |  | -637.47 | -438.07 | -161.14 | |  | -72.89 | -147.16 | |
| -182.31 | | NA |  | NA |  | -14.28 | -80.01 | NA |  | NA |  | NA |  |
| -220.35 | | NA |  | NA |  | -117.4 | -126.83 | NA |  | NA |  | NA |  |
| -206.74 | | NA |  | NA |  | -340.53 | -213.88 | -694.94 | | -301.17 | | -408.14 | |
|  | -380 |  | -56.47 | -170.99 | | -104.93 | -204.83 |  | -47.55 | -121.61 | | -163.45 | |
| -514.32 | |  | -39.17 | -145.22 | | -391.52 | -455.01 | -257.86 | | -312.23 | |  | -340.9 |
|  | -25.78 | NA |  | NA |  | -591.74 | -152.07 | -194.23 | |  | -64.5 | -155.04 | |
| NA |  | NA |  | NA |  | -318.48 | -58.09 |  | -70.28 |  | -88.51 |  | -77.06 |
| NA |  | NA |  | NA |  | -223.97 | -23.43 |  | -22.22 |  | -73.79 |  | -38.52 |
| -110.62 | | NA |  | NA |  | -412.2 | -125.17 | -1000.55 | | -498.04 | |  | -557.5 |
| NA |  | NA |  | NA |  | -194.98 | -10.57 |  | -27.24 |  | -95.62 |  | -56.07 |
| -207.45 | | NA |  | NA |  | -1124.79 | -380.89 |  | -553.4 | -283.82 | | -587.22 | |
| NA |  | NA |  | NA |  | -201.3 | -14.77 | -111.03 | | -155.94 | | -141.56 | |
| NA |  | NA |  | NA |  | -201.3 | -14.77 | -111.03 | | -155.94 | | -141.56 | |
| -143.01 | | NA |  |  | -20.7 | -281.69 | -146.2 | -255.05 | | -166.82 | | -285.86 | |
| -194.24 | | NA |  | NA |  | -347.84 | -262.26 | -807.27 | | -311.75 | | -378.76 | |
| -194.24 | | NA |  | NA |  | -347.84 | -262.26 | -807.27 | | -311.75 | | -378.76 | |
| -194.24 | | NA |  | NA |  | -347.84 | -262.26 | -807.27 | | -311.75 | | -378.76 | |
| -194.24 | | NA |  | NA |  | -347.84 | -262.26 | -807.27 | | -311.75 | | -378.76 | |
| -207.66 | | NA |  | NA |  | -374.34 | -341.73 | -195.05 | |  | -84.43 | -172.32 | |
| -207.66 | | NA |  | NA |  | -374.34 | -341.73 | -195.05 | |  | -84.43 | -172.32 | |
| -207.66 | | NA |  | NA |  | -374.34 | -341.73 | -195.05 | |  | -84.43 | -172.32 | |
| -207.66 | | NA |  | NA |  | -374.34 | -341.73 | -195.05 | |  | -84.43 | -172.32 | |
|  | -64.61 | NA |  | NA |  | -1191.22 | -515.67 | -1393.85 | | -1242.67 | | -1301.98 | |
|  | -64.61 | NA |  | NA |  | -1191.22 | -515.67 | -1393.85 | | -1242.67 | | -1301.98 | |
|  | -40.38 | NA |  | NA |  | -427.64 | -174.5 | -662.78 | | -550.72 | | -618.52 | |
|  | -40.38 | NA |  | NA |  | -427.64 | -174.5 | -662.78 | | -550.72 | | -618.52 | |
| -358.43 | | NA |  |  | -15.74 | -505.33 | -378.65 | -1192.5 | | -575.97 | | -639.82 | |
| -309.14 | | NA |  | NA |  | -993.49 | -422.2 | -428.43 | | -195.32 | | -471.72 | |
| -309.14 | | NA |  | NA |  | -993.49 | -422.2 | -428.43 | | -195.32 | | -471.72 | |
| -188.19 | | NA |  | NA |  | -1215.21 | -381.05 | -611.64 | | -376.46 | | -691.36 | |
| -188.19 | | NA |  | NA |  | -1215.21 | -381.05 | -611.64 | | -376.46 | | -691.36 | |

| -239.57 | | NA |  | NA |  | -809.38 | | -234.41 | | -386.61 | | -168.19 | | -371.68 | |
| --- | --- | --- | --- | --- | --- | --- | --- | --- | --- | --- | --- | --- | --- | --- | --- |
| -413.37 | | NA |  |  | -7.3 | -1236.03 | | -586.23 | | -473.89 | | -217.74 | | -500.24 | |
| -195.18 | | NA |  | NA |  | -910.02 | | -283.23 | | -370.79 | |  | -78.98 | -257.56 | |
|  | -39.17 | NA |  | NA |  | -219.44 | |  | -43.02 | -118.74 | |  | -10.77 |  | -43.65 |
| -307.47 | | NA |  | NA |  | -332.13 | | -366.89 | | -279.93 | |  | -58.55 | -117.05 | |
|  | -149.8 | NA |  | NA |  | -113.65 | | -297.39 | | -636.81 | | -153.51 | | -246.97 | |
| -387.86 | | NA |  | NA |  | -192.09 | | -293.54 | | -170.03 | |  | -15.2 |  | -68.48 |
| -205.75 | | NA |  | NA |  | -2732.93 | | -2373.54 | | -1059.93 | | -575.59 | | -867.23 | |
| -267.81 | | NA |  | NA |  | -3040.38 | | -2708.39 | | -1175.57 | | -621.68 | |  | -940.2 |
|  | -19.36 | NA |  | NA |  | -365.99 | | -186.03 | | -112.41 | |  | -51.03 |  | -76.08 |
| -248.79 | | NA |  | NA |  | -3265.99 | | -2884.8 | | -1343.66 | | -733.44 | | -1107.53 | |
| -108.52 | | NA |  | NA |  | -1942.43 | | -778.59 | | -685.78 | | -432.46 | | -590.19 | |
| NA |  | NA |  |  | -8.26 | NA |  | NA |  | NA |  | NA |  | NA |  |
|  | -83.64 | NA |  | NA |  | NA |  | -138.24 | | NA |  | NA |  | NA |  |
| NA |  | NA |  | NA |  | NA |  | NA |  | NA |  | NA |  | NA |  |
| NA |  | NA |  | NA |  | NA |  | NA |  | NA |  | NA |  | NA |  |
| NA |  | NA |  | NA |  | NA |  | NA |  | NA |  | NA |  | NA |  |
| NA |  | NA |  | NA |  |  | -27.17 |  | -19.17 | NA |  | NA |  | NA |  |
| NA |  | NA |  | NA |  | NA |  | NA |  | NA |  | NA |  | NA |  |
| NA |  | NA |  | NA |  | NA |  | NA |  | NA |  | NA |  | NA |  |
| NA |  | NA |  | NA |  | NA |  | NA |  | NA |  | NA |  | NA |  |
| NA |  | NA |  | NA |  | NA |  | NA |  | NA |  | NA |  | NA |  |
| NA |  | NA |  | NA |  |  | -9.3 | NA |  | NA |  | NA |  | NA |  |
| NA |  | NA |  | NA |  |  | -18.52 | NA |  |  | -10.01 | NA |  |  | -6.14 |
|  | -21.02 | NA |  | NA |  |  | -11.6 |  | -12.22 |  | -16.69 | NA |  |  | -5.13 |
| NA |  | NA |  | NA |  | -190.64 | |  | -11.41 |  | -20.16 |  | -81.54 |  | -41.26 |
| NA |  | NA |  | NA |  | -215.42 | |  | -18.61 |  | -18.32 |  | -76.92 |  | -37.86 |
| NA |  | NA |  | NA |  |  | -190.1 | NA |  |  | -41.69 | -109.78 | |  | -62.5 |
| -258.49 | | NA |  | NA |  | -628.42 | | -389.01 | | -1289.13 | | -627.09 | | -720.84 | |
|  | -96.4 |  | -9.23 |  | -21.02 |  | -14.54 |  | -205.4 |  | -62 |  | -18.66 |  | -29.44 |
|  | -96.4 |  | -9.23 |  | -21.02 |  | -14.54 |  | -205.4 |  | -62 |  | -18.66 |  | -29.44 |
| NA |  | NA |  | NA |  | -102.66 | | -147.39 | |  | -255.3 |  | -235.7 | -257.64 | |
| NA |  | NA |  | NA |  |  | -60.31 | -181.77 | |  | -9.91 | NA |  | NA |  |
| NA |  | NA |  | NA |  |  | -35.56 | -319.12 | | -121.96 | |  | -59.39 |  | -78.87 |
| NA |  | NA |  | NA |  |  | -83.18 | -497.86 | |  | -395.2 | -291.19 | | -413.37 | |
| -599.03 | |  | -5.89 |  | -15.98 |  | -48.08 |  | -304 | -112.25 | |  | -61.97 |  | -77.04 |
| NA |  | NA |  | NA |  | -363.56 | | -708.23 | | -723.64 | | -613.95 | | -882.98 | |
| NA |  | NA |  | NA |  | NA |  |  | -82.63 |  | -14.3 |  | -21.59 |  | -5.49 |
| NA |  | NA |  | NA |  | -287.49 | | -700.72 | | -479.25 | | -339.44 | | -521.31 | |

| NA |  | NA |  | NA |  | -337.09 | | -522.83 | | -533.44 | | -464.76 | | -579.75 | |
| --- | --- | --- | --- | --- | --- | --- | --- | --- | --- | --- | --- | --- | --- | --- | --- |
| NA |  | NA |  | NA |  | -908.17 | | -329.06 | | -259.22 | | -210.99 | | -317.05 | |
| NA |  | NA |  | NA |  | -550.85 | | -347.67 | | -321.25 | | -269.11 | | -341.18 | |
| NA |  | NA |  | NA |  | -642.88 | | -288.41 | | -217.01 | | -194.76 | | -267.22 | |
|  | -7.91 | NA |  | NA |  | -1205.04 | | -142.58 | |  | -75.13 |  | -36.1 |  | -95.2 |
| -241.42 | | NA |  | NA |  |  | -788.8 | -508.97 | | -175.69 | |  | -58.59 | -140.15 | |
| NA |  | NA |  | NA |  | -1003.03 | | NA |  | NA |  |  | -12.37 | NA |  |
| NA |  | NA |  | NA |  | -1003.03 | | NA |  | NA |  |  | -12.37 | NA |  |
| NA |  | NA |  | NA |  | -1003.03 | | NA |  | NA |  |  | -12.37 | NA |  |
| NA |  | NA |  | NA |  |  | -46.13 | NA |  | NA |  |  | -39.75 | NA |  |
|  | -58.36 | NA |  | NA |  |  | -59.03 | -490.83 | |  | -12.95 | NA |  |  | -10.27 |
|  | -29.87 | NA |  | NA |  |  | -72.01 | -372.21 | |  | -18.04 | NA |  | NA |  |
| -197.32 | | NA |  | NA |  | -385.03 | | -426.07 | | -248.57 | |  | -97.13 |  | -169.5 |
| NA |  | NA |  | NA |  |  | -32.31 | NA |  | NA |  | NA |  | NA |  |
|  | -49.19 | NA |  | NA |  | NA |  | -357.27 | | NA |  | NA |  | NA |  |
| NA |  | NA |  | NA |  | -160.05 | | -379.44 | |  | -63.16 |  | -62.77 |  | -64.17 |
|  | -34.87 | NA |  | NA |  | -136.27 | | -202.81 | | -118.33 | |  | -33.62 |  | -51.2 |
|  | -45.41 | NA |  | NA |  | NA |  | -141.58 | | NA |  | NA |  | NA |  |
| NA |  | NA |  | NA |  | -134.89 | |  | -12.7 | NA |  |  | -6.14 | NA |  |
| -140.11 | |  | -20.4 |  | -48.71 | NA |  | NA |  | NA |  | NA |  | NA |  |
|  | -194.4 |  | -20.01 |  | -77.63 | NA |  |  | -93.92 | NA |  | NA |  | NA |  |
|  | -17.13 | NA |  | NA |  | -266.69 | | -378.68 | | -288.06 | | -192.55 | | -297.22 | |
|  | -16.58 | NA |  | NA |  | -256.63 | | -668.18 | | -608.02 | | -470.61 | |  | -656.1 |
| -595.67 | | NA |  | NA |  | -167.15 | |  | -24.62 |  | -23.22 | -430.01 | | -434.32 | |
|  | -94.04 | NA |  | NA |  |  | -275 | -208.59 | |  | -82.26 |  | -15.68 |  | -66.39 |
|  | -13.3 | NA |  | NA |  |  | -62.39 | -156.41 | |  | -44.57 | NA |  |  | -20.93 |
|  | -97.62 | NA |  | NA |  | NA |  | -160.46 | |  | -5.55 | NA |  | NA |  |
|  | -32.57 | NA |  | NA |  | NA |  | -117.18 | | NA |  | NA |  | NA |  |
|  | -189.4 | NA |  | NA |  | -466.22 | | -201.59 | | -142.97 | |  | -63.94 | -130.39 | |
|  | -51.33 | NA |  | NA |  | -110.28 | |  | -57.83 |  | -8.14 | NA |  | NA |  |
|  | -30.04 | NA |  | NA |  | -418.23 | | -107.03 | | -130.93 | |  | -68.11 | -120.38 | |
|  | -53.46 | NA |  | NA |  | -309.53 | |  | -89.04 |  | -68.49 |  | -22.5 |  | -49.52 |
|  | -64.73 | NA |  | NA |  | -471.24 | | -151.76 | | -134.53 | |  | -84.96 | -157.06 | |
|  | -65.18 | NA |  | NA |  | -209.35 | | -138.18 | |  | -16.56 | NA |  | NA |  |
|  | -23.37 | NA |  | NA |  | -391.59 | | -100.85 | |  | -95.11 |  | -60.77 |  | -97.36 |
| NA |  | NA |  | NA |  |  | -169.5 |  | -23.59 |  | -36.32 |  | -20.74 |  | -38.93 |
| NA |  | NA |  | NA |  | NA |  | NA |  | NA |  | NA |  | NA |  |
| NA |  | NA |  | NA |  | NA |  | NA |  | NA |  | NA |  | NA |  |
| NA |  | NA |  | NA |  | NA |  | NA |  | NA |  | NA |  | NA |  |

| -428.73 | | NA |  | NA |  | NA |  |  | -44.43 |  | -39.11 | NA |  | NA |  |
| --- | --- | --- | --- | --- | --- | --- | --- | --- | --- | --- | --- | --- | --- | --- | --- |
|  | -618.8 | NA |  | NA |  | NA |  |  | -74.88 |  | -61.08 | NA |  |  | -8.77 |
| -292.03 | | NA |  | NA |  | NA |  |  | -18.86 | NA |  | NA |  | NA |  |
|  | -7.88 | NA |  | NA |  | -233.99 | | -686.93 | |  | -582.3 | -482.91 | | -655.55 | |
| NA |  | NA |  | NA |  | -133.39 | | -369.07 | | -121.86 | | -104.38 | | -135.08 | |
| NA |  | NA |  | NA |  | -220.03 | | -455.71 | | -576.11 | | -529.68 | | -666.78 | |
| NA |  | NA |  | NA |  | -203.09 | | -543.57 | | -610.21 | |  | -589.8 | -715.64 | |
| NA |  | NA |  | NA |  | -184.63 | | -198.45 | |  | -59.71 |  | -23.56 |  | -37.57 |
|  | -97.03 | NA |  | NA |  |  | -81.29 | -110.24 | |  | -7.54 | NA |  | NA |  |
|  | -79.83 | NA |  | NA |  |  | -325.6 | -170.96 | |  | -87.82 |  | -12.54 |  | -65.21 |
| -108.18 | | NA |  | NA |  | -383.76 | | -305.15 | |  | -92.92 |  | -19.85 |  | -80.5 |
| -108.18 | | NA |  | NA |  | -383.76 | | -305.15 | |  | -92.92 |  | -19.85 |  | -80.5 |
| -139.54 | | NA |  | NA |  | -288.77 | | -240.67 | | -149.88 | |  | -51.74 | -116.52 | |
| -214.71 | | NA |  | NA |  |  | -24.76 | -174.65 | | -296.61 | |  | -25.63 |  | -54.42 |
| -214.71 | | NA |  | NA |  |  | -24.76 | -174.65 | | -296.61 | |  | -25.63 |  | -54.42 |
| NA |  | NA |  | NA |  | -273.96 | |  | -25.43 | -215.21 | | -263.05 | | -308.39 | |
| NA |  | NA |  | NA |  |  | -60.86 |  | -5.48 | -150.34 | | -238.52 | | -178.05 | |
| NA |  | NA |  | NA |  | -287.54 | | NA |  | -230.45 | | -270.96 | | -321.97 | |
|  | -9.32 |  | -17.17 |  | -36.94 | NA |  | NA |  | -198.11 | | -101.76 | | -189.05 | |
| -1129.18 | | NA |  | NA |  | -414.31 | | -974.21 | | -859.64 | | -512.02 | | -642.35 | |
| -1526.77 | | NA |  | NA |  |  | -30.47 | -263.78 | | -485.99 | | -321.62 | | -316.91 | |
|  | -401.5 | NA |  | NA |  | NA |  | -121.51 | | -156.51 | | -124.98 | |  | -71.79 |
| -1140.95 | | NA |  | NA |  | -137.55 | | -525.66 | | -398.85 | | -257.05 | |  | -292.2 |
| -2346.72 | | NA |  | NA |  |  | -83.96 | -478.99 | | -926.13 | | -647.95 | | -630.02 | |
| -1343.45 | | NA |  | NA |  |  | -60.69 | -351.81 | | -721.63 | | -536.09 | | -505.14 | |
|  | -84.8 | NA |  | NA |  |  | -287.6 | -318.37 | | -171.79 | |  | -96.5 | -145.41 | |
| -114.26 | | NA |  | NA |  |  | -63.52 | -226.58 | | -448.24 | | -496.96 | | -297.43 | |
|  | -76.91 | NA |  | NA |  | -368.69 | | -451.27 | | -322.32 | | -146.44 | | -270.24 | |
|  | -8.76 | NA |  | NA |  | -244.61 | |  | -38.37 | -115.35 | |  | -8.52 |  | -48.49 |
|  | -301 |  | -7.87 |  | -27.45 | -684.95 | | -341.55 | | -265.09 | |  | -66.29 | -242.69 | |
| -223.75 | | NA |  | NA |  | -1161.48 | | -476.92 | |  | -615.3 | -314.62 | | -559.13 | |
| NA |  | NA |  | NA |  | -207.11 | | NA |  | -178.68 | |  | -83.76 | -180.39 | |
| NA |  | NA |  | NA |  | -188.78 | |  | -91.59 | -533.48 | | -630.39 | | -555.18 | |
| NA |  | NA |  | NA |  | -339.26 | | -204.52 | |  | -70.58 |  | -31.36 |  | -77.21 |
| NA |  | NA |  | NA |  | -226.93 | | -106.07 | |  | -20.36 |  | -10.96 |  | -23.45 |
|  | -143.9 |  | -16.94 |  | -55.57 | NA |  |  | -21.38 | NA |  | NA |  | NA |  |
|  | -245.3 |  | -17.01 |  | -63.26 |  | -27.33 | -230.06 | | NA |  | NA |  | NA |  |
| -257.51 | |  | -34.1 | -130.58 | | NA |  |  | -81.79 | NA |  | NA |  | NA |  |
| NA |  |  | -29.46 |  | -59.89 | NA |  | NA |  | NA |  | NA |  | NA |  |

| -260.22 | |  | -27.06 |  | -76.32 |  | -12.53 |  | -157.2 | NA |  | NA |  | NA |  |
| --- | --- | --- | --- | --- | --- | --- | --- | --- | --- | --- | --- | --- | --- | --- | --- |
| -456.84 | | NA |  |  | -23.45 | -855.45 | | -595.58 | | -232.01 | |  | -66.85 | -208.05 | |
| -186.72 | | NA |  | NA |  | -362.95 | | -230.93 | | -723.43 | | -303.18 | | -374.94 | |
| -154.98 | | NA |  | NA |  | -263.45 | | -199.62 | | -543.68 | | -209.18 | |  | -245.5 |
| -114.75 | | NA |  | NA |  | -170.63 | | -118.85 | |  | -296.8 | -106.22 | | -135.92 | |
|  | -41.49 | NA |  | NA |  | -391.14 | |  | -90.87 | -140.23 | |  | -36.7 |  | -86.6 |
|  | -49 | NA |  | NA |  | -461.78 | | -474.28 | | -120.09 | |  | -49.78 |  | -68.27 |
| -187.91 | | NA |  | NA |  |  | -317.7 | -209.58 | | -525.68 | |  | -194 | -264.77 | |
| -132.93 | | NA |  | NA |  | -168.22 | | -104.78 | | -378.87 | | -117.67 | | -157.33 | |
| NA |  | NA |  | NA |  |  | -229.1 |  | -56.37 |  | -7.44 |  | -20.17 |  | -7.52 |
| NA |  | NA |  | NA |  | -259.77 | |  | -71.83 |  | -11.71 |  | -26.71 |  | -16.62 |
| NA |  | NA |  | NA |  |  | -81.16 | NA |  | NA |  |  | -10.7 | NA |  |
| -668.71 | |  | -36.94 | -147.54 | | -237.04 | | -102.63 | | -114.01 | | -555.86 | |  | -558.6 |
| -271.37 | |  | -12.88 |  | -37.96 |  | -83.57 | -148.27 | | -189.77 | |  | -84.52 | -128.19 | |
|  | -13.12 | NA |  | NA |  |  | -62.45 |  | -6.65 |  | -40.35 | NA |  |  | -5.58 |
| -130.74 | |  | -18.56 |  | -14.97 |  | -49.28 | -103.37 | |  | -65.76 |  | -31.99 |  | -23.02 |
|  | -6.96 | NA |  | NA |  |  | -54.19 | NA |  |  | -34.55 | NA |  | NA |  |
|  | -17.3 |  | -16.25 |  | -64.19 | NA |  | NA |  | NA |  | NA |  | NA |  |
| -121.75 | |  | -12.05 |  | -53.78 | NA |  |  | -36 |  | -5.96 | NA |  | NA |  |
| NA |  | NA |  | NA |  | -271.49 | |  | -10.35 |  | -20.9 |  | -77.59 |  | -31.33 |
| -462.68 | |  | -22.38 |  | -52.29 | -573.27 | | -433.61 | |  | -300 | -243.69 | | -308.94 | |
| NA |  | NA |  | NA |  | -125.62 | | -108.04 | |  | -82.45 |  | -39.22 |  | -47.41 |
|  | -49.69 |  | -15.2 |  | -69.73 | NA |  | NA |  | NA |  | NA |  | NA |  |
|  | -38.71 |  | -7.26 |  | -19.01 | NA |  | NA |  | NA |  | NA |  | NA |  |
| -184.16 | |  | -17.89 |  | -49.35 | NA |  |  | -5.7 | NA |  | NA |  | NA |  |
|  | -76.33 |  | -19.42 |  | -41.06 | NA |  |  | -43.13 | NA |  | NA |  | NA |  |
| -177.15 | |  | -27.34 |  | -64.13 |  | -18.14 |  | -67.41 | NA |  | NA |  | NA |  |
| -190.62 | |  | -17.45 |  | -28.96 | NA |  |  | -33.53 | NA |  | NA |  | NA |  |
|  | -19.88 | NA |  | NA |  | NA |  |  | -10.46 | NA |  | NA |  | NA |  |
|  | -193.8 | NA |  | NA |  | -166.21 | |  | -96.25 |  | -215.7 | -301.15 | | -287.64 | |
|  | -92.39 |  | -10.64 |  | -10.52 |  | -11.41 |  | -97.82 | -167.77 | |  | -98.76 | -101.33 | |
| -294.93 | |  | -10.62 |  | -23.03 | -286.15 | | -297.02 | | -115.49 | |  | -65.47 | -109.12 | |
|  | -60.85 | NA |  | NA |  |  | -67.44 | -107.78 | |  | -22.72 | NA |  |  | -11.99 |
|  | -36.1 | NA |  | NA |  | -211.46 | | -147.86 | |  | -64.05 | NA |  |  | -43.25 |
| -135.83 | |  | -30.96 |  | -83.6 | NA |  | NA |  | NA |  |  | -36.2 |  | -6.83 |
| -103.74 | | NA |  | NA |  | -218.57 | | -251.46 | | -209.29 | | -170.61 | | -180.66 | |
| -251.52 | | NA |  | NA |  | -239.87 | | -370.85 | | -301.91 | | -235.15 | | -248.73 | |
|  | -79.09 | NA |  | NA |  |  | -36.6 | -109.14 | |  | -8.2 | NA |  | NA |  |
| -346.03 | |  | -37.97 |  | -102.6 | -205.46 | | -177.75 | | -107.43 | | -246.56 | | -249.21 | |

| -103.23 | | NA |  | NA |  |  | -41.3 | -101.62 | | NA |  | NA |  | NA |  |
| --- | --- | --- | --- | --- | --- | --- | --- | --- | --- | --- | --- | --- | --- | --- | --- |
| -167.92 | | NA |  | NA |  |  | -95.14 | -211.29 | |  | -19.27 | NA |  |  | -6.99 |
| -187.12 | | NA |  | NA |  | -785.16 | | -565.67 | | -791.41 | | -553.94 | | -629.57 | |
|  | -26.01 | NA |  | NA |  | NA |  |  | -15.39 | NA |  | NA |  | NA |  |
| -457.36 | | NA |  |  | -16.25 | -777.95 | | -383.37 | | -732.49 | | -1464.73 | | -1532.45 | |
| NA |  | NA |  | NA |  | -211.12 | | -138.69 | | -417.24 | | NA |  |  | -33.38 |
| -304.65 | | NA |  | NA |  | -522.03 | | -420.53 | | -186.83 | |  | -85.67 | -183.81 | |
| NA |  | NA |  | NA |  | -203.98 | | -199.62 | |  | -23.63 | NA |  |  | -18.46 |
| -176.47 | |  | -12.05 |  | -16.76 | -117.02 | | -131.04 | |  | -41.38 |  | -8.98 |  | -30.38 |
|  | -52.94 | NA |  | NA |  |  | -48.39 |  | -63.91 |  | -8.64 | NA |  |  | -9.35 |
| -106.22 | | NA |  |  | -10.73 | NA |  |  | -109.2 | NA |  | NA |  | NA |  |
| NA |  | NA |  | NA |  | -106.53 | |  | -19.25 | -596.81 | |  | -947.1 | -845.03 | |
|  | -87.51 | NA |  | NA |  | NA |  |  | -51.18 |  | -5.29 | NA |  | NA |  |
|  | -55.19 |  | -25.35 |  | -87.18 | NA |  | NA |  |  | -49.74 |  | -19.19 |  | -16 |
| -108.13 | |  | -27.91 |  | -54.53 | NA |  |  | -7.55 |  | -50.73 |  | -26.95 |  | -32.1 |
|  | -565.4 |  | -13.59 |  | -50.02 | -679.37 | | -621.84 | | -165.65 | |  | -46.45 | -162.02 | |
|  | -42.07 | NA |  |  | -5.66 |  | -31.1 |  | -20.74 |  | -12.05 | NA |  |  | -5.97 |
| -257.16 | | NA |  |  | -12.53 | -123.57 | | -144.46 | |  | -15.16 |  | -5.67 |  | -25.38 |
| NA |  | NA |  | NA |  | NA |  |  | -9.38 | NA |  | NA |  | NA |  |
|  | -28.83 | NA |  | NA |  | NA |  |  | -38.99 | NA |  | NA |  | NA |  |
|  | -57.77 | NA |  | NA |  |  | -56.65 | -103.31 | |  | -55.45 | NA |  |  | -28.79 |
| NA |  | NA |  | NA |  | NA |  |  | -14.35 |  | -59.64 | -113.19 | |  | -37.96 |
| -177.56 | | NA |  |  | -10.07 |  | -54.8 |  | -86.24 |  | -19.96 | NA |  |  | -7.16 |
| -105.15 | | NA |  | NA |  | NA |  |  | -68.81 |  | -6.09 | NA |  | NA |  |
|  | -16.46 | NA |  | NA |  | -118.76 | | -165.18 | |  | -37.75 | NA |  |  | -26.59 |
| -100.36 | | NA |  | NA |  |  | -49.02 |  | -89.8 | -159.13 | | -143.45 | | -123.87 | |
|  | -91.32 | NA |  | NA |  | -296.27 | | -195.59 | | -110.22 | |  | -46.56 |  | -88.74 |
|  | -63.09 |  | -21.91 |  | -69.12 | NA |  |  | -82.72 |  | -18.87 | NA |  |  | -5.18 |
|  | -80.02 | NA |  | NA |  | -275.19 | |  | -169.1 |  | -83.12 |  | -15.2 |  | -56.66 |
|  | -11.69 | NA |  | NA |  | -116.07 | |  | -58 |  | -26.7 | NA |  |  | -11.38 |
| -107.31 | | NA |  | NA |  | NA |  |  | -103.8 |  | -59 |  | -7.36 |  | -13.7 |
| -368.62 | |  | -48.2 | -162.41 | | -119.95 | | -108.96 | | -357.57 | | -402.13 | | -378.21 | |
| -108.08 | |  | -13.48 |  | -29.36 | NA |  |  | -76.92 | NA |  | NA |  | NA |  |
|  | -46.09 | NA |  | NA |  |  | -28.86 |  | -47.82 | -178.37 | |  | -60.35 | -106.08 | |
| NA |  | NA |  | NA |  | NA |  | NA |  | NA |  |  | -92.86 |  | -48.54 |
|  | -83.33 | NA |  | NA |  | -113.48 | | -119.87 | |  | -21.34 | NA |  |  | -15.85 |
|  | -86.86 | NA |  | NA |  |  | -53.64 |  | -56.64 |  | -50.2 |  | -24.09 |  | -41.11 |
|  | -64.83 |  | -5.33 |  | -10.23 | NA |  |  | -93.61 | NA |  | NA |  | NA |  |

liver..crest macropha macropha mature_co mature_eo mature_ne megakaryomegakaryo

-129.34	-126	-168.97	-234.56	-180.51	-171.82	-215.87	-287.31

NA	NA	NA	NA	NA	NA	NA	NA

-673.59	-140.55	-332.11	-341.38	-395.11	-201.57	-223.29	-422.8

-342.07	-322.99	-113.32	-156.03	-261.19	-301.4	-137.94	-254.54

-293.31	-60.02	-22.15	-29.37	-47.63	-66.32	-17.79	-53.51

-583.85	-420.05	-517.19	-674.3	-841.39	-657.03	-691.81	-600.07

-786.13	-196.35	-310.65	-387.31	-388.17	-278.82	-187.68	-496.88

-187.02	-43.69	-40.43	-52.57	-58.92	-61.01	-37.19	-149.62

-187.02	-43.69	-40.43	-52.57	-58.92	-61.01	-37.19	-149.62

-196.94	-41.66	-187.22	-191.83	-211.48	-120.24 NA	-58.67

-399.72	-214.1	-381.16	-487.07	-572.55	-361.03	-489.75	-461.35

-320.81	-89.18	-77.09	-114.72	-197.92	-148.64	-149.31	-140.56

-44.32	-256.5	-614.06	-640.69	-161.38	-180.71	-119.91	-198.26

-141.71	-278.86	-638.1	-706.09	-217.69	-209.12	-139.49	-230.18

NA	NA	NA	NA	NA	NA	NA	NA NA	NA	NA	NA	NA	NA	NA	NA

-10.22 NA	-167.05	-168.23	-26.87 NA	NA	NA

-10.22 NA	-167.05	-168.23	-26.87 NA	NA	NA

-22.54	-519.82	-6133.92	-5656.58	-2086.24	-946.43	-1980.85	-2094.43

-794.92	-138.92	-212.74	-241.4	-339.51	-229.1	-178.62	-298.4

NA	NA	NA	NA	NA	NA	NA	NA

-73.91	-463.92	-1790.94	-1974	-1887.32	-936.39	-1640.05	-1658.44

NA	NA	NA	NA	NA	NA	NA	NA NA	NA	NA	NA	NA	NA	NA	NA

-208.35	-24.84	-476.11	-405.23	-597.74	-336.91 NA	NA

-985.05	-211.34	-1435.17	-1157.66	-1026.81	-1285.25 NA	NA

-1043.12	-225.38	-1508.82	-1239.88	-1057.14	-1307.03 NA	NA

-590.05	-133.39	-1007.33	-795.25	-712.21	-908.19 NA	NA

-837.81	-95.66	-295.65	-275.31	-383.94	-186.96	-221.99	-441.37

-837.81	-95.66	-295.65	-275.31	-383.94	-186.96	-221.99	-441.37

-1034.57	-215.32	-430.47	-469.87	-546.95	-345.35	-330.94	-661.17

-1108.68	-187.82	-527.56	-577.35	-629.42	-367.11	-374.1	-760.64

-225.65	-35.04	-275.17	-280.73	-299.83	-134.48 NA	-50.23

-203.18	-38.41	-788.23	-653.78	-557.07	-315.58 NA	NA NA	NA	NA	NA	NA	NA	NA	NA NA	NA	NA	NA	NA	NA	NA	NA NA	NA	NA	NA	NA	NA	NA	NA

-21.14	-168.57	-100.24	-117.92	-47.4	-153.18	-214.46	-281.58

| -114.87 | |  | -26.36 |  | -22.11 |  | -50.19 |  | -19.44 |  | -62.48 |  | -61.67 | -112.27 | |
| --- | --- | --- | --- | --- | --- | --- | --- | --- | --- | --- | --- | --- | --- | --- | --- |
| -114.87 | |  | -26.36 |  | -22.11 |  | -50.19 |  | -19.44 |  | -62.48 |  | -61.67 | -112.27 | |
| -114.87 | |  | -26.36 |  | -22.11 |  | -50.19 |  | -19.44 |  | -62.48 |  | -61.67 | -112.27 | |
|  | -30.34 |  | -47.23 | -154.64 | | -242.73 | |  | -30.73 |  | -23.29 | -179.02 | | -221.85 | |
|  | -27.74 |  | -66.2 |  | -84.86 | -143.18 | |  | -27.26 |  | -28.49 | -126.81 | |  | -167.3 |
|  | -46.49 |  | -980.3 | -5966.52 | | -5955.7 | | -3657.56 | | -2388.53 | | -4101.01 | | -4595.46 | |
| -135.47 | | -1478.56 | | -7984.97 | | -7838.85 | | -5199.72 | | -3506.53 | | -4819.18 | | -5110.31 | |
| -167.83 | | -2295.29 | | -11132.8 | |  | -10959 | -7464.94 | | -5011.52 | | -6970.52 | | -7389.03 | |
|  | -166.2 | -1372.13 | | -7898.65 | | -7931.81 | | -4939.12 | | -3133.09 | | -5589.02 | | -5829.46 | |
|  | -151.5 | -1009.24 | | -5132.59 | | -4994.35 | | -3469.67 | | -2383.39 | | -3043.51 | | -3344.68 | |
| -129.82 | | -1436.07 | | -6500.98 | | -6447.7 | | -4294.55 | | -3146.45 | | -3989.12 | | -3916.49 | |
|  | -96.16 | -1807.71 | | -11380.3 | | -10933.7 | | -7451.47 | | -4503.82 | | -6331.8 | | -6314.89 | |
| -343.43 | | -1635.41 | | -5472.19 | | -5745.29 | | -4238.41 | | -3524.3 | | -4161.93 | | -4866.1 | |
| -241.08 | | -1743.6 | | -6975.54 | | -7160.88 | | -5244.79 | | -3983.25 | | -4902.72 | | -5610.31 | |
| -267.31 | | -2157.69 | | -8040.3 | | -8398.59 | | -6394.23 | | -4733.43 | | -5988.29 | |  | -6833 |
| NA |  | NA |  | NA |  | NA |  | NA |  | NA |  | NA |  | NA |  |
| -611.86 | |  | -60.87 |  | -59.19 |  | -72.78 | -111.17 | |  | -85.04 |  | -52.09 | -218.81 | |
| -195.35 | | -2107.19 | | -8608.36 | | -8713.13 | | -6497.94 | | -4677.09 | | -6076.19 | | -6599.21 | |
| -178.67 | | -2453.56 | | -9544.11 | | -9679.31 | | -7184.72 | | -5321.23 | | -6956.85 | | -7836.94 | |
|  | -65.71 | -234.47 | | -630.55 | | -669.86 | | -474.78 | | -455.61 | |  | -525.9 | -574.32 | |
| -134.87 | | -2551.82 | | -12430.9 | | -12574.6 | | -8911.11 | | -6177.1 | | -8933.49 | | -9538.15 | |
| -773.76 | | NA |  | NA |  | NA |  | NA |  | NA |  | NA |  | NA |  |
| NA |  | NA |  | NA |  | NA |  | NA |  | NA |  | NA |  | NA |  |
| -148.01 | | -2387.25 | | -10720.7 | | -10876.6 | | -8064.03 | | -5681.2 | | -7607.11 | | -8592.41 | |
|  | -25.41 | -124.01 | |  | -372.8 | -389.45 | | -252.43 | | -239.95 | | -299.32 | | -392.64 | |
|  | -51.18 | -1195.9 | | -6511.62 | | -6541.08 | | -3924.4 | | -2749.91 | | -3983.47 | | -4196.22 | |
| -246.03 | | -2438.88 | | -9993.27 | | -10239.1 | | -7751.82 | | -5544.17 | | -7191.72 | | -7986.21 | |
| -118.98 | | -1771.46 | | -7776.91 | | -7886.93 | | -5777.38 | | -4027.07 | | -5382.96 | | -6057.15 | |
| -233.01 | | -882.43 | |  | -3005 | -3122.97 | | -2376.22 | | -1894.21 | | -2159.52 | | -2433.42 | |
| -233.01 | | -882.43 | |  | -3005 | -3122.97 | | -2376.22 | | -1894.21 | | -2159.52 | | -2433.42 | |
| -156.99 | | -2562.96 | | -11469.9 | | -11634.1 | | -8476.55 | | -5944.02 | | -8053.19 | | -8805.89 | |
| -147.84 | | -1173.32 | | -4461.68 | | -4657.98 | | -3486.37 | | -2643.75 | | -3493.7 | | -3938.72 | |
| NA |  | -891.22 | | -4158.26 | | -4103.02 | | -2341.91 | | -1907.28 | | -2315.15 | | -2195.14 | |
|  | -84.64 | -717.53 | | -3559.34 | | -3675.45 | | -2472.36 | | -1703.55 | | -2449.95 | | -2844.55 | |
| NA |  | NA |  | NA |  | NA |  | NA |  | NA |  | NA |  | NA |  |
| -337.68 | | -105.73 | | -118.49 | | -166.03 | | -242.16 | | -178.85 | | -185.07 | | -196.86 | |
|  | -76.8 | -2120.25 | | -11311.7 | | -11405.9 | | -8132.78 | | -5201.71 | | -7355.38 | | -8216.93 | |
| -157.37 | | -2738.33 | | -12235.4 | | -12425.5 | | -9040.97 | | -6333.18 | | -8784.62 | | -9566.79 | |
|  | -84.14 | -258.51 | | -678.89 | | -717.05 | | -482.64 | |  | -503.4 |  | -578.3 |  | -666.4 |

-10.1	-207.08	-762.45	-837.65	-110.03	-94.22	-81.08	-119.03

-47.14	-128.39	-765.72	-808.81	-208.33	-89.02	-130.63	-199.14

-75.33	-275.19	-892.96	-1009.07	-231.6	-206.43	-179.11	-267.54

-7.4	-153.01	-334.74	-396.34	-18.21	-36.78	-8.56	-32.23

-1661.91 NA	NA	NA	NA	NA	NA	NA

-933.52 NA	NA	NA	NA	NA	NA	NA

-902.97 NA	NA	NA	NA	NA	NA	NA

-474.7 NA	NA	NA	NA	NA	NA	NA

-1008.07 NA	NA	NA	NA	NA	NA	NA

-837.41 NA	NA	NA	NA	NA	NA	NA

-328.38 NA	NA	NA	NA	NA	NA	NA

-328.38 NA	NA	NA	NA	NA	NA	NA

-328.38 NA	NA	NA	NA	NA	NA	NA

-845.94 NA	NA	NA	NA	NA	NA	NA

-850.47 NA	NA	NA	NA	NA	NA	NA

-855.39 NA	-14	-9.88	-25.25 NA	-10.96 NA

-273.87 NA	NA	NA	NA	NA	NA	NA

-492.28 NA	NA	NA	NA	NA	NA	NA

-624.89 NA	-51.27	-41.8	-48.53 NA	-48.94	-20.14

-1007.36 NA	-9.03 NA	-21.1 NA	-13.31 NA

-25.06 NA	NA	NA	NA	NA	NA	NA

-295.13	-2316.27	-8944	-9207.16	-6696.22	-5067.7	-6467.32	-7025.89

-262.1 NA	-166.21	-143.71	-688.18	-34.95	-300.21	-837.31

-262.1 NA	-166.21	-143.71	-688.18	-34.95	-300.21	-837.31

-7.68 NA	-17.14 NA	-136.44 NA	-14.52	-102.44

-7.68 NA	-17.14 NA	-136.44 NA	-14.52	-102.44

-69.48 NA	-15.59	-5.74	-537.68 NA	-124.77	-591.2

-292.3 NA	-116.3	-96.81	-1278.08	-16.79	-448.21	-1292.06

-37.98 NA	NA	NA	-137.9 NA	-6.84	-175.46

-13.28 NA	NA	NA	-213.15 NA	-27.03	-260.34

-13.28 NA	NA	NA	-213.15 NA	-27.03	-260.34

-13.28 NA	NA	NA	-213.15 NA	-27.03	-260.34

NA	NA	NA	NA	NA	NA	NA	NA NA	NA	NA	NA	NA	NA	NA	NA NA	NA	NA	NA	NA	NA	NA	NA NA	NA	NA	NA	NA	NA	NA	NA

| -305.07 | -41.79 | -41.02 | -60.9 | -73.79 | -70.51 | -82.8 | -131.1 |
| --- | --- | --- | --- | --- | --- | --- | --- |
| -359.85 | -147.61 | -21.28 | -39.9 | -70.67 | -188.08 | -62.75 | -199.36 |
| -359.85 | -147.61 | -21.28 | -39.9 | -70.67 | -188.08 | -62.75 | -199.36 |

| -690.34 | | -325.73 | |  | -62.56 |  | -89.23 | -179.23 | | -315.84 | | -107.04 | | -238.52 | |
| --- | --- | --- | --- | --- | --- | --- | --- | --- | --- | --- | --- | --- | --- | --- | --- |
|  | -84.21 |  | -6.58 | NA |  | NA |  | NA |  |  | -5.1 | NA |  | NA |  |
| -314.39 | | -169.16 | | -146.45 | | -179.99 | | -185.68 | | -217.87 | | -190.25 | | -242.07 | |
|  | -197.8 |  | -57.67 | NA |  | NA |  |  | -8.55 |  | -70.99 |  | -12.09 |  | -58.72 |
| -250.46 | | -158.73 | | -359.53 | | -431.36 | | -314.56 | | -322.82 | |  | -307.6 | -438.18 | |
| -272.27 | |  | -48.18 | -674.38 | | -527.86 | | -429.25 | | -424.88 | | NA |  | NA |  |
|  | -50.1 | NA |  | NA |  | NA |  | NA |  | NA |  | NA |  | NA |  |
|  | -69.58 | NA |  | NA |  | NA |  | NA |  | NA |  | NA |  | NA |  |
| -4294.55 | |  | -8.68 | NA |  | NA |  |  | -9.24 |  | -7.37 | NA |  |  | -11.39 |
| -4223.17 | | NA |  | NA |  |  | -8.34 |  | -15.94 |  | -6.46 |  | -5.67 |  | -31.97 |
| -3969.98 | |  | -31.29 |  | -27.83 |  | -52.94 |  | -74.71 |  | -65.38 |  | -49.9 |  | -86.17 |
| NA |  | NA |  | NA |  | NA |  | NA |  | NA |  | NA |  | NA |  |
| NA |  | NA |  | NA |  | NA |  | NA |  | NA |  | NA |  | NA |  |
| NA |  | NA |  | NA |  | NA |  | NA |  | NA |  | NA |  | NA |  |
| NA |  | NA |  | NA |  | NA |  | NA |  | NA |  | NA |  | NA |  |
| NA |  | NA |  | NA |  | NA |  | NA |  | NA |  | NA |  | NA |  |
| NA |  | NA |  | NA |  | NA |  | NA |  | NA |  | NA |  | NA |  |
| NA |  | NA |  | NA |  | NA |  | NA |  | NA |  | NA |  | NA |  |
| NA |  | NA |  | NA |  | NA |  | NA |  | NA |  | NA |  | NA |  |
| NA |  | NA |  |  | -24.37 |  | -11.38 |  | -12.29 | NA |  |  | -74.88 | NA |  |
| NA |  | NA |  | NA |  | NA |  | NA |  | NA |  | NA |  | NA |  |
| NA |  | NA |  | NA |  | NA |  | NA |  | NA |  | NA |  | NA |  |
| NA |  | NA |  | NA |  | NA |  | NA |  | NA |  | NA |  | NA |  |
| NA |  | NA |  | NA |  | NA |  | NA |  | NA |  | NA |  | NA |  |
| NA |  | NA |  | NA |  | NA |  | NA |  | NA |  | NA |  | NA |  |
| NA |  | NA |  |  | -20.55 |  | -15.23 |  | -17.34 | NA |  |  | -62.62 |  | -6.31 |
| NA |  | NA |  | NA |  | NA |  | NA |  | NA |  | NA |  | NA |  |
| NA |  | NA |  | NA |  | NA |  | NA |  | NA |  | NA |  | NA |  |
| NA |  | NA |  | NA |  | NA |  | NA |  | NA |  | NA |  | NA |  |
| NA |  | NA |  | NA |  | NA |  | NA |  | NA |  | NA |  | NA |  |
|  | -22.23 | NA |  |  | -55.7 |  | -44.94 |  | -55.93 | NA |  |  | -45.41 |  | -45.84 |
| NA |  | NA |  | NA |  | NA |  | NA |  | NA |  | NA |  | NA |  |
| NA |  | NA |  | NA |  | NA |  | NA |  | NA |  | NA |  | NA |  |
| NA |  | NA |  | NA |  | NA |  | NA |  | NA |  | NA |  | NA |  |
| NA |  | NA |  | NA |  | NA |  | NA |  | NA |  | NA |  | NA |  |
| NA |  | NA |  |  | -9.67 | NA |  |  | -12.98 | NA |  | NA |  | NA |  |
| NA |  | NA |  | NA |  | NA |  | NA |  | NA |  | NA |  | NA |  |
| NA |  | NA |  |  | -7.74 | NA |  |  | -11.63 | NA |  | NA |  | NA |  |
| NA |  | NA |  | NA |  | NA |  | NA |  | NA |  | NA |  | NA |  |

| NA |  | NA |  | NA |  | NA |  | NA |  | NA |  | NA |  | NA |  |
| --- | --- | --- | --- | --- | --- | --- | --- | --- | --- | --- | --- | --- | --- | --- | --- |
| NA |  | NA |  |  | -51.84 |  | -34.52 |  | -29.88 | NA |  | -114.47 | |  | -10.92 |
| NA |  | NA |  | NA |  | NA |  | NA |  | NA |  | NA |  | NA |  |
| NA |  | NA |  | NA |  | NA |  | NA |  | NA |  | NA |  | NA |  |
| NA |  | NA |  | NA |  | NA |  | NA |  | NA |  | NA |  | NA |  |
| NA |  | NA |  |  | -27.04 |  | -10.65 |  | -26.62 | NA |  |  | -5.29 | NA |  |
| NA |  | NA |  | NA |  | NA |  | NA |  | NA |  | NA |  | NA |  |
| NA |  | NA |  | NA |  | NA |  | NA |  | NA |  | NA |  | NA |  |
| NA |  | NA |  | NA |  | NA |  | NA |  | NA |  | NA |  | NA |  |
| NA |  | NA |  |  | -16.66 | NA |  |  | -8.68 | NA |  | NA |  | NA |  |
|  | -12.54 | -215.86 | | -2542.91 | | -2463.14 | | -1355.92 | | -654.29 | | -1321.98 | | -1351.07 | |
| -721.27 | | NA |  | -523.71 | |  | -98.62 |  | -58 |  | -12.52 |  | -16.02 | -647.56 | |
| NA |  | NA |  | -367.51 | | -217.17 | | NA |  | NA |  |  | -5.96 | NA |  |
|  | -15.1 |  | -43.43 | -1466.36 | | -1238.89 | | -377.75 | | -117.33 | | -352.04 | | -326.83 | |
| -127.45 | |  | -90.18 |  | -680.5 | -627.09 | | -237.88 | |  | -113.9 | -185.67 | | -120.41 | |
| -120.34 | |  | -57.01 | -426.12 | | -400.64 | | -173.67 | |  | -75.9 | -115.88 | | -127.67 | |
|  | -87.21 |  | -29.51 |  | -52.06 |  | -43.3 |  | -31.85 |  | -32.55 | NA |  | NA |  |
|  | -12.01 | NA |  | -397.51 | | -275.58 | |  | -54.98 | NA |  |  | -29.42 |  | -25.43 |
|  | -86.09 | -167.31 | | -1646.56 | | -1493.94 | | -418.03 | | -191.93 | | -344.03 | | -264.62 | |
|  | -23.82 | NA |  | -332.37 | | -293.67 | |  | -47.53 |  | -21.45 |  | -41.34 |  | -5.72 |
| NA |  | -134.13 | | -510.39 | | -570.79 | |  | -46.17 |  | -32.03 |  | -31.76 |  | -59.1 |
|  | -5.96 | -190.24 | | -568.79 | | -607.97 | |  | -77.68 |  | -83.9 |  | -36.24 |  | -76.44 |
|  | -27.75 | -214.63 | | -825.97 | | -907.65 | | -164.28 | | -142.18 | |  | -93.67 | -125.24 | |
| NA |  |  | -74.12 | -260.28 | | -286.18 | |  | -6.93 |  | -10.24 | NA |  |  | -18.29 |
| NA |  | NA |  |  | -19.16 |  | -49.21 | NA |  | NA |  |  | -31.38 | -101.13 | |
| -106.91 | |  | -12.7 | -110.61 | | -101.74 | |  | -87.48 |  | -80.83 |  | -72.45 | -276.47 | |
|  | -25.34 |  | -69.35 |  | -18.38 |  | -40.62 |  | -8.81 |  | -42.63 |  | -28.31 |  | -84.28 |
|  | -21.86 |  | -42.11 |  | -32.98 |  | -61.79 |  | -29.14 |  | -29.15 |  | -34.07 |  | -53.13 |
|  | -21.86 |  | -42.11 |  | -32.98 |  | -61.79 |  | -29.14 |  | -29.15 |  | -34.07 |  | -53.13 |
| NA |  |  | -18.59 | NA |  |  | -17.37 | NA |  | NA |  |  | -21.78 |  | -30.81 |
|  | -5.36 |  | -18.14 |  | -75.63 | -123.06 | |  | -6.37 |  | -7.12 | -161.76 | | -186.39 | |
| NA |  |  | -9.76 |  | -12.09 |  | -33.31 | NA |  | NA |  |  | -31.29 |  | -88.98 |
| NA |  |  | -9.76 |  | -12.09 |  | -33.31 | NA |  | NA |  |  | -31.29 |  | -88.98 |
| NA |  |  | -9.76 |  | -12.09 |  | -33.31 | NA |  | NA |  |  | -31.29 |  | -88.98 |
|  | -5.9 |  | -33.79 | -150.71 | | -193.17 | |  | -13.96 |  | -22.42 | -219.81 | | -262.78 | |
|  | -59.42 | -173.93 | |  | -158.2 | -204.65 | | -109.12 | | -152.58 | | -349.29 | | -303.71 | |
| NA |  | NA |  | NA |  | NA |  | NA |  | NA |  | NA |  |  | -15.53 |
| NA |  | NA |  | NA |  | NA |  | NA |  | NA |  | NA |  | NA |  |
| -289.15 | | NA |  |  | -46.81 |  | -35.29 |  | -22.43 | NA |  |  | -11.28 | NA |  |

| NA |  | NA |  | NA |  | NA |  | NA |  | NA |  | NA |  | NA |  |
| --- | --- | --- | --- | --- | --- | --- | --- | --- | --- | --- | --- | --- | --- | --- | --- |
| NA |  | NA |  | NA |  | NA |  | NA |  | NA |  | NA |  | NA |  |
| NA |  | NA |  | NA |  | NA |  | NA |  | NA |  | NA |  | NA |  |
| NA |  | NA |  | NA |  | NA |  | NA |  | NA |  | NA |  | NA |  |
| -326.63 | | -300.02 | | -194.54 | | -286.93 | |  | -375.5 | -405.71 | | -273.15 | | -348.94 | |
| -633.11 | | -195.64 | | -340.42 | | -388.38 | | -422.26 | |  | -284.8 |  | -273.1 | -430.16 | |
| -385.99 | |  | -94.61 |  | -51.22 |  | -84.88 |  | -90.43 | -140.56 | | -116.14 | | -147.07 | |
|  | -454.7 |  | -275.6 | -513.29 | |  | -583.5 | -666.55 | | -448.95 | | -455.87 | | -588.51 | |
| -101.23 | | -154.13 | |  | -66.56 |  | -75.92 |  | -67.37 | -161.67 | |  | -35.08 |  | -59.1 |
| -172.92 | | -174.25 | | -157.86 | | -169.29 | | -165.77 | | -217.68 | |  | -89.08 |  | -112.3 |
| -655.28 | | -155.61 | | -273.19 | | -291.52 | | -358.18 | |  | -240.1 | -216.04 | | -385.14 | |
| -278.27 | | -364.25 | | -166.54 | | -193.62 | | -193.67 | | -307.68 | | -225.82 | | -298.01 | |
| -449.24 | | -484.59 | | -352.72 | | -383.29 | | -349.96 | | -596.21 | |  | -394.9 | -550.02 | |
| -224.71 | |  | -11.45 |  | -31.58 |  | -50.89 |  | -91.45 |  | -44.71 |  | -52.06 |  | -90.47 |
| NA |  | NA |  | NA |  | NA |  | NA |  | NA |  | NA |  | NA |  |
| NA |  | NA |  | NA |  | NA |  | NA |  | NA |  | NA |  | NA |  |
| -745.93 | |  | -79.11 | -139.08 | | -153.53 | | -237.85 | | -158.48 | | -108.36 | | -213.07 | |
| NA |  | NA |  | NA |  | NA |  | NA |  | NA |  | NA |  | NA |  |
| -428.34 | | -150.09 | | -233.24 | | -308.98 | | -400.11 | | -261.71 | | -310.21 | | -245.32 | |
| NA |  | NA |  | NA |  | NA |  | NA |  | NA |  | NA |  | NA |  |
| NA |  | NA |  | NA |  | NA |  | NA |  | NA |  | NA |  | NA |  |
| -374.27 | | -128.36 | | -158.97 | | -171.36 | | -208.44 | | -178.08 | | -149.02 | | -187.89 | |
| -742.38 | | -148.19 | |  | -290.3 | -309.92 | | -359.07 | | -223.53 | | -224.28 | |  | -447.8 |
| -742.38 | | -148.19 | |  | -290.3 | -309.92 | | -359.07 | | -223.53 | | -224.28 | |  | -447.8 |
| -742.38 | | -148.19 | |  | -290.3 | -309.92 | | -359.07 | | -223.53 | | -224.28 | |  | -447.8 |
| -742.38 | | -148.19 | |  | -290.3 | -309.92 | | -359.07 | | -223.53 | | -224.28 | |  | -447.8 |
| -357.23 | | -164.96 | | -572.79 | | -611.94 | | -616.99 | | -348.89 | | -477.11 | | -544.73 | |
| -357.23 | | -164.96 | | -572.79 | | -611.94 | | -616.99 | | -348.89 | | -477.11 | | -544.73 | |
| -357.23 | | -164.96 | | -572.79 | | -611.94 | | -616.99 | | -348.89 | | -477.11 | | -544.73 | |
| -357.23 | | -164.96 | | -572.79 | | -611.94 | | -616.99 | | -348.89 | | -477.11 | | -544.73 | |
| -824.07 | |  | -41.27 | -462.26 | | -439.57 | | -529.97 | | -146.89 | | -254.26 | | -397.94 | |
| -824.07 | |  | -41.27 | -462.26 | | -439.57 | | -529.97 | | -146.89 | | -254.26 | | -397.94 | |
| -351.45 | |  | -20.97 | -316.25 | | -316.11 | | -362.74 | | -104.01 | |  | -171.2 | -253.79 | |
| -351.45 | |  | -20.97 | -316.25 | | -316.11 | | -362.74 | | -104.01 | |  | -171.2 | -253.79 | |
| -1099.82 | | -290.36 | |  | -509.5 | -530.26 | | -620.34 | | -402.47 | | -406.37 | | -755.06 | |
| -400.98 | | -243.32 | | -182.86 | | -267.05 | | -426.23 | | -364.22 | | -325.17 | | -263.89 | |
| -400.98 | | -243.32 | | -182.86 | | -267.05 | | -426.23 | | -364.22 | | -325.17 | | -263.89 | |
| -426.38 | | -144.22 | |  | -335.2 | -393.78 | |  | -478.9 | -264.82 | | -341.42 | | -305.82 | |
| -426.38 | | -144.22 | |  | -335.2 | -393.78 | |  | -478.9 | -264.82 | | -341.42 | | -305.82 | |

| -378.75 | | -202.27 | | -125.53 | |  | -187.4 | -337.05 | |  | -316.1 | -288.97 | | -216.07 | |
| --- | --- | --- | --- | --- | --- | --- | --- | --- | --- | --- | --- | --- | --- | --- | --- |
| -611.16 | | -331.77 | | -300.91 | | -427.28 | | -579.34 | | -492.67 | | -496.29 | | -448.67 | |
| -498.46 | | -158.41 | | -141.08 | | -220.02 | | -339.18 | | -235.45 | | -277.54 | | -258.05 | |
|  | -42.31 |  | -25.85 |  | -90.69 | -112.11 | | -153.81 | |  | -69.87 |  | -93.47 |  | -109.6 |
| -111.59 | | -220.55 | | -835.29 | |  | -881.6 | -447.61 | | -290.22 | | -338.75 | | -486.68 | |
| NA |  | -120.87 | | -396.61 | | -431.14 | |  | -25.33 |  | -17.81 |  | -16.22 |  | -49.41 |
| -179.82 | | -328.48 | | -688.46 | | -748.62 | | -441.56 | | -351.93 | | -358.02 | | -545.77 | |
| -1965.4 | | -216.88 | | -201.95 | | -249.68 | | -316.81 | |  | -413.8 | -386.24 | | -352.97 | |
| -2299.58 | | -279.82 | | -244.82 | | -307.85 | | -378.81 | | -492.56 | | -478.21 | | -411.94 | |
| -588.85 | |  | -19.4 | NA |  | NA |  | NA |  |  | -31.81 | NA |  | NA |  |
| -2527.16 | |  | -260.7 | -256.14 | | -313.83 | | -390.24 | | -461.85 | | -486.65 | | -454.74 | |
| -1497.42 | |  | -82.28 |  | -182 | -204.73 | | -240.84 | | -194.14 | | -247.24 | | -206.75 | |
| NA |  | NA |  | NA |  | NA |  | NA |  | NA |  | NA |  | NA |  |
|  | -57.58 |  | -62.39 | -188.28 | | -262.68 | |  | -86.4 |  | -127.8 | -184.67 | | -222.57 | |
| NA |  | NA |  | NA |  | NA |  | NA |  | NA |  | NA |  | NA |  |
| NA |  | NA |  | NA |  | NA |  | NA |  | NA |  | NA |  | NA |  |
| NA |  | NA |  | NA |  | NA |  | NA |  | NA |  | NA |  | NA |  |
|  | -17.6 | NA |  |  | -51.45 |  | -41.87 |  | -26.99 | NA |  |  | -19.1 |  | -35.15 |
| NA |  | NA |  | NA |  | NA |  | NA |  | NA |  | NA |  | NA |  |
| NA |  | NA |  | NA |  | NA |  | NA |  | NA |  | NA |  | NA |  |
| NA |  | NA |  | NA |  | NA |  | NA |  | NA |  | NA |  | NA |  |
| NA |  | NA |  | NA |  | NA |  | NA |  | NA |  | NA |  | NA |  |
| NA |  | NA |  | NA |  | NA |  | NA |  | NA |  | NA |  | NA |  |
| NA |  | NA |  |  | -80.71 |  | -82.23 |  | -64.68 | NA |  |  | -37.05 |  | -47.66 |
| NA |  |  | -16.19 | -320.07 | | -318.08 | | -174.68 | |  | -52.92 | -201.35 | | -207.68 | |
| NA |  | NA |  | NA |  | NA |  | NA |  | NA |  | NA |  | NA |  |
| NA |  | NA |  | NA |  | NA |  | NA |  | NA |  | NA |  | NA |  |
| NA |  | NA |  | NA |  | NA |  | NA |  | NA |  | NA |  | NA |  |
| -1127.01 | | -199.61 | | -519.33 | | -527.18 | | -616.02 | | -338.64 | | -391.15 | | -736.76 | |
|  | -98.37 |  | -72.65 | -344.19 | | -363.59 | | -121.15 | | -100.92 | |  | -355.6 |  | -517.6 |
|  | -98.37 |  | -72.65 | -344.19 | | -363.59 | | -121.15 | | -100.92 | |  | -355.6 |  | -517.6 |
|  | -633.2 | NA |  | NA |  | NA |  | NA |  | NA |  | NA |  | NA |  |
| -865.11 | | NA |  | NA |  | NA |  | NA |  |  | -10.06 | NA |  |  | -6.98 |
| -1331.15 | | NA |  | NA |  | NA |  | NA |  | NA |  | NA |  | NA |  |
| -1929.04 | | NA |  | NA |  |  | -8.61 | NA |  | NA |  |  | -7.54 |  | -30.94 |
| -109.59 | | -492.45 | | -851.38 | | -934.06 | | -718.03 | |  | -975.9 | -802.78 | | -940.07 | |
| -3155.53 | | NA |  |  | -6.27 |  | -13.35 |  | -6.17 |  | -11.6 | NA |  |  | -15.92 |
| -751.04 | | NA |  | NA |  | NA |  | NA |  | NA |  | NA |  | NA |  |
| -2537.44 | |  | -5.21 | NA |  |  | -6.77 | NA |  |  | -8.37 | NA |  |  | -21.15 |

| -1912.45 | | NA |  |  | -29.89 |  | -29.59 |  | -11.75 | NA |  |  | -15.51 |  | -25.12 |
| --- | --- | --- | --- | --- | --- | --- | --- | --- | --- | --- | --- | --- | --- | --- | --- |
| -918.52 | | NA |  | NA |  | NA |  | NA |  | NA |  | NA |  | NA |  |
| -1018.35 | | NA |  | NA |  | NA |  | NA |  | NA |  | NA |  | NA |  |
| -1385.1 | | NA |  | NA |  | NA |  | NA |  | NA |  | NA |  | NA |  |
| -807.15 | | NA |  | NA |  | NA |  | NA |  | NA |  | NA |  | NA |  |
| -635.11 | | -186.48 | | -433.62 | | -461.52 | | -493.15 | | -293.76 | | -295.95 | | -516.85 | |
| -225.68 | | NA |  | NA |  | NA |  | NA |  | NA |  | NA |  | NA |  |
| -225.68 | | NA |  | NA |  | NA |  | NA |  | NA |  | NA |  | NA |  |
| -225.68 | | NA |  | NA |  | NA |  | NA |  | NA |  | NA |  | NA |  |
| NA |  | NA |  | NA |  | NA |  | NA |  | NA |  | NA |  | NA |  |
|  | -65.72 |  | -50.05 |  | -51.76 |  | -50.26 |  | -50.4 |  | -68.76 |  | -27.89 |  | -58.7 |
| -184.28 | |  | -27.85 |  | -30.22 |  | -35.01 |  | -50.76 |  | -53.9 | NA |  |  | -36.79 |
| -408.15 | | -141.53 | | -378.38 | | -424.89 | | -314.36 | | -242.78 | | -159.01 | | -333.23 | |
| NA |  | NA |  | NA |  | NA |  | NA |  | NA |  | NA |  | NA |  |
|  | -83.02 |  | -36.53 | NA |  | NA |  | NA |  |  | -60.6 | NA |  | NA |  |
|  | -44.41 | NA |  |  | -67.54 |  | -72.5 |  | -49.56 |  | -11.48 |  | -7.26 |  | -37.46 |
| -170.65 | |  | -19.95 |  | -40.34 |  | -55.32 |  | -36.91 |  | -18.77 |  | -19.64 |  | -67.78 |
| -124.59 | |  | -40.59 | NA |  | NA |  | NA |  |  | -81.16 | NA |  |  | -11.97 |
| NA |  | NA |  | NA |  | NA |  | NA |  | NA |  | NA |  | NA |  |
|  | -10.73 | -139.29 | | NA |  |  | -34.51 |  | -29.85 | -146.75 | | -172.16 | | -178.44 | |
|  | -99.77 |  | -172.4 | -126.72 | | -202.43 | | -148.69 | | -204.35 | | -352.98 | | -344.86 | |
| -1331.9 | |  | -8.9 |  | -60.15 |  | -64.12 |  | -32.34 |  | -32.59 |  | -62.07 |  | -95.9 |
| -2291.89 | |  | -11.42 |  | -56.32 |  | -63.33 |  | -40.78 |  | -29.08 |  | -53.1 |  | -97.59 |
| -381.16 | | -551.57 | | -677.99 | | -736.97 | | -888.97 | |  | -815.5 | -516.66 | | -741.14 | |
|  | -291.8 |  | -73.97 | -186.65 | | -229.55 | | -166.27 | | -147.07 | | -206.82 | | -230.49 | |
| -527.11 | |  | -5.54 |  | -7.86 |  | -15.55 |  | -8.12 |  | -7.62 | NA |  |  | -29.98 |
|  | -54.84 |  | -71.03 | -215.54 | |  | -297.2 | -102.44 | | -146.59 | |  | -211.1 |  | -259.4 |
|  | -92.17 |  | -22.7 |  | -47.2 |  | -70.4 |  | -17.21 |  | -50.56 |  | -51.68 |  | -54.57 |
| -330.62 | | -150.73 | | -313.95 | | -366.52 | | -389.23 | |  | -323.2 |  | -256.4 | -321.98 | |
|  | -81.28 |  | -42.19 |  | -8.53 |  | -19.75 |  | -33.68 |  | -78.87 |  | -21.81 |  | -35.41 |
| -141.68 | |  | -22.35 |  | -84.53 |  | -84.88 | -112.59 | |  | -65.49 |  | -71.4 |  | -69.98 |
|  | -117.5 |  | -47.83 |  | -41.58 |  | -66.5 |  | -95.61 |  | -84.91 |  | -64.97 |  | -72.63 |
| -165.49 | |  | -49.88 | -115.75 | | -131.58 | | -158.43 | |  | -110.3 | -104.57 | | -139.77 | |
| -168.15 | |  | -50.99 |  | -87.62 | -117.83 | | -121.87 | | -145.17 | | -141.61 | | -140.92 | |
|  | -97.29 |  | -20.88 |  | -69.02 |  | -87.51 | -102.25 | |  | -47.31 |  | -60.14 |  | -65.38 |
|  | -20.82 | NA |  |  | -7.13 |  | -12.75 |  | -19.72 |  | -11.58 | NA |  | NA |  |
|  | -36.9 | NA |  | NA |  | NA |  | NA |  | NA |  | NA |  | NA |  |
|  | -36.9 | NA |  | NA |  | NA |  | NA |  | NA |  | NA |  | NA |  |
|  | -36.9 | NA |  | NA |  | NA |  | NA |  | NA |  | NA |  | NA |  |

| NA |  | -305.76 | | -1757.06 | | -1853.53 | | -1784.57 | | -807.32 | | -1518.84 | | -1652.23 | |
| --- | --- | --- | --- | --- | --- | --- | --- | --- | --- | --- | --- | --- | --- | --- | --- |
| NA |  | -440.92 | |  | -2165 | -2369.26 | | -2274.03 | | -1049.92 | | -1926.65 | | -2053.75 | |
| NA |  | -241.44 | |  | -973.2 | -1040.26 | |  | -890.5 | -480.15 | | -1023.58 | | -953.83 | |
| -2504.25 | | NA |  |  | -22.25 |  | -31.92 |  | -12.69 |  | -7.33 |  | -20.04 |  | -53.44 |
| -1785.94 | | NA |  | NA |  | NA |  | NA |  | NA |  | NA |  | NA |  |
| -1817.23 | | NA |  | NA |  | NA |  | NA |  | NA |  | NA |  | NA |  |
| -2084.12 | | NA |  | NA |  | NA |  | NA |  | NA |  | NA |  | NA |  |
|  | -80.78 | NA |  |  | -70.48 |  | -67.6 |  | -69.12 |  | -15.26 |  | -11.27 |  | -26.27 |
| -241.82 | |  | -67.72 |  | -84.64 | -123.79 | | -118.09 | | -118.36 | | -168.59 | |  | -237.2 |
|  | -415.3 |  | -59.58 |  | -43.12 |  | -69.36 |  | -94.51 |  | -99.02 | -144.26 | | -170.54 | |
| -494.46 | |  | -94.14 | -107.78 | | -130.01 | |  | -149.8 | -154.01 | | -151.87 | | -280.21 | |
| -494.46 | |  | -94.14 | -107.78 | | -130.01 | |  | -149.8 | -154.01 | | -151.87 | | -280.21 | |
| -390.06 | | -115.59 | | -142.05 | | -173.14 | | -191.29 | | -178.36 | | -160.47 | |  | -265.5 |
| NA |  | -147.48 | | -382.39 | | -431.85 | |  | -41.2 |  | -73.65 |  | -12.34 |  | -42.68 |
| NA |  | -147.48 | | -382.39 | | -431.85 | |  | -41.2 |  | -73.65 |  | -12.34 |  | -42.68 |
|  | -95.3 | NA |  |  | -35 |  | -28.14 |  | -44.75 | NA |  |  | -14.39 |  | -10.82 |
|  | -15.86 | NA |  |  | -21.59 |  | -12.53 | NA |  | NA |  | NA |  | NA |  |
|  | -48.64 | NA |  |  | -16.45 |  | -10.58 |  | -21.43 | NA |  | NA |  | NA |  |
| NA |  |  | -9.95 |  | -40.93 |  | -68.09 | NA |  | NA |  |  | -89.31 | -115.85 | |
|  | -197.1 | -861.53 | | -3400.19 | | -3556.8 | | -2639.76 | | -2002.15 | | -2468.82 | | -2773.16 | |
| NA |  | -1041.67 | | -10030.3 | | -9033.25 | | -5652.85 | | -2541.26 | | -4263.99 | | -3092.64 | |
| NA |  | -236.06 | | -4754.74 | | -4079.37 | | -1688.7 | | -663.88 | | -1631.9 | | -1602.56 | |
|  | -61.49 | -821.85 | | -5669.47 | | -5391.49 | | -3332.12 | | -1878.7 | | -3131.38 | | -2933.74 | |
|  | -5.76 | -1572.83 | | -14197.8 | |  | -12868 | -8049.68 | | -3764.56 | | -5787.97 | | -4538.58 | |
|  | -6.27 | -919.09 | | -9369.53 | | -8502.84 | | -5205.39 | | -2432.43 | | -4131.61 | | -3491.75 | |
|  | -101 |  | -54.98 | -392.64 | | -440.42 | |  | -203.6 | -122.27 | | -216.93 | | -226.29 | |
|  | -27.32 |  | -56.02 | -1894.37 | | -1615.5 | | -387.55 | | -122.08 | | -343.15 | | -289.56 | |
| -234.22 | |  | -43.67 | -306.03 | | -356.98 | | -180.27 | | -138.37 | | -241.12 | | -280.81 | |
| -160.56 | |  | -5.39 |  | -25.97 |  | -38.2 |  | -83.09 |  | -38.42 |  | -47.41 |  | -34.99 |
| -460.66 | | -262.06 | | -212.47 | | -297.26 | | -401.49 | |  | -360.4 | -376.08 | | -338.82 | |
| -373.48 | | -175.58 | | -417.84 | | -480.62 | | -537.55 | |  | -283.1 | -388.54 | | -330.27 | |
|  | -90.86 | NA |  | NA |  | NA |  |  | -6.01 | NA |  | NA |  | NA |  |
| -272.54 | | NA |  |  | -9.95 | NA |  | NA |  | NA |  | NA |  | NA |  |
|  | -82.32 | NA |  | NA |  | NA |  | NA |  | NA |  | NA |  | NA |  |
|  | -20.85 | NA |  | NA |  | NA |  | NA |  | NA |  | NA |  | NA |  |
|  | -67.16 | -145.11 | | NA |  | NA |  |  | -7.46 | -120.99 | |  | -33.75 |  | -53.88 |
|  | -328.6 | -206.88 | |  | -62.42 | -124.06 | | -120.71 | | -241.54 | | -198.69 | | -244.95 | |
| -113.21 | | -248.73 | |  | -17.25 |  | -53.27 |  | -67.92 | -228.84 | | -153.07 | | -143.44 | |
| NA |  |  | -8.19 | NA |  | NA |  | NA |  | NA |  | NA |  | NA |  |

| -264.69 | | -226.23 | |  | -35 |  | -85.19 |  | -93.92 | -241.92 | | -173.06 | | -194.03 | |
| --- | --- | --- | --- | --- | --- | --- | --- | --- | --- | --- | --- | --- | --- | --- | --- |
| -653.69 | |  | -346.7 |  | -337.8 | -469.21 | | -599.27 | | -500.13 | | -494.99 | | -500.42 | |
| -694.08 | | -138.17 | |  | -244.8 | -269.95 | | -290.29 | | -205.48 | | -148.95 | | -312.07 | |
|  | -544 | -115.51 | | -234.61 | | -251.42 | | -286.26 | | -201.81 | |  | -147.7 | -285.55 | |
| -378.56 | |  | -77.22 | -127.08 | | -148.77 | | -181.12 | | -138.06 | |  | -78.96 |  | -163.2 |
| -175.44 | |  | -33.42 |  | -42.17 |  | -59.03 |  | -100.3 |  | -45.33 |  | -48.28 |  | -65.11 |
| -527.31 | |  | -61.91 | NA |  | NA |  |  | -24.67 | -132.02 | |  | -15.1 |  | -24.36 |
| -641.87 | | -142.99 | | -220.28 | | -252.08 | | -274.48 | | -239.76 | | -152.92 | | -286.53 | |
| -511.62 | |  | -93.58 |  | -89.71 | -114.58 | | -154.76 | | -145.61 | |  | -76.16 | -177.57 | |
| NA |  | NA |  | NA |  | NA |  | NA |  | NA |  | NA |  | NA |  |
| NA |  | NA |  | NA |  | NA |  | NA |  | NA |  | NA |  | NA |  |
| NA |  | NA |  | NA |  | NA |  | NA |  | NA |  | NA |  | NA |  |
| -425.33 | | -596.33 | | -629.94 | | -759.21 | | -904.49 | | -788.18 | | -698.74 | | -921.75 | |
| -413.35 | | -227.24 | | -183.91 | | -240.05 | | -277.13 | |  | -266.7 |  | -232.5 | -375.01 | |
|  | -23.76 |  | -5.69 | NA |  | NA |  |  | -24.38 |  | -19.71 |  | -18.16 | NA |  |
| -208.93 | | -117.32 | |  | -67.5 | -109.88 | | -133.37 | | -159.31 | | -171.61 | | -351.65 | |
|  | -13.89 | NA |  | NA |  | NA |  |  | -18.97 |  | -12.72 |  | -16.96 | NA |  |
| NA |  |  | -20.81 | NA |  | NA |  |  | -9.81 |  | -10.21 |  | -18.27 |  | -44.1 |
| -163.08 | | -111.53 | |  | -7.05 |  | -27.04 |  | -40.71 | -112.71 | |  | -80.9 | -108.22 | |
| NA |  | NA |  | NA |  | NA |  | NA |  | NA |  | NA |  | NA |  |
| -857.79 | | -437.07 | |  | -415.8 | -510.88 | | -614.01 | | -607.65 | | -433.22 | | -703.07 | |
|  | -256 | NA |  |  | -22.08 |  | -29.37 |  | -17.78 |  | -31.52 |  | -15.51 |  | -57.95 |
|  | -26.86 |  | -77.54 | NA |  | NA |  | NA |  |  | -9.27 | NA |  | NA |  |
|  | -67.09 |  | -50.85 | NA |  | NA |  | NA |  |  | -12.47 | NA |  | NA |  |
|  | -96 | -175.85 | |  | -29.45 |  | -70.77 |  | -91.81 |  | -165.5 | -154.02 | | -184.57 | |
|  | -48.88 |  | -67.71 | -139.92 | | -194.14 | | -105.54 | |  | -88.81 | -286.56 | | -330.63 | |
|  | -76.41 |  | -185.7 | -121.25 | | -165.17 | |  | -143.5 |  | -175.5 | -236.37 | | -227.24 | |
| -132.86 | | -181.98 | |  | -36.8 |  | -72.83 | -100.69 | | -221.66 | | -120.78 | | -173.12 | |
|  | -25.25 |  | -22.47 |  | -60.75 |  | -74.19 |  | -21.1 |  | -29.87 | -103.98 | | -154.22 | |
| -436.89 | | -173.22 | | -241.13 | | -288.21 | | -363.56 | | -302.43 | | -233.24 | | -411.38 | |
| -314.24 | |  | -71.32 |  | -335.1 | -381.58 | | -191.27 | | -116.95 | | -364.55 | | -581.34 | |
| -422.89 | |  | -252.4 | -288.04 | | -332.09 | | -411.25 | | -398.22 | | -270.86 | |  | -439.6 |
| -206.05 | |  | -46.03 | -151.01 | | -181.13 | | -184.63 | | -114.23 | | -123.65 | |  | -258.6 |
|  | -257.2 |  | -12.03 |  | -184.9 | -212.56 | | -208.39 | |  | -79.11 | -174.03 | | -268.33 | |
|  | -20.77 | -121.84 | |  | -40.56 |  | -54.37 |  | -31.01 | -114.23 | |  | -38.26 | -120.78 | |
|  | -53.65 |  | -70.14 | -633.47 | | -598.73 | | -316.35 | |  | -208 | -406.08 | | -464.86 | |
|  | -29.95 | -165.78 | | -1477.52 | | -1382.58 | | -866.02 | |  | -544.6 | -781.61 | | -864.41 | |
| -183.22 | |  | -64.9 |  | -62.85 |  | -102.7 |  | -70.29 |  | -86.19 | -118.17 | | -180.16 | |
| -358.54 | | -345.73 | | -240.75 | | -280.26 | | -403.95 | | -332.96 | | -201.37 | | -361.93 | |

| -115.69 | |  | -93.49 | -140.49 | |  | -179.4 | -198.92 | | -183.87 | |  | -174.4 | -212.49 | |
| --- | --- | --- | --- | --- | --- | --- | --- | --- | --- | --- | --- | --- | --- | --- | --- |
|  | -235.5 | -139.13 | | -321.38 | | -408.89 | | -309.27 | | -270.39 | | -427.29 | | -479.97 | |
| -1312.97 | | -138.14 | |  | -384.6 | -429.77 | | -450.83 | |  | -299.7 | -244.53 | | -503.01 | |
|  | -95.95 |  | -19.75 |  | -24.58 |  | -47.15 |  | -59.31 |  | -61.95 |  | -48.97 |  | -93.44 |
| -1125.88 | | -403.14 | | -1528.59 | | -1553.76 | | -1678.83 | | -928.26 | | -688.63 | | -1299.02 | |
| -246.31 | | NA |  |  | -66.06 | NA |  | NA |  | NA |  | NA |  | -158.65 | |
| -397.15 | | -246.89 | | -448.37 | | -504.48 | | -488.07 | | -378.87 | | -469.72 | | -510.25 | |
| -200.43 | | NA |  |  | -46.28 |  | -68.92 |  | -97.29 | -165.33 | |  | -90.25 | -119.25 | |
| -411.91 | | -146.18 | | -106.64 | | -133.32 | | -163.93 | | -225.79 | | -167.84 | | -239.46 | |
| -115.23 | |  | -36.13 | -127.59 | | -142.54 | | -139.82 | |  | -87.36 | -102.73 | | -195.05 | |
| -211.45 | |  | -81.14 | -139.26 | | -169.82 | |  | -85.19 |  | -114 | -192.95 | | -296.71 | |
| NA |  | NA |  | NA |  | NA |  | NA |  | NA |  | NA |  | NA |  |
|  | -27.02 |  | -52.68 |  | -184.2 | -213.68 | | -168.88 | | -188.71 | |  | -183 | -212.42 | |
|  | -24.92 |  | -47.14 |  | -33.45 |  | -45.18 |  | -9.41 |  | -32.74 | -107.69 | | -117.65 | |
|  | -83.16 |  | -95.32 |  | -96.54 | -128.52 | |  | -81.91 |  | -101.9 | -216.35 | |  | -341.1 |
| -523.16 | | -484.11 | | -429.83 | |  | -558 | -643.13 | | -632.69 | | -620.14 | | -648.42 | |
| -122.04 | |  | -43.18 | NA |  | NA |  |  | -10.65 |  | -26.32 |  | -22.79 |  | -43.18 |
| -250.08 | | -232.67 | |  | -55.5 | -106.91 | | -186.33 | | -280.26 | | -208.96 | | -192.86 | |
|  | -45.52 | NA |  |  | -7.9 | NA |  | NA |  | NA |  | NA |  |  | -57.01 |
|  | -60 |  | -19.08 | -127.65 | | -135.27 | |  | -95.71 |  | -82.3 | -113.62 | | -170.29 | |
| -186.12 | |  | -36 | -283.55 | | -372.22 | | -245.39 | | -110.65 | | -300.79 | | -476.15 | |
| NA |  | NA |  |  | -518.8 | -355.45 | | -101.97 | |  | -10.11 | -121.35 | | -252.46 | |
| -272.02 | |  | -149 |  | -57.31 | -102.12 | | -114.77 | | -191.27 | | -141.96 | |  | -206.9 |
| NA |  |  | -69.61 | -691.31 | | -640.29 | | -238.06 | | -180.23 | |  | -580 |  | -602.8 |
| -348.05 | |  | -10.12 |  | -45.16 |  | -81.45 |  | -87.28 | -165.82 | | -159.46 | | -212.51 | |
|  | -77.25 |  | -79.58 | -667.26 | | -564.88 | | -361.41 | | -189.82 | | -314.15 | | -385.71 | |
| -424.14 | |  | -66.94 | -234.91 | | -246.34 | | -252.66 | | -169.39 | |  | -180 | -310.93 | |
|  | -43.15 |  | -46.07 | -125.05 | | -174.01 | |  | -41.51 |  | -48.22 | -292.08 | | -284.17 | |
| -273.05 | |  | -59.92 |  | -96.66 | -104.85 | | -157.54 | | -107.65 | |  | -77.7 | -191.78 | |
| -136.42 | | NA |  |  | -68.34 |  | -84.49 |  | -81.3 |  | -35.48 |  | -40.15 |  | -115.1 |
|  | -21.03 |  | -72.44 | -753.18 | | -714.04 | | -343.65 | | -162.62 | | -420.81 | | -497.54 | |
| -433.31 | | -350.71 | | -198.27 | | -249.25 | |  | -244.9 | -320.27 | | -295.26 | | -484.04 | |
| -128.68 | |  | -94.72 |  | -87.99 | -126.39 | |  | -61.53 |  | -97.62 | -211.46 | | -231.59 | |
|  | -32.33 |  | -35.09 | -276.28 | | -299.17 | |  | -318.9 | -108.39 | | -249.32 | | -218.64 | |
|  | -24.8 | NA |  | NA |  | NA |  | NA |  | NA |  | NA |  |  | -12.03 |
| -326.97 | |  | -65.54 |  | -69.43 |  | -93.02 | -117.09 | | -116.01 | |  | -72.43 | -146.51 | |
| -129.63 | |  | -65.14 | -181.61 | | -205.24 | | -204.35 | | -140.98 | | -161.49 | | -219.76 | |
|  | -80.47 |  | -58.77 | -282.93 | | -302.16 | |  | -93.13 |  | -72.89 | -307.48 | | -434.48 | |

memory_b mesangial mesenchy monocyte. monocyte. monocyte. monocyte_multipoten

-139.15	-205.2	-581.76	-204.14	-162.88	-153.09	-272.2	-214.48

NA	NA	NA	NA	NA	NA	NA	NA

-352.32	-193.26	-2082.68	-307.95	-302.11	-299.82	-320.54	-152.75

-525.29	-185.03	-2847.81	-197.52	-101.55	-111.32	-188.98	-104.65

-217.43	-35.41	-1245.16	-30.17	-11.98	-7.59	-43.17	-9.84

-546.92	-672.3	-1751.39	-748.61	-573.35	-488.61	-676.78	-694.14

-427.8	-140.92	-2949.23	-322.56	-273.59	-255.5	-329.38	-129.04

-181.61	-63.5	-1695.3	-52.72	-27.01	-25.28	-75.08	-33.18

-181.61	-63.5	-1695.3	-52.72	-27.01	-25.28	-75.08	-33.18

-14.84	-82.19	-316.65	-162.73	-178.32	-175.92	-122.09 NA

-243.53	-520.99	-1592.72	-509.02	-427.64	-353.08	-452.88	-403.92

-247.7	-163.09	-1074.41	-142.62	-88.99	-59.78	-166.54	-134.85

-140.66	-367.66	-788.44	-542.87	-474.01	-394.26	-353.02	-27.88

-237.52	-638.85	-1369.89	-579.69	-478.68	-374.53	-426.54	-61.19

NA	NA	-126.39 NA	NA	NA	NA	NA NA	NA	NA	NA	NA	NA	NA	NA

-27.44	-75.27	-36.58	-71.23	-150.04	-115.78	-26.23 NA

-27.44	-75.27	-36.58	-71.23	-150.04	-115.78	-26.23 NA

-621.91	-77.36	-144.31	-4466.41	-5571.37	-6196.42	-3925.57	-1815.54

-487.31	-136.61	-2682.17	-238.24	-190.69	-194.05	-262.89	-141.44

NA	NA	NA	NA	NA	NA	NA	NA

-464.18	-104.19	-2058.45	-1642.43	-1940.15	-1763.85	-1536.61	-1089.26

NA	NA	NA	NA	NA	NA	NA	NA NA	NA	NA	NA	NA	NA	NA	NA NA	NA	-11.87	-286.39	-532.93	-499.51	-243.51 NA NA	NA	-59.21	-1069.84	-1624.68	-1652.3	-834.71 NA NA	NA	-29.34	-1169.27	-1710.16	-1747.13	-898.76 NA NA	NA	NA	-737.09	-1144.7	-1129.01	-542.41 NA

-323.98	-164.13	-2273.14	-241.34	-293.96	-278.16	-312.37	-167.08

-323.98	-164.13	-2273.14	-241.34	-293.96	-278.16	-312.37	-167.08

-526.63	-280.87	-3285.84	-413.52	-445.53	-393.48	-472.66	-258.56

-546.62	-256.19	-3571.35	-485.42	-529.57	-485.66	-542.64	-309.96

NA	-74.28	-177.77	-173.29	-256.49	-258.85	-147.38 NA NA	NA	NA	-414.11	-884.62	-898.75	-342.21 NA NA	NA	NA	NA	NA	NA	NA	NA NA	NA	NA	NA	NA	NA	NA	NA NA	NA	NA	NA	NA	NA	NA	NA

-88.26	-186.06	-652.04	-183.57	-76.8	-129.51	-252.71	-289.17

| -363.22 | | -249.32 | | -1627.6 | |  | -59.72 |  | -18.97 |  | -9.46 |  | -83.01 |  | -66.26 |
| --- | --- | --- | --- | --- | --- | --- | --- | --- | --- | --- | --- | --- | --- | --- | --- |
| -363.22 | | -249.32 | | -1627.6 | |  | -59.72 |  | -18.97 |  | -9.46 |  | -83.01 |  | -66.26 |
| -363.22 | | -249.32 | | -1627.6 | |  | -59.72 |  | -18.97 |  | -9.46 |  | -83.01 |  | -66.26 |
| -169.05 | | -364.24 | | -568.47 | | -148.03 | |  | -66.23 |  | -70.95 | -246.59 | |  | -213.5 |
|  | -151 | -315.72 | | -511.13 | | -106.72 | |  | -39.57 |  | -38.9 | -178.08 | | -155.98 | |
| -711.11 | | -216.59 | | -383.75 | |  | -5100 | -5752.03 | | -6032.86 | | -4991.45 | | -4052.06 | |
| -973.96 | | -420.67 | | -877.14 | | -6471.61 | | -7917.15 | | -7951.12 | | -6173.84 | | -4646.11 | |
| -1579.71 | | -493.45 | | -1214.47 | | -9279.44 | | -10836.4 | | -10978.4 | | -8823.73 | | -6789.2 | |
| -1097.89 | | -425.29 | | -1037.98 | | -6756.44 | | -7700.66 | | -7888.02 | | -6678.98 | | -5515.34 | |
| -736.44 | | -327.51 | | -707.16 | | -4180.8 | | -5058.85 | | -5072.17 | | -3903.58 | | -2945.9 | |
|  | -996.4 | -373.91 | | -696.16 | | -5479.16 | | -6418.8 | | -6390.26 | | -5165.73 | | -3935.44 | |
| -1185.36 | | -321.76 | | -626.42 | | -8789.9 | | -10995.4 | | -11188.4 | | -8219.08 | | -5937.7 | |
| -916.88 | | -529.95 | | -2037.9 | | -4987.59 | | -5687.56 | | -5366.06 | | -4762.55 | | -3995.15 | |
| -1174.96 | | -503.29 | | -1421.13 | | -6129.08 | | -7230.25 | | -6922.1 | | -5654.3 | | -4814.48 | |
| -1381.35 | | -597.25 | | -1779.33 | | -7158.55 | | -8240.99 | | -7963.53 | | -6763.9 | | -5730.89 | |
| NA |  | NA |  | NA |  | NA |  | NA |  | NA |  | NA |  | NA |  |
| -335.98 | |  | -81 | -3248.58 | |  | -65.55 |  | -41.51 |  | -34.95 | -115.41 | |  | -30.3 |
| -1397.52 | | -479.05 | | -1408.75 | | -7370.69 | | -8828.74 | | -8558.42 | | -7018.38 | | -6019.85 | |
| -1670.31 | | -516.06 | | -1685.89 | | -8272.98 | | -9854.17 | | -9527.63 | | -7723.45 | | -6630.61 | |
| -125.93 | |  | -95.94 | -388.62 | | -583.03 | | -664.45 | | -634.66 | | -526.02 | | -495.36 | |
| -1879.32 | | -447.03 | | -983.11 | | -10677.7 | | -12558.6 | | -12408.2 | | -10275.8 | | -8720.2 | |
| NA |  | NA |  | -181.59 | | NA |  | NA |  | NA |  | NA |  | NA |  |
| NA |  | NA |  | NA |  | NA |  | NA |  | NA |  | NA |  | NA |  |
| -1707.49 | | -468.14 | | -1330.26 | | -9227.54 | |  | -11012 | -10702.6 | | -8735.5 | | -7378.75 | |
|  | -94.72 |  | -49.42 | -204.09 | | -377.71 | | -346.35 | | -367.42 | | -377.52 | | -298.38 | |
| -751.53 | | -261.12 | | -663.88 | | -5436.23 | | -6369.92 | | -6440.59 | | -5327.99 | | -3657.11 | |
| -1635.58 | | -545.41 | | -1662.03 | | -8794.95 | | -10219.9 | | -9893.21 | | -8262.33 | | -7034.24 | |
| -1226.21 | | -349.66 | | -950.28 | | -6446.19 | | -8111.33 | | -7880.51 | | -6033.62 | | -5320.34 | |
| -558.68 | | -261.81 | | -1077.31 | | -2571.3 | | -3110.2 | | -2928.98 | | -2482.59 | | -2109.86 | |
| -558.68 | | -261.81 | | -1077.31 | | -2571.3 | | -3110.2 | | -2928.98 | | -2482.59 | | -2109.86 | |
| -1801.89 | | -479.79 | | -1322.88 | | -9874.87 | | -11710.2 | | -11380.8 | | -9417.17 | | -7810.4 | |
| -785.56 | | -298.85 | | -1238.33 | | -4111.38 | | -4562.88 | | -4422.82 | | -3905.96 | | -3339.98 | |
| -558.55 | |  | -80.05 | -228.02 | | -3566.87 | | -3880.33 | | -4178.58 | | -3638.83 | | -2455.11 | |
| -619.55 | | -189.96 | |  | -699.7 | -3202.88 | | -3607.93 | | -3583.13 | | -2982.41 | | -2365.87 | |
| NA |  | NA |  | NA |  | NA |  | NA |  | NA |  | NA |  | NA |  |
| -250.52 | |  | -175.9 |  | -952.6 | -196.75 | | -135.45 | |  | -87.03 | -203.09 | | -161.52 | |
| -1480.3 | | -364.12 | | -1046.7 | | -9288.86 | | -11543.2 | | -11361.9 | | -8670.32 | | -6881.93 | |
| -1892.21 | | -546.99 | | -1359.53 | | -10605.4 | | -12445.7 | | -12196.8 | | -10076.9 | | -8564.98 | |
| -151.65 | | -114.95 | | -499.08 | | -627.82 | | -709.88 | | -687.18 | | -565.29 | | -546.72 | |

| -122.33 | | -594.04 | | -775.61 | | -533.36 | | -544.64 | |  | -443.6 | -315.73 | |  | -26.06 |
| --- | --- | --- | --- | --- | --- | --- | --- | --- | --- | --- | --- | --- | --- | --- | --- |
|  | -91.81 | -501.48 | | -534.58 | | -542.23 | | -606.73 | | -505.76 | | -338.97 | |  | -39.08 |
| -222.48 | | -636.83 | | -1001.78 | | -750.36 | | -699.06 | | -558.61 | | -518.39 | |  | -75.86 |
| -103.58 | | -405.37 | | -888.96 | | -262.35 | | -201.66 | | -142.92 | | -124.84 | | NA |  |
| NA |  | NA |  | NA |  | NA |  | NA |  | NA |  | NA |  | NA |  |
| NA |  | NA |  | NA |  | NA |  | NA |  | NA |  | NA |  | NA |  |
| NA |  | NA |  | NA |  | NA |  | NA |  | NA |  | NA |  | NA |  |
| NA |  | NA |  | NA |  | NA |  | NA |  | NA |  | NA |  | NA |  |
| NA |  |  | -43.44 | NA |  | NA |  | NA |  | NA |  | NA |  | NA |  |
| NA |  |  | -50.49 | NA |  | NA |  | NA |  | NA |  | NA |  | NA |  |
| NA |  | NA |  | NA |  | NA |  | NA |  | NA |  | NA |  | NA |  |
| NA |  | NA |  | NA |  | NA |  | NA |  | NA |  | NA |  | NA |  |
| NA |  | NA |  | NA |  | NA |  | NA |  | NA |  | NA |  | NA |  |
| NA |  | NA |  | NA |  | NA |  | NA |  | NA |  | NA |  | NA |  |
| NA |  |  | -41.91 | NA |  | NA |  | NA |  | NA |  | NA |  | NA |  |
| NA |  |  | -64.83 | NA |  | NA |  |  | -19.26 |  | -17.78 | NA |  |  | -5.49 |
| NA |  | NA |  | NA |  | NA |  | NA |  | NA |  | NA |  | NA |  |
| NA |  |  | -6.46 | NA |  | NA |  | NA |  | NA |  | NA |  | NA |  |
| NA |  |  | -87.41 | NA |  |  | -17.85 |  | -57.67 |  | -55.6 |  | -6.45 |  | -39.62 |
| NA |  |  | -63.63 | NA |  | NA |  |  | -12.11 |  | -10.28 | NA |  | NA |  |
| NA |  | NA |  | NA |  | NA |  | NA |  | NA |  | NA |  | NA |  |
| -1479.68 | | -595.68 | | -1626.62 | | -7851.82 | | -9048.43 | | -8800.49 | | -7609.64 | | -6372.63 | |
| NA |  | NA |  |  | -10.91 | -104.36 | | -176.45 | | -197.42 | | -102.79 | | -145.49 | |
| NA |  | NA |  |  | -10.91 | -104.36 | | -176.45 | | -197.42 | | -102.79 | | -145.49 | |
| NA |  | NA |  | NA |  | NA |  |  | -13.91 |  | -37.86 | NA |  | NA |  |
| NA |  | NA |  | NA |  | NA |  |  | -13.91 |  | -37.86 | NA |  | NA |  |
| NA |  | NA |  | NA |  | NA |  |  | -20.87 |  | -35.68 | NA |  |  | -19.39 |
| NA |  | NA |  | NA |  |  | -59.92 | -138.72 | | -166.35 | |  | -52.69 | -174.03 | |
| NA |  | NA |  | NA |  | NA |  | NA |  | NA |  | NA |  | NA |  |
| NA |  | NA |  | NA |  | NA |  | NA |  | NA |  | NA |  | NA |  |
| NA |  | NA |  | NA |  | NA |  | NA |  | NA |  | NA |  | NA |  |
| NA |  | NA |  | NA |  | NA |  | NA |  | NA |  | NA |  | NA |  |
| NA |  | NA |  | NA |  | NA |  | NA |  | NA |  | NA |  | NA |  |
| NA |  | NA |  | NA |  | NA |  | NA |  | NA |  | NA |  | NA |  |
| NA |  | NA |  | NA |  | NA |  | NA |  | NA |  | NA |  | NA |  |
| NA |  | NA |  | NA |  | NA |  | NA |  | NA |  | NA |  | NA |  |
|  | -30.44 | -166.64 | | -1228.86 | |  | -76.2 |  | -41.63 |  | -35.58 |  | -63.46 |  | -39.31 |
| -364.29 | | -100.13 | | -2382.91 | |  | -89.69 |  | -17.49 |  | -15.64 | -107.41 | |  | -45.02 |
| -364.29 | | -100.13 | | -2382.91 | |  | -89.69 |  | -17.49 |  | -15.64 | -107.41 | |  | -45.02 |

| -557.11 | | -170.86 | | -3733.4 | | -170.34 | |  | -48.4 |  | -51.84 | -193.67 | |  | -88.39 |
| --- | --- | --- | --- | --- | --- | --- | --- | --- | --- | --- | --- | --- | --- | --- | --- |
|  | -71.84 | NA |  | -518.08 | | NA |  | NA |  | NA |  | NA |  | NA |  |
| -342.73 | | -144.83 | | -1279.55 | | -202.75 | | -124.49 | | -134.26 | | -244.87 | | -162.12 | |
|  | -80.06 |  | -42.98 | -990.26 | |  | -8.05 | NA |  | NA |  |  | -12.18 | NA |  |
| -217.77 | | -239.31 | | -1417.55 | | -429.35 | | -328.64 | | -324.82 | | -425.05 | | -313.04 | |
| NA |  | NA |  | NA |  | -378.44 | | -745.75 | | -779.04 | |  | -311.6 | NA |  |
| NA |  | NA |  | NA |  | NA |  | NA |  | NA |  | NA |  | NA |  |
| NA |  |  | -10.96 | NA |  | NA |  | NA |  | NA |  | NA |  | NA |  |
|  | -5.13 | -117.45 | | -628.36 | |  | -10.85 | NA |  | NA |  | NA |  | NA |  |
| NA |  | -116.78 | | -447.84 | |  | -10.8 | NA |  | NA |  | NA |  | NA |  |
|  | -39.13 | -149.94 | | -1000.67 | |  | -69.77 |  | -33.11 |  | -17.42 |  | -55.8 |  | -18.15 |
| NA |  | NA |  | NA |  | NA |  | NA |  | NA |  | NA |  | NA |  |
| NA |  | NA |  | NA |  | NA |  | NA |  | NA |  | NA |  | NA |  |
| NA |  | NA |  | NA |  | NA |  | NA |  | NA |  | NA |  | NA |  |
| NA |  | NA |  | NA |  | NA |  | NA |  | NA |  | NA |  | NA |  |
| NA |  | NA |  | NA |  | NA |  | NA |  | NA |  | NA |  | NA |  |
| NA |  | NA |  | NA |  | NA |  | NA |  | NA |  | NA |  | NA |  |
| NA |  |  | -9.74 | NA |  | NA |  | NA |  | NA |  | NA |  | NA |  |
| NA |  | NA |  | NA |  | NA |  | NA |  | NA |  | NA |  | NA |  |
| NA |  | -124.88 | | NA |  | NA |  |  | -27.86 |  | -30.09 | NA |  |  | -42.79 |
| NA |  | NA |  | NA |  | NA |  | NA |  | NA |  | NA |  | NA |  |
| NA |  |  | -16.58 | NA |  | NA |  | NA |  | NA |  | NA |  | NA |  |
| NA |  |  | -17.85 | NA |  | NA |  | NA |  | NA |  | NA |  | NA |  |
| NA |  |  | -17.72 | NA |  | NA |  | NA |  | NA |  | NA |  | NA |  |
| NA |  | NA |  | NA |  | NA |  | NA |  | NA |  | NA |  | NA |  |
| NA |  | -117.17 | | NA |  | NA |  |  | -31.42 |  | -31.17 | NA |  |  | -26.54 |
| NA |  | NA |  | NA |  | NA |  | NA |  | NA |  | NA |  | NA |  |
| NA |  | NA |  | NA |  | NA |  | NA |  | NA |  | NA |  | NA |  |
| NA |  | NA |  | NA |  | NA |  | NA |  | NA |  | NA |  | NA |  |
| NA |  | NA |  | NA |  | NA |  | NA |  | NA |  | NA |  | NA |  |
| NA |  | -258.92 | |  | -23.57 |  | -21.27 |  | -53.44 |  | -45.48 |  | -11.14 |  | -25.52 |
| NA |  | NA |  | NA |  | NA |  | NA |  | NA |  | NA |  | NA |  |
| NA |  |  | -40.9 | NA |  | NA |  | NA |  | NA |  | NA |  | NA |  |
| NA |  |  | -16.72 | NA |  | NA |  | NA |  | NA |  | NA |  | NA |  |
| NA |  | NA |  | NA |  | NA |  | NA |  | NA |  | NA |  | NA |  |
| NA |  |  | -32 | NA |  | NA |  |  | -8.63 |  | -13.35 | NA |  | NA |  |
| NA |  | NA |  | NA |  | NA |  | NA |  | NA |  | NA |  | NA |  |
| NA |  | NA |  | NA |  | NA |  |  | -7.53 |  | -11.22 | NA |  | NA |  |
| NA |  | NA |  | NA |  | NA |  | NA |  | NA |  | NA |  | NA |  |

| NA |  |  | -39.46 | NA |  | NA |  | NA |  | NA |  | NA |  | NA |  |
| --- | --- | --- | --- | --- | --- | --- | --- | --- | --- | --- | --- | --- | --- | --- | --- |
| NA |  | -127.39 | | NA |  | NA |  |  | -63.15 |  | -55.11 | NA |  |  | -68.29 |
| NA |  | NA |  | NA |  | NA |  | NA |  | NA |  | NA |  | NA |  |
| NA |  | NA |  | NA |  | NA |  | NA |  | NA |  | NA |  | NA |  |
| NA |  | NA |  | NA |  | NA |  | NA |  | NA |  | NA |  | NA |  |
| NA |  |  | -21.68 | NA |  | NA |  |  | -32.07 |  | -36.39 | NA |  | NA |  |
| NA |  | NA |  | NA |  | NA |  | NA |  | NA |  | NA |  | NA |  |
| NA |  | NA |  | NA |  | NA |  | NA |  | NA |  | NA |  | NA |  |
| NA |  | NA |  | NA |  | NA |  | NA |  | NA |  | NA |  | NA |  |
| NA |  |  | -12.96 | NA |  | NA |  |  | -15.37 |  | -17.65 | NA |  | NA |  |
| -213.63 | |  | -48.3 | -135.22 | | -1724.48 | | -2452.98 | | -2419.14 | | -1743.52 | | -1231.16 | |
| NA |  | NA |  | -2491.46 | |  | -29.26 | -534.68 | | -535.26 | |  | -24.42 | NA |  |
| NA |  | NA |  | NA |  |  | -90.93 | -368.03 | | -484.01 | |  | -42.75 | NA |  |
|  | -102.2 | NA |  |  | -61.72 | -857.28 | | -1386.29 | | -1640.71 | | -656.36 | | -303.73 | |
| -163.37 | |  | -65.64 | -256.51 | | -459.63 | |  | -697.4 | -688.15 | | -322.86 | | -145.71 | |
| -135.49 | |  | -19.84 | -702.56 | | -305.36 | | -446.55 | | -456.94 | | -213.46 | |  | -76.36 |
|  | -203.9 | NA |  | -1428.05 | |  | -42.08 |  | -43.8 |  | -62.22 |  | -28.17 | NA |  |
| NA |  | NA |  |  | -88.2 |  | -143.9 | -396.57 | | -442.91 | |  | -66.96 |  | -9.98 |
| -247.72 | |  | -47.54 | -274.37 | | -1076.28 | | -1644.72 | | -1739.08 | | -804.38 | | -269.02 | |
|  | -30.92 | NA |  |  | -17.35 | -195.78 | | -374.28 | | -400.73 | | -109.95 | |  | -17.33 |
|  | -88.52 | -522.83 | | -562.77 | | -340.46 | | -352.58 | | -263.79 | | -201.14 | | NA |  |
| -144.38 | | -541.62 | | -725.51 | | -414.94 | | -399.01 | | -320.91 | | -238.72 | |  | -8.76 |
| -181.06 | | -526.33 | | -540.81 | | -612.39 | | -627.65 | | -511.15 | | -403.91 | |  | -38.4 |
|  | -66.69 | -308.07 | | -558.94 | | -178.19 | |  | -163.7 | -112.75 | |  | -77.77 | NA |  |
|  | -23.35 |  | -81.48 | -212.72 | |  | -61.27 |  | -7.28 | NA |  | -111.76 | |  | -21.05 |
| -111.38 | |  | -64.94 | -975.42 | |  | -76.71 | -110.27 | |  | -79.95 | -136.51 | |  | -68.61 |
| -116.14 | |  | -85.82 | -830.98 | |  | -84.56 |  | -13.71 |  | -7.78 |  | -98.37 |  | -14 |
|  | -95.96 |  | -43.58 | -358.93 | |  | -70.07 |  | -21.44 |  | -22.11 |  | -78.47 |  | -37.49 |
|  | -95.96 |  | -43.58 | -358.93 | |  | -70.07 |  | -21.44 |  | -22.11 |  | -78.47 |  | -37.49 |
|  | -89.89 |  | -64.9 | -365.42 | |  | -21.21 | NA |  | NA |  |  | -77.98 |  | -31.43 |
|  | -79.86 | -270.94 | |  | -343 | -112.27 | |  | -26.77 |  | -36.8 | -268.63 | | -197.19 | |
|  | -35.32 | -129.26 | | -344.85 | |  | -49.35 | NA |  | NA |  | -112.05 | |  | -31.03 |
|  | -35.32 | -129.26 | | -344.85 | |  | -49.35 | NA |  | NA |  | -112.05 | |  | -31.03 |
|  | -35.32 | -129.26 | | -344.85 | |  | -49.35 | NA |  | NA |  | -112.05 | |  | -31.03 |
|  | -61.71 | -356.89 | | -269.65 | | -206.33 | |  | -90.48 | -123.69 | | -358.98 | | -229.84 | |
| -140.98 | | -404.34 | | -920.74 | | -264.58 | | -115.06 | | -136.95 | | -412.57 | | -392.39 | |
|  | -64.43 |  | -33.77 | -431.31 | |  | -14.06 | NA |  | NA |  |  | -41.61 | NA |  |
| NA |  |  | -5.13 | NA |  | NA |  | NA |  | NA |  | NA |  | NA |  |
| NA |  | NA |  |  | -16.09 |  | -20.69 |  | -42.07 |  | -66.6 | NA |  | NA |  |

| NA |  | NA |  | NA | NA |  | NA |  | NA |  | NA |  | NA |  |
| --- | --- | --- | --- | --- | --- | --- | --- | --- | --- | --- | --- | --- | --- | --- |
| NA |  | NA |  | NA | NA |  | NA |  | NA |  | NA |  | NA |  |
| NA |  | NA |  | NA | NA |  | NA |  | NA |  | NA |  | NA |  |
| NA |  | NA |  | NA | NA |  | NA |  | NA |  | NA |  | NA |  |
|  | -324.2 | -428.35 | | -1589.1 | -369.32 | | -194.55 | | -168.36 | | -313.16 | | -292.69 | |
| -144.55 | | -309.37 | | -1304.69 | -360.34 | | -317.43 | | -282.66 | | -311.09 | | -230.36 | |
| -119.88 | | -199.19 | | -1599.22 | -130.31 | |  | -43.28 |  | -32.22 | -110.13 | |  | -99.67 |
| -252.17 | | -414.18 | | -1495.05 | -559.24 | | -466.76 | |  | -458.2 | -483.25 | | -398.49 | |
|  | -97.36 |  | -89.51 | -544.27 | -123.73 | |  | -50.42 |  | -47.08 |  | -75.3 |  | -17.79 |
| -111.06 | | -115.97 | | -655.61 | -209.29 | | -138.02 | | -130.91 | | -131.11 | |  | -65.07 |
| -404.56 | | -184.56 | | -2031.1 | -279.19 | | -251.56 | | -223.95 | | -324.64 | | -164.15 | |
| -179.78 | | -408.93 | | -1759.33 | -237.29 | | -144.53 | | -112.94 | | -222.19 | | -264.03 | |
| -198.89 | | -757.11 | | -2434.98 | -459.62 | | -335.53 | |  | -324.4 | -458.18 | | -351.63 | |
|  | -75 |  | -74.01 | -463.37 |  | -54.2 |  | -43.91 |  | -22.98 |  | -34.74 |  | -33.4 |
| NA |  |  | -33.34 | NA | NA |  | NA |  | NA |  | NA |  | NA |  |
| NA |  | NA |  | NA | NA |  | NA |  | NA |  | NA |  | NA |  |
| -418.24 | |  | -59.62 | -2087.54 | -131.71 | | -116.97 | | -108.47 | | -182.22 | |  | -81.58 |
| NA |  | NA |  | NA | NA |  | NA |  | NA |  | NA |  | NA |  |
| -265.34 | | -307.13 | | -728.24 | -323.55 | | -273.64 | | -222.39 | | -290.21 | | -348.39 | |
| NA |  | NA |  | NA | NA |  | NA |  | NA |  | NA |  | NA |  |
| NA |  | NA |  | NA | NA |  | NA |  | NA |  | NA |  | NA |  |
| -159.98 | | -133.02 | | -816.51 | -178.18 | |  | -158.7 | -142.57 | | -188.59 | |  | -117.5 |
| -308.95 | | -199.66 | | -2708.51 | -287.33 | | -272.23 | | -240.48 | | -377.09 | | -170.08 | |
| -308.95 | | -199.66 | | -2708.51 | -287.33 | | -272.23 | | -240.48 | | -377.09 | | -170.08 | |
| -308.95 | | -199.66 | | -2708.51 | -287.33 | | -272.23 | | -240.48 | | -377.09 | | -170.08 | |
| -308.95 | | -199.66 | | -2708.51 | -287.33 | | -272.23 | | -240.48 | | -377.09 | | -170.08 | |
|  | -127.3 | -234.25 | | -1076.37 | -580.63 | | -578.51 | | -546.13 | | -567.46 | | -425.52 | |
|  | -127.3 | -234.25 | | -1076.37 | -580.63 | | -578.51 | | -546.13 | | -567.46 | | -425.52 | |
|  | -127.3 | -234.25 | | -1076.37 | -580.63 | | -578.51 | | -546.13 | | -567.46 | | -425.52 | |
|  | -127.3 | -234.25 | | -1076.37 | -580.63 | | -578.51 | | -546.13 | | -567.46 | | -425.52 | |
|  | -93.77 | -175.02 | | -1562.4 | -303.34 | | -522.36 | |  | -523.4 | -256.69 | | -188.46 | |
|  | -93.77 | -175.02 | | -1562.4 | -303.34 | | -522.36 | |  | -523.4 | -256.69 | | -188.46 | |
|  | -48.03 |  | -50.68 | -1001.5 | -225.96 | |  | -329.8 | -333.39 | | -179.41 | | -152.99 | |
|  | -48.03 |  | -50.68 | -1001.5 | -225.96 | |  | -329.8 | -333.39 | | -179.41 | | -152.99 | |
| -691.49 | | -327.18 | | -4023.59 | -492.77 | |  | -464.9 | -432.34 | | -584.49 | | -326.77 | |
| -417.37 | | -445.77 | | -1215.32 | -343.89 | | -201.27 | | -162.13 | | -320.67 | | -361.36 | |
| -417.37 | | -445.77 | | -1215.32 | -343.89 | | -201.27 | | -162.13 | | -320.67 | | -361.36 | |
| -216.36 | | -265.42 | | -524.09 | -383.53 | | -371.51 | | -313.75 | |  | -322.4 | -346.31 | |
| -216.36 | | -265.42 | | -524.09 | -383.53 | | -371.51 | | -313.75 | |  | -322.4 | -346.31 | |

| -399.34 | | -299.97 | | -1121.51 | | -293.16 | | -147.47 | | -114.24 | | -270.15 | | -321.99 | |
| --- | --- | --- | --- | --- | --- | --- | --- | --- | --- | --- | --- | --- | --- | --- | --- |
| -465.33 | | -663.56 | | -1562.55 | | -529.08 | | -343.69 | | -271.32 | | -507.34 | | -519.04 | |
| -307.25 | |  | -345 | -1416.3 | | -268.62 | | -168.33 | | -122.63 | | -297.15 | | -249.85 | |
|  | -68.37 |  | -57.55 | -514.11 | | -105.12 | | -100.47 | |  | -75.31 |  | -96.82 |  | -75.27 |
| -122.11 | | -298.84 | | -528.36 | |  | -685.5 | -797.07 | | -676.74 | |  | -517.2 | -131.67 | |
|  | -61.29 | -439.58 | | -572.73 | | -258.68 | |  | -260.7 | -189.86 | | -144.64 | | NA |  |
| -145.19 | | -327.72 | | -923.81 | | -650.25 | |  | -658.4 | -553.33 | | -474.17 | | -152.82 | |
|  | -80.02 | -1741.79 | | -2938.79 | | -308.96 | | -249.53 | | -181.21 | | -309.55 | |  | -81.86 |
| -111.86 | |  | -2011 | -3334.51 | | -360.78 | | -310.84 | | -221.23 | | -381.08 | | -119.24 | |
|  | -6.49 | -158.81 | | -1427.71 | | NA |  | NA |  | NA |  | NA |  | NA |  |
| -107.69 | | -2140.32 | | -3535.58 | | -374.18 | | -321.35 | | -230.08 | | -383.95 | | -102.89 | |
|  | -41.44 | -648.79 | | -2282.1 | | -209.15 | | -229.01 | | -174.41 | | -188.72 | |  | -67.21 |
| -193.46 | | NA |  | -186.46 | | NA |  | NA |  | NA |  | NA |  | NA |  |
| -186.85 | | -159.15 | | -576.05 | | -183.85 | | -153.68 | | -137.77 | | -226.77 | | -197.91 | |
| -194.75 | | NA |  | -139.97 | | NA |  | NA |  | NA |  | NA |  | NA |  |
| NA |  | NA |  |  | -62.64 | NA |  | NA |  | NA |  | NA |  | NA |  |
| NA |  | NA |  | NA |  | NA |  | NA |  | NA |  | NA |  | NA |  |
|  | -6.17 |  | -6.99 | -118.03 | |  | -26.41 |  | -45.89 |  | -36.89 |  | -20.69 |  | -11.08 |
| NA |  | NA |  | NA |  | NA |  | NA |  | NA |  | NA |  | NA |  |
| NA |  | NA |  | NA |  | NA |  | NA |  | NA |  | NA |  | NA |  |
| NA |  | NA |  |  | -41.11 | NA |  | NA |  | NA |  | NA |  | NA |  |
| NA |  | NA |  | -138.81 | | NA |  | NA |  | NA |  | NA |  | NA |  |
| NA |  | NA |  |  | -72.39 | NA |  | NA |  | NA |  | NA |  | NA |  |
| NA |  | NA |  |  | -23.83 |  | -37.93 |  | -80.45 |  | -68.7 |  | -35.4 |  | -20.92 |
|  | -32.8 | NA |  |  | -64.26 | -220.34 | | -294.46 | | -290.83 | | -251.85 | | -163.75 | |
| NA |  | NA |  | NA |  | NA |  | NA |  | NA |  | NA |  | NA |  |
| NA |  | NA |  | NA |  | NA |  | NA |  | NA |  | NA |  | NA |  |
| NA |  | NA |  | NA |  | NA |  | NA |  | NA |  | NA |  | NA |  |
| -585.02 | | -259.21 | | -3683.8 | | -473.14 | | -476.47 | | -445.54 | | -578.38 | | -308.61 | |
|  | -99.95 | -356.24 | | -716.87 | | -377.12 | |  | -255 | -328.21 | | -474.32 | | -365.83 | |
|  | -99.95 | -356.24 | | -716.87 | | -377.12 | |  | -255 | -328.21 | | -474.32 | | -365.83 | |
| NA |  |  | -8.35 |  | -39.31 | NA |  | NA |  | NA |  | NA |  | NA |  |
|  | -34.47 |  | -68.68 | -519.15 | | NA |  | NA |  | NA |  | NA |  | NA |  |
| NA |  |  | -34.84 | -427.21 | | NA |  | NA |  | NA |  | NA |  | NA |  |
|  | -26.35 | -106.59 | | -406.24 | |  | -18.34 | NA |  | NA |  |  | -28.62 |  | -9.6 |
| -303.78 | |  | -187.5 | -836.71 | | -953.66 | | -892.59 | | -837.91 | | -937.62 | |  | -774.7 |
|  | -15.02 |  | -90.06 | -609.49 | |  | -16.14 |  | -5.4 | NA |  |  | -12.84 | NA |  |
| NA |  | NA |  | -498.43 | | NA |  | NA |  | NA |  | NA |  | NA |  |
|  | -17.76 | -121.93 | | -664.55 | |  | -10.18 | NA |  | NA |  |  | -12.01 | NA |  |

-6.11	-73.6	-336.68	-31.86	-27.77	-21.29	-17.8	-12.07

NA	-34.55	-27.12 NA	NA	NA	NA	NA NA	-24.21	-44.62 NA	NA	NA	NA	NA NA	-33.93	-109.75 NA	NA	NA	NA	NA

-14.24	-37.36	-375.58 NA	NA	NA	NA	NA

-131	-333.58	-1137.95	-403.58	-397.57	-359.9	-299.89	-250.02

NA	NA	NA	NA	NA	NA	NA	NA NA	NA	NA	NA	NA	NA	NA	NA NA	NA	NA	NA	NA	NA	NA	NA NA	NA	NA	NA	NA	NA	NA	NA

-374.69	-370.39	-316.67	-52.08	-44.21	-40.61	-44.27	-18.4

-323.97	-298.33	-936.09	-42.08	-26.77	-19.78	-28.74 NA

-246.07	-242.29	-932.4	-383.54	-360.77	-333.12	-269.02	-112.97

NA	NA	NA	NA	NA	NA	NA	NA

-433.02	-302.3	-847.37 NA	NA	NA	-6.1 NA

-154.91	-196.76	-180.88	-42.2	-65	-62.47	-27.54	-5.79

-68.32	-149.78	-858.52	-49.28	-42.14	-24.03	-29.97	-9.85

-178	-219.96	-907.08 NA	NA	NA	-13.77 NA NA	NA	NA	NA	NA	NA	NA	NA

-165.47	-99.24	-935.99	-106.37 NA	NA	-161.79	-197.32

-201.12	-277.51	-989.84	-261.61	-123.39	-109.64	-347.22	-363.04

-30.81	-129.14	-483.1	-77.2	-55.54	-60.55	-99.56	-63.28

-17.8	-179.58	-477.34	-73.29	-39.93	-47.48	-77	-40.99

-255.77	-219.15	-1844.82	-758.13	-652.77	-705.74	-709.59	-429.77

-134.73	-151.17	-770.56	-227.41	-193.73	-185.92	-250.71	-180.92

-44.71	-56.36	-371.3	-30.58 NA	NA	-29.77	-9.24

-257.57	-158.57	-577.71	-168.55	-175.12	-153.75	-219.36	-196.93

-116.87	-121.97	-735.14	-55.21	-33	-26.71	-57.26	-50.32

-142.76	-188.64	-1091.94	-355.51	-348.83	-293.83	-333.64	-174.25

-51.21	-69.9	-698.58	-28.85	-15.57	-10.15	-37.99	-15.85

-20.23	-93.66	-295.6	-87.64	-90.44	-88.64	-70.76	-40.95

-49.61	-92.69	-610.32	-62.19	-62.88	-41.21	-64.87	-44.1

-50.71	-123.31	-472.43	-125.92	-134.82	-121.13	-108.99	-98.52

-105.26	-169.36	-926.77	-133.16	-92.09	-78.69	-144.39	-113.17

-13.71	-87.89	-278.03	-69.76	-89.18	-66.57	-65.69	-36.74

-8.93	-19.15	-105.32	-7.3	-9.19	-7.74	-6.72 NA NA	NA	-537.66 NA	NA	NA	NA	NA NA	NA	-537.66 NA	NA	NA	NA	NA NA	NA	-537.66 NA	NA	NA	NA	NA

-415 NA	-2248.1	-1538.94	-1910.28	-1764.04	-1426.17	-962.29

-477.04	-20.39	-2194.05	-1945.59	-2362.66	-2116.01	-1824.45	-1258.41

-282.05	-30.27	-1292.53	-879.53	-1006.61	-945.01	-884.62	-682.82

-22	-155.58	-386.17	-38.12	-13.14	-16.12	-41.44	-16.58

-14.55	-37.42	-606.8 NA	NA	NA	NA	NA NA	-61.2	-74.09 NA	NA	NA	NA	NA NA	-61.55	-101.61 NA	NA	NA	NA	NA

NA	-73.33	-281.81	-48.02	-74.69	-60.66	-21.51	-7.88

-104.42	-136.96	-1154.38	-188.48	-79.98	-74.44	-178.41	-134.9

-124.96	-182.19	-924.01	-114.96	-47.46	-37.64	-121.69	-95.44

-80.45	-274.29	-1226.96	-170.83	-106.77	-92.88	-155.3	-81.73

-80.45	-274.29	-1226.96	-170.83	-106.77	-92.88	-155.3	-81.73

-88.44	-225.53	-1221.68	-208.81	-142.11	-131.91	-169.33	-106.3

-160.43	-347.38	-684.78	-306.52	-247.83	-193.71	-172.59 NA

-160.43	-347.38	-684.78	-306.52	-247.83	-193.71	-172.59 NA NA	NA	NA	-13.79	-38.82	-35.7 NA	NA NA	NA	NA	NA	-16.91	-28.23 NA	NA NA	NA	NA	NA	-18.7	-19.15 NA	NA

-44.94	-210.03	-308.75	-68.79	-7.63	-17.91	-202.02	-122.18

-542.25	-252.94	-1077.26	-2966.69	-3580.16	-3376.13	-2780.23	-2301.48

-877.54 NA	-47.06	-7125.2	-9236.79	-10062.3	-6143.66	-4061.17

-289.08 NA	-31.72	-3294.01	-4122.56	-4967.1	-3027.48	-1704.31

-549.25	-161.23	-316.21	-4373.93	-5325.03	-5642.57	-4027.73	-2999.49

-1208.84	-30.48	-100.35	-10208.8	-13105.3	-14048.3	-8915.52	-5548.38

-684.78	-16.4	-111.81	-6737.84	-8762.72	-9540.87	-6068.77	-4124.11

-16.18	-146.13	-552.65	-261.62	-406.41	-358.57	-189.9	-164.81

-157.84 NA	-77.01	-1136.92	-1855.93	-2099.91	-769.54	-254.58

-35.91	-236.07	-779.39	-236.44	-328.26	-272.47	-216.09	-171.77

-48.81	-14.17	-407.44	-54.27	-43.17	-30.99	-33.37	-30.08

-515.56	-442.82	-1393.33	-396.88	-229.96	-189.56	-371.17	-403.42

-202.47	-346.71	-767.62	-467.46	-453.73	-400.32	-386.26	-365.81

-279.93 NA	-384.39 NA	NA	NA	NA	-7.74

NA	NA	NA	NA	-10.89	-21.82 NA	NA NA	-254.93	-577.85 NA	NA	NA	NA	NA NA	-215.53	-230.18 NA	NA	NA	NA	NA

-174.12	-170.71	-1407.31	-31.63 NA	NA	-52.58	-59.24

-240.4	-462.53	-2015.58	-198.17	-60.02	-46.63	-231.2	-229.81

-229.87	-305.11	-1431.44	-125.78	-17.95	-7.04	-188.8	-189.41

-30.95 NA	-360.02 NA	NA	NA	NA	NA

| -247.6 | -417.85 | -1892.4 | -154.9 | -30.66 | -20.34 | -201.04 | -191.02 |
| --- | --- | --- | --- | --- | --- | --- | --- |
| -312.61 | -669.86 | -1904.78 | -547.18 | -340.96 | -278.24 | -477.49 | -461.59 |
| -379.61 | -119.28 | -2264 | -242.57 | -217.95 | -184.95 | -256.64 | -95.28 |
| -341.66 | -98.35 | -2086.95 | -227.77 | -192.67 | -181.89 | -233.18 | -79.36 |
| -218.77 | -54.6 | -1415.33 | -134.96 | -102.28 | -90.32 | -131.53 | -31.77 |
| -58.95 | -74.95 | -249.81 | -63.87 | -45.67 | -33.81 | -50.11 | -40.44 |

-31.52	-342.65	-1164.13	-19.5 NA	NA	-18.46 NA

-378.49	-123.54	-2078.66	-236.92	-190.75	-166.75	-257.68	-93.63

-289.52	-61.9	-1807.91	-118.15	-77.24	-59.5	-131.48	-37.73

NA	NA	NA	NA	NA	NA	NA	NA NA	-18.28 NA	NA	NA	NA	NA	NA NA	NA	NA	NA	NA	NA	NA	NA

-434.22	-415.1	-2471.52	-758.73	-591.2	-606.08	-775.15	-605.23

-283.69	-208.81	-2408.17	-282.79	-170.05	-163.13	-285.98	-228.45

-68.85 NA	-459.64	-9.36 NA	NA	-9.6	-15.66

-340.77	-119.96	-2315.02	-118.34	-60.52	-57.54	-161.61	-156.89

-57.45 NA	-377.81	-8.7 NA	NA	-7.08	-8.12

-126.29 NA	-867.04 NA	NA	NA	-29.22	-22.2

-102.38	-125.79	-901.78	-66.22	-6.07 NA	-89.06	-92.36

NA	NA	NA	NA	NA	NA	NA	NA

-332.9	-483.06	-3520.43	-518.77	-433.07	-421.38	-491.37	-372.94

-24.35	-31.64	-1001.58	-40.54	-23.81	-26.13	-30.38	-6.49

-67.85	-8.43	-1265.66 NA	NA	NA	NA	NA

-109.86 NA	-1408.33 NA	NA	NA	NA	NA

| -182.78 | -147.2 | -907.12 | -108.37 | -25.76 | -13.67 | -194.46 | -161.34 |
| --- | --- | --- | --- | --- | --- | --- | --- |
| -82.76 | -222.5 | -510.4 | -201.03 | -101.88 | -113.2 | -387.54 | -283.82 |
| -118.25 | -215.2 | -483.46 | -194.28 | -108.04 | -92.34 | -262.82 | -258.3 |
| -149.01 | -122.41 | -1122.22 | -106.51 | -35.51 | -30.32 | -143.52 | -146.55 |
| -64.81 | -58.95 | -756.2 | -124.97 | -31.36 | -55.45 | -157.79 | -101.24 |
| -235.19 | -112.19 | -2452.44 | -295.09 | -244.55 | -252.07 | -284.84 | -206.95 |
| -157.34 | -143.7 | -2125.01 | -434.81 | -259.8 | -338.86 | -507.24 | -327.73 |
| -283.84 | -310 | -1657.18 | -368.5 | -301.98 | -269.55 | -324.23 | -277.52 |
| -100.12 | -95.88 | -890.61 | -167.48 | -149.17 | -131.84 | -177.04 | -130.08 |
| -96.94 | -91.17 | -1174.25 | -170.06 | -165.22 | -158.16 | -165.47 | -134.93 |
| -137.94 | -50.44 | -775.25 | -97.06 | -25.03 | -44.11 | -125.84 | -55.44 |
| -34.6 | -115.84 | -270.38 | -452.48 | -639.06 | -673.92 | -409.42 | -370.44 |
| -91.21 | -127.38 | -233.19 | -1008.62 | -1542.78 | -1521.17 | -925 | -710.69 |
| -118.41 | -130 | -965.62 | -153.1 | -60.66 | -56.74 | -139.91 | -108.34 |
| -166.69 | -239.94 | -1103.97 | -272.07 | -230.81 | -257.69 | -222.41 | -201.64 |

| -95.57 | -107.2 | -781.09 | -180.41 | -131.82 | -120.88 | -196.62 | -139.78 |
| --- | --- | --- | --- | --- | --- | --- | --- |
| -156.55 | -258.08 | -924.84 | -393.82 | -324.48 | -285.71 | -440.95 | -402.58 |
| -192.48 | -206.39 | -2944.99 | -377.28 | -389.15 | -376.85 | -325.41 | -221.69 |
| -69.63 | -23.1 | -804.12 | -48.38 | -26.21 | -24.62 | -62.71 | -67.29 |
| -156.62 | -270.82 | -4851.95 | -1226.9 | -1536.07 | -1849.52 | -1181.62 | -545.39 |

NA	NA	-3732.8 NA	-73.18	-50.77 NA	NA

-209.49	-384.89	-1015.04	-538.34	-468.06	-418.99	-474.06	-428.04

-108.13 NA	-935.62	-112.03	-51.34	-35.48	-102.28	-88.64

-160.35	-189.71	-2006.95	-191.42	-94.39	-85.94	-197.39	-188.98

-54.71	-41.21	-566.61	-115.29	-127.51	-104.62	-131	-97.43

-110.07	-215.47	-1253.64	-242.52	-113.11	-114.37	-260.44	-189.11

NA	NA	NA	NA	NA	NA	NA	NA

-91.48	-20.94	-825.74	-227.65	-196.16	-192.53	-221.31	-188.07

-115.58	-117.09	-599.6	-69.75	-20.26	-30.17	-153.02	-146.42

-250.38	-128.8	-1375.72	-176.78	-72.27	-93.6	-325.06	-210.03

-370.61	-739.01	-2045.17	-630.45	-462.9	-402.1	-584.97	-578.01

-89.46	-75	-745.87	-19.32 NA	NA	-29.84	-24.68

-231.22	-237.7	-1442.19	-187.56	-71.13	-47.62	-164.84	-208.3

-17 NA	-583.86 NA	-12.45	-6.97 NA	NA

-46.89	-23.85	-680.42	-153.65	-116.1	-93.63	-155.96	-99.45

-177.74	-124.55	-1455.72	-359.6	-260.19	-241.43	-386.15	-244.91

-5.7 NA	-26.93	-371.23	-459.2	-700.36	-263.56	-114.84

-130.4	-154.78	-1685.06	-150.7	-58.3	-40.93	-145.8	-118.76

-40.49	-43.18	-208.16	-587.38	-594.89	-756.75	-687.06	-555.32

-150.96	-232.64	-1448.75	-139.43	-46.65	-38.35	-168.95	-135.93

-75.29	-18.05	-475.98	-534.5	-630.11	-710.17	-425	-314.63

-64.81	-158.66	-969.67	-218.35	-234.12	-212.28	-245.91	-146.99

-91.45	-301.44	-575.57	-184.18	-90.08	-95.91	-327.48	-320.82

-52.5	-86.9	-568.46	-103.58	-100.57	-78.17	-77.81	-70.8

-22.18	-27.12	-514.26	-63.66	-71.99	-55.85	-73.5	-35.97

-71.03	-80.95	-249.78	-595.7	-649.19	-728.12	-614.77	-424.38

-318.12	-304.62	-3901.6	-304.92	-170.95	-190.65	-361.53	-308.4

-111.81	-206.76	-1045.71	-166.2	-68.74	-65	-238.03	-216.44

-43.73	-6.77	-1242.57	-215.47	-314.96	-285.16	-221.91	-165.71

-92.21 NA	-915.46	-11.7 NA	NA	-15.83	-5.47

-131.16	-152.03	-1075.56	-129.84	-67.49	-60.18	-102.45	-67.3

-61.9	-29.72	-525.75	-197.5	-191.94	-187.85	-156.72	-122.03

-114.97	-203.8	-856.02	-370.79	-207.63	-307.55	-461.62	-327.31

multipotenmuscle..ce myeloid_c naive_b_c naive_cd4 natural_kil neural_pro neurosphe

-185.07	-272.9	-158.13	-238.1	-146.91	-319.3	-33.11	-198.47

NA	NA	NA	NA	NA	NA	NA	-928.58

-234.64	-648.8	-200	-254.16	-105.53	-442.67 NA	-120.44

-151.2	-196.33	-181.25	-293.47	-355.83	-325.67	-14.48	-208.54

-18.61	-140.85	-55.67	-64.31	-30.47	-118.08 NA	NA

-658.53	-6513.38	-747.5	-1165.24	-231.95	-518.93	-5.7	-901.54

-188.2	-586.21	-221.97	-239.57	-100.13	-447.79 NA	-21.36

-37.58	-110.23	-44.35	-62.74	-70.7	-191.68 NA	-7.09

-37.58	-110.23	-44.35	-62.74	-70.7	-191.68 NA	-7.09

-6.75	-258.9	-30.56	-5.96 NA	-11.56 NA	-52.68

-423.54	-5032.41	-493.95	-608.47	-75.94	-265.22 NA	-634.6

-132.9	-2631.46	-176.91	-361.31	-43.5	-221.53 NA	-117.24

-80.65	-267.08	-342.44	-92.75 NA	-99.13 NA	NA

-100.93	-544.7	-330.47	-116.37	-27.09	-141.4 NA	-35.32

NA	NA	NA	NA	NA	NA	NA	-24.05

NA	NA	NA	NA	NA	NA	NA	-905.98

NA	-30.65	-24.21 NA	NA	NA	NA	-27.89

NA	-30.65	-24.21 NA	NA	NA	NA	-27.89

-1823.93	-150.18	-1528.69	-1749.78	-276.64	-1139.59 NA	-53.83

-174.69	-525.32	-195.55	-295.15	-123.45	-359.55 NA	-82.72

NA	NA	NA	NA	NA	NA	NA	-933.44

-1369.06	-213.6	-1776.91	-1449.84	-470.19	-2434.4 NA	-72.8

NA	NA	NA	NA	NA	NA	NA	NA NA	NA	NA	NA	NA	NA	NA	NA NA	-7.5	-70.74 NA	NA	NA	NA	NA NA	NA	-136.67 NA	NA	NA	NA	NA NA	NA	-147 NA	NA	NA	NA	NA NA	NA	-83.55 NA	NA	NA	NA	NA

-231.92	-856.24	-229.03	-258.13	-84.41	-457.21 NA	-203.24

-231.92	-856.24	-229.03	-258.13	-84.41	-457.21 NA	-203.24

-348.45	-1126.68	-370.18	-405.57	-135.48	-550.56 NA	-307.89

-387.86	-1165.35	-379.61	-420.01	-131.32	-579.13 NA	-278.14

-8.1	-274.62	-32.3 NA	NA	-5.23 NA	-25.62

NA	NA	-21.05 NA	NA	NA	NA	NA

NA	NA	NA	NA	NA	NA	NA	-922.06

NA	NA	NA	NA	NA	NA	NA	-928.33

NA	NA	NA	NA	NA	NA	NA	-1074.14

-212.71	-76.42	-122.78	-163.55	-249.82	-239.57	-74.56	-138.13

-63.93	-899.22	-58.16	-639.93 NA	-38.35 NA	-99.6

-63.93	-899.22	-58.16	-639.93 NA	-38.35 NA	-99.6

-63.93	-899.22	-58.16	-639.93 NA	-38.35 NA	-99.6

-166.65	-19.38	-114.52	-122.81	-195.07	-406.99	-29.96	-109.6

-130.89	-30.11	-101.76	-109.25	-173.27	-271.22	-36.11	-120.72

-3958.17	-322.07	-2775.41	-2651.8	-870.8	-2797.63 NA	-33.5

-4650.3	-787.33	-3688.18	-3479.28	-1160.66	-3280.18 NA	-146.63

-6650.31	-937.23	-5370.49	-5211.3	-1815.04	-4862.48 NA	-195.38

-5440.02	-904.5	-3625.67	-3657.8	-1153.28	-3690.77 NA	-274.55

-2947.8	-534.25	-2483.6	-2332.23	-859.59	-2086.51 NA	-82.13

-3857.05	-494.35	-3424.62	-3176.6	-1164.16	-2661.2	-9.88	-60.27

-5913.01	-646.87	-4826.8	-4600.68	-1241.59	-3717.98 NA	-82.13

-4097.78	-1315.88	-3141.66	-3234.94	-1571.09	-3095.92	-10.25	-326.12

-4847.71	-1306.22	-3878.83	-3954.27	-1678.33	-3973.88 NA	-274.33

-5869.79	-1480.34	-4453.75	-4620.22	-1954.71	-4535.89	-9.29	-373.11

NA	NA	NA	NA	NA	NA	NA	-1082.46

-56.19	-490.59	-99.6	-80.05	-48.11	-301.32 NA	-66.49

-6027.87	-1235.6	-4725.94	-4793.07	-1952.1	-4801.8 NA	-299.06

-6870.3	-1432.7	-5276.01	-5435.9	-2198.39	-5452.04 NA	-344.69

-510.46	-285.44	-392.19	-381.56	-180.3	-307.21 NA	-63.41

-8662.09	-1149.02	-6626.22	-6697.57	-2222.87	-6585.15 NA	-207.89

NA	-59.8 NA	NA	NA	NA	NA	NA

NA	NA	NA	NA	NA	NA	NA	-1008.29

-7577.04	-1333.25	-5817.78	-6010.8	-2088.15	-5969.14 NA	-250.35

-327.31	-158.82	-195.62	-212.32	-130.04	-209.37 NA	-55.55

-3779.82	-447.69	-2940.36	-2677.03	-906.95	-2618.96 NA	-97.62

-7116.42	-1450.29	-5595.16	-5673.28	-2303.61	-5640 NA	-374.59

-5309.03	-1017.54	-4137.34	-4207.68	-1648.2	-4151.35 NA	-227.12

-2136.44	-757.51	-1751.59	-1728.14	-926.51	-1692.71 NA	-168.63

-2136.44	-757.51	-1751.59	-1728.14	-926.51	-1692.71 NA	-168.63

-7922.55	-1236.45	-6273.05	-6249.64	-2301.17	-6106.46 NA	-273.64

-3389.8	-820.01	-2657.77	-2549.09	-1110.9	-2781.57 NA	-218.11

-2174.11	-52.15	-1860.09	-1639.78	-588.18	-1341.77 NA	NA

-2414.63	-448.34	-1903	-1887.09	-614.26	-1981.68 NA	-71.22

NA	NA	NA	NA	NA	NA	NA	-1242.51

-140.75	-2735.06	-188.24	-353.79	-44.26	-251.82 NA	-94.88

-7270.64	-1100.43	-5457.64	-5513.83	-1886.2	-5552.05 NA	-175.26

-8613	-1362.97	-6701.46	-6743.45	-2435.38	-6652.47 NA	-295.74

-559.48	-324.09	-459.41	-429.39	-215.06	-350.84	-6.78	-71.89

-50.57	-456.34	-295.27	-50.81 NA	-57.68 NA	-46.19

-97.85	-430.16	-277.91	-69.02 NA	-93.25 NA	-130.83

-142.15	-594.39	-391.13	-136.46	-13.57	-161.85 NA	-86.19

NA	-194.49	-172.14	-5.3 NA	-23.53 NA	NA NA	NA	NA	NA	NA	NA	NA	NA NA	NA	NA	NA	NA	NA	NA	NA NA	NA	NA	NA	NA	NA	NA	NA

NA	NA	NA	NA	NA	NA	NA	-8.45

NA	-16.24 NA	NA	NA	NA	NA	-51.94

NA	-9.38 NA	NA	NA	NA	NA	-53.76

NA	NA	NA	NA	NA	NA	NA	NA NA	NA	NA	NA	NA	NA	NA	NA NA	NA	NA	NA	NA	NA	NA	NA NA	NA	NA	NA	NA	NA	NA	NA

NA	-18.62 NA	NA	NA	NA	NA	-49.03

-10.64	-50.11 NA	-5.54 NA	NA	NA	-86.14

NA	NA	NA	NA	NA	NA	NA	-53.9

NA	NA	NA	NA	NA	NA	NA	-47.14

-47.1	-57.04 NA	-35.38 NA	NA	NA	-110.37

-10.86	-22.37 NA	NA	NA	NA	NA	-69.79

NA	NA	NA	NA	NA	NA	NA	NA

-6284.59	-1341.43	-5040.77	-5089.34	-2159.02	-5034.36	-14.32	-330.69

-199.19 NA	-79.83	-50.89 NA	-93.03 NA	-6.48

-199.19 NA	-79.83	-50.89 NA	-93.03 NA	-6.48

-5.62 NA	NA	NA	NA	NA	NA	NA

-5.62 NA	NA	NA	NA	NA	NA	NA

-65.81 NA	NA	NA	NA	NA	NA	NA

-303.76 NA	-40.4	-21.12 NA	-65.88 NA	NA NA	NA	NA	NA	NA	NA	NA	NA

-6.11 NA	NA	NA	NA	NA	NA	NA

-6.11 NA	NA	NA	NA	NA	NA	NA

-6.11 NA	NA	NA	NA	NA	NA	NA

NA	NA	NA	NA	NA	NA	NA	-978.6

NA	NA	NA	NA	NA	NA	NA	-1042.81

NA	NA	NA	NA	NA	NA	NA	-1328.13

NA	NA	NA	NA	NA	NA	NA	-1343.11

-87.71	-1009.05	-87.28	-40.55	-10.8	-67.9 NA	-268.18

-57.49	-376.61	-145.41	-141.99	-81.59	-244.68	-12.43	-181.54

-57.49	-376.61	-145.41	-141.99	-81.59	-244.68	-12.43	-181.54

| -102.82 | | -592.03 | | -210.68 | | -244.36 | | -249.41 | | -333.26 | |  | -43.85 | -229.58 | |
| --- | --- | --- | --- | --- | --- | --- | --- | --- | --- | --- | --- | --- | --- | --- | --- |
| NA |  |  | -72.04 | NA |  | NA |  |  | -7.58 |  | -8.11 | NA |  | NA |  |
| -176.98 | | -484.51 | | -194.98 | | -274.56 | | -125.71 | | -306.33 | |  | -35.2 |  | -144.9 |
|  | -15.08 | -343.52 | |  | -51.37 |  | -48.97 |  | -15.06 |  | -52.47 |  | -5.56 | -340.08 | |
| -306.47 | |  | -478.8 | -308.04 | | -288.66 | | -111.23 | | -303.57 | | NA |  | -316.49 | |
| NA |  | NA |  |  | -18.09 | NA |  | NA |  | NA |  | NA |  | NA |  |
| NA |  | NA |  | NA |  | NA |  | NA |  | NA |  | NA |  | NA |  |
| NA |  | NA |  | NA |  | NA |  | NA |  | NA |  | NA |  | NA |  |
| NA |  | -305.29 | |  | -12.87 | NA |  | NA |  |  | -6.08 | NA |  |  | -29.78 |
| NA |  | -256.06 | |  | -5.78 | NA |  | NA |  |  | -8.67 | NA |  | NA |  |
|  | -55.25 | -607.53 | |  | -74.31 |  | -35.2 | NA |  |  | -39.16 | NA |  | -114.05 | |
| NA |  | NA |  | NA |  | NA |  | NA |  | NA |  | NA |  | -1335.55 | |
| NA |  | NA |  | NA |  | NA |  | NA |  | NA |  | NA |  |  | -7.47 |
| NA |  | NA |  | NA |  | NA |  | NA |  | NA |  | NA |  | NA |  |
| NA |  | NA |  | NA |  | NA |  | NA |  | NA |  | NA |  | NA |  |
| NA |  | NA |  | NA |  | NA |  | NA |  | NA |  | NA |  | NA |  |
| NA |  | NA |  | NA |  | NA |  | NA |  | NA |  | NA |  | -1293.64 | |
| NA |  | NA |  | NA |  | NA |  | NA |  | NA |  | NA |  | -887.69 | |
| NA |  | NA |  | NA |  | NA |  | NA |  | NA |  | NA |  | -918.68 | |
|  | -79.61 |  | -113.5 | NA |  | NA |  | NA |  | NA |  | NA |  |  | -11.07 |
| NA |  | NA |  | NA |  | NA |  | NA |  | NA |  | NA |  |  | -56.81 |
| NA |  |  | -6.36 | NA |  | NA |  | NA |  | NA |  | NA |  | -1169.94 | |
| NA |  | NA |  | NA |  | NA |  | NA |  | NA |  | NA |  | -1375.93 | |
| NA |  | NA |  | NA |  | NA |  | NA |  | NA |  | NA |  | -1620.53 | |
| NA |  | NA |  | NA |  | NA |  | NA |  | NA |  | NA |  | NA |  |
|  | -58.86 | -147.94 | | NA |  | NA |  | NA |  | NA |  | NA |  |  | -32.16 |
| NA |  | NA |  | NA |  | NA |  | NA |  | NA |  | NA |  |  | -17.83 |
| NA |  | NA |  | NA |  | NA |  | NA |  | NA |  | NA |  |  | -74.42 |
| NA |  | NA |  | NA |  | NA |  | NA |  | NA |  | NA |  | -1253.91 | |
| NA |  | NA |  | NA |  | NA |  | NA |  | NA |  | NA |  | -1259.8 | |
|  | -34.75 | -139.43 | | NA |  | NA |  | NA |  | NA |  | NA |  | -301.66 | |
| NA |  | NA |  | NA |  | NA |  | NA |  | NA |  | NA |  | -1388.19 | |
| NA |  | NA |  | NA |  | NA |  | NA |  | NA |  | NA |  |  | -68.64 |
| NA |  | NA |  | NA |  | NA |  | NA |  | NA |  | NA |  |  | -67.43 |
| NA |  | NA |  | NA |  | NA |  | NA |  | NA |  | NA |  |  | -23.83 |
| NA |  |  | -12.65 | NA |  | NA |  | NA |  | NA |  | NA |  |  | -66.18 |
| NA |  | NA |  | NA |  | NA |  | NA |  | NA |  | NA |  | NA |  |
| NA |  |  | -25.5 | NA |  | NA |  | NA |  | NA |  | NA |  |  | -12.37 |
| NA |  | NA |  | NA |  | NA |  | NA |  | NA |  | NA |  |  | -45.91 |

| NA |  | NA |  | NA |  | NA |  | NA |  | NA |  | NA |  | -1541.02 | |
| --- | --- | --- | --- | --- | --- | --- | --- | --- | --- | --- | --- | --- | --- | --- | --- |
| -116.14 | | -148.57 | | NA |  |  | -12.49 | NA |  | NA |  | NA |  |  | -14.2 |
| NA |  | NA |  | NA |  | NA |  | NA |  | NA |  | NA |  | -1314.92 | |
| NA |  | NA |  | NA |  | NA |  | NA |  | NA |  | NA |  | NA |  |
| NA |  | NA |  | NA |  | NA |  | NA |  | NA |  | NA |  | NA |  |
|  | -6.25 |  | -28.65 | NA |  | NA |  | NA |  | NA |  | NA |  |  | -26.52 |
| NA |  | NA |  | NA |  | NA |  | NA |  | NA |  | NA |  |  | -14.99 |
| NA |  | NA |  | NA |  | NA |  | NA |  | NA |  | NA |  |  | -45.19 |
| NA |  | NA |  | NA |  | NA |  | NA |  | NA |  | NA |  | -1413.56 | |
| NA |  |  | -45.4 | NA |  | NA |  | NA |  | NA |  | NA |  |  | -41.43 |
| -1240.49 | | -147.09 | | -866.07 | | -800.54 | | -126.26 | | -578.77 | | NA |  |  | -31.64 |
|  | -8.8 | -963.87 | |  | -21.26 |  | -10.07 | NA |  |  | -21.9 | NA |  | -448.87 | |
|  | -13.02 | NA |  | NA |  |  | -44.4 | NA |  | NA |  | NA |  | NA |  |
| -353.98 | |  | -53.62 | -199.33 | |  | -451.3 |  | -31.37 | -232.17 | | NA |  |  | -31.58 |
| -162.32 | | -152.33 | | -151.79 | |  | -357 |  | -99.8 | -232.75 | | NA |  |  | -12.37 |
| -105.81 | |  | -94.6 | -118.99 | | -259.22 | |  | -46.36 | -196.03 | | NA |  |  | -13.34 |
| NA |  | NA |  |  | -12.03 |  | -47.67 |  | -13.76 |  | -14.8 | NA |  | NA |  |
|  | -34.89 |  | -32.86 |  | -8.05 |  | -48.24 | NA |  |  | -8.29 | NA |  |  | -10.15 |
| -309.12 | | -167.83 | | -251.93 | | -612.99 | | -123.74 | | -310.41 | | NA |  |  | -30.6 |
|  | -42.84 | NA |  |  | -27.98 | -127.21 | |  | -7.29 |  | -64.97 | NA |  | NA |  |
|  | -12.6 | -385.77 | | -173.99 | |  | -15.75 | NA |  |  | -20.03 | NA |  |  | -35.71 |
|  | -25.14 | -309.22 | | -215.18 | |  | -45.88 | NA |  |  | -38.26 | NA |  |  | -40.19 |
|  | -60.87 | -373.28 | | -306.69 | |  | -64.71 | NA |  |  | -88.79 | NA |  |  | -54.51 |
| NA |  | -139.27 | |  | -96.24 | NA |  | NA |  | NA |  | NA |  | NA |  |
|  | -26.34 | NA |  |  | -10.24 |  | -12.27 |  | -23.99 | -233.22 | | NA |  | NA |  |
|  | -59.19 | -208.91 | | -108.51 | |  | -90.54 | NA |  | -155.91 | | NA |  | -169.88 | |
|  | -33.41 |  | -10.73 |  | -41.82 |  | -38.92 |  | -37.3 | -210.05 | |  | -17.8 | NA |  |
|  | -31.7 |  | -12.08 |  | -36.75 |  | -30.72 |  | -46.07 |  | -84.53 |  | -5.96 |  | -40.71 |
|  | -31.7 |  | -12.08 |  | -36.75 |  | -30.72 |  | -46.07 |  | -84.53 |  | -5.96 |  | -40.71 |
|  | -26.5 | NA |  | NA |  |  | -17.93 |  | -74.97 | -179.38 | |  | -23.63 | NA |  |
| -153.42 | | NA |  |  | -74.84 |  | -95.25 | -107.48 | |  | -347 |  | -12.93 |  | -44.87 |
|  | -23.11 | NA |  |  | -5.61 |  | -21.91 |  | -45.29 | -252.34 | | NA |  | NA |  |
|  | -23.11 | NA |  |  | -5.61 |  | -21.91 |  | -45.29 | -252.34 | | NA |  | NA |  |
|  | -23.11 | NA |  |  | -5.61 |  | -21.91 |  | -45.29 | -252.34 | | NA |  | NA |  |
| -209.22 | |  | -8.91 |  | -97.93 | -110.79 | | -109.14 | | -374.94 | |  | -13.85 |  | -37.66 |
| -312.41 | | -155.24 | | -228.64 | | -258.91 | | -220.14 | | -393.35 | |  | -58.62 | -432.79 | |
| NA |  | NA |  | NA |  |  | -6.67 |  | -29.7 | -137.18 | |  | -6.13 | NA |  |
| NA |  | NA |  | NA |  | NA |  | NA |  | NA |  | NA |  | -1748.49 | |
|  | -6.22 | NA |  |  | -5.13 |  | -11.7 |  | -25 |  | -13 | NA |  | -224.33 | |

| NA |  | NA |  | NA |  | NA |  | NA |  | NA |  | NA |  | -1736.59 | |
| --- | --- | --- | --- | --- | --- | --- | --- | --- | --- | --- | --- | --- | --- | --- | --- |
| NA |  | NA |  | NA |  | NA |  | NA |  | NA |  | NA |  | -1025.86 | |
| NA |  | NA |  | NA |  | NA |  | NA |  | NA |  | NA |  | -1189.24 | |
| NA |  | NA |  | NA |  | NA |  | NA |  | NA |  | NA |  | -1026.98 | |
|  | -269.2 | -4124.37 | | -471.26 | | -690.64 | | -135.55 | | -301.55 | | NA |  | -503.51 | |
| -233.14 | | -972.25 | | -313.29 | | -229.34 | |  | -75.79 | -263.36 | | NA |  | -351.57 | |
|  | -83.85 | -491.76 | | -153.98 | | -107.62 | |  | -24.16 |  | -168.6 | NA |  | -121.84 | |
| -415.89 | | -1596.78 | |  | -456.8 | -444.73 | | -145.36 | | -419.15 | | NA |  | -690.26 | |
|  | -28.71 | -109.74 | | -138.67 | |  | -73.06 |  | -12.96 |  | -65.13 | NA |  |  | -10.47 |
|  | -84.31 | -243.31 | | -196.87 | | -141.25 | |  | -31.62 | -109.28 | | NA |  | -102.55 | |
|  | -213.6 | -628.67 | | -249.29 | | -291.22 | |  | -98.67 | -465.23 | | NA |  |  | -73.53 |
| -222.15 | | -589.62 | | -215.38 | | -209.99 | |  | -533.9 | -223.97 | |  | -95.58 | -410.78 | |
| -366.95 | | -963.27 | | -342.04 | | -409.53 | |  | -832.9 | -330.41 | |  | -71.01 | -703.27 | |
|  | -47.53 | -1092.07 | |  | -48.69 | -102.02 | | NA |  |  | -94.43 | NA |  |  | -92.9 |
| NA |  | NA |  | NA |  | NA |  | NA |  | NA |  | NA |  |  | -2305 |
| NA |  | NA |  | NA |  | NA |  | NA |  | NA |  | NA |  | -1367.16 | |
| -113.97 | | -406.94 | | -125.06 | | -187.49 | |  | -53.47 | -281.91 | | NA |  | NA |  |
| NA |  | NA |  | NA |  | NA |  | NA |  | NA |  | NA |  | -1374.4 | |
| -302.91 | | -4382.5 | | -327.61 | | -574.09 | |  | -96.53 | -281.39 | | NA |  | -480.11 | |
| NA |  | NA |  | NA |  | NA |  | NA |  | NA |  | NA |  | -1338.89 | |
| NA |  | NA |  | NA |  | NA |  | NA |  | NA |  | NA |  | -1338.89 | |
| -156.24 | | -332.76 | |  | -128.2 | -193.78 | |  | -120.8 | -218.65 | |  | -19.75 | -258.17 | |
| -214.37 | | -793.29 | | -246.49 | | -248.16 | |  | -76.5 | -435.02 | | NA |  | -124.63 | |
| -214.37 | | -793.29 | | -246.49 | | -248.16 | |  | -76.5 | -435.02 | | NA |  | -124.63 | |
| -214.37 | | -793.29 | | -246.49 | | -248.16 | |  | -76.5 | -435.02 | | NA |  | -124.63 | |
| -214.37 | | -793.29 | | -246.49 | | -248.16 | |  | -76.5 | -435.02 | | NA |  | -124.63 | |
| -479.09 | |  | -936.3 | -412.13 | | -356.56 | | -141.98 | | -426.49 | | NA |  | -399.59 | |
| -479.09 | |  | -936.3 | -412.13 | | -356.56 | | -141.98 | | -426.49 | | NA |  | -399.59 | |
| -479.09 | |  | -936.3 | -412.13 | | -356.56 | | -141.98 | | -426.49 | | NA |  | -399.59 | |
| -479.09 | |  | -936.3 | -412.13 | | -356.56 | | -141.98 | | -426.49 | | NA |  | -399.59 | |
| -211.48 | | -499.47 | | -167.01 | | -188.78 | |  | -53.31 | -192.51 | | NA |  | -378.06 | |
| -211.48 | | -499.47 | | -167.01 | | -188.78 | |  | -53.31 | -192.51 | | NA |  | -378.06 | |
| -148.93 | | -336.18 | | -116.34 | | -141.11 | |  | -38.77 | -139.39 | | NA |  | -185.82 | |
| -148.93 | | -336.18 | | -116.34 | | -141.11 | |  | -38.77 | -139.39 | | NA |  | -185.82 | |
| -402.57 | | -1010.44 | | -456.95 | | -449.57 | |  | -243.5 | -761.94 | | NA |  | -247.09 | |
| -315.78 | | -6194.67 | | -416.43 | | -783.34 | | -126.41 | | -294.01 | | NA |  | -398.07 | |
| -315.78 | | -6194.67 | | -416.43 | | -783.34 | | -126.41 | | -294.01 | | NA |  | -398.07 | |
| -323.49 | | -4201.6 | | -350.89 | | -620.79 | |  | -89.62 | -268.88 | | NA |  | -409.58 | |
| -323.49 | | -4201.6 | | -350.89 | | -620.79 | |  | -89.62 | -268.88 | | NA |  | -409.58 | |

| -296.27 | | -4530.5 | | -340.59 | | -739.25 | | -123.79 | | -283.52 | | NA |  | -269.82 | |
| --- | --- | --- | --- | --- | --- | --- | --- | --- | --- | --- | --- | --- | --- | --- | --- |
| -496.66 | | -6436.71 | | -550.54 | | -995.99 | | -176.41 | | -486.57 | |  | -12.97 | -771.85 | |
| -249.08 | | -3982.64 | | -322.21 | | -511.41 | |  | -77.86 | -264.75 | | NA |  | -339.19 | |
|  | -73.12 | -1582.56 | |  | -99.62 | -166.63 | |  | -7.69 |  | -90.28 | NA |  |  | -33.59 |
| -272.57 | | -469.75 | | -455.24 | | -193.37 | |  | -8.96 | -148.96 | | NA |  | -100.36 | |
| NA |  | -300.06 | | -128.83 | |  | -7.72 | NA |  |  | -12.02 | NA |  |  | -9.31 |
| -293.11 | | -490.46 | |  | -515.3 | -219.48 | |  | -19.88 | -206.41 | | NA |  | -128.34 | |
| -382.47 | | -4942.37 | |  | -377.6 | -234.79 | |  | -23.27 | -171.87 | | NA |  | -3631.56 | |
| -473.97 | | -5692.25 | | -451.39 | | -285.71 | |  | -37.72 | -204.39 | | NA |  | -4052.19 | |
| NA |  | -1591.34 | |  | -12.74 | NA |  | NA |  | NA |  | NA |  | -642.89 | |
| -482.35 | | -6048.55 | | -440.98 | | -291.03 | |  | -28.92 |  | -214.1 | NA |  | -4261.66 | |
| -230.23 | | -4258.81 | |  | -216.2 | -142.62 | |  | -10.44 |  | -81.03 | NA |  | -2475.51 | |
| NA |  | NA |  | NA |  |  | -34.5 | NA |  |  | -7.92 | NA |  | NA |  |
| -179.35 | | -184.87 | |  | -97.95 | -217.97 | |  | -31.09 | -118.14 | | NA |  |  | -36.94 |
| NA |  | NA |  | NA |  |  | -9.16 | NA |  | NA |  | NA |  | NA |  |
| NA |  | NA |  | NA |  | NA |  | NA |  | NA |  | NA |  | NA |  |
| NA |  | NA |  | NA |  | NA |  | NA |  | NA |  | NA |  | NA |  |
|  | -18.87 |  | -41.07 |  | -22.32 |  | -15.9 | NA |  |  | -31.98 | NA |  |  | -38.78 |
| NA |  | NA |  | NA |  | NA |  | NA |  | NA |  | NA |  | NA |  |
| NA |  | NA |  | NA |  | NA |  | NA |  | NA |  | NA |  | NA |  |
| NA |  | NA |  | NA |  | NA |  | NA |  | NA |  | NA |  | NA |  |
| NA |  | NA |  | NA |  | NA |  | NA |  | NA |  | NA |  | NA |  |
| NA |  | NA |  | NA |  | NA |  | NA |  | NA |  | NA |  | NA |  |
|  | -30.95 | NA |  |  | -24.91 |  | -13.22 | NA |  |  | -61.5 | NA |  | NA |  |
| -185.29 | |  | -5.58 | -166.35 | |  | -81.96 |  | -34.74 | -189.36 | | NA |  | NA |  |
| NA |  | NA |  | NA |  | NA |  | NA |  | NA |  | NA |  | -1364.66 | |
| NA |  | NA |  | NA |  | NA |  | NA |  | NA |  | NA |  | -1313.19 | |
| NA |  | NA |  | NA |  | NA |  | NA |  | NA |  | NA |  | -1142.52 | |
| -388.73 | | -1057.26 | |  | -415.6 | -433.56 | | -153.71 | | -746.54 | | NA |  | -257.57 | |
| -326.17 | | -237.28 | |  | -198.6 | -173.46 | | -133.04 | | -280.42 | |  | -12.84 | -302.03 | |
| -326.17 | | -237.28 | |  | -198.6 | -173.46 | | -133.04 | | -280.42 | |  | -12.84 | -302.03 | |
| NA |  |  | -10.33 | NA |  | NA |  | NA |  | NA |  | NA |  | NA |  |
| NA |  |  | -87.21 | NA |  | NA |  | NA |  |  | -8.25 | NA |  | NA |  |
| NA |  |  | -62.75 | NA |  | NA |  | NA |  | NA |  | NA |  | NA |  |
|  | -12.75 |  | -45.67 |  | -10.44 |  | -5.76 | NA |  |  | -37.49 | NA |  | NA |  |
| -807.11 | | -275.81 | | -681.35 | | -717.98 | |  | -451.6 | -598.65 | |  | -19.32 |  | -61.6 |
| NA |  | -175.11 | |  | -10.79 | NA |  | NA |  |  | -7.89 | NA |  | NA |  |
| NA |  |  | -6.96 | NA |  | NA |  | NA |  | NA |  | NA |  | NA |  |
| NA |  | -194.29 | |  | -7.09 | NA |  | NA |  |  | -9.63 | NA |  | NA |  |

|  | -12.87 | -111.6 | -26.75 NA | NA |  | -6.04 NA | NA |
| --- | --- | --- | --- | --- | --- | --- | --- |
| NA |  | -218.68 NA | NA | NA | NA | NA | NA |
| NA |  | -160.61 NA | NA | NA | NA | NA | NA |
| NA |  | -89.83 NA | NA | NA | NA | NA | NA |
| NA |  | -179.46 NA | NA | NA | NA | NA | NA |

-243.06	-965.28	-324.37	-235.33	-69.29	-248.99 NA	-379.88

NA	NA	NA	NA	NA	NA	NA	NA NA	NA	NA	NA	NA	NA	NA	NA NA	NA	NA	NA	NA	NA	NA	NA

NA	NA	NA	NA	NA	NA	NA	-946.73

-25.39	-163.33	-30.7	-338.89	-16.56	-43.88 NA	-126.35

NA	-136.08	-39.55	-197.85 NA	-34.67 NA	-68.46

-140.69	-853.26	-226.31	-217.98	-15.61	-173.43 NA	-70.39

NA	NA	NA	NA	NA	NA	NA	-1318.5

NA	-66.49	-15.8	-301.71	-8.57	-18.79 NA	-132.32

-16.53	-86.2	-16.05	-178.12 NA	-19.34 NA	-329.02

-18.01	-210.19	-30.83	-56.38 NA	-23.83 NA	-257.87

NA	-166.09	-11.44	-130.49 NA	-17.15 NA	-131.04

NA	NA	NA	NA	NA	NA	NA	-1849.25

-144.05	-22.09	-134.69	-168.18	-68.9	-176.35	-43.25	-191.92

-311.26	-205.29	-237.12	-281.35	-125.67	-319.32	-48.77	-391.99

-64.58	-206.61	-44.01	-22.65 NA	-49.35 NA	NA

-52.89	-185.62	-38.81	-17.39 NA	-48.71 NA	NA

-500.91	-232.77	-386.51	-631.9	-450.42	-518 NA	-564.06

-174.82	-940.55	-179.26	-230.23	-39.74	-189.19 NA	-242.88

-12.3	-106.64	-16.43 NA	NA	-28.71 NA	NA

-199.05	-215.53	-107.89	-248.3	-40.48	-152.84 NA	-61.02

| -36.67 | -228.11 | -29.04 | -86.97 NA | |  | -37.57 | | -9.52 | -78.23 |
| --- | --- | --- | --- | --- | --- | --- | --- | --- | --- |
| -230.45 | -747.29 | -223.04 | -232.27 | | -52 | -209.84 NA | |  | -545.8 |
| -27.23 | -308.76 | -52.54 | -65.56 | | -11.67 | -46.3 NA | |  | -549.15 |
| -68.66 | -259.32 | -41.21 | -60.96 | | -12.89 | -41.48 NA | |  | -566.07 |
| -64.2 | -395.29 | -68.54 | -79.85 | | -19.69 | -94.97 NA | |  | -799.22 |
| -123.43 | -421.85 | -94.4 | -96.86 | | -35.3 | -91.35 NA | |  | -534.06 |
| -123.86 | -656.68 | -119.01 | -177.57 | | -29 | -154.69 NA | |  | -655.51 |
| -54 | -273.92 | -51.83 | -42.39 | | -5.82 | -53.33 NA | |  | -861.97 |
| -5.1 -87.86 -5.31 -13.77 NA -10.71 NA -652.52 | | | | | | | | | |
| NA | -118.78 NA | | NA | NA |  | NA | NA |  | -12.89 |
| NA | -118.78 NA | | NA | NA |  | NA | NA |  | -12.89 |
| NA | -118.78 NA | | NA | NA |  | NA | NA |  | -12.89 |

| -1257.41 | |  | -9.57 | -1612.73 | | -1285.23 | | -428.28 | | -2558.74 | | NA |  | NA |  |
| --- | --- | --- | --- | --- | --- | --- | --- | --- | --- | --- | --- | --- | --- | --- | --- |
| -1631.05 | |  | -16.55 | -2031.77 | | -1588.97 | | -578.46 | | -3064.93 | | NA |  | NA |  |
|  | -872.7 | NA |  | -1081.51 | |  | -882.1 | -469.92 | | -1813.55 | | NA |  | NA |  |
|  | -27.7 | -119.51 | |  | -23.87 | NA |  | NA |  |  | -29.9 | NA |  | NA |  |
| NA |  | -120.18 | | NA |  | NA |  | NA |  | NA |  | NA |  | NA |  |
| NA |  |  | -39.44 | NA |  | NA |  | NA |  | NA |  | NA |  | NA |  |
| NA |  |  | -54.93 | NA |  | NA |  | NA |  | NA |  | NA |  | NA |  |
|  | -12.53 | -1224.49 | |  | -15.06 |  | -7.31 | NA |  |  | -6.07 | NA |  | NA |  |
| -150.68 | | -437.15 | |  | -159.7 | -141.86 | |  | -27.44 | -165.72 | | NA |  | -257.54 | |
| -118.55 | | -628.41 | | -113.12 | | -135.35 | |  | -32.32 | -188.12 | | NA |  | -366.81 | |
| -148.94 | | -1082.3 | |  | -193 | -115.41 | |  | -33.86 | -169.35 | | NA |  | -508.06 | |
| -148.94 | | -1082.3 | |  | -193 | -115.41 | |  | -33.86 | -169.35 | | NA |  | -508.06 | |
|  | -151.1 | -747.52 | | -189.03 | | -122.92 | |  | -49.78 | -193.07 | | NA |  | -317.26 | |
| NA |  | -148.44 | | -165.73 | |  | -40.47 | NA |  |  | -36.21 | NA |  | NA |  |
| NA |  | -148.44 | | -165.73 | |  | -40.47 | NA |  |  | -36.21 | NA |  | NA |  |
|  | -9.11 | NA |  |  | -14.55 | NA |  | NA |  | NA |  | NA |  | -623.35 | |
| NA |  | NA |  | NA |  | NA |  | NA |  | NA |  | NA |  | -748.77 | |
| NA |  | NA |  | NA |  | NA |  | NA |  | NA |  | NA |  | -965.18 | |
|  | -93 | NA |  |  | -27.36 |  | -37.4 |  | -82.32 |  | -281 |  | -18.36 |  | -6.25 |
| -2496.82 | | -857.84 | | -1885.28 | | -1921.29 | | -819.61 | | -1744.94 | | NA |  | -143.19 | |
| -3796.71 | |  | -23.54 | -3878.48 | | -3256.33 | | -417.91 | | -1680.11 | | NA |  | NA |  |
| -1465.48 | | NA |  | -1277.65 | | -1094.56 | | -138.95 | | -804.03 | | NA |  |  | -10.18 |
| -2922.62 | | -358.09 | | -2219.06 | | -2126.15 | | -511.78 | | -1515.1 | | NA |  |  | -112.2 |
| -5136.59 | | -102.51 | | -5114.7 | | -4333.7 | | -597.88 | | -2313.59 | | NA |  |  | -19.05 |
| -3719.14 | |  | -51.34 | -3392.95 | | -2915.91 | | -418.54 | | -1718.46 | | NA |  |  | -24.44 |
| -220.18 | | -595.42 | | -114.81 | |  | -98.89 |  | -15.73 |  | -37.14 | NA |  | -170.89 | |
| -323.31 | |  | -64.58 | -207.44 | | -564.06 | |  | -23.95 | -205.55 | | NA |  |  | -28.7 |
| -251.64 | | -809.05 | | -172.94 | |  | -109.3 |  | -29.46 |  | -84.1 | NA |  | -247.33 | |
|  | -32.17 | -676.38 | |  | -50.73 |  | -67.5 | NA |  |  | -39.3 | NA |  |  | -28.06 |
| -374.17 | | -3607.76 | | -457.14 | | -796.48 | | -150.07 | |  | -369.3 |  | -23.28 | -559.97 | |
| -381.38 | | -4969.21 | | -386.12 | | -549.35 | |  | -88.86 | -280.33 | | NA |  | -495.48 | |
| NA |  | -1110.76 | |  | -16.29 | -232.74 | |  | -18.81 |  | -40.08 | NA |  | NA |  |
| NA |  | NA |  | NA |  | NA |  |  | -5.7 | NA |  | NA |  | -141.43 | |
| NA |  | -501.69 | | NA |  | NA |  | NA |  | NA |  | NA |  |  | -55.46 |
| NA |  | -344.32 | | NA |  | NA |  | NA |  | NA |  | NA |  |  | -7.51 |
|  | -39.22 | -117.01 | |  | -75.63 | -119.76 | |  | -48.07 |  | -99.77 |  | -38.62 |  | -85.88 |
|  | -191.8 | -622.66 | | -219.31 | |  | -299.4 |  | -56.52 | -239.72 | |  | -38.17 | -389.89 | |
| -153.07 | | -373.28 | |  | -164.3 |  | -266.3 | -114.13 | | -197.07 | |  | -77.62 | -276.78 | |
| NA |  | NA |  | NA |  | NA |  | NA |  | NA |  |  | -38.75 | NA |  |

| -165.88 | | -465.95 | |  | -184.5 | -272.55 | |  | -76.85 | -230.38 | |  | -44.13 | -303.15 | |
| --- | --- | --- | --- | --- | --- | --- | --- | --- | --- | --- | --- | --- | --- | --- | --- |
| -474.92 | | -4771.89 | | -562.66 | | -745.32 | | -147.95 | | -478.45 | |  | -19.59 | -749.66 | |
| -142.56 | | -528.61 | | -197.79 | | -210.39 | |  | -78.26 | -326.63 | | NA |  |  | -32.8 |
| -118.43 | | -529.26 | | -177.32 | | -176.47 | |  | -57.93 | -314.74 | | NA |  |  | -8.52 |
|  | -65.22 | -365.99 | | -103.84 | |  | -100.2 |  | -37.29 | -191.07 | | NA |  | NA |  |
|  | -40.93 | -790.87 | |  | -48.31 |  | -95.33 |  | -8.81 |  | -82.57 | NA |  |  | -33.53 |
|  | -16.6 | -1049.48 | |  | -46.98 |  | -38.79 | NA |  |  | -22.58 | NA |  | -806.13 | |
| -136.11 | | -613.96 | | -180.75 | | -204.68 | |  | -49.95 | -314.14 | | NA |  |  | -19.6 |
|  | -66.34 | -384.28 | | -114.92 | | -111.81 | |  | -27.94 | -226.37 | | NA |  | NA |  |
| NA |  | NA |  | NA |  | NA |  | NA |  | NA |  | NA |  | -1962.93 | |
| NA |  | NA |  | NA |  | NA |  | NA |  | NA |  | NA |  | -2102.99 | |
| NA |  | NA |  | NA |  | NA |  | NA |  | NA |  | NA |  | -1555.3 | |
| -675.67 | | -503.89 | | -499.76 | | -752.57 | | -618.82 | | -753.75 | |  | -54.69 |  | -668.9 |
| -233.61 | | -411.22 | | -258.16 | | -296.84 | | -144.49 | | -349.59 | |  | -35.26 | -364.44 | |
|  | -11.6 | -1094.4 | |  | -54 | -106.16 | | NA |  |  | -20.95 | NA |  | NA |  |
| -159.48 | | -323.67 | | -143.77 | | -276.26 | | -107.61 | | -204.53 | |  | -11.18 | -137.49 | |
|  | -8.7 | -954.04 | |  | -41.12 |  | -84.97 | NA |  |  | -18.52 | NA |  | NA |  |
|  | -14.98 | NA |  |  | -38.04 |  | -41.51 |  | -67.03 | -222.77 | |  | -25.44 | NA |  |
|  | -79 | -161.14 | |  | -99.1 |  | -98.52 |  | -48.72 | -112.76 | |  | -26.03 | -188.67 | |
| NA |  | NA |  | NA |  | NA |  | NA |  | NA |  | NA |  | -1015.05 | |
| -407.23 | | -1445.46 | | -448.82 | | -478.29 | | -224.89 | | -469.55 | |  | -26.88 |  | -950.8 |
|  | -12.65 | -360.18 | |  | -29.62 |  | -11.94 | NA |  | NA |  | NA |  | -143.41 | |
| NA |  | NA |  | NA |  | NA |  |  | -10.97 | NA |  |  | -31.7 | NA |  |
| NA |  |  | -26.36 | NA |  | NA |  | NA |  | NA |  |  | -11.84 |  | -16.46 |
| -130.17 | | -112.61 | | -132.25 | |  | -191.3 | -101.29 | | -287.74 | |  | -34.36 | -435.42 | |
| -275.23 | | -115.73 | | -131.13 | | -184.52 | |  | -92.6 | -374.99 | |  | -29.45 | -323.76 | |
| -221.97 | | -205.36 | | -149.47 | | -231.83 | |  | -97.29 | -277.98 | |  | -35.42 | -579.66 | |
|  | -131.2 |  | -200.1 | -135.47 | | -260.43 | | -106.37 | | -233.93 | |  | -44.82 | -356.15 | |
|  | -84.69 |  | -20.38 |  | -73.53 |  | -34.94 |  | -25.05 | -128.22 | | NA |  |  | -14.38 |
| -214.86 | | -490.64 | | -229.04 | | -293.46 | | -125.47 | | -256.13 | | NA |  |  | -325.4 |
| -323.14 | | -189.39 | | -230.67 | | -157.52 | | -120.37 | | -389.64 | |  | -9.06 | -103.87 | |
| -249.77 | | -1063.37 | | -359.97 | | -403.25 | | -174.79 | | -332.35 | |  | -33.85 | -582.66 | |
| -120.39 | | -573.99 | | -125.83 | | -126.49 | |  | -34.52 | -182.92 | | NA |  | -174.24 | |
| -151.97 | | -608.82 | | -125.65 | | -184.85 | | NA |  | -165.88 | | NA |  | -252.61 | |
|  | -34.09 | NA |  |  | -31.11 |  | -74.85 | -159.41 | |  | -109.1 |  | -43.99 |  | -63.62 |
| -411.66 | | -326.79 | | -197.69 | | -207.88 | |  | -53.15 | -119.87 | | NA |  | -159.34 | |
| -781.85 | |  | -407.5 | -471.38 | | -488.38 | | -103.01 | | -313.58 | | NA |  |  | -143 |
|  | -99.48 | -422.83 | | -109.34 | |  | -82.91 |  | -49.52 | -127.78 | |  | -5.12 | -118.38 | |
|  | -197.1 | -707.29 | | -227.35 | | -287.91 | | -236.49 | | -212.74 | |  | -58.44 |  | -851.1 |

| -155.19 | | -448.58 | | -104.22 | | -153.31 | |  | -51.96 | -164.32 | |  | -8.11 |  | -166 |
| --- | --- | --- | --- | --- | --- | --- | --- | --- | --- | --- | --- | --- | --- | --- | --- |
| -408.45 | |  | -431.6 | -299.18 | | -312.65 | | -105.46 | | -352.72 | |  | -6.58 | -386.51 | |
| -216.65 | | -1487.19 | |  | -318.3 | -261.48 | |  | -74.11 | -228.42 | | NA |  | -201.29 | |
|  | -47.66 |  | -89.36 |  | -53.51 |  | -97.9 |  | -28.33 | -174.27 | | NA |  |  | -69.6 |
| -631.53 | | -849.95 | | -453.54 | | -496.43 | | -378.12 | |  | -409.8 | NA |  | -660.43 | |
| NA |  | -534.83 | | NA |  | NA |  | NA |  | NA |  | NA |  | -301.54 | |
|  | -442.4 | -1086.32 | | -409.84 | | -433.99 | | -130.23 | | -340.25 | | NA |  | -661.35 | |
|  | -87.25 | -1486.57 | | -158.88 | | -194.16 | | NA |  | -116.89 | | NA |  | -318.68 | |
|  | -177.3 | -560.48 | | -177.47 | |  | -164.3 |  | -78.35 | -220.47 | |  | -26.3 | -371.96 | |
| -118.33 | | -256.36 | |  | -91.05 | -113.75 | |  | -44.7 | -171.78 | | NA |  | -166.42 | |
| -201.75 | | -393.96 | | -159.44 | | -124.35 | |  | -68.65 | -232.06 | |  | -16.6 | -201.68 | |
| NA |  | NA |  | NA |  | NA |  | NA |  | NA |  | NA |  | -106.14 | |
| -194.58 | | -136.79 | | -162.18 | | -186.68 | |  | -51.89 | -157.81 | | NA |  | -182.72 | |
| -110.83 | | NA |  |  | -54.62 |  | -75.83 | -116.17 | | -200.66 | |  | -43.65 |  | -34.91 |
| -206.86 | |  | -31.59 | -138.92 | | -170.36 | | -193.02 | |  | -456.9 |  | -23.49 |  | -51.44 |
| -593.89 | | -2343.85 | | -595.04 | | -722.37 | | -266.64 | | -567.11 | |  | -34.8 | -1421.45 | |
|  | -19.48 | -219.27 | |  | -28.82 |  | -47.63 |  | -17.42 |  | -68.27 |  | -9.98 | -164.43 | |
| -173.67 | | -1017.6 | | -248.61 | | -286.06 | |  | -159 | -314.09 | |  | -21.06 | -404.42 | |
| NA |  |  | -63.89 | NA |  | NA |  | NA |  | NA |  | NA |  | -116.56 | |
| -106.41 | | -163.07 | |  | -96.74 |  | -80.78 |  | -9.76 |  | -93.23 | NA |  |  | -48.57 |
| -271.92 | | -356.82 | | -222.43 | | -185.53 | |  | -70.25 | -496.72 | | NA |  | -296.84 | |
| -123.87 | | NA |  |  | -75.34 |  | -26.52 | NA |  |  | -38.31 | NA |  | NA |  |
| -132.32 | | -684.37 | | -165.98 | | -136.63 | |  | -36.61 | -130.84 | |  | -11.59 | -369.09 | |
| -575.05 | |  | -8.91 | -308.66 | | -198.87 | |  | -93.23 |  | -233.2 | NA |  |  | -7.72 |
|  | -159.4 | -680.02 | | -152.85 | | -157.02 | | NA |  | -199.32 | | NA |  | -353.04 | |
| -296.88 | |  | -96.97 |  | -257.9 | -207.11 | |  | -41.67 | -141.36 | | NA |  |  | -64.16 |
| -159.96 | | -663.56 | | -145.03 | | -106.19 | |  | -26.29 | -123.93 | | NA |  |  | -452.9 |
| -276.66 | |  | -93.35 |  | -137.2 | -135.19 | |  | -99.54 |  | -280.3 |  | -36.32 | -165.77 | |
|  | -71.52 | -724.76 | | -101.77 | | -110.28 | |  | -27.38 |  | -108.8 | NA |  | -184.48 | |
|  | -42.8 | -220.63 | |  | -41.25 |  | -40.41 | NA |  |  | -62.86 | NA |  | -192.19 | |
| -386.82 | |  | -90.23 | -292.12 | | -214.19 | |  | -61.11 | -223.54 | | NA |  |  | -33.87 |
| -292.91 | | -485.12 | | -264.06 | |  | -327.2 | -328.64 | | -387.68 | |  | -71.51 | -595.75 | |
| -181.84 | | -288.11 | | -141.47 | |  | -132.4 |  | -63.41 | -158.74 | |  | -19.34 | -172.19 | |
| -221.76 | |  | -19.97 | -259.48 | | -222.61 | |  | -71.07 | -351.76 | | NA |  | NA |  |
| NA |  | NA |  |  | -6.07 |  | -18.6 | NA |  |  | -37.82 | NA |  |  | -24.67 |
|  | -58.29 | -451.26 | | -101.35 | | -128.44 | |  | -28.69 | -134.61 | | NA |  |  | -226.4 |
| -152.28 | | -188.29 | | -123.68 | | -121.52 | |  | -42.53 | -172.12 | | NA |  | -130.75 | |
| -270.45 | | -113.18 | | -191.22 | | -129.33 | |  | -88.46 | -340.76 | |  | -13.67 | -149.28 | |

neurosphe neutrophil neutrophil neutrophil normal_hu osteoclast plasma_ce podocyte..

-219.64	-287.22	-162.31	-163.74	-133.26	-210.73	-278.64	-279.92

NA	NA	NA	NA	NA	NA	NA	NA

-77.05	-320.01	-261.89	-274.94	-618.88	-362.18	-1413.79	-430.76

-196.77	-203.52	-216.85	-195.36	-722.05	-129.55	-1313.42	-242.68

-5.5	-47.67	-67.8	-54.34	-216.74	-22.91	-1891.22	-146.28

-744.83	-714.66	-809.9	-823.67	-1401.66	-707.8	-1976.05	-828.99

-33.16	-336.22	-314.08	-331.82	-535.55	-349.56	-2676.82	-431.79

-68.56	-91.13	-53.27	-53.76	-75.89	-41.17	-1088.47	-128.6

-68.56	-91.13	-53.27	-53.76	-75.89	-41.17	-1088.47	-128.6

-21.85	-171.83	-160.83	-141.58	-121.24	-172.59	-49.86	-148.58

-476.15	-502.7	-487.14	-507.91	-576.26	-491.73	-1079.48	-552.93

-121.05	-182.62	-168.46	-183.83	-585.6	-116.24	-1277.95	-262.75

-52.68	-143.15	-266.79	-272.73	-138.73	-582.14	-743.85	-1654.36

-123.27	-201.48	-287.3	-276.33	-493.02	-612.37	-972.19	-2266.29

NA	NA	NA	NA	-20.3 NA	NA	NA

-39.79 NA	NA	NA	NA	NA	NA	-5.13

NA	NA	NA	-6.78	-75.03	-138.07	-59.48	-637.67

NA	NA	NA	-6.78	-75.03	-138.07	-59.48	-637.67

-27.27	-2732.26	-1521.71	-1564.64	-148.69	-4979.7	-471.6	-108.04

-57.29	-287.64	-262.47	-260.92	-906.67	-235.87	-2877.12	-409.09

-45.85 NA	NA	NA	NA	NA	NA	-7.42

-50.83	-1476.45	-1262.69	-1325.12	-72.57	-1925.42	-962.58	-244.14

NA	NA	NA	NA	-1120.24 NA	NA	NA

NA	NA	NA	NA	-2460.29 NA	NA	-78.27

NA	-518.96	-570.8	-490.21	-42.02	-417.25 NA	NA NA	-1725.82	-1847.39	-1544.31 NA	-1236.37 NA	NA NA	-1830.64	-1945.67	-1596.8 NA	-1309.83 NA	NA NA	-1203.74	-1383.55	-1101.48 NA	-836.97 NA	NA

-83.69	-310.91	-258.64	-274.34	-668.11	-281.14	-1951.2	-382.34

-83.69	-310.91	-258.64	-274.34	-668.11	-281.14	-1951.2	-382.34

-197.02	-495.76	-437.18	-457.96	-927.85	-440.01	-3028.38	-547.73

-158.3	-569.2	-463.94	-487.9	-1052.67	-543.17	-3027.13	-559.06

NA	-216.73	-202.55	-176.87	-98.54	-255.6	-22.43	-150.86

NA	-686.75	-518.94	-425.67 NA	-661.1 NA	NA NA	NA	NA	NA	NA	NA	NA	NA NA	NA	NA	NA	NA	NA	NA	NA

-40.79 NA	NA	NA	NA	NA	NA	NA

-334.34	-288.02	-105.26	-118.72	-25.72	-121.46	-53.21	-84.87

-99.18	-87.43	-91.82	-87.35	-45.38	-57.57	-1533.39	-221.82

-99.18	-87.43	-91.82	-87.35	-45.38	-57.57	-1533.39	-221.82

-99.18	-87.43	-91.82	-87.35	-45.38	-57.57	-1533.39	-221.82

-433.4	-229.35	-18.07	-27.21	-158.17	-121.31	-69.43	-427.86

-400.67	-175.71	-22.55	-31.84	-195.41	-79.53	-96.34	-306.31

-86	-5474.09	-3474.56	-3499.96	-588.22	-5640.37	-1002.81	-271.23

-100.47	-6567.07	-4856.18	-4869.89	-1827.48	-7465.41	-1299.09	-773.92

-168.14	-9225.97	-6893.65	-6844.6	-1675.82	-10455.9	-2177.02	-874.21

-220.41	-7022.7	-4431.38	-4485.61	-1081.01	-7668.48	-1202.56	-744.13

-66.19	-4244.61	-3277.4	-3196.38	-1749.83	-4808.09	-1059.24	-635.45

-77.2	-5288.21	-4342.95	-4215.55	-1207	-6109.34	-1458.76	-579.76

-40.36	-8513.74	-6432.28	-6435.01	-2062.98	-10340	-1607.28	-661.12

-241.72	-5318.75	-4572.48	-4517.14	-1156.25	-5553.27	-1593.19	-992.2

-149.74	-6220.62	-5265.47	-5268.51	-1134.91	-6996.99	-1892.73	-871.77

-236.39	-7554.94	-6235.02	-6219.8	-1406.14	-8215.35	-2203.7	-1095.74

-47.19 NA	NA	NA	NA	NA	NA	NA

-51.53	-128.14	-100.5	-77.39	-386.13	-61.26	-2918.15	-234.93

-167.73	-7457.24	-6081.81	-6111.94	-932.49	-8553.3	-1784.82	-961.3

-205.82	-8242.94	-6991.34	-6947.5	-999.59	-9528.59	-2499.08	-1009.22

-34.56	-566.65	-544.55	-528.74	-71.79	-680.7	-206.41	-138.62

-150.26	-11021.4	-8440.53	-8548.02	-1324.14	-12163.5	-2618.49	-846.92

NA	NA	NA	NA	-300.58 NA	-228.84	-93.33

-13.52 NA	NA	NA	NA	NA	NA	NA

-159.91	-9433.44	-7692.67	-7658.88	-1238.56	-10647.1	-2643.1	-994.2

-51.43	-368.26	-294.44	-301.99	-48.13	-382.69	-141.12	-73.36

-106.58	-5395.8	-3843.56	-3797.39	-682.88	-6213.72	-795.84	-450.91

-233.06	-9079.61	-7345.46	-7316.27	-1256.56	-9987.76	-2387.58	-1097.04

-117.56	-6438.86	-5287.69	-5293.79	-749.48	-7757.09	-1569.04	-802.83

-88.1	-2687.66	-2363.06	-2346.75	-581.79	-3060.69	-854.69	-586.15

-88.1	-2687.66	-2363.06	-2346.75	-581.79	-3060.69	-854.69	-586.15

-197.3	-10031.8	-7982.2	-7943.4	-1193.7	-11317.8	-2346.6	-973.57

-184.21	-4369.5	-3518.09	-3495.93	-441.2	-4561.94	-1288.81	-523.6

-17.07	-3782.43	-2533.67	-2434.19	-132	-3864.9	-614.93	-98.81

-49.78	-3197.17	-2366.38	-2397.8	-589.77	-3601.04	-1099.94	-379.81

-22.57 NA	NA	NA	NA	NA	NA	-43.1

-98.24	-218.9	-223.57	-234.55	-563.67	-162.94	-1374.71	-347.98

-98.09	-9120.37	-7238.8	-7207.13	-1133.26	-11078.2	-1982.51	-826.44

-197.9	-10810.5	-8567.71	-8573.48	-1289.6	-12044.9	-2704.72	-1031.64

-38.78	-605.58	-608.6	-583.92	-85.21	-704.14	-285.08	-158.87

-91.7	-78.14	-146.43	-161.49	-359.31	-690.43	-673.97	-2844.03

-108.8	-132.41	-157.65	-173.07	-336.01	-708.28	-400.32	-2160.47

-132.71	-231.4	-296.56	-311.8	-446.7	-873.93	-913.51	-2696.55

-26.65	-7.18	-56.13	-59.48	-325.23	-313.52	-966.52	-2089.34

NA	NA	NA	NA	-2865.95 NA	NA	-139.14

NA	NA	NA	NA	-1283.81 NA	NA	-58.95

NA	NA	NA	NA	-1754.49 NA	NA	-176.49

NA	NA	NA	NA	-1121.28 NA	NA	-68.87

NA	NA	NA	NA	-2352.25 NA	NA	-260.76

NA	NA	NA	NA	-1996 NA	NA	-245.78

NA	NA	NA	NA	-525.49 NA	NA	-21.83

NA	NA	NA	NA	-525.49 NA	NA	-21.83

NA	NA	NA	NA	-525.49 NA	NA	-21.83

NA	NA	NA	NA	-1566.95 NA	NA	-102.81

NA	NA	NA	NA	-2053.45 NA	NA	-265.5

NA	NA	NA	NA	-1906.74	-12.17 NA	-303.19

NA	NA	NA	NA	-784.22 NA	NA	-149.83

NA	NA	NA	NA	-943.79 NA	NA	-146.98

-13.95	-13.09	-10.95	-11.44	-1638.64	-48.21 NA	-295.42

NA	NA	NA	NA	-2349.04	-7.31 NA	-271.81

NA	NA	NA	NA	-337.42 NA	NA	-10.12

| -264.64 | | -8275.68 | -6602.24 | -6555.98 | -1304.59 | -8879.18 -2224.34 | | | -989.75 | |
| --- | --- | --- | --- | --- | --- | --- | --- | --- | --- | --- |
| NA | | -103.42 | -55.45 | -68.03 | -334.04 | -135.07 NA | | | NA | |
| NA | | -103.42 | -55.45 | -68.03 | -334.04 | -135.07 NA | | | NA | |
| NA | | NA | NA | NA | -75.76 NA | | NA | | NA | |
| NA | | NA | NA | NA | -75.76 NA | | NA | | NA | |
| NA | | NA | NA | NA | -347.98 | | -8.41 NA | | NA | |
| NA -50.54 -54.72 -63.81 -948.33 -104.82 NA NA | | | | | | | | | | |
| NA | NA | | NA | NA | -131.17 NA | | NA | | NA |  |
| NA | NA | | NA | NA | -161.86 NA | | NA | | NA |  |
| NA | NA | | NA | NA | -161.86 NA | | NA | | NA |  |
| NA | NA | | NA | NA | -161.86 NA | | NA | | NA |  |
| NA | NA | | NA | NA | NA NA | | NA | | NA |  |
|  | -16.21 NA | | NA | NA | NA NA | | NA | | NA |  |
|  | -35.13 NA | | NA | NA | NA NA | | NA | |  | -13.63 |
|  | -36.1 NA | | NA | NA | NA NA | | NA | |  | -15.33 |
| -126.27 | | -74.23 | -87.75 | -99.95 | -205.85 | - | 61.71 | -443.84 | -181.11 | |
| -228.93 | | -161.19 | -182.36 | -166.28 | -134.76 | - | 39.54 | -2041.59 | -147.97 | |
| -228.93 | | -161.19 | -182.36 | -166.28 | -134.76 | - | 39.54 | -2041.59 | -147.97 | |

-240.4	-215.11	-260.01	-244.29	-593.17	-87.88	-3332.85	-349.04

NA	NA	NA	NA	-37.81 NA	-725	-5.18

-145.86	-260.77	-208.52	-212.98	-289.7	-175.81	-1147.68	-305.4

-231.69	-24.12	-79.38	-70.69	-28.62 NA	-1142.86	-44.63

-256.72	-519.6	-403.61	-422.59	-241.19	-432.18	-742.94	-327.59

NA	-697.93	-649.33	-514.59 NA	-540.66 NA	NA

NA	NA	NA	NA	-73.47 NA	NA	-138.21

NA	NA	NA	NA	-115.97 NA	NA	-258.91

-36.14 NA	-20.8	-23.68	-2647.07	-7.43	-213.14	-154.93

-11.82	-9.87	-12.5	-10.67	-2548.92	-6.95	-127.51	-201.09

-110.36	-60.86	-100.46	-95.31	-2400.52	-56.8	-402.8	-244.12

-44.03 NA	NA	NA	NA	NA	NA	-23.71

NA	NA	NA	NA	-2000.54 NA	NA	-73.68

NA	NA	NA	NA	-1066.3 NA	NA	NA NA	NA	NA	NA	-1578.81 NA	NA	NA NA	NA	NA	NA	-470.21 NA	NA	NA

-24.6 NA	NA	NA	NA	NA	NA	-30.84

-22.54 NA	NA	NA	NA	NA	NA	-98.17

-27.84 NA	NA	NA	NA	NA	NA	-79.37

NA	NA	NA	NA	-260.82	-13.65 NA	-588.98

NA	NA	NA	NA	-1162.05 NA	NA	-14.71

-42.02 NA	NA	NA	-7.51 NA	NA	-76.95

-33.35 NA	NA	NA	NA	NA	NA	-106.69

-66.64 NA	NA	NA	NA	NA	NA	-138.9

NA	NA	NA	NA	-2158.13 NA	NA	-153.64

NA	NA	NA	NA	-799.92	-18.58 NA	-682.97

NA	NA	NA	NA	-1800.15 NA	NA	-61.88

NA	NA	NA	NA	-1317.34 NA	NA	-85.71

-19.36 NA	NA	NA	NA	NA	NA	-23.59

-19.23 NA	NA	NA	NA	NA	NA	-23.19

-47.52 NA	NA	NA	-42.22	-54.56 NA	-537.96

-41.7 NA	NA	NA	NA	NA	NA	-17.72

NA	NA	NA	NA	NA	NA	NA	-238.94

NA	NA	NA	NA	NA	NA	NA	-97.61

NA	NA	NA	NA	-2014.12 NA	NA	-131.61

NA	NA	NA	NA	-1930.38 NA	NA	-275.09

NA	NA	NA	NA	-2099.52 NA	NA	-67.38

NA	NA	NA	NA	-2249.77 NA	NA	-194.15

NA	NA	NA	NA	-1114.33 NA	NA	-62.23

-83.13 NA	NA	NA	NA	NA	NA	-155.6

NA	NA	NA	NA	-327.22	-41.54 NA	-665.15

-27.58 NA	NA	NA	NA	NA	NA	-8.6

NA	NA	NA	NA	-932.65 NA	NA	NA NA	NA	NA	NA	-1155.03 NA	NA	NA

NA	NA	NA	NA	-2137.91	-11.28 NA	-254.75

NA	NA	NA	NA	-1631.13 NA	NA	-29.84

NA	NA	NA	NA	-1496.64 NA	NA	-93.75

-54.84 NA	NA	NA	NA	NA	NA	-23.35

NA	NA	NA	NA	-2491.69 NA	NA	-208.33

NA	-1797.09	-975.45	-1025.23	-68.6	-2317.38	-98.04	-94.03

NA	-22.69	-27.57	-27.12	-977.67	-49.49	-1011.91	-716.45

NA	NA	-5.14 NA	NA	-184.37 NA	NA

NA	-373.11	-233.14	-236.01	-19.3	-1113.08	-97.45	-51.15

NA	-161.03	-177.63	-186.4	-146.56	-580.41	-463.74	-121.88

NA	-94.8	-102.86	-127.77	-37.96	-379.63	-422.86	-79.55

NA	-5.74	-28.01	-16.9 NA	-37.09	-1328.39	-14.83

NA	-14.46	-9.91	-14.68 NA	-249.93 NA	NA

NA	-312.11	-296.88	-315.98	-78.63	-1315.19	-446.07	-117.23

NA	-32.38	-35.81	-29.74	-14.3	-263.77	-87.05	-12.24

-63.3	-23.03	-59.5	-57.33	-342.93	-460.32	-487.19	-2406.65

-55.26	-54.59	-125.37	-134.45	-239.9	-504.72	-687.07	-2499.02

-98.25	-159.76	-232.79	-242.66	-388.15	-771.9	-635.57	-2429.63

-9.94 NA	-22.63	-24.06	-276.69	-224.1	-623.42	-1759.21

-5.85	-67.9 NA	NA	-2114.84	-30.83	-127.18	-13.7

-127.26	-147.14	-87.38	-84.67	-35.03	-97.15	-613.61	-186.43

-17.38	-52.25	-29.41	-21.47	-2182.53	-36.43	-728.76	-14.63

-37.51	-52.08	-23.17	-29	-543.9	-51.81	-252.35	-29.06

-37.51	-52.08	-23.17	-29	-543.9	-51.81	-252.35	-29.06

-65.25	-50.76 NA	NA	-893.97	-6.63	-141.97	-29.9

-197.04	-222.12	-5.18	-10.2	-663.31	-99.63	-84.03	-166.11

-14.27	-56.87 NA	NA	-2283.17	-22.41	-229.06	-15.28

-14.27	-56.87 NA	NA	-2283.17	-22.41	-229.06	-15.28

-14.27	-56.87 NA	NA	-2283.17	-22.41	-229.06	-15.28

-172.99	-293.08	-22.53	-29.4	-1232.85	-172.28	-41.78	-142.78

-612.47	-382.35	-136.64	-167.89	-34.14	-196.61	-324.58	-301.77

NA	-5.29 NA	NA	-2366.42 NA	-312.09	-5.34

-65.13 NA	NA	NA	NA	NA	NA	-49.29

-34.06 NA	NA	NA	-327.65	-39.47 NA	-24.34

|  | -54.7 NA | | NA | NA |  | -11.05 NA | | NA | -38.71 |
| --- | --- | --- | --- | --- | --- | --- | --- | --- | --- |
|  | -14.68 NA | | NA | NA | NA | NA | | NA | -16.85 |
|  | -5.86 NA | | NA | NA | NA | NA | | NA | -7.54 |
| NA | NA | | NA | NA | NA | NA | | NA | -41.33 |
| -588.41 | | -309.4 | -446.18 | -440.39 | -567.7 | | -274.39 | -1754.33 | -466.05 |
| -268.19 | | -321.77 | -394.61 | -388.99 | -373.4 | | -394.72 | -817.63 | -515.76 |
| -176.85 | | -126.01 | -185.62 | -171.42 | -124.52 | | -89.86 | -1090.85 | -280.09 |
| -463.1 | | -504.85 | -585.94 | -584.29 | -388.76 | | -577.96 | -821.27 | -569.25 |
| -28.66 | | -77.47 | -171.14 | -170.33 NA | |  | -88.83 | -647.32 | -161.94 |
| -73.08 | | -141.11 | -249.4 | -241.82 | | -45.76 | -183.74 | -625.26 | -200.72 |
| -64.16 | | -343.02 | -304.72 | -304.45 | -611.08 | | -304.93 | -2439.7 | -359.67 |
| -650.52 | | -259.08 | -227.27 | -239.18 | -258.2 | | -182.55 | -255.59 | -469.58 |
| -679.24 | | -501.18 | -572.61 | -582.08 | -443.26 | | -382.98 | -317.57 | -1044.14 |
| -87.05 | | -35.84 | -69.03 | -65.21 | -355.56 | | -53.02 | -639.82 | -207.38 |
| -147.85 NA | | | NA | NA |  | -19.49 NA | | NA | -115.6 |
| -36.49 NA | | | NA | NA | NA | NA | | NA | -42.86 |

NA	-196.9	-174.47	-161.94	-913.9	-146.02	-2903.95	-236.01

-20.87 NA	NA	NA	NA	NA	NA	-20.26

-300.22	-311.12	-360.06	-352.25	-1150.56	-323.2	-1059.19	-538.98

-88.78 NA	NA	NA	-52.16 NA	NA	-17.13

-88.78 NA	NA	NA	-52.16 NA	NA	-17.13

| -230.13 | -204.49 | -188.24 | -188.97 | -400.23 | -161.26 | -513.02 | -224.41 |
| --- | --- | --- | --- | --- | --- | --- | --- |
| -68.44 | -353.62 | -310.71 | -321.78 | -609.34 | -313.76 | -2638.79 | -415 |
| -68.44 | -353.62 | -310.71 | -321.78 | -609.34 | -313.76 | -2638.79 | -415 |
| -68.44 | -353.62 | -310.71 | -321.78 | -609.34 | -313.76 | -2638.79 | -415 |
| -68.44 | -353.62 | -310.71 | -321.78 | -609.34 | -313.76 | -2638.79 | -415 |
| -221.59 | -573.45 | -499.86 | -503.53 | -370.16 | -599.14 | -357.84 | -388.49 |
| -221.59 | -573.45 | -499.86 | -503.53 | -370.16 | -599.14 | -357.84 | -388.49 |
| -221.59 | -573.45 | -499.86 | -503.53 | -370.16 | -599.14 | -357.84 | -388.49 |
| -221.59 | -573.45 | -499.86 | -503.53 | -370.16 | -599.14 | -357.84 | -388.49 |
| -66.39 | -276.77 | -228.43 | -247.9 | -1049.07 | -411.7 | -355.7 | -600.3 |
| -66.39 | -276.77 | -228.43 | -247.9 | -1049.07 | -411.7 | -355.7 | -600.3 |
| -22.33 | -197.98 | -155.27 | -141.44 | -546.33 | -287.57 | -150.13 | -249.03 |
| -22.33 | -197.98 | -155.27 | -141.44 | -546.33 | -287.57 | -150.13 | -249.03 |
| -191.76 | -596.98 | -524.38 | -529.01 | -821.49 | -533.75 | -3687.99 | -602.76 |
| -470.33 | -322.72 | -422.6 | -420.06 | -1193.83 | -288.23 | -1894.25 | -586.71 |
| -470.33 | -322.72 | -422.6 | -420.06 | -1193.83 | -288.23 | -1894.25 | -586.71 |
| -251.63 | -358.51 | -356.54 | -378.31 | -1184.08 | -389.77 | -815.19 | -504.19 |
| -251.63 | -358.51 | -356.54 | -378.31 | -1184.08 | -389.77 | -815.19 | -504.19 |

| -326.59 | | -321.78 | | -346.24 | | -343.21 | | -1185.02 | | -209.14 | | -1860.17 | | -442.23 | |
| --- | --- | --- | --- | --- | --- | --- | --- | --- | --- | --- | --- | --- | --- | --- | --- |
| -719.43 | | -509.26 | | -573.38 | | -576.81 | | -1391.12 | | -446.58 | | -2035.28 | | -793.26 | |
| -324.53 | |  | -295.6 | -313.59 | | -312.99 | | -877.15 | | -225.56 | | -1598.08 | | -439.96 | |
|  | -20.02 | -115.93 | | -103.74 | | -116.73 | |  | -126.7 | -108.17 | | -409.14 | |  | -74.16 |
|  | -51.32 | -317.91 | | -431.34 | | -478.63 | | -229.32 | | -832.21 | | -532.52 | | -1210.15 | |
|  | -42.18 |  | -12.36 |  | -39.54 |  | -37.67 | -324.02 | | -353.67 | |  | -435.3 | -1972.92 | |
| -102.27 | | -351.43 | | -502.52 | | -522.67 | | -218.12 | | -704.78 | |  | -969.3 | -1077.86 | |
| -1838.54 | | -390.33 | | -575.45 | | -587.55 | | -1993.66 | | -293.24 | | -934.38 | | -1488.71 | |
| -2054.66 | | -453.24 | | -670.73 | | -668.26 | | -2240.96 | | -349.28 | | -1076.47 | | -1775.57 | |
| -146.52 | | NA |  |  | -41.66 |  | -34.57 | -222.35 | | NA |  | -381.55 | |  | -82.07 |
| -2067.61 | | -449.16 | | -649.68 | | -654.38 | | -2435.59 | | -358.03 | | -1063.79 | | -1892.86 | |
| -689.36 | |  | -215.7 | -300.78 | |  | -302.7 | -1035.58 | | -232.42 | | -398.72 | | -612.42 | |
| NA |  | NA |  | NA |  | NA |  | NA |  | NA |  | -935.49 | | NA |  |
|  | -48.48 | -220.67 | | -170.71 | | -188.85 | |  | -30.57 | -242.28 | | -907.55 | | -208.15 | |
| NA |  | NA |  | NA |  | NA |  | NA |  | NA |  | -967.27 | | NA |  |
|  | -5.2 | NA |  | NA |  | NA |  | NA |  | NA |  | -107.56 | | NA |  |
| NA |  | NA |  | NA |  | NA |  | NA |  | NA |  | NA |  | NA |  |
|  | -58.54 |  | -24.93 |  | -13.73 |  | -14.59 |  | -6.82 |  | -35.4 |  | -48.85 |  | -14.44 |
| NA |  | NA |  | NA |  | NA |  | NA |  | NA |  |  | -17.07 | NA |  |
| NA |  | NA |  | NA |  | NA |  | NA |  | NA |  |  | -37.88 | NA |  |
| NA |  | NA |  | NA |  | NA |  | NA |  | NA |  | -156.79 | | NA |  |
| NA |  | NA |  | NA |  | NA |  | NA |  | NA |  | -196.91 | | NA |  |
|  | -30.71 | NA |  | NA |  | NA |  | NA |  | NA |  |  | -70.49 | NA |  |
| NA |  |  | -33.68 |  | -12.41 |  | -11.94 |  | -8.39 |  | -72.67 |  | -27.72 | NA |  |
|  | -11.86 | -237.93 | |  | -88.67 |  | -94.47 |  | -7.98 | -294.33 | |  | -31.99 |  | -6.59 |
|  | -37.6 | NA |  | NA |  | NA |  | NA |  | NA |  | NA |  |  | -17.86 |
|  | -27.64 | NA |  | NA |  | NA |  | NA |  | NA |  | NA |  |  | -35.89 |
|  | -59.69 | NA |  | NA |  | NA |  | NA |  | NA |  | NA |  |  | -9.36 |
| -147.59 | | -583.01 | | -483.15 | | -482.78 | | -924.47 | | -543.28 | | -3350.82 | | -596.74 | |
| -434.99 | | -497.14 | | -120.53 | | -148.37 | |  | -63.68 | -317.01 | |  | -90.41 | -416.94 | |
| -434.99 | | -497.14 | | -120.53 | | -148.37 | |  | -63.68 | -317.01 | |  | -90.41 | -416.94 | |
| NA |  | NA |  | NA |  | NA |  | -386.34 | | NA |  | NA |  |  | -31.23 |
|  | -17.57 | NA |  |  | -16.16 |  | -12.96 | -144.64 | | NA |  | -665.42 | | -289.18 | |
| NA |  | NA |  | NA |  | NA |  | -295.61 | | NA |  | -363.76 | | -153.54 | |
| NA |  |  | -25.02 | NA |  | NA |  | -921.31 | |  | -9.26 | -165.16 | | -181.32 | |
|  | -70.14 | -1063.34 | | -1111.57 | | -1083.04 | |  | -197.9 | -947.26 | | -864.22 | | -273.36 | |
| NA |  |  | -12.51 |  | -16.74 |  | -13.94 | -1566.88 | |  | -7.81 | -235.43 | | -212.21 | |
| NA |  | NA |  | NA |  | NA |  |  | -96.22 | NA |  | -535.67 | |  | -34.91 |
| NA |  |  | -13.64 |  | -17.68 |  | -12.4 | -1214.56 | | NA |  |  | -355.1 | -297.01 | |

| NA |  |  | -22.55 |  | -10.45 |  | -8.45 | -947.75 | |  | -25.49 |  | -75.83 | -203.16 | |
| --- | --- | --- | --- | --- | --- | --- | --- | --- | --- | --- | --- | --- | --- | --- | --- |
| NA |  | NA |  | NA |  | NA |  | -425.36 | | NA |  | NA |  | -149.26 | |
| NA |  | NA |  | NA |  | NA |  | -418.96 | | NA |  | NA |  | -148.05 | |
| NA |  | NA |  | NA |  | NA |  | -568.64 | | NA |  | -103.04 | | -163.95 | |
| NA |  | NA |  | NA |  | NA |  | -339.32 | | NA |  | -333.99 | | -103.91 | |
| -243.94 | |  | -334.7 | -409.48 | |  | -413.3 | -364.43 | | -459.01 | |  | -652.6 | -582.28 | |
| NA |  | NA |  | NA |  | NA |  | NA |  | NA |  | NA |  | NA |  |
| NA |  | NA |  | NA |  | NA |  | NA |  | NA |  | NA |  | NA |  |
| NA |  | NA |  | NA |  | NA |  | NA |  | NA |  | NA |  | NA |  |
| NA |  | NA |  | NA |  | NA |  | NA |  | NA |  | NA |  | NA |  |
|  | -47.52 |  | -49.12 |  | -67.19 |  | -58.17 |  | -21 |  | -43.57 | -685.06 | | -1329.52 | |
|  | -37.88 |  | -32.9 |  | -56.56 |  | -48.84 |  | -36.68 |  | -30.3 | -1165.23 | |  | -1351 |
|  | -85.97 | -216.74 | | -311.16 | | -308.57 | | -246.03 | | -375.96 | | -711.11 | | -1065.6 | |
|  | -10.43 | NA |  | NA |  | NA |  | NA |  | NA |  | NA |  | NA |  |
|  | -88.27 |  | -9.67 |  | -36.33 |  | -29.64 | NA |  | NA |  | -1652.82 | | -1147.95 | |
|  | -27.06 |  | -17.31 |  | -19.21 |  | -24.47 |  | -73.19 |  | -59.87 | -263.41 | | -1018.51 | |
| -126.69 | |  | -20.94 |  | -30.2 |  | -30.98 | -149.12 | |  | -49.26 | -426.08 | | -637.82 | |
| -155.15 | |  | -18.89 |  | -59.16 |  | -41.11 | NA |  | NA |  | -1352.33 | | -487.71 | |
|  | -40.88 | NA |  | NA |  | NA |  | NA |  | NA |  | NA |  |  | -29.4 |
| -320.95 | | -179.57 | | -140.19 | | -147.29 | | NA |  |  | -34.83 | -1267.18 | |  | -27.5 |
|  | -421.6 | -353.78 | | -229.78 | | -252.91 | |  | -59.93 | -208.97 | | -837.34 | | -200.22 | |
|  | -17.18 | -106.45 | |  | -52.43 |  | -58.45 | -667.55 | |  | -72.02 | -169.34 | | -182.53 | |
|  | -29.69 |  | -89.96 |  | -37 |  | -40.51 | -1239.32 | |  | -62.44 | -113.11 | | -283.56 | |
| -437.32 | | -868.39 | | -806.64 | | -790.42 | | -443.48 | | -700.76 | |  | -30.21 | -358.49 | |
|  | -202.1 | -235.87 | | -224.98 | | -234.22 | | -261.11 | |  | -219.5 | -621.93 | | -254.68 | |
| NA |  |  | -25.08 |  | -11.51 |  | -9.88 | -134.04 | |  | -13.62 | -311.63 | | -153.84 | |
|  | -58.72 | -226.91 | | -191.09 | | -206.35 | |  | -47.51 |  | -273.7 | -946.08 | | -260.53 | |
|  | -59.95 |  | -82.22 |  | -71.79 |  | -77.39 |  | -19.67 |  | -69.82 | -659.54 | | -150.69 | |
| -288.23 | | -377.29 | | -412.88 | | -390.73 | | -327.33 | | -358.18 | | -759.22 | | -294.94 | |
| -395.66 | |  | -53.77 |  | -92.4 |  | -83.61 |  | -44.82 |  | -18.65 | -629.43 | | -100.88 | |
| -318.13 | |  | -79.71 |  | -95.83 |  | -90.18 |  | -189.6 |  | -84.34 | -123.22 | | -154.73 | |
| -470.36 | |  | -97.22 | -118.84 | | -122.86 | | -141.05 | |  | -64.68 | -406.78 | | -181.99 | |
| -254.12 | | -115.45 | | -142.66 | | -149.32 | | -250.28 | | -131.62 | | -253.41 | | -227.51 | |
| -485.17 | | -173.89 | | -166.85 | | -165.94 | | -145.21 | | -115.94 | | -815.52 | | -235.11 | |
| -468.46 | |  | -81.28 |  | -79.86 |  | -80.38 | -141.83 | |  | -80.1 | -129.26 | | -172.25 | |
| -389.14 | |  | -16.39 |  | -18.03 |  | -16.92 |  | -46.37 |  | -12.91 |  | -80.84 |  | -67.46 |
| NA |  | NA |  | NA |  | NA |  |  | -9.32 | NA |  | -168.17 | | NA |  |
| NA |  | NA |  | NA |  | NA |  |  | -9.32 | NA |  | -168.17 | | NA |  |
| NA |  | NA |  | NA |  | NA |  |  | -9.32 | NA |  | -168.17 | | NA |  |

| NA |  | -1404.76 | | -1091.97 | | -1130.7 | |  | -7.35 | -1873.38 | | -995.62 | |  | -52.56 |
| --- | --- | --- | --- | --- | --- | --- | --- | --- | --- | --- | --- | --- | --- | --- | --- |
| NA |  | -1831.84 | | -1390.97 | | -1454.62 | |  | -20.06 | -2389.85 | | -856.58 | |  | -88.95 |
|  | -13.96 | -866.83 | | -562.69 | | -617.82 | | NA |  | -1070.2 | | -351.82 | |  | -43.64 |
|  | -8.24 |  | -38.19 |  | -17.01 |  | -16.65 | -1283.66 | |  | -30.55 | -161.59 | | -273.73 | |
| NA |  | NA |  | NA |  | NA |  | -467.92 | | NA |  | -562.92 | | -175.83 | |
| NA |  | NA |  | NA |  | NA |  | -999.96 | | NA |  |  | -11.69 | -112.37 | |
| NA |  | NA |  | NA |  | NA |  | -1106.84 | | NA |  |  | -25.58 | -167.12 | |
| NA |  |  | -28.3 |  | -29.23 |  | -30.7 |  | -29.39 |  | -67.02 |  | -55.78 | -406.78 | |
| -287.34 | | -188.25 | | -161.71 | | -166.06 | | -193.76 | | -130.12 | | -794.02 | | -192.56 | |
| -260.68 | |  | -123.3 | -121.16 | | -132.52 | | -348.19 | |  | -92.93 | -759.51 | | -299.37 | |
| -294.36 | | -148.46 | |  | -181.2 | -181.59 | | -382.94 | | -138.29 | | -846.75 | |  | -368.6 |
| -294.36 | | -148.46 | |  | -181.2 | -181.59 | | -382.94 | | -138.29 | | -846.75 | |  | -368.6 |
| -229.84 | | -170.01 | | -200.54 | | -205.68 | | -360.63 | | -179.31 | | -665.83 | | -291.74 | |
|  | -27.4 |  | -35.36 |  | -121.2 | -116.89 | | -151.04 | | -355.23 | | -928.53 | | -1748.86 | |
|  | -27.4 |  | -35.36 |  | -121.2 | -116.89 | | -151.04 | | -355.23 | | -928.53 | | -1748.86 | |
| -374.79 | |  | -7.19 |  | -13.3 |  | -11.05 | -267.36 | |  | -29.11 | NA |  |  | -9.16 |
| -390.01 | | NA |  | NA |  | NA |  |  | -85.91 |  | -9.77 | NA |  | NA |  |
| -581.62 | | NA |  | NA |  | NA |  | -266.75 | |  | -8.9 | NA |  | NA |  |
| -145.54 | | -158.83 | | NA |  | NA |  | -722.89 | |  | -47.11 |  | -46.8 |  | -64.19 |
|  | -90.09 | -3011.27 | | -2714.75 | | -2645.97 | | -861.34 | | -3497.33 | | -1138.84 | | -530.16 | |
| NA |  | -5678.97 | | -3890.22 | | -3952.77 | | -145.85 | | -8267.27 | | -801.73 | |  | -25.61 |
|  | -17.65 | -2626.05 | | -1189.34 | | -1197.05 | |  | -44.73 | -3726.88 | |  | -54.56 |  | -12.89 |
|  | -68.32 | -4152.46 | | -2733.48 | | -2771.28 | | -266.43 | | -5103.37 | | -466.34 | | -243.67 | |
| NA |  | -8261.43 | | -5839.38 | | -5883.19 | | -386.41 | | -11852.3 | | -1008.28 | |  | -75.12 |
| NA |  | -6104.34 | | -3857.96 | | -3913.36 | | -287.42 | | -7940.38 | | -446.07 | |  | -65.79 |
|  | -48.87 | -198.39 | | -214.79 | | -219.31 | | -175.19 | | -373.82 | |  | -51.68 | -261.63 | |
| NA |  | -298.93 | | -245.15 | | -256.57 | |  | -45.71 | -1380.3 | | -167.33 | |  | -72.84 |
|  | -87.4 | -234.75 | |  | -230.6 | -237.03 | |  | -400.8 | -311.14 | | -146.41 | | -377.72 | |
|  | -5.64 |  | -54.9 |  | -62.91 |  | -60.26 | -168.26 | |  | -49.33 | -383.82 | |  | -42.61 |
| -540.71 | |  | -402.2 | -436.34 | | -420.72 | | -966.61 | | -306.03 | | -1927.91 | | -569.43 | |
| -299.43 | | -372.98 | | -413.17 | | -411.24 | | -844.35 | | -504.51 | | -661.94 | | -578.51 | |
| NA |  | NA |  | NA |  | NA |  |  | -826.2 | NA |  | -1747.03 | |  | -32.08 |
|  | -17.56 | NA |  | NA |  | NA |  | -309.78 | | NA |  | NA |  |  | -15.69 |
| NA |  | NA |  | NA |  | NA |  | -107.73 | | NA |  | NA |  | -745.99 | |
| NA |  | NA |  | NA |  | NA |  |  | -35.7 | NA |  | NA |  | -703.13 | |
| -184.36 | |  | -68.3 | -103.14 | | -103.16 | |  | -5.1 | NA |  | -1359.43 | | -115.76 | |
| -351.37 | | -272.98 | | -289.61 | | -291.43 | | -154.22 | | -135.34 | | -1629.06 | | -495.15 | |
| -430.76 | | -170.13 | | -226.31 | | -214.64 | |  | -30.01 |  | -58.82 | -1216.3 | | -219.93 | |
|  | -7.91 | NA |  | NA |  | NA |  | NA |  | NA |  |  | -562.2 | NA |  |

| -317.24 | |  | -237.1 | -254.97 | | -265.02 | | -141.35 | |  | -95.56 | -1543.2 | | -420.72 | |
| --- | --- | --- | --- | --- | --- | --- | --- | --- | --- | --- | --- | --- | --- | --- | --- |
| -745.45 | | -516.86 | | -603.55 | | -598.11 | | -775.15 | | -448.88 | | -1699.94 | | -816.31 | |
|  | -26.04 | -279.28 | | -260.03 | |  | -261.1 | -612.11 | | -275.98 | | -2592.18 | | -359.28 | |
|  | -12.71 | -223.99 | | -240.64 | | -232.09 | | -485.72 | | -245.54 | | -2477.04 | | -293.85 | |
| NA |  | -135.67 | | -172.07 | | -165.37 | | -349.16 | | -140.75 | | -1788.97 | | -175.97 | |
|  | -42.63 |  | -48.14 |  | -65.78 |  | -76.92 | -214.35 | |  | -58.2 | -341.31 | | -159.68 | |
| -431.02 | |  | -60.1 | -151.77 | | -132.94 | |  | -294.8 | NA |  | -687.93 | | -236.56 | |
|  | -19.79 | -261.76 | | -278.16 | | -271.19 | | -604.53 | | -239.16 | | -2607.11 | | -306.18 | |
| NA |  | -138.43 | |  | -172.7 |  | -160.3 | -433.18 | | -106.91 | | -2490.83 | | -189.04 | |
|  | -73.02 | NA |  | NA |  | NA |  | NA |  | NA |  | NA |  |  | -72.52 |
|  | -99.02 | NA |  | NA |  | NA |  | NA |  | NA |  | NA |  |  | -94.07 |
|  | -13.72 | NA |  | NA |  | NA |  | NA |  | NA |  | NA |  | NA |  |
| -620.33 | |  | -859.2 | -725.02 | |  | -756.7 | -722.17 | | -713.09 | | -254.37 | | -562.82 | |
| -406.73 | | -296.66 | | -291.89 | | -275.79 | | -342.66 | | -221.19 | | -1632.36 | |  | -380.9 |
| NA |  |  | -9.83 |  | -32.45 |  | -28.83 |  | -71 | NA |  | -624.65 | |  | -6.21 |
| -167.02 | | -193.47 | | -152.15 | | -160.06 | |  | -99.01 | -106.83 | | -1395.27 | | -189.46 | |
| NA |  | NA |  |  | -25.49 |  | -20.44 |  | -55.71 | NA |  | -515.47 | | NA |  |
|  | -14.85 |  | -34.68 | NA |  |  | -6.98 | NA |  | NA |  |  | -574.3 | NA |  |
| -248.34 | |  | -97.33 | -113.02 | |  | -111.8 | -105.86 | |  | -30.8 | -844.01 | |  | -86.93 |
| NA |  | NA |  | NA |  | NA |  | NA |  | NA |  | NA |  |  | -16.84 |
| -811.24 | | -526.55 | | -659.11 | | -641.51 | | -502.18 | | -469.53 | | -1142.33 | | -702.19 | |
|  | -28.11 |  | -31.71 |  | -47.54 |  | -46.1 |  | -188.7 |  | -32.76 |  | -432.3 |  | -136.2 |
|  | -86.11 | NA |  | NA |  | NA |  |  | -12.82 | NA |  | -1355.7 | | NA |  |
|  | -99.29 | NA |  | NA |  | NA |  |  | -69.09 | NA |  | -1658.96 | | NA |  |
| -427.43 | | -210.36 | | -172.02 | | -157.34 | |  | -49.13 |  | -73.3 | -966.09 | | -137.92 | |
| -376.14 | | -351.66 | |  | -87.75 | -110.23 | |  | -45.37 | -191.09 | | -183.77 | | -205.45 | |
| -499.51 | |  | -276.4 | -189.58 | | -180.57 | |  | -34.47 | -173.14 | | -389.92 | | -223.71 | |
| -324.96 | |  | -183.6 | -215.41 | |  | -203.5 | NA |  |  | -77.65 | -955.96 | | -165.75 | |
| -120.35 | | -177.25 | |  | -30.62 |  | -45.81 |  | -11.66 |  | -70.81 |  | -107.1 |  | -31.54 |
| -222.81 | | -338.88 | | -310.11 | | -309.32 | | -348.71 | | -272.09 | | -700.74 | | -306.81 | |
| -247.13 | | -521.65 | | -153.71 | | -163.93 | | -242.26 | | -368.93 | | -373.76 | | -203.92 | |
| -478.13 | | -344.91 | | -448.21 | | -451.96 | | -262.95 | | -330.66 | | -1056.25 | | -484.96 | |
| -112.42 | | -207.29 | | -178.01 | | -176.89 | | -101.13 | | -170.66 | | -560.09 | | -150.57 | |
| -111.43 | | -172.92 | | -144.89 | | -156.26 | | -207.25 | | -205.44 | | -644.54 | | -226.55 | |
| -140.84 | | -137.44 | |  | -67.36 |  | -59.9 |  | -21.08 |  | -44.28 |  | -17.14 |  | -40.08 |
|  | -62.32 | -459.84 | | -334.09 | | -347.09 | | -124.61 | | -589.97 | |  | -27.32 | -162.63 | |
|  | -34.09 | -1071.81 | | -834.17 | | -835.81 | | -154.51 | | -1370.33 | |  | -69.52 | -185.27 | |
|  | -174.5 | -128.59 | |  | -93.84 |  | -99.51 |  | -91.74 |  | -89.57 | -380.26 | | -172.08 | |
| -376.59 | | -223.94 | | -306.75 | | -311.47 | | -391.65 | | -282.63 | | -415.55 | | -500.96 | |

| -178.72 | | -212.57 | | -238.59 | | -215.29 | |  | -39.99 | -160.72 | | -409.17 | | -165.91 | |
| --- | --- | --- | --- | --- | --- | --- | --- | --- | --- | --- | --- | --- | --- | --- | --- |
| -345.18 | | -475.97 | | -340.83 | | -343.38 | | -139.23 | | -395.51 | | -512.45 | | -262.93 | |
| -200.26 | | -369.73 | | -390.92 | | -384.46 | | -818.98 | | -415.21 | | -1000.77 | | -531.05 | |
| -104.33 | |  | -75.16 |  | -53.25 |  | -51.88 |  | -11.71 |  | -44.23 |  | -441.5 |  | -76.79 |
| -118.86 | | -1473.37 | | -1156.44 | | -1091.03 | | -1295.06 | | -1497.91 | | -161.91 | | -818.15 | |
| NA |  | NA |  | NA |  | NA |  | -191.59 | | NA |  |  | -640.3 |  | -193.6 |
| -411.08 | | -469.88 | | -507.51 | | -515.97 | | -466.59 | | -513.74 | | -808.78 | | -523.77 | |
| -280.53 | | -100.74 | | -182.94 | | -174.67 | | -182.01 | |  | -76.61 | -844.01 | | -274.69 | |
| -363.27 | | -235.36 | | -247.51 | | -241.64 | | -181.12 | | -131.68 | | -887.56 | | -255.33 | |
|  | -99.39 | -132.87 | | -108.81 | |  | -116.9 |  | -62.68 | -154.54 | | -193.48 | | -105.66 | |
| -343.32 | | -267.94 | | -130.93 | | -134.67 | |  | -90.6 | -170.57 | | -451.05 | | -192.73 | |
| NA |  | NA |  | NA |  | NA |  | -507.14 | | NA |  | NA |  |  | -27.22 |
|  | -92.64 | -242.48 | | -292.94 | | -270.82 | |  | -32.42 | -234.75 | | -540.12 | |  | -51.92 |
| -177.68 | | -148.09 | |  | -12.78 |  | -18.5 | -100.31 | |  | -45.11 | -121.76 | |  | -40.52 |
| -203.25 | | -284.53 | |  | -67.08 |  | -86.09 | -171.74 | | -124.69 | | -428.03 | |  | -94.13 |
| -1006.05 | | -586.78 | | -725.87 | | -738.45 | | -551.49 | | -568.99 | | -1493.97 | | -886.84 | |
| -163.66 | |  | -23.88 |  | -31.59 |  | -31.71 | -123.69 | | NA |  | -638.37 | |  | -81.86 |
| -424.25 | | -164.44 | | -259.94 | | -270.25 | | -206.17 | | -118.15 | | -1088.58 | | -256.18 | |
| NA |  | NA |  | NA |  | NA |  |  | -5.79 | NA |  | -430.41 | |  | -16.18 |
|  | -52.22 | -146.55 | | -111.85 | | -114.93 | |  | -30.58 | -140.31 | | -293.65 | |  | -57.28 |
| -329.06 | | -399.64 | | -145.42 | | -138.65 | | -166.71 | | -350.09 | | -363.88 | | -246.14 | |
| NA |  | -282.36 | |  | -58.09 |  | -60.01 | NA |  | -338.24 | | NA |  | NA |  |
| -283.78 | | -132.42 | | -238.49 | | -223.76 | | -163.97 | |  | -96.45 | -1159.9 | | -175.23 | |
|  | -44.11 | -729.48 | | -325.76 | |  | -339.3 |  | -13.76 | -629.35 | | NA |  |  | -35.87 |
| -342.93 | | -181.77 | |  | -187.4 | -185.06 | |  | -279.3 |  | -82.14 |  | -997.1 | -257.46 | |
|  | -16.57 | -441.56 | | -298.85 | | -298.63 | |  | -78.59 | -559.79 | |  | -74.67 |  | -60.23 |
| -271.59 | | -284.72 | | -236.32 | | -238.12 | | -280.22 | | -263.01 | | -389.59 | |  | -333.1 |
| -313.94 | | -312.51 | |  | -50.68 |  | -62.19 | -163.61 | | -170.67 | | -103.61 | | -167.94 | |
| -172.74 | |  | -88.89 | -145.97 | | -142.06 | | -156.69 | | -109.46 | | -456.96 | | -191.55 | |
|  | -45.65 |  | -80.36 |  | -57.83 |  | -62.76 |  | -61.42 |  | -85.59 | -245.01 | | -108.32 | |
|  | -55.17 | -617.59 | | -254.39 | | -269.83 | |  | -62.51 | -677.17 | |  | -86.39 |  | -80.87 |
|  | -577.5 | -372.12 | | -273.41 | | -270.73 | | -561.62 | |  | -241.4 | -801.01 | | -409.66 | |
| -243.51 | | -245.83 | | -120.45 | | -145.07 | |  | -60.94 | -115.21 | |  | -637.7 | -132.43 | |
| NA |  |  | -237.2 | -152.73 | | -169.73 | |  | -88.33 | -300.27 | |  | -70.99 |  | -82.35 |
|  | -94.47 |  | -14.08 |  | -17.32 |  | -9.3 | -110.08 | | NA |  | -159.34 | | NA |  |
| -194.32 | |  | -83.31 | -120.75 | | -116.52 | |  | -189 | -102.35 | | -692.61 | | -257.12 | |
|  | -78.77 | -176.28 | | -170.04 | | -172.44 | | -103.94 | | -204.87 | | -226.73 | |  | -56.41 |
| -339.68 | | -448.85 | |  | -90.84 | -113.35 | |  | -47.97 | -300.59 | |  | -136.6 | -182.84 | |

preadipoc precursor_ precursor_ regulatory segmente sigmoid_c t_cell..cee thyroid..ce

-225.01	-239.33	-125.94	-149.58	-182.28	-31.7	-146.27	-156.15

NA	NA	NA	NA	NA	NA	NA	NA

-359.73	-337.5	-91.33	-304.3	-219.19	-197.04	-256.13	-401.43

-257.91	-208.46	-159.18	-198.11	-383.54	-37.95	-131.3	-456.19

-5.52	-45.4	-18.9	-97.47	-74.81 NA	-37.97	-260.92

-1248.89	-1362.04	-584.75	-593.8	-771.72	-638.37	-436.52	-355.99

-245.16	-289.09	-81.18	-297.85	-278.92	-104.51	-213.23	-216.25

-90.54	-77.55	-33.24	-101.56	-54.81 NA	-46.35	-108

-90.54	-77.55	-33.24	-101.56	-54.81 NA	-46.35	-108

-512.73	-12.22 NA	-11.21	-121.69	-47.4	-11.66	-5.75

-1310.63	-746.95	-289.52	-277.32	-439.86	-437.42	-207.04	-145.98

-165.01	-377.26	-163.44	-182.28	-180.27	-181.44	-94.23	-32.63

-468.57	-54.68	-10.24	-450.78	-245.54	-77.29	-188.96	-55.82

-893.66	-135.6	-40	-561.91	-241.8	-168.03	-264.56	-137.47

NA	NA	NA	NA	NA	-23.27 NA	NA NA	NA	NA	NA	NA	-9.61 NA	NA

-166.03	-19.96 NA	-238.87 NA	-100.9	-179.68	-26.33

-166.03	-19.96 NA	-238.87 NA	-100.9	-179.68	-26.33

-189.08	-2767.29	-380.47	-989.14	-985.78	-455.34	-1746.68	-65.64

-145.55	-325.84	-78.79	-371.92	-248.37	-155.85	-260.12	-378.38

NA	NA	NA	NA	NA	-14.22 NA	NA

-169.76	-2134.05	-596.73	-1309.98	-1018.48	-179.36	-2233.09	-84.7

NA	NA	NA	NA	NA	-591.62 NA	NA NA	NA	NA	NA	NA	-1254.05 NA	NA

-43.73 NA	NA	NA	-349.85	-37.35	-5.24 NA

-18.51 NA	NA	NA	-1316.23 NA	NA	NA

-30.76 NA	NA	NA	-1375.26 NA	NA	NA NA	NA	NA	NA	-928.65 NA	NA	NA

-376.03	-373.41	-79.71	-327.29	-205.15	-255.97	-299.27	-333.67

-376.03	-373.41	-79.71	-327.29	-205.15	-255.97	-299.27	-333.67

-405.67	-474.47	-161.18	-471.89	-395.99	-264.32	-376.26	-432.88

-454.94	-531.75	-151.2	-481.95	-400.73	-297.88	-396.55	-428.67

-485.94	-12.31 NA	-10.59	-136.3	-59.42	-21.87 NA

-63.59 NA	NA	NA	-287.22	-42.98 NA	NA NA	NA	NA	NA	NA	NA	NA	NA NA	NA	NA	NA	NA	NA	NA	NA NA	NA	NA	NA	NA	NA	NA	NA

-166.7	-146.1	-155.29	-70.42	-145.63 NA	-52.07	-66.83

| -1070.4 | | -1807.66 | | -429.78 | |  | -28.76 |  | -75.24 |  | -12.3 | NA |  | -109.6 |
| --- | --- | --- | --- | --- | --- | --- | --- | --- | --- | --- | --- | --- | --- | --- |
| -1070.4 | | -1807.66 | | -429.78 | |  | -28.76 |  | -75.24 |  | -12.3 | NA |  | -109.6 |
| -1070.4 | | -1807.66 | | -429.78 | |  | -28.76 |  | -75.24 |  | -12.3 | NA |  | -109.6 |
|  | -465.6 | -162.17 | |  | -35.87 | -303.88 | |  | -33.27 | NA |  | -274.09 |  | -85.17 |
| -345.15 | | -135.89 | |  | -38.34 | -219.88 | |  | -47.33 | NA |  | -128.69 |  | -63.14 |
| -209.99 | | -4579.22 | | -1022.37 | | -1682.91 | | -2532.91 | | -701.94 | | -2774.93 |  | -81.12 |
| -457.35 | | -5404.32 | | -1383.69 | | -2474.86 | | -3715.7 | | -1790.67 | | -3899.98 | -215.77 | |
| -589.24 | | -7761.31 | | -2033.56 | | -3558.56 | | -5312.41 | | -1976.25 | | -5520.1 | -282.43 | |
| -643.02 | | -5957.22 | | -1388.6 | | -2464.01 | | -3232.34 | | -1350.73 | | -3860.85 | -211.94 | |
| -316.02 | | -3390.12 | |  | -932.7 | -1704.35 | | -2531.13 | | -1458.97 | | -2462.98 | -203.62 | |
| -310.38 | | -4423.84 | | -1298.71 | | -2123.75 | | -3321.01 | | -1300.95 | | -3118.22 | -221.72 | |
| -394.67 | | -7000.47 | | -1618.1 | | -2775.06 | | -4643.14 | | -2247.07 | | -4775.24 | -160.32 | |
| -741.37 | | -4589.26 | | -1496.56 | | -2439.13 | | -3627.22 | | -1239.79 | | -3476.59 | -443.63 | |
| -700.37 | | -5679.31 | | -1682.6 | | -3272.69 | | -4129.52 | | -1511.56 | | -4650.08 |  | -284.3 |
| -792.52 | | -6774.88 | | -2073.61 | | -3617.15 | | -4935.05 | | -1709.52 | | -5309.41 | -386.19 | |
| NA |  | NA |  | NA |  | NA |  | NA |  |  | -19.67 | NA | NA |  |
| -120.19 | | -120.22 | |  | -10.51 | -199.82 | |  | -95.55 |  | -30.25 | -125.27 | -140.25 | |
| -686.69 | | -6783.54 | | -2051.2 | | -3723.45 | | -4739.8 | | -1546.3 | | -5628.25 | -242.61 | |
| -755.52 | | -7897.62 | | -2382.26 | | -4478.85 | | -5450.4 | | -1716.79 | | -6550.7 | -293.06 | |
| -160.89 | | -511.63 | | -208.68 | | -295.96 | | -450.13 | |  | -116.1 | -366.58 |  | -69.37 |
| -606.34 | | -10260.2 | | -2650.21 | | -4882.92 | | -6418.5 | | -2119.15 | | -7601.6 | -244.94 | |
| NA |  | NA |  | NA |  | NA |  | NA |  |  | -16.17 | NA | NA |  |
| NA |  | NA |  | NA |  | NA |  | NA |  |  | -9.71 | NA | NA |  |
| -671.69 | | -8862.99 | | -2450.22 | | -4796.18 | | -5870.28 | | -2054.6 | | -7198.34 | -238.32 | |
| -109.78 | |  | -301 | -100.98 | | -162.24 | | -229.24 | |  | -70.17 | -195.94 |  | -25.13 |
| -383.27 | | -4277.46 | | -984.06 | | -1860.31 | | -2856.16 | | -845.09 | | -2923.49 | -116.47 | |
| -759.54 | | -8183.81 | | -2446.25 | | -4434.14 | | -5742.88 | | -1800.11 | | -6517.66 | -344.48 | |
| -538.06 | | -6118.38 | | -1753.7 | | -3418.95 | | -4090.24 | | -1426.81 | | -5372.83 | -219.88 | |
| -393.43 | | -2337.38 | | -799.19 | | -1519.49 | | -1890.35 | | -677.05 | | -2072.72 | -262.41 | |
| -393.43 | | -2337.38 | | -799.19 | | -1519.49 | | -1890.35 | | -677.05 | | -2072.72 | -262.41 | |
| -671.11 | | -9263.15 | | -2503.78 | | -4753.75 | | -6078.25 | | -1931.68 | | -7056.5 | -276.59 | |
| -453.22 | | -3766.94 | | -1193.08 | | -2013.51 | | -2766.88 | | -638.66 | | -2761.8 | -117.24 | |
|  | -88.46 | -2442.16 | | -617.89 | |  | -848.9 | -2054.45 | | -237.83 | | -1305.79 |  | -46.7 |
| -253.19 | | -2773.81 | |  | -713.8 | -1513.66 | | -1770.37 | | -737.38 | | -2194.88 | -104.18 | |
| NA |  | NA |  | NA |  | NA |  | NA |  | NA |  | NA | NA |  |
| -211.71 | | -408.38 | |  | -167.8 |  | -213 |  | -199.8 | -194.85 | | -125.85 |  | -68.09 |
| -557.69 | | -8474.76 | | -2094.9 | | -4461.34 | | -5351.46 | | -1992.97 | | -7066.78 | -182.22 | |
| -700.24 | | -9960.72 | | -2766.9 | | -5071.48 | | -6610.96 | | -2116.12 | | -7760.38 | -282.02 | |
| -177.37 | | -554.99 | | -229.63 | | -358.95 | | -503.38 | | -124.64 | | -438.74 |  | -65.96 |

| -954.14 | |  | -76.31 | NA |  | -594.27 | | -105.16 | |  | -200.1 | -323.01 | |  | -31.51 |
| --- | --- | --- | --- | --- | --- | --- | --- | --- | --- | --- | --- | --- | --- | --- | --- |
| -882.82 | | -113.66 | | NA |  | -477.73 | |  | -99.74 | -247.16 | | -352.15 | |  | -49.09 |
| -1020.88 | | -204.72 | |  | -22.73 | -712.96 | | -233.67 | | -220.73 | |  | -417.8 | -122.16 | |
| -506.56 | | NA |  | NA |  | -412.15 | |  | -45.63 |  | -95.62 | -130.93 | | NA |  |
| NA |  | NA |  | NA |  | NA |  | NA |  | -1239.57 | | NA |  | -413.48 | |
| NA |  | NA |  | NA |  | NA |  | NA |  | -580.97 | | NA |  | -209.97 | |
| NA |  | NA |  | NA |  | NA |  | NA |  | -884.59 | | NA |  | -662.97 | |
| NA |  | NA |  | NA |  | NA |  | NA |  | -621.51 | | NA |  | -153.24 | |
|  | -72.8 | NA |  | NA |  | NA |  | NA |  | -1343.84 | | NA |  | -227.33 | |
|  | -90.08 | NA |  | NA |  | NA |  | NA |  | -1223.74 | | NA |  | -197.88 | |
| NA |  | NA |  | NA |  | NA |  | NA |  | -449.67 | | NA |  | -140.54 | |
| NA |  | NA |  | NA |  | NA |  | NA |  | -449.67 | | NA |  | -140.54 | |
| NA |  | NA |  | NA |  | NA |  | NA |  | -449.67 | | NA |  | -140.54 | |
| NA |  | NA |  | NA |  | NA |  | NA |  | -921.37 | | NA |  | -167.15 | |
|  | -94.07 | NA |  | NA |  | NA |  | NA |  | -1234.26 | |  | -5.52 | -221.59 | |
| -168.91 | | NA |  | NA |  | NA |  | NA |  | -1211.21 | |  | -30.46 | -278.71 | |
|  | -33.96 | NA |  | NA |  | NA |  | NA |  | -618.98 | | NA |  | -339.42 | |
|  | -55.16 | NA |  | NA |  | NA |  | NA |  | -654.74 | | NA |  | -314.95 | |
| -227.86 | |  | -41.88 | NA |  |  | -15.67 | NA |  | -1138.78 | |  | -50.52 | -271.23 | |
| -112.65 | | NA |  | NA |  | NA |  | NA |  | -1312.3 | |  | -9.03 | -243.66 | |
| NA |  | NA |  | NA |  | NA |  | NA |  | -379.64 | | NA |  |  | -37.29 |
| -744.93 | | -7263.65 | | -2210.22 | | -3773.12 | | -5213.63 | | -1639.25 | | -5404.7 | | -385.82 | |
| -157.39 | |  | -51.81 | NA |  |  | -56 |  | -25.64 | -159.98 | | -155.44 | | NA |  |
| -157.39 | |  | -51.81 | NA |  |  | -56 |  | -25.64 | -159.98 | | -155.44 | | NA |  |
| NA |  | NA |  | NA |  | NA |  | NA |  |  | -33.24 |  | -22.49 | NA |  |
| NA |  | NA |  | NA |  | NA |  | NA |  |  | -33.24 |  | -22.49 | NA |  |
|  | -22.94 | NA |  | NA |  | NA |  | NA |  | -180.25 | |  | -34.67 | NA |  |
|  | -73.92 |  | -9.23 | NA |  |  | -37.1 |  | -18.95 | -405.09 | | -141.14 | | NA |  |
| NA |  | NA |  | NA |  | NA |  | NA |  |  | -81.57 | NA |  | NA |  |
| NA |  | NA |  | NA |  | NA |  | NA |  |  | -75.81 | NA |  | NA |  |
| NA |  | NA |  | NA |  | NA |  | NA |  |  | -75.81 | NA |  | NA |  |
| NA |  | NA |  | NA |  | NA |  | NA |  |  | -75.81 | NA |  | NA |  |
| NA |  | NA |  | NA |  | NA |  | NA |  | NA |  | NA |  | NA |  |
| NA |  | NA |  | NA |  | NA |  | NA |  |  | -7.64 | NA |  | NA |  |
| NA |  | NA |  | NA |  | NA |  | NA |  | NA |  | NA |  | NA |  |
| NA |  | NA |  | NA |  | NA |  | NA |  | NA |  | NA |  | NA |  |
| -562.06 | |  | -59.26 |  | -35.02 |  | -65.69 |  | -73.86 |  | -94.7 |  | -19.87 | -133.22 | |
| -140.79 | | -124.65 | |  | -86.55 | -145.52 | | -220.75 | |  | -6.28 |  | -36.42 | -275.56 | |
| -140.79 | | -124.65 | |  | -86.55 | -145.52 | | -220.75 | |  | -6.28 |  | -36.42 | -275.56 | |

| -114.52 | | -170.44 | | -140.96 | |  | -272.5 | -320.66 | |  | -37.56 |  | -83 | -447.89 | |
| --- | --- | --- | --- | --- | --- | --- | --- | --- | --- | --- | --- | --- | --- | --- | --- |
| NA |  | NA |  | NA |  | NA |  |  | -6.01 | NA |  | NA |  |  | -59.75 |
|  | -73.12 | -273.15 | |  | -137.1 | -183.62 | | -213.12 | |  | -40.86 | -136.61 | | -272.24 | |
| NA |  |  | -15.49 |  | -28.29 |  | -62.61 | -101.31 | | NA |  | NA |  |  | -71.19 |
| -353.49 | | -309.38 | | -130.92 | | -262.48 | | -338.04 | |  | -64.98 | -178.21 | | -179.73 | |
|  | -9.38 | NA |  | NA |  | NA |  | -419.77 | |  | -8.46 | NA |  | NA |  |
| NA |  | NA |  | NA |  | NA |  | NA |  |  | -61.02 | NA |  | NA |  |
| NA |  | NA |  | NA |  | NA |  | NA |  |  | -68.82 | NA |  | NA |  |
|  | -205.8 | NA |  | NA |  |  | -28.51 |  | -15.22 | -635.91 | | NA |  |  | -99.07 |
|  | -153.2 |  | -7.43 | NA |  |  | -13.54 |  | -8.54 | -645.86 | | NA |  |  | -53.1 |
| -348.09 | |  | -47.22 |  | -26.3 |  | -61.83 |  | -82.72 | -629.68 | |  | -7.43 | -138.24 | |
| NA |  | NA |  | NA |  | NA |  | NA |  | NA |  | NA |  | NA |  |
| NA |  | NA |  | NA |  | NA |  | NA |  | -980.78 | | NA |  | NA |  |
| NA |  | NA |  | NA |  | NA |  | NA |  | -544.49 | | NA |  | NA |  |
| NA |  | NA |  | NA |  | NA |  | NA |  | -685.87 | | NA |  | NA |  |
| NA |  | NA |  | NA |  | NA |  | NA |  | -232.26 | | NA |  | NA |  |
| NA |  | NA |  | NA |  | NA |  | NA |  | NA |  | NA |  | NA |  |
|  | -21.12 | NA |  | NA |  | NA |  | NA |  |  | -13.13 | NA |  | NA |  |
| NA |  | NA |  | NA |  | NA |  | NA |  | NA |  | NA |  | NA |  |
| -150.87 | | NA |  | NA |  | NA |  | NA |  |  | -415.8 |  | -7.79 | NA |  |
| NA |  | NA |  | NA |  | NA |  | NA |  | -586.81 | | NA |  | NA |  |
|  | -15.87 | NA |  | NA |  | NA |  | NA |  |  | -31.44 | NA |  | NA |  |
| NA |  | NA |  | NA |  | NA |  | NA |  |  | -7.64 | NA |  | NA |  |
|  | -8.41 | NA |  | NA |  | NA |  | NA |  | NA |  | NA |  | NA |  |
| NA |  | NA |  | NA |  | NA |  | NA |  | -1158.31 | | NA |  | NA |  |
| -125.25 | | NA |  | NA |  | NA |  | NA |  | -695.48 | |  | -16.47 | NA |  |
| NA |  | NA |  | NA |  | NA |  | NA |  | -864.75 | | NA |  | NA |  |
| NA |  | NA |  | NA |  | NA |  | NA |  | -617.03 | | NA |  | NA |  |
| NA |  | NA |  | NA |  | NA |  | NA |  | NA |  | NA |  | NA |  |
| NA |  | NA |  | NA |  | NA |  | NA |  | NA |  | NA |  | NA |  |
| -378.63 | | NA |  | NA |  |  | -10.78 | NA |  |  | -82.21 |  | -15.93 | NA |  |
| NA |  | NA |  | NA |  | NA |  | NA |  | NA |  | NA |  | NA |  |
|  | -14.95 | NA |  | NA |  | NA |  | NA |  |  | -44.76 | NA |  | NA |  |
| NA |  | NA |  | NA |  | NA |  | NA |  |  | -5.73 | NA |  | NA |  |
| NA |  | NA |  | NA |  | NA |  | NA |  | -1065.5 | | NA |  | NA |  |
|  | -7.04 | NA |  | NA |  | NA |  | NA |  | -966.11 | | NA |  | NA |  |
| NA |  | NA |  | NA |  | NA |  | NA |  | -1033.46 | | NA |  | NA |  |
| NA |  | NA |  | NA |  | NA |  | NA |  | -1043.18 | | NA |  | NA |  |
| NA |  | NA |  | NA |  | NA |  | NA |  |  | -542.2 | NA |  | NA |  |

| -6.74 NA | NA | NA | NA |
| --- | --- | --- | --- |
| -183.48 NA | NA | NA | NA |

| NA NA NA NA NA | | | | | NA NA | NA |
| --- | --- | --- | --- | --- | --- | --- |
|  |  |  |  |  | -460.25 | -15.73 NA |
|  |  |  |  |  | NA NA | NA |
| NA | NA | NA | NA | NA | -506.89 NA | NA |
| NA | NA | NA | NA | NA | -611.6 NA | NA |
|  | -6.72 NA | NA | NA | NA | -1060.74 | -22.65 NA |
| NA | NA | NA | NA | NA | -814.17 NA | NA |
| NA | NA | NA | NA | NA | -747.44 NA | NA |
| NA | NA | NA | NA | NA | NA NA | NA |
|  | -11.95 NA | NA | NA | NA | -1203.61 | -10.83 NA |

N

| -153.83 -1388.12 -187.74 -446.5 | | | | -617.29 | -248.07 | -905.25 | -19.46 |
| --- | --- | --- | --- | --- | --- | --- | --- |
| -18.67 -31.75 NA -28.34 | | | | -12.19 | -350.31 | -325.48 | -499.63 |
| A -125.01 NA -67.81 N | | | | A | -25.07 | -316.88 | -12.83 |
| -43.62 -753.14 -48.04 -380.33 | | | | -125.9 | -170.53 | -907.11 | -75.46 |
| -144.76 | -448.64 | -95.75 | -511.67 | -110.02 | -268.09 | -702.7 | -73.28 |
| -98.67 | -257.92 | -40.9 | -364.54 | -79.97 | -96.72 | -496.12 | -63.3 |

NA	-42.29 NA	-94.67	-35.28 NA	-59.76	-45.31

-38.65	-117.15 NA	-101.1 NA	-93.19	-326.16	-23.73

-202.23	-793.03	-119.65	-672.87	-206.46	-285.84	-1042.56	-83.14

-11.12	-182.23 NA	-223.38	-14.79	-124.77	-435.46	-29.85

-758.48	-33.94 NA	-436.81	-36.96	-209.58	-230.55	-22.68

-754.69	-51.01 NA	-432.49	-101.16	-139.64	-200.5	-38.94

-888.62	-95.01	-7.12	-654.3	-163.64	-220.22	-367.4	-40.4

-422.55 NA	NA	-374.45	-9.76	-105.76	-141.9 NA

-151.27	-28.29 NA	-26.49 NA	-225.97	-29.45 NA

-112.41	-147.19	-30.74	-59.53	-62.05	-6.37	-95.75	-64.05

-110.89	-30	-8.85	-51.52	-48.16	-204.96	-24.22	-15.09

-52.92	-38.75	-20.6	-57.5	-33.49	-77.44	-34.22	-16.29

-52.92	-38.75	-20.6	-57.5	-33.49	-77.44	-34.22	-16.29

-59.56	-23.33 NA	-31.12 NA	-16.23	-13.82 NA

-222.78	-165.9	-30.21	-108.01	-13.63	-45.44	-90.7 NA

-139.41	-27.59 NA	-28.88 NA	-192.11	-21.84 NA

-139.41	-27.59 NA	-28.88 NA	-192.11	-21.84 NA

-139.41	-27.59 NA	-28.88 NA	-192.11	-21.84 NA

-408.4	-188.05	-31.45	-85.41	-31.72	-115.66	-97.2	-9.6

-383.58	-299.82	-193.68	-135.12	-176.43 NA	-75.61	-62.39

-47.36 NA	NA	-17.52 NA	-128.29	-5.27 NA NA	NA	NA	NA	NA	-10.18 NA	NA

NA	-91.67 NA	-129.79 NA	-157.1	-313.84	-35.21

| NA |  | NA |  | NA |  | NA |  | NA |  |  | -10.18 | NA |  | NA |  |
| --- | --- | --- | --- | --- | --- | --- | --- | --- | --- | --- | --- | --- | --- | --- | --- |
| NA |  | NA |  | NA |  | NA |  | NA |  | NA |  | NA |  | NA |  |
| NA |  | NA |  | NA |  | NA |  | NA |  | NA |  | NA |  | NA |  |
| NA |  | NA |  | NA |  | NA |  | NA |  | NA |  | NA |  | NA |  |
| -866.65 | |  | -802 | -385.07 | | -309.36 | | -476.07 | | -213.23 | | -176.34 | | -193.05 | |
| -767.21 | | -209.01 | | -154.63 | | -361.84 | | -333.17 | | -151.56 | | -217.18 | | -238.89 | |
| -314.54 | | -118.74 | |  | -71.31 | -150.56 | | -178.01 | |  | -15.63 |  | -42.84 | -160.04 | |
| -954.67 | | -459.37 | | -266.76 | | -420.92 | | -493.28 | | -233.22 | | -374.98 | | -316.75 | |
|  | -92.05 |  | -27.77 |  | -36.42 |  | -94.57 | -201.55 | | NA |  |  | -10.63 |  | -61.06 |
|  | -232.5 |  | -64.77 |  | -58.56 | -144.37 | | -253.46 | |  | -8.25 |  | -55.77 | -135.54 | |
| -234.87 | | -368.37 | | -100.87 | | -375.34 | | -270.11 | | -164.26 | | -263.15 | |  | -197.8 |
| -660.57 | | -112.81 | | -224.92 | | -106.52 | | -305.97 | |  | -13.06 |  | -35.24 | -234.51 | |
|  | -1118 |  | -302.8 | -292.78 | | -169.42 | | -598.95 | | -127.94 | | -142.81 | | -572.84 | |
|  | -45.58 | -106.11 | |  | -30.42 | -114.07 | |  | -53.85 | -160.77 | |  | -80.66 |  | -43.42 |
|  | -14.44 | NA |  | NA |  | NA |  | NA |  |  | -26.72 | NA |  | NA |  |
| NA |  | NA |  | NA |  | NA |  | NA |  | NA |  | NA |  | NA |  |
|  | -23.4 | -216.56 | |  | -43.94 |  | -294.1 | -155.45 | | -111.38 | | -196.59 | | -181.79 | |
| NA |  | NA |  | NA |  | NA |  | NA |  | NA |  | NA |  | NA |  |
|  | -547.4 |  | -665.1 | -246.21 | | -331.09 | | -308.82 | |  | -504.6 | -251.48 | | -289.76 | |
| NA |  | NA |  | NA |  | NA |  | NA |  |  | -55.83 | NA |  | NA |  |
| NA |  | NA |  | NA |  | NA |  | NA |  |  | -55.83 | NA |  | NA |  |
| -201.87 | | -171.67 | | -101.34 | | -171.52 | | -209.96 | | -133.19 | | -153.13 | | -239.92 | |
|  | -303.8 |  | -364.1 |  | -94.68 | -273.91 | |  | -265.9 | -166.58 | | -262.91 | | -209.83 | |
|  | -303.8 |  | -364.1 |  | -94.68 | -273.91 | |  | -265.9 | -166.58 | | -262.91 | | -209.83 | |
|  | -303.8 |  | -364.1 |  | -94.68 | -273.91 | |  | -265.9 | -166.58 | | -262.91 | | -209.83 | |
|  | -303.8 |  | -364.1 |  | -94.68 | -273.91 | |  | -265.9 | -166.58 | | -262.91 | | -209.83 | |
| -416.19 | | -506.06 | | -174.81 | | -286.83 | | -367.32 | | -258.01 | | -323.19 | | -234.57 | |
| -416.19 | | -506.06 | | -174.81 | | -286.83 | | -367.32 | | -258.01 | | -323.19 | | -234.57 | |
| -416.19 | | -506.06 | | -174.81 | | -286.83 | | -367.32 | | -258.01 | | -323.19 | | -234.57 | |
| -416.19 | | -506.06 | | -174.81 | | -286.83 | | -367.32 | | -258.01 | | -323.19 | | -234.57 | |
| -422.84 | | -278.78 | |  | -57.9 | -204.02 | | -138.89 | | -556.87 | | -339.62 | | -297.93 | |
| -422.84 | | -278.78 | |  | -57.9 | -204.02 | | -138.89 | | -556.87 | | -339.62 | | -297.93 | |
| -186.96 | | -189.08 | |  | -34.48 | -137.91 | |  | -83.18 | -309.86 | | -203.23 | | -136.74 | |
| -186.96 | | -189.08 | |  | -34.48 | -137.91 | |  | -83.18 | -309.86 | | -203.23 | | -136.74 | |
| -569.12 | | -593.88 | |  | -193.8 | -623.74 | |  | -462.7 | -254.33 | | -501.06 | | -314.97 | |
| -703.68 | | -987.37 | | -390.91 | | -371.75 | | -400.74 | | -495.61 | | -207.08 | | -188.86 | |
| -703.68 | | -987.37 | | -390.91 | | -371.75 | | -400.74 | | -495.61 | | -207.08 | | -188.86 | |
| -523.15 | | -728.56 | | -245.03 | | -339.08 | | -305.91 | | -583.27 | | -294.16 | |  | -328.9 |
| -523.15 | | -728.56 | | -245.03 | | -339.08 | | -305.91 | | -583.27 | | -294.16 | |  | -328.9 |

| -262.8 | -772.9 | -328.45 | -330.52 | -363.24 | -387.46 | -194.92 | -162.95 |
| --- | --- | --- | --- | --- | --- | --- | --- |
| -872.93 | -1189.15 | -494.01 | -503.63 | -573.04 | -521.73 | -320.13 | -325.5 |
| -569.24 | -569.34 | -244.9 | -261.56 | -285.03 | -287.51 | -134.7 | -92.93 |
| -182.54 | -240.37 | -47.2 | -62.68 | -89.02 | -86.75 | -46.81 | NA |
| -528.11 | -186.36 | -46.5 | -449.56 | -335.69 | -173.7 | -317.26 | -140.57 |
| -632.45 | -11.48 | NA | -358.6 | -26.76 | -182.08 | -173.71 | -22.19 |
| -436.66 | -181.06 | -69.76 | -419.77 | -459.22 | -106.04 | -225.78 | -150.34 |
| -2086.86 | -73.17 | -36.36 | -129.38 | -553.38 | -1209.41 | -54.96 | -2466.21 |
| -2517.92 | -100.54 | -55.5 | -141.15 | -640.82 | -1335.27 | -64.69 | -2774.08 |

-614.08 NA	NA	NA	-47.67	-71.82 NA	-316.93

-2817.82	-92.31	-50.08	-143.4	-601.65	-1451.45	-69.95	-2954.26

-1487.27	-99.51	-30.71	-108.74	-234.98	-536.44	-65.44	-1082.37

NA	-17.48	-8.63	-42.7 NA	NA	NA	NA

-229.15	-215.73	-102.95	-218.08	-131.42	-13.47	-112.35	-24.12

NA	NA	NA	-13.37 NA	NA	NA	NA

NA	NA	NA	NA	NA	NA	NA	-823.2

NA	NA	NA	NA	NA	NA	NA	-639.14

-19.28	-34.73 NA	-10.61 NA	-16.02	-19.81	-1807.03

NA	NA	NA	NA	NA	NA	NA	-1909.46

NA	NA	NA	NA	NA	NA	NA	-713.58

NA	NA	NA	NA	NA	NA	NA	-684.06

NA	NA	NA	NA	NA	NA	NA	-1551.83

NA	NA	NA	-5.62 NA	NA	NA	-1722.47

NA	-50.32 NA	-30.07 NA	-30.98	-72.53	-772.36

NA	-173.7	-9.92	-74.19	-63.13	-34.35	-151.8	-663.85

NA	NA	NA	NA	NA	NA	NA	NA NA	NA	NA	NA	NA	NA	NA	NA NA	NA	NA	NA	NA	-15.13 NA	NA

-529.15	-615.89	-141.45	-569.79	-350.06	-296.37	-493.53	-347.64

-563.67	-369.45	-88.12	-130.27	-115.5	-13.69	-164.86	-133.35

-563.67	-369.45	-88.12	-130.27	-115.5	-13.69	-164.86	-133.35

NA	NA	NA	NA	NA	-111.34 NA	-16.42

-7.25 NA	NA	-5.41	-15.29 NA	NA	-48.45

-28.59 NA	NA	NA	NA	-35.89 NA	-8.66

-41.2	-7.98 NA	-21.8 NA	-191.83 NA	-14.06

-207.13	-831.13	-420.94	-465.94	-1041.57	-142.64	-517.65	-128.15

-115.93	-6.05 NA	-24.57	-13.18	-379.69 NA	-28.74

NA	NA	NA	NA	NA	NA	NA	NA

-100.82 NA	NA	-14.15	-14.02	-292.45 NA	-34.2

-61.82	-20.18 NA	-27.16 NA	-243.87 NA	-54.85

-65.04 NA	NA	NA	NA	-137.26 NA	-15.69

-33.4 NA	NA	NA	NA	-148.79 NA	-27.06

-8.31 NA	NA	-14.61 NA	-107.72 NA	-7.7

-18.01 NA	NA	NA	-5.87	-43.35 NA	-15.41

-866.58	-198.29	-139.43	-375.15	-342.09	-197.99	-273.28	-275.71

NA	NA	NA	NA	NA	NA	NA	NA NA	NA	NA	NA	NA	NA	NA	NA NA	NA	NA	NA	NA	NA	NA	NA NA	NA	NA	NA	NA	NA	NA	NA

-40.08	-814.77	-135.34	-44	-70.45	-12.86	-22.78	-2086.34

-63.86	-481.44	-65.41	-49.45	-52.13	-5.48	-9.32	-1853.14

-288.55	-298.16	-85.12	-199.64	-253.63	-117.29	-123.72	-387.65

NA	NA	NA	NA	NA	NA	NA	NA

NA	-656.57	-152.1	-16.06	-62.42 NA	NA	-2461.73

-33.56	-503.62	-45.56	-25.96	-6.73	-38.24	-36.82	-1972.86

-146.99	-128.5	-17.34	-70.04	-26.06	-45.04	-26.05	-712.92

-21.55	-187.23	-78.36	-8.17	-98.75 NA	NA	-1043.53

NA	NA	NA	NA	NA	NA	NA	NA

-71.58	-177.41	-203.52	-76.58	-168.46 NA	NA	NA

-236.98	-321.21	-234.81	-174.38	-229.47 NA	-78	-46.28

-108.28	-45.81	-7.31	-40.67	-38.21	-163.75	-21.41	-47.95

-138.45	-43.83 NA	-41.01	-32.02	-336.76	-6.98	-48.76

-669.82	-296.74	-285.15	-236.45	-808.45	-39.86	-204.19	-557.18

-295.96	-314.22	-106.27	-178.31	-181.39	-79.28	-135.44	-230.12

-72.41 NA	NA	-38.09	-11.94	-22.33 NA	-14.83

-233.06	-258.47	-114.3	-293.33	-153.68	-24.41	-177.55	-32.16

-215.91	-63.71	-18.24	-99.7	-62.57 NA	-33.88	-38.28

-289.09	-271.51	-109.6	-198.62	-338.14	-151.19	-161.6	-211.91

-68.21	-58.82	-36.14	-61.79	-85.45	-22.61	-32.11	-122.11

-140.54	-43.41	-26.02	-53.36	-73.65	-99.56	-39.9	-123.59

-112.96	-85.74	-29.17	-83.67	-101.6	-62.56	-79.24	-82.29

-237.99	-89.75	-53.48	-118.22	-115.74	-118.64	-99.54	-155.22

-166.5	-167.91	-81.01	-133.29	-145.34	-56.58	-76.2	-106.54

-141.06	-61.67	-9.79	-69.87	-55.92	-84.33	-77.22	-107.53

-26.61	-9.17 NA	-18.28	-13.71	-34.88	-21.6	-48.55

-15.69 NA	NA	NA	NA	NA	NA	NA

-15.69 NA	NA	NA	NA	NA	NA	NA

-15.69 NA	NA	NA	NA	NA	NA	NA

| NA |  | -2220.78 | | -419.64 | | -1323.75 | |  | -804.7 | -109.48 | | -2361.71 | | NA |  |
| --- | --- | --- | --- | --- | --- | --- | --- | --- | --- | --- | --- | --- | --- | --- | --- |
|  | -18.15 | -2654.05 | | -526.13 | | -1655.39 | | -1035.71 | | -136.11 | | -2865.87 | | NA |  |
| NA |  | -1393.28 | | -327.93 | | -784.27 | | -447.98 | |  | -37.74 | -1390.43 | | NA |  |
|  | -86.11 |  | -15.78 | NA |  |  | -24.85 |  | -14.73 | -305.64 | | NA |  |  | -32.65 |
|  | -25.64 | NA |  | NA |  | NA |  | NA |  |  | -48.3 | NA |  | NA |  |
|  | -42.95 | NA |  | NA |  | NA |  | NA |  | -268.94 | | NA |  |  | -17.89 |
|  | -55.17 | NA |  | NA |  | NA |  | NA |  | -285.58 | | NA |  |  | -13.92 |
| -112.53 | |  | -27.36 | NA |  |  | -33.7 |  | -15.27 |  | -29.23 |  | -50.88 |  | -31.09 |
| -225.74 | | -158.78 | |  | -66.98 | -100.53 | |  | -156.1 |  | -23.19 |  | -49.46 | -127.99 | |
| -226.92 | | -151.58 | |  | -67 | -153.33 | | -128.77 | |  | -94.15 |  | -91.82 | -194.96 | |
| -492.48 | | -126.65 | |  | -84.21 | -169.14 | | -165.64 | | -140.34 | |  | -80.4 | -189.84 | |
| -492.48 | | -126.65 | |  | -84.21 | -169.14 | | -165.64 | | -140.34 | |  | -80.4 | -189.84 | |
| -466.82 | |  | -167.1 |  | -88.9 | -171.28 | | -188.93 | | -133.06 | | -117.53 | | -130.24 | |
| -454.41 | |  | -36.31 | NA |  |  | -448.3 |  | -97.53 |  | -68.04 | -196.87 | |  | -33.45 |
| -454.41 | |  | -36.31 | NA |  |  | -448.3 |  | -97.53 |  | -68.04 | -196.87 | |  | -33.45 |
| NA |  |  | -17.9 | NA |  |  | -26.21 | NA |  | -112.23 | |  | -42.19 |  | -55.65 |
| NA |  | NA |  | NA |  | NA |  | NA |  |  | -29.48 |  | -32.55 |  | -74.22 |
| NA |  | NA |  | NA |  |  | -5.58 | NA |  |  | -93.85 |  | -16.03 |  | -34.92 |
| -151.19 | |  | -76.66 |  | -8.21 |  | -47.13 | NA |  |  | -17.51 |  | -38.21 | NA |  |
|  | -376.2 | -2644.33 | | -835.16 | | -1658.01 | | -2097.13 | | -918.57 | | -2366.89 | | -110.16 | |
|  | -33.91 | -4874.81 | | -912.58 | | -1115.05 | | -2642.75 | | -670.92 | | -2345.95 | |  | -23.92 |
|  | -35.31 | -2120.97 | | -184.87 | | -402.39 | |  | -713.1 |  | -184.4 | -1118.15 | |  | -15.83 |
| -313.32 | | -3447.89 | |  | -731.8 | -1027.67 | | -1922.54 | | -464.67 | | -1906.68 | |  | -180.1 |
|  | -94.49 | -6431.05 | | -1276.45 | | -1600.24 | | -3961.5 | | -1075.09 | |  | -3265 |  | -44.46 |
|  | -65.08 | -4581.27 | | -766.97 | | -1164.08 | | -2551.17 | | -797.56 | | -2497.84 | |  | -24.49 |
| -642.57 | | -209.91 | |  | -43.84 |  | -99.8 | -138.08 | | -156.29 | | -130.96 | | -137.46 | |
| -106.75 | | -858.97 | |  | -46.02 | -459.76 | | -129.36 | | -301.17 | | -1003.41 | | -105.05 | |
| -593.36 | | -162.44 | |  | -44.15 | -185.08 | | -154.13 | | -236.55 | | -199.81 | | -172.13 | |
|  | -66.29 | -103.12 | |  | -12.65 |  | -55.53 |  | -45.22 |  | -57.44 |  | -36.52 |  | -12.45 |
| -553.89 | | -863.39 | | -378.86 | | -433.89 | |  | -402.6 | -278.86 | | -259.97 | | -245.55 | |
| -911.27 | | -792.22 | | -264.55 | | -294.23 | | -320.98 | | -574.73 | | -269.04 | | -230.44 | |
| NA |  | -145.39 | |  | -65.15 |  | -121.7 | NA |  |  | -67.82 |  | -26.09 | NA |  |
| NA |  |  | -36.21 | NA |  |  | -89.81 | NA |  | -190.02 | | -231.52 | |  | -35.68 |
| -387.75 | | NA |  | NA |  | NA |  | NA |  |  | -42.89 | NA |  | -354.04 | |
| -232.61 | | NA |  | NA |  | NA |  | NA |  | NA |  | NA |  |  | -292.3 |
|  | -92.46 |  | -83.7 | -127.69 | |  | -48.34 | -166.62 | | NA |  | NA |  |  | -49.72 |
| -482.19 | | -374.03 | | -266.48 | | -150.95 | | -308.23 | |  | -13.58 |  | -33.47 | -143.93 | |
| -265.26 | | -283.03 | |  | -302 |  | -80.87 | -294.74 | | NA |  | NA |  |  | -57.48 |
| NA |  | NA |  | NA |  | NA |  | NA |  | NA |  | NA |  | NA |  |

| -379.8 | -289.04 | -235.92 | -135.23 | -297.39 | NA | -16.27 | -106.29 |
| --- | --- | --- | --- | --- | --- | --- | --- |
| -1082.73 | -906.31 | -421.26 | -443.76 | -570.97 | -328.81 | -276.46 | -352.88 |
| -107.2 | -260.45 | -65.55 | -312.75 | -218.4 | -117.81 | -204.68 | -212.69 |
| -82.31 | -196.31 | -47.68 | -268.71 | -200.77 | -76.45 | -169.76 | -163.66 |
| -41.5 | -82.68 | -31.64 | -183.83 | -158.78 | -35.55 | -93 | -98.07 |
| -30.76 | -82.7 | -49.48 | -71.81 | -58.69 | -99.97 | -50.24 | -16.16 |

-201.62 NA	NA	-18.83	-190.8	-175.6 NA	-720.35

-93.46	-219.46	-68.09	-274.23	-252.81	-89.98	-149.08	-183.41

-18.68	-87.03	-44.24	-184.51	-169.59	-35.69	-64.17	-97.42

NA	NA	NA	NA	NA	NA	NA	NA NA	NA	NA	NA	NA	NA	NA	NA NA	NA	NA	NA	NA	NA	NA	NA

-882.83	-457.02	-350	-357.67	-767.98	-89.63	-294.99	-625.91

-309.38	-247.43	-160.62	-263.19	-275.43	-73.74	-167.31	-343.13

-144.69	-143.78	-38.23	-40.98	-19.95	-43.32	-18.05 NA

-318.74	-351.2	-145.02	-147.51	-155.97 NA	-83.99	-291.68

-127.49	-117.79	-34.28	-32.44	-13.91	-44.72	-13.02 NA NA	-39.73	-14.11	-72.59	-10.96 NA	-54.62 NA

-69.71	-78.11	-101.91	-73.25	-140.73 NA	NA	-54.78

NA	NA	NA	NA	NA	NA	NA	NA

-963.31	-385.97	-310.28	-354.85	-603.73	-184.67	-226.28	-510.42

-104.82	-19.52 NA	-51	-39.67	-43.65	-14.46	-51.92

NA	NA	-16.49 NA	-18.15 NA	NA	NA

-9.41 NA	-12.8 NA	-18.84 NA	NA	NA

| -68.85 | -181.14 | -196.88 | -104.19 | -183.62 NA | |  | -42.7 | -14.5 |
| --- | --- | --- | --- | --- | --- | --- | --- | --- |
| -216.79 | -205.38 | -86.04 | -103.75 | -77.87 NA | |  | -86.03 | -15.34 |
| -191.53 | -236.05 | -207.38 | -107.04 | -186.73 NA | |  | -68.04 | -45.59 |
| -83.02 | -189.01 | -212.67 | -94.89 | -260.29 NA | |  | -22.46 | -83.97 |
| -56.29 | -66.22 | -13.74 | -44.64 | -32.85 NA | |  | -19.39 | -5.38 |
| -420.82 | -264.19 | -111.79 | -187.91 | -290.73 | | -68.16 | -136.83 | -330.84 |
| -313.62 | -260.16 | -70.13 | -188.12 | -139.7 | | -9.01 | -179.59 | -67.36 |
| -464.01 | -339.53 | -239.48 | -336.89 | -412.95 | -132.47 | | -222.67 | -372.78 |
| -247.12 | -165.63 | -41.11 | -166.47 | -134.49 | -34.7 | | -100.65 | -54.2 |
| -179.03 | -224.97 | -36.65 | -177.78 | -102.07 | -78.36 | | -152.41 | -113.01 |
| -120.25 | -62.42 | -48.93 | -26.29 | -112.53 | NA | | -7.04 | -119.64 |
| -377.02 | -407.44 | -72.35 | -157.63 | -219.14 | -154.28 | | -302.84 | -77.29 |
| -356.7 | -845.48 | -146.87 | -361.99 | -553.35 | -305.18 | | -655.63 | -28.14 |
| -185.41 | -90.72 | -47.05 | -90.58 | -100.42 | -7.62 | | -44.21 | -100.02 |
| -252.91 | -106.26 | -245.35 | -237.53 | -345.83 | -69.94 | | -146.21 | -474.56 |

| -239.51 | | -168.11 | |  | -85.7 | -135.75 | | -197.05 | |  | -14.3 |  | -86.38 | -108.46 | |
| --- | --- | --- | --- | --- | --- | --- | --- | --- | --- | --- | --- | --- | --- | --- | --- |
|  | -362.7 | -458.39 | | -177.49 | | -204.61 | | -279.89 | |  | -59.64 | -182.75 | | -172.07 | |
|  | -723.8 | -362.24 | | -144.81 | | -308.13 | | -299.97 | | -322.54 | | -257.02 | | -220.85 | |
|  | -20.2 |  | -73.55 |  | -38.24 |  | -65.84 |  | -51.13 | NA |  |  | -43.73 |  | -51.94 |
| -1114.19 | | -464.83 | | -192.67 | | -229.87 | | -878.38 | | -362.02 | | -511.37 | | -716.65 | |
|  | -14.82 | NA |  | NA |  |  | -13 | NA |  |  | -59.46 |  | -58.9 |  | -95.45 |
| -725.76 | | -515.89 | | -267.26 | | -344.39 | | -429.44 | | -260.88 | |  | -291.1 | -361.69 | |
| -406.36 | |  | -208.5 | NA |  | -109.52 | | -199.36 | |  | -76.6 |  | -44.72 | -162.45 | |
| -309.76 | | -124.68 | | -112.13 | | -178.14 | | -250.41 | |  | -34.54 |  | -61.04 | -172.21 | |
| -114.11 | |  | -157.7 |  | -54.69 |  | -93.22 | -103.21 | |  | -18.86 | -120.42 | |  | -54.13 |
| -250.42 | |  | -149.6 |  | -64.36 | -128.28 | | -138.48 | |  | -6.74 |  | -70.46 |  | -167.5 |
| NA |  | NA |  | NA |  | NA |  | NA |  | -309.73 | | NA |  | NA |  |
|  | -66.33 | -241.82 | |  | -68.97 |  | -165.7 | -216.03 | |  | -20.86 |  | -142.2 |  | -33.53 |
|  | -57.29 |  | -82.22 |  | -42.46 |  | -47.64 |  | -35.15 | NA |  |  | -33.8 |  | -6.18 |
|  | -64.2 | -206.15 | |  | -83.37 |  | -99.53 |  | -94.26 | NA |  |  | -82.5 |  | -94.99 |
| -1265.78 | | -693.85 | | -520.59 | | -535.15 | | -713.24 | | -304.35 | | -356.36 | | -559.24 | |
| -160.12 | |  | -33.69 |  | -37.04 |  | -50.45 |  | -35.07 | NA |  |  | -8.11 | -272.18 | |
| -422.75 | | -235.31 | | -268.74 | | -251.22 | | -302.35 | |  | -56.62 | -119.32 | | -176.92 | |
| NA |  | NA |  | NA |  | NA |  | NA |  | NA |  |  | -17.94 |  | -17.73 |
|  | -48.26 | -135.41 | |  | -16.44 |  | -88.8 |  | -83.63 | NA |  |  | -72.01 |  | -54.1 |
| -221.68 | | -293.84 | |  | -42.83 | -245.86 | | -116.35 | |  | -35.27 | -235.95 | | -189.42 | |
| NA |  | -146.07 | | NA |  |  | -28.94 |  | -26.88 | NA |  | -120.47 | | NA |  |
| -270.49 | | -112.99 | | -105.33 | |  | -119.9 | -233.15 | |  | -27.67 |  | -26.44 | -117.91 | |
| -137.96 | | -474.22 | |  | -53.97 |  | -77.93 | -203.86 | | NA |  |  | -91.81 |  | -21.48 |
|  | -181 | -128.72 | | NA |  | -128.14 | | -209.36 | |  | -44.18 |  | -42.22 | -171.72 | |
|  | -118.1 | -328.77 | |  | -43.72 | -176.33 | | -210.73 | |  | -70.61 |  | -281.7 |  | -52.98 |
| -352.91 | | -130.49 | |  | -51.28 | -121.37 | | -178.73 | |  | -118.9 |  | -76.35 | -114.49 | |
| -245.08 | |  | -202.3 |  | -68.56 |  | -81.35 |  | -51.97 |  | -6.01 |  | -58.44 |  | -20.13 |
| -239.66 | | -136.52 | |  | -59.69 | -123.68 | | -124.08 | |  | -87.78 |  | -82.57 |  | -75.29 |
|  | -85.11 |  | -73.15 |  | -15.12 |  | -72.96 |  | -36.58 |  | -27.01 |  | -70.13 |  | -91.97 |
| -208.13 | | -461.15 | |  | -79.75 | -111.45 | |  | -171.8 |  | -41.22 | -212.83 | |  | -34.95 |
| -475.99 | | -267.58 | | -200.34 | |  | -188.3 | -315.46 | |  | -39.89 |  | -95.91 | -316.92 | |
| -331.99 | | -201.96 | | -129.01 | |  | -80.23 | -127.87 | | NA |  |  | -22.5 |  | -9.96 |
|  | -11.98 | -322.05 | |  | -64.74 | -214.48 | |  | -96.71 |  | -117 |  | -394.6 |  | -196.2 |
|  | -12.91 | NA |  | NA |  | NA |  | NA |  | NA |  | NA |  |  | -52.1 |
| -287.71 | | -102.04 | |  | -55.11 | -127.03 | | -121.88 | |  | -32.25 |  | -51.79 |  | -245.7 |
|  | -66.44 | -138.42 | |  | -69.51 | -135.45 | | -137.73 | |  | -36.8 | -133.64 | |  | -39.15 |
|  | -302.1 | -265.22 | |  | -54.41 | -121.99 | |  | -92.86 | NA |  | -126.54 | | -173.27 | |

venous_bl Motif	geneid	type	adipocyte. adult_end alternative band_form

-350.24 AHR_1.mo AHR	Direct	1.16899	1.11973	1.11986	1.12207

NA	ALX3_1.m ALX3	Direct	NA	NA	NA	NA

-1560.41 ARNT_1.m ARNT	Direct	1.15347	1.23185	1.14782	1.10516

-1037.95 ARNT_2.m ARNT	Direct	1.10087	1.25858	1.23857	1.14292

-1047.76 ARNTL_1. ARNTL	Direct	NA	1.30451	1.12524	1.08147

-1234.96 ASCL2_1. ASCL2	Direct	0.962352 0.970366 0.966622 0.981782

-1986.5 ATF3_1.m ATF3	Direct	1.126	1.28246	1.21111	1.07645

-665.91 ATF6_1.m ATF6	Direct	1.04801	1.304	1.06829	1.05043

-665.91 ATF6_1.m CREBZF	Inferred	1.04801	1.304	1.06829	1.05043

-58.19 ATF7_1.m ATF7	Direct	1.11812	1.0769	1.33162	1.1144

-678.27 ATOH1_1. ATOH1	Direct	0.982967 0.992499 0.961175 0.975797

-655.72 ATOH7_1. ATOH7	Direct	0.982942 0.988913	1.04386	1.07558

-208.37 BACH1_1. BACH1	Direct	1.05018	1.04154	1.00356	1.0788

-428.41 BACH2_1. BACH2	Direct	1.0556	0.99643	1.00926	1.06235

-128.07 BARHL2_1BARHL2	Direct	NA	1.14868 NA	NA NA	BARX1_2. BARX1	Direct	NA	NA	NA	NA

-177.3 BATF_1.m BATF	Direct	1.00875 0.976989 NA	NA

-177.3 BATF_1.m BATF2	Inferred	1.00875 0.976989 NA	NA

-2400.51 BCL11A_1 BCL11A	Direct	0.972016 0.988449 0.884921 0.985045

-2422.71 BHLHE40_BHLHE40 Direct	1.18461	1.36777	1.06585	1.11456

NA	BSX_1.mo BSX	Direct	NA	NA	NA	NA

-1590.83 CBFB_1.m CBFB	Direct	0.99012 0.993385	1.04671	1.00123

NA	CDX1_1.m CDX1	Direct	NA	NA	NA	NA NA	CDX2_1.m CDX2	Direct	NA	NA	NA	NA

NA	CEBPA_1. CEBPA	Direct	1.00876 NA	1.04301	1.01094

NA	CEBPB_1. CEBPB	Direct	1.05046 NA	1.09072	1.03282

NA	CEBPD_1. CEBPD	Direct	1.06235 NA	1.10231	1.05345

NA	CEBPE_1. CEBPE	Direct	1.06374 NA	1.1413	1.02956

-2121.83 CLOCK_1. CLOCK	Direct	1.13551	1.28662	1.04193	1.06532

-2121.83 CLOCK_1. SOHLH1	Inferred	1.13551	1.28662	1.04193	1.06532

-2559.08 CREB3L2_ CREB3L2 Direct	1.11683	1.4425	1.11642	1.09428

-2830.74 CREB3L3_ CREB3L3 Direct	1.12829	1.4579	1.16368	1.15648

-48.31 CREB5_1. CREB5	Direct	1.15566	1.06605	1.22776	1.0673

NA	DBP_1.mo DBP	Direct	1.03593 NA	0.948548 0.982976

NA	DLX1_1.m DLX1	Direct	NA	NA	NA	NA NA	DLX2_1.m DLX2	Direct	NA	NA	NA	NA NA	DLX6_1.m DLX6	Direct	NA	NA	NA	NA

NA	E2F1_1.m E2F1	Direct	1.05137	1.04826	1.06832	1.07116

-537.35 EBF1_1.m EBF1	Direct	1.05179 0.995744	1.00089 0.967699

-537.35 EBF1_1.m EBF2	Inferred	1.05179 0.995744	1.00089 0.967699

-537.35 EBF1_1.m EBF4	Inferred	1.05179 0.995744	1.00089 0.967699

-548.68 EGR2_1.m EGR2	Direct	1.09195	1.09487 0.954979	1.00492

-338.73 EGR4_1.m EGR4	Direct	1.07248	1.06535 0.975107 0.957474

-2309.58 EHF_1.mo EHF	Direct	0.98741	1.00264 0.918891 0.934633

-2784.24 EHF_2.mo EHF	Direct	1.02705	1.01757	1.05925	1.06374

-4170.87 ELF1_1.m ELF1	Direct	1.04135	1.01868	1.0475	1.04002

-3262 ELF2_1.m ELF2	Direct	0.961997 0.991624 0.949689 0.965112

-1965.56 ELF3_1.m ELF3	Direct	1.06924	1.03388	1.09464	1.06685

-2305.76 ELF4_1.m ELF4	Direct	1.06334 0.998983	1.09656	1.08986

-3950.1 ELF5_1.m ELF5	Direct	1.00621	1.01211	1.00968	1.06501

-2215.48 ELK1_1.m ELK1	Direct	1.08771	1.01461	1.1266	1.08742

-3061.82 ELK3_1.m ELK3	Direct	1.04453	1.00361	1.08024	1.0471

-3402.76 ELK4_1.m ELK4	Direct	1.03714	1.0054	1.08601	1.04035

NA	EN1_2.mo EN1	Direct	NA	NA	NA	NA

-1815.48 ENO1_1.m ENO1	Direct	1.10432	1.28326	1.04501	1.04462

-3370.9 ERF_1.mo ERF	Direct	1.08316	1.05407	1.11377	1.06536

-4172 ERG_1.mo ERG	Direct	1.04334	1.01113	1.07453	1.04163

-259.88 ERG_2.mo ERG	Direct	1.13032	1.072	1.13974	1.05275

-5339.88 ERG_3.mo ERG	Direct	0.996444	1.00957 0.992934 0.980298

-24.1 ESRRG_3. ESRRG	Direct	1.00168 0.999487 NA	NA NA	ESX1_1.m ESX1	Direct	NA	NA	NA	NA

-4734.48 ETS1_1.m ETS1	Direct	1.03338	1.00403	1.08285	1.04313

-162.81 ETS1_2.m ETS1	Direct	0.978261	1.0515	1.14118	1.04494

-1831.71 ETS2_1.m ETS2	Direct	1.01792	1.03898	1.05765	1.04405

-3936.83 ETV1_1.m ETV1	Direct	1.04518	1.02398	1.12029	1.0756

-3376.98 ETV2_1.m ETV2	Direct	1.05699	1.01815	1.03001	1.04667

-1250.07 ETV3_1.m ETV3	Direct	1.07156	1.01436	1.10571	1.05731

-1250.07 ETV3_1.m ETV3L	Inferred	1.07156	1.01436	1.10571	1.05731

-4596.16 ETV4_1.m ETV4	Direct	1.04075	1.01578	1.06056	1.03987

-1668.42 ETV5_1.m ETV5	Direct	1.09353	1.02618	1.04419	1.05215

-1229.53 ETV6_1.m ETV6	Direct	0.993723	1.00809	1.128	1.06247

-1930.55 ETV7_1.m ETV7	Direct	0.958309	1.02221 0.973067 0.976112

NA	EVX2_1.m EVX2	Direct	NA	NA	NA	NA

-758.85 FERD3L_1 FERD3L	Direct	0.989605	1.04904 0.976818	1.03309

-4697.18 FEV_1.mo FEV	Direct	1.06216	1.03576	1.10159	1.04756

-5012.95 FLI1_1.mo FLI1	Direct	1.01405	1.03413	1.05666	1.01444

-320.7 FLI1_2.mo FLI1	Direct	1.23228	1.14035	1.19919	1.10087

-350.61 FOS_1.mo FOS	Direct	1.04002	1.01721 0.959924	1.02748

-408.95 FOSB_1.m FOSB	Direct	1.01975 0.990033 0.947059 0.988773

-576.66 FOSL1_1. FOSL1	Direct	1.00529	1.02273 0.955572	1.01729

-202.03 FOSL2_1. FOSL2	Direct	1.02366	1.0176 0.951864	1.01814

NA	FOXA1_1. FOXA1	Direct	0.992299 NA	NA	NA NA	FOXA2_1. FOXA2	Direct	NA	NA	NA	NA NA	FOXC1_1. FOXC1	Direct	NA	0.943081 NA	NA NA	FOXD1_1. FOXD1	Direct	1.03978 0.997838 NA	NA NA	FOXD2_2. FOXD2	Direct	1.04742	0.99592 NA	NA NA	FOXD4_1. FOXD4	Direct	1.05868 0.991601 NA	NA NA	FOXF1_1. FOXF1	Direct	NA	NA	NA	NA NA	FOXF1_1. FOXN1	Inferred	NA	NA	NA	NA NA	FOXF1_1. FOXN4	Inferred	NA	NA	NA	NA NA	FOXF2_1. FOXF2	Direct	NA	NA	NA	NA NA	FOXG1_1. FOXG1	Direct	1.06221	1.00906 NA	NA NA	FOXK1_1. FOXK1	Direct	1.04666 0.999362 NA	NA NA	FOXO3_1. FOXO3	Direct	0.977706 0.982738 NA	NA NA	FOXP1_1. FOXP1	Direct	0.968947	0.99226 NA	NA

-6.75 FOXP2_1. FOXP2	Direct	1.05777	1.01162 NA	NA NA	FOXP3_1. FOXP3	Direct	1.06866 0.985207 NA	NA NA	FOXQ1_1. FOXQ1	Direct	NA	NA	NA	NA

-3427.67 GABPA_1. GABPA	Direct	1.05502	1.02853	1.11377	1.06272

-70.64 GATA1_1. GATA1	Direct	0.938742 0.960839 NA	0.919132

-70.64 GATA1_1. GATAD2B Inferred	0.938742 0.960839 NA	0.919132

NA	GATA2_1. GATA2	Direct	0.978155 0.996283 NA	NA NA	GATA2_1. ZGLP1	Inferred	0.978155 0.996283 NA	NA NA	GATA3_2. GATA3	Direct	0.959833 NA	NA	NA

NA	GATA4_1. GATA4	Direct	0.978495	1.01389 NA	0.997282

NA	GATA5_1. GATA5	Direct	0.932163 NA	NA	NA NA	GATA6_1. GATA6	Direct	1.01163 NA	NA	NA NA	GATA6_1. MTA2	Inferred	1.01163 NA	NA	NA NA	GATA6_1. RERE	Inferred	1.01163 NA	NA	NA NA	GBX1_1.m GBX1	Direct	NA	NA	NA	NA NA	GBX2_1.m GBX2	Direct	NA	NA	NA	NA NA	GSX1_1.m GSX1	Direct	NA	NA	NA	NA NA	GSX2_1.m GSX2	Direct	NA	NA	NA	NA

-136.6 HAND1_1. HAND1	Direct	0.982496	1.0546 0.842362 0.950084

-693 HES1_1.m HES1	Direct	1.10403	1.1677	1.05048	1.10086

-693 HES1_1.m HES6	Inferred	1.10403	1.1677	1.05048	1.10086

-1374.59 HES4_1.m HES4	Direct	1.18545	1.31559	1.21074	1.10095

-224.06 HES7_1.m HES7	Direct	NA	1.22044	1.06108	1.0384

-790.81 HEYL_1.m HEYL	Direct	1.04936	1.14556	1.21379	1.08989

-157.82 HIC1_1.moHIC1	Direct	1.03509	1.0215 0.989653 0.965724

-503.92 HIVEP2_1. HIVEP2	Direct	1.06963	1.04826	1.06571	1.10746

NA	HLF_1.mo HLF	Direct	1.02836 NA	0.977663	1.01433

NA	HNF1A_1. HNF1A	Direct	NA	NA	NA	NA NA	HNF1B_1. HNF1B	Direct	NA	NA	NA	NA

-56.97 HNF4A_1. HNF4A	Direct	0.982703	1.01914 0.910242 0.940315

-67.48 HNF4A_2. HNF4A	Direct	1.01239	1.02875 NA	1.04005

-151.85 HNF4G_1. HNF4G	Direct	0.961368	1.01293 0.965746 0.977982

NA	HOXA1_1. HOXA1	Direct	NA	NA	NA	NA NA	HOXA10_1HOXA10	Direct	NA	NA	NA	NA NA	HOXA11_1HOXA11	Direct	NA	NA	NA	NA NA	HOXA13_1HOXA13	Direct	NA	NA	NA	NA NA	HOXA13_2HOXA13	Direct	NA	NA	NA	NA NA	HOXA2_1. HOXA2	Direct	NA	NA	NA	NA NA	HOXA5_2. HOXA5	Direct	1.00011 NA	NA	NA NA	HOXA6_1. HOXA6	Direct	NA	NA	NA	NA NA	HOXA6_10HOXA6	Direct	0.974156 NA	NA	NA NA	HOXA6_4. HOXA6	Direct	NA	NA	NA	NA NA	HOXA6_7. HOXA6	Direct	0.993039 NA	NA	NA NA	HOXA6_8. HOXA6	Direct	NA	NA	NA	NA NA	HOXA6_9. HOXA6	Direct	0.992859 NA	NA	NA NA	HOXA9_1. HOXA9	Direct	NA	NA	NA	NA NA	HOXA9_2. HOXA9	Direct	1.01235 NA	NA	NA NA	HOXB13_1HOXB13	Direct	NA	NA	NA	NA NA	HOXB13_2HOXB13	Direct	NA	NA	NA	NA NA	HOXB2_1. HOXB2	Direct	NA	NA	NA	NA NA	HOXB3_1. HOXB3	Direct	NA	NA	NA	NA NA	HOXB4_1. HOXB4	Direct	1.02797 0.993401 NA	NA NA	HOXB5_1. HOXB5	Direct	NA	NA	NA	NA NA	HOXB7_1. HOXB7	Direct	0.982107 NA	NA	NA NA	HOXB8_1. HOXB8	Direct	0.986588 NA	NA	NA NA	HOXC10_1HOXC10	Direct	NA	NA	NA	NA NA	HOXC11_1HOXC11	Direct	1.05692 NA	NA	NA NA	HOXC11_2HOXC11	Direct	NA	NA	NA	NA NA	HOXC12_2HOXC12	Direct	1.04443 NA	NA	NA NA	HOXC13_2HOXC13	Direct	NA	NA	NA	NA

| NA | HOXC5_1. HOXC5 | | Direct | 1.02366 NA | | NA | NA |
| --- | --- | --- | --- | --- | --- | --- | --- |
| NA | HOXC9_1. HOXC9 | | Direct | 0.9694 NA | | NA | NA |
| NA | HOXD1_1. HOXD1 | | Direct | NA NA | | NA | NA |
| NA | HOXD11_ | HOXD11 | Direct | NA | NA | NA | NA |
| NA | HOXD12_ | HOXD12 | Direct | NA | NA | NA | NA |
| NA | HOXD12_ | HOXD12 | Direct |  | 1.0438 NA | NA | NA |
| NA | HOXD13_ | HOXD13 | Direct | NA | NA | NA | NA |
| NA | HOXD13_ | HOXD13 | Direct | NA | NA | NA | NA |
| NA | HOXD3_1. | HOXD3 | Direct | NA | NA | NA | NA |

NA	HOXD9_2. HOXD9	Direct	1.03932 NA	NA	NA

-895.09 IKZF3_1.mIKZF3	ZifRC	1.01167 0.993524 0.949718 0.937765

-737.13 IKZF5_1.mIKZF5	ZifRC	1.25549	1.27153 NA	1.03547

-177.18 IRF1_1.mo IRF1	Direct	1.0083 NA	NA	NA

-872.05 IRF3_1.mo IRF3	Direct	1.02405	1.0079 0.876528 0.971161

-699.33 IRF4_2.mo IRF4	Direct	1.03319	1.10487	1.09415	1.09156

-651.03 IRF5_1.mo IRF5	Direct	1.04882	1.08227	0.95839	1.06723

-682.86 IRF6_1.Inf IRF6	Indirect	NA	1.24895	1.06442	1.09088

-305.13 IRF7_1.mo IRF7	Direct	1.00841	1.03602 NA	NA

-1321.17 IRF8_1.mo IRF8	Direct	0.990378	1.03146 0.915049	1.01569

-336.67 IRF9_1.mo IRF9	Direct	1.00812 NA	0.927336	1.05483

-263.87 JDP2_1.m JDP2	Direct	1.04343	1.02163 0.966146	1.0488

-267.04 JUN_1.mo JUN	Direct	1.0313	1.02994 0.965687 0.996348

-407.53 JUNB_1.m JUNB	Direct	1.01565	1.02382 0.947439	1.0499

-166.51 JUND_1.m JUND	Direct	1.03895	1.01906	1.01036	1.00255

-178.77 KLF1_1.m KLF1	Direct	NA	1.02013 NA	NA

-263 KLF12_1.mKLF12	Direct	1.01689	1.04649	1.00863	1.09181

-330.72 KLF12_2.mKLF12	Direct	NA	1.10248	1.13224	1.02188

-250.91 KLF13_1.mKLF11	Inferred	1.07153	1.19457 0.987805 0.995536

-250.91 KLF13_1.mKLF13	Direct	1.07153	1.19457 0.987805 0.995536

-210.14 KLF14_1.mKLF14	Direct	NA	1.10028	1.04053 NA

-315.68 KLF16_1.mKLF16	Direct	NA	1.02935 0.987992 0.979731

-198.85 KLF4_1.m KLF17	Inferred	NA	1.01425	1.07083 NA

-198.85 KLF4_1.m KLF2	Inferred	NA	1.01425	1.07083 NA

-198.85 KLF4_1.m KLF4	Direct	NA	1.01425	1.07083 NA

-192.2 KLF5_1.m KLF5	Direct	0.989315	1.01005 0.988782	1.00877

-207.78 KLF6_1.m KLF6	Direct	1.03294 0.996306 0.954613 0.990039

-202.25 KLF7_1.m KLF7	Direct	NA	1.05338 NA	NA NA	LBX1_1.m LBX1	Direct	NA	NA	NA	NA

-56.52 LEF1_1.m LEF1	Direct	1.00208	0.97383 NA	NA

NA	LHX2_1.m LHX2	Direct	NA	NA	NA	NA NA	LHX6_1.m LHX6	Direct	NA	NA	NA	NA NA	LMX1A_1. LMX1A	Direct	NA	NA	NA	NA NA	LMX1B_1. LMX1B	Direct	NA	NA	NA	NA

-629.51 LYL1_1.In LYL1	Indirect	1.00623 0.965478	1.02035 0.971535

-391.55 MAF_1.mo MAF	Direct	0.976744	1.03892	1.13628	1.02657

-252.51 MAFA_1.mMAFA	Direct	1.0173 0.997023 0.991717 0.994461

-573.91 MAFB_1.mMAFB	Direct	1.03076	1.03665	1.10206	1.04809

-24.92 MAFF_2.mMAFF	Direct	1.00894 0.938797 0.846306 0.944609

-54.39 MAFG_1.mMAFG	Direct	1.04639 0.989252 0.911884	1.01608

-2026.03 MAX_2.moMAX	Direct	1.1046	1.25693	1.06669	1.06052

NA	MBD2_1.mMBD2	Direct	1.17549	1.10204	1.23191	1.16553

NA	MECP2_1. MECP2	Direct	1.26058	1.20086	1.32543	1.1727

-297.52 MEIS2_1. MEIS2	Direct	0.951424 0.968296 0.972932 0.973949

NA	MEOX1_1. MEOX1	Direct	1.03982 NA	NA	NA NA	MEOX2_1. MEOX2	Direct	NA	NA	NA	NA

-2516.08 MLXIPL_1 MLXIPL	Direct	1.09517	1.31618	1.12709	1.11686

NA	MNX1_1.mMNX1	Direct	NA	NA	NA	NA

-807.94 MSC_1.moMSC	Direct	0.946889 0.994839	1.03101 0.982844

NA	MSX1_1.mMSX1	Direct	1.02546 NA	NA	NA NA	MSX1_1.mNKX1-2	Inferred	1.02546 NA	NA	NA

-329.77 MTF1_1.m MTF1	Direct	1.05664	1.14207	1.18655	1.07704

-2439.94 MXI1_1.m MXD1	Inferred	1.10876	1.26751	1.10638	1.08096

-2439.94 MXI1_1.m MXD3	Inferred	1.10876	1.26751	1.10638	1.08096

-2439.94 MXI1_1.m MXD4	Inferred	1.10876	1.26751	1.10638	1.08096

-2439.94 MXI1_1.m MXI1	Direct	1.10876	1.26751	1.10638	1.08096

-469.66 MYB_1.moMYB	Direct	1.02242	1.05363	1.05268	1.07009

-469.66 MYB_1.moRCOR2	Inferred	1.02242	1.05363	1.05268	1.07009

-469.66 MYB_1.moRCOR3	Inferred	1.02242	1.05363	1.05268	1.07009

-469.66 MYB_1.moSNAPC4	Inferred	1.02242	1.05363	1.05268	1.07009

-720.48 MYBL1_1. DNAJC2	Inferred	1.13476	1.20484	1.05838	1.1029

-720.48 MYBL1_1. MYBL1	Direct	1.13476	1.20484	1.05838	1.1029

-374.91 MYBL1_2. DNAJC2	Inferred	1.04497	1.11419 0.958419	1.08443

-374.91 MYBL1_2. MYBL1	Direct	1.04497	1.11419 0.958419	1.08443

-3303.66 MYCN_1.mMYCL	Inferred	1.14256	1.39437	1.0957	1.10797

-1005.68 MYF5_1.m ASCL1	Inferred	0.961542 0.981215 0.963467 0.925659

-1005.68 MYF5_1.m ASCL5	Inferred	0.961542 0.981215 0.963467 0.925659

-735.28 MYF6_1.m ASCL3	Inferred	0.99608	1.00594 0.982756	1.01744

-735.28 MYF6_1.m MYF6	Direct	0.99608	1.00594 0.982756	1.01744

| -952.57 MYOD1_1 MYOD1 | Direct | 0.968075 | 1.0356 | 0.960901 | 0.969136 |
| --- | --- | --- | --- | --- | --- |
| -1198.72 MYOG_1. MYOG Direct | | 0.989234 | 0.987024 | 0.960437 | 0.939653 |
| -849.6 NEUROD1 NEUROD1 Direct | | 1.01309 | 1.01405 | 1.00503 | 1.03095 |
| -289.59 NEUROD4 NEUROD4 Direct | | 1.00334 | 1.00903 | 0.885316 | 0.992131 |

-358.45 NFE2_1.m NFE2	Direct	1.0156	1.0226	1.01371	1.01039

-179.01 NFE2_2.m NFE2	Direct	1.06648	1.03681 0.890973	1.08053

-341.74 NFE2L2_1 NFE2L2	Direct	1.05493	1.01187 0.976471	1.01952

-202.45 NFIA_1.moNFIA	Direct	1.03921	1.05405 0.995316	1.01721

-241.88 NFIB_1.m NFIB	Direct	1.0157	1.04678	1.05169	1.01755

-17.45 NFIC_1.m NFIC	Direct	1.02751	1.00457	0.97823 0.991753

-247.36 NFIX_1.moNFIX	Direct	1.03008	1.0415 0.987627 0.971561

-162.63 NFIX_2.moNFIX	Direct	1.00313 0.990337 0.995917	1.00994

-431.45 NFKB1_1. NFKB1	Direct	NA	NA	NA	NA

-776.89 NFKB1_2. NFKB1	Direct	0.960326	1.02528 0.931731 0.980798

-348.18 NFKB2_1. NFKB2	Direct	NA	NA	NA	NA NA	NKX2_1_1 NKX2-1	Direct	NA	NA	NA	NA NA	NKX2_2_1 NKX2-2	Direct	NA	NA	NA	NA

-58.88 NKX2_3_1 NKX2-3	Direct	0.951062 0.976733 NA	0.937746

NA	NKX2_4_2 NKX2-4	Direct	NA	NA	NA	NA NA	NKX2_5_1 NKX2-5	Direct	NA	NA	NA	NA NA	NKX2_5_3 NKX2-5	Direct	NA	NA	NA	NA NA	NKX2_6_1 NKX2-6	Direct	NA	NA	NA	NA

-23.01 NKX2_8_1 NKX2-8	Direct	NA	0.948086 NA	NA

-80.64 NKX3_1_2 NKX3-1	Direct	NA	NA	NA	NA

-143.09 NKX3_2_1 NKX3-2	Direct	0.950069 0.979775 0.978419	1.00248

NA	NKX6_2_1 NKX6-2	Direct	NA	NA	NA	NA NA	NKX6_3_1 NKX6-3	Direct	NA	NA	NA	NA NA	NOBOX_1. NOBOX	Direct	NA	NA	NA	NA

-3344.61 NPAS2_1. NPAS2	Direct	1.12504	1.34681	1.1183	1.10723

-431.6 NR0B1_1. NR0B1	Direct	1.06513	1.07954	1.04321	1.07235

-431.6 NR0B1_1. NR0B2	Inferred	1.06513	1.07954	1.04321	1.07235

NA	NR1H2_1. NR1H2	Direct	0.946281 0.948244 NA	NA

-116.97 NR1H4_1. NR1H4	Direct	1.0046	1.08804 NA	1.10054

-60.39 NR2C1_1. NR2C1	Direct	0.986774 0.996258 NA	NA

-108.56 NR2C2_1. NR2C2	Direct	1.02627	1.00314 NA	NA

-317.1 NR2C2_2. NR2C2	Direct	1.008	1.01044	1.10747	1.10304

-102.04 NR2F1_1. NR2F1	Direct	1.01135	1.00842 NA	0.986365

-7.17 NR2F1_2. NR2F1	Direct	NA	1.04308 NA	NA

-128.96 NR2F2_2. NR2F2	Direct	1.03365	1.03789 NA	1.05477

2

-471.04 PAX1_1.m PAX1	Direct	1.00813	1.01895	1.10645	1.00814

|  | -99.03 NR2F6_3. | NR2F6 | Direct | 0.995294 | 1.02509 0.9 | | 3288 NA | |
| --- | --- | --- | --- | --- | --- | --- | --- | --- |
| NA | NR4A1_1. | NR4A1 | Direct | 0.902803 | 0.951471 NA | | NA | |
|  | -8.22 NR4A2_3. | NR4A2 | Direct | 0.95824 | 0.974429 NA | | NA | |
|  | -16.97 NR4A3_1. | NR4A3 | Direct | 0.989971 | 0.994117 NA | | NA | |
|  | -25.54 NR5A1_1. | NR5A1 | Direct | 0.989604 | 1.02356 NA | | 1.02472 | |
| -403.51 NRL_1.mo NRL | | | Direct | 0.968212 | 1.0391 | 1.10483 | | 1.0325 |
| NA | ONECUT1 ONECUT | | 1 Direct | NA | NA | NA |  | NA |
| NA | ONECUT1 SATB1 | | Inferred | NA | NA | NA |  | NA |
| NA | ONECUT1 SATB2 | | Inferred | NA | NA | NA |  | NA |
| NA | OTP_1.mo OTP | | Direct | NA | NA | NA |  | NA |

-542.76 PAX2_1.m PAX2	Direct	1.08649	1.05825	1.12254	1.06083

-466.46 PAX2_3.m PAX2	Direct	1.06174	1.13085	1.04121	1.04164

NA	PAX4_5.m PAX4	Direct	NA	NA	NA	NA

-458.63 PAX5_2.m PAX5	Direct	NA	1.01001	1.13059	1.11186

-380.25 PAX6_1.m PAX6	Direct	1.04214	1.03454	1.10568	1.07724

-283.05 PAX8_1.m PAX8	Direct	1.03762	1.0856	1.13321	1.06164

-165.63 PAX8_2.m PAX8	Direct	1.0211 0.999446	1.15924	1.01254

NA	PDX1_2.m PDX1	Direct	NA	NA	NA	NA

-282.28 PLAGL1_1 PLAGL1	Direct	NA	1.03834 0.894691 0.959957

-405.73 PLAGL2_2 PLAGL2	ZifRC	1.019	1.01731 0.973865 0.975691

-131.7 PPARD_1. PPARD	Direct	1.01803	1.02094 0.942515 0.960042

-106.39 PPARG_1. PPARG	Direct	0.989062 0.982577 0.956359 0.937315

NA	PRDM15_ PRDM15	ZifRC	1.17907	1.12015	1.18228	1.16604

-454.7 PTF1A_1. PTF1A	Direct	1.04213	1.01276	1.03176	1.04486

-111.66 RARA_1.m RARA	Direct	1.03254	1.04213	1.12394	1.01473

-897.35 RELA_1.m RELA	Direct	0.964617	1.00214	0.97392	1.00334

-442.48 RELB_1.m RELB	Direct	0.979725 0.994807 0.989108	1.04421

-473.17 RFX1_1.m RFX1	Direct	1.01309	1.05441 0.953927	1.02089

-135.41 RFX1_2.m RFX1	Direct	1.05527	1.03862 0.935395	1.01594

-121.79 RFX2_3.m RFX2	Direct	1.0092	1.03565	1.05882 0.965418

-174 RFX3_1.m RFX3	Direct	1.02513 0.986764	1.0376	1.08624

-161.6 RFX4_2.m RFX4	Direct	0.972764	0.98822 0.806452 0.850932

-256.44 RFX5_1.m RFX5	Direct	1.02209	0.99728 0.947805	1.04808

-103.55 RFX5_2.m RFX5	Direct	1.09518 0.933128 0.853383	1.02167

-36.41 RFX6_4.m RFX6	Direct	1.09 0.972783 NA	0.967054

NA	RHOXF1_1RHOXF1 Direct	NA	1.01481 NA	NA NA	RHOXF1_1RHOXF2 Inferred	NA	1.01481 NA	NA NA	RHOXF1_1RHOXF2B Inferred	NA	1.01481 NA	NA

-1490.85 RUNX1_1. RUNX1	Direct	NA	0.992811 0.983189 0.972718

-1627.29 RUNX2_1. RUNX2	Direct	NA	0.984411	0.97298 0.942477

-658.76 RUNX3_1. RUNX3	Direct	NA	NA	1.02627	1.10516

-97.32 RXRA_3.m RXRA	Direct	1.02122	1.01524 0.917253	0.95745

-31.84 RXRA_4.m RXRA	Direct	0.992804	1.02011 NA	NA

-9.08 RXRB_1.m RXRB	Direct	0.997705 0.961325 NA	NA

-24.6 RXRG_1.m RXRG	Direct	1.0135 0.981519 NA	NA

-128.79 SIX1_1.mo SIX1	Direct	1.01223	1.02627 NA	1.03726

-267.98 SMAD1_1. SMAD9	Inferred	1.02698 0.996689	1.12123	1.14865

-293.52 SMAD2_2. SMAD2	Direct	1.00107	1.00596	1.0443	1.09481

-254.68 SMAD3_2. SMAD3	Direct	0.973643 0.981697	1.07022	1.0358

-254.68 SMAD3_2. SMAD7	Inferred	0.973643 0.981697	1.07022	1.0358

-297.2 SMAD4_1. SMAD6	Inferred	1.05198	1.02029	1.05449	1.05736

-315.51 SMARCC1 SMARCC1 Direct	0.987489 0.995626 0.879672 0.998014

-315.51 SMARCC1 TADA2A	Inferred	0.987489 0.995626 0.879672 0.998014

NA	SOX10_5. SOX10	Direct	0.957214 0.934299 NA	1.00212

NA	SOX3_1.m SOX3	Direct	0.933411 0.994223 NA	NA NA	SOX6_1.m SOX6	Direct	NA	0.979357 NA	NA

-190.59 SP3_1.mo SP3	Direct	NA	1.0597	1.01134 NA

-1456.04 SPDEF_1. SPDEF	Direct	1.04552	1.05076	1.08638	1.02275

-3087.01 SPI1_1.mo SPI1	Direct	1.01526 0.936573	1.07786	1.11579

-1152.55 SPI1_2.mo SPI1	Direct	0.973635 0.970901 0.969294	1.00559

-1744.39 SPIB_1.moSPIB	Direct	0.99934 0.988134 0.956075 0.977679

-4138.65 SPIB_2.moSPIB	Direct	1.01962 0.980303	1.0106	1.04347

-2671.28 SPIC_1.moSPIC	Direct	0.96327 0.975337	1.09347	1.05826

-158.55 STAT1_1. STAT1	Direct	1.00811	1.03006 0.994821	1.00407

-1039.02 STAT2_1. STAT2	Direct	1.03405 0.995181 0.840556 0.963461

-161.6 STAT3_1. STAT3	Direct	1.04149 0.989314	1.06866	1.01423

-261.58 TAL1_1.m TAL1	Direct	0.985262 0.998922	0.89547 0.925912

-1093.53 TCF12_1. TCF12	Direct	0.990874	1.01418	1.10131	1.06782

-612.77 TCF21_1. TCF21	Direct	0.927352 0.969395 0.930999 0.922477

-695.2 TCF3_1.m TCF3	Direct	NA	0.989178 NA	NA

-25.02 TCF7_1.m TCF7	Direct	0.984856 0.970705 NA	NA NA	TEAD1_1. TEAD2	Inferred	0.994747 0.993174 NA	NA NA	TEAD3_2. TEAD3	Direct	0.963389 0.973506 NA	NA

-91.62 TFAP2A_1 TFAP2A	Direct	1.02847	1.05031	1.07659	1.06853

-388 TFAP2A_2 TFAP2A	Direct	1.02066	1.05857 0.986509	1.0288

-182.65 TFAP2A_3 TFAP2A	Direct	1.05643	1.04374 0.998638	1.05722

NA	TFAP2D_1 TFAP2D	Direct	NA	NA	NA	NA

-251.26 TFAP2E_1 TFAP2E	Direct	1.03256	1.05244	1.07564	1.02491

-787.79 TFAP4_2. TFAP4	Direct	1.01518 0.983182 0.896034 0.886717

-2004.56 TFE3_1.m TFE3	Direct	1.09427	1.23707	1.18972	1.12207

-1628.79 TFEB_1.m TFEB	Direct	1.06322	1.1853	1.06009	1.08535

-990.21 TFEC_1.m TFEC	Direct	1.01219	1.10492	1.06245	1.07669

-196 TGIF2LX_ TGIF2LX	Direct	0.922089	1.00102	1.09632	1.07364

-37.36 TLX1_2.m TLX1	Direct	1.03509	1.03851 0.891325 0.948983

-1615.59 USF1_1.m USF1	Direct	1.0525	1.17857	1.04482	1.07942

-1291.48 USF2_1.m USF2	Direct	1.08025	1.1684	1.18704	1.0704

NA	VAX1_1.m VAX1	Direct	NA	NA	NA	NA NA	VAX2_1.m VAX2	Direct	NA	NA	NA	NA NA	VSX1_1.m VSX1	Direct	NA	NA	NA	NA

-7.79 ZBTB1_1.I ZBTB1	Indirect	1.20756	1.14466	1.24595	1.17431

-922.61 ZBTB14_1 ZBTB14	ZifRC	1.12479	1.137	1.13566	1.10203

-253.12 ZBTB18_2 ZBTB18	Direct	1.00465 0.984304 NA	0.926808

-689.63 ZBTB33_1 ZBTB33	Direct	1.11181	1.19414 0.948366	1.00594

-216.31 ZBTB42_1 ZBTB42	Direct	0.998474 0.969711 NA	0.872582

-345.78 ZBTB7A_2 ZBTB7A	Direct	NA	1.07178 0.990234 0.980582

-182.97 ZFAT_2.m ZFAT	ZifRC	1.02517	1.03573	1.01855	1.01576

NA	ZFHX2_1. ZFHX2	Direct	NA	NA	NA	NA

-586.12 ZFP57_1.mZFP57	ZifRC	1.13463	1.16814	1.05873	1.10675

-130.05 ZFP64_1.mZFP64	ZifRC	1.03259	1.05678 0.899306 0.982486

-17.07 ZFX_1.mo ZFX	Direct	NA	1.07915	1.02001	1.03274

-93.37 ZFY_1.mo ZFY	Direct	NA	1.06965 0.993396	1.02926

-302.37 ZIC4_1.mo ZIC4	Direct	NA	1.06912	1.01503	1.00883

-179.78 ZIC5_1.mo ZIC5	Direct	1.01137	1.02774 0.965395 0.998263

-184.81 ZIC5_2.mo ZIC5	Direct	1.03019	1.05714	1.01622	1.04357

-169.36 ZIC5_3.mo ZIC5	Direct	1.02915	1.02055 0.902978	1.03561

-121.55 ZNF100_1 ZNF100	ZifRC	NA	0.988654	1.07253 0.991139

-464.9 ZNF142_3 ZNF142	ZifRC	1.13925	1.16476	1.15175	1.08164

-605.14 ZNF19_1. ZNF19	ZifRC	1.04086	1.10945	1.09021	1.00082

-444.54 ZNF2_1.m ZNF2	ZifRC	1.03721	1.10258	1.13498	1.0725

-284.13 ZNF208_1 ZNF208	ZifRC	1.02104	1.07692	1.16108	1.15039

-475.14 ZNF208_2 ZNF208	ZifRC	1.05466	1.07154 0.972263	1.00727

-6.38 ZNF292_1 ZNF292	ZifRC	1.09109	1.13407	1.22389	1.16418

-295.21 ZNF296_1 ZNF296	ZifRC	0.999167	1.01613 0.982357	0.9898

-501.49 ZNF296_2 ZNF296	ZifRC	0.987203	0.9933 0.960874	1.02005

-176.22 ZNF329_1 ZNF329	ZifRC	1.04331	1.08148	1.01544 0.987948

-51.27 ZNF335_1 ZNF335	ZifRC	1.10486	1.03569	1.05549	1.08799

-128.67 ZNF343_1 ZNF343	ZifRC	0.997743	1.02861	1.08095 0.974824

-356.87 ZNF350_1 ZNF350	Direct	0.994369	1.02677 0.985447 0.991832

-893.91 ZNF433_2 ZNF433	ZifRC	1.08367	1.19565	1.03554	1.04879

-154.19 ZNF441_1 ZNF441	ZifRC	NA	0.996158	1.04138	1.07468

-378.06 ZNF445_2 ZNF445	ZifRC	1.21235	1.20903	1.1798	1.19875

-194.82 ZNF445_3 ZNF445	ZifRC	0.980787	1.07388 NA	NA

-545.89 ZNF516_2 ZNF516	ZifRC	0.995595 0.992309	1.01885	1.02219

-211.5 ZNF536_2 ZNF536	ZifRC	1.01432	0.99276 NA	1.00918

-318.46 ZNF547_1 ZNF547	ZifRC	1.09881	1.10783	1.03525	1.08678

-126.04 ZNF548_1 ZNF548	ZifRC	1.05018	1.03989	1.0906	1.06252

-185.85 ZNF550_1 ZNF550	ZifRC	1.01659	1.00486 0.963145	1.03156

NA	ZNF560_3 ZNF560	ZifRC	1.01413 NA	NA	NA

-208.52 ZNF566_1 ZNF566	ZifRC	1.00898	1.04473	1.11381	1.0189

-178.67 ZNF579_1 ZNF579	ZifRC	NA	1.1002	1.08377	1.12913

-500.98 ZNF579_3 ZNF579	ZifRC	NA	1.15417	1.00126	1.04809

-645.08 ZNF594_2 ZNF594	ZifRC	1.02666	1.02518	1.09207	1.05824

-153.82 ZNF594_3 ZNF594	ZifRC	0.996638	1.02984	1.14174	1.1227

-264.39 ZNF594_4 ZNF594	ZifRC	0.989453 0.988107	1.13932	1.14115

-122.22 ZNF597_2 ZNF597	ZifRC	0.94816 0.992762 NA	NA

-199.65 ZNF606_1 ZNF606	ZifRC	0.99182	1.01001	1.01707	1.12556

-504.33 ZNF676_1 ZNF676	ZifRC	1.00638	1.0558 0.973451 0.966051

-64.03 ZNF681_1 ZNF681	ZifRC	1.04902 0.983046 NA	0.990685

-263.95 ZNF699_1 ZNF699	ZifRC	1.01265	1.02743 0.981839 0.971375

-76.64 ZNF705D_ ZNF705D ZifRC	0.982023 0.974876 0.982071 0.938651

-213.26 ZNF716_1 ZNF716	ZifRC	1.01212	0.98721	1.04984	1.01112

-370.29 ZNF721_2 ZNF721	ZifRC	1.02595	1.06864	1.09094	1.01187

-220.16 ZNF732_1 ZNF732	ZifRC	1.06613	1.01917 0.975214	1.01139

-188.7 ZNF746_1 ZNF746	ZifRC	0.995284	1.01048	0.93593 0.950622

-222.67 ZNF763_2 ZNF763	ZifRC	0.981484 0.978948	1.00983	1.04811

-160.82 ZNF768_1 ZNF768	ZifRC	1.05692 0.973014	1.0623	1.00291

-320.6 ZNF770_2 ZNF770	ZifRC	1.02301	1.02106 0.964336	1.01775

-329.46 ZNF777_2 ZNF777	ZifRC	1.17409	1.2205	1.17918	1.10115

-269.18 ZNF781_1 ZNF781	ZifRC	1.02188	1.05684 0.957431 0.990489

-1374.05 ZNF80_2. ZNF80	ZifRC	0.989285	1.04377	1.06724	1.00946

NA	ZNF827_1 ZNF827	ZifRC	NA	1.10116 NA	NA

-225.2 ZNF836_2 ZNF836	ZifRC	1.0274	1.03632	1.21102	1.11279

-200.41 ZNF91_3. ZNF91	ZifRC	1.00947	1.03426 0.974359	1.08413

-244.39 ZSCAN25_ZSCAN25 ZifRC	0.998556	1.00832 0.959085	1.04172

b_cell..cee b_cell_fro brain..ceehbrain_hipp cd14_posi cd19positi cd34_negacd38_nega

| 1.13565 | 1.09771 | 1.05274 | 1.11362 | 1.08183 | 1.1174 | 1.16088 | 1.04715 |
| --- | --- | --- | --- | --- | --- | --- | --- |

NA	NA	NA	NA	NA	NA	NA	NA

1.26828	1.2089	1.1615	1.05091	1.1834	1.31407	1.11123	1.12847

1.38242	1.16379	1.09421	1.01102	1.14017	1.41787	1.07903	1.02142

1.37873	1.16672 NA	NA	1.1009	1.3674	1.01327	1.07792

0.939737 0.909465 0.958182 0.998625 0.966364 0.963789 0.840312 0.966796

1.31224	1.09919	1.19371	1.15534	1.14159	1.36375	1.0684	1.12334

1.26555	1.11558 NA	1.28455	1.1402	1.31514	1.05929	1.09863

1.26555	1.11558 NA	1.28455	1.1402	1.31514	1.05929	1.09863

1.08633 NA	1.31538	1.19327	1.27655	1.13928 NA	NA

0.987415 0.965317	1.07014	1.06238 0.941657 0.966226	0.97053 0.953101

0.973462 0.995635	1.00686 0.978072	1.03223 0.982771	1.25394 0.971609

1.04429	1.02326 NA	1.05992	1.00586	1.01859	0.94236 0.967672

1.00802 0.985762 NA	0.936626	1.04765	1.01554 0.941256 0.953282

1.13771 NA	NA	NA	NA	1.17488 NA	NA NA	NA	1.03928 NA	NA	NA	NA	NA

0.965476 NA	1.03339 0.994865 0.970848 0.973247 NA	NA

0.965476 NA	1.03339 0.994865 0.970848 0.973247 NA	NA

1.01504 0.988775 0.874424 0.950308 0.974415 0.968291	0.91986 0.924542

1.38247	1.24894	1.14787	1.08972	1.06823	1.3946	1.08262	1.08858

NA	NA	1.05213 NA	NA	NA	NA	NA

1.03107 0.995043 0.946535 0.952715	1.04496 0.988071	1.01507	1.02561

NA	NA	NA	NA	NA	NA	NA	NA NA	NA	NA	NA	NA	NA	NA	NA

1.00871 NA	0.937991 NA	1.0326 NA	NA	NA NA	NA	NA	NA	1.08528 NA	NA	NA NA	NA	NA	NA	1.12346 NA	NA	NA NA	NA	NA	NA	1.14967 NA	NA	NA

1.3236	1.15493	1.17375	1.09341	1.0107	1.33625	1.10656	1.06341

1.3236	1.15493	1.17375	1.09341	1.0107	1.33625	1.10656	1.06341

1.4724	1.15285	1.11635	1.08702	1.09211	1.5103	1.01311	1.07008

1.59258	1.16943	1.1329	1.08047	1.11747	1.62885	1.14439	1.0321

1.08824 NA	NA	1.19894	1.15458	1.11402 NA	NA NA	NA	NA	NA	1.02832 NA	NA	NA NA	NA	NA	NA	NA	NA	NA	NA NA	NA	1.00069 NA	NA	NA	NA	NA NA	NA	0.991462 NA	NA	NA	NA	NA

NA	1.04445	1.05809	1.01493	1.01045 NA	1.10005	1.13034

| 0.96874 | | 0.971016 | | 0.93659 | | 0.920522 | | 0.945102 | | 0.977678 | | 0.998931 | | 1.01763 | |
| --- | --- | --- | --- | --- | --- | --- | --- | --- | --- | --- | --- | --- | --- | --- | --- |
| 0.96874 | | 0.971016 | | 0.93659 | | 0.920522 | | 0.945102 | | 0.977678 | | 0.998931 | | 1.01763 | |
| 0.96874 | | 0.971016 | | 0.93659 | | 0.920522 | | 0.945102 | | 0.977678 | | 0.998931 | | 1.01763 | |
| 1.09347 | | 1.06065 | | NA |  | 1.07942 | | 0.980662 | | 1.11794 | | 0.939358 | | 0.913146 | |
| 1.11125 | | 1.03697 | | NA |  |  | 1.0367 | 1.00171 | | 1.10862 | | 0.948424 | | 0.898098 | |
|  | 1.0115 | 1.00302 | | 1.00758 | | 0.937645 | | 0.950956 | | 1.00793 | | 1.01807 | | 0.987108 | |
| 1.05213 | | 1.05539 | | 1.04719 | | 0.97473 | | 1.04115 | | 1.04341 | | 1.01828 | | 0.996665 | |
| 1.04407 | | 1.03963 | |  | 1.0233 | 1.01354 | |  | 1.0295 | 1.02958 | | 1.05362 | | 0.993215 | |
| 1.02132 | | 1.00374 | | 0.947152 | | 0.977799 | | 0.95472 | | 0.987113 | | 0.971363 | | 0.972099 | |
|  | 1.0565 | 1.02518 | | 1.02367 | | 1.02359 | | 1.05712 | |  | 1.0406 | 1.06061 | | 0.988294 | |
| 1.05158 | | 1.10804 | | 1.02406 | | 0.987785 | | 1.03996 | |  | 1.0691 | 1.03406 | | 0.995394 | |
| 1.03667 | | 1.07225 | | 0.966347 | | 0.978467 | | 1.05071 | | 1.03021 | | 0.984897 | | 0.998814 | |
| 1.04597 | | 1.13378 | | 1.13773 | | 1.04432 | | 1.06216 | | 1.05699 | | 1.03525 | | 1.17156 | |
| 1.03436 | | 1.06316 | | 1.04784 | | 0.986828 | | 1.05187 | | 1.02833 | | 1.05945 | | 1.08914 | |
| 1.00351 | | 1.06497 | | 1.02184 | | 1.01039 | | 1.03123 | | 0.998842 | | 0.997104 | | 1.08793 | |
| NA |  | NA |  | 1.05257 | | NA |  | NA |  | NA |  | NA |  | NA |  |
|  | 1.336 | 1.27617 | | 1.10845 | | 1.13343 | | 0.989878 | | 1.35926 | | 1.06007 | | 1.05631 | |
| 1.06091 | | 1.07958 | | 1.06938 | | 0.99291 | | 1.06518 | | 1.05852 | | 1.02749 | | 1.02458 | |
| 1.05389 | | 1.08071 | | 1.06579 | | 0.994063 | | 1.06146 | | 1.02318 | | 1.04656 | | 1.10282 | |
| 1.09827 | |  | 1.2542 | 0.942029 | | 0.977394 | | 1.23432 | | 1.07749 | | 0.934511 | | 1.26923 | |
| 1.00351 | | 1.05521 | | 0.932202 | | 0.903466 | |  | 1.0156 | 0.969527 | | 0.94871 | | 0.973153 | |
| 1.05674 | | NA |  | NA |  | NA |  | NA |  | 1.08741 | | NA |  | NA |  |
| NA |  | NA |  | 1.05547 | | NA |  | NA |  | NA |  | NA |  | NA |  |
| 1.01402 | | 1.03139 | |  | 1.0081 | 0.977313 | |  | 1.0444 | 0.993904 | |  | 1.0397 |  | 1.0443 |
| 1.21253 | | 1.07514 | | 1.02632 | | 1.04428 | | 1.02273 | | 1.10651 | | 1.09091 | | 1.15789 | |
|  | 1.0198 | 1.11011 | | 0.975164 | | 0.970043 | | 1.00754 | | 1.03143 | | 1.02804 | | 1.08413 | |
| 1.04757 | |  | 1.0836 | 1.09803 | | 0.992695 | | 1.04886 | | 1.03098 | | 1.03739 | | 1.13916 | |
| 1.05624 | | 1.03474 | | 1.00896 | | 0.973312 | |  | 1.1055 | 1.03639 | | 1.02275 | | 1.01837 | |
| 1.02289 | | 1.09498 | | 1.03474 | | 1.00009 | | 1.04606 | |  | 1.0354 | 1.01553 | | 1.01531 | |
| 1.02289 | | 1.09498 | | 1.03474 | | 1.00009 | | 1.04606 | |  | 1.0354 | 1.01553 | | 1.01531 | |
| 1.02924 | | 1.07338 | | 1.04496 | | 0.985791 | | 1.05494 | | 1.01167 | | 1.04441 | | 1.08428 | |
| 1.04622 | | 1.06911 | | 1.13047 | | 1.08255 | | 1.08815 | | 1.01092 | |  | 1.0415 | 1.13151 | |
| 0.994732 | | 1.08541 | |  | 0.98 | 0.978747 | |  | 1.1087 | 0.993431 | | 0.933216 | | 1.07362 | |
| 1.03452 | | 1.07469 | | 0.918346 | | 0.963515 | | 0.958236 | | 1.02452 | | 0.832465 | | 0.91343 | |
| NA |  | NA |  | 1.08418 | | NA |  | NA |  | NA |  | NA |  | NA |  |
| 1.01019 | | 1.04574 | | 1.03359 | | 1.02272 | | 0.934994 | | 1.00171 | | 1.05661 | | 1.00086 | |
| 1.04011 | | 1.08228 | | 1.05023 | | 0.981163 | | 1.04687 | | 1.02408 | | 1.03383 | |  | 1.0233 |
| 1.01673 | | 1.06263 | | 1.02887 | | 0.967837 | | 1.02996 | | 1.00498 | | 1.00908 | | 1.03922 | |
| 1.09713 | | 1.23461 | | 1.07417 | | 1.18178 | | 1.22252 | | 1.09081 | |  | 1.1174 | 1.37349 | |

| 0.999666 | 1.01664 | NA |  | 0.995552 | | 0.973533 | 1.01209 | 1.03271 | 0.958779 |
| --- | --- | --- | --- | --- | --- | --- | --- | --- | --- |
| 1.00389 | 0.992132 |  | 1.0237 | 0.973969 | | 0.956093 | 1.01258 | 0.912055 | 0.918919 |
| 1.01825 | 1.03177 | 1.03793 | | 0.995209 | | 0.939038 | 0.992376 | 0.949723 | 0.918462 |
| 0.993489 | NA | NA |  | NA |  | 0.948009 | 0.998409 | 1.08808 | NA |
| NA | NA | NA |  | NA |  | NA | NA | NA | NA |
| NA | NA | NA |  | NA |  | NA | NA | NA | NA |
| NA | NA | NA |  | NA |  | NA | NA | NA | NA |
| NA | NA | NA |  | NA |  | NA | NA | NA | NA |
| 0.976294 | NA | 1.02476 | | 0.994727 | | NA | NA | NA | NA |
| 0.985017 | NA |  | 1.0227 | 1.02674 | | NA | NA | NA | NA |
| NA | NA | NA |  | NA |  | NA | NA | NA | NA |
| NA | NA | NA |  | NA |  | NA | NA | NA | NA |
| NA | NA | NA |  | NA |  | NA | NA | NA | NA |
| NA | NA | NA |  | NA |  | NA | NA | NA | NA |
| 0.976574 | NA | 1.03229 | |  | 1.0156 | NA | NA | NA | NA |
| 0.993584 | NA | 1.03075 | | 0.98611 | | NA | 0.978617 | NA | NA |
| 0.990376 | NA | 1.00967 | | 0.968442 | | NA | 0.987967 | NA | NA |
| 0.932574 | NA | 1.01968 | | 0.958612 | | NA | 0.953259 | NA | NA |
| 0.999449 | 1.02164 | 1.05261 | |  | 1.0089 | 1.02949 | 0.967846 | NA | NA |
| 0.971236 | NA | 1.01957 | | 0.975909 | | NA | NA | NA | NA |
| NA | NA | NA |  | NA |  | NA | NA | NA | NA |
| 1.05071 | 1.05841 | 1.07791 | | 1.00515 | | 1.04553 | 1.04189 | 1.04971 | 1.03489 |
| 0.974867 | 0.95267 | 0.945055 | | NA |  | 1.00939 | 0.969998 | 1.09843 | NA |
| 0.974867 | 0.95267 | 0.945055 | | NA |  | 1.00939 | 0.969998 | 1.09843 | NA |
| 0.991224 | NA | NA |  | NA |  | NA | 0.968329 | NA | NA |
| 0.991224 | NA | NA |  | NA |  | NA | 0.968329 | NA | NA |
| 0.987881 | NA | NA |  | NA |  | NA | 0.969521 | 1.07973 | NA |
| 0.96196 | 0.966648 | NA |  | NA |  | 1.04497 | 0.971815 | 1.13145 | NA |
| 0.957359 | NA | NA |  | NA |  | NA | 0.941123 | NA | NA |
| NA | NA | NA |  | NA |  | NA | NA | NA | NA |
| NA | NA | NA |  | NA |  | NA | NA | NA | NA |
| NA | NA | NA |  | NA |  | NA | NA | NA | NA |
| NA | NA | 1.00265 | | NA |  | NA | NA | NA | NA |
| NA | NA | 1.01708 | | NA |  | NA | NA | NA | NA |
| NA | NA | 1.06049 | | NA |  | NA | NA | NA | NA |
| NA | NA | 1.05774 | | NA |  | NA | NA | NA | NA |
| 1.04139 | 0.992868 | 0.995737 | | 1.04424 | | 0.940616 | 1.01983 | 0.836943 | 0.928949 |
| 1.17854 | 1.07021 | 1.08866 | | 1.09532 | | 1.04821 | 1.18902 | 0.957483 | 1.03764 |
| 1.17854 | 1.07021 | 1.08866 | | 1.09532 | | 1.04821 | 1.18902 | 0.957483 | 1.03764 |

| 1.3819 | 1.18686 | 1.24173 | 1.16775 | 1.0671 | 1.40323 | 1.10444 | 1.10475 |
| --- | --- | --- | --- | --- | --- | --- | --- |

1.29785 NA	NA	NA	NA	1.39763 NA	0.864516

1.21226	1.08441	1.05531 0.975536	1.12879	1.23358	1.09091	1.01462

1.06602	1.00576	1.01822 0.995889	0.93241	1.06529	1.04123	0.94024

1.04907 0.998079 0.923389	0.98011	1.07649	1.06509	1.05292	1.0268

NA	NA	NA	NA	1.11709 NA	NA	NA NA	NA	NA	NA	NA	NA	NA	NA NA	NA	NA	NA	NA	NA	NA	NA NA	NA	0.943874 0.964808 NA	NA	NA	NA

1.01234 NA	NA	0.985507 NA	0.999459 NA	NA

0.982759 0.979116 0.999873	1.02023 0.935075 NA	1.00595 0.931153

NA	NA	1.08337 NA	NA	NA	NA	NA NA	NA	NA	NA	NA	NA	NA	NA NA	NA	NA	NA	NA	NA	NA	NA NA	NA	NA	NA	NA	NA	NA	NA

1.0336 NA	NA	NA	NA	1.05632 NA	NA NA	NA	1.06551 NA	NA	NA	NA	NA

0.976283 NA	1.07553	1.01375 NA	NA	NA	NA NA	NA	1.02283 NA	NA	NA	NA	NA

0.971001 NA	NA	NA	NA	NA	NA	NA NA	NA	NA	NA	NA	NA	NA	NA NA	NA	0.941377 NA	NA	NA	NA	NA NA	NA	1.0125 NA	NA	NA	NA	NA NA	NA	1.0442 NA	NA	NA	NA	NA

NA	NA	NA	NA	NA	NA	NA	NA

0.987558 NA	NA	NA	NA

0.966114 NA	NA

NA	NA	NA	NA	NA	NA	NA	NA

1.05675 NA	NA	NA	NA

1.07889 NA	NA

NA	NA	1.05909 NA	NA	NA	NA	NA NA	NA	1.0432 NA	NA	NA	NA	NA

0.973076 NA	1.03083	1.00839 NA	0.953045 NA	NA

NA	NA

1.07488 NA	NA	NA	NA	NA

NA	NA	NA	NA	NA	NA	NA	NA NA	NA	NA	NA	NA	NA	NA	NA NA	NA	NA	NA	NA	NA	NA	NA

1.08234 NA	NA	NA	NA	1.0946 NA	NA NA	NA	NA	NA	NA	NA	NA	NA

1.06228 NA	NA	NA	NA	1.08993 NA	NA

1.06378 NA	NA	NA	NA	1.07953 NA	NA

| NA |  | NA |  | 1.01192 | | NA |  | NA |  | NA |  | NA |  | NA |
| --- | --- | --- | --- | --- | --- | --- | --- | --- | --- | --- | --- | --- | --- | --- |
| 0.96342 | | 0.988682 | | NA |  | NA |  | NA |  | NA |  | NA |  | NA |
| NA |  | NA |  | 1.09578 | | NA |  | NA |  | NA |  | NA |  | NA |
| NA |  | NA |  | NA |  | NA |  | NA |  | NA |  | NA |  | NA |
| NA |  | NA |  | NA |  | NA |  | NA |  | NA |  | NA |  | NA |
| 1.11198 | | NA |  | NA |  | NA |  | NA |  | 1.10364 | | NA |  | NA |
| NA |  | NA |  | NA |  | NA |  | NA |  | NA |  | NA |  | NA |
| 1.04325 | | NA |  | NA |  | NA |  | NA |  | 1.05714 | | NA |  | NA |
| NA |  | NA |  | 1.06471 | | NA |  | NA |  | NA |  | NA |  | NA |
|  | 1.0412 | NA |  | 0.925394 | | NA |  | NA |  |  | 1.0579 | NA |  | NA |
| 0.974857 | | 1.02948 | | 1.02731 | | 0.975591 | | 0.99892 | | 0.960718 | | 0.896489 | | 0.908587 |
| 1.24719 | | 1.02917 | | 1.26124 | | 0.995752 | | 1.04884 | | 1.28399 | | NA |  | NA |
| 1.03976 | | 0.937755 | | NA |  | NA |  | 0.909689 | | 1.01145 | | NA |  | NA |
| 1.07799 | | 1.02559 | | 1.01743 | | 0.984937 | | 0.894884 | | 1.05963 | | 0.877206 | | 0.905932 |
| 1.13029 | | 1.16486 | | 1.28235 | | 1.11789 | | 1.04873 | | 1.12454 | | 1.31799 | | 1.25152 |
| 1.16046 | | 1.03912 | | 1.09517 | | 1.02679 | | 1.03423 | | 1.16343 | | 1.08397 | | 1.19341 |
| 1.26848 | | 1.06553 | | NA |  | NA |  | 1.09896 | | 1.29377 | | NA |  | 1.18935 |
| 1.10966 | | 0.980714 | |  | 1.0268 | NA |  | 0.916094 | | 1.11061 | | NA |  | NA |
| 1.10059 | | 1.05817 | | 1.02232 | | 1.00196 | | 0.956044 | | 1.09787 | | 1.07997 | | 0.977371 |
| 1.21021 | | 1.10339 | | 1.08377 | | NA |  | 1.00527 | | 1.14912 | | NA |  | 1.19753 |
| 1.02004 | | 0.962194 | | NA |  | 1.04153 | | 0.909689 | | 0.998588 | | 0.960837 | | NA |
| 1.00798 | |  | 1.0149 | NA |  | 0.976148 | | 0.923983 | | 0.986766 | | 1.07701 | | 1.00014 |
| 0.984549 | | 1.00014 | | 0.984016 | | 0.978316 | | 0.968446 | | 0.963329 | | 0.957634 | | 0.994253 |
| 0.981159 | | NA |  | NA |  | NA |  | 0.952843 | | 0.980618 | | NA |  | NA |
| 0.996071 | | 1.03046 | | NA |  | NA |  | NA |  | 1.01835 | | NA |  | NA |
| 1.04232 | | 1.08484 | | 1.11591 | | 1.02195 | | 1.10133 | | 1.03048 | | NA |  | 1.04052 |
| 1.11701 | | 1.07874 | | NA |  | NA |  | 1.09844 | |  | 1.1325 |  | 1.1025 | 1.09551 |
| 1.20199 | | 1.04565 | | NA |  | NA |  | 1.15349 | |  | 1.2524 | 1.04188 | | 1.07143 |
| 1.20199 | | 1.04565 | | NA |  | NA |  | 1.15349 | |  | 1.2524 | 1.04188 | | 1.07143 |
| 1.16516 | | 1.02023 | | NA |  | NA |  | 1.03956 | | 1.19086 | | 1.15343 | | 1.16348 |
| 1.08169 | | 1.01626 | | NA |  | NA |  |  | 1.002 | 1.06247 | | 0.859682 | | 0.931421 |
| 1.04182 | | 1.01969 | | NA |  | NA |  | 1.07144 | |  | 1.0574 | NA |  | NA |
| 1.04182 | | 1.01969 | | NA |  | NA |  | 1.07144 | |  | 1.0574 | NA |  | NA |
| 1.04182 | | 1.01969 | | NA |  | NA |  | 1.07144 | |  | 1.0574 | NA |  | NA |
|  | 1.0357 | 1.01714 | | NA |  | NA |  | 1.01919 | | 1.05442 | | 1.12463 | | 0.956718 |
|  | 1.0074 | 0.992136 | | 0.952634 | |  | 1.0083 | 0.929299 | | 1.01555 | | 0.999183 | | 0.908979 |
|  | 1.11 | 1.08422 | | NA |  | NA |  | NA |  | 1.12378 | | NA |  | NA |
| NA |  | NA |  | 0.966082 | | 1.02455 | | NA |  | NA |  | NA |  | NA |
| 1.01722 | | 1.04856 | |  | 1.0247 | 0.966742 | | NA |  | 1.00039 | | NA |  | NA |

| NA | NA |  | 0.990675 | | NA |  | NA | NA |  | NA |  | NA |  |
| --- | --- | --- | --- | --- | --- | --- | --- | --- | --- | --- | --- | --- | --- |
| NA | NA |  | 1.12357 | | NA |  | NA | NA |  | NA |  | NA |  |
| NA | NA |  | 1.10366 | | NA |  | NA | NA |  | NA |  | NA |  |
| NA | NA |  | 1.07112 | | NA |  | NA | NA |  | NA |  | NA |  |
| 0.955339 | 0.920115 | | 1.02437 | | 1.02728 | | 0.98263 | 0.948175 | | 1.02723 | | 1.06003 | |
| 1.01428 | 0.936359 | | 1.04832 | | 1.01705 | | 1.07433 | 1.00177 | |  | 1.1016 | 1.02596 | |
| 1.04659 | 1.00595 | | 0.973154 | | 0.970454 | | 0.976551 | 1.04253 | |  | 1 | 0.996564 | |
| 1.04344 | 1.01344 | | 1.00561 | | 1.03064 | | 1.02781 | 1.02759 | | 1.07831 | | 1.17541 | |
| NA | 1.01442 | | 1.00822 | | 1.07305 | | 0.929424 | NA |  | 0.910836 | | 0.872093 | |
| NA | 1.03384 | | 1.07952 | | 1.11081 | | 1.04715 | NA |  | 0.878096 | | 1.03492 | |
| 1.26952 |  | 1.1444 | 1.12123 | | 1.03898 | | 1.03697 | 1.29829 | | 1.12118 | | 1.08996 | |
| NA | 1.09234 | | 1.33861 | | 1.17333 | | 1.26775 | NA |  | 1.10884 | | 1.00978 | |
| NA | 1.14788 | |  | 1.3283 | 1.15796 | | 1.29436 | NA |  | 1.29898 | | 1.17236 | |
| 0.965947 | 0.94743 | | 1.01544 | | 0.98592 | | 0.909546 | 0.939594 | | 0.969745 | | 0.870685 | |
| NA | NA |  | 1.03683 | | 1.04923 | | NA | NA |  | NA |  | NA |  |
| NA | NA |  | 1.07267 | | NA |  | NA | NA |  | NA |  | NA |  |
| 1.30486 | 1.17206 | | NA |  | 1.09603 | | 1.06173 | 1.36242 | | 1.01247 | | 1.02687 | |
| NA | NA |  |  | 1.0517 | NA |  | NA | NA |  | NA |  | NA |  |
| 0.950329 | 0.971037 | | 1.05731 | | 1.09858 | | 0.946793 | 0.959334 | | 0.937243 | | 0.994688 | |
| NA | NA |  | 1.03428 | | 0.968166 | | NA | NA |  | NA |  | NA |  |
| NA | NA |  | 1.03428 | | 0.968166 | | NA | NA |  | NA |  | NA |  |
| 1.16966 | 1.14827 | | 1.14953 | | 1.07967 | | 1.17347 |  | 1.1702 | 1.13321 | | 1.26572 | |
| 1.27511 | 1.15822 | | 1.14708 | |  | 1.0706 | 1.04624 | 1.31859 | | 1.16269 | | 1.17041 | |
| 1.27511 | 1.15822 | | 1.14708 | |  | 1.0706 | 1.04624 | 1.31859 | | 1.16269 | | 1.17041 | |
| 1.27511 | 1.15822 | | 1.14708 | |  | 1.0706 | 1.04624 | 1.31859 | | 1.16269 | | 1.17041 | |
| 1.27511 | 1.15822 | | 1.14708 | |  | 1.0706 | 1.04624 | 1.31859 | | 1.16269 | | 1.17041 | |
| 1.05901 | 1.11404 | | 1.03852 | | 1.00881 | | 1.10042 | 1.06453 | | 1.09688 | | 1.02483 | |
| 1.05901 | 1.11404 | | 1.03852 | | 1.00881 | | 1.10042 | 1.06453 | | 1.09688 | | 1.02483 | |
| 1.05901 | 1.11404 | | 1.03852 | | 1.00881 | | 1.10042 | 1.06453 | | 1.09688 | | 1.02483 | |
| 1.05901 | 1.11404 | | 1.03852 | | 1.00881 | | 1.10042 | 1.06453 | | 1.09688 | | 1.02483 | |
| 1.25784 | 1.19475 | | 1.14767 | | 1.15199 | | 1.06855 | 1.27474 | | 1.09181 | | 1.00726 | |
| 1.25784 | 1.19475 | | 1.14767 | | 1.15199 | | 1.06855 | 1.27474 | | 1.09181 | | 1.00726 | |
| 1.16432 | 1.08396 | | 1.05556 | | 1.04622 | | 1.01037 | 1.18925 | | 0.778727 | | 0.947368 | |
| 1.16432 | 1.08396 | | 1.05556 | | 1.04622 | | 1.01037 | 1.18925 | | 0.778727 | | 0.947368 | |
| 1.43191 | 1.19084 | | 1.14696 | | 1.07976 | | 1.06148 | 1.50382 | | 1.17108 | | 1.05963 | |
| 0.938301 | 0.925435 | | 1.00155 | |  | 1.006 | 0.922708 | 0.940151 | | 0.982862 | | 1.04248 | |
| 0.938301 | 0.925435 | | 1.00155 | |  | 1.006 | 0.922708 | 0.940151 | | 0.982862 | | 1.04248 | |
| 0.978387 | 0.968598 | | 0.978386 | | 1.03385 | | 1.02539 | 0.978968 | | 0.840617 | |  | 1.0551 |
| 0.978387 | 0.968598 | | 0.978386 | | 1.03385 | | 1.02539 | 0.978968 | | 0.840617 | |  | 1.0551 |

| 0.970961 | | 0.953127 | | 1.00441 | 1.06247 | | 0.995959 | | 0.965353 | | 0.959432 | 1.04479 |
| --- | --- | --- | --- | --- | --- | --- | --- | --- | --- | --- | --- | --- |
| 0.969568 | | 0.968248 | | 0.986118 | 1.05089 | | 1.01012 | | 0.983366 | | 0.987322 | 0.987228 |
| 1.00285 | | 0.985027 | | 0.977031 | 0.985803 | | 0.963504 | | 0.986117 | | 0.912863 | 0.952153 |
| 0.952854 | | 0.982986 | | 1.02545 | 1.07492 | | 0.927761 | | 0.964038 | | 1.03779 | 0.934122 |
| 0.975625 | | 0.981617 | | 0.982701 |  | 1.0645 | 1.01003 | | 0.980642 | | 0.906067 | 0.938849 |
| 0.989085 | | NA |  | NA | 0.990325 | | 0.941243 | | 0.991196 | | 0.894671 | NA |
| 0.987657 | | 0.986865 | | 0.964825 | 1.03997 | | 1.05749 | | 0.987042 | | 0.874416 | 0.885794 |
| NA |  | 1.04562 | | 0.992791 | 0.970113 | | 0.97247 | | NA |  | 0.992857 | 0.964533 |
| 1.00701 | | 1.03847 | | 0.993051 | 0.971248 | | 1.01745 | | NA |  | 1.04838 | 1.01263 |
| NA |  | NA |  | 1.06904 | 1.07293 | | 0.993555 | | NA |  | NA | NA |
| 1.01991 | |  | 1.0138 | 0.979131 | 0.948081 | | 0.969131 | | 1.00316 | | 0.946542 | 0.929402 |
| 0.992764 | | 1.05798 | | 1.03344 | 1.01063 | | 0.971621 | | 0.971445 | | 0.923889 | 0.977828 |
| 1.00821 | | 0.980741 | | NA | NA |  | NA |  |  | 1.014 | NA | NA |
| 0.997223 | | 1.03161 | | NA | 0.978028 | | 1.04586 | | 0.996129 | | 1.05635 | 1.02971 |
| 1.04114 | | 0.965078 | | NA | NA |  | NA |  | 1.04853 | | NA | NA |
| NA |  | NA |  | NA | NA |  | NA |  | NA |  | NA | NA |
| NA |  | NA |  | NA | NA |  | NA |  | NA |  | NA | NA |
| 0.966304 | | 1.03678 | | 0.94562 | 0.958318 | | NA |  | 0.978217 | | NA | NA |
| NA |  | NA |  | NA | NA |  | NA |  | NA |  | NA | NA |
| NA |  | NA |  | NA | NA |  | NA |  | NA |  | NA | NA |
| NA |  | NA |  | NA | NA |  | NA |  | NA |  | NA | NA |
| NA |  | NA |  | NA | NA |  | NA |  | NA |  | NA | NA |
| 0.980226 | | NA |  | NA | NA |  | NA |  | 0.952904 | | NA | NA |
| 0.957744 | | 0.993182 | | NA | NA |  | NA |  | 0.966539 | | NA | NA |
| 0.958618 | | 1.04417 | | NA | NA |  | 0.994156 | | 0.971014 | | 1.05013 | NA |
| NA |  | NA |  | 1.06374 | NA |  | NA |  | NA |  | NA | NA |
| NA |  | NA |  | 1.06717 | NA |  | NA |  | NA |  | NA | NA |
| NA |  | NA |  | 0.994364 | NA |  | NA |  | NA |  | NA | NA |
|  | 1.3591 | 1.18839 | | 1.12714 | 1.04266 | | 1.05108 | | 1.46975 | | 1.10567 | 1.07578 |
| 1.09724 | | 1.02386 | | 1.10435 | 1.11428 | |  | 1.058 | 1.11694 | | 1.04436 | 1.04111 |
| 1.09724 | | 1.02386 | | 1.10435 | 1.11428 | |  | 1.058 | 1.11694 | | 1.04436 | 1.04111 |
| NA |  | NA |  | NA | NA |  | NA |  | 0.926108 | | NA | NA |
| NA |  | NA |  | NA | NA |  | NA |  | NA |  | NA | NA |
| NA |  | NA |  | NA | NA |  | NA |  | 0.993319 | | NA | NA |
| 1.02788 | | 0.976772 | | NA | NA |  | NA |  | 1.01333 | | NA | NA |
| 1.07627 | | 1.04005 | | 1.10009 | 0.982495 | | 1.10603 | |  | 1.0721 | 1.02641 | 1.06678 |
| 0.987916 | | NA |  | NA | NA |  | NA |  | 0.974288 | | NA | NA |
| NA |  | NA |  | NA | NA |  | NA |  | 1.04679 | | NA | NA |
| 1.02321 | | 1.02691 | | NA | NA |  | NA |  |  | 1.0212 | NA | NA |

| 0.984229 | | 0.972941 | | NA |  | NA | NA |  | 0.981089 | | NA |  | NA |  |
| --- | --- | --- | --- | --- | --- | --- | --- | --- | --- | --- | --- | --- | --- | --- |
| 0.964788 | | NA |  | NA |  | NA | NA |  | 0.949544 | | NA |  | NA |  |
| 0.982229 | | NA |  | NA |  | NA | NA |  | 0.975291 | | NA |  | NA |  |
| NA |  | NA |  | NA |  | NA | NA |  | 0.996559 | | NA |  | NA |  |
| NA |  | NA |  | NA |  | NA | NA |  | NA |  | NA |  | NA |  |
| 1.01249 | | 0.939547 | |  | 1.1119 | 1.03361 | 1.04572 | | 1.01128 | | 1.02985 | | 1.00121 | |
| NA |  | NA |  | NA |  | NA | NA |  | NA |  | NA |  | NA |  |
| NA |  | NA |  | NA |  | NA | NA |  | NA |  | NA |  | NA |  |
| NA |  | NA |  | NA |  | NA | NA |  | NA |  | NA |  | NA |  |
| NA |  | NA |  | NA |  | NA | NA |  | NA |  | NA |  | NA |  |
|  | 1.0328 | 0.976894 | | 0.741117 | | 0.956044 | 0.99918 | | 1.07647 | | 0.946695 | | 1.04505 | |
|  | 1.0575 | 1.01854 | | NA |  | 1.07926 | 1.05257 | | 1.07371 | | NA |  | 1.01381 | |
| 1.09904 | | 1.07112 | | 1.06286 | | 1.01559 | 1.11245 | | 1.09639 | | 1.05427 | | 0.982906 | |
| NA |  | NA |  | 1.03134 | | NA | NA |  | NA |  | NA |  | NA |  |
| 1.06378 | | 1.00221 | | 0.856004 | | 1.00693 | 1.08467 | |  | 1.0789 | 1.02088 | | 0.98505 | |
| 1.01998 | | 1.00396 | | 1.00209 | | 1.01347 | 1.02938 | | 1.03246 | | NA |  | 1.06748 | |
| 1.07173 | | 1.02869 | | 0.993835 | | 0.91524 | 1.02509 | | 1.09361 | | NA |  | 0.995095 | |
| 0.993471 | | 0.893806 | | 0.896306 | | 0.98856 | 1.01515 | | 0.998266 | | 0.954598 | | 0.976214 | |
| NA |  | NA |  | 1.01844 | | NA | NA |  | NA |  | NA |  | NA |  |
| NA |  | 0.984532 | | NA |  | 1.01333 | 0.963158 | | 1.05246 | | 0.851322 | | 0.899549 | |
| 1.02076 | |  | 1.0457 | 0.959318 | | 0.994345 | 0.992658 | |  | 1.0257 | 1.02867 | | 1.02422 | |
| 0.959989 | | 0.958995 | | NA |  | 0.953569 | 0.986836 | | 0.954196 | | NA |  | NA |  |
| 0.959822 | | 0.960276 | | NA |  | 0.985423 | 0.911616 | | 0.963448 | |  | 0.96 | NA |  |
| NA |  | 1.18579 | | 1.17508 | | 1.11362 | 1.17929 | | NA |  | 1.07497 | | 1.13569 | |
|  | 1.008 | 1.00351 | | 0.910913 | | 0.965247 |  | 1.0402 | 1.00778 | | 1.10386 | | 1.05577 | |
| 1.06904 | | 0.993282 | | NA |  | NA |  | 0.982 | 1.05453 | | NA |  | NA |  |
| 1.02199 | | 1.07711 | | NA |  | 0.941797 | 1.04365 | | 0.984283 | | 1.07932 | | 1.03266 | |
| 1.01144 | | 1.06747 | | 0.979114 | | 0.963236 |  | 1.0506 | 1.00522 | | 1.05259 | | 1.08566 | |
| 1.07183 | | 0.967484 | | 0.991199 | | 1.02557 | 1.04457 | | 1.07399 | | 0.902878 | | 0.918058 | |
| 1.03035 | | 1.00224 | | 1.06717 | | 1.05266 | 1.01248 | | 1.03997 | | 0.895897 | | 0.908704 | |
|  | 1.0933 | 1.04516 | | 1.14388 | | 1.06278 | 1.02586 | | 1.03614 | |  | 1.05 |  | 1.1087 |
| 1.05189 | | 1.02074 | | 1.09844 | | 1.02508 | 1.03022 | | 1.05435 | | 0.973077 | | 1.09477 | |
| 1.03567 | | 1.01128 | | 1.11283 | | 1.00676 | 0.94533 | | 1.02845 | | 0.763158 | | 0.801887 | |
| 1.02334 | | 1.04504 | | 1.08182 | | 1.05189 | 1.05763 | | 1.01884 | | 1.00593 | | 0.928693 | |
|  | 1.0116 | 0.94391 | | 1.14124 | | 1.11159 |  | 1 | 0.985061 | | 1.06429 | | 0.857895 | |
| 1.02728 | | NA |  | 1.05145 | | 0.977909 | 0.872881 | | 0.985838 | | NA |  | 0.916667 | |
| NA |  | NA |  | NA |  | NA | NA |  | NA |  | NA |  | NA |  |
| NA |  | NA |  | NA |  | NA | NA |  | NA |  | NA |  | NA |  |
| NA |  | NA |  | NA |  | NA | NA |  | NA |  | NA |  | NA |  |

| 0.989028 | | 1.00686 | | NA |  | NA |  | 0.950982 | | 0.980445 | | 0.969735 | | 0.919674 | |
| --- | --- | --- | --- | --- | --- | --- | --- | --- | --- | --- | --- | --- | --- | --- | --- |
| 0.982231 | | 1.00891 | | NA |  | 0.941851 | | 0.941952 | | 0.982611 | | 0.930967 | | 0.928564 | |
| 1.02287 | | 1.08594 | | NA |  | 0.986994 | | 1.12217 | | 1.02374 | | 1.07116 | |  | 1.0514 |
| 1.00329 | | 1.01457 | | NA |  | NA |  | NA |  | 0.991768 | | NA |  | NA |  |
| NA |  | NA |  | NA |  | NA |  | NA |  | 1.02064 | | NA |  | NA |  |
| NA |  | NA |  | NA |  | NA |  | NA |  |  | 1.0027 | NA |  | NA |  |
| NA |  | NA |  | NA |  | NA |  | NA |  | 1.03344 | | NA |  | NA |  |
| 1.04094 | | NA |  | NA |  | NA |  | 1.13866 | | 1.04145 | | NA |  | NA |  |
| 0.965624 | | 1.07371 | | 1.00534 | | 0.97558 | |  | 1.0607 | 0.960011 | | 1.14193 | | 1.12203 | |
| 1.03234 | | 0.974681 | | 0.905366 | | 0.968529 | | 0.998728 | | 0.994627 | | 1.08196 | | 1.04903 | |
|  | 1.0196 | 1.05453 | | 0.884231 | | 0.976913 | | 0.987508 | | 0.985581 | |  | 1.0099 | 0.964626 | |
|  | 1.0196 | 1.05453 | | 0.884231 | | 0.976913 | | 0.987508 | | 0.985581 | |  | 1.0099 | 0.964626 | |
| 1.04691 | | 1.02464 | | 0.971731 | | 1.02592 | | 1.04952 | |  | 1.0324 | 1.07176 | | 1.06463 | |
| 0.995607 | | 1.04023 | | NA |  | 1.00581 | | 0.882579 | | 0.984877 | | 0.983124 | | 0.861194 | |
| 0.995607 | | 1.04023 | | NA |  | 1.00581 | | 0.882579 | | 0.984877 | | 0.983124 | | 0.861194 | |
| 0.949652 | | NA |  | 0.919065 | | 0.967267 | | NA |  | 0.95416 | | NA |  | NA |  |
| 0.940664 | | NA |  | 0.985807 | | 0.997377 | | NA |  | 0.947177 | | NA |  | NA |  |
| NA |  | NA |  | 0.96655 | | 1.02871 | | NA |  | NA |  | NA |  | NA |  |
| 1.11247 | | 1.01293 | | NA |  | NA |  | 1.08919 | | 1.11854 | | 0.982537 | | 0.924899 | |
| 1.03737 | | 1.05313 | | 1.15539 | | 1.04686 | | 1.01687 | | 1.03822 | | 0.962524 | | 1.00818 | |
| 1.03484 | | 1.06083 | | 0.900745 | | 0.96069 | | 1.11206 | |  | 1.0121 | 1.00518 | |  | 1.1069 |
| 0.963572 | |  | 1.004 | 0.947617 | | 0.940767 | | 1.02451 | | 0.941558 | | 0.989764 | | 1.06829 | |
| 0.98223 | | 0.971379 | | 0.931645 | | 0.979012 | | 1.03508 | | 0.987753 | | 0.973299 | | 1.00787 | |
| 1.04862 | | 1.05783 | | 0.935352 | | 0.986532 | | 1.02572 | | 1.02038 | | 0.962358 | |  | 1.0768 |
| 1.02253 | | 1.03192 | | 1.01244 | | 0.98049 | | 1.05425 | | 1.00841 | | 0.99027 | |  | 1.0169 |
| 1.01861 | | 1.01015 | | 1.04415 | | 1.02773 | |  | 1.0347 | 1.01965 | | 0.995807 | | 0.971515 | |
|  | 1.0361 | 1.01711 | | 0.942047 | | 0.955022 | | 0.951569 | | 1.01882 | | NA |  | 0.912262 | |
| 0.995793 | |  | 1.0508 |  | 1.0439 | 1.05064 | | 0.996448 | | 0.980728 | | 0.971014 | | 1.05313 | |
| 1.01301 | | 0.936179 | | 1.03198 | | 0.97539 | | 0.847893 | | 1.02705 | | NA |  | 0.853982 | |
| 0.964196 | | 1.01693 | | 1.01698 | | 1.01762 | |  | 1.0547 | 1.00357 | | 0.954383 | | 1.02522 | |
| 0.939012 | | 0.905255 | | 0.99995 | | 1.04502 | | 0.929852 | | 0.941442 | | 0.862179 | | 0.898257 | |
| 0.984415 | | 0.921214 | | 1.04273 | | 1.06959 | | NA |  | 0.991679 | | NA |  | 0.99083 | |
| 0.974499 | | NA |  | 1.04825 | | 0.997041 | | NA |  | 0.946944 | | NA |  | NA |  |
| NA |  | NA |  | NA |  | NA |  | NA |  | 0.982625 | | NA |  | NA |  |
| NA |  | NA |  | NA |  | NA |  | NA |  | NA |  | NA |  | NA |  |
| NA |  | 1.09957 | | 0.99716 | | 1.05374 | | 1.05162 | | NA |  |  | 1.0197 | 1.06309 | |
|  | 1.0124 |  | 1.0965 | 0.938021 | |  | 1.0087 | 0.97827 | | 1.03871 | | 1.13387 | | 0.952353 | |
| NA |  | 1.03766 | | 1.00095 | | 1.01929 | | 0.962957 | | NA |  | 1.10052 | | 0.990051 | |
| NA |  | NA |  | NA |  | NA |  | NA |  | NA |  | NA |  | NA |  |

| NA | 1.07458 | | 0.940017 | |  | 1.0232 | 1.01531 | | NA |  | 1.23967 | | 1.07056 | |
| --- | --- | --- | --- | --- | --- | --- | --- | --- | --- | --- | --- | --- | --- | --- |
| 0.945739 | 0.926076 | |  | 0.979 | 0.994294 | | 0.907436 | | 0.963968 | | 0.833516 | | 0.816416 | |
| 1.25691 | 1.15897 | | 1.10346 | | 1.08186 | | 1.05378 | | 1.29383 | | 1.04686 | | 1.04959 | |
| 1.23425 | 1.12653 | | 1.05473 | | 1.05761 | |  | 1.0488 |  | 1.2596 | 0.968949 | | 1.02286 | |
| 1.14807 | 1.07265 | | 0.999699 | | 1.03927 | | 1.02798 | | 1.16512 | | 0.928842 | | 1.00973 | |
| 0.957913 | 0.905083 | | 1.07156 | | 0.972264 | |  | 1.0186 | 0.912513 | | 1.17647 | | 1.02411 | |
| NA | 1.01282 | |  | 1.0381 | 0.997717 | | 0.901522 | | NA |  | 0.937619 | | 0.876432 | |
| 1.21468 |  | 1.0991 | 1.05234 | | 1.04751 | | 1.04965 | | 1.26305 | | 0.949717 | | 1.04174 | |
| 1.20218 | 1.09976 | | 1.08358 | | 1.04806 | | 1.10288 | | 1.21744 | | 1.00359 | | 1.16477 | |
| NA | NA |  | 1.02064 | | NA |  | NA |  | NA |  | NA |  | NA |  |
| NA | NA |  | 1.05105 | | NA |  | NA |  | NA |  | NA |  | NA |  |
| NA | NA |  | 0.985238 | | NA |  | NA |  | NA |  | NA |  | NA |  |
| NA | 1.17382 | | 1.09913 | | 1.11242 | | 1.16052 | | 1.13219 | | 1.12417 | | 1.14307 | |
| 1.22725 | 1.10246 | | 1.08485 | | 1.09802 | | 1.05451 | | 1.23818 | |  | 1.096 | 1.02926 | |
| 0.980291 | 0.983995 | | 1.01085 | | 1.05016 | | 0.886997 | | 0.976994 | | NA |  | 0.987242 | |
| 1.17101 | 1.06062 | | 1.13908 | | 1.12218 | | 0.997965 | | 1.20278 | | 0.882468 | | 1.00782 | |
| 0.958955 | 0.956311 | | 1.00009 | | 1.05833 | | 0.864573 | | 0.956054 | | NA |  | 0.94015 | |
| 1.13201 | 1.04255 | | NA |  | NA |  | NA |  | 1.14446 | | 0.917014 | | 0.965237 | |
| 1.04224 | 1.01987 | | 1.01709 | | 0.989719 | | 1.02294 | | 1.04604 | | 1.02816 | | 1.01435 | |
| NA | NA |  | 1.08251 | | NA |  | NA |  | NA |  | NA |  | NA |  |
| 1.16422 |  | 1.121 | 1.21864 | | 1.10292 | | 1.13389 | | 1.16772 | | 1.05298 | |  | 1.0642 |
| 0.994377 | 0.990375 | | 0.996845 | | 1.00157 | | 0.939464 | | 1.01615 | | NA |  | NA |  |
| NA | NA |  | NA |  | NA |  | NA |  | NA |  | 1.10317 | | 0.973312 | |
| NA | NA |  | NA |  | NA |  | NA |  | 1.10124 | | 1.03628 | | 0.950824 | |
| 1.08865 | 1.04055 | | 1.10251 | | 1.03265 | | 0.935906 | | 1.08603 | | 0.962726 | | 0.882149 | |
| NA | 0.996595 | | 1.00102 | | 0.996229 | | 1.00462 | | NA |  | 0.90239 | | 0.942609 | |
| NA | 1.01209 | | 1.03533 | | 1.02925 | | 0.952646 | | 1.08765 | | 0.985112 | | 0.895908 | |
| NA | 1.08911 | | 1.02827 | | 1.01229 | | 0.987909 | | NA |  | 1.08979 | | 1.07039 | |
| 1.00506 | 0.996554 | | 0.978143 | | 0.935023 | | 0.962392 | | 1.03131 | | 1.01273 | | 0.974654 | |
| 1.24765 | 1.15048 | |  | 1.181 | 1.12416 | | 1.07948 | | 1.20696 | | 0.932075 | | 1.02329 | |
| 1.13396 | 1.12936 | | 0.996721 | | 1.02831 | | 1.01089 | | 1.13844 | | 0.990079 | | 1.06375 | |
| 1.13576 | 1.17362 | | 1.16907 | | 1.10501 | | 1.14028 | | 1.11115 | | 1.30638 | |  | 1.2203 |
| 1.06459 | 1.02665 | | 1.12915 | | 1.06024 | | 1.14229 | | 1.05343 | | 0.941527 | | 1.05337 | |
| 1.05061 | 0.99541 | | 1.12349 | | 1.10005 | | 0.962911 | | 1.08686 | | 1.01339 | | 1.07393 | |
| 1.16259 | 1.13416 | | 0.993786 | | 1.02063 | | 1.24157 | | 1.22417 | | 1.22953 | | 1.22816 | |
| 0.989399 | 1.01542 | | 0.978513 | | 0.980174 | | 1.01643 | | 0.993328 | | 1.00839 | | 0.980043 | |
| 0.963985 | 1.07465 | | 0.995909 | | 0.947855 | | 1.05676 | | 0.967537 | | 0.946926 | | 1.05081 | |
| 1.08383 | 0.965882 | | 0.985012 | | 1.14849 | |  | 1.0141 | 1.06658 | | 0.971963 | | 0.983784 | |
| NA | 1.03366 | |  | 1.1287 | 1.08283 | | 1.14024 | | 1.10887 | | 1.20457 | | 1.01787 | |

| NA |  | 1.11977 | | 1.08321 | | 1.01028 | 1.01871 | | NA |  | 0.934307 | | 0.954839 |
| --- | --- | --- | --- | --- | --- | --- | --- | --- | --- | --- | --- | --- | --- |
|  | 1.0486 | 1.02122 | | 1.03122 | | 0.998174 | 1.02495 | | 1.03339 | | 1.17079 | | 1.10536 |
| 1.21231 | |  | 1.0905 | 1.09248 | | 1.04371 | 1.07778 | | 1.21574 | | 1.20457 | | 1.13269 |
| NA |  |  | 1.0242 | 1.05595 | | 1.03355 | 1.01101 | | 1.03732 | | 0.874286 | | 0.980769 |
| 1.16107 | | 1.22791 | | 1.26886 | | 1.13385 | 1.24808 | | 1.22745 | | 1.09676 | | 1.06802 |
| 1.09571 | | NA |  |  | 1.0179 | NA | NA |  | 1.16034 | | NA |  | NA |
| 0.981179 | | 0.992141 | | 1.00226 | | 1.00303 |  | 1.0251 | 0.984096 | | 1.04923 | | 1.05665 |
| 0.955051 | | 0.973538 | | 1.04103 | | 1.03348 | 1.00699 | | NA |  | NA |  | NA |
| 1.10087 | | 1.10189 | | 1.07959 | | 1.07207 | 1.09752 | | 1.12825 | | 0.892157 | | 0.978947 |
| 0.992011 | | 1.05856 | | 0.925144 | | 1.00137 | 1.05775 | | NA |  | 1.00602 | | 0.923077 |
| NA |  | 0.98847 | | 1.04377 | | 1.12215 |  | 1.0086 | 0.982684 | | 0.929825 | | 1.03185 |
| NA |  | NA |  | NA |  | NA | NA |  | NA |  | NA |  | NA |
| 1.02892 | | 1.05506 | | 1.02005 | | 1.01966 | 0.910481 | | 1.02911 | | 0.913354 | | 1.03867 |
| 1.10583 | | 1.06275 | | NA |  | NA | 1.06973 | | 1.09809 | | 1.11382 | | 1.12571 |
| 1.22131 | | 1.09738 | | 1.04376 | | 1.05066 | 1.11786 | | 1.20974 | | 1.05366 | | 1.09401 |
| 1.02439 | | 1.01561 | | 1.01412 | | 1.00887 |  | 1.0563 | 1.01754 | | 1.04321 | | 1.08816 |
| 1.01191 | | 1.07474 | | 1.04873 | | 1.00136 |  | 1.1146 |  | 1.0233 | 1.13458 | | 1.15221 |
| 0.994629 | | 0.99482 | | 0.935696 | | 1.02521 | 1.03949 | | 0.991584 | | 1.06046 | | 1.11486 |
| NA |  | NA |  | 0.96804 | | NA | NA |  | 0.987432 | | NA |  | NA |
| 1.00053 | | 1.03575 | | 0.964312 | | 0.992331 | 1.04116 | | 0.996423 | | 0.954327 | | 1.14437 |
| 1.06529 | | 1.02918 | | 0.925836 | | 1.02923 | 1.05236 | | 1.07209 | | 1.06061 | | 1.09322 |
| 1.01439 | | 0.91301 | | 1.02173 | | NA | 1.00323 | | 1.02289 | | NA |  | NA |
| 1.00768 | | 0.961884 | | 1.06293 | | 0.97652 | 1.00313 | | 1.00258 | |  | 1.0198 | 0.933722 |
| 0.998609 | | 0.976765 | | 1.00175 | | 0.930493 | 0.956038 | | 0.989862 | | 1.05075 | | 1.01402 |
| 0.991747 | | 1.02284 | | 1.03476 | | 1.00264 | 1.02326 | | 0.974199 | | NA |  | NA |
| 1.09262 | | 1.08978 | | 1.08967 | | 1.02913 | 1.03941 | | 1.07243 | | 1.01803 | | 1.22258 |
| 1.01316 | | 1.03082 | | 1.13196 | | 1.14156 | 1.03217 | | 1.01083 | | 0.992647 | | 1.00616 |
| 1.07948 | | 1.03118 | | 0.990224 | | 0.990668 | 0.982051 | | 1.08008 | | 0.966221 | | 0.984762 |
| 0.987117 | | 0.961076 | | 0.94554 | | 0.960904 |  | 0.9379 | 0.988754 | | 0.986968 | | 0.914195 |
| 0.971281 | | 0.917792 | | 1.04148 | | 1.02989 | 0.902899 | | 1.01775 | | NA |  | 1.11735 |
| 1.00447 | | 1.01229 | | 0.96844 | | 0.995719 | 1.02186 | | 1.00976 | | 1.01011 | | 0.999598 |
| 1.26346 | | 1.17665 | | 1.18465 | | 1.11959 | 1.13316 | | 1.30379 | | 1.11494 | | 1.21093 |
| 1.07319 | | 0.991348 | | 0.973087 | | 0.99634 | 0.991259 | | 1.04621 | | 1.10833 | | 0.990887 |
| 1.07478 | | 1.08809 | | NA |  | 0.972727 | 1.10349 | | 1.05492 | | 1.08902 | | 1.04532 |
| 1.14487 | | 1.13582 | | NA |  | NA | 1.11983 | | 1.19008 | | NA |  | NA |
| 1.01543 | | 1.03698 | | 1.06792 | | 0.990377 | 1.03162 | | 1.00933 | | 1.06881 | | 1.10066 |
| 1.06457 | | 1.07198 | | 1.01182 | | 0.906499 | 0.990551 | | 1.05085 | | 0.849624 | | 0.955882 |
| 1.04664 | | 1.03732 | | 1.05733 | | 1.01379 | 1.11174 | | 1.05071 | | 1.02646 | | 1.11706 |

cd3_negat cd4_naive. cd4_positi cd4_positi cd4_positi cd4_positi cd4positiv cd8_positi

1.09826	1.07056	1.02188	1.00666	1.11153 0.978328	1.05154	1.28632

NA	NA	NA	NA	NA	NA	NA	NA

1.24303	1.27698	1.26296	1.37596	1.23178	1.24374	1.21419	1.41801

1.07933	1.12212	1.17935	1.26426	1.11082	1.12773	1.1535	1.17418

1.07515	1.11861	1.35539	1.23551	1.09284	1.23057	1.03733	1.27011

0.927674	0.85576 0.898867 0.754975 0.903234 0.891857 0.871746 0.989676

1.08623	1.08708	1.29288	1.00278	1.09213	1.15541	1.12908	1.28013

1.10418	1.07047	1.13503	1.07846	1.07253	1.04433	1.12579 0.882883

1.10418	1.07047	1.13503	1.07846	1.07253	1.04433	1.12579 0.882883

NA	1.18574	1.0827 NA	NA	1.09432 NA	NA

0.960621 0.932686 0.834486 0.964621 0.961394 0.970186	0.98742	1.06977

1.0175 0.943079 0.926822 0.930233 0.998713 0.968564 0.938067 0.992632

0.998496	1.01996	1.00781	1.19885	1.00785 0.999792 0.946074 0.891394

0.986582	1.04488	1.02668	1.04088 0.951771 0.964396 0.961214 0.931012

NA	NA	NA	NA	NA	NA	NA	NA NA	NA	NA	NA	NA	NA	NA	NA NA	1.08778 0.914345	1.02318 NA	0.986379 NA	NA NA	1.08778 0.914345	1.02318 NA	0.986379 NA	NA

0.941622	1.01369 0.980557 0.899142 0.961618 0.988137 0.971109 0.890428

1.22476	1.20694	1.3365	1.26267	1.21377	1.32399	1.23862	1.26343

NA	NA	NA	NA	NA	NA	NA	NA

1.02295	1.001	1.06135 0.983067	1.02842	1.06302 0.994711 0.965118

NA	NA	NA	NA	NA	NA	NA	NA NA	NA	NA	0.970117 NA	NA	NA	NA NA	NA	NA	1.10954 NA	1.0601 NA	NA NA	NA	NA	NA	NA	NA	NA	NA NA	NA	NA	NA	NA	NA	NA	NA NA	NA	NA	NA	NA	NA	NA	NA

1.10327	1.08357	1.20049	1.27403	1.09936	1.19454	1.17157	1.1085

1.10327	1.08357	1.20049	1.27403	1.09936	1.19454	1.17157	1.1085

1.05602	1.06332	1.34155	1.29766	1.08802	1.17788	1.02461	1.08824

1.08536	1.07681	1.33666	1.22876	1.13785	1.22113	1.08278	1.20968

NA	NA	NA	1.08725 NA	1.04732 NA	NA NA	NA	NA	1.1018 NA	NA	NA	NA NA	NA	NA	NA	NA	NA	NA	NA NA	NA	NA	NA	NA	NA	NA	NA NA	NA	NA	NA	NA	NA	NA	NA

1.07702	1.07407 0.993386	1.11221	1.07865	1.09137	1.09026	1.09106

| 0.977508 | NA |  | 0.993299 | | NA |  | 0.951569 | | NA |  | 0.966881 | | 1.02189 | |
| --- | --- | --- | --- | --- | --- | --- | --- | --- | --- | --- | --- | --- | --- | --- |
| 0.977508 | NA |  | 0.993299 | | NA |  | 0.951569 | | NA |  | 0.966881 | | 1.02189 | |
| 0.977508 | NA |  | 0.993299 | | NA |  | 0.951569 | | NA |  | 0.966881 | | 1.02189 | |
| 0.977122 | 0.992438 | | 1.05129 | | 0.951514 | | 0.969426 | | 1.04179 | | 0.988996 | | 0.837876 | |
| 1.00016 | 1.05105 | | 1.09768 | | NA |  | 1.02567 | | 1.08307 | | 1.00367 | | 0.900993 | |
| 1.04079 | 1.04375 | | 0.984609 | | 1.08653 | | 1.03358 | | 1.00947 | | 0.993724 | | 1.01999 | |
| 1.11314 | 1.09254 | |  | 1.0341 | 1.14286 | | 1.10649 | |  | 1.077 | 1.09931 | | 1.07158 | |
| 1.02051 | 1.07156 | | 1.01805 | | 1.11673 | | 1.06424 | | 1.05838 | | 1.00868 | | 1.06697 | |
| 1.07927 | 1.07065 | | 0.98372 | | 1.03883 | | 1.06047 | | 1.05171 | | 1.04498 | | 1.01159 | |
| 1.13055 | 1.16766 | | 1.04311 | | 1.14407 | | 1.12237 | | 1.12228 | | 1.05666 | | 1.16272 | |
| 1.12517 | 1.15211 | | 1.00007 | | 1.21629 | | 1.13908 | | 1.08065 | | 1.07636 | | 1.11338 | |
| 1.15446 | 1.14132 | | 1.05971 | | 1.12116 | | 1.17153 | | 1.08906 | | 1.08253 | | 1.03025 | |
| 1.14048 | 1.17842 | | 1.03538 | | 1.23973 | | 1.13566 | | 1.09924 | | 1.11178 | | 1.04531 | |
| 1.05897 | 1.05857 | | 0.993315 | |  | 1.1059 | 1.06681 | | 1.06412 | | 1.02887 | | 1.10742 | |
| 1.06388 | 1.07753 | | 1.00835 | | 1.14137 | | 1.07517 | | 1.04773 | | 1.04767 | | 1.04929 | |
| NA | NA |  | NA |  | NA |  | NA |  | NA |  | NA |  | NA |  |
| 1.16721 | 1.13235 | | 1.19479 | | 1.23818 | | 1.14616 | | 1.23409 | | 1.19149 | | 1.24393 | |
| 1.11586 | 1.08563 | | 1.03491 | | 1.10055 | | 1.08418 | | 1.07866 | | 1.08923 | | 0.921989 | |
| 1.11646 | 1.15395 | | 1.06855 | |  | 1.18 | 1.11471 | | 1.12613 | | 1.08329 | | 1.06957 | |
| 1.20118 | 1.20208 | | 1.15789 | | 1.07609 | | 1.14128 | | 1.09527 | | 1.04243 | | 1.16981 | |
| 1.07629 | 1.04058 | | 0.971691 | | 1.05599 | | 1.04218 | | 1.04912 | | 1.01075 | | 0.92187 | |
| NA | NA |  | NA |  | NA |  | NA |  | NA |  | NA |  | NA |  |
| NA | NA |  | NA |  | NA |  | NA |  | NA |  | NA |  | NA |  |
| 1.09404 | 1.08196 | | 0.976045 | | 1.04856 | | 1.08707 | | 1.07174 | | 1.05615 | | 0.968838 | |
| 1.08419 | 1.24297 | | 1.07092 | | 1.13208 | | 1.11089 | | 1.04376 | | 1.05804 | | 1.02857 | |
| 1.05645 | 1.12798 | | 1.02244 | | 1.22532 | | 1.09887 | | 1.13796 | | 1.07512 | | 1.07676 | |
| 1.11716 | 1.09398 | | 0.952794 | | 1.15506 | |  | 1.082 | 1.07099 | | 1.04278 | | 1.06093 | |
| 1.06292 | 1.07793 | | 1.01212 | | 1.12278 | | 1.06262 | | 1.07524 | | 1.04783 | | 0.916524 | |
| 1.05683 | 1.07162 | | 0.938274 | | 1.17265 | | 1.03748 | | 1.04462 | | 1.04849 | | 0.979725 | |
| 1.05683 | 1.07162 | | 0.938274 | | 1.17265 | | 1.03748 | | 1.04462 | | 1.04849 | | 0.979725 | |
| 1.13746 | 1.12936 | | 0.987565 | | 1.12351 | | 1.11459 | | 1.09489 | | 1.06089 | | 1.01608 | |
| 1.07716 | 1.13214 | | 0.990369 | | 1.19298 | | 1.10407 | | 1.07854 | | 1.03185 | | 1.16214 | |
| 1.10652 | 1.06014 | | 1.08355 | | 1.08649 | | 1.09477 | | 1.09055 | | 0.95615 | | 1.08967 | |
| 1.04428 | 1.08236 | | 0.946784 | | 1.07433 | | 1.04526 | | 1.08875 | |  | 1.0265 | 0.89625 | |
| NA | NA |  | NA |  | NA |  | NA |  | NA |  | NA |  | NA |  |
| 0.993145 | 0.972137 | | 1.03571 | | 0.900593 | | 1.00142 | | 0.991346 | | 1.02287 | |  | 1.0683 |
| 1.11719 | 1.13371 | | 1.04168 | | 1.05364 | | 1.12202 | |  | 1.1175 | 1.08405 | | 0.989031 | |
| 1.13531 | 1.11477 | | 0.98094 | | 1.08898 | | 1.10895 | | 1.09081 | | 1.05416 | | 1.01136 | |
| 1.28246 |  | 1.223 | 1.25778 | | 1.15548 | | 1.21212 | |  | 1.1096 |  | 1.0908 | 1.44231 | |

| 0.99693 | 1.11678 | | 1.04291 | | 0.979666 | 1.02668 | 0.994364 | | 1.01637 | | NA |
| --- | --- | --- | --- | --- | --- | --- | --- | --- | --- | --- | --- |
| 0.976882 | 1.03523 | | 0.992123 | | 1.00756 | 1.00129 | 1.01206 | | 0.959316 | | NA |
| 0.984146 | 1.04703 | | 0.975948 | | 1.03229 | 0.981796 | 0.994818 | | 0.982991 | | 1.00891 |
| NA | NA |  | 1.00766 | | 1.03703 | NA | 1.02887 | | NA |  | NA |
| NA | NA |  | NA |  | NA | NA | NA |  | NA |  | NA |
| NA | NA |  | NA |  | NA | NA | NA |  | NA |  | NA |
| NA | NA |  | NA |  | NA | NA | NA |  | NA |  | NA |
| NA | NA |  | NA |  | 0.950226 | NA | NA |  | NA |  | NA |
| NA | 0.974842 | | NA |  | 1.01318 | NA | 1.02996 | | NA |  | NA |
| NA | 1.00202 | | NA |  | 1.01515 | NA | 1.04774 | | NA |  | NA |
| NA | NA |  | NA |  | NA | NA | NA |  | NA |  | NA |
| NA | NA |  | NA |  | NA | NA | NA |  | NA |  | NA |
| NA | NA |  | NA |  | NA | NA | NA |  | NA |  | NA |
| NA | NA |  | NA |  | NA | NA | NA |  | NA |  | NA |
| NA | 1.01448 | | NA |  | 1.05351 | NA |  | 1.0607 | NA |  | NA |
| NA | 1.02554 | | NA |  | 1.09596 | 1.03232 | 1.08445 | | NA |  | NA |
| NA | NA |  | NA |  | 0.968926 | NA | 1.01708 | | NA |  | NA |
| NA | NA |  | NA |  | 0.944774 | NA | NA |  | NA |  | NA |
| 1.03833 |  | 1.0641 | NA |  | 1.08991 | 1.03933 | 1.11118 | | 1.01682 | | NA |
| NA | 0.998315 | | NA |  | 0.987618 | 0.992281 | 1.05301 | | NA |  | NA |
| NA | NA |  | NA |  | NA | NA | NA |  | NA |  | NA |
| 1.11798 | 1.11126 | |  | 1.0069 | 1.13926 | 1.10538 | 1.08079 | | 1.02569 | | 1.07599 |
| 0.957644 | 0.984126 | | NA |  | 1.02832 | 0.968475 | 0.968792 | | 0.983628 | | NA |
| 0.957644 | 0.984126 | | NA |  | 1.02832 | 0.968475 | 0.968792 | | 0.983628 | | NA |
| 1.03121 | 1.04327 | | NA |  | 1.03035 | 1.06132 |  | 1.0656 | NA |  | NA |
| 1.03121 | 1.04327 | | NA |  | 1.03035 | 1.06132 |  | 1.0656 | NA |  | NA |
| 0.989289 | 0.982546 | | NA |  | 1.07931 | 0.994666 | 1.05175 | | NA |  | NA |
| 0.928804 | 0.958639 | | NA |  | 1.00847 | 0.972808 | 0.968619 | | 1.00315 | | NA |
| NA | NA |  | NA |  | 0.974882 | NA | 0.980732 | | NA |  | NA |
| NA | NA |  | NA |  | 1.06621 | NA | NA |  | NA |  | NA |
| NA | NA |  | NA |  | 1.06621 | NA | NA |  | NA |  | NA |
| NA | NA |  | NA |  | 1.06621 | NA | NA |  | NA |  | NA |
| NA | NA |  | NA |  | NA | NA | NA |  | NA |  | NA |
| NA | NA |  | NA |  | NA | NA | NA |  | NA |  | NA |
| NA | NA |  | NA |  | NA | NA | NA |  | NA |  | NA |
| NA | NA |  | NA |  | NA | NA | NA |  | NA |  | NA |
| 1.01894 | 0.971722 | | 0.934122 | | NA | 1.02855 | 0.942227 | |  | 0.9941 | 0.866521 |
| 1.08403 | 1.13544 | | 1.02455 | | NA | 1.06395 | 1.13796 | |  | 1.1125 | 1.05316 |
| 1.08403 | 1.13544 | | 1.02455 | | NA | 1.06395 | 1.13796 | |  | 1.1125 | 1.05316 |

| 1.12123 | | 1.11529 | | 1.32801 |  | 1.2495 | 1.14171 |  | 1.1832 |  | 1.1469 | 1.19406 | |
| --- | --- | --- | --- | --- | --- | --- | --- | --- | --- | --- | --- | --- | --- |
| NA |  | NA |  | 1.20962 | NA |  | NA | NA |  | NA |  | NA |  |
| 1.06848 | | 1.09117 | | 1.10101 | 1.17281 | | 1.05594 | 1.06728 | | 1.03599 | | 1.14833 | |
| NA |  | NA |  | 1.00456 | NA |  | NA | NA |  | 0.97594 | | 0.982439 | |
| 1.01373 | | 0.947102 | | 1.08767 | 0.999468 | | 0.961278 | 1.05067 | | 1.02138 | |  | 1.0898 |
| NA |  | NA |  | NA | NA |  | NA | NA |  | NA |  | NA |  |
| NA |  | NA |  | NA | NA |  | NA | NA |  | NA |  | NA |  |
| NA |  | NA |  | NA | NA |  | NA | NA |  | NA |  | NA |  |
| NA |  | NA |  | 0.965315 | NA |  | NA | NA |  | NA |  | NA |  |
| NA |  | NA |  | 1.01627 | NA |  | NA | NA |  | NA |  | NA |  |
| 1.02256 | | NA |  | 0.964207 | NA |  | 0.952106 | 0.904304 | | 0.957743 | | NA |  |
| NA |  | NA |  | NA | NA |  | NA | NA |  | NA |  | NA |  |
| NA |  | NA |  | NA | NA |  | NA | NA |  | NA |  | NA |  |
| NA |  | NA |  | NA | NA |  | NA | NA |  | NA |  | NA |  |
| NA |  | NA |  | NA | NA |  | NA | NA |  | NA |  | NA |  |
| NA |  | NA |  | NA | NA |  | NA | NA |  | NA |  | NA |  |
| NA |  | NA |  | NA | NA |  | NA | NA |  | NA |  | NA |  |
| NA |  | NA |  | NA | NA |  | NA | NA |  | NA |  | NA |  |
| NA |  | NA |  | NA | NA |  | NA | NA |  | NA |  | NA |  |
| NA |  | 0.971352 | | NA | 0.990241 | | NA | 0.95898 | | NA |  | NA |  |
| NA |  | NA |  | NA | NA |  | NA | NA |  | NA |  | NA |  |
| NA |  | NA |  | NA | NA |  | NA | NA |  | NA |  | NA |  |
| NA |  | NA |  | NA | NA |  | NA | NA |  | NA |  | NA |  |
| NA |  | NA |  | NA | NA |  | NA | NA |  | NA |  | NA |  |
| NA |  | NA |  | NA | 0.975075 | | NA | NA |  | NA |  | NA |  |
|  | 1.0189 | 0.896627 | | NA | 1.01443 | | NA | 0.920041 | | NA |  | NA |  |
| NA |  | NA |  | NA | NA |  | NA | NA |  | NA |  | NA |  |
| NA |  | NA |  | NA | 1.00103 | | NA | NA |  | NA |  | NA |  |
| NA |  | NA |  | NA | NA |  | NA | NA |  | NA |  | NA |  |
| NA |  | NA |  | NA | NA |  | NA | NA |  | NA |  | NA |  |
| NA |  | NA |  | NA | 1.11664 | | NA | 1.03196 | | NA |  | NA |  |
| NA |  | NA |  | NA | NA |  | NA | NA |  | NA |  | NA |  |
| NA |  | NA |  | NA | 0.96421 | | NA | NA |  | NA |  | NA |  |
| NA |  | NA |  | NA | NA |  | NA | NA |  | NA |  | NA |  |
| NA |  | NA |  | NA | NA |  | NA | NA |  | NA |  | NA |  |
| NA |  | NA |  | NA | 1.16161 | | NA | 1.11506 | | NA |  | NA |  |
| NA |  | NA |  | NA | NA |  | NA | NA |  | NA |  | NA |  |
| NA |  |  | 1.042 | NA | 1.10708 | | NA | 1.08406 | | NA |  | NA |  |
| NA |  | NA |  | NA |  | 1.0266 | NA | NA |  | NA |  | NA |  |

| NA |  | NA |  | NA |  | NA |  | NA |  | NA |  | NA |  | NA |
| --- | --- | --- | --- | --- | --- | --- | --- | --- | --- | --- | --- | --- | --- | --- |
| 1.05108 | | 0.990647 | | NA |  | 1.00316 | | NA |  | 0.96386 | | NA |  | NA |
| NA |  | NA |  | NA |  | NA |  | NA |  | NA |  | NA |  | NA |
| NA |  | NA |  | NA |  | NA |  | NA |  | NA |  | NA |  | NA |
| NA |  | NA |  | NA |  | NA |  | NA |  | NA |  | NA |  | NA |
| NA |  |  | 1.1131 | NA |  | 1.22263 | | NA |  | 1.17724 | | NA |  | NA |
| NA |  | NA |  | NA |  | NA |  | NA |  | NA |  | NA |  | NA |
| NA |  | NA |  | NA |  | 1.11412 | | NA |  | NA |  | NA |  | NA |
| NA |  | NA |  | NA |  | NA |  | NA |  | NA |  | NA |  | NA |
| NA |  | 1.02853 | | NA |  |  | 1.1499 | NA |  | 1.09206 | | NA |  | NA |
| 1.00234 | | 0.986838 | | 0.992287 | | 1.09783 | |  | 1.0359 | 0.969917 | | 0.996885 | | 0.727273 |
| 1.03032 | | 1.24993 | | NA |  | 1.30382 | | 1.04096 | | 1.23313 | | 1.02468 | | NA |
| 1.02195 | |  | 1.0544 | NA |  | 1.09368 | | 0.970879 | |  | 1.0882 | 1.00668 | | NA |
| 1.01566 | | 1.02959 | | 1.04498 | | 1.04369 | | 0.972485 | | 1.02903 | | 1.05221 | | 1.05396 |
| 1.01084 | | 0.993662 | | 0.866641 | | 0.999516 | | 0.937623 | | 0.999326 | | 0.981886 | | 0.906934 |
| 1.02237 | | 0.997318 | |  | 1.1283 | 1.12999 | | 1.01722 | | 1.03815 | | 1.06408 | | 1.12245 |
| NA |  | 1.06185 | | 1.20448 | | 1.02272 | | 1.05595 | |  | 1.0803 | 1.06939 | | 1.15043 |
| 0.959802 | | 1.02432 | | NA |  | 1.08701 | | 0.960115 | | 1.08838 | | 0.948661 | | NA |
| 0.980552 | | 0.973611 | | 1.09786 | | 1.01502 | | 0.99097 | | 1.03888 | | 0.958414 | | 0.892806 |
| 1.09589 | | 1.08997 | | 1.01527 | | 1.16938 | | 1.01002 | | 1.09853 | | 1.05812 | | NA |
| NA |  | NA |  | 0.951524 | | 1.12128 | | NA |  | 1.10511 | | 1.03563 | | NA |
| NA |  | 1.06135 | |  | 1.0047 | 1.04505 | | NA |  | 1.00334 | | 1.03037 | | NA |
| 0.949295 | | 1.03883 | | 0.971758 | | 1.11675 | | 0.934027 | | 1.04219 | | 1.03421 | | 0.834862 |
| NA |  | NA |  | 0.952948 | | 0.946087 | | NA |  |  | 1.0019 | NA |  | NA |
| 1.08649 | | 1.11038 | | 1.02704 | | NA |  | 1.03909 | |  | 1.0486 | 1.09901 | | NA |
| 1.03545 | | 1.01401 | | 1.06522 | | NA |  | 1.00714 | | 0.999777 | | 1.04925 | | NA |
| 1.03516 | | 1.02659 | | 1.06052 | | NA |  | NA |  | 1.04972 | | 1.04841 | | 1.08747 |
| 1.15995 | | 1.10873 | | 1.15902 | | NA |  | 1.11244 | | 1.16821 | | 1.05089 | | 1.24138 |
| 1.15995 | | 1.10873 | | 1.15902 | | NA |  | 1.11244 | | 1.16821 | | 1.05089 | | 1.24138 |
|  | 1.0859 |  | 1.0719 | 1.13237 | | NA |  | 1.04381 | | NA |  | 0.967138 | | 1.17602 |
| 1.08061 | | 1.08676 | | 1.06924 | | NA |  | 1.07868 | | 1.10853 | | 1.03656 | | 0.904518 |
| 1.09136 | | 1.06543 | | 1.02996 | | NA |  | 1.03261 | | 1.04474 | |  | 1.0678 | NA |
| 1.09136 | | 1.06543 | | 1.02996 | | NA |  | 1.03261 | | 1.04474 | |  | 1.0678 | NA |
| 1.09136 | | 1.06543 | | 1.02996 | | NA |  | 1.03261 | | 1.04474 | |  | 1.0678 | NA |
| 1.02849 | | 1.00421 | | 1.02151 | | NA |  | 0.987229 | | 0.984077 | | 0.985308 | | 1.05704 |
| 0.992559 | | 0.949604 | | 1.02272 | | NA |  |  | 1.0197 | 0.968091 | | 0.989065 | | 0.787186 |
| NA |  | NA |  | 1.11536 | | NA |  | NA |  | NA |  | 1.04063 | | NA |
| NA |  | NA |  | NA |  | NA |  | NA |  | NA |  | NA |  | NA |
| 1.12697 | | 1.08401 | | NA |  | 1.10714 | | 1.10872 | | 1.10398 | | 1.13249 | | 0.970326 |

| NA |  | NA | NA |  | NA |  | NA | NA |  | NA | NA |  |
| --- | --- | --- | --- | --- | --- | --- | --- | --- | --- | --- | --- | --- |
| NA |  | NA | NA |  | NA |  | NA | NA |  | NA | NA |  |
| NA |  | NA | NA |  | NA |  | NA | NA |  | NA | NA |  |
| NA |  | NA | NA |  | NA |  | NA | NA |  | NA | NA |  |
| 0.926917 | | 0.896151 | 0.944225 | | 0.838755 | | 0.924932 | 0.876167 | | 0.908707 |  | 1.1213 |
| 1.02525 | | 1.01951 | 1.00525 | | 0.934951 | | 1.00661 | 1.01361 | | 1.01874 | 0.921692 | |
| 0.986376 | | 0.970508 | 0.978217 | | NA |  | 0.971999 |  | 1.0475 | 0.927536 |  | 1 |
| 1.03115 | | 1.00416 | 0.969862 | | 0.959953 | | 0.982534 | 0.976228 | | 1.00481 | 1.01333 | |
| 0.996827 | | NA | 0.97972 | | NA |  | 1.01377 | 0.940187 | | 0.963304 | 0.953538 | |
| 0.996903 | | 1.03114 | 1.04453 | | 0.987661 | | 1.02984 | 0.984085 | | 0.938611 | 0.888991 | |
|  | 1.0997 | 1.03551 |  | 1.281 | 1.11039 | | 1.09247 | 1.16152 | | 1.10989 | 1.27535 | |
| 1.03238 | | 1.03721 | 1.11369 | |  | 1.3208 | 1.04984 | 1.07502 | | 0.976719 | 1.01212 | |
| 1.12121 | | 1.11982 | 1.04898 | | 1.32508 | | 1.14185 | 1.12289 | | 1.11054 | 1.20566 | |
| 0.980886 | | 0.98934 | 0.929569 | | 0.987474 | | 0.975056 | 0.937974 | | 0.96939 | 1.24548 | |
| NA |  | NA | NA |  | NA |  | NA | NA |  | NA | NA |  |
| NA |  | NA | NA |  | NA |  | NA | NA |  | NA | NA |  |
| 1.07264 | | 1.04021 | 1.20646 | | 1.07601 | | 1.10609 | 1.15596 | | 1.07637 | 1.16071 | |
| NA |  | NA | NA |  | NA |  | NA | NA |  | NA | NA |  |
| 0.963081 | | 0.884999 | 0.954678 | | 0.880671 | | 0.937863 | 0.898997 | | 0.972543 | 1.20385 | |
| NA |  | NA | NA |  | NA |  | NA | NA |  | NA | NA |  |
| NA |  | NA | NA |  | NA |  | NA | NA |  | NA | NA |  |
| 1.10923 | | 1.09458 | 1.13178 | | 1.28078 | | 1.11294 | 1.16489 | | 1.13725 |  | 1.3841 |
| 1.11673 | | 1.08518 | 1.30434 | | 1.30667 | | 1.10037 |  | 1.1826 | 1.15938 | 1.45777 | |
| 1.11673 | | 1.08518 | 1.30434 | | 1.30667 | | 1.10037 |  | 1.1826 | 1.15938 | 1.45777 | |
| 1.11673 | | 1.08518 | 1.30434 | | 1.30667 | | 1.10037 |  | 1.1826 | 1.15938 | 1.45777 | |
| 1.11673 | | 1.08518 | 1.30434 | | 1.30667 | | 1.10037 |  | 1.1826 | 1.15938 | 1.45777 | |
| 1.16988 | | 1.11954 | 1.04934 | | 1.14759 | | 1.17755 | 1.11696 | | 1.13638 | 1.00721 | |
| 1.16988 | | 1.11954 | 1.04934 | | 1.14759 | | 1.17755 | 1.11696 | | 1.13638 | 1.00721 | |
| 1.16988 | | 1.11954 | 1.04934 | | 1.14759 | | 1.17755 | 1.11696 | | 1.13638 | 1.00721 | |
| 1.16988 | | 1.11954 | 1.04934 | | 1.14759 | | 1.17755 | 1.11696 | | 1.13638 | 1.00721 | |
| 1.19365 | | 1.22932 | 1.19702 | | 1.21821 | | 1.18669 | 1.13297 | | 1.17455 | 1.06147 | |
| 1.19365 | | 1.22932 | 1.19702 | | 1.21821 | | 1.18669 | 1.13297 | | 1.17455 | 1.06147 | |
| 1.09635 | | 1.10882 | 1.13192 | | 0.980392 | | 1.03099 | 1.02825 | | 1.05724 |  | 1 |
| 1.09635 | | 1.10882 | 1.13192 | | 0.980392 | | 1.03099 | 1.02825 | | 1.05724 |  | 1 |
| 1.18047 | | 1.11859 | 1.47307 | | 1.28908 | | 1.14381 | 1.26481 | | 1.17991 | 1.32994 | |
| 0.991405 | | 0.910845 | 0.912949 | | 0.810337 | | 0.948355 | 0.877363 | | 0.921701 | 1.07859 | |
| 0.991405 | | 0.910845 | 0.912949 | | 0.810337 | | 0.948355 | 0.877363 | | 0.921701 | 1.07859 | |
| 0.985569 | | 0.956346 | 0.905959 | | 0.840332 | | 0.955344 | 0.961348 | | 0.963181 | 1.10985 | |
| 0.985569 | | 0.956346 | 0.905959 | | 0.840332 | | 0.955344 | 0.961348 | | 0.963181 | 1.10985 | |

| 0.965913 | | 0.94475 | 0.915421 | | 0.846043 | | 0.965187 | 0.964275 | | 0.94325 | 1.11232 |
| --- | --- | --- | --- | --- | --- | --- | --- | --- | --- | --- | --- |
| 0.991841 | | 0.967861 | 0.965925 | | 0.856846 | | 0.980052 | 0.945986 | | 0.982559 | 1.07845 |
| 0.991025 | | 1.03142 | 0.957628 | | 0.979719 | | 0.993633 | 0.970586 | | 1.00554 | 1.20058 |
| 1.04985 | | 0.945617 | 0.970113 | | 0.873418 | | 1.00343 | 0.944323 | | 0.970884 | 1.03096 |
| 1.02969 | | 1.02508 | 1.04783 | | 1.07154 | | 1.04617 |  | 1.0509 | 0.981588 | 0.951289 |
| NA |  | NA | 0.963371 | | 1.17224 | | NA | 1.11114 | | NA | NA |
|  | 1.0087 | 1.03125 | 1.02812 | | 1.15075 | | 1.04593 | 1.08415 | | 0.989174 | 0.889474 |
| 1.06205 | | 1.00832 | 1.01364 | | NA |  | 1.03867 | 1.06176 | | 1.04232 | 0.975069 |
| 1.01888 | | 0.968364 | 1.02897 | |  | 0.9538 | 0.995408 | 1.04647 | | 1.01625 | 1.02941 |
| NA |  | NA | 0.998428 | | NA |  | NA | NA |  | NA | NA |
| 1.02274 | | 0.962925 | 1.02595 | | 0.90176 | | 1.00018 | 1.00015 | | 0.99987 | 0.956522 |
| 1.04664 | | 1.05927 | 1.00059 | | 1.07332 | | 1.03547 | 1.02963 | | 1.06161 | 1.00041 |
| NA |  | NA | 0.978643 | | NA |  | NA | NA |  | NA | NA |
| 1.06534 | | 0.983127 |  | 1.0017 | NA |  | 1.03455 | 1.04706 | | 1.00693 | 0.834457 |
| NA |  | NA | 1.08098 | | NA |  | NA | NA |  | NA | NA |
| NA |  | NA | NA |  | NA |  | NA | NA |  | NA | NA |
| NA |  | NA | NA |  | NA |  | NA | NA |  | NA | NA |
| 0.943417 | | 0.973772 | 0.979187 | | NA |  | 0.960413 | 0.981094 | | 0.979136 | NA |
| NA |  | NA | NA |  | NA |  | NA | NA |  | NA | NA |
| NA |  | NA | NA |  | NA |  | NA | NA |  | NA | NA |
| NA |  | NA | NA |  | NA |  | NA | NA |  | NA | NA |
| NA |  | NA | NA |  | NA |  | NA | NA |  | NA | NA |
| NA |  | NA | NA |  | NA |  | NA | NA |  | NA | NA |
| 0.99037 | | 1.00653 | 0.948811 | | 1.08853 | | 0.973178 |  | 1.0154 | 1.03686 | NA |
| 0.983251 | | 1.00337 | 0.976215 | | 1.01418 | | 0.979719 | 1.00993 | | 0.994551 | NA |
| NA |  | NA | NA |  | NA |  | NA | NA |  | NA | NA |
| NA |  | NA | NA |  | NA |  | NA | NA |  | NA | NA |
| NA |  | NA | NA |  | NA |  | NA | NA |  | NA | NA |
| 1.12913 | | 1.07388 | 1.36224 | | 1.22836 | | 1.11542 | 1.20754 | | 1.18014 | 1.45142 |
| 1.03838 | | 1.01425 | 0.997148 | | 0.987282 | | 1.05023 | 1.04727 | | 1.05162 | 0.945439 |
| 1.03838 | | 1.01425 | 0.997148 | | 0.987282 | | 1.05023 | 1.04727 | | 1.05162 | 0.945439 |
| NA |  | NA | NA |  | NA |  | NA | NA |  | NA | NA |
| NA |  | NA | 0.985142 | | NA |  | NA | NA |  | NA | NA |
| NA |  | NA | 0.964248 | | NA |  | NA | NA |  | NA | NA |
| NA |  | NA | 0.929822 | | NA |  | NA | NA |  | 1.00084 | NA |
| 1.08066 | | 1.12519 | 1.01517 | | 1.18211 | | 1.07639 | 1.11175 | | 1.08129 | 1.06154 |
| NA |  | NA | 1.02284 | | NA |  | NA | NA |  | NA | NA |
| NA |  | NA |  | 1.0411 | NA |  | NA | NA |  | NA | NA |
| NA |  | NA | 0.96882 | | NA |  | NA | NA |  | NA | NA |

| 0.951773 | | NA | 1.00067 | NA |  | 0.987401 | | 0.999329 | | NA | NA |  |
| --- | --- | --- | --- | --- | --- | --- | --- | --- | --- | --- | --- | --- |
| NA |  | NA | 0.972771 | NA |  | NA |  | NA |  | NA | NA |  |
| NA |  | NA | NA | NA |  | NA |  | NA |  | NA | NA |  |
| NA |  | NA | 0.931401 | NA |  | NA |  | NA |  | NA | NA |  |
| NA |  | NA | 1.05948 | NA |  | NA |  | NA |  | NA | NA |  |
| 1.06543 | | 1.04874 | 1.03342 | 0.941642 | | 1.04859 | | 1.02955 | | 1.06359 | 0.980976 | |
| NA |  | NA | NA | NA |  | NA |  | NA |  | NA | NA |  |
| NA |  | NA | NA | NA |  | NA |  | NA |  | NA | NA |  |
| NA |  | NA | NA | NA |  | NA |  | NA |  | NA | NA |  |
| NA |  | NA | NA | NA |  | NA |  | NA |  | NA | NA |  |
| 0.835607 | | 0.859715 | 1.02846 |  | 1.1358 | 0.852229 | | 0.875942 | | 0.88287 | 1.14545 | |
| NA |  | NA | 1.06618 | NA |  | NA |  | 0.93869 | | 0.998495 | 0.879911 | |
| 1.09099 | | 1.05835 | 1.08414 | 1.11014 | | 1.05567 | | 1.07363 | | 1.07469 | 0.930126 | |
| NA |  | NA | NA | NA |  | NA |  | NA |  | NA | NA |  |
| NA |  | NA | 0.997961 | NA |  | NA |  | NA |  | NA | 1.09091 | |
|  | 1.0022 | 0.935688 | NA | 0.940998 | | 1.00818 | | 0.961346 | | 0.991298 | NA |  |
| 1.01786 | | 1.03061 | 1.01151 | NA |  | 1.02474 | | 1.00428 | | 1.10905 | NA |  |
| NA |  | NA | 0.976758 | NA |  | NA |  | NA |  | NA | 1.04377 | |
| NA |  | NA | NA | NA |  | NA |  | NA |  | NA | NA |  |
| 1.00386 | | NA | 1.08041 | NA |  | 1.02066 | | NA |  | 0.976119 | 0.925802 | |
| 1.09347 | | 1.07216 | 1.03439 | NA |  | 1.09161 | | 1.06176 | | 1.03273 |  | 1.0015 |
| 0.993872 | | 0.958847 | 0.985249 | NA |  | 0.987177 | | 0.891437 | | 1.02952 | NA |  |
| 0.988896 | | NA | 1.00491 | NA |  | 0.964719 | | 0.920501 | | 0.976432 | NA |  |
| 1.13057 | | 1.14937 | 1.07148 | 1.27685 | | 1.13194 | | 1.08905 | | 1.16372 | 1.06789 | |
| 0.997766 | | 0.988622 | 0.973075 | 0.845912 | | 1.07009 | | 0.984039 | | 0.973129 | 0.95283 | |
| 0.987856 | | NA | 1.04855 | NA |  | NA |  | NA |  | 0.969994 | NA |  |
| 1.09333 | | 0.990736 | 0.980363 | 1.00387 | | 1.06132 | |  | 1.0438 | 1.05952 | 0.83378 | |
| 1.02236 | | NA | 0.939128 | NA |  | 1.04123 | | 0.982309 | | 1.01919 | NA |  |
| 1.00162 | | 1.05987 | 1.09076 | 1.06117 | | 1.02301 | | 1.06546 | | 1.01763 | 0.889358 | |
| 1.00919 | | 1.04612 | 0.969653 | 0.986998 | | 0.979408 | | 1.04257 | | 1.09843 | 1.01287 | |
| 0.954386 | | 0.789668 | 1.00348 | 0.844828 | | 0.873065 | | 0.982206 | | 0.941634 | 0.814815 | |
| 1.05055 | | 1.10742 | 1.18867 | 1.01994 | | 0.98304 | | 1.03366 | | 1.10558 | 0.985294 | |
| 0.981611 | | 0.940608 | 0.960567 | 0.891176 | | 0.896126 | | 0.96409 | | 1.03105 | 0.779006 | |
| 1.11619 | | 1.04404 | 1.11899 | 0.967949 | | 1.03539 | | 1.08727 | | 1.05267 | 0.883289 | |
| 0.897993 | | 0.978764 | 1.07971 |  | 1.155 | 0.861702 | | 0.97893 | | 1.01829 | 0.882353 | |
| 0.92735 | | 1.09701 | NA | 1.04444 | |  | 0.9 | 1.05439 | | 1.09142 | NA |  |
| NA |  | NA | NA | NA |  | NA |  | NA |  | NA | NA |  |
| NA |  | NA | NA | NA |  | NA |  | NA |  | NA | NA |  |
| NA |  | NA | NA | NA |  | NA |  | NA |  | NA | NA |  |

| 1.00898 | | 0.963911 | | 1.01208 | | 0.92146 | | 1.00235 | | 1.01284 | | 0.983464 | | 0.952655 |
| --- | --- | --- | --- | --- | --- | --- | --- | --- | --- | --- | --- | --- | --- | --- |
| 0.993219 | | 0.975417 | | 1.02614 | | 0.91332 | | 1.00033 | |  | 1.0136 | 0.988217 | | 0.944987 |
|  | 1.0752 |  | 1.0931 |  | 1.0662 | 1.01847 | | 1.11968 | | 1.14785 | |  | 1.1084 | 1.14718 |
| NA |  | NA |  | 0.947087 | | NA |  | 0.998225 | | NA |  | 0.966458 | | NA |
| NA |  | NA |  | 0.976238 | | NA |  | NA |  | NA |  | NA |  | NA |
| NA |  | NA |  | NA |  | NA |  | NA |  | NA |  | NA |  | NA |
| NA |  | NA |  | 1.06301 | | NA |  | NA |  | NA |  | NA |  | NA |
| 1.05171 | | 1.00917 | | NA |  | 1.07285 | | 1.06755 | | 1.06799 | | 1.05308 | | NA |
| 1.14037 | | 0.993051 | | 1.04541 | | NA |  | 1.05925 | | 1.03206 | | 1.02809 | | 1.14653 |
| 1.08794 | | 1.06388 | | 1.07052 | | 1.06499 | | 1.05587 | | 1.02236 | | 1.06663 | | 1.09612 |
| 1.04253 | | 1.02424 | | 1.00466 | | 0.942858 | | 1.03868 | | 0.964883 | | 1.03467 | | 0.961508 |
| 1.04253 | | 1.02424 | | 1.00466 | | 0.942858 | | 1.03868 | | 0.964883 | | 1.03467 | | 0.961508 |
| 1.08334 | |  | 1.0902 | 1.05994 | |  | 1.1815 | 1.07237 | | 1.04184 | | 1.06401 | | 1.14349 |
| NA |  | NA |  | 0.961343 | | 1.00291 | | NA |  | 0.966424 | | 1.02683 | | 0.996337 |
| NA |  | NA |  | 0.961343 | | 1.00291 | | NA |  | 0.966424 | | 1.02683 | | 0.996337 |
| 0.966464 | | 0.938533 | | NA |  | 0.976302 | | 0.984934 | | 0.950627 | | 0.96502 | | NA |
| 0.973458 | | 0.945127 | | NA |  |  | 0.9615 | 0.976371 | | 0.963884 | | NA |  | NA |
| 0.898115 | | 0.952079 | | NA |  | 0.847866 | | 0.918911 | | 0.920075 | | NA |  | NA |
| 1.10385 | | 1.05671 | | 1.08278 | | NA |  | 1.06566 | | 1.06257 | | 1.04339 | | NA |
| 1.09912 | | 1.13234 | | 1.00637 | | 1.16189 | | 1.09471 | | 1.10869 | | 1.05292 | | 1.03607 |
| 1.10001 | | 1.12709 | | 0.977987 | | 1.15902 | | 1.07849 | |  | 1.0653 | 1.05587 | | 1.07111 |
| 1.00945 | | 1.00241 | | 0.919576 | | 1.02355 | | 0.988452 | | 0.949954 | |  | 0.9636 | 1.09706 |
| 1.05693 | | 1.01772 | | 0.997861 | | 1.03405 | | 1.03046 | | 1.03674 | |  | 1.0039 | 0.888675 |
|  | 1.0325 | 1.06627 | |  | 1.0045 | 1.12902 | | 1.04105 | | 1.01342 | | 1.03653 | | 1.02069 |
| 1.11474 | | 1.07569 | | 1.00102 | | 1.11522 | | 1.09142 | | 1.01996 | | 1.04085 | | 1.02681 |
| 1.02249 | | 1.01233 | | 0.94458 | | 1.09452 | |  | 1.0255 |  | 1.0434 | 1.06887 | | 0.984663 |
| 1.02754 | |  | 1.0123 | 1.08378 | | 1.01288 | | 0.98211 | | 1.06475 | |  | 1.0082 | NA |
| 1.11224 | | 1.05053 | | 1.02364 | | 1.05783 | | 1.04766 | |  | 1.0317 | 1.02808 | | 1.00923 |
| 0.91816 | | 0.901545 | | 1.06115 | | NA |  | 0.929368 | | 0.880993 | | 0.94842 | | NA |
| 1.05908 | | 0.975196 | | 1.00438 | | 0.863809 | | 1.00354 | | 1.00215 | | 1.03562 | | 1.15173 |
| 0.934065 | | 0.875537 | | 0.918936 | | 0.815993 | | 0.925993 | | 0.894894 | | 0.988869 | | 1.15134 |
| 0.965431 | | 0.908638 | | 0.935105 | | NA |  | 0.949383 | |  | 0.9053 | 0.902097 | | 0.962494 |
| 0.935127 | | 0.985948 | | NA |  | 0.956291 | | 0.954109 | | 0.972225 | | 0.991636 | | NA |
| NA |  | NA |  | NA |  | NA |  | NA |  | NA |  | NA |  | NA |
| NA |  | NA |  | NA |  | NA |  | NA |  | NA |  | NA |  | NA |
| 1.07697 | | NA |  | 1.02721 | | NA |  | NA |  | NA |  | 1.02032 | | 0.934884 |
| 1.02666 | | 0.993749 | | 1.06285 | | NA |  | 1.03346 | | 0.973307 | | 1.00192 | | 1.02873 |
| 1.01131 | | NA |  | 1.06137 | | NA |  | 0.982898 | | NA |  | 1.01918 | | 0.987704 |
| NA |  | NA |  | 1.06863 | | NA |  | NA |  | NA |  | NA |  | 1.06796 |

| 1.07697 | | 1.08702 | | 1.02156 | | NA |  | 1.08804 | | NA | 0.996139 | | 1.15222 | |
| --- | --- | --- | --- | --- | --- | --- | --- | --- | --- | --- | --- | --- | --- | --- |
|  | 0.9109 | 0.903602 | | 0.904669 | | 0.790621 | | 0.895805 | | 0.894123 | 0.961004 | | 1.00336 | |
| 1.09931 | | 1.06942 | | 1.20611 | | 1.10082 | | 1.10203 | | 1.16805 | 1.12076 | | 1.09438 | |
| 1.06346 | | 1.05564 | | 1.19681 | | 1.03618 | | 1.06841 | | 1.12955 |  | 1.0612 | 1.09535 | |
| 1.10171 | | 1.10347 | | 1.14141 | | 1.04894 | | 1.15685 | | 1.10212 | 1.05317 | | 1.13348 | |
| 0.948966 | |  | 1 | 0.85749 | | 0.915663 | | 0.990164 | | 0.922018 | 1.02998 | | 1.25203 | |
| NA |  | NA |  | 1.02664 | | NA |  | NA |  | NA | 1.02608 | | 0.906666 | |
| 1.06983 | | 1.02232 | | 1.17937 | | 0.980676 | | 1.06621 | | 1.11636 | 1.08277 | | 1.18605 | |
| 1.13116 | | 1.12736 | | 1.21899 | | NA |  | 1.13519 | | 1.17419 | 1.12374 | | 1.20629 | |
| NA |  | NA |  | NA |  | NA |  | NA |  | NA | NA |  | NA |  |
| NA |  | NA |  | NA |  | NA |  | NA |  | NA | NA |  | NA |  |
| NA |  | NA |  | NA |  | NA |  | NA |  | NA | NA |  | NA |  |
| 1.19691 | | 1.25924 | | 1.07237 | | 1.30063 | | 1.19707 | | 1.15526 | 1.20742 | | 1.07273 | |
|  | 1.0961 | 1.22387 | | 1.24835 | | 1.29139 | | 1.09031 | | 1.16786 | 1.14675 | | 1.34748 | |
| 0.96084 | | 0.936953 | | 0.896366 | | NA |  | 0.981029 | | 0.894532 | 0.950922 | | 1.12813 | |
| 1.06013 | | 1.14601 | | 1.18354 | | 1.15028 | |  | 1.141 | 1.18226 | 1.18426 | | 0.938111 | |
| 0.95579 | | 0.900828 | | 0.864446 | | NA |  | 0.970787 | | 0.892042 | 0.952579 | | 1.15957 | |
| 1.03489 | | 1.03251 | | 1.01456 | | NA |  | 1.04783 | | 1.04709 | 1.06303 | | 0.977929 | |
| 1.03799 | | NA |  | 1.04031 | | NA |  | 1.03242 | | NA | 1.01852 | | 1.00383 | |
| NA |  | NA |  | NA |  | NA |  | NA |  | NA | NA |  | NA |  |
| 1.08118 | | 1.09595 | | 1.05531 | | 1.13966 | |  | 1.1012 | 1.03032 | 1.05524 | | 1.02028 | |
| 0.979987 | | 0.967353 | | 1.01773 | | NA |  | 0.982068 | | 0.998155 | 1.00553 | | NA |  |
| NA |  | NA |  | 1.08601 | | NA |  | NA |  | NA | NA |  | 1.01434 | |
| NA |  | NA |  | 1.08444 | | NA |  | NA |  | NA | NA |  | 0.921246 | |
| 1.02272 | | 0.985164 | | 1.08904 | | NA |  | 0.979067 | | 0.931169 | 0.961735 | | 0.855363 | |
|  | 1.0088 | 1.01633 | |  | 1.0077 | NA |  | 1.02493 | | 0.960799 | 1.02089 | | 0.916877 | |
| 0.986437 | | 0.997447 | | 1.01582 | | 0.969631 | | 0.924531 | | 0.950321 | 0.973777 | | 0.938581 | |
| 1.08157 | | 1.13044 | | 1.04653 | | 1.09106 | | 1.07282 | | 1.08631 | 1.03665 | |  | 1.0634 |
| 1.03454 | | 1.08627 | | 1.05735 | | NA |  | 1.04199 | | 1.11393 | 0.983425 | | NA |  |
| 1.14619 | | 1.16983 | |  | 1.1177 | 1.18371 | | 1.20916 | | 1.10581 | 1.08592 | | 0.886473 | |
| 1.02432 | | 1.01941 | | 1.08501 | |  | 1 | 0.963613 | | 0.983186 | 0.964319 | | 1.09062 | |
| 1.17526 | | 1.17959 | | 1.14638 | | 1.40144 | | 1.13029 | | 1.16513 | 1.15238 | | 1.12882 | |
| 1.05054 | | 1.06391 | | 1.02707 | | 1.11229 | | 1.05017 | | 1.04336 | 1.02377 | |  | 1.1648 |
| 1.00571 | | 1.00269 | | 1.01995 | | 1.00804 | | 0.978203 | | 0.969078 | 0.911143 | | 0.971098 | |
| 1.03079 | | 1.03801 | | 1.10324 | | 1.21111 | | 1.06471 | | NA |  | 1.0294 | 1.10961 | |
| 1.01118 | | 1.00995 | | 1.04917 | | 1.06352 | | 1.01882 | | 1.04378 | 1.02863 | | 0.942487 | |
| 1.01334 | | 0.982207 | | 1.07118 | | 1.02954 | | 1.01186 | | 1.04617 | 1.03326 | | 1.00397 | |
| 1.09057 | | 1.05072 | | 1.07145 | | 1.10983 | | 1.11395 | | 1.06171 | 1.02611 | |  | 1.0124 |
| 1.03413 | | 1.21197 | | 1.00832 | | 1.28544 | | 1.13619 | | 1.11673 | 1.05661 | | 1.12678 | |

| 1.19687 | | 1.16667 | | 1.06674 | 1.24113 | | 1.15962 | | 1.13555 | | 1.05966 | | 1.03415 | |
| --- | --- | --- | --- | --- | --- | --- | --- | --- | --- | --- | --- | --- | --- | --- |
| 1.05997 | |  | 1.0224 | 1.02243 | 0.969125 | | 1.06292 | | 1.07855 | |  | 1.0278 | 1.14506 | |
|  | 1.1624 | 1.14478 | | 1.17711 | 1.14594 | | 1.14389 | | 1.09695 | | 1.10966 | | 1.21801 | |
| 0.984183 | | 0.955769 | | 0.99665 | 1.03906 | | 0.967742 | | 1.00933 | | 0.949091 | | 0.93154 | |
| 1.11915 | | 1.17973 | | 1.13052 | 1.34862 | | 1.15234 | | 1.14128 | |  | 1.1158 | 1.08433 | |
| NA |  | 0.982532 | | NA | 0.924063 | | NA |  | 0.975857 | | NA |  | NA |  |
| 0.978713 | | 0.955563 | | 0.994659 | 0.950621 | | 0.965327 | | 0.979438 | | 0.983797 | | 1.01067 | |
| 1.00523 | | 0.965864 | | 0.978014 | NA |  |  | 1.0056 | 0.958153 | | 0.987851 | | NA |  |
| 1.05336 | | 1.11869 | | 1.03875 | 1.14209 | | 1.12118 | | 1.17981 | | 1.09093 | | 0.934247 | |
| 1.04399 | | 1.05097 | | 0.912338 | 1.07407 | | 1.01308 | | 0.997743 | | 0.952583 | | 0.837037 | |
| 0.977895 | | 0.951232 | | 0.937158 | NA |  | 0.985914 | | 0.955006 | | 0.969578 | | 0.905556 | |
| NA |  | NA |  | NA | NA |  | NA |  | NA |  | NA |  | NA |  |
| 1.03956 | | 1.00253 | | 1.03558 | 1.04381 | | 1.04216 | | 1.02365 | | 0.991076 | | 0.978082 | |
| 1.08459 | | 1.03935 | | 1.08452 | NA |  | 1.04344 | | 1.02603 | | 1.03942 | | 1.06192 | |
|  | 1.0283 |  | 1.0623 | 1.11656 | NA |  | 1.05946 | | 1.05861 | | 1.04651 | |  | 1.0224 |
| 1.04178 | | 1.04897 | | 1.02937 | 0.968339 | | 1.04034 | | 1.03026 | | 1.04944 | | 1.07231 | |
| 1.12903 | |  | 1.0497 | 1.02168 | NA |  | 1.09103 | | 1.06711 | | 1.06245 | | 1.18335 | |
| 1.05701 | | 1.02275 | | 0.971005 | 1.02961 | | 1.00588 | | 0.972872 | | 0.985944 | | 1.26181 | |
| NA |  | 1.03003 | | 0.99232 | NA |  | NA |  | 1.02103 | | NA |  | NA |  |
| 1.05037 | | 0.999257 | | 1.03775 | NA |  | 1.03471 | | 1.02619 | | 1.02845 | |  | 1.0805 |
| 1.04606 | |  | 1.0533 | 1.02028 | 1.02093 | | 1.02166 | | 1.00168 | | 1.00425 | | 0.957265 | |
| 1.02233 | |  | 1.0081 | NA | 1.00746 | | 1.01826 | | 0.983229 | | 0.947918 | | NA |  |
| 0.992054 | | 0.99442 | | 1.00073 | NA |  | 0.975746 | | 1.01587 | | 0.981855 | | 0.980672 | |
| 1.03148 | | 0.994935 | | 0.985679 | NA |  | 1.01424 | | 1.02697 | | 1.02474 | | 0.978817 | |
| 1.00128 | | 0.996971 | | 1.03441 | NA |  | 0.994512 | | 0.99009 | | 1.01117 | | NA |  |
| 1.10584 | | 1.15159 | | 1.01466 | 1.16952 | | 1.14157 | |  | 1.1656 | 1.07798 | | 1.12758 | |
| 1.05916 | | 1.08372 | | 1.02685 | 1.14706 | | 1.05285 | | 1.06212 | | 1.04493 | |  | 1.1067 |
| 1.02205 | | 0.996282 | | 1.02304 | NA |  | 1.01515 | | 1.03008 | | 1.02682 | | 0.876902 | |
| 1.00593 | | 0.974545 | | 0.994531 | 0.882095 | | 1.00384 | | 0.93892 | | 0.974698 | | 0.812121 | |
| 0.921368 | | 0.955338 | | 0.917737 | 1.05423 | | 0.938636 | | 0.966331 | | 0.91048 | | NA |  |
| 1.04617 | | 1.01728 | | 1.05365 | 1.01448 | | 1.03997 | | 1.02251 | |  | 1.0123 | 0.965279 | |
|  | 1.228 | 1.26349 | | 1.15093 | 1.28578 | | 1.21198 | | 1.20661 | | 1.23047 | | 1.23462 | |
| 0.988042 | | 0.953429 | | 0.962943 | NA |  | 0.976985 | | 0.972838 | | 0.974129 | | 0.945103 | |
| 1.04241 | | 1.03933 | | 1.07101 | 0.916142 | | 1.06338 | | 1.05016 | | 1.07611 | | 1.13768 | |
| NA |  | NA |  | 1.02954 | NA |  | NA |  | NA |  | NA |  | NA |  |
| 1.06467 | |  | 1.0865 | 1.06064 |  | 1.0099 | 1.07169 | | 1.07076 | | 1.03761 | | 1.11178 | |
|  | 1 | 1.03438 | | 1.01978 | 0.90121 | | 1.02177 | | 0.990939 | | 0.990741 | | 0.967273 | |
| 1.02808 | | 1.04435 | | 1.05483 | NA |  | 1.04438 | | 1.03697 | | 1.05427 | | 1.20067 | |

cd8_positi cd8positiv central_m central_m central_m class_swit common_l common_

| 1.08231 | 1.12705 0.997012 | 1.01464 | 1.11083 | 1.08988 | 1.08186 | 1.10787 |
| --- | --- | --- | --- | --- | --- | --- |

NA	NA	NA	NA	NA	NA	NA	NA

1.21301	1.17839	1.23385	1.29048	1.29131	1.18735	1.19285	1.2427

1.06552	1.18556	1.12836	1.19701	1.17335	1.17016	1.13904	1.13079

1.12	1.19429	1.16269	1.17808	1.25373	1.3993	1.13487	1.15474

0.919055	0.87643	0.8967 0.937874 0.996205 0.964309 0.907657 0.927738

1.06394	1.11537	1.14941	1.18277	1.16844	1.25223	1.07246	1.11164

1.05806	1.02507	1.10458	1.15459	1.08873	1.19229	1.04156	1.09241

1.05806	1.02507	1.10458	1.15459	1.08873	1.19229	1.04156	1.09241

NA	NA	1.03015	1.02173 NA	1.19222 NA	NA

0.958952 0.941725 0.986857	1.00568 0.969557	0.98002 0.911446 0.965011

0.983721 0.940409 0.930093 0.925794	0.93323	1.01761	1.04145	1.09702

0.99163 0.995488	1.00973	0.9991 0.978987	1.00272	1.04089	1.01196

0.988907	0.98645	0.99121 0.997719 0.938511 0.985299 0.993499	1.00343

NA	NA	NA	NA	NA	NA	NA	NA NA	NA	NA	NA	NA	NA	NA	NA NA	NA	1.04415	1.03259	1.1307 0.986943 NA	NA NA	NA	1.04415	1.03259	1.1307 0.986943 NA	NA

0.956735	1.02573 0.930291 0.989635 0.898292	1.02984 0.981074 0.982102

1.20541	1.22224	1.2913	1.34223	1.26879	1.31805	1.23362	1.22168

NA	NA	NA	NA	NA	NA	NA	NA

1.04292	1.0558	1.013	1.01147 0.922307	1.01493	0.99175	1.00241

NA	NA	NA	NA	NA	NA	NA	NA NA	NA	NA	NA	NA	NA	NA	NA NA	NA	NA	1.00895 NA	NA	NA	NA NA	NA	NA	NA	NA	NA	NA	NA NA	NA	NA	NA	NA	NA	NA	NA NA	NA	NA	NA	NA	NA	NA	NA

1.10137	1.07939	1.16377	1.25548	1.07012	1.21019	1.1874	1.16571

1.10137	1.07939	1.16377	1.25548	1.07012	1.21019	1.1874	1.16571

1.07012	1.13463	1.25926	1.28753	1.18895	1.37005	1.07899	1.12029

1.10248	1.22527	1.24373	1.29359	1.15385	1.35935	1.06811	1.11752

NA	NA	NA	1.00453 NA	1.18675 NA	NA NA	NA	NA	NA	NA	NA	NA	NA NA	NA	NA	NA	NA	NA	NA	NA NA	NA	NA	NA	NA	NA	NA	NA NA	NA	NA	NA	NA	NA	NA	NA

1.07795	1.0159	1.1276	1.06005 0.973774	1.02817	1.05093	1.04684

| 0.96038 | | 0.953109 | | 0.934684 | | 0.934474 | | 0.854423 | | 0.980232 | | 0.91831 | | 0.94052 | |
| --- | --- | --- | --- | --- | --- | --- | --- | --- | --- | --- | --- | --- | --- | --- | --- |
| 0.96038 | | 0.953109 | | 0.934684 | | 0.934474 | | 0.854423 | | 0.980232 | | 0.91831 | | 0.94052 | |
| 0.96038 | | 0.953109 | | 0.934684 | | 0.934474 | | 0.854423 | | 0.980232 | | 0.91831 | | 0.94052 | |
| 0.982425 | | 0.999103 | |  | 1.0176 |  | 1.0633 |  | 0.8795 | 1.04748 | | 1.03634 | |  | 1.0396 |
| 1.03946 | | 1.02719 | | 1.03632 | | 1.10854 | | 0.986469 | | 1.00206 | | 1.02134 | | 1.01309 | |
| 1.03035 | | 1.01675 | | 1.02844 | | 1.00641 | | 0.918379 | | 0.996829 | |  | 1.0219 | 1.03039 | |
| 1.10547 | | 1.06587 | | 1.07896 | | 1.00939 | | 0.903516 | | 0.999365 | | 1.07638 | | 1.04664 | |
| 1.04387 | | 1.07546 | | 1.03671 | | 0.984634 | | 0.959323 | |  | 1.0231 |  | 1.0458 | 1.04977 | |
| 1.05246 | | 1.05073 | | 1.00208 | | 1.01325 | | 0.87351 | | 0.960345 | | 1.02397 | | 1.02094 | |
| 1.12651 | | 1.08787 | | 1.09265 | | 1.04417 | | 0.993281 | | 1.03563 | | 1.09003 | |  | 1.0541 |
| 1.13116 | |  | 1.1273 | 1.06152 | | 0.985819 | | 0.916643 | | 1.03466 | |  | 1.0985 | 1.12394 | |
| 1.18033 | | 1.07464 | | 1.05571 | | 0.98837 | | 0.938314 | | 1.00618 | | 1.10126 | | 1.05703 | |
| 1.13645 | | 1.14731 | | 1.07111 | | 1.05933 | | 0.968537 | | 0.956239 | | 1.13705 | | 1.13905 | |
| 1.04625 | | 1.10445 | | 1.07993 | | 1.02443 | | 1.04361 | | 0.986814 | | 1.06311 | | 1.06509 | |
| 1.07769 | | 1.09242 | | 1.02607 | | 1.01614 | |  | 0.9117 | 0.97682 | | 1.07424 | | 1.05851 | |
| NA |  | NA |  | NA |  | NA |  | NA |  | NA |  | NA |  | NA |  |
| 1.11931 | | 1.24384 | | 1.16352 | | 1.25734 | | 1.12337 | | 1.24012 | | 1.16526 | | 1.20449 | |
|  | 1.0829 |  | 1.1411 | 1.06812 | | 1.04757 | | 0.909815 | | 1.01297 | | 1.08803 | | 1.09818 | |
| 1.12218 | | 1.11955 | | 1.09675 | | 1.06131 | | 1.04279 | | 1.05792 | | 1.13566 | | 1.13339 | |
| 1.15137 | | 1.03933 | |  | 1.1362 | 1.03035 | | 1.14493 | |  | 1.053 | 1.14944 | | 1.13417 | |
| 1.04165 | | 1.06676 | | 1.00641 | | 0.97834 | | 0.880229 | | 0.99301 | | 1.05351 | |  | 1.0341 |
| NA |  | NA |  | NA |  | NA |  | NA |  | 1.01457 | | NA |  | NA |  |
| NA |  | NA |  | NA |  | NA |  | NA |  | NA |  | NA |  | NA |  |
| 1.08771 | | 1.13427 | | 1.04442 | | 1.01532 | | 0.926042 | | 1.02249 | | 1.07919 | | 1.07924 | |
| 1.07014 | | 0.951542 | | 1.07838 | | 1.05612 | | 1.28571 | | 1.11281 | | 1.23611 | | 1.18987 | |
|  | 1.105 | 1.09368 | | 1.09436 | | 1.04143 | | 1.00891 | | 0.987984 | |  | 1.1104 |  | 1.0766 |
| 1.09549 | | 1.13738 | | 1.05152 | | 1.05169 | | 0.965696 | | 1.02467 | | 1.09735 | | 1.10693 | |
| 1.04971 | | 1.06083 | | 1.06699 | |  | 1.0676 | 0.953608 | | 0.998939 | | 1.05888 | | 1.10554 | |
|  | 1.0573 | 1.17436 | | 1.04398 | | 1.05006 | | 1.03119 | | 0.910177 | | 1.08392 | | 1.12107 | |
|  | 1.0573 | 1.17436 | | 1.04398 | | 1.05006 | | 1.03119 | | 0.910177 | | 1.08392 | | 1.12107 | |
| 1.11996 | | 1.12544 | | 1.07532 | |  | 1.0553 | 0.969297 | | 0.996009 | | 1.11694 | | 1.10306 | |
| 1.07666 | | 1.13462 | | 1.05311 | | 1.05962 | | 1.01909 | | 1.04242 | | 1.12458 | | 1.08807 | |
| 1.09471 | | 1.04713 | | 1.03609 | | 1.04162 | | 0.954424 | | 1.01036 | | 1.07771 | | 1.07318 | |
| 1.03226 | | 1.02914 | | 1.08622 | | 1.02394 | | 0.932044 | | 0.972528 | | 1.03415 | | 1.02067 | |
| NA |  | NA |  | NA |  | NA |  | NA |  | NA |  | NA |  | NA |  |
| 0.962654 | | 1.04105 | | 1.00108 | | 1.01056 | | 1.08132 | |  | 1.0839 |  | 1.0138 | 1.03733 | |
|  | 1.1161 | 1.12828 | | 1.08313 | | 1.03162 | | 0.899739 | | 1.00108 | | 1.07864 | | 1.07789 | |
| 1.11233 | | 1.10905 | | 1.07155 | | 1.02612 | | 0.906079 | | 1.00819 | | 1.07831 | | 1.07822 | |
| 1.26258 | |  | 1.1271 | 1.14494 | | 1.07893 | | 1.41361 | | 1.16034 | | 1.22336 | | 1.21869 | |

| 1.01624 | | 1.01898 | 1.05643 | 1.01312 | | 0.992237 | 0.982259 | 0.993542 | 1.04803 | |
| --- | --- | --- | --- | --- | --- | --- | --- | --- | --- | --- |
| 1.02242 | | 0.95801 | 0.961352 | 1.00537 | | 1.01077 | 0.968884 | 0.985302 | 0.931279 | |
| 1.00296 | | 1.01721 | 1.00722 | 1.01422 | | 0.911423 | 0.977735 | 1.01136 | 1.02247 | |
| NA |  | NA | 1.08204 | 1.00376 | | 0.955083 | 1.00587 | NA |  | 1.0145 |
| NA |  | NA | NA | NA |  | NA | NA | NA | NA |  |
| NA |  | NA | NA | NA |  | NA | NA | NA | NA |  |
| NA |  | NA | NA | NA |  | NA | NA | NA | NA |  |
| NA |  | NA | NA | NA |  | NA | NA | NA | NA |  |
| NA |  | NA | NA | NA |  | NA | NA | NA | NA |  |
| NA |  | NA | NA | NA |  | NA | NA | NA | NA |  |
| NA |  | NA | NA | NA |  | NA | NA | NA | NA |  |
| NA |  | NA | NA | NA |  | NA | NA | NA | NA |  |
| NA |  | NA | NA | NA |  | NA | NA | NA | NA |  |
| NA |  | NA | NA | NA |  | NA | NA | NA | NA |  |
| NA |  | NA | NA | NA |  | NA | NA | NA | NA |  |
| 1.02483 | | NA | NA | 1.02595 | | NA | NA | NA |  | 1.0366 |
| NA |  | NA | NA | NA |  | NA | NA | NA | NA |  |
| NA |  | NA | NA | NA |  | NA | NA | NA | NA |  |
| 1.04976 | | 1.08094 | NA | 1.05598 | | NA | NA | 1.04923 | 1.05412 | |
| NA |  | NA | NA | NA |  | NA | NA | NA | 1.04227 | |
| NA |  | NA | NA | NA |  | NA | NA | NA | NA |  |
| 1.09184 | | 1.12398 | 1.05503 | 1.04033 | | 0.950687 | 0.995524 | 1.09549 | 1.09879 | |
| 0.978956 | | 1.05617 | 0.968831 | 0.986759 | | NA | NA | 0.967472 | 0.96136 | |
| 0.978956 | | 1.05617 | 0.968831 | 0.986759 | | NA | NA | 0.967472 | 0.96136 | |
| 1.05474 | | NA | NA | NA |  | NA | NA | NA | 1.04521 | |
| 1.05474 | | NA | NA | NA |  | NA | NA | NA | 1.04521 | |
| 0.991504 | | NA | NA | 0.986499 | | NA | NA | 0.981025 | 0.982796 | |
| 0.949561 | | 1.02421 | 0.946608 | 0.994239 | | NA | NA | 0.980084 | 1.00851 | |
| NA |  | NA | NA | NA |  | NA | NA | NA | 0.95443 | |
| NA |  | NA | NA | NA |  | NA | NA | NA | 1.04396 | |
| NA |  | NA | NA | NA |  | NA | NA | NA | 1.04396 | |
| NA |  | NA | NA | NA |  | NA | NA | NA | 1.04396 | |
| NA |  | NA | NA | NA |  | NA | NA | NA | NA |  |
| NA |  | NA | NA | NA |  | NA | NA | NA | NA |  |
| NA |  | NA | NA | NA |  | NA | NA | NA | NA |  |
| NA |  | NA | NA | NA |  | NA | NA | NA | NA |  |
|  | 1.0178 | 1.00848 | 0.915571 | 0.978243 | | 0.827157 | 1.00194 | 0.990606 | 0.989674 | |
| 1.09015 | | 1.13248 | 1.09686 |  | 1.1519 | 1.14826 | 1.16164 | 1.09702 | 1.05909 | |
| 1.09015 | | 1.13248 | 1.09686 |  | 1.1519 | 1.14826 | 1.16164 | 1.09702 | 1.05909 | |

| 1.10675 |  | 1.276 | 1.20711 | | 1.20743 | 1.22337 | 1.30768 | | 1.09105 | | 1.13364 |
| --- | --- | --- | --- | --- | --- | --- | --- | --- | --- | --- | --- |
| NA | NA |  | NA |  | NA | 1.17889 |  | 1.3422 | NA |  | NA |
| 1.05796 | 1.12966 | | 1.09121 | | 1.07999 | 1.02331 | 1.14379 | | 1.00656 | | 1.07387 |
| NA | 0.961697 | | 1.08135 | | 0.989052 | 1.03283 | 1.02264 | | 0.933691 | | 0.878176 |
| 1.00858 | 1.01434 | |  | 1.1159 | 1.06854 | 1.11023 | 1.02801 | | 1.03542 | | 1.02243 |
| NA | NA |  | NA |  | NA | NA | NA |  | NA |  | NA |
| NA | NA |  | NA |  | NA | NA | NA |  | NA |  | NA |
| NA | NA |  | NA |  | NA | NA | NA |  | NA |  | NA |
| NA | NA |  | 0.975605 | | 0.918564 | 1.06712 | 0.962533 | | NA |  | NA |
| NA | NA |  | 1.10086 | | 1.03313 | 1.18182 | 1.02463 | | NA |  | NA |
| 0.965031 | 0.948382 | | 0.981621 | | 0.938694 | 1.01923 | 0.99341 | | 0.944125 | | 0.947773 |
| NA | NA |  | NA |  | NA | NA | NA |  | NA |  | NA |
| NA | NA |  | NA |  | NA | NA | NA |  | NA |  | NA |
| NA | NA |  | NA |  | NA | NA | NA |  | NA |  | NA |
| NA | NA |  | NA |  | NA | NA | NA |  | NA |  | NA |
| NA | NA |  | NA |  | NA | NA | NA |  | NA |  | NA |
| NA | NA |  | NA |  | NA | NA | NA |  | NA |  | NA |
| NA | NA |  | NA |  | NA | NA | NA |  | NA |  | NA |
| NA | NA |  | NA |  | NA | NA | NA |  | NA |  | NA |
| NA | NA |  | NA |  | NA | NA | NA |  |  | 1.0268 | 1.06124 |
| NA | NA |  | NA |  | NA | NA | NA |  | NA |  | NA |
| NA | NA |  | NA |  | NA | NA | NA |  | NA |  | NA |
| NA | NA |  | NA |  | NA | NA | NA |  | NA |  | NA |
| NA | NA |  | NA |  | NA | NA | NA |  | NA |  | NA |
| NA | NA |  | NA |  | NA | NA | NA |  | NA |  | NA |
| NA | NA |  | NA |  | NA | NA | NA |  | 0.971767 | | 0.976804 |
| NA | NA |  | NA |  | NA | NA | NA |  | NA |  | NA |
| NA | NA |  | NA |  | NA | NA | NA |  | NA |  | NA |
| NA | NA |  | NA |  | NA | NA | NA |  | NA |  | NA |
| NA | NA |  | NA |  | NA | NA | NA |  | NA |  | NA |
| NA | NA |  | NA |  | 1.04812 | NA | NA |  | 0.988863 | | 0.996443 |
| NA | NA |  | NA |  | NA | NA | NA |  | NA |  | NA |
| NA | NA |  | NA |  | NA | NA | NA |  | NA |  | NA |
| NA | NA |  | NA |  | NA | NA | NA |  | NA |  | NA |
| NA | NA |  | NA |  | NA | NA | NA |  | NA |  | NA |
| NA | NA |  | NA |  | NA | NA | NA |  | NA |  | NA |
| NA | NA |  | NA |  | NA | NA | NA |  | NA |  | NA |
| NA | NA |  | NA |  | NA | NA | NA |  | NA |  | NA |
| NA | NA |  | NA |  | NA | NA | NA |  | NA |  | NA |

| NA |  | NA |  | NA |  | NA |  | NA | NA |  | NA |  | NA |
| --- | --- | --- | --- | --- | --- | --- | --- | --- | --- | --- | --- | --- | --- |
| NA |  | NA |  | NA |  | NA |  | NA | NA |  | 1.02306 | | 0.981426 |
| NA |  | NA |  | NA |  | NA |  | NA | NA |  | NA |  | NA |
| NA |  | NA |  | NA |  | NA |  | NA | NA |  | NA |  | NA |
| NA |  | NA |  | NA |  | NA |  | NA | NA |  | NA |  | NA |
| NA |  | NA |  | NA |  | 1.13821 | | NA | NA |  | 1.15723 | | 1.09337 |
| NA |  | NA |  | NA |  | NA |  | NA | NA |  | NA |  | NA |
| NA |  | NA |  | NA |  | NA |  | NA | NA |  | NA |  | NA |
| NA |  | NA |  | NA |  | NA |  | NA | NA |  | NA |  | NA |
| NA |  | NA |  | NA |  | NA |  | NA | NA |  | NA |  | NA |
| 1.00675 | | 1.00267 | | 0.955513 | |  | 1.0137 | 0.866124 | 0.962974 | | 0.998121 | | 0.952603 |
| 1.00841 | | 0.988772 | |  | 1.0524 | 1.27754 | | NA | 0.985244 | | 1.02822 | | 1.02763 |
| 1.00983 | | NA |  | 1.04381 | | 1.06956 | | NA | NA |  | 1.01705 | | 0.999744 |
| 0.98712 | | 1.03202 | | 1.08637 | | 1.04786 | | 0.993594 | 1.07573 | |  | 1.0186 | 1.02241 |
| 0.93622 | | 0.975391 | | 0.951291 | | 1.09671 | | 0.794442 | 1.06607 | | 1.03302 | | 1.12832 |
| 1.01959 | | 1.00813 | | 1.08267 | | 1.14608 | | 1.07692 | 1.10447 | | 0.984726 | | 1.01122 |
| 1.07857 | | 1.09505 | |  | 1.0453 | 1.12715 | | 1.04958 | 1.20374 | | NA |  | NA |
| 0.985612 | | 0.962779 | | 1.04569 | | 1.10351 | | NA | NA |  | 0.978822 | | 0.998125 |
| 1.00588 | | 0.973917 | | 0.979985 | | 1.06363 | | 0.905218 |  | 1.0434 |  | 1.0201 | 1.04744 |
|  | 1.0801 | 1.08426 | | 1.10952 | | 1.20121 | | 0.936471 | 1.00598 | | 1.11858 | | 1.14479 |
| NA |  | 1.03278 | | 1.02578 | | 1.06323 | | 0.947012 | 0.95978 | | 0.982981 | | 0.996846 |
| NA |  |  | 1.0169 | 1.03507 | | 0.997304 | | 1.07463 | 0.948211 | | 1.00574 | | 1.02473 |
| 0.970776 | | 0.992813 | | 1.01393 | | 1.03166 | | 0.905338 |  | 0.987 | 1.04724 | | 0.987273 |
| NA |  | NA |  | 1.08663 | | 1.03741 | | 0.965667 | 0.958753 | | NA |  | 1.04172 |
| 1.05867 | |  | 1.161 | 1.04314 | | 1.01921 | | NA | 0.972321 | | 1.07985 | | 1.03386 |
| 1.00731 | | 1.12957 | | 1.12988 | | 0.956014 | | 1.03705 | 1.02918 | | 1.09702 | | 1.04324 |
| 1.04076 | | 1.12709 | | 1.05941 | | 1.10418 | | 1.04891 | 1.15214 | |  | 1.0274 | 1.01566 |
| 1.07093 | | 1.16318 | | 1.09597 | | 1.06029 | | 1.11275 |  | 1.1191 | 1.13351 | | 1.03704 |
| 1.07093 | | 1.16318 | | 1.09597 | | 1.06029 | | 1.11275 |  | 1.1191 | 1.13351 | | 1.03704 |
| 1.02596 | | 1.09926 | | 1.05386 | | 1.05189 | | 0.970696 |  | 1.0952 | 1.03788 | | 1.00868 |
| 1.03696 | | 1.04705 | | 1.06642 | | 1.06934 | | 0.896289 | 1.02775 | | 1.06713 | | 1.04963 |
| 1.05047 | | 1.13441 | | 1.04846 | | 1.02582 | | NA | 1.01498 | | 1.04679 | | 1.03859 |
| 1.05047 | | 1.13441 | | 1.04846 | | 1.02582 | | NA | 1.01498 | | 1.04679 | | 1.03859 |
| 1.05047 | | 1.13441 | | 1.04846 | | 1.02582 | | NA | 1.01498 | | 1.04679 | | 1.03859 |
| 0.974418 | | 1.00677 | | 1.00205 | | 0.998782 | | 0.875649 | 0.973207 | | 0.995851 | | 1.01179 |
| 0.991953 | | 1.00259 | | 1.02542 | | 1.01339 | | 0.875671 | 0.969582 | | 1.02401 | | 0.973625 |
| NA |  | 1.17197 | | 1.16777 | | 1.16185 | | NA | 1.05286 | | NA |  | NA |
| NA |  | NA |  | NA |  | NA |  | NA | NA |  | NA |  | NA |
| 1.10667 | | 1.06767 | |  | 1.1131 | 1.00336 | | 1.00548 | NA |  | 1.03831 | | 1.12732 |

| NA | NA |  | NA |  | NA | NA | NA |  | NA |  | NA |  |
| --- | --- | --- | --- | --- | --- | --- | --- | --- | --- | --- | --- | --- |
| NA | NA |  | NA |  | NA | NA | NA |  | NA |  | NA |  |
| NA | NA |  | NA |  | NA | NA | NA |  | NA |  | NA |  |
| NA | NA |  | NA |  | NA | NA | NA |  | NA |  | NA |  |
| 0.934229 | 0.871662 | | 0.94135 | | 0.951042 | 1.06604 | 0.989274 | | 0.863168 | | 0.932193 | |
| 0.978211 | 1.00848 | | 1.01173 | | 1.01773 | 1.08144 | 1.02064 | | 0.956693 | | 1.00459 | |
| 0.997378 | 1.00069 | | 1.02219 | | 1.04897 | 1.18229 | 1.00096 | | 1.04127 | | 1.02612 | |
| 1.01841 | 1.01881 | | 0.92674 | | 1.00528 | 1.02595 | 1.05815 | | 1.00062 | | 1.02009 | |
| 0.996673 | 1.01675 | | 0.969698 | | 0.93773 | 1.00398 |  | 0.9799 | 0.97801 | | 0.973586 | |
| 1.00659 | 1.00619 | | 0.950214 | | 0.982332 | 1.08564 | 1.05316 | | 1.01338 | |  | 1.0349 |
| 1.05878 | 1.09924 | | 1.14809 | | 1.19004 | 1.17279 | 1.23622 | | 1.10791 | | 1.10918 | |
| 1.05141 | 1.04096 | | 1.10641 | | 1.18774 | 1.02683 | 1.04674 | | 1.13603 | | 1.07493 | |
| 1.10094 |  | 1.2074 | 1.06873 | | 1.16412 | 1.02304 | 1.05498 | |  | 1.2009 | 1.15737 | |
| 0.963506 | 0.975517 | | 0.916731 | | 0.928119 | 0.904498 | 0.941379 | | 0.96763 | | 0.975736 | |
| NA | NA |  | NA |  | NA | NA | NA |  | NA |  | NA |  |
| NA | NA |  | NA |  | NA | NA | NA |  | NA |  | NA |  |
| 1.08651 | 1.13149 | | 1.09309 | | 1.17965 | 1.10909 | 1.20522 | | 1.11373 | | 1.13211 | |
| NA | NA |  | NA |  | NA | NA | NA |  | NA |  | NA |  |
| 0.936091 | 0.921332 | | 0.940127 | | 0.939676 | 0.915597 | 0.97287 | | 0.943561 | | 0.981957 | |
| NA | NA |  | NA |  | NA | NA | NA |  | NA |  | NA |  |
| NA | NA |  | NA |  | NA | NA | NA |  | NA |  | NA |  |
| 1.08639 | 1.23025 | | 1.17871 | | 1.12635 | 1.26116 | 1.11065 | | 1.10123 | | 1.14435 | |
| 1.08842 |  | 1.1097 |  | 1.1985 | 1.25031 | 1.16744 | 1.30496 | | 1.13989 | | 1.11795 | |
| 1.08842 |  | 1.1097 |  | 1.1985 | 1.25031 | 1.16744 | 1.30496 | | 1.13989 | | 1.11795 | |
| 1.08842 |  | 1.1097 |  | 1.1985 | 1.25031 | 1.16744 | 1.30496 | | 1.13989 | | 1.11795 | |
| 1.08842 |  | 1.1097 |  | 1.1985 | 1.25031 | 1.16744 | 1.30496 | | 1.13989 | | 1.11795 | |
| 1.20679 | 1.05563 | | 1.16956 | | 1.13486 | 1.09091 | 1.08407 | | 1.07875 | | 1.09774 | |
| 1.20679 | 1.05563 | | 1.16956 | | 1.13486 | 1.09091 | 1.08407 | | 1.07875 | | 1.09774 | |
| 1.20679 | 1.05563 | | 1.16956 | | 1.13486 | 1.09091 | 1.08407 | | 1.07875 | | 1.09774 | |
| 1.20679 | 1.05563 | | 1.16956 | | 1.13486 | 1.09091 | 1.08407 | | 1.07875 | | 1.09774 | |
| 1.18453 | 1.12446 | |  | 1.0948 | 1.17673 | 1.17029 | 1.14943 | | 1.12445 | | 1.08866 | |
| 1.18453 | 1.12446 | |  | 1.0948 | 1.17673 | 1.17029 | 1.14943 | | 1.12445 | | 1.08866 | |
| 1.01933 | 0.966756 | | 1.00062 | | 1.10991 | 1.01865 | 1.12329 | | 1.01306 | | 1.05689 | |
| 1.01933 | 0.966756 | | 1.00062 | | 1.10991 | 1.01865 | 1.12329 | | 1.01306 | | 1.05689 | |
| 1.11078 | 1.19806 | | 1.30847 | | 1.35087 | 1.19709 | 1.36044 | |  | 1.2155 | 1.20011 | |
| 0.961737 | 0.925819 | | 0.91089 | | 0.910432 | 0.917847 | 0.927265 | | 0.895461 | | 0.928514 | |
| 0.961737 | 0.925819 | | 0.91089 | | 0.910432 | 0.917847 | 0.927265 | | 0.895461 | | 0.928514 | |
| 0.979784 | 0.923816 | | 0.89891 | | 0.950345 | 0.889401 | 0.960608 | | 0.971521 | | 0.963081 | |
| 0.979784 | 0.923816 | | 0.89891 | | 0.950345 | 0.889401 | 0.960608 | | 0.971521 | | 0.963081 | |

| 0.951537 | | 0.929928 | 0.950349 | | 0.970885 | | 0.978393 | 0.990374 | | 0.931498 | | 0.993723 |
| --- | --- | --- | --- | --- | --- | --- | --- | --- | --- | --- | --- | --- |
| 0.985667 | | 0.93679 | 0.902548 | | 0.957822 | | 0.860923 | 0.961585 | | 0.914909 | | 0.988576 |
| 0.993315 | | 1.00283 | 1.03736 | | 1.02533 | | 1.05529 | 0.987785 | | 0.986758 | | 1.02015 |
| 1.05173 | | 1.00634 | 1.02522 | | 0.983411 | | 1.01254 | 0.934531 | |  | 1.0536 | 1.00013 |
|  | 1.0517 | 0.990682 | 1.06742 | | 1.03972 | | 1.05782 | 1.04099 | | 0.97272 | | 1.04114 |
| NA |  | NA | 1.00269 | | 1.06525 | | 0.912195 | 0.989193 | | 1.00269 | | 1.01617 |
| 1.01218 | | 0.98046 | 1.05182 | | 0.986495 | | 1.08419 | 1.03184 | | 0.993198 | | 1.00633 |
| 1.04829 | | 1.03727 | 1.04286 | |  | 1.0126 | 0.993068 | 1.02663 | | 1.03642 | | 1.05097 |
| 1.00374 | | 1.00095 | 1.05994 | | 1.03047 | | 0.973016 | 1.01451 | | 1.03589 | | 1.04041 |
| NA |  | NA | NA |  | NA |  | 1.01229 | 0.982015 | | NA |  | NA |
| 1.00439 | | 0.981625 | 1.09499 | | 1.01971 | | 0.996753 | 1.03997 | | 0.996588 | | 1.02875 |
| 1.03461 | | 1.04711 | 0.979801 | | 0.997293 | | 1.01746 | 0.977945 | | 1.05556 | | 1.01576 |
| NA |  | NA | 1.02297 | | 1.00862 | | 0.980743 | 0.999095 | | NA |  | NA |
| 1.04167 | | 0.999972 | 0.995667 | | 1.00996 | | 0.960211 | 1.00238 | | 1.05685 | | 1.12266 |
| NA |  | NA | 1.08157 | | 1.09791 | | 0.890217 | 1.07748 | | NA |  | NA |
| NA |  | NA | NA |  | NA |  | NA | 0.936913 | | NA |  | NA |
| NA |  | NA | NA |  | NA |  | NA | NA |  | NA |  | NA |
| 0.995057 | | 0.951167 | 0.942637 | | 0.953264 | | NA | 1.00603 | | 0.998611 | | 0.997744 |
| NA |  | NA | NA |  | NA |  | NA | 0.953754 | | NA |  | NA |
| NA |  | NA | NA |  | NA |  | NA | 1.04744 | | NA |  | NA |
| NA |  | NA | NA |  | NA |  | NA | 0.929465 | | NA |  | NA |
| NA |  | NA | NA |  | NA |  | NA | 1.02922 | | NA |  | NA |
| NA |  | NA | NA |  | NA |  | NA | 1.01104 | | NA |  | NA |
| 0.987443 | | 0.993121 | 0.995509 | | 1.00045 | | NA | 0.916777 | |  | 1.0652 | 1.04477 |
| 1.00202 | | 1.00656 | 0.986976 | | 0.974048 | | NA | 0.98311 | | 1.07915 | | 1.03403 |
| NA |  | NA | NA |  | NA |  | NA | NA |  | NA |  | NA |
| NA |  | NA | NA |  | NA |  | NA | NA |  | NA |  | NA |
| NA |  | NA | NA |  | NA |  | NA | NA |  | NA |  | NA |
| 1.07103 | | 1.15722 | 1.26169 | | 1.29586 | | 1.17844 | 1.32445 | | 1.13018 | | 1.16186 |
| 1.02557 | | 1.01372 | 1.00046 | | 1.04011 | | 0.967823 |  | 1.107 | 1.02801 | | 1.04996 |
| 1.02557 | | 1.01372 | 1.00046 | | 1.04011 | | 0.967823 |  | 1.107 | 1.02801 | | 1.04996 |
| NA |  | NA | NA |  | NA |  | NA | NA |  | NA |  | NA |
| NA |  | NA | 1.13074 | | NA |  | 0.947877 | 1.05476 | | NA |  | NA |
| NA |  | NA | NA |  | NA |  | NA | 0.983192 | | NA |  | NA |
| NA |  | 0.954271 | 1.00133 | | 0.940782 | | NA | 1.03779 | | 0.962199 | | 0.928668 |
| 1.07002 | | 1.09249 | 0.981188 | | 0.990458 | | 1.08403 | 1.04066 | | 1.00604 | | 1.06329 |
| NA |  | NA |  | 1.116 | 1.06229 | | 1.27815 | 1.01865 | | NA |  | NA |
| NA |  | NA | NA |  | NA |  | NA | 1.09152 | | NA |  | NA |
| NA |  | NA |  | 1.1052 | NA |  | 1.20284 | 1.01842 | | NA |  | NA |

| 0.993724 | | NA |  | 0.953963 | | 1.00585 | | NA | 1.00399 | | 0.980901 | | 0.960631 | |
| --- | --- | --- | --- | --- | --- | --- | --- | --- | --- | --- | --- | --- | --- | --- |
| NA |  | NA |  | NA |  | NA |  | NA | NA |  | NA |  | NA |  |
| NA |  | NA |  | NA |  | NA |  | NA | NA |  | NA |  | NA |  |
| NA |  | NA |  | NA |  | NA |  | 0.897402 | 0.944023 | | NA |  | NA |  |
| NA |  | NA |  | NA |  | NA |  | 1.15644 |  | 1.0269 | NA |  | NA |  |
| 1.00465 | | 1.03357 | | 0.992998 | | 0.986888 | | 1.09589 | 1.02313 | | 0.960333 | | 1.00545 | |
| NA |  | NA |  | NA |  | NA |  | NA | NA |  | NA |  | NA |  |
| NA |  | NA |  | NA |  | NA |  | NA | NA |  | NA |  | NA |  |
| NA |  | NA |  | NA |  | NA |  | NA | NA |  | NA |  | NA |  |
| NA |  | NA |  | NA |  | NA |  | NA | NA |  | NA |  | NA |  |
| 0.822991 | | 0.873152 | | 0.942384 | | 0.918018 | | 1.02299 | 1.04855 | | 0.906274 | |  | 1.0076 |
| NA |  | 0.942906 | | 0.957441 | | 0.984756 | | 0.994083 | 0.969241 | | NA |  | NA |  |
| 1.06173 | |  | 0.9829 | 1.05617 | | 1.11105 | | 1.18883 |  | 1.0906 | 1.08024 | | 1.06119 | |
| NA |  | NA |  | NA |  | NA |  | NA | NA |  | NA |  | NA |  |
| NA |  | 1.06329 | | 1.00898 | | NA |  | 1.03435 | 0.999721 | | NA |  | NA |  |
| 0.981059 | | NA |  |  | 1.0236 | 0.991176 | | NA | 0.990755 | |  | 1 | 1.01084 | |
| 1.03965 | | 1.00242 | | 1.02742 | | 1.06391 | | 1.05104 | 1.02518 | | 1.06949 | | 1.04315 | |
| NA |  | NA |  | 0.980234 | | NA |  | 1.06819 | 1.04403 | | NA |  | NA |  |
| NA |  | NA |  | NA |  | NA |  | NA | NA |  | NA |  | NA |  |
| 0.996494 | | 0.975143 | | 1.01771 | | 1.02364 | | 1.09315 | 0.995481 | | 1.06292 | |  | 1.0394 |
| 1.07853 | | 1.05081 | | 1.01351 | | 1.00819 | | 0.909833 | 1.00562 | |  | 1.0541 | 1.01456 | |
| 0.966722 | | 1.03343 | | 0.953516 | | 0.955101 | | 1.07857 | 0.996883 | | 1.03022 | | 1.00347 | |
| 0.973264 | | 0.940866 | | 0.983304 | | 0.933839 | | NA | 1.00368 | | 0.97396 | | 0.897039 | |
| 1.11607 | | 1.15838 | | 1.03195 | |  | 1.0669 | 0.960915 | 1.04835 | | 1.20639 | | 1.20753 | |
| 1.03905 | | 0.965514 | | 0.94493 | | 1.00345 | | 0.968692 | 1.04925 | | 0.982616 | | 0.995426 | |
| NA |  | 0.957751 | | 0.994243 | |  | 0.9749 | 1.18182 | 1.01848 | | NA |  | 1.01574 | |
| 1.09482 | | 1.00752 | | 0.982284 | | 1.06411 | | 0.979592 | 1.02266 | | 1.08037 | | 1.12617 | |
| 1.01219 | | 1.00237 | | 0.966453 | |  | 1.0001 | 0.915517 |  | 1.0011 | 1.05776 | | 1.09349 | |
|  | 1.0139 | 1.03126 | | 1.05831 | | 1.12022 | | 1.05105 | 1.06327 | | 1.02552 | | 1.00754 | |
| 1.00691 | | 1.00347 | | 1.06017 | | 1.02079 | | 1.04126 | 0.965998 | | 1.05465 | | 1.00433 | |
| 0.859016 | | 0.826255 | | 0.915385 | | 0.930748 | | 0.941176 | 1.08274 | | 1.04724 | | 0.980519 | |
| 1.04813 | | 1.06275 | | 1.04122 | | 1.03115 | | 1.06052 | 1.00772 | |  | 1.0302 | 1.03704 | |
| 0.92887 | | 0.991221 | | 0.900774 | | 0.956768 | | 0.939297 | 0.993726 | | 1.03024 | | 0.99633 | |
|  | 1.0984 | 1.10116 | |  | 1.035 | 1.01603 | | 0.978554 | 1.01322 | | 1.03961 | | 1.02544 | |
| 0.968121 | | 1.07076 | | 0.940945 | | 0.950882 | | 1.02062 | 0.945221 | | 0.916667 | | 1.01742 | |
| 1.01064 | |  | 1.0638 | 0.955882 | | 0.970032 | | 1.10897 | 0.945402 | | NA |  |  | 1.025 |
| NA |  | NA |  | NA |  | NA |  | NA | 1.02418 | | NA |  | NA |  |
| NA |  | NA |  | NA |  | NA |  | NA | 1.02418 | | NA |  | NA |  |
| NA |  | NA |  | NA |  | NA |  | NA | 1.02418 | | NA |  | NA |  |

| 0.999445 | | 0.98041 | | 1.01316 | | 0.965669 | | 0.951458 | 1.00959 | | 0.936562 | | 0.954626 | |
| --- | --- | --- | --- | --- | --- | --- | --- | --- | --- | --- | --- | --- | --- | --- |
| 0.990675 | | 1.00151 | |  | 1.0311 | 1.00848 | | 0.944894 | 0.979492 | | 0.982296 | | 0.965041 | |
| 1.13274 | | 1.15072 | | 1.06023 | | 1.01878 | | 1.13476 | 0.997822 | | 1.11672 | | 1.08911 | |
| 0.978672 | | 0.950485 | | 0.969384 | | 0.936508 | | NA | 0.996223 | | 0.991984 | | 0.970854 | |
| NA |  | NA |  | NA |  | NA |  | 1.27051 | 1.02307 | | NA |  | NA |  |
| NA |  | NA |  | NA |  | NA |  | NA | 1.09008 | | NA |  | NA |  |
| NA |  | NA |  | NA |  | NA |  | NA | 1.09314 | | NA |  | NA |  |
| 1.07718 | | 1.03795 | |  | 1.0046 | 0.986436 | | NA |  | 1.1201 | 1.14372 | | 1.15242 | |
| 1.09167 | | 0.983987 | | 1.02335 | | 1.01602 | | 1.08333 | 1.01053 | | 1.13855 | | 1.07994 | |
| 1.09209 | | 1.08271 | | 1.05049 | | 0.989483 | | 1.02047 | 1.11237 | | 1.08651 | | 1.03682 | |
|  | 1.0871 | 1.05508 | | 0.987637 | | 0.933634 | | 0.89115 |  | 1.0138 | 1.05398 | | 1.03124 | |
|  | 1.0871 | 1.05508 | | 0.987637 | | 0.933634 | | 0.89115 |  | 1.0138 | 1.05398 | | 1.03124 | |
| 1.10032 | | 1.10553 | | 1.01349 | | 1.01732 | | 1.02484 | 1.06873 | | 1.06484 | | 1.01659 | |
| NA |  | 0.964994 | |  | 1.0327 | 0.978275 | | 0.971673 | 0.971995 | | 0.926639 | | 0.94347 | |
| NA |  | 0.964994 | |  | 1.0327 | 0.978275 | | 0.971673 | 0.971995 | | 0.926639 | | 0.94347 | |
| 0.977899 | | 0.949103 | | 0.969775 | | 0.951129 | | NA | NA |  | 0.958044 | | 0.984931 | |
| 0.993789 | | NA |  | NA |  | 0.885151 | | NA | NA |  | NA |  | NA |  |
| 0.921376 | | NA |  | NA |  | NA |  | NA | NA |  | NA |  | NA |  |
|  | 1.0436 | 1.06961 | | 1.02498 | | 1.08357 | | NA | 1.00835 | | 1.10885 | | 1.13128 | |
| 1.10651 | | 1.13881 | | 1.03457 | | 1.01708 | | 1.01008 | 0.986733 | | 1.07504 | | 1.07152 | |
| 1.08286 | | 1.10396 | | 0.985597 | | 1.08475 | | 0.920382 | 1.04099 | |  | 1.0376 | 1.08289 | |
| 0.982157 | | 1.00556 | | 0.955489 | | 0.928736 | | 0.978182 | 0.990586 | | 1.02371 | | 1.04833 | |
| 1.03254 | | 0.998917 | | 1.01271 | | 1.00571 | | 0.956638 | 0.965377 | | 1.01591 | | 1.03209 | |
| 1.05371 | | 0.994155 | | 0.961163 | | 1.00435 | | 0.926784 | 1.01155 | | 0.999495 | | 1.05236 | |
| 1.13033 | | 1.02035 | | 0.96524 | |  | 1.0035 | 0.889571 | 0.993275 | | 1.01779 | | 1.06374 | |
| 1.02085 | | 0.987167 | | 1.02028 | | 1.03062 | | 0.895904 | 1.00951 | |  | 1.0422 | 0.97842 | |
| 1.01156 | | 1.04203 | | 1.02396 | |  | 1.0584 | 1.01095 | 0.984048 | | 1.02757 | | 1.04233 | |
| 1.08192 | |  | 1.0764 |  | 1.048 |  | 1.0666 | 0.957483 | 1.02863 | | 1.07257 | |  | 1.0552 |
| 0.926664 | | 0.929675 | | 0.920186 | | 0.949376 | | 0.882212 | 1.01784 | | 0.958485 | | 0.913525 | |
| 1.02156 | | 0.987236 | | 1.01481 | | 1.04564 | | 1.07398 | 1.02029 | | 1.03193 | | 0.980527 | |
| 0.92328 | | 0.956878 | | 0.919349 | | 0.928075 | | 0.936092 | 0.961913 | | 0.91416 | |  | 0.9228 |
| 0.951796 | | 0.971503 | | 0.904934 | | 0.919013 | | 0.913999 | 0.977805 | | 0.950573 | | NA |  |
| 0.942405 | | 0.980413 | | 0.994294 | | 0.998799 | | NA | NA |  | NA |  | NA |  |
| NA |  | NA |  | NA |  | NA |  | NA | NA |  | NA |  | NA |  |
| NA |  | NA |  | NA |  | NA |  | NA | NA |  | NA |  | NA |  |
| 1.07481 | | 1.07113 | | 1.07347 | | NA |  | 1.12201 | 1.06225 | | 1.04044 | | 1.02039 | |
| 1.01607 | | 1.04945 | | 1.03752 | | 0.973309 | | 1.14206 | 0.982091 | | 1.03031 | | 1.04682 | |
| 1.00265 | | 0.990058 | | 1.04837 | | 1.02108 | | 1.12182 | 1.03058 | | 1.04083 | | 1.04218 | |
| NA |  | NA |  | NA |  | NA |  | 1.24124 | 1.06196 | | NA |  | NA |  |

1.0877	1.0418	1.04506	1.01579	1.11967	1.00922	1.02746	1.07318

0.910426	0.9163 0.917636 0.955927 0.860171 0.993519 0.868549 0.895054

1.08949	1.09161	1.13355	1.16402	1.12246	1.22382	1.11881	1.16104

1.03031	1.08723	1.13283	1.09363	1.22836	1.22088	1.06972	1.10212

1.09461	1.10376	1.09594	1.06055	1.1426	1.16583	1.0566	1.06381

0.956549	1.00351 0.926097 0.991742	1.01288	1.04171 0.963333	1.00628

NA	NA	1.08988 NA	1.11988	1.05791 0.971355 0.948799

1.02879	1.10983	1.10638	1.10769	1.17421	1.20437	1.0457	1.06237

1.10933	1.20158	1.10847	1.15086	1.21064	1.1572	1.10934	1.11413

NA	NA	NA	NA	NA	NA	NA	NA NA	NA	NA	NA	NA	NA	NA	NA NA	NA	NA	NA	NA	NA	NA	NA

1.1476	1.17162	1.17086	1.10963	1.09127	1.04971	1.22577	1.23308

1.13611	1.24421	1.18069	1.22657	1.21919	1.21002	1.15803	1.13025

0.9819	0.96725	1.00033 0.965386 0.974928	1.03422 0.913181	0.92421

1.11155	1.13358	1.13958	1.16209	1.05926	1.0996	1.04669	1.11531

0.973906 0.933311 0.961086 0.932953 0.959859 0.999199 0.921918 0.910767

1.03639	1.06664	1.07165	1.06893	1.06239	1.08652	1.06217	1.07522

1.02905	1.0154	1.00979	0.99721	1.00754	1.03389	1.02615	1.03081

NA	NA	NA	NA	NA	NA	NA	NA

1.10642	1.11485	1.08468	1.1484	1.11043	1.1233	1.08086	1.07757

0.958769 0.966288	1.07722	1.00814 0.904594	1.00075	1.03483 0.997465

NA	NA	1.03078 NA	1.02584	1.09553 NA	NA NA	NA	1.03966 NA	1.12947	1.04986 NA	NA

1.03192 0.986418	1.07198	1.00789 0.950967	1.02345	1.03073	1.0172

0.974126	1.0305 0.963481 0.996877 0.880811	1.00463	1.02638	1.03802

0.978045	1.00149 0.983007 0.950023	0.96861	1.03127	1.03411	1.07283

1.08527	1.09901	1.13938	1.07615 0.999067	1.09875	1.07326	1.12464

1.0339 0.981771	1.11833	1.09261 NA	0.989811 0.978782 0.963348

1.17584	1.05267	1.07424	1.21217	1.00701	1.17629	1.08401	1.07942

0.988876	1.02152	1.03016	1.03942	1.11645 0.989099	1.02373	1.06005

1.1764	1.09133	1.05585	1.13245 0.982187	1.06599	1.14475	1.18755

1.05825	1.05195	1.03706	1.00365	0.9477	1.04861	1.08142	1.04491

0.988292 0.991029 0.948791	1.01014	1.07705	1.05257	1.02374 0.955776

1.08758	1.11315	1.074	1.12297	1.09813	1.07287	1.11928	1.14648

1.02255	1.02544	1.03304	1.02393	1.01565 0.991509	1.0335	1.00679

1.03167	1.03482	1.05533	1.01867	1.01899	1.01081	1.03831 0.994133

1.0715	1.04225	1.06008	1.08339	1.01163	1.04344	1.07344	1.01502

1.13248	1.06593	1.12199	1.10095	1.05426	1.0234	1.0204	1.03689

| 1.17146 | |  | 1.032 | 1.01149 | |  | 1.0798 | 1.11111 | |  | 1.0716 | 1.10327 | 1.05263 | |
| --- | --- | --- | --- | --- | --- | --- | --- | --- | --- | --- | --- | --- | --- | --- |
| 1.04025 | | 1.08706 | | 1.11268 | | 1.09436 | | 1.13737 | | 1.07447 | | 1.06458 | 1.10888 | |
|  | 1.1238 | 1.13324 | | 1.21905 | | 1.14371 | | 1.13573 | | 1.19284 | | 1.10221 | 1.13433 | |
| 0.995935 | | 0.941571 | | 1.03228 | | 1.03523 | | 1.01047 | | 0.986842 | | 1.01547 | 1.05572 | |
| 1.12422 | |  | 1.1524 | 1.07532 | | 1.17083 | | 0.900144 | | 1.03436 | | 1.21082 | 1.32718 | |
| NA |  | NA |  | 1.00813 | | 1.00581 | | NA |  | NA |  | NA | NA |  |
| 0.965507 | | 0.975887 | | 0.993933 | | 0.966756 | | 1.00597 | | 0.997206 | | 0.99292 | 0.97224 | |
| 1.00155 | | 0.959381 | | 0.997195 | | 1.00131 | | 0.983191 | | 0.999211 | | 0.962016 | 0.991512 | |
| 1.12475 | |  | 1.1072 | 1.06686 | | 1.12072 | | 0.993139 | | 1.08199 | | 1.07848 | 1.07131 | |
| 1.02392 | | 1.03468 | | 0.900632 | | 1.03569 | | 0.918552 | | 0.942886 | | 1.00074 | 1.00647 | |
| 0.977581 | | 0.976861 | | 0.981068 | | 0.981211 | | 0.886855 | | 1.00105 | | 0.973028 |  | 1.0021 |
| NA |  | NA |  | NA |  | NA |  | NA |  | NA |  | NA | NA |  |
| 1.05578 | | 1.00187 | | 1.03285 | | 1.02829 | | 0.953846 | | 1.02377 | | 0.997692 | 0.975675 | |
| 1.03268 | | 1.02197 | | 1.07612 | | 1.00711 | | 0.971972 | | 1.05824 | | 1.07063 |  | 1.0261 |
| 1.07273 | | 1.03402 | | 1.14168 | | 1.12295 | | 1.05034 | |  | 1.1295 | 1.09213 |  | 1.0698 |
|  | 1.043 | 1.04974 | | 1.05121 | | 1.06875 | | 1.03156 | | 1.02629 | | 1.05887 | 1.03414 | |
| 1.09575 | | 1.08694 | | 1.01336 | | 1.01982 | | 0.931334 | | 1.06309 | | 1.07321 | 1.05251 | |
| 1.02708 | | 0.962728 | | 1.01065 | | 0.982351 | | 1.03686 | | 0.994094 | | 1.10168 | 1.05138 | |
| NA |  | NA |  | NA |  |  | 1.0279 | NA |  | 1.01523 | | NA | NA |  |
| 1.02896 | | 1.00542 | | 1.06764 | | 1.00221 | |  | 1.1636 | 1.00621 | | 1.15503 | 1.10401 | |
| 1.03722 | | 1.05223 | | 1.05478 | | 1.02431 | | 1.02541 | | 1.08932 | | 1.08196 | 1.07702 | |
| 1.01627 | | 0.924084 | | 0.89526 | | 0.991664 | | NA |  | NA |  | 0.898391 | 0.91082 | |
| 0.95624 | | 1.03971 | | 0.965285 | | 0.991398 | | 0.992791 | | 0.902627 | | 1.02118 | 1.06619 | |
| 1.05437 | | 1.00433 | | 0.969732 | |  | 1.0307 | NA |  | NA |  | 0.974698 | 1.02264 | |
| 1.00168 | | 0.999436 | | 0.989711 | | 0.992411 | | 1.02655 | | 0.990129 | | 0.998346 | 1.00384 | |
| 1.17006 | | 1.07756 | | 1.13816 | | 1.06798 | |  | 1.0808 | 1.04737 | | 1.12237 | 1.12033 | |
| 1.04625 | | 1.05404 | |  | 1.057 | 1.05806 | | 1.18727 | | 1.05607 | | 1.03332 | 1.08511 | |
| 0.967531 | | 0.981209 | | 0.980802 | | 1.01597 | | 0.859893 | | 1.03115 | | 1.01483 | 1.05435 | |
| 1.01547 | | 1.00881 | | 0.993948 | | 0.973255 | | 0.883197 | | 1.01295 | | 0.972248 | 0.981974 | |
| 0.972376 | | 0.98183 | | 0.944674 | | 0.980702 | | 0.921776 | | 0.961017 | | 0.933551 | 0.922948 | |
| 1.04542 | | 1.03689 | | 0.989731 | | 0.991275 | | NA |  | 1.00378 | | 1.03615 | 1.01374 | |
| 1.20149 | | 1.20115 | | 1.22821 | | 1.20627 | | 1.07235 | | 1.17208 | | 1.20574 | 1.24065 | |
| 1.02834 | | 0.983182 | | 0.987304 | | 0.985622 | | 1.04939 | | 1.00899 | | 0.97193 | 0.962233 | |
| 1.02851 | | 1.11955 | | 1.02026 | | 1.03084 | |  | 1.1752 | 1.06691 | | 1.08016 | 1.02148 | |
| NA |  | 1.09812 | | 1.09504 | | NA |  | 1.05509 | | 1.08673 | | NA | NA |  |
| 1.06211 | | 1.07115 | | 1.08407 | | 1.03943 | | 1.05181 | | 1.02877 | | 1.12759 | 1.09245 | |
| 1.00984 | | 1.06876 | | 0.966281 | |  | 1.0325 | 0.897321 | | 1.07612 | | 1.14447 |  | 1.0712 |
|  | 1.0745 | 1.09852 | | 1.06694 | | 0.986315 | | 1.17764 | | 0.971163 | | 1.05653 |  | 1.0462 |

conventioncytotoxic_ early_neur effector_meffector_meffector_mendothelia endothelia

1.10909	1.13517	1.59375	1.02814	1.04988	1.10237	1.1505	1.215

NA	NA	NA	NA	NA	NA	NA	NA

1.12054	1.20107 NA	1.27875	1.19589	1.22138	1.09538	1.13785

1.14675	1.23702 NA	1.15237	1.20039	1.23375	1.10032	1.12604

1.0827	1.0849 NA	1.36026	1.3535	1.33616	1.03286	1.05229

1.00084 0.864458	1.08046 0.929911 0.916792 0.968013 0.938158	1.19178

1.09587	1.12868 NA	1.20985	1.19512	1.26906	1.10689	1.24829

1.03578	1.05811 NA	1.17529	1.22637	1.21244	1.11535	1.12464

1.03578	1.05811 NA	1.17529	1.22637	1.21244	1.11535	1.12464

1.17139	1.16337 NA	1.00922	1.11367	1.03869	1.07706 NA

1.03749 0.911111 NA	0.972144 0.904496 0.963471	1.0621 0.981982

1.00754 0.871278 NA	0.952736	0.94278	1.02254	1.01746	1.01942

1.05353 0.961679 NA	1.03096 0.951097 0.987826 0.953314	0.78178

1.08547 0.994012 NA	1.01191 0.937688	1.00754 0.899106 0.753153

NA	NA	NA	NA	NA	NA	NA	NA NA	NA	1.21099 NA	NA	NA	NA	NA

0.992751 NA	NA	1.02719	1.056	1.01777 NA	NA

0.992751 NA	NA	1.02719	1.056	1.01777 NA	NA

0.960622 0.986911 NA	0.96449 0.957002 0.977319 0.908152 NA

1.13123	1.09277 NA	1.36821	1.34611	1.34105	1.16055	1.13102

NA	NA	1.22764 NA	NA	NA	NA	NA

1.03306	0.98931 NA	1.0001	1.04793	1.01525 0.972502 0.900361

NA	NA	NA	NA	NA	NA	NA	NA NA	NA	NA	NA	NA	NA	NA	NA

1.05315 NA	NA	NA	NA	NA	NA	NA

1.07169 NA	NA	NA	NA	NA	NA	NA

1.12289 NA	NA	NA	NA	NA	NA	NA

1.11561 NA	NA	NA	NA	NA	NA	NA

1.00198	1.03662 NA	1.26867	1.30163	1.28636	1.11583 0.966767

1.00198	1.03662 NA	1.26867	1.30163	1.28636	1.11583 0.966767

1.08971	1.0726 NA	1.3054	1.36886	1.42798	1.14463	1.01493

1.12005	1.10099 NA	1.3012	1.41761	1.48373	1.14437 0.956341

1.14067 NA	NA	1.03739 NA	1.01005	1.07072 NA

0.92431 NA	NA	NA	NA	NA	NA	NA NA	NA	NA	NA	NA	NA	NA	NA NA	NA	NA	NA	NA	NA	NA	NA NA	NA	1.14408 NA	NA	NA	NA	NA

1.07706	1.0541 0.742512	1.09006	1.05298	1.05728	1.11979 0.947463

| 0.933819 | | 0.947081 | |  | 0.8 | 0.933105 | | 0.944411 | | 0.942346 | | 0.933014 | | 0.913621 | |
| --- | --- | --- | --- | --- | --- | --- | --- | --- | --- | --- | --- | --- | --- | --- | --- |
| 0.933819 | | 0.947081 | |  | 0.8 | 0.933105 | | 0.944411 | | 0.942346 | | 0.933014 | | 0.913621 | |
| 0.933819 | | 0.947081 | |  | 0.8 | 0.933105 | | 0.944411 | | 0.942346 | | 0.933014 | | 0.913621 | |
| 0.968801 | | 0.893477 | | 0.800608 | | 1.04238 | | 1.01249 | | 1.02301 | | 1.08376 | | 0.968226 | |
| 0.957315 | | 1.00394 | | 0.759354 | | 1.07192 | |  | 1.0612 | 1.05167 | | 0.960165 | | 1.03569 | |
| 0.926629 | | 1.07338 | | NA |  | 1.00239 | | 1.00688 | | 0.965817 | | 0.981089 | | 1.03849 | |
| 1.05139 | | 1.08836 | | NA |  | 1.02398 | | 0.973515 | | 0.969536 | | 0.985251 | | 1.12696 | |
| 1.05001 | |  | 1.0222 | NA |  | 1.00765 | | 0.983111 | | 1.01998 | | 1.05354 | | 1.09272 | |
| 0.995596 | | 1.07277 | | NA |  | 0.989525 | | 0.976447 | | 1.01259 | | 0.97365 | | 0.973232 | |
| 1.04962 | | 1.11876 | | NA |  | 1.03224 | |  | 1.0298 | 1.06174 | | 1.02479 | | 1.06577 | |
| 1.06189 | | 1.11136 | | NA |  |  | 1.0199 | 1.00511 | | 1.05687 | | 1.01341 | | 1.13355 | |
| 0.993657 | | 1.07227 | | NA |  | 1.03675 | | 0.995256 | | 0.985635 | | 0.993674 | | 0.994424 | |
| 1.04272 | | 1.16388 | | 1.12291 | |  | 1.0032 | 1.00533 | | 0.99047 | | 1.05018 | | 1.05937 | |
| 1.05432 | | 1.14371 | | NA |  | 0.998458 | | 0.983357 | | 0.998014 | | 0.949333 | | 0.99435 | |
| 1.01885 | | 1.06291 | | 1.29167 | | 0.95658 | | 0.920922 | | 0.948428 | | 1.02036 | | 0.948548 | |
| NA |  | NA |  | NA |  | NA |  | NA |  | NA |  | NA |  | NA |  |
| 1.03824 | | 1.13006 | | NA |  | 1.26687 | |  | 1.2631 | 1.26518 | | 1.13944 | | 1.21324 | |
|  | 1.0701 | 1.10487 | | NA |  | 1.00432 | | 0.985857 | | 1.03048 | | 1.09434 | | 0.979757 | |
| 1.05314 | | 1.06922 | | NA |  |  | 1.0107 | 1.02004 | | 1.01839 | | 0.974623 | | 1.00633 | |
| 1.15878 | | 1.23276 | | NA |  |  | 1.0144 | 1.02709 | | 0.950661 | | 1.05298 | | 0.925926 | |
| 0.975449 | | 1.01629 | | NA |  | 1.01051 | | 0.969357 | | 0.972141 | | 0.97544 | | 0.971969 | |
| NA |  | NA |  | NA |  | NA |  | 1.02696 | | 1.02118 | | 1.02459 | | 1.17521 | |
| NA |  | NA |  | NA |  | NA |  | NA |  | NA |  | NA |  | NA |  |
| 1.01948 | |  | 1.0687 | NA |  | 0.998677 | | 0.977482 | | 1.02601 | | 0.96863 | | 1.02452 | |
| 1.08594 | | 1.16814 | | NA |  | 1.09661 | |  | 1.1269 | 0.939335 | |  | 1.2638 | 0.971429 | |
|  | 1.0231 | 1.05686 | | NA |  | 1.02735 | |  | 1.0568 | 1.05244 | | 1.08696 | | 0.97832 | |
| 1.03587 | | 1.08474 | | 0.907407 | | 1.00479 | | 1.01336 | | 1.04464 | | 1.03397 | | 0.974565 | |
| 1.01957 | | 0.992091 | | NA |  | 1.01188 | | 0.981915 | | 1.01506 | | 0.972852 | | 1.02057 | |
| 1.07479 | | 1.14484 | | NA |  | 0.966341 | | 1.00359 | | 0.96971 | | 1.11542 | | 1.07048 | |
| 1.07479 | | 1.14484 | | NA |  | 0.966341 | | 1.00359 | | 0.96971 | | 1.11542 | | 1.07048 | |
| 1.03411 | | 1.05785 | | NA |  | 1.00267 | | 0.99007 | | 1.02063 | | 1.04388 | | 1.00204 | |
| 1.04634 | | 1.09383 | | NA |  | 1.04615 | | 0.967553 | | 0.999682 | | 0.971993 | | 0.909931 | |
|  | 1.1032 |  | 1.1015 | NA |  | 1.03063 | | 0.967873 | | 1.03151 | | 1.03929 | | 1.22477 | |
| 1.00814 | | 0.947398 | | NA |  | 1.03513 | |  | 1.0265 | 1.02824 | |  | 1.0051 | 1.15366 | |
| NA |  | NA |  | NA |  | NA |  | NA |  | NA |  | NA |  | NA |  |
| 0.981486 | | 0.85108 | | NA |  | 1.03566 | | 1.01591 | |  | 1.1127 | 1.08851 | | 1.08184 | |
| 1.03435 | | 1.11352 | | NA |  | 1.03241 | | 0.976111 | | 1.00407 | | 1.00845 | |  | 1.0372 |
| 1.00608 | | 1.05752 | | NA |  |  | 1.0212 | 0.99543 | | 1.02057 | | 1.01291 | | 1.00715 | |
| 1.22834 | | 1.26862 | |  | 0.9 | 1.11857 | | 1.14539 | | 1.00321 | | 1.22477 | | 1.23077 | |

| 0.981598 | | 1.03713 | | NA |  | 1.02741 | 0.966228 | 1.03585 | 0.989728 | | 0.864465 |
| --- | --- | --- | --- | --- | --- | --- | --- | --- | --- | --- | --- |
| 0.922276 | | 0.881294 | | NA |  | 0.985971 | 0.924314 | 0.960367 | 0.973099 | | 0.853333 |
| 0.982696 | | 0.948455 | | NA |  | 0.998798 | 0.936521 | 0.991314 | 0.893689 | | 0.727794 |
| 0.999937 | | 0.955293 | | NA |  | 1.02201 | 0.960291 | 1.01177 |  | 1.0509 | 0.839341 |
| NA |  | NA |  | NA |  | NA | NA | NA | NA |  | NA |
| NA |  | NA |  | NA |  | NA | NA | NA | NA |  | NA |
| NA |  | NA |  | NA |  | NA | NA | NA | NA |  | NA |
| NA |  | NA |  | NA |  | NA | NA | NA | NA |  | NA |
| NA |  | NA |  | NA |  | NA | NA | NA | NA |  | NA |
| NA |  | NA |  | NA |  | NA | NA | NA | NA |  | NA |
| NA |  | NA |  | NA |  | NA | NA | NA | NA |  | NA |
| NA |  | NA |  | NA |  | NA | NA | NA | NA |  | NA |
| NA |  | NA |  | NA |  | NA | NA | NA | NA |  | NA |
| NA |  | NA |  | NA |  | NA | NA | NA | NA |  | NA |
| NA |  | NA |  | NA |  | NA | NA | NA | NA |  | NA |
| NA |  | NA |  | NA |  | NA | NA | NA | 1.08283 | | NA |
| NA |  | NA |  | NA |  | NA | NA | NA | NA |  | NA |
| NA |  | NA |  | NA |  | NA | NA | NA | NA |  | NA |
| NA |  | NA |  | NA |  | NA | NA | NA | 1.09847 | | 1.05047 |
| NA |  | NA |  | NA |  | NA | NA | NA | 1.03133 | | NA |
| NA |  | NA |  | NA |  | NA | NA | NA | NA |  | NA |
| 1.05441 | | 1.08705 | |  | 1.1875 | 0.987387 | 0.994052 | 1.02206 |  | 1.0103 | 1.02407 |
| 0.987035 | | 0.952157 | | NA |  | 1.03976 | 0.994816 | 0.942041 | 0.941141 | | NA |
| 0.987035 | | 0.952157 | | NA |  | 1.03976 | 0.994816 | 0.942041 | 0.941141 | | NA |
| NA |  | NA |  | NA |  | NA | NA | NA | NA |  | NA |
| NA |  | NA |  | NA |  | NA | NA | NA | NA |  | NA |
| NA |  | NA |  | NA |  | NA | NA | NA | NA |  | NA |
| NA |  | 0.970461 | | NA |  | NA | NA | NA | 1.17153 | | NA |
| NA |  | NA |  | NA |  | NA | NA | NA | NA |  | NA |
| NA |  | NA |  | NA |  | NA | NA | NA | NA |  | NA |
| NA |  | NA |  | NA |  | NA | NA | NA | NA |  | NA |
| NA |  | NA |  | NA |  | NA | NA | NA | NA |  | NA |
| NA |  | NA |  | NA |  | NA | NA | NA | NA |  | NA |
| NA |  | NA |  | 1.20025 | | NA | NA | NA | NA |  | NA |
| NA |  | NA |  | 1.04305 | | NA | NA | NA | NA |  | NA |
| NA |  | NA |  | 1.00787 | | NA | NA | NA | NA |  | NA |
|  | 0.9334 | 0.938462 | | NA |  | 0.986801 | 0.942782 | 0.95766 | 0.953984 | | 1.19081 |
| 1.06374 | |  | 1.1928 | 0.925714 | | 1.14304 | 1.19035 | 1.23873 | 1.04686 | | 0.844086 |
| 1.06374 | |  | 1.1928 | 0.925714 | | 1.14304 | 1.19035 | 1.23873 | 1.04686 | | 0.844086 |

| 1.07047 | | 1.25159 | | 0.834656 | | 1.27153 | 1.35685 | | 1.38291 | 1.14204 | 1.06707 |
| --- | --- | --- | --- | --- | --- | --- | --- | --- | --- | --- | --- |
| NA |  |  | 1.0622 | NA |  | 1.06741 | 1.26249 | | 1.22487 | NA | 1.06452 |
| 1.13607 | | 0.994138 | | 1.45455 | | 1.03478 | 1.08083 | | 1.04373 | 1.10837 | 1.36937 |
| 0.923458 | | 1.01743 | | NA |  | 0.982201 | 0.970879 | | 1.01018 | 1.05094 | 1.06265 |
| 1.08221 | | 1.05487 | | 0.878788 | | 1.07262 | 1.03573 | | 1.03054 | 0.989901 | 0.980645 |
|  | 1.0003 | NA |  | NA |  | NA | NA |  | NA | NA | NA |
| NA |  | NA |  | NA |  | NA | NA |  | NA | NA | NA |
| NA |  | NA |  | NA |  | NA | NA |  | NA | NA | NA |
| 0.961131 | | NA |  | 1.27451 | | 0.979638 | 0.962847 | | 1.01849 | 0.940851 | 0.931751 |
| 1.00905 | | NA |  | 2.28571 | | 1.07323 |  | 1.0354 | 1.07553 | 1.02649 | 0.972222 |
| 0.926269 | | 0.903456 | | 0.861538 | | 0.924385 | 0.946213 | | 1.00507 | 0.956967 | 0.977273 |
| NA |  | NA |  | 1.03774 | | NA | NA |  | NA | NA | NA |
| NA |  | NA |  | NA |  | NA | NA |  | NA | NA | NA |
| NA |  | NA |  | NA |  | NA | NA |  | NA | NA | NA |
| NA |  | NA |  | NA |  | NA | NA |  | NA | NA | NA |
| NA |  | NA |  | NA |  | NA | NA |  | NA | NA | NA |
| NA |  | NA |  | NA |  | NA | NA |  | NA | NA | NA |
| NA |  | NA |  | NA |  | NA | NA |  | NA | NA | NA |
| NA |  | NA |  | NA |  | NA | NA |  | NA | NA | NA |
| NA |  | NA |  | NA |  | NA | NA |  | NA | NA | NA |
| NA |  | NA |  | NA |  | NA | NA |  | NA | NA | NA |
| NA |  | NA |  | 0.923126 | | NA | NA |  | NA | NA | NA |
| NA |  | NA |  | 0.782353 | | NA | NA |  | NA | NA | NA |
| NA |  | NA |  |  | 1.08 | NA | NA |  | NA | NA | NA |
| NA |  | NA |  | NA |  | NA | NA |  | NA | NA | NA |
| NA |  | NA |  | NA |  | NA | NA |  | NA | NA | NA |
| NA |  | NA |  | NA |  | NA | NA |  | NA | NA | NA |
| NA |  | NA |  | NA |  | NA | NA |  | NA | NA | NA |
| NA |  | NA |  | NA |  | NA | NA |  | NA | NA | NA |
| NA |  | NA |  | NA |  | NA | NA |  | NA | NA | NA |
| NA |  | NA |  | NA |  | NA | NA |  | NA | NA | NA |
| NA |  | NA |  | NA |  | NA | NA |  | NA | NA | NA |
| NA |  | NA |  | NA |  | NA | NA |  | NA | NA | NA |
| NA |  | NA |  | NA |  | NA | NA |  | NA | NA | NA |
| NA |  | NA |  | NA |  | NA | NA |  | NA | NA | NA |
| NA |  | NA |  | NA |  | NA | NA |  | NA | NA | NA |
| NA |  | NA |  | NA |  | NA | NA |  | NA | NA | NA |
| NA |  | NA |  | NA |  | NA | NA |  | NA | NA | NA |
| NA |  | NA |  | NA |  | NA | NA |  | NA | NA | NA |

| NA |  | NA | 0.921429 | | NA |  | NA |  | NA |  | NA | NA |
| --- | --- | --- | --- | --- | --- | --- | --- | --- | --- | --- | --- | --- |
| NA |  | NA | NA |  | NA |  | NA |  | NA |  | NA | NA |
| NA |  | NA | NA |  | NA |  | NA |  | NA |  | NA | NA |
| NA |  | NA | NA |  | NA |  | NA |  | NA |  | NA | NA |
| NA |  | NA | NA |  | NA |  | NA |  | NA |  | NA | NA |
| NA |  | NA | NA |  | NA |  | NA |  | NA |  | NA | NA |
| NA |  | NA | NA |  | NA |  | NA |  | NA |  | NA | NA |
| NA |  | NA | NA |  | NA |  | NA |  | NA |  | NA | NA |
| NA |  | NA | 1.02357 | | NA |  | NA |  | NA |  | NA | NA |
| NA |  | NA | NA |  | NA |  | NA |  | NA |  | NA | NA |
| 0.945157 | | 0.895512 | NA |  | 0.945281 | | 0.962532 | | 0.967721 | | 0.830375 | 1.01476 |
| 1.02086 | | 0.98439 | NA |  | 1.26742 | |  | 1.21 | 1.21363 | | NA | NA |
| NA |  | NA | NA |  | 1.00812 | | NA |  | NA |  | NA | NA |
| 0.919495 | | 0.93825 | NA |  | 1.02616 | | 1.06928 | | 1.05958 | | 0.984871 | NA |
| 1.04285 | | 0.765993 | NA |  | 1.01402 | | 1.04019 | | 1.14481 | | 0.981982 | NA |
| 1.04514 | | 1.03379 | NA |  | 1.10995 | | 1.11564 | | 1.14192 | | NA | NA |
| 1.05299 | | 1.12058 | NA |  | 1.14739 | | 1.19057 | | 1.21538 | | NA | NA |
| NA |  | NA | NA |  | 1.05456 | | 1.07454 | | 1.11164 | | NA | NA |
| 0.964295 | | 0.995723 | NA |  | 1.03262 | | 1.06155 | | 1.11136 | | 0.900906 | NA |
| 1.12607 | | 1.01714 | NA |  | 1.07393 | | 1.04829 | | 1.10813 | | NA | NA |
| 0.979116 | | 0.949666 | NA |  | 1.04203 | | 0.959357 | | 1.02078 | | 0.972554 | 0.866397 |
| 0.960245 | | 0.921992 | NA |  |  | 1.009 | 0.931458 | | 0.996063 | | 1.01983 | 0.846743 |
| 0.981286 | | 0.83745 | NA |  |  | 1.0168 | 0.978352 | | 0.971474 | | 0.934772 | 0.738462 |
| 0.977401 | | NA | NA |  | 1.01808 | | 0.965781 | | 1.01079 | | 1.04699 | 0.858804 |
| 1.03585 | | 0.997521 | NA |  | 0.995494 | | 1.00676 | | 1.03114 | | 1.00753 | NA |
| 1.02952 | | 0.995837 | 1.18421 | | 1.04249 | | 1.02185 | | 1.05198 | | 1.02752 | NA |
| 1.04523 | | 1.06474 | NA |  | 1.04439 | | 1.15797 | |  | 1.1141 | 1.08466 | 1.17383 |
|  | 1.05 | 1.23022 | NA |  | 1.07578 | | 1.14516 | | 1.14274 | | 1.00974 | 1.34043 |
|  | 1.05 | 1.23022 | NA |  | 1.07578 | | 1.14516 | | 1.14274 | | 1.00974 | 1.34043 |
| 1.04645 | | 1.03117 | 1.26087 | | 1.09527 | | 1.15593 | | 1.11934 | | 1.01204 | 1.00737 |
| 1.00242 | | 1.01315 | 0.882353 | | 1.01715 | | 1.02976 | | 1.05214 | | 0.978687 | NA |
|  | 1.0179 | 1.02648 | 0.651515 | | 0.99373 | | 1.04722 | | 1.06152 | | 0.966807 | NA |
|  | 1.0179 | 1.02648 | 0.651515 | | 0.99373 | | 1.04722 | | 1.06152 | | 0.966807 | NA |
|  | 1.0179 | 1.02648 | 0.651515 | | 0.99373 | | 1.04722 | | 1.06152 | | 0.966807 | NA |
| 0.961688 | | 0.953605 |  | 1.25 | 0.942497 | | 0.981768 | | 0.978778 | | 1.09766 | 1.19723 |
| 1.05149 | | 0.974619 | 0.765991 | | 0.965775 | | 0.988758 | | 1.05155 | | 1.00659 | 1.15359 |
| NA |  | 1.01121 | NA |  | 1.02948 | | 1.09689 | | 1.10109 | | 1.02428 | NA |
| NA |  | NA | 1.27215 | | NA |  | NA |  | NA |  | NA | NA |
| NA |  | NA | 0.83871 | | 1.04355 | | 1.06447 | | 1.05174 | | NA | NA |

| NA |  | NA |  |  | 1.08 | NA |  | NA |  | NA |  | NA |  | NA |  |
| --- | --- | --- | --- | --- | --- | --- | --- | --- | --- | --- | --- | --- | --- | --- | --- |
| NA |  | NA |  | NA |  | NA |  | NA |  | NA |  | NA |  | NA |  |
| NA |  | NA |  | NA |  | NA |  | NA |  | NA |  | NA |  | NA |  |
| NA |  | NA |  | NA |  | NA |  | NA |  | NA |  | NA |  | NA |  |
| 1.01925 | | 0.951974 | |  | 1.3773 | 0.943077 | | 0.968769 | | 0.981849 | | 1.00832 | | 1.18901 | |
| 1.09765 | | 1.00044 | |  | 2.345 | 1.02428 | | 1.00696 | | 1.02268 | | 1.13153 | | 1.02459 | |
| 0.971802 | | 0.988755 | | 0.655172 | | 1.02918 | | 1.09843 | | 1.04869 | | 1.00106 | |  | 1 |
|  | 1.098 | 1.09716 | | 1.18056 | | 1.01214 | | 1.05036 | |  | 1.032 | 1.04401 | | 1.23305 | |
| 0.93998 | | 0.976601 | | NA |  | 0.966677 | | 0.939888 | | 0.985108 | | 1.12562 | | 1.11556 | |
| 0.966491 | | 1.06786 | | 1.07143 | | 1.06808 | | 0.972713 | |  | 1.0438 | 1.04917 | | 0.903114 | |
| 1.02759 | | 1.11981 | | NA |  | 1.22883 | | 1.28561 | | 1.27549 | | 1.10197 | | 1.09263 | |
| 1.15334 | | 0.998862 | |  | 1.4153 | 1.03073 | | 1.02044 | | 1.02925 | | 1.16114 | |  | 1.1511 |
|  | 1.1688 | 1.12001 | |  | 1.588 | 1.01398 | | 1.01394 | |  | 1.0148 | 1.30201 | | 1.14678 | |
| 0.950109 | | 1.01738 | | NA |  | 0.961441 | | 0.944718 | | 1.00304 | | 0.938026 | | NA |  |
| NA |  | NA |  | 1.16082 | | NA |  | NA |  | NA |  | NA |  | NA |  |
| NA |  | NA |  | 1.16297 | | NA |  | NA |  | NA |  | NA |  | NA |  |
|  | 1.0835 | 1.05062 | | NA |  | 1.20428 | | 1.21315 | | 1.23476 | | 1.13648 | | NA |  |
| NA |  | NA |  | NA |  | NA |  | NA |  | NA |  | NA |  | NA |  |
| 0.923703 | | 0.944915 | | NA |  | 0.92126 | | 0.973296 | | 0.93321 | | 0.973636 | | 0.97251 | |
| NA |  | NA |  | 1.47826 | | NA |  | NA |  | NA |  | NA |  | NA |  |
| NA |  | NA |  | 1.47826 | | NA |  | NA |  | NA |  | NA |  | NA |  |
| 1.14243 | | 1.23766 | | 1.32353 | | 1.20858 | | 1.19444 | | 1.11315 | |  | 1.302 | 1.21012 | |
|  | 1.0325 |  | 1.1097 | NA |  | 1.25455 | | 1.30446 | | 1.29341 | | 1.18817 | |  | 1.2984 |
|  | 1.0325 |  | 1.1097 | NA |  | 1.25455 | | 1.30446 | | 1.29341 | | 1.18817 | |  | 1.2984 |
|  | 1.0325 |  | 1.1097 | NA |  | 1.25455 | | 1.30446 | | 1.29341 | | 1.18817 | |  | 1.2984 |
|  | 1.0325 |  | 1.1097 | NA |  | 1.25455 | | 1.30446 | | 1.29341 | | 1.18817 | |  | 1.2984 |
| 1.03547 | | 1.07784 | | 0.783784 | | 1.06006 | | 1.12147 | | 1.08609 | | 1.06639 | | 1.11811 | |
| 1.03547 | | 1.07784 | | 0.783784 | | 1.06006 | | 1.12147 | | 1.08609 | | 1.06639 | | 1.11811 | |
| 1.03547 | | 1.07784 | | 0.783784 | | 1.06006 | | 1.12147 | | 1.08609 | | 1.06639 | | 1.11811 | |
| 1.03547 | | 1.07784 | | 0.783784 | | 1.06006 | | 1.12147 | | 1.08609 | | 1.06639 | | 1.11811 | |
| 1.09246 | | 1.13137 | | 0.646154 | | 1.09212 | |  | 1.1782 | 1.11475 | | 1.05842 | | 1.10646 | |
| 1.09246 | | 1.13137 | | 0.646154 | | 1.09212 | |  | 1.1782 | 1.11475 | | 1.05842 | | 1.10646 | |
| 1.00785 | |  | 1.0283 | 0.714286 | | 1.09963 | | 1.04184 | | 1.08655 | | 0.894273 | | NA |  |
| 1.00785 | |  | 1.0283 | 0.714286 | | 1.09963 | | 1.04184 | | 1.08655 | | 0.894273 | | NA |  |
| 1.06156 | | 1.19936 | | NA |  |  | 1.3599 | 1.46711 | | 1.44405 | | 1.09962 | |  | 1.1052 |
| 0.925796 | | 0.929351 | | 1.08333 | | 0.91613 | | 0.929366 | | 0.973856 | | 0.951132 | | 1.03929 | |
| 0.925796 | | 0.929351 | | 1.08333 | | 0.91613 | | 0.929366 | | 0.973856 | | 0.951132 | | 1.03929 | |
| 0.984666 | | 0.847531 | | NA |  | 0.886264 | | 0.927096 | | 0.947554 | | 1.01645 | | 1.08562 | |
| 0.984666 | | 0.847531 | | NA |  | 0.886264 | | 0.927096 | | 0.947554 | | 1.01645 | | 1.08562 | |

| 0.982332 | | 0.974066 | | 1.25786 | | 0.994469 | | 0.964753 | | 1.02376 | | 1.03429 | | 0.994681 | |
| --- | --- | --- | --- | --- | --- | --- | --- | --- | --- | --- | --- | --- | --- | --- | --- |
| 1.01826 | | 0.929718 | | 0.96875 | | 0.899635 | | 0.917793 | | 0.922624 | | 1.02193 | | 1.10266 | |
| 1.02206 | | 0.942907 | | NA |  | 1.01471 | | 1.01413 | | 1.01914 | | 1.02838 | | 0.927894 | |
| 0.957388 | | 0.863187 | | NA |  | 0.991506 | | 0.961729 | | 1.01988 | | NA |  | NA |  |
| 0.996459 | | 1.03113 | | NA |  |  | 1.1604 | 1.00928 | | 1.01406 | | 1.00385 | | 0.950311 | |
| 0.960073 | | NA |  | NA |  | 1.01231 | | 0.942792 | | 1.04183 | | 0.96781 | | 0.837963 | |
| 1.04028 | | 0.981839 | | 0.981132 | | 1.11751 | | 0.96009 | | 1.00818 | | 1.10461 | | 1.01212 | |
| 0.962175 | | 1.02384 | | NA |  | 1.02681 | | 1.03468 | | 1.04489 | | 1.04605 | | 1.12112 | |
| 0.996958 | |  | 1.0318 | NA |  | 1.06984 | | 1.06048 | | 1.04336 | | 1.05467 | | 1.09688 | |
| NA |  | NA |  | NA |  | 0.966288 | | 1.00827 | | 0.998738 | |  | 1.0372 |  | 1.0569 |
| 0.955994 | | 0.937317 | | NA |  | 1.07996 | | 1.09328 | | 1.05086 | | 0.979483 | | 1.01626 | |
| 1.00218 | | 0.949379 | | NA |  | 0.98147 | | 0.991504 | | 1.00737 | | 1.02848 | | 0.908009 | |
| 0.932263 | | NA |  | NA |  | 0.992769 | | 0.991938 | | 0.95705 | | NA |  | NA |  |
|  | 1.0226 | 0.99722 | | 1.36842 | | 0.947979 | | 1.01844 | | 0.957537 | | 1.16085 | | 1.06784 | |
| NA |  | NA |  | NA |  | 1.06396 | |  | 1.0929 | 1.04917 | | NA |  | NA |  |
| NA |  | NA |  | NA |  | NA |  | NA |  | NA |  | NA |  | NA |  |
| NA |  | NA |  | NA |  | NA |  | NA |  | NA |  | NA |  | NA |  |
| NA |  | NA |  | NA |  | 0.992762 | | 0.988451 | | 0.980808 | | NA |  | NA |  |
| NA |  | NA |  | NA |  | NA |  | NA |  | NA |  | NA |  | NA |  |
| NA |  | NA |  | NA |  | NA |  | NA |  | NA |  | NA |  | NA |  |
| NA |  | NA |  | NA |  | NA |  | NA |  | NA |  | NA |  | NA |  |
| NA |  | NA |  | NA |  | NA |  | NA |  | 0.955316 | | NA |  | NA |  |
| NA |  | NA |  | NA |  | 1.00179 | | 1.05622 | | 1.00589 | | NA |  | NA |  |
| NA |  | NA |  | NA |  | 0.995236 | | 0.971022 | | 0.991082 | | NA |  | NA |  |
| 0.97243 | | 0.915196 | | NA |  | 0.987858 | | 0.949837 | | 0.997903 | | NA |  | NA |  |
| NA |  | NA |  | NA |  | NA |  | NA |  | NA |  | NA |  | NA |  |
| NA |  | NA |  | 1.11483 | | NA |  | NA |  | NA |  | NA |  | NA |  |
| NA |  | NA |  |  | 1.246 | NA |  | NA |  | NA |  | NA |  | NA |  |
| 1.07483 | | 1.17199 | | NA |  | 1.32253 | | 1.42288 | | 1.39461 | | 1.11427 | | 1.08259 | |
| 1.04951 | | 0.983541 | | 0.849315 | | 1.05491 | | 1.03674 | | 1.05573 | | 1.12192 | | 0.868474 | |
| 1.04951 | | 0.983541 | | 0.849315 | | 1.05491 | | 1.03674 | | 1.05573 | | 1.12192 | | 0.868474 | |
| NA |  | NA |  |  | 1.5 | NA |  | NA |  | NA |  | 1.13462 | | NA |  |
| 1.07152 | | 0.946265 | | 0.444444 | | 1.03928 | | 1.06895 | | 1.08157 | | 0.956316 | | 1.12243 | |
| NA |  | NA |  | 0.821429 | | 1.02681 | | 1.00548 | | 1.00009 | | 0.956427 | | 0.929188 | |
| 0.94078 | | NA |  | 1.21311 | | 1.00536 | | 1.03795 | | 1.02846 | | 1.01849 | | 0.873118 | |
|  | 1.0888 | 1.01581 | | 0.925926 | | 0.964949 | | 0.977853 | |  | 1.0598 | 0.986898 | | 1.01437 | |
| 1.03679 | | NA |  | 1.10714 | | 1.07917 | | 1.02734 | | 1.07795 | | 1.06092 | | 0.981073 | |
| NA |  | NA |  | 1.09524 | | NA |  | 1.00231 | | 1.07533 | | 0.909726 | | NA |  |
| 1.02192 | | NA |  | 1.57143 | | 1.05363 | | 1.09319 | | 1.07267 | | 1.03261 | | 0.938008 | |

| 0.907504 | | NA |  | 0.885246 | | 1.01925 | | 1.00306 | | 1.00961 | | 0.822709 | 0.766667 | |
| --- | --- | --- | --- | --- | --- | --- | --- | --- | --- | --- | --- | --- | --- | --- |
| NA |  | NA |  | NA |  | NA |  | NA |  | NA |  | 0.810074 | 0.874154 | |
| NA |  | NA |  | NA |  | NA |  | NA |  | NA |  | 0.916437 | 1.03309 | |
| NA |  | NA |  | NA |  | 1.00513 | | 1.00322 | |  | 1.039 | 1.07752 | 0.952824 | |
| 1.01293 | | NA |  | NA |  | 0.995534 | | 1.04737 | | 1.05164 | | 0.986387 | 0.956039 | |
| 1.06657 | | 0.994544 | | 1.86124 | | 1.05372 | | 0.997373 | |  | 1.0303 | 1.07041 | 1.07264 | |
| NA |  | NA |  | NA |  | NA |  | NA |  | NA |  | NA | NA |  |
| NA |  | NA |  | NA |  | NA |  | NA |  | NA |  | NA | NA |  |
| NA |  | NA |  | NA |  | NA |  | NA |  | NA |  | NA | NA |  |
| NA |  | NA |  | NA |  | NA |  | NA |  | NA |  | NA | NA |  |
| 0.947318 | | 0.957854 | |  | 1 | 0.988354 | | 0.912201 | | 0.936426 | | 0.909774 |  | 0.96 |
| 1.04063 | | 0.975097 | | 0.918919 | | 1.03521 | | 0.994396 | | 1.04875 | | 1.12197 | NA |  |
| 1.03731 | | 1.00964 | | 0.883155 | | 1.12525 | | 1.13072 | | 1.10408 | | 1.08231 | 1.09723 | |
| NA |  | NA |  | 1.15217 | | NA |  | NA |  | NA |  | NA | NA |  |
|  | 1.1302 | 1.09652 | | 0.789474 | | 1.03662 | | 0.982585 | | 1.00368 | | 1.04908 | 0.882943 | |
| 1.16449 | |  | 1.0742 |  | 1 | 1.04015 | | 1.01162 | | 1.05109 | | NA | NA |  |
| 1.06597 | | NA |  | 0.72639 | | 1.08251 | | 1.10123 | | 1.11652 | | 1.02229 | 0.785019 | |
| 0.996635 | | 1.01156 | | 0.909091 | | 1.02524 | | 0.989184 | | 1.01304 | | 1.04412 | 1.05654 | |
| NA |  | NA |  | 1.05806 | | NA |  | NA |  | NA |  | NA | NA |  |
| 0.942985 | | 0.957706 | | 1.06061 | | 1.02512 | | 0.985314 | | 0.996003 | | 1.04141 | 0.970326 | |
| 0.998756 | | 1.01684 | | 0.997707 | |  | 1.0054 | 1.00941 | | 1.02004 | | 1.00021 | 0.995793 | |
| 0.900939 | | NA |  |  | 1.2381 | 0.946668 | | 0.97535 | | 0.994485 | | 1.03402 | 1.03268 | |
| 0.910897 | | NA |  | 1.17647 | | 0.991591 | | 0.952069 | | 0.99197 | | 1.03704 | 0.903424 | |
| 1.10203 | | 1.15378 | | 1.15219 | | 0.980375 | | 0.915132 | | 0.939216 | | 1.14192 | 1.08383 | |
| 1.11279 | | 0.864799 | | 1.34783 | | 1.02032 | |  | 1.0395 | 1.05149 | | 1.01094 | 1.04422 | |
| 0.976676 | | 1.06104 | | 0.382353 | | 1.06885 | | 0.996064 | | 1.04955 | | 0.992126 | 0.835634 | |
| 0.985002 | | 0.952968 | | 1.31579 | | 0.972678 | | 1.05114 | | 0.998854 | | 1.11349 | 1.07942 | |
| 0.99471 | | 0.908285 | | 0.818777 | | 0.908077 | | 0.94618 | | 0.937588 | | 1.09194 | 0.897527 | |
| 1.00881 | | 0.980444 | |  | 1.4 |  | 1.1072 | 1.11101 | | 1.10793 | | 0.93101 | 0.839823 | |
| 0.986876 | | 0.988127 | | 1.13947 | | 1.02698 | | 1.01443 | | 1.05812 | | 1.00357 | 0.962199 | |
|  | 1 |  | 1.0303 | 1.42857 | |  | 1.0163 | 0.935294 | | 1.03465 | | 1.14474 | 1.21739 | |
| 1.06318 | | 0.955769 | | 0.565217 | | 1.07669 | | 1.06619 | | 1.10717 | | 1.02669 | 0.912791 | |
| 0.876454 | | 0.843091 | | 1.36364 | | 0.977358 | | 1.00343 | | 0.932798 | | 1.00689 | 1.19751 | |
| 1.02027 | | 0.965753 | | 1.27586 | |  | 1.0426 | 1.02741 | | 1.01207 | | 1.02058 | 1.19433 | |
|  | 1.0237 | 0.934641 | | 0.647059 | | 1.06706 | | 1.01035 | | 1.04963 | | 1.00673 | 1.08333 | |
| NA |  | 0.935484 | |  | 0.5625 | 1.06061 | | 1.07078 | | 1.12888 | | 1.02905 | NA |  |
| NA |  | NA |  | 1.03571 | | NA |  | NA |  | 1.09482 | | NA | NA |  |
| NA |  | NA |  | 1.03571 | | NA |  | NA |  | 1.09482 | | NA | NA |  |
| NA |  | NA |  | 1.03571 | | NA |  | NA |  | 1.09482 | | NA | NA |  |

| 0.958564 | | 0.958248 | | NA |  | 1.01938 | | 1.02167 | | 0.999912 | | NA | NA |  |
| --- | --- | --- | --- | --- | --- | --- | --- | --- | --- | --- | --- | --- | --- | --- |
| 0.933188 | | 0.914242 | | NA |  | 1.03679 | | 1.03739 | | 1.02004 | | NA | NA |  |
| 1.08458 | | 1.07775 | | NA |  | 1.04148 | | 0.996196 | | 1.06797 | | NA | NA |  |
| 0.914149 | | NA |  | 1.16923 | | 0.986588 | | 0.960241 | | 0.995613 | | 1.02727 | 0.94105 | |
| NA |  | NA |  | 1.89655 | | 1.07782 | | 1.01497 | | 1.05755 | | 1.02046 | 0.95966 | |
| NA |  | NA |  | 1.28571 | | 1.19399 | | 1.05748 | | 1.13754 | | 1.07982 | 0.885714 | |
| NA |  | NA |  |  | 1.3 |  | 1.0676 | NA |  | 1.04578 | | 1.22756 | 0.908257 | |
| 1.06979 | | NA |  | NA |  | 1.01662 | | 0.984188 | | 0.994067 | | NA | NA |  |
| 1.10881 | | 1.07581 | | 1.08113 | | 1.00614 | |  | 1.0101 | 0.999056 | | 1.16299 | 1.06485 | |
| 1.08624 | | 1.12677 | | 1.09375 | | 1.02773 | | 1.10555 | | 1.06092 | | 1.09439 | 0.956306 | |
| 1.03927 | |  | 0.9889 | 1.15385 | | 0.965059 | | 0.976133 | | 0.971525 | | 0.952255 | 0.914706 | |
| 1.03927 | |  | 0.9889 | 1.15385 | | 0.965059 | | 0.976133 | | 0.971525 | | 0.952255 | 0.914706 | |
| 1.04045 | | 1.16397 | | 1.47059 | | 1.04296 | | 1.05598 | | 1.01396 | | 1.03151 | 0.995516 | |
| 0.945991 | | 0.893473 | | NA |  | 1.00228 | | 0.959294 | | 1.02795 | | 0.969867 | 0.679602 | |
| 0.945991 | | 0.893473 | | NA |  | 1.00228 | | 0.959294 | | 1.02795 | | 0.969867 | 0.679602 | |
| NA |  | NA |  | 1.13775 | |  | 1.007 | NA |  | NA |  | 0.996141 | NA |  |
| NA |  | NA |  | 1.04762 | | NA |  | NA |  | NA |  | 0.96438 | NA |  |
| NA |  | NA |  |  | 1.4153 | NA |  | NA |  | NA |  | 0.943375 | 1.05897 | |
| 1.04557 | | 0.988384 | | 1.17742 | | 1.01064 | | 1.03026 | | 1.06003 | | 1.01696 | NA |  |
|  | 1.0039 | 1.05252 | | NA |  | 1.00957 | | 1.04999 | | 1.03805 | | 1.10482 | 0.952756 | |
| 1.05923 | | 1.06228 | | NA |  | 1.03482 | | 0.961538 | | 0.98953 | | 0.811364 | NA |  |
| 0.991227 | | 0.917411 | | NA |  | 0.95041 | | 0.960472 | | 0.952807 | | 0.95564 | NA |  |
| 0.993604 | | 0.983353 | | NA |  | 0.999598 | | 1.00632 | | 0.988677 | | 0.909406 | 0.792552 | |
| 1.05881 | | 0.970463 | | NA |  | 0.988117 | | 0.979791 | | 0.974903 | | 0.957295 | 1.01068 | |
| 1.01211 | | 1.03157 | | NA |  | 0.986525 | | 0.905045 | | 0.974971 | | 0.895442 | NA |  |
| 0.997264 | | 0.941481 | | NA |  | 1.02607 | | 1.03106 | | 0.938762 | | 0.970684 | 0.869318 | |
| 0.942309 | | 0.921595 | | NA |  | 1.03218 | | 1.00193 | | 1.01666 | | 1.02576 | NA |  |
| 1.06277 | | 1.04574 | | NA |  | 1.00992 | | 1.01735 | | 1.03473 | | 1.10826 | 1.02339 | |
| 0.886387 | | 0.940199 | | NA |  | 0.988702 | | 1.04814 | |  | 1.0608 | NA | NA |  |
| 1.10932 | | 0.978435 | |  | 0.825 | 1.04672 | | 0.999949 | | 1.02867 | | 1.00717 | 1.03312 | |
| 0.925975 | | 0.847432 | | NA |  | 0.921592 | | 0.902281 | | 0.930034 | | 0.977827 |  | 0.8107 |
| NA |  | 0.976914 | | NA |  | 0.927931 | | 0.948462 | | 0.973114 | | NA | NA |  |
| NA |  | NA |  | NA |  | 1.00956 | | 1.03805 | | NA |  | NA | NA |  |
| NA |  | NA |  | 0.878049 | | NA |  | NA |  | NA |  | 0.995122 | NA |  |
| NA |  | NA |  | NA |  | NA |  | NA |  | NA |  | 0.942828 | NA |  |
| 1.09798 | | 1.13378 | | 0.737864 | | 1.06349 | | 1.06344 | | 1.04109 | | 1.03902 | 0.839981 | |
| 1.01681 | | 1.10585 | |  | 1.0303 | 0.976264 | | 1.02826 | | 0.989005 | | 1.10581 | 0.904043 | |
| 1.00935 | | 1.03163 | | 0.983716 | | 0.997465 | | 1.04239 | | 1.05416 | | 1.00648 | 1.06418 | |
| NA |  | 1.22776 | | NA |  | NA |  | NA |  | 1.06348 | | 1.10136 |  | 1.1117 |

| 1.0613 | 1.14924 1 | 1.00923 | 1.00953 | 1.01686 | 1.06305 | 0.947312 |
| --- | --- | --- | --- | --- | --- | --- |
| 0.949585 | 0.827726 0.83871 | 0.98085 | 0.954699 | 0.998568 | 0.960096 | 0.841257 |
| 1.11234 | 1.06825 NA | 1.21091 | 1.20553 | 1.22939 | 1.10121 | 1.01131 |
| 1.03946 | 1.09132 NA | 1.19218 | 1.19459 | 1.26982 | 0.930293 | 0.888889 |
| 1.02109 | 1.09908 NA | 1.12171 | 1.12075 | 1.1502 | 1.01551 | 0.879085 |
| 1.06933 | 0.980924 NA | 0.939656 | 1.05486 | 1.01858 | 0.937981 | 1.07035 |
| 0.885972 NA NA 1.05058 1.10986 1.04646 1.14663 1.14643 | | | | | | |
| 1.01028 | 1.08065 NA | 1.13722 | 1.18082 | 1.21048 | 0.99555 | 0.859425 |
| 1.08645 | 1.10653 NA | 1.14713 | 1.21019 | 1.22101 | 1.01292 | 0.951096 |

NA	NA	1.12452 NA	NA	NA	NA	NA NA	NA	1.08553 NA	NA	NA	NA	NA NA	NA	NA	NA	NA	NA	NA	NA

1.24618	1.24831	1.09038	1.08155	1.03702	1.02213	1.21549	1.22158

1.08099	1.06372	1.20922	1.24422	1.14647	1.2045	1.25032	1.11859

0.926226 0.906625 NA	0.99342 0.943962 0.989539 NA	NA

1.00251	1.19232	0.875	1.13361	1.20105	1.15593	1.11068	1.05686

0.899242 0.882726 NA	0.961938 0.924482 0.971181 NA	NA

0.977875 0.940794 NA	1.03583	1.01462	1.01287 NA	NA

1.02185	1.04585 0.965182	1.01609	1.02953	1.02493	1.04192	1.01383

NA	NA	NA	NA	NA	NA	NA	NA

| 1.12925 | 1.14917 | 0.875 | 1.08239 | 1.09572 | 1.10922 | 1.12933 | 1.06094 |
| --- | --- | --- | --- | --- | --- | --- | --- |
| 0.969861 | NA | NA | 1.01002 | 1.00525 | 1.02033 | 0.983296 | 0.83125 |
| 0.996947 | 1.00773 | 0.962963 | 1.06179 | 1.06072 | 1.07185 | 1.12227 | 1.17924 |
| 1.01098 | 1.05257 | 1.09746 | 1.02 | 1.02546 | 1.09212 | 1.03065 | 0.885843 |
| 1.00514 | 0.975833 | 1.01538 | 1.01542 | 1.05895 | 1.07977 | 0.982626 | 0.929746 |
| 0.980319 | 1.00815 | 1.15714 | 1.02698 | 1.01936 | 1.04965 | 0.910173 | 0.927336 |
| 0.954187 | 0.957096 | 1.17857 | 0.980453 | 1.0552 | 1.08978 | 0.935185 | 0.951049 |
| 0.978496 | 1.00064 | 0.959097 | 1.09651 | 1.06238 | 1.11236 | 1.03349 | 0.824608 |
| 1.01537 | 1.01627 | 0.761364 | 1.09134 | 1.03693 | 1.02689 | 1.04471 | NA |
| 1.12282 | 1.11253 | 0.83871 | 1.19734 | 1.25546 | 1.1689 | 0.996602 | 0.957929 |
| 1.00463 | 1.04836 | 0.617647 | 0.999547 | 1.04545 | 1.06567 | 1.10862 | 1.14839 |
| 1.02411 | 1.20413 | 0.952381 | 1.13748 | 1.08839 | 1.13953 | 1.15052 | 1.1558 |
| 1.10997 | 1.00183 | 0.875 | 1.05081 | 1.02963 | 1.00389 | 1.04068 | 0.93007 |
| 1.03953 | 0.993672 | NA | 1.037 | 1.04296 | 1.04887 | 1.06532 | NA |
| 1.15105 | 1.16608 | 1.10427 | 1.10527 | 1.08268 | 1.0952 | 1.17221 | 1.15032 |
| 0.996992 | 1.0181 | NA | 1.013 | 1.01206 | 1.01651 | 1.02874 | 1.04574 |
| 1.04986 | 1.04955 | NA | 1.01307 | 0.949033 | 0.972562 | 1.02082 | 1.02307 |
| 0.936119 | 0.946237 | 1.04762 | 0.999156 | 1.01586 | 1.05579 | 1.00898 | 1.04444 |
| 1.10762 | 1.10333 | 0.680256 | 1.05341 | 1.04786 | 1.06152 | 1.12461 | 0.986721 |

1.1135	1.07177 0.807692	1.0998	1.04203	1.06261 0.972689	1.13924

0.909425	1.17725 0.971429	1.07135	1.06395	1.08985	1.03027 0.869969

1.12547	1.14554 0.728571	1.25992	1.19213	1.18644	1.14	1.27915

1.07191 0.911915	1.40909 0.978482 0.995458 0.991505 0.921525	1.13592

1.17292	1.11698	1.57681	1.02248 0.977896 0.964852	1.119 0.985001

NA	NA	NA	1.01255 0.991345	1.00552 NA	NA

1.02963 0.996436 0.894893 0.987564	0.97989 0.997135	1.04114	1.03966

NA	NA	1.20509	1.02	1.01061 0.992755 NA	NA

1.08216 0.994683	1.05357	1.10934	1.0672	1.09493	1.11587	1.1028

1	0.943066 NA	0.940447 0.965595 0.938019	1.12414	1.15238

0.960327 0.903955 0.909091 0.976253 0.973591 0.966192	1.03663	1.11392

NA	NA	NA	NA	NA	NA	NA	NA

1.01245 0.941291	1.16667	1.05945 0.998279 0.998809	1.04322	1.12411

1.12935	1.04663	1.45371	1.02381	1.06015	1.06961	1.15695	1.19687

0.985172	1.08752	1.2381	1.1924	1.2041	1.15455	1.15218	1.06509

1.08842	1.08201	1.04765	1.05032	1.07204	1.04306	1.06058	1.07628

1.12056	1.13276	1.11846	0.99865 0.981642	1.04942	1.10857	1.11653

1.07964	1.14117	1.21212 0.995052	1.01682	1.02868 0.991763 0.959041

NA	NA	NA	1.03949	1.04208	1.03044 NA	NA

1.07979	1.09491 NA	1.05233 0.995724	1.06552	1.05128 NA

1.01015 0.986364	1.07077	1.03079	1.11604	1.10041 0.972256 NA

1.0683 NA	NA	0.929803	1.00976	1.00532 NA	NA

| 1.02556 | 1.0145 | 1.17857 | 1.0098 | 0.996769 | 1.03525 | 0.992539 | 0.997207 |
| --- | --- | --- | --- | --- | --- | --- | --- |

0.997038	1.01879 NA	0.992076 0.975491	1.01618 0.945909	0.92051

| 1.01387 | 0.994326 | 0.991839 | 0.995139 | 0.999119 | 1.03314 |
| --- | --- | --- | --- | --- | --- |

1.00821 NA

1.06893	1.0815 NA	1.03915	1.07889	1.04608	1.1554 NA

1.0055	1.01514	1.23077	1.01939	1.05186 0.995734	1.0881	1.2674

0.965873 0.999924	0.92437 0.994623	1.03355	1.0272	1.0832 0.991704

1.02954 0.937575 NA	0.993526 0.993376	0.99321 0.964544	1.1145

1.09959 0.963636	0.84 0.953998	1.01272 0.980667 0.935199 NA

0.999566	1.01666 NA	1.02177 0.983188 0.999129 0.963732 0.911346

1.20105	1.17095	1.05396	1.16074	1.17681	1.14718	1.12006	1.14277

1.03566	1.07726 0.887783 0.979398 0.929335	1.00419	1.11513	1.30679

1.04624	1.01419 NA	1.08402	1.09211	1.05681 NA	NA NA	NA	0.867321 NA	NA	1.09878 NA	NA

| 1.09839 | 1.03597 | 0.925926 | 1.06217 | 1.0303 | 1.09511 | 1.05502 | 1.12109 |
| --- | --- | --- | --- | --- | --- | --- | --- |
| 0.985011 | 0.977492 | 1.1875 | 1.03168 | 1.09453 | 1.08299 | 0.93921 | 0.867769 |
| 1.05226 | 1.11183 | 1.45455 | 1.0482 | 0.982114 | 0.998776 | 1.12579 | 1.53431 |

eosinophil erythrobla fetal_intes germinal_cgranulocyt hapatocyt heart..nih_ hematopoi

| 1.03867 | 1.3125 | 1.05913 | 1.07416 | 1.16773 | 1.10944 | 1.144 | 1.07877 |
| --- | --- | --- | --- | --- | --- | --- | --- |

NA	NA	NA	NA	NA	NA	NA	NA

1.16973	1.4972	1.09786	1.20392	1.21209	1.17344	1.1198	1.13476

1.11642	1.0016	1.15874	1.16205	1.11072	1.22832	0.98999	1.05237

1.17769	1.23306	1.0995	1.30046	1.14881	1.18856	1.21707	1.03297

0.921313	1.15096	1.03756 0.918912 0.961433 0.948664	1.0057 0.889162

1.04911	1.26536	1.09919	1.20216	1.12253	1.18918	1.19412	1.08018

1.09749	1.03617 NA	1.12026	1.10059	1.16869	1.05856	1.01263

1.09749	1.03617 NA	1.12026	1.10059	1.16869	1.05856	1.01263

1.27273 NA	1.26146	1.22165	1.37073	1.2279	1.16022	1.42953

0.969276	1.05098	1.00342 0.952567 0.980165 0.959005	1.04389 0.924693

1.037	1.09195 0.956583	0.99581	1.06451 0.990488	1.02714 0.905853

1.00855	1.06646	1.03193	1.05382	1.02454 0.991478	1.02358	1.02671

1.06822	1.01743	1.04947	1.03436	1.0058	1.04342 0.972027	1.09253

NA	NA	1.06746 NA	NA	1.23673 NA	NA NA	NA	1.01856 NA	NA	NA	NA	NA

1.03495 NA	0.991957	1.01724 0.990195 0.962344 0.952411 NA

1.03495 NA	0.991957	1.01724 0.990195 0.962344 0.952411 NA

1.01439	0.90678 0.949167 0.998969	1.02135 0.963135 0.930586	0.93788

1.11757	1.21176	1.12203	1.28488	1.16014	1.21373	1.12999	1.05499

NA	NA	1.00335 NA	NA	NA	NA	NA

1.01812	1.0788 0.999008	1.03214 0.974635 0.938613	1.03167 0.973307

NA	NA	1.07188 NA	NA	NA	NA	NA NA	NA	1.08248 NA	NA	NA	NA	NA

1.06851 NA	1.09165 NA	1.00506 0.980906 NA	NA

1.09255 NA	NA	NA	NA	0.994835 NA	NA

1.09009 NA	NA	NA	NA	1.02634 NA	NA

1.04215 NA	NA	NA	NA	1.02665 NA	NA

1.13687	1.0804	1.13199	1.0984	1.13272	1.22349	1.1093	1.04013

1.13687	1.0804	1.13199	1.0984	1.13272	1.22349	1.1093	1.04013

1.10841	1.05978	1.1456	1.22108	1.03884	1.27254	1.13301	1.00855

1.13017	1.20886	1.07739	1.14645	1.11147	1.33712	1.14126	1.00879

1.16236 NA	1.20981	1.16426	1.25016	1.16516	1.12078 NA

1.03788 NA	1.02697 NA	NA	1.05499 NA	NA NA	NA	NA	NA	NA	NA	NA	NA NA	NA	NA	NA	NA	NA	NA	NA NA	NA	0.997759 NA	NA	NA	NA	NA

1.04796	1.03076 NA	1.05053	1.05852	1.0034 0.967913	1.0463

| 0.953868 | | 0.991019 | | 0.990866 | | 0.905141 | | 0.919356 | 0.960126 | | 1.00059 | | 0.953159 | |
| --- | --- | --- | --- | --- | --- | --- | --- | --- | --- | --- | --- | --- | --- | --- |
| 0.953868 | | 0.991019 | | 0.990866 | | 0.905141 | | 0.919356 | 0.960126 | | 1.00059 | | 0.953159 | |
| 0.953868 | | 0.991019 | | 0.990866 | | 0.905141 | | 0.919356 | 0.960126 | | 1.00059 | | 0.953159 | |
| 0.991727 | | 0.907762 | | NA |  | 1.01191 | | 1.01067 | 1.08472 | | 1.06617 | | 0.933162 | |
| 1.03272 | | 0.938782 | | NA |  | 0.980145 | | 0.993193 | 1.01479 | | 0.986977 | | 0.922654 | |
| 0.999197 | | 0.839895 | | 0.969205 | | 1.01007 | | 1.00641 | 0.985303 | | 0.949698 | | 0.968149 | |
| 1.09434 | |  | 1.03 | 1.17712 | | 1.02642 | | 1.03677 | 1.01534 | | 0.996037 | | 1.04301 | |
| 1.05774 | | 0.98725 | | 1.04921 | | 1.01652 | | 1.03777 | 1.02989 | | 0.973393 | |  | 1.0551 |
| 1.04991 | | 0.986702 | | 1.00458 | | 0.956918 | | 0.979804 | 0.990922 | |  | 1 | 1.00473 | |
| 1.07308 | | 0.916084 | | 1.10633 | | 1.05741 | | 1.02643 | 1.05236 | | 1.00822 | | 1.06128 | |
| 1.08766 | | 0.925884 | | 1.16841 | | 1.03283 | | 1.07093 | 1.08388 | | 1.02471 | | 1.08138 | |
| 1.08071 | | 0.864802 | | 1.06245 | | 1.01732 | | 1.06256 | 0.987277 | | 0.934827 | | 1.06818 | |
| 1.13339 | | 1.03705 | | 1.14235 | | 1.06587 | | 1.11231 | 1.04859 | |  | 1.0145 | 1.04515 | |
| 1.08045 | | 0.961618 | | 1.05775 | | 1.05283 | | 1.05104 | 1.03584 | | 1.01781 | | 1.05437 | |
|  | 1.0678 | 0.936703 | | 1.04905 | | 1.05673 | | 1.03043 |  | 1.0265 | 0.987103 | | 1.00411 | |
| NA |  | NA |  | 1.00634 | | NA |  | NA | NA |  | NA |  | NA |  |
| 1.08651 | | 1.41127 | |  | 1.0954 | 1.12683 | | 1.11571 | 1.22787 | | 1.14427 | | 1.10568 | |
| 1.07876 | | 0.977317 | | 1.09978 | | 1.05944 | | 1.06254 | 1.07644 | | 1.04762 | | 1.07504 | |
| 1.12059 | | 1.01113 | | 1.02885 | | 1.04206 | | 1.09199 | 1.04082 | | 1.02728 | | 1.06318 | |
|  | 1.2066 | 0.740741 | | 1.05055 | | 1.03892 | | 1.20452 | 1.07298 | | 1.01663 | | 1.02112 | |
| 1.03508 | | 0.864516 | | 0.99216 | | 0.997816 | | 1.00168 | 0.987135 | | 0.967049 | | 0.982077 | |
| NA |  | NA |  | 0.999002 | | NA |  | NA | 1.02426 | | NA |  | NA |  |
| NA |  | NA |  | 0.99786 | | NA |  | NA | NA |  | NA |  | NA |  |
| 1.05953 | | 0.887702 | | 0.978084 | | 1.03662 | | 1.04051 | 1.01276 | | 1.04594 | | 1.01634 | |
| 1.12389 | | 1.47619 | | 1.00917 | | 1.14607 | | 1.15476 | 1.06262 | | 0.895833 | | 1.18085 | |
| 1.08451 | | 0.982555 | | 1.08189 | | 1.01942 | | 1.03479 | 1.03764 | |  | 1.0376 | 1.02408 | |
| 1.08133 | | 1.08523 | |  | 1.0317 |  | 1.0547 | 1.05972 | 1.04493 | | 1.05114 | | 1.06981 | |
| 1.06945 | | 1.09633 | | 1.05607 | | 1.01696 | | 1.05372 | 1.04303 | |  | 1.0309 | 1.03169 | |
| 1.09241 | | 0.846448 | | 1.13011 | | 1.03701 | | 1.07506 | 1.08964 | | 1.04781 | | 1.03462 | |
| 1.09241 | | 0.846448 | | 1.13011 | | 1.03701 | | 1.07506 | 1.08964 | | 1.04781 | | 1.03462 | |
| 1.07985 | | 0.891195 | | 0.984614 | | 1.02137 | | 1.06864 | 1.01257 | | 1.07603 | | 1.04654 | |
| 1.09046 | | 0.921677 | | 1.00615 | | 1.06349 | | 1.07299 | 1.05308 | | 1.04147 | |  | 1.0148 |
| 1.08146 | | 1.00837 | | 1.14671 | | 1.06134 | | 1.04402 | NA |  | 1.03045 | | 1.04354 | |
| 1.06509 | | 0.983146 | | 0.97434 | | 0.995803 | | 0.989591 | 0.970872 | | 0.994026 | | 0.963083 | |
| NA |  | NA |  |  | 1.0682 | NA |  | NA | NA |  | NA |  | NA |  |
| 0.997447 | | 1.11567 | | 1.00514 | | 1.00057 | | 0.99679 | 1.01928 | | 1.03705 | | 0.922679 | |
| 1.08149 | | 0.911435 | | 1.04413 | | 1.00197 | | 1.04047 | 1.00926 | | 1.06664 | | 1.06017 | |
| 1.05656 | | 0.916449 | | 0.986121 | | 1.03424 | | 1.04292 | 1.02055 | | 1.02442 | | 1.06638 | |
| 1.24743 | | 0.923077 | | 1.11775 | | 1.17542 | | 1.24968 | 1.17181 | | 1.11172 | | 1.07591 | |

| 1.03607 | | 1.12582 | 1.00028 | | 1.05374 | | 1.02072 | 1.05258 | | 1.03463 | | 1.00249 |
| --- | --- | --- | --- | --- | --- | --- | --- | --- | --- | --- | --- | --- |
|  | 1.0253 | NA | 0.995749 | | 1.00237 | | 0.969937 | 0.996793 | | 1.05533 | | 0.901625 |
| 1.04735 | | 1.11992 |  | 1.033 | 1.06185 | | 1.00655 | 1.03355 | | 1.00844 | | 0.976199 |
|  | 1.0167 | NA | 1.06227 | |  | 1.0902 | 0.971966 | 1.05922 | | 1.03427 | | NA |
| NA |  | NA | 0.998764 | | NA |  | NA | 0.997001 | | 0.97545 | | NA |
| NA |  | NA | 0.998172 | | NA |  | NA | 1.01694 | | NA |  | NA |
| NA |  | NA | 0.977525 | | NA |  | NA | 0.998942 | | 0.969826 | | NA |
| NA |  | NA | 1.07781 | | NA |  | NA |  | 1.0371 | 0.946941 | | NA |
| 1.00689 | | NA | 1.05898 | | NA |  | NA | 0.998748 | | 1.04218 | | NA |
| 0.964014 | | NA | 1.04831 | | NA |  | NA | 0.991785 | | 1.04253 | | NA |
| NA |  | NA | 0.986899 | | NA |  | NA | 0.99336 | | NA |  | NA |
| NA |  | NA | 0.986899 | | NA |  | NA | 0.99336 | | NA |  | NA |
| NA |  | NA | 0.986899 | | NA |  | NA | 0.99336 | | NA |  | NA |
| NA |  | NA | 1.05206 | | NA |  | NA | 0.998479 | | NA |  | NA |
| 0.991889 | | NA | 1.05071 | | NA |  | NA | 1.00754 | | 1.03813 | | NA |
| 0.991824 | | NA | 1.05777 | | NA |  | 1.07119 |  | 1 |  | 1.0475 | NA |
| NA |  | NA | 1.06059 | | NA |  | NA | 1.02564 | | 1.02749 | | NA |
| NA |  | NA | 1.03662 | | NA |  | NA | 0.988115 | | 0.961605 | | NA |
| 0.98796 | | NA |  | 1.0603 | NA |  | 1.07905 | 1.00127 | | 1.02997 | | NA |
| 0.995348 | | NA | 1.06703 | | NA |  | 1.06048 | 1.02771 | | 1.01208 | | NA |
| NA |  | NA | 0.987022 | | NA |  | NA | 0.973578 | | NA |  | NA |
| 1.10128 | | 0.972721 |  | 1.094 |  | 1.04 | 1.07737 |  | 1.0604 | 1.02363 | | 1.05043 |
| 0.959494 | | 1.01365 |  | 1.002 | NA |  | 0.952308 | 1.02639 | | 1.02185 | | 0.985104 |
| 0.959494 | | 1.01365 |  | 1.002 | NA |  | 0.952308 | 1.02639 | | 1.02185 | | 0.985104 |
| 0.985199 | | NA | 1.02306 | | NA |  | 1.05075 | 1.02346 | | 0.962706 | | NA |
| 0.985199 | | NA | 1.02306 | | NA |  | 1.05075 | 1.02346 | | 0.962706 | | NA |
| 0.991346 | | NA | 1.04205 | | NA |  | 0.991023 | 1.01016 | | 1.10814 | | NA |
| 0.992631 | | 0.986867 | 1.02526 | | NA |  | 1.03542 | 1.05712 | |  | 1.1299 | 1.00374 |
| 0.984854 | | NA | 1.01519 | | NA |  | 0.98544 | 0.95991 | | 1.05499 | | NA |
| 1.04848 | | NA | 1.05118 | | NA |  | 1.04716 | 1.04539 | | 1.08667 | | NA |
| 1.04848 | | NA | 1.05118 | | NA |  | 1.04716 | 1.04539 | | 1.08667 | | NA |
| 1.04848 | | NA | 1.05118 | | NA |  | 1.04716 | 1.04539 | | 1.08667 | | NA |
| NA |  | NA | 1.00851 | | NA |  | NA | NA |  | NA |  | NA |
| NA |  | NA | 0.998377 | | NA |  | NA | NA |  | NA |  | NA |
| NA |  | NA | 1.02423 | | NA |  | NA | NA |  | NA |  | NA |
| NA |  | NA |  | 1.0249 | NA |  | NA | NA |  | NA |  | NA |
| 0.96275 | | 0.740586 | 0.980513 | | 0.912924 | | 1.01888 | 1.05604 | | 1.00753 | | 0.830736 |
| 1.05933 | | 1.11251 | NA |  | 1.16792 | | 1.08531 |  | 1.1156 | 1.07666 | | 0.990515 |
| 1.05933 | | 1.11251 | NA |  | 1.16792 | | 1.08531 |  | 1.1156 | 1.07666 | | 0.990515 |

| 1.08035 | 1.22677 | 1.26246 | | 1.24358 | 1.07049 | | 1.22661 | | 1.19764 | 1.08299 |
| --- | --- | --- | --- | --- | --- | --- | --- | --- | --- | --- |
| NA | NA | NA |  | 1.25611 | NA |  | 1.14817 | | NA | NA |
| 1.04388 | 1.13559 | 1.11954 | | 1.12482 | 0.993064 | | 1.08844 | | 0.979723 | 1.11496 |
| NA | 0.926302 | NA |  | 1.03335 | NA |  |  | 1.0564 | 1.01766 | NA |
| 1.06625 | 1.13793 | 1.05586 | | 1.07253 | 1.01382 | | 0.987892 | | 0.991359 | 1.01989 |
| 1.04261 | NA | NA |  | NA | NA |  | 1.04591 | | NA | NA |
| NA | NA | 1.01475 | | NA | NA |  | 0.987564 | | NA | NA |
| NA | NA | 1.11551 | | NA | NA |  | 1.02424 | | NA | NA |
| NA | NA | 1.01249 | | 0.970854 | NA |  | 1.05145 | | 0.938314 | NA |
| 1.02892 | NA | 1.05671 | | 1.14475 | NA |  | 1.07603 | | 0.965278 | NA |
| 0.907072 | NA | 1.01745 | | 1.03232 | 0.983245 | | 1.05739 | | 0.983763 | 0.978898 |
| NA | NA | 1.04684 | | NA | NA |  | NA |  | NA | NA |
| NA | NA | 1.09978 | | NA | NA |  | NA |  | NA | NA |
| NA | NA | 1.07065 | | NA | NA |  | NA |  | NA | NA |
| NA | NA | 1.08441 | | NA | NA |  | NA |  | NA | NA |
| NA | NA | 1.06364 | | NA | NA |  | NA |  | NA | NA |
| NA | NA | 1.06362 | | NA | NA |  | NA |  | NA | NA |
| NA | NA |  | 1.0619 | NA | NA |  | NA |  | NA | NA |
| NA | NA | 1.00506 | | NA | NA |  | NA |  | NA | NA |
| 1.05944 | NA | 1.04084 | | NA | 1.08891 | | NA |  | NA | NA |
| NA | NA | 1.10958 | | NA | NA |  | NA |  | NA | NA |
| NA | NA | 1.01482 | | NA | NA |  | NA |  | NA | NA |
| NA | NA | 1.00178 | | NA | NA |  | NA |  | NA | NA |
| NA | NA | 0.959274 | | NA | NA |  | NA |  | NA | NA |
| NA | NA | 1.07339 | | NA | NA |  | NA |  | NA | NA |
| 0.993702 | NA | 1.08707 | | NA |  | 1.0156 | NA |  | NA | NA |
| NA | NA |  | 1.0898 | NA | NA |  | NA |  | NA | NA |
| NA | NA | 1.10564 | | NA | NA |  | NA |  | NA | NA |
| NA | NA | 1.06231 | | NA | NA |  | NA |  | NA | NA |
| NA | NA | 1.06121 | | NA | NA |  | NA |  | NA | NA |
| 1.06717 | NA |  | 1.0111 | NA | 0.961324 | | 1.01083 | | 1.01825 | NA |
| NA | NA | 1.03521 | | NA | NA |  | NA |  | NA | NA |
| NA | NA | 0.995165 | | NA | NA |  | NA |  | NA | NA |
| NA | NA | 1.01551 | | NA | NA |  | NA |  | NA | NA |
| NA | NA | 1.04291 | | NA | NA |  | NA |  | NA | NA |
| 1.07304 | NA | 1.13973 | | NA | NA |  | NA |  | NA | NA |
| NA | NA | 1.07987 | | NA | NA |  | NA |  | NA | NA |
| 1.08069 | NA | 1.18032 | | NA | NA |  | NA |  | NA | NA |
| NA | NA | 1.02339 | | NA | NA |  | NA |  | NA | NA |

| NA |  | NA |  | 1.01972 | NA |  | NA |  | NA | NA |  | NA |  |
| --- | --- | --- | --- | --- | --- | --- | --- | --- | --- | --- | --- | --- | --- |
|  | 1.0245 | NA |  | 1.04518 | NA |  | 1.03131 | | NA | NA |  | 1.04741 | |
| NA |  | NA |  | 1.05921 | NA |  | NA |  | NA | NA |  | NA |  |
| NA |  | NA |  | 1.07014 | NA |  | NA |  | NA | NA |  | NA |  |
| NA |  | NA |  | 1.12674 | NA |  | NA |  | NA | NA |  | NA |  |
| 1.09987 | | NA |  | 1.12412 | NA |  | 1.12614 | | NA | NA |  | NA |  |
| NA |  | NA |  | 1.07923 | NA |  | NA |  | NA | NA |  | NA |  |
| NA |  | NA |  | 1.04763 | NA |  | NA |  | NA | NA |  | NA |  |
| NA |  | NA |  | 1.02418 | NA |  | NA |  | NA | NA |  | NA |  |
| 1.06517 | | NA |  | 1.10142 | NA |  | NA |  | NA | NA |  | NA |  |
| 0.997846 | | 0.835955 | | 1.01548 | 0.96544 | | 0.949205 | | 0.971165 | 0.991517 | | 0.908072 | |
| 1.23057 | | NA |  | 1.24065 | 1.00768 | | 1.00579 | | 1.21107 | 1.25331 | | NA |  |
| 0.983518 | | NA |  | NA | 0.93659 | | 0.964141 | | NA | NA |  | NA |  |
| 1.03539 | | NA |  | 0.935256 | 1.09654 | |  | 1.0468 | 0.986983 | 0.978382 | | 0.956841 | |
|  | 1.1408 |  | 1.0922 | 1.03863 | 1.04626 | | 1.07491 | | 1.02333 | 0.934783 | | 1.01222 | |
| 1.10077 | | NA |  | 0.999788 | 1.06772 | | 0.995361 | | 1.07309 | 1.03341 | | 1.05735 | |
| NA |  | NA |  | NA | 1.18734 | | NA |  | 1.17125 | NA |  | NA |  |
| 1.00363 | | NA |  | 0.939174 | 0.961446 | | 0.974961 | | 1.03333 | 0.998079 | | NA |  |
| 1.02206 | | NA |  | 0.925029 | 0.979556 | | 1.07259 | | 1.04635 | 0.926376 | |  | 1.0143 |
| 1.10523 | | NA |  | 1.03193 | 1.05004 | | 1.10969 | | 1.12728 |  | 1.0261 | NA |  |
| 1.03818 | | NA |  | 1.00838 | 0.988027 | | 0.961549 | | NA | 1.01781 | | 1.02432 | |
| 1.06718 | |  | 1.0378 | 0.982479 | 1.01649 | | 1.00107 | | 1.07989 | 1.02601 | | 0.949884 | |
| 1.06125 | | 0.974803 | | 1.05239 | 1.03665 | | 1.02755 | | 1.00772 | 0.995881 | | 0.95206 | |
| 1.02165 | | NA |  | 1.02101 | 1.07315 | | 1.04998 | | NA | 1.00085 | | NA |  |
| NA |  | NA |  | 0.897153 | NA |  |  | 1.0554 | 0.977031 | NA |  | NA |  |
| 1.03896 | | NA |  | 1.05144 | 1.01001 | | 1.08245 | | 1.02006 | 1.06316 | | 1.10633 | |
| NA |  | 1.05946 | | 0.991115 | 1.11472 | | 1.01935 | | 0.997246 | NA |  | 1.02427 | |
| 0.95837 | |  | 1.1875 | 1.01578 | 1.04993 | | 0.975657 | | 1.03887 | NA |  | 1.16993 | |
| 0.95837 | |  | 1.1875 | 1.01578 | 1.04993 | | 0.975657 | | 1.03887 | NA |  | 1.16993 | |
| NA |  | 1.29826 | | NA | 1.02802 | | 1.00421 | | NA | NA |  | 1.13477 | |
| 1.03442 | | 0.911058 | | 1.03916 |  | 1.0015 | 1.01026 | | 0.97914 | NA |  | 1.04332 | |
| NA |  | NA |  | 0.919649 | 1.03605 | | 1.06855 | | NA | NA |  | NA |  |
| NA |  | NA |  | 0.919649 | 1.03605 | | 1.06855 | | NA | NA |  | NA |  |
| NA |  | NA |  | 0.919649 | 1.03605 | | 1.06855 | | NA | NA |  | NA |  |
| 0.977022 | | 0.977251 | | 0.980339 | 0.97213 | | 0.976743 | | 0.936945 | 0.92932 | | 1.01638 | |
| 0.971377 | | 0.979453 | | 1.03721 | 1.02611 | | 1.01488 | | 1.00762 |  | 1.0296 | 0.986695 | |
| NA |  | NA |  | 0.948669 |  | 1.1023 | NA |  | NA | NA |  | NA |  |
| NA |  | NA |  | 1.02394 | NA |  | NA |  | NA | NA |  | NA |  |
| 1.08291 | | NA |  | 1.01437 | NA |  | 1.07042 | | 0.961544 | NA |  | NA |  |

| NA |  | NA |  | 0.971278 | | NA |  | NA |  | NA |  | NA |  | NA |  |
| --- | --- | --- | --- | --- | --- | --- | --- | --- | --- | --- | --- | --- | --- | --- | --- |
| NA |  | NA |  | 1.07584 | | NA |  | NA |  | NA |  | NA |  | NA |  |
| NA |  | NA |  | 1.03415 | | NA |  | NA |  | NA |  | NA |  | NA |  |
| NA |  | NA |  | 0.955162 | | NA |  | NA |  | NA |  | NA |  | NA |  |
| 0.947686 | | 1.29488 | | 0.977809 | | 1.02099 | | 0.945329 | | 0.946862 | | 0.994477 | | 1.01645 | |
| 1.00455 | | 1.09494 | | 0.998022 | | 0.996183 | | 1.00241 | |  | 1.0097 | 0.964917 | | 1.06086 | |
|  | 1.0136 | 0.90411 | | NA |  | 0.957938 | | 0.982899 | | 0.977199 | | 0.937358 | | 1.02661 | |
|  | 1.0699 | 1.09408 | | 0.997282 | | 1.05602 | | 0.992038 | | 0.990718 | | 0.992758 | | 1.03219 | |
| 0.927042 | | 0.940828 | | NA |  | 0.97612 | | 0.982268 | | 0.961982 | | 1.01059 | | 0.948161 | |
| 0.997796 | | 0.98248 | | 1.05021 | |  | 1.0073 | 1.00458 | | 0.955256 | | 1.01241 | | 0.973075 | |
| 1.06447 | | 1.21734 | | 1.02493 | | 1.19084 | | 1.06297 | | 1.13237 | | 1.04644 | | 1.00326 | |
| 1.10929 | |  | 1.104 | 1.35467 | | 1.01807 | | 1.12436 | | 1.23189 | | 1.08951 | | 1.02463 | |
| 1.21777 | | 1.17072 | | 1.48525 | |  | 1.0415 | 1.22036 | | 1.27184 | | 1.19145 | | 1.16968 | |
| 0.998139 | | NA |  | 1.02044 | | 0.89506 | | 1.01335 | | 0.979648 | | 0.971994 | | 0.993573 | |
| NA |  | NA |  |  | 1.0441 | NA |  | NA |  | NA |  | NA |  | NA |  |
| NA |  | NA |  | 1.07027 | | NA |  | NA |  | NA |  | NA |  | NA |  |
| 1.06246 | | 1.23428 | |  | 1.0326 | 1.09987 | | 1.11881 | | 1.16068 | | 1.07912 | |  | 1.0072 |
| NA |  | NA |  | 1.03978 | | NA |  | NA |  | NA |  | NA |  | NA |  |
| 0.939667 | | 1.25661 | | 0.998673 | | 0.936626 | | 0.965431 | | 0.954759 | | 1.06788 | | 0.864013 | |
| NA |  | NA |  | 1.00815 | | NA |  | NA |  | NA |  | NA |  | NA |  |
| NA |  | NA |  | 1.00815 | | NA |  | NA |  | NA |  | NA |  | NA |  |
| 1.06494 | | 1.24717 | |  | 1.2022 | 1.12622 | | 1.12223 | | 1.16421 | | 1.20226 | | 1.10611 | |
| 1.07863 | | 1.23782 | |  | 1.0319 | 1.24518 | | 1.10453 | | 1.16126 | | 1.08323 | | 1.05393 | |
| 1.07863 | | 1.23782 | |  | 1.0319 | 1.24518 | | 1.10453 | | 1.16126 | | 1.08323 | | 1.05393 | |
| 1.07863 | | 1.23782 | |  | 1.0319 | 1.24518 | | 1.10453 | | 1.16126 | | 1.08323 | | 1.05393 | |
| 1.07863 | | 1.23782 | |  | 1.0319 | 1.24518 | | 1.10453 | | 1.16126 | | 1.08323 | | 1.05393 | |
| 1.07536 | | 1.19929 | | 1.14613 | | 1.08194 | | 1.01014 | | 1.00555 | | 1.06918 | | 1.07787 | |
| 1.07536 | | 1.19929 | | 1.14613 | | 1.08194 | | 1.01014 | | 1.00555 | | 1.06918 | | 1.07787 | |
| 1.07536 | | 1.19929 | | 1.14613 | | 1.08194 | | 1.01014 | | 1.00555 | | 1.06918 | | 1.07787 | |
| 1.07536 | | 1.19929 | | 1.14613 | | 1.08194 | | 1.01014 | | 1.00555 | | 1.06918 | | 1.07787 | |
| 1.15548 | | 0.923351 | | 1.19267 | | 1.10406 | | 1.13287 | | 1.21044 | | 1.09514 | | 1.06295 | |
| 1.15548 | | 0.923351 | | 1.19267 | | 1.10406 | | 1.13287 | | 1.21044 | | 1.09514 | | 1.06295 | |
| 1.09313 | | 0.916667 | | 1.05014 | | 1.06925 | |  | 1.0236 | 1.15162 | | 1.04691 | | 0.975275 | |
| 1.09313 | | 0.916667 | | 1.05014 | | 1.06925 | |  | 1.0236 | 1.15162 | | 1.04691 | | 0.975275 | |
| 1.13856 | | 1.13846 | | 1.06825 | | 1.24874 | | 1.12709 | | 1.29206 | |  | 1.1185 | 1.05892 | |
| 0.918474 | | 1.18363 | | 1.01024 | | 0.932333 | | 0.958156 | | 0.992698 | | 1.00206 | | 0.875937 | |
| 0.918474 | | 1.18363 | | 1.01024 | | 0.932333 | | 0.958156 | | 0.992698 | | 1.00206 | | 0.875937 | |
| 0.947357 | | 1.26872 | | 0.960768 | | 0.962249 | | 0.982751 | | 0.950653 | | 0.992263 | | 0.954643 | |
| 0.947357 | | 1.26872 | | 0.960768 | | 0.962249 | | 0.982751 | | 0.950653 | | 0.992263 | | 0.954643 | |

| 0.959176 | | 1.20182 | 0.986659 | | 0.98875 | | 0.983714 | | 0.957196 | | 1.01476 | | 0.965683 | |
| --- | --- | --- | --- | --- | --- | --- | --- | --- | --- | --- | --- | --- | --- | --- |
| 0.951983 | | 1.25632 | 1.01223 | | 0.933149 | | 0.980239 | | 0.990258 | | 0.990279 | | 0.914933 | |
| 1.05502 | | 1.05091 | 0.989594 | | 0.977447 | | 0.994308 | | 0.977263 | | 0.995912 | | 0.873561 | |
| 0.993715 | | 1.03753 | NA |  | 0.903017 | | 1.05021 | |  | 1.0205 | 1.00879 | | 0.950049 | |
| 1.01409 | | 1.05512 | 0.972716 | | 1.05447 | | 1.00413 | | 0.996174 | | 0.986643 | | 1.00738 | |
| 1.04452 | | NA | 1.07742 | | 0.967607 | | 1.01923 | | NA |  | 1.03886 | | NA |  |
| 1.02568 | | 0.95082 | 1.00771 | | 1.04379 | | 0.972979 | | 0.996264 | | 0.993477 | | 0.955556 | |
| 1.02537 | | 0.928814 | 1.04576 | | 1.01111 | | 1.03495 | | 1.03944 | | 1.00099 | | 1.01302 | |
| 1.03141 | | 0.958991 | 1.04194 | | 1.00283 | | 1.01948 | | 1.03102 | | 0.99846 | | 1.04423 | |
| NA |  | NA | NA |  | 0.96083 | | NA |  |  | 1.0171 | 1.02135 | | NA |  |
| 1.01536 | | 0.864706 | 1.05771 | | 1.02272 | | 0.996717 | | 1.04144 | |  | 1.0062 | 0.961395 | |
| 1.03449 | | 0.94332 |  | 1.0057 | 0.957156 | | 0.981057 | | 1.03089 | | 1.03713 | | 1.04491 | |
| NA |  | NA | NA |  | 0.961082 | | NA |  | NA |  | NA |  | NA |  |
| 1.03454 | | 0.94386 | NA |  | 0.995147 | | 1.11862 | | 0.962098 | |  | 1.0168 | 1.05285 | |
| NA |  | NA | NA |  |  | 1.058 | NA |  | NA |  | NA |  | NA |  |
| NA |  | NA | NA |  | 1.00062 | | NA |  | NA |  | NA |  | NA |  |
| NA |  | NA | NA |  | NA |  | NA |  | NA |  | NA |  | NA |  |
|  | 1.0342 | NA | NA |  | 0.949786 | | 0.961991 | | 0.981451 | | 1.04288 | | NA |  |
| NA |  | NA | NA |  | NA |  | NA |  | NA |  | NA |  | NA |  |
| NA |  | NA | NA |  | NA |  | NA |  | NA |  | NA |  | NA |  |
| NA |  | NA | NA |  | 0.970154 | | NA |  | NA |  | NA |  | NA |  |
| NA |  | NA | NA |  | NA |  | NA |  | NA |  | NA |  | NA |  |
| NA |  | NA | NA |  | NA |  | NA |  | NA |  | 1.03013 | | NA |  |
| 1.05434 | | NA | NA |  | 0.926565 | | 1.02244 | | NA |  | NA |  | NA |  |
|  | 1.0563 | NA | NA |  | 0.980181 | | 0.995559 | | NA |  | 1.04798 | | 1.06871 | |
| NA |  | NA | 1.01993 | | NA |  | NA |  | NA |  | NA |  | NA |  |
| NA |  | NA | 1.04274 | | NA |  | NA |  | NA |  | NA |  | NA |  |
| NA |  | NA | 0.989584 | | NA |  | NA |  | NA |  | NA |  | NA |  |
| 1.09668 | | 1.15724 | 1.07533 | | 1.21976 | | 1.10619 | | 1.25664 | | 1.06697 | | 1.06825 | |
| 1.05009 | | 1.02721 | 0.988025 | |  | 1.0972 | 1.01152 | | 0.998754 | |  | 1.0723 |  | 1.0581 |
| 1.05009 | | 1.02721 | 0.988025 | |  | 1.0972 | 1.01152 | | 0.998754 | |  | 1.0723 |  | 1.0581 |
| NA |  | NA | 1.28652 | | NA |  | NA |  | 1.04685 | | NA |  | NA |  |
| NA |  | NA |  | 1.0132 | 1.09747 | | NA |  | 1.03217 | | 1.00627 | | NA |  |
| NA |  | NA | 0.976891 | | 0.945452 | | NA |  | 1.00414 | | 0.907419 | | NA |  |
| NA |  | NA | 0.985833 | | 1.02905 | |  | 1.0383 | 1.05606 | | NA |  | NA |  |
| 1.04793 | | 0.99472 | 1.00789 | | 1.08456 | | 1.05859 | | 1.02779 | | 0.951075 | | 0.999297 | |
| 0.97148 | | NA | 1.01844 | | 1.03163 | | NA |  | 1.05568 | | 1.02724 | | NA |  |
| NA |  | NA | 1.00055 | | NA |  | NA |  | 1.03207 | | NA |  | NA |  |
| NA |  | NA | 1.00593 | | 1.02592 | | NA |  | 1.02318 | | 1.02093 | | NA |  |

| 0.932987 | | NA |  | 1.02037 | | NA |  | 0.96718 | | 1.04799 | | 0.978127 | | NA |  |
| --- | --- | --- | --- | --- | --- | --- | --- | --- | --- | --- | --- | --- | --- | --- | --- |
| NA |  | NA |  | 0.977843 | | NA |  | NA |  | 0.925015 | | 0.914956 | | NA |  |
| NA |  | NA |  |  | 1.0121 | NA |  | NA |  | 0.986945 | | 0.980941 | | NA |  |
| NA |  | NA |  | 1.03681 | | NA |  | NA |  |  | 0.9851 | 1.04706 | | NA |  |
| NA |  | NA |  | 0.986831 | | 1.03774 | | NA |  | 0.998501 | | 1.02779 | | NA |  |
| 1.01715 | | 1.10392 | | 0.954057 | | 1.03184 | | 0.990363 | | 0.991532 | | 0.950973 | | 1.04322 | |
| NA |  | NA |  | 1.07469 | | NA |  | NA |  | 1.00534 | | NA |  | NA |  |
| NA |  | NA |  | 1.07469 | | NA |  | NA |  | 1.00534 | | NA |  | NA |  |
| NA |  | NA |  | 1.07469 | | NA |  | NA |  | 1.00534 | | NA |  | NA |  |
| NA |  | NA |  | NA |  | NA |  | NA |  | NA |  | NA |  | NA |  |
| 1.00871 | | 1.48649 | | 1.05541 | | 1.01426 | | 0.901662 | | 0.977291 | | 0.91073 | | 0.857143 | |
|  | 1.0307 | NA |  |  | 1.0403 | 1.04913 | | NA |  |  | 1.0293 |  | 1.0953 | 0.954941 | |
|  | 1.0922 | 1.09737 | | 1.06572 | | 1.04733 | | 1.10368 | | 1.03408 | | 1.08994 | |  | 1.0679 |
| NA |  | NA |  | NA |  | NA |  | NA |  | NA |  | NA |  | NA |  |
| NA |  | 1.15254 | | NA |  | 1.04299 | | NA |  | 1.01755 | | 1.00487 | | 1.09329 | |
| 1.05135 | | NA |  | 0.994447 | | 1.04585 | | 1.02968 | | 1.02728 | | 1.04841 | | 1.02802 | |
| 1.02192 | | NA |  | 1.02887 | | 1.04257 | | 1.02078 | | 1.07848 | | 1.05135 | | 1.05214 | |
| NA |  | 1.10417 | | NA |  | 1.03756 | | NA |  | 0.989846 | | 0.964336 | | 0.934939 | |
| NA |  | NA |  | NA |  | NA |  | NA |  | NA |  | NA |  | NA |  |
| NA |  | 0.849343 | | NA |  | 0.995032 | | 0.972517 | | 0.990667 | | 1.01024 | | 0.951168 | |
| 1.00449 | | 1.04805 | | 0.975369 | |  | 1.0144 | 1.00757 | | 1.00293 | | 0.941905 | | 1.03732 | |
| 0.926361 | | NA |  |  | 1.0486 | 0.939758 | |  | 1.007 | 1.01743 | | 1.01171 | | 1.02365 | |
| 0.912213 | | NA |  | 1.00261 | | 0.99413 | | 0.984073 | | 1.00554 | | 0.982285 | | 0.971333 | |
| 1.14204 | | 1.05424 | | 1.22185 | |  | 1.1042 | 1.19715 | | 1.18115 | | 1.03863 | | 1.10126 | |
| 1.02208 | |  | 1 | 1.00533 | | 1.04868 | | 1.05226 | | 0.982963 | | 1.00487 | | 1.16516 | |
| 0.999135 | | NA |  | 0.974465 | | 0.985982 | | 0.926969 | | 1.01416 | | 0.991825 | | 0.975744 | |
| 1.05635 | | 0.917226 | | NA |  | 1.02732 | | 1.10812 | | 0.959456 | | 1.04137 | | 1.06245 | |
| 1.05394 | | 0.942029 | | 0.959602 | | 1.02274 | | 1.05536 | | 0.945903 | | 1.02796 | | 0.960424 | |
|  | 1.0462 | 0.977778 | | 0.935693 | | 0.979307 | | 0.951083 | | 0.990712 | | 1.01537 | | 0.925611 | |
| 1.05709 | | 0.969657 | | 0.991929 | | 0.970865 | | 1.07244 | | 1.04677 | | 1.02099 | | 0.980657 | |
| 1.06087 | |  | 0.75 | 1.04381 | | 0.917847 | | 0.86747 | | 1.05811 | | 1.02809 | | 1.11905 | |
| 1.05121 | | 1.27586 | | 1.04149 | | 1.03111 | | 1.12447 | | 1.05319 | | 1.00461 | | 1.00212 | |
| 1.03907 | | 0.732143 | | 1.07165 | | 0.871224 | | 0.91844 | | 0.952565 | | 1.01548 | | 0.896797 | |
| 1.05867 | | 0.932624 | | 1.01756 | | 0.935539 | | 0.979045 | | 0.938814 | | 1.01916 | |  | 1.0653 |
| 0.926221 | | NA |  | 0.959762 | | 0.904011 | | 1.07986 | | 1.06618 | | 1.11867 | | 0.902685 | |
| 0.946528 | | NA |  | 1.04635 | |  | 1 | NA |  | 1.07296 | | 1.03417 | | NA |  |
| NA |  | NA |  | NA |  | NA |  | NA |  | 0.977746 | | NA |  | NA |  |
| NA |  | NA |  | NA |  | NA |  | NA |  | 0.977746 | | NA |  | NA |  |
| NA |  | NA |  | NA |  | NA |  | NA |  | 0.977746 | | NA |  | NA |  |

| 1.01109 | | 0.980522 | | NA |  | 1.02948 | | 0.970335 | | NA |  | NA |  | 0.930106 | |
| --- | --- | --- | --- | --- | --- | --- | --- | --- | --- | --- | --- | --- | --- | --- | --- |
| 1.01099 | | 0.954732 | | NA |  | 1.01028 | | 0.986784 | | NA |  | NA |  | 0.903379 | |
| 1.13311 | | 1.02265 | | NA |  | 0.985465 | | 1.06393 | | NA |  | NA |  | 1.05199 | |
| 0.970523 | | NA |  | 0.963754 | | 1.02572 | | 1.02713 | | 1.01961 | | 0.995706 | | NA |  |
| NA |  | NA |  | 1.00009 | |  | 1.0146 | NA |  | 1.00119 | | 1.00225 | | NA |  |
| NA |  | NA |  | 0.983614 | | NA |  | NA |  | 1.02667 | | 0.930646 | | NA |  |
| NA |  | NA |  | 1.05173 | | NA |  | NA |  | 1.02016 | | 1.03672 | | NA |  |
| 1.09375 | | NA |  | 1.00854 | | 1.05979 | | 1.14969 | | 1.03275 | | 1.05649 | | NA |  |
| 1.07833 | | 1.21479 | | 0.963663 | | 1.05321 | | 1.11785 | | 0.965672 | | 1.03067 | | 1.10651 | |
| 1.04183 | |  | 1.1717 |  | 1.0606 | 1.09224 | | 1.06284 | | 1.00991 | | 0.997087 | | 1.10458 | |
| 0.991144 | | 0.977941 | | 0.975772 | | 1.04965 | | 1.04365 | | 0.995312 | | 0.988055 | | 1.02058 | |
| 0.991144 | | 0.977941 | | 0.975772 | | 1.04965 | | 1.04365 | | 0.995312 | | 0.988055 | | 1.02058 | |
| 0.996497 | | 1.09126 | | 1.03033 | | 1.04896 | | 1.02703 | | 1.06234 | | 1.07835 | |  | 1.115 |
| 0.955799 | | NA |  | 1.02016 | | 1.00883 | | 0.991175 | | 1.03456 | | 0.976602 | | NA |  |
| 0.955799 | | NA |  | 1.02016 | | 1.00883 | | 0.991175 | | 1.03456 | | 0.976602 | | NA |  |
| 0.978157 | | NA |  | 1.00327 | | NA |  | 0.953554 | | 1.00415 | | 0.968644 | | NA |  |
| 0.982826 | | NA |  | 0.931901 | | NA |  | NA |  | 0.959231 | | NA |  | NA |  |
| 0.927568 | | NA |  | 0.934907 | | NA |  | NA |  | 0.989183 | | 1.00082 | | NA |  |
| NA |  | 0.993951 | | NA |  | NA |  | 1.07273 | | NA |  | NA |  | 1.07805 | |
| 1.07932 | | 0.923118 | | 1.08129 | | 1.03321 | | 1.08765 | | 1.08091 | | 1.05871 | | 1.03272 | |
| 1.08451 | | 0.966292 | | 1.04225 | | 1.10563 | | 1.04772 | | 0.973394 | | 0.941312 | | 1.06364 | |
|  | 1.0197 | NA |  | 0.934205 | |  | 1.0132 | 1.07375 | | NA |  | 0.915865 | | 1.03774 | |
| 1.05711 | | 0.87164 | | 1.00884 | |  | 1.005 | 1.01516 | | 1.01127 | | 0.989204 | |  | 1.003 |
| 1.09013 | | 0.939808 | | 1.02462 | | 1.03672 | | 1.04388 | | 0.990477 | | 0.96666 | | 1.06351 | |
| 1.07706 | | 0.813665 | | 0.976809 | | 1.01885 | | 1.05323 | | 0.96476 | | 0.962451 | | 1.05394 | |
| 1.01076 | |  | 1.0183 | 0.953058 | | 1.03695 | | 1.00699 | | 1.02351 | | 1.02361 | | 0.996445 | |
| 1.00112 | | NA |  | 0.970698 | | 0.961385 | | 1.03275 | | 0.990814 | | 1.01467 | | 1.00806 | |
| 1.06943 | | 0.899637 | | 0.96772 | |  | 1.0179 |  | 1.0597 | 1.01934 | |  | 1.0135 | 1.06339 | |
| 0.879902 | | NA |  | 1.03239 | | 0.996119 | | 0.911765 | | 1.03953 | | 0.97197 | | 0.846721 | |
| 0.996806 | | 1.00208 | | 1.03259 | | 0.997508 | | 1.01731 | | 0.991876 | | 1.06071 | | 0.993947 | |
| 0.889346 | | 1.13255 | |  | 1.0132 | 0.916869 | | 0.913135 | | 0.934737 | | 0.968276 | | 0.837525 | |
| NA |  | NA |  | 0.920222 | | 0.986463 | | NA |  | 0.997809 | | NA |  | 0.886428 | |
| 0.98691 | | NA |  | 0.992752 | | NA |  | NA |  | 0.977946 | | NA |  | NA |  |
| NA |  | NA |  | 0.975606 | | NA |  | NA |  | 0.989751 | | 1.03912 | | NA |  |
| NA |  | NA |  | 0.952229 | | NA |  | NA |  | 0.933591 | | 0.983634 | | NA |  |
| NA |  | 0.855847 | | NA |  | 1.07484 | | 1.00954 | |  | 1.0176 | 1.01072 | | 1.05238 | |
| 1.02579 | | 0.95076 | | 1.09063 | | 0.968902 | |  | 1.0218 | 1.06979 | | 1.08142 | | 1.01263 | |
|  | 1.0264 | 0.968604 | | NA |  | 1.01257 | | 1.01824 | | 1.04046 | | 1.06354 | | 0.975825 | |
| NA |  | 1.11176 | | NA |  | 1.08699 | | NA |  | NA |  | NA |  | NA |  |

| 1.04226 | | 0.96715 | | 1.07513 | | 1.03676 | | 1.02431 | | 1.08813 | | 1.03494 | | 1.02299 | |
| --- | --- | --- | --- | --- | --- | --- | --- | --- | --- | --- | --- | --- | --- | --- | --- |
| 0.890031 | | 0.956596 | | 0.935099 | | 0.93004 | | 0.906341 | | 0.979789 | | 1.05161 | | 0.868375 | |
| 1.11042 | | 1.10811 | | 1.05541 | | 1.15915 | | 1.10591 | | 1.15753 | | 1.07818 | | 1.01367 | |
| 1.08843 | | 0.99711 | | 1.02374 | |  | 1.1664 | 1.04917 | | 1.14177 | | 1.08011 | | 0.937761 | |
| 1.03443 | | 1.00775 | | 0.980245 | | 1.11874 | | 0.999279 | | 1.06536 | | 1.02238 | | 0.997547 | |
| 0.999894 | | 1.21739 | | 0.952571 | | 0.968673 | | 1.02322 | | 0.991554 | | 0.887591 | | 1.01653 | |
| NA |  | NA |  | NA |  | 0.974388 | | 0.947094 | |  | 1.0086 | 1.06037 | | NA |  |
| 1.03694 | | 1.06107 | | 1.00399 | | 1.15089 | |  | 0.9912 | 1.13661 | | 1.03904 | | 0.872727 | |
| 1.10537 | | 1.05929 | | 1.02995 | | 1.17508 | | 1.05601 | | 1.10903 | |  | 1.0465 | 0.997716 | |
| NA |  | NA |  |  | 1.0766 | NA |  | NA |  | NA |  | NA |  | NA |  |
| NA |  | NA |  |  | 1.0556 | NA |  | NA |  | NA |  | NA |  | NA |  |
| NA |  | NA |  | NA |  | NA |  | NA |  | NA |  | NA |  | NA |  |
|  | 1.1499 | 1.02177 | | 1.20288 | | 1.13045 | | 1.19639 | | 1.14411 | | 1.10087 | | 1.15518 | |
| 1.14124 | | 1.10794 | |  | 1.1034 | 1.10109 | | 1.14079 | | 1.18029 | | 1.11307 | | 1.10601 | |
| 0.870116 | | NA |  | NA |  | 0.99202 | | 0.961224 | | 1.04108 | | 1.00584 | | 0.861281 | |
| 1.07408 | | 0.966972 | | 1.17913 | | 1.02486 | |  | 1.1487 | 1.14992 | | 1.04801 | | 0.946329 | |
| 0.862862 | | NA |  | NA |  | 0.938035 | | 0.962666 | | 1.01784 | | 1.01183 | | 0.825074 | |
| NA |  | 0.926138 | | NA |  | 1.06484 | | 1.01568 | | 1.09035 | | NA |  | 0.927671 | |
| 1.01084 | | 1.03692 | | 1.01431 | | 1.01877 | | 1.00448 | | 1.02434 | | 0.990271 | | 1.01994 | |
| NA |  | NA |  | 1.06919 | | NA |  | NA |  | NA |  | NA |  | NA |  |
| 1.18234 | | 0.91029 | | 1.17772 | | 1.10969 | | 1.10853 | | 1.21595 | | 1.14154 | | 1.10805 | |
| 0.963719 | | NA |  |  | 1.073 |  | 1.0593 | 0.991834 | | 1.05063 | |  | 1.0827 | 0.911471 | |
| NA |  | 1.17135 | | NA |  | 1.04429 | | NA |  | 1.04637 | | NA |  | NA |  |
| NA |  | 0.948333 | | NA |  | 1.01835 | | NA |  | 1.04704 | | NA |  | NA |  |
| 1.01384 | | 1.06388 | | NA |  | 0.980356 | | 0.989789 | |  | 1.0963 | NA |  | 0.939647 | |
| 0.969632 | | 1.15302 | | 0.985971 | | 1.02679 | | 1.02465 | | 1.02469 | | 0.951419 | | 0.934791 | |
| 1.04664 | | 0.904255 | | 1.03132 | | 0.99036 | | 0.973326 | | 1.03539 | | 1.07269 | | 0.907223 | |
| 1.07607 | | 1.05955 | | NA |  | 1.03174 | | 1.11134 | | 1.01266 | |  | 1.0963 | 1.03753 | |
| 0.981149 | | NA |  | NA |  | 0.963888 | | 0.94609 | | 1.00594 | | 0.99923 | | 1.04471 | |
|  | 1.0827 | 0.914089 | | 1.13997 | | 1.12874 | | 1.08388 | | 1.17126 | |  | 1.0815 | 1.13309 | |
| 0.978072 | | 1.16236 | | 1.03011 | | 0.996128 | | 0.990127 | | 1.06588 | | 1.00739 | |  | 1.076 |
| 1.09199 | | 1.21392 | | 1.08255 | | 1.07344 | | 1.14505 | | 1.16287 | | 1.16579 | | 1.19184 | |
|  | 1.0474 | 1.11905 | | 1.11542 | | 0.987631 | | 1.02661 | | 1.04307 | | 1.12308 | | 1.09877 | |
| 0.952347 | | NA |  | 1.02802 | | 1.02196 | | 0.984618 | | 1.06019 | | 1.04212 | | 0.982021 | |
| NA |  | 1.06207 | | NA |  | 1.12765 | | 1.10317 | | 1.13024 | | NA |  | 1.13031 | |
| 1.02262 | | 1.04041 | | 1.00477 | | 0.99073 | | 1.02487 | | 1.00193 | | 0.998057 | | 1.02785 | |
| 1.02145 | |  | 1.0529 |  | 1.0372 | 0.976272 | | 0.967627 | | 0.983005 | | 0.961844 | | 1.02737 | |
| 1.02413 | | 0.946809 | | 1.09073 | | 1.04101 | | 0.984511 | | 1.07746 | | 1.10932 | | 1.04798 | |
| 1.10934 | |  | 1.0229 | 1.19554 | | 1.07262 | | 1.10295 | | 1.05128 | | 1.06553 | | 0.94384 | |

| 1.10601 | | 0.965116 | | 1.09436 | | 1.09892 | | 1.02581 | |  | 1.0251 | 0.947195 | 1.07306 | |
| --- | --- | --- | --- | --- | --- | --- | --- | --- | --- | --- | --- | --- | --- | --- |
| 1.04293 | | 1.08017 | | 0.997888 | | 1.07365 | |  | 1.081 | 1.03833 | | 0.991481 |  | 1.0495 |
| 1.14246 | | 1.19887 | | 1.14838 | | 1.15382 | | 1.12039 | | 1.15169 | | 1.15396 |  | 1.1327 |
| 1.02949 | | 0.935961 | | NA |  | 1.06169 | | 0.988044 | | 0.984304 | | 0.971002 | 1.04018 | |
| 1.26685 | | 1.01026 | | 1.19837 | |  | 1.0874 | 1.26558 | | 1.29416 | | 1.10386 | 1.20238 | |
| 1.00124 | | NA |  | 0.985122 | | NA |  | NA |  | 0.995361 | | 0.989351 | NA |  |
| 0.970236 | | 1.09695 | | 0.979744 | | 0.976036 | | 0.990069 | | 1.00554 | | 0.995241 | 1.03797 | |
| 0.983665 | | NA |  | 0.995403 | | 1.00652 | | 0.998931 | | 0.985771 | | 1.02944 | NA |  |
| 1.11769 | | 0.853821 | | 1.07275 | |  | 1.0615 | 1.03433 | | 1.10813 | | 1.10763 | 1.00856 | |
| 1.04899 | | 0.821782 | | 1.06121 | | 0.941729 | | 1.00711 | | 1.08186 | | 1.04381 | 1.05939 | |
| 1.00314 | | 1.09328 | | 0.976327 | | 0.961124 | | 0.961317 | | 1.00141 | | 1.04939 | 0.906213 | |
| NA |  | NA |  | 0.987299 | | NA |  | NA |  | NA |  | NA | NA |  |
| 1.00625 | |  | 1.0751 | NA |  | 0.990241 | | 0.989689 | | 0.99472 | | 0.959337 | 1.05667 | |
| 1.09736 | | 1.05088 | | 1.13143 | | 1.11465 | | 1.04193 | | 1.08114 | | NA | 1.08384 | |
| 1.06176 | | 0.957441 | | NA |  | 1.09021 | | 1.03141 | | 1.04908 | | 0.988316 | 0.966635 | |
| 1.02704 | | 1.08901 | | 1.04179 | | 1.04795 | | 1.06507 | | 0.992229 | | 0.996094 | 1.09825 | |
| NA |  | 1.16301 | | 1.08495 | | 1.04636 | |  | 1.048 | 1.06999 | | 0.974465 | 1.14084 | |
| 1.02077 | | 1.01656 | | 1.01516 | | 1.00587 | | 1.06392 | | 0.94728 | | 0.965899 | 1.06987 | |
| 1.05489 | | NA |  | NA |  | 1.05063 | | NA |  | 0.97054 | | 0.950953 | NA |  |
| 1.03353 | | NA |  | NA |  | 1.03784 | | 1.04175 | | 0.990271 | | 0.989418 | 1.03273 | |
| 1.04044 | | 1.13537 | | 1.02628 | | 1.00998 | | 1.10479 | |  | 1.0837 | 1.04961 | 1.02483 | |
| 1.01638 | | NA |  | 1.00399 | | NA |  | 0.96322 | | NA |  | 1.02258 | 1.02978 | |
| 1.01893 | | 1.05226 | | 0.962591 | | 0.925976 | | 0.994323 | | 0.975331 | | 1.07525 | 0.920871 | |
| 1.03383 | | 0.900561 | | 0.979393 | | 0.937933 | | 1.00798 | | 1.00322 | | 0.99119 | 1.04175 | |
| 1.02292 | | NA |  | 1.01698 | | 1.00616 | | 1.02927 | | 0.988971 | | 0.983685 | NA |  |
| 1.09241 | | 1.02857 | | 1.12981 | | 1.08133 | | 1.12036 | | 1.07295 | | 1.05299 | 1.06608 | |
| 1.05318 | | 1.18944 | | 0.987604 | | 0.986625 | | 1.04141 | | 1.07606 | | 1.16372 | 0.921227 | |
| 1.05238 | | 0.899543 | | 0.893008 | |  | 1.0101 | 1.07193 | | 0.993235 | | 0.914591 | 0.984369 | |
| 0.992671 | | 1.28769 | | 1.03036 | | 0.988698 | | 0.977742 | | 1.03256 | | 0.966284 | 0.980318 | |
| 0.987336 | | NA |  | 0.918575 | | 0.927307 | | 0.942964 | | 1.03463 | | 0.961087 | 0.939706 | |
|  | 1.0434 | 0.909327 | | 0.999382 | | 0.99258 | |  | 1.0343 | 0.997273 | | 1.01275 | 1.04211 | |
| 1.14601 | | 1.17439 | | 1.21668 | | 1.13981 | | 1.18657 | | 1.16654 | | 1.15007 | 1.18047 | |
| 0.936989 | | 1.03103 | | 1.09084 | | 1.04577 | | 0.979316 | | 1.03942 | | 1.03114 | 0.977371 | |
| 1.02697 | | NA |  | 0.966937 | | 1.05376 | | 1.00761 | | 1.00656 | | 0.966228 | 1.05232 | |
| NA |  | NA |  | NA |  | 1.09994 | | NA |  | 1.08421 | | NA | NA |  |
| 1.03991 | | 1.34599 | |  | 1.006 |  | 1.0409 |  | 1.0137 | 1.05197 | | 1.04566 | 1.14472 | |
| 1.11788 | | 0.948276 | | 0.992689 | | 1.11829 | | 1.04251 | | 1.02243 | | 0.956484 | 0.912975 | |
| 1.01232 | | 1.24344 | | 1.07277 | | 1.05184 | | 0.996745 | | 0.984053 | | 1.00489 | 1.11866 | |

hematopoi hematopoi hematopoi hematopoi hematopoi hepatocyt hesc_deriv hesc_deriv

1.07551	1.16807	1.12777	1.09763	1.17156	1.072 NA	1.36364

NA	NA	NA	NA	NA	NA	NA	NA

1.24717	1.15294	1.20317	1.1073	1.17568	1.15125 NA	NA

1.19487	1.13355	1.11307	1.19565	1.1886	1.16603 NA	NA NA	1.1521	1.10801	1.05905	1.11727	1.14931 NA	NA

0.938225 0.906981 0.964234 0.889404 0.953544 0.945317 NA	1.11377

1.07005	1.03062	1.0409	1.00613	1.10205	1.18472 NA	NA

1.07709	1.10437	1.07401	1.1195	1.08334	1.16459 NA	NA

1.07709	1.10437	1.07401	1.1195	1.08334	1.16459 NA	NA

1.28939	1.20028 NA	NA	1.19405	1.2206 NA	NA

0.983739 0.951592	1.01242 0.999449	1.00861 0.930116 NA	0.768293

1.03627	1.05645	1.03478	1.05762	1.04344 0.989227 NA	0.977778

1.07904	1.06255 0.979417 0.957372	1.04507	1.01451 NA	NA

1.04706	1.04497	1.01451	1.01035 0.963643	1.0548 NA	NA NA	NA	NA	NA	NA	1.23623 NA	NA NA	NA	NA	NA	NA	NA	NA	NA

1.0049 NA	NA	NA	NA	0.9421 NA	NA

1.0049 NA	NA	NA	NA	0.9421 NA	NA

1.0008 0.957803 0.964821 0.941055 0.931133	0.92946 NA	NA

| 1.14791 | 1.17373 | 1.20004 | 1.12503 | 1.20141 | 1.21476 NA | NA |
| --- | --- | --- | --- | --- | --- | --- |

NA	NA	NA	NA	NA	NA	NA	NA

1.0534 0.981344	1.07592 0.996409	1.03864 0.897516 NA	NA NA	NA	NA	NA	NA	NA	NA	NA NA	NA	NA	NA	NA	NA	NA	NA

1.07922 NA	NA	NA	NA	0.966868 NA	NA NA	NA	NA	NA	NA	1.03505 NA	NA NA	NA	NA	NA	NA	1.04609 NA	NA NA	NA	NA	NA	NA	1.02214 NA	NA

1.19237	1.1363	1.12193	1.09312	1.22388	1.18704 NA	NA

1.19237	1.1363	1.12193	1.09312	1.22388	1.18704 NA	NA

1.1409	1.10876	1.12354	1.16355	1.24989	1.24611 NA	NA

1.1395	1.13488	1.17868	1.19074	1.25084	1.30464 NA	NA

1.24724	1.14266 NA	NA	1.15665	1.20602 NA	NA NA	NA	NA	NA	NA	1.0874 NA	NA NA	NA	NA	NA	NA	NA	NA	NA NA	NA	NA	NA	NA	NA	NA	NA NA	NA	NA	NA	NA	NA	NA	NA

1.11247	1.05587	1.05724	1.04983	1.11844 NA	NA	1.34615

| 0.977314 | | 0.939681 | | 0.973773 | | 0.93476 | | 0.980016 | | 0.977137 | NA | 0.703704 | |
| --- | --- | --- | --- | --- | --- | --- | --- | --- | --- | --- | --- | --- | --- |
| 0.977314 | | 0.939681 | | 0.973773 | | 0.93476 | | 0.980016 | | 0.977137 | NA | 0.703704 | |
| 0.977314 | | 0.939681 | | 0.973773 | | 0.93476 | | 0.980016 | | 0.977137 | NA | 0.703704 | |
| 1.05417 | | 1.04154 | | 1.03002 | | 1.07045 | | 1.05611 | | NA | NA | 1.40683 | |
| 1.09963 | | 1.00904 | | 0.97923 | | 1.01952 | | 0.988469 | | NA | NA |  | 1 |
| 1.05518 | | 1.03199 | | 1.03119 | | 0.990114 | | 1.01272 | | 0.961693 | NA | NA |  |
| 1.07411 | | 1.03139 | | 1.06362 | | 1.03747 | | 1.07646 | | 1.00962 | NA | NA |  |
|  | 1.0704 | 1.01914 | | 1.00836 | | 1.00178 | | 1.05858 | | 1.02156 | NA | NA |  |
| 1.10811 | | 1.03571 | | 1.02687 | | 0.977122 | | 0.999311 | | 0.960764 | 0.777778 | NA |  |
| 1.06292 | |  | 1.053 | 1.05913 | | 1.01378 | | 1.08825 | | 1.05169 | NA | NA |  |
|  | 1.1114 | 1.07122 | |  | 1.0718 | 1.02957 | | 1.07385 | | 1.05498 | NA | NA |  |
| 1.11406 | | 1.04629 | | 1.06136 | | 1.05484 | | 1.03179 | | 1.01026 | NA | NA |  |
|  | 1.1693 | 1.11943 | | 1.09862 | | 1.09345 | | 1.10945 | | 1.00939 | NA | NA |  |
|  | 1.0936 | 1.02623 | | 1.08952 | | 1.01174 | | 1.04785 | | 0.997988 | NA | NA |  |
|  | 1.0885 | 1.06693 | | 1.03431 | | 1.00973 | | 1.05256 | | 1.00307 | NA | NA |  |
| NA |  | NA |  | NA |  | NA |  | NA |  | NA | NA | NA |  |
| 1.13857 | | 1.11933 | | 1.16052 | | 1.12523 | | 1.17163 | | 1.21629 | NA | NA |  |
| 1.11088 | | 1.06097 | | 1.11223 | | 1.07334 | | 1.12229 | | 1.00324 | NA | NA |  |
| 1.13627 | | 1.13633 | | 1.11878 | | 1.10153 | |  | 1.1058 | 1.00506 | NA | NA |  |
| 1.04238 | |  | 1.0771 | 1.07703 | | 1.12545 | | 1.04946 | | 1.04595 | NA | NA |  |
| 1.09303 | | 1.00314 | | 1.03611 | | 1.02392 | | 1.01768 | | 0.942727 | NA | NA |  |
| NA |  | NA |  | NA |  | NA |  | NA |  | 1.02706 | NA | NA |  |
| NA |  | NA |  | NA |  | NA |  | NA |  | NA | NA | NA |  |
| 1.08686 | | 1.02358 | | 1.05506 | | 1.03571 | | 1.04377 | | 0.99634 | NA | NA |  |
|  | 1 | 1.13816 | | 1.04516 | | 1.02174 | | 1.12049 | | 1.07309 | NA | NA |  |
| 1.12192 | | 1.04828 | | 1.06486 | | 1.08377 | | 1.07994 | | 1.00289 | NA | NA |  |
| 1.15078 | |  | 1.0331 | 1.11256 | | 1.08426 | | 1.08982 | | 1.00613 | NA | NA |  |
| 1.08266 | | 1.04276 | | 1.09165 | | 1.04188 | | 1.06446 | | 1.00736 | NA | NA |  |
| 1.07144 | | 1.06978 | | 1.07484 | | 1.04252 | | 1.11447 | | 1.04302 | NA | NA |  |
| 1.07144 | | 1.06978 | | 1.07484 | | 1.04252 | | 1.11447 | | 1.04302 | NA | NA |  |
| 1.15385 | |  | 1.0756 | 1.11068 | |  | 1.0777 | 1.08627 | | 0.977366 | NA | NA |  |
| 1.14111 | | 1.09316 | | 1.03441 | |  | 1.0742 |  | 1.0858 | 1.02279 | NA | NA |  |
| 1.05841 | | 1.03058 | |  | 1.0241 | 1.01821 | | 1.04579 | | NA | NA | NA |  |
| 1.09205 | | 1.04117 | | 0.998848 | | 1.00934 | | 0.981081 | | 0.919238 | NA | NA |  |
| NA |  | NA |  | NA |  | NA |  | NA |  | NA | NA | NA |  |
| 0.991486 | | 1.03088 | | 0.982338 | | 0.981248 | | 1.00038 | | 0.960911 | NA | 1.02979 | |
| 1.10469 | | 1.01207 | | 1.10042 | | 1.06774 | | 1.06425 | | 1.01029 | NA | NA |  |
| 1.14605 | | 1.04162 | |  | 1.0944 | 1.07162 | | 1.06436 | | 0.981114 | NA | NA |  |
| 1.09999 | |  | 1.1735 | 1.08469 | | 1.22039 | | 1.09159 | | 1.08234 | NA | NA |  |

| 0.993104 | 1.08132 | 0.936766 | 0.999033 | 1.04891 | 1.03956 | | NA |  | NA |  |
| --- | --- | --- | --- | --- | --- | --- | --- | --- | --- | --- |
| 1.01204 | 0.99466 | 0.894979 | 0.954096 | 0.970132 | 1.00426 | | NA |  | NA |  |
| 1.03485 | 1.04236 | 0.997089 | 1.00112 | 1.02074 | 0.987615 | | NA |  | NA |  |
| 1.04696 | 1.07169 | NA | NA | 1.02709 | NA |  | NA |  | NA |  |
| NA | NA | NA | NA | NA | 1.00209 | | 0.333333 | | NA |  |
| NA | NA | NA | NA | NA | 1.01639 | | 0.378378 | | NA |  |
| NA | NA | NA | NA | NA | 1.04161 | |  | 0.7 | NA |  |
| NA | NA | NA | NA | NA | 1.03042 | | NA |  | NA |  |
| 0.958761 | NA | NA | NA | NA | 1.02306 | | 0.424581 | | NA |  |
| 0.968803 | NA | NA | NA | NA |  | 1.0101 | NA |  | NA |  |
| NA | NA | NA | NA | NA | 1.02384 | | NA |  | NA |  |
| NA | NA | NA | NA | NA | 1.02384 | | NA |  | NA |  |
| NA | NA | NA | NA | NA | 1.02384 | | NA |  | NA |  |
| NA | NA | NA | NA | NA | 1.01023 | | NA |  | NA |  |
| 1.01289 | NA | NA | NA | NA | 1.03033 | | NA |  | NA |  |
| 1.00913 | 1.07441 | 1.04769 | 1.05413 | 1.01196 | 1.02664 | | 0.30622 | | NA |  |
| NA | NA | NA | NA | NA | 1.03164 | | NA |  | NA |  |
| NA | NA | NA | NA | NA | 1.00713 | | NA |  | NA |  |
| 1.04113 | 1.11573 | 1.06732 | 1.07612 | 1.03423 | 1.01057 | | 0.463054 | | NA |  |
| 0.966555 | NA | NA | 1.00629 | 0.983105 | 1.04681 | | 0.433497 | | NA |  |
| NA | NA | NA | NA | NA |  | 1.0129 | NA |  | NA |  |
| 1.16103 | 1.06682 | 1.08583 | 1.08948 | 1.12086 | 1.01309 | | NA |  | NA |  |
| 0.986565 | 0.949137 | 0.971122 | 0.971065 | 0.947525 | 0.986763 | | 0.923077 | | NA |  |
| 0.986565 | 0.949137 | 0.971122 | 0.971065 | 0.947525 | 0.986763 | | 0.923077 | | NA |  |
| 0.989641 | NA | NA | 1.09165 | NA | 0.960127 | | 1.05882 | | NA |  |
| 0.989641 | NA | NA | 1.09165 | NA | 0.960127 | | 1.05882 | | NA |  |
| 1.01989 | 1.01317 | 1.02258 | 1.03044 | 1.00164 |  | 1.0163 | 1.11232 | | NA |  |
| 1.03147 | 1.05223 | 1.01131 | 1.07804 | 1.01412 | 1.03208 | |  | 1 | NA |  |
| 1.00008 | NA | NA | 1.04673 | NA | 0.961412 | | 1.16667 | | NA |  |
| 1.05858 | NA | NA | 1.07891 | NA | 1.02209 | | 1.01716 | | NA |  |
| 1.05858 | NA | NA | 1.07891 | NA | 1.02209 | | 1.01716 | | NA |  |
| 1.05858 | NA | NA | 1.07891 | NA | 1.02209 | | 1.01716 | | NA |  |
| NA | NA | NA | NA | NA | NA |  | NA |  | NA |  |
| NA | NA | NA | NA | NA | NA |  | NA |  | NA |  |
| NA | NA | NA | NA | NA | NA |  | NA |  | NA |  |
| NA | NA | NA | NA | NA | NA |  | NA |  | NA |  |
| 0.978455 | 1.01962 | 0.931241 | 0.97738 | 1.00596 | 1.02407 | | NA |  | NA |  |
| 1.19127 | 1.06488 | 1.06932 | 1.03113 | 1.11561 | 1.09087 | | NA |  |  | 1.26 |
| 1.19127 | 1.06488 | 1.06932 | 1.03113 | 1.11561 | 1.09087 | | NA |  |  | 1.26 |

| 1.12225 | 1.08757 | 1.08722 | 1.12951 | | 1.20543 | | 1.17096 | | NA | NA |  |
| --- | --- | --- | --- | --- | --- | --- | --- | --- | --- | --- | --- |
| NA | NA | NA | NA |  | NA |  | 1.13468 | | NA | NA |  |
| 1.17608 | 1.03563 | 1.08048 |  | 1.0212 | 1.02902 | | 1.08308 | | NA | NA |  |
| NA | 0.962068 | NA | NA |  | 0.954426 | |  | 1.0829 | NA |  | 1.8 |
| 1.03599 | 1.02498 | 0.968786 | 1.04461 | | 1.00209 | | 0.976858 | | NA | 0.636364 | |
| NA | NA | NA | NA |  | NA |  | 1.08269 | | NA | NA |  |
| NA | NA | NA | NA |  | NA |  | 0.97709 | | NA | NA |  |
| NA | NA | NA | NA |  | NA |  | 1.01283 | | NA | NA |  |
| NA | NA | NA | NA |  | 0.907429 | | 1.04435 | | NA | 0.909091 | |
| NA | NA | NA | NA |  | 1.01235 | | 1.03386 | | NA |  | 0.8125 |
| 0.906935 | 0.962528 | 0.932127 | 0.958514 | | 0.922139 | | 1.03702 | | NA | 1.53846 | |
| NA | NA | NA | NA |  | NA |  | NA |  | NA | NA |  |
| NA | NA | NA | NA |  | NA |  | NA |  | NA | NA |  |
| NA | NA | NA | NA |  | NA |  | NA |  | NA | NA |  |
| NA | NA | NA | NA |  | NA |  | NA |  | NA | NA |  |
| NA | NA | NA | NA |  | NA |  | NA |  | NA | NA |  |
| NA | NA | NA | NA |  | NA |  | NA |  | NA | NA |  |
| NA | NA | NA | NA |  | NA |  | NA |  | NA | NA |  |
| NA | NA | NA | NA |  | NA |  | NA |  | NA | NA |  |
| 1.03902 | 1.02814 | 0.989782 | 1.06857 | |  | 1.0306 | NA |  | NA | NA |  |
| NA | NA | NA | NA |  | NA |  | NA |  | NA | NA |  |
| NA | NA | NA | NA |  | NA |  | NA |  | NA | NA |  |
| NA | NA | NA | NA |  | NA |  | NA |  | NA | NA |  |
| NA | NA | NA | NA |  | NA |  | NA |  | NA | NA |  |
| NA | NA | NA | NA |  | NA |  | NA |  | NA | NA |  |
| 0.951558 | 0.938104 | 0.913534 | 0.969839 | | 0.976742 | | NA |  | NA | NA |  |
| NA | NA | NA | NA |  | NA |  | NA |  | NA | NA |  |
| 1.01746 | NA | NA | NA |  | NA |  | NA |  | NA | NA |  |
| NA | NA | NA | NA |  | NA |  | NA |  | NA | NA |  |
| NA | NA | NA | NA |  | NA |  | NA |  | NA | NA |  |
| 0.98705 | 0.979502 | 0.979873 | 0.996267 | | 1.00605 | | 1.03328 | | NA | 1.81818 | |
| NA | NA | NA | NA |  | NA |  | NA |  | NA | NA |  |
| 0.938478 | NA | NA | NA |  | NA |  | NA |  | NA | NA |  |
| NA | NA | NA | NA |  | NA |  | NA |  | NA | NA |  |
| NA | NA | NA | NA |  | NA |  | NA |  | NA | NA |  |
| 1.06641 | NA | NA | NA |  | NA |  | NA |  | NA | NA |  |
| NA | NA | NA | NA |  | NA |  | NA |  | NA | NA |  |
| 1.06467 | NA | NA | NA |  | NA |  | NA |  | NA | NA |  |
| 0.959839 | NA | NA | NA |  | NA |  | NA |  | NA | NA |  |

| NA | NA |  | NA |  | NA | NA |  | NA |  | NA | NA |  |
| --- | --- | --- | --- | --- | --- | --- | --- | --- | --- | --- | --- | --- |
| 1.03424 | 0.992519 | | 0.968925 | | 1.07391 | 1.00501 | | NA |  | NA | NA |  |
| NA | NA |  | NA |  | NA | NA |  | NA |  | NA | NA |  |
| NA | NA |  | NA |  | NA | NA |  | NA |  | NA | NA |  |
| NA | NA |  | NA |  | NA | NA |  | NA |  | NA | NA |  |
| 1.10317 | NA |  | 1.12585 | | NA | NA |  | NA |  | NA | NA |  |
| NA | NA |  | NA |  | NA | NA |  | NA |  | NA | NA |  |
| 1.04683 | NA |  | NA |  | NA | NA |  | NA |  | NA | NA |  |
| NA | NA |  | NA |  | NA | NA |  | NA |  | NA | NA |  |
| 1.04598 | NA |  | NA |  | NA | NA |  | NA |  | NA | NA |  |
| 0.988118 | 0.979177 | | 0.993976 | | 0.964927 | 1.01366 | | 0.984855 | | NA | NA |  |
| 1.29788 | 1.03801 | | NA |  | 1.02774 | 1.23374 | |  | 1.2039 | NA | NA |  |
| 1.08152 | 1.01232 | | NA |  | 0.961038 | 1.02278 | | NA |  | NA | NA |  |
| 1.01183 | 1.03217 | | 0.985239 | | 0.993412 | 0.968337 | | 0.990933 | | NA | NA |  |
| 1.24278 | 1.14677 | | 1.09462 | | 1.15922 | 1.11612 | | 1.01794 | | NA | NA |  |
| 1.12279 | 1.00142 | |  | 1.0178 | 1.00706 | 1.08152 | |  | 1.1019 | NA | NA |  |
| NA | NA |  | NA |  | NA | NA |  | 1.16036 | | NA | NA |  |
| 1.00206 | 0.991122 | | 0.999217 | | 0.964245 | 1.00654 | | 1.02683 | | NA | NA |  |
| 1.02279 | 1.04741 | | 1.00556 | | 1.00444 | 1.09544 | | 1.06886 | | NA | NA |  |
| 1.17431 | 1.18367 | | 1.06667 | | 1.08937 | 1.11747 | | 1.09601 | | NA | NA |  |
| 1.04617 | 1.04856 | | 0.982525 | | NA | 0.990363 | | NA |  | NA | NA |  |
| 1.02657 |  | 1.0601 | 0.930728 | | 1.01171 | 0.987826 | | NA |  | NA | NA |  |
| 1.08643 |  | 1.0415 | 0.936324 | | 0.982781 | 1.01308 | | 0.989183 | | NA | NA |  |
| 1.02495 | NA |  | NA |  | NA | NA |  | NA |  | NA | NA |  |
| NA | 1.04315 | | 1.07468 | | 1.03318 | 1.00842 | | NA |  | NA | NA |  |
| 1.04625 | 1.06685 | | 1.11073 | | 1.05804 | 1.03111 | | 1.00759 | | NA | 1.37636 | |
| NA | 1.02578 | | 1.00398 | | 1.00633 | 1.06614 | | NA |  | NA | NA |  |
| 1.06235 |  | 1.0316 | 1.12065 | | 1.04842 | 1.09704 | | 1.02584 | | NA | NA |  |
| 1.06235 |  | 1.0316 | 1.12065 | | 1.04842 | 1.09704 | | 1.02584 | | NA | NA |  |
| NA | 1.02853 | | 1.08341 | | 1.04936 |  | 1.0351 | NA |  | NA | NA |  |
| 1.08756 | 1.05731 | | 1.05185 | | 1.02078 | 1.05705 | | NA |  | NA |  | 0.6 |
| NA |  | 1.0248 | 1.02831 | | 1.05022 | 1.07308 | | NA |  | NA | NA |  |
| NA |  | 1.0248 | 1.02831 | | 1.05022 | 1.07308 | | NA |  | NA | NA |  |
| NA |  | 1.0248 | 1.02831 | | 1.05022 | 1.07308 | | NA |  | NA | NA |  |
| 1.06127 | 0.989228 | | 0.999125 | | 1.02784 |  | 1.0314 | NA |  | NA | 0.555556 | |
| 0.973715 | 0.961509 | | 1.02783 | | 1.02451 | 0.99702 | | NA |  | NA | 0.522523 | |
| NA | NA |  | NA |  | NA |  | 1.0765 | NA |  | NA | NA |  |
| NA | NA |  | NA |  | NA | NA |  | NA |  | NA | NA |  |
| 1.11167 | NA |  | 1.05442 | | NA | 1.05916 | | 0.972814 | | NA |  | 1 |

| NA |  | NA |  | NA | NA |  | NA |  | NA |  | NA |  | NA |  |
| --- | --- | --- | --- | --- | --- | --- | --- | --- | --- | --- | --- | --- | --- | --- |
| NA |  | NA |  | NA | NA |  | NA |  | NA |  | NA |  | NA |  |
| NA |  | NA |  | NA | NA |  | NA |  | NA |  | NA |  | NA |  |
| NA |  | NA |  | NA | NA |  | NA |  | NA |  | NA |  | NA |  |
| 0.948919 | | 0.933754 | | 0.975659 | 0.888603 | | 0.98035 | | 0.915656 | | NA |  | 1.26466 | |
| 0.982707 | | 0.950666 | | 0.957445 | 0.955416 | | 1.05476 | |  | 1.002 | 0.857143 | |  | 1.5396 |
| 0.99478 | | 0.987621 | | 0.97153 | 0.976833 | | 0.939203 | | 0.986716 | | NA |  |  | 1 |
| 0.978794 | | 0.996671 | | 1.00856 | 0.987382 | | 1.00472 | | 0.990069 | | NA |  | 0.97619 | |
| 0.962277 | | 0.974603 | | 0.930318 | 0.911476 | | 1.03778 | | 0.96621 | | NA |  | 1.40909 | |
| 1.04312 | | 1.00422 | | 0.95983 | 0.953403 | | 1.00277 | | 0.992504 | | NA |  | NA |  |
|  | 1.0488 | 1.04848 | | 1.05455 |  | 1.0645 | 1.15493 | |  | 1.1068 | NA |  | NA |  |
| 1.09683 | | 1.10673 | | 1.07089 | 1.06653 | | 1.06894 | | 1.19613 | | NA |  | 1.47368 | |
| 1.23074 | |  | 1.2151 | 1.21371 | 1.19294 | |  | 1.2215 |  | 1.271 | NA |  | 0.837209 | |
| 0.930159 | | 1.02321 | | 0.963788 | 0.96807 | | 0.99113 | | 0.977987 | | NA |  | 1.73333 | |
| NA |  | NA |  | NA | NA |  | NA |  | NA |  | NA |  | NA |  |
| NA |  | NA |  | NA | NA |  | NA |  | NA |  | NA |  | NA |  |
| 1.02187 | |  | 1.1049 | 1.09556 | 1.03671 | | 1.14416 | | 1.13213 | | NA |  | NA |  |
| NA |  | NA |  | NA | NA |  | NA |  | NA |  | NA |  | NA |  |
| 0.952481 | | 0.892644 | | 0.931566 | 0.881434 | | 0.969931 | | 0.973774 | |  | 1.1 | 0.745763 | |
| NA |  | NA |  | NA | NA |  | NA |  | NA |  | NA |  | NA |  |
| NA |  | NA |  | NA | NA |  | NA |  | NA |  | NA |  | NA |  |
| 1.13089 | | 1.17457 | | 1.19297 | 1.09267 | | 1.20207 | | 1.13313 | | NA |  | NA |  |
|  | 1.0944 | 1.06368 | | 1.08514 | 1.07999 | | 1.23067 | | 1.16327 | | NA |  | NA |  |
|  | 1.0944 | 1.06368 | | 1.08514 | 1.07999 | | 1.23067 | | 1.16327 | | NA |  | NA |  |
|  | 1.0944 | 1.06368 | | 1.08514 | 1.07999 | | 1.23067 | | 1.16327 | | NA |  | NA |  |
|  | 1.0944 | 1.06368 | | 1.08514 | 1.07999 | | 1.23067 | | 1.16327 | | NA |  | NA |  |
| 1.11902 | | 1.06291 | | 1.08753 | 1.08138 | | 1.05049 | | 1.01626 | | NA |  | NA |  |
| 1.11902 | | 1.06291 | | 1.08753 | 1.08138 | | 1.05049 | | 1.01626 | | NA |  | NA |  |
| 1.11902 | | 1.06291 | | 1.08753 | 1.08138 | | 1.05049 | | 1.01626 | | NA |  | NA |  |
| 1.11902 | | 1.06291 | | 1.08753 | 1.08138 | | 1.05049 | | 1.01626 | | NA |  | NA |  |
|  | 1.1966 | 1.14586 | | 1.14754 | 1.15899 | | 1.13703 | | 1.20682 | | NA |  | NA |  |
|  | 1.1966 | 1.14586 | | 1.14754 | 1.15899 | | 1.13703 | | 1.20682 | | NA |  | NA |  |
| 1.10059 | | 1.03869 | | 0.96635 | 0.995936 | | 1.06149 | | 1.12607 | | NA |  | NA |  |
| 1.10059 | | 1.03869 | | 0.96635 | 0.995936 | | 1.06149 | | 1.12607 | | NA |  | NA |  |
| 1.17219 | | 1.11339 | | 1.12719 | 1.15283 | | 1.21836 | | 1.25923 | | NA |  | NA |  |
| 0.959775 | | 0.89587 | | 0.913559 | 0.88841 | | 0.995883 | | 0.969948 | | NA |  |  | 1.2622 |
| 0.959775 | | 0.89587 | | 0.913559 | 0.88841 | | 0.995883 | | 0.969948 | | NA |  |  | 1.2622 |
| 0.977238 | | 0.936616 | | 0.954977 | 0.904675 | | 1.04922 | | 0.957984 | | NA |  | 0.793651 | |
| 0.977238 | | 0.936616 | | 0.954977 | 0.904675 | | 1.04922 | | 0.957984 | | NA |  | 0.793651 | |

| 0.980343 | 0.957472 | | 0.985031 | | 0.947878 | | 1.01724 | | 0.957295 | | NA | 0.810345 | |
| --- | --- | --- | --- | --- | --- | --- | --- | --- | --- | --- | --- | --- | --- |
| 0.926867 | 0.94856 | | 0.941149 | | 0.898278 | | 0.998673 | | 0.977043 | | NA | 0.527778 | |
| 1.00063 | 0.972426 | | 0.994847 | | 0.992507 | | 0.994877 | | 0.994012 | | NA | 0.596154 | |
| 0.996864 | 1.03317 | | 1.00914 | | 0.995644 | | 1.04172 | | 0.985309 | | NA | NA |  |
| 1.01108 | 0.982972 | | 0.999638 | | 0.996947 | | 1.02234 | |  | 1.0051 | NA | NA |  |
| 1.08092 | 1.04831 | | NA |  | NA |  | 1.01516 | | NA |  | NA | NA |  |
| 1.04628 | 1.01975 | | 0.95437 | | 0.944601 | | 1.08893 | | 1.04676 | | NA | NA |  |
| 1.04675 |  | 1.0418 | 1.05257 | | 1.07613 | |  | 1.019 | 1.04482 | | NA | NA |  |
| 1.03487 | 1.04657 | | 1.03194 | |  | 1.0296 | 1.01395 | | 1.02587 | | NA | NA |  |
| NA | NA |  | NA |  | NA |  | 1.00144 | | 0.998068 | | NA | NA |  |
| 1.01909 | 1.00391 | |  | 1.0052 | 1.01044 | | 0.978151 | | 1.03571 | | NA | NA |  |
| 1.05867 | 1.02583 | | 1.02684 | | 1.02656 | | 1.04619 | | 0.993235 | | NA | NA |  |
| NA | NA |  | NA |  | NA |  |  | 1.0059 | NA |  | NA | NA |  |
| 1.02262 | 1.13679 | | 1.07935 | | 1.04747 | | 1.07109 | | NA |  | NA | 1.38889 | |
| NA | NA |  | NA |  | NA |  | NA |  | NA |  | NA | NA |  |
| NA | NA |  | NA |  | NA |  | NA |  | NA |  | NA | NA |  |
| NA | NA |  | NA |  | NA |  | NA |  | NA |  | NA | NA |  |
| 0.985956 | 0.982705 | | 0.974812 | | 1.01344 | | 1.00115 | | NA |  | NA | NA |  |
| NA | NA |  | NA |  | NA |  | NA |  | NA |  | NA | NA |  |
| NA | NA |  | NA |  | NA |  | NA |  | NA |  | NA | NA |  |
| NA | NA |  | NA |  | NA |  | NA |  | NA |  | NA | NA |  |
| NA | NA |  | NA |  | NA |  | NA |  | NA |  | NA | NA |  |
| NA | NA |  | NA |  | NA |  | NA |  | NA |  | NA | NA |  |
| 1.01294 | 1.03299 | | 1.01791 | | 0.923896 | | 1.05564 | | NA |  | NA | NA |  |
| 1.02958 | 1.01947 | | 1.01011 | | 0.996633 | |  | 1.0292 | NA |  | NA | NA |  |
| NA | NA |  | NA |  | NA |  | NA |  | NA |  | NA | NA |  |
| NA | NA |  | NA |  | NA |  | NA |  | NA |  | NA | NA |  |
| NA | NA |  | NA |  | NA |  | NA |  | NA |  | NA | NA |  |
| 1.11197 |  | 1.0765 | 1.13604 | | 1.11881 | |  | 1.2108 | 1.21322 | | NA | NA |  |
| 1.08086 | 1.01788 | | 1.06229 | | 1.06605 | | 1.00715 | | 0.983936 | | NA |  | 1 |
| 1.08086 | 1.01788 | | 1.06229 | | 1.06605 | | 1.00715 | | 0.983936 | | NA |  | 1 |
| NA | NA |  | NA |  | NA |  | NA |  | 1.15429 | | NA | 0.333333 | |
| NA | NA |  | NA |  | NA |  | 0.967326 | | 1.02893 | | NA | 0.826087 | |
| NA | NA |  | NA |  | NA |  | NA |  | 0.984446 | | NA | 0.842105 | |
| NA | 0.957129 | | 0.935683 | | 1.01252 | | 0.950224 | | 1.03399 | | NA | 0.764706 | |
| 1.11004 | 1.12329 | | 1.05986 | | 1.06715 | | 1.07707 | | 1.04394 | | NA | NA |  |
| NA | NA |  | NA |  | NA |  | 1.01741 | | 1.05461 | | NA | 1.14286 | |
| NA | NA |  | NA |  | NA |  | NA |  | 1.06284 | | NA | NA |  |
| NA | NA |  | NA |  | NA |  | 0.947192 | | 1.01749 | | NA | 0.842105 | |

| 0.91676 | | 0.942436 | | 0.956275 | | 1.00165 | | 0.956274 | | 1.02766 | | NA |  | 1.4 |
| --- | --- | --- | --- | --- | --- | --- | --- | --- | --- | --- | --- | --- | --- | --- |
| NA |  | NA |  | NA |  | NA |  | NA |  | 0.968478 | | NA | NA |  |
| NA |  | NA |  | NA |  | NA |  | NA |  | 1.00342 | | NA | NA |  |
| NA |  | NA |  | NA |  | NA |  | NA |  | 0.996373 | | NA | NA |  |
| NA |  | NA |  | NA |  | NA |  | NA |  | 0.984772 | | NA | NA |  |
| 0.935351 | | 0.957753 | | 0.932032 | | 0.942093 | | 1.01919 | | 1.00024 | | NA | 1.32039 | |
| NA |  | NA |  | NA |  | NA |  | NA |  | 1.04253 | | NA | NA |  |
| NA |  | NA |  | NA |  | NA |  | NA |  | 1.04253 | | NA | NA |  |
| NA |  | NA |  | NA |  | NA |  | NA |  | 1.04253 | | NA | NA |  |
| NA |  | NA |  | NA |  | NA |  | NA |  | NA |  | NA | NA |  |
|  | 1.1129 | 0.999312 | | 0.988955 | | 1.09803 | | 1.00426 | | 0.964615 | | NA | 0.066667 | |
| NA |  | NA |  | NA |  | 1.02542 | | 1.12124 | | 1.01613 | | NA | 0.818182 | |
| 1.10379 | | 1.04278 | | 1.15816 | | 1.07744 | | 1.11477 | | 1.02141 | | NA | 1.19672 | |
| NA |  | NA |  | NA |  | NA |  | NA |  | NA |  | NA | NA |  |
| NA |  | NA |  | NA |  | NA |  | 1.12125 | |  | 1.0045 | NA | NA |  |
| 0.935957 | |  | 1 | 0.982612 | | 1.06618 | | 1.08415 | | 1.03457 | | NA |  | 0.75 |
| 1.00151 | | 1.05508 | | 0.975202 | | 1.07489 | | 1.06576 | | 1.08559 | | NA |  | 1.1875 |
| NA |  | NA |  | NA |  | NA |  | 1.04438 | | 1.02291 | | NA | 0.791667 | |
| NA |  | NA |  | NA |  | NA |  | NA |  | NA |  | NA | NA |  |
| 0.974824 | | 1.00379 | | 0.990855 | | 0.99514 | | 0.981427 | | NA |  | NA | 0.895833 | |
| 1.05773 | | 1.00033 | | 1.06951 | |  | 1.0475 | 1.01933 | | 0.988138 | | NA | 1.07831 | |
| 0.967757 | | 0.996494 | | 0.996748 | | 1.00245 | | 1.02127 | | 1.01003 | | NA | 0.764706 | |
| 0.863285 | | 0.948413 | | 0.952915 | | 0.967615 | | 0.948064 | | 0.991767 | | NA |  | 0.625 |
| 1.22262 | | 1.18167 | | 1.15339 | | 1.20006 | |  | 1.232 | 1.06533 | | NA | NA |  |
| 1.04653 | | 1.02339 | | 1.01371 | | 1.00353 | | 0.995977 | | 0.94866 | | NA | 1.22222 | |
| NA |  | 0.92581 | | 0.967265 | | 1.00115 | | 0.97079 | | 1.06046 | | NA | NA |  |
| 1.03288 | | 1.11509 | | 1.08913 | | 1.06071 | | 1.05765 | | 0.980534 | | NA | 2.15385 | |
| 1.01392 | | 1.05048 | |  | 1.0355 | 0.998489 | | 0.962134 | | 0.990567 | | NA | 1.07407 | |
| 1.03833 | | 1.01478 | | 0.969381 | | 0.988279 | | 1.02296 | | 0.998907 | | NA | NA |  |
| 1.05284 | | 1.01916 | | 0.982975 | |  | 1.0808 | 1.08908 | | 1.06055 | | NA | 0.923077 | |
| 1.00351 | | 0.958621 | | 1.12963 | | 0.990625 | | 1.03562 | | 1.09505 | | NA |  | 0.75 |
| 1.10135 | | 1.07314 | | 1.03492 | | 1.05343 | | 1.02402 | | 1.02865 | | NA |  | 0.65 |
| 1.04792 | | 0.965116 | | 0.968326 | |  | 1.074 | 0.959459 | | 0.967855 | | NA | NA |  |
|  | 1.0105 | 1.08284 | | 1.05251 | | 1.07151 | | 1.02016 | | 0.942773 | | NA |  | 1.125 |
| 1.05647 | | 0.976534 | | 0.896761 | | 0.965116 | | 1.04919 | | 1.03982 | | NA | NA |  |
| 1.13768 | | NA |  | NA |  | 1.11062 | | 1.05028 | | 1.00777 | | NA | 0.461538 | |
| NA |  | NA |  | NA |  | NA |  | NA |  | NA |  | NA | 2.22222 | |
| NA |  | NA |  | NA |  | NA |  | NA |  | NA |  | NA | 2.22222 | |
| NA |  | NA |  | NA |  | NA |  | NA |  | NA |  | NA | 2.22222 | |

| 0.999703 | | 0.961736 | | 0.976482 | | 0.984365 | | 0.955689 | | NA |  | NA |  | NA |  |
| --- | --- | --- | --- | --- | --- | --- | --- | --- | --- | --- | --- | --- | --- | --- | --- |
| 1.02413 | | 0.945797 | | 0.981919 | | 0.959688 | | 0.97973 | | NA |  | NA |  | NA |  |
| 1.14718 | | 1.04285 | | 1.12419 | | 1.09154 | | 1.08125 | | NA |  | NA |  | NA |  |
| NA |  |  | 1.001 | 1.03504 | | 1.03986 | | 0.974534 | | 1.01113 | | NA |  | 0.607143 | |
| NA |  | NA |  | NA |  | NA |  | NA |  |  | 1.0004 | NA |  | 1.47222 | |
| NA |  | NA |  | NA |  | NA |  | NA |  | 1.07605 | | NA |  |  | 0.6 |
| NA |  | NA |  | NA |  | NA |  | NA |  | 1.01833 | | NA |  | 1.61538 | |
| 1.19812 | | 1.15315 | | 1.18585 | | 1.08357 | | 1.09137 | | 1.00884 | | NA |  | NA |  |
| 1.04916 | | 1.05498 | | 1.06864 | | 1.09507 | | 1.07287 | | 0.95333 | | NA |  | 0.921053 | |
| 1.05184 | |  | 1.0424 | 1.05647 | | 1.02947 | | 1.02834 | | 0.973599 | |  | 0.75 |  | 1 |
| 1.03706 | | 1.02654 | |  | 1.0412 | 1.05622 | | 1.00359 | | 0.980517 | | NA |  | 1.11628 | |
| 1.03706 | | 1.02654 | |  | 1.0412 | 1.05622 | | 1.00359 | | 0.980517 | | NA |  | 1.11628 | |
| 1.07165 | | 1.04553 | | 1.04116 | | 1.09265 | | 1.03125 | | 1.04083 | | NA |  | 1.26168 | |
| 0.987184 | | 0.971004 | | NA |  | NA |  | 0.965588 | | NA |  | NA |  | NA |  |
| 0.987184 | | 0.971004 | | NA |  | NA |  | 0.965588 | | NA |  | NA |  | NA |  |
| 0.97135 | | NA |  | NA |  | NA |  | 0.977134 | | 0.995302 | | NA |  | 0.932067 | |
| 1.03408 | | NA |  | NA |  | NA |  | 0.983627 | | 0.947904 | | NA |  | 1.32222 | |
| 0.927859 | | NA |  | NA |  | NA |  | 0.939853 | | 0.990955 | | 0.784616 | | 1.08187 | |
| 1.11161 | | 1.08877 | | 1.07204 | | 1.06957 | | 1.12478 | | NA |  | NA |  | 0.562091 | |
| 1.11063 | | 1.03301 | | 1.06157 | | 1.04979 | |  | 1.0917 | 1.06746 | | NA |  | NA |  |
| 1.06107 | | 0.997667 | | 1.06954 | | 0.946501 | | 1.02731 | | 0.972907 | | NA |  | NA |  |
| 1.06096 | |  | 1.0055 | 0.997094 | | 1.01792 | | 0.961994 | | NA |  | NA |  | NA |  |
| 1.06202 | | 1.00742 | | 0.973817 | |  | 1.0407 | 1.00607 | | 1.00842 | | 1.26829 | | NA |  |
|  | 1.0812 | 0.992879 | | 1.03655 | | 0.981985 | | 1.01446 | | 1.03241 | | NA |  | NA |  |
| 1.06884 | |  | 1.0318 | 1.00993 | |  | 1.0065 | 1.00964 | | 1.00717 | | NA |  | NA |  |
| 1.04093 | | 1.03156 | | 1.00968 | | 1.05005 | |  | 1.0259 |  | 1.0066 | NA |  | NA |  |
| 1.01363 | | 1.04441 | | 1.01113 | | 1.05186 | | 1.01905 | | 1.01354 | | NA |  | NA |  |
| 1.03582 | | 1.04313 | | 1.06724 | | 1.09608 | | 1.08805 | | 0.988794 | | NA |  | NA |  |
| 0.906684 | | 0.861088 | | 0.903365 | | 0.942935 | | 0.95265 | | 1.01475 | | NA |  | NA |  |
| 0.970227 | | 1.00279 | | 1.03409 | | 0.918415 | | 1.05812 | | 0.995328 | | NA |  | 1.13333 | |
| 0.909058 | | 0.88589 | | 0.876579 | | 0.806373 | | 0.918117 | | 0.952454 | | NA |  | 0.956522 | |
| NA |  | NA |  | 0.918892 | | NA |  | 0.975595 | | 0.990934 | | NA |  | NA |  |
| 0.987768 | | NA |  | NA |  | NA |  | NA |  | 0.967539 | | NA |  | 1.31579 | |
| NA |  | NA |  | NA |  | NA |  | NA |  | 1.01284 | | NA |  | NA |  |
| NA |  | NA |  | NA |  | NA |  | NA |  | 0.953472 | | NA |  | NA |  |
| NA |  | 1.05938 | | 1.03018 | | 1.03319 | | 1.04181 | | NA |  | NA |  | 0.946602 | |
| 1.01553 | | 1.07274 | | 1.06579 | | 1.06055 | | 1.04557 | | 1.02308 | | NA |  | 1.14084 | |
| 1.00286 | | 0.998464 | | 1.05894 | | 1.03687 | | 1.03209 | | 1.02645 | | NA |  |  | 0.65 |
| NA |  | NA |  | NA |  | NA |  | NA |  | NA |  | NA |  | NA |  |

| 1.08349 | |  | 1.0674 |  | 1.0738 | 1.08728 | |  | 1.0988 | 1.03813 | | NA | 0.71223 | |
| --- | --- | --- | --- | --- | --- | --- | --- | --- | --- | --- | --- | --- | --- | --- |
| 0.902781 | | 0.861256 | | 0.920471 | | 0.878299 | | 0.941866 | | 0.990526 | | NA | 0.974603 | |
| 1.07424 | | 1.09467 | | 1.09955 | | 1.09603 | | 1.13496 | | 1.10747 | | NA | NA |  |
| 1.04949 | | 1.01096 | | 1.01282 | | 1.01867 | | 1.08392 | | 1.07779 | | NA | NA |  |
| 0.956475 | | 1.01172 | | 0.978567 | | 0.997867 | | 1.04095 | | 1.00625 | | NA | NA |  |
| 0.952507 | | 0.988871 | | 0.971042 | | 1.03545 | | 1.02771 | | 0.966456 | | NA | NA |  |
| NA |  | 0.964349 | | NA |  | NA |  | 1.01092 | | 1.03001 | | NA | NA |  |
| 0.990404 | |  | 1.0018 | 0.950667 | | 0.982844 | | 1.04893 | | 1.07494 | | NA | NA |  |
| 1.05934 | | 1.08584 | | 1.00747 | | 1.00458 | | 1.05971 | | 1.09197 | | NA | NA |  |
| NA |  | NA |  | NA |  | NA |  | NA |  | NA |  | NA | NA |  |
| NA |  | NA |  | NA |  | NA |  | NA |  | NA |  | NA | NA |  |
| NA |  | NA |  | NA |  | NA |  | NA |  | NA |  | NA | NA |  |
| 1.24555 | | 1.14059 | | 1.20903 | | 1.24272 | | 1.26064 | | 1.05716 | | NA | 1.51175 | |
| 1.08898 | | 1.17054 | | 1.12303 | | 1.08195 | | 1.12891 | | 1.18266 | | NA |  | 1 |
| NA |  | 0.884467 | | 0.909784 | | 0.937264 | | 0.988757 | | 1.02983 | | NA | NA |  |
| 1.04841 | | 1.05739 | | 1.06452 | | 1.03834 | | 1.10828 | | 1.14073 | | NA | 1.66667 | |
| NA |  | 0.873684 | | 0.922138 | | NA |  | 0.942715 | | NA |  | NA | NA |  |
| NA |  | 1.02898 | | 1.02276 | | 1.01433 | | 1.04224 | | NA |  | NA | NA |  |
| NA |  | 1.00753 | | 1.01248 | | 1.02955 | | 1.01396 | | 1.01836 | | NA | 0.951675 | |
| NA |  | NA |  | NA |  | NA |  | NA |  | NA |  | NA | NA |  |
| 1.08989 | | 1.05455 | | 1.03079 | | 1.10506 | | 1.07108 | | 1.22919 | | NA | 1.57895 | |
| 0.975082 | | 0.970663 | | 0.983116 | | 0.999404 | | 1.03071 | | 1.03574 | | NA | 1.85714 | |
| NA |  | NA |  | NA |  | NA |  | NA |  | NA |  | NA | 1.11111 | |
| NA |  | NA |  | NA |  | NA |  | NA |  | NA |  | NA | 0.730769 | |
| 0.982506 | | 1.01218 | | 1.04053 | | 1.04189 | | 1.03517 | | NA |  | NA | 0.71875 | |
| 0.999812 | | 1.01464 | | 1.05769 | | 1.03268 | | 0.97965 | | NA |  | NA | 0.705882 | |
| 1.00084 | | 1.03058 | | 0.993735 | | 1.03304 | | 1.04539 | | 1.03015 | | 0.666667 | 0.952381 | |
| 1.05691 | | 1.02973 | | 1.05788 | | 1.04686 | | 1.08981 | | 1.00981 | | 0.785714 | 0.913793 | |
| 0.993088 | | 0.949575 | | 1.00772 | |  | 1.005 | 0.990472 | | NA |  | NA | NA |  |
| 1.12602 | | 1.07738 | | 1.12546 | |  | 1.0995 | 1.10333 | |  | 1.145 | NA | NA |  |
| 0.990605 | | 1.03506 | | 1.00989 | | 1.00148 | | 1.06047 | | 1.06942 | | NA | 0.731707 | |
| 1.23757 | | 1.17899 | | 1.09916 | | 1.09763 | | 1.13562 | | 1.13739 | | NA | 1.81818 | |
| 1.02528 | | 1.02408 | | 1.08896 | | 1.06762 | | 1.03321 | | 1.06078 | | NA |  | 1.4 |
| 0.97753 | | 0.975626 | | 0.975167 | | 1.00449 | | 0.975156 | | 1.06645 | | NA | 1.04545 | |
| 1.14679 | | 1.11468 | | 1.15514 | | 1.09526 | | 1.14906 | | NA |  | NA | 0.894295 | |
|  | 1.0163 | 1.04521 | | 1.02087 | | 1.03733 | | 1.02115 | |  | 1.0188 | NA | NA |  |
| 0.974609 | | 1.00856 | | 0.978655 | | 1.03434 | | 1.02383 | | 0.982384 | | NA | NA |  |
| 0.967635 | | 1.03276 | | 1.04087 | | 1.01924 | | 1.09905 | | 1.06794 | | NA | NA |  |
| 1.15446 | | 1.07406 | | 1.04944 | | 1.11412 | | 1.06159 | | 1.05349 | | NA | 0.797743 | |

| 1.11559 | |  | 1.0431 | 0.990476 | |  | 1.0168 | 1.08959 | | 1.04679 | | NA | NA |  |
| --- | --- | --- | --- | --- | --- | --- | --- | --- | --- | --- | --- | --- | --- | --- |
| 0.970687 | | 1.07216 | | 1.03504 | | 1.04211 | | 1.00823 | | 1.03173 | | NA | 1.26316 | |
| 1.13453 | | 1.17789 | | 1.07549 | | 1.11683 | |  | 1.068 | 1.17304 | | NA | 1.06061 | |
| 1.04805 | | 1.00121 | | 1.06401 | | 1.05083 | | 1.05302 | | 0.979023 | | NA | 0.826087 | |
| 1.27913 | | 1.20395 | |  | 1.2544 | 1.24424 | | 1.32283 | |  | 1.2589 | NA | NA |  |
| 0.993229 | | NA |  | NA |  | NA |  | 1.03135 | | 1.15535 | | NA | 0.973616 | |
| 0.969751 | | 0.966211 | | 0.984049 | | 0.984141 | | 1.00292 | | 0.999184 | | NA | 0.989677 | |
|  | 0.9724 | 0.985794 | | 0.990897 | | 0.981334 | | 0.997557 | | 0.976055 | | NA | 0.977042 | |
|  | 1.1003 | 1.04975 | | 1.01432 | | 1.09665 | | 1.08095 | | 1.10628 | | NA | 0.703704 | |
| 1.05212 | | 1.07009 | | 0.986486 | | 1.05383 | | 1.01668 | | 1.10644 | | NA | 1.11111 | |
| 0.956839 | | 0.979365 | | 0.987609 | | 0.949739 | | 0.961127 | | 0.979983 | | NA |  | 0.56 |
| NA |  | NA |  | NA |  | NA |  | NA |  | NA |  | NA | NA |  |
| 0.979703 | | 1.06134 | | 1.05099 | | 1.01775 | |  | 1.0605 | NA |  | NA |  | 1 |
| 1.10786 | | 1.02082 | | 1.09134 | |  | 1.0461 | 1.04104 | | NA |  | NA | NA |  |
| 1.11681 | | 1.10005 | | 1.09089 | |  | 1.0505 | 1.14888 | | 1.01211 | | NA | NA |  |
| 1.04779 | | 1.03939 | | 1.05062 | | 1.04389 | | 1.04492 | | 1.01501 | | NA | 1.14878 | |
| NA |  | 1.06182 | | 1.09251 | | 1.05117 | | 1.03015 | | 1.05492 | | NA | 1.28814 | |
| 1.06166 | | 1.03748 | | 1.07246 | | 1.02297 | | 1.05979 | | 0.960537 | | NA |  | 1.25 |
| 1.06711 | | NA |  | NA |  | NA |  | 0.996361 | | 0.98939 | | NA | 0.856435 | |
| 1.00361 | | 1.01886 | |  | 1 | 0.984371 | | 1.02184 | | 1.01958 | | NA |  | 0.92 |
| 1.02136 | | 1.05328 | | 1.06995 | |  | 1.0852 | 1.06722 | | 1.04273 | | NA | 1.33333 | |
| 1.03298 | | 0.975742 | | 1.00129 | |  | 1.0283 | 1.05246 | | NA |  | NA | NA |  |
| 0.958224 | | 0.989729 | | 1.03756 | | 1.03753 | | 1.02771 | | 0.987171 | | NA | 0.913043 | |
| 1.01875 | | 1.01819 | | 1.02542 | | 1.01685 | | 1.00576 | | NA |  | NA | NA |  |
| 1.02063 | | 1.03764 | | 0.999751 | | 1.01628 | | 0.998233 | | 0.979705 | | NA | 0.964507 | |
| 1.14775 | | 1.13172 | | 1.14117 | | 1.08114 | | 1.16568 | | 1.08141 | | NA | NA |  |
| 1.01476 | | 1.07914 | | 1.05328 | |  | 1.105 | 1.06754 | | 1.06384 | | NA | 0.965217 | |
| 1.03257 | | 1.06577 | | 1.03525 | | 1.02614 | | 1.04562 | | NA |  | NA | 0.849817 | |
| 0.907066 | | 1.04053 | | 0.977231 | | 1.05006 | | 0.991204 | | 0.99425 | | NA | 1.90831 | |
| 0.943498 | | 1.03323 | | 0.951919 | | 1.04541 | | 1.01493 | |  | 1.0229 | NA | 1.22222 | |
| 1.01068 | | 1.01087 | | 1.03628 | | 1.01088 | | 1.00483 | | 0.993622 | | NA | NA |  |
|  | 1.3292 | 1.18366 | | 1.22983 | | 1.25588 | | 1.22948 | | 1.14471 | | NA | 0.872363 | |
| 1.04143 | | 1.00972 | | 1.03158 | | 1.03448 | | 1.03292 | | 1.03652 | | NA | 1.15217 | |
| 1.01108 | | 1.00537 | | 1.04925 | | 1.00365 | | 1.02764 | | 1.00018 | | NA | NA |  |
| NA |  | NA |  | NA |  | NA |  | 1.08054 | | NA |  | NA | 0.82872 | |
| 1.00173 | | 1.10212 | | 1.03403 | | 0.990638 | | 1.06039 | | 1.06031 | | NA | 0.794872 | |
| 1.02924 | | 1.01055 | | 1.06007 | | 1.07015 | | 0.966565 | | 1.01342 | | NA | 2.28571 | |
| 1.06222 | | 1.05529 | | 1.02386 | | 1.04391 | | 1.13767 | | 0.997996 | | NA | 0.782609 | |

hesc_deriv immature_ immature_ immature_ immature_ immature_ inflammat ips..ceehrc

1	1.08752	1.09987	1.11442	1.12391	1.0931	1.13358	2

NA	NA	NA	NA	NA	NA	NA	NA NA

| 1.12377 | 1.17629 | 1.18241 | 1.15484 | 1.24266 | 1.09441 NA |
| --- | --- | --- | --- | --- | --- |

NA	1.14558	1.17225	1.13419	1.11389	1.09242	1.17867	1.22538

NA	1.11114	1.16911	1.13279	1.13245	1.19237	1.09129 NA NA	0.935978 0.932419 0.912832 0.921062 0.875644 0.944475 NA NA	1.08712	1.07017	1.04428	1.04972	1.11332	1.09048 NA NA	1.08615	1.04074	1.07929	1.12874	1.02095 0.993491 NA NA	1.08615	1.04074	1.07929	1.12874	1.02095 0.993491 NA NA	1.2023 NA	NA	NA	NA	1.20833 NA NA	0.992042	0.9384 0.927286 0.964114 0.948216	1.00485 NA NA	1.01852	1.04258	1.04415	1.05241	1.00178	1.02205 NA

0.666667	1.03636	1.00992	1.02254 0.974786	1.02364	1.04574 NA

0.775	1.05543 0.981053 0.975472	1.00945	1.00217	1.06143 NA NA	NA	NA	NA	NA	NA	NA	NA NA	NA	NA	NA	NA	NA	NA	NA

1.125 0.975203 NA	NA	NA	NA	NA	NA

1.125 0.975203 NA	NA	NA	NA	NA	NA NA	1.01182 0.972094 0.947982 0.960031 0.985302 0.955417 NA

| NA | 1.13266 | 1.1755 | 1.19626 | 1.16471 | 1.19554 | 1.0086 NA |
| --- | --- | --- | --- | --- | --- | --- |

NA	NA	NA	NA	NA	NA	NA	NA NA	1.0378 0.978422 0.944148	0.97613 0.994589	1.02008 NA NA	NA	NA	NA	NA	NA	NA	NA NA	NA	NA	NA	NA	NA	NA	NA NA	1.06629 NA	NA	NA	NA	1.05996 NA NA	1.07818 NA	NA	NA	NA	1.02074 NA NA	1.0829 NA	NA	NA	NA	1.00823 NA NA	1.08603 NA	NA	NA	NA	1.07964 NA NA	1.06602	1.15428	1.16801	1.17542	1.16857	1.03758 NA NA	1.06602	1.15428	1.16801	1.17542	1.16857	1.03758 NA

NA	1.15799	1.00585	1.04673	1.10931	1.05351	1.06358 0.941176

NA	1.19972	1.06878	1.06222	1.10269	1.10098	1.12577	1.66667

NA	1.14336 NA	NA	NA	NA	1.1256 NA NA	1.00355 NA	NA	NA	NA	0.894395 NA NA	NA	NA	NA	NA	NA	NA	NA NA	NA	NA	NA	NA	NA	NA	NA NA	NA	NA	NA	NA	NA	NA	NA

NA	1.05029	1.06554	1.05903	1.0591	1.11514	1.06988 0.777778

| NA |  | 0.908752 | | 0.975585 | | 0.918958 | | 0.954293 | | 0.951935 | | 1.02187 | | NA |  |
| --- | --- | --- | --- | --- | --- | --- | --- | --- | --- | --- | --- | --- | --- | --- | --- |
| NA |  | 0.908752 | | 0.975585 | | 0.918958 | | 0.954293 | | 0.951935 | | 1.02187 | | NA |  |
| NA |  | 0.908752 | | 0.975585 | | 0.918958 | | 0.954293 | | 0.951935 | | 1.02187 | | NA |  |
| NA |  | 1.01602 | | 1.00282 | | 1.02005 | | 1.01234 | |  | 1.0131 | 0.942058 | | 0.790794 | |
| NA |  |  | 1.0297 | 0.994301 | | 0.975737 | | 1.05372 | | 0.97657 | | 0.966509 | |  | 1.2 |
|  | 1.25 | 1.01866 | | 1.05149 | | 0.983397 | | 1.01945 | | 1.01052 | | 0.902788 | | NA |  |
|  | 1.5 |  | 1.0947 | 1.09622 | |  | 1.069 | 1.06419 | | 1.05015 | | 1.05006 | | NA |  |
| 1.16667 | | 1.06415 | |  | 1.0634 |  | 1.0145 | 1.02317 | | 1.04947 | | 1.07075 | | NA |  |
|  | 3 | 1.02858 | | 1.01323 | | 1.01202 | | 1.00714 | | 0.996435 | | 0.915202 | | NA |  |
|  | 0.75 | 1.09961 | | 1.13335 | | 1.04045 | | 1.07296 | | 1.07613 | |  | 1.0367 | NA |  |
| 0.833333 | | 1.12042 | | 1.14165 | | 1.08748 | | 1.11659 | | 1.13453 | | 1.06769 | | NA |  |
| 1.33333 | | 1.08346 | | 1.12959 | | 1.05825 | | 1.09066 | | 1.09245 | | 0.997802 | | NA |  |
|  | 1.5 | 1.12522 | | 1.15346 | |  | 1.1268 | 1.18008 | | 1.13689 | | 1.05813 | |  | 1 |
|  | 1 | 1.08558 | | 1.07407 | | 1.06433 | | 1.06452 | | 1.04529 | |  | 1.0468 | NA |  |
|  | 1.4 | 1.07588 | |  | 1.0562 | 1.02802 | | 1.03761 | | 1.02078 | | 1.03412 | | NA |  |
| NA |  | NA |  | NA |  | NA |  | NA |  | NA |  | NA |  | NA |  |
| NA |  |  | 1.0954 |  | 1.1392 | 1.14826 | | 1.15628 | | 1.22103 | | 0.969512 | | NA |  |
|  | 0.8 | 1.10819 | | 1.10515 | | 1.08792 | | 1.08497 | | 1.09639 | | 1.06007 | | NA |  |
|  | 0.9 | 1.12861 | | 1.11779 | | 1.07008 | | 1.10347 | | 1.09629 | | 0.995597 | | NA |  |
| NA |  | 1.16581 | | 1.07423 | | 1.10634 | | 1.23612 | | 1.14449 | | 1.00986 | | NA |  |
| 1.33333 | | 1.06769 | | 1.05341 | | 1.02317 | | 1.03307 | | 1.04161 | | 0.945569 | | NA |  |
| NA |  | NA |  | NA |  | NA |  | NA |  | NA |  | NA |  | NA |  |
| NA |  | NA |  | NA |  | NA |  | NA |  | NA |  | NA |  | NA |  |
| 0.777778 | | 1.09863 | | 1.04513 | | 1.03441 | | 1.03814 | | 1.04493 | | 1.04148 | | NA |  |
| NA |  | 1.05138 | | 1.12752 | | 1.01629 | | 1.14189 | | 1.12583 | | 1.20455 | | NA |  |
| NA |  | 1.08257 | | 1.10451 | | 1.04828 | | 1.12061 | | 1.04694 | | 1.03048 | | NA |  |
| 1.22222 | | 1.11117 | | 1.09173 | | 1.08935 | | 1.09175 | | 1.07667 | | 1.05431 | | NA |  |
| 0.444444 | | 1.09244 | | 1.07696 | | 1.04442 | | 1.03937 | | 1.07809 | | 1.01347 | | NA |  |
| 0.571429 | | 1.10747 | | 1.08591 | | 1.07867 | |  | 1.1197 | 1.10767 | | 1.03811 | | NA |  |
| 0.571429 | | 1.10747 | | 1.08591 | | 1.07867 | |  | 1.1197 | 1.10767 | | 1.03811 | | NA |  |
| 1.17647 | | 1.12247 | | 1.07997 | | 1.08378 | | 1.09318 | | 1.09315 | |  | 1.0385 | NA |  |
| 0.545455 | | 1.11571 | | 1.07116 | | 1.07708 | | 1.05958 | | 1.07238 | | 1.01937 | | NA |  |
| NA |  | 1.07241 | | 1.03989 | | 1.03606 | | 1.01995 | | 1.03882 | | 1.12391 | | NA |  |
| NA |  | 1.05436 | | 1.03902 | |  | 1.0255 | 1.07021 | | 0.996098 | | 0.969551 | | NA |  |
| NA |  | NA |  | NA |  | NA |  | NA |  | NA |  | NA |  | NA |  |
| NA |  | 1.04608 | | 1.04075 | | 1.03662 | | 1.00278 | |  | 1.0043 | 0.95506 | | NA |  |
| 0.484848 | | 1.11482 | | 1.10376 | | 1.03981 | | 1.05989 | | 1.05685 | | 1.04038 | | NA |  |
|  | 1 | 1.09735 | | 1.07592 | | 1.05741 | | 1.07279 | | 1.06417 | |  | 1.0065 | NA |  |
| NA |  | 1.15402 | | 1.15508 | | 1.19473 | | 1.26326 | |  | 1.2308 | 1.13892 | | NA |  |

| 0.666667 | | 1.02187 | | 0.997639 | 0.993849 |  | 1.0054 | 1.06046 | 0.991661 | NA |
| --- | --- | --- | --- | --- | --- | --- | --- | --- | --- | --- |
| 1.41667 | | 1.01849 | | 0.954834 | 0.933337 | 0.935881 | | 1.01677 | 0.917679 | NA |
|  | 0.625 | 1.00988 | | 1.01265 | 0.98799 |  | 1.0058 | 1.02211 | 0.968955 | NA |
|  | 1.5 | 1.03996 | | NA | 1.00765 | NA |  | NA | 1.02351 | NA |
| NA |  | NA |  | NA | NA | NA |  | NA | NA | NA |
| NA |  | NA |  | NA | NA | NA |  | NA | NA | NA |
| NA |  | NA |  | NA | NA | NA |  | NA | NA | NA |
| NA |  | NA |  | NA | NA | NA |  | NA | NA | NA |
| NA |  | NA |  | NA | NA | NA |  | NA | NA | NA |
| NA |  | NA |  | NA | NA | NA |  | NA | NA | NA |
| NA |  | NA |  | NA | NA | NA |  | NA | NA | NA |
| NA |  | NA |  | NA | NA | NA |  | NA | NA | NA |
| NA |  | NA |  | NA | NA | NA |  | NA | NA | NA |
| NA |  | NA |  | NA | NA | NA |  | NA | NA | NA |
| NA |  | NA |  | NA | NA | NA |  | NA | NA | NA |
| NA |  | 1.00766 | | NA | NA | NA |  | NA | NA | NA |
| NA |  | NA |  | NA | NA | NA |  | NA | NA | NA |
| NA |  | NA |  | NA | NA | NA |  | NA | NA | NA |
| NA |  | 1.02001 | | 1.04207 | 1.06907 | 1.02819 | | 1.09944 | NA | NA |
| NA |  | 1.02252 | | NA | NA | NA |  | NA | NA | NA |
| NA |  | NA |  | NA | NA | NA |  | NA | NA | NA |
|  | 1 |  | 1.1114 | 1.09518 | 1.09296 | 1.10215 | | 1.07822 | 1.02463 | 0.846154 |
|  | 1 | 0.951098 | | 0.961264 | 0.983662 | 0.951006 | | 0.95583 | NA | NA |
|  | 1 | 0.951098 | | 0.961264 | 0.983662 | 0.951006 | | 0.95583 | NA | NA |
|  | 1.5 | NA |  | NA | NA | NA |  | NA | NA | NA |
|  | 1.5 | NA |  | NA | NA | NA |  | NA | NA | NA |
|  | 1.126 | 0.976671 | | 0.978498 | 1.01177 | 0.982387 | | 1.00019 | NA | NA |
| 1.03472 | | 0.990106 | | 1.03792 | 1.02097 | 0.971884 | | 0.990763 | NA | NA |
|  | 1 | NA |  | NA | NA | NA |  | NA | NA | NA |
|  | 1.0496 | NA |  | NA | NA | NA |  | NA | NA | NA |
|  | 1.0496 | NA |  | NA | NA | NA |  | NA | NA | NA |
|  | 1.0496 | NA |  | NA | NA | NA |  | NA | NA | NA |
| NA |  | NA |  | NA | NA | NA |  | NA | NA | NA |
| NA |  | NA |  | NA | NA | NA |  | NA | NA | NA |
| NA |  | NA |  | NA | NA | NA |  | NA | NA | NA |
| NA |  | NA |  | NA | NA | NA |  | NA | NA | NA |
| NA |  | 0.981232 | | 0.985884 | 1.00023 | 0.976345 | | 0.938917 | 0.869052 | NA |
| NA |  | 1.07084 | | 1.11363 | 1.11159 | 1.11732 | | 1.07389 | 1.09348 | 0.708333 |
| NA |  | 1.07084 | | 1.11363 | 1.11159 | 1.11732 | | 1.07389 | 1.09348 | 0.708333 |

| NA | 1.14309 | 1.10947 |  | 1.0975 | 1.06863 | 1.07308 | | 1.11508 | 1.33667 |
| --- | --- | --- | --- | --- | --- | --- | --- | --- | --- |
| NA | NA | NA | NA |  | NA | NA |  | NA | NA |
| NA | 1.06014 | 1.03418 | 1.07966 | | 1.07284 | 1.04834 | | 1.12065 | 2.14286 |
| NA | NA | 0.926908 | 0.91423 | | 0.938103 | 0.909753 | | 0.977965 | NA |
| NA | 1.08593 | 0.988673 | 1.04773 | | 0.994425 | 0.969883 | | 1.08719 | NA |
| NA | 1.09337 | NA | NA |  | NA | NA |  | 0.976486 | NA |
| NA | NA | NA | NA |  | NA | NA |  | NA | NA |
| NA | NA | NA | NA |  | NA | NA |  | NA | NA |
| NA | 0.912485 | NA | NA |  | NA | NA |  | 0.899292 | NA |
| NA | 1.00939 | NA | NA |  | NA | NA |  | NA | NA |
| NA | 0.904383 | 0.944094 | 0.971843 | | 0.960872 | 0.912173 | | 0.869622 | NA |
| NA | NA | NA | NA |  | NA | NA |  | NA | NA |
| NA | NA | NA | NA |  | NA | NA |  | NA | NA |
| NA | NA | NA | NA |  | NA | NA |  | NA | NA |
| NA | NA | NA | NA |  | NA | NA |  | NA | NA |
| NA | NA | NA | NA |  | NA | NA |  | NA | NA |
| NA | NA | NA | NA |  | NA | NA |  | NA | NA |
| NA | NA | NA | NA |  | NA | NA |  | NA | NA |
| NA | NA | NA | NA |  | NA | NA |  | NA | NA |
| NA | 1.06786 | 1.05237 | 1.06677 | | 1.06844 | 1.03517 | | NA | NA |
| NA | NA | NA | NA |  | NA | NA |  | NA | NA |
| NA | NA | NA | NA |  | NA | NA |  | NA | NA |
| NA | NA | NA | NA |  | NA | NA |  | NA | NA |
| NA | NA | NA | NA |  | NA | NA |  | NA | NA |
| NA | NA | NA | NA |  | NA | NA |  | NA | NA |
| NA | 0.974219 | 0.970756 | 1.00441 | | 0.976496 | 0.98583 | | NA | NA |
| NA | NA | NA | NA |  | NA | NA |  | NA | NA |
| NA | NA | NA | NA |  | NA | NA |  | NA | NA |
| NA | NA | NA | NA |  | NA | NA |  | NA | NA |
| NA | NA | NA | NA |  | NA | NA |  | NA | NA |
| NA | 1.01132 | 0.972581 | 1.00207 | | 0.997012 | 0.942224 | | NA | NA |
| NA | NA | NA | NA |  | NA | NA |  | NA | NA |
| NA | NA | NA | NA |  | NA | NA |  | NA | NA |
| NA | NA | NA | NA |  | NA | NA |  | NA | NA |
| NA | NA | NA | NA |  | NA | NA |  | NA | NA |
| NA | NA | NA | NA |  | NA | NA |  | NA | NA |
| NA | NA | NA | NA |  | NA | NA |  | NA | NA |
| NA | NA | NA | NA |  | NA |  | 1.0484 | NA | NA |
| NA | NA | NA | NA |  | NA | NA |  | NA | NA |

| NA |  | NA |  | NA |  | NA |  | NA |  | NA |  | NA | NA |
| --- | --- | --- | --- | --- | --- | --- | --- | --- | --- | --- | --- | --- | --- |
| NA |  | 1.01546 | | 1.03004 | | 1.02165 | | 1.01766 | | 1.01658 | | NA | NA |
| NA |  | NA |  | NA |  | NA |  | NA |  | NA |  | NA | NA |
| NA |  | NA |  | NA |  | NA |  | NA |  | NA |  | NA | NA |
| NA |  | NA |  | NA |  | NA |  | NA |  | NA |  | NA | NA |
| NA |  | 1.09548 | | NA |  | 1.11808 | | 1.13653 | | 1.12484 | | NA | NA |
| NA |  | NA |  | NA |  | NA |  | NA |  | NA |  | NA | NA |
| NA |  | NA |  | NA |  | NA |  | NA |  | NA |  | NA | NA |
| NA |  | NA |  | NA |  | NA |  | NA |  | NA |  | NA | NA |
| NA |  | NA |  | NA |  | NA |  | NA |  | NA |  | NA | NA |
| NA |  | 1.00657 | | 0.996265 | | 0.954999 | | 1.00565 | | 0.982928 | | 0.923626 | NA |
| NA |  | 1.04902 | | 1.02637 | | 1.01244 | | 1.01443 | | 1.02511 | | NA | NA |
| NA |  |  | 1.002 | 0.99164 | | 0.976564 | | 1.01003 | | 0.967295 | | NA | NA |
| NA |  | 0.988771 | |  | 1.03 | 0.97862 | | 0.988542 | | 1.00815 | | 0.879197 | NA |
| NA |  | 1.13265 | | 1.01767 | | 1.04524 | | 1.01338 | | 0.986698 | | 1.16461 | NA |
| NA |  | 1.03529 | | 1.01601 | |  | 1.0094 | 1.02896 | | 1.00434 | | 1.01449 | NA |
| NA |  | 1.12532 | | NA |  | NA |  | NA |  | NA |  | 1.03447 | NA |
| NA |  | 1.02568 | | 0.945833 | | 0.934783 | | 0.989719 | | 1.00371 | | NA | NA |
| NA |  | 1.04175 | | 1.05202 | | 0.99334 | | 1.01284 | | 0.992243 | | 0.970797 | NA |
| NA |  | 1.12439 | | 1.14192 | | 1.11814 | | 1.04585 | | 1.04941 | | 1.06463 | NA |
| 0.675325 | | 1.01882 | | 1.02942 | | 1.00183 | |  | 1.0064 | 1.05326 | | 1.00828 | NA |
|  | 1.6 | 1.02938 | | 1.00151 | | 1.03761 | | 1.04225 | | 1.07514 | | 0.980439 | NA |
|  | 1.5 | 1.05232 | | 0.970514 | |  | 1.0071 |  | 1.0022 | 1.02577 | | 1.02747 | NA |
|  | 1.25 | 1.00388 | | NA |  | 0.991564 | | NA |  | NA |  | 0.977447 | NA |
| NA |  | 1.04969 | | 1.06994 | | 1.03107 | | 1.10593 | |  | 1.0519 | NA | NA |
| NA |  | 1.04279 | | 1.02912 | | 1.09001 | | 1.06762 | | 1.04535 | | 1.08075 | NA |
| NA |  | 1.02289 | | 0.964049 | | 1.02611 | | 1.10076 | | 1.00745 | | 1.05539 | 0.805556 |
| NA |  | 0.997586 | | 1.16868 | |  | 1.0969 |  | 1.1902 | 1.06968 | | 0.91623 | NA |
| NA |  | 0.997586 | | 1.16868 | |  | 1.0969 |  | 1.1902 | 1.06968 | | 0.91623 | NA |
| NA |  | 1.02493 | |  | 1.0223 | 1.04355 | | 1.01837 | | 0.995008 | | 1.01913 | 0.857143 |
| NA |  | 1.02128 | | 1.06606 | | 1.07711 | | 1.08447 | | 1.05217 | | 0.923739 | 1.21739 |
| NA |  | 1.06173 | | 1.05718 | | 1.03101 | | 1.08568 | | 1.06284 | | 0.993554 | NA |
| NA |  | 1.06173 | | 1.05718 | | 1.03101 | | 1.08568 | | 1.06284 | | 0.993554 | NA |
| NA |  | 1.06173 | | 1.05718 | | 1.03101 | | 1.08568 | | 1.06284 | | 0.993554 | NA |
| NA |  | 0.992857 | | 1.03465 | | 0.981348 | | 1.00403 | | 0.970476 | | 0.986526 | 0.869929 |
| NA |  | 0.978174 | | 1.03662 | | 1.05492 | | 1.04503 | | 1.06049 | | 0.963215 | 1.38898 |
| NA |  | NA |  | NA |  | NA |  | NA |  | NA |  | NA | NA |
| NA |  | NA |  | NA |  | NA |  | NA |  | NA |  | NA | NA |
|  | 0.625 | 1.11018 | | 1.10588 | | NA |  | 1.15661 | | NA |  | NA | NA |

| NA |  | NA | NA |  | NA | NA |  | NA | NA |  | NA |
| --- | --- | --- | --- | --- | --- | --- | --- | --- | --- | --- | --- |
| NA |  | NA | NA |  | NA | NA |  | NA | NA |  | NA |
| NA |  | NA | NA |  | NA | NA |  | NA | NA |  | NA |
| NA |  | NA | NA |  | NA | NA |  | NA | NA |  | NA |
| NA |  | 0.963962 | 0.92858 | | 0.907129 | 0.911928 | | 0.882885 | 1.01865 | | NA |
| NA |  | 0.983308 | 0.975348 | | 0.958755 | 0.977255 | | 0.988137 | 1.08837 | | NA |
| NA |  | 0.980145 | 1.03959 | | 0.984719 | 1.02429 | | 1.03305 | 0.984262 | | NA |
| NA |  | 1.03794 | 1.05003 | | 0.975235 | 1.02708 | | 0.989486 | 1.09203 | | NA |
| NA |  | 0.946856 | 0.929196 | | 0.902897 | 0.935123 | | 0.951227 | 0.926528 | | NA |
| NA |  | 1.01405 | 0.923805 | | 0.976727 | 0.949083 | | 0.954046 | 0.940429 | | NA |
| NA |  | 1.02944 | 1.10154 | | 1.07059 | 1.09999 | | 1.10223 | 1.04861 | | NA |
| NA |  | 1.09565 | 1.11166 | | 1.10842 | 1.10471 | | 1.12722 |  | 1.14 | 1.16667 |
|  | 1 | 1.16023 | 1.17984 | | 1.11337 | 1.17953 | | 1.14749 | 1.25214 | | 0.741635 |
| NA |  | 1.01327 | 1.01106 | | 0.951542 | 0.997658 | | 1.01048 | 0.928571 | | NA |
| NA |  | NA | NA |  | NA | NA |  | NA | NA |  | NA |
| NA |  | NA | NA |  | NA | NA |  | NA | NA |  | NA |
| NA |  | 1.07743 | 1.10738 | | 1.11486 | 1.09092 | | 1.08999 | 1.02749 | | NA |
| NA |  | NA | NA |  | NA | NA |  | NA | NA |  | NA |
| NA |  | 0.958422 | 0.928568 | | 0.938502 | 0.945858 | | 0.910916 |  | 1.0296 | NA |
| NA |  | NA | NA |  | NA | NA |  | NA | NA |  | NA |
| NA |  | NA | NA |  | NA | NA |  | NA | NA |  | NA |
| NA |  | 1.08087 | 1.20279 | | 1.17172 |  | 1.1936 | 1.14278 | 1.14337 | | NA |
| NA |  | 1.02168 | 1.16034 | | 1.10027 | 1.15913 | | 1.16249 | 1.06356 | | NA |
| NA |  | 1.02168 | 1.16034 | | 1.10027 | 1.15913 | | 1.16249 | 1.06356 | | NA |
| NA |  | 1.02168 | 1.16034 | | 1.10027 | 1.15913 | | 1.16249 | 1.06356 | | NA |
| NA |  | 1.02168 | 1.16034 | | 1.10027 | 1.15913 | | 1.16249 | 1.06356 | | NA |
| NA |  | 1.06786 |  | 1.0915 | 1.05734 | 1.11117 | | 1.01831 | 1.01786 | | NA |
| NA |  | 1.06786 |  | 1.0915 | 1.05734 | 1.11117 | | 1.01831 | 1.01786 | | NA |
| NA |  | 1.06786 |  | 1.0915 | 1.05734 | 1.11117 | | 1.01831 | 1.01786 | | NA |
| NA |  | 1.06786 |  | 1.0915 | 1.05734 | 1.11117 | | 1.01831 | 1.01786 | | NA |
| NA |  | 1.14437 | 1.17011 | | 1.15275 |  | 1.1325 | 1.12653 | 1.03581 | | NA |
| NA |  | 1.14437 | 1.17011 | | 1.15275 |  | 1.1325 | 1.12653 | 1.03581 | | NA |
| NA |  | 1.02608 | 1.01466 | | 1.03802 | 1.02837 | | 1.11018 | 0.960337 | | NA |
| NA |  | 1.02608 | 1.01466 | | 1.03802 | 1.02837 | | 1.11018 | 0.960337 | | NA |
| NA |  | 1.09203 | 1.19554 | | 1.15067 | 1.18403 | | 1.19624 | 1.07893 | | NA |
| NA |  | 0.929147 | 0.946733 | | 0.935185 | 0.944188 | | 0.868722 | 0.918118 | | NA |
| NA |  | 0.929147 | 0.946733 | | 0.935185 | 0.944188 | | 0.868722 | 0.918118 | | NA |
| NA |  | 0.988095 | 0.955606 | | 0.975845 | 0.962252 | | 0.915996 | 1.03926 | | NA |
| NA |  | 0.988095 | 0.955606 | | 0.975845 | 0.962252 | | 0.915996 | 1.03926 | | NA |

| NA |  | 0.957403 | | 0.966184 | | 0.922237 | | 0.933483 | 0.935862 | | 1.01966 | NA |
| --- | --- | --- | --- | --- | --- | --- | --- | --- | --- | --- | --- | --- |
| NA |  | 0.964555 | |  | 0.9375 | 0.967015 | | 0.940297 | 0.921936 | | 1.00042 | NA |
| NA |  | 1.00822 | | 0.978663 | | 0.956465 | | 1.01978 | 0.974876 | | 1.00557 | NA |
| NA |  | 1.01718 | | 1.04203 | | 1.05284 | | 1.04387 |  | 1.0387 | 1.00292 | NA |
|  | 0.5 | 0.986846 | | 0.944303 | | 0.987025 | | 0.940038 | 0.941305 | | 0.997844 | NA |
|  | 1.375 |  | 1.0078 | 1.02055 | | 1.02656 | | NA | NA |  | 0.987945 | NA |
|  | 1.2 | 1.01341 | | 0.971623 | | 0.98305 | | 0.935224 | 0.99687 | | 1.02786 | NA |
| NA |  | 0.991649 | | 1.02234 | | 1.07266 | | 0.99967 |  | 1.0278 | 0.973291 | NA |
| NA |  | 1.00294 | | 1.03011 | | 1.06451 | | 0.981526 | 1.04233 | | 1.03413 | NA |
| NA |  | NA |  | NA |  | NA |  | NA | NA |  | 1.00896 | NA |
| NA |  | 0.991688 | | 0.991508 | | 1.03907 | | 0.939879 | 0.976751 | | 0.952525 | NA |
| NA |  | 1.04421 | | 1.05677 | | 1.04281 | | 1.04762 | 1.03621 | | 1.03646 | NA |
| NA |  | NA |  | NA |  | NA |  | NA | NA |  | NA | NA |
| NA |  |  | 1.0431 | 1.08127 | |  | 1.0901 | 1.04673 | 1.07431 | | 1.00183 | NA |
| NA |  | NA |  | NA |  | NA |  | NA | NA |  | NA | NA |
| NA |  | NA |  | NA |  | NA |  | NA | NA |  | NA | NA |
| NA |  | NA |  | NA |  | NA |  | NA | NA |  | NA | NA |
| NA |  | 0.949481 | | 0.99749 | | 0.950801 | | 0.94927 | 0.999729 | | NA | NA |
| NA |  | NA |  | NA |  | NA |  | NA | NA |  | NA | NA |
| NA |  | NA |  | NA |  | NA |  | NA | NA |  | NA | NA |
| NA |  | NA |  | NA |  | NA |  | NA | NA |  | NA | NA |
| NA |  | NA |  | NA |  | NA |  | NA | NA |  | NA | NA |
| NA |  | NA |  | NA |  | NA |  | NA | NA |  | NA | NA |
| NA |  | 0.988665 | | 1.03789 | | 1.03561 | | 1.03146 | 1.00264 | | NA | NA |
| NA |  | 0.989231 | | 1.08371 | | 1.03323 | | 1.02402 | 1.07216 | | 0.943176 | NA |
| NA |  | NA |  | NA |  | NA |  | NA | NA |  | NA | NA |
| NA |  | NA |  | NA |  | NA |  | NA | NA |  | NA | NA |
| NA |  | NA |  | NA |  | NA |  | NA | NA |  | NA | NA |
| NA |  | 1.07938 | |  | 1.129 | 1.09157 | | 1.12632 | 1.14102 | | 1.09371 | NA |
| NA |  |  | 1.0471 | 0.992271 | | 1.00427 | | 1.02922 | 1.01255 | | 1.09275 | 0.928571 |
| NA |  |  | 1.0471 | 0.992271 | | 1.00427 | | 1.02922 | 1.01255 | | 1.09275 | 0.928571 |
| NA |  | NA |  | NA |  | NA |  | NA | NA |  | NA | NA |
| NA |  | NA |  | NA |  | NA |  | NA | NA |  | NA | NA |
| NA |  | NA |  | NA |  | NA |  | NA | NA |  | NA | NA |
| NA |  | 0.99542 | | 0.981381 | | 0.95903 | | 0.961959 | 0.998319 | | NA | NA |
| NA |  | 1.07631 | | 1.06556 | |  | 1.0168 | 1.01288 | 1.05769 | | 1.07002 | 0.384615 |
| NA |  |  | 1.0134 | NA |  | NA |  | NA | NA |  | NA | NA |
| NA |  | NA |  | NA |  | NA |  | NA | NA |  | NA | NA |
|  | 1.12 | 1.05564 | | NA |  | NA |  | NA | NA |  | NA | NA |

| NA |  | 0.976205 | 0.953968 | | 0.945619 | | 0.937596 | | NA |  | NA |  | NA |
| --- | --- | --- | --- | --- | --- | --- | --- | --- | --- | --- | --- | --- | --- |
| NA |  | NA | NA |  | NA |  | NA |  | NA |  | NA |  | NA |
| NA |  | NA | NA |  | NA |  | NA |  | NA |  | NA |  | NA |
| NA |  | NA | NA |  | NA |  | NA |  | NA |  | NA |  | NA |
| NA |  | NA | NA |  | NA |  | NA |  | NA |  | 0.988371 | | NA |
| 1.16667 | | 0.984869 | 0.984109 | | 0.957167 | | 0.962246 | | 0.972531 | | 1.03463 | | NA |
| NA |  | NA | NA |  | NA |  | NA |  | NA |  | NA |  | NA |
| NA |  | NA | NA |  | NA |  | NA |  | NA |  | NA |  | NA |
| NA |  | NA | NA |  | NA |  | NA |  | NA |  | NA |  | NA |
| NA |  | NA | NA |  | NA |  | NA |  | NA |  | NA |  | NA |
| NA |  | 1.01619 | 0.974939 | | 1.00342 | | 1.11586 | | 0.905576 | | 1.04046 | | NA |
| NA |  | 1.07676 | NA |  | NA |  | NA |  | NA |  | 1.12938 | | NA |
| 0.688776 | | 1.09151 | 1.07662 | | 1.08645 | | 1.04808 | |  | 1.0639 |  | 1.0909 | NA |
| NA |  | NA | NA |  | NA |  | NA |  | NA |  | NA |  | NA |
| NA |  | NA | NA |  | NA |  | NA |  | NA |  | 1.10222 | | NA |
| NA |  | 1.08951 | 0.995273 | |  | 1.0134 | 1.02759 | | 1.01416 | | NA |  | NA |
|  | 2 | 0.992449 | 1.06699 | | 1.08057 | | 1.02176 | | 1.04485 | | 1.05033 | | NA |
| NA |  | NA | NA |  | NA |  | NA |  | NA |  |  | 1.1039 | NA |
| NA |  | NA | NA |  | NA |  | NA |  | NA |  | NA |  | NA |
| NA |  | 0.982974 | 1.04086 | | 1.01957 | | 0.994439 | | 1.00154 | | 0.864853 | | 1.14074 |
| NA |  | 0.977379 |  | 1.0325 | 1.03661 | | 1.02304 | | 1.02074 | | 0.995122 | | 1.15754 |
| NA |  | 0.954574 | 1.00775 | | 0.942884 | | 0.935577 | | 0.969057 | | 0.994076 | | NA |
| NA |  | 0.94707 | 0.972951 | | 0.95579 | | 0.970145 | | 0.942452 | | 0.904867 | | NA |
| NA |  | 1.17526 |  | 1.2206 | 1.23968 | | 1.20708 | | 1.19775 | | 1.17652 | | NA |
| NA |  | 1.04887 | 1.03977 | | 1.02529 | | 0.99635 | |  | 1.0149 | 1.11154 | | NA |
| NA |  | 1.00866 | 0.984683 | | 0.922325 | | 0.911206 | | 0.893097 | | 1.08529 | | NA |
| NA |  | 1.03279 | 1.10432 | | 1.11096 | | 1.08097 | |  | 1.0545 | 1.00618 | | NA |
| NA |  | 1.02081 |  | 1.0816 | 1.05268 | | 1.07671 | | 1.03484 | | 1.02131 | | NA |
| NA |  | 1.07528 | 1.02986 | | 1.02571 | | 1.00966 | | 0.989596 | | 0.960938 | | NA |
| NA |  | 1.04774 | 1.05351 | | 1.10027 | | 1.05994 | | 1.04427 | | 0.981147 | | NA |
| NA |  | 1.05485 |  | 1.032 | 0.904412 | | 0.978022 | | 1.05147 | | 1.01316 | | NA |
| NA |  | 1.04519 | 1.10262 | | 1.10401 | | 1.07257 | | 1.03072 | | 1.00409 | | NA |
| NA |  | 0.974359 | 0.926407 | | 0.970852 | | 1.04479 | | 0.934959 | | 0.854489 | | NA |
| NA |  | 1.06859 | 1.05986 | | 1.04959 | | 1.04369 | | 1.01225 | | 1.02987 | | NA |
| NA |  | 0.996933 | 0.955078 | | 1.00391 | | 0.986538 | | 0.956044 | | 0.890411 | | NA |
| NA |  | 0.959079 | NA |  | 1.04061 | |  | 1.0314 | 1.02913 | | NA |  | NA |
| NA |  | NA | NA |  | NA |  | NA |  | NA |  | NA |  | NA |
| NA |  | NA | NA |  | NA |  | NA |  | NA |  | NA |  | NA |
| NA |  | NA | NA |  | NA |  | NA |  | NA |  | NA |  | NA |

| NA |  | 0.988345 | | 0.945097 | 0.934247 | | 0.966257 | | 0.971138 | | 0.946243 | | NA |  |
| --- | --- | --- | --- | --- | --- | --- | --- | --- | --- | --- | --- | --- | --- | --- |
| NA |  | 0.97926 | | 0.936887 | 0.955773 | | 0.989864 | | 0.984927 | | 0.914381 | | NA |  |
| NA |  | 1.11611 | | 1.01097 |  | 1.0357 | 1.10772 | | 1.07896 | | 1.08828 | | NA |  |
| NA |  | 0.992131 | | 1.00494 | 1.01277 | | 0.984227 | | 0.967858 | | 0.885488 | | NA |  |
| NA |  | NA |  | NA | NA |  | NA |  | NA |  | NA |  | NA |  |
| NA |  | NA |  | NA | NA |  | NA |  | NA |  | NA |  | NA |  |
| NA |  | NA |  | NA | NA |  | NA |  | NA |  | NA |  | NA |  |
| NA |  | 1.01399 | | NA | 1.09704 | | 1.17194 | |  | 1.1811 | NA |  | NA |  |
| NA |  | 1.03437 | | 1.05796 | 1.13776 | | 1.08134 | | 1.10262 | |  | 1.1911 | NA |  |
|  | 1.8 | 1.06392 | | 1.06002 | 1.09386 | | 0.995975 | | 1.05849 | | 1.10326 | | NA |  |
| 1.09091 | | 0.997529 | | 1.03835 | 1.09375 | | 1.03477 | | 1.03609 | | 1.03375 | | NA |  |
| 1.09091 | | 0.997529 | | 1.03835 | 1.09375 | | 1.03477 | | 1.03609 | | 1.03375 | | NA |  |
| 0.631579 | | 0.998407 | | 1.05957 | 1.04956 | | 1.03982 | | 1.04328 | | 1.08453 | | NA |  |
| 1.55882 | | 0.983481 | | 0.90746 | 0.94483 | | NA |  | NA |  | 0.986002 | | NA |  |
| 1.55882 | | 0.983481 | | 0.90746 | 0.94483 | | NA |  | NA |  | 0.986002 | | NA |  |
| NA |  | 0.973587 | | 0.97391 | NA |  | 0.961414 | | NA |  | NA |  | NA |  |
| NA |  | 0.94977 | | NA | NA |  | NA |  | NA |  | NA |  | NA |  |
| NA |  | 0.96793 | | NA | NA |  | NA |  | NA |  | NA |  | NA |  |
| NA |  | 1.05188 | | 1.10017 | 1.09885 | | 1.11185 | | 1.08479 | | 0.978708 | | 0.956522 | |
| 0.777778 | | 1.10382 | | 1.09114 | 1.01756 | |  | 1.0447 | 1.06928 | | 1.02772 | | NA |  |
| NA |  | 1.11241 | | 1.06684 | 1.05549 | | 1.01802 | | 1.01766 | | 1.07417 | | NA |  |
| NA |  | 1.03976 | | 0.989908 | 0.998746 | | 0.94123 | | 1.02317 | | 1.02097 | | NA |  |
| 1.96296 | | 1.04887 | | 1.00605 |  | 1.0048 | 1.00737 | | 0.993881 | | 0.961213 | | NA |  |
| NA |  | 1.08625 | | 1.01532 | 1.01932 | | 0.990545 | | 1.01879 | |  | 1.0058 | NA |  |
| NA |  | 1.09354 | | 1.03394 | 1.01494 | | 1.02374 | |  | 1.0159 | 1.02273 | | NA |  |
|  | 1.2 |  | 1.0644 | 1.02192 | 1.06717 | | 1.04281 | | 1.03654 | | 0.972454 | | NA |  |
| NA |  | 1.00425 | | 1.04687 | 0.980903 | | 0.998507 | | 0.992344 | | 0.886498 | | NA |  |
| NA |  | 1.07572 | | 1.08277 | 1.05511 | | 1.05865 | | 1.04836 | | 1.07298 | | NA |  |
| NA |  | 0.934524 | | 0.954028 | 0.929785 | | 0.91448 | | 0.934609 | | 0.876336 | | NA |  |
| NA |  | 1.03535 | | 1.02798 | 1.02283 | | 0.997663 | | 0.96969 | | 1.07367 | |  | 0.4375 |
| NA |  | 0.927065 | | 0.878195 | 0.912831 | | 0.95564 | | 0.912672 | | 0.938773 | | NA |  |
| NA |  | NA |  | 0.947678 | 0.931871 | | 0.926267 | | 0.89955 | | NA |  | NA |  |
| NA |  | NA |  | NA | NA |  | NA |  | NA |  | NA |  | NA |  |
|  | 1.625 | NA |  | NA | NA |  | NA |  | NA |  | NA |  | NA |  |
|  | 0.875 | NA |  | NA | NA |  | NA |  | NA |  | NA |  | NA |  |
| NA |  | NA |  | 1.04055 | 1.03294 | |  | 1.0158 | 1.01169 | | 1.08732 | | 1.23385 | |
| NA |  | 1.04379 | | 1.02874 | 0.99347 | | 1.01616 | | 1.01409 | | 1.02509 | |  | 1.1 |
| NA |  | 0.980521 | | 0.981324 | 1.05496 | |  | 1.0772 | 0.987797 | | 1.01239 | | 1.58333 | |
| NA |  | NA |  | NA | NA |  | NA |  | NA |  | NA |  | 1.20833 | |

| NA | 1.03881 | |  | 1.0343 | 1.02183 | |  | 1.0217 | 0.978509 | | 1.05533 | | 1.38462 | |
| --- | --- | --- | --- | --- | --- | --- | --- | --- | --- | --- | --- | --- | --- | --- |
| NA | 0.949412 | | 0.901706 | | 0.904001 | | 0.868193 | | 0.858609 | | 0.956356 | | NA |  |
| NA | 1.09558 | | 1.15033 | | 1.16669 | | 1.10577 | | 1.15198 | | 1.08465 | | NA |  |
| NA | 1.06172 | | 1.08343 | | 1.07446 | |  | 1.027 | 1.13065 | | 0.977735 | | NA |  |
| NA | 1.02803 | | 1.07049 | | 1.06702 | | 1.02855 | | 1.09982 | | 0.992942 | | NA |  |
| NA | 1.06239 | | 0.988966 | | 0.957377 | | 1.04874 | | 0.991051 | | 1.02579 | | NA |  |
| NA | NA |  | 0.969343 | | 0.989926 | | 0.934805 | | 0.971085 | | 0.909528 | | NA |  |
| NA |  | 1.013 | 1.04028 | | 1.03448 | | 1.01302 | | 1.09021 | | 1.00518 | | NA |  |
| NA | 1.08019 | | 1.08884 | | 1.11128 | | 1.11879 | | 1.12628 | | 1.04141 | | NA |  |
| NA | NA |  | NA |  | NA |  | NA |  | NA |  | NA |  | NA |  |
| NA | NA |  | NA |  | NA |  | NA |  | NA |  | NA |  | NA |  |
| NA | NA |  | NA |  | NA |  | NA |  | NA |  | NA |  | NA |  |
| NA | 1.19696 | | 1.27361 | | 1.20901 | | 1.20216 | | 1.22699 | | 1.26759 | | 1.84125 | |
| NA | 1.12012 | | 1.21871 | | 1.17528 | | 1.11267 | | 1.16133 | | 1.11942 | | 0.888889 | |
| NA | NA |  | 0.956488 | | 0.898831 | | 0.913592 | | 0.93876 | | 0.98477 | | NA |  |
| NA | 1.02889 | | 1.09231 | | 1.02193 | | 1.07866 | | 1.04497 | | 0.94243 | | 0.794118 | |
| NA | 0.878959 | | 0.934602 | | 0.897815 | | 0.924957 | | 0.922903 | | 0.939201 | | NA |  |
| NA | NA |  | 1.02256 | | 1.01087 | | 1.02584 | | 1.01499 | | 0.921705 | | 1.56863 | |
| NA | 1.01384 | |  | 1.004 |  | 1.0202 | 1.01994 | | 1.01954 | | 1.01759 | | 0.941453 | |
| NA | NA |  | NA |  | NA |  | NA |  | NA |  | NA |  | NA |  |
| NA |  | 1.0997 | 1.06403 | | 1.08511 | | 1.05899 | | 1.08463 | | 1.06788 | | 0.470588 | |
| NA | 1.01785 | |  | 1 | 1.01832 | | 0.963675 | | 0.965446 | | NA |  | NA |  |
| NA | NA |  | NA |  | NA |  | NA |  | NA |  | 1.07281 | | 0.666667 | |
| NA | NA |  | NA |  | NA |  | NA |  | NA |  | 0.930703 | | 1.07692 | |
| NA | 1.02341 | | 0.973394 | | 0.979815 | | 1.04174 | |  | 1.0233 | 0.992695 | | 0.896552 | |
| NA | 0.969414 | | 0.979106 | | 1.01108 | | 1.06789 | | 0.985417 | | 0.964748 | |  | 1.24 |
| NA | 0.983277 | | 0.982494 | | 0.996183 | | 1.09125 | | 1.06897 | | 1.05829 | | 1.29167 | |
| NA | 1.02008 | |  | 1.0943 |  | 1.0627 | 1.13922 | | 1.10144 | | 0.989488 | |  | 1 |
| NA | 0.984563 | | 0.983023 | | 0.969353 | | 0.939675 | | 0.927917 | | 1.04986 | | NA |  |
| NA | 1.09113 | | 1.07472 | | 1.09891 | |  | 1.0889 | 1.08754 | | 1.05156 | | NA |  |
| NA |  | 1.0131 | 1.03216 | |  | 1.0528 | 1.00312 | | 1.01253 | | 1.01839 | | 0.928571 | |
| NA | 1.12285 | | 1.15891 | | 1.13497 | |  | 1.1567 |  | 1.146 | 1.07641 | |  | 1.4 |
| NA | 1.04367 | | 1.01532 | | 1.01194 | | 1.01457 | | 1.03601 | |  | 1.1505 | NA |  |
| NA | 0.976517 | | 0.975137 | | 1.00321 | | 0.928808 | | 0.981002 | | 0.974225 | | NA |  |
| NA |  | 1.1391 | 1.11659 | | 1.17596 | | 1.08551 | | 1.17046 | | 1.17678 | |  | 1.3648 |
| NA | 1.02465 | | 1.02868 | | 1.02228 | | 1.02943 | | 1.03093 | | 0.986403 | | NA |  |
| NA | 1.01823 | | 1.01985 | | 0.998839 | | 0.985134 | | 1.04183 | | 1.01652 | | NA |  |
| NA | 1.01344 | | 1.00257 | | 1.04231 | | 1.04151 | | 1.03783 | | 0.905063 | | NA |  |
| NA | 1.08927 | | 1.06566 | | 1.08111 | | 1.03801 | | 1.02599 | | 1.05385 | | 0.943844 | |

| NA |  | 1.02918 | | 1.05249 | | 1.07317 | | 1.04859 | | 1.01728 | | 1.00408 | | NA |  |
| --- | --- | --- | --- | --- | --- | --- | --- | --- | --- | --- | --- | --- | --- | --- | --- |
|  | 0.625 | 0.991078 | | 1.10918 | | 1.05392 | | 1.04644 | | 1.03504 | | 0.939542 | | NA |  |
| NA |  | 1.15993 | |  | 1.1033 | 1.14325 | | 1.08574 | | 1.09156 | | 1.07571 | | NA |  |
| NA |  | 1.04116 | | 0.928526 | | 1.04088 | |  | 1.0086 | 1.00031 | | 1.00154 | | NA |  |
| NA |  | 1.21093 | | 1.20057 | | 1.26121 | | 1.27282 | | 1.24721 | | 1.14966 | | NA |  |
| NA |  | NA |  | NA |  | NA |  | NA |  | NA |  | NA |  | NA |  |
| NA |  | 0.986246 | | 0.977824 | | 0.980711 | | 0.987084 | | 0.977546 | | 1.02664 | | NA |  |
| NA |  | 0.99991 | | 0.98684 | | 0.979009 | | 0.979352 | | 0.985839 | | NA |  | NA |  |
| NA |  | 1.11761 | | 1.01749 | |  | 1.0574 | 1.08661 | | 1.07877 | | 1.07636 | | 0.777778 | |
| NA |  | 1.03539 | | 1.00898 | | 1.04878 | | 1.03802 | | 1.06225 | |  | 1.05 | NA |  |
| NA |  | 0.995754 | | 0.97121 | | 0.991643 | | 1.01907 | | 1.03433 | | 0.985456 | | NA |  |
| NA |  | NA |  | NA |  | NA |  | NA |  | NA |  | NA |  | NA |  |
| NA |  | 0.990386 | | 0.998329 | | 0.983374 | | 1.01195 | |  | 1.0171 | 1.07129 | | NA |  |
| NA |  |  | 1.0762 | 1.07992 | | 1.07378 | | 1.07182 | | 1.04786 | | 1.03727 | | 2.25447 | |
| NA |  |  | 1.063 | 1.06068 | | 1.12273 | | 1.09276 | | 1.04197 | | 1.03051 | | 1.75479 | |
| NA |  | 1.04991 | | 1.04454 | | 1.06903 | | 1.06272 | | 1.04192 | | 1.07733 | | 0.981586 | |
| NA |  | NA |  | 1.08029 | | 1.04261 | | 1.06102 | | 1.08919 | | 1.25259 | | NA |  |
| NA |  | 1.00886 | | 1.06182 | | 1.08011 | | 1.09763 | | 1.01524 | | 1.07276 | | NA |  |
| NA |  | NA |  | NA |  | NA |  | NA |  | NA |  | NA |  | NA |  |
| NA |  | 1.05108 | | 1.01831 | | 1.03534 | | 1.10463 | | 1.07146 | |  | 1.088 | NA |  |
| NA |  | 1.03965 | | 1.05023 | | 1.02329 | | 1.04248 | | 1.08883 | | 1.02778 | | NA |  |
| NA |  | 0.991551 | | 0.933657 | | 0.943542 | | 0.972462 | | 0.905377 | | NA |  | NA |  |
| NA |  | 1.01465 | | 1.00491 | | 1.01311 | | 1.04196 | | 1.03653 | | 0.957136 | | NA |  |
| NA |  | 1.00218 | | 0.978213 | | 0.981309 | | 0.994235 | | 0.99176 | | 0.993677 | | NA |  |
| NA |  | 0.993182 | | 1.00268 | | 1.01089 | | 1.02524 | | 1.00117 | | 1.02127 | | NA |  |
| NA |  | 1.12524 | | 1.14043 | | 1.15732 | | 1.12818 | | 1.18136 | | 1.07235 | | NA |  |
| NA |  | 1.01136 | | 1.04738 | | 1.01224 | | 1.04758 | | 1.05497 | | 1.01519 | | NA |  |
| NA |  | 1.03353 | | 1.03564 | | 1.04567 | | 1.02705 | | 1.08685 | | 0.975809 | | 1.13784 | |
| NA |  | 1.03707 | | 0.979114 | | 0.996585 | | 1.01922 | | 0.999862 | | 1.02852 | | NA |  |
| NA |  | 0.964309 | | 0.936854 | | 0.965043 | | 0.944086 | | 0.956045 | | 1.06821 | | NA |  |
| NA |  | 1.03789 | | 1.02909 | | 1.02487 | | 1.02315 | | 1.02295 | | 0.966982 | | NA |  |
| NA |  | 1.19241 | | 1.21571 | | 1.22724 | | 1.20705 | | 1.20315 | | 1.16663 | | 1.15591 | |
| 1.33333 | | 0.989143 | | 0.933494 | |  | 1.0046 | 1.02144 | | 0.968276 | | 0.996323 | | 0.424242 | |
| NA |  | 1.02811 | | 1.00026 | | 1.01732 | | 1.08676 | | 1.03488 | | 1.03714 | | NA |  |
| NA |  | NA |  | NA |  | NA |  | NA |  | NA |  | NA |  | NA |  |
| NA |  |  | 1.0462 |  | 1.0669 | 1.11434 | | 1.01243 | | 1.06301 | | 1.16486 | | NA |  |
| NA |  | 1.05035 | | 1.05521 | | 1.06096 | |  | 1.0662 |  | 1.116 | 0.969072 | | NA |  |
| NA |  | 1.07144 | | 1.03297 | | 1.05972 | | 1.06022 | | 1.01517 | | 1.05709 | |  | 1.3913 |

| ips..knih | | islet_cell.. | | kidney..ce | large_inte | | large_inte | | large_inte | liver..crest | | macropha | |
| --- | --- | --- | --- | --- | --- | --- | --- | --- | --- | --- | --- | --- | --- |
|  | 1.25 |  | 1.1104 | 1.11613 | 1.12674 | | 1.22486 | | 1.13913 | 1.14113 | | 1.10874 | |
| NA |  | 1.00166 | | NA | NA |  | 0.995959 | | NA | NA |  | NA |  |
| NA |  | 1.12465 | | 1.09474 | 1.25623 | | 1.28027 | | 1.24225 |  | 1.175 | 1.08227 | |
| 1.65634 | | 1.10143 | | 0.995526 | 1.28282 | | 1.36218 | | 1.31186 | 1.20748 | | 1.14001 | |
| NA |  | 1.11386 | | 1.10607 | 1.30519 | |  | 1.2848 | 1.26069 | 1.14155 | | 1.17677 | |
| 0.722222 | | 0.955768 | | 0.882212 | 0.962219 | | 0.975522 | | 0.976117 | 0.966737 | | 0.963779 | |
| NA |  | 1.16576 | | 1.07518 | 1.26272 | | 1.22325 | | 1.23273 | 1.19042 | | 1.18236 | |
| 1.52083 | |  | 1.0631 | 1.10845 | 1.26376 | | 1.20238 | | 1.16968 | 1.10859 | | 1.04072 | |
| 1.52083 | |  | 1.0631 | 1.10845 | 1.26376 | | 1.20238 | | 1.16968 | 1.10859 | | 1.04072 | |
| NA |  |  | 1.1476 | 1.31771 | 1.24823 | | 1.33904 | | 1.29345 | 1.25961 | |  | 1.4877 |
| NA |  | 0.948767 | | 0.959942 | 0.969215 | | 0.962209 | | 0.975009 | 0.942193 | | 0.955353 | |
| NA |  | 0.980482 | | 0.94533 | 0.964251 | | 1.01126 | | 0.976958 | 0.982461 | | 1.03989 | |
| NA |  | 1.05786 | | 1.00284 | 1.06378 | | 1.01715 | | 1.03604 | 1.01346 | | 1.00849 | |
| NA |  | 1.07163 | | 1.02707 |  | 1.0873 | 1.09086 | | 1.06926 | 1.03798 | | 1.00645 | |
| NA |  | 1.09952 | | NA | 1.22747 | | 1.19089 | | 1.00706 | NA |  | NA |  |
| NA |  | 0.973439 | | NA | 0.98012 | | 0.99051 | | 0.994455 | NA |  | NA |  |
| NA |  | 0.976278 | | 0.873593 | 0.94499 | | 0.993549 | | 0.950802 | 0.958825 | | NA |  |
| NA |  | 0.976278 | | 0.873593 | 0.94499 | | 0.993549 | | 0.950802 | 0.958825 | | NA |  |
| NA |  | 0.917702 | | 0.920057 | 0.967405 | | 0.905398 | | 0.882375 | 0.913924 | | 0.85198 | |
| NA |  |  | 1.1411 | 1.17272 | 1.33816 | | 1.33958 | | 1.28512 | 1.23246 | | 1.06437 | |
| NA |  | 0.978323 | | NA | 0.979412 | | 0.991855 | | 0.995702 | NA |  | NA |  |
| NA |  | 0.960104 | | 1.02349 | 0.93214 | | NA |  | 0.928868 | 0.933999 | |  | 1.0874 |
| NA |  | NA |  | NA | 1.00507 | | 1.02705 | | 1.02334 | NA |  | NA |  |
| NA |  | NA |  | 0.942004 | 1.04523 | | 1.01749 | | 1.00323 | NA |  | NA |  |
| NA |  | 0.970529 | | NA | 1.07405 | |  | 1.0502 | 1.09446 | 0.982786 | | 1.07443 | |
| NA |  | NA |  | NA |  | 0.9468 | 0.957858 | | NA | 0.993277 | | 1.02714 | |
| NA |  | NA |  | NA | 0.943754 | | 0.984556 | | NA | 1.02462 | | 1.04441 | |
| NA |  | NA |  | NA | NA |  | 1.02525 | | NA | 1.04919 | | 1.14492 | |
| NA |  | 1.10172 | | 1.13303 | 1.28772 | | 1.24136 | | 1.20363 | 1.19695 | | 1.05593 | |
| NA |  | 1.10172 | | 1.13303 | 1.28772 | | 1.24136 | | 1.20363 | 1.19695 | | 1.05593 | |
|  | 0.875 | 1.19461 | | 1.10246 | 1.38851 | | 1.38327 | | 1.32937 | 1.19846 | | 1.04119 | |
| 0.454545 | | 1.14437 | | 1.09749 | 1.50549 | |  | 1.3924 | 1.41037 | 1.24301 | | 1.15869 | |
| NA |  |  | 1.095 | 1.23339 | 1.10138 | | 1.11938 | | 1.10682 | 1.16525 | | 1.36119 | |
| NA |  | 1.02763 | | NA | 1.02227 | | 1.03103 | | 1.05542 | 1.03913 | | 0.964785 | |
| NA |  | 0.99987 | | NA | NA |  | 1.00007 | | NA | NA |  | NA |  |
| NA |  | 0.980134 | | NA | 0.952829 | | 0.980004 | | 0.996154 | NA |  | NA |  |
| NA |  | 0.984437 | | NA | 0.971279 | | 0.981902 | | 0.992481 | NA |  | NA |  |
| 1.13889 | | NA |  | 0.99894 | NA |  | 1.14356 | | NA | 1.00215 | | 1.11673 | |

| 1.07143 | | 0.978555 | | 0.976132 | | NA |  | NA |  | NA |  | 0.96923 | | 0.986268 | |
| --- | --- | --- | --- | --- | --- | --- | --- | --- | --- | --- | --- | --- | --- | --- | --- |
| 1.07143 | | 0.978555 | | 0.976132 | | NA |  | NA |  | NA |  | 0.96923 | | 0.986268 | |
| 1.07143 | | 0.978555 | | 0.976132 | | NA |  | NA |  | NA |  | 0.96923 | | 0.986268 | |
| 0.880734 | | NA |  | 1.04574 | | 1.03712 | | 1.08206 | | 1.08484 | | 1.06277 | | 0.958174 | |
| 0.71904 | | NA |  | 1.02062 | |  | 1.0547 | 1.06567 | | 1.08155 | | 1.04311 | | 0.892167 | |
| NA |  | 1.01694 | | 0.971569 | | 1.05564 | | 0.983571 | | 1.02866 | | 0.952685 | | 0.930065 | |
| NA |  | 1.09582 | | 1.04649 | | 1.07556 | | 1.09366 | | 1.09984 | | 1.00439 | | 1.05878 | |
|  | 2 |  | 1.0616 | 1.01028 | | 1.05766 | | 1.07942 | | 1.07909 | | 1.03474 | | 1.08531 | |
| NA |  | 0.996625 | | 1.04224 | | 1.01244 | | 1.04407 | |  | 1.0391 | 0.957197 | | 0.955429 | |
| NA |  | 1.10533 | | 1.10204 | | 1.07414 | | 1.06714 | | 1.08305 | | 1.05022 | |  | 1.0732 |
|  | 1.375 | 1.12226 | | 1.05236 | | 1.11446 | | 1.11564 | | 1.16839 | | 1.07108 | | 1.12987 | |
| NA |  | 1.02838 | | 0.997421 | | 1.07213 | | 1.02273 | | 1.05802 | | 0.992032 | | 0.991105 | |
|  | 1.24 |  | 1.1503 |  | 1.0547 | 1.06779 | | 1.13283 | |  | 1.1544 | 1.06835 | |  | 1.0922 |
| NA |  | 1.06396 | | 1.03455 | | 1.07761 | | 1.14074 | |  | 1.1209 | 1.04095 | | 1.08815 | |
| 0.911765 | | 1.08531 | | 1.01697 | | 1.04285 | | 1.11791 | | 1.08765 | | 1.03399 | | 1.06316 | |
| NA |  | 0.992658 | | NA |  | 0.983201 | | 0.986575 | | 0.988153 | | NA |  | NA |  |
| NA |  | 1.16889 | |  | 1.1187 | 1.20041 | | 1.16038 | | 1.15722 | | 1.19955 | | 0.953519 | |
| NA |  | 1.12111 | | 1.07811 | | 1.06725 | | 1.09731 | | 1.08023 | | 1.07517 | | 1.06663 | |
| 1.61905 | | 1.10526 | | 1.05045 | | 1.00942 | | 1.07767 | |  | 1.0471 | 1.06058 | | 1.01331 | |
| 0.666667 | | 1.07778 | | 0.96663 | | 1.01856 | | 1.00385 | | 0.925714 | | 1.03929 | | 0.936107 | |
| NA |  | 0.992099 | | 1.00258 | | 0.971409 | | 0.949643 | | 0.978657 | | 0.969455 | | 0.956428 | |
| NA |  | 1.02927 | | 0.999412 | | 1.02716 | | 1.01222 | | 0.979519 | | 1.00671 | | NA |  |
| NA |  | 0.973392 | | NA |  | 0.987113 | | 0.991025 | | 0.985719 | | NA |  | NA |  |
| NA |  | 1.03294 | |  | 1.0526 | 0.992968 | | 0.996128 | | 0.999877 | |  | 1.0156 | 1.01226 | |
| NA |  | 1.09309 | | 1.09465 | | 1.06835 | | 1.07788 | |  | 1.042 | 1.12829 | |  | 1.125 |
| NA |  | 1.07054 | | 1.08486 | | 1.02966 | | 1.10176 | | 1.05062 | | 1.03116 | | 1.00601 | |
| NA |  |  | 1.0655 | 1.04125 | | 1.03508 | | 1.05782 | | 1.03259 | | 1.06233 | | 1.05436 | |
| NA |  | 1.08191 | | 1.05612 | | 1.07697 | | 1.11864 | |  | 1.0946 | 1.07653 | | 0.99627 | |
| NA |  | 1.13036 | | 1.03493 | | 1.09114 | | 1.14479 | | 1.13329 | | 1.11548 | | 1.04574 | |
| NA |  | 1.13036 | | 1.03493 | | 1.09114 | | 1.14479 | | 1.13329 | | 1.11548 | | 1.04574 | |
| NA |  | 1.04063 | | 1.06377 | | 1.03419 | | 1.03706 | | 1.03002 | |  | 1.0366 | 0.998427 | |
| NA |  |  | 1.0546 | 1.01451 | | 1.04263 | | 1.12646 | | 1.04865 | | 1.04131 | |  | 1.042 |
| NA |  | NA |  | 1.04448 | | 1.04774 | | 1.03051 | | 1.02423 | | NA |  | 1.08364 | |
| NA |  | 1.01076 | | 1.05479 | | 1.00117 | | 1.00989 | | 0.984125 | | 0.945361 | | 0.92199 | |
| NA |  | 1.02028 | | 0.951351 | | 1.00271 | | 1.02126 | |  | 1.0249 | NA |  | NA |  |
| NA |  | 0.969662 | | 1.01334 | | 1.01109 | |  | 1.0466 | 1.07177 | | 1.03196 | | 0.95162 | |
| NA |  | 1.05874 | | 1.08707 | | 0.99585 | | 1.01696 | | 1.01891 | | 1.00987 | | 1.03859 | |
| NA |  | 1.03983 | | 1.03375 | | 1.03206 | | 1.04753 | | 1.06373 | |  | 1.0179 | 0.971123 | |
|  | 1 | 1.21734 | | 1.03643 | | 1.03878 | |  | 1.089 | 1.01124 | | 1.16971 | | 1.10365 | |

| NA |  | 1.0508 | 1.03839 | | 1.05068 | 1.03146 | | 1.04755 |  | 1.0083 | 0.988952 |
| --- | --- | --- | --- | --- | --- | --- | --- | --- | --- | --- | --- |
| NA |  | 1.00776 | 1.01315 | | 1.02828 | 0.940728 | | 0.990017 | 0.981903 | | 0.934383 |
| NA |  | 1.03225 | 1.01787 | | 1.05546 |  | 1.0436 | 1.06489 | 0.992763 | | 0.959842 |
| NA |  | 1.06208 | 0.983557 | | 1.05808 | 1.03835 | | 1.02719 | 1.02223 | | 1.03789 |
| NA |  | 1.00649 | 0.974089 | | 0.937615 | 0.978615 | | 0.976602 | 1.00829 | | NA |
| NA |  | 0.991379 | 1.03196 | | 0.968678 | 0.967463 | | 0.992271 | 1.01415 | | NA |
| NA |  | 1.01422 | 0.951481 | | 0.985674 | 0.996319 | | 0.997658 | 0.997942 | | NA |
| NA |  | 1.06363 | 0.963908 | | 1.0312 | 1.03395 | | 1.02733 | 1.06845 | | NA |
| NA |  | 1.05389 | 0.968496 | | 1.03023 | 1.04299 | | 1.01911 | 1.03918 | | NA |
| NA |  | 1.02825 | 0.989852 | | 1.0105 | 1.02049 | | 0.996513 |  | 1.0217 | NA |
| NA |  | 0.988749 | 0.967888 | | 1.01212 | 1.01184 | | 1.00004 | 0.992692 | | NA |
| NA |  | 0.988749 | 0.967888 | | 1.01212 | 1.01184 | | 1.00004 | 0.992692 | | NA |
| NA |  | 0.988749 | 0.967888 | | 1.01212 | 1.01184 | | 1.00004 | 0.992692 | | NA |
| NA |  | 1.03631 | 0.934428 | | 0.999104 | 1.01996 | | 0.985392 | 1.01912 | | NA |
| NA |  | 1.0362 |  | 1.0017 | 1.02097 | 1.01138 | | 1.00906 | 1.03206 | | NA |
| NA |  | 1.07176 | 1.02511 | | 1.03552 | 1.04236 | | 0.999701 |  | 1.0215 | NA |
| NA |  | 1.06412 | 0.995041 | | 1.02522 | 1.01073 | | 1.02698 | 1.06839 | | NA |
| NA |  | 1.03458 | 0.992079 | | 1.03777 | 1.02123 | | 1.03632 | 1.03259 | | NA |
| NA |  | 1.06793 | 1.06288 | | 1.02265 | 1.02771 | | 1.01206 | 1.03713 | | NA |
| NA |  | 1.06524 | 0.987031 | | 1.02278 | 1.02909 | | 1.01764 | 1.04854 | | NA |
| NA |  | 1.01726 | NA |  | 1.01409 | 1.00327 | | 1.00972 | 1.00441 | | NA |
| 1.41667 | | 1.10322 | 1.04471 | | 1.06363 | 1.08754 | | 1.07059 | 1.05738 | | 1.06156 |
| NA |  | 0.985598 | 1.00404 | | 1.02482 | 0.96805 | | 0.993701 | 1.02264 | | NA |
| NA |  | 0.985598 | 1.00404 | | 1.02482 | 0.96805 | | 0.993701 | 1.02264 | | NA |
| NA |  | 1.03098 | NA |  | 1.03489 | 1.02048 | | 1.01989 | 1.00397 | | NA |
| NA |  | 1.03098 | NA |  | 1.03489 | 1.02048 | | 1.01989 | 1.00397 | | NA |
| NA |  | 1.06047 | NA |  | 1.00828 | 0.991508 | | 1.01283 | 0.989288 | | NA |
| NA |  | 1.05138 | NA |  | 1.0377 | 0.972865 | | 1.03035 | 1.01874 | | NA |
| NA |  | 0.977691 | NA |  | 0.953573 | 0.952613 | | 0.942769 | 0.956493 | | NA |
| NA |  | 1.0931 | NA |  | 1.03514 | 1.03286 | | 1.05536 | 1.02646 | | NA |
| NA |  | 1.0931 | NA |  | 1.03514 | 1.03286 | | 1.05536 | 1.02646 | | NA |
| NA |  | 1.0931 | NA |  | 1.03514 | 1.03286 | | 1.05536 | 1.02646 | | NA |
| NA |  | 0.991173 | NA |  | 0.976489 | 0.998242 | | 1.00165 | NA |  | NA |
| NA |  | 0.984872 | NA |  | 0.973756 | 0.990633 | | 0.990905 | NA |  | NA |
| NA |  | 1.00321 | 0.965355 | | 0.992121 | 1.01364 | | 1.00404 | NA |  | NA |
| NA |  | 1.00323 | 0.967425 | | 0.972485 | 0.978117 | | 1.00543 | NA |  | NA |
| NA |  | 1.02513 | 1.04332 | | 0.97979 | NA |  | 0.959811 | 1.02371 | | 0.855496 |
|  | 0.75 | 1.08819 | 1.02406 | | 1.16934 | 1.19284 | | 1.14278 | 1.10174 | | 1.02876 |
|  | 0.75 | 1.08819 | 1.02406 | | 1.16934 | 1.19284 | | 1.14278 | 1.10174 | | 1.02876 |

| 0.957573 |  | 1.179 | 1.14727 | | 1.38093 | | 1.43785 | | 1.30509 | | 1.19914 | | 1.09049 |
| --- | --- | --- | --- | --- | --- | --- | --- | --- | --- | --- | --- | --- | --- |
| NA | NA |  | NA |  | 1.29791 | |  | 1.4182 | 1.25685 | | 1.14594 | | 1.00355 |
| 1.53846 | 1.01282 | | 1.05017 | | 1.18646 | | 1.18574 | | 1.11944 | | 1.10817 | | 1.14786 |
| NA | 1.01942 | | 1.06373 | | NA |  | NA |  | NA |  | 1.06316 | | 0.954121 |
| NA | 1.01278 | | 1.12025 | | 1.07025 | | 1.08491 | | 1.09702 | | 0.968248 | | 1.13991 |
| NA | NA |  | NA |  | 1.01959 | | 1.01958 | | 1.03269 | | 1.04551 | | 0.993609 |
| NA | 1.00547 | | 0.94116 | | 0.976325 | | 1.00021 | | 1.00277 | | 0.980052 | | NA |
| NA | 1.02541 | | 1.02208 | |  | 1.0206 | 1.04188 | | 1.09561 | | 1.01627 | | NA |
| NA | 0.946519 | | 0.971419 | | 1.01117 | | 1.00709 | |  | 1.0004 | 1.06672 | | 0.885445 |
| NA | 1.01857 | | 1.01391 | |  | 1.0592 | 1.02037 | | 1.02359 | | 1.07636 | | NA |
| NA | 0.985544 | | 1.04523 | | 1.02459 | | 0.967221 | | 0.985286 | |  | 1.0526 | 0.899628 |
| NA | 1.01009 | |  | 1.0018 | 0.992225 | | 1.02346 | | 1.01505 | | NA |  | NA |
| NA | 1.05855 | | 0.999786 | | 1.05196 | | 1.05151 | | 1.01764 | | NA |  | NA |
| NA | NA |  | NA |  | 1.00767 | | 0.999016 | | 1.00602 | | NA |  | NA |
| NA | NA |  | NA |  | 1.02267 | | 1.03112 | | 1.00008 | | NA |  | NA |
| NA | NA |  | NA |  | 1.03173 | | 1.06307 | | 1.08189 | | NA |  | NA |
| NA | 1.01139 | | 0.948163 | | 0.995749 | | 1.01609 | | 1.01723 | | NA |  | NA |
| NA |  | 1.0625 |  | 1.0606 | 1.01393 | | 1.03471 | | 1.03626 | | NA |  | NA |
| NA | 1.01758 | | 0.981933 | | 0.975786 | | 0.977159 | | 0.981263 | | NA |  | NA |
| NA | 0.979321 | | 0.952713 | | 0.984772 | | 0.972702 | | 0.981183 | | NA |  | NA |
| NA | 0.977266 | | 0.935176 | |  | 1.0059 | 0.99069 | | 0.997884 | | NA |  | NA |
| NA | 1.00866 | | 1.00336 | | 0.97567 | | 1.01612 | | 1.02501 | | NA |  | NA |
| NA | 1.01693 | | 1.02365 | | 0.99439 | | 1.02488 | | 1.02677 | | NA |  | NA |
| NA | 0.989304 | | 0.948733 | | 0.948029 | | 0.972013 | | 0.986351 | | NA |  | NA |
| NA | 1.00616 | | 0.939558 | | 1.04955 | | 1.04423 | | 1.06191 | | NA |  | NA |
| NA | 1.01476 | | 0.974877 | | 1.00725 | | 1.01153 | | 0.998066 | | NA |  | NA |
| NA | 1.00472 | | 1.05318 | | 1.05316 | | 1.04466 | |  | 1.0183 | NA |  | NA |
| NA | 1.01991 | | 1.00873 | | 1.08533 | | 1.07997 | | 1.07358 | | NA |  | NA |
| NA | 1.01602 | | 0.942437 | | 0.991731 | | 1.01495 | | 1.01365 | | NA |  | NA |
| NA | 1.01305 | | 0.945881 | | 0.991755 | | 1.01111 | | 1.01259 | | NA |  | NA |
| NA | 1.08337 | | 1.04704 | | 0.980449 | | 0.963303 | | 1.00419 | | 0.999769 | | NA |
| NA | 1.01053 | | 0.961511 | | 0.983897 | | 1.01965 | | 1.01624 | | NA |  | NA |
| NA | 0.98954 | | 0.889646 | | 0.921032 | | 0.937658 | | 0.978483 | | NA |  | NA |
| NA | 1.00161 | | 0.915662 | | 0.94036 | | 0.946921 | | 0.946086 | | NA |  | NA |
| NA | 1.02858 | | 0.962338 | | 1.05357 | | 1.04666 | | 1.02074 | | NA |  | NA |
| NA |  | 1.0603 | 1.05824 | | 1.12467 | | 1.10808 | | 1.10409 | | NA |  | NA |
| NA | 1.04592 | | 0.970228 | | 1.05463 | | 1.04566 | | 1.04363 | | NA |  | NA |
| NA | 1.04065 | | 1.07795 | | 1.06211 | | 1.10251 | | 1.07903 | | NA |  | NA |
| NA | 0.991339 | | 0.952182 | |  | 1.0686 | 1.08956 | | 1.06646 | | NA |  | NA |

| NA | 1.03992 | | 1.00903 | | 0.999076 | | 0.997787 | | 1.00207 | | NA | NA |  |
| --- | --- | --- | --- | --- | --- | --- | --- | --- | --- | --- | --- | --- | --- |
| NA | 0.987578 | |  | 0.9521 | 0.969588 | | 0.974601 | | 0.977008 | | NA | NA |  |
| NA | 1.01302 | |  | 0.9704 | 0.984427 | | 1.01734 | |  | 1.0136 | NA | NA |  |
| NA | NA |  | NA |  | 1.04805 | |  | 1.0175 | 1.03395 | | NA | NA |  |
| NA | 1.06536 | | NA |  |  | 1.0433 |  | 1.046 | 1.01637 | | NA | NA |  |
| NA | 1.02795 | |  | 1.109 | 1.11161 | | 1.09024 | | 1.09789 | | NA | NA |  |
| NA | NA |  | 0.952898 | | 1.02812 | | 1.02228 | | 1.00219 | | NA | NA |  |
| NA | 0.983006 | | 0.981113 | | 1.03966 | | 1.05696 | | 1.04939 | | NA | NA |  |
| NA | 0.997221 | | 0.983157 | | 0.99322 | | 1.02193 | | 1.01511 | | NA | NA |  |
| NA | 1.00622 | | 1.01619 | | 1.03288 | | 1.04718 | | 1.04422 | | NA | NA |  |
| NA | 0.974162 | |  | 1.0106 | 0.996918 | | 0.99119 | | 0.989708 | | 0.992724 | 0.862137 | |
| NA | 1.24013 | | 1.20326 | | 1.35609 | | 1.30964 | | 1.29136 | | 1.21011 | NA |  |
| NA | NA |  | 1.04813 | | 1.03324 | | 0.975035 | | 0.960415 | | NA | NA |  |
| NA | 1.04182 | | 1.01241 | | 1.03076 | | 0.993059 | | 0.990693 | | 0.989977 | 0.876668 | |
| NA | 1.07759 | | 1.21716 | | 1.27412 | | 1.31087 | | 1.29436 | | 1.10524 | 1.11123 | |
| NA | 1.02363 | |  | 1.0406 | 1.12897 | | 1.10974 | | 1.13631 | | 1.06793 | 1.02738 | |
| NA | NA |  | NA |  | 1.24266 | |  | 1.1843 |  | 1.1531 | 1.12021 | 1.08228 | |
| NA | 0.985331 | | 0.997512 | | 1.08672 | | 1.00216 | | 1.05347 | | 1.01014 | NA |  |
| NA |  | 1.0143 | 0.946372 | | 1.08772 | | 1.04272 | | 1.08571 | | 1.06214 | 0.968249 | |
| NA | NA |  | 1.17173 | | 1.24304 | | 1.14061 | | 1.13971 | | 1.11814 | NA |  |
| NA | 1.08354 | | 0.993508 | | 1.07876 | | 1.05151 | | 1.03781 | | NA |  | 1.0042 |
| NA | 1.10156 | | 1.02668 | | 1.07211 | | 1.02534 | | 1.02195 | | 1.07703 | 1.03912 | |
| NA | 1.05671 | | 1.00409 | | 1.01174 | | 0.966064 | | 0.987481 | | 0.986812 | 1.00366 | |
| NA | 1.08581 | | 0.992428 | | 1.01428 | | 1.07009 | | 1.02477 | | NA | 1.03914 | |
| NA | NA |  | NA |  | 1.00468 | | 1.02571 | | 0.992006 | | NA | NA |  |
| NA | 1.02258 | | 1.02876 | | NA |  | NA |  | NA |  | 1.02228 | 1.07356 | |
| 0.736842 | NA |  | NA |  | 1.10106 | | 1.16289 | | 1.09374 | | 1.01742 | 1.03244 | |
| 0.777778 | NA |  | NA |  | 1.17401 | | 1.22619 | | 1.16824 | | 1.09012 |  | 1.0069 |
| 0.777778 | NA |  | NA |  | 1.17401 | | 1.22619 | | 1.16824 | | 1.09012 |  | 1.0069 |
| 0.695652 | NA |  | NA |  | 1.09749 | | 1.11968 | | 1.12738 | | NA | 1.08287 | |
| 0.615385 | NA |  | 0.997772 | | 1.05239 | | 1.05731 | | 1.02543 | | 0.996538 | 0.964938 | |
| 0.552632 | NA |  | NA |  | 1.02712 | | 1.03571 | | 0.996703 | | NA | 1.02653 | |
| 0.552632 | NA |  | NA |  | 1.02712 | | 1.03571 | | 0.996703 | | NA | 1.02653 | |
| 0.552632 | NA |  | NA |  | 1.02712 | | 1.03571 | | 0.996703 | | NA | 1.02653 | |
| 1.03636 | NA |  | 0.934817 | | 0.999425 | | 1.02009 | | 0.98868 | | 0.92258 | 1.06047 | |
| 0.702296 | NA |  | 1.01278 | | NA |  | NA |  | NA |  | 0.99018 | 0.946313 | |
| 0.364583 | NA |  | NA |  | 1.04939 | | 1.03628 | | 1.02836 | | NA | NA |  |
| NA | 1.05234 | |  | 1.002 | 0.990187 | | 1.03277 | | 1.01375 | | NA | NA |  |
| NA | 1.00814 | | 0.981151 | | 1.00109 | | 0.973164 | | 0.999722 | | 0.978121 | NA |  |

| NA | 1.00575 | 1.03315 | 0.954531 | | 0.934165 | | 0.960196 | | NA | NA |  |
| --- | --- | --- | --- | --- | --- | --- | --- | --- | --- | --- | --- |
| NA | 1.03711 | 1.01396 | 0.980783 | | 1.00432 | | 1.01341 | | NA | NA |  |
| NA | 1.02622 | 0.986402 | 1.00962 | | 0.999072 | | 1.00326 | | NA | NA |  |
| NA | 0.99672 | 1.01185 | 0.945865 | | 0.969381 | | 1.01095 | | NA | NA |  |
| NA | 1.01311 | 0.896223 | 0.912908 | | 0.950247 | | 0.94255 | | 0.94531 | 1.03045 | |
| NA | 1.09948 | 1.02068 | 1.03881 | | 1.10479 | | 1.04633 | | 1.00149 | 1.08094 | |
| NA | 0.99668 | 1.04547 | NA |  | NA |  | NA |  | 0.991171 | 0.982522 | |
| NA | 1.07058 | 1.03213 | 1.04638 | | 1.01479 | | 1.02283 | | 1.01432 | 1.04007 | |
| NA | 1.03213 | 0.99116 | NA |  | NA |  | NA |  | 0.986263 | 0.830412 | |
| NA | 1.05249 | 1.04287 | NA |  | NA |  | NA |  | 0.97701 | 0.888831 | |
| NA | 1.0924 | 1.04693 | 1.21502 | |  | 1.1922 | 1.18306 | | 1.13442 | 1.06604 | |
| 1.17391 | 1.19858 | 1.17436 | 1.23378 | | 1.33904 | | 1.26263 | | 1.21684 |  | 1.0905 |
| 1.12411 | 1.33378 | 1.25921 | 1.45056 | | 1.46752 | | 1.37479 | | 1.30041 |  | 1.2802 |
| NA | 1.00137 | 1.03062 | 0.989674 | | 1.04987 | | 0.974195 | | 0.990149 | 1.01937 | |
| NA | 1.03016 | 1.02062 | 0.975148 | | 1.04382 | | 1.00955 | | NA | NA |  |
| NA | 1.013 | 0.953879 | 0.986909 | | 1.01696 | | 1.01411 | | NA | NA |  |
| NA | 1.11824 | 1.07127 |  | 1.2812 | 1.25902 | | 1.22972 | | 1.18236 | 1.13302 | |
| NA | 1.00844 | 0.977101 | 0.992632 | | 1.02496 | | 1.01144 | | NA | NA |  |
| NA | 0.998857 | 0.948689 | 1.00301 | | 0.983562 | | 1.00388 | | 0.981671 | 0.978175 | |
| NA | 1.02398 | 1.07598 | 0.94706 | | 0.994692 | | 0.992193 | | NA | NA |  |
| NA | 1.02398 | 1.07598 | 0.94706 | | 0.994692 | | 0.992193 | | NA | NA |  |
| 1.08333 | 1.17444 | 1.13443 | 1.16011 | | 1.12233 | | 1.13586 | | 1.13532 | 1.15708 | |
| NA | 1.117 | 1.08995 | 1.21601 | | 1.17921 | |  | 1.1722 | 1.13752 | 1.10844 | |
| NA | 1.117 | 1.08995 | 1.21601 | | 1.17921 | |  | 1.1722 | 1.13752 | 1.10844 | |
| NA | 1.117 | 1.08995 | 1.21601 | | 1.17921 | |  | 1.1722 | 1.13752 | 1.10844 | |
| NA | 1.117 | 1.08995 | 1.21601 | | 1.17921 | |  | 1.1722 | 1.13752 | 1.10844 | |
| NA | 1.03468 | 1.08596 | 1.07565 | | 1.10999 | | 1.07276 | | 1.01191 | 0.980189 | |
| NA | 1.03468 | 1.08596 | 1.07565 | | 1.10999 | | 1.07276 | | 1.01191 | 0.980189 | |
| NA | 1.03468 | 1.08596 | 1.07565 | | 1.10999 | | 1.07276 | | 1.01191 | 0.980189 | |
| NA | 1.03468 | 1.08596 | 1.07565 | | 1.10999 | | 1.07276 | | 1.01191 | 0.980189 | |
| NA | 1.08383 | 1.2309 | 1.35651 | | 1.24404 | | 1.23279 | | 1.14691 | 1.04747 | |
| NA | 1.08383 | 1.2309 | 1.35651 | | 1.24404 | | 1.23279 | | 1.14691 | 1.04747 | |
| NA | 1.17817 | 1.08054 | 1.18419 | | 1.21096 | | 1.17781 | | 1.12034 | 0.981034 | |
| NA | 1.17817 | 1.08054 | 1.18419 | | 1.21096 | | 1.17781 | | 1.12034 | 0.981034 | |
| 1.16327 | 1.18174 | 1.11554 | 1.38356 | | 1.31609 | |  | 1.2711 | 1.25344 | 1.09431 | |
| NA | 0.964277 | 0.933153 | 0.984539 | | 0.980286 | | 0.999953 | | 0.993057 | 0.94785 | |
| NA | 0.964277 | 0.933153 | 0.984539 | | 0.980286 | | 0.999953 | | 0.993057 | 0.94785 | |
| NA | 0.974836 | 0.956862 | 1.00671 | | 0.978917 | | 0.99637 | | 0.966149 | 1.03312 | |
| NA | 0.974836 | 0.956862 | 1.00671 | | 0.978917 | | 0.99637 | | 0.966149 | 1.03312 | |

| NA |  | 0.972597 | | 0.948786 | | 1.01399 | | 0.998301 | | 0.981571 | | 0.968436 | | 1.09746 |
| --- | --- | --- | --- | --- | --- | --- | --- | --- | --- | --- | --- | --- | --- | --- |
|  | 0.9375 | 0.966297 | | 0.961243 | | 1.02186 | | 0.986955 | | 1.02951 | | 1.01488 | | 0.988934 |
| NA |  | 0.988933 | | 0.939996 | | 0.959576 | | 0.968475 | | 0.944442 | | 0.975384 | | 1.05116 |
| NA |  | 1.01469 | | 0.998854 | | 0.989996 | | 0.976161 | | 1.00335 | | 1.02765 | | 0.953704 |
| NA |  | 1.04081 | | 0.973782 | |  | 0.9999 | 1.01756 | | 1.03488 | | 0.992563 | | 0.961443 |
| NA |  | 1.04019 | | 1.04319 | | 1.11488 | | 1.07893 | | 1.05518 | | NA |  | 0.977143 |
| NA |  |  | 1.0896 | 1.08936 | | 1.06641 | | 1.00613 | | 1.06294 | | 1.00752 | | 0.972438 |
| NA |  | 1.07774 | | 1.03165 | | 1.04373 | | 1.02395 | |  | 1.0387 | 1.07748 | | 0.97875 |
| NA |  | 1.04694 | | 0.995616 | | 1.06247 | | 1.02594 | | 1.02229 | | 1.06403 | | 1.06716 |
| NA |  | 1.01033 | | 1.01867 | | 1.00526 | | 1.01101 | | 1.00736 | | 1.02544 | | 0.963815 |
| NA |  | 1.04169 | | 0.977643 | | 1.04946 | | 1.01104 | | 1.02272 | | 1.05608 | | 0.986715 |
| NA |  | 1.01543 | | 0.977883 | | 0.983832 | | 0.993311 | | 0.983207 | | 1.01669 | | 0.951322 |
| 0.818182 | | NA |  | NA |  | NA |  | NA |  | NA |  | NA |  | NA |
| NA |  | NA |  | 1.05869 | | NA |  | NA |  | NA |  | 0.984761 | | 0.996743 |
| NA |  | NA |  | NA |  | NA |  | NA |  | NA |  | NA |  | NA |
| NA |  | NA |  | NA |  | NA |  | NA |  | NA |  | NA |  | NA |
| NA |  | NA |  | NA |  | NA |  | NA |  | NA |  | NA |  | NA |
| NA |  | 0.955811 | | 0.931161 | | NA |  | NA |  | NA |  | 0.988493 | | NA |
| NA |  | NA |  | NA |  | NA |  | NA |  | NA |  | NA |  | NA |
| NA |  | NA |  | NA |  | NA |  | NA |  | NA |  | NA |  | NA |
| NA |  | NA |  | NA |  | NA |  | NA |  | NA |  | NA |  | NA |
| NA |  | NA |  | NA |  | NA |  | NA |  | NA |  | NA |  | NA |
| NA |  | 0.978537 | | NA |  | NA |  | NA |  | NA |  | NA |  | NA |
| NA |  |  | 1.0294 | NA |  | 1.04308 | | NA |  | 0.996739 | | NA |  | NA |
| NA |  | 0.971593 | | 1.01487 | | 0.987497 | | NA |  | 0.961668 | | NA |  | 0.978361 |
| NA |  | 1.00117 | | 0.96217 | | 0.988324 | | 1.02188 | | 1.01126 | | NA |  | NA |
| NA |  |  | 1.0137 | 0.950562 | | 0.998226 | | 1.01975 | |  | 1.0207 | NA |  | NA |
| NA |  | 0.980572 | | NA |  | 0.963933 | | 0.965288 | | 0.986579 | | NA |  | NA |
| NA |  | 1.15259 | | 1.06409 | |  | 1.3059 |  | 1.2673 | 1.22854 | | 1.21963 | | 1.08003 |
| 0.888889 | |  | 1.0155 | 1.02466 | | 1.04566 | | 1.02082 | | 1.07284 | | 0.983876 | | 1.10277 |
| 0.888889 | |  | 1.0155 | 1.02466 | | 1.04566 | | 1.02082 | | 1.07284 | | 0.983876 | | 1.10277 |
| NA |  | 0.875776 | |  | 1.02 | 1.08621 | |  | 1.2605 | 1.06935 | | 1.03815 | | NA |
| NA |  | 0.975637 | | 1.04842 | | 1.04575 | | NA |  | NA |  | 1.04827 | | NA |
| NA |  | 0.90643 | | 0.982463 | | 1.01477 | | 1.01364 | | 1.00667 | | 0.983563 | | NA |
| NA |  | 0.973579 | | 1.01481 | | 1.11089 | | 1.05391 | |  | 1.0636 | 1.04237 | | NA |
| 0.956522 | | 1.03867 | | 1.02958 | | 1.08072 | | 1.08547 | | 1.07385 | |  | 1.0345 | 1.03315 |
| NA |  | 1.00145 | | 1.00902 | | 0.982654 | | 0.985712 | | 0.967855 | | 1.03234 | | NA |
| NA |  | NA |  | 0.968579 | | 1.07658 | |  | 1.0862 | 1.05916 | | 1.01694 | | NA |
| NA |  | 0.971922 | | 1.04373 | | 1.06103 | | 1.01546 | | 1.02679 | | 1.04943 | | 1.01979 |

| NA | 0.978393 | | 0.982448 | | 1.04761 | | 0.976519 | | 1.00901 | | 1.04982 | | NA |  |
| --- | --- | --- | --- | --- | --- | --- | --- | --- | --- | --- | --- | --- | --- | --- |
| NA | 0.995933 | | 0.901794 | | 1.02954 | | 1.01247 | | 0.988321 | | 0.925228 | | NA |  |
| NA | 1.01898 | | 1.00593 | | 1.02341 | | 1.02802 | | 0.990195 | | 0.982659 | | NA |  |
| NA | 0.983183 | | 1.00428 | | 1.01819 | | 0.960363 | | 0.960728 | | 0.962811 | | NA |  |
| NA | 1.04437 | | 0.994384 | | 1.01814 | | 1.04345 | | 1.05101 | |  | 0.9814 | NA |  |
| NA | 1.06706 | | 1.01045 | | 0.97686 | | 0.998322 | | 1.00712 | | 0.986861 | |  | 1.0422 |
| NA | 1.02619 | | NA |  | NA |  |  | 1.0422 | NA |  | 1.02627 | | NA |  |
| NA | 1.02619 | | NA |  | NA |  |  | 1.0422 | NA |  | 1.02627 | | NA |  |
| NA | 1.02619 | | NA |  | NA |  |  | 1.0422 | NA |  | 1.02627 | | NA |  |
| NA | 1.00579 | | NA |  | NA |  | 1.01018 | | NA |  | NA |  | NA |  |
| NA | 0.992871 | | 1.05685 | | 0.980294 | | NA |  |  | 1.0047 | 1.00797 | | 1.10728 | |
| NA | 0.97719 | |  | 1.0963 | 1.06586 | | NA |  | NA |  | 1.06793 | |  | 1.1097 |
| NA | 1.07435 | | 1.13141 | | 1.07235 | | 1.05178 | | 1.11309 | | 1.04057 | | 1.03363 | |
| NA | 0.980127 | | NA |  | NA |  | NA |  | NA |  | NA |  | NA |  |
| NA | NA |  | 1.02231 | | NA |  | NA |  | NA |  | 1.02577 | | 1.06244 | |
| NA | 0.973015 | | 1.00692 | | 1.04917 | | 1.06297 | | 1.09086 | | 1.05216 | | NA |  |
| NA | 0.951819 | | 1.08723 | |  | 1.0587 | 1.09346 | | 1.04994 | | 1.06592 | | 1.04684 | |
| NA | NA |  | 1.02807 | | NA |  | NA |  | NA |  | 1.01208 | | 1.05329 | |
| NA | 0.988087 | | 0.997182 | | NA |  | 0.989505 | | NA |  | NA |  | NA |  |
| 0.519231 | NA |  | NA |  | NA |  | NA |  | NA |  | 1.01931 | | 0.876476 | |
| 0.892975 | NA |  | 1.02775 | | NA |  | NA |  | NA |  | 0.985085 | | 1.00511 | |
| NA | 0.99665 | | 1.03137 | | 1.01037 | | 0.982365 | | 0.980829 | | 1.06214 | | 0.975645 | |
| NA | 0.953968 | | 1.01105 | | 1.02922 | | 0.962879 | | 0.963801 | | 0.995346 | | 0.946839 | |
| NA | 1.13191 | | 0.969454 | | 1.21424 | | 1.41557 | | 1.34845 | | 1.22572 | | 1.14663 | |
| NA | 1.02276 | | 0.96654 | | 1.03544 | | 1.04333 | | 0.994781 | | 0.969558 | |  | 1.0277 |
| NA | 1.02074 | | 1.13665 | | 1.05097 | | NA |  | 1.03604 | | 1.01285 | | 1.05747 | |
| NA | NA |  | 0.993057 | | 0.969595 | | NA |  | NA |  | 0.99865 | | 1.10119 | |
| NA | NA |  | 1.03077 | | NA |  | NA |  | NA |  | 0.980087 | | 1.02422 | |
| NA | 0.998895 | | 1.02436 | | 1.08606 | | 1.05508 | | 1.05665 | | 0.991664 | | 0.979518 | |
| NA | 1.01993 | | 0.962287 | | 1.00371 | | NA |  | NA |  | 1.05612 | | 0.909576 | |
| NA | 1.19872 | | 1.04717 | | 1.16186 | | 1.01293 | | 1.16187 | |  | 1 | 1.17544 | |
| NA |  | 1.0639 | 0.971206 | | 1.00325 | | 0.964085 | | 0.994157 | | 1.05251 | | 0.907105 | |
| NA | 1.14341 | | 0.936275 | | 1.08585 | | 0.983178 | | 1.04911 | | 0.939791 | |  | 0.872 |
| NA | 1.01663 | | 1.04144 | | 1.03283 | | NA |  | NA |  | 0.970511 | | 0.957359 | |
| NA | 1.05833 | | 0.996078 | | 0.940585 | | 0.917201 | | 0.930285 | |  | 1.066 | 0.814961 | |
| NA | 1.04747 | | 1.07642 | | 1.00888 | | 0.948722 | | 0.939484 | | 1.04783 | | NA |  |
| NA | NA |  | NA |  | NA |  | NA |  | NA |  | 1.00826 | | NA |  |
| NA | NA |  | NA |  | NA |  | NA |  | NA |  | 1.00826 | | NA |  |
| NA | NA |  | NA |  | NA |  | NA |  | NA |  | 1.00826 | | NA |  |

| NA | NA |  | 0.931268 | | 0.944287 | NA |  | NA |  | NA |  | 0.977077 | |
| --- | --- | --- | --- | --- | --- | --- | --- | --- | --- | --- | --- | --- | --- |
| NA | NA |  | 0.90742 | | 0.956245 | NA |  | 0.921496 | | NA |  | 0.972123 | |
| NA | NA |  | 1.02886 | | NA | NA |  | NA |  | NA |  | 1.12376 | |
| NA | 0.946487 | | 1.02244 | | 1.03163 | 0.948664 | | 0.988034 | | 1.03419 | | NA |  |
| NA | 0.973748 | | 1.03883 | | 1.06103 | 1.04645 | |  | 1.0201 | 0.98378 | | NA |  |
| NA | 0.981471 | | 1.00345 | | 1.16073 | 1.10337 | | 1.03501 | | 1.05234 | | NA |  |
| NA | 0.959846 | | 0.976448 | | 1.12332 | 1.08235 | | 1.00904 | | 1.02452 | | NA |  |
| NA | 0.995329 | | 1.11568 | | 1.02476 | 1.02678 | | 1.02698 | |  | 1.0094 | NA |  |
| NA |  | 1.0223 | 0.985379 | | 0.96491 | NA |  | NA |  | 0.975701 | | 1.18135 | |
| NA | 1.00069 | | 1.03159 | | 0.993826 | 0.975168 | | 0.975324 | | 1.02074 | | 1.07077 | |
| NA | 0.976517 | | 1.01208 | | 0.989514 | 0.964653 | | 0.937252 | | 0.974294 | | 0.955376 | |
| NA | 0.976517 | | 1.01208 | | 0.989514 | 0.964653 | | 0.937252 | | 0.974294 | | 0.955376 | |
| NA | 1.04999 | | 1.05103 | | 1.06691 | 1.07395 | | 1.04961 | | 1.04767 | | 1.01133 | |
| NA | 1.08584 | | 1.02612 | | 1.04332 | 1.04088 | | 1.04481 | | NA |  | 0.940803 | |
| NA | 1.08584 | | 1.02612 | | 1.04332 | 1.04088 | | 1.04481 | | NA |  | 0.940803 | |
| NA | 0.984938 | | 0.964567 | | 0.972174 | 0.981033 | | 0.960952 | | 1.02287 | | NA |  |
| NA | 1.00863 | | 0.934213 | | 0.967695 | 0.967017 | | 0.969285 | | 0.941957 | | NA |  |
| NA | 1.00917 | | NA |  | 0.944781 | 0.955809 | | 0.958623 | | 1.02349 | | NA |  |
| 0.848485 | NA |  | NA |  | 1.04622 | 1.02997 | | 1.01469 | | NA |  |  | 1.0069 |
| NA | 1.09796 | | 1.12166 | | 1.09478 | 1.10623 | | 1.15232 | | 1.10172 | | 1.05759 | |
| NA | 0.977811 | | 1.04581 | | 0.985866 | 0.990135 | | 1.00949 | | NA |  | 1.10505 | |
| NA | NA |  | 0.99409 | | 0.97448 | 0.997333 | | 0.997214 | | NA |  | 0.928078 | |
| NA | 1.01255 | | 0.961725 | | 1.00287 | 0.970193 | | 0.974198 | | 1.01193 | | 0.970138 | |
| NA | 0.981386 | | 1.02354 | | 1.01244 |  | 1.0126 |  | 1.0167 | 0.993941 | | 1.01592 | |
| NA | 0.903431 | | 0.96235 | | 0.92861 | 0.939677 | | 0.929439 | | 0.960003 | | 1.04697 | |
| NA | 1.03272 | | 1.06499 | | 1.02147 | 1.02243 | | 1.03322 | | 1.02052 | | 0.975395 | |
| NA | 1.00149 | | 0.97825 | | 1.04928 | 1.01718 | | 1.00521 | | 0.989913 | | 0.858609 | |
| NA | 1.01128 | | 1.09179 | | 1.00659 | 0.994925 | | 1.00213 | | 1.01169 | | 1.10863 | |
| NA | 1.01403 | | 0.999135 | | 0.997436 | 0.95271 | | 0.977959 | | 1.04857 | | 0.829545 | |
| 0.491228 | 1.01057 | | 0.955993 | | 1.02528 | 1.05622 | | 1.02163 | | 0.997002 | | 1.10883 | |
| NA | 0.917258 | |  | 0.9076 | 1.00822 | 0.995678 | | 0.986933 | | 0.954995 | | 0.927156 | |
| NA | 1.00102 | | NA |  | 0.975645 | 0.997037 | | 0.980901 | | 1.01106 | | NA |  |
| NA | 0.981784 | | 1.01305 | | 1.03454 | 0.966978 | | 0.986743 | | 0.999241 | | NA |  |
| NA | 1.01216 | | 0.997042 | | 0.960365 | 1.00585 | | 0.990612 | | 1.00086 | | NA |  |
| NA | 0.992962 | | 1.01114 | | 0.94577 | 0.960235 | |  | 0.9636 | 0.929256 | | NA |  |
| 1.37179 | NA |  |  | 1.0514 | NA | NA |  | NA |  | 1.04493 | | 1.00004 | |
| 2.16393 | 1.03837 | |  | 1.0066 | NA | NA |  | NA |  | 1.05737 | | 0.946377 | |
| 1.12623 | NA |  | 0.971356 | | NA | NA |  | NA |  | 1.02221 | | 1.04957 | |
| 1.61538 | NA |  | NA |  | NA | NA |  | NA |  | NA |  | 1.19194 | |

| 1.71429 | | 1.02111 | | 1.05151 | | NA | NA |  | NA |  | 1.06056 | | 1.01042 | |
| --- | --- | --- | --- | --- | --- | --- | --- | --- | --- | --- | --- | --- | --- | --- |
| 0.785714 | | 0.944717 | | 0.992504 | | 1.01919 | 0.979788 | | 1.00704 | | 0.992823 | | 0.928356 | |
| NA |  | 1.09673 | | 1.09937 | | 1.23402 | 1.25325 | | 1.22387 | | 1.17027 | | 1.10576 | |
| NA |  | 1.10439 | | 0.99485 | | 1.19515 | 1.21748 | | 1.18371 | | 1.14975 | | 0.988374 | |
| NA |  | 1.03465 | | 0.996514 | | 1.10502 | 1.14766 | | 1.11636 | | 1.05002 | | 1.01417 | |
| NA |  | 0.947595 | | 1.01834 | | 0.928824 | 0.977798 | | 0.941124 | | 0.984036 | | 1.07168 | |
| NA |  | 1.07294 | |  | 1.0209 | 1.04169 | 1.00598 | | 0.972904 | | 1.06807 | | 0.86836 | |
| NA |  | 1.05995 | | 0.943977 | | 1.11925 | 1.19363 | | 1.16543 | | 1.11964 | | 1.01622 | |
| NA |  | 1.09532 | | 1.00424 | | 1.15792 | 1.16229 | | 1.15149 | | 1.11603 | | 1.09845 | |
| NA |  | 1.03875 | | 1.02697 | | 0.999814 | 1.02446 | | 1.02132 | | NA |  | NA |  |
| NA |  |  | 1.0426 | 1.01025 | | 0.989258 | 1.01806 | | 1.00869 | | NA |  | NA |  |
| NA |  | 1.06076 | | NA |  | NA | 1.00835 | | NA |  | NA |  | NA |  |
| 1.93013 | | 1.14811 | |  | 1.0385 | 1.18621 | 1.38287 | | 1.27993 | | 1.15441 | | 1.27906 | |
| 0.701064 | | 1.10839 | | 1.15737 | | 1.13481 | 1.10645 | | 1.09948 | | 1.15395 | | 1.13056 | |
| NA |  | 0.964762 | | 0.934074 | | 0.986823 | NA |  | 0.95338 | | 1.04804 | | 0.956808 | |
| 0.72549 | |  | 1.1202 | 1.07163 | | 1.19588 |  | 1.1611 | 1.13622 | | 1.14719 | | 0.933917 | |
| NA |  | 0.97963 | | NA |  | 0.968481 | NA |  | NA |  | 1.03817 | | NA |  |
| 0.786632 | | NA |  | NA |  | NA | NA |  | NA |  | NA |  | 1.03581 | |
| 0.928579 | | NA |  | 1.00618 | | 1.02238 | NA |  | NA |  | 1.02942 | | 1.01701 | |
| NA |  | 1.03099 | | 1.00013 | | 0.995972 | 1.00502 | |  | 1.0083 | NA |  | NA |  |
| 0.769231 | | 1.13359 | | 1.07748 | | 1.19477 | 1.23043 | |  | 1.1966 | 1.17667 | | 1.05954 | |
| NA |  | 1.05022 | | 1.11551 | | 1.04423 | 1.01571 | | 0.995945 | | 1.04858 | | NA |  |
| 0.704545 | | NA |  | NA |  | NA | NA |  | NA |  | 1.03002 | | 1.08163 | |
| 0.64387 | | NA |  | NA |  | NA | NA |  | NA |  |  | 1.0443 | 0.993363 | |
| 0.761905 | | NA |  | 1.02604 | | NA | NA |  | NA |  | 1.04227 | | 0.94069 | |
| 2.18182 | | NA |  | 0.952071 | | NA | NA |  | NA |  | 1.00267 | | 0.962851 | |
| 0.714286 | | 1.05427 | | 1.01847 | | NA | NA |  | NA |  | 1.02236 | | 1.03035 | |
| 1.11142 | | NA |  | 0.99196 | | NA | NA |  | NA |  | 1.03757 | | 0.969338 | |
| NA |  | NA |  | 1.07897 | | NA | NA |  | NA |  | 1.03024 | | 1.13225 | |
| NA |  | 1.14026 | | 1.12968 | | 1.16003 | 1.21464 | | 1.10639 | |  | 1.1794 | 1.03954 | |
| 1.35714 | | 1.05607 | | 1.01472 | | 1.04416 | 1.06378 | | 1.03646 | | 1.08992 | |  | 1.0394 |
|  | 2 | 1.12895 | | 1.13638 | | 1.12354 | 1.15923 | | 1.09549 | |  | 1.1182 | 1.15843 | |
| NA |  | 1.02252 | | 1.06877 | | 1.08618 | NA |  | 1.07774 | | 1.05093 | | 1.15097 | |
| NA |  | 1.05054 | | 1.04612 | | 1.03593 | NA |  | 1.07815 | | 1.03698 | | 0.974063 | |
| 1.12204 | | NA |  | NA |  | NA | 1.14649 | | 1.11597 | | 1.12593 | | 1.18678 | |
| NA |  | 1.01901 | | 1.02189 | | 1.01214 | 1.00663 | | 1.00498 | | 0.998376 | | 0.987736 | |
| NA |  | 1.00361 | | 1.03364 | | 0.976743 | 1.00349 | | 0.998248 | | 0.993992 | | 0.986856 | |
| NA |  | 1.08862 | | 1.11105 | | 1.06872 | NA |  | NA |  | 1.06903 | | 0.905812 | |
| 0.693493 | | 1.11336 | | 1.01374 | | 1.18574 | 1.29402 | | 1.24646 | | 1.05978 | | 1.08758 | |

| NA |  | 1.02809 | | 0.873484 | | NA |  | NA |  | NA |  | 1.02723 | | 1.05759 | |
| --- | --- | --- | --- | --- | --- | --- | --- | --- | --- | --- | --- | --- | --- | --- | --- |
| NA |  | 0.992692 | | 0.934497 | | 0.983033 | | NA |  | 0.994355 | | 1.02249 | | 1.07865 | |
| NA |  | 1.09984 | | 1.11905 | | 1.16111 | | 1.13818 | | 1.10935 | | 1.09221 | |  | 1.1012 |
| NA |  | NA |  | 0.997484 | | NA |  | NA |  | NA |  | 1.02301 | | 0.984344 | |
|  | 1.6616 | 1.21213 | | 1.02806 | | 1.26873 | | 1.43486 | | 1.36206 | | 1.29813 | | 1.15641 | |
| NA |  | 1.00648 | | 1.00758 | | 1.22101 | | NA |  | 0.980829 | | 0.986308 | | NA |  |
| NA |  | 0.985966 | | 1.01739 | | 0.948445 | | 0.953117 | | 0.956711 | | 1.00958 | | 1.07326 | |
| NA |  | 0.994971 | | 1.01715 | | 0.971228 | | NA |  | 0.960786 | | 0.989532 | | NA |  |
| 1.18182 | | 1.12441 | | 1.14688 | | 1.15424 | | 1.18756 | | 1.17761 | | 1.10983 | |  | 1.0524 |
| NA |  | 1.03716 | | 0.971045 | | 1.06199 | | NA |  | 1.07938 | | 1.12642 | | 1.04423 | |
| 1.55556 | | NA |  | 0.958012 | | NA |  | NA |  | NA |  | 0.979531 | | 1.00108 | |
| NA |  | 0.978897 | | 0.932868 | | 0.978316 | | 0.981814 | | 0.97489 | | NA |  | NA |  |
| NA |  | NA |  | 0.980805 | | 1.05681 | | NA |  | NA |  | 1.00473 | | 1.09234 | |
| 1.53693 | | NA |  | NA |  | 1.08348 | | 1.12615 | | 1.14208 | | 1.12015 | | 1.10431 | |
|  | 1 | NA |  | 0.963163 | |  | 1.1704 | 1.11745 | | 1.11705 | | 1.05846 | | 1.01062 | |
| 1.07256 | | 1.04191 | | 1.03535 | | 1.01761 | | 1.05377 | | 1.03112 | | 1.01538 | | 1.10332 | |
| 0.951956 | | 1.01146 | | 0.96426 | | 1.02032 | | NA |  | 1.06715 | | 1.07416 | |  | 1.2403 |
| 0.547619 | | 0.966223 | | 0.90756 | | 0.96765 | | 0.938934 | | 0.97931 | | 0.969955 | |  | 1.1086 |
| NA |  | NA |  | 0.985396 | | NA |  | NA |  | NA |  | 0.991412 | | NA |  |
| NA |  | NA |  | 1.04857 | | NA |  | NA |  | NA |  | 1.00718 | | 1.10877 | |
| NA |  |  | 1.0578 | 1.06507 | | 1.11668 | | NA |  |  | 1.1157 | 1.04514 | | 0.931133 | |
| NA |  | NA |  | 1.09884 | | 1.00477 | | 1.04482 | | 1.07706 | | NA |  | NA |  |
| 0.785714 | | 0.98234 | | 1.03159 | |  | 1.0295 | NA |  | 1.08104 | | 0.984991 | | 0.917955 | |
| NA |  | NA |  | 0.942141 | | 1.01007 | | NA |  | NA |  | NA |  | 0.969469 | |
| NA |  | 1.01736 | | 1.01636 | | 0.99205 | | NA |  | 1.01468 | | 0.991122 | | 1.02759 | |
| NA |  | 1.05921 | |  | 1.072 | 1.04186 | | 1.08652 | | 1.02307 | | 1.04721 | | 1.07577 | |
| NA |  | 1.06001 | | 1.05339 | | 1.08772 | | 1.12831 | | 1.08984 | | 1.09593 | | 1.04383 | |
| 1.03409 | | NA |  | 0.974983 | | 1.02359 | | NA |  | 0.995078 | | 1.00572 | | 0.988266 | |
| NA |  | 1.01434 | | 0.953228 | | 1.05856 | | 1.07571 | | 1.08657 | | 1.01981 | | 1.01373 | |
| NA |  | 1.03371 | | 1.01506 | | 1.08296 | | NA |  | 1.05171 | | 1.01615 | | NA |  |
| NA |  | NA |  | 1.01051 | | 1.01318 | |  | 1.0053 | 1.00621 | | 0.985479 | | 0.977502 | |
| 1.05234 | | 1.18615 | | 1.07381 | | 1.34094 | | 1.35348 | | 1.30373 | | 1.17128 | | 1.17312 | |
| 1.10598 | | NA |  | 1.01178 | | NA |  | NA |  | NA |  | 1.01014 | | 0.929082 | |
| NA |  | 0.989679 | | 0.948901 | | 1.07426 | | 1.08051 | | 1.04306 | |  | 1.0065 | 1.13248 | |
| NA |  | NA |  | NA |  | NA |  | 1.16064 | | 1.12713 | | 1.09801 | | NA |  |
| NA |  | 1.09674 | | 1.08727 | | 1.03712 | | NA |  | 1.06865 | | 1.06835 | | 1.10157 | |
| NA |  | 1.07224 | | 0.979223 | | 1.07615 | | 1.09037 | | 1.09684 | | 1.03345 | | 0.97075 | |
|  | 3.125 | NA |  | 1.08686 | | NA |  | NA |  | NA |  | 0.989664 | | 0.983357 | |

macropha mature_co mature_eo mature_ne megakaryomegakaryomemory_b mesangial

| 1.0517 | 1.10265 | 1.1037 | 1.08962 | 1.10652 | 1.09928 | 1.09872 | 1.21671 |
| --- | --- | --- | --- | --- | --- | --- | --- |

NA	NA	NA	NA	NA	NA	NA	NA

1.1242	1.12397	1.14028	1.11531	1.22197	1.1518	1.20699	1.14287

1.10749	1.13826	1.18038	1.13026	1.16328	1.12388	1.28708	1.03536

1.14342	1.15351	1.22573	1.12552	1.15813	1.12707	1.26228	1.00975

0.943789 0.943752 0.945268 0.970493 0.968791 0.989306 0.879866 0.955492

1.11796	1.10416	1.12342	1.15361	1.09799	1.1766	1.15134	1.11183

1.08121	1.08252	1.08046	1.12625	1.04935	1.13685	1.00271	1.11993

1.08121	1.08252	1.08046	1.12625	1.04935	1.13685	1.00271	1.11993

1.25432	1.18746	1.25338	1.26726 NA	1.14249	1.12329	1.14507

0.996114 0.974892 0.953635 0.952381 0.945497 0.969718 0.942451 0.943734

1.04797	1.00945	1.06059	1.03028	1.0717	1.01995 0.927505 0.999774

1.01055	1.03685	1.06379	1.05533	1.02769 0.990604	1.02201	1.06818

1.04223	1.07249	1.11418	1.10747	1.0232 0.989585 0.963546	1.01369

NA	NA	NA	NA	NA	NA	NA	NA NA	NA	NA	NA	NA	NA	NA	NA

0.99938 0.961263	1.01269 NA	NA	NA	1.06988 0.987691

0.99938 0.961263	1.01269 NA	NA	NA	1.06988 0.987691

1.00347	1.01035	1.00185 0.989941 0.998837	1.00891 0.971261 0.971628

1.13081	1.15821	1.14983	1.10749	1.20845	1.15373	1.21475	1.01974

NA	NA	NA	NA	NA	NA	NA	NA

1.04633	1.04612 0.994991	1.02272 0.998646	1.05387 0.950174	1.02229

NA	NA	NA	NA	NA	NA	NA	NA NA	NA	NA	NA	NA	NA	NA	NA

1.04924	1.05496	1.05051	1.06582 NA	NA	NA	NA

1.06004	1.04664	1.05145	1.03435 NA	NA	NA	NA

1.08622	1.06747	1.03462	1.07607 NA	NA	NA	NA

1.08631	1.09409	1.02828	1.06279 NA	NA	NA	NA

1.08872	1.09246	1.14743	1.04709	1.16939	1.18856	1.15843	1.06745

1.08872	1.09246	1.14743	1.04709	1.16939	1.18856	1.15843	1.06745

1.10921	1.16302	1.15108	1.05357	1.12707	1.23018	1.21948	1.03779

1.15445	1.20366	1.18138	1.13705	1.08999	1.25436	1.16538	1.09273

1.17759	1.13887	1.14223	1.16324 NA	1.0744 NA	1.03271

0.989671	1.01466	1.00834 0.969228 NA	NA	NA	NA NA	NA	NA	NA	NA	NA	NA	NA NA	NA	NA	NA	NA	NA	NA	NA NA	NA	NA	NA	NA	NA	NA	NA

1.04179	1.04519	1.08763	1.05354	1.03395	1.06913 0.985202	1.07554

| 0.901692 | | 0.91105 | | 0.969486 | | 0.990091 | | 0.963105 | | 0.970538 | | 1.08557 | | 1.01005 | |
| --- | --- | --- | --- | --- | --- | --- | --- | --- | --- | --- | --- | --- | --- | --- | --- |
| 0.901692 | | 0.91105 | | 0.969486 | | 0.990091 | | 0.963105 | | 0.970538 | | 1.08557 | | 1.01005 | |
| 0.901692 | | 0.91105 | | 0.969486 | | 0.990091 | | 0.963105 | | 0.970538 | | 1.08557 | | 1.01005 | |
| 1.02322 | | 1.01505 | | 1.00481 | | 1.01194 | | 1.03841 | | 1.07941 | | 1.04801 | | 0.984266 | |
| 1.02293 | | 1.03288 | | 1.02223 | | 0.975247 | | 1.04252 | |  | 1.0045 | 1.06543 | | 0.923786 | |
| 1.02095 | | 1.01286 | | 0.99132 | | 0.935047 | | 1.01347 | | 1.01294 | | 1.03836 | | 0.927011 | |
| 1.09198 | | 1.10121 | | 1.03954 | | 1.11254 | | 1.05811 | | 1.12978 | | 1.06466 | | 0.983882 | |
| 1.07394 | | 1.05584 | | 1.05722 | | 1.08119 | | 1.00055 | | 1.06243 | | 1.05887 | | 1.01618 | |
| 1.06032 | | 1.03978 | |  | 1.0101 | 1.00466 | |  | 1.0005 | 1.03508 | | 1.03906 | |  | 1.0339 |
| 1.10444 | | 1.09393 | | 1.01266 | | 1.08694 | | 1.08825 | |  | 1.119 | 1.07569 | | 1.02025 | |
| 1.10895 | | 1.11229 | | 1.05783 | | 1.11656 | | 1.08714 | | 1.07216 | | 1.07021 | | 1.04888 | |
| 1.10573 | | 1.08551 | | 1.03498 | | 1.05823 | | 1.05022 | | 1.05228 | | 1.10083 | | 1.02057 | |
| 1.12383 | | 1.13168 | | 1.10395 | | 1.09281 | | 1.17519 | | 1.08889 | | 1.08878 | | 1.00731 | |
| 1.06472 | | 1.08501 | | 1.05752 | | 1.08181 | | 1.07906 | | 1.04657 | | 1.03793 | | 0.955824 | |
|  | 1.0761 | 1.08395 | | 1.07518 | | 1.06607 | | 1.07119 | | 1.06601 | |  | 1.072 | 1.02827 | |
| NA |  | NA |  | NA |  | NA |  | NA |  | NA |  | NA |  | NA |  |
| 1.05878 | | 1.09115 | | 1.10438 | | 1.01535 | | 1.13059 | |  | 1.1429 | 1.25015 | | 1.09206 | |
| 1.06654 | | 1.09207 | | 1.08787 | | 1.04118 | | 1.10612 | | 1.08006 | | 1.08186 | | 1.04421 | |
| 1.13307 | | 1.12557 | | 1.10047 | | 1.03779 | | 1.14286 | |  | 1.0804 | 1.06375 | | 1.00931 | |
| 1.16284 | | 1.15385 | | 1.20193 | |  | 1.1309 | 1.14055 | | 1.08271 | | 1.12717 | | 1.06299 | |
|  | 1.0741 | 1.06606 | | 1.01037 | | 0.985912 | | 1.04142 | | 1.02046 | | 1.07498 | | 0.937116 | |
| NA |  | NA |  | NA |  | NA |  | NA |  | NA |  | NA |  | NA |  |
| NA |  | NA |  | NA |  | NA |  | NA |  | NA |  | NA |  | NA |  |
| 1.09656 | | 1.08564 | | 1.03648 | | 1.03412 | | 1.05034 | | 1.04121 | | 1.06842 | | 1.02447 | |
| 1.03557 | |  | 1.068 | 1.09174 | | 1.08108 | | 1.06211 | | 1.10105 | | 1.24074 | | 1.23602 | |
| 1.10584 | | 1.08148 | | 1.05053 | | 1.01527 | |  | 1.0756 | 1.04515 | | 1.08337 | | 1.05521 | |
| 1.11837 | | 1.11682 | | 1.07226 | | 1.08473 | | 1.10049 | | 1.09431 | | 1.06028 | | 1.00708 | |
|  | 1.0728 | 1.07738 | |  | 1.0941 | 1.05761 | | 1.12493 | | 1.05402 | | 1.05983 | | 1.04202 | |
|  | 1.0646 | 1.08874 | | 1.09607 | | 1.08083 | | 1.13821 | | 1.07688 | |  | 0.9847 |  | 1.0241 |
|  | 1.0646 | 1.08874 | | 1.09607 | | 1.08083 | | 1.13821 | | 1.07688 | |  | 0.9847 |  | 1.0241 |
| 1.12501 | | 1.12033 | | 1.07196 | | 1.04216 | | 1.09887 | | 1.07202 | | 1.05745 | | 1.03471 | |
| 1.10502 | | 1.10665 | | 1.05518 | | 1.10285 | | 1.09148 | | 1.07378 | | 1.09886 | |  | 1.0384 |
| 1.07609 | | 1.09041 | | 1.10806 | | 1.06734 | | 1.03907 | | 1.06345 | | 1.06063 | | 1.07637 | |
| 1.06465 | | 1.03814 | | 1.03488 | | 1.00365 | |  | 1.0216 | 1.00576 | | 1.04419 | | 0.924171 | |
| NA |  | NA |  | NA |  | NA |  | NA |  | NA |  | NA |  | NA |  |
| 1.00873 | | 1.03053 | | 0.999471 | | 1.02287 | | 1.02745 | | 1.02857 | | 0.944795 | | 1.02482 | |
| 1.10695 | | 1.10333 | | 1.06365 | |  | 1.0601 | 1.07695 | | 1.06758 | | 1.05291 | | 1.07502 | |
| 1.09701 | | 1.09222 | | 1.03249 | | 1.02512 | | 1.08137 | | 1.05417 | | 1.05723 | | 1.02234 | |
| 1.16855 | | 1.14202 | | 1.24404 | |  | 1.1334 | 1.22791 | | 1.15562 | | 1.13603 | | 1.03279 | |

| 1.02594 | | 1.02431 | |  | 1.0658 | 1.02407 | | 1.04285 | 0.998793 | | 1.04047 | | 1.06643 |
| --- | --- | --- | --- | --- | --- | --- | --- | --- | --- | --- | --- | --- | --- |
| 1.03226 | | 1.01484 | | 0.995104 | | 0.988285 | | 0.950163 | 0.949898 | | 0.913693 | | 1.11015 |
|  | 1.0212 | 1.02988 | |  | 1.0811 |  | 1.013 | 1.00836 | 1.02169 | |  | 1.0076 | 1.03296 |
| 1.03502 | | 1.04416 | | 1.08313 | | 1.02734 | | 1.02005 | 0.996721 | | 0.970137 | | 0.982154 |
| NA |  | NA |  | NA |  | NA |  | NA | NA |  | NA |  | NA |
| NA |  | NA |  | NA |  | NA |  | NA | NA |  | NA |  | NA |
| NA |  | NA |  | NA |  | NA |  | NA | NA |  | NA |  | NA |
| NA |  | NA |  | NA |  | NA |  | NA | NA |  | NA |  | NA |
| NA |  | NA |  | NA |  | NA |  | NA | NA |  | NA |  | 0.985327 |
| NA |  | NA |  | NA |  | NA |  | NA | NA |  | NA |  | 0.981738 |
| NA |  | NA |  | NA |  | NA |  | NA | NA |  | NA |  | NA |
| NA |  | NA |  | NA |  | NA |  | NA | NA |  | NA |  | NA |
| NA |  | NA |  | NA |  | NA |  | NA | NA |  | NA |  | NA |
| NA |  | NA |  | NA |  | NA |  | NA | NA |  | NA |  | NA |
| NA |  | NA |  | NA |  | NA |  | NA | NA |  | NA |  | 0.997611 |
| 1.03755 | | 1.01774 | | 0.999427 | | NA |  | 1.03628 | NA |  | NA |  | 0.989532 |
| NA |  | NA |  | NA |  | NA |  | NA | NA |  | NA |  | NA |
| NA |  | NA |  | NA |  | NA |  | NA | NA |  | NA |  | 0.978811 |
| 1.04436 | |  | 1.0344 | 1.01095 | | NA |  | 1.06472 | 1.02817 | | NA |  | 1.03531 |
| 1.02773 | | NA |  | 1.01329 | | NA |  | 1.05481 | NA |  | NA |  | 0.973256 |
| NA |  | NA |  | NA |  | NA |  | NA | NA |  | NA |  | NA |
| 1.11742 | | 1.13204 | | 1.09064 | | 1.10351 | | 1.10811 | 1.06938 | | 1.06482 | | 1.11297 |
| 0.97046 | | 0.934012 | | 0.981163 | | 0.926685 | | 0.926684 | 1.03172 | | NA |  | NA |
| 0.97046 | | 0.934012 | | 0.981163 | | 0.926685 | | 0.926684 | 1.03172 | | NA |  | NA |
| 0.981142 | | NA |  | 0.983857 | | NA |  | 1.02241 |  | 1.0895 | NA |  | NA |
| 0.981142 | | NA |  | 0.983857 | | NA |  | 1.02241 |  | 1.0895 | NA |  | NA |
| 0.983693 | | 0.982848 | | 1.03378 | | NA |  | 1.02479 | 1.04283 | | NA |  | NA |
| 0.981658 | | 1.00629 | | 1.05296 | | 0.987852 | | 1.03249 | 1.06625 | | NA |  | NA |
| NA |  | NA |  | 1.02771 | | NA |  | 0.967139 | 1.10836 | | NA |  | NA |
| NA |  | NA |  | 1.07523 | | NA |  | 1.05935 | 1.07465 | | NA |  | NA |
| NA |  | NA |  | 1.07523 | | NA |  | 1.05935 | 1.07465 | | NA |  | NA |
| NA |  | NA |  | 1.07523 | | NA |  | 1.05935 | 1.07465 | | NA |  | NA |
| NA |  | NA |  | NA |  | NA |  | NA | NA |  | NA |  | NA |
| NA |  | NA |  | NA |  | NA |  | NA | NA |  | NA |  | NA |
| NA |  | NA |  | NA |  | NA |  | NA | NA |  | NA |  | NA |
| NA |  | NA |  | NA |  | NA |  | NA | NA |  | NA |  | NA |
| 0.979552 | | 0.999375 | | 0.946342 | | 0.921757 | | 1.01822 | 0.985883 | | 0.933692 | | 1.05393 |
| 1.07971 | | 1.09495 | | 1.08666 | | 1.11171 | | 1.09987 | 1.13107 | | 1.27931 | | 1.08135 |
| 1.07971 | | 1.09495 | | 1.08666 | | 1.11171 | | 1.09987 | 1.13107 | | 1.27931 | | 1.08135 |

| 1.13666 | 1.14299 | | 1.19357 | | 1.13343 | 1.13943 | | 1.14848 | | 1.30166 | | 1.14934 | |
| --- | --- | --- | --- | --- | --- | --- | --- | --- | --- | --- | --- | --- | --- |
| NA | NA |  | NA |  | 1.10506 | NA |  | NA |  | 1.44051 | | NA |  |
| 1.05115 | 1.05347 | | 1.05222 | | 1.11463 |  | 1.0338 | 1.08713 | |  | 1.1883 | 1.04045 | |
| NA | NA |  | 0.944424 | | 0.994291 | 0.917355 | | 1.04822 | | 1.04829 | |  | 1.052 |
| 1.08086 | 1.08983 | | 1.07166 | | 1.07747 | 1.02694 | |  | 1.0341 | 1.01661 | | 1.04311 | |
| 1.09327 |  | 1.0666 | 1.04292 | | 1.02888 | NA |  | NA |  | NA |  | NA |  |
| NA | NA |  | NA |  | NA | NA |  | NA |  | NA |  | NA |  |
| NA | NA |  | NA |  | NA | NA |  | NA |  | NA |  | 0.90569 | |
| NA | NA |  | 0.959819 | | 0.932592 | NA |  | 0.922998 | | 1.02632 | | 0.870869 | |
| NA | 0.983291 | |  | 1.0148 | 1.03113 | 1.02494 | | 0.995951 | | NA |  |  | 1.0069 |
| 0.895487 | 0.903702 | | 0.968228 | | 0.980705 | 0.966189 | | 0.965952 | | 1.06535 | | 0.926558 | |
| NA | NA |  | NA |  | NA | NA |  | NA |  | NA |  | NA |  |
| NA | NA |  | NA |  | NA | NA |  | NA |  | NA |  | NA |  |
| NA | NA |  | NA |  | NA | NA |  | NA |  | NA |  | NA |  |
| NA | NA |  | NA |  | NA | NA |  | NA |  | NA |  | NA |  |
| NA | NA |  | NA |  | NA | NA |  | NA |  | NA |  | NA |  |
| NA | NA |  | NA |  | NA | NA |  | NA |  | NA |  | NA |  |
| NA | NA |  | NA |  | NA | NA |  | NA |  | NA |  |  | 1.1119 |
| NA | NA |  | NA |  | NA | NA |  | NA |  | NA |  | NA |  |
| 1.06247 | 1.04624 | | 1.08032 | | NA | 1.06331 | | NA |  | NA |  | 0.970349 | |
| NA | NA |  | NA |  | NA | NA |  | NA |  | NA |  | NA |  |
| NA | NA |  | NA |  | NA | NA |  | NA |  | NA |  | 0.996453 | |
| NA | NA |  | NA |  | NA | NA |  | NA |  | NA |  | 1.11562 | |
| NA | NA |  | NA |  | NA | NA |  | NA |  | NA |  | 1.04478 | |
| NA | NA |  | NA |  | NA | NA |  | NA |  | NA |  | NA |  |
| 0.993344 | 0.979661 | | 1.00222 | | NA | 0.993284 | | 0.984405 | | NA |  | 1.05473 | |
| NA | NA |  | NA |  | NA | NA |  | NA |  | NA |  | NA |  |
| NA | NA |  | NA |  | NA | NA |  | NA |  | NA |  | NA |  |
| NA | NA |  | NA |  | NA | NA |  | NA |  | NA |  | NA |  |
| NA | NA |  | NA |  | NA | NA |  | NA |  | NA |  | NA |  |
| 1.04224 | 1.02387 | | 1.04833 | | NA | 1.00111 | | 1.02602 | | NA |  | 1.04598 | |
| NA | NA |  | NA |  | NA | NA |  | NA |  | NA |  | NA |  |
| NA | NA |  | NA |  | NA | NA |  | NA |  | NA |  | 1.01652 | |
| NA | NA |  | NA |  | NA | NA |  | NA |  | NA |  | 1.04422 | |
| NA | NA |  | NA |  | NA | NA |  | NA |  | NA |  | NA |  |
| 1.06575 | NA |  | 1.07234 | | NA | NA |  | NA |  | NA |  | 1.03212 | |
| NA | NA |  | NA |  | NA | NA |  | NA |  | NA |  | NA |  |
| 1.05189 | NA |  | 1.00662 | | NA | NA |  | NA |  | NA |  | NA |  |
| NA | NA |  | NA |  | NA | NA |  | NA |  | NA |  | NA |  |

| NA |  | NA |  | NA |  | NA | NA |  | NA | NA |  |  | 1.0261 |
| --- | --- | --- | --- | --- | --- | --- | --- | --- | --- | --- | --- | --- | --- |
| 1.01661 | | 1.01238 | | 1.03497 | | NA | 1.04007 | | 0.96925 | NA |  | 0.998691 | |
| NA |  | NA |  | NA |  | NA | NA |  | NA | NA |  | NA |  |
| NA |  | NA |  | NA |  | NA | NA |  | NA | NA |  | NA |  |
| NA |  | NA |  | NA |  | NA | NA |  | NA | NA |  | NA |  |
| 1.09524 | | 1.08884 | | 1.09732 | | NA | 1.10239 | | NA | NA |  |  | 1.1014 |
| NA |  | NA |  | NA |  | NA | NA |  | NA | NA |  | NA |  |
| NA |  | NA |  | NA |  | NA | NA |  | NA | NA |  | NA |  |
| NA |  | NA |  | NA |  | NA | NA |  | NA | NA |  | NA |  |
| 1.06896 | | NA |  | 1.02623 | | NA | NA |  | NA | NA |  | 0.985591 | |
| 1.00654 | | 0.999942 | | 0.976156 | | 0.986687 | 0.995441 | | 1.01759 | 0.958141 | |  | 0.9502 |
| 1.22601 | | 1.05228 | |  | 1.0197 | 1.01959 | 0.994309 | | 1.25869 | NA |  | NA |  |
| 0.989905 | | 0.996808 | | NA |  | NA | 1.02716 | | NA | NA |  | NA |  |
| 0.996112 | | 0.989645 | | 0.979103 | | 0.945035 | 0.988549 | | 1.02989 | 1.02031 | | NA |  |
| 1.12064 | |  | 1.0937 | 1.13095 | | 0.984351 | 1.09024 | | 1.15895 | 0.994787 | | 1.23026 | |
| 1.03399 | | 1.04129 | | 1.08549 | | 1.03931 | 1.02138 | | 1.10029 | 1.04601 | | 1.04712 | |
| 1.14526 | | 1.12787 | | 1.08935 | | 1.03948 | NA |  | NA | 1.22687 | | NA |  |
| 1.00082 | | 1.00973 | | 0.997323 | | NA | 0.980547 | | 0.96534 | NA |  | NA |  |
| 1.03533 | | 1.02053 | | 1.03428 | | 1.00399 |  | 1.047 | 1.03123 | 1.02638 | | 0.885257 | |
| 1.11793 | | 1.08464 | | 1.13977 | | 1.02658 | 1.05566 | | 1.18143 | 0.869539 | | NA |  |
|  | 1.0312 | 1.02601 | | 1.06239 | | 1.05089 | 1.02583 | | 1.00344 | 0.999569 | | 0.98318 | |
| 1.02292 | | 1.01942 | | 1.07953 | | 0.986518 | 0.979425 | | 1.03582 | 0.982877 | | 0.971921 | |
| 1.03092 | | 1.05302 | | 1.05403 | | 1.04737 | 1.02632 | | 1.00122 | 1.02404 | | 1.00313 | |
| 1.01279 | | 1.01632 | | 1.03366 | | 1.03096 | NA |  | 0.993079 | 1.04243 | | 1.00701 | |
| 1.02899 | |  | 1.0611 | NA |  | NA | 1.04201 | | 0.999835 | 1.00171 | | 0.976628 | |
| 1.04761 | |  | 1.036 | 1.04068 | | 1.08529 | 1.11669 | | 1.02316 | 1.05101 | | 1.01189 | |
| 1.01524 | | 1.03513 | | 1.02524 | | 1.03893 | 1.01504 | | 1.02323 | 1.13709 | | 0.939949 | |
| 0.969084 | | 0.995672 | | 0.95444 | | 1.01852 | 1.08291 | | 1.02581 | 1.16382 | | 0.969072 | |
| 0.969084 | | 0.995672 | | 0.95444 | | 1.01852 | 1.08291 | | 1.02581 | 1.16382 | | 0.969072 | |
| NA |  | 1.01517 | | NA |  | NA | 1.01932 | | 1.02312 | 1.16546 | | 0.929901 | |
| 1.02487 | | 1.03284 | | 1.04097 | | 0.988691 | 1.06271 | | 1.05202 | 1.00036 | | 1.00175 | |
|  | 1.0403 | 1.07536 | | NA |  | NA | 1.07121 | | 1.01344 |  | 1.0413 |  | 0.9631 |
|  | 1.0403 | 1.07536 | | NA |  | NA | 1.07121 | | 1.01344 |  | 1.0413 |  | 0.9631 |
|  | 1.0403 | 1.07536 | | NA |  | NA | 1.07121 | | 1.01344 |  | 1.0413 |  | 0.9631 |
| 1.00304 | | 1.00742 | | 1.02738 | | 1.00361 |  | 1.0182 | 1.01195 | 1.01245 | | 0.966653 | |
| 0.977325 | | 0.99784 | | 1.00489 | | 1.00826 | 0.988762 | | 1.05307 | 0.994467 | | 0.950282 | |
| NA |  | NA |  | NA |  | NA | NA |  | 1.04586 | 1.08007 | | 0.897447 | |
| NA |  | NA |  | NA |  | NA | NA |  | NA | NA |  |  | 1.05 |
| 1.04319 | | 1.09365 | | 1.05982 | | NA | 1.02307 | | NA | NA |  | NA |  |

| NA |  | NA | NA |  | NA |  | NA |  | NA |  | NA |  | NA |  |
| --- | --- | --- | --- | --- | --- | --- | --- | --- | --- | --- | --- | --- | --- | --- |
| NA |  | NA | NA |  | NA |  | NA |  | NA |  | NA |  | NA |  |
| NA |  | NA | NA |  | NA |  | NA |  | NA |  | NA |  | NA |  |
| NA |  | NA | NA |  | NA |  | NA |  | NA |  | NA |  | NA |  |
| 0.973044 | | 0.955134 | 0.951809 | | 0.967355 | | 0.939768 | | 0.977402 | | 0.992473 | | 0.901702 | |
| 1.04021 | | 0.989938 | 1.04689 | | 1.04093 | | 0.985165 | | 1.01971 | | 1.03885 | | 1.07508 | |
| 0.991728 | | 0.988876 | 1.00687 | | 0.987978 | | 1.03751 | | 1.02526 | | 0.990903 | | 1.00112 | |
| 1.03817 | | 1.05192 | 1.02572 | | 1.10775 | |  | 1.0232 | 1.05242 | | 1.02435 | | 1.00405 | |
| 0.920876 | | 0.94601 |  | 0.9761 | 0.93663 | | 0.970424 | | 0.938424 | | 0.914521 | | 1.01024 | |
|  | 1.0323 | 1.00635 |  | 1.0355 | 1.02723 | | 1.01292 | | 0.94461 | | 0.987613 | | 1.07071 | |
| 1.05435 | | 1.05093 | 1.06429 | | 1.03042 | |  | 1.0905 | 1.10602 | | 1.15623 | | 1.04228 | |
| 1.12998 | | 1.13198 | 1.17272 | | 1.22024 | | 1.09655 | | 1.15688 | |  | 1.0825 |  | 1.2008 |
| 1.18426 | | 1.15694 | 1.17323 | | 1.19684 | | 1.19094 | | 1.21415 | | 1.13375 | | 1.23641 | |
| 1.00794 | | 1.00916 | 0.944456 | | 0.967516 | | 1.03038 | | 0.978353 | | 0.868106 | | 0.961664 | |
| NA |  | NA | NA |  | NA |  | NA |  | NA |  | NA |  | 0.988848 | |
| NA |  | NA | NA |  | NA |  | NA |  | NA |  | NA |  | NA |  |
| 1.08078 | | 1.10117 | 1.13281 | | 1.10063 | | 1.07659 | | 1.15135 | | 1.09151 | | 0.994993 | |
| NA |  | NA | NA |  | NA |  | NA |  | NA |  | NA |  | NA |  |
| 0.958395 | | 0.96508 | 0.992474 | | 0.971707 | | 0.923987 | | 1.00546 | | 0.921395 | | 0.970545 | |
| NA |  | NA | NA |  | NA |  | NA |  | NA |  | NA |  | NA |  |
| NA |  | NA | NA |  | NA |  | NA |  | NA |  | NA |  | NA |  |
| 1.07514 | | 1.05997 | 1.09737 | | 1.12216 | | 1.09686 | | 1.14245 | | 1.14174 | | 1.15329 | |
| 1.04529 | | 1.04457 | 1.08314 | | 1.06044 | | 1.12971 | | 1.09534 | | 1.24495 | | 1.02825 | |
| 1.04529 | | 1.04457 | 1.08314 | | 1.06044 | | 1.12971 | | 1.09534 | | 1.24495 | | 1.02825 | |
| 1.04529 | | 1.04457 | 1.08314 | | 1.06044 | | 1.12971 | | 1.09534 | | 1.24495 | | 1.02825 | |
| 1.04529 | | 1.04457 | 1.08314 | | 1.06044 | | 1.12971 | | 1.09534 | | 1.24495 | | 1.02825 | |
|  | 1.0664 | 1.04876 | 1.10423 | | 1.08853 | | 1.02009 | |  | 1.0068 | 1.11298 | | 0.983578 | |
|  | 1.0664 | 1.04876 | 1.10423 | | 1.08853 | | 1.02009 | |  | 1.0068 | 1.11298 | | 0.983578 | |
|  | 1.0664 | 1.04876 | 1.10423 | | 1.08853 | | 1.02009 | |  | 1.0068 | 1.11298 | | 0.983578 | |
|  | 1.0664 | 1.04876 | 1.10423 | | 1.08853 | | 1.02009 | |  | 1.0068 | 1.11298 | | 0.983578 | |
|  | 1.1328 | 1.13663 | 1.10763 | | 1.01119 | | 1.12197 | | 1.20905 | | 1.12567 | | 1.19961 | |
|  | 1.1328 | 1.13663 | 1.10763 | | 1.01119 | | 1.12197 | | 1.20905 | | 1.12567 | | 1.19961 | |
| 1.03904 | | 1.01636 | 1.04404 | |  | 1.0346 | 1.03294 | |  | 1.018 | 1.03263 | | 1.08312 | |
| 1.03904 | | 1.01636 | 1.04404 | |  | 1.0346 | 1.03294 | |  | 1.018 | 1.03263 | | 1.08312 | |
| 1.12019 | | 1.11868 | 1.14524 | | 1.08162 | | 1.19999 | | 1.18969 | | 1.34592 | | 1.06146 | |
| 0.956853 | | 0.92907 | 0.943211 | | 0.934269 | | 0.95081 | | 0.987318 | | 0.955177 | | 0.897612 | |
| 0.956853 | | 0.92907 | 0.943211 | | 0.934269 | | 0.95081 | | 0.987318 | | 0.955177 | | 0.897612 | |
| 1.00062 | | 0.986475 | 1.00277 | | 0.977039 | | 0.999032 | | 1.00371 | | 0.972811 | | 0.980167 | |
| 1.00062 | | 0.986475 | 1.00277 | | 0.977039 | | 0.999032 | | 1.00371 | | 0.972811 | | 0.980167 | |

| 0.982473 | | 0.95988 | | 0.977932 | | 0.991691 | | 0.981746 | | 0.999549 | | 0.985367 | | 1.01266 | |
| --- | --- | --- | --- | --- | --- | --- | --- | --- | --- | --- | --- | --- | --- | --- | --- |
| 0.993793 | | 0.974148 | | 0.959016 | | 0.94368 | | 0.950285 | | 1.00955 | | 0.935177 | | 0.971081 | |
| 1.01718 | | 0.991172 | | 1.01497 | | 0.992768 | | 1.00159 | | 0.949473 | | 1.06019 | | 0.941225 | |
| 0.995346 | | 1.01752 | | 0.995435 | | 1.00361 | | 1.05357 | |  | 1.0265 | 0.940998 | | 0.934968 | |
|  | 1.0204 | 0.997123 | | 0.984519 | | 1.03885 | | 1.03698 | | 0.999481 | | 1.00462 | |  | 1.0017 |
|  | 1.0219 | 1.03041 | | 1.05844 | | 1.02789 | | 1.02281 | | 1.00584 | | 0.944112 | | 1.03552 | |
| 1.02585 | | 1.01441 | | 1.03089 | | 1.05423 | | 1.01429 | | 0.991152 | | 1.06469 | | 1.06465 | |
| 0.999817 | | 1.01714 | | 0.976424 | |  | 1.0122 |  | 1.0535 |  | 1.0253 | 1.02826 | | 1.04644 | |
| 1.01188 | | 1.03366 | | 0.973812 | | 1.02965 | | 1.04927 | | 1.01088 | | 1.01192 | |  | 1.0254 |
| NA |  | NA |  | NA |  | 0.991694 | | NA |  | NA |  | 0.958126 | | 0.983539 | |
| 0.974995 | | 1.01276 | | 0.963068 | | 0.967293 | | 1.00937 | | 0.991081 | | 1.00995 | |  | 1.0558 |
| 1.02496 | |  | 1.0321 | 1.02867 | | 0.996564 | | 1.02676 | | 1.01408 | | 1.00511 | | 0.981579 | |
| NA |  | NA |  | NA |  | NA |  | NA |  | NA |  | 0.952903 | | NA |  |
| 1.04223 | | 1.05227 | |  | 1.0316 | 1.01531 | | 1.07821 | | 1.02437 | | 0.922786 | | 1.01625 | |
| NA |  | NA |  | NA |  | NA |  | NA |  | NA |  | 0.986867 | | NA |  |
| NA |  | NA |  | NA |  | NA |  | NA |  | NA |  | NA |  | NA |  |
| NA |  | NA |  | NA |  | NA |  | NA |  | NA |  | NA |  | NA |  |
| 0.97264 | | 0.964334 | | 1.02473 | | NA |  | 0.974517 | |  | 1.018 | 1.02783 | | 1.11652 | |
| NA |  | NA |  | NA |  | NA |  | NA |  | NA |  | NA |  | NA |  |
| NA |  | NA |  | NA |  | NA |  | NA |  | NA |  | NA |  | NA |  |
| NA |  | NA |  | NA |  | NA |  | NA |  | NA |  | NA |  | NA |  |
| NA |  | NA |  | NA |  | NA |  | NA |  | NA |  | NA |  | NA |  |
| NA |  | NA |  | NA |  | NA |  | NA |  | NA |  | NA |  | NA |  |
| 1.03167 | | 1.00806 | | 1.03762 | | NA |  |  | 1.0301 | 0.971361 | | NA |  | NA |  |
| 1.01177 | | 1.00995 | | 1.03267 | | 1.00287 | | 1.04652 | | 1.02694 | | 0.969253 | | NA |  |
| NA |  | NA |  | NA |  | NA |  | NA |  | NA |  | NA |  | NA |  |
| NA |  | NA |  | NA |  | NA |  | NA |  | NA |  | NA |  | NA |  |
| NA |  | NA |  | NA |  | NA |  | NA |  | NA |  | NA |  | NA |  |
| 1.09843 | | 1.10055 | | 1.12744 | | 1.06465 | | 1.14997 | |  | 1.1526 | 1.25057 | | 1.08929 | |
|  | 1.0639 | 1.04901 | | 1.09009 | | 1.09923 | | 1.03679 | |  | 1.0934 | 1.07853 | | 1.06743 | |
|  | 1.0639 | 1.04901 | | 1.09009 | | 1.09923 | | 1.03679 | |  | 1.0934 | 1.07853 | | 1.06743 | |
| NA |  | NA |  | NA |  | NA |  | NA |  | NA |  | NA |  | 0.87037 | |
| NA |  | NA |  | NA |  | 1.10374 | | NA |  | 1.04332 | | 1.09684 | | 0.960553 | |
| NA |  | NA |  | NA |  | NA |  | NA |  | NA |  | NA |  | 1.02977 | |
| NA |  | 0.980339 | | NA |  | NA |  | 0.941558 | | 1.01584 | | 0.998832 | | 1.11518 | |
| 1.07209 | |  | 1.0971 | 1.11601 | | 1.08809 | | 1.06813 | | 1.12423 | | 1.10095 | | 0.954005 | |
| 0.979676 | | 1.00666 | | 0.984332 | | 0.980743 | | NA |  | 1.01678 | |  | 1.0475 | 1.02061 | |
| NA |  | NA |  | NA |  | NA |  | NA |  | NA |  | NA |  | NA |  |
| NA |  | 1.03608 | | NA |  |  | 1.0391 | NA |  | 1.07435 | | 1.03587 | | 1.08467 | |

| 0.964424 | | 0.965836 | | 0.937981 | | NA |  | 0.985286 | | 1.02675 | | 1.03659 | | 1.02172 | |
| --- | --- | --- | --- | --- | --- | --- | --- | --- | --- | --- | --- | --- | --- | --- | --- |
| NA |  | NA |  | NA |  | NA |  | NA |  | NA |  | NA |  | 0.949612 | |
| NA |  | NA |  | NA |  | NA |  | NA |  | NA |  | NA |  | 0.917118 | |
| NA |  | NA |  | NA |  | NA |  | NA |  | NA |  | NA |  | 1.01611 | |
| NA |  | NA |  | NA |  | NA |  | NA |  | NA |  | 0.952078 | | 1.03468 | |
| 1.02255 | | 1.00015 | | 1.01546 | | 1.04264 | | 1.01629 | | 1.01427 | | 1.01014 | | 1.01899 | |
| NA |  | NA |  | NA |  | NA |  | NA |  | NA |  | NA |  | NA |  |
| NA |  | NA |  | NA |  | NA |  | NA |  | NA |  | NA |  | NA |  |
| NA |  | NA |  | NA |  | NA |  | NA |  | NA |  | NA |  | NA |  |
| NA |  | NA |  | NA |  | NA |  | NA |  | NA |  | NA |  | NA |  |
| 0.976688 | | 1.02159 | | 1.00644 | | 0.92159 | | 0.976454 | | 0.946491 | | 1.09722 | | 1.02111 | |
| 1.05433 | | 1.07754 | | 1.10959 | | 1.10633 | | NA |  | 1.07322 | | 1.10977 | | 1.10645 | |
|  | 1.069 |  | 1.1019 |  | 1.0864 | 1.08785 | |  | 1.0473 | 1.05866 | | 1.03767 | | 1.10828 | |
| NA |  | NA |  | NA |  | NA |  | NA |  | NA |  | NA |  | NA |  |
| NA |  | NA |  | NA |  | 1.14544 | | NA |  | NA |  | 1.10114 | | 1.01238 | |
|  | 1.0621 | 1.09571 | |  | 1.1449 | 1.09989 | | 0.990347 | | 1.01881 | | 1.08813 | | 1.02773 | |
| 1.00769 | | 1.01609 | | 0.985857 | | 1.07965 | | 1.06919 | | 1.00293 | | 0.960601 | | 1.04444 | |
| NA |  | NA |  | NA |  | 1.01016 | | NA |  | 1.02668 | | 1.06305 | | 0.99154 | |
| NA |  | NA |  | NA |  | NA |  | NA |  | NA |  | NA |  | NA |  |
| NA |  | 0.983104 | | 0.999914 | | 0.991501 | | 0.997241 | | 0.975215 | | 1.02763 | | 1.00607 | |
| 0.978862 | | 0.994088 | | 1.02743 | | 0.984988 | | 1.01167 | | 0.983932 | | 1.02331 | | 0.952641 | |
| 0.94218 | | 0.949011 | | 0.940704 | | 0.947186 | | 0.969718 | | 1.01381 | | 1.00479 | | 1.02978 | |
| 0.922584 | | 0.930492 | | 0.908281 | | 0.919842 | | 0.943615 | | 0.938243 | |  | 1.0421 | 1.05175 | |
| 1.17165 | | 1.19212 | | 1.20644 | | 1.17859 | | 1.20758 | | 1.24828 | | 1.10361 | | 1.11044 | |
| 1.05743 | | 1.07509 | | 1.06563 | |  | 1.0437 | 1.05288 | | 0.995176 | | 1.02936 | | 0.982023 | |
| 0.997335 | | 1.01873 | | 1.03493 | | 1.06369 | | NA |  |  | 0.9514 | 1.07158 | | 1.13374 | |
| 1.06745 | | 1.04998 | | 1.02725 | | 0.99512 | | 1.08805 | | 1.07647 | | 0.95323 | | 0.992091 | |
| 1.02888 | | 1.03432 | | 1.03582 | | 1.08285 | | 1.05327 | | 1.00868 | | 0.967607 | | 0.95698 | |
| 1.08166 | | 1.07152 | | 1.02185 | | 0.980554 | | 0.989497 | | 1.10838 | |  | 1.0505 | 1.08186 | |
|  | 1.0523 | 1.04445 | |  | 1.0384 | 1.04764 | | 1.05154 | | 1.01055 | | 0.970195 | | 0.94729 | |
| 1.12609 | |  | 1.0121 | 0.982906 | | 0.985075 | |  | 1.0378 | 1.03817 | | 1.04215 | | 0.982609 | |
| 1.05272 | | 1.03737 | |  | 1.0795 | 1.09844 | | 1.06891 | | 1.02685 | | 1.02571 | | 0.914717 | |
|  | 1 | 0.945856 | | 0.960929 | | 0.911647 | |  | 0.8879 | 1.00701 | | 0.891616 | | 0.994792 | |
| 1.05163 | | 1.07407 | | 1.04208 | | 1.09073 | | 1.03367 | | 1.04139 | | 1.01491 | | 1.01501 | |
| 1.02283 | | 0.959245 | | 0.960784 | | 0.953668 | | 1.01017 | | 1.02922 | | 0.982206 | |  | 1.0873 |
| 0.967866 | | 0.955224 | | 0.932296 | | 0.95086 | | NA |  | NA |  | 0.981395 | | 0.984424 | |
| NA |  | NA |  | NA |  | NA |  | NA |  | NA |  | NA |  | NA |  |
| NA |  | NA |  | NA |  | NA |  | NA |  | NA |  | NA |  | NA |  |
| NA |  | NA |  | NA |  | NA |  | NA |  | NA |  | NA |  | NA |  |

| 0.989164 | | 0.990209 | | 0.997476 | | 0.978345 | | 0.986245 | | 1.00552 | | 0.970597 | | NA |  |
| --- | --- | --- | --- | --- | --- | --- | --- | --- | --- | --- | --- | --- | --- | --- | --- |
| 0.98671 | | 0.968169 | | 0.992455 | | 0.947476 | | 0.988133 | | 0.975802 | | 0.929585 | | 0.957338 | |
| 1.09734 | | 1.12405 | | 1.08781 | | 1.13544 | | 1.10689 | | 1.04859 | | 1.00055 | | 1.09421 | |
| 0.979055 | | 0.972633 | | 0.962235 | | 0.967879 | | 1.00683 | | 1.00738 | | 1.02444 | | 1.08107 | |
| NA |  | NA |  | NA |  | NA |  | NA |  | NA |  | 1.07003 | | 1.03957 | |
| NA |  | NA |  | NA |  | NA |  | NA |  | NA |  | NA |  | 1.06218 | |
| NA |  | NA |  | NA |  | NA |  | NA |  | NA |  | NA |  | 1.04277 | |
| 1.03079 | |  | 1.0281 | 1.02487 | | 1.09899 | | 1.12595 | | 1.07896 | | NA |  | 1.02697 | |
| 1.04358 | | 1.04324 | |  | 1.1114 | 1.13372 | | 1.12675 | |  | 1.0638 | 1.03589 | | 1.00323 | |
|  | 1.0559 | 1.07328 | |  | 1.0615 | 1.06419 | | 1.04205 | | 1.02127 | | 0.987285 | |  | 1.1104 |
| 1.01533 | | 1.02158 | | 1.01721 | | 1.02766 | | 1.02708 | | 0.984118 | | 0.953912 | | 0.952632 | |
| 1.01533 | | 1.02158 | | 1.01721 | | 1.02766 | | 1.02708 | | 0.984118 | | 0.953912 | | 0.952632 | |
| 1.00609 | |  | 1.0244 | 1.03425 | | 1.04538 | | 1.03516 | |  | 1.0084 |  | 1.012 | 0.98462 | |
| 0.951952 | | 0.985351 | | 1.00117 | | 0.953001 | | 0.960418 | | 0.981987 | | 0.955379 | | 1.01274 | |
| 0.951952 | | 0.985351 | | 1.00117 | | 0.953001 | | 0.960418 | | 0.981987 | | 0.955379 | | 1.01274 | |
| 0.956653 | | 0.974119 | | 0.98217 | | NA |  | 0.95188 | | 1.00519 | | NA |  | NA |  |
| 0.940054 | | 0.970825 | | NA |  | NA |  | NA |  | NA |  | NA |  | NA |  |
| 0.932202 | | 0.958934 | | 0.947727 | | NA |  | NA |  | NA |  | NA |  | NA |  |
| 1.07913 | |  | 1.0755 | NA |  | NA |  | 1.09323 | | 1.07074 | | 1.08474 | | 0.998967 | |
| 1.10986 | | 1.10459 | | 1.08324 | | 1.07081 | | 1.08731 | | 1.07257 | | 1.04563 | | 0.974084 | |
| 1.10359 | | 1.13571 | | 1.09538 | | 1.10625 | | 1.00322 | | 1.02248 | | 1.05045 | | NA |  |
| 1.05571 | | 1.02813 | | 1.03958 | | 1.03745 | | 1.02459 | | 1.01882 | | 1.02783 | | NA |  |
| 1.05639 | | 1.04612 | | 1.04125 | | 1.01319 | | 1.02845 | | 1.01766 | | 1.10181 | | 0.985036 | |
| 1.07656 | | 1.11118 | | 1.08113 | | 1.07583 | |  | 1.0032 |  | 1.0324 |  | 1.0026 | 0.974948 | |
| 1.09326 | | 1.10963 | | 1.04248 | | 1.05417 | | 1.00465 | | 1.03827 | | 1.02855 | | 0.971228 | |
| 1.03931 | | 1.06916 | | 0.982267 | | 0.990313 | | 1.03441 | | 1.04601 | | 1.04772 | | 0.992488 | |
| 1.00482 | | 0.99517 | | 0.98775 | | 0.94385 | | 1.05317 | | 0.971667 | | 1.02078 | | NA |  |
| 1.09465 | |  | 1.057 | 1.04879 | | 0.976959 | | 1.05897 | | 0.984451 | | 1.07588 | | 0.967164 | |
| 0.905651 | | 0.929353 | | 0.957436 | | 0.896724 | | 0.900487 | | 0.979209 | | 1.01049 | | 0.832936 | |
| 0.988774 | | 1.03196 | | 1.00866 | | 1.09803 | | 1.00422 | | 0.997317 | | 1.00831 | | 0.959714 | |
| 0.920819 | | 0.931024 | | 0.924485 | | 0.962551 | | 0.920516 | | 0.923544 | | 0.924433 | | 0.954467 | |
| NA |  | NA |  | 0.979844 | | NA |  | NA |  | NA |  | 1.00362 | | NA |  |
| 0.98788 | | NA |  | NA |  | NA |  | NA |  | NA |  | NA |  | NA |  |
| NA |  | NA |  | NA |  | NA |  | NA |  | NA |  | NA |  | 1.00402 | |
| NA |  | NA |  | NA |  | NA |  | NA |  | NA |  | NA |  | 1.00196 | |
| NA |  | NA |  | 1.07883 | |  | 1.0613 | 1.02219 | | 1.01061 | | 1.12352 | | 0.99294 | |
| 1.05189 | | 1.05448 | | 1.07389 | | 0.972253 | | 1.04077 | | 1.01678 | | 1.09301 | | 0.984234 | |
| 0.973747 | | 0.988984 | | 1.07766 | | 1.07491 | | 0.974582 | | 0.997075 | | 1.05179 | | 0.935693 | |
| NA |  | NA |  | NA |  | NA |  | NA |  | NA |  | 1.11284 | | NA |  |

1.0672	1.03922	1.08649	1.02841	1.096	1.04232	1.13593	1.04386

0.958714 0.948482 0.924089 0.885558 0.907534 0.956488 0.962739	0.91751

1.10968	1.12373	1.09897	1.10791	1.10649	1.11043	1.05659	1.07944

1.08346	1.07307	1.09508	1.0543	1.07894	1.07482	1.06602	1.03081

1.0356	1.03341	1.04347	1.02716	1.05375	1.01067	1.04504	1.03674

1.02179	1.06407	1.05673	1.09236	1.03407 0.998439 0.895928 0.955247

NA	NA	0.967986 0.919979	1.01584 0.999722	1.0029	1.10733

1.0434	1.01433	1.05937	1.04887	1.03499	1.05248	1.11166	1.04705

1.10891	1.09335	1.11255	1.10696	1.14018	1.08258	1.07088	1.0283

NA	NA	NA	NA	NA	NA	NA	NA

NA	NA	NA	NA	NA	NA	NA	1.04604

NA	NA	NA	NA	NA	NA	NA	NA

1.19917	1.21773	1.21215	1.18442	1.20674	1.22747	1.16487	1.10965

1.1343	1.11663	1.11607	1.07871	1.18048	1.14476	1.16884	1.07328

NA	NA	0.870397 0.907726 0.940545 NA	1.01156 NA

1.02908	1.03165	1.08493 0.966963	1.06204	1.12132	1.1686	1.15971

NA	NA	0.84693 0.846088 0.941841 NA	0.96545 NA NA	NA	0.989294 0.991481	1.03474	1.05832	1.07806 NA

1.01817	1.01743	1.01768	1.01224	1.01134	1.01813	1.0206	1.01019

NA	NA	NA	NA	NA	NA	NA	NA

1.12523	1.11426	1.15899	1.12794	1.0336	1.14443	1.13427	1.04061

1.01703	1.0201 0.984664 0.937458 0.995544	1.01989 0.968393 0.974684

NA	NA	NA	1.0095 NA	NA	1.10024 0.964644

NA	NA	NA	1.04636 NA	NA	1.06069 NA

1.02646	1.00498	1.01974	1.00426 0.969106	1.05635	1.03836	1.09336

0.98699 0.955813	1.02135	1.00035	1.00901	1.01146	1.02718	0.93066

1.01142 0.971477	1.01669	1.02727 0.985432	1.05557	1.03459	1.02877

1.02282	1.02754	1.0269	1.05446	1.12622	1.005 0.980921	1.05604

1.02553 0.998758	1.01358	1.04875	0.94765	0.97687	0.93975	1.01452

1.11858	1.09622	1.12644	1.11673	1.09401	1.12431	1.15131 0.923768

1.04002	1.02662	1.00576	1.05288	1.0482	1.05558	1.09012	1.0248

1.11894	1.10387	1.10729	1.06477	1.18252	1.11362	1.1509	1.08416

1.03295	1.05153	1.05728	1.10713	1.0279	1.04818 0.987387	1.10609

1.00145 0.986302 0.971908	1.01115 0.998099	1.00048 0.995457 0.960759

1.11932	1.13953	1.14423	1.16408	1.10991	1.10452	1.19418	1.08711

1.03121	1.03539	1.00571 0.998246	1.03951	1.01953 0.984128	1.02274

1.02304	1.02901	1.00646	1.00649	1.04038	1.00636 0.983158 0.993004

1.00897	1.00406	1.01907 0.962539	1.04389	1.05231	1.00735	1.03099

1.10981	1.06095	1.13201	1.08774	1.01366	1.13049	1.09921 0.969777

| 1.09056 | | 1.03832 | 1.08045 | | 1.01847 | | 1.00457 | | 1.01293 | | 0.996178 | | 1.00181 | |
| --- | --- | --- | --- | --- | --- | --- | --- | --- | --- | --- | --- | --- | --- | --- |
| 1.03381 | | 1.00513 | 1.08288 | | 1.00514 | | 1.04701 | | 1.05131 | | 1.05376 | | 0.906253 | |
| 1.15605 | | 1.15403 | 1.15959 | | 1.12006 | | 1.12635 | | 1.15249 | | 1.14739 | | 1.16784 | |
| 1.04022 | | 1.04931 | 1.01554 | | 1.02039 | | 1.02458 | | 0.989502 | | 0.968202 | | 0.94282 | |
| 1.24623 | | 1.25791 | 1.31085 | | 1.20212 | | 1.27186 | | 1.29606 | | 1.17438 | | 1.14629 | |
| 0.998625 | | NA | NA |  | NA |  | NA |  | 1.00552 | | NA |  | NA |  |
| 0.983592 | | 0.988542 | 0.986168 | |  | 1.0468 | 0.988762 | | 1.00668 | | 0.998122 | | 1.02973 | |
| 1.00192 | | 1.00299 | 0.997625 | | 1.00948 | | 0.980849 | | 0.975034 | | 1.00925 | | NA |  |
| 1.12637 | | 1.11502 | 1.08678 | | 1.12261 | | 1.08297 | |  | 1.0289 |  | 1.0145 | 1.01698 | |
| 1.04446 | | 1.05131 | 1.05947 | | 1.06308 | | 0.983115 | | 1.04033 | | 0.969286 | |  | 1.1047 |
| 0.985679 | | 0.993416 | 1.00895 | |  | 1.0227 | 0.971428 | | 0.999618 | | 1.00458 | | 1.08233 | |
| NA |  | NA | NA |  | NA |  | NA |  | NA |  | NA |  | NA |  |
| 0.975949 | | 0.97949 | 1.00973 | | 0.960558 | | 1.03598 | | 1.01746 | | 1.00653 | | 1.08655 | |
| 1.08903 | | 1.07596 | 1.10855 | | 1.09902 | | 1.09232 | |  | 1.0408 | 1.11256 | | 1.13761 | |
| 1.08304 | | 1.07088 | 1.07059 | | 1.04686 | | 1.03764 | | 1.05274 | | 1.13651 | | 0.871482 | |
| 1.04411 | | 1.03868 | 1.06517 | | 1.08192 | | 1.04467 | | 1.04699 | | 1.02865 | | 1.01007 | |
| NA |  | NA | 1.10624 | | 1.13392 | | 1.01012 | | 1.05504 | | 1.08072 | | 1.02127 | |
|  | 1.0317 | 1.00069 | 1.07552 | | 1.14591 | | 1.03101 | | 1.02304 | | 0.995626 | | 0.970344 | |
| 1.00896 | | NA | NA |  | NA |  | NA |  | 0.998567 | | 0.986837 | | NA |  |
| 1.04892 | | 1.04545 | 1.05967 | | 1.05747 | |  | 1.013 | 1.00725 | | 0.966007 | | 1.04702 | |
| 1.04223 | | 1.03547 | 1.05145 | | 1.00227 | | 1.05574 | | 1.07565 | | 1.07884 | | 1.00469 | |
| 1.04847 | | 0.991719 | 0.997389 | | 1.02586 | | 0.959021 | | 1.03508 | | 0.930856 | | NA |  |
| 1.02885 | | 1.01073 | 1.02915 | | 0.96024 | |  | 1.0389 | 1.03668 | | 0.926919 | | 0.894407 | |
| 1.00292 | | 1.00811 | 0.993312 | | 0.936558 | | 0.987681 | | 1.04149 | | 0.915583 | | 0.908197 | |
| 1.00962 | | 0.99721 | 1.02293 | | 1.01998 | | 1.02225 | | 1.02016 | | 1.01336 | | 1.03167 | |
| 1.13447 | | 1.10703 | 1.08031 | | 1.04523 | | 1.11883 | | 1.14416 | | 1.06603 | | 1.05353 | |
| 1.01819 | | 1.03208 | 1.03065 | | 0.981049 | |  | 1.0313 | 1.02101 | |  | 1.0301 | 1.00525 | |
|  | 1.0166 | 1.04313 |  | 1.0398 | 0.946594 | | 1.09836 | | 0.99575 | | 0.905601 | | 0.956879 | |
| 0.99051 | | 1.02058 | 1.01681 | | 1.00668 | | 1.00606 | |  | 1.0244 | 0.996876 | | 1.00123 | |
| 1.00404 | | 0.949861 | 1.03581 | | 0.974176 | | 0.953138 | | 0.945492 | | 0.915981 | | 0.951111 | |
| 1.02915 | | 1.02856 | 1.03313 | | 1.00117 | | 1.03553 | | 1.01573 | |  | 1.0219 | 1.03145 | |
| 1.21142 | | 1.20901 | 1.19732 | |  | 1.1177 | 1.20515 | | 1.20813 | | 1.13158 | | 1.12076 | |
| 0.976599 | | 0.998233 | 0.952059 | | 0.98689 | | 0.949472 | | 1.00435 | | 0.997492 | | 0.955527 | |
| 1.01226 | | 1.03951 | 1.02663 | | 1.00203 | | 1.00605 | | 1.04702 | | 1.09364 | | 0.955937 | |
| NA |  | NA | NA |  | NA |  | NA |  | 1.08086 | | 1.15077 | | NA |  |
| 1.04044 | | 1.05034 | 1.02329 | | 1.05088 | | 1.07286 | | 1.02391 | |  | 1.0047 | 1.10337 | |
| 1.07658 | | 1.06429 | 1.06285 | |  | 1.0278 | 1.04334 | | 0.997491 | | 1.02073 | | 1.00143 | |
| 1.05274 | | 1.05687 | 1.04661 | | 1.06477 | | 1.05123 | | 1.03306 | | 1.06562 | | 0.992367 | |

mesenchy monocyte. monocyte. monocyte. monocyte_multipotenmultipotenmuscle..ce

| 1.11224 | 1.09661 | 1.05921 | 1.06212 | 1.06339 | 1.09321 | 1.09311 | 1.14375 |
| --- | --- | --- | --- | --- | --- | --- | --- |

NA	NA	NA	NA	NA	NA	NA	NA

1.19557	1.11498	1.18733	1.16942	1.16736	1.1729	1.216	1.10965

1.31018	1.14114	1.11449	1.15937	1.14683	1.06738	1.11902	1.04559

1.25736	1.14056	1.09161	1.10654	1.04772	1.12847	1.14531	1.10118

0.966905	0.94204 0.953434 0.938328 0.973004 0.971483 0.935788 0.992486

1.26401	1.11342	1.09639	1.13675	1.12741	1.09559	1.10504	1.15414

1.21294	1.07714	1.10633	1.09878	1.09624	1.14487	1.07169	1.08946

1.21294	1.07714	1.10633	1.09878	1.09624	1.14487	1.07169	1.08946

1.21233	1.17702	1.20131	1.22671	1.21418 NA	1.2384	1.10354

0.940576 0.938411	0.9759 0.979689	0.96959 0.957836 0.984336 0.996592

0.966066	1.04831	1.02504	1.03783	1.01087	1.02741	1.0491	1.02967

1.01636	1.03169	1.03812	1.0191	1.00836	1.10583	1.03821	1.03292

1.019	1.05385	1.06107	1.06469	1.03627	1.03387	1.00638	1.02744

1.12347 NA	NA	NA	NA	NA	NA	NA NA	NA	NA	NA	NA	NA	NA	NA

0.981164	1.00282 0.981503	1.00069 0.957026 NA	NA	1.05724

0.981164	1.00282 0.981503	1.00069 0.957026 NA	NA	1.05724

0.974784	1.00401 0.995579	1.01604	1.0085 0.935537 0.981551 0.949601

1.24203	1.1826	1.15254	1.16744	1.16381	1.18448	1.21706	1.11996

NA	NA	NA	NA	NA	NA	NA	NA

0.975256	1.0264	1.02796	1.0318	1.04511	1.037 0.999675 0.974701

NA	NA	NA	NA	NA	NA	NA	NA NA	NA	NA	NA	NA	NA	NA	NA

0.988805	1.03623	1.0355	1.04767	1.05205 NA	NA	1.04742

0.99307	1.12072	1.09339	1.07949	1.06433 NA	NA	NA

0.994504	1.17184	1.09694	1.10279	1.04331 NA	NA	NA NA	1.15657	1.09357	1.1065	1.09627 NA	NA	NA

1.20156	1.08054	1.10474	1.1024	1.11153	1.13101	1.20922	1.17074

1.20156	1.08054	1.10474	1.1024	1.11153	1.13101	1.20922	1.17074

1.27862	1.12606	1.15027	1.14897	1.11688	1.11091	1.11328	1.19314

1.36716	1.17289	1.17782	1.1898	1.18988	1.1412	1.1028	1.19208

1.16868	1.1228	1.16129	1.18075	1.21239 NA	1.15117	1.09378

NA	1.01053	1.03592	1.03008	1.04071 NA	NA	NA NA	NA	NA	NA	NA	NA	NA	NA NA	NA	NA	NA	NA	NA	NA	NA NA	NA	NA	NA	NA	NA	NA	NA

1.07822	1.05684	1.04005	1.05854	1.04673	1.06189	1.06207	1.00163

| 1.01146 | | 0.884884 | | 0.895741 | | 0.911634 | | 0.976076 | | 0.966348 | | 0.954471 | | 1.02248 | |
| --- | --- | --- | --- | --- | --- | --- | --- | --- | --- | --- | --- | --- | --- | --- | --- |
| 1.01146 | | 0.884884 | | 0.895741 | | 0.911634 | | 0.976076 | | 0.966348 | | 0.954471 | | 1.02248 | |
| 1.01146 | | 0.884884 | | 0.895741 | | 0.911634 | | 0.976076 | | 0.966348 | | 0.954471 | | 1.02248 | |
| 1.13002 | | 1.01991 | | 1.02164 | |  | 1.0205 | 0.986644 | | 0.978372 | | 1.02672 | | 1.06194 | |
| 1.10838 | | 1.01085 | | 1.03376 | | 1.03129 | | 1.01378 | | 0.926093 | | 0.979123 | | 1.01181 | |
| 0.947699 | | 0.991884 | | 1.00934 | |  | 1.0471 | 0.994213 | | 1.04137 | | 1.03871 | | 0.989008 | |
|  | 1.0298 |  | 1.0619 | 1.09854 | |  | 1.1007 | 1.05979 | | 1.08044 | |  | 1.0894 | 1.02449 | |
| 1.04199 | | 1.03066 | | 1.05622 | | 1.07294 | | 1.03967 | | 1.04468 | |  | 1.0411 | 1.03001 | |
| 0.991177 | | 1.00648 | | 1.03016 | | 1.06512 | | 1.03435 | | 1.04315 | | 1.03353 | | 0.995929 | |
| 1.05754 | | 1.06723 | | 1.08911 | | 1.11532 | | 1.07636 | | 1.05808 | | 1.08045 | | 1.03037 | |
| 1.07525 | | 1.08427 | | 1.11766 | | 1.14102 | | 1.08212 | | 1.07127 | | 1.09752 | | 0.999408 | |
| 1.00479 | | 1.07701 | | 1.09111 | | 1.13388 | | 1.05095 | | 1.09078 | |  | 1.058 | 1.01771 | |
| 1.08436 | | 1.07433 | | 1.14485 | | 1.15897 | |  | 1.0986 | 1.07309 | | 1.13725 | |  | 1.0014 |
| 1.03016 | | 1.04114 | | 1.07279 | |  | 1.0725 | 1.03615 | |  | 1.0216 | 1.04219 | | 0.989398 | |
| 1.02986 | | 1.04481 | | 1.07029 | | 1.09115 | | 1.04724 | | 1.00669 | | 1.04417 | | 1.01424 | |
| NA |  | NA |  | NA |  | NA |  | NA |  | NA |  | NA |  | NA |  |
| 1.19505 | | 1.06637 | | 1.08304 | | 1.06288 | | 1.11957 | | 1.10495 | |  | 1.1422 | 1.16739 | |
| 1.07985 | | 1.07154 | | 1.07664 | | 1.08574 | | 1.08366 | | 1.05857 | | 1.08963 | | 1.03659 | |
| 1.03379 | | 1.06549 | | 1.12698 | | 1.13659 | | 1.09166 | | 1.09495 | | 1.13831 | | 1.03141 | |
| 1.05818 | |  | 1.0745 | 1.14499 | | 1.16485 | | 1.04834 | | 1.18584 | | 1.23647 | | 1.06695 | |
| 0.977587 | | 1.02443 | | 1.04991 | | 1.06007 | | 1.03265 | | 1.00209 | | 1.02818 | | 0.991165 | |
| 1.00568 | | NA |  | NA |  | NA |  | NA |  | NA |  | NA |  | 1.07124 | |
| NA |  | NA |  | NA |  | NA |  | NA |  | NA |  | NA |  | NA |  |
| 1.00349 | | 1.03766 | | 1.06795 | | 1.07424 | | 1.02417 | | 1.03721 | | 1.06435 | | 1.00345 | |
| 1.03922 | | 1.10633 | | 1.08403 | | 1.03306 | |  | 1 | 1.06711 | | 1.02395 | | 0.973384 | |
| 1.02769 | | 1.06482 | | 1.09432 | | 1.09566 | | 1.07822 | | 1.08313 | | 1.09151 | | 0.993956 | |
| 1.03466 | | 1.07625 | |  | 1.1059 | 1.11391 | | 1.07207 | | 1.04378 | | 1.08439 | | 1.02483 | |
| 1.04135 | |  | 1.0509 | 1.06154 | | 1.07202 | | 1.05198 | | 1.02821 | | 1.08911 | | 0.96529 | |
| 1.05765 | | 1.05539 | | 1.08576 | | 1.07726 | | 1.05928 | | 1.04793 | | 1.11411 | | 1.01364 | |
| 1.05765 | | 1.05539 | | 1.08576 | | 1.07726 | | 1.05928 | | 1.04793 | | 1.11411 | | 1.01364 | |
| 1.00312 | | 1.05209 | | 1.09353 | | 1.11659 | | 1.07575 | | 1.06592 | | 1.10755 | | 1.03105 | |
| 1.03338 | | 1.07461 | | 1.11332 | | 1.11686 | | 1.08122 | | 1.03321 | |  | 1.1215 | 1.08279 | |
| 1.01989 | |  | 1.0861 | 1.06961 | | 1.09911 | | 1.04818 | | 1.04306 | | 1.00773 | | 1.01785 | |
| 0.987792 | | 1.03172 | | 1.04236 | | 1.05566 | | 1.00255 | | 0.995879 | | 1.01704 | | 1.07324 | |
| NA |  | NA |  | NA |  | NA |  | NA |  | NA |  | NA |  | NA |  |
|  | 1.0195 | 1.01729 | | 1.03503 | | 1.00507 | | 1.03474 | | 1.00438 | | 1.02032 | | 1.04554 | |
| 1.01491 | | 1.05922 | | 1.09379 | | 1.10193 | |  | 1.087 | 1.07118 | | 1.08841 | | 0.999415 | |
| 0.997976 | | 1.04124 | | 1.07463 | | 1.08334 | | 1.04649 | | 1.05636 | | 1.07936 | | 1.02364 | |
| 1.09993 | | 1.04825 | | 1.16504 | | 1.19886 | | 1.13092 | | 1.28952 | | 1.29516 | | 1.09493 | |

| 1.01785 | | 1.02757 | 1.02094 | 0.987414 | |  | 1.0155 | 1.03017 | | 1.03895 | 1.04574 | |
| --- | --- | --- | --- | --- | --- | --- | --- | --- | --- | --- | --- | --- |
| 1.01286 | | 1.00283 | 1.02443 |  | 1.0348 | 0.98531 | | 0.933477 | | 0.972193 |  | 1.0511 |
| 0.987959 | | 1.02672 | 1.01148 | 0.996788 | | 1.02132 | | 1.05188 | | 1.01171 | 1.03129 | |
| 1.01418 | | 1.04786 | 1.03267 | 1.02698 | | 1.02025 | | NA |  | NA | 1.06755 | |
| NA |  | NA | NA | NA |  | NA |  | NA |  | NA | NA |  |
| NA |  | NA | NA | NA |  | NA |  | NA |  | NA | NA |  |
| NA |  | NA | NA | NA |  | NA |  | NA |  | NA | NA |  |
| NA |  | NA | NA | NA |  | NA |  | NA |  | NA | NA |  |
| NA |  | NA | NA | NA |  | NA |  | NA |  | NA | 1.03649 | |
| NA |  | NA | NA | NA |  | NA |  | NA |  | NA | 1.03587 | |
| NA |  | NA | NA | NA |  | NA |  | NA |  | NA | NA |  |
| NA |  | NA | NA | NA |  | NA |  | NA |  | NA | NA |  |
| NA |  | NA | NA | NA |  | NA |  | NA |  | NA | NA |  |
| NA |  | NA | NA | NA |  | NA |  | NA |  | NA | NA |  |
| NA |  | NA | NA | NA |  | NA |  | NA |  | NA | 1.05373 | |
| NA |  | NA | 1.04114 | 1.02735 | | NA |  |  | 1.0386 | 1.01838 |  | 1.0533 |
| NA |  | NA | NA | NA |  | NA |  | NA |  | NA | NA |  |
| NA |  | NA | NA | NA |  | NA |  | NA |  | NA | NA |  |
| NA |  | 1.05459 | 1.05938 | 1.04773 | | 1.07675 | | 1.07954 | | 1.05596 | 1.04226 | |
| NA |  | NA | 1.03244 | 1.02605 | | NA |  | NA |  | 1.01921 | 1.02329 | |
| NA |  | NA | NA | NA |  | NA |  | NA |  | NA | NA |  |
| 1.03534 | | 1.07586 | 1.10774 | 1.13008 | |  | 1.0759 | 1.06131 | | 1.09727 | 1.05479 | |
| 0.954602 | | 0.986543 | 0.974873 | 0.986475 | | 0.996741 | | 0.991935 | | 0.948485 | NA |  |
| 0.954602 | | 0.986543 | 0.974873 | 0.986475 | | 0.996741 | | 0.991935 | | 0.948485 | NA |  |
| NA |  | NA | 0.967888 | 0.966067 | | NA |  | NA |  | 1.03229 | NA |  |
| NA |  | NA | 0.967888 | 0.966067 | | NA |  | NA |  | 1.03229 | NA |  |
| NA |  | NA | 0.987548 | 1.00133 | | NA |  | 1.04071 | | 0.972908 | NA |  |
| NA |  | 0.98877 | 0.974594 | 1.00575 | | 1.00571 | |  | 1.0119 | 1.01999 | NA |  |
| NA |  | NA | NA | NA |  | NA |  | NA |  | NA | NA |  |
| NA |  | NA | NA | NA |  | NA |  | NA |  | 1.02514 | NA |  |
| NA |  | NA | NA | NA |  | NA |  | NA |  | 1.02514 | NA |  |
| NA |  | NA | NA | NA |  | NA |  | NA |  | 1.02514 | NA |  |
| NA |  | NA | NA | NA |  | NA |  | NA |  | NA | NA |  |
| NA |  | NA | NA | NA |  | NA |  | NA |  | NA | NA |  |
| NA |  | NA | NA | NA |  | NA |  | NA |  | NA | NA |  |
| NA |  | NA | NA | NA |  | NA |  | NA |  | NA | NA |  |
|  | 1.0425 | 1.02953 | 0.998587 |  | 0.9848 | 1.00594 | | 0.999182 | | 1.02207 | 1.00111 | |
| 1.15305 | | 1.10775 | 1.08421 | 1.08175 | | 1.04709 | | 1.05874 | | 1.07543 | 1.04997 | |
| 1.15305 | | 1.10775 | 1.08421 | 1.08175 | | 1.04709 | | 1.05874 | | 1.07543 | 1.04997 | |

| 1.28572 | 1.18626 |  | 1.0849 | 1.13284 | 1.13129 | | 1.08785 | | 1.14271 | 1.19528 | |
| --- | --- | --- | --- | --- | --- | --- | --- | --- | --- | --- | --- |
| 1.23199 | NA | NA |  | NA | NA |  | NA |  | NA | 1.18609 | |
| 1.09283 | 1.06427 | 1.03063 | | 1.07427 | 1.06492 | |  | 1.0959 | 1.00098 | 1.11048 | |
| 1.02383 | 0.949039 | NA |  | NA | 0.971105 | | NA |  | 0.903209 | 1.03612 | |
| 1.09475 | 1.07506 | 1.06522 | | 1.07611 | 1.10111 | | 1.07096 | | 1.00063 |  | 1.0648 |
| NA | 1.09366 | 1.09986 | | 1.09567 |  | 1.1007 | NA |  | NA | NA |  |
| NA | NA | NA |  | NA | NA |  | NA |  | NA | NA |  |
| NA | NA | NA |  | NA | NA |  | NA |  | NA | NA |  |
| 0.981999 | 0.937833 | NA |  | NA | NA |  | NA |  | NA | 0.986327 | |
| 0.977638 | 1.03643 | NA |  | NA | NA |  | NA |  | NA | 0.990783 | |
| 0.979054 | 0.938637 | 0.89928 | | 0.886774 | 0.91542 | |  | 1.0089 | 0.946681 | 1.01387 | |
| NA | NA | NA |  | NA | NA |  | NA |  | NA | NA |  |
| NA | NA | NA |  | NA | NA |  | NA |  | NA | NA |  |
| NA | NA | NA |  | NA | NA |  | NA |  | NA | NA |  |
| NA | NA | NA |  | NA | NA |  | NA |  | NA | NA |  |
| NA | NA | NA |  | NA | NA |  | NA |  | NA | NA |  |
| NA | NA | NA |  | NA | NA |  | NA |  | NA | NA |  |
| NA | NA | NA |  | NA | NA |  | NA |  | NA | NA |  |
| NA | NA | NA |  | NA | NA |  | NA |  | NA | NA |  |
| NA | NA | 1.02662 | | 1.05495 | NA |  | 1.01669 | | 1.07129 | 0.980652 | |
| NA | NA | NA |  | NA | NA |  | NA |  | NA | NA |  |
| NA | NA | NA |  | NA | NA |  | NA |  | NA | 0.935427 | |
| NA | NA | NA |  | NA | NA |  | NA |  | NA | NA |  |
| NA | NA | NA |  | NA | NA |  | NA |  | NA | NA |  |
| NA | NA | NA |  | NA | NA |  | NA |  | NA | NA |  |
| NA | NA | 0.968337 | | 0.989058 | NA |  | 0.940298 | | 0.977424 | 0.966783 | |
| NA | NA | NA |  | NA | NA |  | NA |  | NA | NA |  |
| NA | NA | NA |  | NA | NA |  | NA |  | NA | NA |  |
| NA | NA | NA |  | NA | NA |  | NA |  | NA | NA |  |
| NA | NA | NA |  | NA | NA |  | NA |  | NA | NA |  |
| 0.966944 | 1.04413 | 1.03463 | | 1.06902 | 1.01124 | | 0.934475 | | 0.972048 | 0.981003 | |
| NA | NA | NA |  | NA | NA |  | NA |  | NA | NA |  |
| NA | NA | NA |  | NA | NA |  | NA |  | NA | NA |  |
| NA | NA | NA |  | NA | NA |  | NA |  | NA | NA |  |
| NA | NA | NA |  | NA | NA |  | NA |  | NA | NA |  |
| NA | NA | 1.07239 | | 1.08252 | NA |  | NA |  | NA | 1.02486 | |
| NA | NA | NA |  | NA | NA |  | NA |  | NA | NA |  |
| NA | NA | 1.03672 | | 1.06138 | NA |  | NA |  | NA | 1.03869 | |
| NA | NA | NA |  | NA | NA |  | NA |  | NA | NA |  |

| NA |  | NA |  | NA |  | NA |  | NA |  | NA |  | NA |  | NA |  |
| --- | --- | --- | --- | --- | --- | --- | --- | --- | --- | --- | --- | --- | --- | --- | --- |
| NA |  | NA |  | 0.990128 | | 1.01101 | | NA |  | 0.999726 | | 1.04177 | | 0.967138 | |
| NA |  | NA |  | NA |  | NA |  | NA |  | NA |  | NA |  | NA |  |
| NA |  | NA |  | NA |  | NA |  | NA |  | NA |  | NA |  | NA |  |
| NA |  | NA |  | NA |  | NA |  | NA |  | NA |  | NA |  | NA |  |
| NA |  | NA |  | 1.09298 | | 1.11379 | | NA |  | NA |  | 1.12618 | | 1.02593 | |
| NA |  | NA |  | NA |  | NA |  | NA |  | NA |  | NA |  | NA |  |
| NA |  | NA |  | NA |  | NA |  | NA |  | NA |  | NA |  | NA |  |
| NA |  | NA |  | NA |  | NA |  | NA |  | NA |  | NA |  | NA |  |
| NA |  | NA |  | 1.05988 | | 1.09713 | | NA |  | NA |  | NA |  | 1.03061 | |
| 1.00682 | | 0.967084 | | 1.00792 | | 1.01417 | | 1.00854 | | 0.975673 | | 1.01685 | | 0.990688 | |
| 1.30442 | | 1.00133 | | 1.21195 | | 1.22854 | | 0.997943 | | NA |  |  | 1.0419 | 1.31739 | |
| NA |  | 1.00018 | | 1.00362 | | 1.00896 | | 1.00149 | | NA |  | 0.955153 | | NA |  |
| 0.984263 | | 1.01083 | | 1.01145 | | 0.990682 | | 0.992957 | | 1.00442 | | 0.982617 | | 0.994946 | |
| 1.15271 | | 1.05277 | | 1.07057 | | 1.08505 | | 1.08391 | | 1.03171 | | 1.13888 | | 1.05106 | |
| 1.13218 | | 1.01943 | |  | 1.0596 | 1.04624 | | 0.98539 | | 1.06162 | | 1.01965 | | 1.02865 | |
| 1.20968 | | 1.12357 | | 1.12037 | | 1.14925 | | 1.11486 | | NA |  | NA |  | NA |  |
| 1.05135 | | 0.990319 | | 1.00946 | | 1.01419 | | 0.937629 | | 0.997613 | | 0.973856 | | 0.988278 | |
| 1.06865 | | 1.04066 | | 1.02717 | | 1.04342 | | 1.02152 | | 1.06875 | | 0.996229 | | 0.96357 | |
| 1.08248 | | 1.12759 | | 1.09972 | | 1.09051 | | 1.08561 | | 1.21647 | |  | 1.1135 | NA |  |
| 0.997828 | | 1.02909 | | 1.02327 | | 1.03131 | | 1.04109 | | NA |  | 1.01402 | | 1.04911 | |
| 1.02451 | | 1.00829 | | 1.02289 | | 1.02707 | |  | 1.042 | 1.04453 | | 1.02836 | | 1.05423 | |
| 1.00633 | |  | 1.0737 | 1.05914 | |  | 1.052 | 1.00034 | | 0.983675 | | 1.01579 | | 1.03237 | |
| 1.01302 | | 1.01769 | | 1.03198 | | 1.01412 | | 1.00305 | | NA |  | NA |  | 1.03818 | |
| 1.03296 | | 1.01151 | | 1.02497 | | NA |  | 1.04136 | | 1.05714 | | 1.01366 | | NA |  |
| 1.03427 | | 1.04888 | | 1.04472 | | 1.02632 | | 1.07414 | | 1.08307 | | 1.07046 | |  | 1.0983 |
| 1.10614 | | 1.02458 | | 1.02195 | | 0.994266 | | 1.04298 | | 1.07315 | | 1.06453 | | 0.986762 | |
| 1.17652 | | 1.00669 | | 1.01026 | | 0.978261 | | 1.03915 | |  | 1.0707 | 1.10964 | | 1.03128 | |
| 1.17652 | | 1.00669 | | 1.01026 | | 0.978261 | | 1.03915 | |  | 1.0707 | 1.10964 | | 1.03128 | |
|  | 1.125 | 1.02817 | | NA |  | NA |  | 1.02758 | |  | 1.0652 | 1.09914 | | NA |  |
| 1.07895 | | 1.00628 | | 1.02096 | | 0.999342 | | 1.04387 | | 1.06711 | |  | 1.0753 | NA |  |
| 1.04047 | | 1.07091 | | NA |  | NA |  | 1.03798 | | 1.07625 | | 1.09075 | | NA |  |
| 1.04047 | | 1.07091 | | NA |  | NA |  | 1.03798 | | 1.07625 | | 1.09075 | | NA |  |
| 1.04047 | | 1.07091 | | NA |  | NA |  | 1.03798 | | 1.07625 | | 1.09075 | | NA |  |
| 1.01996 | | 1.00439 | |  | 1.003 | 0.982423 | | 1.01232 | | 0.985448 | | 1.07078 | | 0.950476 | |
| 1.03028 | | 0.99954 | | 0.998621 | | 0.997551 | | 1.02738 | | 1.04101 | | 1.02372 | | 1.01138 | |
| 1.09849 | | 1.06619 | | NA |  | NA |  | 1.05822 | | NA |  | NA |  | NA |  |
| NA |  | NA |  | NA |  | NA |  | NA |  | NA |  | NA |  | NA |  |
| 0.970637 | | 1.06762 | | 1.10615 | | 1.09894 | | NA |  | NA |  | 1.08525 | | NA |  |

| NA |  | NA |  | NA |  | NA | NA |  | NA |  | NA |  | NA |  |
| --- | --- | --- | --- | --- | --- | --- | --- | --- | --- | --- | --- | --- | --- | --- |
| NA |  | NA |  | NA |  | NA | NA |  | NA |  | NA |  | NA |  |
| NA |  | NA |  | NA |  | NA | NA |  | NA |  | NA |  | NA |  |
| NA |  | NA |  | NA |  | NA | NA |  | NA |  | NA |  | NA |  |
| 0.976637 | | 0.994239 | | 0.966463 | | 0.952615 | 0.966491 | | 0.928932 | | 0.930133 | | 1.01005 | |
| 1.00733 | | 1.01712 | | 1.00087 | | 0.99756 | 1.06146 | | 0.963911 | | 1.00593 | | 1.08098 | |
| 1.00052 | | 0.986922 | | 1.00988 | | 0.986271 | 0.992813 | | 0.988095 | | 1.03264 | | 0.987592 | |
| 1.04404 | | 1.05409 | | 1.03292 | | 1.05268 | 0.999665 | | 0.996619 | |  | 1.0394 | 1.03295 | |
| 1.02878 | | 0.920455 | | 0.928645 | | 0.92965 | 0.952681 | | 0.982662 | | 0.950735 | | 1.05595 | |
| 1.03252 | | 0.976042 | | 1.00473 | | 1.01206 | 0.986383 | | 0.976438 | | 0.969877 | | 1.06378 | |
|  | 1.1824 | 1.06727 | | 1.06896 | | 1.07122 | 1.05873 | | 1.09543 | | 1.09679 | | 1.08148 | |
| 1.25586 | | 1.14501 | | 1.13466 | | 1.13887 | 1.09896 | | 1.05001 | | 1.07821 | | 1.21548 | |
| 1.41421 | | 1.18732 | |  | 1.2012 | 1.21528 | 1.21875 | | 1.19437 | | 1.20099 | | 1.22152 | |
| 0.964622 | | 0.97341 | | 0.998566 | | 1.01304 | 1.00783 | | 0.951825 | | 1.00186 | |  | 1.008 |
| NA |  | NA |  | NA |  | NA | NA |  | NA |  | NA |  | NA |  |
| NA |  | NA |  | NA |  | NA | NA |  | NA |  | NA |  | NA |  |
| 1.22396 | | 1.09755 | | 1.09074 | | 1.06491 | 1.10509 | |  | 1.1052 | 1.16744 | |  | 1.0864 |
| NA |  | NA |  | NA |  | NA | NA |  | NA |  | NA |  | NA |  |
| 0.963659 | | 0.950578 | | 0.955166 | | 0.934407 | 0.944777 | | 0.894656 | | 0.959193 | | 1.03701 | |
| NA |  | NA |  | NA |  | NA | NA |  | NA |  | NA |  | NA |  |
| NA |  | NA |  | NA |  | NA | NA |  | NA |  | NA |  | NA |  |
| 1.11375 | | 1.05218 | |  | 1.0883 | 1.09898 | 1.06346 | | 1.17571 | | 1.17744 | |  | 1.1106 |
| 1.21499 | | 1.05919 | | 1.06037 | | 1.07419 |  | 1.0444 | 1.14789 | | 1.11808 | | 1.12746 | |
| 1.21499 | | 1.05919 | | 1.06037 | | 1.07419 |  | 1.0444 | 1.14789 | | 1.11808 | | 1.12746 | |
| 1.21499 | | 1.05919 | | 1.06037 | | 1.07419 |  | 1.0444 | 1.14789 | | 1.11808 | | 1.12746 | |
| 1.21499 | | 1.05919 | | 1.06037 | | 1.07419 |  | 1.0444 | 1.14789 | | 1.11808 | | 1.12746 | |
| 1.01299 | | 1.11179 | | 1.07584 | | 1.06493 | 0.984613 | | 1.07595 | | 1.05342 | | 1.00398 | |
| 1.01299 | | 1.11179 | | 1.07584 | | 1.06493 | 0.984613 | | 1.07595 | | 1.05342 | | 1.00398 | |
| 1.01299 | | 1.11179 | | 1.07584 | | 1.06493 | 0.984613 | | 1.07595 | | 1.05342 | | 1.00398 | |
| 1.01299 | | 1.11179 | | 1.07584 | | 1.06493 | 0.984613 | | 1.07595 | | 1.05342 | | 1.00398 | |
| 1.21238 | |  | 1.1404 | 1.11386 | | 1.13565 | 1.18819 | | 1.08834 | | 1.09945 | | 1.12161 | |
| 1.21238 | |  | 1.1404 | 1.11386 | | 1.13565 | 1.18819 | | 1.08834 | | 1.09945 | | 1.12161 | |
| 1.13944 | | 0.996847 | | 1.04041 | | 1.05024 | 1.01117 | | 0.913921 | | 1.00626 | | 1.08799 | |
| 1.13944 | | 0.996847 | | 1.04041 | | 1.05024 | 1.01117 | | 0.913921 | | 1.00626 | | 1.08799 | |
| 1.30257 | | 1.13484 | | 1.14234 | | 1.12849 | 1.14842 | |  | 1.2018 | 1.19268 | | 1.13441 | |
| 0.961859 | | 0.928308 | | 0.931896 | | 0.940847 | 0.932448 | | 0.89131 | | 0.945733 | | 1.02344 | |
| 0.961859 | | 0.928308 | | 0.931896 | | 0.940847 | 0.932448 | | 0.89131 | | 0.945733 | | 1.02344 | |
| 0.959878 | | 0.971288 | | 0.996537 | | 0.971035 | 0.967352 | | 1.00953 | | 0.978839 | | 1.04619 | |
| 0.959878 | | 0.971288 | | 0.996537 | | 0.971035 | 0.967352 | | 1.00953 | | 0.978839 | | 1.04619 | |

| 0.969718 | | 0.995014 | | 0.973505 | 0.968982 | | 0.973918 | | 0.984822 | | 0.966697 | | 0.996774 | |
| --- | --- | --- | --- | --- | --- | --- | --- | --- | --- | --- | --- | --- | --- | --- |
| 0.95502 | | 0.977123 | | 0.981531 | 0.990568 | | 0.991574 | | 0.929895 | | 0.969981 | | 1.01859 | |
| 0.978483 | | 1.00839 | | 1.00015 | 1.00055 | | 0.997096 | | 0.974363 | |  | 1.0038 |  | 1.0479 |
| 1.01314 | | 0.99188 | | 0.980523 | 0.999168 | | 0.957468 | |  | 1.0505 | 1.04209 | | 1.01856 | |
| 1.01816 | | 0.992205 | | 1.00807 | 0.987101 | | 0.977241 | | 0.99972 | | 0.993183 | | 1.00924 | |
| 1.00898 | |  | 1.0086 | 1.02031 | 1.00912 | | 1.00925 | | NA |  | NA |  | 1.06374 | |
|  | 1.02 | 1.02216 | | 1.03147 | 1.01921 | | 1.00954 | | 0.914396 | | 1.01719 | | 1.03276 | |
| 1.03381 | | 1.00726 | | 1.01861 | 1.03144 | | 0.990233 | | 1.01955 | | 1.05769 | | 1.03506 | |
| 1.02491 | |  | 1.0392 | 1.01289 | 1.02369 | | 0.996181 | | 1.00429 | | 1.05737 | | 1.02552 | |
| 1.01361 | | NA |  | NA | NA |  | NA |  | NA |  | NA |  | 1.00976 | |
| 1.01174 | | 0.992029 | | 0.988849 | 1.01223 | | 0.963418 | | 0.993368 | | 1.01355 | | 1.01452 | |
| 0.997623 | | 1.00645 | | 1.02143 | 1.02742 | | 0.974546 | | 1.14749 | | 1.06191 | | 1.01421 | |
| 1.04091 | | NA |  | NA | NA |  | NA |  | NA |  | NA |  | NA |  |
| 1.00791 | |  | 1.0214 | 1.02101 |  | 1.012 | 1.02371 | | 1.08113 | | 1.09425 | | 1.01239 | |
| 1.05678 | | NA |  | NA | NA |  | NA |  | NA |  | NA |  | NA |  |
| 0.985197 | | NA |  | NA | NA |  | NA |  | NA |  | NA |  | NA |  |
| NA |  | NA |  | NA | NA |  | NA |  | NA |  | NA |  | NA |  |
| 0.931823 | | 0.986657 | | 0.975752 | 0.970263 | | 1.02583 | | 0.976677 | | 0.985914 | | 0.995463 | |
| NA |  | NA |  | NA | NA |  | NA |  | NA |  | NA |  | NA |  |
| NA |  | NA |  | NA | NA |  | NA |  | NA |  | NA |  | NA |  |
| 0.971122 | | NA |  | NA | NA |  | NA |  | NA |  | NA |  | NA |  |
| 0.968571 | | NA |  | NA | NA |  | NA |  | NA |  | NA |  | NA |  |
| 0.964947 | | NA |  | NA | NA |  | NA |  | NA |  | NA |  | NA |  |
| 0.942675 | | 0.997725 | | 1.01476 | 1.01916 | |  | 1.0051 | 0.972723 | | 0.978474 | | NA |  |
| 0.937206 | | 1.00775 | | 1.00679 |  | 1.0014 | 1.01982 | | 1.01533 | | 1.02866 | | 1.00353 | |
| NA |  | NA |  | NA | NA |  | NA |  | NA |  | NA |  | NA |  |
| NA |  | NA |  | NA | NA |  | NA |  | NA |  | NA |  | NA |  |
| NA |  | NA |  | NA | NA |  | NA |  | NA |  | NA |  | NA |  |
| 1.24827 | | 1.09986 | | 1.10972 | 1.08626 | | 1.10059 | | 1.14108 | | 1.13661 | | 1.13347 | |
| 1.08139 | | 1.02954 | | 1.04488 | 1.06904 | | 1.07582 | | 1.00761 | | 0.997955 | | 1.03516 | |
| 1.08139 | | 1.02954 | | 1.04488 | 1.06904 | | 1.07582 | | 1.00761 | | 0.997955 | | 1.03516 | |
| 0.91188 | | NA |  | NA | NA |  | NA |  | NA |  | NA |  | 0.894118 | |
| 1.03655 | | NA |  | NA | NA |  | NA |  | NA |  | NA |  | 1.00512 | |
| 0.997292 | | NA |  | NA | NA |  | NA |  | NA |  | NA |  | 0.983094 | |
| 1.00758 | | 0.998704 | | NA | NA |  | 0.95322 | | 0.950269 | | 1.01765 | | 1.00753 | |
| 1.06233 | |  | 1.0718 | 1.08563 | 1.07767 | | 1.06658 | | 1.06655 | | 1.06616 | | 0.972309 | |
| 0.960737 | | 1.00821 | | 0.99209 | NA |  | 1.00856 | | NA |  | NA |  | 1.02447 | |
| 1.00524 | | NA |  | NA | NA |  | NA |  | NA |  | NA |  | 0.966916 | |
| 0.985823 | | 1.05867 | | NA | NA |  | 1.02577 | | NA |  | NA |  | 1.01929 | |

| 0.970063 | | 0.975424 | | 0.954954 | | 0.946109 | | 0.993038 | | 0.965124 |  | 1.0145 |  | 1.0307 |
| --- | --- | --- | --- | --- | --- | --- | --- | --- | --- | --- | --- | --- | --- | --- |
| 0.940875 | | NA |  | NA |  | NA |  | NA |  | NA | NA |  | 0.956833 | |
| 0.956433 | | NA |  | NA |  | NA |  | NA |  | NA | NA |  | 0.982882 | |
| 0.991088 | | NA |  | NA |  | NA |  | NA |  | NA | NA |  | 1.02651 | |
| 1.01424 | | NA |  | NA |  | NA |  | NA |  | NA | NA |  | 1.03701 | |
| 0.990611 | | 1.00307 | | 1.00107 | | 0.979487 | | 1.02007 | | 0.936067 | 1.00281 | | 1.03746 | |
| NA |  | NA |  | NA |  | NA |  | NA |  | NA | NA |  | NA |  |
| NA |  | NA |  | NA |  | NA |  | NA |  | NA | NA |  | NA |  |
| NA |  | NA |  | NA |  | NA |  | NA |  | NA | NA |  | NA |  |
| NA |  | NA |  | NA |  | NA |  | NA |  | NA | NA |  | NA |  |
| 1.02772 | | 1.05838 | | 1.01621 | | 1.02194 | | 0.976744 | | 0.98299 | 1.01524 | | 1.03106 | |
| 1.08825 | | 1.10823 | | 1.06987 | | 1.07508 | | 1.05715 | | NA | NA |  | 1.03091 | |
| 1.08428 | | 1.07677 | | 1.07673 | | 1.10998 | | 1.07465 | | 1.15332 | 1.09925 | | 1.02529 | |
| NA |  | NA |  | NA |  | NA |  | NA |  | NA | NA |  | NA |  |
| 1.04809 | | NA |  | NA |  | NA |  | 1.03183 | | NA | NA |  | 1.00192 | |
| 1.00675 | | 1.12605 | | 1.09419 | | 1.07911 | | 1.12016 | | 1.07793 | 1.03417 | | 1.01878 | |
| 1.05634 | | 0.99261 | | 0.985458 | | 1.00381 | | 1.03513 | | 0.97629 | 1.05112 | | 1.04762 | |
| 1.02295 | | NA |  | NA |  | NA |  | 0.981332 | | NA | NA |  | 1.03047 | |
| NA |  | NA |  | NA |  | NA |  | NA |  | NA | NA |  | NA |  |
| 1.01011 | | 0.960371 | | NA |  | NA |  | 0.96753 | | 0.970878 | 0.975151 | | 1.00922 | |
| 1.01188 | | 0.993585 | | 0.989059 | | 0.975354 | | 1.00476 | | 1.05457 | 0.992653 | | 0.994218 | |
|  | 0.9745 | 0.971162 | | 0.936102 | | 0.923806 | | 0.986281 | | 0.987874 | 1.03242 | | 1.01395 | |
| 0.923456 | | 0.934815 | | 0.904434 | | 0.909228 | | 0.946231 | | 0.95234 | 1.00423 | | 0.978021 | |
| 1.26641 | |  | 1.1716 | 1.16351 | | 1.17806 | | 1.19933 | | 1.13544 | 1.21697 | |  | 1.0377 |
| 1.03296 | | 1.05417 | | 1.03733 | | 1.05629 | | 1.09046 | | 1.01574 | 1.01673 | | 1.00265 | |
| 1.01444 | | 1.04519 | | NA |  | NA |  | 1.03669 | | 0.965539 | 1.07378 | | 1.06765 | |
| 1.03256 | | 1.02108 | | 1.04106 | | 1.04635 | | 1.05421 | | 1.09532 | 1.09091 | | 1.02257 | |
|  | 1.0024 | 1.00208 | | 1.02411 | | 1.00301 | | 1.01593 | | 1.06964 | 1.07931 | | 0.972005 | |
| 1.04899 | | 1.03361 | | 1.06974 | |  | 1.0862 | 1.07348 | | 0.985779 | 1.02542 | | 0.980034 | |
| 1.04208 | | 1.04663 | | 1.04425 | | 1.08555 | | 1.09693 | | 1.01869 | 1.03304 | | 0.997014 | |
| 1.04067 | |  | 0.9375 | 1.03579 | |  | 1.0883 |  | 1.045 | 0.982906 | 1.07194 | | 1.08221 | |
| 1.01705 | | 1.09066 | | 1.03252 | | 1.06844 | | 1.05141 | | 1.08366 | 1.07446 | | 1.00284 | |
| 1.00504 | | 0.854981 | | 0.952795 | | 0.969803 | | 0.985481 | | 0.944039 | 0.95499 | | 0.986614 | |
| 1.01032 | | 1.04879 | | 1.06286 | | 1.07057 | | 1.01995 | | 1.02187 | 1.06637 | | 1.04217 | |
| 0.991857 | | 1.04478 | |  | 1 | 1.00436 | | 1.02655 | | 0.91498 | 0.975265 | | 1.13575 | |
| 1.01272 | | 1.00774 | | 0.983117 | | 0.961942 | | 1.00769 | | NA | 1.10462 | | 1.05596 | |
| 0.988978 | | NA |  | NA |  | NA |  | NA |  | NA | NA |  | 1.01386 | |
| 0.988978 | | NA |  | NA |  | NA |  | NA |  | NA | NA |  | 1.01386 | |
| 0.988978 | | NA |  | NA |  | NA |  | NA |  | NA | NA |  | 1.01386 | |

| 0.98825 | | 0.981816 | 0.984263 | | 0.974879 | | 0.985129 | | 1.02266 | | 1.00431 | | 0.966662 | |
| --- | --- | --- | --- | --- | --- | --- | --- | --- | --- | --- | --- | --- | --- | --- |
| 1.00232 | | 0.995854 | 0.973724 | | 0.980402 | | 0.996956 | | 0.990819 | | 1.02846 | | 0.972978 | |
| 1.03662 | | 1.12468 | 1.13429 | | 1.10629 | | 1.09236 | | 1.12249 | | 1.03632 | | NA |  |
| 0.975381 | | 0.960767 | 0.95895 | | 0.970758 | | 0.978728 | | 0.968846 | | 1.05456 | | 0.985502 | |
| 0.97554 | | NA | NA |  | NA |  | NA |  | NA |  | NA |  | 1.05697 | |
| 0.921671 | | NA | NA |  | NA |  | NA |  | NA |  | NA |  | 0.922785 | |
| 0.943639 | | NA | NA |  | NA |  | NA |  | NA |  | NA |  | 0.997201 | |
| 1.01357 | | 1.01978 | 1.06001 | | 1.04681 | | 1.11127 | | 1.16296 | | 1.16528 | | 1.04803 | |
| 0.992959 | | 1.07523 | 1.04128 | | 1.02897 | | 1.10345 | | 1.08865 | | 1.13492 | | 0.998537 | |
| 1.01059 | | 1.08682 | 1.09474 | | 1.08845 | | 1.08396 | | 1.04565 | | 1.02513 | | 1.02471 | |
| 1.00539 | | 1.04902 | 1.00115 | | 0.990839 | | 1.01126 | | 1.08181 | | 1.04832 | |  | 0.9581 |
| 1.00539 | | 1.04902 | 1.00115 | | 0.990839 | | 1.01126 | | 1.08181 | | 1.04832 | |  | 0.9581 |
| 1.03301 | | 1.05756 | 1.02313 | | 1.01408 | | 1.06158 | | 1.06209 | | 1.03959 | | 1.07316 | |
| 0.985117 | | 0.978938 | 0.976121 | | 0.940245 | | 0.963403 | | NA |  | NA |  | 1.06516 | |
| 0.985117 | | 0.978938 | 0.976121 | | 0.940245 | | 0.963403 | | NA |  | NA |  | 1.06516 | |
| NA |  | 0.958547 | 0.965452 | | 0.951973 | | NA |  | NA |  | 0.960283 | | NA |  |
| NA |  | NA | 0.947835 | | 0.926903 | | NA |  | NA |  | NA |  | NA |  |
| NA |  | NA | 0.913384 | | 0.930284 | | NA |  | NA |  | NA |  | NA |  |
| 1.09833 | | 1.06573 | 1.06255 | | 1.04181 | | 1.11844 | | 1.07086 | | 1.12274 | | NA |  |
| 1.03767 | | 1.05475 | 1.09438 | | 1.10412 | | 1.08003 | | 1.05119 | | 1.06338 | | 1.09112 | |
| 0.956376 | | 1.12416 | 1.09856 | |  | 1.0934 | 1.07441 | | 1.07504 | | 1.08263 | |  | 1.0039 |
| 0.93511 | | 1.04602 |  | 1.0515 | 1.04531 | | 1.03872 | | 1.01359 | | 1.03273 | | NA |  |
| 0.974939 | | 1.01022 | 1.05778 | | 1.04999 | |  | 1.0488 | 1.00562 | | 1.02949 | | 0.930167 | |
| 0.985754 | | 1.06933 | 1.07957 | | 1.09743 | | 1.02575 | | 1.03306 | |  | 1.0588 | 0.996192 | |
|  | 1.0046 | 1.06918 |  | 1.0863 | 1.10765 | | 1.06379 | | 1.03496 | | 1.07292 | | 0.977326 | |
| 1.02547 | | 1.05894 | 1.03504 | | 1.03429 | | 1.06532 | | 1.03619 | | 1.07477 | |  | 1.0393 |
|  | 1.007 | 0.990038 | 1.01194 | | 1.02457 | | 0.983488 | |  | 1.0567 | 1.04834 | | 0.962819 | |
| 0.984158 | | 1.05687 | 1.08841 | |  | 1.0458 | 1.02038 | | 1.12065 | | 1.06475 | |  | 1.0777 |
| 1.02999 | | 0.916064 | 0.900756 | | 0.932765 | | 0.952285 | | 0.873742 | | 0.894987 | | 1.04616 | |
| 0.98566 | | 1.03879 | 1.02157 | | 0.997202 | | 1.02332 | |  | 1.0019 | 1.07433 | | 1.04968 | |
| 0.936611 | | 0.898222 | 0.946234 | | 0.901235 | |  | 0.9014 | 0.85406 | | 0.924419 | | 0.995775 | |
| 0.964173 | | NA | NA |  | NA |  | NA |  | 0.897454 | | NA |  | 1.00368 | |
| NA |  | NA | 1.02058 | |  | 1.0098 | NA |  | NA |  | NA |  | NA |  |
| 0.984692 | | NA | NA |  | NA |  | NA |  | NA |  | NA |  | 1.02592 | |
| 0.978732 | | NA | NA |  | NA |  | NA |  | NA |  | NA |  | 0.985512 | |
| 1.06657 | | 1.10095 | NA |  | NA |  |  | 1.0764 | 1.06669 | | 1.02671 | | 0.987665 | |
|  | 1.0517 | 1.04947 | 1.00701 | | 1.02911 | | 1.04398 | | 1.04149 | | 1.00752 | | 1.03368 | |
| 1.03129 | | 0.99906 | 0.972079 | | 0.975912 | | 1.02031 | | 1.06042 | | 1.00037 | | 1.05606 | |
| 1.10489 | | NA | NA |  | NA |  | NA |  | NA |  | NA |  | NA |  |

| 1.06188 | | 1.06614 | | 1.04426 | | 1.03859 | | 1.01606 | | 1.11364 | | 0.998083 | 1.02156 | |
| --- | --- | --- | --- | --- | --- | --- | --- | --- | --- | --- | --- | --- | --- | --- |
| 0.987339 | | 0.953147 | | 0.925973 | | 0.928238 | | 0.951746 | | 0.884947 | | 0.928323 | 1.02925 | |
| 1.16401 | | 1.16044 | | 1.10508 | | 1.09649 | | 1.09237 | | 1.11935 | | 1.11774 | 1.07955 | |
| 1.17028 | | 1.08144 | | 1.06088 | | 1.06463 | | 1.03321 | | 1.06588 | | 1.07683 | 1.08645 | |
| 1.11848 | | 1.03703 | |  | 1.028 | 1.00331 | | 1.04417 | | 1.04442 | | 1.02244 | 1.05741 | |
| 0.97519 | | 1.02536 | | 0.989448 | | 1.02209 | |  | 1.0492 | 0.99053 | | 0.995025 | 0.977221 | |
|  | 1.0002 | 0.948134 | | NA |  | NA |  | 0.972808 | | NA |  | 0.953262 | 1.04199 | |
| 1.12937 | |  | 1.0402 | 1.00445 | | 1.05715 | | 1.02862 | | 0.994056 | | 1.03628 | 1.05973 | |
| 1.13717 | | 1.12262 | | 1.08554 | | 1.10251 | | 1.05676 | | 1.10518 | | 1.13741 | 1.09462 | |
| NA |  | NA |  | NA |  | NA |  | NA |  | NA |  | NA | NA |  |
| NA |  | NA |  | NA |  | NA |  | NA |  | NA |  | NA | NA |  |
| NA |  | NA |  | NA |  | NA |  | NA |  | NA |  | NA | NA |  |
| 1.22695 | | 1.25473 | | 1.19092 | | 1.20703 | | 1.22469 | | 1.15435 | | 1.19229 |  | 1.0364 |
| 1.16584 | | 1.09602 | | 1.12887 | | 1.11812 | |  | 1.1539 | 1.14881 | | 1.11887 | 1.14329 | |
| 0.970625 | | 0.899999 | | NA |  | NA |  | 0.898496 | | 0.937582 | | 0.936861 |  | 1.0483 |
| 1.16849 | | 1.01772 | | 1.04704 | | 1.05708 | | 1.06386 | | 1.05771 | | 1.09516 | 1.11687 | |
| 0.970249 | | 0.895948 | | NA |  | NA |  | 0.87939 | | 0.954463 | | 0.908512 |  | 1.0313 |
| 1.10502 | | NA |  | NA |  | NA |  |  | 1.0093 | 0.985555 | | 1.00602 | NA |  |
| 1.03127 | | 1.01286 | | 1.01749 | | NA |  | 1.01784 | | 1.03203 | | 1.00481 |  | 1.0141 |
| NA |  | NA |  | NA |  | NA |  | NA |  | NA |  | NA | NA |  |
| 1.17157 | | 1.13417 | | 1.12255 | | 1.14689 | | 1.10939 | |  | 1.0329 | 1.05927 | 1.11462 | |
| 1.01026 | | 0.986987 | | 0.997032 | | 1.00416 | |  | 1.0296 | 0.942161 | | 1.00538 | 1.03734 | |
| 1.08529 | | NA |  | NA |  | NA |  | NA |  | NA |  | NA | NA |  |
| 1.11703 | | NA |  | NA |  | NA |  | NA |  | NA |  | NA |  | 1.0997 |
| 1.10729 | | 1.03052 | |  | 1.0304 | 0.997522 | | 1.05017 | | 1.03204 | | 1.04749 | 1.04395 | |
| 1.04594 | | 0.992454 | | 0.973243 | | 0.973407 | | 0.986262 | | 1.03184 | | 1.08432 | 1.03471 | |
| 1.10222 | | 1.02042 | | 0.999483 | | 1.01608 | |  | 1.0706 | 1.01209 | | 1.03521 | 1.03242 | |
| 1.04562 | | 1.01034 | | 1.04362 | | 1.02354 | | 0.998306 | | 1.07551 | | 1.05244 | 1.04429 | |
| 1.04373 | | 1.02904 | | 1.04955 | | 1.02939 | | 1.00745 | | 0.946675 | | 0.934508 | 1.02503 | |
| 1.19343 | | 1.12575 | | 1.09252 | | 1.11432 | | 1.11969 | | 1.06706 | | 1.08361 | 1.09059 | |
| 1.11834 | | 1.02787 | | 0.999897 | | 1.02072 | | 1.02617 | | 0.993588 | | 1.03514 | 0.984602 | |
| 1.07609 | | 1.12493 | | 1.12844 | | 1.14626 | | 1.07246 | | 1.17448 | | 1.23388 | 1.05786 | |
| 1.09443 | | 1.07872 | | 1.02131 | | 1.05178 | | 1.01995 | | 1.00911 | | 1.06334 | 1.04529 | |
| 1.06856 | | 0.966517 | |  | 0.9934 |  | 1.0056 | 0.988836 | | 0.948311 | | 0.931059 | 1.02511 | |
| 1.14378 | | 1.16584 | | 1.13627 | |  | 1.1303 | 1.14006 | | 1.04328 | | 1.13079 | NA |  |
| 0.998948 | | 1.01599 | | 1.03588 | | 1.02664 | | 1.01823 | | 1.01921 | | 1.02125 | 0.987599 | |
| 0.990806 | | 1.00475 | | 1.03316 | | 1.00777 | | 1.00291 | |  | 1.0135 | 0.999335 | 0.970695 | |
| 1.08563 | | 0.989529 | | 1.03511 | | 1.02102 | | 0.972022 | | 1.05589 | | 0.98991 | 0.993507 | |
| 1.07643 | | 1.11923 | | 1.11682 | | 1.09166 | |  | 1.0797 | 1.05619 | | 1.04634 | 1.09523 | |

| 1.05415 | | 1.02974 | | 1.07116 | | 1.08504 | | 1.04066 | | 1.02222 | | 0.99757 | | 1.04086 | |
| --- | --- | --- | --- | --- | --- | --- | --- | --- | --- | --- | --- | --- | --- | --- | --- |
| 1.00826 | | 1.00255 | | 1.01133 | | 1.03605 | | 1.02742 | | 1.08399 | | 1.03691 | | 0.988359 | |
| 1.17195 | | 1.14089 | | 1.12943 | | 1.17209 | | 1.16295 | | 1.13501 | | 1.09933 | | 1.11492 | |
| 1.05352 | | 1.03037 | | 1.06161 | | 1.03924 | | 1.05993 | | 1.02722 | | 0.976059 | | 1.06215 | |
|  | 1.3206 | 1.20716 | | 1.26345 | | 1.25579 | | 1.21429 | | 1.17925 | | 1.29962 | | 1.10849 | |
| 1.27059 | | NA |  | 0.98717 | |  | 0.9975 | NA |  | NA |  | NA |  | 1.01405 | |
| 0.991422 | | 0.988056 | | 0.977624 | | 0.971068 | | 0.98789 | | 0.978567 | | 0.98931 | | 1.01772 | |
| 0.97912 | | 0.995643 | | 0.99473 | | 0.995117 | | 0.990114 | | 0.989012 | | 0.993782 | | 0.998194 | |
| 1.11816 | | 1.12752 | | 1.11957 | | 1.11441 | | 1.06859 | |  | 1.0326 | 1.09463 | | 1.08845 | |
| 1.05979 | | 1.03533 | | 1.04012 | | 1.04293 | | 0.976098 | | 1.05482 | |  | 1.0235 | 1.02357 | |
|  | 1.0526 | 1.03762 | | 0.986672 | | 0.985273 | | 0.989032 | | 0.982309 | | 0.992209 | |  | 1.0557 |
| NA |  | NA |  | NA |  | NA |  | NA |  | NA |  | NA |  | NA |  |
| 1.04768 | | 0.969835 | | 0.968806 | | 0.987311 | | 0.984082 | | 0.991035 | | 1.01497 | | 0.984696 | |
| 1.12579 | | 1.12145 | | 1.09422 | | 1.09491 | | 1.10964 | | 1.08519 | | 1.07376 | | NA |  |
| 1.12246 | | 1.09799 | | 1.09902 | | 1.08432 | | 1.10531 | | 1.03567 | | 1.07681 | | 0.983276 | |
| 1.02954 | | 1.05568 | | 1.03129 | | 1.04607 | |  | 1.0403 | 1.05027 | | 1.05369 | | 1.02237 | |
| 1.04979 | | 1.12566 | | NA |  | NA |  | 1.12308 | | 1.07339 | | 1.06413 | | 0.968312 | |
| 1.04355 | |  | 1.0429 | 0.993799 | | 1.04332 | | 1.02899 | | 1.01971 | | 1.06743 | |  | 1.0071 |
| 0.962631 | | NA |  | 1.00176 | |  | 1.0174 | NA |  | NA |  | NA |  | 1.01978 | |
| 1.03805 | | 1.08866 | |  | 1.0371 | 1.06542 | | 1.08375 | | 1.07853 | | 1.10744 | | 0.975239 | |
| 1.06331 | | 1.04906 | | 1.02487 | | 1.02672 | | 1.02962 | |  | 1.0325 | 1.03222 | | 1.03723 | |
| 0.978789 | | 0.999179 | | 1.02628 | | 1.04152 | | 0.93954 | | 0.93197 | | 0.963834 | | NA |  |
| 1.01176 | | 0.997454 | | 1.00289 | | 0.973444 | | 0.968355 | | 0.985992 | | 0.992201 | | 1.03538 | |
| 0.988971 | | 1.00427 | | 1.00767 | | 0.993815 | | 0.991465 | | 1.03868 | | 1.02596 | | 0.987513 | |
| 0.99973 | | 1.00695 | | 1.00488 | | 1.00842 | | 0.998758 | | 1.01487 | | 1.01019 | | 1.01695 | |
| 1.07833 | | 1.12685 | | 1.13082 | | 1.14475 | | 1.03487 | | 1.09996 | | 1.13452 | | 1.06144 | |
| 1.07741 | | 1.02556 | | 1.04064 | | 1.01375 | | 1.02258 | | 1.06153 | | 1.04696 | | 1.07137 | |
| 1.09177 | | 1.04156 | | 1.04167 | | 1.01747 | | 1.05618 | | 1.00901 | | 1.04939 | | 0.971575 | |
| 1.01845 | | 1.02892 | | 1.00885 | | 1.00696 | | 1.02092 | | 1.01269 | | 1.00287 | | 0.99682 | |
|  | 1.0048 |  | 1.0149 | 0.968825 | |  | 0.9576 | 0.96554 | | 0.983174 | | 0.991883 | |  | 0.9875 |
| 0.999361 | |  | 1.0173 | 1.03647 | | 1.03479 | | 1.02875 | | 1.02421 | | 1.03773 | | 0.994656 | |
| 1.30188 | | 1.22619 | | 1.18795 | | 1.21484 | | 1.21809 | |  | 1.1934 | 1.20933 | | 1.14587 | |
| 1.03931 | | 0.960141 | | 0.978269 | | 0.971055 | | 0.995363 | | 0.973383 | | 0.966644 | | 1.01505 | |
| 1.01659 | | 1.02503 | | 1.01585 | | 1.01871 | | 1.01245 | | 1.09402 | | 1.04726 | | 0.957278 | |
| 1.15835 | | 1.11625 | | NA |  | NA |  | 1.10898 | | 1.05951 | | NA |  | NA |  |
| 1.01558 | | 1.04801 | | 1.05529 | | 1.03384 | | 1.07594 | | 1.05823 | | 1.08262 | | 1.01507 | |
| 1.07049 | | 1.03149 | | 1.08331 | | 1.08594 | | 1.05271 | |  | 1.0302 | 1.06976 | | 1.01789 | |
| 1.00966 | | 1.01833 | | 1.03545 | | 1.05128 | | 1.02564 | | 1.04002 | | 1.04557 | | 1.02075 | |

myeloid_c naive_b_c naive_cd4 natural_kil neural_pro neurosphe neurosphe neutrophil

1.00357	1.07143	1.04093	1.17665	1	1.15425	1.19501	1.0389

NA	NA	NA	NA	NA	1.05098 NA	NA

1.18222	1.13945	1.15393	1.15449 NA	1.06865	1.02204	1.15829

1.14842	1.15017	1.2248	1.13831	1.15667	1.09585	1.14787	1.08273

1.22207	1.26705	1.1073	1.1203 NA	NA	0.990074	1.06344

0.912888 0.958421 0.872382 0.956512 0.727273 0.953383 0.965355 0.946655

1.14928	1.10634	1.10867	1.07213 NA	1.09859	1.0177	1.09675

1.04144	1.13547	1.11396	1.13795 NA	1.16414	1.15175	1.08917

1.04144	1.13547	1.11396	1.13795 NA	1.16414	1.15175	1.08917

1.27457	1.29464 NA	1.01815 NA	1.17154	1.39478	1.2454

0.950215 0.988197	1.00186 0.951343 NA	1.05978	1.05563 0.964787

0.999606	1.02159	1.00369 0.976157 NA	1.04755	1	1.02199

0.99096	1.04962 NA	0.962526 NA	NA	1.13255	1.05456

1.07121 0.990183 0.921551	1.03108 NA	0.951625 0.901196	1.04412

NA	NA	NA	NA	NA	0.985483 NA	NA NA	NA	NA	NA	NA	1.02668	1.09772 NA

0.984523 NA	NA	NA	NA	0.978909 NA	NA

0.984523 NA	NA	NA	NA	0.978909 NA	NA

0.94711	1.00074	1.01052 0.941697 NA	0.948101 0.797223	1.00878

1.25547	1.22092	1.15391	1.12949 NA	1.11328	1.072	1.12215

NA	NA	NA	NA	NA	1.02342	1.08276 NA

1.0269	0.99949	1.04012	1.06372 NA	1.03695 0.995514	1.00372

NA	NA	NA	NA	NA	NA	NA	NA NA	NA	NA	NA	NA	NA	NA	NA

1.0151 NA	NA	NA	NA	NA	NA	1.00601

1.06637 NA	NA	NA	NA	NA	NA	1.02654

1.0861 NA	NA	NA	NA	NA	NA	1.03463

1.04829 NA	NA	NA	NA	NA	NA	1.02087

1.17443	1.10464	1.00303	1.13126 NA	1.14187	1.07669	1.14716

1.17443	1.10464	1.00303	1.13126 NA	1.14187	1.07669	1.14716

1.1588	1.05997	1.13561	1.09367 NA	1.18239	1.03661	1.17774

1.18126	1.03724	1.08824	1.16748 NA	1.24636 0.976909	1.28686

1.16352 NA	NA	1.04951 NA	1.14129 NA	1.11706

0.913313 NA	NA	NA	NA	NA	NA	1.03545

NA	NA	NA	NA	NA	1.05926 NA	NA NA	NA	NA	NA	NA	1.04046 NA	NA NA	NA	NA	NA	NA	1.03724	1.10246 NA

1.03525	1.09017	1.15319	1.06572	0.65625 0.989029	1.02444	1.07281

| 0.959948 | | 0.920759 | | NA |  | 0.964598 | | NA |  | 0.99528 | | 0.932517 | |  | 0.9521 |
| --- | --- | --- | --- | --- | --- | --- | --- | --- | --- | --- | --- | --- | --- | --- | --- |
| 0.959948 | | 0.920759 | | NA |  | 0.964598 | | NA |  | 0.99528 | | 0.932517 | |  | 0.9521 |
| 0.959948 | | 0.920759 | | NA |  | 0.964598 | | NA |  | 0.99528 | | 0.932517 | |  | 0.9521 |
| 0.936132 | |  | 1.0856 | 1.05777 | | 0.936376 | | 0.540541 | | 1.04399 | | 1.11346 | | 1.06373 | |
| 0.994818 | | 1.05326 | | 1.16241 | | 0.963115 | | 0.486486 | | 0.999976 | | 1.10548 | | 1.02685 | |
| 0.955149 | | 1.00376 | | 0.926027 | | 1.01768 | | NA |  | 1.06071 | | 0.879675 | | 0.984821 | |
|  | 1.0326 | 1.02115 | | 0.988434 | |  | 1.0487 | NA |  | 1.05189 | | 0.960517 | | 1.07587 | |
| 1.01794 | | 1.03064 | | 0.98431 | | 1.03156 | | NA |  | 1.06033 | | 0.877901 | | 1.04619 | |
| 0.974685 | | 0.990533 | | 0.941649 | | 1.04663 | | NA |  | 1.00862 | | 0.939337 | | 1.00935 | |
| 1.02694 | | 1.00086 | | 0.995254 | | 1.04329 | | NA |  | 1.04702 | | 0.979768 | | 1.05651 | |
| 1.04757 | | 1.09406 | | 1.03028 | | 1.03663 | |  | 1 | 1.03938 | | 0.926113 | | 1.09758 | |
| 1.04447 | | 1.03237 | | 0.965889 | | 1.01896 | | NA |  | 0.986471 | | 0.916503 | | 1.07406 | |
|  | 1.0729 |  | 1.1244 | 1.15073 | | 1.13931 | |  | 0.72 | 1.07964 | |  | 1.0442 | 1.09327 | |
| 1.07174 | | 1.06195 | | 1.07595 | | 1.05102 | | NA |  | 1.01084 | | 0.945636 | | 1.06598 | |
|  | 1.0398 | 1.09693 | | 1.02602 | | 1.09108 | |  | 0.875 | 1.03733 | | 1.02548 | | 1.05711 | |
| NA |  | NA |  | NA |  | NA |  | NA |  |  | 1.0461 | 1.10654 | | NA |  |
|  | 1.1028 | 1.07472 | | 1.15996 | | 1.14552 | | NA |  |  | 1.0704 | 1.09155 | | 1.09105 | |
|  | 1.0647 | 1.11773 | | 1.02085 | | 1.10187 | | NA |  | 1.04359 | | 0.984094 | | 1.07007 | |
| 1.02565 | | 1.09752 | | 1.06314 | | 1.10252 | | NA |  | 1.02016 | | 1.02072 | | 1.12845 | |
| 0.976632 | | 1.10118 | |  | 1.3625 | 1.18255 | | NA |  | 0.911364 | | 0.969697 | |  | 1.1342 |
| 1.01357 | | 1.07441 | | 0.916873 | | 1.04827 | | NA |  | 0.951718 | | 0.837201 | | 1.01542 | |
| NA |  | NA |  | NA |  | NA |  | NA |  | NA |  | NA |  | NA |  |
| NA |  | NA |  | NA |  | NA |  | NA |  |  | 1.0368 | 1.11967 | | NA |  |
|  | 1.0346 | 1.06786 | | 1.01909 | | 1.08917 | | NA |  | 1.00363 | | 0.942432 | | 1.05138 | |
| 1.12698 | | 1.29286 | | 1.34615 | | 1.07018 | | NA |  | 1.10309 | | 0.947368 | | 1.02941 | |
| 1.09949 | | 1.05966 | | 1.11765 | | 1.05689 | | NA |  | 1.01687 | | 0.969817 | | 1.05801 | |
| 1.07751 | | 1.11713 | | 1.07244 | | 1.08589 | | NA |  | 1.03829 | | 0.990335 | | 1.08156 | |
| 0.960605 | | 1.08975 | |  | 1.1195 | 1.02832 | | NA |  | 1.05467 | | 1.01653 | | 1.04954 | |
| 1.02345 | | 1.07405 | | 1.11145 | | 1.06011 | | NA |  |  | 1.0907 | 1.01665 | | 1.08102 | |
| 1.02345 | | 1.07405 | | 1.11145 | | 1.06011 | | NA |  |  | 1.0907 | 1.01665 | | 1.08102 | |
| 1.02479 | | 1.10619 | | 1.03537 | | 1.09017 | | NA |  | 0.991768 | | 0.950024 | | 1.08419 | |
| 1.10397 | | 1.07719 | |  | 1.0602 | 1.06823 | | NA |  | 1.12953 | | 0.97824 | | 1.08969 | |
| 1.03749 | | 1.07925 | | 0.956566 | | 1.04479 | | NA |  | NA |  | 0.93914 | | 1.05928 | |
| 0.950836 | |  | 1.0036 | 0.972806 | | 1.05659 | | NA |  | 1.00555 | | 0.944523 | | 1.01261 | |
| NA |  | NA |  | NA |  | NA |  | NA |  | 1.01975 | | 1.07015 | | NA |  |
| 0.99313 | | 1.07851 | | 0.953344 | | 0.979574 | | NA |  | 1.02815 | |  | 1.0564 | 1.03004 | |
| 1.04971 | | 1.07476 | |  | 1.0006 | 1.10604 | | NA |  | 1.04223 | | 1.00719 | | 1.07613 | |
| 1.02674 | | 1.08491 | | 1.04813 | | 1.06829 | | NA |  | 0.993027 | | 0.917365 | | 1.04945 | |
| 1.09378 | | 1.23795 | |  | 1.5679 | 1.18096 | |  | 1 |  | 1.0314 | 0.986842 | | 1.16332 | |

| 1.00878 | | 1.03076 | NA |  |  | 1.0275 | NA | 0.945036 | | 0.970301 | 1.02416 |
| --- | --- | --- | --- | --- | --- | --- | --- | --- | --- | --- | --- |
| 0.968198 | | 0.966186 | NA |  | 0.908315 | | NA | 1.00303 | | 1.00317 | 0.98488 |
| 1.04623 | | 1.05196 | 1.03482 | | 0.991019 | | NA | 1.01641 | | 1.03003 | 1.04378 |
| 1.00656 | | 1.04118 | NA |  | 1.02135 | | NA | NA |  | 1.03796 | 0.981527 |
| NA |  | NA | NA |  | NA |  | NA | NA |  | NA | NA |
| NA |  | NA | NA |  | NA |  | NA | NA |  | NA | NA |
| NA |  | NA | NA |  | NA |  | NA | NA |  | NA | NA |
| NA |  | NA | NA |  | NA |  | NA | 1.00398 | | NA | NA |
| NA |  | NA | NA |  | NA |  | NA | 1.00212 | | NA | NA |
| NA |  | NA | NA |  | NA |  | NA | 1.00584 | | NA | NA |
| NA |  | NA | NA |  | NA |  | NA | NA |  | NA | NA |
| NA |  | NA | NA |  | NA |  | NA | NA |  | NA | NA |
| NA |  | NA | NA |  | NA |  | NA | NA |  | NA | NA |
| NA |  | NA | NA |  | NA |  | NA | NA |  | NA | NA |
| NA |  | NA | NA |  | NA |  | NA | 0.996983 | | NA | NA |
| NA |  | 1.01695 | NA |  | NA |  | NA | 1.00194 | | NA | NA |
| NA |  | NA | NA |  | NA |  | NA | 1.00697 | | NA | NA |
| NA |  | NA | NA |  | NA |  | NA |  | 0.9969 | NA | NA |
| NA |  | 1.05718 | NA |  | NA |  | NA | 1.02475 | | 0.908092 | 1.03727 |
| NA |  | NA | NA |  | NA |  | NA | 0.985289 | | NA | NA |
| NA |  | NA | NA |  | NA |  | NA | NA |  | NA | NA |
| 1.06283 | | 1.08703 |  | 1.0356 | 1.08937 | | 1.29167 | 1.09777 | | 0.992715 | 1.08918 |
| 0.919811 | | 0.922077 | NA |  | 0.974558 | | NA | 0.924265 | | NA | 0.964228 |
| 0.919811 | | 0.922077 | NA |  | 0.974558 | | NA | 0.924265 | | NA | 0.964228 |
| NA |  | NA | NA |  | NA |  | NA | NA |  | NA | NA |
| NA |  | NA | NA |  | NA |  | NA | NA |  | NA | NA |
| NA |  | NA | NA |  | NA |  | NA | NA |  | NA | NA |
| 1.02373 | | 0.962152 | NA |  | 1.02011 | | NA | NA |  | NA | 1.00675 |
| NA |  | NA | NA |  | NA |  | NA | NA |  | NA | NA |
| NA |  | NA | NA |  | NA |  | NA | NA |  | NA | NA |
| NA |  | NA | NA |  | NA |  | NA | NA |  | NA | NA |
| NA |  | NA | NA |  | NA |  | NA | NA |  | NA | NA |
| NA |  | NA | NA |  | NA |  | NA |  | 1.0322 | NA | NA |
| NA |  | NA | NA |  | NA |  | NA | 1.02409 | | 1.10451 | NA |
| NA |  | NA | NA |  | NA |  | NA |  | 1.0518 | 1.08531 | NA |
| NA |  | NA | NA |  | NA |  | NA | 1.04913 | | 1.06985 | NA |
| 0.966768 | | 0.978356 | 0.849197 | | 0.96162 | | NA | 0.998282 | | 1.03449 | 1.00628 |
|  | 1.069 | 1.07529 | 1.17916 | | 1.11087 | | 0.857143 | 1.13311 | | 1.01714 | 1.12282 |
|  | 1.069 | 1.07529 | 1.17916 | | 1.11087 | | 0.857143 | 1.13311 | | 1.01714 | 1.12282 |

| 1.19118 | 1.14089 | | 1.19529 | | 1.14267 | | 0.290646 | 1.27277 | | 1.28168 | 1.11525 | |
| --- | --- | --- | --- | --- | --- | --- | --- | --- | --- | --- | --- | --- |
| NA | NA |  | 0.990826 | | 1.03944 | | NA | NA |  | NA | NA |  |
| 1.16506 |  | 1.0521 |  | 1.1383 | 1.08731 | | 1.33333 | 1.12477 | | 0.979855 | 1.05834 | |
| 0.970108 | 0.953592 | | 0.999813 | | 0.983378 | | 0.923077 | 1.01966 | | 1.08355 | 0.943026 | |
| 1.03928 | 1.00683 | | 1.03191 | |  | 1.0652 | NA | 0.954889 | | 0.97964 | 1.08258 | |
| 0.984854 | NA |  | NA |  | NA |  | NA | NA |  | NA | 1.06071 | |
| NA | NA |  | NA |  | NA |  | NA | NA |  | NA | NA |  |
| NA | NA |  | NA |  | NA |  | NA | NA |  | NA | NA |  |
| 0.905303 | NA |  | NA |  | 0.971441 | | NA | 0.977862 | | 0.977534 | NA |  |
| 1.08122 | NA |  | NA |  | 1.05269 | | NA | NA |  | 0.978426 | 0.992743 | |
| 0.94088 | 0.994135 | | NA |  | 0.965615 | | NA |  | 1.0014 | 1.05185 |  | 0.9397 |
| NA | NA |  | NA |  | NA |  | NA | 1.05592 | | 1.11028 | NA |  |
| NA | NA |  | NA |  | NA |  | NA | 0.989114 | | NA | NA |  |
| NA | NA |  | NA |  | NA |  | NA | NA |  | NA | NA |  |
| NA | NA |  | NA |  | NA |  | NA | NA |  | NA | NA |  |
| NA | NA |  | NA |  | NA |  | NA | NA |  | NA | NA |  |
| NA | NA |  | NA |  | NA |  | NA | 1.03316 | | 1.10044 | NA |  |
| NA | NA |  | NA |  | NA |  | NA | 1.03781 | | 1.05423 | NA |  |
| NA | NA |  | NA |  | NA |  | NA | 1.00769 | | 1.08549 | NA |  |
| NA | NA |  | NA |  | NA |  | NA | 0.985151 | | NA | NA |  |
| NA | NA |  | NA |  | NA |  | NA | 0.956691 | | NA | NA |  |
| NA | NA |  | NA |  | NA |  | NA | 0.993531 | | 1.05722 | NA |  |
| NA | NA |  | NA |  | NA |  | NA | 1.01703 | | 1.04344 | NA |  |
| NA | NA |  | NA |  | NA |  | NA | 1.05598 | | 1.04531 | NA |  |
| NA | NA |  | NA |  | NA |  | NA | NA |  | NA | NA |  |
| NA | NA |  | NA |  | NA |  | NA | 0.987415 | | NA | NA |  |
| NA | NA |  | NA |  | NA |  | NA | 0.971834 | | NA | NA |  |
| NA | NA |  | NA |  | NA |  | NA | 1.06529 | | NA | NA |  |
| NA | NA |  | NA |  | NA |  | NA | 1.03405 | | 1.08718 | NA |  |
| NA | NA |  | NA |  | NA |  | NA | 1.03079 | | 1.08698 | NA |  |
| NA | NA |  | NA |  | NA |  | NA | 1.02155 | | 1.06691 | NA |  |
| NA | NA |  | NA |  | NA |  | NA | 1.05333 | | 1.09184 | NA |  |
| NA | NA |  | NA |  | NA |  | NA | 0.978655 | | NA | NA |  |
| NA | NA |  | NA |  | NA |  | NA | 0.985503 | | NA | NA |  |
| NA | NA |  | NA |  | NA |  | NA | 1.01559 | | NA | NA |  |
| NA | NA |  | NA |  | NA |  | NA | 1.08828 | | NA | NA |  |
| NA | NA |  | NA |  | NA |  | NA | NA |  | NA | NA |  |
| NA | NA |  | NA |  | NA |  | NA | 1.00168 | | NA | NA |  |
| NA | NA |  | NA |  | NA |  | NA |  | 1.0835 | NA | NA |  |

| NA |  | NA | NA | NA |  | NA |  |  | 1.0138 | 1.03775 | | NA |
| --- | --- | --- | --- | --- | --- | --- | --- | --- | --- | --- | --- | --- |
| NA |  | 0.989553 | NA | NA |  | NA |  | 1.01862 | | NA |  | NA |
| NA |  | NA | NA | NA |  | NA |  | 1.05507 | | 1.12708 | | NA |
| NA |  | NA | NA | NA |  | NA |  | NA |  | NA |  | NA |
| NA |  | NA | NA | NA |  | NA |  | NA |  | NA |  | NA |
| NA |  | NA | NA | NA |  | NA |  | 1.08167 | | NA |  | NA |
| NA |  | NA | NA | NA |  | NA |  | 1.03124 | | NA |  | NA |
| NA |  | NA | NA | NA |  | NA |  | 1.06939 | | NA |  | NA |
| NA |  | NA | NA | NA |  | NA |  | 1.04453 | |  | 1.0641 | NA |
| NA |  | NA | NA | NA |  | NA |  |  | 1.0379 | NA |  | NA |
| 1.00592 | | 1.00377 | 0.960805 | 0.99043 | | NA |  | 0.917334 | | NA |  | 0.99561 |
| 1.01039 | | 1.03183 | NA | 0.997511 | | NA |  | 1.24508 | | NA |  | 1.00924 |
| NA |  | 1.03383 | NA | NA |  | NA |  | NA |  | NA |  | NA |
|  | 1.0223 | 0.980566 | 0.997365 | 1.00882 | | NA |  | 1.03516 | | NA |  | 0.993159 |
| 1.05698 | | 1.25666 | 0.877289 | 1.08369 | | NA |  |  | 1.104 | NA |  | 1.05049 |
| 1.10258 | | 1.11418 | 1.03119 | 1.05659 | | NA |  | 1.03288 | | NA |  | 1.01023 |
| 1.07941 | | 1.12873 | 1.08474 | 1.12649 | | NA |  | NA |  | NA |  | 1.06933 |
| 0.905632 | | 1.03507 | NA |  | 1.0736 | NA |  | 0.985045 | | NA |  | 1.00446 |
| 1.05162 | | 1.07673 | 0.877069 | 0.990038 | | NA |  | 1.01498 | | NA |  | 1.00917 |
|  | 1.1645 | 1.19545 | 0.960452 | 1.01183 | | NA |  | NA |  | NA |  | 1.06276 |
|  | 1.0461 | 1.05353 | NA | 1.05633 | | NA |  | 1.00895 | | 0.92304 | | 1.03002 |
|  | 1.0216 | 1.02629 | NA | 1.02311 | | NA |  | 0.976383 | | 0.957436 | | 1.04673 |
| 1.02307 | | 1.04949 | NA | 0.96814 | | NA |  | 0.997157 | | 1.05317 | | 1.01585 |
| 1.01166 | | NA | NA | NA |  | NA |  | NA |  | 1.01499 | | NA |
| 0.982364 | | 1.03863 | 1.11593 | 1.01155 | | NA |  | NA |  | 0.91604 | | 1.04957 |
|  | 1.0849 | 1.07124 | NA | 1.10243 | | NA |  | 1.04881 | | 1.09492 | | 1.04849 |
| 1.02299 | | 1.10756 | 1.07192 | 1.05766 | | 0.692308 | | NA |  | 1.09487 | | 0.988298 |
| 1.06157 | | 1.02078 | 1.13242 | 1.06052 | | 1.33333 | | 1.14146 | | 1.24832 | | 0.962462 |
| 1.06157 | | 1.02078 | 1.13242 | 1.06052 | | 1.33333 | | 1.14146 | | 1.24832 | | 0.962462 |
| NA |  | 1.05435 | 1.05903 | 1.00835 | | 0.956522 | | NA |  | 1.03719 | | 0.986526 |
| 1.00518 | | 1.03747 | 0.973085 | 1.06429 | | 0.571429 | | 0.953405 | | 0.98957 | | 1.01059 |
| 1.03803 | | 1.09509 | 1.05124 |  | 1.0294 | NA |  | NA |  | 0.953591 | | 1.05467 |
| 1.03803 | | 1.09509 | 1.05124 |  | 1.0294 | NA |  | NA |  | 0.953591 | | 1.05467 |
| 1.03803 | | 1.09509 | 1.05124 |  | 1.0294 | NA |  | NA |  | 0.953591 | | 1.05467 |
| 0.923385 | | 1.01315 | 1.01903 | 0.935435 | |  | 0.75 | 0.970547 | | 0.979269 | | 0.955282 |
| 0.943004 | | 1.02264 | 0.974217 | 1.00508 | |  | 0.654 | 0.965755 | | 1.00062 | | 1.03835 |
| NA |  | 1.15203 | 1.13968 | 1.08168 | |  | 0.85 | NA |  | NA |  | 1.02269 |
| NA |  | NA | NA | NA |  | NA |  | 1.03936 | | 1.07162 | | NA |
| 1.00264 | | 1.11787 | 1.12084 | 1.05113 | | NA |  | 1.06817 | | 0.996689 | | NA |

| NA |  | NA |  | NA |  | NA |  | NA |  | 0.984269 | | 1.03804 | | NA |  |
| --- | --- | --- | --- | --- | --- | --- | --- | --- | --- | --- | --- | --- | --- | --- | --- |
| NA |  | NA |  | NA |  | NA |  | NA |  | 1.04188 | | 1.03898 | | NA |  |
| NA |  | NA |  | NA |  | NA |  | NA |  | 1.07344 | | 1.10484 | | NA |  |
| NA |  | NA |  | NA |  | NA |  | NA |  | 1.04896 | | NA |  | NA |  |
| 0.901969 | |  | 1.028 | 0.974961 | | 0.96271 | | NA |  | 1.03445 | | 1.05409 | | 0.955728 | |
| 1.03444 | | 0.992125 | | 1.19131 | | 1.00783 | | NA |  | 0.985022 | | 1.11729 | | 1.00787 | |
| 0.901173 | | 1.02039 | | 0.953571 | | 1.00041 | | NA |  | 0.978916 | | 0.955665 | | 1.01859 | |
| 1.03138 | | 0.978308 | | 1.15906 | |  | 1.1122 | NA |  | 1.08657 | | 1.05416 | |  | 1.0612 |
| 0.94856 | | 0.974088 | | 0.903753 | | 0.94652 | | NA |  | 1.01841 | | 1.09841 | | 0.943311 | |
| 1.00282 | | 1.00992 | | 0.90132 | | 0.987713 | | NA |  | 1.02587 | | 1.04412 | | 0.979669 | |
|  | 1.1073 | 1.06028 | | 1.08935 | | 1.10816 | | NA |  | 1.07043 | | 1.03214 | |  | 1.0907 |
| 1.07932 | | 1.09065 | | 1.33806 | | 1.10536 | | 1.38462 | |  | 1.1535 | 1.15912 | | 1.16483 | |
|  | 1.1776 | 1.08884 | | 1.28707 | | 1.16601 | | 1.59333 | |  | 1.2908 | 1.36293 | | 1.18156 | |
| 1.00139 | | 0.915581 | | NA |  | 1.02361 | | NA |  | 0.938294 | | 0.985098 | | 1.02242 | |
| NA |  | NA |  | NA |  | NA |  | NA |  | 1.02201 | | 1.09577 | | NA |  |
| NA |  | NA |  | NA |  | NA |  | NA |  | 1.03166 | |  | 1.1037 | NA |  |
| 1.15182 | | 1.09155 | | 1.07647 | | 1.07135 | | NA |  | NA |  | NA |  | 1.12398 | |
| NA |  | NA |  | NA |  | NA |  | NA |  | 1.03881 | | 1.07598 | | NA |  |
| 0.903499 | | 0.959141 | | 0.980478 | | 0.888789 | | NA |  | 0.975585 | | 0.972109 | | 0.942551 | |
| NA |  | NA |  | NA |  | NA |  | NA |  | 1.03463 | | 1.02605 | | NA |  |
| NA |  | NA |  | NA |  | NA |  | NA |  | 1.03463 | | 1.02605 | | NA |  |
| 1.04722 | | 1.04305 | | 1.20792 | | 1.18428 | |  | 1.1 | 1.08013 | | 1.03117 | |  | 1.094 |
|  | 1.1411 |  | 1.1056 | 1.08471 | | 1.12635 | | NA |  | 1.10725 | | 1.10921 | | 1.12659 | |
|  | 1.1411 |  | 1.1056 | 1.08471 | | 1.12635 | | NA |  | 1.10725 | | 1.10921 | | 1.12659 | |
|  | 1.1411 |  | 1.1056 | 1.08471 | | 1.12635 | | NA |  | 1.10725 | | 1.10921 | | 1.12659 | |
|  | 1.1411 |  | 1.1056 | 1.08471 | | 1.12635 | | NA |  | 1.10725 | | 1.10921 | | 1.12659 | |
| 1.06453 | | 1.17068 | | 1.09347 | | 1.06485 | | NA |  |  | 1.005 | 0.992071 | | 1.02985 | |
| 1.06453 | | 1.17068 | | 1.09347 | | 1.06485 | | NA |  |  | 1.005 | 0.992071 | | 1.02985 | |
| 1.06453 | | 1.17068 | | 1.09347 | | 1.06485 | | NA |  |  | 1.005 | 0.992071 | | 1.02985 | |
| 1.06453 | | 1.17068 | | 1.09347 | | 1.06485 | | NA |  |  | 1.005 | 0.992071 | | 1.02985 | |
| 1.08536 | | 1.17983 | | 1.02788 | |  | 1.1798 | NA |  | 1.09685 | | 1.14482 | |  | 1.0555 |
| 1.08536 | | 1.17983 | | 1.02788 | |  | 1.1798 | NA |  | 1.09685 | | 1.14482 | |  | 1.0555 |
| 0.999499 | | 1.11591 | | 1.12469 | | 1.11007 | | NA |  | 1.11202 | | 1.14441 | | 1.10629 | |
| 0.999499 | | 1.11591 | | 1.12469 | | 1.11007 | | NA |  | 1.11202 | | 1.14441 | | 1.10629 | |
| 1.22647 | | 1.11162 | |  | 1.1341 | 1.21684 | | NA |  | 1.06915 | | 1.02145 | | 1.15326 | |
| 0.886272 | | 0.951656 | | 0.922441 | | 0.915955 | | NA |  | 0.993719 | | 1.05348 | | 0.912737 | |
| 0.886272 | | 0.951656 | | 0.922441 | | 0.915955 | | NA |  | 0.993719 | | 1.05348 | | 0.912737 | |
| 0.966089 | | 0.988118 | | 0.903485 | | 0.917475 | | NA |  | 0.93738 | | 1.02558 | | 0.976268 | |
| 0.966089 | | 0.988118 | | 0.903485 | | 0.917475 | | NA |  | 0.93738 | | 1.02558 | | 0.976268 | |

| 0.924359 | | 0.987805 | 0.982108 | | 1.00276 | | NA |  | 1.01334 | | 1.03579 | | 0.947653 |
| --- | --- | --- | --- | --- | --- | --- | --- | --- | --- | --- | --- | --- | --- |
| 0.917545 | | 0.96139 | 0.954522 | | 0.96673 | | 1.04545 | |  | 1.0174 |  | 1.0416 | 0.948097 |
| 0.986018 | | 0.970588 | 0.971794 | | 1.01929 | | NA |  | 0.964901 | | 0.967449 | | 0.996946 |
| 0.976124 | | 0.938778 | 0.952941 | | 0.99617 | | NA |  |  | 1.0997 | 1.08366 | | 1.06841 |
| 0.993181 | | 0.982614 |  | 1.026 | 0.97079 | | NA |  | 0.995706 | | 1.05518 | | 0.998187 |
| 0.975836 | | 1.03466 | NA |  | 1.03629 | | NA |  |  | 0.95 | 0.895555 | | 1.05282 |
|  | 1.0304 | 0.975549 | 1.00933 | | 0.95246 | | NA |  | 1.04869 | | 1.10474 | | 0.975524 |
|  | 1.0132 | 0.979814 |  | 1.1357 |  | 1.0487 | NA |  | 1.03399 | | 1.00141 | | 1.01736 |
| 1.06309 | | 0.98857 | 1.13018 | | 1.05724 | | NA |  | 1.03632 | | 1.02152 | | 1.00018 |
| 0.972052 | | NA | NA |  | NA |  | NA |  | 1.03822 | | 1.06561 | | NA |
| 1.01307 | | 0.988982 | 1.12326 | | 1.04114 | | NA |  | 1.01691 | | 1.00996 | | 0.970193 |
| 0.985929 | | 1.02908 | 0.948107 | | 1.04817 | | NA |  | 0.994037 | | 1.07879 | | 1.02583 |
| NA |  | 0.919307 | NA |  | 0.898609 | | NA |  | NA |  | NA |  | NA |
| 0.982132 | | 0.96978 | 1.08377 | | 1.01007 | | NA |  | 1.01771 | | 1.05955 | | 1.00047 |
| NA |  | 1.01674 | NA |  | NA |  | NA |  | NA |  | NA |  | NA |
| NA |  | NA | NA |  | NA |  | NA |  | NA |  | 1.00148 | | NA |
| NA |  | NA | NA |  | NA |  | NA |  | NA |  | NA |  | NA |
| 0.937342 | | 0.961465 | NA |  |  | 1.0206 | NA |  | 0.987523 | | 0.990286 | | 1.00885 |
| NA |  | NA | NA |  | NA |  | NA |  | NA |  | NA |  | NA |
| NA |  | NA | NA |  | NA |  | NA |  | NA |  | NA |  | NA |
| NA |  | NA | NA |  | NA |  | NA |  | NA |  | NA |  | NA |
| NA |  | NA | NA |  | NA |  | NA |  | NA |  | NA |  | NA |
| NA |  | NA | NA |  | NA |  | NA |  | NA |  | 0.994063 | | NA |
| 0.955852 | | 0.984901 | NA |  | 1.03695 | | NA |  | NA |  | NA |  | 1.00986 |
| 0.97955 | | 1.01787 | 0.973452 | | 0.979812 | | NA |  | NA |  | 1.10271 | | 1.03543 |
| NA |  | NA | NA |  | NA |  | NA |  | 1.04811 | | 1.07201 | | NA |
| NA |  | NA | NA |  | NA |  | NA |  | 1.03034 | | 1.07945 | | NA |
| NA |  | NA | NA |  | NA |  | NA |  | 1.02308 | | 1.08385 | | NA |
| 1.20542 | | 1.07856 | 1.12787 | | 1.15828 | | NA |  | 1.07762 | |  | 1.063 | 1.13253 |
| 1.03609 | | 1.04664 | 1.17155 | | 0.938484 | |  | 0.95 | 1.08856 | | 1.03718 | | 1.02246 |
| 1.03609 | | 1.04664 | 1.17155 | | 0.938484 | |  | 0.95 | 1.08856 | | 1.03718 | | 1.02246 |
| NA |  | NA | NA |  | NA |  | NA |  | NA |  | NA |  | NA |
| NA |  | NA | NA |  | 1.00732 | | NA |  | NA |  | 1.01333 | | NA |
| NA |  | NA | NA |  | NA |  | NA |  | NA |  | NA |  | NA |
| 0.95883 | | 1.00729 | NA |  | 1.00616 | | NA |  | NA |  | NA |  | 0.949354 |
|  | 1.0341 | 1.08588 | 1.11197 | | 1.05696 | | 1.09524 | | 1.04651 | | 0.918129 | | 1.03469 |
| 1.00869 | | NA | NA |  | 1.05042 | | NA |  | NA |  | NA |  | 0.961019 |
| NA |  | NA | NA |  | NA |  | NA |  | NA |  | NA |  | NA |
| 1.06412 | | NA | NA |  | 1.05908 | | NA |  | NA |  | NA |  | 0.994102 |

| 0.971483 | NA |  | NA |  | 1.02335 | | NA | NA |  | NA |  | 0.929266 | |
| --- | --- | --- | --- | --- | --- | --- | --- | --- | --- | --- | --- | --- | --- |
| NA | NA |  | NA |  | NA |  | NA | NA |  | NA |  | NA |  |
| NA | NA |  | NA |  | NA |  | NA | NA |  | NA |  | NA |  |
| NA | NA |  | NA |  | NA |  | NA | NA |  | NA |  | NA |  |
| NA | NA |  | NA |  | NA |  | NA | NA |  | NA |  | NA |  |
| 1.00995 | 1.00581 | |  | 1.1229 | 1.03237 | | NA | 1.02238 | | 1.13582 | | 0.99766 | |
| NA | NA |  | NA |  | NA |  | NA | NA |  | NA |  | NA |  |
| NA | NA |  | NA |  | NA |  | NA | NA |  | NA |  | NA |  |
| NA | NA |  | NA |  | NA |  | NA | NA |  | NA |  | NA |  |
| NA | NA |  | NA |  | NA |  | NA | 1.05576 | | NA |  | NA |  |
| 0.959578 | 1.03424 | | 0.988506 | | 0.959565 | | NA | 0.824077 | |  | 0.908 | 1.02777 | |
| 1.07936 | 1.08159 | | NA |  | 1.06501 | | NA | 1.05127 | | 1.06807 | | 1.08928 | |
| 1.06742 | 1.02832 | | 0.985264 | | 1.12679 | | NA | 1.06809 | | 1.13946 | | 1.06279 | |
| NA | NA |  | NA |  | NA |  | NA | 1.03081 | |  | 1.1529 | NA |  |
| 1.13151 |  | 1.0633 | 0.965063 | | 1.02562 | | NA | 0.981232 | | 0.935643 | | 1.07071 | |
| 1.08229 | 1.02071 | | NA |  | 0.972833 | | NA | 0.944776 | | 0.975302 | | 1.07022 | |
| 0.961107 | 0.942061 | | NA |  | 1.00197 | | NA | 1.05666 | | 0.939223 | | 1.00724 | |
| 1.05982 | 0.935189 | | NA |  | 0.987912 | | NA | 0.908729 | |  | 1.0057 | 1.02655 | |
| NA | NA |  | NA |  | NA |  | NA | 1.03644 | | 1.09821 | | NA |  |
| 0.947529 | 1.04815 | | 0.929048 | | 0.980968 | | 0.382353 | 1.02766 | | 1.11117 | | 0.997593 | |
| 1.03609 | 1.06909 | | 1.06723 | | 1.00801 | | 1.09813 | 0.996667 | | 0.991259 | | 0.988585 | |
| 0.917948 | 0.957446 | | NA |  |  | 1.0283 | NA | NA |  | 1.01607 | | 0.927917 | |
| 0.965078 | 0.999044 | | NA |  | 1.01141 | | NA | NA |  | 0.94357 | | 0.903317 | |
| 1.12899 | 1.19424 | | 1.28549 | | 1.17526 | | NA | 1.20219 | | 1.13733 | | 1.16648 | |
| 1.04182 | 0.991145 | | 0.860806 | | 0.985474 | | NA | 1.01542 | | 0.977806 | |  | 1.0513 |
| 0.974737 | NA |  | NA |  | 1.02056 | | NA | NA |  | NA |  | 0.996615 | |
| 0.964029 | 1.01533 | | 1.12387 | | 1.01493 | | NA | 0.975489 | | 0.943943 | | 1.01625 | |
| 0.964646 | 0.976564 | | NA |  | 0.988402 | | 0.818182 | 0.926108 | | 0.925396 | | 1.01593 | |
| 1.05494 | 0.957409 | | 1.00056 | | 1.07709 | | NA | 1.05141 | |  | 1.1218 | 1.03276 | |
| 1.06218 | 0.989953 | | 0.882276 | | 0.967257 | | NA | 1.05579 | | 1.01882 | | 1.06309 | |
| 1.00826 | 1.01418 | | 0.933333 | | 0.903427 | | NA | 1.15267 | | 1.32075 | | 0.977778 | |
| 1.11253 |  | 1.0684 | 0.951299 | | 1.04111 | | NA | 1.05415 | | 0.954151 | | 1.02854 | |
| 0.918605 | 0.874046 | | 0.891892 | | 0.891153 | | NA | 1.11085 | | 1.04469 | | 0.899406 | |
| 1.04668 | 1.04543 | | 0.893039 | | 1.02401 | | NA |  | 1.1063 | 1.11427 | | 1.05185 | |
| 1.05682 | 1.04231 | | 0.987805 | | 1.06801 | | NA | 1.10156 | | 0.993865 | | 0.960954 | |
| 0.962209 | 1.04327 | | NA |  | 0.968699 | | NA | 1.09576 | | 0.880342 | | 0.96672 | |
| NA | NA |  | NA |  | NA |  | NA | 0.962695 | | NA |  | NA |  |
| NA | NA |  | NA |  | NA |  | NA | 0.962695 | | NA |  | NA |  |
| NA | NA |  | NA |  | NA |  | NA | 0.962695 | | NA |  | NA |  |

| 0.982634 | | 0.960192 | | 0.889012 | | 1.05035 | | NA | NA |  | NA |  | 0.985343 | |
| --- | --- | --- | --- | --- | --- | --- | --- | --- | --- | --- | --- | --- | --- | --- |
| 0.937376 | | 0.947625 | | 0.975528 | | 1.04451 | | NA | NA |  | NA |  | 1.01705 | |
| 1.13387 | | 1.11906 | | 1.09254 | | 1.11149 | | NA | NA |  | 1.01518 | | 1.12221 | |
| 0.997815 | | NA |  | NA |  | 0.968043 | | NA | NA |  | 0.916622 | | 0.954838 | |
| NA |  | NA |  | NA |  | NA |  | NA | NA |  | NA |  | NA |  |
| NA |  | NA |  | NA |  | NA |  | NA | NA |  | NA |  | NA |  |
| NA |  | NA |  | NA |  | NA |  | NA | NA |  | NA |  | NA |  |
|  | 1.0846 | 1.10918 | | NA |  | 1.09095 | | NA | NA |  | NA |  |  | 1.037 |
| 1.05769 | | 1.05516 | | 1.15062 | | 1.10727 | | NA | 1.02266 | | 1.01769 | | 1.06422 | |
| 1.02536 | | 1.04088 | | 1.14958 | | 1.04533 | | NA | 1.02548 | | 1.10129 | | 1.04891 | |
|  | 1.1464 | 1.01621 | | 0.961125 | | 0.994505 | | NA | 0.940357 | | 0.959478 | | 0.97968 | |
|  | 1.1464 | 1.01621 | | 0.961125 | | 0.994505 | | NA | 0.940357 | | 0.959478 | | 0.97968 | |
|  | 1.1128 | 1.04747 | |  | 1.0766 |  | 1.0675 | NA | 0.986314 | | 0.960813 | | 0.980782 | |
| 0.94976 | | 1.00084 | | NA |  | 0.980315 | | NA | NA |  | 0.996713 | | 0.982431 | |
| 0.94976 | | 1.00084 | | NA |  | 0.980315 | | NA | NA |  | 0.996713 | | 0.982431 | |
| 0.941469 | | NA |  | NA |  | NA |  | NA | 0.980927 | | 0.977755 | | 1.00456 | |
| NA |  | NA |  | NA |  | NA |  | NA | 0.992011 | | 0.915754 | | NA |  |
| NA |  | NA |  | NA |  | NA |  | NA | 1.00946 | | 1.01669 | | NA |  |
|  | 1.0378 | 1.04115 | | 0.998846 | |  | 1.0558 | 0.833333 | 0.965487 | | 0.989969 | | 1.04779 | |
| 1.03669 | | 1.04288 | | 1.03254 | | 1.05998 | | NA |  | 1.0775 |  | 1.0171 | 1.04859 | |
|  | 1.0976 |  | 1.0898 | 0.945238 | | 1.03308 | | NA | NA |  | NA |  | 1.03714 | |
| 1.00608 | | 0.979888 | | 0.929933 | | 0.914266 | | NA | 0.99804 | | 0.953714 | | 1.01507 | |
| 1.01974 | | 0.992371 | | 0.99193 | | 0.901338 | | NA | 1.00593 | | 0.888556 | | 1.04608 | |
|  | 1.0898 |  | 1.0205 | 0.91148 | | 1.00327 | | NA | 1.05331 | | NA |  | 1.03901 | |
| 1.04633 | | 1.04885 | | 0.893518 | | 1.01795 | | NA | 1.04267 | | NA |  | 1.03747 | |
| 1.03075 | | 1.01301 | | 1.01349 | | 0.944276 | | NA | 1.08284 | | 1.05855 | | 1.01631 | |
| 0.988302 | | 1.02292 | | 0.959128 | | 1.05633 | | NA | 0.907459 | | NA |  | 1.00141 | |
|  | 1.0229 | 1.05574 | | 1.05739 | |  | 1.0002 | NA | 0.999413 | | 1.01019 | | 1.01447 | |
| 0.871642 | | 0.918675 | | NA |  | 0.943919 | | NA | 0.970757 | | 1.03205 | | 0.974066 | |
| 1.01859 | | 1.00573 | | 0.982137 | | 0.986932 | | 0.583333 | 1.00282 | | 1.00543 | | 1.07509 | |
|  | 0.8917 | 0.921614 | | 0.992574 | | 0.880804 | | NA | 0.953941 | | 1.12264 | | 0.908277 | |
| 0.892682 | | 0.942785 | | 0.864597 | | 0.975067 | | NA | NA |  | NA |  | NA |  |
| NA |  | NA |  |  | 1.025 | NA |  | NA | 0.991929 | | 1.01441 | | NA |  |
| NA |  | NA |  | NA |  | NA |  | NA | 0.992425 | | NA |  | NA |  |
| NA |  | NA |  | NA |  | NA |  | NA | 1.04586 | | NA |  | NA |  |
| 1.09019 | | 1.07413 | | 1.11529 | | 1.07396 | | 0.722222 | 1.03932 | | 1.06849 | |  | 1.0968 |
| 0.959867 | | 0.992572 | |  | 1.0532 | 1.10095 | | 1.03689 | 1.03248 | | 1.11792 | | 1.09144 | |
| 1.08268 | | 1.02805 | | 0.948881 | |  | 1.0218 | 1.16667 | 1.01151 | | 1.00429 | | 0.986796 | |
| NA |  | NA |  | NA |  | NA |  | 0.772727 | NA |  | 1.21753 | | NA |  |

| 1.01881 | | 1.02178 | | 1.19656 | | 1.06447 | | 1.22727 | | 1.01663 | | 1.09135 | | 1.09377 | |
| --- | --- | --- | --- | --- | --- | --- | --- | --- | --- | --- | --- | --- | --- | --- | --- |
| 0.95318 | | 0.926283 | | 0.919042 | | 0.94331 | |  | 1 | 0.976534 | | 0.978637 | | 0.922905 | |
| 1.17222 | | 1.11496 | | 1.07198 | | 1.03997 | | NA |  | 1.11654 | |  | 1.0074 |  | 1.1597 |
| 1.15205 | | 1.15191 | | 1.03797 | | 1.02734 | | NA |  | 1.10361 | | 1.05607 | | 1.06745 | |
| 1.10684 | |  | 1.1095 | 1.04811 | | 1.02986 | | NA |  | NA |  | NA |  | 1.03547 | |
| 1.09717 | | 0.930999 | | 0.928571 | | 1.11429 | | NA |  | 1.00686 | | 1.04188 | | 1.00886 | |
| 0.971846 | | 0.955195 | | NA |  | 0.959862 | | NA |  | 1.16731 | | 1.24784 | | 0.996678 | |
| 1.09173 | | 1.11358 | | 1.01057 | | 1.01859 | | NA |  | 1.07137 | | 0.998013 | | 1.05496 | |
| 1.18617 | | 1.13003 | |  | 1.1039 | 1.03585 | | NA |  | NA |  | NA |  | 1.09275 | |
| NA |  | NA |  | NA |  | NA |  | NA |  | 1.04976 | |  | 1.1182 | NA |  |
| NA |  | NA |  | NA |  | NA |  | NA |  | 1.04149 | |  | 1.0984 | NA |  |
| NA |  | NA |  | NA |  | NA |  | NA |  | 1.02448 | | 1.12134 | | NA |  |
| 1.15599 | | 1.18518 | | 1.28243 | | 1.19677 | | 1.68013 | | 1.14504 | |  | 1.1271 | 1.18281 | |
| 1.12165 | | 1.12023 | | 1.21968 | | 1.12495 | | 0.846154 | | 1.08041 | |  | 1.0621 | 1.11769 | |
| 0.961737 | | 0.955962 | | NA |  | 0.927593 | | NA |  | NA |  | NA |  | 0.934943 | |
| 1.07129 | |  | 1.074 | 1.09368 | | 1.17387 | | 0.794872 | |  | 1.1157 | 1.04318 | | 1.04073 | |
| 0.93322 | | 0.945349 | | NA |  | 0.897852 | | NA |  | NA |  | NA |  | NA |  |
| 0.952573 | | 1.10562 | | 0.92003 | | 1.06297 | | 0.787879 | | NA |  | 1.03158 | | 1.05619 | |
| 1.01792 | | 1.02458 | | 1.03359 | | 1.01577 | | 0.985539 | | 1.00387 | |  | 1.0192 | 1.00055 | |
| NA |  | NA |  | NA |  | NA |  | NA |  | 1.03609 | | NA |  | NA |  |
| 1.15404 | | 1.07771 | | 1.04372 | | 1.19475 | | 1.23529 | | 1.19666 | | 1.16603 | | 1.11426 | |
| 1.02106 | | 0.991275 | | NA |  | NA |  | NA |  | 1.02433 | | 0.903564 | | 1.02889 | |
| NA |  | NA |  | 1.05173 | | NA |  | 0.666667 | | NA |  | 1.07729 | | NA |  |
| NA |  | NA |  | NA |  | NA |  | 0.490196 | | 1.00951 | | 1.04608 | | NA |  |
| 0.992839 | | 0.950705 | | 0.941907 | | 1.00137 | | 0.705882 | | 0.992569 | | 0.992588 | | 1.07807 | |
| 1.00046 | | 0.98228 | | 0.965334 | | 1.04633 | |  | 1.4 | 0.976531 | | 1.07946 | | 1.02961 | |
| 1.00634 | | 0.943557 | | 0.987315 | | 0.983425 | | 1.07692 | | 0.985672 | | 0.939887 | | 1.03641 | |
|  | 1.0109 |  | 1.0026 | 1.03713 | | 1.04665 | | 0.937457 | | 0.982652 | | 0.936531 | | 1.01637 | |
| 0.964848 | | 0.982893 | | 1.01961 | | 1.05778 | | NA |  |  | 1 | 1.02342 | | 1.01297 | |
| 1.13984 | | 1.13106 | | 1.03975 | | 1.14408 | | NA |  | 1.05096 | | 1.08872 | | 1.08834 | |
| 1.07807 | | 1.00379 | | 1.09572 | | 1.09617 | | 1.07692 | | 1.04857 | | 0.996179 | | 1.00479 | |
| 1.08395 | | 1.16028 | | 1.31963 | | 1.10139 | |  | 1.9 | 1.10337 | |  | 1.1452 | 1.06347 | |
| 1.06102 | | 1.06009 | | 0.983323 | | 0.993877 | | NA |  | 1.11093 | | 1.15851 | |  | 1.021 |
| 1.01172 | | 0.989982 | | NA |  | 0.978634 | | NA |  | 1.06051 | | 1.04479 | | 0.995383 | |
| 1.16181 | | 1.15961 | | 1.23234 | | 1.13636 | | 0.850744 | | 1.04002 | | 1.02923 | | 1.18186 | |
| 0.987903 | | 1.00848 | | 0.968127 | |  | 1.0262 | NA |  | 0.992224 | | 0.976886 | | 1.02978 | |
| 0.981995 | | 1.01337 | | 1.03791 | | 1.03968 | | NA |  | 0.992182 | | 0.937848 | | 1.01643 | |
| 0.959871 | | 1.05363 | | 0.910995 | |  | 1.0232 |  | 0.5 | 1.04547 | | 1.09589 | | 1.03673 | |
| 1.09663 | | 1.06114 | | 1.14817 | | 1.01515 | | 0.753421 | | 1.09845 | |  | 1.0423 | 1.11288 | |

| 1.05201 | | 1.08229 | | 1.10448 | | 1.09554 | |  | 0.75 |  | 1.0625 | 1.05144 | | 1.04334 | |
| --- | --- | --- | --- | --- | --- | --- | --- | --- | --- | --- | --- | --- | --- | --- | --- |
| 1.08776 | | 1.06899 | | 1.11395 | | 1.04542 | |  | 1.6 | 1.01312 | | 1.00625 | |  | 1.0195 |
| 1.08351 | | 1.10854 | | 1.15466 | | 1.16661 | | NA |  | 1.13094 | | 1.08264 | | 1.13356 | |
| 0.942007 | | 0.945061 | | 0.919118 | | 0.988554 | | NA |  | 1.00709 | | 1.02121 | | 1.04055 | |
| 1.21994 | | 1.21006 | | 1.30294 | |  | 1.1921 | NA |  | 1.17539 | | 1.10458 | | 1.22983 | |
| NA |  | NA |  | NA |  | NA |  | NA |  | 1.00463 | | NA |  | NA |  |
| 1.00715 | | 0.983778 | | 0.993588 | | 0.986098 | | NA |  | 1.00039 | | 0.959837 | | 1.00522 | |
| 1.00855 | | 1.01032 | | NA |  | 0.97818 | | NA |  | 1.00467 | | 1.05888 | | 0.987636 | |
| 1.03603 | | 1.06846 | | 0.933333 | | 1.16109 | | 0.785714 | | 0.975326 | | 0.926222 | | 1.09874 | |
| 1.02245 | | 0.972862 | | 0.85446 | | 0.983662 | | NA |  | 0.994166 | | 1.04213 | | 1.05118 | |
| 0.959741 | | 1.01054 | | 0.920223 | | 0.986756 | | 0.928571 | | 1.08901 | |  | 1.1928 | 0.988824 | |
| NA |  | NA |  | NA |  | NA |  | NA |  | 0.998197 | | NA |  | NA |  |
| 1.01102 | | 0.966033 | | 1.03817 | | 0.953862 | | NA |  | 0.992612 | | 0.97314 | | 0.987685 | |
| 1.09478 | | 1.12368 | | 1.08708 | |  | 1.0744 |  | 4.8913 | 1.06752 | | 1.06088 | | 1.03473 | |
| 1.06804 | | 1.16319 | | 1.13658 | | 1.06574 | |  | 1.375 | 1.03904 | | 1.03967 | | 1.03869 | |
|  | 1.0726 | 1.05731 | | 1.12747 | |  | 1.0579 | 1.05401 | | 1.01587 | | 1.06648 | | 1.07468 | |
| 1.13095 | | 1.08275 | | 1.04168 | |  | 1.0213 | 2.13684 | | 1.08625 | | 1.06465 | | 1.10213 | |
| 1.07312 | | 1.02992 | | 1.04388 | | 1.02963 | | 0.323529 | | 1.03426 | | 0.990532 | | 1.07131 | |
| NA |  | NA |  | NA |  | NA |  | NA |  | 0.946448 | | NA |  | NA |  |
| 1.05943 | | 1.09526 | | 1.05929 | |  | 0.9887 | NA |  |  | 0.9915 | 0.978571 | | 1.07025 | |
| 0.981173 | |  | 1.0102 | 1.07529 | | 1.01764 | | NA |  | 0.991963 | | 1.09289 | | 1.05632 | |
| 1.06507 | | 0.866709 | | NA |  | 0.943266 | | NA |  | NA |  | NA |  | 0.965614 | |
| 1.01118 | | 0.982314 | | 0.91954 | | 0.99118 | |  | 0.4 | 1.07549 | | 0.956894 | | 0.988693 | |
| 0.913263 | | 0.969364 | | 0.939528 | | 0.985399 | | NA |  | 0.997547 | | 0.834638 | | 0.999218 | |
| 1.00084 | | 1.02119 | | NA |  | 1.01866 | | NA |  | 1.01798 | | 1.05094 | | 1.03204 | |
|  | 1.1038 | 1.12462 | | 1.06382 | | 1.06023 | | NA |  | 1.13038 | | 1.05767 | | 1.05918 | |
| 0.958717 | | 0.981698 | | 1.17481 | | 0.985179 | | NA |  | 1.18528 | | 1.20437 | | 1.01612 | |
| 0.940333 | | 1.05291 | | 0.964608 | | 0.990481 | | 1.16944 | | 0.96342 | | 1.16872 | | 1.00603 | |
| 1.04321 | | 0.920175 | |  | 1.0706 | 0.920628 | | NA |  | 1.01237 | | 0.971751 | | 1.05295 | |
| 0.952677 | | 1.01069 | | NA |  | 0.943697 | | NA |  | 1.00809 | | 1.06858 | | 0.95804 | |
| 1.01298 | | 1.01052 | | 0.98507 | | 1.00359 | | NA |  | 0.98347 | | 0.975786 | |  | 1.0479 |
| 1.13904 | | 1.19899 | | 1.32862 | | 1.19709 | | 0.995656 | | 1.22391 | | 1.18601 | | 1.17886 | |
| 1.01615 | | 0.976517 | | 1.01679 | | 1.03257 | |  | 1.375 |  | 1.0467 | 1.01087 | | 0.953982 | |
| 1.04418 | | 1.02945 | | 1.06528 | | 1.05119 | | NA |  | NA |  | NA |  | 1.01595 | |
| 1.05995 | |  | 1.0838 | NA |  | 1.09051 | | NA |  | 1.01135 | | 1.01915 | | 1.10699 | |
| 1.06797 | | 1.08668 | |  | 1.1262 | 1.04055 | | NA |  | 1.01653 | | 1.01363 | | 1.10142 | |
| 1.02383 | | 1.04528 | | 1.04726 | | 1.07357 | | NA |  | 1.07733 | | 0.970506 | | 1.00511 | |
|  | 1.059 | 1.05478 | | 0.96798 | | 1.04781 | |  | 2 | 1.04819 | | 0.932121 | | 1.03017 | |

neutrophil neutrophil normal_hu osteoclast plasma_ce podocyte.. preadipoc precursor_

| 1.0946 | 1.07981 | 1.12004 | 1.05631 | 1.13588 | 1.08483 | 1.11217 | 1.09094 |
| --- | --- | --- | --- | --- | --- | --- | --- |

NA	NA	NA	NA	NA	NA	NA	NA

1.15483	1.14935	1.22557	1.15648	1.23089	1.11784	1.19074	1.1793

1.09522	1.14338	1.24134	1.10575	1.21749	1.01556	1.10363	1.18213

1.11104	1.12998	1.19932	1.16092	1.36812	1.12101	1.17684	1.17255

0.98256 0.980616	0.97551 0.938472 0.959932 0.926595 0.917725 0.923546

1.10974	1.1272	1.18583	1.0973	1.2752	1.1157	1.18123	1.14295

1.06704	1.08364	1.14276	1.08727	1.21516	1.2299	1.1183	1.16189

1.06704	1.08364	1.14276	1.08727	1.21516	1.2299	1.1183	1.16189

1.12522	1.16402	1.1197	1.19122	1.08196	1.12147	1.1452	1.14199

0.957124	0.96624 0.945441 0.981573 0.966235	1.02403 0.973572	0.9763

1.04364	1.06796 0.973118	1.02399 0.956762	0.95216 0.986802 0.967884

1.08288	1.07466	1.01687	1.03992	0.98088	1.02145	1.04655	1.01483

1.08985	1.06287	1.0093	1.06354	1.01035	1.05315	1.03959	1.01397

NA	NA	1.03819 NA	NA	NA	NA	NA NA	NA	NA	NA	NA	1.05051 NA	NA

NA	1.03734 0.988648 0.965425	1.01629	1.01727 0.984564 0.983119

NA	1.03734 0.988648 0.965425	1.01629	1.01727 0.984564 0.983119

1.00587 0.996537 0.904505	1.02148 0.960762 0.950507 0.956134	1.02972

1.14868	1.13844	1.257	1.16831	1.34641	1.25546	1.12237	1.25346

NA	NA	NA	NA	NA	1.04684 NA	NA

1.00807 0.993804 0.943329	1.03414	1.00376 0.977829	1.01323	1.01659

NA	NA	1.01311 NA	NA	NA	NA	NA NA	NA	0.996542 NA	NA	0.970927 NA	NA

1.04596	1.03231	1.03317	1.05829 NA	NA	1.02648 NA

1.06887	1.05895 NA	1.08803 NA	NA	1.07828 NA

1.07249	1.08291 NA	1.08987 NA	NA	1.05182 NA

1.09007	1.07948 NA	1.0837 NA	NA	NA	NA

1.08573	1.07778	1.19964	1.1028	1.26656	1.17365	1.08446	1.20443

1.08573	1.07778	1.19964	1.1028	1.26656	1.17365	1.08446	1.20443

1.10341	1.1634	1.2722	1.14388	1.43479	1.15419	1.15461	1.13233

1.17773	1.22475	1.32623	1.21155	1.48141	1.17709	1.1475	1.11896

1.09044	1.11285 0.999319	1.13089	1.10447	1.09397	1.15064	1.09806

1.00459	1.01182 NA	1.00848 NA	NA	1.02234 NA NA	NA	NA	NA	NA	NA	NA	NA NA	NA	NA	NA	NA	NA	NA	NA NA	NA	NA	NA	NA	NA	NA	NA

1.0702	1.08677	1.04266	1.04246	1.02352	1.00746	1.02627	1.04228

| 0.940899 | | 0.948154 | | 0.93697 | | 0.916749 | | 0.949995 | | 1.02183 | 1.05778 | | 0.93895 | |
| --- | --- | --- | --- | --- | --- | --- | --- | --- | --- | --- | --- | --- | --- | --- |
| 0.940899 | | 0.948154 | | 0.93697 | | 0.916749 | | 0.949995 | | 1.02183 | 1.05778 | | 0.93895 | |
| 0.940899 | | 0.948154 | | 0.93697 | | 0.916749 | | 0.949995 | | 1.02183 | 1.05778 | | 0.93895 | |
| 1.01951 | | 1.03181 | | 1.04656 | | 1.01565 | | 1.07112 | | 1.02774 | 1.05239 | | 1.05936 | |
|  | 1.0005 | 0.992256 | | 1.08222 | | 1.02212 | | 1.07426 | | 1.03418 | 1.03675 | |  | 1.0819 |
| 0.968089 | | 0.959421 | | 1.00351 | | 0.997744 | | 0.997048 | | 0.970858 | 0.960867 | | 1.04835 | |
| 1.04195 | | 1.03254 | | 1.10921 | | 1.07544 | | 1.01947 | | 1.07216 | 1.00997 | | 1.08621 | |
| 1.04545 | | 1.02818 | | 1.05367 | | 1.03616 | | 1.01519 | | 1.07082 |  | 1.032 |  | 1.0653 |
| 0.980227 | | 0.984552 | | 1.02141 | | 1.01902 | | 0.986845 | | 0.98238 | 0.954442 | | 1.04487 | |
| 1.03887 | | 1.04073 | | 1.08283 | | 1.06036 | | 1.02766 | | 1.09538 | 1.07778 | | 1.08482 | |
| 1.09408 | | 1.07447 | |  | 1.1383 | 1.07041 | | 1.04094 | | 1.12692 | 1.07052 | | 1.09151 | |
| 1.04201 | | 1.04092 | | 1.04254 | | 1.07658 | |  | 1.0057 | 1.03744 | 1.02842 | |  | 1.0903 |
| 1.10429 | | 1.09504 | | 1.12638 | |  | 1.1137 |  | 1.0089 | 1.11729 | 1.05957 | | 1.12057 | |
| 1.05856 | | 1.05645 | | 1.07819 | | 1.04936 | | 0.997175 | | 1.00869 | 1.02491 | | 1.05823 | |
|  | 1.0547 | 1.05155 | | 1.05607 | | 1.07614 | | 0.984594 | | 0.98755 | 1.02446 | | 1.04114 | |
| NA |  | NA |  | NA |  | NA |  | NA |  | NA | NA |  | NA |  |
| 1.07221 | | 1.06002 | | 1.14706 | | 1.09714 | | 1.27271 | | 1.22723 | 1.12433 | | 1.18402 | |
| 1.04435 | | 1.05671 | | 1.08336 | | 1.08756 | | 1.06989 | | 1.09924 | 1.07012 | | 1.10895 | |
| 1.04505 | | 1.04916 | |  | 1.0127 | 1.09536 | | 1.05549 | | 1.03627 | 1.04727 | | 1.10471 | |
| 1.08843 | | 1.06976 | | 0.921673 | | 1.17528 | | 1.08211 | | 1.10867 | 1.12987 | | 1.13419 | |
| 1.00308 | | 0.99977 | | 0.95862 | | 1.03228 | | 0.99594 | | 0.975679 | 0.975678 | | 1.07139 | |
| NA |  | NA |  | 1.03097 | | NA |  | 1.07828 | | 1.02629 | NA |  | NA |  |
| NA |  | NA |  | NA |  | NA |  | NA |  | NA | NA |  | NA |  |
| 1.03467 | | 1.03509 | | 0.977422 | | 1.05491 | | 1.03655 | | 1.01903 | 1.02106 | | 1.04744 | |
| 1.04082 | | 1.06297 | | 0.987633 | |  | 1.04 | 1.17167 | | 1.08418 |  | 1.05 | 1.10088 | |
| 1.05198 | | 1.06149 | | 1.05022 | | 1.08474 | | 1.01513 | | 1.03698 | 0.980008 | | 1.09899 | |
| 1.07425 | | 1.07003 | | 1.01996 | |  | 1.0693 | 1.05596 | | 1.03613 | 1.03355 | | 1.09087 | |
| 1.03605 | |  | 1.0373 | 1.09037 | | 1.05892 | | 1.01208 | | 1.03177 | 1.04768 | | 1.05522 | |
| 1.04963 | | 1.04959 | |  | 1.0951 | 1.05734 | | 1.00468 | | 1.07214 |  | 1.0217 | 1.04913 | |
| 1.04963 | | 1.04959 | |  | 1.0951 | 1.05734 | | 1.00468 | | 1.07214 |  | 1.0217 | 1.04913 | |
| 1.04071 | |  | 1.043 | 1.03439 | | 1.07476 | | 1.03539 | | 1.04806 | 1.05008 | | 1.09058 | |
| 1.08019 | | 1.08491 | | 1.04192 | | 1.11757 | | 1.02547 | | 1.08295 | 1.05916 | | 1.05269 | |
| 1.04401 | | 1.05526 | | 1.01701 | | 1.07817 | | 0.994281 | | 1.06038 | 1.02917 | | 1.11261 | |
|  | 1.0035 | 0.993592 | | 0.994242 | | 1.02879 | | 1.01208 | | 1.07012 | 0.916631 | | 1.03737 | |
| NA |  | NA |  | NA |  | NA |  | NA |  | 1.00821 | NA |  | NA |  |
| 1.00898 | | 1.02526 | | 1.02489 | | 1.02644 | | 1.04753 | | 1.04211 | 1.01521 | | 1.00163 | |
| 1.04964 | | 1.06456 | | 1.01602 | | 1.07551 | | 1.02304 | | 1.01828 | 1.06537 | | 1.07563 | |
| 1.01448 | |  | 1.0218 | 1.03006 | | 1.05312 | | 1.03599 | | 1.02478 | 1.03552 | | 1.08657 | |
| 1.11938 | |  | 1.0944 | 1.01169 | | 1.14582 | | 1.13154 | | 1.14652 | 1.19468 | | 1.16508 | |

| 1.02188 | | 1.00416 | | 1.04005 | | 1.02481 | 1.01207 | | 1.07452 | | 1.05546 | | 0.994869 |
| --- | --- | --- | --- | --- | --- | --- | --- | --- | --- | --- | --- | --- | --- |
| 0.971365 | | 0.974901 | | 0.960853 | | 1.02019 | 0.998073 | | 1.02482 | | 1.07891 | | 0.975985 |
| 1.03396 | | 1.00779 | | 1.03716 | | 1.03795 | 1.00865 | | 1.04865 | | 1.02443 | | 0.994604 |
|  | 1.0299 | 0.992951 | | 1.01954 | | 1.03823 |  | 1.0188 | 1.05678 | | 1.02327 | | NA |
| NA |  | NA |  | 1.02109 | | NA | NA |  | 0.967517 | | NA |  | NA |
| NA |  | NA |  | 1.02828 | | NA | NA |  | 0.969594 | | NA |  | NA |
| NA |  | NA |  | 0.98633 | | NA | NA |  | 0.975838 | | NA |  | NA |
| NA |  | NA |  | 1.05684 | | NA | NA |  | 0.984607 | | NA |  | NA |
| NA |  | NA |  | 1.05447 | | NA | NA |  | 0.940061 | | 1.05449 | | NA |
| NA |  | NA |  | 1.02928 | | NA | NA |  | 0.909617 | | 1.06212 | | NA |
| NA |  | NA |  | 1.01283 | | NA | NA |  | 0.954434 | | NA |  | NA |
| NA |  | NA |  | 1.01283 | | NA | NA |  | 0.954434 | | NA |  | NA |
| NA |  | NA |  | 1.01283 | | NA | NA |  | 0.954434 | | NA |  | NA |
| NA |  | NA |  | 1.04027 | | NA | NA |  | 0.946462 | | NA |  | NA |
| NA |  | NA |  | 1.04895 | | NA | NA |  | 0.916723 | | 1.07395 | | NA |
| NA |  | NA |  |  | 1.0258 | 1.02205 | NA |  | 0.942585 | | 1.06906 | | NA |
| NA |  | NA |  | 1.06634 | | NA | NA |  | 0.967435 | | 0.998667 | | NA |
| NA |  | NA |  | 1.01826 | | NA | NA |  | 0.989558 | |  | 1.0021 | NA |
| 1.10011 | | 1.10667 | |  | 1.03 | 1.04756 | NA |  | 0.966983 | | 1.06628 | | 1.05304 |
| NA |  | NA |  | 1.04752 | | 1.01835 | NA |  | 0.946047 | | 1.05688 | | NA |
| NA |  | NA |  | 1.02593 | | NA | NA |  | 0.961215 | | NA |  | NA |
| 1.05673 | | 1.06589 | |  | 1.0536 | 1.09369 | 1.02323 | | 1.09763 | | 1.05539 | | 1.09278 |
| 0.93924 | | 0.92137 | | 1.02961 | | 0.952255 | NA |  | NA |  | 0.984093 | | 0.952006 |
| 0.93924 | | 0.92137 | | 1.02961 | | 0.952255 | NA |  | NA |  | 0.984093 | | 0.952006 |
| NA |  | NA |  | 1.02153 | | NA | NA |  | NA |  | NA |  | NA |
| NA |  | NA |  | 1.02153 | | NA | NA |  | NA |  | NA |  | NA |
| NA |  | NA |  | 1.02814 | | 0.995903 | NA |  | NA |  | 0.975062 | | NA |
| 1.03306 | |  | 1.0437 | 1.05625 | | 0.992912 | NA |  | NA |  | 1.00284 | | 0.95489 |
| NA |  | NA |  | 0.94287 | | NA | NA |  | NA |  | NA |  | NA |
| NA |  | NA |  | 1.03351 | | NA | NA |  | NA |  | NA |  | NA |
| NA |  | NA |  | 1.03351 | | NA | NA |  | NA |  | NA |  | NA |
| NA |  | NA |  | 1.03351 | | NA | NA |  | NA |  | NA |  | NA |
| NA |  | NA |  | NA |  | NA | NA |  | NA |  | NA |  | NA |
| NA |  | NA |  | NA |  | NA | NA |  | NA |  | NA |  | NA |
| NA |  | NA |  | NA |  | NA | NA |  | 1.00989 | | NA |  | NA |
| NA |  | NA |  | NA |  | NA | NA |  | 1.00921 | | NA |  | NA |
| 0.923767 | | 0.929896 | | 0.977127 | | 1.02656 | 1.00968 | | 0.971661 | | 0.991847 | | 1.00243 |
| 1.11289 | | 1.11332 | | 1.11388 | | 1.10229 | 1.18645 | |  | 1.0922 | 1.03946 | | 1.11552 |
| 1.11289 | | 1.11332 | | 1.11388 | | 1.10229 | 1.18645 | |  | 1.0922 | 1.03946 | | 1.11552 |

| 1.10925 | 1.12057 | 1.27388 | | 1.12523 | 1.30653 | | 1.16931 | | 1.21077 | 1.20769 |
| --- | --- | --- | --- | --- | --- | --- | --- | --- | --- | --- |
| NA | NA | 1.27192 | | NA | 1.28714 | | 1.18349 | | NA | NA |
| 1.07382 | 1.07277 | 1.09733 | | 1.02395 | 1.10723 | | 1.07439 | | 1.00275 | 1.10842 |
| 0.987253 | 0.951522 | 1.06225 | | NA |  | 1.0508 | 1.02038 | | NA | 0.985533 |
| 1.06061 | 1.04878 | 1.07251 | | 1.07747 | 1.04104 | | 1.06582 | | 1.08809 | 0.985152 |
| 1.06473 | 1.06466 | NA |  | 1.08309 | NA |  | NA |  | 1.04055 | NA |
| NA | NA | 1.01972 | | NA | NA |  | 0.966629 | | NA | NA |
| NA | NA | 1.05586 | | NA | NA |  | 1.04389 | | NA | NA |
| 0.940459 | 0.949254 | 1.02591 | | 0.902832 | 1.00063 | | 0.954886 | | 0.926689 | NA |
| 1.05105 | 1.02898 |  | 1.0791 | 1.00175 | 1.04638 | | 1.04721 | | 0.980267 | 1.12765 |
| 0.954897 | 0.95532 | 1.00137 | | 0.886749 | 1.00854 | | 0.97354 | | 0.931637 | 0.96209 |
| NA | NA | NA |  | NA | NA |  | 1.01305 | | NA | NA |
| NA | NA | 1.03246 | | NA | NA |  | 1.00744 | | NA | NA |
| NA | NA | 1.01262 | | NA | NA |  | NA |  | NA | NA |
| NA | NA | 0.996691 | | NA | NA |  | NA |  | NA | NA |
| NA | NA | 1.04651 | | NA | NA |  | NA |  | NA | NA |
| NA | NA | NA |  | NA | NA |  | 1.03155 | | NA | NA |
| NA | NA | NA |  | NA | NA |  | 1.07714 | | 1.04978 | NA |
| NA | NA | NA |  | NA | NA |  | 1.01458 | | NA | NA |
| NA | NA | 0.961477 | | 1.05015 | NA |  | 0.960053 | | 0.992466 | NA |
| NA | NA | 1.00714 | | NA | NA |  | 0.928173 | | NA | NA |
| NA | NA | 1.01736 | | NA | NA |  | 1.01038 | | 0.97187 | NA |
| NA | NA | NA |  | NA | NA |  | 1.07338 | | NA | NA |
| NA | NA | NA |  | NA | NA |  | 1.00559 | | 0.928977 | NA |
| NA | NA | 1.05175 | | NA | NA |  | 0.975429 | | NA | NA |
| NA | NA | 1.00513 | | 0.979524 | NA |  | 1.01037 | | 1.02123 | NA |
| NA | NA | 1.01424 | | NA | NA |  |  | 1.029 | NA | NA |
| NA | NA | 1.04256 | | NA | NA |  | 1.06964 | | NA | NA |
| NA | NA | NA |  | NA | NA |  | 1.02705 | | NA | NA |
| NA | NA | NA |  | NA | NA |  | 1.02855 | | NA | NA |
| NA | NA | 0.996529 | | 1.01533 | NA |  |  | 1.0728 | 1.06987 | NA |
| NA | NA | NA |  | NA | NA |  | 1.01164 | | NA | NA |
| NA | NA | NA |  | NA | NA |  | 0.970762 | | 1.00939 | NA |
| NA | NA | NA |  | NA | NA |  | 0.986261 | | NA | NA |
| NA | NA |  | 1.0277 | NA | NA |  | 0.992644 | | NA | NA |
| NA | NA | 1.08394 | | NA | NA |  | 1.01497 | | 1.06515 | NA |
| NA | NA | 1.03295 | | NA | NA |  | 0.981479 | | NA | NA |
| NA | NA | 1.07393 | | NA | NA |  | 1.05687 | | NA | NA |
| NA | NA | 1.03737 | | NA | NA |  | 0.998017 | | NA | NA |

| NA |  | NA |  | NA |  | NA |  | NA | 0.99944 | | 0.993742 | | NA |  |
| --- | --- | --- | --- | --- | --- | --- | --- | --- | --- | --- | --- | --- | --- | --- |
| NA |  | NA |  | 0.962503 | | 1.01037 | | NA | 0.935341 | |  | 1.0253 | NA |  |
| NA |  | NA |  | NA |  | NA |  | NA | 1.01329 | | NA |  | NA |  |
| NA |  | NA |  | 1.04165 | | NA |  | NA | NA |  | NA |  | NA |  |
| NA |  | NA |  | 0.991051 | | NA |  | NA | NA |  | NA |  | NA |  |
| NA |  | NA |  | 1.08879 | | 1.10672 | | NA |  | 1.0839 | 1.07309 | | NA |  |
| NA |  | NA |  | 1.02359 | | NA |  | NA | 1.00843 | | NA |  | NA |  |
| NA |  | NA |  | 1.01758 | | NA |  | NA | 1.03372 | | NA |  | NA |  |
| NA |  | NA |  | NA |  | NA |  | NA | 1.01892 | | NA |  | NA |  |
| NA |  | NA |  | 1.03353 | | NA |  | NA | 1.00301 | | 1.07558 | | NA |  |
| 0.952438 | | 0.980301 | | 1.00426 | | 0.996991 | | 0.982873 | 0.989411 | | 0.988663 | | 1.03609 | |
| 1.03069 | | 1.03367 | | 1.28039 | | 0.999573 | | 1.22922 |  | 1.2605 | 0.955455 | | 0.993471 | |
| 0.90285 | | NA |  | NA |  | 0.983202 | | NA | NA |  | NA |  | 0.995811 | |
| 0.957116 | | 0.952565 | | 0.988734 | | 0.995074 | | 1.08013 | 1.01643 | |  | 1.0318 | 1.00784 | |
|  | 1.0239 | 1.13225 | |  | 1.1625 | 1.13902 | | 1.01603 | 1.16549 | | 1.20237 | |  | 1.0938 |
| 1.02098 | | 1.03775 | | 1.05182 | | 1.06502 | | 1.13229 | 1.02877 | | 1.07736 | | 1.05404 | |
| 1.09078 | | 1.07987 | | NA |  | 1.14646 | | 1.22689 | 1.14994 | | NA |  | 1.17335 | |
| 0.932373 | | 0.946249 | | NA |  | 1.02438 | | NA | NA |  |  | 1.0286 | 1.02593 | |
| 1.02003 | | 1.02189 | | 1.04633 | | 1.04626 | | 1.06464 | 0.943902 | | 0.994406 | | 1.02603 | |
|  | 1.0787 | 1.13257 | |  | 1.0622 | 1.14784 | | 1.05336 | 1.08752 | | 1.02724 | | 1.12515 | |
| 1.05779 | | 1.03087 | | 1.03338 | |  | 1.0245 | 0.987951 | 1.00841 | | 1.06215 | |  | 1.0455 |
| 0.994671 | | 0.993986 | | 1.02482 | | 1.01559 | | 1.00438 | 1.05775 | | 1.05019 | | 0.97781 | |
| 1.03799 | | 1.03254 | | 0.987582 | | 1.04934 | | 1.01586 | 1.05843 | | 1.03315 | | 1.01491 | |
| 1.01427 | |  | 1 | 1.05791 | | 0.987913 | | 1.00757 | 1.08383 | | 1.02137 | | NA |  |
| NA |  | NA |  | 1.02471 | | 1.05032 | | 0.989746 | 0.997711 | | 0.996023 | | 1.04775 | |
|  | 1.0939 | 1.08972 | | 1.01551 | | 1.03612 | | 1.05208 | 1.05477 | | 1.01334 | | 1.08342 | |
| 1.02198 | | 1.03045 | | 1.06977 | | 1.03517 | | 1.14424 | 0.957487 | | 1.08449 | | 1.07522 | |
| 1.02926 | | 1.00943 | |  | 1.1727 | 0.984018 | | 1.10935 | 0.962545 | | 1.15778 | |  | 1.0154 |
| 1.02926 | | 1.00943 | |  | 1.1727 | 0.984018 | | 1.10935 | 0.962545 | | 1.15778 | |  | 1.0154 |
| NA |  | NA |  | 1.08486 | | 1.02521 | | 1.09962 | 0.983936 | | 1.05313 | | 1.04426 | |
| 0.988553 | | 0.984955 | | 1.04198 | | 1.01287 | | 1.06785 | 0.970649 | | 1.03693 | | 1.04091 | |
| NA |  | NA |  | 1.01509 | | 1.07211 | | 1.05061 |  | 0.9359 | 0.983512 | | 1.03171 | |
| NA |  | NA |  | 1.01509 | | 1.07211 | | 1.05061 |  | 0.9359 | 0.983512 | | 1.03171 | |
| NA |  | NA |  | 1.01509 | | 1.07211 | | 1.05061 |  | 0.9359 | 0.983512 | | 1.03171 | |
| 0.956552 | | 0.973338 | | 1.01034 | | 1.00696 | | 1.00333 | 1.00995 | | 1.03922 | | 0.989272 | |
| 0.993687 | | 0.960879 | | 1.05667 | | 0.982298 | | 0.987134 | 1.02062 | | 1.04405 | | 0.994048 | |
| NA |  | NA |  | 1.05603 | | NA |  | 1.10536 | 0.968713 | | 0.997124 | | NA |  |
| NA |  | NA |  | NA |  | NA |  | NA | 1.03561 | | NA |  | NA |  |
| NA |  | NA |  | 0.959273 | | 1.10108 | | NA | 0.968495 | | NA |  | 1.08935 | |

| NA |  | NA |  | 1.0162 | NA |  | NA |  | 1.09069 | NA |  | NA |  |
| --- | --- | --- | --- | --- | --- | --- | --- | --- | --- | --- | --- | --- | --- |
| NA |  | NA | NA |  | NA |  | NA |  | 1.00198 | NA |  | NA |  |
| NA |  | NA | NA |  | NA |  | NA |  | 1.01058 | NA |  | NA |  |
| NA |  | NA | NA |  | NA |  | NA |  | 1.03458 | NA |  | NA |  |
| 0.962147 | | 0.99579 | 0.922921 | | 0.93708 | | 0.950058 | | 0.969767 | 0.988694 | | 0.954735 | |
| 1.04077 | | 1.02777 | 1.04029 | | 0.996461 | | 1.03414 | | 0.999997 | 1.03717 | | 0.994326 | |
| 1.01514 | | 0.992464 | 0.96484 | | 0.998331 | | 1.03281 | | 1.00661 | 0.962193 | | 1.00288 | |
|  | 1.0353 | 1.03814 | 1.01082 | | 1.01858 | | 1.02618 | | 1.04943 | 1.03563 | | 1.02732 | |
| 0.968641 | | 0.948367 | NA |  | 0.955448 | | 0.960405 | | 1.05825 | 1.02703 | | 0.943552 | |
| 1.01157 | | 0.997436 | 1.07131 | | 0.998695 | | 1.00944 | | 1.06161 | 1.02578 | |  | 1.0014 |
| 1.08253 | | 1.07363 | 1.18238 | | 1.08431 | | 1.26243 | | 1.13103 | 1.07222 | | 1.13214 | |
| 1.17672 | | 1.14855 |  | 1.2886 |  | 1.1246 |  | 1.0309 | 1.17507 | 1.20004 | | 1.05007 | |
| 1.14038 | | 1.17877 |  | 1.3228 | 1.14836 | | 1.19369 | | 1.34306 | 1.28484 | | 1.15807 | |
| 0.978953 | | 1.00183 | 0.996226 | | 1.00325 | | 0.995408 | | 0.967396 | 0.972059 | | 0.98124 | |
| NA |  | NA | 1.03638 | | NA |  | NA |  | 1.03812 |  | 1.0066 | NA |  |
| NA |  | NA | NA |  | NA |  | NA |  | 1.02287 | NA |  | NA |  |
| 1.14663 | | 1.11884 | 1.20982 | | 1.12587 | |  | 1.3124 | 1.15591 | 1.08189 | | 1.12748 | |
| NA |  | NA | NA |  | NA |  | NA |  | 1.02265 | NA |  | NA |  |
| 0.97269 | | 0.987844 |  | 1.0269 | 0.943513 | | 0.942473 | | 1.00223 | 0.942847 | | 0.913548 | |
| NA |  | NA | 1.02104 | | NA |  | NA |  | 1.0587 | NA |  | NA |  |
| NA |  | NA | 1.02104 | | NA |  | NA |  | 1.0587 | NA |  | NA |  |
| 1.05746 | | 1.08104 | 1.12332 | | 1.07554 | | 1.14976 | | 1.08852 |  | 1.1267 | 1.13396 | |
| 1.08772 | | 1.08297 | 1.18969 | | 1.05228 | | 1.29684 | | 1.13382 | 1.07394 | | 1.14798 | |
| 1.08772 | | 1.08297 | 1.18969 | | 1.05228 | | 1.29684 | | 1.13382 | 1.07394 | | 1.14798 | |
| 1.08772 | | 1.08297 | 1.18969 | | 1.05228 | | 1.29684 | | 1.13382 | 1.07394 | | 1.14798 | |
| 1.08772 | | 1.08297 | 1.18969 | | 1.05228 | | 1.29684 | | 1.13382 | 1.07394 | | 1.14798 | |
| 1.05684 | | 1.08354 | 1.04301 | | 1.07795 | | 1.02966 | | 1.05859 | 1.06024 | | 1.05986 | |
| 1.05684 | | 1.08354 | 1.04301 | | 1.07795 | | 1.02966 | | 1.05859 | 1.06024 | | 1.05986 | |
| 1.05684 | | 1.08354 | 1.04301 | | 1.07795 | | 1.02966 | | 1.05859 | 1.06024 | | 1.05986 | |
| 1.05684 | | 1.08354 | 1.04301 | | 1.07795 | | 1.02966 | | 1.05859 | 1.06024 | | 1.05986 | |
| 1.06761 | | 1.04847 | 1.18092 | | 1.13608 | | 1.12997 | | 1.20517 | 1.12456 | | 1.15272 | |
| 1.06761 | | 1.04847 | 1.18092 | | 1.13608 | | 1.12997 | | 1.20517 | 1.12456 | | 1.15272 | |
| 1.07665 | | 1.06372 | 1.11079 | |  | 1.0008 | 1.09505 | | 1.09756 | 1.03827 | | 1.02203 | |
| 1.07665 | | 1.06372 | 1.11079 | |  | 1.0008 | 1.09505 | | 1.09756 | 1.03827 | | 1.02203 | |
| 1.12043 | | 1.13117 | 1.26382 | | 1.13608 | | 1.42378 | | 1.18036 | 1.15254 | | 1.22696 | |
| 0.923933 | | 0.895554 | 0.987069 | | 0.910008 | | 0.96079 | | 0.942907 | 0.924731 | | 0.957149 | |
| 0.923933 | | 0.895554 | 0.987069 | | 0.910008 | | 0.96079 | | 0.942907 | 0.924731 | | 0.957149 | |
| 1.00623 | | 1.00611 | 1.00412 | | 0.988592 | | 0.972773 | | 0.970742 | 0.991086 | | 0.968777 | |
| 1.00623 | | 1.00611 | 1.00412 | | 0.988592 | | 0.972773 | | 0.970742 | 0.991086 | | 0.968777 | |

| 0.973458 | | 0.969905 | | 0.982372 | | 0.958274 | | 1.00313 | | 0.98048 | 0.956505 | | 0.974487 | |
| --- | --- | --- | --- | --- | --- | --- | --- | --- | --- | --- | --- | --- | --- | --- |
| 0.924757 | | 0.940257 | | 1.03389 | | 0.950588 | | 0.967299 | | 1.00158 | 0.997968 | | 0.969296 | |
| 1.01273 | | 1.01847 | | 0.951644 | | 1.01206 | | 0.996674 | | 1.00564 | 0.987944 | | 0.989136 | |
| 0.980928 | | 0.995515 | | 0.989505 | | 1.00439 | | 0.985091 | | 1.00391 | 0.944774 | | 0.973109 | |
| 1.03751 | | 1.02472 | | 1.01305 | | 1.01594 | | 0.989833 | | 1.01754 | 1.04164 | | 0.997655 | |
| 1.04573 | | 1.03373 | | 1.03861 | | 1.01955 | | 1.00408 | | 1.03262 | 1.05786 | | 1.04639 | |
| 1.04001 | |  | 1.0528 | 1.01826 | | 1.02403 | | 1.00132 | | 1.09392 | 1.05534 | | 0.968426 | |
| 1.01383 | | 0.994417 | | 1.00676 | | 1.00598 | | 1.04909 | | 1.05041 | 1.04272 | | 0.996963 | |
| 1.00959 | | 1.00283 | | 0.998233 | | 1.02015 | | 1.05678 | | 1.02906 | 1.02737 | | 0.991951 | |
|  | 1.0068 | 0.978252 | | 1.01254 | | NA |  | 1.00977 | | 1.01232 | 1.00958 | | NA |  |
| 0.982143 | | 0.977217 | | 1.02238 | | 1.01006 | | 1.04782 | | 1.01419 | 1.04189 | | 0.986039 | |
|  | 1.0188 | 0.992698 | | 0.998872 | | 1.01315 | | 1.00484 | | 1.00435 | 1.00799 | | 1.01701 | |
| NA |  | NA |  | NA |  | NA |  | 1.02377 | | NA | NA |  | 0.975286 | |
| 1.02227 | | 0.995318 | | 0.976915 | | 1.04068 | | 1.00169 | | 0.98637 | 1.00494 | | 0.964932 | |
| NA |  | NA |  | NA |  | NA |  | 1.04579 | | NA | NA |  | NA |  |
| NA |  | NA |  | NA |  | NA |  | 1.04606 | | NA | NA |  | NA |  |
| NA |  | NA |  | NA |  | NA |  | NA |  | NA | NA |  | NA |  |
|  | 0.9481 | 0.966555 | | 0.963957 | | 0.98395 | | 1.01521 | | 0.99716 | 0.968195 | | 1.05263 | |
| NA |  | NA |  | NA |  | NA |  | 1.01075 | | NA | NA |  | NA |  |
| NA |  | NA |  | NA |  | NA |  | 0.997975 | | NA | NA |  | NA |  |
| NA |  | NA |  | NA |  | NA |  | 0.988225 | | NA | NA |  | NA |  |
| NA |  | NA |  | NA |  | NA |  |  | 1.0419 | NA | NA |  | NA |  |
| NA |  | NA |  | NA |  | NA |  | 1.02621 | | NA | NA |  | NA |  |
| 0.980611 | | 0.962718 | | 1.02262 | | 1.03162 | | 0.976706 | | NA | NA |  |  | 1.0286 |
| 0.99299 | | 0.978981 | | 0.973323 | | 1.02984 | | 1.00314 | | 1.01468 | NA |  | 1.02418 | |
| NA |  | NA |  | NA |  | NA |  | NA |  | 1.01501 | NA |  | NA |  |
| NA |  | NA |  | NA |  | NA |  | NA |  | 1.02998 | NA |  | NA |  |
| NA |  | NA |  | NA |  | NA |  | NA |  | 1.00522 | NA |  | NA |  |
| 1.11288 | | 1.11615 | | 1.22339 | |  | 1.1122 | 1.32764 | | 1.13565 | 1.12068 | | 1.16668 | |
| 1.03803 | |  | 1.0514 |  | 1.01 | 1.04101 | | 1.06891 | | 1.05695 | 1.06488 | | 1.04536 | |
| 1.03803 | |  | 1.0514 |  | 1.01 | 1.04101 | | 1.06891 | | 1.05695 | 1.06488 | | 1.04536 | |
| NA |  | NA |  | 1.32416 | | NA |  | NA |  | 0.849398 | NA |  | NA |  |
| 1.13372 | | 1.13444 | | 1.02085 | | NA |  | 1.09328 | | 1.03426 | 1.02166 | | NA |  |
| NA |  | NA |  | 1.03291 | | NA |  | 1.00765 | | 0.985081 |  | 1.0473 | NA |  |
| NA |  | NA |  | 1.07483 | | 0.987432 | | 1.04076 | | 0.987394 | 1.02642 | | 1.02954 | |
| 1.10316 | | 1.07489 | | 1.04317 | | 1.08476 | | 1.05073 | | 1.03261 | 1.04819 | | 1.10277 | |
| 1.00664 | | 0.974835 | | 0.993766 | | 0.997265 | |  | 1.0099 | 1.03804 | 1.01393 | | 1.08267 | |
| NA |  | NA |  |  | 1.0242 | NA |  | 1.07714 | | 0.961913 | NA |  | NA |  |
| 1.00266 | | 1.01247 | | 1.03235 | | NA |  | 1.01262 | | 1.00957 | 1.02325 | | NA |  |

| 0.939108 | 0.885297 | | 1.02066 | | 0.979501 | 1.03883 | | 0.991594 | | 1.04514 | | 1.03184 | |
| --- | --- | --- | --- | --- | --- | --- | --- | --- | --- | --- | --- | --- | --- |
| NA | NA |  | 0.991679 | | NA | NA |  | 0.934382 | | 0.903424 | | NA |  |
| NA | NA |  | 1.02218 | | NA | NA |  | 0.988347 | | 0.979306 | | NA |  |
| NA | NA |  | 1.01969 | | NA | 1.00936 | | 0.989141 | | 0.999781 | | NA |  |
| NA | NA |  |  | 1.055 | NA | 1.06033 | | 1.01417 | | 1.04063 | | NA |  |
| 1.03257 | 1.02404 | | 1.01797 | | 0.983554 | 0.999347 | | 1.00776 | |  | 1.0144 | 0.980912 | |
| NA | NA |  | NA |  | NA | NA |  | NA |  | NA |  | NA |  |
| NA | NA |  | NA |  | NA | NA |  | NA |  | NA |  | NA |  |
| NA | NA |  | NA |  | NA | NA |  | NA |  | NA |  | NA |  |
| NA | NA |  | NA |  | NA | NA |  | NA |  | NA |  | NA |  |
| 0.989076 | 1.02013 | | 1.01288 | | 1.06868 | 1.02416 | | 1.04374 | | 1.03465 | | 1.11642 | |
| 1.07027 | 1.10243 | | 1.05108 | | 1.09249 | 1.05147 | | 1.06725 | | 1.04297 | | 1.07171 | |
| 1.08006 | 1.05566 | | 1.06239 | | 1.09231 | 1.08092 | | 1.12993 | | 1.06275 | | 1.05969 | |
| NA | NA |  | NA |  | NA | NA |  | NA |  | NA |  | NA |  |
| 1.10053 | 1.08972 | | NA |  | NA | 1.04473 | | 1.01547 | | NA |  | 1.02886 | |
| 1.06877 | 1.07676 | | 1.09852 | | 1.09614 | 1.01016 | | 1.06465 | | 0.976235 | | 1.04142 | |
| 1.02851 | 1.07837 | | 1.03255 | | 1.00967 | 1.09367 | | 1.06481 | | 1.02219 | | 1.00045 | |
| 1.01357 | 1.00479 | | NA |  | NA | 1.02558 | |  | 1.0209 | 1.00116 | | 0.994314 | |
| NA | NA |  | NA |  | NA | NA |  | 1.05564 | | NA |  | NA |  |
| 0.977171 | 0.962244 | | NA |  | 0.986534 | 1.04876 | | 0.981318 | | 1.04784 | |  | 1.0238 |
| 0.997553 | 0.956159 | | 1.01586 | | 0.998561 | 1.01759 | | 0.986583 | | 1.00117 | | 1.06256 | |
| 0.954001 | 0.927366 | | 1.01735 | | 0.951993 | 0.95462 | | 0.97429 | | 1.04117 | | 0.995515 | |
| 0.958189 | 0.922157 | | 1.00933 | | 0.916172 | 0.998494 | | 0.973098 | | 1.00411 | | 1.02313 | |
| 1.12876 |  | 1.1391 | 1.26066 | | 1.17271 | 0.980433 | | 1.15853 | | 1.24408 | | 1.14992 | |
| 1.08613 | 1.07776 | | 0.988491 | | 1.04378 | 1.04536 | | 1.05162 | | 0.982862 | | 1.03713 | |
| 0.976663 | 1.01877 | | 1.03591 | | 1.00877 | 1.02608 | | 1.13656 | | 1.08994 | | NA |  |
| 1.01515 | 1.00945 | | 0.996048 | | 1.06388 |  | 1.0104 | 1.06747 | | 0.966914 | | 1.01766 | |
| 1.05393 | 1.01632 | | 0.984188 | | 1.02903 | 1.00062 | | 0.99927 | | 1.00525 | | 0.988657 | |
| 1.03868 |  | 1.0031 |  | 1.0504 | 1.07346 | 1.01879 | | 1.05877 | | 1.02867 | | 1.03443 | |
| 1.04417 | 1.07448 | | 0.986853 | | 1.07734 | 1.04367 | | 1.03214 | | 1.02817 | | 0.95953 | |
| 0.992021 | 0.973404 | | 1.12091 | | 1.03814 | 1.06111 | | 1.13846 | |  | 1.0369 | 1.07342 | |
| 1.07684 | 1.09045 | | 1.01178 | | 1.06249 | 1.02702 | | 0.982836 | | 0.984526 | | 0.99178 | |
| 0.947518 | 0.913917 | | 1.06539 | | 0.937996 | 1.02381 | | 0.956129 | | 1.05789 | | 1.01769 | |
| 1.05969 | 1.04946 | | 1.05305 | | 1.07108 |  | 1.0171 | 1.00976 | | 0.983572 | | 0.982986 | |
| 1.04408 | 1.03662 | | 0.947803 | | 0.987261 | 0.998804 | |  | 1.0704 | 1.05324 | | 0.933714 | |
| 0.958746 |  | 0.955 | 0.945763 | | 0.99328 | 0.999367 | | 1.09494 | | 1.03023 | | 1.03139 | |
| NA | NA |  | 0.986501 | | NA | 0.99815 | | NA |  | 0.964794 | | NA |  |
| NA | NA |  | 0.986501 | | NA | 0.99815 | | NA |  | 0.964794 | | NA |  |
| NA | NA |  | 0.986501 | | NA | 0.99815 | | NA |  | 0.964794 | | NA |  |

| 0.99564 | | 0.985502 | | 0.952712 | | 1.00534 | | 1.02724 | | 0.958765 | NA | 0.97658 | |
| --- | --- | --- | --- | --- | --- | --- | --- | --- | --- | --- | --- | --- | --- |
| 0.963686 | | 0.963231 | | 0.985199 | | 1.01095 | | 1.01123 | | 0.939305 | 0.964876 | 0.984848 | |
| 1.11522 | | 1.10099 | | NA |  | 1.14385 | | 0.999936 | | 1.05338 | NA | 1.05825 | |
| 0.980762 | | 0.970344 | | 1.01906 | | 0.961799 | | 0.988529 | | 0.990472 | 0.998328 | 1.03355 | |
| NA |  | NA |  | 1.03292 | | NA |  | 1.11383 | | 1.00401 | 1.06127 | NA |  |
| NA |  | NA |  | 1.11469 | | NA |  | 1.02589 | | 0.982971 | 1.07145 | NA |  |
| NA |  | NA |  | 1.06026 | | NA |  | 1.02271 | | 0.937841 | 1.11741 | NA |  |
|  | 1.0436 | 1.08465 | | 1.02521 | | 1.02978 | | 1.01833 | | 1.06718 | 1.01706 | 1.12999 | |
| 1.13001 | | 1.12836 | |  | 1.033 | 1.07836 | | 1.00935 | | 1.03401 | 1.05104 | 1.07015 | |
| 1.11946 | |  | 1.0962 | 1.01756 | | 1.09049 | | 1.07587 | | 1.05107 | 0.969588 |  | 1.051 |
| 1.04612 | |  | 1.0605 | 0.961851 | | 1.01362 | | 0.991293 | | 0.970171 | 0.975824 | 1.05422 | |
| 1.04612 | |  | 1.0605 | 0.961851 | | 1.01362 | | 0.991293 | | 0.970171 | 0.975824 | 1.05422 | |
|  | 1.0658 | 1.04476 | | 1.03963 | |  | 1.0264 | 1.04217 | | 1.00845 | 1.04568 | 1.05502 | |
| 0.994694 | | 0.979533 | | 1.03546 | | 0.956269 | | 0.996531 | | 1.04533 | 0.991907 | 0.965826 | |
| 0.994694 | | 0.979533 | | 1.03546 | | 0.956269 | | 0.996531 | | 1.04533 | 0.991907 | 0.965826 | |
| 1.00369 | | 1.01415 | | 0.977196 | | 0.940708 | | NA |  | 0.916934 | NA | 0.96151 | |
| NA |  | NA |  | 0.982312 | | 0.959718 | | NA |  | NA | NA | NA |  |
| NA |  | NA |  | 0.984742 | | 0.944716 | | NA |  | NA | NA | NA |  |
| NA |  | NA |  | 1.05866 | |  | 1.089 | 1.07474 | | 0.942974 | 1.04491 | 1.04347 | |
| 1.05455 | | 1.04775 | |  | 1.0826 | 1.06076 | | 1.04509 | | 1.10607 | 1.03686 | 1.04696 | |
| 1.09048 | | 1.10635 | | 1.01031 | | 1.11201 | | 1.00289 | | 1.03347 | 0.971789 |  | 1.1111 |
|  | 1.0112 | 0.992962 | | 1.00145 | | 1.03598 | | 0.951406 | | 0.952489 | 0.929536 | 1.01955 | |
| 1.01349 | | 1.01435 | | 0.976685 | | 1.04002 | | 0.977879 | | 1.00805 | 1.03588 | 1.04996 | |
| 1.04724 | | 1.05404 | | 0.998066 | | 1.07987 | | 0.982122 | | 1.01222 | 1.03513 | 1.09452 | |
| 1.05304 | | 1.03609 | | 0.938399 | | 1.08404 | | 1.00406 | | 1.01113 | 0.992507 | 1.06006 | |
| 1.01839 | | 1.02147 | | 1.05138 | | 1.05695 | | 0.979411 | | 1.03517 | 1.01487 | 1.02998 | |
| 0.979674 | | 0.952141 | | 0.961177 | | 0.998526 | |  | 1.0178 | 0.937455 | 1.01001 | 1.04076 | |
| 0.989696 | | 0.997527 | |  | 1.0212 | 1.06218 | | 1.00815 | | 0.999632 | 1.03261 | 1.07682 | |
| 0.897692 | | 0.92814 | | 0.963784 | | 0.951459 | | 1.08881 | | 0.952766 | 0.994125 | 0.944633 | |
| 1.05223 | | 1.06436 | | 1.07113 | |  | 1.0076 | 1.00559 | | 0.989878 | 0.959611 | 1.02302 | |
| 0.923423 | | 0.920121 | | 1.01678 | | 0.912358 | | 0.94182 | | 0.913162 | 0.936976 | 0.894499 | |
| NA |  | NA |  | 0.996394 | | NA |  | 0.990858 | | 0.945121 | NA | 0.965801 | |
| NA |  | NA |  | 0.997236 | | NA |  | NA |  | 0.976454 | NA | 0.911403 | |
| NA |  | NA |  |  | 0.9742 | NA |  | NA |  | 1.04406 | 1.01306 | NA |  |
| NA |  | NA |  | 0.967977 | | NA |  | NA |  | 1.02783 | 0.959897 | NA |  |
| 1.08814 | | 1.08345 | | 1.04082 | | NA |  | 1.04844 | | 1.08552 | 1.03987 | 1.10671 | |
| 1.03163 | | 1.04638 | | 1.04876 | |  | 1.0555 | 1.03689 | | 1.01295 | 1.02496 | 1.05912 | |
| 1.05409 | | 1.05698 | | 0.982542 | | 1.00584 | | 1.05848 | | 1.00742 | 1.07111 | 0.995316 | |
| NA |  | NA |  | NA |  | NA |  | 1.08605 | | NA | NA | NA |  |

| 1.05387 | | 1.04559 | | 1.07739 | | 1.05594 | | 1.03539 | | 1.05362 | 1.03308 | | 1.04766 | |
| --- | --- | --- | --- | --- | --- | --- | --- | --- | --- | --- | --- | --- | --- | --- |
| 0.906534 | | 0.904745 | | 0.999811 | | 0.926099 | | 0.962914 | | 0.986705 | 0.978268 | | 0.92959 | |
| 1.15234 | | 1.13827 | | 1.18327 | | 1.15232 | | 1.25715 | | 1.12178 | 1.12474 | | 1.12133 | |
| 1.10532 | | 1.09071 | | 1.16817 | | 1.10403 | |  | 1.2529 | 1.09469 | 1.05162 | | 1.12062 | |
| 1.09838 | | 1.06683 | | 1.10709 | | 1.06954 | | 1.17985 | | 1.02859 | 1.04602 | |  | 1.0843 |
| 1.02033 | | 1.02434 | | 0.979492 | | 1.03329 | | 0.964467 | | 0.990441 | 0.899157 | | 0.96487 | |
| 0.970732 | | 0.962946 | | 0.986973 | | NA |  | 1.05289 | | 0.985686 |  | 1.1197 | NA |  |
| 1.09129 | | 1.07984 | | 1.13598 | | 1.03206 | |  | 1.224 | 1.14633 | 1.06815 | | 1.10215 | |
| 1.09104 | | 1.11411 | | 1.11774 | | 1.11024 | | 1.20271 | | 1.10249 | 1.07934 | | 1.09609 | |
| NA |  | NA |  | NA |  | NA |  | NA |  | 1.06069 | NA |  | NA |  |
| NA |  | NA |  | NA |  | NA |  | NA |  | 1.04378 | NA |  | NA |  |
| NA |  | NA |  | NA |  | NA |  | NA |  | NA | NA |  | NA |  |
| 1.16213 | | 1.15892 | | 1.23085 | | 1.19623 | | 0.992557 | | 1.16274 | 1.27436 | | 1.21547 | |
| 1.08772 | | 1.10368 | | 1.08661 | | 1.12457 | | 1.17185 | | 1.15552 | 1.10848 | | 1.08676 | |
| 0.925225 | | 0.946542 | | 0.956157 | | NA |  | 0.961134 | | 0.973117 | 0.932079 | | 0.94008 | |
| 1.01329 | | 1.03179 | | 1.08884 | | 1.03341 | |  | 1.1581 | 1.10654 | 1.11105 | | 1.02545 | |
| 0.885186 | | 0.898689 | | 0.948708 | | NA |  | 0.934279 | | NA | 0.936582 | | 0.934783 | |
| NA |  | 1.00935 | | NA |  | NA |  |  | 1.1022 | NA | NA |  | 1.00486 | |
| 1.01798 | | 0.998573 | | 1.02955 | | 1.01882 | | 1.03302 | | 1.00621 | 1.00382 | |  | 1.013 |
| NA |  | NA |  | NA |  | NA |  | NA |  | 0.997697 | NA |  | NA |  |
| 1.12024 | | 1.13895 | | 1.12994 | | 1.08869 | | 1.08381 | | 1.12245 | 1.13666 | | 1.05367 | |
| 0.989923 | | 1.01776 | | 1.03917 | | 1.00038 | |  | 1.0135 | 1.04956 | 1.03243 | | 1.00846 | |
| NA |  | NA |  | 1.04761 | | NA |  | 1.09216 | | NA | NA |  | NA |  |
| NA |  | NA |  |  | 1.0833 | NA |  |  | 1.1198 | NA | 1.02342 | | NA |  |
| 0.985463 | |  | 1.0226 | 1.07259 | | 0.976794 | | 1.05302 | | 1.05722 | 1.08375 | | 1.00542 | |
| 0.960163 | | 0.987293 | | 1.00212 | | 0.960872 | | 1.01258 | | 0.988441 | 1.06542 | | 1.01501 | |
| 0.99988 | | 1.00038 | | 1.06064 | | 0.975367 | | 1.04098 | | 1.10608 | 1.05295 | | 0.975339 | |
| 1.00813 | | 0.993452 | | NA |  | 1.04442 | | 1.04554 | | 1.00513 | 0.986622 | | 1.08356 | |
| 0.965146 | | 0.973975 | | 1.02226 | | 1.01893 | | 1.01465 | | 1.02865 | 0.989332 | | 0.991525 | |
| 1.06331 | | 1.10139 | | 1.12409 | | 1.10081 | | 1.15861 | | 1.12811 | 1.15951 | | 1.11428 | |
| 0.970933 | | 1.01643 | | 1.06903 | | 1.04615 | | 1.09339 | | 1.02042 | 0.990707 | | 0.985765 | |
|  | 1.0803 | 1.11375 | | 1.02802 | | 1.10459 | | 1.10173 | | 1.03045 | 1.03701 | | 1.12171 | |
| 1.12184 | | 1.09312 | | 1.01991 | | 1.05623 | |  | 1.0189 | 1.00539 |  | 1.0212 | 1.04305 | |
| 0.956868 | | 0.972883 | | 1.07558 | | 0.981598 | | 1.09822 | | 1.01597 | 1.04761 | | 1.00787 | |
| 1.11736 | | 1.14719 | |  | 1.1082 | 1.13961 | | 1.05631 | | 1.04764 | 1.10506 | | 1.13465 | |
| 0.986386 | | 0.988942 | | 0.998853 | | 1.03498 | | 0.977426 | | 1.04317 | 1.00008 | | 1.00944 | |
| 0.977815 | | 1.00742 | | 1.01529 | |  | 1.0354 | 0.954578 | | 1.02974 | 0.970901 | | 1.00933 | |
| 0.998713 | | 0.961319 | | 0.989291 | | 1.03222 | | 1.02755 | | 1.04374 | 0.995549 | | 1.07661 | |
| 1.07536 | | 1.07274 | | 1.09652 | | 1.08005 | | 1.01513 | | 1.04562 | 1.09704 | | 1.03782 | |

| 0.963028 | | 1.00275 | | 1.06988 | | 1.07633 | | 1.03391 | | 0.96309 | | 0.953898 | | 1.13757 | |
| --- | --- | --- | --- | --- | --- | --- | --- | --- | --- | --- | --- | --- | --- | --- | --- |
| 1.02941 | | 1.01599 | | 0.987142 | | 0.991106 | | 1.02905 | | 0.924072 | | 0.962671 | | 0.99529 | |
| 1.09961 | | 1.09864 | | 1.05979 | |  | 1.1235 | 1.20113 | | 1.07373 | |  | 1.122 | 1.10668 | |
| 1.05438 | | 1.03717 | | 1.02603 | | 1.04047 | | 1.02943 | | 1.02955 | | 0.940925 | | 1.01695 | |
| 1.20675 | | 1.20642 | | 1.30576 | | 1.22815 | | 1.08644 | | 1.19414 | | 1.23076 | |  | 1.1597 |
| NA |  | NA |  | 1.00966 | | NA |  | 1.00425 | | 1.01699 | | 0.976805 | | NA |  |
| 1.02638 | | 1.02367 | | 0.989467 | | 0.984201 | | 1.00553 | | 1.02023 | | 1.00346 | | 0.992896 | |
| 0.997205 | | 0.998267 | | 0.989574 | | 0.986159 | | 0.981489 | | 1.01102 | | 1.00465 | | 0.99688 | |
| 1.09541 | |  | 1.0931 | 1.08294 | | 1.08392 | | 1.08215 | | 1.06971 | | 1.12737 | | 1.06614 | |
| 1.05133 | | 1.02199 | | 1.06421 | | 1.04292 | | 1.01449 | | 1.06725 | | 1.06302 | | 0.958447 | |
| 1.02884 | | 0.991539 | |  | 1.0107 | 0.985166 | | 0.976881 | | 0.959617 | | 1.00758 | | 0.933106 | |
| NA |  | NA |  | 0.98454 | | NA |  | NA |  |  | 1.0206 | NA |  | NA |  |
| 0.971015 | | 0.960385 | | 1.05407 | | 0.982377 | | 1.05527 | | 0.980748 | |  | 1.0216 | 1.06217 | |
| 1.09807 | |  | 1.0917 | 1.13524 | | 1.11788 | | 1.07632 | | 1.04911 | | 1.10407 | | 1.12384 | |
| 1.04834 | | 1.03142 | | 1.05585 | | 1.09571 | | 1.11315 | | 0.994664 | | 1.07602 | | 1.13781 | |
|  | 1.0625 | 1.07699 | | 1.02443 | | 1.02064 | |  | 1.0251 | 1.02789 | | 1.03762 | | 1.05301 | |
| 1.10579 | | 1.12386 | | 1.03295 | | NA |  | 1.02215 | | 0.993053 | | 1.00041 | | 1.05081 | |
| 1.09804 | | 1.14865 | | 0.949113 | |  | 1.0025 | 0.990238 | | 0.938068 | | 0.982697 | | 1.05058 | |
| NA |  | NA |  | 0.950576 | | NA |  | 1.01702 | | 0.975053 | | NA |  | NA |  |
| 1.14004 | | 1.08601 | | 1.07107 | | 1.05848 | | 1.03036 | | 1.02057 | | 0.95667 | | 1.01547 | |
| 1.02988 | | 1.04042 | |  | 1.1077 | 1.01129 | | 1.03385 | | 1.06704 | | 1.03956 | | 1.03149 | |
| 0.982071 | | 1.00183 | | NA |  | 0.999655 | | NA |  | NA |  | NA |  | 0.930324 | |
| 0.994871 | | 0.987075 | | 1.05506 | | 1.00789 | | 0.990655 | | 1.01036 | | 1.00342 | | 1.01428 | |
| 0.957361 | | 0.937402 | | 0.982991 | | 1.00397 | | NA |  | 0.972058 | | 0.994938 | | 1.03397 | |
| 1.00887 | | 1.01875 | | 1.00672 | | 0.994171 | | 0.998881 | | 1.02015 | | 1.00027 | | 1.00018 | |
| 1.02754 | | 1.02936 | | 1.03746 | | 1.11893 | |  | 1.0713 | 1.05867 | | 1.02641 | | 1.12285 | |
| 1.04487 | | 1.02587 | | 1.04281 | |  | 1.0127 | 1.01511 | | 1.01948 | |  | 1.0679 | 0.970896 | |
| 0.957964 | | 0.944275 | | 1.00776 | | 1.00751 | | 1.03466 | | 1.02601 | | 1.01742 | | 1.03215 | |
| 1.02819 | | 1.04306 | | 1.02852 | | 1.02448 | | 0.983696 | | 0.993272 | | 0.996172 | | 0.985524 | |
| 0.972823 | | 0.958778 | | 1.00071 | | 0.974242 | | 0.967425 | | 0.913311 | | 0.984337 | | 0.934827 | |
| 1.01341 | | 1.00299 | | 0.986913 | | 1.03066 | | 1.00555 | | 1.02529 | |  | 1.025 | 1.04012 | |
| 1.10935 | | 1.13648 | | 1.22818 | | 1.21856 | |  | 1.1512 |  | 1.165 | 1.16456 | | 1.21329 | |
| 0.96556 | | 0.970113 | | 1.00489 | | 0.965748 | | 1.05947 | | 0.972719 | | 0.997982 | | 0.963235 | |
| 1.00457 | | 1.02029 | | 1.01736 | |  | 1.0106 | 1.04576 | | 0.942709 | | 0.969485 | |  | 1.0471 |
| 1.05243 | | 1.05864 | | 1.10562 | | NA |  | 1.05847 | | NA |  |  | 1.0663 | NA |  |
| 1.11778 | | 1.06492 | | 1.07693 | | 1.03762 | | 1.05508 | | 1.06147 | | 1.04806 | | 1.01102 | |
|  | 1.0597 | 1.03647 | | 1.09594 | | 1.07657 | | 1.09433 | | 1.02299 | | 1.02022 | | 1.02959 | |
| 1.05844 | | 1.03653 | | 0.978432 | | 1.03009 | | 1.00424 | | 1.00147 | |  | 1.0511 | 1.03794 | |

precursor_ regulatory segmente sigmoid_c t_cell..cee thyroid..ce venous_bl Motif

1.14862	1.00074	1.08783	1.13993 0.994185	1.13104	1.08057 AHR_1.mo

NA	NA	NA	NA	NA	NA	NA	ALX3_1.m

1.14137	1.29946	1.13578	1.20078	1.22875	1.05765	1.23291 ARNT_1.m

1.04011	1.1737	1.17258	1.13562	1.1299	1.15901	1.31298 ARNT_2.m

1.11569	1.28692	1.05821 NA	1.19435	1.15015	1.34372 ARNTL_1.

0.999384 0.920286	1.00185 0.990126 0.884306	1.01709 0.953249 ASCL2_1.

1.18978	1.15317	1.08972	1.20707	1.12087	1.18012	1.29172 ATF3_1.m

1.01773	1.12486	1.03709 NA	1.01226	1.20257	1.22897 ATF6_1.m

1.01773	1.12486	1.03709 NA	1.01226	1.20257	1.22897 ATF6_1.m

NA	1.0327	1.10619	1.26128	1.11527	1.06474	1.07378 ATF7_1.m

0.997037 0.977909 0.971247 0.991577 0.969237	1.03032 0.956507 ATOH1_1.

0.990461 0.966407	1.05019	1.03226 0.959223	1.08257 0.976871 ATOH7_1.

0.939986	1.06872	1.0792	1.07085	1.02229	1.04696	1.00583 BACH1_1.

0.961538	1.02527	1.08485	1.01807	1.00847	1.02389 0.997963 BACH2_1. NA	NA	NA	0.993417 NA	NA	1.11351 BARHL2_1

NA	NA	NA	0.945827 NA	NA	NA	BARX1_2. NA	1.02368 NA	0.979684	1.02808 0.966241 0.990474 BATF_1.m NA	1.02368 NA	0.979684	1.02808 0.966241 0.990474 BATF_1.m

0.96069 0.936672	1.0138 0.921797 0.977964 0.947911	1.00972 BCL11A_1

1.06533	1.38424	1.14518	1.17991	1.2717	1.22499	1.381 BHLHE40_ NA	NA	NA	0.944378 NA	NA	NA	BSX_1.mo

0.994347	1.03472	1.02817 0.990137	1.07081 0.966306	1.02949 CBFB_1.m NA	NA	NA	1.0313 NA	NA	NA	CDX1_1.m NA	NA	NA	1.03817 NA	NA	NA	CDX2_1.m NA	NA	1.04433	1.11898	1.03059 NA	NA	CEBPA_1. NA	NA	1.03608 NA	NA	NA	NA	CEBPB_1. NA	NA	1.06397 NA	NA	NA	NA	CEBPD_1. NA	NA	1.05507 NA	NA	NA	NA	CEBPE_1.

1.10068	1.20144	1.07273	1.20308	1.14608	1.11434	1.30668 CLOCK_1.

1.10068	1.20144	1.07273	1.20308	1.14608	1.11434	1.30668 CLOCK_1.

1.07932	1.31944	1.05627	1.22892	1.17922	1.22512	1.45015 CREB3L2_

1.0818	1.26966	1.11689	1.24434	1.22968	1.17433	1.49165 CREB3L3_ NA	0.962663	1.05356	1.1764	1.10371 NA	1.04864 CREB5_1. NA	NA	1.01126	1.05835 NA	NA	NA	DBP_1.mo NA	NA	NA	NA	NA	NA	NA	DLX1_1.m NA	NA	NA	NA	NA	NA	NA	DLX2_1.m NA	NA	NA	NA	NA	NA	NA	DLX6_1.m

1.0068	1.15232	1.07302 NA	1.09906	0.99744 NA	E2F1_1.m

| 0.9845 | 0.94823 | 0.993974 | 1.00283 NA | 0.948187 | 0.973365 EBF1_1.m |
| --- | --- | --- | --- | --- | --- |
| 0.9845 | 0.94823 | 0.993974 | 1.00283 NA | 0.948187 | 0.973365 EBF1_1.m |
| 0.9845 | 0.94823 | 0.993974 | 1.00283 NA | 0.948187 | 0.973365 EBF1_1.m |

0.891069	1.01571	1.00277 NA	1.00618	1.07617	1.07459 EGR2_1.m

0.883183	1.06137 0.981221 NA	1.04726	1.00886	1.08234 EGR4_1.m

0.988378	1.0037 0.933583 0.994418	1.00801 0.930479	1.02794 EHF_1.mo

1.091	1.05872	1.04652	1.12265	1.03235	1.05501	1.03991 EHF_2.mo

1.08613	1.03046	1.07342	1.05024	1.00075	1.06998	1.03535 ELF1_1.m

1.03365 0.988726 0.973536	1.0342	1.04192 0.960417	1.02337 ELF2_1.m

1.13333	1.08079	1.06857	1.10593	1.06685	1.05452	1.03397 ELF3_1.m

1.13459	1.03722	1.09548	1.07383	1.05595	1.01208	1.01989 ELF4_1.m

1.10173	1.04679	1.05419	1.04945	1.04885	1.01042	1.0369 ELF5_1.m

1.17651	1.03838	1.10693	1.12829	1.07393	1.03468	1.01425 ELK1_1.m

1.10611	1.01697	1.08496	1.07758	1.01772	1.01004	1.01906 ELK3_1.m

1.16888 0.979593	1.06083	1.05696	1.03433 0.973843 0.982956 ELK4_1.m

NA	NA	NA	0.994529 NA	NA	NA	EN1_2.mo

1.06944	1.24743	1.04136	1.10855	1.26184	1.11667	1.31736 ENO1_1.m

1.12515	1.04993	1.07047	1.01628	1.05798	1.03098	1.06161 ERF_1.mo

1.12936	1.02894	1.04254	1.06799	1.10048 0.988816	1.03897 ERG_1.mo

1.10906	1.03608	1.07849	1.03106	1.1749	1.03279	1.10073 ERG_2.mo

1.07577 0.995265 0.985937 0.982219	1.02718 0.902303	1.01138 ERG_3.mo NA	NA	NA	0.981302 NA	NA	1.04616 ESRRG_3. NA	NA	NA	0.985952 NA	NA	NA	ESX1_1.m

1.12284 0.996627	1.06347	1.01712	1.05806	1.00142	1.0166 ETS1_1.m

1.42373	1.06404	1.04403	1.15217	1.1754 0.995516	1.10121 ETS1_2.m

1.07898	1.08715	1.08495	1.04234	1.09481	1.06111	1.00918 ETS2_1.m

1.13863	1.04124	1.09021	1.00978	1.05463	1.03524	1.02495 ETV1_1.m

1.12079	1.0388	1.02684	1.06869	1.06102	0.96805	1.01703 ETV2_1.m

1.11392	1.03898	1.04386	1.06342	1.0371	1.01914	1.00642 ETV3_1.m

1.11392	1.03898	1.04386	1.06342	1.0371	1.01914	1.00642 ETV3_1.m

1.13885	1.04636	1.06334	1.04548	1.07763 0.981599	1.03017 ETV4_1.m

1.07052	1.05991	1.07273	1.05149	1.07277	1.10218	1.04533 ETV5_1.m

1.23544	1.0282	1.05529	1.03406	1.06154	1.08929	1.00343 ETV6_1.m

1.01428	1.03528	1.00277	1.09426	1.05724 0.964778	1.05927 ETV7_1.m

NA	NA	NA	NA	NA	NA	NA	EVX2_1.m

1.01814	1.02581	1.03751	1.03486 0.981625	1.06694	1.00302 FERD3L_1

1.08503	1.04003	1.05924	1.07996	1.08059 0.988742	1.05152 FEV_1.mo

1.13281	1.03019	1.03488	1.04095	1.05955 0.987482	1.03005 FLI1_1.mo

1.1511	1.07609	1.10019	1.14836	1.15373	1.04181	1.08374 FLI1_2.mo

| NA |  | 1.0509 | 1.02821 |  | 1.0759 | 1.04063 | | 0.960004 | | 0.991736 | FOS_1.mo |
| --- | --- | --- | --- | --- | --- | --- | --- | --- | --- | --- | --- |
| NA | 0.977447 | | 0.974913 |  | 1.0684 | 1.02938 | | 0.938195 | | 0.993395 | FOSB_1.m |
| 0.912275 | 0.992709 | | 1.02512 | 1.08315 | | 1.02624 | | 1.02099 | | 1.00384 | FOSL1_1. |
| NA | 1.02808 | | 1.03803 | 1.12071 | |  | 1.0374 | NA |  | 0.996656 | FOSL2_1. |
| NA | NA |  | NA | 0.977743 | | NA |  | 0.932941 | | NA | FOXA1_1. |
| NA | NA |  | NA | 1.06288 | | NA |  | 0.963002 | | NA | FOXA2_1. |
| NA | NA |  | NA | 0.986767 | | NA |  |  | 1.0441 | NA | FOXC1_1. |
| NA | NA |  | NA |  | 1.0192 | NA |  | 0.963734 | | NA | FOXD1_1. |
| NA | NA |  | NA | 1.04492 | | NA |  | 0.975413 | | NA | FOXD2_2. |
| NA | NA |  | NA | 1.01364 | | NA |  | 0.963755 | | NA | FOXD4_1. |
| NA | NA |  | NA | 1.05851 | | NA |  | 0.96807 | | NA | FOXF1_1. |
| NA | NA |  | NA | 1.05851 | | NA |  | 0.96807 | | NA | FOXF1_1. |
| NA | NA |  | NA | 1.05851 | | NA |  | 0.96807 | | NA | FOXF1_1. |
| NA | NA |  | NA | 1.02634 | | NA |  | 0.935603 | | NA | FOXF2_1. |
| NA | NA |  | NA | 1.03927 | | 1.06792 | | 0.979335 | | NA | FOXG1_1. |
| NA | NA |  | NA | 1.04576 | | 1.10106 | | 0.998316 | | NA | FOXK1_1. |
| NA | NA |  | NA | 1.09013 | | NA |  | 0.988489 | | NA | FOXO3_1. |
| NA | NA |  | NA | 1.03895 | | NA |  | 1.02852 | | NA | FOXP1_1. |
| NA | 1.05346 | | NA | 1.02045 | |  | 1.1094 | 1.01314 | | 1.03381 | FOXP2_1. |
| NA | NA |  | NA | 1.02132 | | 1.07524 | | 0.983922 | | NA | FOXP3_1. |
| NA | NA |  | NA | 1.01645 | | NA |  | 0.962569 | | NA | FOXQ1_1. |
| 1.08199 | 1.04358 | | 1.09718 | 1.03886 | | 1.05544 | | 1.04567 | | 1.02586 | GABPA_1. |
| NA | 0.955693 | | 0.920422 | 1.01693 | | 0.974992 | | NA |  | 0.956458 | GATA1_1. |
| NA | 0.955693 | | 0.920422 | 1.01693 | | 0.974992 | | NA |  | 0.956458 | GATA1_1. |
| NA | NA |  | NA | 0.932215 | | 0.968339 | | NA |  | NA | GATA2_1. |
| NA | NA |  | NA | 0.932215 | | 0.968339 | | NA |  | NA | GATA2_1. |
| NA | NA |  | NA | 1.05124 | | 1.00683 | | NA |  | NA | GATA3_2. |
| NA | 0.990846 | | 1.03773 |  | 1.0592 | 0.964456 | | NA |  | NA | GATA4_1. |
| NA | NA |  | NA | 0.885832 | | NA |  | NA |  | NA | GATA5_1. |
| NA | NA |  | NA | 1.09224 | | NA |  | NA |  | NA | GATA6_1. |
| NA | NA |  | NA | 1.09224 | | NA |  | NA |  | NA | GATA6_1. |
| NA | NA |  | NA | 1.09224 | | NA |  | NA |  | NA | GATA6_1. |
| NA | NA |  | NA | NA |  | NA |  | NA |  | NA | GBX1_1.m |
| NA | NA |  | NA | 0.955426 | | NA |  | NA |  | NA | GBX2_1.m |
| NA | NA |  | NA | NA |  | NA |  | NA |  | NA | GSX1_1.m |
| NA | NA |  | NA | NA |  | NA |  | NA |  | NA | GSX2_1.m |
| 0.883046 | 0.951719 | | 0.948171 | 0.960065 | | 0.965679 | | 0.946533 | | 1.00167 | HAND1_1. |
| 1.08825 | 1.12161 | | 1.11062 | 0.984231 | | 1.15478 | |  | 1.1331 | 1.19306 | HES1_1.m |
| 1.08825 | 1.12161 | | 1.11062 | 0.984231 | | 1.15478 | |  | 1.1331 | 1.19306 | HES1_1.m |

| 1.03223 | | 1.29538 | 1.11938 | | 1.25572 | |  | 1.1744 | 1.24922 | | 1.38528 | HES4_1.m |
| --- | --- | --- | --- | --- | --- | --- | --- | --- | --- | --- | --- | --- |
| NA |  | NA | 1.12124 | | NA |  | NA |  | 1.15485 | | 1.28664 | HES7_1.m |
| 1.15048 | | 1.05424 | 1.07464 | | 1.10885 | | 1.05177 | |  | 1.1725 | 1.16345 | HEYL_1.m |
| 0.977259 | | 1.02001 | 0.999626 | | NA |  | NA |  | 0.974687 | | 1.05945 | HIC1_1.mo |
| 1.03114 | | 1.06224 | 1.10161 | | 1.07483 | | 1.11127 | |  | 0.9604 | 1.06798 | HIVEP2_1. |
| NA |  | NA |  | 1.0377 | 1.00236 | | NA |  | NA |  | NA | HLF_1.mo |
| NA |  | NA | NA |  | 1.08667 | | NA |  | NA |  | NA | HNF1A_1. |
| NA |  | NA | NA |  | 1.20391 | | NA |  | NA |  | NA | HNF1B_1. |
| NA |  | 0.955207 | 0.968709 | | 1.02225 | | NA |  | 0.925203 | | 1.00678 | HNF4A_1. |
| NA |  | 1.08007 | 1.03887 | | 1.04599 | | NA |  | 0.953474 | | 1.02288 | HNF4A_2. |
|  | 1.0198 | 0.960535 | 0.995863 | |  | 1.0183 | 0.951981 | | 0.950874 | | 0.989153 | HNF4G_1. |
| NA |  | NA | NA |  | NA |  | NA |  | NA |  | NA | HOXA1_1. |
| NA |  | NA | NA |  | 1.09767 | | NA |  | NA |  | NA | HOXA10_1 |
| NA |  | NA | NA |  | 1.06504 | | NA |  | NA |  | NA | HOXA11_1 |
| NA |  | NA | NA |  | 1.01276 | | NA |  | NA |  | NA | HOXA13_1 |
| NA |  | NA | NA |  | 1.07054 | | NA |  | NA |  | NA | HOXA13_2 |
| NA |  | NA | NA |  | NA |  | NA |  | NA |  | NA | HOXA2_1. |
| NA |  | NA | NA |  | 1.03402 | | NA |  | NA |  | NA | HOXA5_2. |
| NA |  | NA | NA |  | NA |  | NA |  | NA |  | NA | HOXA6_1. |
| NA |  | NA | NA |  | 1.00414 | | 0.99031 | | NA |  | NA | HOXA6_10 |
| NA |  | NA | NA |  | 0.97776 | | NA |  | NA |  | NA | HOXA6_4. |
| NA |  | NA | NA |  | 1.01133 | | NA |  | NA |  | NA | HOXA6_7. |
| NA |  | NA | NA |  | 1.13306 | | NA |  | NA |  | NA | HOXA6_8. |
| NA |  | NA | NA |  | NA |  | NA |  | NA |  | NA | HOXA6_9. |
| NA |  | NA | NA |  |  | 1.043 | NA |  | NA |  | NA | HOXA9_1. |
| NA |  | NA | NA |  | 1.07946 | | 0.905172 | | NA |  | NA | HOXA9_2. |
| NA |  | NA | NA |  | 1.01313 | | NA |  | NA |  | NA | HOXB13_1 |
| NA |  | NA | NA |  | 1.07618 | | NA |  | NA |  | NA | HOXB13_2 |
| NA |  | NA | NA |  | NA |  | NA |  | NA |  | NA | HOXB2_1. |
| NA |  | NA | NA |  | NA |  | NA |  | NA |  | NA | HOXB3_1. |
| NA |  | 0.980067 | NA |  | 1.07203 | |  | 1.035 | NA |  | NA | HOXB4_1. |
| NA |  | NA | NA |  | NA |  | NA |  | NA |  | NA | HOXB5_1. |
| NA |  | NA | NA |  | 1.02487 | | NA |  | NA |  | NA | HOXB7_1. |
| NA |  | NA | NA |  | 1.04836 | | NA |  | NA |  | NA | HOXB8_1. |
| NA |  | NA | NA |  | 1.04773 | | NA |  | NA |  | NA | HOXC10_1 |
| NA |  | NA | NA |  | 1.09714 | | NA |  | NA |  | NA | HOXC11_1 |
| NA |  | NA | NA |  | 1.03961 | | NA |  | NA |  | NA | HOXC11_2 |
| NA |  | NA | NA |  | 0.998907 | | NA |  | NA |  | NA | HOXC12_2 |
| NA |  | NA | NA |  | 1.05671 | | NA |  | NA |  | NA | HOXC13_2 |

| NA | NA |  | NA |  | NA |  | NA |  | NA |  | NA |  | HOXC5_1. |
| --- | --- | --- | --- | --- | --- | --- | --- | --- | --- | --- | --- | --- | --- |
| NA | NA |  | NA |  | 0.960269 | | 0.97801 | | NA |  | NA |  | HOXC9_1. |
| NA | NA |  | NA |  | NA |  | NA |  | NA |  | NA |  | HOXD1_1. |
| NA | NA |  | NA |  | 1.05834 | | NA |  | NA |  | NA |  | HOXD11_ |
| NA | NA |  | NA |  | 1.06395 | | NA |  | NA |  | NA |  | HOXD12_ |
| NA | NA |  | NA |  | 1.10706 | |  | 1.1899 | NA |  | NA |  | HOXD12_ |
| NA | NA |  | NA |  |  | 1.0523 | NA |  | NA |  | NA |  | HOXD13_ |
| NA | NA |  | NA |  | 1.06234 | | NA |  | NA |  | NA |  | HOXD13_ |
| NA | NA |  | NA |  | NA |  | NA |  | NA |  | NA |  | HOXD3_1. |
| NA | NA |  | NA |  | 1.03112 | | 1.07181 | | NA |  | NA |  | HOXD9_2. |
| 0.916578 | 0.938468 | | 0.971156 | | 0.993676 | | 0.954217 | | 0.953192 | | 0.984663 | | IKZF3_1.m |
| NA | 1.00227 | | 1.01919 | | 1.25217 | | 1.25895 | | 1.25799 | | 1.22639 | | IKZF5_1.m |
| NA | 1.00978 | | NA |  | 1.00821 | |  | 1.0273 | 1.04229 | | 0.995702 | | IRF1_1.mo |
| 0.902549 | 1.02651 | | 0.914969 | | 0.995959 | | 1.06911 | | 1.02112 | | 1.04375 | | IRF3_1.mo |
| 1.18548 | 1.00816 | | 1.04149 | | 1.12605 | | 1.07105 | | 1.13136 | | 1.07938 | | IRF4_2.mo |
| 1.15106 | 1.10716 | | 1.05721 | | 1.02286 | | 1.06011 | | 0.982929 | | 1.10299 | | IRF5_1.mo |
| NA | 1.11399 | | 1.09751 | | NA |  | 1.09709 | | 1.08667 | | 1.24643 | | IRF6_1.Inf |
| NA | 1.04183 | | NA |  | 1.07652 | | 1.08405 | | 1.01412 | | 1.05646 | | IRF7_1.mo |
| 1.00649 | 0.988472 | | 0.966484 | | 1.12611 | | 1.04249 | |  | 1.0689 | 1.10708 | | IRF8_1.mo |
| NA |  | 1.0598 | 1.04843 | | 1.11719 | | 1.15437 | | 1.03781 | | 1.16965 | | IRF9_1.mo |
| NA | 1.02699 | | 1.06225 | | 1.09001 | | 1.10079 | | 0.974497 | | 0.996542 | | JDP2_1.m |
| NA | 1.01275 | | 0.995749 | | 1.11487 | | 1.05059 | | 1.04328 | | 0.97025 | | JUN_1.mo |
| 0.94742 | 1.04286 | |  | 1.0448 | 1.04721 | | 1.07489 | | 1.00881 | | 0.956067 | | JUNB_1.m |
| NA | 1.02481 | | 0.987831 | |  | 1.1042 |  | 1.0438 | NA |  | 0.992108 | | JUND_1.m |
| NA |  | 1.0134 | NA |  | 0.983092 | | 1.06707 | | NA |  | 1.00247 | | KLF1_1.m |
| 1.09742 | 1.09295 | | 1.05905 | | 1.00187 | |  | 1.0158 | 1.05112 | | 1.04001 | | KLF12_1.m |
| 1.14625 | 1.02183 | | 1.05792 | |  | 1.0962 | 1.05826 | | 0.938372 | | 1.11926 | | KLF12_2.m |
| 1.17582 | 1.09961 | | 1.04032 | | 1.06538 | | 1.10448 | | 1.24248 | | 1.15716 | | KLF13_1.m |
| 1.17582 | 1.09961 | | 1.04032 | | 1.06538 | | 1.10448 | | 1.24248 | | 1.15716 | | KLF13_1.m |
| NA | 0.990244 | | NA |  | 1.03818 | | 1.06111 | | NA |  | 1.14931 | | KLF14_1.m |
| 0.975881 | 1.04321 | |  | 1.008 | 1.00765 | | 1.08679 | | NA |  | 1.06555 | | KLF16_1.m |
| NA | 1.03496 | | NA |  | 0.983758 | | 1.04995 | | NA |  | 1.03971 | | KLF4_1.m |
| NA | 1.03496 | | NA |  | 0.983758 | | 1.04995 | | NA |  | 1.03971 | | KLF4_1.m |
| NA | 1.03496 | | NA |  | 0.983758 | | 1.04995 | | NA |  | 1.03971 | | KLF4_1.m |
| 0.908915 | 0.992075 | | 1.01035 | | 0.935149 | | 0.971988 | | 0.884325 | |  | 1.0011 | KLF5_1.m |
| 0.809306 | 0.971654 | | 1.04709 | | NA |  | 0.934216 | | 0.998112 | | 0.979722 | | KLF6_1.m |
| NA | 1.10981 | | NA |  | 0.972293 | | 1.11808 | | NA |  | 1.10391 | | KLF7_1.m |
| NA | NA |  | NA |  | 1.07914 | | NA |  | NA |  | NA |  | LBX1_1.m |
| NA | 1.03733 | | NA |  | 1.04003 | | 1.11328 | | 1.04574 | | 1.02663 | | LEF1_1.m |

| NA |  | NA | NA |  | 1.0303 | NA |  | NA |  | NA |  | LHX2_1.m |
| --- | --- | --- | --- | --- | --- | --- | --- | --- | --- | --- | --- | --- |
| NA |  | NA | NA | NA |  | NA |  | NA |  | NA |  | LHX6_1.m |
| NA |  | NA | NA | NA |  | NA |  | NA |  | NA |  | LMX1A_1. |
| NA |  | NA | NA | NA |  | NA |  | NA |  | NA |  | LMX1B_1. |
| 1.03889 | | 0.928328 | 0.996016 | 0.950508 | | 0.920497 | | 1.07165 | | 0.96543 | | LYL1_1.In |
| 1.03376 | | 1.03808 | 1.03431 | 1.12705 | | 0.996836 | | 0.992582 | | 0.996718 | | MAF_1.mo |
| 0.980424 | | 1.01329 | 0.996085 | 0.936727 | | 1.01454 | | 0.968075 | | 1.01664 | | MAFA_1.m |
| 1.10917 | | 0.992235 | 1.07318 |  | 1.0919 | 0.989866 | | 1.02731 | | 1.03577 | | MAFB_1.m |
| 0.902027 | | 0.97459 | 0.974876 | NA |  | 0.971433 | | 0.993175 | | 0.979976 | | MAFF_2.m |
| 1.05852 | | 0.958404 | 0.999299 | 1.05996 | | 1.00135 | | 1.02019 | | 1.02978 | | MAFG_1.m |
|  | 1.131 | 1.21666 | 1.04507 |  | 1.1062 | 1.12596 | | 1.06751 | | 1.27125 | | MAX_2.mo |
| 0.933499 | | 1.12883 | 1.16469 | 1.09089 | | 1.04448 | |  | 1.1765 | NA |  | MBD2_1.m |
| 1.15409 | | 1.07836 | 1.12183 | 1.35723 | | 1.09169 | | 1.30222 | | NA |  | MECP2_1. |
| 1.00661 | | 0.985411 | 0.97788 | 0.992767 | | 0.997455 | | 0.994248 | | 0.997537 | | MEIS2_1. |
| NA |  | NA | NA | 1.06054 | | NA |  | NA |  | NA |  | MEOX1_1. |
| NA |  | NA | NA | NA |  | NA |  | NA |  | NA |  | MEOX2_1. |
| 1.03802 | | 1.19474 | 1.12055 | 1.15269 | | 1.10035 | | 1.16904 | | 1.26819 | | MLXIPL_1 |
| NA |  | NA | NA | NA |  | NA |  | NA |  | NA |  | MNX1_1.m |
| 0.946047 | | 0.942045 | 0.984756 | 1.05301 | | 0.94449 | | 1.09116 | | 0.969507 | | MSC_1.mo |
| NA |  | NA | NA | 0.96376 | | NA |  | NA |  | NA |  | MSX1_1.m |
| NA |  | NA | NA | 0.96376 | | NA |  | NA |  | NA |  | MSX1_1.m |
| 1.31297 | | 1.24189 | 1.12559 | 1.12303 | | 1.16195 | | 1.05426 | |  | 1.1832 | MTF1_1.m |
| 1.23374 | | 1.18039 | 1.08866 |  | 1.1284 | 1.14046 | | 1.02331 | | 1.28953 | | MXI1_1.m |
| 1.23374 | | 1.18039 | 1.08866 |  | 1.1284 | 1.14046 | | 1.02331 | | 1.28953 | | MXI1_1.m |
| 1.23374 | | 1.18039 | 1.08866 |  | 1.1284 | 1.14046 | | 1.02331 | | 1.28953 | | MXI1_1.m |
| 1.23374 | | 1.18039 | 1.08866 |  | 1.1284 | 1.14046 | | 1.02331 | | 1.28953 | | MXI1_1.m |
| 1.12098 | | 1.10812 | 1.10171 | 1.08785 | |  | 1.1213 | 1.02447 | | 1.04846 | | MYB_1.mo |
| 1.12098 | | 1.10812 | 1.10171 | 1.08785 | |  | 1.1213 | 1.02447 | | 1.04846 | | MYB_1.mo |
| 1.12098 | | 1.10812 | 1.10171 | 1.08785 | |  | 1.1213 | 1.02447 | | 1.04846 | | MYB_1.mo |
| 1.12098 | | 1.10812 | 1.10171 | 1.08785 | |  | 1.1213 | 1.02447 | | 1.04846 | | MYB_1.mo |
| 1.10189 | | 1.10959 | 1.03377 | 1.24621 | | 1.17656 | |  | 1.1208 | 1.17574 | | MYBL1_1. |
| 1.10189 | | 1.10959 | 1.03377 | 1.24621 | | 1.17656 | |  | 1.1208 | 1.17574 | | MYBL1_1. |
| 0.815631 | | 1.05831 | 1.09212 | 1.11238 | | 1.01951 | | 1.20629 | |  | 1.1303 | MYBL1_2. |
| 0.815631 | | 1.05831 | 1.09212 | 1.11238 | | 1.01951 | | 1.20629 | |  | 1.1303 | MYBL1_2. |
| 1.10287 | | 1.29075 | 1.09157 | 1.18414 | | 1.24867 | | 1.10862 | | 1.41688 | | MYCN_1.m |
| 1.01491 | | 0.906428 | 0.952595 | 1.04217 | | 0.923474 | | 1.03074 | | 0.94933 | | MYF5_1.m |
| 1.01491 | | 0.906428 | 0.952595 | 1.04217 | | 0.923474 | | 1.03074 | | 0.94933 | | MYF5_1.m |
| 1.01085 | | 0.927105 | 0.991929 | 0.976728 | | 0.951841 | | 1.01283 | |  | 0.99 | MYF6_1.m |
| 1.01085 | | 0.927105 | 0.991929 | 0.976728 | | 0.951841 | | 1.01283 | |  | 0.99 | MYF6_1.m |

| 1.12714 | 0.977733 | 0.990584 | | 1.08382 | | 0.976781 | 0.989265 | | 0.981172 | | MYOD1_1 |
| --- | --- | --- | --- | --- | --- | --- | --- | --- | --- | --- | --- |
| 0.978539 | 0.927245 | 0.967721 | | 1.04406 | | 0.992205 | 1.07725 | | 0.975657 | | MYOG_1. |
| 0.992492 | 1.00913 | 1.01676 | | 1.00744 | | 0.980149 | 0.946185 | | 0.989987 | | NEUROD1 |
| 0.975754 | 1.01192 | 1.00592 | | 1.06426 | | 0.985381 | NA |  | 0.968527 | | NEUROD4 |
| 0.989655 | 1.08474 |  | 1.0189 | 1.01859 | | 1.07549 | 1.00377 | | 1.00179 | | NFE2_1.m |
| NA | 1.04197 | 1.06528 | |  | 0.9948 | 1.09372 | 1.00693 | | 0.997991 | | NFE2_2.m |
| 0.953664 | 1.07954 | 1.01613 | | 1.02299 | | 1.05932 | 1.04658 | | 0.992117 | | NFE2L2_1 |
| 1.06964 | 0.995257 | 1.00028 | | 0.979458 | | 1.03042 |  | 1.0523 | 1.03698 | | NFIA_1.mo |
| 1.05396 | 1.02853 | 1.00027 | | 0.96467 | | 1.02845 | 0.991398 | | 1.02112 | | NFIB_1.m |
| NA | NA | 1.01866 | | 1.00124 | | NA | 1.02634 | | 0.992877 | | NFIC_1.m |
| 0.968333 | 1.00759 | 0.972356 | | 0.970455 | | 1.01443 | 0.980669 | | 1.01149 | | NFIX_1.mo |
| 1.00095 | 0.98102 | 1.02351 | | 1.00717 | | 1.00862 | 1.02367 | | 0.993008 | | NFIX_2.mo |
| 0.898638 | 0.961344 | NA |  | NA |  | NA | NA |  | 1.02766 | | NFKB1_1. |
| 1.06254 | 0.966617 | 0.965333 | | 0.981804 | | 1.03771 | 1.00407 | |  | 1.003 | NFKB1_2. |
| NA | 1.06048 | NA |  | NA |  | NA | NA |  | 1.05172 | | NFKB2_1. |
| NA | NA | NA |  | NA |  | NA | 1.00947 | | NA |  | NKX2_1_1 |
| NA | NA | NA |  | NA |  | NA |  | 1.0378 | NA |  | NKX2_2_1 |
| NA | 0.996795 | NA |  | 0.953087 | | 0.954708 | 0.967907 | | 1.01025 | | NKX2_3_1 |
| NA | NA | NA |  | NA |  | NA | 1.04798 | | NA |  | NKX2_4_2 |
| NA | NA | NA |  | NA |  | NA | 0.96954 | | NA |  | NKX2_5_1 |
| NA | NA | NA |  | NA |  | NA |  | 1.0172 | NA |  | NKX2_5_3 |
| NA | NA | NA |  | NA |  | NA | 0.999812 | | NA |  | NKX2_6_1 |
| NA | 0.975367 | NA |  | NA |  | NA | 0.963293 | | 1.00722 | | NKX2_8_1 |
| NA | 0.985761 | NA |  |  | 1.0229 | 1.03982 | 1.08593 | | 0.985632 | | NKX3_1_2 |
| 0.949719 | 0.985841 | 1.00455 | | 1.01866 | | 1.02694 |  | 1.0604 | 0.975315 | | NKX3_2_1 |
| NA | NA | NA |  | NA |  | NA | NA |  | NA |  | NKX6_2_1 |
| NA | NA | NA |  | NA |  | NA | NA |  | NA |  | NKX6_3_1 |
| NA | NA | NA |  | 0.919051 | | NA | NA |  | NA |  | NOBOX_1. |
| 1.14773 | 1.26715 | 1.06979 | | 1.11437 | | 1.17668 | 1.08866 | | 1.36557 | | NPAS2_1. |
| 0.968637 | 1.00264 | 1.06879 | | 0.978127 | | 1.06332 | 1.06188 | | 1.10192 | | NR0B1_1. |
| 0.968637 | 1.00264 | 1.06879 | | 0.978127 | | 1.06332 | 1.06188 | | 1.10192 | | NR0B1_1. |
| NA | NA | NA |  | 1.47143 | | NA |  | 0.8 | NA |  | NR1H2_1. |
| NA | 1.10551 | 1.10713 | | NA |  | NA | 0.974469 | | 1.08926 | | NR1H4_1. |
| NA | NA | NA |  | 1.02921 | | NA | 0.958475 | | 0.990935 | | NR2C1_1. |
| NA | 0.981575 | NA |  | 1.03301 | | NA | 1.02451 | | 1.02722 | | NR2C2_1. |
| 1.11541 | 1.00153 | 1.09166 | | 1.03848 | | 1.06671 | 1.01662 | | 1.04048 | | NR2C2_2. |
| NA | 1.02419 | 0.997438 | | 1.07522 | | NA | 0.956983 | | 1.00745 | | NR2F1_1. |
| NA | NA | NA |  | NA |  | NA | NA |  | 1.03206 | | NR2F1_2. |
| NA | 1.08499 | 1.04471 | | 1.06356 | | NA | 0.930185 | | 1.02006 | | NR2F2_2. |

| NA |  | 0.97512 | NA | 0.982759 | | NA |  | 0.979113 | | 1.03063 | | NR2F6_3. |
| --- | --- | --- | --- | --- | --- | --- | --- | --- | --- | --- | --- | --- |
| NA |  | NA | NA | 0.923837 | | NA |  | 0.978527 | | NA |  | NR4A1_1. |
| NA |  | NA | NA | 1.00819 | | NA |  | 0.948762 | | 0.995329 | | NR4A2_3. |
| NA |  | 1.00986 | NA | 1.00785 | | NA |  | 0.986812 | | 1.01587 | | NR4A3_1. |
| NA |  | NA | 0.98494 | 1.00709 | | NA |  | 1.03663 | | 1.01282 | | NR5A1_1. |
| 0.994579 | | 1.06175 | 1.03578 |  | 1.0879 | 1.03082 | | 1.02062 | | 1.01252 | | NRL_1.mo |
| NA |  | NA | NA | NA |  | NA |  | NA |  | NA |  | ONECUT1 |
| NA |  | NA | NA | NA |  | NA |  | NA |  | NA |  | ONECUT1 |
| NA |  | NA | NA | NA |  | NA |  | NA |  | NA |  | ONECUT1 |
| NA |  | NA | NA | NA |  | NA |  | NA |  | NA |  | OTP_1.mo |
| 0.863226 | | 0.996017 | 0.983439 | 1.02198 | | 0.891736 | | 1.06539 | | 0.990372 | | PAX1_1.m |
| 0.925543 | | 0.963174 | 1.04806 | 1.08079 | | 0.946387 | | 1.17519 | | 1.04915 | | PAX2_1.m |
| 0.966445 | | 1.13224 | 1.06924 | 1.07079 | | 1.05289 | | 1.14923 | | 1.08047 | | PAX2_3.m |
| NA |  | NA | NA | NA |  | NA |  | NA |  | NA |  | PAX4_5.m |
| 0.980545 | | 1.00029 | 1.11223 | NA |  | NA |  | 1.14482 | | 1.04962 | | PAX5_2.m |
| 1.00565 | | 1.03817 | 1.06458 | 1.08957 | | 1.00098 | | 1.06824 | |  | 1.0358 | PAX6_1.m |
|  | 1.058 | 1.02669 | 1.04684 | 1.07815 | |  | 1.0161 | 1.02997 | | 1.08351 | | PAX8_1.m |
| 0.958589 | | 1.01359 | 1.02333 | NA |  | NA |  | 1.04999 | | 0.977166 | | PAX8_2.m |
| NA |  | NA | NA | NA |  | NA |  | NA |  | NA |  | PDX1_2.m |
| 0.925586 | | 0.985019 | 0.944844 | NA |  | NA |  | NA |  | 1.06414 | | PLAGL1_1 |
| 1.02423 | | 1.03388 | 0.983788 | NA |  | 1.06148 | | 0.968762 | | 1.03523 | | PLAGL2_2 |
| 1.08766 | | 0.880037 | 0.932033 | 1.05144 | | 0.93106 | | 0.997965 | | 0.983077 | | PPARD_1. |
| NA |  | 0.984826 | 0.955154 | 1.01905 | | 0.906722 | |  | 0.974 | 0.958846 | | PPARG_1. |
| 1.05268 | | 1.05633 | 1.13787 | 1.06012 | |  | 1.0802 | 1.25813 | | NA |  | PRDM15_ |
| 0.954392 | | 1.04676 | 1.08513 | 0.962706 | | 0.982784 | | 0.955942 | | 1.04883 | | PTF1A_1. |
| NA |  | 1.01013 | 1.01654 | 0.976602 | | NA |  | 0.994298 | | 1.02886 | | RARA_1.m |
| 0.982388 | | 0.984769 | 1.03008 | 1.01882 | | 1.05134 | | 1.01858 | | 1.03576 | | RELA_1.m |
| 0.999411 | | 0.975026 | 1.01345 | NA |  | 0.972024 | | 1.04412 | | 1.00164 | | RELB_1.m |
| 0.924266 | | 1.03602 | 1.01581 | 0.995202 | | 1.06868 | | 1.04956 | | 1.09055 | | RFX1_1.m |
| 0.904707 | | 1.08246 | 1.04449 | 0.921137 | | 1.06025 | | 1.00845 | | 1.04791 | | RFX1_2.m |
| 1.22727 | | 0.893471 | 0.940252 | 1.14829 | | 1.03114 | | 1.02841 | | 1.05417 | | RFX2_3.m |
| 0.971347 | | 1.05697 | 1.14305 | 1.00396 | | 1.01669 | |  | 1.0767 | 1.03552 | | RFX3_1.m |
| 0.813084 | | 0.913687 | 0.887946 | 1.00432 | | 0.997033 | | 0.983766 | | 1.06246 | | RFX4_2.m |
| 0.926647 | | 1.04334 | 1.06193 | 1.06244 | | 1.07912 | | 1.00189 | | 1.04449 | | RFX5_1.m |
| 0.917526 | | 1.02154 | 1.04538 | 0.951673 | | 0.984802 | | 1.07367 | |  | 1.0271 | RFX5_2.m |
| NA |  | 0.984848 | 0.950216 | 0.967593 | | 1.09449 | |  | 1.0653 | 1.02531 | | RFX6_4.m |
| NA |  | NA | NA | NA |  | NA |  | NA |  | NA |  | RHOXF1_1 |
| NA |  | NA | NA | NA |  | NA |  | NA |  | NA |  | RHOXF1_1 |
| NA |  | NA | NA | NA |  | NA |  | NA |  | NA |  | RHOXF1_1 |

| 0.937267 | | 0.986937 | | 0.981465 | 1.00322 | | 1.00052 | | NA |  | 1.01569 | | RUNX1_1. |
| --- | --- | --- | --- | --- | --- | --- | --- | --- | --- | --- | --- | --- | --- |
| 0.938004 | | 0.996063 | | 0.951913 | 0.973086 | | 1.02264 | | NA |  | 1.02246 | | RUNX2_1. |
| 0.961593 | | 1.05632 | | 1.16111 | 1.01617 | | 1.12914 | | NA |  | 1.02782 | | RUNX3_1. |
| NA |  | 0.967235 | | 0.989888 | 1.03201 | | NA |  | 0.953567 | | 0.988678 | | RXRA_3.m |
| NA |  | NA |  | NA | 1.00149 | | NA |  | NA |  | 1.02791 | | RXRA_4.m |
| NA |  | NA |  | NA | 1.15625 | | NA |  | 0.834197 | | 1.11522 | | RXRB_1.m |
| NA |  | NA |  | NA | 1.11355 | | NA |  | 0.934866 | | 1.04395 | | RXRG_1.m |
| NA |  | 0.978073 | | 1.04776 | 1.13972 | |  | 1.0412 |  | 1.0525 | 1.04008 | | SIX1_1.mo |
| 1.09506 | | 1.00567 | | 1.10328 |  | 1.0853 | 1.03978 | | 0.927748 | | 1.01303 | | SMAD1_1. |
| 1.00853 | | 1.10238 | | 1.07656 | 1.00404 | | 1.05832 | | 1.03107 | | 1.05204 | | SMAD2_2. |
| 1.07996 | | 0.988841 | | 1.04421 | 1.00424 | | 0.993469 | | 0.955005 | | 0.990687 | | SMAD3_2. |
| 1.07996 | | 0.988841 | | 1.04421 | 1.00424 | | 0.993469 | | 0.955005 | | 0.990687 | | SMAD3_2. |
|  | 1.1951 |  | 1.0387 | 1.03651 | 1.11745 | | 1.03681 | | 1.07787 | | 1.05034 | | SMAD4_1. |
| NA |  | 1.02761 | | 1.00581 | 1.02898 | | 1.01403 | | 1.01993 | | 0.964696 | | SMARCC1 |
| NA |  | 1.02761 | | 1.00581 | 1.02898 | | 1.01403 | | 1.01993 | | 0.964696 | | SMARCC1 |
| NA |  | 0.954764 | | NA | 0.957645 | | 0.940533 | | 0.916635 | | NA |  | SOX10_5. |
| NA |  | NA |  | NA |  | 1.1019 | 0.981522 | | 1.00306 | | NA |  | SOX3_1.m |
| NA |  | 0.97752 | | NA | 1.01073 | | 0.936271 | | 0.995852 | | NA |  | SOX6_1.m |
| 0.996804 | |  | 1.0164 | NA | 0.928505 | | 1.04769 | | NA |  | 1.08919 | | SP3_1.mo |
| 1.06042 | | 1.03394 | | 1.02233 | 1.14009 | | 1.09899 | | 1.05365 | | 1.03863 | | SPDEF_1. |
| 1.14964 | | 1.06784 | | 1.06048 | 1.09236 | |  | 1.0303 | 1.10692 | | 1.05838 | | SPI1_1.mo |
|  | 1.0627 | 0.958177 | | 0.977861 | 0.913872 | | 0.956406 | | 0.947649 | | 0.980392 | | SPI1_2.mo |
| 1.02844 | |  | 1.0147 | 0.994013 | 0.965522 | | 1.02769 | | 0.936395 | | 1.00989 | | SPIB_1.mo |
| 1.12602 | | 0.983492 | | 1.03448 | 1.05427 | | 0.994527 | | 0.958647 | | 1.03904 | | SPIB_2.mo |
|  | 1.0819 | 1.02605 | | 1.02037 | 0.957861 | | 1.02572 | | 1.03493 | | 1.02245 | | SPIC_1.mo |
| 1.00174 | | 1.03967 | | 1.00707 | 1.01999 | | 1.05386 | | 1.00628 | | 0.996819 | | STAT1_1. |
| 1.01666 | | 1.04669 | | 0.924933 |  | 1.0118 | 1.06039 | | 0.994538 | | 1.00574 | | STAT2_1. |
|  | 1 |  | 1.0461 | 1.02977 | 1.06053 | | 1.10044 | | 0.95572 | | 1.00688 | | STAT3_1. |
| 0.871111 | | 0.957576 | | 0.902597 | 0.94761 | | 0.907192 | | 1.02494 | | 1.02601 | | TAL1_1.m |
| 1.06871 | | 1.05549 | | 1.09963 |  | 1.1334 | 1.00002 | | 1.02368 | | 1.01636 | | TCF12_1. |
| 0.928162 | | 0.911032 | | 0.949913 | 0.991657 | | 0.93171 | | 1.01129 | | 0.939889 | | TCF21_1. |
|  | 1.0085 | 0.918351 | | NA | 0.960814 | | 0.920394 | | NA |  | 1.02425 | | TCF3_1.m |
| NA |  |  | 1.0059 | NA | 0.959633 | | 0.964792 | |  | 1.0477 | 0.951122 | | TCF7_1.m |
| NA |  | NA |  | NA | 0.920716 | | NA |  | 1.00177 | | NA |  | TEAD1_1. |
| NA |  | NA |  | NA | NA |  | NA |  | 0.98519 | | NA |  | TEAD3_2. |
| 1.12421 | | 1.10437 | | 1.07126 | NA |  | NA |  | 1.05427 | | 1.03108 | | TFAP2A_1 |
| 0.993707 | |  | 1.025 | 1.02871 | 1.06182 | | 0.995496 | | 1.04452 | |  | 1.0397 | TFAP2A_2 |
| 0.996639 | | 1.00997 | | 1.09555 | NA |  | NA |  | 1.03716 | | 1.06392 | | TFAP2A_3 |
| NA |  | NA |  | NA | NA |  | NA |  | NA |  | NA |  | TFAP2D_1 |

| 1.07331 | | 1.04252 | |  | 1.041 | NA |  | 1.01806 | | 1.05911 | |  | 1.039 | TFAP2E_1 |
| --- | --- | --- | --- | --- | --- | --- | --- | --- | --- | --- | --- | --- | --- | --- |
| 0.844492 | | 0.907915 | | 0.914954 | | 0.947419 | | 0.888717 | | 0.929934 | | 0.961309 | | TFAP4_2. |
| 1.14814 | | 1.21757 | | 1.11151 | | 1.13227 | | 1.12799 | | 1.06294 | | 1.25712 | | TFE3_1.m |
|  | 1.1345 | 1.18478 | | 1.05343 | | 0.98639 | | 1.10854 | | 1.06215 | | 1.25371 | | TFEB_1.m |
|  | 1.1 | 1.13483 | | 1.07423 | | 1.00628 | | 1.09004 | | 1.07224 | | 1.14694 | | TFEC_1.m |
| 1.26522 | | 0.94165 | | 1.09124 | | 0.935403 | | 0.988372 | | 1.13744 | | 0.98908 | | TGIF2LX_ |
| NA |  | 1.00208 | | 0.979412 | | 0.974192 | | NA |  |  | 1.0663 | 1.02881 | | TLX1_2.m |
| 1.14994 | | 1.13873 | | 1.06569 | | 1.01741 | | 1.08105 | | 1.08743 | | 1.20651 | | USF1_1.m |
| 1.13594 | | 1.13393 | | 1.06889 | | 1.04618 | | 1.11669 | | 1.06747 | | 1.17483 | | USF2_1.m |
| NA |  | NA |  | NA |  | NA |  | NA |  | NA |  | NA |  | VAX1_1.m |
| NA |  | NA |  | NA |  | NA |  | NA |  | NA |  | NA |  | VAX2_1.m |
| NA |  | NA |  | NA |  | NA |  | NA |  | NA |  | NA |  | VSX1_1.m |
| 1.11923 | | 1.13131 | |  | 1.1907 | 1.07938 | | 1.13375 | | 1.23963 | | 1.04029 | | ZBTB1_1.I |
| 1.02369 | | 1.21895 | | 1.10571 | | 1.09896 | | 1.18574 | | 1.10442 | | 1.19372 | | ZBTB14_1 |
| 0.97727 | | 0.993425 | | 0.923775 | | 1.01283 | | 0.935445 | | NA |  | 0.967431 | | ZBTB18_2 |
| 1.00846 | | 1.17904 | | 0.992337 | | NA |  | 1.16724 | | 1.13799 | | 1.15555 | | ZBTB33_1 |
| 0.997904 | | 0.941395 | | 0.879033 | | 0.99472 | | 0.933267 | | NA |  | 0.96206 | | ZBTB42_1 |
| 1.00827 | |  | 1.0592 | 0.987808 | | NA |  | 1.04115 | | NA |  | 1.11523 | | ZBTB7A_2 |
| 1.03342 | | 1.02056 | |  | 1.0183 | NA |  | NA |  | 0.996303 | | 1.04525 | | ZFAT_2.m |
| NA |  | NA |  | NA |  | NA |  | NA |  | NA |  | NA |  | ZFHX2_1. |
| 1.03852 | | 1.10159 | | 1.13556 | | 1.13591 | | 1.03741 | | 1.15342 | | 1.13045 | | ZFP57_1.m |
| NA |  | 1.04438 | | 0.966952 | | 1.17468 | | 0.961669 | | 1.07425 | | 1.00302 | | ZFP64_1.m |
| 1.05506 | | NA |  | 1.02499 | | NA |  | NA |  | NA |  | 1.05707 | | ZFX_1.mo |
| 1.04882 | | NA |  | 1.04005 | | NA |  | NA |  | NA |  | 1.07696 | | ZFY_1.mo |
| 0.876091 | | 0.991357 | | 1.03396 | | NA |  | 0.989839 | | 1.08812 | | 1.04377 | | ZIC4_1.mo |
| 0.94865 | | 1.02106 | | 0.99298 | | NA |  | 1.02577 | | 0.996556 | | 1.02084 | | ZIC5_1.mo |
| 0.836942 | | 0.960156 | |  | 1.0705 | NA |  | 0.989046 | | 1.03507 | | 1.03376 | | ZIC5_2.mo |
| 1.05926 | | 1.09121 | | 1.04036 | | NA |  | 1.12169 | | 1.01229 | |  | 1.0652 | ZIC5_3.mo |
| 0.93083 | | 1.06041 | | 0.995381 | | NA |  | 1.11444 | | 1.07637 | | 1.03111 | | ZNF100_1 |
| 1.04647 | | 1.17873 | | 1.08288 | | 1.14749 | | 1.13603 | | 1.13015 | | 1.19268 | | ZNF142_3 |
| 1.03565 | | 0.955775 | | 1.01234 | |  | 1.0135 | 0.960779 | | 0.977079 | | 1.10546 | | ZNF19_1. |
| 1.11628 | |  | 1.1028 | 1.09399 | | 1.20167 | | 1.24199 | | 1.13243 | | 1.12576 | | ZNF2_1.m |
| 1.08585 | | 0.977405 | |  | 1.1126 | 1.08885 | | 1.03105 | | 1.14733 | | 1.05958 | | ZNF208_1 |
| 1.00202 | | 0.923467 | | 1.00857 | | 1.00583 | | 0.991726 | | 0.994635 | |  | 1.0805 | ZNF208_2 |
| 1.10145 | | 1.10996 | |  | 1.1644 | NA |  | 1.06639 | | 1.06758 | | 1.11266 | | ZNF292_1 |
| 0.985374 | | 1.02167 | | 0.993859 | | 1.02933 | | 1.03497 | | 0.959318 | | 0.988193 | | ZNF296_1 |
| 1.00623 | | 1.00754 | | 1.00154 | | 0.991803 | |  | 1.0445 | 0.923598 | | 0.982005 | | ZNF296_2 |
| 1.04847 | | 1.05822 | | 0.942418 | | 1.11762 | | 1.03293 | | 1.05287 | |  | 1.1258 | ZNF329_1 |
|  | 1.0403 | 1.14905 | | 1.08842 | | 1.10348 | | 1.11083 | | 1.05105 | | 1.04937 | | ZNF335_1 |

1.1082	1.08668 0.987443	1.03415	1.14002	1.04019	1.05217 ZNF343_1

1.10215	1.03765 0.971241	1.04028	1.03853 0.915205	1.02609 ZNF350_1

1.25529	1.17707	1.07983	1.14517	1.09112	1.0831	1.16965 ZNF433_2

0.995633	1.02411	1.06044 NA	0.963756 0.928478	1.0198 ZNF441_1

1.0893	1.12505	1.17827	1.16016	1.11955	1.15728	1.12723 ZNF445_2

NA	0.979382 NA	1.03098 0.965498 0.985136 0.994187 ZNF445_3

1.00312 0.974024	1.03208 0.996203 0.961884 0.983308	1.00102 ZNF516_2

NA	0.967667	1.02922	1.00567 0.986284	1.03395	0.97994 ZNF536_2

1.01947	1.05288	1.11884	1.09873	1.11856	1.00686	1.1433 ZNF547_1

0.971491 0.877311	1.02994	1.07591	1.00857	1.02974 0.998254 ZNF548_1

1.03905 0.942618 0.991727	1.03227 0.990296	1.00872 0.992335 ZNF550_1

NA	NA	NA	1.00619 NA	NA	NA	ZNF560_3

1.08769	1.02032 0.980669 0.992757	1.03153	1.02721	1.02947 ZNF566_1

1.08994	1.01819	1.08851 NA	1.00987	1.08679	1.09231 ZNF579_1

1.10215	1.11444	1.04974 NA	1.08206 0.959224	1.19003 ZNF579_3

1.1	1.04103	1.08672	1.03876	1.03855	1.04043	1.03753 ZNF594_2

1.06348	1.06715	1.13513 NA	1.00071	1.01165	1.03218 ZNF594_3

1.03491	1.03228	1.15992 0.963748 0.989539 0.966218 0.976091 ZNF594_4

NA	NA	NA	NA	1.03495 0.988395	1.02846 ZNF597_2

0.945055	1.06828	1.10401 NA	1.04287	1.04746	0.99215 ZNF606_1

0.98579	1.03024 0.986503	1.06845	1.03723	1.09022	1.0554 ZNF676_1

NA	0.921508 0.982456 NA	0.973635 NA	1.02457 ZNF681_1

0.928495	1.01224 0.960022 0.969809 0.976274 0.998139	1.01279 ZNF699_1

0.938426	1.00645 0.946205 NA	1.00299 0.980794 0.996982 ZNF705D_ NA	1.01073	1.02562	1.00676 0.982878 0.980591 0.997374 ZNF716_1

1.13573	1.19517	1.02084	1.0995	1.11695	1.06416	1.07715 ZNF721_2

1.02475	1.08498	1.01607	1.05651	1.05876	1.06908	0.99879 ZNF732_1

0.937729	0.98122 0.951288 0.939517	1.02282 0.980253	1.07066 ZNF746_1

0.987476	1.02034	1.06305	1.03668 0.955887	1.03264 0.969959 ZNF763_2

0.970588 0.894267	1.04755	1.12962 0.969722	1.04456 0.962126 ZNF768_1

1.0434	1.00677	1.00005 0.987956	1.00036 0.949998	1.01956 ZNF770_2

1.11845	1.20645	1.10992	1.08202	1.18118	1.20221	1.21554 ZNF777_2

0.907295 0.962261 0.969893 NA	0.991481 0.956853	1.04694 ZNF781_1

1.15045	1.03873	1.04081	1.02519	1.05102	1.01742	1.10057 ZNF80_2. NA	NA	NA	NA	NA	1.06803 NA	ZNF827_1

| 1.05467 | 1.09453 | 1.13258 | 1.12985 | 1.0727 | 1.11953 | 1.04076 ZNF836_2 |
| --- | --- | --- | --- | --- | --- | --- |
| 0.988399 | 0.992 | 1.06754 | 1.02065 | 0.97852 | 1.13228 | 1.06397 ZNF91_3. |
| 1.02812 | 1.07023 | 1.05191 | NA | 1.0576 | 1.05933 | 1.05957 ZSCAN25_ |

geneid	type	adipocyte. adult_end alternative band_form b_cell..cee b_cell_fro

AHR	Direct	0.105687	0.10579 0.120159	0.11236 0.102559 0.117775

ALX3	Direct	NA	NA	NA	NA	NA	NA

ARNT	Direct	0.10952	0.11197 0.120643 0.113429 0.118218 0.114473

ARNT	Direct	0.100669 0.110393 0.134875 0.114542 0.115342 0.113668

ARNTL	Direct	NA	0.116168 0.115341 0.106778 0.121396 0.105877

ASCL2	Direct	0.118315 0.106515 0.129819 0.125725 0.102598 0.132026

ATF3	Direct	0.105961 0.120025 0.126375 0.116448 0.121658 0.114435

ATF6	Direct	0.097049 0.114488 0.113267 0.104059 0.113237 0.107313

CREBZF	Inferred	0.097049 0.114488 0.113267 0.104059 0.113237 0.107313

ATF7	Direct	0.12766 0.110514 0.124239 0.122171 0.101438 NA

ATOH1	Direct	0.12256	0.1064 0.119479 0.117289 0.099626 0.121148

ATOH7	Direct	0.101373 0.100819 0.109527 0.107556 0.099859 0.115487

BACH1	Direct	0.103662 0.116647 0.127945 0.114972 0.098953 0.102256

BACH2	Direct	0.109132 0.120126 0.129617 0.113579 0.102199 0.105144

BARHL2	Direct	NA	0.098757 NA	NA	0.105725 NA BARX1	Direct	NA	NA	NA	NA	NA	NA BATF	Direct	0.099948 0.102738 NA	NA	0.102478 NA BATF2	Inferred	0.099948 0.102738 NA	NA	0.102478 NA

BCL11A	Direct	0.102998 0.116614 0.136302 0.134193 0.125083	0.15236

BHLHE40 Direct	0.1028 0.117948 0.120961 0.115197	0.12543 0.116616

BSX	Direct	NA	NA	NA	NA	NA	NA

CBFB	Direct	0.098483 0.097295 0.134332 0.132406 0.108813 0.139859

CDX1	Direct	NA	NA	NA	NA	NA	NA CDX2	Direct	NA	NA	NA	NA	NA	NA CEBPA	Direct	0.110412 NA	0.105808 0.118663 0.091967 NA CEBPB	Direct	0.123849 NA	0.127987	0.14659 NA	NA CEBPD	Direct	0.125033 NA	0.129527 0.147653 NA	NA CEBPE	Direct	0.116866 NA	0.12029 0.138089 NA	NA

CLOCK	Direct	0.11127	0.11849 0.115051 0.113112	0.12352 0.115721

SOHLH1	Inferred	0.11127	0.11849 0.115051 0.113112	0.12352 0.115721

CREB3L2 Direct	0.123681 0.141234	0.13902 0.131655	0.14371 0.132353

CREB3L3 Direct	0.124861 0.144152	0.13888 0.133674 0.148682 0.134745

CREB5	Direct	0.121359 0.104202 0.113591 0.116676	0.09851 NA DBP	Direct	0.115679 NA	0.109249 0.119306 NA	NA DLX1	Direct	NA	NA	NA	NA	NA	NA DLX2	Direct	NA	NA	NA	NA	NA	NA DLX6	Direct	NA	NA	NA	NA	NA	NA

E2F1	Direct	0.097602 0.102463 0.119025 0.106984 NA	0.111386

| EBF1 | Direct | 0.131508 | 0.105657 | 0.100374 | 0.099616 | 0.098357 | 0.121162 | |
| --- | --- | --- | --- | --- | --- | --- | --- | --- |
| EBF2 | Inferred | 0.131508 | 0.105657 | 0.100374 | 0.099616 | 0.098357 | 0.121162 | |
| EBF4 | Inferred | 0.131508 | 0.105657 | 0.100374 | 0.099616 | 0.098357 | 0.121162 | |
| EGR2 | Direct | 0.098707 | 0.103204 | 0.105146 | 0.095954 | 0.103324 | 0.113626 | |
| EGR4 | Direct | 0.096799 | 0.101706 | 0.106886 | 0.097556 | 0.099029 | 0.111845 | |
| EHF | Direct | 0.103589 | 0.137873 | 0.155122 | 0.162578 | 0.113475 | 0.170515 | |
| EHF | Direct | 0.11454 | 0.143892 | 0.178946 | 0.182188 | 0.120625 | 0.180435 | |
| ELF1 | Direct | 0.118029 | 0.150503 | 0.198991 | 0.200692 | 0.127018 | 0.201144 | |
| ELF2 | Direct | 0.115555 | 0.148551 | 0.17013 | 0.173834 | 0.122705 | 0.186636 | |
| ELF3 | Direct | 0.114827 | 0.139326 | 0.173122 | 0.175947 | 0.120511 | 0.172535 | |
| ELF4 | Direct | 0.113938 | 0.137537 | 0.186238 | 0.186575 | 0.120511 | 0.184273 | |
| ELF5 | Direct | 0.113719 | 0.143203 | 0.188292 | 0.193769 | 0.127409 | 0.193133 | |
| ELK1 | Direct | 0.121414 | 0.145033 | 0.183582 | 0.18067 | 0.115129 | 0.172951 | |
| ELK3 | Direct | 0.120315 | 0.148281 | 0.187389 | 0.186457 | 0.118666 | 0.180829 | |
| ELK4 | Direct | 0.122568 | 0.153224 | 0.196278 | 0.196426 | 0.120426 | 0.19067 | |
| EN1 | Direct | NA | NA | NA | NA | NA | NA |  |
| ENO1 | Direct | 0.10038 | 0.11474 | 0.109919 | 0.10566 | 0.118683 | 0.105914 | |
| ERF | Direct | 0.121266 | 0.151196 | 0.194708 | 0.193994 | 0.123262 | 0.193643 | |
| ERG | Direct | 0.122202 | 0.159652 | 0.204333 | 0.201683 | 0.127287 | 0.200768 | |
| ERG | Direct | 0.120921 | 0.146418 | 0.164474 | 0.156981 | 0.113037 | 0.148455 | |
| ERG | Direct | 0.116853 | 0.161179 | 0.203757 | 0.210327 | 0.129659 | 0.215591 | |
| ESRRG | Direct | 0.093539 | 0.103212 | NA | NA | 0.093915 | NA |  |
| ESX1 | Direct | NA | NA | NA | NA | NA | NA |  |
| ETS1 | Direct | 0.119443 | 0.157987 | 0.204936 | 0.205609 | 0.126733 | 0.204956 | |
| ETS1 | Direct | 0.117712 | 0.140092 | 0.15376 | 0.14923 | 0.108988 | 0.145869 | |
| ETS2 | Direct | 0.109335 | 0.134302 | 0.165663 | 0.168961 | 0.113888 | 0.169608 | |
| ETV1 | Direct | 0.121612 | 0.155316 | 0.205326 | 0.204267 | 0.124137 | 0.203158 | |
| ETV2 | Direct | 0.119662 | 0.15224 | 0.18824 | 0.188987 | 0.126787 | 0.188559 | |
| ETV3 | Direct | 0.113549 | 0.126512 | 0.15929 | 0.154487 | 0.108038 | 0.150808 | |
| ETV3L | Inferred | 0.113549 | 0.126512 | 0.15929 | 0.154487 | 0.108038 | 0.150808 | |
| ETV4 | Direct | 0.119349 | 0.157088 | 0.205582 | 0.207263 | 0.126513 | 0.210333 | |
| ETV5 | Direct | 0.11351 | 0.136316 | 0.168427 | 0.167182 | 0.109824 | 0.166352 | |
| ETV6 | Direct | 0.101952 | 0.121348 | 0.162585 | 0.164178 | 0.113542 | 0.163268 | |
| ETV7 | Direct | 0.110711 | 0.139632 | 0.156572 | 0.160854 | 0.119533 |  | 0.1653 |
| EVX2 | Direct | NA | NA | NA | NA | NA | NA |  |
| FERD3L | Direct | 0.102949 | 0.103626 | 0.108963 | 0.110838 | 0.101685 | 0.114561 | |
| FEV | Direct | 0.118165 | 0.158577 | 0.19687 | 0.201409 | 0.129109 | 0.200858 | |
| FLI1 | Direct | 0.119703 | 0.160425 | 0.211265 | 0.212426 | 0.128355 | 0.215025 | |
| FLI1 | Direct | 0.122951 | 0.151274 | 0.166105 | 0.159323 | 0.113891 | 0.150716 | |

| FOS | Direct | 0.10974 | | 0.12234 | 0.125423 | 0.107168 | 0.104416 | 0.098566 |
| --- | --- | --- | --- | --- | --- | --- | --- | --- |
| FOSB | Direct | 0.109676 | | 0.11846 | 0.118677 | 0.106413 | 0.10405 | 0.101052 |
| FOSL1 | Direct | 0.110679 | | 0.123185 | 0.128786 | 0.113709 | 0.105458 | 0.104237 |
| FOSL2 | Direct | 0.100212 | | 0.118186 | 0.11961 | 0.101141 | 0.10035 | NA |
| FOXA1 | Direct | 0.091544 | | NA | NA | NA | NA | NA |
| FOXA2 | Direct | NA |  | NA | NA | NA | NA | NA |
| FOXC1 | Direct | NA |  | 0.092007 | NA | NA | NA | NA |
| FOXD1 | Direct | 0.092877 | | 0.09193 | NA | NA | NA | NA |
| FOXD2 | Direct |  | 0.0982 | 0.092531 | NA | NA | 0.089217 | NA |
| FOXD4 | Direct | 0.098261 | | 0.093052 | NA | NA | 0.089547 | NA |
| FOXF1 | Direct | NA |  | NA | NA | NA | NA | NA |
| FOXN1 | Inferred | NA |  | NA | NA | NA | NA | NA |
| FOXN4 | Inferred | NA |  | NA | NA | NA | NA | NA |
| FOXF2 | Direct | NA |  | NA | NA | NA | NA | NA |
| FOXG1 | Direct | 0.098595 | | 0.093793 | NA | NA | 0.088872 | NA |
| FOXK1 | Direct | 0.100775 | | 0.09683 | NA | NA | 0.091555 | NA |
| FOXO3 | Direct | 0.094709 | | 0.094313 | NA | NA | 0.091209 | NA |
| FOXP1 | Direct | 0.095708 | | 0.09244 | NA | NA | 0.091201 | NA |
| FOXP2 | Direct | 0.102348 | | 0.096927 | NA | NA | 0.092231 | 0.095479 |
| FOXP3 | Direct | 0.098759 | | 0.092741 | NA | NA | 0.089043 | NA |
| FOXQ1 | Direct | NA |  | NA | NA | NA | NA | NA |
| GABPA | Direct | 0.121724 | | 0.148266 | 0.201138 | 0.19838 | 0.120175 | 0.197065 |
| GATA1 | Direct | 0.100069 | | 0.100247 | NA | 0.093924 | 0.096003 | 0.098214 |
| GATAD2B | Inferred | 0.100069 | | 0.100247 | NA | 0.093924 | 0.096003 | 0.098214 |
| GATA2 | Direct | 0.089339 | | 0.091346 | NA | NA | 0.091996 | NA |
| ZGLP1 | Inferred | 0.089339 | | 0.091346 | NA | NA | 0.091996 | NA |
| GATA3 | Direct | 0.093299 | | NA | NA | NA | 0.088594 | NA |
| GATA4 | Direct | 0.096809 | | 0.097803 | NA | 0.093477 | 0.092233 | 0.094197 |
| GATA5 | Direct | 0.092199 | | NA | NA | NA | 0.089413 | NA |
| GATA6 | Direct | 0.091684 | | NA | NA | NA | NA | NA |
| MTA2 | Inferred | 0.091684 | | NA | NA | NA | NA | NA |
| RERE | Inferred | 0.091684 | | NA | NA | NA | NA | NA |
| GBX1 | Direct | NA |  | NA | NA | NA | NA | NA |
| GBX2 | Direct | NA |  | NA | NA | NA | NA | NA |
| GSX1 | Direct | NA |  | NA | NA | NA | NA | NA |
| GSX2 | Direct | NA |  | NA | NA | NA | NA | NA |
| HAND1 | Direct | 0.112104 | | 0.102606 | 0.101373 | 0.101992 | 0.093947 | 0.100136 |
| HES1 | Direct | 0.102412 | | 0.108292 | 0.116918 | 0.111939 | 0.103792 | 0.107811 |
| HES6 | Inferred | 0.102412 | | 0.108292 | 0.116918 | 0.111939 | 0.103792 | 0.107811 |

| HES4 | Direct | 0.100497 | | 0.117236 | 0.133796 | 0.118061 | 0.114393 | 0.112607 |
| --- | --- | --- | --- | --- | --- | --- | --- | --- |
| HES7 | Direct | NA |  | 0.106013 | 0.104888 | 0.10091 | 0.111445 | NA |
| HEYL | Direct | 0.101619 | | 0.111629 | 0.125267 | 0.115979 | 0.110327 | 0.120146 |
| HIC1 | Direct |  | 0.094 | 0.099815 | 0.104152 | 0.10466 | 0.092954 | 0.10038 |
| HIVEP2 | Direct | 0.107692 | | 0.10992 | 0.115306 | 0.115933 | 0.101719 | 0.114316 |
| HLF | Direct | 0.110629 | | NA | 0.108863 | 0.122948 | NA | NA |
| HNF1A | Direct | NA |  | NA | NA | NA | NA | NA |
| HNF1B | Direct | NA |  | NA | NA | NA | NA | NA |
| HNF4A | Direct | 0.112095 | | 0.104884 | 0.095517 | 0.094915 | NA | NA |
| HNF4A | Direct | 0.115548 | | 0.107735 | NA | 0.093111 | 0.092935 | NA |
| HNF4G | Direct | 0.115304 | | 0.106928 | 0.10012 | 0.100374 | 0.091385 | 0.095141 |
| HOXA1 | Direct | NA |  | NA | NA | NA | NA | NA |
| HOXA10 | Direct | NA |  | NA | NA | NA | NA | NA |
| HOXA11 | Direct | NA |  | NA | NA | NA | NA | NA |
| HOXA13 | Direct | NA |  | NA | NA | NA | NA | NA |
| HOXA13 | Direct | NA |  | NA | NA | NA | 0.093293 | NA |
| HOXA2 | Direct | NA |  | NA | NA | NA | NA | NA |
| HOXA5 | Direct | 0.093386 | | NA | NA | NA | 0.089558 | NA |
| HOXA6 | Direct | NA |  | NA | NA | NA | NA | NA |
| HOXA6 | Direct | 0.101121 | | NA | NA | NA | 0.089005 | NA |
| HOXA6 | Direct | NA |  | NA | NA | NA | NA | NA |
| HOXA6 | Direct | 0.093736 | | NA | NA | NA | NA | NA |
| HOXA6 | Direct | NA |  | NA | NA | NA | NA | NA |
| HOXA6 | Direct | 0.088005 | | NA | NA | NA | NA | NA |
| HOXA9 | Direct | NA |  | NA | NA | NA | NA | NA |
| HOXA9 | Direct | 0.10159 | | NA | NA | NA | 0.091576 | NA |
| HOXB13 | Direct | NA |  | NA | NA | NA | NA | NA |
| HOXB13 | Direct | NA |  | NA | NA | NA | 0.095283 | NA |
| HOXB2 | Direct | NA |  | NA | NA | NA | NA | NA |
| HOXB3 | Direct | NA |  | NA | NA | NA | NA | NA |
| HOXB4 | Direct | 0.107639 | | 0.095516 | NA | NA | 0.090569 | NA |
| HOXB5 | Direct | NA |  | NA | NA | NA | NA | NA |
| HOXB7 | Direct | 0.094909 | | NA | NA | NA | NA | NA |
| HOXB8 | Direct | 0.091797 | | NA | NA | NA | NA | NA |
| HOXC10 | Direct | NA |  | NA | NA | NA | NA | NA |
| HOXC11 | Direct | 0.093847 | | NA | NA | NA | 0.095826 | NA |
| HOXC11 | Direct | NA |  | NA | NA | NA | NA | NA |
| HOXC12 | Direct | 0.093701 | | NA | NA | NA | 0.096674 | NA |
| HOXC13 | Direct | NA |  | NA | NA | NA | 0.097077 | NA |

| HOXC5 | Direct | 0.089785 | NA | NA | NA | NA | NA |
| --- | --- | --- | --- | --- | --- | --- | --- |
| HOXC9 | Direct | 0.101867 | NA | NA | NA | 0.090097 | 0.093537 |
| HOXD1 | Direct | NA | NA | NA | NA | NA | NA |
| HOXD11 | Direct | NA | NA | NA | NA | NA | NA |
| HOXD12 | Direct | NA | NA | NA | NA | NA | NA |
| HOXD12 | Direct | 0.09483 | NA | NA | NA | 0.096704 | NA |
| HOXD13 | Direct | NA | NA | NA | NA | NA | NA |
| HOXD13 | Direct | NA | NA | NA | NA | 0.09434 | NA |
| HOXD3 | Direct | NA | NA | NA | NA | NA | NA |
| HOXD9 | Direct | 0.095026 | NA | NA | NA | 0.096129 | NA |
| IKZF3 | ZifRC | 0.102214 | 0.112441 | 0.11995 | 0.124663 | 0.108605 | 0.132784 |
| IKZF5 | ZifRC | 0.114144 | 0.11115 | NA | 0.095955 | 0.111437 | 0.094315 |
| IRF1 | Direct | 0.091798 | NA | NA | NA | 0.109578 | 0.097151 |
| IRF3 | Direct | 0.100522 | 0.103117 | 0.107894 | 0.107492 | 0.120815 | 0.118274 |
| IRF4 | Direct | 0.130159 | 0.106982 | 0.147319 | 0.131753 | 0.150189 | 0.146467 |
| IRF5 | Direct | 0.104111 | 0.100516 | 0.110094 | 0.105314 | 0.114253 | 0.109038 |
| IRF6 | Indirect | NA | 0.099303 | 0.106729 | 0.098983 | 0.115244 | 0.096456 |
| IRF7 | Direct | 0.099929 | 0.097915 | NA | NA | 0.112685 | 0.096522 |
| IRF8 | Direct | 0.107953 | 0.101417 | 0.123447 | 0.112301 | 0.123452 | 0.122519 |
| IRF9 | Direct | 0.102857 | NA | 0.107135 | 0.104122 | 0.122881 | 0.111226 |
| JDP2 | Direct | 0.109359 | 0.123868 | 0.123228 | 0.101674 | 0.10437 | 0.095776 |
| JUN | Direct | 0.105844 | 0.118486 | 0.122026 | 0.105827 | 0.102379 | 0.097332 |
| JUNB | Direct | 0.107968 | 0.120193 | 0.12625 | 0.110106 | 0.104183 | 0.100336 |
| JUND | Direct | 0.100422 | 0.116005 | 0.112243 | 0.097606 | 0.101794 | NA |
| KLF1 | Direct | NA | 0.101371 | NA | NA | 0.092672 | 0.101577 |
| KLF12 | Direct | 0.100747 | 0.099941 | 0.099057 | 0.102317 | 0.094897 | 0.104439 |
| KLF12 | Direct | NA | 0.106309 | 0.109161 | 0.100907 | 0.097503 | 0.102296 |
| KLF11 | Inferred | 0.097217 | 0.110507 | 0.111535 | 0.101073 | 0.108347 | 0.105501 |
| KLF13 | Direct | 0.097217 | 0.110507 | 0.111535 | 0.101073 | 0.108347 | 0.105501 |
| KLF14 | Direct | NA | 0.101484 | 0.098597 | NA | 0.09704 | 0.101648 |
| KLF16 | Direct | NA | 0.102713 | 0.091325 | 0.088846 | 0.095645 | 0.109796 |
| KLF17 | Inferred | NA | 0.102161 | 0.094258 | NA | 0.09351 | 0.102667 |
| KLF2 | Inferred | NA | 0.102161 | 0.094258 | NA | 0.09351 | 0.102667 |
| KLF4 | Direct | NA | 0.102161 | 0.094258 | NA | 0.09351 | 0.102667 |
| KLF5 | Direct | 0.094266 | 0.104912 | 0.092838 | 0.089842 | 0.094168 | 0.111158 |
| KLF6 | Direct | 0.099021 | 0.103315 | 0.108432 | 0.104408 | 0.093822 | 0.115722 |
| KLF7 | Direct | NA | 0.100272 | NA | NA | 0.094515 | 0.099034 |
| LBX1 | Direct | NA | NA | NA | NA | NA | NA |
| LEF1 | Direct | 0.095878 | 0.097423 | NA | NA | 0.096929 | 0.094301 |

| LHX2 | Direct | NA | NA | NA | NA |  | NA | NA |  |
| --- | --- | --- | --- | --- | --- | --- | --- | --- | --- |
| LHX6 | Direct | NA | NA | NA | NA |  | NA | NA |  |
| LMX1A | Direct | NA | NA | NA | NA |  | NA | NA |  |
| LMX1B | Direct | NA | NA | NA | NA |  | NA | NA |  |
| LYL1 | Indirect | 0.112607 | 0.102137 | 0.12469 | 0.11903 | | 0.097861 | 0.123465 | |
| MAF | Direct | 0.113669 | 0.106972 | 0.118476 | 0.114367 | | 0.097347 | 0.107367 | |
| MAFA | Direct | 0.104292 | 0.104509 | 0.108431 | 0.108084 | | 0.094305 | 0.10562 | |
| MAFB | Direct | 0.116369 | 0.108155 | 0.124594 | 0.120282 | | 0.099696 | 0.11547 | |
| MAFF | Direct | 0.098571 | 0.097944 | 0.117248 | 0.111392 | | NA | 0.102234 | |
| MAFG | Direct | 0.103917 | 0.099313 | 0.11895 |  | 0.1135 | NA | 0.105752 | |
| MAX | Direct | 0.10548 | 0.114134 | 0.120675 | 0.115103 | | 0.119696 | 0.11606 | |
| MBD2 | Direct | 0.114324 | 0.109066 | 0.140246 | 0.119869 | | NA | 0.113692 | |
| MECP2 | Direct | 0.128828 | 0.115451 | 0.146474 | 0.132604 | | NA | 0.124154 | |
| MEIS2 | Direct | 0.100121 | 0.099701 | 0.099005 | 0.101192 | | 0.097922 | 0.102014 | |
| MEOX1 | Direct | 0.091014 | NA | NA | NA |  | NA | NA |  |
| MEOX2 | Direct | NA | NA | NA | NA |  | NA | NA |  |
| MLXIPL | Direct | 0.095084 | 0.115985 | 0.114263 | 0.109677 | | 0.125625 |  | 0.1117 |
| MNX1 | Direct | NA | NA | NA | NA |  | NA | NA |  |
| MSC | Direct | 0.109198 | 0.102098 | 0.11129 | 0.112021 | | 0.102174 | 0.12016 | |
| MSX1 | Direct | 0.091705 | NA | NA | NA |  | NA | NA |  |
| NKX1-2 | Inferred | 0.091705 | NA | NA | NA |  | NA | NA |  |
| MTF1 | Direct | 0.108691 | 0.106066 | 0.123002 | 0.115506 | | 0.103394 | 0.116579 | |
| MXD1 | Inferred | 0.109502 | 0.119457 | 0.123589 | 0.118735 | | 0.122299 | 0.118707 | |
| MXD3 | Inferred | 0.109502 | 0.119457 | 0.123589 | 0.118735 | | 0.122299 | 0.118707 | |
| MXD4 | Inferred | 0.109502 | 0.119457 | 0.123589 | 0.118735 | | 0.122299 | 0.118707 | |
| MXI1 | Direct | 0.109502 | 0.119457 | 0.123589 | 0.118735 | | 0.122299 | 0.118707 | |
| MYB | Direct | 0.110993 | 0.106771 | 0.115801 | 0.117555 | | 0.101818 | 0.118919 | |
| RCOR2 | Inferred | 0.110993 | 0.106771 | 0.115801 | 0.117555 | | 0.101818 | 0.118919 | |
| RCOR3 | Inferred | 0.110993 | 0.106771 | 0.115801 | 0.117555 | | 0.101818 | 0.118919 | |
| SNAPC4 | Inferred | 0.110993 | 0.106771 | 0.115801 | 0.117555 | | 0.101818 | 0.118919 | |
| DNAJC2 | Inferred | 0.115916 | 0.112113 | 0.108777 | 0.109946 | | 0.115269 | 0.110273 | |
| MYBL1 | Direct | 0.115916 | 0.112113 | 0.108777 | 0.109946 | | 0.115269 | 0.110273 | |
| DNAJC2 | Inferred | 0.108312 | 0.106806 | 0.105771 | 0.106909 | | 0.108882 | 0.109627 | |
| MYBL1 | Direct | 0.108312 | 0.106806 | 0.105771 | 0.106909 | | 0.108882 | 0.109627 | |
| MYCL | Inferred | 0.115431 | 0.126721 | 0.132667 | 0.123759 | | 0.131568 | 0.123224 | |
| ASCL1 | Inferred | 0.110691 | 0.102186 | 0.11948 | 0.116598 | | 0.101364 | 0.125074 | |
| ASCL5 | Inferred | 0.110691 | 0.102186 | 0.11948 | 0.116598 | | 0.101364 | 0.125074 | |
| ASCL3 | Inferred | 0.110367 | 0.101423 | 0.115349 | 0.113954 | | 0.101992 | 0.120403 | |
| MYF6 | Direct | 0.110367 | 0.101423 | 0.115349 | 0.113954 | | 0.101992 | 0.120403 | |

| MYOD1 | Direct | 0.101421 | 0.10155 | | 0.115021 | 0.114826 | 0.101865 | | 0.125019 |
| --- | --- | --- | --- | --- | --- | --- | --- | --- | --- |
| MYOG | Direct | 0.113143 | 0.105842 | | 0.125037 | 0.120947 |  | 0.1024 | 0.13068 |
| NEUROD1 | Direct | 0.109906 | 0.105959 | | 0.114637 | 0.11274 | 0.101374 | | 0.121678 |
| NEUROD4 | Direct | 0.103523 | 0.097563 | | 0.102067 | 0.103217 | 0.096946 | | 0.106794 |
| NFE2 | Direct | 0.109095 | 0.11052 | | 0.123178 | 0.118167 | 0.100039 | | 0.105885 |
| NFE2 | Direct | 0.108469 | 0.123272 | | 0.120979 | 0.100342 | 0.103158 | | NA |
| NFE2L2 | Direct | 0.107154 |  | 0.11 | 0.125273 | 0.120692 | 0.098127 | | 0.108256 |
| NFIA | Direct | 0.147743 | 0.118599 | | 0.117459 | 0.125243 | NA |  | 0.110138 |
| NFIB | Direct | 0.15217 | 0.120606 | | 0.121698 | 0.127122 | 0.093677 | | 0.111754 |
| NFIC | Direct | 0.121761 | 0.102164 | | 0.097507 | 0.100287 | NA |  | NA |
| NFIX | Direct | 0.15543 | 0.121599 | | 0.119871 | 0.126845 | 0.094078 | | 0.110758 |
| NFIX | Direct | 0.140394 | 0.111936 | | 0.106843 | 0.112322 | 0.093822 | | 0.103733 |
| NFKB1 | Direct | NA | NA |  | NA | NA | 0.100731 | | 0.101271 |
| NFKB1 | Direct | 0.101288 | 0.105274 | | 0.10375 | 0.103688 | 0.102869 | | 0.109664 |
| NFKB2 | Direct | NA | NA |  | NA | NA | 0.100678 | | 0.095383 |
| NKX2-1 | Direct | NA | NA |  | NA | NA | NA |  | NA |
| NKX2-2 | Direct | NA | NA |  | NA | NA | NA |  | NA |
| NKX2-3 | Direct | 0.09589 | 0.093082 | | NA | 0.093864 | 0.094224 | | 0.095968 |
| NKX2-4 | Direct | NA | NA |  | NA | NA | NA |  | NA |
| NKX2-5 | Direct | NA | NA |  | NA | NA | NA |  | NA |
| NKX2-5 | Direct | NA | NA |  | NA | NA | NA |  | NA |
| NKX2-6 | Direct | NA | NA |  | NA | NA | NA |  | NA |
| NKX2-8 | Direct | NA | 0.092999 | | NA | NA | 0.093823 | | NA |
| NKX3-1 | Direct | NA | NA |  | NA | NA | 0.093993 | | 0.09681 |
| NKX3-2 | Direct | 0.092577 | 0.094994 | | 0.098998 | 0.09994 | 0.096395 | | 0.105033 |
| NKX6-2 | Direct | NA | NA |  | NA | NA | NA |  | NA |
| NKX6-3 | Direct | NA | NA |  | NA | NA | NA |  | NA |
| NOBOX | Direct | NA | NA |  | NA | NA | NA |  | NA |
| NPAS2 | Direct | 0.114677 | 0.12422 | | 0.125599 | 0.120626 | 0.129835 | | 0.122493 |
| NR0B1 | Direct | 0.106798 | 0.109017 | | 0.102518 | 0.102128 | 0.103651 | | 0.11732 |
| NR0B2 | Inferred | 0.106798 | 0.109017 | | 0.102518 | 0.102128 | 0.103651 | | 0.11732 |
| NR1H2 | Direct | 0.125233 | 0.122359 | | NA | NA | NA |  | NA |
| NR1H4 | Direct | 0.094934 | 0.103331 | | NA | 0.096129 | NA |  | NA |
| NR2C1 | Direct | 0.102116 | 0.106114 | | NA | NA | NA |  | NA |
| NR2C2 | Direct | 0.107144 | 0.107151 | | NA | NA | 0.094778 | | 0.094954 |
| NR2C2 | Direct | 0.104985 | 0.116188 | | 0.141606 | 0.139894 | 0.098654 | | 0.130165 |
| NR2F1 | Direct | 0.109779 | 0.108327 | | NA | 0.092861 | 0.093072 | | NA |
| NR2F1 | Direct | NA | 0.102823 | | NA | NA | NA |  | NA |
| NR2F2 | Direct | 0.111076 | 0.109565 | | NA | 0.094057 | 0.094145 | | 0.093459 |

| NR2F6 | Direct | 0.10779 | 0.107831 | 0.089089 | NA | 0.094792 | 0.091393 |
| --- | --- | --- | --- | --- | --- | --- | --- |
| NR4A1 | Direct | 0.103277 | 0.101249 | NA | NA | 0.090308 | NA |
| NR4A2 | Direct | 0.102616 | 0.102161 | NA | NA | 0.091498 | NA |
| NR4A3 | Direct | 0.100878 | 0.103595 | NA | NA | NA | NA |
| NR5A1 | Direct | 0.098805 | 0.10366 | NA | 0.094094 | NA | NA |
| NRL | Direct | 0.114377 | 0.107568 | 0.117249 | 0.114148 | 0.097474 | 0.105924 |
| ONECUT1 | Direct | NA | NA | NA | NA | NA | NA |
| SATB1 | Inferred | NA | NA | NA | NA | NA | NA |
| SATB2 | Inferred | NA | NA | NA | NA | NA | NA |
| OTP | Direct | NA | NA | NA | NA | NA | NA |
| PAX1 | Direct | 0.101792 | 0.104707 | 0.122483 | 0.114527 | 0.119545 | 0.136803 |
| PAX2 | Direct | 0.097782 | 0.102477 | 0.102944 | 0.10223 | 0.106336 | 0.108749 |
| PAX2 | Direct | 0.107454 | 0.110068 | 0.118068 | 0.113734 | 0.104666 | 0.109407 |
| PAX4 | Direct | NA | NA | NA | NA | NA | NA |
| PAX5 | Direct | NA | 0.097812 | 0.104979 | 0.102365 | 0.102658 | 0.112816 |
| PAX6 | Direct | 0.098201 | 0.098169 | 0.098506 | 0.097349 | 0.106213 | 0.108031 |
| PAX8 | Direct | 0.101813 | 0.105432 | 0.102853 | 0.099436 | 0.100809 | 0.10139 |
| PAX8 | Direct | 0.095067 | 0.097421 | 0.102557 | 0.104353 | 0.09422 | 0.104198 |
| PDX1 | Direct | NA | NA | NA | NA | NA | NA |
| PLAGL1 | Direct | NA | 0.103127 | 0.108484 | 0.105917 | NA | 0.108339 |
| PLAGL2 | ZifRC | 0.097544 | 0.105984 | 0.111308 | 0.107173 | 0.096476 | 0.114369 |
| PPARD | Direct | 0.105637 | 0.107905 | 0.096219 | 0.097224 | 0.093738 | 0.097909 |
| PPARG | Direct | 0.113363 | 0.108537 | 0.092085 | 0.093212 | 0.094123 | 0.095452 |
| PRDM15 | ZifRC | 0.112094 | 0.094174 | 0.148038 | 0.132789 | NA | 0.125454 |
| PTF1A | Direct | 0.106336 | 0.105545 | 0.10768 | 0.108489 | 0.099702 | 0.11147 |
| RARA | Direct | 0.098589 | 0.108094 | 0.096838 | 0.09416 | 0.09516 | 0.094368 |
| RELA | Direct | 0.10206 | 0.104854 | 0.104943 | 0.105367 | 0.104901 | 0.111393 |
| RELB | Direct | 0.101842 | 0.10241 | 0.098027 | 0.09906 | 0.10064 | 0.101374 |
| RFX1 | Direct | 0.109587 | 0.108067 | 0.116778 | 0.115796 | 0.101031 | 0.110769 |
| RFX1 | Direct | 0.100891 | 0.100187 | 0.104146 | 0.104161 | 0.095333 | 0.09972 |
| RFX2 | Direct | 0.121655 | 0.112527 | 0.117487 | 0.116998 | 0.105752 | 0.115323 |
| RFX3 | Direct | 0.104505 | 0.10372 | 0.109448 | 0.109297 | 0.098557 | 0.103889 |
| RFX4 | Direct | 0.118151 | 0.11081 | 0.119712 | 0.116481 | 0.102119 | 0.110592 |
| RFX5 | Direct | 0.104628 | 0.101791 | 0.104927 | 0.107484 | 0.095552 | 0.10707 |
| RFX5 | Direct | 0.109607 | 0.104531 | 0.107839 | 0.106856 | 0.098634 | 0.102689 |
| RFX6 | Direct | 0.09927 | 0.099172 | NA | 0.099826 | 0.096868 | NA |
| RHOXF1 | Direct | NA | 0.094921 | NA | NA | NA | NA |
| RHOXF2 | Inferred | NA | 0.094921 | NA | NA | NA | NA |
| RHOXF2B | Inferred | NA | 0.094921 | NA | NA | NA | NA |

| RUNX1 | Direct | NA | 0.09286 | | 0.127578 | 0.128683 | 0.109452 | 0.139472 |
| --- | --- | --- | --- | --- | --- | --- | --- | --- |
| RUNX2 | Direct | NA | 0.09285 | | 0.134753 | 0.134484 | 0.110327 | 0.145446 |
| RUNX3 | Direct | NA | NA |  | 0.122281 | 0.121086 | 0.101269 | 0.132514 |
| RXRA | Direct | 0.111236 | 0.107932 | | 0.092076 | 0.093415 | 0.093887 | 0.095229 |
| RXRA | Direct | 0.10253 | 0.106518 | | NA | NA | NA | NA |
| RXRB | Direct | 0.12206 | 0.116278 | | NA | NA | NA | NA |
| RXRG | Direct | 0.118673 | 0.114342 | | NA | NA | NA | NA |
| SIX1 | Direct | 0.10354 | 0.10007 | | NA | 0.096977 | 0.099366 | NA |
| SMAD9 | Inferred | 0.103245 | 0.103942 | | 0.106189 | 0.106853 | 0.094197 | 0.106233 |
| SMAD2 | Direct | 0.105009 | 0.103779 | | 0.104793 | 0.106312 | 0.096201 | 0.105179 |
| SMAD3 | Direct | 0.112919 | 0.105044 | | 0.10771 | 0.10776 | 0.094589 | 0.104441 |
| SMAD7 | Inferred | 0.112919 | 0.105044 | | 0.10771 | 0.10776 | 0.094589 | 0.104441 |
| SMAD6 | Inferred | 0.112661 | 0.105404 | | 0.111109 | 0.108957 | 0.098015 | 0.10581 |
| SMARCC1 | Direct | 0.100874 | 0.115141 | | 0.118771 | 0.106052 | 0.100916 | 0.095891 |
| TADA2A | Inferred | 0.100874 | 0.115141 | | 0.118771 | 0.106052 | 0.100916 | 0.095891 |
| SOX10 | Direct | 0.09161 | 0.092985 | | NA | 0.092096 | 0.090721 | NA |
| SOX3 | Direct | 0.088809 | 0.097826 | | NA | NA | 0.091814 | NA |
| SOX6 | Direct | NA |  | 0.1007 | NA | NA | NA | NA |
| SP3 | Direct | NA | 0.102163 | | 0.089217 | NA | 0.094758 | 0.107306 |
| SPDEF | Direct | 0.113419 | 0.134916 | | 0.15682 | 0.159532 | 0.110589 | 0.15271 |
| SPI1 | Direct | 0.103712 | 0.112555 | | 0.186947 | 0.196112 | 0.145068 | 0.206682 |
| SPI1 | Direct | 0.094408 | 0.107169 | | 0.120667 | 0.12795 | 0.114054 | 0.144528 |
| SPIB | Direct | 0.106934 | 0.121497 | | 0.14927 | 0.153041 | 0.115967 | 0.158443 |
| SPIB | Direct | 0.104214 | 0.116911 | | 0.183698 | 0.18823 | 0.135015 | 0.192956 |
| SPIC | Direct | 0.100887 | 0.116288 | | 0.15603 | 0.166046 | 0.126479 | 0.174171 |
| STAT1 | Direct | 0.114411 | 0.108052 | | 0.105336 | 0.105597 | 0.09779 | 0.099455 |
| STAT2 | Direct | 0.102266 |  | 0.0982 | 0.109764 | 0.107392 | 0.120107 | 0.118748 |
| STAT3 | Direct | 0.116888 | 0.110656 | | 0.106604 | 0.108035 | 0.096323 | 0.10084 |
| TAL1 | Direct | 0.100358 | 0.101286 | | 0.097689 | 0.101873 | 0.100617 | 0.103605 |
| TCF12 | Direct | 0.106755 | 0.10561 | | 0.119862 | 0.11741 | 0.101426 | 0.127639 |
| TCF21 | Direct | 0.122558 | 0.104497 | | 0.121002 | 0.119159 | 0.103554 | 0.125898 |
| TCF3 | Direct | NA | 0.093153 | | NA | NA | 0.100526 | 0.108988 |
| TCF7 | Direct | 0.094371 | 0.092951 | | NA | NA | 0.094556 | NA |
| TEAD2 | Inferred | 0.108238 | 0.099679 | | NA | NA | NA | NA |
| TEAD3 | Direct | 0.104769 | 0.095417 | | NA | NA | NA | NA |
| TFAP2A | Direct | 0.097302 | 0.100555 | | 0.112854 | 0.106789 | NA | 0.105273 |
| TFAP2A | Direct | 0.110892 | 0.109281 | | 0.117205 | 0.113037 | 0.094723 | 0.114002 |
| TFAP2A | Direct | 0.105542 | 0.104599 | | 0.122423 | 0.112875 | NA | 0.112666 |
| TFAP2D | Direct | NA | NA |  | NA | NA | NA | NA |

| TFAP2E | Direct | 0.107198 | 0.106792 | 0.119371 | 0.112177 | NA | 0.11286 |
| --- | --- | --- | --- | --- | --- | --- | --- |
| TFAP4 | Direct | 0.117057 | 0.107077 | 0.125817 | 0.120793 | 0.098836 | 0.125135 |
| TFE3 | Direct | 0.101798 | 0.114455 | 0.119623 | 0.113102 | 0.119289 | 0.111623 |
| TFEB | Direct | 0.101108 | 0.113045 | 0.118167 | 0.112675 | 0.116016 | 0.109722 |
| TFEC | Direct | 0.097348 | 0.10662 | 0.113175 | 0.109666 | 0.109262 | 0.104241 |
| TGIF2LX | Direct | 0.103497 | 0.100059 | 0.107761 | 0.107563 | 0.09859 | 0.109895 |
| TLX1 | Direct | 0.112293 | 0.101665 | 0.103587 | 0.110717 | NA | 0.096041 |
| USF1 | Direct | 0.101065 | 0.11289 | 0.119779 | 0.114922 | 0.114769 | 0.111504 |
| USF2 | Direct | 0.095721 | 0.109368 | 0.114904 | 0.109772 | 0.111339 | 0.106734 |
| VAX1 | Direct | NA | NA | NA | NA | NA | NA |
| VAX2 | Direct | NA | NA | NA | NA | NA | NA |
| VSX1 | Direct | NA | NA | NA | NA | NA | NA |
| ZBTB1 | Indirect | 0.112335 | 0.099299 | 0.149648 | 0.130059 | NA | 0.128838 |
| ZBTB14 | ZifRC | 0.105299 | 0.111833 | 0.123581 | 0.114938 | 0.106216 | 0.114358 |
| ZBTB18 | Direct | 0.100076 | 0.094191 | NA | 0.09772 | 0.096154 | 0.102843 |
| ZBTB33 | Direct | 0.109584 | 0.112574 | 0.116886 | 0.109121 | 0.106994 | 0.114482 |
| ZBTB42 | Direct | 0.099776 | 0.093257 | NA | 0.096646 | 0.095993 | 0.10221 |
| ZBTB7A | Direct | NA | 0.096393 | 0.102769 | 0.094826 | 0.099019 | 0.101539 |
| ZFAT | ZifRC | 0.096941 | 0.102755 | 0.11056 | 0.105514 | 0.093258 | 0.104117 |
| ZFHX2 | Direct | NA | NA | NA | NA | NA | NA |
| ZFP57 | ZifRC | 0.121897 | 0.114271 | 0.13656 | 0.125816 | 0.103609 | 0.119905 |
| ZFP64 | ZifRC | 0.102886 | 0.106664 | 0.096613 | 0.099792 | 0.09614 | 0.096071 |
| ZFX | Direct | NA | 0.097241 | 0.10393 | 0.095821 | NA | NA |
| ZFY | Direct | NA | 0.10036 | 0.104534 | 0.095331 | NA | NA |
| ZIC4 | Direct | NA | 0.10309 | 0.115213 | 0.107303 | 0.09531 | 0.111615 |
| ZIC5 | Direct | 0.09396 | 0.100858 | 0.102346 | 0.101014 | NA | 0.114458 |
| ZIC5 | Direct | 0.097604 | 0.104487 | 0.116294 | 0.108072 | NA | 0.113634 |
| ZIC5 | Direct | 0.098142 | 0.101352 | 0.117271 | 0.111757 | NA | 0.112266 |
| ZNF100 | ZifRC | NA | 0.105479 | 0.097267 | 0.097355 | 0.095401 | 0.106207 |
| ZNF142 | ZifRC | 0.108131 | 0.106969 | 0.123851 | 0.116252 | 0.103705 | 0.112352 |
| ZNF19 | ZifRC | 0.103073 | 0.118797 | 0.10696 | 0.106164 | 0.106488 | 0.117694 |
| ZNF2 | ZifRC | 0.116816 | 0.112448 | 0.129152 | 0.124357 | 0.102267 | 0.121076 |
| ZNF208 | ZifRC | 0.107094 | 0.106979 | 0.107341 | 0.108616 | 0.097632 | 0.107157 |
| ZNF208 | ZifRC | 0.105669 | 0.108347 | 0.100075 | 0.10573 | 0.101331 | 0.110911 |
| ZNF292 | ZifRC | 0.095014 | 0.096526 | 0.118315 | 0.104815 | 0.09553 | 0.103684 |
| ZNF296 | ZifRC | 0.106845 | 0.110724 | 0.105207 | 0.109067 | 0.103713 | 0.109183 |
| ZNF296 | ZifRC | 0.108736 | 0.118005 | 0.118359 | 0.123183 | 0.106135 | 0.119659 |
| ZNF329 | ZifRC | 0.105495 | 0.112132 | 0.113214 | 0.10924 | 0.09681 | 0.111134 |
| ZNF335 | ZifRC | 0.109315 | 0.102963 | 0.133013 | 0.116331 | NA | 0.112408 |

| ZNF343 | ZifRC | 0.111569 | | 0.110498 | | 0.117783 | | 0.117925 | NA | 0.114713 |
| --- | --- | --- | --- | --- | --- | --- | --- | --- | --- | --- |
| ZNF350 | Direct | 0.107205 | | 0.109529 | | 0.110709 | | 0.111297 | 0.09631 | 0.117071 |
| ZNF433 | ZifRC | 0.12035 | | 0.115289 | | 0.11776 | | 0.115203 | 0.110417 | 0.112013 |
| ZNF441 | ZifRC | NA |  | 0.101508 | |  | 0.1056 | 0.105747 | NA | 0.108752 |
| ZNF445 | ZifRC | 0.130779 | | 0.117839 | | 0.140526 | | 0.134997 | 0.106132 | 0.123251 |
| ZNF445 | ZifRC | 0.104041 | | 0.107164 | | NA |  | NA | 0.095985 | NA |
| ZNF516 | ZifRC | 0.111288 | | 0.10582 | | 0.119297 | | 0.116463 | 0.09827 | 0.114711 |
| ZNF536 | ZifRC | 0.107252 | | 0.099987 | | NA |  | 0.10889 | 0.09314 | 0.107134 |
| ZNF547 | ZifRC | 0.107575 | | 0.109135 | | 0.118289 | | 0.114699 | 0.096939 | 0.109515 |
| ZNF548 | ZifRC | 0.102287 | | 0.101026 | | 0.107878 | | 0.108099 | 0.093951 | 0.108798 |
| ZNF550 | ZifRC | 0.102247 | |  | 0.108 | 0.106927 | | 0.106981 | NA | 0.108418 |
| ZNF560 | ZifRC | 0.09408 | | NA |  | NA |  | NA | NA | NA |
| ZNF566 | ZifRC | 0.099878 | | 0.105331 | | 0.110457 | | 0.113059 | 0.09725 | 0.109616 |
| ZNF579 | ZifRC | NA |  | 0.101923 | | 0.103514 | | 0.096671 | 0.095969 | 0.10889 |
| ZNF579 | ZifRC | NA |  | 0.104993 | | 0.110647 | | 0.102156 | 0.101281 | 0.115785 |
| ZNF594 | ZifRC | 0.120583 | | 0.109555 | | 0.135273 | | 0.126114 | 0.098496 | 0.123284 |
| ZNF594 | ZifRC | 0.099105 | | 0.100683 | | 0.102947 | | 0.099352 | 0.094594 | 0.100387 |
| ZNF594 | ZifRC | 0.105991 | | 0.102936 | | 0.122616 | | 0.115619 | 0.09426 | 0.110711 |
| ZNF597 | ZifRC | 0.094371 | | 0.096651 | | NA |  | NA | NA | NA |
| ZNF606 | ZifRC | 0.098927 | | 0.104532 | | 0.100429 | | 0.105013 | 0.095698 | 0.106878 |
| ZNF676 | ZifRC |  | 0.1032 | 0.109171 | | 0.103028 | | 0.104181 | 0.100109 | 0.11536 |
| ZNF681 | ZifRC | 0.094765 | | 0.106503 | | NA |  | 0.097521 | 0.104607 | 0.103495 |
| ZNF699 | ZifRC | 0.10491 | | 0.105616 | | 0.110699 | | 0.108275 | 0.095066 | 0.105914 |
| ZNF705D | ZifRC | 0.09597 | | 0.102491 | | 0.103091 | | 0.108769 | 0.09464 | 0.115781 |
| ZNF716 | ZifRC | 0.103906 | | 0.104598 | | 0.097817 | | 0.109874 | 0.094111 | 0.108529 |
| ZNF721 | ZifRC | 0.10145 | | 0.109008 | | 0.109679 | | 0.111191 | 0.108788 | 0.112286 |
| ZNF732 | ZifRC | 0.111484 | | 0.106384 | | 0.108909 | | 0.109634 | 0.096644 | 0.106323 |
| ZNF746 | ZifRC | 0.093883 | | 0.103899 | | 0.095102 | | 0.093163 | 0.09419 | 0.110903 |
| ZNF763 | ZifRC | 0.104746 | | 0.101736 | | 0.107129 | | 0.105601 | 0.096908 | 0.102342 |
| ZNF768 | ZifRC | 0.102614 | | 0.103092 | | 0.098144 | | 0.101117 | 0.096635 | 0.100191 |
| ZNF770 | ZifRC | 0.100849 | | 0.107294 | | 0.104909 | | 0.107146 | 0.101452 | 0.115188 |
| ZNF777 | ZifRC | 0.10619 | | 0.108706 | | 0.133437 | | 0.115371 | 0.101838 | 0.117372 |
| ZNF781 | ZifRC | 0.101356 | | 0.106625 | | 0.10472 | | 0.102598 | 0.094667 | 0.107264 |
| ZNF80 | ZifRC | 0.094084 | | 0.096456 | | 0.103142 | | 0.10432 | 0.112286 | 0.108845 |
| ZNF827 | ZifRC | NA |  | 0.094159 | | NA |  | NA | 0.093681 | 0.096475 |
| ZNF836 | ZifRC | 0.105226 | | 0.10186 | | 0.107693 | | 0.10499 | 0.096255 | 0.104345 |
| ZNF91 | ZifRC | 0.102866 | | 0.106526 | | 0.113014 | | 0.11214 | 0.100985 | 0.108994 |
| ZSCAN25 | ZifRC | 0.100298 | | 0.108003 | | 0.098679 | | 0.100478 | 0.09921 | 0.115924 |

brain..ceehbrain_hipp cd14_posi cd19positi cd34_negacd38_negacd3_negat cd4_naive.

0.107655 0.112316 0.114389 0.101692 0.118793 0.117236 0.115619	0.11466

NA	NA	NA	NA	NA	NA	NA	NA

0.105903 0.108756 0.114007 0.118499	0.11074 0.116999 0.113607	0.11531

0.104532 0.105229 0.113847 0.118759 0.131165 0.132743 0.104789 0.111293

NA	NA	0.106108 0.123329 0.103769 0.118265 0.099962 0.100752

0.164396 0.154617 0.124242 0.097882 0.136225 0.152491 0.140313 0.122034

0.10169 0.107757 0.116549 0.123119 0.117217	0.12193	0.10854 0.110046

NA	0.098776 0.106851 0.116489 0.111065 0.112107 0.102461 0.104522

NA	0.098776 0.106851 0.116489 0.111065 0.112107 0.102461 0.104522

0.103863 0.112046 0.120228 0.104159 NA	NA	NA	0.100062

0.160743 0.148971 0.115831 0.095695 0.122011 0.130777 0.122894 0.108046

0.131267 0.125681 0.106113 0.096921	0.10743 0.124036 0.119239 0.104819

NA	0.100502 0.119505 0.097124 0.112034 0.109179 0.099707 0.100612

NA	0.104132 0.119264 0.101069 0.115054 0.111085 0.102113 0.102672

NA	NA	NA	0.107423 NA	NA	NA	NA

0.094097 NA	NA	NA	NA	NA	NA	NA

0.093808 0.095677 0.096912	0.09938 NA	NA	NA	0.095705

0.093808 0.095677 0.096912	0.09938 NA	NA	NA	0.095705

0.104772 0.098569 0.149962	0.11721	0.12384 0.126979 0.150773 0.151113

0.103826 0.106236 0.113499 0.126262	0.11082 0.120629 0.116197 0.114746

0.094871 NA	NA	NA	NA	NA	NA	NA

0.099096	0.10249 0.137242 0.103489 0.141023	0.14029 0.164313 0.157876

NA	NA	NA	NA	NA	NA	NA	NA NA	NA	NA	NA	NA	NA	NA	NA

0.09199 NA	0.1124 NA	NA	NA	NA	NA NA	NA	0.140237 NA	NA	NA	NA	NA NA	NA	0.141577 NA	NA	NA	NA	NA NA	NA	0.132131 NA	NA	NA	NA	NA

0.112826 0.113137 0.111537 0.123424 0.110617 0.117657 0.113636 0.114538

0.112826 0.113137 0.111537 0.123424 0.110617 0.117657 0.113636 0.114538

0.126499 0.129274 0.129906 0.143789 0.141199 0.143621 0.129744 0.129654

0.125463 0.129465 0.132603 0.149485 0.139594 0.140115	0.13194 0.130631

NA	0.104896 0.114201 0.099446 NA	NA	NA	NA NA	NA	0.11982 NA	NA	NA	NA	NA NA	NA	NA	NA	NA	NA	NA	NA

0.091382 NA	NA	NA	NA	NA	NA	NA

0.092759 NA	NA	NA	NA	NA	NA	NA

0.106208 0.104475 0.108327 NA	0.124496 0.118278 0.110103 0.107808

| 0.093438 | 0.098727 | 0.098459 | | 0.095403 | 0.100979 | 0.128785 | | 0.095763 | NA |  |
| --- | --- | --- | --- | --- | --- | --- | --- | --- | --- | --- |
| 0.093438 | 0.098727 | 0.098459 | | 0.095403 | 0.100979 | 0.128785 | | 0.095763 | NA |  |
| 0.093438 | 0.098727 | 0.098459 | | 0.095403 | 0.100979 | 0.128785 | | 0.095763 | NA |  |
| NA | 0.096352 | 0.096953 | | 0.10458 | 0.103852 | 0.098672 | | 0.108843 | 0.113951 | |
| NA | 0.098225 | 0.098405 | | 0.100835 | 0.108021 | 0.102811 | | 0.10751 | 0.109829 | |
| 0.105177 | 0.100456 | 0.163251 | | 0.106926 | 0.164768 | 0.154056 | | 0.177555 | 0.176461 | |
| 0.118443 | 0.109093 | 0.185391 | | 0.112962 | 0.184291 | 0.172439 | | 0.19163 | 0.193078 | |
| 0.12401 | 0.113199 | 0.205576 | | 0.117503 | 0.204587 | 0.193946 | | 0.212953 | 0.213513 | |
| 0.12083 | 0.111376 | 0.179039 | | 0.11354 | 0.182687 | 0.168729 | | 0.196188 | 0.192629 | |
| 0.116038 | 0.109341 | 0.178995 | | 0.113667 | 0.179214 | 0.167519 | | 0.181206 | 0.18374 | |
| 0.115245 | 0.109114 | 0.189552 | | 0.112966 | 0.188775 | 0.181055 | | 0.190876 | 0.190658 | |
| 0.115781 | 0.10693 | 0.199901 | | 0.117569 | 0.187227 | 0.179122 | | 0.203719 | 0.202717 | |
| 0.125753 | 0.117397 | 0.181903 | | 0.109373 | 0.194478 | 0.183917 | | 0.184828 | 0.186826 | |
| 0.125275 | 0.114006 | 0.187541 | | 0.110701 | 0.198433 | 0.186143 | | 0.196454 | 0.199644 | |
| 0.129504 | 0.118548 | 0.19855 | | 0.112844 | 0.210761 | 0.198165 | | 0.207578 | 0.210006 | |
| 0.094394 | NA | NA |  | NA | NA | NA |  | NA | NA |  |
| 0.103235 | 0.105567 | 0.102733 | | 0.120111 | 0.103365 | 0.106699 | | 0.101119 | 0.102357 | |
| 0.130082 | 0.115797 | 0.196983 | | 0.115055 | 0.209904 | 0.194759 | | 0.210484 | 0.214608 | |
| 0.133064 | 0.116114 | 0.20668 | | 0.117375 | 0.217143 | 0.208675 | | 0.220747 | 0.225332 | |
| 0.12278 | 0.113694 | 0.160714 | | 0.109674 | 0.167934 | 0.161902 | | 0.153219 | 0.153018 | |
| 0.123604 | 0.111606 | 0.214422 | | 0.117644 | 0.220371 | 0.209968 | | 0.23409 | 0.234161 | |
| NA | NA | NA |  | 0.095539 | NA | NA |  | NA | NA |  |
| 0.093036 | NA | NA |  | NA | NA | NA |  | NA | NA |  |
| 0.126527 | 0.114024 | 0.208691 | | 0.116274 | 0.216487 | 0.206393 | | 0.226284 | 0.229919 | |
| 0.12337 | 0.119538 | 0.150906 | | 0.10497 | 0.159893 | 0.145648 | | 0.149037 | 0.151793 | |
| 0.111602 | 0.104355 | 0.172759 | | 0.107876 | 0.169119 | 0.157442 | | 0.178154 | 0.178637 | |
| 0.131608 | 0.117624 | 0.20789 | | 0.115269 | 0.218184 | 0.207008 | | 0.22106 | 0.224531 | |
| 0.126462 | 0.110956 |  | 0.1936 | 0.117442 | 0.202329 | 0.191242 | | 0.208259 | 0.213398 | |
| 0.115949 | 0.108308 | 0.15617 | | 0.103756 | 0.163883 | 0.15849 | | 0.159808 | 0.163007 | |
| 0.115949 | 0.108308 | 0.15617 | | 0.103756 | 0.163883 | 0.15849 | | 0.159808 | 0.163007 | |
| 0.128865 | 0.114038 | 0.212119 | | 0.116613 | 0.218939 | 0.209599 | | 0.228611 | 0.230698 | |
| 0.11931 | 0.111284 | 0.170589 | | 0.104785 | 0.176699 | 0.16934 | | 0.176379 | 0.178196 | |
| 0.104305 | 0.098669 | 0.165121 | | 0.107377 | 0.158889 | 0.151637 | | 0.160996 | 0.159542 | |
| 0.111136 | 0.105628 | 0.161654 | | 0.112757 | 0.164785 | 0.157332 | | 0.173445 | 0.174519 | |
| 0.091014 | NA | NA |  | NA | NA | NA |  | NA | NA |  |
| 0.124162 | 0.122684 | 0.106807 | | 0.098913 | 0.112173 | 0.122649 | | 0.118321 | 0.106455 | |
| 0.122459 | 0.10844 | 0.205823 | | 0.118176 | 0.207813 | 0.195872 | | 0.222414 |  | 0.2284 |
| 0.128919 | 0.11561 | 0.216711 | | 0.117435 | 0.224424 | 0.213939 | | 0.234824 | 0.236228 | |
| 0.124248 | 0.11538 | 0.162986 | | 0.110863 | 0.173515 |  | 0.168 | 0.157408 | 0.159133 | |

| NA | 0.102891 | 0.116701 | | 0.101713 | 0.106972 | 0.099535 | | 0.097276 | 0.098508 |
| --- | --- | --- | --- | --- | --- | --- | --- | --- | --- |
| 0.097882 | 0.10394 | 0.112884 | | 0.100704 | 0.101371 | 0.099576 | | 0.100979 | 0.104266 |
| 0.097592 | 0.106615 |  | 0.1215 | 0.101976 | 0.112568 |  | 0.1063 | 0.103597 | 0.104651 |
| NA | NA | 0.109489 | | 0.098799 | 0.10176 | NA |  | NA | NA |
| NA | NA | NA |  | NA | NA | NA |  | NA | NA |
| NA | NA | NA |  | NA | NA | NA |  | NA | NA |
| NA | NA | NA |  | NA | NA | NA |  | NA | NA |
| NA | NA | NA |  | NA | NA | NA |  | NA | NA |
| 0.09826 | 0.096486 | NA |  | NA | NA | NA |  | NA | 0.091365 |
| 0.097179 | 0.096164 | NA |  | NA | NA | NA |  | NA | 0.091564 |
| NA | NA | NA |  | NA | NA | NA |  | NA | NA |
| NA | NA | NA |  | NA | NA | NA |  | NA | NA |
| NA | NA | NA |  | NA | NA | NA |  | NA | NA |
| NA | NA | NA |  | NA | NA | NA |  | NA | NA |
| 0.097261 | 0.095958 | NA |  | NA | NA | NA |  | NA | 0.091905 |
| 0.100565 | 0.099512 | NA |  | 0.090851 | NA | NA |  | NA | 0.095104 |
| 0.094587 | 0.092162 | NA |  | 0.091192 | NA | NA |  | NA | NA |
| 0.09938 | 0.095156 | NA |  | 0.091554 | NA | NA |  | NA | NA |
| 0.101981 | 0.101454 | 0.093385 | | 0.091677 | NA | NA |  | 0.096815 | 0.09801 |
| 0.097745 | 0.097975 | NA |  | NA | NA | NA |  | NA | 0.093724 |
| NA | NA | NA |  | NA | NA | NA |  | NA | NA |
| 0.130054 | 0.11929 | 0.201618 | | 0.111713 | 0.21387 | 0.201313 | | 0.211675 | 0.213813 |
| 0.092449 | NA | 0.093892 | | 0.095776 | 0.110106 | NA |  | 0.105638 | 0.105997 |
| 0.092449 | NA | 0.093892 | | 0.095776 | 0.110106 | NA |  | 0.105638 | 0.105997 |
| NA | NA | NA |  | 0.091912 | NA | NA |  | 0.093859 | 0.098061 |
| NA | NA | NA |  | 0.091912 | NA | NA |  | 0.093859 | 0.098061 |
| NA | NA | NA |  | 0.088595 | 0.104393 | NA |  | 0.097539 | 0.098779 |
| NA | NA | 0.092389 | | 0.091521 | 0.119229 | NA |  | 0.107312 | 0.108883 |
| NA | NA | NA |  | 0.090113 | NA | NA |  | NA | NA |
| NA | NA | NA |  | NA | NA | NA |  | NA | NA |
| NA | NA | NA |  | NA | NA | NA |  | NA | NA |
| NA | NA | NA |  | NA | NA | NA |  | NA | NA |
| 0.092308 | NA | NA |  | NA | NA | NA |  | NA | NA |
| 0.093367 | NA | NA |  | NA | NA | NA |  | NA | NA |
| 0.091512 | NA | NA |  | NA | NA | NA |  | NA | NA |
| 0.091684 | NA | NA |  | NA | NA | NA |  | NA | NA |
| 0.118926 | 0.118793 | 0.099594 | | 0.093312 | 0.103719 | 0.104159 | | 0.100038 | 0.095697 |
| 0.109308 | 0.110211 | 0.108605 | | 0.104211 | 0.113338 | 0.119391 | | 0.102382 | 0.101558 |
| 0.109308 | 0.110211 | 0.108605 | | 0.104211 | 0.113338 | 0.119391 | | 0.102382 | 0.101558 |

| 0.111689 | 0.113535 | 0.117787 | 0.117037 | 0.13054 | 0.132833 | 0.106381 | 0.106593 |
| --- | --- | --- | --- | --- | --- | --- | --- |
| NA | NA | NA | 0.113709 | NA | 0.107804 | NA | NA |
| 0.111523 | 0.112599 | 0.115218 | 0.109879 | 0.121655 | 0.12793 | 0.117343 | 0.113551 |
| 0.116932 | 0.117855 | 0.099357 | 0.093072 | 0.103869 | 0.109942 | NA | NA |
| 0.111607 | 0.111228 | 0.113615 | 0.100757 | 0.115951 | 0.11529 | 0.111846 | 0.108911 |
| NA | NA | 0.120812 | NA | NA | NA | NA | NA |
| NA | NA | NA | NA | NA | NA | NA | NA |
| NA | NA | NA | NA | NA | NA | NA | NA |
| 0.09719 | 0.101121 | NA | NA | NA | NA | NA | NA |
| NA | 0.094413 | NA | 0.093329 | NA | NA | NA | NA |
| 0.104305 | 0.109052 | 0.096713 | NA | 0.100816 | 0.100223 | 0.093569 | NA |
| 0.093157 | NA | NA | NA | NA | NA | NA | NA |
| NA | NA | NA | NA | NA | NA | NA | NA |
| NA | NA | NA | NA | NA | NA | NA | NA |
| NA | NA | NA | NA | NA | NA | NA | NA |
| NA | NA | NA | 0.092472 | NA | NA | NA | NA |
| 0.091572 | NA | NA | NA | NA | NA | NA | NA |
| 0.097528 | 0.090662 | NA | NA | NA | NA | NA | NA |
| 0.094384 | NA | NA | NA | NA | NA | NA | NA |
| NA | NA | NA | NA | NA | NA | NA | 0.091004 |
| NA | NA | NA | NA | NA | NA | NA | NA |
| 0.094187 | NA | NA | NA | NA | NA | NA | NA |
| 0.093837 | NA | NA | NA | NA | NA | NA | NA |
| 0.096829 | NA | NA | NA | NA | NA | NA | NA |
| NA | NA | NA | NA | NA | NA | NA | NA |
| NA | NA | NA | 0.090836 | NA | NA | 0.092936 | 0.092558 |
| NA | NA | NA | NA | NA | NA | NA | NA |
| NA | NA | NA | 0.095162 | NA | NA | NA | NA |
| 0.090847 | NA | NA | NA | NA | NA | NA | NA |
| 0.090626 | NA | NA | NA | NA | NA | NA | NA |
| 0.100506 | 0.098217 | NA | 0.090045 | NA | NA | NA | NA |
| 0.092672 | NA | NA | NA | NA | NA | NA | NA |
| NA | NA | NA | NA | NA | NA | NA | NA |
| NA | NA | NA | NA | NA | NA | NA | NA |
| NA | NA | NA | NA | NA | NA | NA | NA |
| NA | NA | NA | 0.095817 | NA | NA | NA | NA |
| NA | NA | NA | NA | NA | NA | NA | NA |
| NA | NA | NA | 0.097139 | NA | NA | NA | 0.094617 |
| NA | NA | NA | 0.096418 | NA | NA | NA | NA |

| 0.09682 | NA | NA | NA | NA | NA | NA | NA |  |
| --- | --- | --- | --- | --- | --- | --- | --- | --- |
| NA | NA | NA | NA | NA | NA | 0.09406 | 0.092502 | |
| 0.091461 | NA | NA | NA | NA | NA | NA | NA |  |
| NA | NA | NA | NA | NA | NA | NA | NA |  |
| NA | NA | NA | NA | NA | NA | NA | NA |  |
| NA | NA | NA | 0.096908 | NA | NA | NA | 0.097588 | |
| NA | NA | NA | NA | NA | NA | NA | NA |  |
| NA | NA | NA | 0.09428 | NA | NA | NA | NA |  |
| 0.093335 | NA | NA | NA | NA | NA | NA | NA |  |
| 0.091838 | NA | NA | 0.096114 | NA | NA | NA | 0.096345 | |
| 0.09806 | 0.093096 | 0.131125 | 0.103756 | 0.117049 | 0.114562 | 0.136098 | 0.134184 | |
| 0.118065 | 0.100996 | 0.095847 | 0.113797 | NA | NA | 0.096306 | 0.118752 | |
| NA | NA | 0.098462 | 0.105961 | NA | NA | 0.102875 | 0.111335 | |
| 0.105258 | 0.091202 | 0.116517 | 0.117056 | 0.098549 | 0.107292 | 0.124388 | 0.12943 | |
| 0.117593 | 0.109225 | 0.151324 | 0.144107 | 0.123873 | 0.149944 | 0.158339 | 0.165469 | |
| 0.100491 | 0.096942 | 0.111923 | 0.113315 | 0.103805 | 0.111878 | 0.11458 | 0.118801 | |
| NA | NA | 0.102491 | 0.117528 | NA | 0.101063 | NA | 0.09875 | |
| 0.096688 | NA | 0.097891 | 0.111039 | NA | NA | 0.101353 | 0.108418 | |
| 0.105803 | 0.098836 | 0.12675 | 0.119621 | 0.10487 | 0.119518 | 0.12488 | 0.130486 | |
| 0.098252 | NA | 0.117368 | 0.118521 | NA | 0.105165 | 0.118565 | 0.126509 | |
| NA | 0.100273 | 0.112673 | 0.101045 | 0.103468 | NA | NA | NA |  |
| NA | 0.100235 | 0.113982 | 0.100005 | 0.104656 | 0.100325 | NA | 0.095824 | |
| 0.095318 | 0.103264 | 0.119167 | 0.101015 | 0.10677 | 0.102375 | 0.09817 | 0.101983 | |
| NA | NA | 0.10655 | 0.100237 | NA | NA | NA | NA |  |
| NA | NA | NA | 0.093735 | NA | NA | 0.097308 | 0.099493 | |
| 0.103199 | 0.104875 | 0.101988 | 0.094036 | NA | 0.09703 | 0.09949 | 0.101361 | |
| NA | NA | 0.10336 | 0.099361 | 0.106524 | 0.103215 | 0.096217 | 0.097047 | |
| NA | NA | 0.10594 | 0.110219 | 0.11091 | 0.106813 | 0.104909 | 0.10485 | |
| NA | NA | 0.10594 | 0.110219 | 0.11091 | 0.106813 | 0.104909 | 0.10485 | |
| NA | NA | 0.093271 | 0.099715 | 0.09719 | 0.092994 | 0.096129 | 0.097869 | |
| NA | NA | 0.091744 | 0.096474 | 0.092525 | 0.08914 | 0.106209 | 0.104944 | |
| NA | NA | 0.094429 | 0.095009 | NA | NA | 0.097585 |  | 0.099 |
| NA | NA | 0.094429 | 0.095009 | NA | NA | 0.097585 |  | 0.099 |
| NA | NA | 0.094429 | 0.095009 | NA | NA | 0.097585 |  | 0.099 |
| NA | NA | 0.094144 | 0.095068 | 0.096174 | 0.090074 | 0.107642 | 0.104652 | |
| 0.106437 | 0.106748 | 0.106004 | 0.093974 | 0.116554 | 0.114432 | 0.113733 | 0.106569 | |
| NA | NA | NA | 0.096837 | NA | NA | NA | NA |  |
| 0.096225 | 0.090007 | NA | NA | NA | NA | NA | NA |  |
| 0.098454 | 0.097414 | NA | 0.096345 | NA | NA | 0.112063 | 0.119251 | |

| 0.09882 | | NA | NA | NA | NA | NA | NA | NA |  |
| --- | --- | --- | --- | --- | --- | --- | --- | --- | --- |
| 0.090634 | | NA | NA | NA | NA | NA | NA | NA |  |
| 0.090961 | | NA | NA | NA | NA | NA | NA | NA |  |
| 0.09115 | | NA | NA | NA | NA | NA | NA | NA |  |
| 0.156511 | | 0.14843 | 0.116614 | 0.09489 | 0.12825 | 0.142371 | 0.123895 | 0.109305 | |
| 0.112369 | | 0.117845 | 0.113134 | 0.095991 | 0.120223 | 0.115778 | 0.111099 | 0.109133 | |
| 0.10727 | | 0.111355 | 0.104536 | 0.094567 | 0.111548 | 0.110574 | 0.102673 | 0.098105 | |
| 0.134114 | | 0.134341 | 0.118653 | 0.098398 | 0.129727 | 0.130565 | 0.122471 | 0.118016 | |
| 0.099159 | | 0.103094 | 0.110211 | NA | 0.112701 | 0.112437 | 0.099325 | NA |  |
| 0.107701 | | 0.109957 | 0.112841 | NA | 0.116489 | 0.115051 | 0.10587 | 0.102724 | |
|  | 0.1035 | 0.109171 | 0.11322 | 0.11887 | 0.110645 | 0.120866 | 0.114227 | 0.113464 | |
| 0.125628 | | 0.125851 | 0.124418 | NA | 0.14887 | 0.135695 | 0.114441 | 0.110024 | |
| 0.140798 | | 0.135061 | 0.133988 | NA | 0.153986 | 0.146263 | 0.119629 | 0.12052 | |
| 0.111328 | | 0.109867 | 0.098385 | 0.096422 | 0.100897 | 0.106558 | 0.105732 |  | 0.102 |
| 0.099305 | | 0.093461 | NA | NA | NA | NA | NA | NA |  |
| 0.092739 | | NA | NA | NA | NA | NA | NA | NA |  |
| NA |  | 0.098235 | 0.107947 | 0.125978 | 0.102549 | 0.114944 | 0.110503 | 0.110195 | |
| 0.092902 | | NA | NA | NA | NA | NA | NA | NA |  |
| 0.141929 | | 0.134447 | 0.109254 | 0.098678 | 0.117173 | 0.127184 | 0.127118 | 0.113445 | |
| 0.099766 | | 0.094435 | NA | NA | NA | NA | NA | NA |  |
| 0.099766 | | 0.094435 | NA | NA | NA | NA | NA | NA |  |
| 0.119321 | | 0.119037 | 0.115987 | 0.103124 | 0.120085 | 0.122872 | 0.116157 | 0.117438 | |
| 0.107532 | | 0.115346 | 0.116593 | 0.122139 | 0.113347 | 0.123862 | 0.114083 | 0.112655 | |
| 0.107532 | | 0.115346 | 0.116593 | 0.122139 | 0.113347 | 0.123862 | 0.114083 | 0.112655 | |
| 0.107532 | | 0.115346 | 0.116593 | 0.122139 | 0.113347 | 0.123862 | 0.114083 | 0.112655 | |
| 0.107532 | | 0.115346 | 0.116593 | 0.122139 | 0.113347 | 0.123862 | 0.114083 | 0.112655 | |
| 0.118221 | | 0.117529 | 0.117512 | 0.100194 | 0.119916 | 0.117573 | 0.12404 | 0.11865 | |
| 0.118221 | | 0.117529 | 0.117512 | 0.100194 | 0.119916 | 0.117573 | 0.12404 | 0.11865 | |
| 0.118221 | | 0.117529 | 0.117512 | 0.100194 | 0.119916 | 0.117573 | 0.12404 | 0.11865 | |
| 0.118221 | | 0.117529 | 0.117512 | 0.100194 | 0.119916 | 0.117573 | 0.12404 | 0.11865 | |
| 0.114008 | | 0.106052 | 0.111442 | 0.116083 | 0.11061 | 0.106636 | 0.118703 | 0.118145 | |
| 0.114008 | | 0.106052 | 0.111442 | 0.116083 | 0.11061 | 0.106636 | 0.118703 | 0.118145 | |
| 0.110867 | | 0.104339 | 0.110363 | 0.10992 | 0.105929 | 0.104242 | 0.11693 | 0.116378 | |
| 0.110867 | | 0.104339 | 0.110363 | 0.10992 | 0.105929 | 0.104242 | 0.11693 | 0.116378 | |
| 0.113674 | | 0.118684 | 0.122388 | 0.132228 | 0.125523 | 0.13142 | 0.12186 | 0.122146 | |
| 0.154395 | | 0.147198 | 0.113594 | 0.097672 | 0.124565 | 0.142292 | 0.133003 | 0.113804 | |
| 0.154395 | | 0.147198 | 0.113594 | 0.097672 | 0.124565 | 0.142292 | 0.133003 | 0.113804 | |
| 0.139826 | | 0.133717 | 0.114539 | 0.097706 | 0.117651 | 0.129383 | 0.13017 | 0.117707 | |
| 0.139826 | | 0.133717 | 0.114539 | 0.097706 | 0.117651 | 0.129383 | 0.13017 | 0.117707 | |

| 0.141495 | 0.135099 | 0.112913 | 0.098185 | 0.118957 | 0.138404 | 0.131205 | 0.114684 |
| --- | --- | --- | --- | --- | --- | --- | --- |
| 0.157203 | 0.151518 | 0.118078 | 0.09823 | 0.131817 | 0.14815 | 0.13745 | 0.117822 |
| 0.14667 | 0.138371 | 0.110384 | 0.098113 | 0.115869 | 0.131056 | 0.121881 | 0.106858 |
| 0.121078 | 0.11853 | 0.102907 | 0.094664 | 0.100181 | 0.109688 | 0.108957 | 0.099225 |
| 0.102555 | 0.108575 | 0.121554 | 0.097046 | 0.113242 | 0.109591 | 0.107876 | 0.106067 |
| NA | 0.097443 | 0.111014 | 0.101128 | 0.10258 | NA | NA | NA |
| 0.105053 | 0.112327 | 0.122142 | 0.096718 | 0.119871 | 0.116004 | 0.108618 | 0.105991 |
| 0.173335 | 0.173255 | 0.114817 | NA | 0.118373 | 0.113896 | 0.099243 | 0.096988 |
| 0.178372 | 0.177622 | 0.117669 | NA | 0.121794 | 0.116142 | 0.099711 | 0.097272 |
| 0.125556 | 0.122013 | 0.095359 | NA | NA | NA | NA | NA |
| 0.181429 | 0.179728 | 0.116247 | 0.092984 | 0.119705 | 0.115326 | 0.099463 | 0.09732 |
| 0.158258 | 0.152393 | 0.106337 | 0.092783 | 0.105609 | 0.106882 | 0.10098 | 0.09846 |
| NA | NA | NA | 0.100378 | NA | NA | NA | NA |
| NA | 0.093813 | 0.10773 | 0.101268 | 0.102397 | 0.107961 | 0.103526 | 0.099718 |
| NA | NA | NA | 0.100936 | NA | NA | NA | NA |
| NA | NA | NA | NA | NA | NA | NA | NA |
| NA | NA | NA | NA | NA | NA | NA | NA |
| 0.095441 | 0.09729 | NA | 0.093381 | NA | NA | 0.094299 | 0.09536 |
| NA | NA | NA | NA | NA | NA | NA | NA |
| NA | NA | NA | NA | NA | NA | NA | NA |
| NA | NA | NA | NA | NA | NA | NA | NA |
| NA | NA | NA | NA | NA | NA | NA | NA |
| NA | NA | NA | 0.093222 | NA | NA | NA | NA |
| NA | NA | NA | 0.092963 | NA | NA | 0.098795 | 0.100277 |
| NA | NA | 0.102204 | 0.095381 | 0.097715 | NA | 0.106906 | 0.10689 |
| 0.092738 | NA | NA | NA | NA | NA | NA | NA |
| 0.091968 | NA | NA | NA | NA | NA | NA | NA |
| 0.09449 | NA | NA | NA | NA | NA | NA | NA |
| 0.112232 | 0.116443 | 0.119773 | 0.132239 | 0.117184 | 0.12545 | 0.12114 | 0.120563 |
| 0.108036 | 0.108631 | 0.104124 | 0.103526 | 0.104401 | 0.100244 | 0.112843 | 0.111047 |
| 0.108036 | 0.108631 | 0.104124 | 0.103526 | 0.104401 | 0.100244 | 0.112843 | 0.111047 |
| NA | NA | NA | 0.094037 | NA | NA | NA | NA |
| NA | NA | NA | NA | NA | NA | NA | NA |
| NA | NA | NA | 0.094155 | NA | NA | NA | NA |
| NA | NA | NA | 0.094533 | NA | NA | NA | NA |
| 0.109622 | 0.105892 | 0.138643 | 0.096572 | 0.148291 | 0.140943 | 0.130419 | 0.132546 |
| NA | NA | NA | 0.093186 | NA | NA | NA | NA |
| NA | NA | NA | 0.094901 | NA | NA | NA | NA |
| NA | NA | NA | 0.094281 | NA | NA | NA | NA |

| NA |  | NA |  | NA | 0.094813 | NA | NA | 0.09143 | | NA |
| --- | --- | --- | --- | --- | --- | --- | --- | --- | --- | --- |
| NA |  | NA |  | NA | 0.090241 | NA | NA | NA |  | NA |
| NA |  | NA |  | NA | 0.091571 | NA | NA | NA |  | NA |
| NA |  | NA |  | NA | 0.093449 | NA | NA | NA |  | NA |
| NA |  | NA |  | NA | NA | NA | NA | NA |  | NA |
| 0.113296 | | 0.116609 | | 0.112783 | 0.095936 | 0.120675 | 0.114328 | 0.11205 | | 0.110438 |
| NA |  | NA |  | NA | NA | NA | NA | NA |  | NA |
| NA |  | NA |  | NA | NA | NA | NA | NA |  | NA |
| NA |  | NA |  | NA | NA | NA | NA | NA |  | NA |
| NA |  | NA |  | NA | NA | NA | NA | NA |  | NA |
| 0.106472 | | 0.109693 | | 0.110868 | 0.115695 | 0.11941 | 0.157072 | 0.10215 | | 0.104187 |
| NA |  | 0.099753 | | 0.101872 | 0.10607 | NA | 0.11622 | NA |  | NA |
| 0.100989 | | 0.108655 | | 0.114944 | 0.104312 | 0.11505 | 0.115652 |  | 0.1038 | 0.10508 |
|  | 0.0921 | NA |  | NA | NA | NA | NA | NA |  | NA |
| 0.100514 | | 0.102399 | | 0.100786 | 0.101708 | 0.103371 | 0.129281 | NA |  | NA |
| 0.100966 | | 0.100025 | | 0.098732 | 0.104448 | NA | 0.111824 | 0.096526 | | 0.099736 |
| 0.107501 | | 0.107811 | | 0.099664 | 0.101301 | NA | 0.103343 | 0.095113 | | 0.097233 |
| 0.105351 | | 0.107886 | | 0.101101 | 0.093253 | 0.101855 | 0.118456 | NA |  | NA |
| 0.094198 | | NA |  | NA | NA | NA | NA | NA |  | NA |
| NA |  | 0.098308 | | 0.105036 | 0.094029 | 0.117161 | 0.116533 | 0.103776 | | NA |
| 0.101549 | | 0.104337 | | 0.106631 | 0.096149 | 0.121677 | 0.119744 | 0.111725 | | 0.102208 |
| NA |  |  | 0.0932 | 0.096364 | 0.09389 | NA | NA | 0.095079 | | 0.09291 |
| NA |  | 0.092726 | | 0.092314 | 0.094347 | 0.092489 | NA | 0.092864 | | NA |
| 0.120884 | | 0.117911 | | 0.135548 | NA | 0.142532 | 0.143287 | 0.123506 | | 0.129266 |
| 0.11761 | | 0.117507 | | 0.105125 | 0.097952 | 0.10705 | 0.111258 | 0.112754 | | 0.108054 |
| NA |  | NA |  | 0.094315 | 0.096197 | NA | NA | 0.091773 | | NA |
| NA |  | 0.095419 | | 0.108901 | 0.102757 | 0.102793 | 0.107945 | 0.105246 | | 0.10397 |
| 0.096222 | | 0.095912 | | 0.099146 | 0.100186 | 0.096384 | 0.098242 | 0.096454 | | NA |
| 0.114116 | | 0.117103 | | 0.11469 | 0.100304 | 0.114475 | 0.11646 | 0.11114 | | 0.10861 |
| 0.111754 | | 0.11158 | | 0.10115 | 0.095111 | 0.105642 | 0.107612 | 0.099211 | | 0.099005 |
| 0.146756 | | 0.140938 | | 0.115681 | 0.103983 | 0.123815 | 0.117175 | 0.118508 | | 0.114125 |
| 0.123582 | | 0.120935 | | 0.10745 | 0.098303 | 0.110137 | 0.111759 | 0.104674 | | 0.105077 |
| 0.133166 | | 0.132388 | | 0.116372 | 0.100793 | 0.126317 | 0.122023 | 0.119137 | | 0.116523 |
| 0.11875 | | 0.118642 | | 0.105787 | 0.094518 | 0.110395 | 0.109613 | 0.106811 | | 0.103861 |
| 0.129752 | | 0.126541 | | 0.106326 | 0.098257 | 0.107216 | 0.108307 | 0.107955 | | 0.11101 |
| 0.122529 | | 0.118683 | | 0.097057 | 0.096682 | NA | 0.102776 | 0.099046 | | 0.102362 |
| NA |  | NA |  | NA | NA | NA | NA | NA |  | NA |
| NA |  | NA |  | NA | NA | NA | NA | NA |  | NA |
| NA |  | NA |  | NA | NA | NA | NA | NA |  | NA |

| NA |  | NA | 0.132716 | 0.104723 | 0.130339 | 0.133562 |  | 0.1683 | 0.161615 |
| --- | --- | --- | --- | --- | --- | --- | --- | --- | --- |
| NA |  | 0.095815 | 0.139185 | 0.105294 | 0.135814 | 0.138866 | 0.178588 | | 0.171573 |
| NA |  | 0.094254 | 0.126137 | 0.098472 | 0.126542 | 0.128049 | 0.154781 | | 0.149739 |
| NA |  | NA | NA | 0.093669 | NA | NA | NA |  | NA |
| NA |  | NA | NA | 0.092823 | NA | NA | NA |  | NA |
| NA |  | NA | NA | 0.093157 | NA | NA | NA |  | NA |
| NA |  | NA | NA | 0.092999 | NA | NA | NA |  | NA |
| NA |  | NA | 0.096771 | 0.098875 | NA | NA | 0.095488 | | 0.097808 |
| 0.115722 | | 0.119175 | 0.103969 | 0.093744 | 0.108791 | 0.108821 | 0.105946 | | 0.100765 |
| 0.117642 | | 0.121409 | 0.103108 | 0.09586 | 0.110504 | 0.110233 | 0.104538 | | 0.101891 |
| 0.123083 | | 0.124879 | 0.104056 | 0.093276 | 0.112857 | 0.110751 | 0.104085 | | 0.101082 |
| 0.123083 | | 0.124879 | 0.104056 | 0.093276 | 0.112857 | 0.110751 | 0.104085 | | 0.101082 |
| 0.120398 | | 0.122883 | 0.106779 | 0.097152 | 0.114829 | 0.112697 | 0.107269 | | 0.10477 |
| NA |  | 0.096231 | 0.111315 | 0.098545 | 0.101106 | 0.099725 | NA |  | NA |
| NA |  | 0.096231 | 0.111315 | 0.098545 | 0.101106 | 0.099725 | NA |  | NA |
| 0.115558 | | 0.118893 | NA | 0.089826 | NA | NA | 0.097167 | | 0.098544 |
| 0.117958 | | 0.116905 | NA | 0.091723 | NA | NA | 0.094726 | | 0.097386 |
| 0.120355 | | 0.129427 | NA | NA | NA | NA | 0.093314 | | 0.094702 |
| NA |  | NA | 0.092371 | 0.096526 | 0.090784 | 0.08325 | 0.102326 | | 0.101714 |
| 0.121307 | | 0.111284 | 0.157754 | 0.105832 | 0.163389 | 0.154045 | 0.163614 | | 0.168486 |
| 0.105343 | | 0.09595 | 0.209116 | 0.131058 | 0.17834 | 0.175439 | 0.214248 | | 0.201918 |
| 0.098673 | | 0.089418 | 0.138959 | 0.107837 | 0.113326 | 0.110627 | 0.147191 | | 0.146498 |
| 0.108486 | | 0.103044 | 0.159258 | 0.109232 | 0.150488 | 0.144536 | 0.165211 | | 0.161232 |
| 0.105519 | | 0.09793 | 0.203652 | 0.123351 | 0.172443 | 0.169197 | 0.197748 | | 0.188572 |
|  | 0.1045 | 0.09414 | 0.174131 | 0.117381 | 0.149929 | 0.143893 | 0.177932 | | 0.172927 |
| 0.102236 | | 0.101154 | 0.106473 | 0.096666 | 0.104147 | 0.099226 | 0.104799 | | 0.101923 |
| 0.102619 | | 0.091656 | 0.119232 | 0.114588 | NA | 0.107341 | 0.122254 | | 0.128403 |
| 0.110282 | | 0.109089 | 0.105925 | 0.095145 | 0.108125 | 0.099793 | 0.106393 | | 0.105244 |
| 0.110449 | | 0.106704 | 0.099074 | 0.099194 | NA | 0.103149 | 0.106632 | | 0.100245 |
| 0.144033 | | 0.137919 | 0.111698 | 0.098541 | 0.124764 | 0.141025 | 0.131202 | | 0.116038 |
| 0.155283 | | 0.146322 | 0.118958 | 0.098934 | 0.126621 | 0.135595 | 0.13402 | | 0.121171 |
| 0.112374 | | 0.104203 | NA | 0.098403 | NA | 0.119544 | 0.117593 | | 0.10095 |
| 0.09712 | | 0.094232 | NA | 0.094192 | NA | NA | 0.105173 | | 0.113211 |
| NA |  | NA | NA | 0.090464 | NA | NA | NA |  | NA |
| NA |  | NA | NA | NA | NA | NA | NA |  | NA |
| 0.097081 | | 0.102201 | 0.104156 | NA | 0.118045 | 0.118851 | 0.096482 | | NA |
| 0.108065 | | 0.111771 | 0.110558 | 0.094202 | 0.124539 | 0.125168 | 0.105777 | | 0.09736 |
| 0.105418 | | 0.10926 | 0.111414 | NA | 0.129521 | 0.130631 |  | 0.1046 | NA |
| NA |  | NA | NA | NA | NA | NA | NA |  | NA |

| 0.10537 | 0.109929 | 0.109862 | | NA | 0.127586 | | 0.124756 | 0.104692 | 0.096768 |
| --- | --- | --- | --- | --- | --- | --- | --- | --- | --- |
| 0.14907 | 0.148284 | 0.118577 | | 0.096191 | 0.131553 | | 0.140131 | 0.127126 | 0.113526 |
| 0.099147 | 0.105761 | 0.111826 | | 0.119563 | 0.106339 | | 0.115901 | 0.10874 | 0.109179 |
| 0.098784 | 0.104909 | 0.112279 | | 0.116458 | 0.106107 | | 0.113311 | 0.106931 | 0.106456 |
| 0.096001 | 0.102989 | 0.107729 | | 0.109494 | 0.102381 | | 0.109495 | 0.102158 | 0.101289 |
| 0.118129 | 0.116865 | 0.105447 | | 0.096019 | 0.110746 | | 0.119059 | 0.114063 | 0.107634 |
| 0.130141 | 0.130905 | 0.101939 | | NA | 0.10016 | | 0.102881 | NA | NA |
| 0.100083 | 0.107869 | 0.113721 | | 0.115221 | 0.10694 | | 0.117179 | 0.108722 | 0.10561 |
| 0.095928 | 0.103222 | 0.108287 | | 0.111711 | 0.104921 | | 0.113755 | 0.101436 | 0.098531 |
| 0.095391 | NA | NA |  | NA | NA |  | NA | NA | NA |
| 0.097764 | NA | NA |  | NA | NA |  | NA | NA | NA |
| 0.095028 | NA | NA |  | NA | NA |  | NA | NA | NA |
| 0.121665 | 0.124156 | 0.132754 | | 0.094572 | 0.149366 | | 0.146765 | 0.128212 | 0.130877 |
| 0.114547 | 0.115783 | 0.115314 | | 0.107236 | 0.126467 | | 0.124068 | 0.11202 | 0.111149 |
| 0.112062 | 0.107203 | 0.095733 | | 0.094285 | NA |  | 0.105617 | 0.10905 | 0.098293 |
| 0.104489 | 0.107435 | 0.110033 | | 0.108962 | 0.118622 | | 0.121548 | 0.106788 | 0.104264 |
| 0.109986 | 0.106104 | 0.095375 | | 0.094178 | NA |  | 0.105144 | 0.10862 | 0.097433 |
| NA | NA | NA |  | 0.100997 | 0.103267 | | 0.102322 | 0.102728 | 0.099976 |
| 0.103464 | 0.105952 | 0.103402 | | 0.093758 | 0.115496 | | 0.114043 | 0.099805 | NA |
| 0.089486 | NA | NA |  | NA | NA |  | NA | NA | NA |
| 0.137948 | 0.134075 | 0.127314 | | 0.104474 | 0.14455 | | 0.137118 | 0.120171 | 0.118472 |
| 0.104959 | 0.102264 | 0.096806 | | 0.095941 | NA |  | NA | 0.096301 | 0.095888 |
| NA | NA | NA |  | NA | 0.107618 | | 0.107247 | NA | NA |
| NA | NA | NA |  | 0.094838 | 0.107454 | | 0.104597 | NA | NA |
| 0.099499 | 0.10226 | 0.107368 | | 0.095936 | 0.117573 | | 0.11742 | 0.110225 | 0.103073 |
| 0.099179 | 0.103043 | 0.104425 | | NA | 0.105533 | | 0.103201 | 0.112183 | 0.106268 |
| 0.10587 | 0.106998 |  | 0.1104 | 0.093006 | 0.118106 | | 0.119653 | 0.113043 | 0.105488 |
| 0.102866 | 0.107627 | 0.110327 | | NA | 0.121279 | | 0.123558 | 0.106388 | 0.102786 |
| 0.096838 | 0.09775 | 0.096134 | | 0.097561 | 0.098931 | | 0.095857 | 0.102947 | 0.099726 |
| 0.117434 | 0.115136 | 0.118568 | | 0.105964 | 0.123303 | | 0.121235 | 0.114216 | 0.114244 |
| 0.103445 | 0.102522 | 0.105505 | | 0.108125 | 0.108098 | | 0.10125 | 0.115302 | 0.114334 |
| 0.130992 | 0.133186 | 0.123932 | | 0.101567 | 0.138432 | | 0.136432 | 0.121361 | 0.119758 |
| 0.115545 | 0.115948 | 0.108789 | | 0.09702 | 0.106066 | | 0.105355 | 0.108999 | 0.108405 |
| 0.116585 | 0.114136 | 0.106242 | | 0.100611 | 0.102072 | | 0.102886 | 0.111431 | 0.108445 |
| 0.09917 | 0.098176 | 0.108734 | | 0.099989 |  | 0.1122 | 0.112977 | 0.098646 | 0.102771 |
| 0.103812 | 0.099053 | 0.109543 | | 0.102418 | 0.108227 | | 0.102699 | 0.112433 | 0.113345 |
| 0.106307 | 0.099363 | 0.124425 | | 0.103415 | 0.119896 | | 0.114387 | 0.123322 | 0.125019 |
| 0.113393 | 0.115855 | 0.106822 | | 0.096645 | 0.118239 | | 0.11254 | 0.109019 | 0.103941 |
| 0.123373 | 0.122218 | 0.118759 | | 0.092977 | 0.137294 | | 0.132505 | 0.111641 | 0.118205 |

| 0.118825 | | 0.121068 | 0.118173 | | NA | 0.116967 | | 0.117561 | 0.116246 | 0.113551 | |
| --- | --- | --- | --- | --- | --- | --- | --- | --- | --- | --- | --- |
| 0.112413 | | 0.114572 | 0.111947 | | 0.094585 | 0.115206 | | 0.116063 | 0.116986 | 0.111769 | |
| 0.121405 | | 0.119163 | 0.116093 | | 0.110351 |  | 0.1156 | 0.117497 | 0.115901 | 0.114654 | |
| 0.105525 | | 0.108726 | 0.107123 | | 0.094993 | 0.102721 | | 0.108091 | 0.110153 |  | 0.1082 |
| 0.127922 | | 0.110702 | 0.140078 | | 0.110872 | 0.131512 | | 0.126518 | 0.121985 | 0.128283 | |
| 0.112019 | | NA | NA |  | 0.097845 | NA |  | NA | NA | 0.099629 | |
| 0.121815 | | 0.122814 | 0.114518 | | 0.095959 | 0.125091 | | 0.125089 | 0.117638 | 0.112939 | |
| 0.126575 | | 0.125202 | 0.106786 | | NA | NA |  | NA | 0.10711 | 0.100422 | |
| 0.11885 | | 0.12218 | 0.110794 | | 0.098451 | 0.121255 | | 0.119947 | 0.107426 | 0.104333 | |
| 0.110426 | | 0.111525 | 0.108633 | | NA | 0.106313 | | 0.109969 | 0.112205 | 0.109492 | |
| 0.109077 | | 0.113194 | 0.104141 | | 0.093652 | 0.110679 | | 0.105539 | 0.107477 | 0.104374 | |
| NA |  | NA | NA |  | NA | NA |  | NA | NA | NA |  |
| 0.110785 | | 0.107507 | 0.110069 | | 0.097364 | 0.108174 | | 0.107301 | 0.10891 | 0.109669 | |
| NA |  | NA | 0.09855 | | 0.097533 | 0.106946 | | 0.103279 | 0.105525 | 0.101567 | |
| 0.096331 | | 0.097628 | 0.104946 | | 0.102268 | 0.11277 | | 0.110994 | 0.110534 | 0.107008 | |
| 0.14499 | | 0.143852 | 0.123728 | | 0.096222 | 0.143696 | | 0.146418 | 0.125357 | 0.119292 | |
| 0.107381 | | 0.110272 | 0.097538 | | 0.095658 | 0.107366 | | 0.109684 | 0.099189 | 0.096848 | |
| 0.121811 | | 0.125527 | 0.111338 | | 0.093944 | 0.129168 | | 0.133271 | 0.115017 | 0.108832 | |
| 0.100565 | | NA | NA |  | 0.093009 | NA |  | NA | NA | 0.09582 | |
| 0.104378 | | 0.104057 | 0.104627 | | 0.095343 |  | 0.0998 | 0.101517 | 0.10787 | 0.10596 | |
| 0.110532 | | 0.114419 | 0.105273 | | 0.100281 | 0.104432 | | 0.103216 | 0.117019 | 0.116219 | |
| 0.097458 | | NA | 0.097093 | | 0.104466 | NA |  | NA | 0.10724 | 0.112669 | |
| 0.113561 | | 0.114064 | 0.107386 | | 0.09542 | 0.114879 | | 0.111051 | 0.103223 | 0.096955 | |
| 0.095621 | | 0.094822 |  | 0.1091 | 0.094359 | 0.107312 | | 0.098818 | 0.115157 | 0.111618 | |
| 0.117821 | | 0.117545 | 0.106685 | | 0.093615 | NA |  | NA | 0.104391 | 0.10052 | |
|  | 0.1071 | 0.098146 | 0.113089 | | 0.108958 | 0.107149 | | 0.104555 | 0.115408 | 0.117012 | |
| 0.119302 | | 0.121243 | 0.110714 | | 0.096997 | 0.108209 | | 0.105632 | 0.106812 | 0.105583 | |
| 0.097407 | | 0.099151 | 0.094715 | | 0.094791 | 0.101725 | | 0.096702 | 0.108222 | 0.103014 | |
| 0.110135 | | 0.115718 | 0.103372 | | 0.095824 | 0.108262 | | 0.108273 | 0.104462 | 0.103681 | |
| 0.109912 | | 0.110058 | 0.100247 | | 0.096404 | NA |  | 0.099389 | 0.100379 | 0.102859 | |
| 0.098867 | | 0.098085 | 0.110112 | | 0.100322 | 0.104301 | | 0.100637 | 0.115729 | 0.113674 | |
| 0.120365 | | 0.118359 | 0.11871 | | 0.106355 | 0.136267 | | 0.131162 | 0.11352 | 0.111266 | |
| 0.100489 | | 0.103425 | 0.101309 | | 0.095769 | 0.110961 | | 0.107668 | 0.101937 | 0.095392 | |
| NA |  | 0.098029 | 0.107596 | | 0.108197 | 0.104686 | | 0.10371 | 0.120047 | 0.119864 | |
| NA |  | NA | 0.101853 | | 0.098297 | NA |  | NA | NA | NA |  |
| 0.108955 | | 0.111219 | 0.103605 | | 0.095422 | 0.106978 | | 0.108262 | 0.103863 | 0.102767 | |
| 0.111211 | | 0.112171 | 0.111303 | | 0.100149 | 0.113689 | | 0.112837 | 0.113446 | 0.112628 | |
| 0.106912 | | 0.106304 | 0.10051 | | 0.099324 | 0.099986 | | 0.098494 | 0.112557 | 0.11102 | |

cd4_positi cd4_positi cd4_positi cd4_positi cd4positiv cd8_positi cd8_positi cd8positiv

0.11293 0.105596 0.116072 0.108997 0.118298 0.117922 0.116484 0.120074

NA	NA	NA	NA	NA	NA	NA	NA

0.113213	0.11713 0.115004 0.113324 0.116032 0.116214 0.114615 0.115158

0.111483 0.114518 0.107665 0.104968 0.111971 0.136099 0.107331 0.114625

0.118554 0.102189 0.100086 0.101496	0.10659 0.116977 0.099885 0.107784

0.115875 0.115552	0.13312 0.116526 0.124051 0.134188 0.134583 0.126572

0.125281 0.111621 0.110066 0.111496 0.114126 0.123547 0.110025 0.116554

0.116883 0.103607 0.104259 0.102365 0.108413 0.113014 0.104447 0.109392

0.116883 0.103607 0.104259 0.102365 0.108413 0.113014 0.104447 0.109392

0.098516 NA	NA	0.100959 NA	NA	NA	NA

0.109841 0.106129	0.11765 0.108945 0.113005	0.11545 0.118557 0.112342

0.110308 0.099946 0.114598 0.103759 0.110682 0.115143 0.115405 0.109706

0.107968 0.105528 0.099567 0.110043 0.102306 0.105605 0.099129 0.101234

0.1144 0.106362 0.102041 0.111956 0.105511 0.113821 0.102647 0.105424

NA	NA	NA	NA	NA	NA	NA	NA NA	NA	NA	NA	NA	NA	NA	NA

0.096114 0.103104 NA	0.108028 NA	NA	NA	NA

0.096114 0.103104 NA	0.108028 NA	NA	NA	NA

0.115564 0.148732 0.149421 0.150971	0.14154 0.111778 0.149205 0.138756

0.121533 0.115381	0.11609 0.113414 0.117416 0.120605 0.115732 0.117665

NA	NA	NA	NA	NA	NA	NA	NA

0.126292 0.151414 0.159921 0.155158 0.145181	0.14527	0.16359 0.150829

NA	NA	NA	NA	NA	NA	NA	NA NA	0.092928 NA	NA	NA	NA	NA	NA NA	0.094807 NA	0.093678 NA	NA	NA	NA NA	NA	NA	NA	NA	NA	NA	NA NA	NA	NA	NA	NA	NA	NA	NA NA	NA	NA	NA	NA	NA	NA	NA

0.119007 0.115062 0.114406 0.116024 0.115994 0.115045 0.114143 0.115698

0.119007 0.115062 0.114406 0.116024 0.115994 0.115045 0.114143 0.115698

0.149231 0.134803 0.129713 0.130122 0.132996 0.139425 0.128701 0.132741

0.147477 0.136278 0.131758 0.132748 0.134102 0.137364 0.131365 0.133717

NA	0.105083 NA	0.099526 NA	NA	NA	NA NA	0.095722 NA	NA	NA	NA	NA	NA NA	NA	NA	NA	NA	NA	NA	NA NA	NA	NA	NA	NA	NA	NA	NA NA	NA	NA	NA	NA	NA	NA	NA

0.105324 0.101657 0.108436	0.10062 0.112297 0.124044 0.108577 0.112823

| 0.106277 | NA | 0.093343 | NA | 0.094972 | 0.099533 | 0.094611 | 0.096077 |
| --- | --- | --- | --- | --- | --- | --- | --- |
| 0.106277 | NA | 0.093343 | NA | 0.094972 | 0.099533 | 0.094611 | 0.096077 |
| 0.106277 | NA | 0.093343 | NA | 0.094972 | 0.099533 | 0.094611 | 0.096077 |
| 0.119573 | 0.100739 | 0.11277 | 0.112841 | 0.11814 | 0.102473 | 0.112794 | 0.120635 |
| 0.115925 | NA | 0.109151 | 0.106213 | 0.114557 | 0.106934 | 0.109278 | 0.118425 |
| 0.137172 | 0.164927 | 0.175562 | 0.166033 | 0.170565 | 0.145696 | 0.175707 | 0.171134 |
| 0.141766 | 0.192868 | 0.189943 | 0.183871 | 0.180557 | 0.170388 | 0.188927 | 0.179965 |
| 0.15172 | 0.210341 | 0.210604 | 0.201985 | 0.201671 | 0.188979 | 0.210523 | 0.201577 |
| 0.145974 | 0.182054 | 0.192364 | 0.182835 | 0.185574 | 0.15808 | 0.193056 | 0.18621 |
| 0.13888 | 0.184862 | 0.180125 | 0.174348 | 0.171684 | 0.164281 | 0.179253 | 0.172492 |
| 0.141435 | 0.193045 | 0.188452 | 0.181796 | 0.180831 | 0.179283 | 0.188157 | 0.183403 |
| 0.141625 | 0.20684 | 0.200548 | 0.193222 | 0.185907 | 0.169042 | 0.19973 | 0.185685 |
| 0.141658 | 0.193355 | 0.183108 | 0.177918 | 0.180321 | 0.190741 | 0.182709 | 0.181702 |
| 0.14626 | 0.200101 | 0.196327 | 0.192608 | 0.18953 | 0.189536 | 0.195539 | 0.190699 |
| 0.151277 | 0.212315 | 0.205818 | 0.199468 | 0.199051 | 0.204366 | 0.205789 | 0.200781 |
| NA | NA | NA | NA | NA | NA | NA | NA |
| 0.123273 | 0.103846 | 0.101745 | 0.104672 | 0.108158 | 0.107998 | 0.102458 | 0.107928 |
| 0.151157 | 0.213959 | 0.208629 | 0.20517 | 0.201068 | 0.195651 | 0.209284 | 0.202621 |
| 0.158056 | 0.22452 | 0.220107 | 0.21547 | 0.210131 | 0.210814 | 0.220376 | 0.212116 |
| 0.130425 | 0.162224 | 0.149895 | 0.149653 | 0.145372 | 0.158128 | 0.151149 | 0.146694 |
| 0.161505 | 0.225069 | 0.231381 | 0.221533 | 0.219993 | 0.203143 | 0.232146 | 0.222135 |
| NA | NA | NA | NA | NA | NA | NA | NA |
| NA | NA | NA | NA | NA | NA | NA | NA |
| 0.158571 | 0.224744 | 0.22564 | 0.218877 | 0.214517 | 0.20443 | 0.225289 | 0.215966 |
| 0.128508 | 0.14757 | 0.148985 | 0.142741 | 0.150102 | 0.144804 | 0.148166 | 0.149152 |
| 0.133746 | 0.171198 | 0.176807 | 0.170654 | 0.169812 | 0.15489 | 0.17649 | 0.171768 |
| 0.155884 | 0.221758 | 0.220005 | 0.212477 | 0.211267 | 0.209385 | 0.220197 | 0.21313 |
| 0.148936 | 0.217643 | 0.206788 | 0.204635 | 0.195827 | 0.188924 | 0.206909 | 0.197103 |
| 0.127768 | 0.167024 | 0.158649 | 0.158214 | 0.155665 | 0.159844 | 0.158709 | 0.157246 |
| 0.127768 | 0.167024 | 0.158649 | 0.158214 | 0.155665 | 0.159844 | 0.158709 | 0.157246 |
| 0.158128 | 0.226508 | 0.226527 | 0.218145 | 0.215692 | 0.207138 | 0.226587 | 0.218526 |
| 0.136238 | 0.172236 | 0.174731 | 0.170127 | 0.172317 | 0.173174 | 0.174573 | 0.173601 |
| 0.126107 | 0.151913 | 0.157861 | 0.150273 | 0.154691 | 0.142843 | 0.157732 | 0.154121 |
| 0.139288 | 0.166653 | 0.173875 | 0.169215 | 0.167364 | 0.156772 | 0.172869 | 0.167745 |
| NA | NA | NA | NA | NA | NA | NA | NA |
| 0.108663 | 0.099708 | 0.114085 | 0.105551 | 0.110296 | 0.116351 | 0.114334 | 0.110429 |
| 0.154853 | 0.226688 | 0.222988 | 0.218384 | 0.208658 | 0.192671 | 0.221687 | 0.209248 |
| 0.161484 | 0.230959 | 0.232921 | 0.22408 | 0.221447 | 0.21124 | 0.232942 | 0.223669 |
| 0.134541 | 0.167146 | 0.15521 | 0.156315 | 0.149057 | 0.162758 | 0.155495 | 0.151636 |

| 0.112194 | 0.106087 | 0.098474 | 0.112616 | 0.100191 | NA | 0.097428 | 0.099763 |
| --- | --- | --- | --- | --- | --- | --- | --- |
| 0.108842 | 0.109843 | 0.102674 | 0.115977 | 0.103575 | NA | 0.101874 | 0.100582 |
| 0.114233 | 0.111016 | 0.104376 | 0.117565 | 0.107686 | 0.107703 | 0.103829 | 0.106073 |
| 0.111016 | 0.099672 | NA | 0.105212 | NA | NA | NA | NA |
| NA | NA | NA | NA | NA | NA | NA | NA |
| NA | NA | NA | NA | NA | NA | NA | NA |
| NA | NA | NA | NA | NA | NA | NA | NA |
| NA | 0.09534 | NA | NA | NA | NA | NA | NA |
| NA | 0.093333 | NA | 0.091568 | NA | NA | NA | NA |
| NA | 0.09461 | NA | 0.091575 | NA | NA | NA | NA |
| NA | NA | NA | NA | NA | NA | NA | NA |
| NA | NA | NA | NA | NA | NA | NA | NA |
| NA | NA | NA | NA | NA | NA | NA | NA |
| NA | NA | NA | NA | NA | NA | NA | NA |
| NA | 0.094261 | NA | 0.092101 | NA | NA | NA | NA |
| NA | 0.097793 | 0.093423 | 0.095674 | NA | NA | 0.092541 | NA |
| NA | 0.096727 | NA | 0.092989 | NA | NA | NA | NA |
| NA | 0.095624 | NA | NA | NA | NA | NA | NA |
| NA | 0.099933 | 0.096349 | 0.097927 | 0.094482 | NA | 0.096049 | 0.093082 |
| NA | 0.096523 | 0.092333 | 0.093183 | NA | NA | NA | NA |
| NA | NA | NA | NA | NA | NA | NA | NA |
| 0.154011 | 0.210679 | 0.210518 | 0.201857 | 0.203941 | 0.207132 | 0.21019 | 0.205619 |
| NA | 0.105966 | 0.107131 | 0.10559 | 0.102536 | NA | 0.104306 | 0.100537 |
| NA | 0.105966 | 0.107131 | 0.10559 | 0.102536 | NA | 0.104306 | 0.100537 |
| NA | 0.098983 | 0.096714 | 0.098689 | NA | NA | 0.094382 | NA |
| NA | 0.098983 | 0.096714 | 0.098689 | NA | NA | 0.094382 | NA |
| NA | 0.101418 | 0.098928 | 0.098767 | NA | NA | 0.095876 | NA |
| NA | 0.110943 | 0.109057 | 0.107451 | 0.101452 | NA | 0.105603 | 0.098668 |
| NA | 0.092652 | NA | 0.092078 | NA | NA | NA | NA |
| NA | 0.094101 | NA | NA | NA | NA | NA | NA |
| NA | 0.094101 | NA | NA | NA | NA | NA | NA |
| NA | 0.094101 | NA | NA | NA | NA | NA | NA |
| NA | NA | NA | NA | NA | NA | NA | NA |
| NA | NA | NA | NA | NA | NA | NA | NA |
| NA | NA | NA | NA | NA | NA | NA | NA |
| NA | NA | NA | NA | NA | NA | NA | NA |
| 0.099049 | NA | 0.098687 | 0.096323 | 0.099039 | 0.105256 | 0.098765 | 0.099153 |
| 0.118067 | NA | 0.102444 | 0.097511 | 0.107225 | 0.120459 | 0.102543 | 0.108676 |
| 0.118067 | NA | 0.102444 | 0.097511 | 0.107225 | 0.120459 | 0.102543 | 0.108676 |

| 0.127066 | 0.107591 | 0.106597 | 0.102857 | 0.113134 | 0.138485 | 0.106348 | 0.112786 |
| --- | --- | --- | --- | --- | --- | --- | --- |
| 0.105063 | NA | NA | NA | NA | NA | NA | NA |
| 0.118815 | 0.10736 | 0.116175 | 0.108694 | 0.116871 | 0.125453 | 0.116585 | 0.119855 |
| 0.105786 | NA | NA | NA | 0.098037 | 0.111279 | NA | 0.100338 |
| 0.112623 | 0.105168 | 0.110516 | 0.108374 | 0.112075 | 0.114754 | 0.111449 | 0.11335 |
| NA | NA | NA | NA | NA | NA | NA | NA |
| NA | NA | NA | NA | NA | NA | NA | NA |
| NA | NA | NA | NA | NA | NA | NA | NA |
| 0.104595 | NA | NA | NA | NA | NA | NA | NA |
| 0.103116 | NA | NA | NA | NA | NA | NA | NA |
| 0.111261 | NA | 0.093093 | 0.092213 | 0.094349 | NA | 0.093422 | 0.094348 |
| NA | NA | NA | NA | NA | NA | NA | NA |
| NA | NA | NA | NA | NA | NA | NA | NA |
| NA | NA | NA | NA | NA | NA | NA | NA |
| NA | NA | NA | NA | NA | NA | NA | NA |
| NA | NA | NA | NA | NA | NA | NA | NA |
| NA | NA | NA | NA | NA | NA | NA | NA |
| NA | NA | NA | NA | NA | NA | NA | NA |
| NA | NA | NA | NA | NA | NA | NA | NA |
| NA | 0.101922 | NA | 0.093396 | NA | NA | NA | NA |
| NA | NA | NA | NA | NA | NA | NA | NA |
| NA | NA | NA | NA | NA | NA | NA | NA |
| NA | NA | NA | NA | NA | NA | NA | NA |
| NA | NA | NA | NA | NA | NA | NA | NA |
| NA | 0.093822 | NA | NA | NA | NA | NA | NA |
| NA | 0.103569 | NA | 0.09567 | NA | NA | NA | NA |
| NA | NA | NA | NA | NA | NA | NA | NA |
| NA | 0.099475 | NA | NA | NA | NA | NA | NA |
| NA | NA | NA | NA | NA | NA | NA | NA |
| NA | NA | NA | NA | NA | NA | NA | NA |
| NA | 0.094885 | NA | 0.094162 | NA | NA | NA | NA |
| NA | NA | NA | NA | NA | NA | NA | NA |
| NA | 0.090126 | NA | NA | NA | NA | NA | NA |
| NA | NA | NA | NA | NA | NA | NA | NA |
| NA | NA | NA | NA | NA | NA | NA | NA |
| NA | 0.105338 | NA | 0.096362 | NA | NA | NA | NA |
| NA | NA | NA | NA | NA | NA | NA | NA |
| NA | 0.10566 | NA | 0.096006 | NA | NA | NA | NA |
| NA | 0.100772 | NA | NA | NA | NA | NA | NA |

| NA | NA | NA | NA |  | NA | NA |  | NA |  | NA |
| --- | --- | --- | --- | --- | --- | --- | --- | --- | --- | --- |
| NA | 0.103909 | NA | 0.095538 | | NA | NA |  | NA |  | NA |
| NA | NA | NA | NA |  | NA | NA |  | NA |  | NA |
| NA | NA | NA | NA |  | NA | NA |  | NA |  | NA |
| NA | NA | NA | NA |  | NA | NA |  | NA |  | NA |
| NA | 0.10869 | NA | 0.099366 | | NA | NA |  | NA |  | NA |
| NA | NA | NA | NA |  | NA | NA |  | NA |  | NA |
| NA | 0.099696 | NA | NA |  | NA | NA |  | NA |  | NA |
| NA | NA | NA | NA |  | NA | NA |  | NA |  | NA |
| NA | 0.106012 | NA | 0.096599 | | NA | NA |  | NA |  | NA |
| 0.108709 | 0.130665 | 0.134142 |  | 0.1323 | 0.129873 | 0.103235 | | 0.134113 | | 0.128915 |
| NA | 0.127786 | 0.096347 | 0.116761 | | 0.095453 | NA |  | 0.096519 | | 0.094692 |
| NA | 0.128325 | 0.103814 | 0.116506 | | 0.097445 | NA |  | 0.102968 | | NA |
| 0.099143 | 0.144241 | 0.123931 | 0.134391 | | 0.116907 | 0.097294 | | 0.122804 | | 0.114137 |
| 0.138357 | 0.209692 | 0.154456 | 0.187556 | | 0.145392 | 0.138161 | | 0.153063 | | 0.140072 |
| 0.106386 | 0.136831 | 0.114319 | 0.124473 | | 0.111573 | 0.107367 | | 0.113903 | | 0.108394 |
| 0.104295 | 0.107797 | 0.094721 | 0.100377 | | 0.09525 | 0.104103 | | 0.094821 | | 0.095888 |
| NA | 0.128899 | 0.101524 | 0.115977 | | 0.097458 | NA |  | 0.100092 | | 0.093758 |
| 0.108069 | 0.151016 | 0.124531 | 0.139191 | | 0.11899 |  | 0.1131 | 0.123876 | | 0.115139 |
| 0.101081 | 0.15712 | 0.119141 | 0.140442 | | 0.112032 | NA |  | 0.117345 | | 0.10886 |
| 0.111332 | 0.107743 | NA | 0.114484 | | 0.097208 | NA |  | NA |  | 0.096746 |
| 0.107702 | 0.103234 | NA | 0.10963 | | 0.096905 | NA |  | NA |  | 0.097424 |
| 0.111238 | 0.110284 | 0.099956 | 0.116472 | | 0.103198 | 0.103536 | | 0.099094 | | 0.102016 |
| 0.108413 | 0.100062 | NA | 0.107258 | | NA | NA |  | NA |  | NA |
| 0.109962 | NA | 0.097872 |  | 0.0969 | 0.106098 | NA |  | 0.099265 | | 0.108086 |
| 0.105145 | NA | 0.09876 | 0.101605 | | 0.102559 | NA |  | 0.099896 | | 0.104092 |
| 0.120831 | NA | NA | 0.097234 | | 0.10261 | 0.107041 | | 0.096929 | | 0.10518 |
| 0.117969 | NA | 0.104047 | 0.104163 | | 0.10989 | 0.102513 | | 0.104921 | | 0.112024 |
| 0.117969 | NA | 0.104047 | 0.104163 | | 0.10989 | 0.102513 | | 0.104921 | | 0.112024 |
| 0.112187 | NA | 0.096243 | NA |  | 0.103506 | 0.094547 | | 0.097362 | | 0.107849 |
| 0.114532 | NA | 0.106154 | 0.101563 | | 0.11215 | 0.087969 | | 0.106973 | | 0.116138 |
| 0.111369 | NA | 0.097167 | 0.096072 | | 0.106199 | NA |  | 0.09924 | | 0.109434 |
| 0.111369 | NA | 0.097167 | 0.096072 | | 0.106199 | NA |  | 0.09924 | | 0.109434 |
| 0.111369 | NA | 0.097167 | 0.096072 | | 0.106199 | NA |  | 0.09924 | | 0.109434 |
| 0.109814 | NA | 0.105523 | 0.100748 | | 0.111691 | 0.087336 | | 0.106456 | | 0.114705 |
| 0.117065 | NA | 0.111015 | 0.100928 | | 0.112071 | 0.112718 | | 0.112693 | | 0.116919 |
| 0.108446 | NA | NA | NA |  | 0.099714 | NA |  | NA |  | 0.102925 |
| NA | NA | NA | NA |  | NA | NA |  | NA |  | NA |
| NA | 0.11781 | 0.11714 | 0.116874 | | 0.106965 | 0.099514 | |  | 0.1147 | 0.106426 |

| NA | NA | NA | NA |  | NA | NA |  | NA | NA |
| --- | --- | --- | --- | --- | --- | --- | --- | --- | --- |
| NA | NA | NA | NA |  | NA | NA |  | NA | NA |
| NA | NA | NA | NA |  | NA | NA |  | NA | NA |
| NA | NA | NA | NA |  | NA | NA |  | NA | NA |
| 0.109677 | 0.102382 | 0.11808 | 0.105638 | | 0.113748 | 0.128791 | | 0.120156 | 0.114866 |
| 0.108536 | 0.108426 | 0.110502 | 0.109094 | | 0.110643 | 0.117416 | | 0.110255 | 0.111037 |
| 0.107212 | NA | 0.101317 | 0.097294 | | 0.102711 | 0.106256 | | 0.101652 | 0.102904 |
| 0.109548 | 0.117418 | 0.121959 | 0.115169 | | 0.118516 | 0.133365 | | 0.122251 | 0.118001 |
| 0.099838 | NA | 0.098333 | 0.09603 | | 0.100484 |  | 0.1142 | 0.098277 | 0.100255 |
| 0.101668 | 0.100344 | 0.105135 | 0.102241 | | 0.105312 | 0.117207 | | 0.105022 | 0.104796 |
| 0.123371 | 0.110755 | 0.114909 | 0.11352 | | 0.116832 | 0.117662 | | 0.114159 | 0.117561 |
| 0.122169 | 0.108379 | 0.110869 | 0.10125 | | 0.111461 | 0.138318 | | 0.111644 | 0.114943 |
| 0.115482 | 0.123309 | 0.118545 | 0.112785 | | 0.12414 | 0.150036 | | 0.118555 | 0.126619 |
| 0.101919 | 0.100541 | 0.104215 | 0.102561 | | 0.102016 | 0.102117 | | 0.104589 | 0.102465 |
| NA | NA | NA | NA |  | NA | NA |  | NA | NA |
| NA | NA | NA | NA |  | NA | NA |  | NA | NA |
| 0.121227 | 0.108944 | 0.110616 | 0.110248 | | 0.112752 | 0.112727 | | 0.109801 | 0.11252 |
| NA | NA | NA | NA |  | NA | NA |  | NA | NA |
| 0.107232 | 0.109682 | 0.122147 | 0.110711 | | 0.115367 | 0.12062 | | 0.122318 | 0.114667 |
| NA | NA | NA | NA |  | NA | NA |  | NA | NA |
| NA | NA | NA | NA |  | NA | NA |  | NA | NA |
| 0.112849 | 0.118079 | 0.116441 | 0.113635 | | 0.117563 | 0.130091 | | 0.116929 | 0.118868 |
| 0.125304 | 0.110177 | 0.114284 | 0.112906 | | 0.119282 | 0.118397 | | 0.113795 | 0.119998 |
| 0.125304 | 0.110177 | 0.114284 | 0.112906 | | 0.119282 | 0.118397 | | 0.113795 | 0.119998 |
| 0.125304 | 0.110177 | 0.114284 | 0.112906 | | 0.119282 | 0.118397 | | 0.113795 | 0.119998 |
| 0.125304 | 0.110177 | 0.114284 | 0.112906 | | 0.119282 | 0.118397 | | 0.113795 | 0.119998 |
| 0.11119 | 0.120262 | 0.121423 | 0.116429 | | 0.117888 | 0.111058 | | 0.122425 | 0.117959 |
| 0.11119 | 0.120262 | 0.121423 | 0.116429 | | 0.117888 | 0.111058 | | 0.122425 | 0.117959 |
| 0.11119 | 0.120262 | 0.121423 | 0.116429 | | 0.117888 | 0.111058 | | 0.122425 | 0.117959 |
| 0.11119 | 0.120262 | 0.121423 | 0.116429 | | 0.117888 | 0.111058 | | 0.122425 | 0.117959 |
| 0.102835 | 0.128984 | 0.117091 |  | 0.1183 | 0.112006 | 0.109701 | | 0.116851 | 0.111566 |
| 0.102835 | 0.128984 | 0.117091 |  | 0.1183 | 0.112006 | 0.109701 | | 0.116851 | 0.111566 |
| 0.103507 | 0.120799 | 0.115125 | 0.113947 | | 0.110731 | 0.103547 | | 0.115473 | 0.109743 |
| 0.103507 | 0.120799 | 0.115125 | 0.113947 | | 0.110731 | 0.103547 | | 0.115473 | 0.109743 |
| 0.137525 | 0.125583 | 0.121461 | 0.123129 | | 0.12661 | 0.130867 | | 0.121369 | 0.127206 |
| 0.111335 | 0.108624 | 0.125071 | 0.109292 | | 0.117519 | 0.128305 | | 0.125974 | 0.118183 |
| 0.111335 | 0.108624 | 0.125071 | 0.109292 | | 0.117519 | 0.128305 | | 0.125974 | 0.118183 |
| 0.105615 | 0.113475 | 0.125092 | 0.114089 | | 0.116517 | 0.118314 | | 0.125753 | 0.116467 |
| 0.105615 | 0.113475 | 0.125092 | 0.114089 | | 0.116517 | 0.118314 | | 0.125753 | 0.116467 |

| 0.111505 | 0.105521 | | 0.124958 | 0.109048 | 0.117584 | 0.125454 | 0.125704 | 0.118572 |
| --- | --- | --- | --- | --- | --- | --- | --- | --- |
| 0.115445 | 0.109321 | | 0.129527 | 0.113466 | 0.120992 | 0.13273 | 0.131057 | 0.123401 |
| 0.114179 | 0.102844 | | 0.117012 | 0.105634 | 0.112968 | 0.120221 | 0.117535 | 0.112701 |
| 0.099959 | 0.097818 | | 0.105841 | 0.099058 | 0.101651 | 0.100564 | 0.105468 | 0.100687 |
| 0.103372 | 0.112833 | | 0.107489 | 0.113998 | 0.106075 | 0.106892 | 0.106984 | 0.105736 |
| 0.111047 | 0.104507 | | NA | 0.112753 | NA | NA | NA | NA |
| 0.106658 | 0.10774 | | 0.107687 | 0.111088 | 0.107931 | 0.112162 | 0.108284 | 0.107468 |
| 0.112912 | NA |  | 0.096405 | 0.099515 | 0.108966 | 0.111067 | 0.096725 | 0.107314 |
| 0.114937 |  | 0.0979 | 0.096884 | 0.100199 | 0.109187 | 0.113698 | 0.096952 | 0.108166 |
| 0.106845 | NA |  | NA | NA | NA | NA | NA | NA |
| 0.115135 | 0.098685 | | 0.096802 | 0.09996 | 0.108724 | 0.113426 | 0.097155 | 0.10812 |
| 0.101264 | 0.101014 | | 0.099537 | 0.099847 | 0.102261 | 0.105687 | 0.099376 | 0.102767 |
| 0.108803 | NA |  | NA | NA | NA | NA | NA | NA |
| 0.111257 | NA |  | 0.101128 | 0.103263 | 0.103306 | 0.102157 | 0.102242 | 0.105692 |
| 0.104305 | NA |  | NA | NA | NA | NA | NA | NA |
| NA | NA |  | NA | NA | NA | NA | NA | NA |
| NA | NA |  | NA | NA | NA | NA | NA | NA |
| 0.094664 | NA |  | 0.095064 | 0.097431 | 0.095066 | NA | 0.095584 | 0.095015 |
| NA | NA |  | NA | NA | NA | NA | NA | NA |
| NA | NA |  | NA | NA | NA | NA | NA | NA |
| NA | NA |  | NA | NA | NA | NA | NA | NA |
| NA | NA |  | NA | NA | NA | NA | NA | NA |
| NA | NA |  | NA | NA | NA | NA | NA | NA |
| 0.09459 | 0.099046 | | 0.099473 | 0.102424 | 0.098395 | NA | 0.099303 | 0.09854 |
| 0.101358 | 0.10188 | | 0.106805 | 0.106893 | 0.105746 | NA | 0.106936 | 0.106869 |
| NA | NA |  | NA | NA | NA | NA | NA | NA |
| NA | NA |  | NA | NA | NA | NA | NA | NA |
| NA | NA |  | NA | NA | NA | NA | NA | NA |
| 0.132276 | 0.123501 | | 0.120959 | 0.121506 | 0.124231 | 0.12297 | 0.120758 | 0.124683 |
| 0.11053 | 0.097683 | | 0.112344 | 0.107155 | 0.11525 | 0.099725 | 0.112332 | 0.116293 |
| 0.11053 | 0.097683 | | 0.112344 | 0.107155 | 0.11525 | 0.099725 | 0.112332 | 0.116293 |
| NA | NA |  | NA | NA | NA | NA | NA | NA |
| 0.105284 | NA |  | NA | NA | NA | NA | NA | NA |
| 0.097928 | NA |  | NA | NA | NA | NA | NA | NA |
| 0.101343 | NA |  | NA | NA | 0.094833 | NA | NA | 0.094712 |
| 0.117821 | 0.132029 | | 0.129837 | 0.125867 | 0.131342 | 0.143901 | 0.129811 | 0.132583 |
| 0.10303 | NA |  | NA | NA | NA | NA | NA | NA |
| 0.09587 | NA |  | NA | NA | NA | NA | NA | NA |
| 0.104227 | NA |  | NA | NA | NA | NA | NA | NA |

| 0.101228 | NA | 0.09252 | 0.094233 | NA | NA | 0.09221 | | NA |  |
| --- | --- | --- | --- | --- | --- | --- | --- | --- | --- |
| 0.094257 | NA | NA | NA | NA | NA | NA |  | NA |  |
| NA | NA | NA | NA | NA | NA | NA |  | NA |  |
| 0.094798 | NA | NA | NA | NA | NA | NA |  | NA |  |
| 0.106545 | NA | NA | NA | NA | NA | NA |  | NA |  |
| 0.10658 | 0.110287 | 0.112343 | 0.111627 | 0.110446 | 0.116715 | 0.111506 | | 0.109692 | |
| NA | NA | NA | NA | NA | NA | NA |  | NA |  |
| NA | NA | NA | NA | NA | NA | NA |  | NA |  |
| NA | NA | NA | NA | NA | NA | NA |  | NA |  |
| NA | NA | NA | NA | NA | NA | NA |  | NA |  |
| 0.102904 | 0.108176 | 0.102992 | 0.103287 | 0.105359 | 0.121042 |  | 0.1031 | 0.105706 | |
| 0.097004 | NA | NA | 0.093864 | 0.094787 | 0.101891 | NA |  | 0.095784 | |
| 0.103534 | 0.107601 | 0.105236 | 0.106721 | 0.105023 | 0.107936 | 0.10439 | | 0.106446 | |
| NA | NA | NA | NA | NA | NA | NA |  | NA |  |
| 0.098424 | NA | NA | NA | NA | 0.107203 | NA |  | 0.095369 | |
| NA | 0.101864 | 0.098197 | 0.100239 | 0.097034 | NA | 0.098023 | | NA |  |
| 0.10147 | NA | 0.096254 | 0.098125 | 0.099331 | NA | 0.096419 | | 0.097821 | |
| 0.102677 | NA | NA | NA | NA | 0.106272 | NA |  | NA |  |
| NA | NA | NA | NA | NA | NA | NA |  | NA |  |
| 0.136922 | NA | 0.099765 | NA | 0.103613 | 0.111851 | 0.102373 | | 0.103927 | |
| 0.123596 | NA | 0.107949 | 0.100332 | 0.109884 | 0.11781 | 0.110285 | | 0.113624 | |
| 0.101993 | NA | 0.094318 | 0.094341 | 0.097323 | NA | 0.094969 | | 0.096543 | |
| 0.103941 | NA | 0.093069 | 0.093398 | 0.095443 | NA | 0.093085 | | 0.095268 | |
| 0.102769 | 0.132656 | 0.125243 | 0.113744 | 0.127938 | 0.149586 | 0.125077 | | 0.128234 | |
| 0.105457 | 0.101844 | 0.111989 | 0.107122 | 0.110518 | 0.110778 | 0.11208 | |  | 0.1107 |
| 0.104433 | NA | NA | NA | 0.093661 | NA | NA |  | 0.094036 | |
| 0.112535 | 0.100611 | 0.103927 | 0.108218 | 0.105911 | 0.105078 | 0.104087 | | 0.107694 | |
| 0.104749 | NA | 0.095094 | 0.096962 | 0.097564 | NA | 0.094981 | | 0.097942 | |
| 0.110471 | 0.107874 | 0.110484 | 0.108385 | 0.109211 | 0.113381 | 0.110783 | | 0.111207 | |
| 0.101817 | 0.098242 | 0.098928 | 0.099477 | 0.09995 | 0.106204 | 0.099465 | | 0.101787 | |
| 0.107326 | 0.117121 | 0.117308 | 0.111502 | 0.113714 | 0.121288 | 0.114044 | | 0.113164 | |
| 0.104284 | 0.109105 | 0.104046 | 0.106609 | 0.104679 | 0.110582 | 0.105066 | | 0.106258 | |
| 0.109634 | 0.119391 | 0.116914 | 0.116218 | 0.114929 | 0.12305 | 0.117101 | | 0.11523 | |
| 0.105222 | 0.099249 | 0.104928 | 0.102184 | 0.106121 | 0.107576 | 0.105492 | | 0.106497 | |
| 0.101833 | 0.114841 | 0.107757 | 0.111372 | 0.106958 | 0.113534 | 0.108929 | | 0.108181 | |
| NA | 0.109112 | 0.099179 | 0.104305 | 0.101693 | NA | 0.100045 | | 0.100492 | |
| NA | NA | NA | NA | NA | NA | NA |  | NA |  |
| NA | NA | NA | NA | NA | NA | NA |  | NA |  |
| NA | NA | NA | NA | NA | NA | NA |  | NA |  |

| 0.128015 | 0.154271 | 0.16276 | 0.157314 | 0.14753 | 0.143033 | 0.16716 | 0.152464 |
| --- | --- | --- | --- | --- | --- | --- | --- |
| 0.131705 | 0.160371 | 0.172615 | 0.165247 | 0.156395 | 0.149861 | 0.177247 | 0.161334 |
| 0.12284 | 0.140404 | 0.150378 | 0.142066 | 0.140806 | 0.138355 | 0.154658 | 0.146623 |
| 0.102326 | NA | 0.092752 | NA | 0.094922 | NA | 0.092954 | 0.094597 |
| 0.098077 | NA | NA | NA | NA | NA | NA | NA |
| NA | NA | NA | NA | NA | NA | NA | NA |
| 0.096889 | NA | NA | NA | NA | NA | NA | NA |
| NA | 0.100579 | 0.096436 | 0.101097 | 0.095047 | NA | 0.095382 | 0.094039 |
| 0.109549 | NA | 0.105341 | 0.09898 | 0.104665 | 0.105128 | 0.105521 | 0.104992 |
| 0.106037 | 0.097843 | 0.105714 | 0.102722 | 0.105468 | 0.111177 | 0.105179 | 0.106977 |
| 0.105822 | 0.096696 | 0.103939 | 0.101595 | 0.103899 | 0.109478 | 0.103737 | 0.104369 |
| 0.105822 | 0.096696 | 0.103939 | 0.101595 | 0.103899 | 0.109478 | 0.103737 | 0.104369 |
| 0.106274 | 0.104155 | 0.106924 | 0.10435 | 0.106662 | 0.113003 | 0.106921 | 0.10726 |
| 0.106924 | 0.10278 | NA | 0.107566 | 0.09845 | 0.100412 | NA | 0.096973 |
| 0.106924 | 0.10278 | NA | 0.107566 | 0.09845 | 0.100412 | NA | 0.096973 |
| NA | 0.100343 | 0.099075 | 0.098388 | 0.094415 | NA | 0.098333 | 0.09548 |
| NA | 0.098197 | 0.096566 | 0.095779 | NA | NA | 0.095466 | NA |
| NA | 0.095648 | 0.094484 | 0.095864 | NA | NA | 0.094339 | NA |
| 0.110347 | NA | 0.102016 | 0.097533 | 0.109785 | NA | 0.102856 | 0.112398 |
| 0.131096 | 0.170716 | 0.162789 | 0.163292 | 0.158646 | 0.160351 | 0.163198 | 0.159603 |
| 0.127685 | 0.207961 | 0.203506 | 0.190162 | 0.178279 | 0.141461 | 0.202919 | 0.176854 |
| 0.104337 | 0.132064 | 0.144929 | 0.138563 | 0.135999 | 0.095584 | 0.144153 | 0.133092 |
| 0.12126 | 0.156458 | 0.161345 | 0.152431 | 0.151315 | 0.130902 | 0.161647 | 0.149894 |
| 0.124335 | 0.193239 | 0.189681 | 0.177441 | 0.169359 | 0.140108 | 0.188926 | 0.165397 |
| 0.117243 | 0.173533 | 0.171881 | 0.164385 | 0.157135 | 0.126626 | 0.171411 | 0.155069 |
| 0.094847 | 0.108631 | 0.102402 | 0.105439 | 0.09941 | 0.09623 | 0.101683 | 0.096923 |
| 0.098089 | 0.149068 | 0.123292 | 0.135882 | 0.113345 | NA | 0.121199 | 0.110586 |
| 0.09872 | 0.110452 | 0.105259 | 0.109579 | 0.103222 | 0.100785 | 0.104418 | 0.102437 |
| 0.104171 | NA | 0.105002 | 0.099986 | 0.100406 | NA | 0.103406 | 0.099925 |
| 0.114995 | 0.106028 | 0.125371 | 0.112124 | 0.120221 | 0.129543 | 0.125937 | 0.119598 |
| 0.106306 | 0.118996 | 0.128896 | 0.117317 | 0.120378 | 0.127032 | 0.129199 | 0.120167 |
| 0.107033 | NA | 0.112995 | 0.096517 | 0.105797 | 0.111268 | 0.113369 | 0.103676 |
| NA | 0.111611 | 0.110515 | 0.111265 | 0.100814 | NA | 0.108343 | 0.099784 |
| NA | NA | NA | NA | NA | NA | NA | NA |
| NA | NA | NA | NA | NA | NA | NA | NA |
| 0.119021 | NA | NA | NA | 0.098719 | 0.113741 | 0.096118 | 0.10181 |
| 0.129839 | NA | 0.103183 | 0.096526 | 0.106439 | 0.11876 | 0.104395 | 0.108876 |
| 0.124851 | NA | 0.101392 | NA | 0.105532 | 0.120832 | 0.103081 | 0.107328 |
| 0.101441 | NA | NA | NA | NA | 0.103003 | NA | NA |

| 0.126176 | | NA | 0.101636 | NA | 0.106869 | 0.118965 | | 0.103802 | 0.108446 |
| --- | --- | --- | --- | --- | --- | --- | --- | --- | --- |
| 0.113795 | | 0.107807 | 0.122427 | 0.10989 | 0.118465 | 0.132992 | | 0.123346 | 0.119423 |
|  | 0.1211 | 0.109588 | 0.109592 | 0.110278 | 0.112785 | 0.114779 | | 0.109086 | 0.113421 |
| 0.120996 | | 0.107589 | 0.107064 | 0.108658 | 0.109959 | 0.114087 | | 0.106865 | 0.112431 |
| 0.113392 | | 0.100903 | 0.102916 | 0.103354 | 0.104755 | 0.111022 | | 0.102634 | 0.10607 |
| 0.104072 | | 0.105628 | 0.112481 | 0.107295 | 0.10904 | 0.114308 | | 0.112723 | 0.109878 |
| 0.101032 | | NA | NA | NA | 0.096047 |  | 0.1039 | NA | NA |
| 0.122321 | | 0.106176 | 0.107331 | 0.107664 | 0.112112 | 0.114973 | | 0.10783 | 0.112721 |
| 0.121431 | | NA | 0.101272 | 0.101298 | 0.105772 | 0.112267 | | 0.100934 | 0.106955 |
| NA |  | NA | NA | NA | NA | NA |  | NA | NA |
| NA |  | NA | NA | NA | NA | NA |  | NA | NA |
| NA |  | NA | NA | NA | NA | NA |  | NA | NA |
| 0.110265 | | 0.131788 | 0.128482 | 0.116481 | 0.131428 | 0.15189 | | 0.128881 | 0.131886 |
| 0.120333 | | 0.109384 | 0.111793 | 0.108333 | 0.115345 | 0.129631 | | 0.11251 | 0.117381 |
| 0.099421 | | NA | 0.104978 | 0.09685 | 0.09924 | 0.102297 | | 0.105133 | 0.099647 |
| 0.114424 | | 0.103345 | 0.105338 | 0.102613 | 0.109226 | 0.118165 | | 0.105392 | 0.108438 |
| 0.098146 | | NA | 0.104425 | 0.096357 | 0.098266 | 0.100938 | | 0.104744 | 0.09833 |
| 0.112349 | | NA | 0.102025 | 0.099346 | 0.105386 | 0.111754 | | 0.10409 | 0.109096 |
| 0.117499 | | NA | 0.097646 | NA | 0.101351 | 0.112445 | | 0.099089 | 0.102585 |
| NA |  | NA | NA | NA | NA | NA |  | NA | NA |
| 0.11946 | | 0.118908 | 0.118281 | 0.112896 | 0.11789 | 0.139209 | | 0.119188 | 0.119391 |
| 0.101436 | | NA | 0.096267 | 0.097255 | 0.096827 | NA |  | 0.09519 | 0.095234 |
| 0.122895 | | NA | NA | NA | NA | 0.108718 | | NA | NA |
| 0.142884 | | NA | NA | NA | NA | 0.102367 | | NA | NA |
| 0.115632 | | NA | 0.107108 | 0.099376 | 0.111642 | 0.121088 | | 0.10991 | 0.113599 |
| 0.111297 | | NA | 0.110237 | 0.101244 | 0.113457 | 0.104784 | | 0.112051 | 0.117413 |
| 0.110022 | | 0.099396 | 0.110141 | 0.10294 | 0.111814 | 0.124083 | | 0.112406 | 0.114405 |
| 0.107741 | | 0.098886 | 0.105348 | 0.098296 | 0.108779 | 0.126399 | | 0.106872 | 0.11001 |
| 0.110084 | | NA | 0.10159 | 0.097647 | 0.10663 | NA |  | 0.102751 | 0.107585 |
| 0.108284 | | 0.113991 | 0.113797 | 0.107192 | 0.115434 | 0.12116 | | 0.114127 | 0.115204 |
| 0.118548 | | 0.101357 | 0.11517 | 0.109195 | 0.116984 | 0.102596 | | 0.115506 | 0.119624 |
| 0.116224 | | 0.122827 | 0.12075 | 0.115492 | 0.12006 | 0.139237 | | 0.121413 | 0.120506 |
| 0.106231 | | 0.106145 | 0.109339 | 0.106214 | 0.108967 | 0.109877 | | 0.109479 | 0.110059 |
| 0.107852 | | 0.105399 | 0.110687 | 0.10793 | 0.107849 | 0.102712 | | 0.110847 | 0.108747 |
| 0.101352 | | 0.100387 | 0.100405 | NA | 0.103925 | 0.114294 | | 0.09965 | 0.105381 |
| 0.100384 | | 0.116256 | 0.111443 | 0.114303 | 0.109373 | 0.099621 | | 0.110957 | 0.107346 |
| 0.106244 | | 0.130113 | 0.122566 | 0.126597 | 0.118598 | 0.107811 | | 0.121972 | 0.116698 |
| 0.114333 | | 0.102607 | 0.107708 | 0.103637 | 0.109175 | 0.11072 | | 0.108442 | 0.109346 |
| 0.102335 | | 0.120687 | 0.113026 | 0.108458 | 0.112173 | 0.141563 | | 0.113363 | 0.113477 |

| 0.114327 | 0.110597 | | 0.114601 | 0.108786 | | 0.113008 | | 0.123485 | | 0.114749 | 0.114463 |
| --- | --- | --- | --- | --- | --- | --- | --- | --- | --- | --- | --- |
| 0.114017 | 0.107883 | | 0.114208 | 0.108586 | | 0.114219 | | 0.114142 | | 0.115831 | 0.115727 |
| 0.111688 | 0.117851 | | 0.115728 | 0.112757 | | 0.112833 | | 0.116397 | | 0.115602 | 0.112493 |
| 0.107155 | 0.103512 | | 0.109746 | 0.102592 | | 0.110063 | | 0.112269 | | 0.110634 | 0.111112 |
| 0.104171 | 0.150208 | | 0.122837 | 0.124508 | | 0.122537 | |  | 0.1315 | 0.121446 | 0.122699 |
| NA | 0.097456 | | NA | 0.099625 | | NA |  | NA |  | NA | NA |
| 0.10924 | 0.109784 | | 0.116608 | 0.110982 | | 0.114384 | | 0.121175 | | 0.116708 | 0.114468 |
| 0.102411 | NA |  | 0.10506 | 0.098454 | | 0.103628 | | NA |  | 0.105661 | 0.103936 |
| 0.113922 | 0.100297 | | 0.106899 | 0.101177 | |  | 0.1092 | 0.117854 | | 0.108113 | 0.109399 |
| 0.102262 | 0.108155 | | 0.111773 | 0.107572 | | 0.109241 | | 0.108351 | | 0.112813 | 0.110988 |
| 0.109206 | NA |  | 0.107346 | 0.100839 | | 0.110418 | | 0.105507 | | 0.107965 | 0.111032 |
| NA | NA |  | NA | NA |  | NA |  | NA |  | NA | NA |
| 0.108748 | 0.107612 | | 0.109287 | 0.106611 | | 0.109595 | |  | 0.1106 | 0.109523 | 0.108744 |
| 0.10929 | NA |  | 0.103452 | 0.097914 | | 0.107928 | | 0.101485 | | 0.105084 | 0.110695 |
| 0.118533 | NA |  | 0.108837 | 0.101479 | | 0.113817 | | 0.111545 | | 0.110532 | 0.116671 |
| 0.113712 | 0.115506 | | 0.123866 | 0.114032 | | 0.122258 | | 0.144192 | | 0.124844 | 0.123541 |
| 0.103167 | NA |  | 0.099505 | 0.095559 | | 0.101719 | | 0.110734 | | 0.099755 | 0.101326 |
| 0.111088 | 0.106789 | | 0.113141 | 0.104439 | | 0.112747 | | 0.137928 | | 0.114882 | 0.113349 |
| 0.094936 | NA |  | NA | 0.095816 | | NA |  | NA |  | NA | NA |
| 0.103909 | NA |  | 0.107995 | 0.103768 | | 0.107743 | | 0.101605 | | 0.108822 | 0.107348 |
| 0.114237 | 0.103584 | | 0.117612 | 0.110544 | | 0.11686 | | 0.101924 | | 0.118122 | 0.118616 |
| NA | 0.10314 | | 0.108088 | 0.109383 | | 0.102963 | | NA |  | 0.106934 | 0.102223 |
| 0.113496 | NA |  | 0.101005 | 0.096771 | | 0.102011 | | 0.110325 | | 0.102267 | 0.103614 |
| 0.101833 | NA |  | 0.112187 | 0.103222 | | 0.111865 | | 0.090603 | | 0.111676 | 0.113712 |
| 0.107635 | NA |  | 0.103108 | 0.098903 | | 0.107391 | | NA |  | 0.104073 | 0.108098 |
| 0.099695 | 0.118862 | | 0.115491 | 0.115646 | | 0.111305 | | 0.102621 | | 0.114382 | 0.110393 |
| 0.099743 | 0.105508 | | 0.106372 | 0.103594 | | 0.107908 | | 0.109284 | | 0.106636 | 0.106837 |
| 0.111153 | NA |  | 0.105899 | 0.098154 | | 0.109604 | | 0.093996 | | 0.106379 | 0.112434 |
| 0.100678 | 0.101656 | | 0.104659 | 0.102688 | | 0.103567 | | 0.106431 | | 0.104511 | 0.103389 |
| 0.098376 | 0.102634 | | 0.101233 | 0.104174 | | 0.102026 | | NA |  | 0.101722 | 0.10207 |
| 0.105689 | 0.105236 | | 0.114655 | 0.109818 | | 0.114247 | | 0.095527 | | 0.114503 | 0.113724 |
| 0.113777 | 0.112516 | | 0.110742 | 0.103916 | | 0.116543 | | 0.135196 | | 0.112232 | 0.118441 |
| 0.116282 | NA |  | 0.099077 | 0.094888 | | 0.105266 | | 0.103174 | | 0.10109 | 0.10399 |
| 0.103033 | 0.146675 | | 0.118881 |  | 0.1174 | 0.113564 | | 0.109749 | | 0.12001 | 0.113902 |
| 0.100189 | NA |  | NA | NA |  | NA |  | NA |  | NA | 0.097247 |
| 0.103496 | 0.101002 | | 0.103626 | 0.101404 | | 0.104636 | | 0.108205 | | 0.104736 | 0.10564 |
| 0.104884 |  | 0.1127 | 0.113981 | 0.109905 | | 0.113041 | | 0.116212 | | 0.113876 | 0.111359 |
| 0.108896 | NA |  | 0.112424 | 0.104591 | | 0.116889 | | 0.096345 | | 0.113128 | 0.115979 |

central_m central_m central_m class_swit common_l common_ conventioncytotoxic_

0.114242 0.112025 0.115147 0.108575 0.114856 0.116175 0.117916 0.123496

NA	NA	NA	NA	NA	NA	NA	NA

0.116904 0.116698 0.118599 0.121411 0.112702	0.11311	0.11436 0.126312

0.115939 0.110476 0.118616 0.126818 0.108905 0.108922 0.120008 0.136965

0.113541	0.11018 0.122699 0.138702	0.10172 0.100399 0.107152 0.114956

0.123195 0.112825 0.130361 0.128657 0.130212 0.124446 0.129209 0.127214

0.119819 0.118058 0.126596 0.136843 0.112876	0.11235 0.118081 0.128627

0.114031 0.112544 0.114845 0.122592 0.103956 0.103293 0.109005 0.119695

0.114031 0.112544 0.114845 0.122592 0.103956 0.103293 0.109005 0.119695

0.100279 0.104833 NA	0.104497 NA	NA	0.119226 0.105137

0.111825 0.105176 0.115763 0.116848 0.120205 0.118018 0.119832	0.11226

0.108325 0.103488 0.112267 0.117654 0.110726 0.107053 0.108849 0.107707

0.121836 0.114895 0.119757	0.12157 0.107027 0.107301 0.127524 0.107188

0.123685 0.118297 0.124908 0.122741 0.106773 0.107134 0.128504	0.11057

NA	NA	NA	NA	NA	NA	NA	NA NA	NA	NA	NA	NA	NA	NA	NA

0.109942 0.111826 0.105155 0.101072 NA	NA	0.096755 NA

0.109942 0.111826 0.105155 0.101072 NA	NA	0.096755 NA

0.130994 0.134517 0.109798 0.109884 0.153089 0.153597	0.1523 0.123134

0.121384 0.118723 0.128195 0.135452 0.113902 0.111677 0.113721 0.122296

NA	NA	NA	NA	NA	NA	NA	NA

0.144089 0.140028 0.141617 0.119998 0.149335 0.146086 0.136753 0.162992

NA	NA	NA	NA	NA	NA	NA	NA NA	NA	NA	NA	NA	NA	NA	NA NA	0.091444 NA	NA	NA	NA	0.105384 NA NA	NA	NA	NA	NA	NA	0.124977 NA NA	NA	NA	NA	NA	NA	0.127413 NA NA	NA	NA	NA	NA	NA	0.118242 NA

0.11935 0.121982 0.120942 0.124143 0.114695	0.11409	0.1121 0.125567

0.11935 0.121982 0.120942 0.124143 0.114695	0.11409	0.1121 0.125567

0.140563 0.139279	0.14309 0.153947 0.132636 0.131183	0.13186 0.141241

0.141247 0.141497 0.143398	0.15422	0.13523 0.133523 0.132365 0.142316

NA	0.102132 NA	0.097885 NA	NA	0.111359 NA NA	NA	NA	NA	NA	NA	0.107575 NA NA	NA	NA	NA	NA	NA	NA	NA NA	NA	NA	NA	NA	NA	NA	NA NA	NA	NA	NA	NA	NA	NA	NA

0.10793 0.099711 0.101691 0.099378 0.113671 0.112422 0.111569 0.118295

| 0.095955 | 0.09239 | 0.101409 | 0.127096 | | 0.097332 | 0.098245 | 0.101222 | | 0.097251 |
| --- | --- | --- | --- | --- | --- | --- | --- | --- | --- |
| 0.095955 | 0.09239 | 0.101409 | 0.127096 | | 0.097332 | 0.098245 | 0.101222 | | 0.097251 |
| 0.095955 | 0.09239 | 0.101409 | 0.127096 | | 0.097332 | 0.098245 | 0.101222 | | 0.097251 |
| 0.120737 | 0.119918 | 0.099824 | 0.100407 | | 0.109445 | 0.110235 | 0.10695 | | 0.110863 |
| 0.116385 | 0.111645 | 0.100556 | 0.102645 | | 0.108726 | 0.108475 | 0.106114 | | 0.112904 |
| 0.150473 | 0.146366 | 0.13008 |  | 0.1198 | 0.182518 | 0.183684 | 0.166351 | | 0.152473 |
| 0.164609 | 0.158182 | 0.146098 | 0.12622 | | 0.195439 | 0.194684 | 0.185983 | | 0.171715 |
| 0.182976 | 0.17228 | 0.162769 | 0.138055 | | 0.217816 | 0.21674 | 0.208183 | | 0.191507 |
| 0.164614 | 0.159346 | 0.14416 | 0.123522 | | 0.201599 | 0.202377 | 0.180962 | | 0.167816 |
| 0.160138 | 0.154562 | 0.147301 | 0.127747 | | 0.18465 | 0.183548 | 0.178509 | | 0.165114 |
| 0.167723 | 0.15935 | 0.155579 | 0.134626 | | 0.197121 | 0.195298 | 0.19274 | | 0.177069 |
| 0.167791 | 0.163265 | 0.148725 | 0.128607 | | 0.210748 | 0.208805 | 0.198089 | | 0.172254 |
| 0.168295 | 0.156515 | 0.160846 | 0.130808 | | 0.189019 | 0.187276 | 0.182087 | | 0.181574 |
| 0.179023 | 0.166433 | 0.165188 | 0.134175 | | 0.196416 | 0.195288 | 0.189309 | | 0.189065 |
| 0.18452 | 0.171207 | 0.173034 | 0.13834 | | 0.20907 | 0.207598 | 0.19863 | | 0.197888 |
| NA | NA | NA | NA |  | NA | NA | NA |  | NA |
| 0.11455 | 0.114636 | 0.116847 | 0.132519 | | 0.102921 | 0.102137 | 0.10414 | | 0.113789 |
| 0.186433 | 0.17532 | 0.168733 | 0.135266 | | 0.212764 | 0.210143 | 0.198658 | | 0.19937 |
| 0.195764 | 0.183487 | 0.182091 | 0.143525 | | 0.220944 | 0.218535 | 0.205825 | | 0.210762 |
| 0.146924 | 0.139108 | 0.147805 | 0.126609 | | 0.158962 | 0.160371 | 0.157069 | | 0.153577 |
| 0.197459 | 0.184681 | 0.17669 | 0.142292 | | 0.234658 | 0.233921 | 0.215557 | | 0.208215 |
| NA | NA | NA | 0.102293 | | NA | NA | NA |  | NA |
| NA | NA | NA | NA |  | NA | NA | NA |  | NA |
| 0.197581 | 0.184178 | 0.180603 | 0.143295 | | 0.225264 | 0.223972 | 0.209967 | | 0.20961 |
| 0.139187 | 0.133818 | 0.135716 | 0.116409 | | 0.158292 | 0.157222 | 0.151424 | | 0.146291 |
| 0.154848 | 0.149403 | 0.135968 | 0.118851 | | 0.182289 | 0.183318 | 0.173826 | | 0.158398 |
| 0.194557 | 0.181137 | 0.175957 | 0.141448 | | 0.222517 | 0.221031 |  | 0.2084 | 0.209247 |
| 0.18338 | 0.176035 | 0.162165 | 0.134522 | | 0.20747 | 0.205677 | 0.191982 | | 0.193932 |
| 0.150074 | 0.142015 | 0.141997 | 0.119035 | | 0.161215 | 0.160233 | 0.157272 | | 0.156607 |
| 0.150074 | 0.142015 | 0.141997 | 0.119035 | | 0.161215 | 0.160233 | 0.157272 | | 0.156607 |
| 0.197481 | 0.183782 | 0.176497 | 0.141029 | | 0.230052 | 0.228798 | 0.212523 | | 0.210624 |
| 0.158596 | 0.14932 | 0.152082 | 0.12611 | | 0.179816 | 0.178409 | 0.169478 | | 0.172087 |
| 0.140115 | 0.136711 | 0.124892 | 0.12003 | | 0.170701 | 0.167908 | 0.167976 | | 0.142415 |
| 0.156045 | 0.150395 | 0.147333 | 0.127651 | | 0.173953 | 0.173369 | 0.16388 | | 0.161213 |
| NA | NA | NA | NA |  | NA | NA | NA |  | NA |
| 0.110985 | 0.105902 | 0.115613 | 0.11957 | | 0.111757 | 0.108075 | 0.110787 | | 0.112132 |
| 0.192531 | 0.182766 | 0.169906 | 0.137401 | | 0.221529 | 0.219624 | 0.204533 | | 0.202264 |
| 0.201423 | 0.187438 | 0.18107 | 0.144132 | | 0.235915 | 0.23384 | 0.217366 | | 0.214544 |
| 0.150654 | 0.145083 | 0.15176 | 0.129536 | | 0.164272 | 0.161796 | 0.157295 | | 0.15632 |

| 0.122801 | 0.119773 | 0.119879 | 0.117712 | 0.10332 | 0.105271 | 0.125609 | 0.100224 |
| --- | --- | --- | --- | --- | --- | --- | --- |
| 0.119244 | 0.119736 | 0.117303 | 0.111823 | 0.105331 | 0.106678 | 0.119944 | 0.099795 |
| 0.125606 | 0.123291 | 0.124178 | 0.12111 | 0.109295 | 0.110468 | 0.12889 | 0.106456 |
| 0.11786 | 0.114712 | 0.118442 | 0.120645 | NA | 0.096231 | 0.119041 | 0.098675 |
| NA | NA | NA | NA | NA | NA | NA | NA |
| NA | NA | NA | NA | NA | NA | NA | NA |
| NA | NA | NA | NA | NA | NA | NA | NA |
| NA | NA | NA | NA | NA | NA | NA | NA |
| NA | NA | NA | NA | NA | NA | NA | NA |
| NA | NA | NA | NA | NA | NA | NA | NA |
| NA | NA | NA | NA | NA | NA | NA | NA |
| NA | NA | NA | NA | NA | NA | NA | NA |
| NA | NA | NA | NA | NA | NA | NA | NA |
| NA | NA | NA | NA | NA | NA | NA | NA |
| NA | NA | NA | NA | NA | NA | NA | NA |
| NA | 0.092774 | NA | NA | NA | 0.091992 | NA | NA |
| NA | NA | NA | NA | NA | NA | NA | NA |
| NA | NA | NA | NA | NA | NA | NA | NA |
| NA | 0.093938 | NA | NA | 0.095417 | 0.09546 | NA | NA |
| NA | NA | NA | NA | NA | 0.091341 | NA | NA |
| NA | NA | NA | NA | NA | NA | NA | NA |
| 0.188421 | 0.174025 | 0.173132 | 0.140221 | 0.214914 | 0.212691 | 0.204724 | 0.202507 |
| 0.096738 | 0.101404 | NA | NA | 0.104944 | 0.114399 | 0.091042 | 0.09578 |
| 0.096738 | 0.101404 | NA | NA | 0.104944 | 0.114399 | 0.091042 | 0.09578 |
| NA | NA | NA | NA | NA | 0.097107 | NA | NA |
| NA | NA | NA | NA | NA | 0.097107 | NA | NA |
| NA | 0.091911 | NA | NA | 0.094447 | 0.106784 | NA | NA |
| 0.095542 | 0.098863 | NA | NA | 0.105555 | 0.120446 | NA | 0.094554 |
| NA | NA | NA | NA | NA | 0.095022 | NA | NA |
| NA | NA | NA | NA | NA | 0.099059 | NA | NA |
| NA | NA | NA | NA | NA | 0.099059 | NA | NA |
| NA | NA | NA | NA | NA | 0.099059 | NA | NA |
| NA | NA | NA | NA | NA | NA | NA | NA |
| NA | NA | NA | NA | NA | NA | NA | NA |
| NA | NA | NA | NA | NA | NA | NA | NA |
| NA | NA | NA | NA | NA | NA | NA | NA |
| 0.100593 | 0.097033 | 0.105312 | 0.104647 | 0.102499 | 0.10314 | 0.10058 | 0.100481 |
| 0.11353 | 0.106519 | 0.121409 | 0.130793 | 0.104113 | 0.103195 | 0.111135 | 0.11843 |
| 0.11353 | 0.106519 | 0.121409 | 0.130793 | 0.104113 | 0.103195 | 0.111135 | 0.11843 |

| 0.121065 | 0.11295 | 0.136774 | 0.142892 | 0.109622 | 0.108035 | 0.118558 | 0.133805 |
| --- | --- | --- | --- | --- | --- | --- | --- |
| NA | NA | 0.113242 | 0.136196 | NA | NA | NA | 0.103665 |
| 0.117612 | 0.113877 | 0.12146 | 0.128474 | 0.117619 | 0.115242 | 0.116996 | 0.123874 |
| 0.103131 | 0.09703 | 0.112538 | 0.117178 | 0.097844 | 0.096255 | 0.099647 | 0.102551 |
| 0.114371 | 0.110698 | 0.113312 | 0.112425 | 0.113586 | 0.113521 | 0.11489 | 0.114693 |
| NA | NA | NA | NA | NA | NA | 0.107502 | NA |
| NA | NA | NA | NA | NA | NA | NA | NA |
| NA | NA | NA | NA | NA | NA | NA | NA |
| 0.097456 | 0.091924 | 0.103897 | 0.099969 | NA | NA | 0.095729 | NA |
| 0.094887 | 0.092993 | 0.101115 | 0.098525 | NA | NA | 0.094526 | NA |
| 0.099922 | 0.094121 | 0.107192 | 0.10405 | 0.094079 | 0.09542 | 0.10204 | 0.096178 |
| NA | NA | NA | NA | NA | NA | NA | NA |
| NA | NA | NA | NA | NA | NA | NA | NA |
| NA | NA | NA | NA | NA | NA | NA | NA |
| NA | NA | NA | NA | NA | NA | NA | NA |
| NA | NA | NA | NA | NA | NA | NA | NA |
| NA | NA | NA | NA | NA | NA | NA | NA |
| NA | NA | NA | NA | NA | NA | NA | NA |
| NA | NA | NA | NA | NA | NA | NA | NA |
| NA | NA | NA | NA | 0.102259 | 0.10021 | NA | NA |
| NA | NA | NA | NA | NA | NA | NA | NA |
| NA | NA | NA | NA | NA | NA | NA | NA |
| NA | NA | NA | NA | NA | NA | NA | NA |
| NA | NA | NA | NA | NA | NA | NA | NA |
| NA | NA | NA | NA | NA | NA | NA | NA |
| NA | NA | NA | NA | 0.100138 | 0.098471 | NA | NA |
| NA | NA | NA | NA | NA | NA | NA | NA |
| NA | NA | NA | NA | NA | NA | NA | NA |
| NA | NA | NA | NA | NA | NA | NA | NA |
| NA | NA | NA | NA | NA | NA | NA | NA |
| NA | 0.093114 | NA | NA | 0.095934 | 0.096968 | NA | NA |
| NA | NA | NA | NA | NA | NA | NA | NA |
| NA | NA | NA | NA | NA | NA | NA | NA |
| NA | NA | NA | NA | NA | NA | NA | NA |
| NA | NA | NA | NA | NA | NA | NA | NA |
| NA | NA | NA | NA | NA | NA | NA | NA |
| NA | NA | NA | NA | NA | NA | NA | NA |
| NA | NA | NA | NA | NA | NA | NA | NA |
| NA | NA | NA | NA | NA | NA | NA | NA |

| NA | NA | NA | NA | NA | NA | NA |  | NA |
| --- | --- | --- | --- | --- | --- | --- | --- | --- |
| NA | NA | NA | NA | 0.105465 | 0.10256 | NA |  | NA |
| NA | NA | NA | NA | NA | NA | NA |  | NA |
| NA | NA | NA | NA | NA | NA | NA |  | NA |
| NA | NA | NA | NA | NA | NA | NA |  | NA |
| NA | 0.096444 | NA | NA | 0.096101 | 0.096019 | NA |  | NA |
| NA | NA | NA | NA | NA | NA | NA |  | NA |
| NA | NA | NA | NA | NA | NA | NA |  | NA |
| NA | NA | NA | NA | NA | NA | NA |  | NA |
| NA | NA | NA | NA | NA | NA | NA |  | NA |
| 0.117658 | 0.121001 | 0.101735 | 0.097305 | 0.14143 | 0.142055 | 0.129148 | | 0.113215 |
| 0.096338 | 0.117435 | NA | 0.093724 | 0.095322 | 0.095391 | 0.095494 | | 0.095751 |
| 0.099523 | 0.104585 | NA | NA | 0.096707 | 0.094994 | NA |  | NA |
| 0.117931 | 0.121958 | 0.105127 | 0.100421 | 0.120492 | 0.117828 | 0.115054 | | 0.106457 |
| 0.174122 | 0.17707 | 0.160977 | 0.14326 | 0.148531 | 0.141717 | 0.145895 | | 0.156129 |
| 0.120459 | 0.122667 | 0.116637 | 0.110501 | 0.10896 | 0.107716 | 0.111323 | | 0.115863 |
| 0.105907 | 0.107299 | 0.11075 | 0.120577 | NA | NA | 0.10206 | | 0.103829 |
| 0.102914 | 0.109425 | NA | NA | 0.099802 | 0.097922 | NA |  | NA |
| 0.128118 | 0.129861 | 0.119619 | 0.114392 | 0.121316 | 0.116707 | 0.125335 | | 0.117852 |
| 0.127352 | 0.134903 | 0.116899 | 0.10797 | 0.108287 | 0.106413 | 0.110627 | | 0.108257 |
| 0.122291 | 0.121642 | 0.12184 | 0.116406 | 0.098891 | 0.10093 | 0.123717 | | 0.098904 |
| 0.117226 | 0.115578 | 0.117265 | 0.117269 | 0.098921 | 0.100259 | 0.121651 | | 0.099665 |
| 0.123725 | 0.122058 | 0.121899 | 0.118424 | 0.103473 | 0.104728 | 0.126386 | | 0.10163 |
| 0.116828 | 0.116437 | 0.11596 | 0.116648 | NA | 0.095398 | 0.113385 | | NA |
| 0.102348 | 0.102884 | NA | 0.094981 | 0.099201 | 0.099606 | 0.093471 | | 0.099939 |
| 0.103218 | 0.105137 | 0.103628 | 0.107757 | 0.102725 | 0.102145 | 0.099465 | | 0.102236 |
| 0.107212 | 0.104104 | 0.108965 | 0.11447 | 0.100498 | 0.101021 | 0.105837 | | 0.111847 |
| 0.112801 | 0.110098 | 0.107834 | 0.113994 | 0.104291 | 0.104781 |  | 0.109 | 0.114174 |
| 0.112801 | 0.110098 | 0.107834 | 0.113994 | 0.104291 | 0.104781 |  | 0.109 | 0.114174 |
| 0.104759 | 0.102998 | 0.094667 | 0.102669 | 0.09899 | 0.098869 | 0.096034 | | 0.10348 |
| 0.107199 | 0.108134 | 0.089093 | 0.095741 | 0.107043 | 0.106941 | 0.096013 | | 0.102033 |
| 0.10357 | 0.103038 | NA | 0.09915 | 0.099384 | 0.098673 | 0.095832 | | 0.101824 |
| 0.10357 | 0.103038 | NA | 0.09915 | 0.099384 | 0.098673 | 0.095832 | | 0.101824 |
| 0.10357 | 0.103038 | NA | 0.09915 | 0.099384 | 0.098673 | 0.095832 | | 0.101824 |
| 0.103483 | 0.10381 | 0.086639 | 0.090345 | 0.108524 | 0.108735 | 0.095822 | | 0.101223 |
| 0.108777 | 0.10541 | 0.104608 | 0.105629 | 0.115086 | 0.115205 | 0.111128 | | 0.114614 |
| 0.10138 | 0.100928 | NA | 0.104125 | NA | NA | NA |  | 0.102179 |
| NA | NA | NA | NA | NA | NA | NA |  | NA |
| 0.107508 | 0.106082 | 0.097172 | NA | 0.093104 | 0.09313 | NA |  | NA |

| NA |  | NA | NA | NA | NA | NA | NA | NA |
| --- | --- | --- | --- | --- | --- | --- | --- | --- |
| NA |  | NA | NA | NA | NA | NA | NA | NA |
| NA |  | NA | NA | NA | NA | NA | NA | NA |
| NA |  | NA | NA | NA | NA | NA | NA | NA |
| 0.116672 | | 0.104239 | 0.125619 | 0.126309 | 0.116546 | 0.112184 | 0.122948 | 0.120761 |
|  | 0.1166 | 0.109486 | 0.119456 | 0.114101 | 0.111441 | 0.111615 | 0.118507 | 0.117835 |
| 0.109177 | | 0.10123 | 0.115379 | 0.116568 | 0.104024 | 0.103472 | 0.109517 | 0.108843 |
| 0.119725 | | 0.112477 | 0.121618 | 0.113976 | 0.11922 | 0.117987 | 0.123401 | 0.124416 |
| 0.108257 | | 0.098201 | 0.115178 | 0.116056 | 0.102425 | 0.100857 | 0.112425 | 0.108613 |
| 0.110131 | | 0.101693 | 0.117132 | 0.114621 | 0.106807 | 0.105092 | 0.114564 | 0.111988 |
| 0.120972 | | 0.119056 | 0.12404 | 0.131187 | 0.115095 | 0.113117 | 0.114541 | 0.125757 |
| 0.113651 | | 0.109535 | 0.117123 | 0.111039 | 0.12079 | 0.118178 | 0.127724 | 0.134079 |
| 0.119499 | | 0.118673 | 0.123903 | 0.114805 | 0.127023 | 0.124256 | 0.135545 | 0.136772 |
| 0.105041 | | 0.101538 | 0.107985 | 0.10954 | 0.102036 | 0.100112 | 0.100867 | 0.107285 |
| NA |  | NA | NA | NA | NA | NA | NA | NA |
| NA |  | NA | NA | NA | NA | NA | NA | NA |
| 0.117641 | | 0.117264 | 0.122209 | 0.134672 | 0.109517 | 0.107187 | 0.108043 | 0.116789 |
| NA |  | NA | NA | NA | NA | NA | NA | NA |
| 0.113561 | | 0.106298 | 0.115762 | 0.117623 | 0.116588 | 0.112623 | 0.112344 | 0.112765 |
| NA |  | NA | NA | NA | NA | NA | NA | NA |
| NA |  | NA | NA | NA | NA | NA | NA | NA |
| 0.116467 | | 0.113173 | 0.120751 | 0.116596 | 0.114438 | 0.113422 | 0.115682 | 0.123964 |
| 0.122111 | | 0.12148 | 0.124683 | 0.131817 | 0.118469 | 0.11712 | 0.11808 | 0.126019 |
| 0.122111 | | 0.12148 | 0.124683 | 0.131817 | 0.118469 | 0.11712 | 0.11808 | 0.126019 |
| 0.122111 | | 0.12148 | 0.124683 | 0.131817 | 0.118469 | 0.11712 | 0.11808 | 0.126019 |
| 0.122111 | | 0.12148 | 0.124683 | 0.131817 | 0.118469 | 0.11712 | 0.11808 | 0.126019 |
| 0.116897 | | 0.115676 | 0.110782 | 0.105669 | 0.120638 | 0.122443 | 0.118127 | 0.116823 |
| 0.116897 | | 0.115676 | 0.110782 | 0.105669 | 0.120638 | 0.122443 | 0.118127 | 0.116823 |
| 0.116897 | | 0.115676 | 0.110782 | 0.105669 | 0.120638 | 0.122443 | 0.118127 | 0.116823 |
| 0.116897 | | 0.115676 | 0.110782 | 0.105669 | 0.120638 | 0.122443 | 0.118127 | 0.116823 |
| 0.110714 | | 0.116773 | 0.108935 | 0.10297 | 0.113887 | 0.114563 | 0.108478 | 0.112451 |
| 0.110714 | | 0.116773 | 0.108935 | 0.10297 | 0.113887 | 0.114563 | 0.108478 | 0.112451 |
| 0.108546 | | 0.114773 | 0.10158 | 0.101062 | 0.112794 | 0.111356 | 0.107634 | 0.111053 |
| 0.108546 | | 0.114773 | 0.10158 | 0.101062 | 0.112794 | 0.111356 | 0.107634 | 0.111053 |
| 0.132326 | | 0.131737 | 0.136048 | 0.142268 | 0.124046 | 0.123177 | 0.12427 | 0.140607 |
| 0.116051 | | 0.106118 | 0.123246 | 0.127242 | 0.119808 | 0.113849 | 0.119808 | 0.120577 |
| 0.116051 | | 0.106118 | 0.123246 | 0.127242 | 0.119808 | 0.113849 | 0.119808 | 0.120577 |
| 0.113014 | | 0.108405 | 0.11441 | 0.114897 | 0.117861 | 0.114254 | 0.114447 | 0.114761 |
| 0.113014 | | 0.108405 | 0.11441 | 0.114897 | 0.117861 | 0.114254 | 0.114447 | 0.114761 |

| 0.115389 | 0.107181 | 0.122161 | 0.126395 | 0.118812 | 0.113659 | 0.115813 | 0.115628 |
| --- | --- | --- | --- | --- | --- | --- | --- |
| 0.120385 | 0.110594 | 0.126641 | 0.129415 | 0.124643 | 0.119561 | 0.125306 | 0.124254 |
| 0.113416 | 0.106431 | 0.117703 | 0.121936 | 0.117166 | 0.112098 | 0.114948 | 0.112793 |
| 0.098975 | 0.098445 | 0.10161 | 0.104954 | 0.105403 | 0.103563 | 0.103366 | 0.100644 |
| 0.119932 | 0.113553 | 0.119207 | 0.113485 | 0.114564 | 0.115803 | 0.123624 | 0.106788 |
| 0.120247 | 0.121193 | 0.118491 | 0.117027 | 0.096801 | 0.099151 | 0.121625 | NA |
| 0.118577 | 0.110496 | 0.123065 | 0.11802 | 0.115368 | 0.116884 | 0.127015 | 0.112054 |
| 0.109549 | 0.099523 | 0.117421 | 0.11411 | 0.119119 | 0.118792 | 0.111411 | 0.104019 |
| 0.109756 | 0.100376 | 0.119263 | 0.114801 | 0.121575 | 0.121231 | 0.114065 | 0.106483 |
| NA | NA | 0.09957 | 0.103922 | NA | NA | NA | NA |
| 0.110358 | 0.100695 | 0.118471 | 0.114884 | 0.120659 | 0.121748 | 0.113286 | 0.104062 |
| 0.10399 | 0.098653 | 0.109403 | 0.104206 | 0.109947 | 0.109599 | 0.103875 | 0.10081 |
| 0.101083 | 0.100595 | 0.104936 | 0.123421 | NA | NA | 0.096241 | NA |
| 0.107323 | 0.107952 | 0.108425 | 0.115708 | 0.107304 | 0.107248 | 0.107912 | 0.099667 |
| 0.097788 | 0.097237 | 0.101155 | 0.124689 | NA | NA | NA | NA |
| NA | NA | NA | 0.098942 | NA | NA | NA | NA |
| NA | NA | NA | NA | NA | NA | NA | NA |
| 0.095607 | 0.096688 | NA | 0.094982 | 0.096039 | 0.096704 | NA | NA |
| NA | NA | NA | 0.094462 | NA | NA | NA | NA |
| NA | NA | NA | 0.094995 | NA | NA | NA | NA |
| NA | NA | NA | 0.100549 | NA | NA | NA | NA |
| NA | NA | NA | 0.100908 | NA | NA | NA | NA |
| NA | NA | NA | 0.096438 | NA | NA | NA | NA |
| 0.09817 | 0.099875 | NA | 0.093079 | 0.099302 | 0.099411 | NA | NA |
| 0.102249 | 0.104278 | NA | 0.091491 | 0.108585 | 0.11003 | 0.102063 | 0.10006 |
| NA | NA | NA | NA | NA | NA | NA | NA |
| NA | NA | NA | NA | NA | NA | NA | NA |
| NA | NA | NA | NA | NA | NA | NA | NA |
| 0.128832 | 0.129496 | 0.131177 | 0.137429 | 0.122747 | 0.121287 | 0.118909 | 0.132733 |
| 0.10811 | 0.110131 | 0.09514 | 0.096963 | 0.116135 | 0.116502 | 0.104724 | 0.101974 |
| 0.10811 | 0.110131 | 0.09514 | 0.096963 | 0.116135 | 0.116502 | 0.104724 | 0.101974 |
| NA | NA | NA | NA | NA | NA | NA | NA |
| 0.095777 | NA | 0.103069 | 0.109241 | NA | NA | 0.096963 | 0.093706 |
| NA | NA | NA | 0.103917 | NA | NA | NA | NA |
| 0.096629 | 0.096469 | NA | 0.098956 | 0.093465 | 0.093781 | 0.092632 | NA |
| 0.127281 | 0.119443 | 0.128853 | 0.12105 | 0.135028 | 0.132792 | 0.140054 | 0.136723 |
| 0.096427 | 0.093454 | 0.103309 | 0.102443 | NA | NA | 0.095498 | NA |
| NA | NA | NA | 0.108813 | NA | NA | NA | NA |
| 0.096014 | NA | 0.102661 | 0.104577 | NA | NA | 0.095569 | NA |

| 0.095864 | | 0.095183 | | NA | 0.095479 | 0.091496 | 0.091774 | 0.089595 | NA |
| --- | --- | --- | --- | --- | --- | --- | --- | --- | --- |
| NA |  | NA |  | NA | NA | NA | NA | NA | NA |
| NA |  | NA |  | NA | NA | NA | NA | NA | NA |
| NA |  | NA |  | 0.099952 | 0.098687 | NA | NA | NA | NA |
| NA |  | NA |  | 0.100983 | 0.10531 | NA | NA | 0.096383 | NA |
| 0.115887 | | 0.110111 | | 0.117583 | 0.110438 | 0.111397 | 0.111935 | 0.117683 | 0.116825 |
| NA |  | NA |  | NA | NA | NA | NA | NA | NA |
| NA |  | NA |  | NA | NA | NA | NA | NA | NA |
| NA |  | NA |  | NA | NA | NA | NA | NA | NA |
| NA |  | NA |  | NA | NA | NA | NA | NA | NA |
| 0.11106 | | 0.102487 | | 0.115545 | 0.15014 | 0.104527 | 0.10647 | 0.116651 | 0.116907 |
| 0.101471 | | 0.098031 | | 0.105008 | 0.126473 | NA | NA | 0.102116 | 0.101001 |
| 0.111241 | | 0.107367 | | 0.114517 | 0.11652 | 0.10852 | 0.109642 | 0.115957 | 0.114182 |
| NA |  | NA |  | NA | NA | NA | NA | NA | NA |
| 0.099608 | | NA |  | 0.106082 | 0.135367 | NA | NA | 0.10359 | 0.104533 |
| 0.09771 | |  | 0.0987 | NA | 0.110766 | 0.09686 | 0.097494 | 0.097476 | 0.098578 |
|  | 0.1033 | 0.102677 | | 0.102488 | 0.108284 | 0.096789 | 0.097425 | 0.101744 | NA |
| 0.099224 | | NA |  | 0.109093 | 0.124878 | NA | NA | 0.101756 | 0.102191 |
| NA |  | NA |  | NA | NA | NA | NA | NA | NA |
| 0.105558 | | 0.098084 | | 0.112858 | 0.118191 | 0.107724 | 0.105831 | 0.108872 | 0.108999 |
| 0.109548 | | 0.104702 | | 0.110967 | 0.113031 | 0.113183 | 0.112622 | 0.111853 | 0.114508 |
| 0.09716 | | 0.096835 | | 0.098024 | 0.098023 | 0.09817 | 0.100317 | 0.097292 | NA |
| 0.096189 | | 0.096139 | | NA | 0.095603 | 0.095995 | 0.096076 | 0.09499 | NA |
| 0.118287 | | 0.10632 | | 0.115174 | 0.104776 | 0.127158 | 0.12618 | 0.134274 | 0.143894 |
| 0.110503 | | 0.105986 | | 0.109946 | 0.110716 | 0.110305 | 0.108517 | 0.109315 | 0.107196 |
| 0.099797 | | 0.098311 | | 0.102022 | 0.105052 | NA | 0.095193 | 0.096699 | 0.095733 |
| 0.112484 | | 0.111834 | | 0.111298 | 0.117651 | 0.109138 | 0.10984 | 0.108399 | 0.103087 |
| 0.101861 | | 0.102773 | | 0.102823 | 0.110392 | 0.099331 | 0.099585 | 0.099522 | 0.095414 |
| 0.113404 | | 0.108597 | | 0.115521 | 0.112289 | 0.112886 | 0.111997 | 0.116661 | 0.113331 |
| 0.102339 | | 0.098366 | | 0.107319 | 0.11009 | 0.099363 | 0.098707 | 0.103174 | 0.105252 |
| 0.115737 | | 0.112195 | | 0.120984 | 0.112621 | 0.120149 | 0.117718 | 0.115102 | 0.119203 |
| 0.106659 | | 0.105364 | | 0.110628 | 0.110365 | 0.10554 | 0.105649 | 0.108621 | 0.109862 |
| 0.119483 | | 0.114438 | | 0.124148 | 0.113677 | 0.118784 | 0.118561 | 0.117812 | 0.119716 |
| 0.10721 | | 0.103128 | | 0.110032 | 0.112049 | 0.107921 | 0.106002 | 0.107317 | 0.107791 |
| 0.107306 | | 0.10802 | | 0.105854 | 0.10466 | 0.106824 | 0.107268 | 0.106299 | 0.108775 |
| 0.101074 | | 0.101915 | | 0.105145 | 0.102429 | NA | 0.098877 | NA | 0.100358 |
| NA |  | NA |  | NA | 0.098619 | NA | NA | NA | NA |
| NA |  | NA |  | NA | 0.098619 | NA | NA | NA | NA |
| NA |  | NA |  | NA | 0.098619 | NA | NA | NA | NA |

| 0.145012 | 0.142467 | 0.139297 | 0.119527 | 0.147627 | 0.143928 | 0.130458 | 0.161988 |
| --- | --- | --- | --- | --- | --- | --- | --- |
| 0.151433 | 0.148337 | 0.143129 | 0.118694 | 0.155826 | 0.152192 | 0.137486 | 0.170697 |
| 0.132588 | 0.131034 | 0.124879 | 0.107423 | 0.141509 | 0.136492 | 0.124632 | 0.156162 |
| 0.09645 | 0.095153 | NA | 0.099042 | 0.09422 | 0.095454 | 0.095161 | NA |
| NA | NA | 0.100375 | 0.110565 | NA | NA | NA | NA |
| NA | NA | NA | 0.096436 | NA | NA | NA | NA |
| NA | NA | NA | 0.097092 | NA | NA | NA | NA |
| 0.098468 | 0.099697 | NA | 0.096642 | 0.095573 | 0.096542 | 0.096847 | NA |
| 0.105952 | 0.100958 | 0.110224 | 0.111494 | 0.106098 | 0.107375 | 0.107596 | 0.103575 |
| 0.108119 | 0.102225 | 0.113131 | 0.111956 | 0.10444 | 0.104494 | 0.106871 | 0.111111 |
| 0.106738 | 0.101134 | 0.109028 | 0.110579 | 0.104546 | 0.104741 | 0.108341 | 0.107424 |
| 0.106738 | 0.101134 | 0.109028 | 0.110579 | 0.104546 | 0.104741 | 0.108341 | 0.107424 |
| 0.10847 | 0.104344 | 0.109697 | 0.109375 | 0.106545 | 0.106895 | 0.110082 | 0.111514 |
| 0.118509 | 0.11517 | 0.119834 | 0.12136 | 0.095989 | 0.097273 | 0.11941 | 0.098334 |
| 0.118509 | 0.11517 | 0.119834 | 0.12136 | 0.095989 | 0.097273 | 0.11941 | 0.098334 |
| 0.094707 | 0.092518 | NA | NA | 0.091988 | 0.092196 | NA | NA |
| NA | 0.088614 | NA | NA | NA | NA | NA | NA |
| NA | NA | NA | NA | NA | NA | NA | NA |
| 0.102345 | 0.104857 | NA | 0.093784 | 0.104122 | 0.103989 | 0.093071 | 0.097678 |
| 0.152222 | 0.145732 | 0.145643 | 0.123182 | 0.165974 | 0.164685 | 0.158568 | 0.156563 |
| 0.154071 | 0.156626 | 0.124748 | 0.131301 | 0.221894 | 0.218024 | 0.208276 | 0.157886 |
| 0.116494 | 0.119256 | 0.091266 | 0.095142 | 0.148788 | 0.148246 | 0.137865 | 0.106779 |
| 0.134349 | 0.132901 | 0.118944 | 0.110724 | 0.168703 | 0.168919 | 0.159293 | 0.13673 |
| 0.146508 | 0.147862 | 0.123693 | 0.126097 | 0.207235 | 0.206146 | 0.202617 | 0.150876 |
| 0.137253 | 0.13973 | 0.113468 | 0.112757 | 0.186291 | 0.184874 | 0.172237 | 0.134408 |
| 0.101046 | 0.099421 | 0.098491 | 0.092508 | 0.105609 | 0.106295 | 0.107773 | 0.093702 |
| 0.118046 | 0.12234 | 0.107012 | 0.102109 | 0.118691 | 0.114587 | 0.116601 | 0.106231 |
| 0.107628 | 0.105819 | 0.106771 | 0.095905 | 0.107165 | 0.108811 | 0.108305 | 0.099813 |
| 0.103059 | 0.101079 | 0.103081 | 0.108759 | 0.103159 | 0.100546 | 0.097942 | 0.099754 |
| 0.121654 | 0.110378 | 0.125824 | 0.128516 | 0.122391 | 0.116303 | 0.119155 | 0.11685 |
| 0.115735 | 0.110528 | 0.118239 | 0.117508 | 0.124636 | 0.121092 | 0.123315 | 0.120432 |
| 0.105517 | 0.098686 | 0.109971 | 0.12594 | 0.100058 | NA | NA | 0.100211 |
| 0.102585 | 0.101156 | NA | NA | NA | NA | NA | NA |
| NA | NA | NA | NA | NA | NA | NA | NA |
| NA | NA | NA | NA | NA | NA | NA | NA |
| 0.103795 | NA | 0.114065 | 0.123067 | 0.099774 | 0.098634 | 0.109297 | 0.110403 |
| 0.112042 | 0.101533 | 0.119624 | 0.125282 | 0.109865 | 0.109278 | 0.116525 | 0.115695 |
| 0.107751 | 0.097749 | 0.119178 | 0.125797 | 0.109804 | 0.10748 | 0.117436 | 0.119114 |
| NA | NA | 0.101437 | 0.113532 | NA | NA | NA | 0.098942 |

| 0.111113 | | 0.101032 | 0.120539 | 0.124811 | 0.108714 | | 0.108827 | 0.115999 | 0.117041 |
| --- | --- | --- | --- | --- | --- | --- | --- | --- | --- |
| 0.121079 | | 0.109631 | 0.127084 | 0.125919 | 0.121072 | | 0.118689 | 0.126075 | 0.127146 |
| 0.117014 | | 0.116102 | 0.122206 | 0.131816 | 0.109908 | | 0.109129 | 0.113522 | 0.119018 |
| 0.11643 | | 0.113788 | 0.125142 | 0.131046 | 0.109606 | | 0.10832 | 0.113474 | 0.118331 |
| 0.110679 | | 0.108356 | 0.118148 | 0.125008 | 0.104023 | | 0.103611 | 0.109554 | 0.112232 |
| 0.108175 | | 0.103869 | 0.114598 | 0.113674 | 0.109223 | | 0.107316 | 0.109696 | 0.115702 |
| 0.099469 | | NA | 0.109461 | 0.112716 | 0.096359 | | 0.095696 | 0.099011 | NA |
| 0.116688 | | 0.113542 | 0.126311 | 0.13365 | 0.111069 | | 0.108814 | 0.11386 | 0.119889 |
| 0.112403 | | 0.109498 | 0.121968 | 0.13237 | 0.105206 | | 0.103192 | 0.110606 | 0.113701 |
| NA |  | NA | NA | NA | NA |  | NA | NA | NA |
| NA |  | NA | NA | NA | NA |  | NA | NA | NA |
| NA |  | NA | NA | NA | NA |  | NA | NA | NA |
| 0.124202 | | 0.11095 | 0.12117 | 0.112564 | 0.132276 | | 0.131375 | 0.135029 | 0.148534 |
| 0.118924 | | 0.113358 | 0.124184 | 0.123612 | 0.114946 | | 0.113719 | 0.119014 | 0.124444 |
| 0.100211 | | 0.095881 | 0.101875 | 0.109856 | 0.099997 | | 0.096104 | 0.098779 | 0.099054 |
| 0.111959 | | 0.10884 | 0.115778 | 0.122443 | 0.110206 | | 0.110312 | 0.111338 | 0.116767 |
|  | 0.0989 | 0.095192 | 0.101287 | 0.108608 | 0.099981 | | 0.095392 | 0.097498 | 0.097842 |
| 0.110032 | | 0.106955 | 0.106316 | 0.111186 | 0.098423 | | 0.097202 | 0.098557 | 0.115713 |
| 0.105327 | | 0.098342 | 0.112527 | 0.114039 | 0.10285 | | 0.102531 | 0.108106 | 0.110037 |
| NA |  | NA | NA | NA | NA |  | NA | NA | NA |
| 0.121929 | | 0.11386 | 0.132136 | 0.122072 | 0.121773 | | 0.120554 | 0.128812 | 0.136385 |
| 0.10199 | | 0.098712 | 0.101861 | 0.105572 | 0.096837 | | 0.096193 | 0.097988 | NA |
| 0.096884 | | NA | 0.112123 | 0.122076 | NA |  | NA | 0.09795 | 0.103233 |
| 0.096765 | | NA | 0.113947 | 0.124157 | NA |  | NA | 0.099447 | 0.104849 |
| 0.109102 | | 0.105398 | 0.116244 | 0.117499 | 0.109926 | | 0.107912 | 0.11137 | 0.118414 |
| 0.104768 | | 0.105648 | 0.093827 | 0.098731 | 0.113417 | | 0.113854 | 0.103973 | 0.10956 |
| 0.107999 | | 0.104402 | 0.112064 | 0.110388 | 0.112731 | | 0.111434 | 0.113445 | 0.117345 |
| 0.10824 | | 0.100785 | 0.11693 | 0.119549 |  | 0.1091 | 0.106348 | 0.114576 | 0.117829 |
| 0.103626 | | 0.102861 | NA | 0.099468 | 0.105422 | | 0.105059 | 0.098588 | 0.095994 |
| 0.112984 | | 0.110684 | 0.118038 | 0.116978 | 0.113987 | | 0.111997 | 0.117261 | 0.125012 |
| 0.115429 | | 0.115238 | 0.100032 | 0.103619 | 0.118464 | | 0.119641 | 0.108446 | 0.109573 |
| 0.123772 | | 0.11574 | 0.133639 | 0.123063 | 0.118781 | | 0.118641 | 0.126699 | 0.134795 |
| 0.109699 | | 0.106502 | 0.109312 | 0.106396 | 0.107269 | | 0.107872 | 0.107178 | 0.106379 |
| 0.110291 | | 0.109313 | 0.113357 | 0.111068 | 0.10896 | | 0.108321 | 0.10489 | 0.103961 |
| 0.102951 | | 0.099108 | 0.096961 | 0.101256 | 0.100965 | | 0.101289 | 0.109283 | 0.11517 |
| 0.106289 | | 0.107312 | 0.098667 | 0.09145 | 0.115536 | | 0.115628 | 0.108186 | 0.099938 |
| 0.116128 | | 0.116477 | 0.107073 | 0.096721 | 0.126722 | | 0.127606 | 0.120761 | 0.110663 |
| 0.112016 | | 0.107873 | 0.111981 | 0.111551 | 0.111902 | | 0.110078 | 0.111716 | 0.111854 |
| 0.113916 | | 0.106605 | 0.118909 | 0.111128 | 0.112487 | | 0.112224 | 0.121322 | 0.129267 |

| 0.114238 | 0.109451 | | 0.120559 | 0.116127 | 0.116716 | | 0.118213 | | 0.120403 | 0.119396 |
| --- | --- | --- | --- | --- | --- | --- | --- | --- | --- | --- |
| 0.111194 | 0.105571 | | 0.112577 | 0.108445 | 0.118488 | | 0.118114 | | 0.112522 | 0.113673 |
| 0.116895 | 0.113562 | | 0.119245 | 0.116584 | 0.113618 | | 0.11384 | | 0.11508 | 0.116573 |
| 0.108712 | 0.104584 | | 0.108522 | 0.109815 | 0.108887 | | 0.105712 | | 0.106395 | 0.114215 |
| 0.114542 | 0.121057 | | 0.105042 | 0.09757 |  | 0.1292 | 0.131185 | | 0.133174 | 0.127515 |
| 0.095884 | 0.09996 | | NA | NA | NA |  | NA |  | NA | NA |
| 0.11498 | 0.107172 | | 0.118944 | 0.112998 | 0.117272 | | 0.116551 | | 0.11944 | 0.116164 |
| 0.106818 | 0.098295 | | 0.112607 | 0.114856 | 0.104561 | | 0.103111 | | NA | NA |
| 0.112597 | 0.105919 | | 0.117252 | 0.116848 | 0.111569 | | 0.111928 | | 0.115896 | 0.118267 |
| 0.107958 | 0.108185 | | 0.111449 | 0.101452 | 0.111094 | | 0.110444 | | 0.108821 | 0.110943 |
| 0.109324 | 0.103074 | | 0.105177 | 0.107807 | 0.110111 | | 0.111597 | | 0.107019 | 0.105834 |
| NA | NA |  | NA | NA | NA |  | NA |  | NA | NA |
| 0.110266 | 0.105356 | | 0.109286 | 0.109946 | 0.110371 | | 0.109204 | | 0.110876 | 0.107815 |
| 0.104266 | 0.103406 | | 0.095153 | 0.101458 | 0.107636 | | 0.106188 | | 0.100143 | 0.105896 |
| 0.111119 | 0.108608 | | 0.105175 | 0.110737 | 0.113312 | | 0.111274 | | 0.10613 | 0.119168 |
| 0.123907 | 0.111686 | | 0.132842 | 0.125023 | 0.124406 | | 0.122008 | | 0.130732 | 0.132995 |
| 0.102376 | 0.096352 | | 0.105703 | 0.109646 | 0.098484 | | 0.09855 | | 0.101692 | 0.107726 |
| 0.115915 | 0.10471 | | 0.128118 | 0.12034 | 0.112556 | | 0.109665 | | 0.118727 | 0.128961 |
| NA | 0.096728 | | NA | 0.103524 | NA |  | NA |  | NA | NA |
| 0.108113 | 0.103869 | | 0.103783 | 0.104216 | 0.107095 | | 0.107026 | | 0.106148 | 0.103988 |
| 0.116299 | 0.114511 | | 0.105387 | 0.104826 | 0.116734 | | 0.115755 | | 0.106765 | 0.113687 |
| 0.09923 | 0.101881 | | NA | NA | 0.10534 | | 0.105164 | | 0.098426 | NA |
| 0.10803 | 0.100267 | | 0.11521 | 0.115679 | 0.108105 | | 0.10595 | | 0.110445 | 0.108484 |
| 0.101264 | 0.099681 | | NA | NA | 0.121464 | | 0.122092 | | 0.109506 | 0.096617 |
| 0.108268 | 0.100948 | | 0.112912 | 0.115252 | 0.108383 | | 0.107703 | | 0.109583 | NA |
| 0.108899 | 0.110302 | | 0.100283 | 0.097458 | 0.11665 | | 0.115407 | | 0.11238 | 0.103159 |
| 0.105858 | 0.103956 | | 0.105839 | 0.10426 | 0.109057 | |  | 0.1097 | 0.109097 | 0.106402 |
| 0.101675 | 0.104239 | | 0.08979 | 0.093549 | 0.112304 | | 0.11106 | | 0.097341 | 0.099833 |
| 0.106022 | 0.102188 | | 0.104498 | 0.106204 | 0.10205 | | 0.101717 | | 0.106322 | 0.110614 |
| 0.10381 | 0.103255 | | 0.104062 | 0.102331 | 0.100641 | | 0.100718 | | 0.101093 | 0.102229 |
| 0.104385 | 0.106143 | | NA | 0.094639 | 0.118013 | | 0.118695 | | 0.110839 | 0.100142 |
| 0.115537 | 0.107288 | | 0.115332 | 0.118211 | 0.118261 | | 0.116447 | | 0.121399 | 0.131887 |
| 0.102232 | 0.100904 | | 0.102821 | 0.107046 | 0.106569 | | 0.106083 | | 0.106171 | 0.100678 |
| 0.110382 | 0.110796 | | 0.106001 | 0.097963 | 0.112259 | | 0.111699 | | 0.105899 | 0.117895 |
| 0.097782 | NA |  | 0.100546 | 0.106266 | NA |  | NA |  | NA | NA |
| 0.109948 |  | 0.1037 | 0.112626 | 0.114028 | 0.104404 | | 0.103516 | | 0.107704 | 0.108069 |
| 0.111277 | 0.106973 | | 0.111297 | 0.105817 | 0.112534 | | 0.111625 | | 0.111901 | 0.113078 |
| 0.106718 | 0.105554 | | 0.09521 | 0.097588 | 0.113724 | | 0.114502 | | 0.103033 | 0.102693 |

early_neur effector_meffector_meffector_mendothelia endothelia eosinophil erythrobla

0.135864 0.112019	0.10959 0.110529 0.113201 0.115467 0.107896 0.113499

NA	NA	NA	NA	NA	NA	NA	NA

NA	0.117745 0.118817 0.121147 0.109715 0.112983 0.111215 0.113721

NA	0.11222 0.114718 0.116419 0.125996 0.127268 0.102207 0.128744

NA	0.120374 0.125865 0.129646 0.106539 0.106173 0.096038 0.112056

0.124058 0.120882 0.119539	0.12095 0.125974 0.129906 0.116277 0.139158

NA	0.124023 0.127473 0.130461 0.115818 0.119195 0.109753 0.121881

NA	0.116668 0.118155 0.123084 0.112006 0.115247 0.097674 0.112144

NA	0.116668 0.118155 0.123084 0.112006 0.115247 0.097674 0.112144

NA	0.100965 0.100364 0.100973 0.116736 NA	0.119974 NA

NA	0.111271	0.11053 0.112091 0.119745	0.11746 0.112601	0.12354

NA	0.109244 0.108852 0.111654 0.105242 0.106818 0.102567 0.110637

NA	0.120472	0.11659 0.120027 0.124591 0.114644 0.105138 0.108941

NA	0.123031 0.119628 0.123988 0.130175 0.120919 0.106708 0.113537

NA	NA	NA	NA	NA	NA	NA	NA

0.121024 NA	NA	NA	NA	NA	NA	NA NA	0.1095 0.107352 0.107638 NA	NA	0.098859 NA NA	0.1095 0.107352 0.107638 NA	NA	0.098859 NA

NA	0.122744 0.114703 0.114122 0.108418 NA	0.157015 0.108336

NA	0.125128 0.127828 0.127773	0.11119 0.111272 0.107906 0.114873

0.121785 NA	NA	NA	NA	NA	NA	NA

NA	0.134161 0.132624	0.13374	0.09996 0.098561 0.141789 0.134495

NA	NA	NA	NA	NA	NA	NA	NA NA	NA	NA	NA	NA	NA	NA	NA NA	NA	NA	NA	NA	NA	0.11834 NA NA	NA	NA	NA	NA	NA	0.126714 NA NA	NA	NA	NA	NA	NA	0.127218 NA NA	NA	NA	NA	NA	NA	0.119485 NA

NA	0.1236 0.127248 0.127397 0.109315 0.109901 0.111224 0.113387

NA	0.1236 0.127248 0.127397 0.109315 0.109901 0.111224 0.113387

NA	0.148151 0.153456 0.157965 0.134348 0.135137 0.126025	0.14236

NA	0.14883 0.154561 0.157449 0.132539 0.131369 0.129244 0.143695

NA	0.098854 NA	0.097699 0.104811 NA	0.117961 NA NA	NA	NA	NA	NA	NA	0.122787 NA NA	NA	NA	NA	NA	NA	NA	NA NA	NA	NA	NA	NA	NA	NA	NA

0.116103 NA	NA	NA	NA	NA	NA	NA

0.136422	0.10084 0.096832 0.098133 0.126735 0.124099 0.099243 0.130316

| 0.114472 | 0.09824 | | 0.100576 | 0.102687 | 0.10309 | | 0.099574 | 0.092243 | | 0.101955 |
| --- | --- | --- | --- | --- | --- | --- | --- | --- | --- | --- |
| 0.114472 | 0.09824 | | 0.100576 | 0.102687 | 0.10309 | | 0.099574 | 0.092243 | | 0.101955 |
| 0.114472 | 0.09824 | | 0.100576 | 0.102687 | 0.10309 | | 0.099574 | 0.092243 | | 0.101955 |
| 0.111714 | 0.112771 | | 0.110284 | 0.108789 | 0.101754 | | 0.100611 | 0.100617 | | 0.097681 |
| 0.112041 | 0.108796 | | 0.107826 | 0.107362 | 0.105857 | | 0.104951 | 0.098511 | | 0.104433 |
| NA | 0.138495 | | 0.128696 | 0.128099 | 0.153047 | | 0.138275 | 0.17409 | | 0.14638 |
| NA | 0.147808 | | 0.136278 | 0.13459 | 0.17034 | | 0.158939 | 0.19115 | | 0.164807 |
| NA | 0.160646 | | 0.147351 | 0.146603 | 0.179911 | | 0.173274 | 0.209766 | | 0.180226 |
| NA | 0.14951 | | 0.138107 | 0.137343 | 0.169953 | | 0.155514 | 0.190448 | | 0.159609 |
| NA | 0.145878 | | 0.135683 | 0.134992 | 0.160478 | | 0.15626 | 0.180793 | | 0.157534 |
| NA | 0.15099 | | 0.138704 | 0.138575 | 0.164511 | | 0.162298 | 0.188959 | | 0.170227 |
| NA | 0.149621 | | 0.136759 | 0.136315 | 0.158933 | | 0.154091 | 0.210452 | | 0.164358 |
| 0.108416 | 0.148904 | | 0.137178 | 0.137352 | 0.186166 | | 0.179941 | 0.176269 | | 0.180478 |
| NA | 0.158245 | | 0.146127 | 0.146283 | 0.181771 | | 0.178834 | 0.187838 | | 0.175857 |
| 0.09959 | 0.161445 | | 0.148318 | 0.148545 | 0.195818 | | 0.191553 | 0.197052 | | 0.189029 |
| NA | NA |  | NA | NA | NA |  | NA | NA |  | NA |
| NA | 0.121183 | | 0.125873 | 0.128962 | 0.104104 | | 0.106181 | 0.099039 | | 0.106766 |
| NA | 0.162533 | | 0.149817 | 0.148137 | 0.184942 | | 0.174831 | 0.199825 | | 0.185247 |
| NA | 0.170962 | | 0.158361 | 0.156733 | 0.20065 | | 0.192719 | 0.207187 | | 0.195951 |
| NA | 0.134654 | | 0.129572 | 0.127286 | 0.172014 | | 0.159571 | 0.149688 | | 0.158675 |
| NA | 0.173376 | | 0.158092 | 0.157887 | 0.193312 | | 0.180279 | 0.220995 | | 0.191343 |
| NA | NA |  | 0.09571 | 0.098134 | 0.10329 | | 0.101014 | NA |  | NA |
| NA | NA |  | NA | NA | NA |  | NA | NA |  | NA |
| NA | 0.173196 | | 0.15799 | 0.158205 | 0.193071 | | 0.185976 | 0.213064 | | 0.186787 |
| NA | 0.131333 | | 0.122327 | 0.120176 | 0.162909 | | 0.154271 | 0.146727 | | 0.136168 |
| NA | 0.139038 | | 0.128976 | 0.128329 | 0.157634 | | 0.144704 | 0.177824 | | 0.149119 |
| 0.103639 | 0.168084 | | 0.154261 | 0.153234 | 0.195791 | | 0.187853 | 0.209278 | | 0.19457 |
| NA | 0.162104 | | 0.148995 | 0.146997 |  | 0.1814 | 0.170737 | 0.198284 | | 0.176379 |
| NA | 0.134996 | | 0.126217 | 0.126417 | 0.153678 | | 0.150723 | 0.153545 | | 0.150998 |
| NA | 0.134996 | | 0.126217 | 0.126417 | 0.153678 | | 0.150723 | 0.153545 | | 0.150998 |
| NA | 0.170593 | | 0.155846 | 0.154362 | 0.194833 | | 0.186448 | 0.21753 | | 0.191215 |
| NA | 0.142552 | | 0.134843 | 0.135062 | 0.163417 | | 0.15723 |  | 0.168 | 0.160889 |
| NA | 0.128496 | | 0.118413 | 0.118549 | 0.131933 | | 0.120636 | 0.16379 | | 0.138597 |
| NA | 0.146052 | | 0.139931 | 0.140391 | 0.148853 | | 0.142877 | 0.168248 | | 0.148476 |
| NA | NA |  | NA | NA | NA |  | NA | NA |  | NA |
| NA |  | 0.1118 | 0.113439 | 0.114796 | 0.107184 | | 0.107429 | 0.104132 | | 0.113078 |
| NA | 0.168743 | | 0.154229 | 0.152758 | 0.185221 | | 0.177606 | 0.215894 | | 0.178742 |
| NA | 0.17486 | | 0.159643 | 0.158665 | 0.198117 | | 0.190693 | 0.221656 | | 0.195429 |
| 0.158703 | 0.141545 | | 0.136337 | 0.133714 | 0.17764 | | 0.167062 | 0.15352 | | 0.163089 |

| NA | 0.123042 | 0.118032 | 0.123082 | 0.128722 | 0.11617 | 0.104689 | 0.104174 |
| --- | --- | --- | --- | --- | --- | --- | --- |
| NA | 0.119429 | 0.116015 | 0.11837 | 0.120714 | 0.110173 | 0.109208 | NA |
| NA | 0.126673 | 0.121721 | 0.125761 | 0.128082 | 0.118587 | 0.109551 | 0.10993 |
| NA | 0.11904 | 0.11641 | 0.122381 | 0.124377 | 0.115541 | 0.095745 | NA |
| NA | NA | NA | NA | NA | NA | NA | NA |
| NA | NA | NA | NA | NA | NA | NA | NA |
| NA | NA | NA | NA | NA | NA | NA | NA |
| NA | NA | NA | NA | NA | NA | NA | NA |
| NA | NA | NA | NA | NA | NA | 0.090352 | NA |
| NA | NA | NA | NA | NA | NA | 0.089902 | NA |
| NA | NA | NA | NA | NA | NA | NA | NA |
| NA | NA | NA | NA | NA | NA | NA | NA |
| NA | NA | NA | NA | NA | NA | NA | NA |
| NA | NA | NA | NA | NA | NA | NA | NA |
| NA | NA | NA | NA | NA | NA | 0.090694 | NA |
| NA | NA | NA | NA | 0.098649 | NA | 0.093253 | NA |
| NA | NA | NA | NA | NA | NA | NA | NA |
| NA | NA | NA | NA | NA | NA | NA | NA |
| NA | NA | NA | NA | 0.101236 | 0.099882 | 0.096649 | NA |
| NA | NA | NA | NA | 0.093647 | NA | 0.093296 | NA |
| NA | NA | NA | NA | NA | NA | NA | NA |
| 0.106399 | 0.163371 | 0.14999 | 0.149102 | 0.18935 | 0.185854 | 0.201521 | 0.191235 |
| NA | 0.094387 | 0.091541 | 0.090467 | 0.092716 | NA | 0.115284 | 0.107205 |
| NA | 0.094387 | 0.091541 | 0.090467 | 0.092716 | NA | 0.115284 | 0.107205 |
| NA | NA | NA | NA | NA | NA | 0.101915 | NA |
| NA | NA | NA | NA | NA | NA | 0.101915 | NA |
| NA | NA | NA | NA | NA | NA | 0.110984 | NA |
| NA | NA | NA | NA | 0.101455 | NA | 0.12227 | 0.11364 |
| NA | NA | NA | NA | NA | NA | 0.101211 | NA |
| NA | NA | NA | NA | NA | NA | 0.102593 | NA |
| NA | NA | NA | NA | NA | NA | 0.102593 | NA |
| NA | NA | NA | NA | NA | NA | 0.102593 | NA |
| NA | NA | NA | NA | NA | NA | NA | NA |
| 0.114372 | NA | NA | NA | NA | NA | NA | NA |
| 0.112034 | NA | NA | NA | NA | NA | NA | NA |
| 0.111908 | NA | NA | NA | NA | NA | NA | NA |
| NA | 0.102792 | 0.10164 | 0.103279 | 0.111044 | 0.111446 | 0.096523 | 0.104846 |
| 0.118557 | 0.116048 | 0.118732 | 0.121819 | 0.116024 | 0.115729 | 0.095135 | 0.11922 |
| 0.118557 | 0.116048 | 0.118732 | 0.121819 | 0.116024 | 0.115729 | 0.095135 | 0.11922 |

| 0.130988 | 0.124372 | 0.128894 | 0.132541 | 0.129636 | | 0.136703 | 0.098495 | 0.139507 |
| --- | --- | --- | --- | --- | --- | --- | --- | --- |
| NA | 0.108167 | 0.112295 | 0.119721 | NA |  | 0.10838 | NA | NA |
| 0.12815 | 0.118758 | 0.119713 | 0.121995 | 0.116758 | | 0.12406 | 0.106538 | 0.125332 |
| NA | 0.106304 | 0.10971 | 0.112043 | 0.110598 | | 0.109211 | NA | 0.110175 |
| 0.11932 | 0.111871 | 0.111275 | 0.111711 |  | 0.1153 | 0.107935 | 0.11061 | 0.113413 |
| NA | NA | NA | NA | NA |  | NA | 0.116291 | NA |
| NA | NA | NA | NA | NA |  | NA | NA | NA |
| NA | NA | NA | NA | NA |  | NA | NA | NA |
| 0.111418 | 0.101053 | 0.100844 | 0.102511 |  | 0.1142 | 0.109909 | NA | NA |
| 0.124744 | 0.100987 | 0.100252 | 0.100074 | 0.114206 | | 0.112894 | 0.090804 | NA |
| 0.113425 | 0.103119 | 0.104789 | 0.105683 | 0.120241 | | 0.118698 | 0.093248 | NA |
| 0.115939 | NA | NA | NA | NA |  | NA | NA | NA |
| NA | NA | NA | NA | NA |  | NA | NA | NA |
| NA | NA | NA | NA | NA |  | NA | NA | NA |
| NA | NA | NA | NA | NA |  | NA | NA | NA |
| NA | NA | NA | NA | NA |  | NA | NA | NA |
| NA | NA | NA | NA | NA |  | NA | NA | NA |
| NA | NA | NA | NA | NA |  | NA | NA | NA |
| NA | NA | NA | NA | NA |  | NA | NA | NA |
| NA | NA | NA | NA | NA |  | NA | 0.095091 | NA |
| NA | NA | NA | NA | NA |  | NA | NA | NA |
| 0.11903 | NA | NA | NA | NA |  | NA | NA | NA |
| 0.113579 | NA | NA | NA | NA |  | NA | NA | NA |
| 0.125203 | NA | NA | NA | NA |  | NA | NA | NA |
| NA | NA | NA | NA | NA |  | NA | NA | NA |
| NA | NA | NA | NA | NA |  | NA | 0.095701 | NA |
| NA | NA | NA | NA | NA |  | NA | NA | NA |
| NA | NA | NA | NA | NA |  | NA | NA | NA |
| NA | NA | NA | NA | NA |  | NA | NA | NA |
| NA | NA | NA | NA | NA |  | NA | NA | NA |
| NA | NA | NA | NA | NA |  | NA | 0.097055 | NA |
| NA | NA | NA | NA | NA |  | NA | NA | NA |
| NA | NA | NA | NA | NA |  | NA | NA | NA |
| NA | NA | NA | NA | NA |  | NA | NA | NA |
| NA | NA | NA | NA | NA |  | NA | NA | NA |
| NA | NA | NA | NA | NA |  | NA | 0.096037 | NA |
| NA | NA | NA | NA | NA |  | NA | NA | NA |
| NA | NA | NA | NA | NA |  | NA | 0.096766 | NA |
| NA | NA | NA | NA | NA |  | NA | NA | NA |

| 0.120594 | NA | NA | NA | NA | NA | NA |  | NA |
| --- | --- | --- | --- | --- | --- | --- | --- | --- |
| NA | NA | NA | NA | NA | NA | 0.096526 | | NA |
| NA | NA | NA | NA | NA | NA | NA |  | NA |
| NA | NA | NA | NA | NA | NA | NA |  | NA |
| NA | NA | NA | NA | NA | NA | NA |  | NA |
| NA | NA | NA | NA | NA | NA | 0.098849 | | NA |
| NA | NA | NA | NA | NA | NA | NA |  | NA |
| NA | NA | NA | NA | NA | NA | NA |  | NA |
| 0.112787 | NA | NA | NA | NA | NA | NA |  | NA |
| NA | NA | NA | NA | NA | NA | 0.095766 | | NA |
| NA | 0.109891 | 0.104129 | 0.102881 | 0.105545 | 0.10139 | 0.142364 | | 0.105022 |
| NA | 0.112874 | 0.112839 | 0.113318 | NA | NA | 0.119361 | | NA |
| NA | 0.096392 | NA | NA | NA | NA | 0.101958 | | NA |
| NA | 0.112706 | 0.106655 | 0.106331 | 0.092183 | NA |  | 0.1217 | NA |
| NA | 0.164126 | 0.163199 | 0.160407 | 0.109414 | NA | 0.149228 | | 0.126541 |
| NA | 0.118337 | 0.11539 | 0.114202 | NA | NA | 0.110527 | | NA |
| NA | 0.107729 | 0.110212 | 0.111572 | NA | NA | NA |  | NA |
| NA | 0.102038 | 0.098614 | 0.097904 | NA | NA | 0.10431 | | NA |
| NA | 0.123058 | 0.118006 | 0.117227 | 0.097267 | NA | 0.120636 | | NA |
| NA | 0.121283 | 0.117483 | 0.11523 | NA | NA | 0.112537 | | NA |
| NA | 0.122403 | 0.118115 | 0.122952 | 0.128229 | 0.117279 | 0.102472 | | NA |
| NA | 0.116793 | 0.114181 | 0.117731 | 0.124535 | 0.112443 | 0.101109 | | 0.105396 |
| NA | 0.12259 | 0.117938 | 0.122508 | 0.125189 | 0.11146 | 0.10726 | | 0.10306 |
| NA | 0.118527 | 0.115811 | 0.120698 | 0.116854 | 0.107878 | 0.095446 | | NA |
| NA | 0.099701 | 0.099339 | 0.103612 | 0.096545 | NA | NA |  | NA |
| 0.114833 | 0.106783 | 0.108217 | 0.109166 | 0.096553 | NA | 0.100919 | | NA |
| NA | 0.108337 | 0.110692 | 0.115575 | 0.114543 | 0.114652 | NA |  | 0.113349 |
| NA | 0.110103 | 0.112257 | 0.115414 | 0.108897 | 0.110021 | 0.098689 | | 0.106351 |
| NA | 0.110103 | 0.112257 | 0.115414 | 0.108897 | 0.110021 | 0.098689 | | 0.106351 |
| 0.122122 | 0.102696 | 0.102271 | 0.104789 | 0.09821 | 0.096606 | NA |  | 0.100468 |
| 0.116744 | 0.103708 | 0.101961 | 0.103842 | 0.094959 | NA | 0.097277 | | 0.089453 |
| 0.105879 | 0.100617 | 0.100247 | 0.104757 | 0.100325 | NA | NA |  | NA |
| 0.105879 | 0.100617 | 0.100247 | 0.104757 | 0.100325 | NA | NA |  | NA |
| 0.105879 | 0.100617 | 0.100247 | 0.104757 | 0.100325 | NA | NA |  | NA |
| 0.132666 | 0.099455 | 0.096468 | 0.100052 | 0.101253 | 0.092037 | 0.097847 | | 0.092169 |
| 0.145382 | 0.105936 | 0.105659 | 0.106775 | 0.113175 | 0.110984 | 0.103803 | | 0.117171 |
| NA | 0.099046 | 0.098839 | 0.104562 | 0.098791 | NA | NA |  | NA |
| 0.115127 | NA | NA | NA | NA | NA | NA |  | NA |
| 0.117623 | 0.102171 | 0.096927 | 0.094066 | NA | NA | 0.097065 | | NA |

| 0.12266 | NA | NA |  | NA | NA | NA | NA |  | NA |
| --- | --- | --- | --- | --- | --- | --- | --- | --- | --- |
| NA | NA | NA |  | NA | NA | NA | NA |  | NA |
| NA | NA | NA |  | NA | NA | NA | NA |  | NA |
| NA | NA | NA |  | NA | NA | NA | NA |  | NA |
| 0.123794 | 0.115561 | 0.115624 | | 0.116483 | 0.122014 | 0.12056 | 0.106247 | | 0.132615 |
| 0.12212 | 0.11656 | 0.115033 | | 0.115531 | 0.122673 | 0.116648 | 0.109016 | | 0.120811 |
| 0.122825 | 0.111277 | 0.112448 | | 0.113993 | 0.115669 | 0.108652 | 0.09807 | | 0.106054 |
| 0.12212 | 0.117578 | 0.11633 | | 0.115558 | 0.127211 | 0.126998 | 0.115152 | | 0.125611 |
| NA | 0.106792 | 0.107636 | | 0.10846 | 0.113354 | 0.109907 | 0.096883 | | 0.113407 |
| 0.126396 | 0.108402 | 0.109208 | | 0.108628 | 0.11434 | 0.109833 | 0.102577 | | 0.117306 |
| NA | 0.123465 | 0.127593 | | 0.128246 | 0.110689 | 0.112615 | 0.109663 | | 0.118059 |
| 0.176249 | 0.103693 | 0.104262 | | 0.101238 | 0.15542 | 0.15496 | 0.106496 | | 0.151464 |
| 0.16034 | 0.106683 | 0.103524 | | 0.101385 | 0.157145 | 0.154147 | 0.113463 | | 0.155524 |
| NA | 0.10726 | 0.107774 | | 0.108927 | 0.095514 | NA | 0.099696 | | NA |
| 0.130073 | NA | NA |  | NA | NA | NA | NA |  | NA |
| 0.11142 | NA | NA |  | NA | NA | NA | NA |  | NA |
| NA | 0.123933 | 0.126992 | | 0.128941 | 0.102008 | NA | 0.105348 | | 0.109107 |
| NA | NA | NA |  | NA | NA | NA | NA |  | NA |
| NA | 0.113191 | 0.114066 | | 0.113363 | 0.109662 | 0.110812 | 0.109363 | | 0.117603 |
| 0.118037 | NA | NA |  | NA | NA | NA | NA |  | NA |
| 0.118037 | NA | NA |  | NA | NA | NA | NA |  | NA |
| 0.12598 | 0.115648 | 0.115105 | | 0.115606 | 0.118592 | 0.122406 | 0.109081 | | 0.127667 |
| NA | 0.126405 | 0.130649 | | 0.131971 | 0.114763 | 0.120041 | 0.110958 | | 0.123938 |
| NA | 0.126405 | 0.130649 | | 0.131971 | 0.114763 | 0.120041 | 0.110958 | | 0.123938 |
| NA | 0.126405 | 0.130649 | | 0.131971 | 0.114763 | 0.120041 | 0.110958 | | 0.123938 |
| NA | 0.126405 | 0.130649 | | 0.131971 | 0.114763 | 0.120041 | 0.110958 | | 0.123938 |
| 0.105415 | 0.113591 | 0.111458 | | 0.111521 | 0.111811 | 0.10452 | 0.119321 | | 0.120584 |
| 0.105415 | 0.113591 | 0.111458 | | 0.111521 | 0.111811 | 0.10452 | 0.119321 | | 0.120584 |
| 0.105415 | 0.113591 | 0.111458 | | 0.111521 | 0.111811 | 0.10452 | 0.119321 | | 0.120584 |
| 0.105415 | 0.113591 | 0.111458 | | 0.111521 | 0.111811 | 0.10452 | 0.119321 | | 0.120584 |
| 0.120161 | 0.110045 |  | 0.108 | 0.107182 | 0.104016 | 0.103367 |  | 0.1199 | 0.107244 |
| 0.120161 | 0.110045 |  | 0.108 | 0.107182 | 0.104016 | 0.103367 |  | 0.1199 | 0.107244 |
| 0.112427 | 0.107269 | 0.105876 | | 0.105106 | 0.100036 | NA | 0.116386 | | 0.103823 |
| 0.112427 | 0.107269 | 0.105876 | | 0.105106 | 0.100036 | NA | 0.116386 | | 0.103823 |
| NA | 0.136394 | 0.141558 | | 0.142285 | 0.121746 | 0.126088 | 0.117548 | | 0.13196 |
| 0.110748 | 0.115689 | 0.115503 | | 0.11664 | 0.116617 | 0.116106 | 0.107552 | | 0.125802 |
| 0.110748 | 0.115689 | 0.115503 | | 0.11664 | 0.116617 | 0.116106 | 0.107552 | | 0.125802 |
| NA | 0.11084 | 0.110041 | | 0.110332 | 0.110036 | 0.107481 | 0.113346 | | 0.118731 |
| NA | 0.11084 | 0.110041 | | 0.110332 | 0.110036 | 0.107481 | 0.113346 | | 0.118731 |

| 0.1151 | 0.114601 | 0.11559 | 0.115875 | 0.111546 | 0.108874 | 0.105948 | 0.121386 |
| --- | --- | --- | --- | --- | --- | --- | --- |
| 0.130577 | 0.119934 | 0.118967 | 0.120053 | 0.125971 | 0.124292 | 0.112064 | 0.131779 |
| NA | 0.114375 | 0.113285 | 0.115663 | 0.114527 | 0.1136 | 0.106408 | 0.123435 |
| NA | 0.099932 | 0.100691 | 0.10257 N | A N | A | 0.102993 | 0.102227 |

NA	0.118861 0.114294 0.115122 0.116639 0.110205

| 0.114108 | 0.112181 |
| --- | --- |

NA	0.12158	0.11845 0.123132 0.126654 0.120973 0.099749 NA

0.113825 0.118549 0.115306 0.117111 0.117778	0.11232 0.111988 0.119794

NA	0.109217 0.109963 0.111056	0.14988 0.145207 0.108012 0.116706

NA	0.11032 0.110383 0.112161 0.155968 0.150174 0.109163 0.120259

NA	0.097263 0.099902 0.100876 0.115149 0.114966 NA	NA

NA	0.110785 0.111024 0.112258 0.156762	0.1534 0.109409	0.1195

NA	0.105186 0.107943 0.106855 0.133135 0.131359 0.105983 0.109624

NA	0.10007 0.099299 0.102989 NA	NA	NA	NA

0.106839 0.106284 0.104393 0.106244 0.109233	0.10525 0.101516 0.103547

NA	0.097165 0.096594 0.101059 NA	NA	NA	NA NA	NA	NA	NA	NA	NA	NA	NA NA	NA	NA	NA	NA	NA	NA	NA NA	0.097073 0.096529 0.097592 NA	NA	0.096995 NA NA	NA	NA	NA	NA	NA	NA	NA NA	NA	NA	NA	NA	NA	NA	NA NA	NA	NA	NA	NA	NA	NA	NA NA	NA	NA	0.095508 NA	NA	NA	NA NA	0.09667 0.096537 0.097112 NA	NA	NA	NA NA	0.096129 0.094819 0.095941 NA	NA	0.100324 NA NA	0.099511 0.098344 0.097487 NA	NA	0.108438 NA NA	NA	NA	NA	NA	NA	NA	NA

0.111779 NA	NA	NA	NA	NA	NA	NA

0.118741 NA	NA	NA	NA	NA	NA	NA

NA	0.133112 0.137728 0.137826 0.115865 0.117527 0.116546 0.123148

0.129679 0.105478 0.102407 0.101903 0.102572 0.091993 0.107765 0.102544

0.129679 0.105478 0.102407 0.101903 0.102572 0.091993 0.107765 0.102544

0.215569 NA	NA	NA	0.120482 NA	NA	NA

144374

| 0.121516 | 0.099897 | 0.102182 | 0.103323 | 0.109148 | 0.108351 NA | NA |
| --- | --- | --- | --- | --- | --- | --- |
| 0.128343 | 0.097999 | 0.099066 | 0.100189 | 0.108599 | 0.102527 NA | NA |
| 0.126954 | 0.099464 | 0.099615 | 0.099284 | 0.105702 | 0.101349 NA | NA |
| 0.114747 | 0.119547 | 0.116715 | 0.117658 | 0.145604 | 0.145051 0.12 | 288 0. |
| 0.122738 | 0.101462 | 0.102546 | 0.103429 | 0.114717 | 0.11103 0.0892 | 32 NA |
| 0.131716 | NA | 0.095429 | 0.098916 | 0.101796 N | A NA | NA |
| 0.127415 | 0.102031 | 0.102408 | 0.103954 | 0.116304 | 0.112874 NA | NA |

| 0.127915 | 0.099572 | 0.098732 | 0.098393 | 0.106518 | 0.103015 | 0.092755 | NA |
| --- | --- | --- | --- | --- | --- | --- | --- |
| NA | NA | NA | NA | 0.10447 | 0.100647 | NA | NA |
| NA | NA | NA | NA | 0.102693 | 0.100973 | NA | NA |
| NA | 0.097064 | 0.097834 | 0.099125 | 0.110146 | 0.102444 | NA | NA |
| NA | 0.098095 | 0.101196 | 0.10285 | 0.110403 | 0.108418 | NA | NA |
| 0.121656 | 0.116152 | 0.113284 | 0.112952 | 0.123301 | 0.117987 | 0.111088 | 0.121265 |
| NA | NA | NA | NA | NA | NA | NA | NA |
| NA | NA | NA | NA | NA | NA | NA | NA |
| NA | NA | NA | NA | NA | NA | NA | NA |
| NA | NA | NA | NA | NA | NA | NA | NA |
| 0.151819 | 0.108723 | 0.110133 | 0.109813 | 0.115868 | 0.114627 | 0.103951 | 0.119481 |
| 0.127132 | 0.103679 | 0.103786 | 0.107233 | 0.10159 | NA | 0.095519 | NA |
| 0.117608 | 0.111872 | 0.110988 | 0.111914 | 0.116612 | 0.117108 | 0.109966 | 0.115262 |
| 0.114363 | NA | NA | NA | NA | NA | NA | NA |
| 0.135717 | 0.100362 | 0.101428 | 0.104039 | 0.107923 | 0.106074 | NA | 0.109292 |
| 0.134476 | 0.098197 | 0.097895 | 0.098524 | NA | NA | 0.09976 | NA |
| 0.125445 | 0.105485 | 0.103932 | 0.105428 | 0.104873 | 0.102165 | 0.097136 | NA |
| 0.125319 | 0.101902 | 0.103424 | 0.106459 | 0.107842 | 0.105052 | NA | 0.106232 |
| 0.118866 | NA | NA | NA | NA | NA | NA | NA |
| 0.13513 | 0.108231 | 0.109989 | 0.110939 | 0.111023 | 0.11404 | NA | 0.117628 |
| 0.136698 | 0.110091 | 0.112171 | 0.11197 | 0.116429 | 0.115598 | 0.102472 | 0.12689 |
| 0.126511 | 0.100911 | 0.099862 | 0.100231 | 0.111463 | 0.107583 | 0.095886 | NA |
| 0.136144 | 0.099178 | 0.098473 | 0.098449 | 0.111125 | 0.109329 | 0.094341 | NA |
| 0.145967 | 0.100031 | 0.09599 | 0.094704 | 0.13327 | 0.133644 | 0.120028 | 0.142007 |
| 0.107702 | 0.112257 | 0.111215 | 0.110627 | 0.10875 | 0.102259 | 0.104354 | 0.102024 |
| 0.122442 | 0.104171 | 0.104278 | 0.105546 | 0.10926 | 0.105224 | 0.093443 | NA |
| 0.102867 | 0.109285 | 0.108237 | 0.108976 | 0.110115 | 0.107118 | 0.103545 | 0.103782 |
| 0.125086 | 0.102307 | 0.102484 | 0.103772 | 0.103052 | 0.100601 | 0.095918 | 0.096069 |
| 0.118882 | 0.113907 | 0.113799 | 0.114199 | 0.11274 | 0.107486 | 0.111565 | 0.112213 |
| 0.116615 | 0.10582 | 0.106057 | 0.107356 | 0.108635 | 0.105613 | 0.096319 | 0.107144 |
| 0.156823 | 0.118176 | 0.113474 | 0.11518 | 0.127626 | 0.117115 | 0.117815 | 0.118235 |
| 0.135241 | 0.108761 | 0.109162 | 0.110977 | 0.112149 | 0.106687 | 0.104021 | 0.105379 |
| 0.131643 | 0.118579 | 0.117321 | 0.116427 | 0.126037 | 0.119365 | 0.11505 | 0.120785 |
| 0.126914 | 0.107582 | 0.107635 | 0.10863 | 0.110241 | 0.108064 | 0.101095 | 0.108129 |
| 0.147987 | 0.108807 | 0.106909 | 0.108782 | 0.110131 | 0.109372 | 0.10887 | NA |
| 0.144753 | 0.103148 | 0.10207 | 0.104497 | 0.101524 | NA | 0.101133 | NA |
| 0.120129 | NA | NA | 0.096371 | NA | NA | NA | NA |
| 0.120129 | NA | NA | 0.096371 | NA | NA | NA | NA |
| 0.120129 | NA | NA | 0.096371 | NA | NA | NA | NA |

| NA | 0.135184 | 0.13282 | 0.134322 | | NA | NA | 0.141827 | 0.123967 |
| --- | --- | --- | --- | --- | --- | --- | --- | --- |
| NA | 0.138973 | 0.135107 | 0.136023 | | NA | NA | 0.149606 | 0.126657 |
| NA | 0.122838 | 0.121168 | 0.122846 | | NA | NA | 0.130877 | 0.122223 |
| 0.128263 | 0.09979 | 0.099894 | 0.099781 | | 0.112509 | 0.110226 | 0.093129 | NA |
| 0.128293 | 0.096776 | 0.099986 | 0.102318 | | 0.115611 | 0.113368 | NA | NA |
| 0.174923 | 0.100358 | 0.09883 | 0.100245 | | 0.121215 | 0.117405 | NA | NA |
| 0.172067 | 0.098377 | NA | 0.09886 | | 0.117298 | 0.11355 | NA | NA |
| NA | 0.099977 | 0.099584 |  | 0.0994 | NA | NA | 0.099729 | NA |
| 0.125132 | 0.108022 | 0.109066 | 0.109311 | | 0.113092 | 0.106553 | 0.100299 | 0.107631 |
| 0.111917 | 0.110328 | 0.110656 | 0.112031 | | 0.110074 | 0.105734 | 0.099267 | 0.10762 |
| 0.11722 | 0.109489 | 0.110262 | 0.110092 | | 0.117072 | 0.111197 | 0.100828 | 0.106517 |
| 0.11722 | 0.109489 | 0.110262 | 0.110092 | | 0.117072 | 0.111197 | 0.100828 | 0.106517 |
| 0.123981 | 0.110009 | 0.110269 | 0.11036 | | 0.116253 | 0.112223 | 0.102999 | 0.111652 |
| NA | 0.119936 | 0.115931 | 0.121306 | | 0.119962 | 0.106501 | 0.097153 | NA |
| NA | 0.119936 | 0.115931 | 0.121306 | | 0.119962 | 0.106501 | 0.097153 | NA |
| 0.131795 | 0.092762 | NA | NA |  | 0.096215 | NA | 0.095229 | NA |
| 0.13355 | NA | NA | NA |  | 0.098687 | NA | 0.09126 | NA |
| 0.149255 | NA | NA | NA |  | 0.107044 | 0.102289 | 0.092515 | NA |
| 0.118366 | 0.100253 | 0.098041 | 0.100419 | | 0.093557 | NA | NA | 0.086951 |
| NA | 0.139953 | 0.133228 | 0.133339 | | 0.161893 | 0.157122 | 0.158366 | 0.150916 |
| NA | 0.139531 | 0.125936 | 0.12311 | | 0.104958 | NA | 0.229376 | 0.161116 |
| NA | 0.110085 | 0.101806 | 0.101102 | | 0.089295 | NA | 0.153309 | NA |
| NA | 0.123053 | 0.11535 | 0.113573 | | 0.125765 | 0.113273 | 0.167912 | 0.129929 |
| NA | 0.132332 | 0.12157 | 0.118757 | | 0.115192 | 0.104735 | 0.218642 | 0.150927 |
| NA | 0.125037 | 0.114387 | 0.112879 | | 0.108773 | NA | 0.19244 | 0.128796 |
| NA | 0.100046 | 0.096741 | 0.095428 | | 0.116521 | 0.110143 | 0.105915 | 0.099751 |
| NA | 0.111756 | 0.105814 | 0.104668 | | 0.091395 | NA | 0.121063 | NA |
| NA | 0.105688 | 0.102699 | 0.102599 | | 0.121677 | 0.116944 | 0.10674 | 0.105023 |
| NA | 0.105424 | 0.106641 | 0.10823 | | NA | NA | 0.100705 | NA |
| 0.126957 | 0.119431 | 0.118399 | 0.119298 | | 0.120751 | 0.120677 | 0.107633 | 0.124336 |
| NA | 0.113437 | 0.113604 | 0.112935 | | 0.116495 | 0.114031 | 0.117625 | 0.12324 |
| NA | 0.10673 | 0.108162 | 0.110159 | | NA | NA | NA | NA |
| NA | 0.097709 | 0.092328 | NA |  | NA | NA | 0.094019 | NA |
| 0.113043 | NA | NA | NA |  | 0.098398 | NA | NA | NA |
| NA | NA | NA | NA |  | 0.095709 | NA | NA | NA |
| 0.137951 | 0.106103 | 0.107756 | 0.110688 | | 0.119965 | 0.120877 | NA | 0.12139 |
| 0.141321 | 0.113234 | 0.116063 | 0.117393 | | 0.12871 | 0.129174 | 0.099987 | 0.128583 |
| 0.144558 | 0.108975 | 0.111254 | 0.113127 | | 0.128732 | 0.131127 | 0.095971 | 0.132332 |
| NA | NA | NA | 0.096632 | | 0.099554 | 0.105142 | NA | 0.103202 |

| 0.136729 | 0.111072 | 0.112774 | 0.116027 | | 0.129395 | 0.129041 | 0.097752 | 0.131134 |
| --- | --- | --- | --- | --- | --- | --- | --- | --- |
| 0.128566 | 0.119269 | 0.119169 | 0.119904 | | 0.13118 | 0.131259 | 0.109867 | 0.135005 |
| NA | 0.121877 | 0.124672 | 0.126322 | | 0.106318 | 0.109165 | 0.108049 | 0.110627 |
| NA | 0.12092 | 0.123509 | 0.126648 | | 0.106438 | 0.107687 | 0.106936 | 0.111726 |
| NA | 0.115488 | 0.116877 | 0.120205 | | 0.101814 | 0.100466 | 0.102211 | 0.106849 |
| NA | 0.107252 | 0.111594 | 0.111037 | | 0.100978 | 0.105693 | 0.10606 | 0.114401 |
| NA | 0.101227 | 0.101566 | 0.104743 | | 0.116344 | 0.113823 | NA | NA |
| NA | 0.12118 | 0.123889 | 0.126508 | | 0.107152 | 0.109399 | 0.106693 | 0.114219 |
| NA | 0.117619 | 0.121497 | 0.124241 | | 0.106178 | 0.107659 | 0.100581 | 0.11063 |
| 0.118439 | NA | NA | NA |  | NA | NA | NA | NA |
| 0.11782 | NA | NA | NA |  | NA | NA | NA | NA |
| NA | NA | NA | NA |  | NA | NA | NA | NA |
| 0.152678 | 0.107062 | 0.104506 | 0.103095 | | 0.139377 | 0.137383 | 0.119633 | 0.144589 |
| 0.121253 | 0.120753 | 0.122596 | 0.122365 | | 0.124173 | 0.126588 | 0.107209 | 0.128932 |
| NA | 0.10091 | 0.100833 | 0.103045 | | NA | NA | 0.093758 | NA |
| 0.119076 | 0.111561 | 0.111913 | 0.113382 | | 0.119769 | 0.119879 | 0.101225 | 0.12028 |
| NA | 0.100002 | 0.099956 | 0.102264 | | NA | NA | 0.093795 | NA |
| NA | 0.107517 | 0.109587 | 0.110888 | | NA | NA | NA | 0.105971 |
| 0.125841 | 0.107216 | 0.109138 | 0.110289 | | 0.117728 | 0.118399 | 0.094838 | 0.120926 |
| NA | NA | NA | NA |  | NA | NA | NA | NA |
| 0.154552 | 0.119331 | 0.118967 | 0.118686 | | 0.140147 | 0.140547 | 0.115381 | 0.143079 |
| NA | 0.104301 | 0.104151 | 0.10495 | | 0.105932 | 0.098431 | 0.095529 | NA |
| 0.122445 | 0.10168 | 0.104557 | 0.109717 | | 0.117468 | 0.117945 | NA | 0.119966 |
| 0.126288 | 0.102744 | 0.107563 | 0.112755 | | 0.116891 | 0.114201 | NA | 0.11451 |
| 0.148878 | 0.107876 | 0.110613 | 0.112721 | | 0.111745 | 0.115624 | 0.097997 | 0.121883 |
| 0.128788 | 0.101609 | 0.101562 | 0.102428 | | 0.102519 | 0.099902 | 0.102018 | 0.10974 |
| 0.147549 | 0.105051 | 0.106045 | 0.108646 | | 0.115638 | 0.116526 | 0.103135 | 0.121208 |
| 0.143349 | 0.105634 | 0.106911 | 0.109566 | | 0.120146 | 0.11891 | 0.098243 | 0.123937 |
| 0.120858 | 0.104558 | 0.105071 | 0.105049 | | 0.100222 | NA | 0.09898 | NA |
| 0.129835 | 0.111428 | 0.111898 | 0.111377 | | 0.116691 | 0.114297 | 0.107937 | 0.119461 |
| 0.123622 | 0.114264 | 0.113225 | 0.115033 | | 0.110525 | 0.105046 | 0.110207 | 0.101314 |
| 0.135585 | 0.122266 | 0.122708 | 0.122555 | | 0.134976 | 0.13044 | 0.113982 | 0.140356 |
| 0.100802 | 0.109526 | 0.108678 | 0.108586 | | 0.107869 | 0.106278 | 0.106494 | 0.100417 |
| NA | 0.111444 | 0.111612 | 0.114143 | | 0.102064 | NA | 0.107896 | NA |
| 0.125242 | 0.096162 | 0.095729 | 0.095129 | | 0.108707 | 0.106259 | NA | 0.110448 |
| NA | 0.102437 | 0.097925 | 0.097015 | | 0.117143 | 0.108876 | 0.113994 | 0.100437 |
| NA | 0.108799 | 0.103554 | 0.102082 | | 0.127974 | 0.116877 | 0.130231 | 0.108985 |
| 0.125541 | 0.112197 | 0.113945 |  | 0.1142 | 0.120317 | 0.114713 | 0.10341 | 0.109936 |
| 0.133445 | 0.110013 | 0.10738 | 0.110494 | | 0.131018 | 0.133374 | 0.109095 | 0.140463 |

| 0.136187 | | 0.114902 | | 0.113495 | 0.115145 | | 0.124526 | 0.126895 | 0.110746 | 0.122796 |
| --- | --- | --- | --- | --- | --- | --- | --- | --- | --- | --- |
|  | 0.1183 | 0.110494 | | 0.10963 | 0.110962 | | 0.117437 | 0.113713 | 0.110903 | 0.114285 |
| 0.131654 | | 0.118541 | | 0.118357 | 0.119146 | | 0.118935 | 0.116733 | 0.114345 | 0.117231 |
| 0.118414 | | 0.108347 | | 0.111516 | 0.112695 | | 0.102329 | 0.104826 | 0.101366 | 0.107863 |
| 0.127801 | | 0.109806 | | 0.101168 | 0.10161 | | 0.129718 | 0.124108 | 0.135507 | 0.122274 |
| NA |  | 0.107523 | | 0.107814 | 0.108116 | | NA | NA | 0.099427 | NA |
| 0.124653 | | 0.113367 | | 0.112993 | 0.112333 | | 0.122861 | 0.124033 | 0.111206 | 0.124957 |
| 0.117435 | | 0.10741 | | 0.108101 | 0.108985 | | NA | NA | 0.098033 | NA |
| 0.137435 | | 0.112974 | | 0.114893 | 0.115519 | | 0.124105 | 0.12234 | 0.103929 | 0.121846 |
| NA |  | 0.107102 | | 0.106368 | 0.106509 | | 0.104819 | 0.102548 | 0.10618 | 0.105766 |
| 0.126114 | | 0.109744 | | 0.109655 | 0.109884 | | 0.115008 | 0.110945 | 0.102824 | 0.109632 |
| NA |  | NA |  | NA | NA |  | NA | NA | NA | NA |
| 0.110941 | | 0.109618 | | 0.106949 | 0.108876 | | 0.108924 | 0.103353 | 0.106805 | 0.107902 |
| 0.121756 | | 0.099715 | | 0.099158 | 0.100048 | | 0.10521 | 0.102161 | 0.096559 | 0.108137 |
| 0.131166 | | 0.107997 | | 0.108832 | 0.111044 | | 0.105105 | 0.10003 | 0.101172 | 0.110826 |
| 0.148432 | | 0.121555 | | 0.122092 | 0.120777 | | 0.140306 | 0.142805 | 0.113607 | 0.14606 |
| 0.136674 | | 0.103649 | | 0.104737 | 0.10774 | | 0.105909 | 0.110476 | NA | 0.107374 |
| 0.136224 | | 0.114361 | | 0.114504 | 0.114802 | | 0.123418 | 0.125835 | 0.100993 | 0.135301 |
| NA |  | 0.100981 | | 0.102261 |  | 0.1031 | NA | NA | 0.094074 | NA |
| NA |  | 0.107929 | | 0.107526 | 0.107512 | | 0.101329 | NA | 0.10428 | NA |
| 0.121453 | | 0.113919 | | 0.113876 | 0.115195 | | 0.101546 | NA | 0.110035 | 0.101947 |
| NA |  | 0.098295 | | 0.093979 | 0.093132 | | NA | NA | 0.112362 | NA |
| 0.120466 | | 0.109695 | | 0.111388 | 0.113661 | | 0.11843 | 0.11976 | 0.099549 | 0.11849 |
| NA |  | 0.100158 | | 0.096125 | 0.094398 | | 0.101283 | 0.091764 | 0.113187 | 0.093793 |
| 0.118715 | | 0.108826 | | 0.109193 | 0.110515 | | 0.101098 | 0.101028 | 0.098702 | NA |
| NA |  | 0.10598 | | 0.101899 | 0.100749 | | 0.104928 | NA | 0.116157 | 0.097927 |
| 0.117455 | | 0.10543 | | 0.105077 | 0.105898 | | 0.112323 | 0.106856 | 0.108045 | 0.103725 |
| 0.128643 | | 0.100423 | | 0.098785 | 0.100559 | | 0.102968 | 0.094507 | 0.099791 | 0.099518 |
| NA |  | 0.106326 | | 0.109136 | 0.106997 | | 0.104467 | 0.105419 | 0.102326 | 0.109625 |
| 0.112607 | | 0.104089 | | 0.105016 | 0.10756 | | 0.099165 | NA | 0.101872 | NA |
| NA |  | 0.103196 | | 0.100345 | 0.100094 | | 0.098969 | 0.092104 | 0.116389 | 0.098323 |
| 0.152513 | | 0.108077 | | 0.108148 | 0.109495 | | 0.135047 | 0.135038 | 0.104424 | 0.136526 |
| 0.120888 | | 0.105286 | | 0.104937 | 0.107421 | | 0.112299 | 0.115575 | 0.099175 | 0.111646 |
| NA |  | 0.105881 | | 0.104434 | 0.105437 | | NA | NA | 0.112456 | NA |
| 0.12612 | | NA |  | NA | 0.096123 | | NA | NA | NA | NA |
| 0.118748 | | 0.109453 | | 0.109485 | 0.110762 | | 0.108372 | 0.104722 | 0.100243 | 0.110409 |
|  | 0.1214 | 0.110234 | | 0.108996 | 0.109544 | | 0.111052 | 0.113444 | 0.11134 | 0.115525 |
| 0.131059 | |  | 0.1061 | 0.104007 | 0.103662 | | 0.101552 | 0.095217 | 0.106997 | 0.097526 |

fetal_intes germinal_cgranulocyt hapatocyt heart..nih_ hematopoi hematopoi hematopoi

0.100309 0.111189 0.116229 0.104326 0.110681 0.118799	0.11048 0.116011

NA	NA	NA	NA	NA	NA	NA	NA

0.110651 0.118072	0.11337	0.1154 0.116082	0.11224 0.111122 0.112869

0.1004 0.123544 0.108516 0.108892 0.098661 0.119039 0.107123 0.108494

0.099746 0.122463 0.101775	0.11429 0.101869 0.099588 NA	0.102566

0.113118 0.131017 0.124038 0.108468	0.13011 0.138788 0.118261 0.122313

0.106344 0.125364 0.113054	0.1185 0.115157 0.112694 0.108558 0.113179

NA	0.113792	0.10318 0.109241 0.105193 0.106782 0.100889 0.104227

NA	0.113792	0.10318 0.109241 0.105193 0.106782 0.100889 0.104227

0.113171 0.103817 0.100804 0.114966 0.132694 0.099982 0.105274 0.098642

0.104836 0.117353 0.117673 0.105665 0.129506	0.12433 0.111882	0.11513

0.098044 0.117239 0.106134 0.104599 0.109416 0.108746 0.100367	0.10527

0.100187 0.120733 0.108183 0.096646 0.109078 0.105193 0.107038 0.107562

0.107937 0.120986 0.108363	0.1023 0.113057 0.107088 0.106389 0.106862

0.095676 NA	NA	0.105526 NA	NA	NA	NA

0.096904 NA	NA	NA	NA	NA	NA	NA

0.105183 0.103882 0.093828 0.092777 0.098124 NA	0.09711 NA

0.105183 0.103882 0.093828 0.092777 0.098124 NA	0.09711 NA

0.100078 0.125262 0.153238 0.090173 0.108772 0.150413 0.166356 0.152149

0.110037 0.125349 0.110887 0.118578 0.109001 0.109821 0.107517 0.112512

0.097599 NA	NA	NA	NA	NA	NA	NA

0.093889	0.13036 0.145536 0.095763 0.099686 0.146327 0.147737 0.138361

0.11752 NA	NA	NA	NA	NA	NA	NA

0.135846 NA	NA	NA	NA	NA	NA	NA

0.096106 NA	0.092695 0.101532 NA	NA	0.09454 NA NA	NA	NA	0.11455 NA	NA	NA	NA NA	NA	NA	0.114895 NA	NA	NA	NA NA	NA	NA	0.108599 NA	NA	NA	NA

0.108929 0.117439 0.113163 0.119414 0.119018 0.111353 0.112618 0.113487

0.108929 0.117439 0.113163 0.119414 0.119018 0.111353 0.112618 0.113487

0.121748 0.139318	0.12897 0.136763 0.137885 0.131702 0.126649 0.130172

0.125923 0.140875 0.132362 0.139467 0.137622 0.133058 0.130173 0.133245

0.107958 0.098757 0.099153 0.109896 0.122411 NA	0.101381 0.096939

0.098927 NA	NA	0.10228 NA	NA	NA	NA NA	NA	NA	NA	NA	NA	NA	NA NA	NA	NA	NA	NA	NA	NA	NA

0.094634 NA	NA	NA	NA	NA	NA	NA

NA	0.102618 0.110823 0.094651 0.100785 0.118529 0.110102 0.113777

| 0.094727 | 0.126676 | 0.09617 | | 0.09844 | | 0.118387 | | 0.106427 | 0.093125 | | 0.097251 |
| --- | --- | --- | --- | --- | --- | --- | --- | --- | --- | --- | --- |
| 0.094727 | 0.126676 | 0.09617 | | 0.09844 | | 0.118387 | | 0.106427 | 0.093125 | | 0.097251 |
| 0.094727 | 0.126676 | 0.09617 | | 0.09844 | | 0.118387 | | 0.106427 | 0.093125 | | 0.097251 |
| NA | 0.101762 | 0.110163 | | 0.099092 | | 0.099059 | | 0.102946 | 0.107214 | | 0.109529 |
| NA | 0.103046 | 0.108717 | | 0.098011 | | 0.097421 | | 0.103686 | 0.105623 | | 0.108401 |
| 0.103561 | 0.133378 | 0.181922 | | 0.096592 | | 0.123598 | | 0.186598 | 0.192313 | | 0.182578 |
| 0.118663 | 0.144634 | 0.192417 | | 0.102339 | | 0.139564 | | 0.205373 | 0.208993 | | 0.192683 |
| 0.11721 | 0.159947 | 0.215673 | | 0.103193 | | 0.144222 | | 0.230721 | 0.230383 | | 0.213145 |
| 0.113838 | 0.142466 | 0.199931 | | 0.100962 | | 0.139314 | | 0.204629 | 0.20957 | | 0.200049 |
| 0.122073 | 0.14467 | 0.181248 | | 0.105018 | | 0.137009 | | 0.194933 | 0.195688 | | 0.18205 |
| 0.115056 | 0.153678 | 0.192866 | | 0.102726 | | 0.136898 | | 0.207828 | 0.20571 | | 0.190485 |
| 0.118475 | 0.15073 | 0.207335 | | 0.098921 | | 0.137379 | | 0.218959 | 0.227511 | | 0.205909 |
| 0.11932 | 0.147379 | 0.18524 | | 0.107483 | | 0.144005 | | 0.201957 | 0.195158 | | 0.184297 |
| 0.114219 | 0.152729 | 0.193175 | | 0.10451 | | 0.147793 | | 0.209931 | 0.205074 | | 0.191959 |
| 0.119179 | 0.158905 | 0.204636 | | 0.105985 | | 0.151464 | | 0.222705 | 0.216926 | | 0.204313 |
| 0.097025 | NA | NA |  | NA |  | NA |  | NA | NA |  | NA |
| 0.09583 | 0.115656 |  | 0.1015 | 0.11497 | | 0.107952 | | 0.098996 | 0.097371 | | 0.102954 |
| 0.117952 | 0.157666 | 0.208558 | | 0.103972 | | 0.150375 | | 0.224058 | 0.222696 | | 0.205934 |
| 0.115796 | 0.166435 | 0.215903 | | 0.103548 | | 0.155654 | | 0.236625 | 0.232136 | | 0.215242 |
| 0.109185 | 0.134373 | 0.160489 | | 0.107621 | | 0.141106 | | 0.173315 | 0.166437 | | 0.160835 |
| 0.113073 | 0.168227 | 0.231245 | | 0.098767 | | 0.150579 | | 0.248102 | 0.246806 | | 0.230066 |
| 0.115039 | NA | NA |  | 0.114247 | | NA |  | NA | NA |  | NA |
| 0.096349 | NA | NA |  | NA |  | NA |  | NA | NA |  | NA |
| 0.113666 | 0.166232 | 0.220111 | |  | 0.1009 | 0.153675 | | 0.239481 | 0.236755 | | 0.219917 |
| 0.108949 | 0.126952 | 0.155413 | | 0.103052 | | 0.132914 | | 0.168211 | 0.157408 | | 0.156301 |
| 0.107024 | 0.134866 | 0.181917 | | 0.097589 | | 0.128793 | | 0.188709 | 0.192875 | | 0.18007 |
| 0.116776 | 0.164968 | 0.218099 | | 0.104863 | | 0.153988 | | 0.236112 | 0.233354 | | 0.216047 |
| 0.117065 | 0.155579 | 0.203538 | | 0.10072 | |  | 0.1473 | 0.217999 | 0.220549 | | 0.203143 |
| 0.113125 | 0.132523 | 0.15833 | | 0.104338 | | 0.130865 | | 0.169423 | 0.16624 | | 0.157029 |
| 0.113125 | 0.132523 | 0.15833 | | 0.104338 | | 0.130865 | | 0.169423 | 0.16624 | | 0.157029 |
| 0.115085 | 0.166655 |  | 0.2259 | 0.101807 | | 0.152766 | | 0.242412 |  | 0.2422 | 0.223537 |
| 0.105191 | 0.140912 | 0.177957 | | 0.101968 | | 0.135134 | | 0.190429 | 0.184373 | | 0.175125 |
| 0.096511 | 0.133853 | 0.167254 | | NA |  | 0.112435 | | 0.176496 | 0.175156 | | 0.168162 |
| 0.109845 | 0.139878 | 0.171813 | | 0.101411 | | 0.133043 | | 0.18202 | 0.180312 | | 0.171015 |
| 0.096166 | NA | NA |  | NA |  | NA |  | NA | NA |  | NA |
| 0.103844 | 0.118599 | 0.106955 | | 0.105556 | | 0.111176 | | 0.113549 | 0.101862 | | 0.106964 |
| 0.11386 | 0.16095 | 0.217554 | | 0.098228 | | 0.150161 | | 0.230253 | 0.238315 | | 0.216032 |
| 0.115422 | 0.170414 | 0.230574 | | 0.101592 | | 0.154524 | | 0.249105 | 0.247467 | | 0.229433 |
| 0.110365 | 0.138337 | 0.162276 | | 0.109054 | | 0.146295 | | 0.174671 | 0.170716 | | 0.162047 |

| 0.107854 | 0.118024 | 0.105345 | 0.09471 | 0.112583 | 0.100961 | 0.106067 | 0.104197 |
| --- | --- | --- | --- | --- | --- | --- | --- |
| 0.109671 | 0.113005 | 0.107556 | 0.095409 | 0.113571 | 0.101578 | 0.108776 | 0.105638 |
| 0.11084 | 0.122347 | 0.110229 | 0.099082 | 0.117089 | 0.106751 | 0.110144 | 0.108736 |
| 0.099917 | 0.116037 | 0.097422 | 0.094125 | 0.103832 | NA | 0.096393 | 0.095462 |
| 0.152068 | NA | NA | 0.124352 | 0.094019 | NA | NA | NA |
| 0.133367 | NA | NA | 0.115628 | NA | NA | NA | NA |
| 0.142936 | NA | NA | 0.115846 | 0.095307 | NA | NA | NA |
| 0.133134 | NA | NA | 0.110084 | 0.093329 | NA | NA | NA |
| 0.143872 | NA | NA | 0.117308 | 0.098922 | NA | 0.090526 | NA |
| 0.141328 | NA | NA | 0.114804 | 0.09847 | NA | 0.090073 | NA |
| 0.118966 | NA | NA | 0.104477 | NA | NA | NA | NA |
| 0.118966 | NA | NA | 0.104477 | NA | NA | NA | NA |
| 0.118966 | NA | NA | 0.104477 | NA | NA | NA | NA |
| 0.140879 | NA | NA | 0.113908 | NA | NA | NA | NA |
| 0.141136 | NA | NA | 0.115137 | 0.099335 | NA | 0.091512 | NA |
| 0.13961 | NA | 0.092993 | 0.117214 | 0.102584 | NA | 0.093863 | 0.093683 |
| 0.127283 | NA | NA | 0.104655 | 0.096299 | NA | NA | NA |
| 0.127789 | NA | NA | 0.107872 | 0.094435 | NA | NA | NA |
| 0.136192 | NA | 0.096723 | 0.111015 | 0.102155 | NA | 0.098491 | 0.096823 |
| 0.14393 | NA | 0.092284 | 0.116425 | 0.099435 | NA | 0.092963 | NA |
| 0.111669 | NA | NA | 0.094119 | NA | NA | NA | NA |
| 0.116581 | 0.162798 | 0.210333 | 0.105512 | 0.149778 | 0.229536 | 0.223069 | 0.209278 |
| 0.149894 | NA | 0.116954 | 0.105757 | 0.126043 | 0.09889 | 0.113491 | 0.105981 |
| 0.149894 | NA | 0.116954 | 0.105757 | 0.126043 | 0.09889 | 0.113491 | 0.105981 |
| 0.130456 | NA | 0.098741 | 0.093998 | 0.101637 | NA | 0.100118 | NA |
| 0.130456 | NA | 0.098741 | 0.093998 | 0.101637 | NA | 0.100118 | NA |
| 0.161184 | NA | 0.109812 | 0.099433 | 0.125091 | NA | 0.106407 | 0.097852 |
| 0.185521 | NA | 0.123837 | 0.107365 | 0.143476 | 0.099079 | 0.118814 | 0.109513 |
| 0.143234 | NA | 0.098523 | 0.097145 | 0.110089 | NA | 0.098414 | NA |
| 0.144494 | NA | 0.101354 | 0.095503 | 0.115013 | NA | 0.099107 | NA |
| 0.144494 | NA | 0.101354 | 0.095503 | 0.115013 | NA | 0.099107 | NA |
| 0.144494 | NA | 0.101354 | 0.095503 | 0.115013 | NA | 0.099107 | NA |
| 0.093468 | NA | NA | NA | NA | NA | NA | NA |
| 0.094675 | NA | NA | NA | NA | NA | NA | NA |
| 0.094265 | NA | NA | NA | NA | NA | NA | NA |
| 0.094204 | NA | NA | NA | NA | NA | NA | NA |
| 0.09809 | 0.10147 | 0.102083 | 0.104895 | 0.121373 | 0.102641 | 0.097397 | 0.101594 |
| NA | 0.120182 | 0.103205 | 0.108451 | 0.10813 | 0.108291 | 0.097241 | 0.103092 |
| NA | 0.120182 | 0.103205 | 0.108451 | 0.10813 | 0.108291 | 0.097241 | 0.103092 |

| 0.097209 | 0.128378 | 0.106943 | 0.116529 | 0.11032 | | 0.116236 | 0.102478 | 0.108226 |
| --- | --- | --- | --- | --- | --- | --- | --- | --- |
| NA | 0.118044 | NA | 0.108813 | NA |  | NA | NA | NA |
| 0.099818 | 0.123023 | 0.113087 | 0.11026 | 0.109725 | | 0.116153 | 0.109244 | 0.115666 |
| NA | 0.109009 | NA | 0.102009 | 0.103172 | | NA | NA | 0.095956 |
| 0.100993 | 0.111736 | 0.114453 | 0.104575 | 0.108166 | | 0.116752 | 0.112361 | 0.113634 |
| NA | NA | NA | 0.10242 | NA |  | NA | NA | NA |
| 0.123064 | NA | NA | 0.095261 | NA |  | NA | NA | NA |
| 0.129325 | NA | NA | 0.09753 | NA |  | NA | NA | NA |
| 0.178666 | 0.097501 | NA | 0.144858 | 0.106215 | | NA | NA | NA |
| 0.179551 | 0.095884 | NA | 0.146478 |  | 0.1038 | NA | NA | NA |
| 0.167991 | 0.101257 | 0.094841 | 0.141314 | 0.112435 | | 0.098716 | 0.09059 | 0.095859 |
| 0.096831 | NA | NA | NA | NA |  | NA | NA | NA |
| 0.131906 | NA | NA | NA | NA |  | NA | NA | NA |
| 0.113893 | NA | NA | NA | NA |  | NA | NA | NA |
| 0.114877 | NA | NA | NA | NA |  | NA | NA | NA |
| 0.103997 | NA | NA | NA | NA |  | NA | NA | NA |
| 0.095305 | NA | NA | NA | NA |  | NA | NA | NA |
| 0.099532 | NA | NA | NA | NA |  | NA | NA | NA |
| 0.099015 | NA | NA | NA | NA |  | NA | NA | NA |
| 0.116891 | NA | 0.099633 | NA | NA |  | NA | 0.108115 | 0.101815 |
| 0.120465 | NA | NA | NA | NA |  | NA | NA | NA |
| 0.099392 | NA | NA | NA | NA |  | NA | NA | NA |
| 0.097738 | NA | NA | NA | NA |  | NA | NA | NA |
| 0.098676 | NA | NA | NA | NA |  | NA | NA | NA |
| 0.137238 | NA | NA | NA | NA |  | NA | NA | NA |
| 0.125665 | NA | 0.096857 | NA | NA |  | NA | 0.106532 | 0.10058 |
| 0.120862 | NA | NA | NA | NA |  | NA | NA | NA |
| 0.119456 | NA | NA | NA | NA |  | NA | 0.096178 | NA |
| 0.094183 | NA | NA | NA | NA |  | NA | NA | NA |
| 0.094263 | NA | NA | NA | NA |  | NA | NA | NA |
| 0.110779 | NA | 0.097011 | 0.09384 | 0.098157 | | NA | 0.099591 | 0.097517 |
| 0.094597 | NA | NA | NA | NA |  | NA | NA | NA |
| 0.103838 | NA | NA | NA | NA |  | NA | 0.094064 | NA |
| 0.098581 | NA | NA | NA | NA |  | NA | NA | NA |
| 0.130856 | NA | NA | NA | NA |  | NA | NA | NA |
| 0.135559 | NA | NA | NA | NA |  | NA | 0.099606 | NA |
| 0.130786 | NA | NA | NA | NA |  | NA | NA | NA |
| 0.139938 | NA | NA | NA | NA |  | NA | 0.10134 | NA |
| 0.117945 | NA | NA | NA | NA |  | NA | 0.096094 | NA |

| 0.097607 | NA | NA |  | NA | NA | NA | NA | NA |  |
| --- | --- | --- | --- | --- | --- | --- | --- | --- | --- |
| 0.117657 | NA | 0.101932 | | NA | NA | 0.095099 | 0.11094 | 0.105052 | |
| 0.094804 | NA | NA |  | NA | NA | NA | NA | NA |  |
| 0.114731 | NA | NA |  | NA | NA | NA | NA | NA |  |
| 0.127167 | NA | NA |  | NA | NA | NA | NA | NA |  |
| 0.136465 | NA | 0.096102 | | NA | NA | NA | 0.103074 | NA |  |
| 0.117689 | NA | NA |  | NA | NA | NA | NA | NA |  |
| 0.120739 | NA | NA |  | NA | NA | NA | 0.095408 | NA |  |
| 0.09481 | NA | NA |  | NA | NA | NA | NA | NA |  |
| 0.136167 | NA | NA |  | NA | NA | NA | 0.100106 | NA |  |
| 0.094805 | 0.109343 | 0.14217 | | 0.091451 | 0.109063 | 0.13584 | 0.149614 | 0.140665 | |
| 0.122405 | 0.095112 | 0.095053 | | 0.114259 | 0.118007 | NA | 0.121378 | 0.094648 | |
| NA | 0.093664 | 0.095196 | | NA | NA | NA | 0.11085 | 0.096348 | |
| 0.098256 | 0.109442 | 0.11801 | | 0.09398 | 0.099653 | 0.111108 | 0.13199 | 0.11828 | |
| 0.126623 | 0.148705 | 0.137414 | | 0.115058 | 0.122611 | 0.142883 | 0.15524 | 0.140397 | |
| 0.099061 | 0.113166 | 0.10742 | | 0.10229 | 0.101128 | 0.107734 | 0.114883 | 0.106736 | |
| NA | 0.113096 | NA |  | 0.09973 | NA | NA | NA | NA |  |
| 0.09528 | 0.095836 | 0.096262 | | 0.09357 | 0.095185 | NA | 0.11045 | 0.098316 | |
| 0.104082 | 0.120893 | 0.115086 | | 0.100029 | 0.103198 | 0.117991 | 0.127512 | 0.117754 | |
| 0.097662 | 0.111858 | 0.103196 | | 0.097987 | 0.097439 | NA | 0.117459 | 0.106827 | |
| 0.109304 | 0.115956 | 0.101821 | | NA | 0.115248 | 0.099275 | 0.103943 | 0.10003 | |
| 0.10344 | 0.116626 | 0.101237 | | 0.093789 | 0.107712 | 0.098625 | 0.101371 | 0.099567 | |
| 0.109115 | 0.119174 | 0.105449 | | 0.096405 | 0.111442 | 0.102041 | 0.106257 |  | 0.1035 |
| 0.101474 | 0.114149 | 0.096269 | | NA | 0.103118 | NA | 0.097028 | NA |  |
| 0.101481 | NA | 0.099416 | | 0.093422 | NA | NA | NA | 0.099401 | |
| 0.093492 | 0.103904 | 0.102845 | | 0.098686 | 0.101484 | 0.098332 | 0.100413 | 0.101529 | |
| 0.099634 | 0.104467 | 0.100447 | | 0.096557 | NA | 0.100474 | NA | 0.099841 | |
| 0.103403 | 0.10852 |  | 0.1048 | 0.10054 | NA | 0.10703 | 0.100564 | 0.105052 | |
| 0.103403 | 0.10852 |  | 0.1048 | 0.10054 | NA | 0.10703 | 0.100564 | 0.105052 | |
| NA | 0.09726 | 0.097904 | | NA | NA | 0.093863 | NA | 0.098276 | |
| 0.095232 | 0.093912 | 0.107506 | | 0.094579 | NA | 0.097022 | 0.099197 | 0.106856 | |
| 0.099743 | 0.093551 | 0.099765 | | NA | NA | NA | NA | 0.099324 | |
| 0.099743 | 0.093551 | 0.099765 | | NA | NA | NA | NA | 0.099324 | |
| 0.099743 | 0.093551 | 0.099765 | | NA | NA | NA | NA | 0.099324 | |
| 0.101234 | 0.090227 | 0.109538 | | 0.093884 | 0.096562 | 0.098256 | 0.101402 | 0.108101 | |
| 0.09586 | 0.105865 | 0.115227 | | 0.098519 | 0.103972 | 0.116769 | 0.107672 | 0.114005 | |
| 0.097938 | 0.098358 | NA |  | NA | NA | NA | NA | NA |  |
| 0.096995 | NA | NA |  | NA | NA | NA | NA | NA |  |
| 0.123381 | NA | 0.093546 | | 0.103664 | NA | NA | 0.097937 | NA |  |

| 0.099533 | NA |  | NA | NA | NA | NA | NA |  | NA |
| --- | --- | --- | --- | --- | --- | --- | --- | --- | --- |
| 0.099751 | NA |  | NA | NA | NA | NA | NA |  | NA |
| 0.094908 | NA |  | NA | NA | NA | NA | NA |  | NA |
| 0.094496 | NA |  | NA | NA | NA | NA | NA |  | NA |
| 0.102268 | 0.125967 | | 0.112323 | 0.104723 | 0.121434 | 0.128036 | 0.106653 | | 0.111372 |
| 0.108674 | 0.113389 | | 0.111562 | 0.109986 | 0.120973 | 0.118097 | 0.109813 | | 0.111179 |
| NA | 0.110806 | | 0.10273 | 0.106407 | 0.107682 | 0.109388 | 0.098343 | | 0.103644 |
| 0.111967 | 0.115584 | | 0.11794 | 0.108403 | 0.12516 | 0.127243 | 0.115281 | | 0.117799 |
| NA | 0.111729 | | 0.100851 | 0.097732 | 0.098957 | 0.107559 | 0.096958 | | 0.101932 |
| 0.097896 | 0.112824 | | 0.105121 | 0.100168 | 0.103347 | 0.112126 | 0.103105 | | 0.104622 |
| 0.106125 | 0.121693 | | 0.112056 | 0.114679 | 0.111294 | 0.111383 |  | 0.1066 | 0.113208 |
| 0.112638 | 0.112724 | | 0.11858 | 0.108701 | 0.11862 | 0.137583 | 0.118178 | | 0.118309 |
| 0.125142 | 0.119943 | | 0.125127 | 0.112921 | 0.133028 | 0.140692 | 0.126337 | | 0.12626 |
| 0.103212 | 0.106777 | | 0.09922 | 0.103993 | 0.112636 | 0.103077 | 0.10104 | | 0.100638 |
| 0.102067 | NA |  | NA | NA | NA | NA | NA |  | NA |
| 0.096148 | NA |  | NA | NA | NA | NA | NA |  | NA |
| 0.107549 | 0.121804 | | 0.106076 | 0.117787 | 0.102804 | 0.103967 | 0.102148 | | 0.106812 |
| 0.095496 | NA |  | NA | NA | NA | NA | NA |  | NA |
| 0.113062 |  | 0.1189 | 0.112332 | 0.106987 | 0.121343 | 0.119519 | 0.109454 | | 0.111408 |
| 0.101243 | NA |  | NA | NA | NA | NA | NA |  | NA |
| 0.101243 | NA |  | NA | NA | NA | NA | NA |  | NA |
| 0.115195 | 0.115373 | | 0.113213 | 0.113579 | 0.114922 | 0.117746 | 0.112192 | | 0.113789 |
| 0.106075 | 0.122165 | | 0.11585 | 0.116884 | 0.116711 | 0.113419 | 0.109055 | | 0.115782 |
| 0.106075 | 0.122165 | | 0.11585 | 0.116884 | 0.116711 | 0.113419 | 0.109055 | | 0.115782 |
| 0.106075 | 0.122165 | | 0.11585 | 0.116884 | 0.116711 | 0.113419 | 0.109055 | | 0.115782 |
| 0.106075 | 0.122165 | | 0.11585 | 0.116884 | 0.116711 | 0.113419 | 0.109055 | | 0.115782 |
| 0.11068 | 0.111105 | | 0.121441 | 0.106697 | 0.119775 | 0.122337 | 0.120669 | | 0.119039 |
| 0.11068 | 0.111105 | | 0.121441 | 0.106697 | 0.119775 | 0.122337 | 0.120669 | | 0.119039 |
| 0.11068 | 0.111105 | | 0.121441 | 0.106697 | 0.119775 | 0.122337 | 0.120669 | | 0.119039 |
| 0.11068 | 0.111105 | | 0.121441 | 0.106697 | 0.119775 | 0.122337 | 0.120669 | | 0.119039 |
| 0.135992 | 0.107311 | | 0.114698 | 0.118683 | 0.116212 | 0.112101 | 0.122465 | | 0.113248 |
| 0.135992 | 0.107311 | | 0.114698 | 0.118683 | 0.116212 | 0.112101 | 0.122465 | | 0.113248 |
| 0.120467 | 0.103634 | | 0.111949 | 0.111191 | 0.108393 | 0.109332 | 0.117905 | | 0.11088 |
| 0.120467 | 0.103634 | | 0.111949 | 0.111191 | 0.108393 | 0.109332 | 0.117905 | | 0.11088 |
| 0.111831 | 0.129609 | | 0.121538 | 0.123551 | 0.122242 | 0.122624 | 0.117496 | | 0.121766 |
| 0.109564 | 0.127492 | | 0.113762 | 0.105788 | 0.121347 | 0.128022 | 0.108227 | | 0.112535 |
| 0.109564 | 0.127492 | | 0.113762 | 0.105788 | 0.121347 | 0.128022 | 0.108227 | | 0.112535 |
| 0.114566 | 0.118331 | | 0.114077 | 0.105574 | 0.119824 | 0.122429 | 0.113467 | | 0.113357 |
| 0.114566 | 0.118331 | | 0.114077 | 0.105574 | 0.119824 | 0.122429 | 0.113467 | | 0.113357 |

| 0.106129 | 0.126511 | | 0.113286 | 0.105137 | 0.112611 | 0.122047 | 0.106705 | 0.11165 |
| --- | --- | --- | --- | --- | --- | --- | --- | --- |
| 0.112904 | 0.131077 | | 0.119168 | 0.11008 | 0.126039 | 0.132978 | 0.111986 | 0.118847 |
| 0.104254 | 0.120989 | | 0.111831 | 0.108627 | 0.118654 | 0.11871 | 0.105072 | 0.110771 |
| NA | 0.106074 | | 0.103733 | 0.096217 | 0.106877 | 0.101409 | 0.099564 | 0.101157 |
| 0.106881 | 0.11607 | | 0.115503 | 0.099702 | 0.112614 | 0.110881 | 0.114888 | 0.115187 |
| 0.108763 | 0.114852 | | 0.099487 | NA | 0.11366 | NA | 0.101721 | 0.097244 |
| 0.101903 | 0.118222 | | 0.115639 | 0.101753 | 0.112161 | 0.114469 | 0.114191 | 0.116361 |
| 0.098961 | 0.110186 | | 0.117477 | 0.127194 | 0.181429 | 0.103791 | 0.105564 | 0.117658 |
| 0.099469 | 0.111818 | | 0.12027 | 0.129574 | 0.187359 | 0.10637 | 0.106415 | 0.119843 |
| NA | 0.098317 | | NA | 0.109213 | 0.126423 | NA | NA | NA |
| 0.100545 | 0.112377 | | 0.120175 | 0.131401 | 0.190266 | 0.104582 | 0.10695 | 0.120324 |
| 0.096871 | 0.102799 | | 0.108791 | 0.120707 | 0.153656 | 0.103383 | 0.103325 | 0.108816 |
| NA | 0.116482 | | NA | NA | NA | NA | NA | NA |
| NA |  | 0.1149 | 0.107267 | 0.095755 | 0.103857 | 0.108872 | 0.104581 | 0.109133 |
| NA | 0.116668 | | NA | NA | NA | NA | NA | NA |
| NA | 0.095286 | | NA | NA | NA | NA | NA | NA |
| NA | NA |  | NA | NA | NA | NA | NA | NA |
| NA | 0.095202 | | 0.096018 | 0.092946 | 0.100218 | NA | 0.097039 | 0.096463 |
| NA | NA |  | NA | NA | NA | NA | NA | NA |
| NA | NA |  | NA | NA | NA | NA | NA | NA |
| NA | 0.096667 | | NA | NA | NA | NA | NA | NA |
| NA | NA |  | NA | NA | NA | NA | NA | NA |
| NA | NA |  | NA | NA | 0.094922 | NA | NA | NA |
| NA | 0.092307 | | 0.099241 | NA | NA | NA | 0.100559 | 0.099053 |
| NA | 0.095317 | | 0.109033 | NA | 0.096318 | 0.104193 | 0.110324 | 0.107965 |
| 0.094857 | NA |  | NA | NA | NA | NA | NA | NA |
| 0.095754 | NA |  | NA | NA | NA | NA | NA | NA |
| 0.096385 | NA |  | NA | NA | NA | NA | NA | NA |
| 0.112609 | 0.126974 | | 0.120022 | 0.123267 | 0.121491 | 0.117452 | 0.115963 | 0.120461 |
| 0.101707 | 0.098271 | | 0.118109 | 0.100051 | 0.106025 | 0.111354 | 0.114323 | 0.116019 |
| 0.101707 | 0.098271 | | 0.118109 | 0.100051 | 0.106025 | 0.111354 | 0.114323 | 0.116019 |
| 0.236393 | NA |  | NA | 0.184939 | NA | NA | NA | NA |
| 0.102209 | 0.102817 | | NA | 0.115215 | 0.095259 | NA | NA | NA |
| 0.117271 | 0.096484 | | NA | 0.121434 | 0.096902 | NA | NA | NA |
| 0.140586 | 0.09533 | | 0.094722 | 0.126724 | NA | NA | NA | 0.094729 |
| 0.099899 | 0.126735 | | 0.131319 | 0.100723 | 0.115947 | 0.146388 | 0.133713 | 0.134332 |
| 0.158664 | 0.097936 | | NA | 0.136501 | 0.100905 | NA | NA | NA |
| 0.102972 | NA |  | NA | 0.113615 | NA | NA | NA | NA |
| 0.144925 |  | 0.0991 | NA | 0.131285 | 0.101188 | NA | NA | NA |

| 0.145086 | NA | 0.092387 | 0.127964 | 0.097604 | NA | 0.091169 | 0.092617 |
| --- | --- | --- | --- | --- | --- | --- | --- |
| 0.124561 | NA | NA | 0.113105 | 0.101129 | NA | NA | NA |
| 0.127537 | NA | NA | 0.115628 | 0.099917 | NA | NA | NA |
| 0.131296 | NA | NA | 0.122374 | 0.099913 | NA | NA | NA |
| 0.115532 | 0.097079 | NA | 0.114278 | 0.103973 | NA | NA | NA |
| 0.113076 | 0.111496 | 0.112477 | 0.109956 | 0.122809 | 0.117895 | 0.111311 | 0.111531 |
| 0.097192 | NA | NA | 0.10417 | NA | NA | NA | NA |
| 0.097192 | NA | NA | 0.10417 | NA | NA | NA | NA |
| 0.097192 | NA | NA | 0.10417 | NA | NA | NA | NA |
| NA | NA | NA | NA | NA | NA | NA | NA |
| 0.100241 | 0.152153 | 0.10512 | 0.104715 | 0.106347 | 0.112096 | 0.106818 | 0.105886 |
| 0.097911 | 0.125094 | NA | 0.103589 | 0.098457 | 0.097053 | NA | NA |
| 0.108416 | 0.115952 | 0.10885 | 0.107414 | 0.114023 | 0.110854 | 0.109412 | 0.108717 |
| NA | NA | NA | NA | NA | NA | NA | NA |
| NA | 0.132279 | NA | 0.09916 | 0.095204 | 0.099488 | NA | NA |
| 0.100219 | 0.11404 | 0.096416 | 0.097391 | 0.096504 | 0.097225 | 0.100445 | 0.09763 |
| 0.101274 | 0.107236 | 0.097497 | 0.10291 | 0.104301 | 0.098998 | 0.09645 | 0.097456 |
| NA | 0.118869 | NA | 0.099272 | 0.099989 | 0.097852 | NA | NA |
| NA | NA | NA | NA | NA | NA | NA | NA |
| NA | 0.111915 | 0.106119 | 0.09551 | 0.094639 | 0.115957 | 0.095118 | 0.107634 |
| 0.100442 | 0.110305 | 0.112544 | 0.099047 | 0.099969 | 0.119537 | 0.104092 | 0.112765 |
| 0.125402 | 0.096854 | 0.098431 | 0.119262 | 0.102153 | 0.097914 | 0.095279 | 0.098951 |
| 0.146943 | 0.093995 | 0.096768 | 0.128879 | 0.09984 | 0.094247 | 0.092138 | 0.09792 |
| 0.115724 | 0.119579 | 0.126034 | 0.104995 | 0.104818 | 0.135004 | 0.125526 | 0.124968 |
| 0.101916 | 0.111059 | 0.107484 | 0.104077 | 0.112857 | 0.11361 | 0.105901 | 0.108115 |
| 0.10814 | 0.097982 | 0.094301 | 0.112369 | 0.098702 | 0.096118 | NA | 0.094234 |
| NA | 0.117706 | 0.109038 | 0.096116 | 0.105565 | 0.111617 | 0.107514 | 0.109703 |
| 0.094713 | 0.109383 | 0.098939 | 0.098173 | 0.103249 | 0.099144 | 0.097795 | 0.100647 |
| 0.106974 | 0.111228 | 0.111112 | 0.105486 | 0.115607 | 0.114961 | 0.110209 | 0.111045 |
| 0.096526 | 0.10557 | 0.097791 | 0.098769 | 0.108251 | 0.102722 | 0.096605 | 0.098213 |
| 0.135391 | 0.111906 | 0.117848 | 0.112382 | 0.133446 | 0.11675 | 0.118986 | 0.119926 |
| 0.106751 | 0.107629 | 0.105553 | 0.102871 | 0.115561 | 0.10816 | 0.10345 | 0.104225 |
| 0.125626 | 0.114487 | 0.116644 | 0.109765 | 0.129157 | 0.121547 | 0.118383 | 0.117719 |
| 0.100404 | 0.110369 | 0.105827 | 0.100894 | 0.111252 | 0.106348 | 0.103006 | 0.104813 |
| 0.115749 | 0.101873 | 0.10853 | 0.104371 | 0.122654 | 0.10693 | 0.108274 | 0.106693 |
| 0.11118 | 0.100701 | NA | 0.097669 | 0.111288 | NA | 0.102097 | NA |
| NA | NA | NA | 0.097162 | NA | NA | NA | NA |
| NA | NA | NA | 0.097162 | NA | NA | NA | NA |
| NA | NA | NA | 0.097162 | NA | NA | NA | NA |

| NA |  | 0.127164 | 0.144185 | NA |  | NA | 0.13953 | 0.14754 | 0.13571 |
| --- | --- | --- | --- | --- | --- | --- | --- | --- | --- |
| NA |  | 0.129576 | 0.15204 | NA |  | NA | 0.146987 | 0.154381 | 0.142556 |
| NA |  | 0.116584 | 0.136059 | NA |  | NA | 0.133924 | 0.136408 | 0.12954 |
|  | 0.1479 | 0.096899 | 0.096225 | 0.130345 | | 0.098033 | NA | NA | 0.096447 |
| 0.122993 | | 0.098466 | NA | 0.124784 | | 0.096009 | NA | NA | NA |
| 0.198494 | | NA | NA | 0.164032 | | 0.101958 | NA | NA | NA |
| 0.185606 | | NA | NA | 0.156052 | | 0.100386 | NA | NA | NA |
| 0.103687 | | 0.097142 | 0.096822 | 0.099857 | | 0.10107 | NA | 0.098523 | 0.09533 |
| 0.099358 | | 0.106934 | 0.107236 | 0.102478 | | 0.110557 | 0.110023 | 0.100654 | 0.105079 |
| 0.102653 | | 0.10812 | 0.105189 | 0.108208 | | 0.115119 | 0.108721 | 0.101101 | 0.104849 |
| 0.102941 | | 0.106405 | 0.104867 | 0.108434 | | 0.123647 | 0.108454 | 0.100043 | 0.103862 |
| 0.102941 | | 0.106405 | 0.104867 | 0.108434 | | 0.123647 | 0.108454 | 0.100043 | 0.103862 |
| 0.106505 | | 0.106903 | 0.106648 | 0.108823 | | 0.121564 | 0.112412 | 0.103949 | 0.10572 |
| 0.098026 | | 0.119421 | 0.097676 | 0.093839 | | 0.10345 | NA | 0.097871 | 0.095764 |
| 0.098026 | | 0.119421 | 0.097676 | 0.093839 | | 0.10345 | NA | 0.097871 | 0.095764 |
| 0.113387 | | NA | 0.091937 | 0.097296 | | 0.094234 | NA | 0.094343 | NA |
| 0.105109 | | NA | NA | 0.093717 | | NA | NA | 0.093224 | NA |
| 0.109406 | | NA | NA | 0.096886 | | 0.093572 | NA | 0.092517 | NA |
| NA |  | NA | 0.104968 | NA |  | NA | 0.092875 | 0.096841 | 0.103955 |
| 0.110154 | | 0.133603 | 0.162175 | 0.104636 | | 0.134926 | 0.175367 | 0.172687 | 0.161995 |
| 0.104284 | | 0.159048 | 0.219299 | 0.091379 | | 0.110291 | 0.225293 | 0.248468 | 0.216943 |
| 0.094187 | | 0.110234 | 0.149772 | NA |  | 0.095717 | 0.140589 | 0.162367 | 0.14746 |
| 0.104642 | | 0.126995 | 0.169124 |  | 0.0944 | 0.117604 | 0.174833 | 0.179579 | 0.167005 |
| 0.103427 | | 0.15063 | 0.206404 | 0.091727 | | 0.113819 | 0.215871 | 0.231275 | 0.202369 |
| 0.102381 | | 0.131968 | 0.185912 | 0.092343 | | 0.107024 | 0.184956 | 0.207033 | 0.182099 |
| 0.100873 | | 0.095408 | 0.105104 | 0.096189 | | 0.113999 | 0.108174 | 0.114922 | 0.105551 |
| 0.099434 | | 0.112753 | 0.114984 | 0.091673 | | 0.101063 | 0.111645 | 0.130177 | 0.11508 |
| 0.104187 | | 0.098581 | 0.106781 | 0.100894 | | 0.121337 | 0.108524 | 0.116739 | 0.108369 |
| 0.099276 | | 0.105755 | 0.100731 | 0.10391 | | 0.102573 | 0.101794 | 0.099894 | 0.10045 |
| 0.10771 | | 0.128682 | 0.116011 | 0.107406 | | 0.118186 | 0.127036 | 0.109041 | 0.115775 |
| 0.118504 | | 0.12221 | 0.119535 | 0.108427 | | 0.133077 | 0.130951 | 0.119752 | 0.118872 |
| 0.094187 | | 0.117668 | NA | 0.098193 | | NA | 0.095724 | NA | NA |
| 0.124904 | | NA | NA | 0.102396 | | NA | NA | 0.095059 | NA |
| 0.106803 | | NA | NA | 0.096467 | | 0.114212 | NA | NA | NA |
| 0.102179 | | NA | NA | 0.093393 | | 0.111585 | NA | NA | NA |
| NA |  | 0.113894 | 0.098054 | 0.09833 | | 0.100403 | 0.11114 | NA | 0.101083 |
| 0.100065 | | 0.119361 | 0.109219 | 0.106591 | | 0.112837 | 0.119315 | 0.10101 | 0.109492 |
| NA |  | 0.118683 | 0.107473 | 0.100568 | | 0.106191 | 0.121093 | 0.09766 | 0.10981 |
| NA |  | 0.099504 | NA | NA |  | NA | NA | NA | NA |

| 0.097655 | 0.118731 | 0.107999 | |  | 0.105 | 0.109431 | 0.119234 | 0.099651 | 0.109332 | |
| --- | --- | --- | --- | --- | --- | --- | --- | --- | --- | --- |
| 0.10886 | 0.126915 | 0.118311 | | 0.109366 | | 0.128713 | 0.132187 | 0.10987 | 0.117128 | |
| 0.106709 | 0.120906 | 0.108671 | | 0.115188 | | 0.108094 | 0.105599 | 0.104352 | 0.109077 | |
| 0.103906 | 0.120392 | 0.107913 | | 0.113415 | | 0.109016 | 0.104669 | 0.102973 | 0.108559 | |
| 0.099558 | 0.116319 | 0.102727 | | 0.108811 | | 0.103887 | 0.101158 | 0.097387 | 0.102807 | |
| 0.10575 | 0.112292 | 0.105802 | | 0.107265 | | 0.11791 | 0.11162 | 0.10673 | 0.106366 | |
| NA | 0.106343 | 0.095183 | | 0.108953 | | 0.131912 | NA | NA | 0.095462 | |
| 0.10393 | 0.122764 | 0.108371 | | 0.114169 | | 0.109001 | 0.107061 | 0.10187 | 0.109102 | |
| 0.098823 | 0.119641 | 0.102438 | | 0.111566 | | 0.103816 | 0.10254 | 0.095906 | 0.103973 | |
| 0.094168 | NA | NA |  | NA |  | NA | NA | NA | NA |  |
| 0.095709 | NA | NA |  | NA |  | NA | NA | NA | NA |  |
| NA | NA | NA |  | NA |  | NA | NA | NA | NA |  |
| 0.115459 | 0.122803 | 0.130265 | | 0.105482 | | 0.110467 | 0.141643 | 0.128354 | 0.130099 | |
| 0.101142 | 0.115842 | 0.113274 | | 0.110119 | | 0.109483 | 0.123412 | 0.108013 | 0.113071 | |
| NA | 0.107612 | 0.09659 | | 0.095489 | | 0.100255 | 0.101276 | NA | 0.09545 | |
| 0.100571 | 0.116888 | 0.109816 | | 0.106138 | | 0.111355 | 0.114317 | 0.107771 | 0.109731 | |
| NA | 0.106311 | 0.096554 | | 0.094465 | | 0.100863 | 0.100033 | NA | 0.094616 | |
| NA | 0.105803 | 0.097633 | | 0.093981 | | NA | 0.098148 | NA | 0.097049 | |
| 0.095954 | 0.108553 | 0.10235 | | 0.101553 | | 0.100203 | 0.110293 | NA | 0.102748 | |
| 0.098974 | NA | NA |  | NA |  | NA | NA | NA | NA |  |
| 0.116628 | 0.12205 | 0.120776 | | 0.117794 | | 0.128052 | 0.132487 | 0.120314 | 0.120782 | |
| 0.102716 | 0.101627 | 0.095863 | | 0.105286 | | 0.108469 | 0.096269 | 0.09638 | 0.096957 | |
| NA | 0.107701 | NA |  | 0.096363 | | NA | NA | NA | NA |  |
| NA | 0.10717 | NA |  | 0.098261 | | NA | NA | NA | NA |  |
| NA | 0.111191 | 0.107958 | | 0.100466 | | NA | 0.114844 | 0.100297 | 0.107133 | |
| 0.094102 | 0.099232 | 0.113686 | | 0.097009 | | 0.096716 | 0.10762 | 0.104905 |  | 0.1138 |
| 0.09473 | 0.107586 | 0.111083 | | 0.099996 | | 0.099829 | 0.117908 | 0.105498 | 0.112314 | |
| NA | 0.116102 | 0.106358 | | 0.099399 | | 0.101535 | 0.118428 | 0.100342 | 0.106379 | |
| NA | 0.096852 | 0.105941 | | 0.098006 | | 0.09518 | 0.100747 | 0.100921 | 0.105385 | |
| 0.104381 | 0.117285 | 0.113583 | | 0.109282 | | 0.10962 | 0.119732 | 0.112079 | 0.113761 | |
| 0.104523 | 0.10236 | 0.120363 | | 0.110116 | | 0.106791 | 0.113904 | 0.115298 | 0.11916 | |
| 0.110854 | 0.12322 |  | 0.1178 | 0.113923 | | 0.125643 | 0.132037 | 0.11726 | 0.119043 | |
| 0.099227 | 0.105041 | 0.107158 | | 0.104259 | | 0.111684 | 0.108404 | 0.106858 | 0.106182 | |
| 0.103158 | 0.109357 | 0.108463 | | 0.106703 | | 0.112562 | 0.106227 | 0.109599 | 0.10904 | |
| NA | 0.104094 | 0.099935 | | 0.095168 | | NA | 0.107605 | 0.099028 | 0.101495 | |
| 0.100472 | 0.097427 | 0.11516 | | 0.093795 | | 0.110194 | 0.114553 | 0.121986 | 0.116581 | |
| 0.099158 | 0.106446 | 0.126045 | | 0.092786 | | 0.11526 | 0.127383 | 0.136957 | 0.127349 | |
| 0.097656 | 0.10778 | 0.110431 | | 0.107926 | | 0.11394 | 0.112952 | 0.104679 | 0.110493 | |
| 0.111464 | 0.112373 | 0.112139 | | 0.105163 | | 0.112341 | 0.124314 | 0.114818 | 0.111491 | |

| 0.102247 | 0.116778 | | 0.117974 | 0.104987 | 0.118142 | | 0.12124 | 0.112791 | 0.115298 |
| --- | --- | --- | --- | --- | --- | --- | --- | --- | --- |
| 0.102457 | 0.10873 | | 0.119174 | 0.105747 | 0.110184 | | 0.120943 | 0.113577 | 0.118406 |
| 0.126285 |  | 0.1154 | 0.113519 | 0.125433 | 0.127542 | | 0.118218 | 0.116219 | 0.113382 |
| NA | 0.10787 | | 0.106035 | 0.101755 | 0.102053 | | 0.10779 | 0.102371 | 0.105802 |
| 0.131652 | 0.110973 | | 0.130843 | 0.12092 | 0.124787 | | 0.130705 | 0.135664 | 0.129905 |
| 0.099667 | NA |  | NA | 0.102717 | 0.10952 | | NA | 0.099276 | NA |
| 0.110189 | 0.113796 | | 0.116137 | 0.104674 | 0.118479 | | 0.125071 | 0.112979 | 0.116265 |
| 0.098666 | 0.111819 | | 0.102463 | 0.101282 | 0.11476 | | NA | 0.098789 | 0.103556 |
| 0.102308 | 0.112559 | | 0.111157 | 0.109491 | 0.113766 | | 0.118376 | 0.106082 | 0.109629 |
| 0.098487 | 0.103189 | | 0.110531 | 0.102018 | 0.107518 | | 0.11074 | 0.106683 | 0.109424 |
| 0.097066 | 0.103631 | | 0.109861 | 0.103351 | 0.107933 | | 0.109705 | 0.105256 | 0.110464 |
| 0.115064 | NA |  | NA | NA | NA |  | NA | NA | NA |
| NA | 0.108335 | | 0.109411 | 0.096366 | 0.10218 | | 0.114541 | 0.109598 | 0.110432 |
| 0.096498 | 0.101033 | | 0.106753 | 0.096434 | NA |  | 0.105347 | 0.101097 | 0.106163 |
| NA | 0.106669 | | 0.111976 | 0.099669 | 0.094991 | | 0.107601 | 0.105115 | 0.111079 |
| 0.109688 | 0.125421 | | 0.121593 | 0.108477 |  | 0.13 | 0.138302 | 0.116523 | 0.123052 |
| 0.096112 | 0.105712 | | 0.097951 | 0.100875 | 0.102782 | | 0.103499 | NA | 0.098726 |
| 0.101579 | 0.117969 | | 0.109649 | 0.10326 | 0.113153 | | 0.123618 | 0.10489 | 0.111025 |
| NA | 0.098059 | | NA | 0.094596 | 0.094196 | | NA | 0.094865 | NA |
| NA | 0.103764 | | 0.106286 | 0.099007 | 0.102192 | | 0.105743 | 0.104798 | 0.105978 |
| 0.099489 | 0.104412 | | 0.115798 | 0.105537 | 0.110454 | | 0.108712 | 0.112161 | 0.113788 |
| 0.096759 | NA |  | 0.106731 | NA | 0.094348 | | 0.095242 | 0.119264 | 0.106788 |
| 0.09526 | 0.110413 | | 0.10502 | 0.103965 | 0.110344 | | 0.112062 | 0.099495 | 0.10593 |
| 0.094685 | 0.093421 | | 0.12188 | 0.090419 | 0.095704 | | 0.116976 | 0.121291 | 0.122815 |
| 0.096871 | 0.111398 | | 0.107222 | 0.104795 | 0.112406 | | NA | 0.100607 | 0.108062 |
| 0.102715 |  | 0.1024 | 0.115815 | 0.100274 | 0.101822 | | 0.115833 | 0.122122 | 0.115724 |
| 0.107379 | 0.103902 | | 0.108429 | 0.109065 | 0.114198 | | 0.108961 | 0.10673 | 0.108171 |
| 0.094658 | 0.092074 | | 0.112175 | 0.097446 | 0.096995 | | 0.104127 | 0.103225 | 0.111664 |
| 0.103918 | 0.103859 | | 0.102713 | 0.104993 | 0.110696 | | 0.107604 | 0.101572 | 0.101892 |
| 0.101454 | 0.100816 | | 0.10108 | 0.102379 | 0.105135 | | 0.100416 | 0.100554 | 0.102137 |
| 0.09623 |  | 0.0978 | 0.118951 | 0.094614 | 0.101546 | | 0.11545 | 0.120904 | 0.118032 |
| 0.107994 | 0.117238 | | 0.116437 | 0.108294 | 0.108922 | | 0.126938 | 0.114404 | 0.117165 |
| 0.095559 | 0.102381 | | 0.107288 | 0.10191 | 0.105983 | | 0.107759 | 0.099772 | 0.106592 |
| 0.095613 | 0.102539 | | 0.111424 | 0.095662 | 0.097288 | | 0.10966 | 0.111901 | 0.108732 |
| NA | 0.104034 | | NA | 0.096564 | NA |  | NA | NA | NA |
| 0.101889 | 0.110622 | | 0.102873 | 0.105111 | 0.108081 | | 0.105638 | 0.100747 | 0.10406 |
| 0.10272 | 0.107446 | | 0.111363 | 0.104234 | 0.107134 | | 0.112747 | 0.112569 | 0.110763 |
| 0.100587 | 0.099028 | | 0.11494 | 0.099171 | 0.102055 | | 0.106765 | 0.110055 | 0.114568 |

hematopoi hematopoi hematopoi hepatocyt hesc_deriv hesc_deriv hesc_deriv immature_

0.115317 0.113543 0.112687 0.099001 NA	0.125316 0.148649 0.111457

NA	NA	NA	NA	NA	NA	NA	NA

0.111173 0.111108 0.112807 0.111718 NA	NA	NA	0.111948

0.107961 0.108693 0.108636 0.102715 NA	NA	NA	0.1047

0.098666 0.097893 0.105682 0.108864 NA	NA	NA	0.09884

0.127002 0.118182 0.116172 0.105378 NA	0.156164 NA	0.115371

0.109458 0.109582 0.112032 0.114057 NA	NA	NA	0.113876

0.103247 0.102129	0.10449 0.105025 NA	NA	NA	0.10138

0.103247 0.102129	0.10449 0.105025 NA	NA	NA	0.10138

NA	NA	0.10454 0.114533 NA	NA	NA	0.119438

0.118375 0.112552 0.111498 0.103075 NA	0.139052 NA	0.112011

0.106828 0.103594 0.105412 0.102013 NA	0.118992 NA	0.101256

0.100654 0.102621 0.103616	0.09524 NA	NA	0.218941 0.121083

0.104168 0.102984 0.105618	0.09914 NA	NA	0.229348	0.12051

NA	NA	NA	0.109148 NA	NA	NA	NA NA	NA	NA	NA	NA	NA	NA	NA

NA	NA	NA	0.093791 NA	NA	0.164855 0.103046

NA	NA	NA	0.093791 NA	NA	0.164855 0.103046

0.159062 0.152008 0.135829 0.092765 NA	NA	NA	0.173825

0.110803 0.109679 0.112561 0.115599 NA	NA	NA	0.108793

NA	NA	NA	NA	NA	NA	NA	NA

0.138562 0.134652	0.11935 0.094227 NA	NA	NA	0.13678

NA	NA	NA	NA	NA	NA	NA	NA NA	NA	NA	NA	NA	NA	NA	NA

NA	NA	NA	0.10568 NA	NA	NA	0.112537

NA	NA	NA	0.121315 NA	NA	NA	0.129785

NA	NA	NA	0.12201 NA	NA	NA	0.131184

NA	NA	NA	0.114896 NA	NA	NA	0.12314

0.111877 0.110412 0.115611 0.116871 NA	NA	NA	0.110528

0.111877 0.110412 0.115611 0.116871 NA	NA	NA	0.110528

0.128305 0.128656 0.131006 0.132466 NA	NA	NA	0.126669

0.132745	0.13197	0.13404 0.136084 NA	NA	NA	0.131464

NA	NA	0.099722 0.111176 NA	NA	NA	0.116968

NA	NA	NA	0.108686 NA	NA	NA	0.121344

NA	NA	NA	NA	NA	NA	NA	NA NA	NA	NA	NA	NA	NA	NA	NA NA	NA	NA	NA	NA	NA	NA	NA

0.117914 0.113156 0.111509 NA	NA	0.122189 NA	0.103436

| 0.098259 | | 0.099358 | | 0.103215 | 0.09341 | NA | 0.146667 | NA | 0.094641 |
| --- | --- | --- | --- | --- | --- | --- | --- | --- | --- |
| 0.098259 | | 0.099358 | | 0.103215 | 0.09341 | NA | 0.146667 | NA | 0.094641 |
| 0.098259 | | 0.099358 | | 0.103215 | 0.09341 | NA | 0.146667 | NA | 0.094641 |
| 0.112596 | | 0.11064 | | 0.107723 | NA | NA | 0.116803 | NA | 0.107446 |
|  | 0.1116 | 0.109454 | | 0.105224 | NA | NA | 0.108852 | NA | 0.104788 |
| 0.198037 | | 0.181882 | | 0.163845 | 0.095389 | NA | NA | 0.131491 | 0.175667 |
| 0.208394 | | 0.191233 | | 0.173603 | 0.102222 | NA | NA | 0.160149 | 0.192401 |
| 0.23282 | | 0.211721 | | 0.187909 | 0.102235 | NA | NA | 0.146409 | 0.212122 |
| 0.216056 | | 0.198788 | | 0.177863 | 0.100987 | 0.128109 | NA | 0.141588 | 0.19093 |
| 0.195319 | | 0.180787 | | 0.165208 | 0.104434 | NA | NA | 0.159091 | 0.18231 |
| 0.208512 | | 0.188533 | | 0.170151 | 0.101723 | NA | NA | 0.156553 | 0.191685 |
| 0.223713 | | 0.204628 | | 0.177571 | 0.100088 | NA | NA | 0.140097 | 0.211335 |
| 0.19768 | | 0.182122 | | 0.170349 | 0.104822 | NA | NA | 0.15157 | 0.17711 |
| 0.208502 | | 0.188986 | | 0.175168 | 0.103704 | NA | NA | 0.159035 | 0.186835 |
| 0.220585 | | 0.200959 | | 0.185265 | 0.104588 | NA | NA | 0.161783 | 0.196025 |
| NA |  | NA |  | NA | NA | NA | NA | NA | NA |
| 0.099733 | | 0.101601 | | 0.108004 | 0.11011 | NA | NA | NA | 0.100399 |
| 0.225934 | | 0.202939 | | 0.185671 | 0.103224 | NA | NA | 0.182075 | 0.198712 |
| 0.233485 | | 0.210906 | | 0.194731 | 0.103822 | NA | NA | 0.176415 | 0.205775 |
| 0.166804 | | 0.159109 | | 0.15889 | 0.108967 | NA | NA | NA | 0.152612 |
| 0.253453 | | 0.227155 | | 0.200057 | 0.100202 | NA | NA | 0.14761 | 0.220331 |
| NA |  | NA |  | NA | 0.112738 | NA | NA | NA | NA |
| NA |  | NA |  | NA | NA | NA | NA | NA | NA |
| 0.240407 | |  | 0.2159 | 0.19566 | 0.101728 | NA | NA | 0.152476 | 0.210617 |
| 0.166401 | | 0.158581 | | 0.146939 | 0.101019 | NA | NA | NA | 0.148326 |
| 0.193976 | | 0.178126 | | 0.161232 | 0.096372 | NA | NA | NA | 0.18104 |
| 0.237035 | | 0.212661 | | 0.192777 | 0.10403 | NA | NA | 0.179976 | 0.207693 |
| 0.220361 | | 0.198848 | | 0.186346 | 0.102257 | NA | NA | 0.170146 | 0.196723 |
| 0.16721 | |  | 0.1542 | 0.147356 | 0.102078 | NA | NA | 0.157559 | 0.153458 |
| 0.16721 | |  | 0.1542 | 0.147356 | 0.102078 | NA | NA | 0.157559 | 0.153458 |
| 0.245597 | | 0.22041 | | 0.197974 | 0.102162 | NA | NA | 0.16093 | 0.215627 |
| 0.188719 | | 0.175176 | | 0.160702 | 0.098931 | NA | NA | 0.151268 | 0.167452 |
| 0.181054 | | 0.169239 | | 0.152491 | NA | NA | NA | NA | 0.170648 |
| 0.182293 | | 0.172211 | | 0.157829 | 0.100868 | NA | NA | NA | 0.168276 |
| NA |  | NA |  | NA | NA | NA | NA | NA | NA |
| 0.108054 | | 0.105228 | | 0.106723 | 0.103344 | NA | 0.135777 | NA | 0.10428 |
| 0.236243 | | 0.212948 | | 0.193857 | 0.100348 | NA | NA | 0.156061 | 0.214137 |
| 0.251772 | | 0.225379 | | 0.200985 | 0.101879 | NA | NA | 0.153382 | 0.220083 |
| 0.170967 | | 0.163251 | | 0.16194 | 0.108685 | NA | NA | NA | 0.154374 |

| 0.099834 | 0.099568 | 0.10149 | 0.094415 | NA | NA | 0.264614 | 0.123038 |
| --- | --- | --- | --- | --- | --- | --- | --- |
| 0.102046 | 0.102694 | 0.100785 | 0.095787 | NA | NA | 0.23279 | 0.121117 |
| 0.106351 | 0.104742 | 0.10576 | 0.097251 | NA | NA | 0.243333 | 0.125178 |
| NA | NA | 0.095851 | NA | NA | NA | 0.249538 | 0.113243 |
| NA | NA | NA | 0.129054 | 0.12875 | NA | NA | NA |
| NA | NA | NA | 0.118586 | 0.119226 | NA | NA | NA |
| NA | NA | NA | 0.120634 | 0.122347 | NA | NA | NA |
| NA | NA | NA | 0.112761 | NA | NA | NA | NA |
| NA | NA | NA | 0.120346 | 0.124938 | NA | NA | NA |
| NA | NA | NA | 0.117626 | NA | NA | NA | NA |
| NA | NA | NA | 0.108649 | NA | NA | NA | NA |
| NA | NA | NA | 0.108649 | NA | NA | NA | NA |
| NA | NA | NA | 0.108649 | NA | NA | NA | NA |
| NA | NA | NA | 0.118834 | NA | NA | NA | NA |
| NA | NA | NA | 0.118071 | NA | NA | NA | NA |
| 0.094185 | 0.093062 | 0.09415 | 0.121703 | 0.131131 | NA | NA | 0.090405 |
| NA | NA | NA | 0.108695 | NA | NA | NA | NA |
| NA | NA | NA | 0.111471 | NA | NA | NA | NA |
| 0.098663 | 0.097077 | 0.096946 | 0.113854 | 0.117623 | NA | NA | 0.093723 |
| NA | 0.092421 | 0.092445 | 0.118918 | 0.134457 | NA | NA | 0.08971 |
| NA | NA | NA | 0.097949 | NA | NA | NA | NA |
| 0.228118 | 0.207023 | 0.186978 | 0.103759 | NA | NA | 0.164188 | 0.201835 |
| 0.10666 | 0.115846 | 0.102409 | 0.105701 | 0.21197 | NA | 0.19837 | 0.098628 |
| 0.10666 | 0.115846 | 0.102409 | 0.105701 | 0.21197 | NA | 0.19837 | 0.098628 |
| NA | 0.098326 | NA | 0.096139 | 0.199501 | NA | 0.143116 | NA |
| NA | 0.098326 | NA | 0.096139 | 0.199501 | NA | 0.143116 | NA |
| 0.096251 | 0.108411 | 0.094221 | 0.100953 | 0.20392 | NA | 0.154883 | 0.090364 |
| 0.108436 | 0.121888 | 0.101861 | 0.108659 | 0.286305 | NA | 0.221074 | 0.098467 |
| NA | 0.098451 | NA | 0.099878 | 0.213217 | NA | 0.156987 | NA |
| NA | 0.102015 | NA | 0.097744 | 0.194443 | NA | 0.144038 | NA |
| NA | 0.102015 | NA | 0.097744 | 0.194443 | NA | 0.144038 | NA |
| NA | 0.102015 | NA | 0.097744 | 0.194443 | NA | 0.144038 | NA |
| NA | NA | NA | NA | NA | NA | NA | NA |
| NA | NA | NA | NA | NA | NA | NA | NA |
| NA | NA | NA | NA | NA | NA | NA | NA |
| NA | NA | NA | NA | NA | NA | NA | NA |
| 0.098973 | 0.099575 | 0.102146 | 0.10186 | NA | NA | NA | 0.097158 |
| 0.102742 | 0.103118 | 0.107248 | 0.102251 | NA | 0.126174 | NA | 0.097867 |
| 0.102742 | 0.103118 | 0.107248 | 0.102251 | NA | 0.126174 | NA | 0.097867 |

| 0.107875 | | 0.105397 | 0.110582 | 0.108753 | NA | NA | NA | 0.102501 |
| --- | --- | --- | --- | --- | --- | --- | --- | --- |
| NA |  | NA | NA | 0.104568 | NA | NA | NA | NA |
|  | 0.1154 | 0.110839 | 0.114931 | 0.106793 | NA | NA | NA | 0.109898 |
| NA |  | NA | 0.097632 | 0.097611 | NA | 0.122931 | NA | NA |
| 0.114601 | | 0.112792 | 0.112873 | 0.10116 | NA | 0.126026 | NA | 0.112908 |
| NA |  | NA | NA | 0.108674 | NA | NA | NA | 0.118202 |
| NA |  | NA | NA | 0.101202 | NA | NA | NA | NA |
| NA |  | NA | NA | 0.10314 | NA | NA | NA | NA |
| NA |  | NA | 0.094338 | 0.144189 | NA | 0.120648 | NA | 0.089837 |
| NA |  | NA | 0.094295 | 0.146461 | NA | 0.132371 | NA | 0.090253 |
| 0.094857 | | 0.094986 | 0.097392 | 0.13831 | NA | 0.142145 | NA | 0.093665 |
| NA |  | NA | NA | NA | NA | NA | NA | NA |
| NA |  | NA | NA | NA | NA | NA | NA | NA |
| NA |  | NA | NA | NA | NA | NA | NA | NA |
| NA |  | NA | NA | NA | NA | NA | NA | NA |
| NA |  | NA | NA | NA | NA | NA | NA | NA |
| NA |  | NA | NA | NA | NA | NA | NA | NA |
| NA |  | NA | NA | NA | NA | NA | NA | NA |
| NA |  | NA | NA | NA | NA | NA | NA | NA |
| 0.100988 | | 0.098236 | 0.094545 | NA | NA | NA | NA | 0.09211 |
| NA |  | NA | NA | NA | NA | NA | NA | NA |
| NA |  | NA | NA | NA | NA | NA | NA | NA |
| NA |  | NA | NA | NA | NA | NA | NA | NA |
| NA |  | NA | NA | NA | NA | NA | NA | NA |
| NA |  | NA | NA | NA | NA | NA | NA | NA |
| 0.098227 | | 0.096451 | 0.094837 | NA | NA | NA | NA | 0.092755 |
| NA |  | NA | NA | NA | NA | NA | NA | NA |
| NA |  | NA | NA | NA | NA | NA | NA | NA |
| NA |  | NA | NA | NA | NA | NA | NA | NA |
| NA |  | NA | NA | NA | NA | NA | NA | NA |
| 0.095707 | | 0.097421 | 0.094908 | 0.095137 | NA | 0.110434 | NA | 0.09576 |
| NA |  | NA | NA | NA | NA | NA | NA | NA |
| NA |  | NA | NA | NA | NA | NA | NA | NA |
| NA |  | NA | NA | NA | NA | NA | NA | NA |
| NA |  | NA | NA | NA | NA | NA | NA | NA |
| NA |  | NA | NA | NA | NA | NA | NA | NA |
| NA |  | NA | NA | NA | NA | NA | NA | NA |
| NA |  | NA | NA | NA | NA | NA | NA | NA |
| NA |  | NA | NA | NA | NA | NA | NA | NA |

| NA | NA | NA | NA | NA | NA | NA | NA |
| --- | --- | --- | --- | --- | --- | --- | --- |
| 0.102933 | 0.10134 | 0.096934 | NA | NA | NA | NA | 0.094167 |
| NA | NA | NA | NA | NA | NA | NA | NA |
| NA | NA | NA | NA | NA | NA | NA | NA |
| NA | NA | NA | NA | NA | NA | NA | NA |
| 0.097392 | NA | NA | NA | NA | NA | NA | 0.095747 |
| NA | NA | NA | NA | NA | NA | NA | NA |
| NA | NA | NA | NA | NA | NA | NA | NA |
| NA | NA | NA | NA | NA | NA | NA | NA |
| NA | NA | NA | NA | NA | NA | NA | NA |
| 0.147869 | 0.139696 | 0.126557 | 0.092452 | NA | NA | NA | 0.144191 |
| NA | 0.094216 | 0.115196 | 0.11113 | NA | NA | NA | 0.101662 |
| NA | 0.095188 | 0.095136 | NA | NA | NA | NA | 0.106304 |
| 0.119193 | 0.11644 | 0.112131 | 0.098441 | NA | NA | NA | 0.130256 |
| 0.133683 | 0.128879 | 0.123864 | 0.126354 | NA | NA | NA | 0.163856 |
| 0.104442 | 0.103354 | 0.104202 | 0.103335 | NA | NA | NA | 0.114744 |
| NA | NA | NA | 0.098881 | NA | NA | NA | 0.098342 |
| 0.09653 | 0.095082 | 0.096728 | 0.098972 | NA | NA | NA | 0.108387 |
| 0.116633 | 0.113563 | 0.110069 | 0.104392 | NA | NA | NA | 0.13523 |
| 0.103839 | 0.10123 | 0.10207 | 0.106467 | NA | NA | NA | 0.122347 |
| 0.098365 | NA | 0.099166 | NA | NA | NA | 0.267817 | 0.120995 |
| 0.095772 | 0.096364 | 0.098401 | NA | NA | NA | 0.24683 | 0.117698 |
| 0.100177 | 0.099468 | 0.100618 | 0.095699 | NA | NA | 0.23133 | 0.123495 |
| NA | NA | NA | NA | NA | NA | 0.241966 | 0.110967 |
| 0.098968 | 0.098245 | 0.100708 | NA | NA | NA | NA | 0.098432 |
| 0.100313 | 0.101544 | 0.101544 | 0.095518 | NA | 0.129699 | NA | 0.101261 |
| 0.099205 | 0.098417 | 0.10095 | NA | NA | NA | NA | 0.09916 |
| 0.104763 | 0.10702 | 0.103675 | 0.096957 | NA | NA | NA | 0.10394 |
| 0.104763 | 0.10702 | 0.103675 | 0.096957 | NA | NA | NA | 0.10394 |
| 0.100254 | 0.099484 | 0.098928 | NA | NA | NA | NA | 0.096094 |
| 0.109928 | 0.107312 | 0.104955 | NA | NA | 0.112663 | NA | 0.102138 |
| 0.098955 | 0.099917 | 0.100668 | NA | NA | NA | NA | 0.097762 |
| 0.098955 | 0.099917 | 0.100668 | NA | NA | NA | NA | 0.097762 |
| 0.098955 | 0.099917 | 0.100668 | NA | NA | NA | NA | 0.097762 |
| 0.110731 | 0.109026 | 0.106681 | NA | NA | 0.11098 | NA | 0.104424 |
| 0.119164 | 0.115084 | 0.110617 | NA | NA | 0.141823 | NA | 0.106372 |
| NA | NA | 0.097282 | NA | NA | NA | NA | NA |
| NA | NA | NA | NA | NA | NA | NA | NA |
| 0.093951 | NA | 0.095416 | 0.106196 | NA | 0.139023 | 0.140713 | 0.094811 |

| NA | NA | NA | NA | NA | NA |  | NA | NA |
| --- | --- | --- | --- | --- | --- | --- | --- | --- |
| NA | NA | NA | NA | NA | NA |  | NA | NA |
| NA | NA | NA | NA | NA | NA |  | NA | NA |
| NA | NA | NA | NA | NA | NA |  | NA | NA |
| 0.114795 | 0.108454 | 0.109137 | 0.100461 | NA | 0.15163 | | NA | 0.107515 |
| 0.111769 | 0.111484 | 0.107694 | 0.107599 | 0.130819 | 0.157484 | | NA | 0.110105 |
| 0.103843 | 0.103843 | 0.10363 | 0.101538 | NA | 0.126026 | | NA | 0.099968 |
| 0.118386 | 0.115272 | 0.112784 | 0.105027 | NA |  | 0.13 | NA | 0.114556 |
| 0.098384 | 0.099829 | 0.101351 | 0.09537 | NA | 0.12453 | | NA | 0.101629 |
| 0.10333 | 0.102607 | 0.102956 | 0.099095 | NA | NA |  | NA | 0.10561 |
| 0.109785 | 0.109298 | 0.113457 | 0.11101 | NA | NA |  | NA | 0.110779 |
| 0.123825 | 0.117717 | 0.117644 | 0.101158 | NA | 0.147833 | | NA | 0.110018 |
| 0.129673 | 0.126441 | 0.125522 | 0.107409 | NA | 0.151057 | | 0.163943 | 0.118371 |
| 0.09952 | 0.098976 | 0.100321 | 0.102093 | NA | 0.116098 | | NA | 0.098648 |
| NA | NA | NA | NA | NA | NA |  | NA | NA |
| NA | NA | NA | NA | NA | NA |  | NA | NA |
| 0.104261 | 0.104222 | 0.109216 | 0.114546 | NA | NA |  | NA | 0.10519 |
| NA | NA | NA | NA | NA | NA |  | NA | NA |
| 0.114009 | 0.107966 | 0.10794 | 0.104823 | 0.149146 | 0.141566 | | NA | 0.106168 |
| NA | NA | NA | NA | NA | NA |  | NA | NA |
| NA | NA | NA | NA | NA | NA |  | NA | NA |
| 0.116115 | 0.110629 | 0.11193 | 0.111018 | NA | NA |  | NA | 0.110271 |
| 0.113379 | 0.111816 | 0.117505 | 0.114285 | NA | NA |  | NA | 0.114196 |
| 0.113379 | 0.111816 | 0.117505 | 0.114285 | NA | NA |  | NA | 0.114196 |
| 0.113379 | 0.111816 | 0.117505 | 0.114285 | NA | NA |  | NA | 0.114196 |
| 0.113379 | 0.111816 | 0.117505 | 0.114285 | NA | NA |  | NA | 0.114196 |
| 0.12207 | 0.119891 | 0.112964 | 0.104206 | NA | NA |  | NA | 0.117646 |
| 0.12207 | 0.119891 | 0.112964 | 0.104206 | NA | NA |  | NA | 0.117646 |
| 0.12207 | 0.119891 | 0.112964 | 0.104206 | NA | NA |  | NA | 0.117646 |
| 0.12207 | 0.119891 | 0.112964 | 0.104206 | NA | NA |  | NA | 0.117646 |
| 0.115541 | 0.114704 | 0.114332 | 0.121341 | NA | NA |  | NA | 0.115824 |
| 0.115541 | 0.114704 | 0.114332 | 0.121341 | NA | NA |  | NA | 0.115824 |
| 0.113184 | 0.110183 | 0.109373 | 0.110419 | NA | NA |  | NA | 0.113307 |
| 0.113184 | 0.110183 | 0.109373 | 0.110419 | NA | NA |  | NA | 0.113307 |
| 0.119847 | 0.118784 | 0.121977 | 0.120173 | NA | NA |  | NA | 0.118711 |
| 0.117461 | 0.109292 | 0.109559 | 0.102523 | NA | 0.158618 | | NA | 0.106674 |
| 0.117461 | 0.109292 | 0.109559 | 0.102523 | NA | 0.158618 | | NA | 0.106674 |
| 0.115415 | 0.109751 | 0.108923 | 0.104372 | NA | 0.129697 | | NA | 0.110009 |
| 0.115415 | 0.109751 | 0.108923 | 0.104372 | NA | 0.129697 | | NA | 0.110009 |

| 0.116351 | 0.107768 | 0.109103 | 0.102262 | | NA | 0.140567 | NA | 0.104758 |
| --- | --- | --- | --- | --- | --- | --- | --- | --- |
| 0.122023 | 0.114113 | 0.11333 | 0.105778 | | NA | 0.157042 | NA | 0.110773 |
| 0.11398 | 0.107135 | 0.109153 | 0.104882 | | NA | 0.125808 | NA | 0.105271 |
| 0.101074 | 0.100182 | 0.100958 | 0.095099 | | NA | NA | NA | 0.101581 |
| 0.108057 | 0.111231 | 0.107439 | 0.099329 | | NA | NA | 0.178044 | 0.122734 |
| NA | NA | 0.098174 | NA |  | NA | NA | 0.270513 | 0.118418 |
| 0.109034 | 0.11081 | 0.108391 | 0.099463 | | NA | NA | 0.154545 | 0.120525 |
| 0.107571 | 0.107057 | 0.119844 | 0.124279 | | NA | NA | NA | 0.108403 |
| 0.109683 | 0.10895 | 0.122104 | 0.126659 | | NA | NA | NA | 0.110116 |
| NA | NA | 0.096743 |  | 0.1079 | NA | NA | NA | NA |
| 0.108906 | 0.109001 | 0.122402 | 0.127733 | | NA | NA | NA | 0.110373 |
| 0.102894 | 0.102319 | 0.108657 | 0.120614 | | NA | NA | NA | 0.104498 |
| NA | NA | 0.095327 | NA |  | NA | NA | NA | NA |
| 0.110036 | 0.106875 | 0.106885 | NA |  | NA | 0.127305 | NA | 0.105983 |
| NA | NA | NA | NA |  | NA | NA | NA | NA |
| NA | NA | NA | NA |  | NA | NA | NA | NA |
| NA | NA | NA | NA |  | NA | NA | NA | NA |
| 0.095032 | 0.095671 | 0.095878 | NA |  | NA | NA | NA | 0.096069 |
| NA | NA | NA | NA |  | NA | NA | NA | NA |
| NA | NA | NA | NA |  | NA | NA | NA | NA |
| NA | NA | NA | NA |  | NA | NA | NA | NA |
| NA | NA | NA | NA |  | NA | NA | NA | NA |
| NA | NA | NA | NA |  | NA | NA | NA | NA |
| 0.098071 | 0.096163 | 0.095722 | NA |  | NA | NA | NA | 0.099221 |
| 0.109928 | 0.106668 | 0.102491 | NA |  | NA | NA | NA | 0.108055 |
| NA | NA | NA | NA |  | NA | NA | NA | NA |
| NA | NA | NA | NA |  | NA | NA | NA | NA |
| NA | NA | NA | NA |  | NA | NA | NA | NA |
| 0.118356 | 0.117239 | 0.121253 | 0.120361 | | NA | NA | NA | 0.117471 |
| 0.12074 | 0.118011 | 0.113004 | 0.097524 | | NA | 0.125122 | NA | 0.111194 |
| 0.12074 | 0.118011 | 0.113004 | 0.097524 | | NA | 0.125122 | NA | 0.111194 |
| NA | NA | NA | 0.190567 | | NA | 0.198347 | NA | NA |
| NA | NA | 0.094126 | 0.111394 | | NA | 0.126179 | NA | NA |
| NA | NA | NA | 0.118543 | | NA | 0.131485 | NA | NA |
| 0.095087 | 0.095504 | 0.097774 | 0.125107 | | NA | 0.13406 | NA | 0.092998 |
| 0.13983 | 0.132559 | 0.129746 | 0.097632 | | NA | NA | NA | 0.127711 |
| NA | NA | 0.094441 | 0.135742 | | NA | 0.127515 | NA | 0.090268 |
| NA | NA | NA | 0.110224 | | NA | NA | NA | NA |
| NA | NA | 0.095189 | 0.12965 | | NA | 0.129836 | 0.138768 | 0.092243 |

| 0.093834 | 0.094171 | | 0.095032 | 0.127311 | NA | 0.133666 | NA | 0.093358 | |
| --- | --- | --- | --- | --- | --- | --- | --- | --- | --- |
| NA | NA |  | NA | 0.113769 | NA | NA | NA | NA |  |
| NA | NA |  | NA | 0.117231 | NA | NA | NA | NA |  |
| NA | NA |  | NA | 0.119968 | NA | NA | NA | NA |  |
| NA | NA |  | NA | 0.110715 | NA | NA | NA | NA |  |
| 0.11214 | 0.112367 | | 0.106861 | 0.108283 | NA | 0.156018 | 0.134662 | 0.11143 | |
| NA | NA |  | NA | 0.110813 | NA | NA | NA | NA |  |
| NA | NA |  | NA | 0.110813 | NA | NA | NA | NA |  |
| NA | NA |  | NA | 0.110813 | NA | NA | NA | NA |  |
| NA | NA |  | NA | NA | NA | NA | NA | NA |  |
| 0.106323 | 0.106964 | | 0.102711 | 0.101584 | NA | 0.139738 | NA | 0.106393 | |
| NA | 0.095216 | | 0.096231 | 0.100226 | NA | 0.123312 | NA | 0.097992 | |
| 0.107107 | 0.107703 | | 0.106869 | 0.10573 | NA | 0.122607 | 0.127083 | 0.113468 | |
| NA | NA |  | NA | NA | NA | NA | NA | NA |  |
| NA | NA |  | 0.096572 | 0.09501 | NA | NA | NA | NA |  |
| 0.096267 | 0.097379 | | 0.097603 | 0.097478 | NA | 0.122449 | NA | 0.100742 | |
| 0.096927 | 0.098191 | | 0.098841 | 0.100369 | NA | 0.119578 | 0.143116 | 0.099647 | |
| NA | NA |  | 0.09722 | 0.095276 | NA | 0.117821 | NA | NA |  |
| NA | NA |  | NA | NA | NA | NA | NA | NA |  |
| 0.108903 | 0.107416 | | 0.103793 | NA | NA | 0.14046 | NA | 0.097017 | |
| 0.115939 | 0.11314 | | 0.109143 | 0.094236 | NA | 0.145768 | NA | 0.10376 | |
| 0.099385 | 0.099783 | | 0.101906 | 0.117166 | NA | 0.118699 | NA | 0.09671 | |
| 0.098413 | 0.098334 | | 0.098414 | 0.12821 | NA | 0.138921 | NA | 0.096547 | |
| 0.12576 |  | 0.1231 | 0.121019 | 0.098381 | NA | NA | NA | 0.122675 | |
| 0.10933 | 0.105896 | | 0.107654 | 0.102222 | NA | 0.123202 | NA | 0.105762 | |
| 0.096247 | 0.094685 | | 0.096602 | 0.108769 | NA | NA | NA | 0.094433 | |
| 0.110098 | 0.108461 | | 0.108244 | 0.092625 | NA | 0.124701 | NA | 0.107877 | |
| 0.100325 | 0.100644 | | 0.100894 | 0.095544 | NA | 0.135777 | NA | 0.098733 | |
| 0.110278 | 0.108168 | | 0.108392 | 0.103422 | NA | NA | NA | 0.111851 | |
| 0.097693 | 0.097968 | | 0.099827 | 0.096763 | NA | 0.133373 | NA | 0.096287 | |
| 0.116928 | 0.114461 | | 0.114583 | 0.112661 | NA | 0.160772 | NA |  | 0.1152 |
| 0.102056 | 0.102607 | | 0.103493 | 0.100384 | NA | 0.127119 | NA | 0.102916 | |
| 0.117748 | 0.112912 | | 0.11311 | 0.10954 | NA | NA | NA | 0.113897 | |
| 0.106069 | 0.104894 | | 0.105103 | 0.097872 | NA | 0.128085 | NA | 0.102191 | |
| 0.104394 | 0.105437 | | 0.104128 | 0.102434 | NA | NA | NA | 0.106773 | |
| NA | 0.097902 | | 0.097442 | 0.095952 | NA | 0.130199 | NA | 0.098394 | |
| NA | NA |  | NA | NA | NA | 0.130275 | NA | NA |  |
| NA | NA |  | NA | NA | NA | 0.130275 | NA | NA |  |
| NA | NA |  | NA | NA | NA | 0.130275 | NA | NA |  |

| 0.135949 | 0.132839 | 0.118453 | NA | NA | NA | NA |  | 0.136257 | |
| --- | --- | --- | --- | --- | --- | --- | --- | --- | --- |
| 0.143377 | 0.139019 | 0.123253 | NA | NA | NA | NA |  | 0.142471 | |
| 0.130963 | 0.126027 | 0.114682 | NA | NA | NA | NA |  | 0.127057 | |
| 0.095749 | 0.096165 | 0.098013 | 0.129468 | NA | 0.134513 | NA |  | 0.094938 | |
| NA | NA | NA | 0.121554 | NA | 0.13687 | NA |  | NA |  |
| NA | NA | NA | 0.165199 | NA | 0.178649 | NA |  | NA |  |
| NA | NA | NA | 0.156773 | NA | 0.178512 | NA |  | NA |  |
| 0.096776 | 0.096265 | 0.095555 | 0.100269 | NA | NA | NA |  | 0.098746 | |
| 0.107065 | 0.105866 | 0.10529 | 0.099246 | NA | 0.147801 | NA |  | 0.101255 | |
| 0.105375 | 0.104909 | 0.104488 | 0.103687 | 0.139651 | 0.131887 | 0.139493 | | 0.09981 | |
| 0.104254 | 0.103292 | 0.104081 | 0.103839 | NA | 0.116305 | 0.150837 | | 0.10146 | |
| 0.104254 | 0.103292 | 0.104081 | 0.103839 | NA | 0.116305 | 0.150837 | | 0.10146 | |
| 0.106953 | 0.105861 | 0.105146 | 0.104949 | NA | 0.120066 | 0.158083 | | 0.10371 | |
| NA | NA | 0.097547 | NA | NA | NA | 0.231281 | | 0.113486 | |
| NA | NA | 0.097547 | NA | NA | NA | 0.231281 | | 0.113486 | |
| NA | NA | 0.092863 | 0.098629 | NA | 0.190537 | NA |  | 0.093055 | |
| NA | NA | 0.093362 | 0.095526 | NA | 0.23095 | NA |  | 0.091356 | |
| NA | NA | 0.095711 | 0.097105 | 0.142976 | 0.22714 | NA |  | 0.091457 | |
| 0.106496 | 0.10526 | 0.103525 | NA | NA | 0.111632 | NA |  | 0.099882 | |
| 0.173085 | 0.161827 | 0.151564 | 0.103138 | NA | NA |  | 0.1587 | 0.158297 | |
| 0.23363 | 0.20992 | 0.172231 | 0.09554 | NA | NA | NA |  | 0.234209 | |
| 0.156571 | 0.150323 | 0.130214 | NA | NA | NA | NA |  | 0.160998 | |
| 0.177757 | 0.166655 | 0.146219 | 0.094812 | 0.137303 | NA | 0.149758 | |  | 0.1708 |
| 0.21985 | 0.200008 | 0.164074 | 0.09549 | NA | NA | NA |  | 0.224383 | |
| 0.197413 | 0.179749 | 0.154323 | 0.095485 | NA | NA | NA |  | 0.195058 | |
| 0.107011 | 0.106746 | 0.106331 | 0.098161 | NA | NA | 0.146739 | | 0.110246 | |
| 0.116993 | 0.113237 | 0.108931 | 0.097248 | NA | NA | NA |  | 0.133843 | |
| 0.110224 | 0.109425 | 0.109184 | 0.100922 | NA | NA | NA |  | 0.109035 | |
| 0.101126 | 0.098704 | 0.100471 | 0.101534 | NA | NA | NA |  | 0.098398 | |
| 0.119357 | 0.112023 | 0.111576 | 0.103102 | NA | 0.167644 | NA |  | 0.107319 | |
| 0.122949 | 0.115992 | 0.112364 | 0.106774 | NA | 0.136285 | NA |  | 0.116238 | |
| 0.094201 | NA | 0.095451 | 0.094854 | NA | NA | NA |  | NA |  |
| NA | NA | NA | 0.106151 | NA | 0.13725 | NA |  | NA |  |
| NA | NA | NA | 0.096477 | NA | NA | 0.142468 | | NA |  |
| NA | NA | NA | 0.093311 | NA | NA | 0.151737 | | NA |  |
| 0.102572 | 0.101932 | 0.101375 | NA | NA | 0.141272 | NA |  | NA |  |
| 0.11192 | 0.110086 | 0.108917 | 0.099541 | NA | 0.156909 | NA |  | 0.102428 | |
| 0.111386 | 0.10815 | 0.106596 | 0.094162 | NA | 0.137628 | NA |  | 0.099528 | |
| NA | NA | NA | NA | NA | NA | NA |  | NA |  |

| 0.112078 | 0.109142 | 0.107603 | | 0.097305 | NA | 0.151256 | NA | 0.100803 |
| --- | --- | --- | --- | --- | --- | --- | --- | --- |
| 0.121371 | 0.11452 | 0.112817 | | 0.10518 | NA | 0.166272 | NA | 0.110998 |
| 0.105765 | 0.106133 |  | 0.1096 | 0.111558 | NA | NA | NA | 0.109656 |
| 0.104664 | 0.104013 | 0.108208 | | 0.109194 | NA | NA | NA | 0.109193 |
| 0.099759 | 0.100149 | 0.103671 | | 0.104469 | NA | NA | NA | 0.104307 |
| 0.106193 | 0.103348 | 0.103646 | | 0.105716 | NA | NA | NA | 0.104321 |
| NA | NA | 0.100712 | | 0.106036 | NA | NA | NA | NA |
| 0.104723 | 0.10469 | 0.108698 | | 0.109612 | NA | NA | NA | 0.108984 |
| 0.099453 | 0.099973 | 0.105469 | | 0.106151 | NA | NA | NA | 0.103115 |
| NA | NA | NA |  | NA | NA | NA | NA | NA |
| NA | NA | NA |  | NA | NA | NA | NA | NA |
| NA | NA | NA |  | NA | NA | NA | NA | NA |
| 0.131455 | 0.128026 | 0.122482 | | 0.098037 | NA | 0.131564 | NA | 0.122067 |
| 0.114642 | 0.112053 | 0.113241 | | 0.10337 | NA | 0.129641 | NA | 0.108236 |
| 0.096773 | 0.09361 | 0.096163 | | 0.094242 | NA | NA | NA | NA |
| 0.110302 | 0.111006 | 0.11221 | | 0.100147 | NA | 0.137994 | NA | 0.102886 |
| 0.096794 | NA | 0.095479 | | NA | NA | NA | NA | 0.093356 |
| 0.096883 | 0.096355 | 0.096636 | | NA | NA | NA | NA | NA |
| 0.103877 | 0.102527 | 0.101437 | | 0.096797 | NA | 0.12751 | NA | 0.096656 |
| NA | NA | NA |  | NA | NA | NA | NA | NA |
| 0.121126 | 0.120093 | 0.11825 | | 0.112776 | NA | 0.152468 | NA | 0.115835 |
| 0.097004 | 0.096969 | 0.10078 | | 0.102367 | NA | 0.121994 | NA | 0.096893 |
| NA | NA | NA |  | NA | NA | 0.127672 | NA | NA |
| NA | NA | NA |  | NA | NA | 0.128895 | NA | NA |
| 0.109331 | 0.106717 | 0.105638 | | NA | NA | 0.198537 | NA | 0.100395 |
| 0.115234 | 0.111334 | 0.107221 | | NA | NA | 0.12889 | NA | 0.105046 |
| 0.113548 | 0.111539 | 0.107754 | | 0.094752 | 0.134851 | 0.202148 | NA | 0.105001 |
| 0.109991 | 0.105969 | 0.107459 | | 0.094301 | 0.125935 | 0.17458 | NA | 0.100141 |
| 0.106903 | 0.105939 | 0.103998 | | NA | NA | NA | NA | 0.10137 |
| 0.115547 | 0.111496 | 0.111355 | | 0.104048 | NA | NA | NA | 0.110465 |
| 0.121613 | 0.119591 | 0.116099 | | 0.105233 | NA | 0.112962 | NA | 0.113354 |
| 0.120741 | 0.117879 | 0.11663 | | 0.109343 | NA | 0.132653 | NA | 0.115256 |
| 0.107664 | 0.106965 | 0.105488 | | 0.100052 | NA | 0.12544 | NA | 0.10566 |
| 0.108378 | 0.108718 | 0.108817 | | 0.103053 | NA | 0.121613 | NA | 0.107291 |
| 0.103703 | 0.102625 | 0.102006 | | NA | NA | 0.122564 | NA | 0.099677 |
| 0.118593 | 0.116201 | 0.113126 | | 0.095786 | NA | NA | NA | 0.114918 |
| 0.13063 | 0.126769 | 0.123138 | | 0.095247 | NA | NA | NA | 0.129425 |
| 0.113212 | 0.109878 | 0.10784 | | 0.104368 | NA | NA | NA | 0.10629 |
| 0.114079 | 0.109148 | 0.111408 | | 0.099497 | NA | 0.134665 | NA | 0.109408 |

| 0.116575 | 0.115793 | 0.114439 | 0.101159 | | NA | NA | NA | 0.111663 |
| --- | --- | --- | --- | --- | --- | --- | --- | --- |
| 0.119359 | 0.117374 | 0.11233 | 0.101742 | | NA | 0.130799 | 0.125253 | 0.111752 |
| 0.113305 | 0.113421 | 0.112543 | 0.124173 | | NA | 0.147756 | NA | 0.11385 |
| 0.107728 | 0.106911 | 0.104599 | 0.097278 | | NA | 0.120248 | NA | 0.10149 |
| 0.131062 | 0.127413 | 0.127103 | 0.117909 | | NA | NA | NA | 0.136364 |
| NA | NA | 0.101267 | 0.109635 | | NA | 0.124688 | NA | NA |
| 0.118161 | 0.11422 | 0.110264 | 0.10265 | | NA | 0.134829 | NA | 0.111258 |
| 0.104163 | 0.102582 | 0.101563 | 0.097693 | | NA | 0.130377 | NA | 0.099114 |
| 0.111311 | 0.110238 | 0.10942 | 0.104378 | | NA | 0.119865 | NA | 0.104607 |
| 0.109626 | 0.108925 | 0.10641 | 0.098871 | | NA | 0.125298 | NA | 0.106618 |
| 0.110845 | 0.109256 | 0.108953 |  | 0.0984 | NA | 0.116595 | NA | 0.105664 |
| NA | NA | NA | NA |  | NA | NA | NA | NA |
| 0.111808 | 0.109686 | 0.10797 | NA |  | NA | 0.135781 | NA | 0.107287 |
| 0.108933 | 0.10671 | 0.104895 | NA |  | NA | NA | NA | 0.098762 |
| 0.112971 | 0.111956 | 0.109808 | 0.093033 | | NA | NA | NA | 0.103882 |
| 0.124749 | 0.119899 | 0.115694 | 0.10486 | | NA | 0.150334 | NA | 0.113894 |
| 0.098879 | 0.097898 | 0.098656 | 0.095885 | | NA | 0.135013 | NA | NA |
| 0.111513 | 0.107787 | 0.107592 | 0.098202 | | NA | 0.141147 | NA | 0.101522 |
| NA | NA | 0.096466 | 0.091588 | | NA | 0.132481 | NA | NA |
| 0.107578 | 0.107197 | 0.105358 | 0.096692 | | NA | 0.12276 | NA | 0.105447 |
| 0.11576 | 0.115255 | 0.110979 | 0.100361 | | NA | 0.122135 | NA | 0.111765 |
| 0.110043 | 0.11207 | 0.109464 | NA |  | NA | NA | NA | 0.11353 |
| 0.10601 | 0.106245 | 0.105181 | 0.098718 | | NA | 0.127979 | NA | 0.101216 |
| 0.126783 | 0.121293 | 0.113749 | NA |  | NA | NA | NA | 0.116275 |
| 0.10818 | 0.106941 | 0.106968 | 0.101037 | | NA | 0.119083 | NA | 0.100047 |
| 0.118647 | 0.116009 | 0.111728 | 0.100964 | | NA | NA | NA | 0.117907 |
| 0.109111 | 0.107465 | 0.106373 | 0.105852 | | NA | 0.129027 | NA | 0.108594 |
| 0.114498 | 0.11079 | 0.108065 | NA |  | NA | 0.115914 | NA | 0.103301 |
| 0.101855 | 0.102308 | 0.10149 | 0.102572 | | NA | 0.127159 | NA | 0.100592 |
| 0.100516 | 0.101684 | 0.099313 | 0.100488 | | NA | 0.123758 | NA | 0.102452 |
| 0.121621 | 0.118366 | 0.112104 | 0.093181 | | NA | NA | NA | 0.118769 |
| 0.119644 | 0.115581 | 0.115659 | 0.101643 | | NA | 0.137749 | NA | 0.108924 |
| 0.108848 | 0.107267 | 0.106608 | 0.096664 | | NA | 0.127198 | 0.129529 | 0.101716 |
| 0.110196 | 0.10703 | 0.103066 | 0.095812 | | NA | NA | NA | 0.108535 |
| NA | NA | 0.096462 | NA |  | NA | 0.120438 | NA | NA |
| 0.102507 | 0.101931 | 0.101292 | 0.101523 | | NA | 0.131594 | NA | 0.101534 |
| 0.11081 | 0.112479 | 0.110032 | 0.100941 | | NA | 0.131218 | NA | 0.110051 |
| 0.116674 | 0.115986 | 0.110715 | 0.094944 | | NA | 0.129552 | NA | 0.109922 |

immature_ immature_ immature_ immature_ inflammat ips..ceehrcips..knih	islet_cell..

0.116791 0.116427 0.116406 0.116789 0.119224 0.138577 0.150304 0.099157

| NA | NA | NA | NA | NA | NA | NA | 0.095947 |
| --- | --- | --- | --- | --- | --- | --- | --- |

0.112679 0.112928 0.113175	0.11368 0.118642 NA	NA	0.114258

0.108566 0.109467 0.108937 0.108792 0.127947 0.122384 0.146023 0.098901

0.101397 0.101527 0.100842 0.102333 0.114339 NA	NA	0.105699

0.127635 0.129704 0.129489 0.128626 0.131359 NA	0.115385	0.12615

0.111939 0.112396 0.111286 0.112302 0.123545 NA	NA	0.111876

0.102956	0.10444 0.104425 0.103363 0.109939 NA	0.108838 0.097219

0.102956	0.10444 0.104425 0.103363 0.109939 NA	0.108838 0.097219

NA	NA	NA	NA	0.120432 NA	NA	0.113711

0.118699 0.120719 0.119731 0.120021 0.121285 NA	NA	0.120898

0.109063	0.11069 0.110243	0.11034 0.110507 NA	NA	0.115128

0.106504 0.106883 0.106372 0.105726 0.131771 NA	NA	0.099752

0.107097 0.107503 0.106498 0.105795 0.130201 NA	NA	0.10481

NA	NA	NA	NA	NA	NA	NA	0.105558

NA	NA	NA	NA	NA	NA	NA	0.101528

NA	NA	NA	NA	NA	NA	NA	0.107107

NA	NA	NA	NA	NA	NA	NA	0.107107

0.152681 0.153471 0.152582 0.151466 0.139624 NA	NA	0.093618

0.11406 0.113427 0.113418 0.113238 0.119147 NA	NA	0.115788

| NA | NA | NA | NA | NA | NA | NA | 0.101969 |
| --- | --- | --- | --- | --- | --- | --- | --- |

0.144763 0.146997 0.146934 0.147945 0.134046 NA	NA	0.094213

NA	NA	NA	NA	NA	NA	NA	NA NA	NA	NA	NA	NA	NA	NA	NA

NA	NA	NA	NA	0.104712 NA	NA	0.099929

NA	NA	NA	NA	0.126131 NA	NA	NA NA	NA	NA	NA	0.127209 NA	NA	NA NA	NA	NA	NA	0.118494 NA	NA	NA

0.113666 0.114672 0.114077 0.115104 0.113823 NA	NA	0.116777

0.113666 0.114672 0.114077 0.115104 0.113823 NA	NA	0.116777

0.130868 0.132636 0.130907 0.131515 0.137225	0.13227 0.122449 0.135194

0.134517 0.135387 0.134494 0.134634 0.136117 0.127202 0.109397 0.137842

NA	NA	NA	NA	0.112099 NA	NA	0.109791

NA	NA	NA	NA	0.105782 NA	NA	0.099791

NA	NA	NA	NA	NA	NA	NA	0.09581

NA	NA	NA	NA	NA	NA	NA	0.098498

NA	NA	NA	NA	NA	NA	NA	0.100464

0.11396 0.114392 0.114738 0.114142 0.115869 0.151655 0.177457 NA

| 0.098258 | 0.097064 | 0.097462 | 0.098275 | 0.100648 | NA | 0.113809 | | 0.091138 |
| --- | --- | --- | --- | --- | --- | --- | --- | --- |
| 0.098258 | 0.097064 | 0.097462 | 0.098275 | 0.100648 | NA | 0.113809 | | 0.091138 |
| 0.098258 | 0.097064 | 0.097462 | 0.098275 | 0.100648 | NA | 0.113809 | | 0.091138 |
| 0.110305 | 0.110996 | 0.109652 | 0.110902 | 0.100796 | 0.126073 | 0.149852 | | NA |
| 0.108798 | 0.109358 | 0.108478 | 0.107807 | 0.104222 | 0.135749 | 0.155064 | | NA |
| 0.183866 | 0.183134 | 0.181731 | 0.181302 | 0.157589 | NA | NA |  | 0.096847 |
| 0.194558 | 0.194992 | 0.194092 | 0.193177 | 0.180379 | NA | NA |  | 0.116014 |
| 0.216894 | 0.217275 | 0.215569 | 0.21504 | 0.201293 | NA |  | 0.1149 | 0.114662 |
| 0.201297 | 0.202183 | 0.199892 | 0.199133 | 0.171963 | NA | NA |  | 0.110168 |
| 0.183959 | 0.184832 | 0.184034 | 0.183078 | 0.17421 | NA | NA |  | 0.119006 |
| 0.195166 | 0.196978 | 0.195448 | 0.195454 | 0.187581 | NA | 0.123749 | | 0.114151 |
| 0.209227 | 0.209477 | 0.207297 | 0.207087 | 0.189628 | NA | NA |  | 0.114662 |
| 0.18765 | 0.187838 | 0.187107 | 0.18635 | 0.184092 | 0.111846 | 0.120944 | | 0.117713 |
| 0.194901 | 0.195911 | 0.194344 | 0.19496 | 0.187586 | NA | NA |  | 0.115556 |
| 0.206907 | 0.207813 | 0.207469 | 0.206529 | 0.198143 | NA | 0.119645 | | 0.117849 |
| NA | NA | NA | NA | NA | NA | NA |  | 0.103139 |
| 0.102402 | 0.103163 | 0.102659 | 0.102952 | 0.106862 | NA | NA |  | 0.104171 |
| 0.210952 | 0.21181 | 0.209732 | 0.208842 | 0.194816 | NA | NA |  | 0.116412 |
| 0.21799 | 0.219927 | 0.218261 | 0.217714 | 0.203118 | NA | 0.104851 | | 0.116549 |
| 0.157577 | 0.160268 | 0.160018 | 0.15828 | 0.162128 | NA | 0.130031 | | 0.113027 |
| 0.234867 | 0.234858 | 0.232978 | 0.231693 | 0.206103 | NA | NA |  | 0.110257 |
| NA | NA | NA | NA | NA | NA | NA |  | 0.123545 |
| NA | NA | NA | NA | NA | NA | NA |  | 0.102177 |
| 0.222519 | 0.223866 | 0.222164 | 0.221745 | 0.20466 | NA | NA |  | 0.113608 |
| 0.156759 | 0.158793 | 0.158521 | 0.155612 | 0.153529 | NA | NA |  | 0.108103 |
| 0.182391 | 0.182811 | 0.181727 | 0.180434 | 0.168296 | NA | NA |  | 0.101741 |
| 0.220497 | 0.22202 | 0.220127 | 0.219488 | 0.205144 | NA | NA |  | 0.115866 |
| 0.205851 | 0.206056 | 0.204627 | 0.205374 | 0.187957 | NA | NA |  | 0.114328 |
| 0.160101 | 0.161345 | 0.159471 | 0.160651 | 0.157528 | NA | NA |  | 0.113212 |
| 0.160101 | 0.161345 | 0.159471 | 0.160651 | 0.157528 | NA | NA |  | 0.113212 |
| 0.227691 | 0.229157 | 0.226666 | 0.226684 | 0.206923 | NA | NA |  | 0.112949 |
| 0.178111 | 0.179026 | 0.177462 | 0.177352 | 0.169038 | NA | NA |  | 0.103569 |
| 0.169493 | 0.17016 | 0.169508 | 0.169182 | 0.161369 | NA | NA |  | NA |
| 0.174962 | 0.174742 | 0.173782 | 0.172485 | 0.15835 | NA | NA |  | 0.10771 |
| NA | NA | NA | NA | NA | NA | NA |  | 0.103138 |
| 0.110705 | 0.11155 | 0.111192 | 0.111459 | 0.112809 | NA | NA |  | 0.115138 |
| 0.218381 | 0.220263 | 0.217419 | 0.217271 | 0.197877 | NA | NA |  | 0.112989 |
| 0.233605 | 0.234468 | 0.232436 | 0.232082 | 0.212064 | NA | NA |  | 0.113579 |
| 0.161592 | 0.163314 | 0.163806 | 0.162768 | 0.163313 | NA | 0.129199 | | 0.114833 |

| 0.103485 | 0.103801 | 0.102795 | 0.102296 | 0.126886 | NA | NA | 0.106966 |
| --- | --- | --- | --- | --- | --- | --- | --- |
| 0.105279 | 0.105974 | 0.104936 | 0.104004 | 0.119344 | NA | NA | 0.109804 |
| 0.108684 | 0.109007 | 0.108194 | 0.107629 | 0.12954 | NA | NA | 0.109062 |
| NA | 0.095468 | NA | NA | 0.120661 | NA | NA | 0.097227 |
| NA | NA | NA | NA | NA | NA | NA | 0.155555 |
| NA | NA | NA | NA | NA | NA | NA | 0.137258 |
| NA | NA | NA | NA | NA | NA | NA | 0.143705 |
| NA | NA | NA | NA | NA | NA | NA | 0.128832 |
| NA | NA | NA | NA | NA | NA | NA | 0.14034 |
| NA | NA | NA | NA | NA | NA | NA | 0.137365 |
| NA | NA | NA | NA | NA | NA | NA | 0.120531 |
| NA | NA | NA | NA | NA | NA | NA | 0.120531 |
| NA | NA | NA | NA | NA | NA | NA | 0.120531 |
| NA | NA | NA | NA | NA | NA | NA | 0.136711 |
| NA | NA | NA | NA | NA | NA | NA | 0.137725 |
| NA | NA | NA | NA | NA | NA | NA | 0.138495 |
| NA | NA | NA | NA | NA | NA | NA | 0.124534 |
| NA | NA | NA | NA | NA | NA | NA | 0.12458 |
| 0.096546 | 0.095891 | 0.095523 | 0.096018 | NA | NA | NA | 0.133212 |
| NA | NA | NA | NA | NA | NA | NA | 0.138521 |
| NA | NA | NA | NA | NA | NA | NA | 0.107894 |
| 0.213249 | 0.21439 | 0.212978 | 0.212093 | 0.201662 | 0.114965 | 0.12795 | 0.115766 |
| 0.104272 | 0.104329 | 0.104389 | 0.104062 | NA | NA | NA | 0.121445 |
| 0.104272 | 0.104329 | 0.104389 | 0.104062 | NA | NA | NA | 0.121445 |
| NA | NA | NA | NA | NA | NA | NA | 0.105606 |
| NA | NA | NA | NA | NA | NA | NA | 0.105606 |
| 0.094997 | 0.094836 | 0.09492 | 0.094991 | NA | NA | NA | 0.124586 |
| 0.106483 | 0.105936 | 0.104919 | 0.105615 | NA | NA | NA | 0.138177 |
| NA | NA | NA | NA | NA | NA | NA | 0.117483 |
| NA | NA | NA | NA | NA | NA | NA | 0.119082 |
| NA | NA | NA | NA | NA | NA | NA | 0.119082 |
| NA | NA | NA | NA | NA | NA | NA | 0.119082 |
| NA | NA | NA | NA | NA | NA | NA | 0.099808 |
| NA | NA | NA | NA | NA | NA | NA | 0.101056 |
| NA | NA | NA | NA | NA | NA | NA | 0.101326 |
| NA | NA | NA | NA | NA | NA | NA | 0.101331 |
| 0.102197 | 0.102834 | 0.101744 | 0.102318 | 0.10355 | NA | NA | 0.106066 |
| 0.103264 | 0.105135 | 0.103825 | 0.104296 | 0.117118 | 0.114164 | 0.125123 | 0.096543 |
| 0.103264 | 0.105135 | 0.103825 | 0.104296 | 0.117118 | 0.114164 | 0.125123 | 0.096543 |

| 0.10861 | 0.110447 | 0.109091 | 0.109439 | 0.12886 | 0.144114 | 0.180287 | 0.105537 | |
| --- | --- | --- | --- | --- | --- | --- | --- | --- |
| NA | NA | NA | NA | NA | NA | NA | NA |  |
| 0.116235 | 0.11715 | 0.116388 | 0.116516 | 0.122736 | 0.13758 | 0.149723 | 0.106986 | |
| 0.097034 | 0.096672 | 0.097519 | 0.097314 | 0.104238 | NA | NA | 0.096322 | |
| 0.115426 | 0.115102 | 0.114627 | 0.113199 | 0.114409 | NA | NA | 0.100276 | |
| NA | NA | NA | NA | 0.107432 | NA | NA | NA |  |
| NA | NA | NA | NA | NA | NA | NA | 0.128536 | |
| NA | NA | NA | NA | NA | NA | NA | 0.134465 | |
| NA | NA | NA | NA | 0.096375 | NA | NA | 0.114824 | |
| NA | NA | NA | NA | NA | NA | NA | 0.117655 | |
| 0.094795 | 0.09533 | 0.094648 | 0.095409 | 0.100979 | NA | NA | 0.116681 | |
| NA | NA | NA | NA | NA | NA | NA | 0.103781 | |
| NA | NA | NA | NA | NA | NA | NA | 0.095342 | |
| NA | NA | NA | NA | NA | NA | NA | NA |  |
| NA | NA | NA | NA | NA | NA | NA | NA |  |
| NA | NA | NA | NA | NA | NA | NA | NA |  |
| NA | NA | NA | NA | NA | NA | NA | 0.102471 | |
| NA | NA | NA | NA | NA | NA | NA | 0.107691 | |
| NA | NA | NA | NA | NA | NA | NA | 0.106497 | |
| 0.102785 | 0.102785 | 0.102532 | 0.103146 | NA | NA | NA | 0.112486 | |
| NA | NA | NA | NA | NA | NA | NA | 0.097843 | |
| NA | NA | NA | NA | NA | NA | NA | 0.105227 | |
| NA | NA | NA | NA | NA | NA | NA | 0.106017 | |
| NA | NA | NA | NA | NA | NA | NA | 0.107957 | |
| NA | NA | NA | NA | NA | NA | NA | 0.098405 | |
| 0.100204 | 0.10129 | 0.100514 | 0.100958 | NA | NA | NA | 0.111927 | |
| NA | NA | NA | NA | NA | NA | NA | 0.092991 | |
| NA | NA | NA | NA | NA | NA | NA | 0.096803 | |
| NA | NA | NA | NA | NA | NA | NA | 0.101644 | |
| NA | NA | NA | NA | NA | NA | NA | 0.101747 | |
| 0.097244 | 0.09749 | 0.098448 | 0.096102 | NA | NA | NA | 0.128886 | |
| NA | NA | NA | NA | NA | NA | NA | 0.101832 | |
| NA | NA | NA | NA | NA | NA | NA |  | 0.1239 |
| NA | NA | NA | NA | NA | NA | NA | 0.120051 | |
| NA | NA | NA | NA | NA | NA | NA | 0.095846 | |
| NA | NA | NA | NA | NA | NA | NA | 0.106036 | |
| NA | NA | NA | NA | NA | NA | NA | 0.093526 | |
| NA | NA | NA | 0.094939 | NA | NA | NA | 0.102497 | |
| NA | NA | NA | NA | NA | NA | NA | 0.097197 | |

| NA | NA |  | NA | NA |  | NA | NA | NA |  | 0.108253 |
| --- | --- | --- | --- | --- | --- | --- | --- | --- | --- | --- |
| 0.10574 | 0.10631 | | 0.105824 | 0.10599 | | NA | NA | NA |  | 0.110126 |
| NA | NA |  | NA | NA |  | NA | NA | NA |  | 0.101702 |
| NA | NA |  | NA | NA |  | NA | NA | NA |  | NA |
| NA | NA |  | NA | NA |  | NA | NA | NA |  | 0.094083 |
| NA | 0.097207 | | 0.096075 | 0.09663 | | NA | NA | NA |  | 0.105879 |
| NA | NA |  | NA | NA |  | NA | NA | NA |  | NA |
| NA | NA |  | NA | NA |  | NA | NA | NA |  | 0.09651 |
| NA | NA |  | NA | NA |  | NA | NA | NA |  | 0.102193 |
| NA | NA |  | NA | NA |  | NA | NA | NA |  | 0.101174 |
| 0.141695 | 0.142266 | | 0.141335 | 0.139685 | | 0.12264 | NA | NA |  | 0.094788 |
| 0.094947 | 0.094453 | | 0.095306 | 0.09479 | | NA | NA | NA |  | 0.120548 |
| 0.096356 | 0.096057 | | 0.095149 |  | 0.0955 | NA | NA | NA |  | NA |
| 0.119423 | 0.118971 | | 0.118048 | 0.11836 | | 0.107192 | NA | NA |  | 0.093261 |
| 0.142187 | 0.14291 | | 0.143703 | 0.146095 | | 0.143477 | NA | NA |  | 0.109394 |
| 0.108647 | 0.109306 | | 0.109359 | 0.109398 | | 0.109454 | NA | NA |  | 0.096568 |
| NA | NA |  | NA | NA |  | 0.103472 | NA | NA |  | NA |
| 0.098048 | 0.098598 | | 0.097212 | 0.099332 | | NA | NA | NA |  | 0.09425 |
| 0.118271 | 0.119286 | | 0.117563 | 0.118332 | | 0.121386 | NA | NA |  | 0.097434 |
| 0.107891 | 0.106276 | | 0.106321 | 0.108409 | | 0.10406 | NA | NA |  | NA |
| 0.099345 | 0.100893 | | 0.098502 | 0.097599 | | 0.123909 | NA | NA |  | 0.108248 |
| 0.099779 | 0.09963 | | 0.098667 | 0.098518 | | 0.122725 | NA | NA |  | 0.104374 |
| 0.103839 | 0.103804 | | 0.102055 | 0.102234 | | 0.127613 | NA | NA |  | 0.107775 |
| NA | 0.093656 | | NA | NA |  | 0.11327 | NA | NA |  | 0.100483 |
| 0.100287 | 0.098596 | | 0.09844 | 0.099177 | | NA | NA | NA |  | NA |
| 0.102414 | 0.101672 | | 0.101838 | 0.102379 | | 0.099002 | NA | NA |  | 0.094351 |
| 0.100183 | 0.099648 | | 0.099839 |  | 0.1011 | 0.106865 | 0.11815 | 0.125874 | | NA |
| 0.105898 | 0.10408 | | 0.104854 | 0.104629 | | 0.109533 | NA | 0.123953 | | NA |
| 0.105898 | 0.10408 | | 0.104854 | 0.104629 | | 0.109533 | NA | 0.123953 | | NA |
| 0.098894 | 0.099003 | | 0.098058 | 0.099378 | | 0.09594 | 0.131492 | 0.134234 | | NA |
| 0.108631 | 0.107937 | | 0.107576 | 0.107972 | | 0.090878 | 0.118552 | 0.122963 | | NA |
| 0.100458 | 0.098438 | | 0.098337 | 0.099344 | | 0.093054 | NA | 0.097735 | | NA |
| 0.100458 | 0.098438 | | 0.098337 | 0.099344 | | 0.093054 | NA | 0.097735 | | NA |
| 0.100458 | 0.098438 | | 0.098337 | 0.099344 | | 0.093054 | NA | 0.097735 | | NA |
| 0.109854 | 0.108427 | | 0.108431 | 0.108428 | | 0.092762 | 0.122327 |  | 0.1176 | NA |
| 0.116204 |  | 0.1171 | 0.115991 | 0.116483 | | 0.111288 | 0.136644 | 0.153702 | | NA |
| NA | NA |  | NA | NA |  | NA | NA | 0.10845 | | NA |
| NA | NA |  | NA | NA |  | NA | NA | NA |  | 0.101487 |
| 0.092799 | NA |  | 0.093451 | NA |  | NA | NA | NA |  | 0.101492 |

| NA |  | NA |  | NA | NA | NA | NA | NA | 0.103591 |
| --- | --- | --- | --- | --- | --- | --- | --- | --- | --- |
| NA |  | NA |  | NA | NA | NA | NA | NA | 0.106294 |
| NA |  | NA |  | NA | NA | NA | NA | NA | 0.100734 |
| NA |  | NA |  | NA | NA | NA | NA | NA | 0.104637 |
| 0.116367 | | 0.116999 | | 0.116589 | 0.116461 | 0.126275 | NA | NA | 0.112479 |
| 0.113499 | | 0.111403 | | 0.111855 | 0.111532 | 0.122112 | NA | NA | 0.115617 |
| 0.10331 | | 0.104361 | | 0.104988 | 0.103592 | 0.109318 | NA | NA | 0.10005 |
| 0.11913 | | 0.119118 | | 0.118947 | 0.118905 | 0.126221 | NA | NA | 0.11574 |
| 0.101818 | | 0.101304 | | 0.102022 | 0.100821 | 0.119344 | NA | NA | 0.096198 |
| 0.105899 | | 0.10556 | | 0.10628 | 0.105772 | 0.120343 | NA | NA | 0.103388 |
| 0.114146 | | 0.113937 | | 0.11422 | 0.114459 | 0.119925 | NA | NA | 0.111579 |
| 0.120381 | | 0.11967 | | 0.120591 | 0.120831 | 0.137751 | 0.15117 | 0.185583 | 0.105161 |
| 0.126759 | | 0.125875 | | 0.127475 | 0.12693 | 0.144629 | 0.135582 | 0.180224 | 0.119929 |
| 0.101053 | | 0.101937 | | 0.101376 | 0.101411 | 0.101076 | NA | NA | 0.115911 |
| NA |  | NA |  | NA | NA | NA | NA | NA | 0.107328 |
| NA |  | NA |  | NA | NA | NA | NA | NA | 0.103394 |
|  | 0.1091 | 0.109049 | | 0.109353 | 0.108478 | 0.113156 | NA | NA | 0.114237 |
| NA |  | NA |  | NA | NA | NA | NA | NA | 0.101905 |
| 0.114979 | | 0.116474 | | 0.116988 | 0.11622 | 0.113861 | NA | NA | 0.123649 |
| NA |  | NA |  | NA | NA | NA | NA | NA | 0.103827 |
| NA |  | NA |  | NA | NA | NA | NA | NA | 0.103827 |
| 0.114407 | | 0.115492 | | 0.114426 | 0.11452 | 0.120483 | NA | 0.133261 | 0.115208 |
| 0.11709 | | 0.117796 | | 0.117569 | 0.118109 | 0.122754 | NA | NA | 0.112573 |
| 0.11709 | | 0.117796 | | 0.117569 | 0.118109 | 0.122754 | NA | NA | 0.112573 |
| 0.11709 | | 0.117796 | | 0.117569 | 0.118109 | 0.122754 | NA | NA | 0.112573 |
| 0.11709 | | 0.117796 | | 0.117569 | 0.118109 | 0.122754 | NA | NA | 0.112573 |
| 0.121113 | | 0.12191 | | 0.121809 | 0.121138 | 0.115643 | NA | NA | 0.111182 |
| 0.121113 | | 0.12191 | | 0.121809 | 0.121138 | 0.115643 | NA | NA | 0.111182 |
| 0.121113 | | 0.12191 | | 0.121809 | 0.121138 | 0.115643 | NA | NA | 0.111182 |
| 0.121113 | | 0.12191 | | 0.121809 | 0.121138 | 0.115643 | NA | NA | 0.111182 |
| 0.112299 | | 0.113491 | | 0.113403 | 0.112489 | 0.107166 | NA | NA | 0.129942 |
| 0.112299 | | 0.113491 | | 0.113403 | 0.112489 | 0.107166 | NA | NA | 0.129942 |
| 0.112012 | | 0.110792 | | 0.111439 | 0.112579 | 0.103822 | NA | NA | 0.117315 |
| 0.112012 | | 0.110792 | | 0.111439 | 0.112579 | 0.103822 | NA | NA | 0.117315 |
| 0.122812 | | 0.123743 | | 0.123433 | 0.123335 | 0.13082 | NA | 0.122877 | 0.116964 |
| 0.118682 | |  | 0.1197 | 0.119237 | 0.118969 | 0.121429 | NA | NA | 0.121998 |
| 0.118682 | |  | 0.1197 | 0.119237 | 0.118969 | 0.121429 | NA | NA | 0.121998 |
| 0.116339 | | 0.118303 | | 0.117863 | 0.118512 | 0.116776 | NA | NA | 0.124874 |
| 0.116339 | | 0.118303 | | 0.117863 | 0.118512 | 0.116776 | NA | NA | 0.124874 |

| 0.118572 | 0.119526 | 0.119176 | 0.118808 | 0.11792 | NA | NA | 0.119501 |
| --- | --- | --- | --- | --- | --- | --- | --- |
| 0.124002 | 0.12543 | 0.123483 | 0.124236 | 0.127479 | NA | 0.112056 | 0.125546 |
| 0.115326 | 0.117165 | 0.116674 | 0.116704 | 0.116952 | NA | NA | 0.121381 |
| 0.104987 | 0.106544 | 0.105538 | 0.105843 | 0.102495 | NA | NA | 0.106842 |
| 0.115031 | 0.114617 | 0.113717 | 0.112616 | 0.125664 | NA | NA | 0.109658 |
| 0.096985 | 0.098677 | NA | NA | 0.12165 | NA | NA | 0.105395 |
| 0.116014 | 0.115658 | 0.115061 | 0.114596 | 0.129449 | NA | NA | 0.106039 |
| 0.118941 | 0.119285 | 0.118561 | 0.118567 | 0.11984 | NA | NA | 0.149372 |
| 0.121787 | 0.121589 | 0.121317 | 0.121662 | 0.123727 | NA | NA | 0.151504 |
| NA | NA | NA | NA | 0.099862 | NA | NA | 0.11068 |
| 0.121112 | 0.121702 | 0.121472 | 0.121217 | 0.122595 | NA | NA | 0.153924 |
| 0.109305 | 0.109295 | 0.10903 | 0.110064 | 0.109802 | NA | NA | 0.135515 |
| NA | NA | NA | NA | NA | NA | 0.102516 | NA |
| 0.10685 | 0.107113 | 0.106643 | 0.106836 | 0.104844 | NA | NA | NA |
| NA | NA | NA | NA | NA | NA | NA | NA |
| NA | NA | NA | NA | NA | NA | NA | NA |
| NA | NA | NA | NA | NA | NA | NA | NA |
| 0.097841 | 0.095945 | 0.095892 | 0.096873 | NA | NA | NA | 0.096185 |
| NA | NA | NA | NA | NA | NA | NA | NA |
| NA | NA | NA | NA | NA | NA | NA | NA |
| NA | NA | NA | NA | NA | NA | NA | NA |
| NA | NA | NA | NA | NA | NA | NA | NA |
| NA | NA | NA | NA | NA | NA | NA | 0.094214 |
| 0.099442 | 0.09915 | 0.098815 | 0.100554 | NA | NA | NA | 0.093775 |
| 0.110018 | 0.10961 | 0.109276 | 0.109843 | 0.098164 | NA | NA | 0.093169 |
| NA | NA | NA | NA | NA | NA | NA | 0.101969 |
| NA | NA | NA | NA | NA | NA | NA | 0.103084 |
| NA | NA | NA | NA | NA | NA | NA | 0.102022 |
| 0.121525 | 0.12192 | 0.121463 | 0.122163 | 0.123841 | NA | NA | 0.118932 |
| 0.116418 | 0.116043 | 0.115291 | 0.115055 | 0.103699 | 0.120588 | 0.117539 | 0.094561 |
| 0.116418 | 0.116043 | 0.115291 | 0.115055 | 0.103699 | 0.120588 | 0.117539 | 0.094561 |
| NA | NA | NA | NA | NA | NA | NA | 0.140548 |
| NA | NA | NA | NA | NA | NA | NA | 0.099473 |
| NA | NA | NA | NA | NA | NA | NA | 0.09742 |
| 0.093548 | 0.09469 | 0.094013 | 0.093435 | NA | NA | NA | 0.100133 |
| 0.134671 | 0.134565 | 0.134564 | 0.135324 | 0.141879 | 0.120043 | 0.123442 | 0.099663 |
| NA | NA | NA | NA | NA | NA | NA | 0.109937 |
| NA | NA | NA | NA | NA | NA | NA | NA |
| NA | NA | NA | NA | NA | NA | NA | 0.107896 |

| 0.091789 | 0.092238 | 0.091385 | NA | NA | NA |  | NA | 0.108744 |
| --- | --- | --- | --- | --- | --- | --- | --- | --- |
| NA | NA | NA | NA | NA | NA |  | NA | 0.121667 |
| NA | NA | NA | NA | NA | NA |  | NA | 0.114356 |
| NA | NA | NA | NA | NA | NA |  | NA | 0.119433 |
| NA | NA | NA | NA | 0.096844 | NA |  | NA | 0.128886 |
| 0.11289 | 0.110436 | 0.110871 | 0.111203 | 0.119699 | NA |  | NA | 0.118029 |
| NA | NA | NA | NA | NA | NA |  | NA | 0.126855 |
| NA | NA | NA | NA | NA | NA |  | NA | 0.126855 |
| NA | NA | NA | NA | NA | NA |  | NA | 0.126855 |
| NA | NA | NA | NA | NA | NA |  | NA | 0.096086 |
| 0.106117 | 0.104607 | 0.105641 | 0.105044 | 0.11946 | NA |  | NA | 0.107313 |
| NA | NA | NA | NA | 0.102648 | NA |  | NA | 0.100659 |
| 0.109166 | 0.108882 | 0.108103 | 0.10884 | 0.118584 | NA |  | NA | 0.111535 |
| NA | NA | NA | NA | NA | NA |  | NA | 0.09487 |
| NA | NA | NA | NA | 0.105686 | NA |  | NA | NA |
| 0.096109 | 0.097249 | 0.096677 | 0.095925 | NA | NA |  | NA | 0.104733 |
| 0.097081 | 0.097127 | 0.095709 | 0.096805 | 0.103086 | NA |  | NA | 0.103415 |
| NA | NA | NA | NA | 0.104725 | NA |  | NA | NA |
| NA | NA | NA | NA | NA | NA |  | NA | 0.100631 |
| 0.106957 | 0.10704 | 0.107216 | 0.107334 | 0.109123 | 0.114258 | | 0.119703 | NA |
| 0.113889 | 0.113757 | 0.113637 | 0.113566 | 0.112417 |  | 0.1296 | 0.151961 | NA |
| 0.099115 | 0.099094 | 0.097631 | 0.097422 | 0.096888 | NA |  | NA | 0.105783 |
| 0.095891 | 0.096833 | 0.095578 | 0.095699 | 0.091754 | NA |  | NA | 0.105992 |
| 0.127043 | 0.128093 | 0.126634 | 0.127408 | 0.142246 | NA |  | NA | 0.105999 |
| 0.110549 | 0.109573 | 0.109486 | 0.11035 | 0.107875 | NA |  | NA | 0.107067 |
| 0.094502 | 0.095015 | 0.095061 | 0.092517 | 0.096807 | NA |  | NA | 0.099618 |
| 0.108739 | 0.108513 | 0.108695 | 0.108216 | 0.106512 | NA |  | NA | NA |
| 0.099122 | 0.09843 | 0.099185 | 0.098694 | 0.099243 | NA |  | NA | NA |
| 0.111501 | 0.113365 | 0.112438 | 0.112193 | 0.116564 | NA |  | NA | 0.112498 |
| 0.098914 | 0.100206 | 0.099177 | 0.099065 | 0.104402 | NA |  | NA | 0.102584 |
| 0.118609 | 0.118861 | 0.118629 | 0.117422 | 0.114401 | NA |  | NA | 0.138723 |
| 0.105164 | 0.10576 | 0.10556 | 0.105784 | 0.109498 | NA |  | NA | 0.114327 |
| 0.116844 | 0.116847 | 0.119834 | 0.116361 | 0.116797 | NA |  | NA | 0.130086 |
| 0.10707 | 0.106923 | 0.107166 | 0.10789 | 0.104997 | NA |  | NA | 0.106014 |
| 0.105407 | 0.108337 | 0.106377 | 0.105818 | 0.10663 | NA |  | NA | 0.123326 |
| NA | 0.09915 | 0.098925 | 0.097545 | NA | NA |  | NA | 0.114501 |
| NA | NA | NA | NA | NA | NA |  | NA | NA |
| NA | NA | NA | NA | NA | NA |  | NA | NA |
| NA | NA | NA | NA | NA | NA |  | NA | NA |

| 0.14351 | 0.145185 | | 0.14432 | 0.14708 | | 0.128391 | | NA | NA | NA |
| --- | --- | --- | --- | --- | --- | --- | --- | --- | --- | --- |
| 0.150651 | 0.153379 | | 0.152037 | 0.155073 | | 0.135505 | | NA | NA | NA |
| 0.136826 | 0.137734 | | 0.138202 |  | 0.1401 | 0.122412 | | NA | NA | NA |
| 0.094813 | 0.095451 | | 0.094505 | 0.093838 | |  | 0.0925 | NA | NA | 0.105833 |
| NA | NA |  | NA | NA |  | NA |  | NA | NA | 0.103259 |
| NA | NA |  | NA | NA |  | NA |  | NA | NA | 0.123607 |
| NA | NA |  | NA | NA |  | NA |  | NA | NA | 0.117227 |
| NA | 0.094492 | | 0.094042 | 0.094548 | | NA |  | NA | NA | 0.10349 |
| 0.107022 | 0.10642 | | 0.106477 | 0.107104 | | 0.108352 | | NA | NA | 0.100003 |
| 0.104105 | 0.105205 | | 0.105559 | 0.106041 | | 0.107907 | | NA | NA | 0.109266 |
| 0.105139 | 0.104823 | | 0.104779 | 0.105508 | | 0.110061 | | NA | NA | 0.109695 |
| 0.105139 | 0.104823 | | 0.104779 | 0.105508 | | 0.110061 | | NA | NA | 0.109695 |
| 0.106039 | 0.106392 | | 0.106332 | 0.106131 | | 0.112389 | | NA | NA | 0.108373 |
| 0.096222 | 0.096004 | | NA | NA |  | 0.120404 | | NA | NA | 0.096487 |
| 0.096222 | 0.096004 | | NA | NA |  | 0.120404 | | NA | NA | 0.096487 |
| 0.092455 | NA |  | 0.091875 | NA |  | NA |  | NA | NA | 0.104459 |
| NA | NA |  | NA | NA |  | NA |  | NA | NA | 0.096165 |
| NA | NA |  | NA | NA |  | NA |  | NA | NA | 0.105382 |
| 0.105574 | 0.104251 | | 0.104727 | 0.104865 | | 0.088712 | | 0.115228 | 0.125952 | NA |
| 0.164125 | 0.164733 | | 0.16402 | 0.164592 | | 0.156945 | | NA | NA | 0.112472 |
| 0.220926 | 0.219211 | | 0.220338 | 0.216084 | | 0.190951 | | NA | NA | 0.096491 |
| 0.149454 | 0.148455 | | 0.147986 | 0.146456 | | 0.123186 | | NA | NA | NA |
| 0.168982 | 0.168079 | | 0.167146 | 0.16662 | | 0.151358 | | NA | NA | 0.098406 |
| 0.205507 | 0.206549 | | 0.205078 | 0.203145 | | 0.186214 | | NA | NA | 0.098619 |
| 0.185575 | 0.186112 | | 0.184273 | 0.18258 | | 0.159209 | | NA | NA | 0.09608 |
| 0.105723 | 0.105111 | | 0.104774 | 0.104185 | | 0.104265 | | NA | NA | 0.102538 |
| 0.116407 |  | 0.1161 | 0.115523 | 0.115692 | | 0.108855 | | NA | NA | 0.093862 |
| 0.10835 | 0.107235 | | 0.106292 | 0.105911 | |  | 0.1064 | NA | NA | 0.108385 |
| 0.10169 | 0.103848 | | 0.101973 | 0.101019 | | 0.097307 | | NA | NA | 0.109531 |
| 0.120323 | 0.121654 | | 0.120777 | 0.120277 | | 0.121476 | | 0.11299 | 0.129698 | 0.11688 |
| 0.12295 | 0.125018 | | 0.124389 | 0.124002 | | 0.122224 | | NA | NA | 0.13022 |
| 0.09731 | 0.099207 | | 0.09837 | 0.099082 | | NA |  | NA | NA | 0.10609 |
| NA | NA |  | NA | NA |  | NA |  | NA | NA | 0.101481 |
| NA | NA |  | NA | NA |  | NA |  | NA | NA | 0.108853 |
| NA | NA |  | NA | NA |  | NA |  | NA | NA | 0.106027 |
| 0.100514 | 0.100856 | | 0.101197 | 0.101118 | | 0.113675 | | 0.127264 | 0.148086 | NA |
| 0.110591 | 0.109994 | | 0.111115 | 0.111185 | | 0.120434 | | 0.110287 | 0.149298 | 0.096666 |
| 0.10917 | 0.109714 | | 0.109722 | 0.10945 | | 0.121885 | | 0.139054 | 0.181116 | NA |
| NA | NA |  | NA | NA |  | NA |  | 0.137593 | 0.152636 | NA |

| 0.11012 | | 0.109764 | | 0.109836 | | 0.109725 | 0.121383 | 0.131385 | 0.161754 | 0.094798 |
| --- | --- | --- | --- | --- | --- | --- | --- | --- | --- | --- |
| 0.121813 | | 0.122068 | | 0.120662 | | 0.121653 | 0.128194 | NA | 0.13232 | 0.119348 |
| 0.110002 | | 0.110038 | | 0.108987 | | 0.110083 | 0.118805 | NA | NA | 0.111984 |
| 0.108259 | | 0.109469 | | 0.108659 | | 0.109236 | 0.116699 | NA | NA | 0.109349 |
| 0.102932 | | 0.103508 | | 0.103338 | | 0.104075 | 0.11288 | NA | NA | 0.105486 |
| 0.109375 | | 0.110138 | | 0.109657 | | 0.108645 | 0.111806 | NA | NA | 0.121986 |
| 0.096581 | | 0.096524 | |  | 0.0959 | 0.096813 | 0.10458 | NA | NA | 0.11493 |
|  | 0.1098 | 0.110667 | | 0.109923 | | 0.11045 | 0.119198 | NA | NA | 0.11113 |
| 0.103669 | | 0.104828 | | 0.103923 | | 0.105115 | 0.114778 | NA | NA | 0.105714 |
| NA |  | NA |  | NA |  | NA | NA | NA | NA | 0.103551 |
| NA |  | NA |  | NA |  | NA | NA | NA | NA | 0.104736 |
| NA |  | NA |  | NA |  | NA | NA | NA | NA | 0.099518 |
| 0.13177 | | 0.133209 | | 0.130723 | | 0.13181 | 0.144257 | 0.157412 | 0.195313 | 0.107927 |
| 0.114705 | | 0.114646 | | 0.114555 | | 0.115066 | 0.122739 | 0.124385 | 0.143777 | 0.101492 |
| 0.09875 | | 0.099786 | | 0.099096 | | 0.100954 | 0.097908 | NA | NA | 0.099108 |
| 0.110281 | | 0.109677 | | 0.110306 | | 0.109912 | 0.113887 | 0.128984 | 0.116308 | 0.09926 |
| 0.098309 | | 0.099314 | | 0.098755 | | 0.100234 | 0.096422 | NA | NA | 0.098338 |
| 0.098522 | | 0.09852 | | 0.098779 | | 0.099329 | 0.099743 | 0.115191 | 0.144755 | NA |
| 0.103035 | | 0.10321 | | 0.102851 | | 0.103147 | 0.110648 | 0.120795 | 0.144965 | NA |
| NA |  | NA |  | NA |  | NA | NA | NA | NA | 0.105154 |
| 0.120645 | | 0.121254 | | 0.122328 | | 0.120851 | 0.13333 | 0.127326 | 0.133751 | 0.117397 |
| 0.096069 | | 0.096383 | | 0.096218 | | 0.096539 | NA | NA | NA | 0.102547 |
| NA |  | NA |  | NA |  | NA | 0.105007 | 0.115206 | 0.15048 | NA |
| NA |  | NA |  | NA |  | NA | 0.102889 | 0.111331 | 0.129319 | NA |
| 0.10985 | | 0.109553 | | 0.109754 | | 0.110405 | 0.113325 | 0.12749 | 0.142379 | NA |
| 0.114051 | | 0.113773 | | 0.113101 | | 0.113952 | 0.101652 | 0.124753 | 0.127884 | NA |
| 0.112763 | | 0.112722 | | 0.113042 | | 0.113911 | 0.114897 | 0.134088 | 0.143376 | 0.09526 |
| 0.108097 | | 0.10787 | | 0.108749 | | 0.109084 | 0.117696 | 0.118773 | 0.13213 | NA |
| 0.106717 | | 0.106198 | | 0.106547 | | 0.105354 | 0.098043 | NA | NA | NA |
| 0.114515 | | 0.114165 | | 0.113751 | | 0.114179 | 0.119056 | NA | NA | 0.10512 |
| 0.119496 | | 0.120119 | | 0.119464 | | 0.119031 | 0.106683 | 0.106061 | 0.10685 | 0.096139 |
| 0.119688 | |  | 0.1199 | 0.120149 | | 0.120152 | 0.129633 | 0.121212 | 0.133741 | 0.113346 |
| 0.107524 | | 0.107989 | | 0.10726 | | 0.107663 | 0.105574 | NA | NA | 0.100702 |
| 0.109227 | | 0.109923 | | 0.108672 | | 0.109606 | 0.102909 | NA | NA | 0.107744 |
| 0.10115 | | 0.102224 | | 0.101278 | | 0.101953 | 0.114623 | 0.152327 | 0.165874 | NA |
| 0.115814 | | 0.115163 | | 0.115038 | | 0.114578 | 0.105903 | NA | NA | 0.101091 |
| 0.127217 | | 0.126586 | | 0.125521 | | 0.126104 | 0.119192 | NA | NA | 0.102614 |
| 0.110838 | | 0.112004 | | 0.111906 | | 0.111755 | 0.112813 | NA | NA | 0.101172 |
| 0.113119 | | 0.112588 | | 0.113425 | | 0.112493 | 0.128164 | 0.158952 | 0.174897 | 0.106467 |

| 0.118045 | 0.116506 | 0.117266 | | 0.117006 | 0.11905 | NA | NA | 0.102296 |
| --- | --- | --- | --- | --- | --- | --- | --- | --- |
| 0.119745 | 0.119138 | 0.118584 | | 0.119468 | 0.11004 | NA | NA | 0.100049 |
| 0.112524 | 0.113859 | 0.113482 | | 0.113073 | 0.116812 | NA | NA | 0.121743 |
| 0.106447 | 0.107122 | 0.107365 | | 0.107586 | 0.104479 | NA | NA | NA |
| 0.129003 | 0.130327 | 0.129543 | | 0.129787 | 0.134744 | NA | 0.122339 | 0.121692 |
| NA | NA | NA |  | NA | NA | NA | NA | 0.103921 |
| 0.117433 | 0.117482 | 0.117039 | | 0.116833 | 0.120848 | NA | NA | 0.110024 |
| 0.104414 | 0.104895 |  | 0.1049 | 0.104335 | NA | NA | NA | 0.10462 |
| 0.111265 | 0.111199 | 0.110869 | | 0.112186 | 0.117521 | 0.116701 | 0.130699 | 0.103802 |
| 0.111349 | 0.111988 | 0.111196 | | 0.110714 | 0.107245 | NA | NA | 0.10125 |
| 0.110386 | 0.11178 | 0.110874 | | 0.110113 | 0.107475 | NA | 0.126782 | NA |
| NA | NA | NA |  | NA | NA | NA | NA | 0.098511 |
| 0.110882 | 0.110918 | 0.110339 | | 0.109792 | 0.109056 | NA | NA | NA |
| 0.107731 | 0.107436 | 0.107343 | | 0.107898 | 0.102615 | 0.136727 | 0.154744 | NA |
| 0.112885 | 0.113356 | 0.112745 | | 0.112978 | 0.109578 | 0.145087 | 0.144952 | NA |
| 0.124069 | 0.12478 | 0.124345 | | 0.123915 | 0.135537 | 0.124815 | 0.147216 | 0.115685 |
| 0.09993 | 0.099307 | 0.097964 | | 0.100288 | 0.103901 | NA | 0.114493 | 0.096603 |
| 0.111241 | 0.112405 | 0.113009 | | 0.111521 | 0.120922 | NA | 0.123799 | 0.101884 |
| NA | NA | NA |  | NA | NA | NA | NA | NA |
| 0.10773 | 0.105704 | 0.107442 | | 0.107086 | 0.101752 | NA | NA | NA |
| 0.115947 | 0.11684 | 0.116643 | | 0.117123 | 0.103292 | NA | NA | 0.100581 |
| 0.107041 | 0.106034 | 0.107348 | | 0.105462 | NA | NA | NA | NA |
| 0.106645 | 0.108412 | 0.107228 | | 0.10685 | 0.112642 | NA | 0.110538 | 0.098319 |
| 0.121897 | 0.121915 | 0.121282 | | 0.120636 | 0.104207 | NA | NA | NA |
| 0.108519 | 0.109014 | 0.108531 | | 0.108337 | 0.099566 | NA | NA | 0.10238 |
| 0.11585 | 0.116331 | 0.115735 | | 0.115974 | 0.109512 | NA | NA | 0.099053 |
| 0.10843 | 0.110324 | 0.108421 | | 0.109317 | 0.109626 | NA | NA | 0.109919 |
| 0.11301 | 0.112906 | 0.112308 | | 0.113275 | 0.097382 | 0.116094 | 0.140286 | NA |
| 0.101568 | 0.103483 | 0.102597 | | 0.102697 | 0.106951 | NA | NA | 0.107302 |
| 0.100384 | 0.102258 | 0.100828 | | 0.101287 | 0.098622 | NA | NA | 0.103627 |
| 0.118116 | 0.118434 | 0.117973 | | 0.117396 | 0.105986 | NA | NA | NA |
| 0.117873 | 0.119062 | 0.117805 | | 0.118164 | 0.129444 | 0.159103 | 0.189747 | 0.102771 |
| 0.106341 | 0.106464 | 0.106834 | | 0.10636 | 0.107245 | 0.117042 | 0.123021 | NA |
| 0.111223 | 0.111088 | 0.110939 | | 0.111841 | 0.10172 | NA | NA | 0.096829 |
| NA | NA | NA |  | NA | NA | NA | NA | NA |
| 0.104006 | 0.103321 | 0.103497 | | 0.103444 | 0.10893 | NA | NA | 0.103013 |
| 0.111773 | 0.111825 | 0.112176 | | 0.112018 | 0.111939 | NA | NA | 0.101995 |
| 0.115235 | 0.115244 | 0.114837 | | 0.114468 | 0.099762 | 0.116401 | 0.113536 | NA |

| kidney..ce | large_inte | large_inte | large_inte | | liver..crest | macropha | macropha | mature_co |
| --- | --- | --- | --- | --- | --- | --- | --- | --- |
| 0.107986 | 0.101075 | 0.09863 | 0.100985 | | 0.104048 | 0.118146 | 0.109129 | 0.112008 |
| NA | NA | 0.09377 | NA |  | NA | NA | NA | NA |
| 0.115215 | 0.118744 | 0.115694 |  | 0.1155 | 0.114727 | 0.11786 | 0.11173 | 0.111857 |
| 0.098828 | 0.113375 | 0.121413 | 0.116969 | | 0.107708 | 0.132155 | 0.103077 | 0.105054 |
| 0.106909 | 0.115016 | 0.10412 | 0.10493 | | 0.113252 | 0.114822 | 0.098319 | 0.099387 |
| 0.120738 | 0.110213 | 0.104409 | 0.110788 | | 0.108606 | 0.132079 | 0.112362 | 0.115123 |
| 0.117031 | 0.117025 | 0.110475 | 0.110626 | | 0.117893 | 0.124066 | 0.111893 | 0.114168 |
| 0.103818 | 0.103587 | 0.098437 | 0.097015 | | 0.107539 | 0.109726 | 0.100357 | 0.10146 |
| 0.103818 | 0.103587 | 0.098437 | 0.097015 | | 0.107539 | 0.109726 | 0.100357 | 0.10146 |
| 0.114508 | 0.11538 | 0.117233 | 0.113709 | | 0.115872 | 0.118435 | 0.12108 | 0.12129 |
| 0.118258 | 0.107318 | 0.097605 | 0.103419 | | 0.105485 | 0.121063 | 0.110212 | 0.112019 |
| 0.105937 | 0.105978 | 0.098322 | 0.102478 | | 0.105101 | 0.109771 | 0.099651 | 0.101112 |
| 0.117043 | 0.106837 | 0.096275 | 0.098754 | | 0.097935 | 0.129081 | 0.120152 | 0.120794 |
| 0.11997 | 0.114217 | 0.105199 | 0.107787 | | 0.101816 | 0.128182 | 0.119245 | 0.120734 |
| NA | 0.121931 | 0.123707 | 0.099308 | | NA | NA | NA | NA |
| NA | 0.093146 | 0.097604 | 0.094597 | | NA | NA | NA | NA |
| 0.107746 | 0.103969 | 0.099605 | 0.099766 | | 0.092185 | NA | 0.103661 | 0.103255 |
| 0.107746 | 0.103969 | 0.099605 | 0.099766 | | 0.092185 | NA | 0.103661 | 0.103255 |
| 0.114856 | 0.106348 | 0.104778 | 0.101579 | | 0.088598 | 0.132941 | 0.177635 | 0.173756 |
| 0.113893 | 0.124977 | 0.118625 | 0.118186 | | 0.117615 | 0.118218 | 0.107899 | 0.108876 |
| NA | 0.093443 | 0.097867 | 0.09511 | | NA | NA | NA | NA |
| 0.106819 | 0.097765 | NA | 0.093995 | | 0.095478 | 0.133559 | 0.134434 | 0.13677 |
| NA | 0.123846 | 0.135366 | 0.131408 | | NA | NA | NA | NA |
| 0.100471 | 0.13726 | 0.150738 | 0.147921 | | NA | NA | NA | NA |
| NA | 0.098631 | 0.097263 | 0.097561 | | 0.103236 | 0.10235 | 0.114304 | 0.112537 |
| NA | 0.094192 | 0.095427 | NA |  | 0.118567 | 0.12385 | 0.134176 | 0.129558 |
| NA | 0.095848 | 0.097301 | NA |  | 0.11937 | 0.125169 | 0.135194 | 0.130918 |
| NA | NA | 0.094354 | NA |  | 0.112443 | 0.117418 | 0.126863 | 0.122776 |
| 0.115339 | 0.124348 | 0.115707 | 0.116978 | | 0.118192 | 0.112761 | 0.110947 | 0.110127 |
| 0.115339 | 0.124348 | 0.115707 | 0.116978 | | 0.118192 | 0.112761 | 0.110947 | 0.110127 |
| 0.133244 | 0.142925 | 0.128393 | 0.129459 | | 0.134815 | 0.13762 | 0.125823 | 0.127163 |
| 0.135403 | 0.147249 | 0.134173 | 0.134975 | | 0.13707 | 0.135772 | 0.130085 | 0.131616 |
| 0.11049 | 0.109236 | 0.109426 | 0.10844 | | 0.11091 | 0.109261 | 0.118134 | 0.118045 |
| NA | 0.100261 | 0.104839 | 0.102132 | | 0.104589 | 0.104848 | 0.123867 | 0.121387 |
| NA | NA | 0.093735 | NA |  | NA | NA | NA | NA |
| NA | 0.092707 | 0.095912 | 0.092476 | | NA | NA | NA | NA |
| NA | 0.092944 | 0.096728 | 0.093676 | | NA | NA | NA | NA |
| 0.098382 | NA | 0.095287 | NA |  | 0.095462 | 0.118619 | 0.102356 | 0.103205 |

| 0.105807 | NA |  | NA |  | NA | 0.097246 | 0.099231 | 0.092515 | 0.095166 |
| --- | --- | --- | --- | --- | --- | --- | --- | --- | --- |
| 0.105807 | NA |  | NA |  | NA | 0.097246 | 0.099231 | 0.092515 | 0.095166 |
| 0.105807 | NA |  | NA |  | NA | 0.097246 | 0.099231 | 0.092515 | 0.095166 |
| 0.105118 | 0.098124 | | 0.095887 | | 0.09847 | 0.096188 | 0.099884 | 0.105005 | 0.108055 |
| 0.100369 | 0.097705 | | 0.097254 | | 0.099609 | 0.096432 | 0.103625 | 0.102221 | 0.105085 |
| 0.127798 | 0.112119 | | 0.105744 | | 0.107782 | 0.094782 | 0.153482 | 0.177241 | 0.176456 |
| 0.144797 | 0.133194 | |  | 0.1318 | 0.131553 | 0.101369 | 0.176009 | 0.194412 | 0.193009 |
| 0.150105 | 0.131714 | | 0.128295 | | 0.12854 | 0.102207 | 0.19654 | 0.214457 | 0.212348 |
| 0.143507 | 0.122417 | | 0.116326 | | 0.118205 | 0.099945 | 0.166906 | 0.193203 | 0.192484 |
| 0.144636 | 0.139145 | | 0.136881 | | 0.136662 | 0.104194 | 0.170647 | 0.184098 | 0.182548 |
| 0.143731 | 0.129788 | | 0.127556 | | 0.127735 | 0.102753 | 0.183604 | 0.193382 | 0.192183 |
| 0.143351 | 0.136682 | | 0.134456 | | 0.13373 | 0.097553 | 0.183155 | 0.215335 | 0.21189 |
| 0.145868 | 0.123343 | | 0.122798 | | 0.124148 | 0.107648 | 0.182936 | 0.176117 | 0.177551 |
| 0.149522 | 0.123196 | | 0.119539 | | 0.121053 | 0.104567 | 0.183031 | 0.186588 | 0.187259 |
| 0.153735 | 0.126649 | | 0.125019 | | 0.125856 | 0.105572 | 0.197173 | 0.194457 | 0.19648 |
| NA | 0.094164 | | 0.097843 | | 0.095548 | NA | NA | NA | NA |
| 0.106196 | 0.113037 | | 0.106391 | | 0.10535 | 0.113613 | 0.108259 | 0.09991 | 0.100821 |
| 0.152369 | 0.124309 | | 0.121442 | | 0.122556 | 0.10386 | 0.191816 | 0.198874 | 0.198917 |
| 0.156437 | 0.126044 | | 0.121869 | | 0.122822 | 0.103233 | 0.201113 | 0.20622 | 0.206083 |
| 0.13598 | 0.114182 | | 0.111008 | | 0.111311 | 0.108649 | 0.160289 | 0.152042 | 0.153072 |
| 0.156305 | 0.12702 | | 0.119928 | | 0.121277 | 0.097883 | 0.200702 | 0.220786 | 0.220434 |
| 0.107947 | 0.101608 | | 0.101323 | | 0.102618 | 0.115134 | NA | NA | NA |
| NA | 0.094228 | | 0.098265 | | 0.095099 | NA | NA | NA | NA |
| 0.157296 |  | 0.1263 | 0.120138 | | 0.121706 | 0.100754 | 0.198469 | 0.211399 | 0.21076 |
| 0.133189 | 0.11382 | | 0.109561 | | 0.109948 | 0.102898 | 0.150251 | 0.147785 | 0.148967 |
| 0.131821 |  | 0.1136 | 0.111652 | | 0.112293 | 0.096801 | 0.163544 | 0.182729 | 0.182102 |
| 0.156322 | 0.125695 | | 0.122858 | | 0.123911 | 0.104796 | 0.200731 | 0.207563 | 0.208158 |
| 0.150921 | 0.124086 | | 0.121613 | | 0.121519 | 0.100887 | 0.18479 | 0.196915 | 0.196798 |
| 0.13171 | 0.114072 | | 0.114359 | | 0.115554 | 0.104873 | 0.155911 | 0.153092 | 0.153656 |
| 0.13171 | 0.114072 | | 0.114359 | | 0.115554 | 0.104873 | 0.155911 | 0.153092 | 0.153656 |
| 0.15559 | 0.125731 | | 0.122192 | | 0.122725 | 0.101505 | 0.201991 | 0.216313 | 0.216199 |
| 0.134005 | 0.107928 | | 0.106535 | | 0.107609 | 0.101175 | 0.165635 | 0.166791 | 0.167839 |
| 0.116403 | 0.105996 | | 0.105088 | | 0.10287 | NA | 0.160042 | 0.172104 | 0.170965 |
| 0.135576 | 0.118777 | | 0.112595 | | 0.113462 | 0.100592 | 0.153583 | 0.168378 | 0.168951 |
| 0.09562 | 0.091084 | | 0.095474 | | 0.092563 | NA | NA | NA | NA |
| 0.108049 | 0.107366 | | 0.099955 | | 0.104122 | 0.105756 | 0.111787 | 0.102015 | 0.103731 |
| 0.155159 | 0.128005 | | 0.123113 | | 0.123196 | 0.09778 | 0.191808 | 0.214707 | 0.214369 |
| 0.159196 | 0.127111 | | 0.121959 | | 0.123316 | 0.101061 | 0.206903 | 0.220333 | 0.220155 |
| 0.140534 | 0.115531 | | 0.112241 | | 0.112175 | 0.110752 | 0.161154 | 0.154051 | 0.155102 |

| 0.123335 | 0.117849 | 0.105192 | 0.108213 | 0.094132 | 0.124544 | 0.122245 | 0.123112 |
| --- | --- | --- | --- | --- | --- | --- | --- |
| 0.120196 | 0.11453 | 0.102929 | 0.105957 | 0.095658 | 0.116334 | 0.120961 | 0.121551 |
| 0.122592 | 0.116036 | 0.102618 | 0.106264 | 0.098169 | 0.128076 | 0.123965 | 0.125437 |
| 0.112627 | 0.113014 | 0.102427 | 0.104159 | 0.093671 | 0.119749 | 0.112007 | 0.113673 |
| 0.100216 | 0.139761 | 0.138485 | 0.146387 | 0.126377 | NA | NA | NA |
| 0.098228 | 0.125783 | 0.12561 | 0.129528 | 0.11777 | NA | NA | NA |
| 0.101395 | 0.132704 | 0.132969 | 0.138387 | 0.116861 | NA | NA | NA |
| 0.097419 | 0.124336 | 0.125105 | 0.128081 | 0.109531 | NA | NA | NA |
| 0.10546 | 0.135249 | 0.134556 | 0.140361 | 0.118123 | NA | NA | NA |
| 0.105267 | 0.132309 | 0.132307 | 0.137899 | 0.115588 | NA | NA | NA |
| 0.095996 | 0.115324 | 0.116628 | 0.119563 | 0.106497 | NA | NA | NA |
| 0.095996 | 0.115324 | 0.116628 | 0.119563 | 0.106497 | NA | NA | NA |
| 0.095996 | 0.115324 | 0.116628 | 0.119563 | 0.106497 | NA | NA | NA |
| 0.098964 | 0.130672 | 0.132665 | 0.136325 | 0.116406 | NA | NA | NA |
| 0.105609 | 0.131885 | 0.131861 | 0.137634 | 0.115775 | NA | NA | NA |
| 0.107751 | 0.13321 | 0.130461 | 0.135948 | 0.115605 | NA | 0.090879 | 0.09031 |
| 0.101078 | 0.120972 | 0.121818 | 0.125305 | 0.104941 | NA | NA | NA |
| 0.102586 | 0.122585 | 0.124194 | 0.125977 | 0.109545 | NA | NA | NA |
| 0.109236 | 0.128397 | 0.128917 | 0.133438 | 0.111928 | NA | 0.094264 | 0.093691 |
| 0.105549 | 0.132474 | 0.133037 | 0.138495 | 0.117813 | NA | 0.089856 | NA |
| NA | 0.11034 | 0.11435 | 0.116498 | 0.095689 | NA | NA | NA |
| 0.15307 | 0.12473 | 0.121931 | 0.123859 | 0.105884 | 0.19833 | 0.201374 | 0.202256 |
| 0.093854 | 0.107496 | 0.109112 | 0.108604 | 0.104405 | NA | 0.100112 | 0.09871 |
| 0.093854 | 0.107496 | 0.109112 | 0.108604 | 0.104405 | NA | 0.100112 | 0.09871 |
| NA | 0.100146 | 0.104287 | 0.101271 | 0.091598 | NA | 0.092449 | NA |
| NA | 0.100146 | 0.104287 | 0.101271 | 0.091598 | NA | 0.092449 | NA |
| NA | 0.106579 | 0.112848 | 0.112288 | 0.09768 | NA | 0.091713 | 0.089966 |
| NA | 0.11689 | 0.122047 | 0.12117 | 0.105331 | NA | 0.099115 | 0.098209 |
| NA | 0.103181 | 0.10802 | 0.105481 | 0.095502 | NA | NA | NA |
| NA | 0.104083 | 0.109614 | 0.107534 | 0.094509 | NA | NA | NA |
| NA | 0.104083 | 0.109614 | 0.107534 | 0.094509 | NA | NA | NA |
| NA | 0.104083 | 0.109614 | 0.107534 | 0.094509 | NA | NA | NA |
| NA | 0.093082 | 0.097177 | 0.093554 | NA | NA | NA | NA |
| NA | 0.092816 | 0.097322 | 0.094257 | NA | NA | NA | NA |
| 0.093414 | 0.091235 | 0.096054 | 0.092972 | NA | NA | NA | NA |
| 0.093637 | 0.093068 | 0.097418 | 0.092992 | NA | NA | NA | NA |
| 0.107139 | 0.098478 | NA | 0.096989 | 0.104952 | 0.103766 | 0.096095 | 0.097309 |
| 0.103528 | 0.099414 | 0.095514 | 0.095344 | 0.108211 | 0.118881 | 0.096788 | 0.098565 |
| 0.103528 | 0.099414 | 0.095514 | 0.095344 | 0.108211 | 0.118881 | 0.096788 | 0.098565 |

| 0.108818 | 0.114193 | | 0.111366 | | 0.109988 | | 0.116792 | | 0.134063 | 0.10074 | 0.102453 |
| --- | --- | --- | --- | --- | --- | --- | --- | --- | --- | --- | --- |
| NA | 0.105963 | | 0.104223 | | 0.099827 | | 0.109351 | | 0.106221 | NA | NA |
| 0.110678 | 0.10734 | | 0.102673 | | 0.103757 | | 0.111181 | | 0.124912 | 0.107953 | 0.10955 |
| 0.098513 | NA |  | NA |  | NA |  |  | 0.1036 | 0.106327 | NA | NA |
| 0.110544 | 0.101271 | | 0.096835 | | 0.099258 | | 0.104356 | | 0.115286 | 0.111394 | 0.112835 |
| NA | 0.096419 | | 0.100459 | | 0.097125 | | 0.106131 | | 0.106091 | 0.121217 | 0.117656 |
| 0.107448 | 0.104358 | | 0.109649 | | 0.109064 | | 0.095531 | | NA | NA | NA |
| 0.113852 | 0.105821 | | 0.11092 | | 0.110848 | | 0.097405 | | NA | NA | NA |
| 0.13221 | 0.125168 | | 0.127539 | | 0.129258 | | 0.148882 | | 0.095749 | NA | NA |
| 0.135295 | 0.127459 | | 0.129131 | | 0.130893 | | 0.15008 | | NA | NA | 0.090836 |
| 0.13199 | 0.120583 | | 0.119849 | | 0.124453 | | 0.144821 | | 0.100252 | 0.092224 | 0.094137 |
| 0.094767 | 0.09258 | | 0.096731 | |  | 0.0943 | NA |  | NA | NA | NA |
| 0.098438 | 0.132967 | | 0.146836 | | 0.143621 | | NA |  | NA | NA | NA |
| NA | 0.121893 | | 0.132273 | | 0.131516 | | NA |  | NA | NA | NA |
| NA | 0.122546 | | 0.131068 | | 0.13599 | | NA |  | NA | NA | NA |
| NA | 0.110174 | | 0.114038 | | 0.120677 | | NA |  | NA | NA | NA |
| 0.094977 | 0.090947 | | 0.09544 | | 0.092311 | | NA |  | NA | NA | NA |
| 0.102314 | 0.094032 | | 0.093883 | | 0.093495 | | NA |  | NA | NA | NA |
| 0.097625 | 0.092322 | | 0.095099 | | 0.09306 | | NA |  | NA | NA | NA |
| 0.11985 | 0.109352 | | 0.11116 | | 0.111126 | | NA |  | NA | 0.093248 | 0.091719 |
| 0.095584 | 0.121874 | | 0.131365 | | 0.132218 | | NA |  | NA | NA | NA |
| 0.098491 | 0.096368 | |  | 0.0991 | 0.095912 | | NA |  | NA | NA | NA |
| 0.100984 | 0.092943 | | 0.097159 | | 0.096175 | | NA |  | NA | NA | NA |
| 0.101655 | 0.091065 | | 0.093254 | | 0.091868 | | NA |  | NA | NA | NA |
| 0.101943 | 0.135349 | | 0.148368 | | 0.146136 | | NA |  | NA | NA | NA |
| 0.121636 | 0.120011 | | 0.124676 | | 0.124192 | | NA |  | NA | 0.093608 | 0.092443 |
| 0.099366 | 0.122013 | | 0.12806 | | 0.13501 | | NA |  | NA | NA | NA |
| 0.101944 | 0.123609 | | 0.125891 | | 0.134475 | | NA |  | NA | NA | NA |
| 0.094265 | 0.090282 | |  | 0.0951 | 0.09186 | | NA |  | NA | NA | NA |
| 0.094171 | 0.090245 | | 0.095235 | | 0.091871 | | NA |  | NA | NA | NA |
| 0.114438 | 0.097955 | | 0.095442 | | 0.096287 | | 0.094126 | | NA | 0.09617 | 0.095954 |
| 0.093885 | 0.091589 | | 0.095781 | | 0.092977 | | NA |  | NA | NA | NA |
| 0.110124 |  | 0.0966 | 0.09965 | | 0.097727 | | NA |  | NA | NA | NA |
| 0.104502 | 0.092389 | | 0.094992 | | 0.093574 | | NA |  | NA | NA | NA |
| 0.101281 | 0.132585 | | 0.144384 | | 0.143382 | | NA |  | NA | NA | NA |
| 0.109031 | 0.133854 | | 0.143766 | | 0.144666 | | NA |  | NA | 0.094353 | NA |
| 0.099317 | 0.134255 | | 0.147463 | | 0.145342 | | NA |  | NA | NA | NA |
| 0.107158 | 0.138698 | | 0.150516 | | 0.152405 | | NA |  | NA | 0.094765 | NA |
| 0.100291 | 0.123152 | | 0.124675 | | 0.132673 | | NA |  | NA | NA | NA |

| 0.103128 | 0.090758 | | 0.09239 | 0.091223 | | NA | NA | NA | NA |
| --- | --- | --- | --- | --- | --- | --- | --- | --- | --- |
| 0.122768 | 0.11097 | | 0.112583 | 0.112821 | | NA | NA | 0.095199 | 0.09373 |
| 0.093255 | 0.091306 | | 0.09573 | 0.092745 | | NA | NA | NA | NA |
| NA | 0.121346 | | 0.131835 | 0.131072 | | NA | NA | NA | NA |
| NA | 0.124546 | | 0.136617 | 0.133318 | | NA | NA | NA | NA |
| 0.108878 | 0.136778 | | 0.147147 | 0.148409 | | NA | NA | 0.097223 | 0.095562 |
| 0.09536 | 0.125295 | | 0.133634 | 0.137919 | | NA | NA | NA | NA |
| 0.101668 | 0.122688 | | 0.128474 | 0.136942 | | NA | NA | NA | NA |
| 0.094801 | 0.091571 | | 0.095733 | 0.093106 | | NA | NA | NA | NA |
| 0.106411 | 0.135412 | | 0.144325 | 0.147674 | | NA | NA | 0.094302 | NA |
| 0.109595 | 0.10095 | | 0.097123 | 0.098086 | | 0.089896 | 0.116114 | 0.145937 | 0.144186 |
| 0.118236 | 0.123446 | | 0.126306 | 0.123146 | | 0.115356 | NA | 0.117035 | 0.101746 |
| 0.096678 | 0.099399 | | 0.103003 | 0.094817 | | NA | NA | 0.110914 | 0.106457 |
| 0.107532 |  | 0.1077 | 0.110302 | 0.103578 | | 0.092853 | 0.102627 | 0.133939 | 0.130211 |
| 0.149315 | 0.142183 | | 0.144438 | 0.134719 | | 0.117829 | 0.141007 | 0.166848 | 0.164072 |
| 0.106902 | 0.103908 | | 0.105596 | 0.102438 | | 0.102076 | 0.108809 | 0.115545 | 0.114984 |
| NA | 0.098936 | | 0.101469 | 0.095615 | | 0.09942 | 0.103567 | 0.099271 | 0.09845 |
| 0.100888 | 0.104665 | | 0.106388 | 0.10057 | | 0.092684 | NA | 0.111344 | 0.108187 |
| 0.113718 | 0.110178 | | 0.11103 | 0.106357 | | 0.099719 | 0.118539 | 0.137381 | 0.134827 |
| 0.115752 | 0.113093 | | 0.115476 |  | 0.1061 | 0.099593 | NA | 0.124063 | 0.121804 |
| 0.123744 | 0.121039 | | 0.106721 | 0.110486 | | NA | 0.121211 | 0.119957 | 0.121261 |
| 0.118713 | 0.113767 | | 0.102076 | 0.104191 | | 0.09367 | 0.122066 | 0.117322 | 0.117752 |
| 0.118672 | 0.116142 | | 0.104676 | 0.107509 | | 0.095668 | 0.124463 | 0.12249 | 0.123831 |
| 0.111411 | 0.113069 | | 0.105049 | 0.105214 | | NA | 0.111441 | 0.110005 | 0.110801 |
| NA | 0.118937 | | 0.112659 | 0.119498 | | NA | NA | 0.096075 | 0.09883 |
| 0.102901 | NA |  | NA | NA |  | 0.09849 | 0.095753 | 0.101475 | 0.101104 |
| NA | 0.125419 | | 0.120564 | 0.12539 | | 0.096344 | 0.108402 | 0.096809 | 0.099153 |
| NA | 0.12439 | | 0.119234 | 0.123125 | | 0.099293 | 0.109542 | 0.101603 | 0.104549 |
| NA | 0.12439 | | 0.119234 | 0.123125 | | 0.099293 | 0.109542 | 0.101603 | 0.104549 |
| NA | 0.110499 | | 0.111182 | 0.113482 | | NA | 0.096758 | NA | 0.096271 |
| 0.096057 | 0.104309 | | 0.099174 | 0.10441 | | 0.093524 | 0.089849 | 0.100001 | 0.102118 |
| NA | 0.119433 | | 0.114088 | 0.119907 | | NA | 0.093188 | 0.095515 | 0.097924 |
| NA | 0.119433 | | 0.114088 | 0.119907 | | NA | 0.093188 | 0.095515 | 0.097924 |
| NA | 0.119433 | | 0.114088 | 0.119907 | | NA | 0.093188 | 0.095515 | 0.097924 |
| 0.097684 | 0.110691 | | 0.104861 | 0.110039 | | 0.09281 | 0.090731 | 0.103477 | 0.104599 |
| 0.102034 | NA |  | NA | NA |  | 0.097639 | 0.111877 | 0.104818 | 0.106097 |
| NA | 0.121087 | | 0.118899 | 0.122801 | | NA | NA | NA | NA |
| 0.096071 | 0.092153 | | 0.094842 | 0.094994 | | NA | NA | NA | NA |
| 0.101367 | 0.109403 | | 0.111171 |  | 0.1078 | 0.104046 | NA | 0.09638 | 0.095214 |

| 0.09528 | 0.095129 | 0.097068 | 0.096462 | | NA | NA | NA | NA |
| --- | --- | --- | --- | --- | --- | --- | --- | --- |
| 0.094496 | 0.092459 | 0.096523 | 0.094034 | | NA | NA | NA | NA |
| 0.094138 | 0.092642 | 0.097372 | 0.094555 | | NA | NA | NA | NA |
| 0.095765 | 0.093823 | 0.097379 | 0.096902 | | NA | NA | NA | NA |
| 0.113858 | 0.09932 | 0.095452 | 0.099604 | | 0.105231 | 0.127711 | 0.104796 | 0.107348 |
| 0.118546 | 0.100097 | 0.095298 | 0.098646 | | 0.110626 | 0.120028 | 0.10983 | 0.110413 |
| 0.109829 | NA | NA | NA |  | 0.106954 | 0.110574 | 0.098579 | 0.100307 |
| 0.116729 | 0.103265 | 0.100797 | 0.10321 | | 0.108483 | 0.124468 | 0.113422 | 0.114866 |
| 0.105434 | NA | NA | NA |  | 0.099853 | 0.119733 | 0.101073 | 0.10169 |
| 0.107778 | NA | NA | NA |  | 0.102003 | 0.120016 | 0.105581 | 0.105917 |
| 0.113042 | 0.118123 | 0.110758 | 0.112441 | | 0.114531 | 0.11878 | 0.109798 | 0.110327 |
| 0.117426 | 0.09993 | 0.106063 | 0.10757 | | 0.109865 | 0.141286 | 0.108942 | 0.110115 |
| 0.131562 | 0.113475 | 0.117504 | 0.116303 | | 0.116552 | 0.148509 | 0.117691 | 0.11836 |
| 0.108064 | 0.104351 | 0.099703 |  | 0.1028 | 0.103941 | 0.099183 | 0.097478 | 0.098395 |
| 0.100208 | 0.096508 | 0.098605 | 0.096928 | | NA | NA | NA | NA |
| 0.096363 | 0.091419 | 0.095593 | 0.092929 | | NA | NA | NA | NA |
| 0.108287 | 0.124493 | 0.117124 | 0.116747 | | 0.116717 | 0.112035 | 0.104649 | 0.105278 |
| 0.093945 | 0.092176 | 0.09698 | 0.094343 | | NA | NA | NA | NA |
| 0.114002 | 0.111699 | 0.10657 | 0.112135 | | 0.106848 | 0.113736 | 0.10445 | 0.105931 |
| 0.094917 | 0.098944 | 0.101425 | 0.100102 | | NA | NA | NA | NA |
| 0.094917 | 0.098944 | 0.101425 | 0.100102 | | NA | NA | NA | NA |
| 0.113154 | 0.112585 | 0.110238 | 0.114378 | | 0.114138 | 0.122926 | 0.109707 | 0.110157 |
| 0.115925 | 0.120559 | 0.111288 | 0.112339 | | 0.116925 | 0.121665 | 0.11096 | 0.113773 |
| 0.115925 | 0.120559 | 0.111288 | 0.112339 | | 0.116925 | 0.121665 | 0.11096 | 0.113773 |
| 0.115925 | 0.120559 | 0.111288 | 0.112339 | | 0.116925 | 0.121665 | 0.11096 | 0.113773 |
| 0.115925 | 0.120559 | 0.111288 | 0.112339 | | 0.116925 | 0.121665 | 0.11096 | 0.113773 |
| 0.116951 | 0.105413 | 0.101926 | 0.105288 | | 0.106784 | 0.115395 | 0.117067 | 0.117309 |
| 0.116951 | 0.105413 | 0.101926 | 0.105288 | | 0.106784 | 0.115395 | 0.117067 | 0.117309 |
| 0.116951 | 0.105413 | 0.101926 | 0.105288 | | 0.106784 | 0.115395 | 0.117067 | 0.117309 |
| 0.116951 | 0.105413 | 0.101926 | 0.105288 | | 0.106784 | 0.115395 | 0.117067 | 0.117309 |
| 0.126555 | 0.130582 | 0.132609 | 0.130532 | | 0.118064 | 0.105558 | 0.116227 | 0.115492 |
| 0.126555 | 0.130582 | 0.132609 | 0.130532 | | 0.118064 | 0.105558 | 0.116227 | 0.115492 |
| 0.112041 | 0.121062 | 0.121406 | 0.121234 | | 0.110572 | 0.103334 | 0.113346 | 0.113137 |
| 0.112041 | 0.121062 | 0.121406 | 0.121234 | | 0.110572 | 0.103334 | 0.113346 | 0.113137 |
| 0.121676 | 0.128033 | 0.119367 | 0.118856 | | 0.122811 | 0.130334 | 0.117749 | 0.118129 |
| 0.11596 | 0.109258 | 0.103876 | 0.110163 | | 0.106062 | 0.12179 | 0.103958 | 0.106024 |
| 0.11596 | 0.109258 | 0.103876 | 0.110163 | | 0.106062 | 0.12179 | 0.103958 | 0.106024 |
| 0.115174 | 0.112658 | 0.107764 | 0.113176 | | 0.106018 | 0.117087 | 0.109185 | 0.109669 |
| 0.115174 | 0.112658 | 0.107764 | 0.113176 | | 0.106018 | 0.117087 | 0.109185 | 0.109669 |

| 0.109545 | 0.108387 | 0.103215 |  | 0.1081 | 0.105583 | | 0.11909 | 0.102115 | 0.104148 |
| --- | --- | --- | --- | --- | --- | --- | --- | --- | --- |
| 0.119939 | 0.110637 | 0.104301 | 0.110864 | | 0.110568 | | 0.12768 | 0.10769 | 0.110316 |
| 0.112816 | 0.108853 | 0.100415 | 0.105802 | | 0.108722 | | 0.117569 | 0.103165 | 0.105449 |
| 0.100253 | 0.101083 | 0.094725 | 0.097378 | | 0.096568 | | 0.101874 | 0.10087 | 0.101717 |
| 0.117869 | 0.106195 | 0.098397 | 0.100967 | | 0.099301 | | 0.122557 | 0.122153 | 0.122647 |
| 0.122357 | 0.121893 | 0.108116 | 0.111266 | | NA |  | 0.122378 | 0.117951 | 0.118899 |
| 0.115976 | 0.103694 | 0.095599 | 0.099241 | | 0.101839 | | 0.12976 | 0.119888 | 0.120561 |
| 0.164645 | 0.123776 | 0.117894 | 0.121344 | | 0.131327 | | 0.122995 | 0.106984 | 0.108714 |
| 0.169135 | 0.125682 | 0.118826 | 0.12217 | | 0.134306 | | 0.127519 | 0.108544 | 0.11034 |
| 0.110459 | 0.10108 | 0.098904 | 0.099606 | | 0.111632 | | 0.100514 | NA | NA |
| 0.170953 | 0.12786 | 0.120284 | 0.124468 | | 0.135899 | | 0.126194 | 0.108696 | 0.110623 |
| 0.129587 | 0.11481 | 0.111582 | 0.113538 | | 0.12344 | | 0.109649 | 0.103811 | 0.104507 |
| NA | NA | NA | NA |  | NA |  | NA | NA | NA |
| 0.106446 | NA | NA | NA |  | 0.095469 | | 0.103566 | 0.104144 | 0.106757 |
| NA | NA | NA | NA |  | NA |  | NA | NA | NA |
| NA | NA | NA | NA |  | NA |  | NA | NA | NA |
| NA | NA | NA | NA |  | NA |  | NA | NA | NA |
| 0.097217 | NA | NA | NA |  | 0.093194 | | NA | 0.097158 | 0.096746 |
| NA | NA | NA | NA |  | NA |  | NA | NA | NA |
| NA | NA | NA | NA |  | NA |  | NA | NA | NA |
| NA | NA | NA | NA |  | NA |  | NA | NA | NA |
| NA | NA | NA | NA |  | NA |  | NA | NA | NA |
| NA | NA | NA | NA |  | NA |  | NA | NA | NA |
| NA | 0.093059 | NA | 0.091384 | | NA |  | NA | 0.099588 | 0.099545 |
| 0.096211 | 0.092453 | NA | 0.091277 | | NA |  | 0.09633 | 0.108581 | 0.108585 |
| 0.094157 | 0.091796 | 0.096114 | 0.093487 | | NA |  | NA | NA | NA |
| 0.095681 | 0.091358 | 0.096035 | 0.093214 | | NA |  | NA | NA | NA |
| NA | 0.093427 | 0.097208 | 0.09493 | | NA |  | NA | NA | NA |
| 0.120597 | 0.127935 | 0.119465 | 0.119515 | | 0.121908 | | 0.122374 | 0.116902 | 0.117016 |
| 0.108619 | 0.098884 | 0.096281 | 0.097103 | | 0.098844 | | 0.099546 | 0.11127 | 0.111141 |
| 0.108619 | 0.098884 | 0.096281 | 0.097103 | | 0.098844 | | 0.099546 | 0.11127 | 0.111141 |
| 0.175499 | 0.163515 | 0.168328 | 0.164175 | | 0.189139 | | NA | NA | NA |
| 0.110361 | 0.095025 | NA | NA |  | 0.11594 | | NA | NA | NA |
| 0.116648 | 0.102146 | 0.099715 | 0.100174 | | 0.122696 | | NA | NA | NA |
| 0.122311 | 0.110035 | 0.109044 | 0.109743 | |  | 0.1289 | NA | NA | 0.093043 |
| 0.118781 | 0.102975 | 0.101071 | 0.101341 | | 0.101437 | | 0.142212 | 0.125889 | 0.127643 |
| 0.128439 | 0.116081 | 0.115499 | 0.118383 | | 0.139467 | | NA | 0.089039 | 0.090938 |
| 0.104906 | 0.095279 | 0.096381 | 0.094372 | | 0.115186 | | NA | NA | NA |
| 0.126977 | 0.112176 | 0.109913 | 0.112695 | | 0.134445 | | 0.094652 | NA | 0.092428 |

| 0.122263 | | 0.112804 | | 0.113552 | 0.113588 | 0.129924 | NA | 0.093211 | 0.093827 |
| --- | --- | --- | --- | --- | --- | --- | --- | --- | --- |
| 0.114326 | | 0.104425 | | 0.104062 | 0.106069 | 0.115504 | NA | NA | NA |
| 0.116464 | | 0.106799 | | 0.10649 | 0.107444 | 0.117593 | NA | NA | NA |
| 0.116927 | | 0.105177 | | 0.105503 | 0.106937 | 0.124993 | NA | NA | NA |
| 0.10837 | | 0.099409 | | 0.097538 | 0.100833 | 0.115324 | NA | NA | NA |
| 0.119395 | | 0.101828 | | 0.096796 | 0.100215 | 0.110634 | 0.118118 | 0.111198 | 0.111456 |
| NA |  | NA |  | 0.094715 | NA | 0.105817 | NA | NA | NA |
| NA |  | NA |  | 0.094715 | NA | 0.105817 | NA | NA | NA |
| NA |  | NA |  | 0.094715 | NA | 0.105817 | NA | NA | NA |
| NA |  | NA |  | 0.093849 | NA | NA | NA | NA | NA |
| 0.14932 | | 0.098855 | | NA | 0.098626 | 0.105415 | 0.11983 | 0.106783 | 0.106519 |
| 0.120421 | | 0.09611 | | NA | NA | 0.103616 | 0.103591 | 0.097769 | 0.09839 |
| 0.120143 | | 0.106787 | | 0.102178 | 0.104613 | 0.108185 | 0.116496 | 0.112855 | 0.113879 |
| NA |  | NA |  | NA | NA | NA | NA | NA | NA |
| 0.119712 | | NA |  | NA | NA | 0.099801 | 0.105255 | NA | NA |
| 0.119533 | |  | 0.0998 | 0.100698 | 0.100261 | 0.097818 | NA | 0.100803 | 0.101062 |
| 0.111257 | | 0.102172 | | 0.097966 | 0.098899 | 0.102963 | 0.101382 | 0.098198 | 0.099446 |
| 0.109067 | | NA |  | NA | NA | 0.101056 | 0.105095 | NA | NA |
| 0.095131 | | NA |  | 0.091385 | NA | NA | NA | NA | NA |
| NA |  | NA |  | NA | NA | 0.094163 | 0.110449 | NA | 0.097024 |
| 0.100085 | | NA |  | NA | NA | 0.097988 | 0.113771 | 0.101389 | 0.103821 |
|  | 0.1161 | 0.105533 | | 0.103693 | 0.105893 | 0.121019 | 0.096456 | 0.096689 | 0.096898 |
| 0.125286 | | 0.113009 | | 0.111835 | 0.113608 | 0.131321 | 0.090714 | 0.095718 | 0.096062 |
| 0.09796 | | 0.096496 | | 0.115858 | 0.114171 | 0.10965 | 0.145593 | 0.121936 | 0.123001 |
| 0.108745 | | 0.099683 | | 0.095398 | 0.098399 | 0.104157 | 0.107639 | 0.105028 | 0.105714 |
| 0.109923 | | 0.098969 | | NA | 0.096623 | 0.112654 | 0.095072 | 0.093573 | 0.094418 |
| 0.107553 | | 0.094198 | | NA | NA | 0.094729 | 0.106646 | 0.106323 | 0.108733 |
| 0.104369 | | NA |  | NA | NA | 0.097953 | 0.098494 | 0.097478 | 0.099243 |
| 0.110431 | | 0.103207 | | 0.100435 | 0.103191 | 0.10594 | 0.115919 | 0.11068 | 0.111866 |
| 0.102461 | | 0.094702 | | NA | NA | 0.099372 | 0.104174 | 0.095098 | 0.096437 |
| 0.124871 | | 0.11581 | | 0.112291 | 0.116094 | 0.114771 | 0.115904 | 0.114794 | 0.114392 |
| 0.10792 | | 0.101937 | | 0.098441 | 0.100752 | 0.102855 | 0.109539 | 0.100919 | 0.102946 |
| 0.119599 | | 0.112291 | | 0.110596 | 0.114034 | 0.110027 | 0.118053 | 0.11245 | 0.11395 |
| 0.108418 | | 0.095727 | | NA | NA | 0.101928 | 0.105049 | 0.10087 | 0.102718 |
| 0.113932 | | 0.106272 | | 0.104863 | 0.107579 | 0.104809 | 0.106646 | 0.105127 | 0.106323 |
| 0.103661 | | 0.101718 | | 0.100228 | 0.102544 | 0.097952 | NA | 0.096528 | 0.099114 |
| NA |  | NA |  | NA | NA | 0.096591 | NA | NA | NA |
| NA |  | NA |  | NA | NA | 0.096591 | NA | NA | NA |
| NA |  | NA |  | NA | NA | 0.096591 | NA | NA | NA |

| 0.099285 | 0.096349 | | NA | NA | NA | 0.12634 | 0.135154 | | 0.135769 |
| --- | --- | --- | --- | --- | --- | --- | --- | --- | --- |
| 0.101455 | 0.097633 | | NA | 0.093501 | NA | 0.133458 | 0.140179 | | 0.142063 |
| 0.097771 | NA |  | NA | NA | NA | 0.122122 | 0.125394 | | 0.126561 |
| 0.126366 | 0.11286 | | 0.112719 | 0.113931 | 0.133162 | NA | 0.094439 | | 0.09515 |
| 0.118443 | 0.102183 | | 0.102262 | 0.102867 | 0.126834 | NA | NA |  | NA |
| 0.15649 |  | 0.1415 | 0.143708 | 0.143217 | 0.167329 | NA | NA |  | NA |
| 0.149108 | 0.13281 | | 0.136658 | 0.135346 | 0.159201 | NA | NA |  | NA |
| 0.110222 | 0.098253 | | 0.095785 | 0.095752 | 0.09833 | NA | 0.099209 | | 0.098817 |
| 0.103967 | 0.093574 | | NA | NA | 0.102594 | 0.10641 | 0.100456 | | 0.101663 |
| 0.108625 | 0.100155 | | 0.094808 | 0.098554 | 0.108433 | 0.106767 | 0.098265 | | 0.099959 |
| 0.113302 | 0.099029 | | 0.095242 | 0.098429 | 0.108589 | 0.11047 | 0.10065 | | 0.101815 |
| 0.113302 | 0.099029 | | 0.095242 | 0.098429 | 0.108589 | 0.11047 | 0.10065 | | 0.101815 |
| 0.111309 | 0.102816 | | 0.099363 | 0.101454 | 0.107538 | 0.112263 | 0.102752 | | 0.103932 |
| 0.110631 | 0.108038 | | 0.096705 | 0.098708 | NA | 0.118056 | 0.112397 | | 0.113626 |
| 0.110631 | 0.108038 | | 0.096705 | 0.098708 | NA | 0.118056 | 0.112397 | | 0.113626 |
| 0.094782 | 0.101746 | | 0.104309 | 0.10456 | 0.097825 | NA | 0.093495 | | 0.093194 |
| 0.091657 | 0.098787 | | 0.101739 | 0.099973 | 0.092982 | NA | 0.092718 | | 0.091677 |
| NA | 0.102657 | | 0.105008 | 0.104989 | 0.096543 | NA | 0.092182 | | 0.091429 |
| NA | 0.105183 | | 0.102351 | 0.105468 | NA | 0.087613 | 0.098587 | | 0.100415 |
| 0.136367 | 0.120574 | | 0.116788 | 0.117829 | 0.103602 | 0.155756 | 0.157544 | | 0.158135 |
| 0.121408 | 0.119353 | | 0.116705 | 0.11423 | NA | 0.178984 | 0.242855 | | 0.234675 |
| 0.103685 | 0.099327 | | 0.098285 | 0.09564 | NA | 0.114396 | 0.167967 | | 0.161143 |
| 0.119344 | 0.106483 | | 0.104994 | 0.104122 | 0.09333 | 0.147351 | 0.173979 | | 0.171283 |
| 0.121204 | 0.118641 | | 0.116502 | 0.114085 | 0.090449 | 0.175683 | 0.232466 | | 0.224917 |
| 0.11552 | 0.114758 | | 0.113634 | 0.11091 | 0.090653 | 0.151213 | 0.202298 | | 0.195648 |
| 0.113407 | 0.100143 | | 0.097978 | 0.099303 | 0.095423 | 0.102297 | 0.108911 | | 0.110096 |
| 0.110599 | 0.108118 | | 0.110515 | 0.103233 | 0.091241 | 0.10415 | 0.137564 | | 0.133824 |
| 0.118918 | 0.105522 | | 0.101309 | 0.104681 | 0.100935 | 0.104182 | 0.107523 | | 0.108683 |
| 0.101624 | 0.103897 | | 0.09634 | 0.099975 | 0.103605 | 0.096521 | 0.097021 | | 0.098138 |
| 0.113802 | 0.105583 | | 0.098358 | 0.104702 | 0.107164 | 0.121762 | 0.104653 | | 0.106773 |
| 0.123911 | 0.116843 | | 0.109889 | 0.115448 | 0.107734 | 0.123503 | 0.114876 | | 0.115949 |
| NA | 0.104159 | | 0.10111 | 0.104025 | 0.098722 | NA | NA |  | NA |
| 0.101833 | 0.108963 | | 0.112411 | 0.109132 | 0.103248 | NA |  | 0.0918 | NA |
| 0.108523 | 0.096624 | | 0.094897 | 0.097383 | 0.096695 | NA | NA |  | NA |
| 0.104728 | 0.093645 | | 0.092613 | 0.094128 | 0.094203 | NA | NA |  | NA |
| 0.098352 | NA |  | NA | NA | 0.098244 | 0.116369 | NA |  | NA |
| 0.111621 | NA |  | NA | NA | 0.106332 | 0.120641 | 0.099746 | | 0.103111 |
| 0.103677 | NA |  | NA | NA | 0.100589 | 0.12431 | 0.096486 | | 0.099757 |
| NA | NA |  | NA | NA | NA | 0.09978 | NA |  | NA |

| 0.107998 | NA | NA | NA | 0.105285 | | 0.122921 | | 0.098177 | 0.101404 |
| --- | --- | --- | --- | --- | --- | --- | --- | --- | --- |
| 0.121767 | 0.105743 | 0.099996 | 0.104964 | 0.110824 | | 0.12814 | | 0.108088 | 0.111188 |
| 0.113886 | 0.118329 | 0.110641 | 0.111395 | 0.115012 | | 0.117857 | | 0.108787 | 0.109567 |
| 0.112578 | 0.115162 | 0.107637 | 0.107818 | 0.112685 | | 0.115587 | | 0.108674 | 0.10919 |
| 0.107291 | 0.108484 | 0.102696 | 0.10333 | 0.10867 | | 0.110607 | | 0.103718 | 0.104673 |
| 0.113015 | 0.107822 | 0.100496 | 0.103337 | 0.107747 | | 0.112154 | | 0.102959 | 0.103929 |
| 0.123557 | 0.102366 | 0.099292 | 0.100051 | 0.111793 | | 0.108139 | | NA | NA |
| 0.112658 | 0.114683 | 0.106916 | 0.108434 | 0.114528 | | 0.118421 | | 0.10805 | 0.109132 |
| 0.106758 | 0.111154 | 0.103607 | 0.104513 | 0.112046 | | 0.11331 | | 0.101971 | 0.103253 |
| 0.099691 | 0.090099 | 0.092916 | 0.090266 | NA |  | NA |  | NA | NA |
| 0.100814 | 0.090474 | 0.09323 | 0.091029 | NA |  | NA |  | NA | NA |
| NA | NA | 0.093622 | NA | NA |  | NA |  | NA | NA |
| 0.104321 | 0.101782 | 0.117634 | 0.115881 | 0.109667 | | 0.147199 | | 0.119623 | 0.122324 |
| 0.108953 | 0.105032 | 0.101649 | 0.103248 | 0.109603 | | 0.122707 | | 0.106337 | 0.108434 |
| 0.094689 | 0.096851 | NA | 0.093711 | 0.095215 | | 0.096362 | | NA | NA |
| 0.10673 | 0.099843 | 0.09812 | 0.096893 | 0.104672 | | 0.11542 | | 0.100794 | 0.103285 |
| NA | 0.096478 | NA | NA | 0.094204 | | NA |  | NA | NA |
| NA | NA | NA | NA | NA |  | 0.101964 | | NA | NA |
| 0.09911 | 0.094046 | NA | NA | 0.101758 | | 0.112299 | | 0.094678 | 0.096779 |
| 0.094132 | 0.091922 | 0.096193 | 0.093525 | NA |  | NA |  | NA | NA |
| 0.12104 | 0.10862 | 0.108826 | 0.109611 | 0.117687 | | 0.136529 | | 0.113884 | 0.116207 |
| 0.105352 | 0.100768 | 0.098433 | 0.098704 | 0.104676 | | NA |  | 0.096462 | 0.096992 |
| NA | NA | NA | NA | 0.096324 | | 0.110653 | | NA | NA |
| NA | NA | NA | NA | 0.098697 | | 0.105936 | | NA | NA |
| 0.095983 | NA | NA | NA | 0.100522 | | 0.115071 | | 0.097741 | 0.100397 |
| 0.098635 | NA | NA | NA | 0.096028 | | 0.101099 | | 0.102946 | 0.104868 |
| 0.100359 | NA | NA | NA |  | 0.0991 | 0.117611 | | 0.102762 | 0.104947 |
| 0.09944 | NA | NA | NA | 0.100836 | | 0.118743 | | 0.098153 | 0.100663 |
| 0.097257 | NA | NA | NA | 0.097082 | | 0.099149 | | 0.101476 | 0.10214 |
| 0.105987 | 0.105847 | 0.110585 | 0.108691 | 0.110077 | | 0.120755 | | 0.108708 | 0.110308 |
| 0.105644 | 0.104765 | 0.102822 | 0.102293 | 0.108053 | | 0.105424 | | 0.112423 | 0.113256 |
| 0.118997 | 0.104572 | 0.102517 | 0.104613 | 0.113519 | | 0.130597 | | 0.113951 | 0.114703 |
| 0.106915 | 0.096574 | NA | 0.095636 | 0.104296 | | 0.104676 | | 0.10504 | 0.105992 |
| 0.10889 | 0.101358 | NA | 0.100186 | 0.10702 | | 0.098809 | | 0.107271 | 0.107614 |
| NA | NA | 0.097804 | 0.094489 | 0.095527 | |  | 0.116 | 0.098517 | 0.099477 |
| 0.10895 | 0.101134 | 0.100839 | 0.100069 | 0.093145 | | 0.10382 | | 0.116116 | 0.114891 |
| 0.115124 | 0.104934 | 0.104287 | 0.102944 | 0.092327 | | 0.115481 | | 0.131268 | 0.129461 |
| 0.109995 | 0.096917 | NA | NA | 0.108237 | | 0.113323 | | 0.103292 | 0.105947 |
| 0.107375 | 0.101485 | 0.108618 | 0.107528 | 0.107667 | | 0.132798 | | 0.108547 | 0.109826 |

| 0.110861 | NA | NA |  | NA |  | 0.106457 | 0.118484 | 0.110349 | | 0.111808 |
| --- | --- | --- | --- | --- | --- | --- | --- | --- | --- | --- |
| 0.109132 | 0.096817 | NA |  | 0.095399 | | 0.105333 | 0.109788 | 0.110277 | | 0.111885 |
| 0.126435 | 0.119565 | 0.117391 | | 0.117253 | | 0.123561 | 0.116283 | 0.112977 | | 0.113593 |
| 0.100247 | NA | NA |  | NA |  | 0.103358 | 0.102989 | 0.099558 | | 0.102094 |
| 0.117728 | 0.118256 | 0.134826 | | 0.132853 | | 0.121496 | 0.136611 | 0.13619 | | 0.136358 |
| 0.10539 | 0.111469 | NA |  | 0.096523 | | 0.102822 | NA | 0.098515 | | NA |
| 0.114619 | 0.100887 | 0.097457 | | 0.101158 | | 0.104496 | 0.120551 | 0.110279 | | 0.111295 |
| 0.108711 | 0.095103 | NA |  | 0.093621 | | 0.101726 | NA | 0.097748 | | 0.099147 |
| 0.109344 | 0.098578 | 0.095789 | | 0.097906 | | 0.110558 | 0.118167 | 0.103742 | | 0.104989 |
| 0.10354 | 0.096084 | NA |  | 0.09631 | | 0.102771 | 0.105969 | 0.105834 | | 0.106383 |
| 0.104149 | NA | NA |  | NA |  | 0.103835 | 0.107557 | 0.104832 | | 0.106027 |
| 0.095172 | 0.112466 | 0.12124 | | 0.117594 | | NA | NA | NA |  | NA |
| 0.10145 | 0.094773 | NA |  | NA |  | 0.095596 | 0.107955 | 0.106151 | | 0.107153 |
| NA | 0.098177 | 0.096134 | | 0.095641 | | 0.095719 | 0.104309 | 0.097875 | | 0.098814 |
| 0.095112 | 0.098412 | 0.097014 | | 0.097156 | | 0.099459 | 0.109153 | 0.102294 | | 0.103759 |
| 0.121811 | 0.103094 | 0.098894 | | 0.103001 | | 0.109366 | 0.137086 |  | 0.1118 | 0.113982 |
| 0.096273 | 0.095004 | NA |  | 0.094421 | | 0.100563 | 0.104711 | NA |  | NA |
| 0.106026 | 0.095054 | 0.094311 | | 0.09681 | | 0.104437 | 0.124072 | 0.099589 | | 0.10171 |
| 0.093764 | NA | NA |  | NA |  | 0.094937 | NA | 0.092464 | | NA |
| 0.100831 | NA | NA |  | NA |  | 0.099094 | 0.100716 | 0.104958 | | 0.105303 |
| 0.10595 | 0.099921 | NA |  | 0.098293 | | 0.104757 | 0.101198 | 0.109909 | | 0.111839 |
| 0.096631 | 0.097453 | 0.099774 | | 0.095479 | | NA | NA | 0.118287 | | 0.112948 |
| 0.102815 | 0.095963 | NA |  | 0.094631 | | 0.104093 | 0.112317 | 0.099174 | | 0.101367 |
| 0.100113 | 0.091263 | NA |  | NA |  | NA | 0.100129 | 0.117596 | | 0.116231 |
| 0.108216 | 0.09699 | NA |  | 0.096344 | | 0.105599 | 0.099087 | 0.098541 | | 0.100338 |
| 0.102815 | 0.103836 | 0.10451 | | 0.102999 | | 0.09933 | 0.107376 | 0.119924 | | 0.117312 |
| 0.109451 | 0.101906 |  | 0.0994 | 0.101168 | | 0.109906 | 0.108162 | 0.107145 | | 0.108252 |
| 0.097593 | 0.095751 | NA |  | 0.094127 | | 0.096236 | 0.096567 | 0.102104 | | 0.10336 |
| 0.107633 | 0.100068 | 0.095875 | | 0.098996 | | 0.104805 | 0.105975 | 0.100855 | | 0.101046 |
| 0.102411 | 0.097995 | NA |  | 0.096312 | | 0.102426 | NA | 0.101356 | | 0.102008 |
| 0.102704 | 0.097812 | 0.094241 | |  | 0.0945 | 0.092878 | 0.103304 | 0.120165 | | 0.118874 |
| 0.106463 | 0.10992 | 0.113428 | | 0.111272 | | 0.109775 | 0.132811 | 0.106899 | | 0.108733 |
| 0.101106 | NA | NA |  | NA |  | 0.10007 | 0.106348 | 0.100109 | | 0.101563 |
| 0.101025 | 0.104083 | 0.099666 | | 0.10143 | | 0.095892 | 0.102609 | 0.108128 | | 0.108641 |
| NA | NA | 0.101804 | | 0.097982 | | 0.095822 | NA | NA |  | NA |
| 0.105757 | 0.096033 | NA |  | 0.095815 | | 0.105698 | 0.108269 | 0.099985 | | 0.101622 |
| 0.101927 | 0.100779 | 0.098804 | | 0.100548 | | 0.104512 | 0.111797 | 0.109299 | | 0.110105 |
| 0.102996 | NA | NA |  | NA |  | 0.098088 | 0.098684 | 0.109837 | | 0.109916 |

mature_eo mature_ne megakaryomegakaryomemory_b mesangial mesenchy monocyte.

0.110225 0.114089 0.116057 0.112661	0.11154 0.118395 0.107187 0.111865

NA	NA	NA	NA	NA	NA	NA	NA

0.114568 0.113461 0.113259 0.112509 0.119591 0.115815 0.115194 0.112609

0.110044 0.118097 0.108483 0.107618 0.126396 0.114363 0.119285 0.108263

0.102594 0.108495 0.100404 0.102046 0.122449 0.106418 0.118935 0.100749

0.121327 0.126692 0.125016 0.112697 0.122269 0.126771 0.109981 0.119356

0.115286 0.118324 0.112323 0.115491 0.124114 0.113406 0.121484 0.114007

0.102702 0.106231 0.102942 0.107516 0.117178 0.109919 0.120662 0.102517

0.102702 0.106231 0.102942 0.107516 0.117178 0.109919 0.120662 0.102517

0.124369 0.122712 NA	0.106864	0.10336 0.122195 0.109396 0.120667

0.115742 0.117394 0.117622 0.110414 0.111483 0.121953 0.108386 0.114267

0.105966 0.107619 0.106713 0.102109 0.111916 0.109471 0.106383 0.103683

0.10701 0.113279 0.108029 0.106772 0.109284 0.126908	0.10686 0.121028

0.108345 0.113476 0.108161 0.106659 0.113066 0.136271 0.110052	0.12007

NA	NA	NA	NA	NA	NA	0.096947 NA NA	NA	NA	NA	NA	NA	NA	NA

0.0953 NA	NA	NA	0.097233 0.107117 0.093087 0.100211

0.0953 NA	NA	NA	0.097233 0.107117 0.093087 0.100211

0.141144 0.132117 0.152443	0.13602 0.123292 0.100117 0.093543 0.170269

0.1136 0.115596 0.111579 0.109637 0.126303	0.11314 0.119604 0.110709

NA	NA	NA	NA	NA	NA	NA	NA

0.138557 0.133901 0.145922 0.131034 0.119662 0.104666 0.110824 0.136411

NA	NA	NA	NA	NA	NA	NA	NA NA	NA	NA	NA	NA	NA	NA	NA

0.118785 0.118602 NA	NA	NA	NA	0.092355 0.110592

0.129131	0.14881 NA	NA	NA	NA	0.094923 0.131462

0.129281 0.149398 NA	NA	NA	NA	0.094045	0.13339

0.121929 0.139661 NA	NA	NA	NA	NA	0.124587

0.114762 0.112453 0.113647	0.11348 0.119224 0.114652 0.116994 0.110527

0.114762 0.112453 0.113647	0.11348 0.119224 0.114652 0.116994 0.110527

0.131898 0.132974 0.130076 0.131008	0.14193	0.13342 0.137767 0.127752

0.135337 0.135092 0.133361 0.134097	0.14342 0.131886 0.139849 0.131308

0.119989 0.116421 NA	0.102139 NA	0.112518 0.101484 0.113828

0.120443 0.120884 NA	NA	NA	NA	NA	0.117686

NA	NA	NA	NA	NA	NA	NA	NA NA	NA	NA	NA	NA	NA	NA	NA NA	NA	NA	NA	NA	NA	NA	NA

0.099432 0.109678 0.112905 0.108801	0.10483 0.110619 0.104724 0.107506

| 0.093244 | 0.099459 | 0.098031 | 0.09877 | 0.11632 | 0.115196 | 0.110247 | 0.096148 |
| --- | --- | --- | --- | --- | --- | --- | --- |
| 0.093244 | 0.099459 | 0.098031 | 0.09877 | 0.11632 | 0.115196 | 0.110247 | 0.096148 |
| 0.093244 | 0.099459 | 0.098031 | 0.09877 | 0.11632 | 0.115196 | 0.110247 | 0.096148 |
| 0.097604 | 0.095775 | 0.109238 | 0.106791 | 0.110705 | 0.120519 | 0.104133 | 0.105488 |
| 0.097916 | 0.097251 | 0.107889 | 0.104892 | 0.110786 | 0.116541 | 0.103851 | 0.103851 |
| 0.159635 | 0.163417 | 0.182849 | 0.159563 | 0.126946 | 0.110485 | 0.099277 | 0.176154 |
| 0.177288 | 0.184637 | 0.194907 | 0.166538 | 0.134933 | 0.122895 | 0.10594 | 0.190961 |
| 0.19495 | 0.203505 | 0.216854 | 0.181243 | 0.148077 | 0.126189 | 0.108647 | 0.211877 |
| 0.173568 | 0.175971 | 0.201488 | 0.171579 | 0.138403 | 0.121434 | 0.105825 | 0.191866 |
| 0.170983 | 0.178117 | 0.183715 | 0.160305 | 0.135966 | 0.125678 | 0.107677 | 0.18174 |
| 0.177207 | 0.189254 | 0.19558 | 0.163174 | 0.141594 | 0.126624 | 0.107271 | 0.191899 |
| 0.193973 | 0.196098 | 0.209401 | 0.173023 | 0.138954 | 0.118118 | 0.102648 | 0.207742 |
| 0.168842 | 0.185291 | 0.187969 | 0.164365 | 0.134719 | 0.128651 | 0.114556 | 0.178188 |
| 0.176497 | 0.190567 | 0.195049 | 0.168882 | 0.140299 | 0.126199 | 0.10976 | 0.187244 |
| 0.187362 | 0.201207 | 0.207645 | 0.178488 | 0.145316 | 0.129214 | 0.112368 | 0.196524 |
| NA | NA | NA | NA | NA | NA | NA | NA |
| 0.103652 | 0.105379 | 0.101853 | 0.106588 | 0.119303 | 0.107764 | 0.121471 | 0.101141 |
| 0.188604 | 0.199891 | 0.209807 | 0.178082 | 0.146388 | 0.127626 | 0.110968 | 0.198335 |
| 0.19432 | 0.207753 | 0.218939 | 0.185853 | 0.151884 | 0.127827 | 0.112273 | 0.205406 |
| 0.146794 | 0.162088 | 0.160582 | 0.146231 | 0.127183 | 0.125855 | 0.115358 | 0.153114 |
| 0.203387 | 0.214494 | 0.23388 | 0.193614 | 0.153322 | 0.122576 | 0.104988 | 0.219577 |
| NA | NA | NA | NA | NA | NA | 0.098139 | NA |
| NA | NA | NA | NA | NA | NA | NA | NA |
| 0.197966 | 0.210461 | 0.222043 | 0.187865 | 0.150592 | 0.125255 | 0.108382 | 0.209961 |
| 0.14052 | 0.152926 | 0.156413 | 0.146113 | 0.12676 | 0.123719 | 0.111863 | 0.153395 |
| 0.163825 | 0.170963 | 0.183114 | 0.157311 | 0.129551 | 0.115278 | 0.103234 | 0.180121 |
| 0.196823 | 0.210121 | 0.219778 | 0.185708 | 0.149685 | 0.128884 | 0.111606 | 0.208296 |
| 0.18517 | 0.194291 | 0.205208 | 0.176799 | 0.143894 | 0.123012 | 0.107572 | 0.193122 |
| 0.148448 | 0.158901 | 0.159357 | 0.142118 | 0.124965 | 0.118548 | 0.108278 | 0.152661 |
| 0.148448 | 0.158901 | 0.159357 | 0.142118 | 0.124965 | 0.118548 | 0.108278 | 0.152661 |
| 0.20191 | 0.213414 | 0.227786 | 0.19024 | 0.152184 | 0.126084 | 0.10893 | 0.215213 |
| 0.160057 | 0.171287 | 0.17811 | 0.156145 | 0.131735 | 0.117816 | 0.108925 | 0.168645 |
| 0.155139 | 0.165323 | 0.169347 | 0.146327 | 0.128144 | 0.108388 | 0.100264 | 0.172414 |
| 0.158249 | 0.162305 | 0.173107 | 0.154373 | 0.132491 | 0.11591 | 0.106365 | 0.169292 |
| NA | NA | NA | NA | NA | NA | NA | NA |
| 0.107561 | 0.110316 | 0.108392 | 0.104347 | 0.110921 | 0.110476 | 0.106073 | 0.106322 |
| 0.198674 | 0.205179 | 0.219887 | 0.186186 | 0.146422 | 0.120829 | 0.106158 | 0.210595 |
| 0.204974 | 0.217652 | 0.233475 | 0.194233 | 0.154239 | 0.127827 | 0.108537 | 0.219473 |
| 0.147733 | 0.164116 | 0.163696 | 0.150555 | 0.130852 | 0.128266 | 0.119067 | 0.154394 |

| 0.103591 | 0.10652 | | 0.104526 | 0.102738 | 0.107601 | | 0.136258 | 0.105477 | 0.119115 |
| --- | --- | --- | --- | --- | --- | --- | --- | --- | --- |
| 0.106916 | 0.105529 | | 0.106297 | 0.104199 | 0.104748 | | 0.130892 | 0.10206 | 0.118438 |
| 0.108664 |  | 0.1131 | 0.110088 | 0.107159 |  | 0.1127 | 0.135949 | 0.106418 | 0.123824 |
| 0.096344 | 0.100609 | | 0.09611 | 0.096623 | 0.10593 | | 0.127321 | 0.106889 | 0.111177 |
| NA | NA |  | NA | NA | NA |  | NA | NA | NA |
| NA | NA |  | NA | NA | NA |  | NA | NA | NA |
| NA | NA |  | NA | NA | NA |  | NA | NA | NA |
| NA | NA |  | NA | NA | NA |  | NA | NA | NA |
| NA | NA |  | NA | NA | NA |  | 0.10177 | NA | NA |
| NA | NA |  | NA | NA | NA |  | 0.102452 | NA | NA |
| NA | NA |  | NA | NA | NA |  | NA | NA | NA |
| NA | NA |  | NA | NA | NA |  | NA | NA | NA |
| NA | NA |  | NA | NA | NA |  | NA | NA | NA |
| NA | NA |  | NA | NA | NA |  | NA | NA | NA |
| NA | NA |  | NA | NA | NA |  | 0.101298 | NA | NA |
| 0.092117 | NA |  | 0.092527 | NA | NA |  | 0.103352 | NA | NA |
| NA | NA |  | NA | NA | NA |  | NA | NA | NA |
| NA | NA |  | NA | NA | NA |  | 0.095037 | NA | NA |
| 0.094343 | NA |  | 0.096944 | 0.092773 | NA |  | 0.105631 | NA | 0.092502 |
| 0.092496 | NA |  | 0.092292 | NA | NA |  | 0.102879 | NA | NA |
| NA | NA |  | NA | NA | NA |  | NA | NA | NA |
| 0.189466 | 0.205034 | | 0.212851 | 0.179927 | 0.148019 | | 0.13205 | 0.111472 | 0.201881 |
| 0.119026 | 0.092224 | | 0.110784 | 0.11899 | NA |  | NA | 0.089084 | 0.097722 |
| 0.119026 | 0.092224 | | 0.110784 | 0.11899 | NA |  | NA | 0.089084 | 0.097722 |
| 0.101761 | NA |  | 0.095197 | 0.101263 | NA |  | NA | NA | NA |
| 0.101761 | NA |  | 0.095197 | 0.101263 | NA |  | NA | NA | NA |
| 0.116687 | NA |  | 0.104396 | 0.11523 | NA |  | NA | NA | NA |
| 0.130942 | 0.09215 | | 0.117237 | 0.127171 | NA |  | NA | NA | 0.096417 |
| 0.103326 | NA |  | 0.094232 | 0.104415 | NA |  | NA | NA | NA |
| 0.10688 | NA |  | 0.097394 | 0.107124 | NA |  | NA | NA | NA |
| 0.10688 | NA |  | 0.097394 | 0.107124 | NA |  | NA | NA | NA |
| 0.10688 | NA |  | 0.097394 | 0.107124 | NA |  | NA | NA | NA |
| NA | NA |  | NA | NA | NA |  | NA | NA | NA |
| NA | NA |  | NA | NA | NA |  | NA | NA | NA |
| NA | NA |  | NA | NA | NA |  | NA | NA | NA |
| NA | NA |  | NA | NA | NA |  | NA | NA | NA |
| 0.098945 | 0.10126 | | 0.103256 | 0.100527 | 0.098405 | | 0.112486 | 0.107686 | 0.099755 |
| 0.101296 | 0.112585 | | 0.103132 | 0.105913 | 0.120365 | | 0.109754 | 0.117014 | 0.102903 |
| 0.101296 | 0.112585 | | 0.103132 | 0.105913 | 0.120365 | | 0.109754 | 0.117014 | 0.102903 |

| 0.107785 | 0.120389 | 0.107476 | 0.108201 | 0.129946 | 0.116469 | 0.126336 | 0.108086 |
| --- | --- | --- | --- | --- | --- | --- | --- |
| NA | 0.101074 | NA | NA | 0.119114 | NA | 0.118745 | NA |
| 0.110812 | 0.118194 | 0.115393 | 0.110763 | 0.125186 | 0.115673 | 0.115112 | 0.112098 |
| 0.09509 | 0.10436 | 0.096011 | 0.099123 | 0.103779 | 0.103133 | 0.106787 | 0.095137 |
| 0.110483 | 0.11703 | 0.114355 | 0.110538 | 0.110683 | 0.115095 | 0.109927 | 0.114522 |
| 0.115995 | 0.123941 | NA | NA | NA | NA | NA | 0.11564 |
| NA | NA | NA | NA | NA | NA | NA | NA |
| NA | NA | NA | NA | NA | 0.097839 | NA | NA |
| 0.092787 | 0.093178 | NA | 0.09111 | 0.09509 | 0.106385 | 0.102476 | 0.092187 |
| 0.092486 | 0.092236 | 0.090118 | 0.092995 | NA | 0.106878 | 0.10132 | 0.092431 |
| 0.096051 | 0.098297 | 0.095155 | 0.095934 | 0.098219 | 0.108317 | 0.104712 | 0.096444 |
| NA | NA | NA | NA | NA | NA | NA | NA |
| NA | NA | NA | NA | NA | NA | NA | NA |
| NA | NA | NA | NA | NA | NA | NA | NA |
| NA | NA | NA | NA | NA | NA | NA | NA |
| NA | NA | NA | NA | NA | NA | NA | NA |
| NA | NA | NA | NA | NA | NA | NA | NA |
| NA | NA | NA | NA | NA | 0.096052 | NA | NA |
| NA | NA | NA | NA | NA | NA | NA | NA |
| 0.091262 | NA | 0.101499 | NA | NA | 0.10876 | NA | NA |
| NA | NA | NA | NA | NA | NA | NA | NA |
| NA | NA | NA | NA | NA | 0.098694 | NA | NA |
| NA | NA | NA | NA | NA | 0.098791 | NA | NA |
| NA | NA | NA | NA | NA | 0.097525 | NA | NA |
| NA | NA | NA | NA | NA | NA | NA | NA |
| 0.091899 | NA | 0.099596 | 0.09272 | NA | 0.108754 | NA | NA |
| NA | NA | NA | NA | NA | NA | NA | NA |
| NA | NA | NA | NA | NA | NA | NA | NA |
| NA | NA | NA | NA | NA | NA | NA | NA |
| NA | NA | NA | NA | NA | NA | NA | NA |
| 0.095573 | NA | 0.097164 | 0.096775 | NA | 0.115837 | 0.092558 | 0.093982 |
| NA | NA | NA | NA | NA | NA | NA | NA |
| NA | NA | NA | NA | NA | 0.103813 | NA | NA |
| NA | NA | NA | NA | NA | 0.099826 | NA | NA |
| NA | NA | NA | NA | NA | NA | NA | NA |
| 0.094772 | NA | NA | NA | NA | 0.099755 | NA | NA |
| NA | NA | NA | NA | NA | NA | NA | NA |
| 0.09498 | NA | NA | NA | NA | NA | NA | NA |
| NA | NA | NA | NA | NA | NA | NA | NA |

| NA | NA | NA |  | NA | NA |  | 0.100372 | NA | NA |  |
| --- | --- | --- | --- | --- | --- | --- | --- | --- | --- | --- |
| 0.093173 | NA | 0.104022 | | 0.093373 | NA |  | 0.10955 | NA | NA |  |
| NA | NA | NA |  | NA | NA |  | NA | NA | NA |  |
| NA | NA | NA |  | NA | NA |  | NA | NA | NA |  |
| NA | NA | NA |  | NA | NA |  | NA | NA | NA |  |
| 0.097679 | NA | 0.095693 | | NA | NA |  | 0.099655 | NA | NA |  |
| NA | NA | NA |  | NA | NA |  | NA | NA | NA |  |
| NA | NA | NA |  | NA | NA |  | NA | NA | NA |  |
| NA | NA | NA |  | NA | NA |  | NA | NA | NA |  |
| 0.09325 | NA | NA |  | NA | NA |  | 0.096173 | NA | NA |  |
| 0.131006 | 0.127169 | 0.141947 | | 0.126539 | 0.109004 | | 0.098726 | 0.093598 | 0.139164 | |
| 0.097997 | 0.095713 | 0.095349 | | 0.117685 | NA |  | NA | 0.117367 | 0.096024 | |
| NA | NA | 0.094765 | | NA | NA |  | NA | NA | 0.101914 | |
| 0.112854 | 0.106459 | 0.117292 | | 0.109056 | 0.104208 | | NA | 0.094987 | 0.126171 | |
| 0.137898 | 0.129044 | 0.138571 | | 0.121716 | 0.137809 | | 0.122604 | 0.115848 | 0.156965 | |
| 0.10768 | 0.105038 | 0.106531 | | 0.103986 | 0.108591 | | 0.098557 | 0.106428 | 0.112973 | |
| 0.097761 | 0.099682 | NA |  | NA | 0.112764 | | NA | 0.110796 | 0.098999 | |
| 0.098061 | NA | 0.096942 | | 0.094856 | NA |  | NA | 0.095772 | 0.103939 | |
| 0.114671 | 0.111752 | 0.116191 | | 0.106802 | 0.113394 | | 0.098367 | 0.100448 | 0.131422 | |
| 0.104605 | 0.103371 | 0.104944 | | 0.097121 | 0.103062 | | NA | 0.095669 | 0.118894 | |
| 0.100395 | 0.101254 |  | 0.1008 | 0.100111 | 0.106204 | | 0.138537 | 0.104932 | 0.116615 | |
| 0.101335 | 0.104882 | 0.099538 | | 0.100107 | 0.108486 | | 0.132054 | 0.104835 | 0.115002 | |
| 0.105712 | 0.109479 | 0.104268 | | 0.10257 | 0.109603 | | 0.132061 | 0.102661 | 0.120657 | |
| 0.094771 | 0.096227 | NA |  | 0.095688 | 0.104244 | | 0.124251 | 0.104018 | 0.108201 | |
| NA | NA | 0.099138 | | 0.100079 | 0.097149 | | 0.104891 | 0.098694 | 0.099905 | |
| 0.100743 | 0.103634 | 0.102686 | | 0.106457 | 0.10456 | | 0.103177 | 0.104872 | 0.100377 | |
| 0.095758 | 0.101174 | 0.099767 | | 0.101125 | 0.108756 | | 0.106089 | 0.108088 | 0.103175 | |
| 0.100793 | 0.102406 | 0.104148 | | 0.10345 | 0.116685 | | 0.109364 | 0.109326 | 0.106616 | |
| 0.100793 | 0.102406 | 0.104148 | | 0.10345 | 0.116685 | | 0.109364 | 0.109326 | 0.106616 | |
| NA | NA | 0.098921 | | 0.097474 | 0.106829 | | 0.102832 | 0.102599 | 0.097218 | |
| 0.094159 | 0.088732 | 0.106869 | | 0.10311 | 0.103502 | | 0.112954 | 0.101073 | 0.101786 | |
| NA | NA | 0.099115 | | 0.099607 | 0.099965 | | 0.108406 | 0.10091 | 0.099837 | |
| NA | NA | 0.099115 | | 0.099607 | 0.099965 | | 0.108406 | 0.10091 | 0.099837 | |
| NA | NA | 0.099115 | | 0.099607 | 0.099965 | | 0.108406 | 0.10091 | 0.099837 | |
| 0.093651 | 0.090439 | 0.108648 | | 0.105558 | 0.099089 | | 0.116229 | 0.099026 |  | 0.1059 |
| 0.102657 | 0.106473 | 0.115834 | | 0.107692 |  | 0.1073 | 0.119809 | 0.106284 | 0.109058 | |
| NA | NA | NA |  | 0.095278 | 0.103326 | | 0.100558 | 0.102004 | 0.095937 | |
| NA | NA | NA |  | NA | NA |  | 0.094341 | NA | NA |  |
| 0.092839 | NA | 0.093578 | | NA | NA |  | NA | 0.090508 | 0.094784 | |

| NA | NA |  | NA | NA | NA |  | NA | NA | NA |
| --- | --- | --- | --- | --- | --- | --- | --- | --- | --- |
| NA | NA |  | NA | NA | NA |  | NA | NA | NA |
| NA | NA |  | NA | NA | NA |  | NA | NA | NA |
| NA | NA |  | NA | NA | NA |  | NA | NA | NA |
| 0.111661 |  | 0.1201 | 0.112064 | 0.107759 | 0.11593 | | 0.120547 | 0.109289 | 0.111993 |
| 0.112623 | 0.114554 | | 0.111639 | 0.10973 | 0.107787 | | 0.116883 | 0.107738 | 0.111375 |
| 0.101481 | 0.107508 | | 0.104905 | 0.101829 | 0.105648 | | 0.11158 | 0.110734 | 0.102788 |
| 0.118665 | 0.122074 | | 0.117738 | 0.114457 | 0.113008 | | 0.121034 | 0.109907 | 0.116802 |
| 0.101775 | 0.112183 | | 0.100922 | 0.100166 | 0.105634 | | 0.108782 | 0.10375 | 0.105521 |
| 0.106951 | 0.114286 | | 0.105659 | 0.10244 | 0.106764 | | 0.110567 | 0.104296 | 0.109062 |
| 0.113409 | 0.115265 | | 0.112847 | 0.111651 | 0.122039 | | 0.115409 | 0.115151 | 0.111528 |
| 0.110931 | 0.123972 | | 0.118063 | 0.113239 | 0.11622 | | 0.135315 | 0.118929 | 0.114063 |
| 0.118183 | 0.136808 | | 0.126767 | 0.121997 | 0.117548 | | 0.151872 | 0.128547 | 0.12332 |
| 0.101356 | 0.101325 | | 0.101499 | 0.099804 | 0.10213 | | 0.103757 | 0.101326 | 0.099121 |
| NA | NA |  | NA | NA | NA |  | 0.100314 | NA | NA |
| NA | NA |  | NA | NA | NA |  | NA | NA | NA |
| 0.109785 | 0.111454 | | 0.106886 | 0.106814 | 0.123461 | | 0.105838 | 0.11612 | 0.105575 |
| NA | NA |  | NA | NA | NA |  | NA | NA | NA |
| 0.111288 | 0.11232 | | 0.111168 | 0.104054 | 0.112526 | | 0.115531 | 0.103008 | 0.108546 |
| NA | NA |  | NA | NA | NA |  | NA | NA | NA |
| NA | NA |  | NA | NA | NA |  | NA | NA | NA |
| 0.113029 | 0.117165 | | 0.112952 | 0.109441 |  | 0.1158 | 0.115122 | 0.111152 | 0.111278 |
| 0.116735 | 0.117721 | | 0.116351 | 0.113953 | 0.121918 | | 0.119475 | 0.119205 | 0.114763 |
| 0.116735 | 0.117721 | | 0.116351 | 0.113953 | 0.121918 | | 0.119475 | 0.119205 | 0.114763 |
| 0.116735 | 0.117721 | | 0.116351 | 0.113953 | 0.121918 | | 0.119475 | 0.119205 | 0.114763 |
| 0.116735 | 0.117721 | | 0.116351 | 0.113953 | 0.121918 | | 0.119475 | 0.119205 | 0.114763 |
| 0.118585 | 0.118561 | | 0.121301 | 0.114576 | 0.107163 | | 0.114389 | 0.108076 | 0.118944 |
| 0.118585 | 0.118561 | | 0.121301 | 0.114576 | 0.107163 | | 0.114389 | 0.108076 | 0.118944 |
| 0.118585 | 0.118561 | | 0.121301 | 0.114576 | 0.107163 | | 0.114389 | 0.108076 | 0.118944 |
| 0.118585 | 0.118561 | | 0.121301 | 0.114576 | 0.107163 | | 0.114389 | 0.108076 | 0.118944 |
| 0.119178 | 0.110067 | | 0.115449 | 0.112658 | 0.106229 | | 0.116266 | 0.112677 | 0.113152 |
| 0.119178 | 0.110067 | | 0.115449 | 0.112658 | 0.106229 | | 0.116266 | 0.112677 | 0.113152 |
| 0.115951 | 0.108845 | | 0.112321 | 0.110089 | 0.103201 | | 0.10657 | 0.110249 | 0.111604 |
| 0.115951 | 0.108845 | | 0.112321 | 0.110089 | 0.103201 | | 0.10657 | 0.110249 | 0.111604 |
| 0.121845 | 0.123719 | | 0.122256 | 0.121049 | 0.133451 | | 0.124697 | 0.126466 | 0.119515 |
| 0.113222 | 0.117878 | | 0.114149 | 0.104927 | 0.118901 | | 0.120783 | 0.106976 | 0.110409 |
| 0.113222 | 0.117878 | | 0.114149 | 0.104927 | 0.118901 | | 0.120783 | 0.106976 | 0.110409 |
| 0.114884 | 0.115307 | | 0.114475 | 0.10626 | 0.111244 | | 0.114263 | 0.100973 | 0.111423 |
| 0.114884 | 0.115307 | | 0.114475 | 0.10626 | 0.111244 | | 0.114263 | 0.100973 | 0.111423 |

| 0.111111 | 0.116466 | 0.113833 | 0.103952 | 0.118997 | 0.114478 | 0.10648 | | 0.108918 |
| --- | --- | --- | --- | --- | --- | --- | --- | --- |
| 0.11689 | 0.122418 | 0.118743 | 0.109782 | 0.120317 | 0.127301 | 0.109014 | | 0.114934 |
| 0.11063 | 0.113384 | 0.112379 | 0.105245 | 0.115882 | 0.117382 | 0.108483 | | 0.108598 |
| 0.10493 | 0.103613 | 0.103826 | 0.101368 | 0.102447 | 0.101545 | 0.101921 | | 0.102317 |
| 0.114374 | 0.117046 | 0.116628 | 0.112461 | 0.105278 | 0.121052 | 0.101896 | | 0.121998 |
| 0.098606 | 0.099337 | 0.098633 | 0.099853 | 0.104496 | 0.13667 | 0.106394 | | 0.114355 |
| 0.114449 | 0.119554 | 0.117623 | 0.113905 | 0.107965 | 0.121908 | 0.106091 | | 0.12126 |
| 0.111374 | 0.122804 | 0.120149 | 0.110158 | 0.104499 | 0.167799 | 0.118544 | | 0.112405 |
| 0.11292 | 0.12505 | 0.122439 | 0.111399 | 0.106294 | 0.173995 | 0.120114 | | 0.113973 |
| NA | 0.099421 | NA | NA | 0.095145 | 0.112729 | 0.109061 | | NA |
| 0.112866 | 0.123891 | 0.122691 | 0.112252 | 0.10553 | 0.176226 | 0.120515 | | 0.114366 |
| 0.106745 | 0.110423 | 0.110782 | 0.103742 | 0.099227 | 0.133659 | 0.11302 | | 0.106028 |
| NA | NA | NA | NA | 0.11313 | NA | 0.099756 | | NA |
| 0.099775 | 0.106228 | 0.107704 | 0.104348 | 0.108476 | 0.107582 | 0.101635 | | 0.104345 |
| NA | NA | NA | NA | 0.114706 | NA | 0.098966 | | NA |
| NA | NA | NA | NA | NA | NA | 0.09438 | | NA |
| NA | NA | NA | NA | NA | NA | NA |  | NA |
| 0.096328 | NA | 0.095744 | 0.095956 | 0.094224 | 0.096865 | 0.095238 | | 0.095965 |
| NA | NA | NA | NA | NA | NA | NA |  | NA |
| NA | NA | NA | NA | NA | NA | NA |  | NA |
| NA | NA | NA | NA | NA | NA | 0.09295 | | NA |
| NA | NA | NA | NA | NA | NA | 0.096835 | | NA |
| NA | NA | NA | NA | NA | NA | 0.094552 | | NA |
| 0.098454 | NA | 0.098921 | 0.096076 | NA | NA | 0.092509 | | 0.097301 |
| 0.10418 | 0.099911 | 0.108848 | 0.103486 | 0.097825 | NA | 0.093321 | | 0.106852 |
| NA | NA | NA | NA | NA | NA | NA |  | NA |
| NA | NA | NA | NA | NA | NA | NA |  | NA |
| NA | NA | NA | NA | NA | NA | NA |  | NA |
| 0.120585 | 0.12001 | 0.120481 | 0.119574 | 0.128548 | 0.120087 | 0.123527 | | 0.117828 |
| 0.103243 | 0.102261 | 0.116757 | 0.114249 | 0.104899 | 0.119045 | 0.105074 | | 0.112674 |
| 0.103243 | 0.102261 | 0.116757 | 0.114249 | 0.104899 | 0.119045 | 0.105074 | | 0.112674 |
| NA | NA | NA | NA | NA | 0.119688 | 0.102878 | | NA |
| NA | 0.095117 | NA | 0.093236 | 0.100059 | 0.104117 | 0.102106 | | NA |
| NA | NA | NA | NA | NA | 0.102481 | 0.101661 | | NA |
| NA | NA | 0.093871 | 0.095809 | 0.094933 | 0.105552 |  | 0.0999 | 0.094874 |
| 0.124879 | 0.142367 | 0.134564 | 0.125114 | 0.118083 | 0.115364 | 0.107742 | | 0.131005 |
| 0.091097 | 0.092147 | NA | 0.091749 | 0.095581 | 0.105528 | 0.10259 | | 0.092116 |
| NA | NA | NA | NA | NA | NA | 0.102767 | | NA |
| NA | 0.093475 | NA | 0.094587 | 0.096785 | 0.108284 | 0.102975 | | 0.093936 |

| 0.09162 | NA | 0.092514 | 0.093511 | | 0.091811 | 0.102034 | | 0.099041 | 0.094449 |
| --- | --- | --- | --- | --- | --- | --- | --- | --- | --- |
| NA | NA | NA | NA |  | NA | 0.098496 | | 0.091814 | NA |
| NA | NA | NA | NA |  | NA | 0.09909 | | 0.093191 | NA |
| NA | NA | NA | NA |  | NA | 0.100979 | | 0.096523 | NA |
| NA | NA | NA | NA |  | 0.095776 | 0.100879 | | 0.100931 | NA |
| 0.113615 | 0.113917 | 0.111923 | 0.110885 | | 0.106298 | 0.11603 | | 0.106259 | 0.112307 |
| NA | NA | NA | NA |  | NA | NA |  | NA | NA |
| NA | NA | NA | NA |  | NA | NA |  | NA | NA |
| NA | NA | NA | NA |  | NA | NA |  | NA | NA |
| NA | NA | NA | NA |  | NA | NA |  | NA | NA |
| 0.107288 | 0.115207 | 0.10632 | 0.106432 | | 0.144754 | 0.150184 | | 0.109162 | 0.10824 |
| 0.099902 | 0.103227 | NA | 0.097987 | | 0.118767 | 0.123125 | | 0.107517 | 0.099601 |
| 0.111931 | 0.114928 | 0.108903 | 0.109946 | | 0.114032 | 0.117154 | | 0.106734 | 0.114759 |
| NA | NA | NA | NA |  | NA | NA |  | NA | NA |
| NA | 0.10393 | NA | NA |  | 0.122915 | 0.123403 | | 0.106754 | NA |
| 0.100129 | 0.097456 | 0.095818 | 0.098089 | | 0.110548 | 0.117249 | | 0.098674 | 0.099807 |
| 0.098385 | 0.098045 | 0.097581 | 0.099581 | | 0.101872 | 0.112839 | | 0.106523 | 0.099722 |
| NA | 0.105219 | NA | 0.095554 | | 0.110619 | 0.117596 | | 0.105904 | NA |
| NA | NA | NA | NA |  | NA | NA |  | NA | NA |
| 0.097005 | 0.107285 | 0.106373 | 0.102384 | | 0.108969 | 0.104095 | | 0.106871 | 0.101162 |
| 0.103006 | 0.108077 | 0.113463 | 0.107159 | | 0.109661 | 0.111682 | | 0.10584 | 0.107024 |
| 0.094766 | 0.096036 | 0.098636 | 0.09915 | | 0.096725 | 0.107071 | | 0.099997 | 0.098613 |
| 0.094131 | 0.092994 | 0.096402 | 0.097288 | | 0.094441 | 0.109065 | | 0.09913 | 0.097561 |
| 0.127908 | 0.137603 | 0.126226 | 0.120924 | | 0.116385 | 0.11761 | | 0.115521 | 0.126383 |
| 0.104096 | 0.107481 | 0.109295 | 0.104828 | | 0.105945 | 0.106246 | | 0.105279 | 0.107766 |
| 0.094544 | 0.094541 | NA |  | 0.0962 | 0.098714 | 0.104561 | | 0.102357 | 0.096876 |
| 0.10127 | 0.10703 | 0.109246 | 0.106433 | | 0.111095 | 0.108067 | | 0.10227 | 0.104915 |
| 0.095145 | 0.099979 | 0.098859 | 0.097988 | | 0.104818 | 0.105526 | | 0.103475 | 0.098166 |
| 0.113172 | 0.116921 | 0.112226 | 0.108633 | | 0.108179 | 0.113111 | | 0.107737 | 0.112933 |
| 0.098339 | 0.104755 | 0.098599 | 0.097538 | | 0.102091 | 0.105144 | | 0.104515 | 0.09783 |
| 0.118585 | 0.11898 | 0.117898 | 0.111404 | | 0.107342 | 0.129949 | | 0.112039 | 0.114657 |
| 0.106004 | 0.108863 | 0.105172 | 0.102487 | | 0.105339 | 0.109948 | | 0.106929 | 0.103502 |
| 0.116252 | 0.117596 | 0.115533 | 0.112073 | | 0.108512 | 0.122259 | | 0.110727 | 0.113961 |
| 0.103418 | 0.107775 | 0.10705 | 0.102948 | | 0.105715 | 0.111022 | | 0.106216 | 0.103774 |
| 0.108488 | 0.107128 | 0.108614 | 0.104108 | | 0.101209 |  | 0.1153 | 0.104198 | 0.105835 |
| 0.100625 | 0.101263 | NA | NA |  | 0.101407 | 0.104021 | | 0.099844 | 0.09784 |
| NA | NA | NA | NA |  | NA | NA |  | 0.102433 | NA |
| NA | NA | NA | NA |  | NA | NA |  | 0.102433 | NA |
| NA | NA | NA | NA |  | NA | NA |  | 0.102433 | NA |

| 0.138148 | 0.130864 | | 0.145019 | 0.131324 | 0.118788 | NA | 0.112558 | 0.135433 | |
| --- | --- | --- | --- | --- | --- | --- | --- | --- | --- |
| 0.144571 | 0.136704 | | 0.152476 | 0.135825 | 0.120594 | 0.09845 | 0.112455 | 0.141585 | |
| 0.125698 | 0.124047 | | 0.136259 | 0.122343 | 0.113466 | 0.099122 | 0.108898 | 0.12628 | |
| 0.09361 | 0.092334 | | 0.095298 | 0.096866 | 0.095611 | 0.108581 | 0.099313 | 0.096447 | |
| NA | NA |  | NA | NA | 0.097227 | 0.102284 | 0.103437 | NA |  |
| NA | NA |  | NA | NA | NA | 0.117508 | 0.100215 | NA |  |
| NA | NA |  | NA | NA | NA | 0.112139 | 0.099201 | NA |  |
| 0.099257 | 0.096133 | | 0.095176 | 0.096496 | NA | 0.10516 | 0.099674 | 0.098724 | |
| 0.10243 | 0.105932 | | 0.107282 | 0.104764 | 0.103971 | 0.108061 | 0.106217 | 0.105211 | |
| 0.101735 | 0.105371 | | 0.105698 | 0.10427 | 0.104709 | 0.113171 | 0.105842 | 0.102774 | |
| 0.103189 | 0.107859 | | 0.105852 | 0.105619 | 0.102525 | 0.116429 | 0.10715 | 0.104091 | |
| 0.103189 | 0.107859 | | 0.105852 | 0.105619 | 0.102525 | 0.116429 | 0.10715 | 0.104091 | |
| 0.105876 | 0.109476 | | 0.107436 | 0.10643 | 0.103237 | 0.114438 | 0.10827 | 0.106592 | |
| 0.098482 | 0.104211 | | 0.096663 | 0.09787 | 0.108658 | 0.124331 | 0.104079 | 0.111833 | |
| 0.098482 | 0.104211 | | 0.096663 | 0.09787 | 0.108658 | 0.124331 | 0.104079 | 0.111833 | |
| 0.09491 | NA |  | 0.092475 | 0.090983 | NA | NA | NA | 0.092064 | |
| NA | NA |  | NA | NA | NA | NA | NA | NA |  |
| 0.092527 | NA |  | NA | NA | NA | NA | NA | NA |  |
| NA | NA |  | 0.103077 | 0.101058 | 0.10107 | 0.109361 | 0.100389 | 0.100448 | |
| 0.151942 | 0.161445 | | 0.164011 | 0.14545 | 0.124503 | 0.116428 | 0.108353 | 0.158175 | |
| 0.209506 | 0.195375 | | 0.218242 | 0.168111 | 0.146678 | NA | 0.090906 | 0.228922 | |
| 0.136864 | 0.12653 | | 0.147404 | 0.129641 | 0.109801 | NA | 0.089233 | 0.159497 | |
| 0.155551 | 0.154589 | | 0.16897 | 0.144122 | 0.121927 | 0.106836 | 0.096809 | 0.16842 | |
| 0.199895 | 0.187703 | | 0.205098 | 0.160118 | 0.13899 | 0.097398 | 0.092877 | 0.21899 | |
| 0.174212 | 0.165272 | | 0.183846 | 0.150078 | 0.125394 | 0.094104 | 0.093078 |  | 0.1911 |
| 0.102479 | 0.104306 | | 0.106463 | 0.103178 | 0.093821 | 0.106044 | 0.100326 | 0.106929 | |
| 0.111598 | 0.105647 | | 0.115246 | 0.105869 | 0.105071 | NA | 0.091696 | 0.129208 | |
| 0.102754 | 0.107257 | | 0.108613 | 0.10487 | 0.096058 | 0.114303 | 0.103478 | 0.106248 | |
| 0.103379 | 0.101654 | | 0.101117 | 0.098271 | 0.103673 | 0.097621 | 0.103708 | 0.099821 | |
| 0.111927 | 0.117663 | | 0.116054 | 0.107788 | 0.122144 | 0.118827 | 0.108504 | 0.111718 | |
| 0.120515 | 0.120103 | | 0.120349 | 0.110924 | 0.114033 | 0.12409 | 0.105699 | 0.118339 | |
| 0.093546 | NA |  | NA | NA | 0.114574 | NA | 0.100647 | NA |  |
| NA | NA |  | NA | NA | NA | NA | NA | NA |  |
| NA | NA |  | NA | NA | NA | 0.115902 | 0.101732 | NA |  |
| NA | NA |  | NA | NA | NA | 0.114426 | 0.097975 | NA |  |
| 0.095155 | 0.10745 | | 0.099366 | 0.098782 | 0.110171 | 0.111741 | 0.110378 | 0.098366 | |
| 0.103397 | 0.113092 | | 0.110196 | 0.107044 | 0.113811 | 0.12514 | 0.114048 | 0.107127 | |
| 0.101653 |  | 0.1147 | 0.107968 | 0.104061 | 0.114421 | 0.117225 | 0.112768 | 0.104703 | |
| NA | NA |  | NA | NA | 0.101482 | NA | 0.103661 | NA |  |

| 0.10238 | 0.112753 | | 0.109591 | 0.105618 | | 0.114319 | | 0.124059 | 0.113522 | 0.105969 |
| --- | --- | --- | --- | --- | --- | --- | --- | --- | --- | --- |
| 0.115989 | 0.121417 | | 0.117882 | 0.111246 | | 0.115164 | | 0.127922 | 0.11143 | 0.114899 |
| 0.111167 |  | 0.1134 | 0.109186 | 0.109499 | | 0.120742 | | 0.110607 | 0.116226 | 0.110189 |
| 0.11137 | 0.113701 | | 0.109292 | 0.108986 | | 0.119815 | | 0.109394 | 0.115749 | 0.109854 |
| 0.106936 | 0.10955 | | 0.104351 | 0.104362 | | 0.113558 | | 0.104739 | 0.110844 | 0.105397 |
| 0.108264 | 0.107965 | | 0.108083 | 0.10339 | | 0.108662 | | 0.110098 | 0.102589 | 0.105346 |
| 0.096415 | 0.108695 | | 0.09683 | 0.096242 | | 0.100054 | | 0.12569 | 0.108493 | 0.096197 |
| 0.110847 | 0.115624 | | 0.109524 | 0.108953 | | 0.121327 | | 0.111462 | 0.115684 | 0.110125 |
| 0.105697 | 0.110293 | | 0.104266 | 0.105189 | | 0.117227 | | 0.10562 | 0.114062 | 0.104642 |
| NA | NA |  | NA | NA |  | NA |  | NA | NA | NA |
| NA | NA |  | NA | NA |  | NA |  | 0.097654 | NA | NA |
| NA | NA |  | NA | NA |  | NA |  | NA | NA | NA |
| 0.126978 | 0.135673 | | 0.13101 | 0.123029 | | 0.122825 | | 0.126017 | 0.117326 | 0.125257 |
| 0.110667 | 0.116212 | | 0.113215 |  | 0.1115 | 0.116934 | | 0.115922 | 0.117343 | 0.111464 |
| 0.096554 | 0.098108 | | 0.096892 | NA |  | 0.103044 | | NA | 0.101251 | 0.095054 |
| 0.105111 | 0.110901 | | 0.110963 | 0.111159 | | 0.120151 | | 0.111373 | 0.117386 | 0.104752 |
| 0.096118 | 0.096768 | | 0.097162 | NA |  | 0.102256 | | NA | 0.100268 | 0.094932 |
| 0.093893 | 0.096034 | | 0.097418 | 0.098068 | | 0.108628 | | NA | 0.106979 | NA |
| 0.098337 | 0.105841 | | 0.102547 | 0.100934 | | 0.105238 | | 0.107232 | 0.105912 | 0.100102 |
| NA | NA |  | NA | NA |  | NA |  | NA | NA | NA |
| 0.120256 | 0.129434 | | 0.121671 | 0.118803 | | 0.118937 | | 0.127835 | 0.122602 | 0.118946 |
| 0.096338 | 0.099249 | | 0.096686 | 0.09902 | | 0.097292 | | 0.099296 | 0.107064 | 0.098318 |
| NA | 0.096985 | | NA | NA |  | 0.104112 | | 0.097691 | 0.110316 | NA |
| NA | 0.096962 | | NA | NA |  | 0.106896 | | NA | 0.111013 | NA |
| 0.102376 | 0.108981 | | 0.107599 | 0.105734 | | 0.110618 | | 0.107849 | 0.107758 | 0.102944 |
| 0.101574 | 0.102359 | | 0.112614 | 0.10773 | | 0.102522 | | 0.109777 | 0.102752 | 0.106239 |
| 0.10525 | 0.110085 | | 0.111282 | 0.107421 | | 0.106813 | | 0.111022 | 0.104434 | 0.107112 |
| 0.102564 | 0.113247 | | 0.106433 | 0.104181 | | 0.108797 | | 0.108071 | 0.107934 | 0.10345 |
| 0.09738 | 0.098076 | | 0.106161 | 0.104297 | | 0.103414 | | 0.10165 | 0.107448 | 0.105316 |
| 0.113731 | 0.118688 | | 0.113749 | 0.112422 | | 0.114524 | | 0.110582 | 0.117413 | 0.11225 |
| 0.107803 | 0.106403 | | 0.119487 | 0.116923 | | 0.110464 | | 0.109356 | 0.116537 | 0.11681 |
| 0.118324 | 0.12575 | | 0.118488 | 0.116376 | | 0.119832 | | 0.12371 | 0.115988 | 0.11761 |
| 0.107061 | 0.107762 | | 0.106659 | 0.10786 | |  | 0.1049 | 0.107012 | 0.106922 | 0.106097 |
| 0.107831 | 0.104954 | | 0.108886 | 0.108798 | | 0.105801 | | 0.105485 | 0.110714 | 0.106576 |
| 0.097824 | 0.107983 | | 0.100527 | 0.102533 | | 0.109205 | | 0.102076 | 0.105566 | 0.103292 |
| 0.107852 | 0.108952 | | 0.115927 | 0.110527 | | 0.09644 | | 0.103394 | 0.096155 | 0.113032 |
| 0.122546 | 0.12424 | | 0.127581 | 0.119068 | | 0.102445 | | 0.106181 | 0.096218 | 0.12623 |
| 0.104837 | 0.109697 | | 0.111649 | 0.109126 | | 0.112355 | | 0.113778 | 0.113294 | 0.109253 |
| 0.114746 | 0.119471 | | 0.111506 | 0.110808 | | 0.110417 | | 0.113848 | 0.10772 | 0.111235 |

| 0.11545 | 0.120565 | 0.117377 | 0.112594 | 0.112339 | | 0.115229 | 0.112707 | 0.113692 |
| --- | --- | --- | --- | --- | --- | --- | --- | --- |
| 0.110624 | 0.112901 | 0.117912 | 0.11245 | 0.106589 | | 0.112223 | 0.107012 | 0.113082 |
| 0.115657 | 0.116544 | 0.112763 | 0.114284 | 0.112091 | | 0.116273 | 0.119773 | 0.114301 |
| 0.103271 | 0.107011 | 0.105119 | 0.104747 | 0.107219 | | 0.103528 | 0.110382 | 0.103024 |
| 0.140656 | 0.139779 | 0.130454 | 0.129132 | 0.109981 | | 0.120438 | 0.128256 | 0.134815 |
| NA | NA | NA | 0.101743 | NA |  | NA | 0.123448 | NA |
| 0.112001 | 0.116602 | 0.116733 | 0.109859 | 0.108512 | | 0.11668 | 0.104337 | 0.113923 |
| 0.101561 | 0.109437 | 0.102988 | 0.100532 | 0.10534 | | NA | 0.104698 | 0.102251 |
| 0.107011 | 0.115782 | 0.110726 | 0.107943 | 0.110817 | | 0.115215 | 0.116096 | 0.109052 |
| 0.106807 | 0.107671 | 0.108846 | 0.10738 | 0.103112 | | 0.103683 | 0.105844 | 0.106229 |
| 0.102327 | 0.106748 | 0.110327 | 0.108001 | 0.10557 | | 0.114296 | 0.109544 | 0.109752 |
| NA | NA | NA | NA | NA |  | NA | NA | NA |
| 0.106194 | 0.112115 | 0.110338 | 0.105923 | 0.105154 | | 0.098903 | 0.105895 | 0.109113 |
| 0.095379 | 0.098008 | 0.106143 | 0.101991 | 0.10723 | | 0.105938 | 0.103862 | 0.101446 |
| 0.101793 | 0.104552 | 0.111827 | 0.110186 | 0.114994 | | 0.106383 | 0.110636 | 0.106993 |
| 0.117563 | 0.127596 | 0.122605 | 0.114407 | 0.117973 | | 0.131481 | 0.112752 | 0.118106 |
| 0.095234 | 0.099332 | 0.097744 | 0.097877 | 0.10412 | | 0.103742 | 0.104471 | 0.096705 |
| 0.10732 | 0.11677 | 0.110567 | 0.104974 | 0.111862 | | 0.114035 | 0.110238 | 0.106682 |
| NA | NA | NA | 0.095905 | 0.096855 | | NA | 0.102185 | NA |
| 0.103888 | 0.105583 | 0.107131 | 0.105139 | 0.099704 | | 0.097394 | 0.105197 | 0.107414 |
| 0.109109 | 0.104565 | 0.115958 | 0.114473 | 0.11067 | | 0.109765 | 0.112413 | 0.11321 |
| 0.102023 | 0.094635 | 0.106564 | 0.108265 | 0.093223 | | NA | 0.093578 | 0.115228 |
| 0.102528 | 0.109013 | 0.107012 | 0.105028 | 0.106499 | | 0.107295 | 0.111418 | 0.104176 |
| 0.105443 | 0.107314 | 0.122178 | 0.113634 | 0.096332 | | 0.098024 | 0.095325 | 0.117359 |
| 0.101606 | 0.109427 | 0.108289 | 0.104829 | 0.108118 | | 0.114885 | 0.108793 | 0.104054 |
| 0.112897 | 0.110652 | 0.115458 | 0.110796 | 0.103097 | | 0.097996 | 0.102791 | 0.118254 |
| 0.109105 | 0.110314 | 0.109585 | 0.109386 | 0.102122 | | 0.111797 | 0.107237 | 0.107778 |
| 0.096058 | 0.093746 | 0.112474 | 0.105848 |  | 0.1003 | 0.110653 | 0.102803 | 0.104261 |
| 0.104141 | 0.105924 | 0.10201 | 0.104124 | 0.101069 | | 0.105879 | 0.102865 | 0.101712 |
| 0.102899 | 0.100098 | 0.100452 | 0.10189 | 0.099303 | | 0.097604 | 0.103791 | 0.100746 |
| 0.110771 | 0.10747 | 0.118394 | 0.112064 | 0.101158 | | 0.101485 | 0.097864 | 0.117942 |
| 0.109367 | 0.11895 | 0.116547 | 0.113959 | 0.118263 | | 0.120233 | 0.124274 | 0.11244 |
| 0.098337 | 0.102674 | 0.107288 | 0.104599 | 0.104016 | | 0.108181 | 0.107112 | 0.103899 |
| 0.110693 | 0.105292 | 0.111398 | 0.105246 | 0.09946 | | 0.096807 | 0.10764 | 0.107221 |
| NA | NA | NA | 0.095141 | 0.10568 | | NA | 0.106782 | 0.094758 |
| 0.103412 | 0.105704 | 0.103023 | 0.101908 | 0.107036 | | 0.109274 | 0.10745 | 0.10412 |
| 0.111069 | 0.111445 | 0.11304 | 0.10928 | 0.105609 | | 0.100398 | 0.106118 | 0.110513 |
| 0.102103 | 0.099626 | 0.114425 | 0.111437 | 0.106172 | | 0.109437 | 0.105784 | 0.112267 |

monocyte. monocyte. monocyte_multipotenmultipotenmuscle..ce myeloid_c naive_b_c

0.108843	0.10847 0.114744 0.119225 0.115288 0.109745 0.115291 0.118267

NA	NA	NA	NA	NA	NA	NA	NA

0.110843	0.11104 0.113152 0.111566 0.114579 0.117058	0.11469 0.115583

0.102458	0.10318 0.107891 0.107844 0.109922 0.105051 0.113629 0.117359

0.09676 0.096136 0.102481 0.099448 0.100741 0.108387 0.108152 0.108115

0.113558 0.111655 0.117887 0.129214 0.123747 0.168962 0.131364 0.138862

0.110696 0.110333	0.11432 0.110744 0.113077 0.116921 0.117134	0.1158

0.098942 0.098789 0.104631 0.103651 0.103362 0.105179 0.105448 0.106512

0.098942 0.098789 0.104631 0.103651 0.103362 0.105179 0.105448 0.106512

0.120324 0.120527 0.116742 NA	0.098999 0.121868 0.107997 0.099851

0.110771 0.109519 0.112687 0.119335 0.116594 0.157931 0.121744	0.12363

0.100266 0.099058 0.104766 0.106969 0.105919 0.141141 0.110767 0.116963

0.117056 0.115099	0.11451 0.101206 0.105337 0.108581 0.123015 0.107179

0.115394 0.113153 0.115362 0.104011 0.105513 0.114163 0.120695 0.107411

NA	NA	NA	NA	NA	NA	NA	NA NA	NA	NA	NA	NA	NA	NA	NA

0.102481 0.100954 0.095978 NA	NA	0.095819 0.096737 NA

0.102481 0.100954 0.095978 NA	NA	0.095819 0.096737 NA

0.173556 0.179746 0.165008 0.158068 0.152369 0.097075 0.150459 0.149845

0.10699 0.107438 0.111685 0.111239 0.112147	0.1151 0.115171 0.118427

NA	NA	NA	NA	NA	NA	NA	NA

0.137276 0.135429 0.135196 0.140839 0.142801 0.100662 0.155992 0.144406

NA	NA	NA	NA	NA	NA	NA	NA NA	NA	NA	NA	NA	NA	NA	NA

0.115535 0.115135 0.108517 NA	NA	0.091749 0.102401 NA

0.137302 0.138173 0.126328 NA	NA	NA	0.110528 NA

0.138303	0.1394 0.127597 NA	NA	NA	0.111026 NA

0.129377 0.129521 0.119775 NA	NA	NA	0.106605 NA

0.110933 0.110725	0.11335 0.112981 0.114914 0.121811 0.116961 0.116116

0.110933 0.110725	0.11335 0.112981 0.114914 0.121811 0.116961 0.116116

0.126548 0.125002 0.130022 0.129229 0.132541	0.14134 0.137785	0.1352

0.13023 0.129163 0.133441 0.133867 0.135244 0.142503	0.13933 0.137029

0.117269 0.117915	0.1121 NA	0.098094	0.11489 0.104837 NA

0.126121 0.127019 0.115137 NA	NA	NA	0.099267 NA NA	NA	NA	NA	NA	NA	NA	NA NA	NA	NA	NA	NA	NA	NA	NA NA	NA	NA	NA	NA	NA	NA	NA

0.10109 0.104074 0.110373 0.119244 0.113181 0.099343	0.10907 0.110619

| 0.092556 | 0.091885 | 0.097458 | | 0.099984 | 0.098491 | 0.118808 | 0.099392 | 0.125438 |
| --- | --- | --- | --- | --- | --- | --- | --- | --- |
| 0.092556 | 0.091885 | 0.097458 | | 0.099984 | 0.098491 | 0.118808 | 0.099392 | 0.125438 |
| 0.092556 | 0.091885 | 0.097458 | | 0.099984 | 0.098491 | 0.118808 | 0.099392 | 0.125438 |
| 0.100351 | 0.100984 | 0.109624 | | 0.113197 | 0.109373 | 0.095056 | 0.107157 | 0.106976 |
| 0.098937 | 0.098977 | 0.107508 | | 0.111722 | 0.108329 | 0.09539 | 0.107544 | 0.107559 |
| 0.175198 | 0.179162 | 0.175334 | | 0.194088 | 0.185602 | 0.105951 | 0.174408 | 0.166126 |
| 0.194198 | 0.195725 | 0.188428 | | 0.206279 | 0.196823 | 0.116809 | 0.190697 | 0.180292 |
| 0.212675 | 0.215486 | 0.209389 | | 0.231456 | 0.219107 | 0.119319 | 0.21424 | 0.201434 |
| 0.191506 | 0.194455 | 0.191601 | | 0.215728 | 0.204484 | 0.11731 | 0.188886 | 0.181245 |
| 0.183887 | 0.185256 | 0.178424 | | 0.193302 | 0.185047 | 0.117269 | 0.182937 | 0.173258 |
| 0.193074 | 0.194632 | 0.188837 | | 0.206833 | 0.197838 | 0.115682 | 0.197675 | 0.186493 |
| 0.21319 | 0.216452 | 0.203783 | | 0.220981 | 0.209635 | 0.112618 | 0.205137 | 0.193043 |
| 0.178014 | 0.177091 | 0.176401 | | 0.196414 | 0.190791 | 0.125331 | 0.183816 | 0.17887 |
| 0.188589 | 0.187854 | 0.183742 | | 0.206585 | 0.19829 | 0.124552 | 0.19429 | 0.186098 |
| 0.196156 | 0.196079 | 0.193839 | | 0.218531 | 0.210851 | 0.127462 | 0.203045 | 0.196478 |
| NA | NA | NA |  | NA | NA | NA | NA | NA |
| 0.098652 | 0.09808 | 0.10439 | | 0.100073 | 0.102401 | 0.113755 | 0.107825 | 0.104784 |
| 0.200229 | 0.200365 | 0.196566 | | 0.222733 | 0.211776 | 0.124907 | 0.206289 | 0.198296 |
| 0.207821 | 0.20806 | 0.201687 | | 0.230752 | 0.22236 | 0.126913 | 0.213364 | 0.206216 |
| 0.153365 | 0.154117 | 0.149447 | | 0.163552 | 0.161991 | 0.125909 | 0.158874 | 0.152631 |
| 0.221223 | 0.222631 | 0.217826 | | 0.249825 | 0.236924 | 0.120532 | 0.226082 | 0.216737 |
| NA | NA | NA |  | NA | NA | 0.099031 | NA | NA |
| NA | NA | NA |  | NA | NA | NA | NA | NA |
| 0.212625 | 0.213081 | 0.207199 | | 0.236462 | 0.226497 | 0.123977 | 0.21798 | 0.209257 |
| 0.145764 | 0.148612 | 0.152411 | | 0.162767 | 0.160498 | 0.124077 | 0.150832 | 0.146943 |
| 0.181408 | 0.183743 | 0.180116 | | 0.190026 | 0.184546 | 0.109464 | 0.178064 | 0.16739 |
| 0.208771 | 0.20886 | 0.204562 | | 0.23327 | 0.223282 | 0.126682 | 0.216968 | 0.207826 |
| 0.19851 | 0.199006 | 0.191195 | | 0.216824 | 0.207461 | 0.12209 | 0.200668 | 0.193037 |
| 0.153916 | 0.153226 | 0.151313 | | 0.165683 | 0.161669 | 0.117268 | 0.158975 | 0.153467 |
| 0.153916 | 0.153226 | 0.151313 | | 0.165683 | 0.161669 | 0.117268 | 0.158975 | 0.153467 |
| 0.217245 | 0.217523 | 0.212515 | | 0.242018 | 0.231136 | 0.122765 | 0.22359 | 0.212659 |
| 0.167471 | 0.168092 | 0.167018 | | 0.18511 | 0.181016 | 0.118281 | 0.175382 | 0.167712 |
| 0.169353 | 0.173952 | 0.173059 | | 0.180139 | 0.169601 | 0.099753 | 0.169135 | 0.157812 |
| 0.168473 | 0.169594 | 0.166415 | | 0.181054 | 0.175083 | 0.114055 | 0.169909 | 0.164778 |
| NA | NA | NA |  | NA | NA | NA | NA | NA |
| 0.102981 | 0.100504 |  | 0.1064 | 0.109111 | 0.107279 | 0.141138 | 0.111422 | 0.118109 |
| 0.215935 | 0.216523 | 0.207075 | | 0.231511 | 0.223588 | 0.120234 | 0.213621 | 0.204568 |
| 0.221272 | 0.222156 | 0.217116 | | 0.249604 | 0.236671 | 0.124517 | 0.228077 | 0.217649 |
| 0.155639 | 0.156506 | 0.151501 | | 0.166579 | 0.165276 | 0.129352 | 0.162567 | 0.155918 |

| 0.116984 | 0.114455 | 0.112486 | 0.100306 | 0.102193 | 0.112596 | 0.119469 | 0.102036 |
| --- | --- | --- | --- | --- | --- | --- | --- |
| 0.117411 | 0.115528 | 0.112019 | 0.101182 | 0.104505 | 0.110636 | 0.11736 | 0.10367 |
| 0.119781 | 0.117292 | 0.117817 | 0.10515 | 0.10787 | 0.114538 | 0.123082 | 0.108823 |
| 0.107255 | 0.104836 | 0.104681 | NA | NA | 0.105499 | 0.112672 | 0.095412 |
| NA | NA | NA | NA | NA | NA | NA | NA |
| NA | NA | NA | NA | NA | NA | NA | NA |
| NA | NA | NA | NA | NA | NA | NA | NA |
| NA | NA | NA | NA | NA | NA | NA | NA |
| NA | NA | NA | NA | NA | 0.091412 | NA | NA |
| NA | NA | NA | NA | NA | 0.090636 | NA | NA |
| NA | NA | NA | NA | NA | NA | NA | NA |
| NA | NA | NA | NA | NA | NA | NA | NA |
| NA | NA | NA | NA | NA | NA | NA | NA |
| NA | NA | NA | NA | NA | NA | NA | NA |
| NA | NA | NA | NA | NA | 0.091428 | NA | NA |
| 0.092242 | 0.091316 | NA | 0.093832 | 0.093991 | 0.093768 | NA | 0.091516 |
| NA | NA | NA | NA | NA | NA | NA | NA |
| NA | NA | NA | NA | NA | NA | NA | NA |
| 0.095636 | 0.094704 | 0.09236 | 0.098823 | 0.098146 | 0.093983 | NA | 0.095048 |
| 0.091284 | 0.090383 | NA | NA | 0.093409 | 0.091775 | NA | NA |
| NA | NA | NA | NA | NA | NA | NA | NA |
| 0.201885 | 0.202493 | 0.200602 | 0.226439 | 0.215478 | 0.126087 | 0.209834 | 0.201239 |
| 0.100802 | 0.101886 | 0.098079 | 0.107044 | 0.107854 | NA | 0.098533 | 0.097106 |
| 0.100802 | 0.101886 | 0.098079 | 0.107044 | 0.107854 | NA | 0.098533 | 0.097106 |
| 0.09214 | 0.094186 | NA | NA | 0.093572 | NA | NA | NA |
| 0.09214 | 0.094186 | NA | NA | 0.093572 | NA | NA | NA |
| 0.092727 | 0.093583 | NA | 0.096306 | 0.100413 | NA | NA | NA |
| 0.100148 | 0.101413 | 0.096545 | 0.108836 | 0.112018 | NA | 0.097807 | 0.09414 |
| NA | NA | NA | NA | NA | NA | NA | NA |
| NA | NA | NA | NA | 0.093908 | NA | NA | NA |
| NA | NA | NA | NA | 0.093908 | NA | NA | NA |
| NA | NA | NA | NA | 0.093908 | NA | NA | NA |
| NA | NA | NA | NA | NA | NA | NA | NA |
| NA | NA | NA | NA | NA | NA | NA | NA |
| NA | NA | NA | NA | NA | NA | NA | NA |
| NA | NA | NA | NA | NA | NA | NA | NA |
| 0.097098 | 0.096015 | 0.098834 | 0.099408 | 0.103646 | 0.119968 | 0.103832 | 0.099604 |
| 0.096445 | 0.09618 | 0.103933 | 0.102654 | 0.103197 | 0.110967 | 0.111361 | 0.109573 |
| 0.096445 | 0.09618 | 0.103933 | 0.102654 | 0.103197 | 0.110967 | 0.111361 | 0.109573 |

| 0.099718 | 0.100092 | 0.109157 | 0.107645 | 0.107731 | 0.117655 | 0.116972 | 0.116526 |
| --- | --- | --- | --- | --- | --- | --- | --- |
| NA | NA | NA | NA | NA | 0.109771 | NA | NA |
| 0.106657 | 0.10745 | 0.114137 | 0.116257 | 0.11555 | 0.118657 | 0.11908 | 0.120738 |
| NA | NA | 0.095723 | NA | 0.097214 | 0.109018 | 0.102927 | 0.100922 |
| 0.110453 | 0.110548 | 0.114577 | 0.115421 | 0.113925 | 0.111387 | 0.115453 | 0.113582 |
| 0.122861 | 0.123926 | 0.113374 | NA | NA | NA | 0.097757 | NA |
| NA | NA | NA | NA | NA | NA | NA | NA |
| NA | NA | NA | NA | NA | NA | NA | NA |
| NA | NA | NA | NA | NA | 0.105826 | 0.094355 | NA |
| NA | NA | NA | NA | NA | 0.104648 | 0.093617 | NA |
| 0.092897 | 0.091281 | 0.094763 | 0.094743 | 0.096018 | 0.111621 | 0.099661 | 0.096453 |
| NA | NA | NA | NA | NA | NA | NA | NA |
| NA | NA | NA | NA | NA | NA | NA | NA |
| NA | NA | NA | NA | NA | NA | NA | NA |
| NA | NA | NA | NA | NA | NA | NA | NA |
| NA | NA | NA | NA | NA | NA | NA | NA |
| NA | NA | NA | NA | NA | NA | NA | NA |
| NA | NA | NA | NA | NA | NA | NA | NA |
| NA | NA | NA | NA | NA | NA | NA | NA |
| 0.093752 | 0.093795 | NA | 0.099354 | 0.101653 | 0.09835 | NA | NA |
| NA | NA | NA | NA | NA | NA | NA | NA |
| NA | NA | NA | NA | NA | 0.091415 | NA | NA |
| NA | NA | NA | NA | NA | NA | NA | NA |
| NA | NA | NA | NA | NA | NA | NA | NA |
| NA | NA | NA | NA | NA | NA | NA | NA |
| 0.094753 | 0.094227 | NA | 0.097179 | 0.100101 | 0.101492 | NA | NA |
| NA | NA | NA | NA | NA | NA | NA | NA |
| NA | NA | NA | NA | NA | NA | NA | NA |
| NA | NA | NA | NA | NA | NA | NA | NA |
| NA | NA | NA | NA | NA | NA | NA | NA |
| 0.096346 | 0.095833 | 0.093785 | 0.095679 | 0.097113 | 0.10014 | NA | NA |
| NA | NA | NA | NA | NA | NA | NA | NA |
| NA | NA | NA | NA | NA | NA | NA | NA |
| NA | NA | NA | NA | NA | NA | NA | NA |
| NA | NA | NA | NA | NA | NA | NA | NA |
| 0.094832 | 0.095187 | NA | NA | NA | 0.093435 | NA | NA |
| NA | NA | NA | NA | NA | NA | NA | NA |
| 0.095118 | 0.095909 | NA | NA | NA | 0.094726 | NA | NA |
| NA | NA | NA | NA | NA | NA | NA | NA |

| NA | NA | NA |  | NA | NA | NA | NA | NA |  |
| --- | --- | --- | --- | --- | --- | --- | --- | --- | --- |
| 0.096234 | 0.095721 | NA |  | 0.101906 | 0.104855 | 0.100278 | NA | 0.093781 | |
| NA | NA | NA |  | NA | NA | NA | NA | NA |  |
| NA | NA | NA |  | NA | NA | NA | NA | NA |  |
| NA | NA | NA |  | NA | NA | NA | NA | NA |  |
| 0.097987 | 0.098503 | NA |  | NA | 0.096319 | 0.095301 | NA | NA |  |
| NA | NA | NA |  | NA | NA | NA | NA | NA |  |
| NA | NA | NA |  | NA | NA | NA | NA | NA |  |
| NA | NA | NA |  | NA | NA | NA | NA | NA |  |
| 0.094633 | 0.095085 | NA |  | NA | NA | 0.096134 | NA | NA |  |
| 0.144571 | 0.145285 | 0.140266 | | 0.146456 | 0.142802 | 0.099622 | 0.134156 | 0.12995 | |
| 0.117398 | 0.117718 | 0.095533 | | NA | 0.094641 | 0.122849 | 0.09626 | 0.095049 | |
| 0.110829 | 0.11433 | 0.098602 | | NA | 0.096223 | NA | NA | 0.100371 | |
| 0.132905 | 0.136894 | 0.12192 | | 0.119317 | 0.118938 | 0.09361 | 0.11254 | 0.12108 | |
| 0.168132 | 0.168904 | 0.145577 | | 0.139633 | 0.140134 | 0.120577 | 0.142466 | 0.158876 | |
| 0.116403 | 0.116886 | 0.109302 | | 0.106597 | 0.106337 | 0.101721 | 0.110324 | 0.115422 | |
| 0.098647 | 0.100138 | 0.097645 | | NA | NA | NA | 0.097026 | 0.101602 | |
| 0.111251 | 0.112909 | 0.099161 | | 0.09577 | 0.097691 | 0.091926 | 0.094429 |  | 0.0997 |
| 0.137158 | 0.139591 | 0.125398 | | 0.117403 | 0.116047 | 0.101186 | 0.116451 | 0.128097 | |
| 0.12611 | 0.127725 | 0.111692 | | 0.103923 | 0.105416 | NA | 0.105478 | 0.117393 | |
| 0.114813 | 0.112133 | 0.111251 | | NA | 0.09821 | 0.113711 | 0.115482 | 0.098553 | |
| 0.11294 | 0.110931 | 0.109295 | | 0.096948 | 0.098786 | 0.108564 | 0.114623 | 0.101369 | |
| 0.118466 | 0.116161 | 0.114548 | | 0.100785 | 0.102134 | 0.109413 | 0.119009 | 0.103374 | |
| 0.106116 | 0.103547 | 0.10239 | | NA | NA | 0.103531 | 0.108407 | NA |  |
| 0.094509 | NA | 0.102976 | | 0.098377 | 0.098628 | NA | 0.093989 | 0.095513 | |
| 0.101293 | 0.100108 | 0.103745 | | 0.102758 | 0.101635 | 0.103283 | 0.105192 | 0.104014 | |
| 0.096315 | 0.095419 | 0.104191 | | 0.09836 | 0.100947 | 0.095045 | 0.102722 | 0.101598 | |
| 0.099854 | 0.100196 | 0.107989 | | 0.105934 | 0.10501 | 0.096636 | 0.105826 | 0.104554 | |
| 0.099854 | 0.100196 | 0.107989 | | 0.105934 | 0.10501 | 0.096636 | 0.105826 | 0.104554 | |
| NA | NA | 0.102437 | | 0.100133 | 0.09955 | NA | NA | 0.097234 | |
| 0.096228 | 0.097362 | 0.109027 | | 0.111151 | 0.107082 | NA | 0.101797 | 0.101486 | |
| NA | NA |  | 0.1033 | 0.099487 | 0.098376 | NA | 0.095406 | 0.097648 | |
| NA | NA |  | 0.1033 | 0.099487 | 0.098376 | NA | 0.095406 | 0.097648 | |
| NA | NA |  | 0.1033 | 0.099487 | 0.098376 | NA | 0.095406 | 0.097648 | |
| 0.100142 | 0.102239 | 0.111505 | | 0.110526 | 0.108452 | 0.093024 | 0.100921 | 0.101919 | |
| 0.102892 | 0.104166 | 0.113803 | | 0.120352 | 0.115356 | 0.101159 | 0.113082 | 0.112455 | |
| NA | NA | 0.098897 | | NA | NA | NA | NA | 0.09582 | |
| NA | NA | NA |  | NA | NA | NA | NA | NA |  |
| 0.096746 | 0.097707 | NA |  | NA | 0.092909 | NA | 0.092478 | 0.093327 | |

| NA | NA | NA | NA | NA | NA | NA | NA |
| --- | --- | --- | --- | --- | --- | --- | --- |
| NA | NA | NA | NA | NA | NA | NA | NA |
| NA | NA | NA | NA | NA | NA | NA | NA |
| NA | NA | NA | NA | NA | NA | NA | NA |
| 0.104937 | 0.10395 | 0.10966 | 0.11576 | 0.112224 | 0.151487 | 0.123987 | 0.127751 |
| 0.108649 | 0.108173 | 0.108205 | 0.111734 | 0.111759 | 0.118718 | 0.116467 | 0.110288 |
| 0.098041 | 0.097278 | 0.102121 | 0.105932 | 0.103699 | 0.111469 | 0.108593 | 0.105509 |
| 0.11273 | 0.112679 | 0.114509 | 0.119452 | 0.117814 | 0.127127 | 0.123008 | 0.119705 |
| 0.100037 | 0.099731 | 0.102399 | 0.099252 | 0.10039 | 0.102858 | 0.111862 | 0.105104 |
| 0.104653 | 0.104468 | 0.105256 | 0.104941 | 0.105291 | 0.106779 | 0.114084 | 0.109058 |
| 0.109075 | 0.108257 | 0.113279 | 0.112075 | 0.113456 | 0.116849 | 0.117618 | 0.117202 |
| 0.107771 | 0.105921 | 0.113239 | 0.123038 | 0.118632 | 0.121731 | 0.120919 | 0.117717 |
| 0.117113 | 0.117097 | 0.123058 | 0.128157 | 0.126718 | 0.131895 | 0.128604 | 0.128348 |
| 0.098076 | 0.096829 | 0.097779 | 0.099579 | 0.101226 | 0.122655 | 0.101367 | 0.10443 |
| NA | NA | NA | NA | NA | NA | NA | NA |
| NA | NA | NA | NA | NA | NA | NA | NA |
| 0.103704 | 0.103449 | 0.108103 | 0.106544 | 0.108056 | 0.11221 | 0.110468 | 0.112601 |
| NA | NA | NA | NA | NA | NA | NA | NA |
| 0.105621 | 0.104159 | 0.107108 | 0.115071 | 0.112015 | 0.154686 | 0.116226 | 0.123205 |
| NA | NA | NA | NA | NA | NA | NA | NA |
| NA | NA | NA | NA | NA | NA | NA | NA |
| 0.109738 | 0.109093 | 0.112692 | 0.113522 | 0.115239 | 0.113772 | 0.113548 | 0.116638 |
| 0.11041 | 0.109473 | 0.118369 | 0.115563 | 0.11678 | 0.120997 | 0.121167 | 0.118505 |
| 0.11041 | 0.109473 | 0.118369 | 0.115563 | 0.11678 | 0.120997 | 0.121167 | 0.118505 |
| 0.11041 | 0.109473 | 0.118369 | 0.115563 | 0.11678 | 0.120997 | 0.121167 | 0.118505 |
| 0.11041 | 0.109473 | 0.118369 | 0.115563 | 0.11678 | 0.120997 | 0.121167 | 0.118505 |
| 0.117308 | 0.117508 | 0.118665 | 0.122616 | 0.122435 | 0.120372 | 0.12102 | 0.119367 |
| 0.117308 | 0.117508 | 0.118665 | 0.122616 | 0.122435 | 0.120372 | 0.12102 | 0.119367 |
| 0.117308 | 0.117508 | 0.118665 | 0.122616 | 0.122435 | 0.120372 | 0.12102 | 0.119367 |
| 0.117308 | 0.117508 | 0.118665 | 0.122616 | 0.122435 | 0.120372 | 0.12102 | 0.119367 |
| 0.117935 | 0.118344 | 0.111358 | 0.113909 | 0.113978 | 0.114634 | 0.112306 | 0.111568 |
| 0.117935 | 0.118344 | 0.111358 | 0.113909 | 0.113978 | 0.114634 | 0.112306 | 0.111568 |
| 0.113758 | 0.114424 | 0.109858 | 0.112431 | 0.110974 | 0.112478 | 0.111095 | 0.110905 |
| 0.113758 | 0.114424 | 0.109858 | 0.112431 | 0.110974 | 0.112478 | 0.111095 | 0.110905 |
| 0.116615 | 0.116021 | 0.122211 | 0.122245 | 0.12336 | 0.125133 | 0.128563 | 0.125047 |
| 0.104574 | 0.103217 | 0.109716 | 0.118612 | 0.113919 | 0.167276 | 0.121395 | 0.130383 |
| 0.104574 | 0.103217 | 0.109716 | 0.118612 | 0.113919 | 0.167276 | 0.121395 | 0.130383 |
| 0.110056 | 0.10865 | 0.109384 | 0.117687 | 0.114683 | 0.153756 | 0.118663 | 0.125445 |
| 0.110056 | 0.10865 | 0.109384 | 0.117687 | 0.114683 | 0.153756 | 0.118663 | 0.125445 |

| 0.103144 | 0.10169 | 0.10853 | 0.116663 | 0.11399 | 0.155435 | 0.118031 | 0.129695 |
| --- | --- | --- | --- | --- | --- | --- | --- |
| 0.10869 | 0.107301 | 0.114551 | 0.123237 | 0.11944 | 0.168016 | 0.126992 | 0.135377 |
| 0.104109 | 0.102415 | 0.109436 | 0.113586 | 0.112343 | 0.152091 | 0.118094 | 0.122778 |
| 0.101151 | 0.099835 | 0.101841 | 0.104847 | 0.10278 | 0.128508 | 0.105312 | 0.108975 |
| 0.121658 | 0.119633 | 0.117194 | 0.108818 | 0.113738 | 0.11179 | 0.125511 | 0.111262 |
| 0.112542 | 0.109816 | 0.10894 | NA | NA | 0.112793 | 0.112745 | 0.096889 |
| 0.119338 | 0.117725 | 0.116378 | 0.109976 | 0.115895 | 0.112485 | 0.127687 | 0.113429 |
| 0.108876 | 0.106483 | 0.111612 | 0.10573 | 0.120469 | 0.162926 | 0.122717 | 0.114199 |
| 0.110794 | 0.107968 | 0.113823 | 0.108063 | 0.123728 | 0.167626 | 0.124966 | 0.115534 |
| NA | NA | NA | NA | NA | 0.12959 | 0.09715 | NA |
| 0.11098 | 0.108272 | 0.113744 | 0.106515 | 0.123886 | 0.17012 | 0.124591 | 0.116095 |
| 0.105557 | 0.103603 | 0.105119 | 0.101818 | 0.111394 | 0.154052 | 0.112761 | 0.105811 |
| NA | NA | NA | NA | NA | NA | NA | 0.101365 |
| 0.102842 | 0.101616 | 0.105708 | 0.109882 | 0.108365 | 0.102347 | 0.103769 | 0.109274 |
| NA | NA | NA | NA | NA | NA | NA | 0.096136 |
| NA | NA | NA | NA | NA | NA | NA | NA |
| NA | NA | NA | NA | NA | NA | NA | NA |
| 0.096893 | 0.096159 | 0.095961 | 0.094793 | 0.095945 | 0.095865 | 0.096726 | 0.096203 |
| NA | NA | NA | NA | NA | NA | NA | NA |
| NA | NA | NA | NA | NA | NA | NA | NA |
| NA | NA | NA | NA | NA | NA | NA | NA |
| NA | NA | NA | NA | NA | NA | NA | NA |
| NA | NA | NA | NA | NA | NA | NA | NA |
| 0.09962 | 0.098835 | 0.096925 | 0.096878 | 0.098147 | NA | 0.098215 | 0.095787 |
| 0.107736 | 0.107695 | 0.107971 | 0.109804 | 0.109026 | 0.092092 | 0.108083 | 0.102355 |
| NA | NA | NA | NA | NA | NA | NA | NA |
| NA | NA | NA | NA | NA | NA | NA | NA |
| NA | NA | NA | NA | NA | NA | NA | NA |
| 0.115884 | 0.115288 | 0.120919 | 0.120239 | 0.121547 | 0.12466 | 0.125569 | 0.12319 |
| 0.108111 | 0.11126 | 0.116614 | 0.119512 | 0.116037 | 0.104258 | 0.111512 | 0.108526 |
| 0.108111 | 0.11126 | 0.116614 | 0.119512 | 0.116037 | 0.104258 | 0.111512 | 0.108526 |
| NA | NA | NA | NA | NA | 0.104558 | NA | NA |
| NA | NA | NA | NA | NA | 0.099755 | NA | NA |
| NA | NA | NA | NA | NA | 0.098974 | NA | NA |
| NA | NA | 0.09603 | 0.095312 | 0.09494 | 0.096244 | 0.093609 | 0.092562 |
| 0.126992 | 0.126642 | 0.130265 | 0.138257 | 0.136476 | 0.108642 | 0.136151 | 0.133894 |
| 0.088937 | NA | 0.090809 | NA | NA | 0.10253 | 0.093777 | NA |
| NA | NA | NA | NA | NA | 0.091874 | NA | NA |
| NA | NA | 0.093225 | NA | NA | 0.103013 | 0.095087 | NA |

| 0.092795 | 0.093066 | 0.093111 | 0.091909 | 0.091911 | 0.09903 | 0.092284 | NA |
| --- | --- | --- | --- | --- | --- | --- | --- |
| NA | NA | NA | NA | NA | 0.103454 | NA | NA |
| NA | NA | NA | NA | NA | 0.101926 | NA | NA |
| NA | NA | NA | NA | NA | 0.100841 | NA | NA |
| NA | NA | NA | NA | NA | 0.104237 | NA | NA |
| 0.110506 | 0.10948 | 0.107645 | 0.112337 | 0.111552 | 0.11776 | 0.116015 | 0.109762 |
| NA | NA | NA | NA | NA | NA | NA | NA |
| NA | NA | NA | NA | NA | NA | NA | NA |
| NA | NA | NA | NA | NA | NA | NA | NA |
| NA | NA | NA | NA | NA | NA | NA | NA |
| 0.10608 | 0.105326 | 0.106949 | 0.105719 | 0.106354 | 0.114115 | 0.109528 | 0.14376 |
| 0.097404 | 0.096773 | 0.098046 | NA | NA | 0.103207 | 0.101953 | 0.112924 |
| 0.112265 | 0.111864 | 0.110801 | 0.10826 | 0.109143 | 0.118602 | 0.115248 | 0.113304 |
| NA | NA | NA | NA | NA | NA | NA | NA |
| NA | NA | 0.094179 | NA | NA | 0.09975 | 0.098757 | 0.118352 |
| 0.100838 | 0.100712 | 0.097795 | 0.096858 | 0.098565 | 0.099723 | 0.098984 | 0.112528 |
| 0.098279 | 0.096881 | 0.097975 | 0.096298 | 0.096648 | 0.104858 | 0.100168 | 0.102674 |
| NA | NA | 0.095904 | NA | NA | 0.104366 | 0.097968 | 0.108479 |
| NA | NA | NA | NA | NA | NA | NA | NA |
| NA | NA | 0.103684 | 0.110018 | 0.106294 | 0.095699 | 0.108907 | 0.108296 |
| 0.101071 | 0.10091 | 0.109774 | 0.117329 | 0.112749 | 0.101113 | 0.112912 | 0.112711 |
| 0.096242 | 0.096476 | 0.099732 | 0.099644 | 0.098704 | 0.10152 | 0.096871 | 0.094796 |
| 0.094369 | 0.095229 | 0.098091 | 0.097717 | 0.097607 | 0.100641 | 0.096629 | 0.093591 |
| 0.121429 | 0.123137 | 0.12522 | 0.126684 | 0.126969 | 0.106533 | 0.125287 | 0.131327 |
| 0.105447 | 0.105469 | 0.107953 | 0.110162 | 0.107017 | 0.118715 | 0.111331 | 0.111591 |
| NA | NA | 0.096747 | 0.094364 | 0.095031 | 0.102514 | 0.097137 | NA |
| 0.104919 | 0.10385 | 0.106445 | 0.111584 | 0.110261 | 0.103755 | 0.104796 | 0.11239 |
| 0.096837 | 0.095809 | 0.098332 | 0.100393 | 0.099579 | 0.102971 | 0.098267 | 0.102908 |
| 0.11167 | 0.110326 | 0.112676 | 0.110863 | 0.112788 | 0.116001 | 0.114967 | 0.112363 |
| 0.095999 | 0.095587 | 0.099044 | 0.098248 | 0.099874 | 0.107924 | 0.102816 | 0.10313 |
| 0.115275 | 0.115444 | 0.113237 | 0.115681 | 0.119634 | 0.128553 | 0.114009 | 0.117289 |
| 0.102255 | 0.10098 | 0.103752 | 0.10402 | 0.105763 | 0.114429 | 0.108657 | 0.107842 |
| 0.11372 | 0.112736 | 0.111938 | 0.117442 | 0.119033 | 0.125389 | 0.118656 | 0.116899 |
| 0.101479 | 0.100608 | 0.10434 | 0.106984 | 0.10717 | 0.11497 | 0.108017 | 0.110186 |
| 0.105906 | 0.104954 | 0.106566 | 0.104882 | 0.107202 | 0.115356 | 0.107656 | 0.107034 |
| 0.097399 | 0.097405 | 0.098196 | NA | 0.099693 | 0.105947 | 0.099259 | 0.102002 |
| NA | NA | NA | NA | NA | 0.101467 | NA | NA |
| NA | NA | NA | NA | NA | 0.101467 | NA | NA |
| NA | NA | NA | NA | NA | 0.101467 | NA | NA |

| 0.137097 | 0.135818 | 0.133479 | | 0.138938 | 0.141281 | 0.093744 | | 0.152391 | 0.141863 |
| --- | --- | --- | --- | --- | --- | --- | --- | --- | --- |
| 0.142574 | 0.140706 | 0.139652 | | 0.145944 | 0.149082 | 0.09481 | | 0.160816 | 0.148192 |
| 0.126559 | 0.125615 | 0.126007 | | 0.132666 | 0.134411 | NA |  | 0.142962 | 0.134531 |
| 0.092988 | 0.093659 | 0.096907 | | 0.096188 | 0.095656 | 0.098742 | | 0.096153 | NA |
| NA | NA | NA |  | NA | NA | 0.101982 | | NA | NA |
| NA | NA | NA |  | NA | NA | 0.102012 | | NA | NA |
| NA | NA | NA |  | NA | NA | 0.102259 | | NA | NA |
| 0.099467 | 0.099206 | 0.096363 | | 0.09508 | 0.09626 | 0.124933 | | 0.095836 | 0.095341 |
| 0.10008 | 0.099878 | 0.105116 | | 0.107053 | 0.107493 | 0.109206 | | 0.109171 | 0.105345 |
| 0.098585 | 0.09786 | 0.103323 | | 0.104342 | 0.105062 | 0.114669 | | 0.106758 | 0.106778 |
| 0.100426 | 0.099721 | 0.103508 | | 0.103469 | 0.105336 | 0.12128 | | 0.109425 | 0.104657 |
| 0.100426 | 0.099721 | 0.103508 | | 0.103469 | 0.105336 | 0.12128 | | 0.109425 | 0.104657 |
| 0.102845 | 0.102437 | 0.105491 | | 0.105432 | 0.10704 | 0.116712 | | 0.110724 | 0.105998 |
| 0.108296 | 0.106429 | 0.106515 | | NA | NA | 0.103176 | | 0.111938 | 0.100461 |
| 0.108296 | 0.106429 | 0.106515 | | NA | NA | 0.103176 | | 0.111938 | 0.100461 |
| 0.094253 | 0.093719 | NA |  | NA | 0.092376 | NA |  | 0.093273 | NA |
| 0.093008 | 0.094075 | NA |  | NA | NA | NA |  | NA | NA |
| 0.09222 | 0.092764 | NA |  | NA | NA | NA |  | NA | NA |
| 0.094873 | 0.096551 | 0.107572 | | 0.106712 | 0.104169 | NA |  | 0.097295 | 0.098226 |
| 0.159358 | 0.158536 | 0.154937 | | 0.170834 | 0.166102 | 0.118986 | | 0.162252 | 0.156939 |
| 0.237357 | 0.246369 | 0.220301 | | 0.232047 | 0.216837 | 0.093075 | | 0.227535 | 0.205084 |
| 0.162023 | 0.170828 | 0.15674 | | 0.156007 | 0.146788 | NA |  | 0.146478 | 0.13696 |
| 0.171423 | 0.175125 | 0.166159 | | 0.177495 | 0.169089 | 0.104409 | | 0.164236 | 0.156658 |
| 0.226951 | 0.234536 | 0.210875 | | 0.218464 | 0.203382 | 0.097102 | | 0.21205 | 0.192061 |
| 0.198216 | 0.205144 | 0.186508 | | 0.196355 | 0.183391 | 0.092815 | | 0.185219 | 0.17037 |
| 0.109521 | 0.108406 | 0.103375 | | 0.104981 | 0.107474 | 0.108637 | | 0.104123 | 0.101349 |
| 0.136987 | 0.140712 | 0.122053 | | 0.115487 | 0.114913 | 0.094168 | | 0.111785 | 0.12341 |
| 0.108444 | 0.106728 |  | 0.1049 | 0.107649 | 0.109977 | 0.116133 | | 0.107402 | 0.101461 |
| 0.098405 | 0.097803 | 0.098842 | | 0.100437 | 0.100084 | 0.120426 | | 0.103541 | 0.104734 |
| 0.105563 | 0.103985 | 0.110523 | | 0.120214 | 0.116508 | 0.147553 | | 0.122595 | 0.131175 |
| 0.115958 | 0.11437 | 0.114628 | | 0.122692 | 0.121295 | 0.170609 | | 0.124622 | 0.12921 |
| NA | NA | NA |  | 0.095491 | NA |  | 0.1237 | 0.095871 | 0.112996 |
| 0.092817 | 0.093288 | NA |  | NA | NA | NA |  | NA | NA |
| NA | NA | NA |  | NA | NA | 0.110273 | | NA | NA |
| NA | NA | NA |  | NA | NA | 0.108312 | | NA | NA |
| NA | NA | 0.099657 | | 0.103102 | 0.100522 | 0.10188 | | 0.10514 | 0.106579 |
| 0.099617 | 0.098796 | 0.107826 | | 0.113049 | 0.110918 | 0.113987 | | 0.112984 | 0.115822 |
| 0.096595 | 0.09499 | 0.10686 | | 0.111883 | 0.109638 | 0.110238 | | 0.112354 | 0.115197 |
| NA | NA | NA |  | NA | NA | NA |  | NA | NA |

| 0.097839 | 0.096747 | | 0.106902 | 0.112385 | | 0.110053 | 0.111695 | 0.112288 | 0.114565 |
| --- | --- | --- | --- | --- | --- | --- | --- | --- | --- |
| 0.108132 | 0.106615 | | 0.113496 | 0.120605 | | 0.118799 | 0.157022 | 0.124972 | 0.129278 |
| 0.107835 | 0.106721 | | 0.11078 | 0.107311 | | 0.109453 | 0.114348 | 0.114571 | 0.113353 |
| 0.107081 |  | 0.1068 | 0.110052 | 0.105792 | | 0.108042 | 0.114987 | 0.113679 | 0.111809 |
| 0.102397 | 0.101883 | | 0.105169 | 0.100488 | | 0.10335 | 0.110558 | 0.108199 | 0.106601 |
| 0.103095 | 0.101729 | | 0.104253 | 0.106444 | | 0.106891 | 0.132633 | 0.108768 | 0.113146 |
| NA | NA |  | 0.096315 | NA |  | 0.09696 | 0.124089 | 0.102947 | 0.100913 |
| 0.106755 | 0.106053 | | 0.110986 | 0.107022 | | 0.109234 | 0.116555 | 0.113903 | 0.113257 |
| 0.10109 | 0.100106 | | 0.105276 |  | 0.1016 | 0.103738 | 0.111311 | 0.10933 | 0.107664 |
| NA | NA |  | NA | NA |  | NA | NA | NA | NA |
| NA | NA |  | NA | NA |  | NA | NA | NA | NA |
| NA | NA |  | NA | NA |  | NA | NA | NA | NA |
| 0.118789 | 0.119518 | | 0.125668 | 0.132489 | | 0.131736 | 0.113017 | 0.129005 | 0.134054 |
| 0.105771 | 0.10564 | | 0.111697 | 0.115638 | | 0.114188 | 0.111254 | 0.117647 | 0.117312 |
| NA | NA |  | 0.094114 | 0.097645 | | 0.096496 | 0.122665 | 0.102138 | 0.106009 |
| 0.100453 | 0.100329 | | 0.107121 | 0.112193 | | 0.110708 | 0.109964 | 0.111688 | 0.117024 |
| NA | NA |  | 0.093409 | 0.096598 | | 0.095865 | 0.121044 | 0.10101 | 0.104656 |
| NA | NA |  | 0.097796 | 0.099181 | | 0.097325 | NA | 0.101808 | 0.101237 |
| 0.094516 | NA |  | 0.101274 | 0.104607 | | 0.102852 | 0.102352 | 0.10649 | 0.104534 |
| NA | NA |  | NA | NA |  | NA | NA | NA | NA |
| 0.114429 | 0.114464 | | 0.118166 | 0.122467 | | 0.12189 | 0.129643 | 0.125697 | 0.124287 |
| 0.096507 | 0.096929 | | 0.09747 | 0.095015 | | 0.096677 | 0.109085 | 0.099118 | 0.096831 |
| NA | NA |  | NA | NA |  | NA | NA | NA | NA |
| NA | NA |  | NA | NA |  | NA | 0.096681 | NA | NA |
| 0.097241 | 0.096248 | | 0.106478 | 0.110352 | | 0.10724 | 0.102151 | 0.10968 | 0.110143 |
| 0.101344 | 0.102179 | | 0.112637 | 0.115411 | | 0.113188 | 0.099934 | 0.106428 | 0.107759 |
| 0.102016 | 0.101968 | | 0.109743 | 0.115423 | | 0.111195 | 0.104859 | 0.110761 | 0.11196 |
| 0.098083 | 0.09762 | | 0.104893 | 0.108861 | | 0.107224 | 0.104969 | 0.109154 | 0.114089 |
| 0.098766 | 0.101182 | | 0.10671 | 0.106644 | | 0.104922 | 0.097287 | 0.105002 | 0.100506 |
| 0.108921 | 0.109302 | | 0.111963 | 0.114728 | | 0.113523 | 0.113902 | 0.116706 | 0.117574 |
| 0.109802 | 0.112818 | | 0.119255 | 0.12062 | | 0.11899 | 0.105396 | 0.117038 | 0.110023 |
| 0.114315 | 0.11365 | | 0.116103 | 0.121681 | | 0.118507 | 0.129031 | 0.125971 | 0.125333 |
| 0.104779 | 0.104293 | | 0.105662 | 0.106805 | | 0.107477 | 0.114432 | 0.109313 | 0.106669 |
| 0.106145 | 0.106403 | | 0.107206 | 0.108597 | | 0.108023 | 0.116492 | 0.110284 | 0.112048 |
| 0.09674 |  | 0.0989 | 0.105003 | 0.103055 | | 0.100431 | NA | 0.100743 | 0.104612 |
| 0.116342 | 0.117191 | | 0.112218 | 0.118185 | | 0.117407 | 0.102939 | 0.109258 | 0.108421 |
| 0.132429 | 0.13236 | | 0.124916 | 0.129939 | | 0.129428 | 0.105611 | 0.121922 | 0.121015 |
| 0.103158 | 0.103324 | | 0.108689 | 0.111317 | | 0.109853 | 0.116259 | 0.113052 | 0.108565 |
| 0.108233 | 0.109447 | | 0.109142 | 0.113365 | | 0.112461 | 0.115609 | 0.116385 | 0.116918 |

| 0.109878 | 0.108997 | 0.11505 | 0.115811 | | 0.117695 | 0.120017 | 0.115139 | | 0.116151 |
| --- | --- | --- | --- | --- | --- | --- | --- | --- | --- |
| 0.110252 | 0.11014 | 0.115227 | 0.120739 | | 0.118878 | 0.111006 | 0.118072 | | 0.115291 |
| 0.113045 | 0.113273 | 0.112786 | 0.114053 | | 0.112231 | 0.129744 | 0.118201 | | 0.113985 |
| 0.099873 | 0.099801 | 0.104288 | 0.108715 | | 0.104739 | 0.104117 | 0.106681 | | 0.110091 |
| 0.136424 | 0.141679 | 0.134041 | 0.129542 | | 0.130067 | 0.120547 | 0.126955 | | 0.125448 |
| 0.098662 | 0.097596 | NA | NA |  | NA | 0.111174 | NA |  | NA |
| 0.110589 | 0.109838 | 0.112266 | 0.118769 | | 0.116432 | 0.118257 | 0.118587 | | 0.117304 |
| 0.098147 | 0.097007 | 0.101369 | 0.104653 | | 0.103527 | 0.126542 | 0.109639 | | 0.110529 |
| 0.10306 | 0.102674 | 0.108814 | 0.112881 | | 0.111869 | 0.116184 | 0.11288 | | 0.111774 |
| 0.105769 | 0.104855 | 0.108064 | 0.110117 | | 0.110826 | 0.110188 | 0.108129 | | 0.10989 |
| 0.10327 | 0.103998 | 0.110291 | 0.111368 | | 0.110803 | 0.109284 | 0.110014 | | 0.107215 |
| NA | NA | NA | NA |  | NA | NA | NA |  | NA |
| 0.106605 | 0.106466 | 0.109091 | 0.112065 | | 0.111458 | 0.102909 | 0.111599 | | 0.110707 |
| 0.096624 | 0.09766 | 0.10608 | 0.110462 | | 0.107094 | NA | 0.103579 | | 0.104094 |
| 0.100825 | 0.102307 | 0.112497 | 0.113006 | | 0.111876 | 0.096532 | 0.110404 | | 0.11031 |
| 0.112478 | 0.111541 | 0.116709 | 0.125389 | | 0.122826 | 0.135041 | 0.128035 | | 0.12868 |
| NA | NA | 0.097753 | 0.099716 | | 0.098226 | 0.104082 | 0.100295 | | 0.101752 |
| 0.100324 | 0.09952 | 0.105283 | 0.112734 | | 0.109965 | 0.121146 | 0.115428 | | 0.115999 |
| 0.092847 | 0.092383 | NA | NA |  | NA | 0.097635 | NA |  | NA |
| 0.104623 | 0.103506 | 0.107159 | 0.108004 | | 0.107609 | 0.104665 | 0.107085 | | 0.105652 |
| 0.108786 | 0.108624 | 0.114423 | 0.115442 | | 0.115256 | 0.110713 | 0.114648 | | 0.111583 |
| 0.116267 | 0.123271 | 0.110409 | 0.109075 | | 0.107127 | NA |  | 0.1038 | 0.096861 |
| 0.099291 | 0.097965 | 0.104253 | 0.106693 | | 0.105998 | 0.115669 | 0.110067 | | 0.106981 |
| 0.115567 | 0.119599 | 0.119053 | 0.125828 | | 0.123437 | 0.092743 | 0.114539 | | 0.107718 |
| 0.098509 | 0.098148 | 0.105487 | 0.108731 | | 0.108637 | 0.115298 | 0.109906 | | 0.108565 |
| 0.119026 | 0.121549 | 0.115267 | 0.118133 | | 0.115844 | 0.098108 | 0.115596 | | 0.110842 |
| 0.107543 | 0.106893 | 0.109534 | 0.109987 | | 0.108974 | 0.115219 | 0.107565 | | 0.106073 |
| 0.09992 | 0.101285 | 0.110393 | 0.115138 | | 0.111881 | 0.096862 | 0.104214 | | 0.102678 |
| 0.100768 | 0.099994 | 0.100345 | 0.10318 | | 0.102728 | 0.11549 | 0.105863 | | 0.104112 |
| 0.101307 | 0.100395 | 0.101205 | 0.100224 | | 0.101368 | 0.105689 | 0.101691 | | 0.100911 |
| 0.117727 | 0.120025 | 0.118803 | 0.121805 | | 0.118386 | 0.098014 | 0.115311 | | 0.110115 |
| 0.10579 | 0.106824 | 0.11438 | 0.120202 | | 0.117399 | 0.113365 | 0.118221 | | 0.118875 |
| 0.099162 | 0.099121 | 0.106417 | 0.108362 | | 0.106432 | 0.10536 | 0.106229 | | 0.104574 |
| 0.109045 | 0.108198 | 0.106836 | 0.111213 | | 0.11075 | 0.094602 | 0.113769 | | 0.111327 |
| NA | NA | 0.096075 | 0.094882 | | NA | NA | 0.094727 | | 0.097756 |
| 0.099702 | 0.09948 | 0.102634 | 0.103742 | | 0.10227 | 0.110819 | 0.106072 | | 0.107919 |
| 0.109899 | 0.110045 | 0.109449 | 0.111895 | | 0.112877 | 0.106359 | 0.11225 | | 0.110413 |
| 0.106948 | 0.110805 | 0.11559 |  | 0.1176 | 0.113718 | 0.100263 | 0.108913 | | 0.106508 |

naive_cd4 natural_kil neural_pro neurosphe neurosphe neutrophil neutrophil neutrophil

0.124051 0.120668 0.135492 0.110921 0.122997	0.1145 0.110921 0.111081

NA	NA	NA	0.121626 NA	NA	NA	NA

0.117852 0.120048 NA	0.104973	0.10814 0.112104 0.112315 0.113266

0.140419 0.115848 0.119992 0.109373 0.118675 0.107718 0.110289 0.109677

0.110394 0.112019 NA	NA	0.098266 0.102055 0.105961 0.104781

0.122314 0.119334 0.124633	0.12296 0.140232 0.117273 0.123847 0.124749

0.117942 0.121494 NA	0.097656 0.103229 0.113452 0.115206 0.116372

0.118227 0.115324 NA	0.096585 0.111786 0.105285 0.103236 0.103554

0.118227 0.115324 NA	0.096585 0.111786 0.105285 0.103236 0.103554

NA	0.101412 NA	0.109391 0.109307 0.119843 0.122294	0.12094

0.106888 0.109436 NA	0.117968 0.129095 0.112601 0.115736 0.116554

0.105899 0.108627 NA	0.103707 0.111169 0.105132 0.106222 0.107295

NA	0.105404 NA	NA	0.105555 0.105514 0.113842 0.114048

0.1048 0.107101 NA	0.09866 0.111395	0.10725 0.113179 0.112993

NA	NA	NA	0.093632 NA	NA	NA	NA NA	NA	NA	0.121164	0.09695 NA	NA	NA

NA	NA	NA	0.096517 NA	NA	NA	0.09377

NA	NA	NA	0.096517 NA	NA	NA	0.09377

0.127133 0.133109 NA	0.092585	0.09372	0.14912 0.136694 0.139114

0.120615 0.118106 NA	0.103016 0.106497 0.111678	0.11305 0.113427

NA	NA	NA	0.121767 0.097364 NA	NA	NA

0.1382 0.155514 NA	0.095854 0.101794 0.132015 0.132489 0.134259

NA	NA	NA	NA	NA	NA	NA	NA NA	NA	NA	NA	NA	NA	NA	NA

NA	NA	NA	NA	NA	0.115982 0.120608 0.119028

NA	NA	NA	NA	NA	0.140313 0.148662 0.144672

NA	NA	NA	NA	NA	0.141551 0.149927 0.145499

NA	NA	NA	NA	NA	0.132025 0.140573	0.13629

0.114789 0.121372 NA	0.109711 0.109605 0.112214 0.112502 0.113348

0.114789 0.121372 NA	0.109711 0.109605 0.112214 0.112502 0.113348

0.133272 0.138523 NA	0.124374 0.128925 0.129147 0.130764 0.132539

0.13313 0.140167 NA	0.123027 0.125445 0.132326 0.132635 0.134551

NA	0.096864 NA	0.100948 NA	0.115267 0.116937 0.115801

NA	NA	NA	NA	NA	0.123858 0.122933	0.1206

NA	NA	NA	0.121661 NA	NA	NA	NA NA	NA	NA	0.121524 NA	NA	NA	NA NA	NA	NA	0.123772 0.096894 NA	NA	NA

0.130035 0.111743 0.172785 0.104617 0.123495 0.110894 0.104313 0.105639

| NA | 0.095868 | | NA | 0.098907 | 0.103474 | 0.097303 | 0.099572 | 0.099635 | |
| --- | --- | --- | --- | --- | --- | --- | --- | --- | --- |
| NA | 0.095868 | | NA | 0.098907 | 0.103474 | 0.097303 | 0.099572 | 0.099635 | |
| NA | 0.095868 | | NA | 0.098907 | 0.103474 | 0.097303 | 0.099572 | 0.099635 | |
| 0.125775 | 0.116926 | | 0.126787 | 0.103127 | 0.126875 | 0.108762 | 0.094698 | 0.096324 | |
| 0.125264 | 0.113536 | | 0.132951 | 0.104345 | 0.127316 | 0.107299 | 0.096174 | 0.097324 | |
| 0.158556 | 0.159996 | | NA | 0.095963 | 0.103957 | 0.175965 | 0.164658 | 0.166024 | |
| 0.174326 | 0.168159 | | NA | 0.103887 | 0.107145 | 0.187704 | 0.18234 | 0.184483 | |
| 0.196057 | 0.186199 | | NA | 0.104961 | 0.110938 | 0.206877 | 0.200904 | 0.202999 | |
| 0.172137 | 0.173774 | | NA | 0.10673 | 0.114979 | 0.189685 | 0.17531 | 0.177657 | |
| 0.172717 | 0.160564 | | NA | 0.103147 | 0.10672 | 0.178459 | 0.176036 | 0.176494 | |
| 0.185435 | 0.168418 | | 0.121554 | 0.102107 | 0.10921 | 0.185959 | 0.187016 | 0.187413 | |
| 0.174198 | 0.171714 | | NA | 0.09872 | 0.098766 | 0.200645 | 0.195613 | 0.197634 | |
| 0.191118 | 0.167655 | | 0.111542 | 0.112256 | 0.118705 | 0.177649 | 0.180532 | 0.181288 | |
| 0.191526 | 0.176799 | | NA | 0.107665 | 0.111402 | 0.184169 | 0.186255 | 0.188095 | |
| 0.203282 | 0.183984 | | 0.113164 | 0.111995 | 0.117808 | 0.194801 | 0.195894 | 0.197919 | |
| NA | NA |  | NA | 0.124031 | 0.098538 | NA | NA | NA |  |
| 0.10899 | 0.115212 | | NA | 0.101722 | 0.105323 | 0.104478 | 0.10437 | 0.102979 | |
| 0.201148 | 0.187514 | | NA | 0.110082 | 0.113592 | 0.194534 | 0.194003 | 0.196083 | |
| 0.208545 | 0.194123 | | NA | 0.111521 | 0.115999 | 0.200083 | 0.20156 | 0.204064 | |
| 0.159812 | 0.142265 | | NA | 0.109625 | 0.11421 | 0.148994 | 0.15458 | 0.155688 | |
| 0.205651 | 0.202416 | | NA | 0.103942 | 0.109219 | 0.216761 | 0.211269 |  | 0.2144 |
| NA | NA |  | NA | NA | NA | NA | NA | NA |  |
| NA | NA |  | NA | 0.122761 | 0.094642 | NA | NA | NA |  |
| 0.202851 |  | 0.1968 | NA | 0.10608 | 0.110668 | 0.206553 | 0.206195 | 0.208369 | |
| 0.15976 | 0.140944 | | NA | 0.115442 | 0.126611 | 0.148646 | 0.148106 | 0.14924 | |
| 0.161582 | 0.159948 | | NA | 0.10075 | 0.106389 | 0.177098 | 0.169736 | 0.171033 | |
| 0.211092 | 0.194644 | | NA | 0.110963 | 0.115868 | 0.205246 | 0.204704 | 0.206646 | |
| 0.195072 | 0.18255 | | NA | 0.107351 | 0.109499 | 0.189365 | 0.188948 | 0.191051 | |
| 0.165694 | 0.14615 | | NA | 0.10589 | 0.107105 | 0.151262 | 0.153721 | 0.154622 | |
| 0.165694 | 0.14615 | | NA | 0.10589 | 0.107105 | 0.151262 | 0.153721 | 0.154622 | |
| 0.20945 | 0.198643 | | NA | 0.107639 | 0.11315 | 0.211372 | 0.208969 | 0.211082 | |
| 0.174028 | 0.162176 | | NA | 0.106763 | 0.113279 | 0.16803 | 0.167404 | 0.168725 | |
| 0.156487 | 0.146776 | | NA | NA | 0.100226 | 0.171973 | 0.163118 | 0.163165 | |
| 0.155575 | 0.159774 | | NA | 0.100736 | 0.105465 | 0.166491 | 0.162395 | 0.163769 | |
| NA | NA |  | NA | 0.126927 | 0.093149 | NA | NA | NA |  |
| 0.104993 |  | 0.1101 | NA | 0.102199 | 0.109287 | 0.106693 | 0.109065 | 0.110101 | |
| 0.195479 | 0.193172 | | NA | 0.102512 | 0.104779 | 0.204833 | 0.203028 | 0.205177 | |
| 0.212836 | 0.203175 | | NA | 0.107962 | 0.112712 | 0.216004 | 0.213324 | 0.215994 | |
| 0.166087 | 0.144336 | | 0.158491 | 0.110662 | 0.114766 | 0.150962 | 0.156952 | 0.157457 | |

| NA | 0.101334 | NA | 0.099426 | 0.108578 | 0.101688 | 0.106912 | 0.108051 |
| --- | --- | --- | --- | --- | --- | --- | --- |
| NA | 0.103077 | NA | 0.103055 | 0.108713 | 0.103675 | 0.106911 | 0.107633 |
| 0.101092 | 0.107352 | NA | 0.102088 | 0.112001 | 0.107853 | 0.11327 | 0.114168 |
| NA | 0.098379 | NA | NA | 0.101692 | 0.094748 | 0.100832 | 0.101202 |
| NA | NA | NA | NA | NA | NA | NA | NA |
| NA | NA | NA | NA | NA | NA | NA | NA |
| NA | NA | NA | NA | NA | NA | NA | NA |
| NA | NA | NA | 0.090322 | NA | NA | NA | NA |
| NA | NA | NA | 0.095103 | NA | NA | NA | NA |
| NA | NA | NA | 0.094422 | NA | NA | NA | NA |
| NA | NA | NA | NA | NA | NA | NA | NA |
| NA | NA | NA | NA | NA | NA | NA | NA |
| NA | NA | NA | NA | NA | NA | NA | NA |
| NA | NA | NA | NA | NA | NA | NA | NA |
| NA | NA | NA | 0.094235 | NA | NA | NA | NA |
| NA | NA | NA | 0.096768 | NA | NA | NA | NA |
| NA | NA | NA | 0.094391 | NA | NA | NA | NA |
| NA | NA | NA | 0.093954 | NA | NA | NA | NA |
| NA | NA | NA | 0.097844 | 0.092791 | 0.092266 | 0.092868 | 0.093045 |
| NA | NA | NA | 0.096081 | NA | NA | NA | NA |
| NA | NA | NA | NA | NA | NA | NA | NA |
| 0.209541 | 0.189083 | 0.123661 | 0.111691 | 0.119631 | 0.200611 | 0.198343 | 0.200457 |
| NA | 0.10038 | NA | 0.089471 | NA | 0.096608 | 0.09456 | 0.095395 |
| NA | 0.10038 | NA | 0.089471 | NA | 0.096608 | 0.09456 | 0.095395 |
| NA | NA | NA | NA | NA | NA | NA | NA |
| NA | NA | NA | NA | NA | NA | NA | NA |
| NA | NA | NA | NA | NA | NA | NA | NA |
| NA | 0.099224 | NA | NA | NA | 0.095236 | 0.095171 | 0.097186 |
| NA | NA | NA | NA | NA | NA | NA | NA |
| NA | NA | NA | NA | NA | NA | NA | NA |
| NA | NA | NA | NA | NA | NA | NA | NA |
| NA | NA | NA | NA | NA | NA | NA | NA |
| NA | NA | NA | 0.122445 | NA | NA | NA | NA |
| NA | NA | NA | 0.123255 | 0.094094 | NA | NA | NA |
| NA | NA | NA | 0.127796 | 0.09429 | NA | NA | NA |
| NA | NA | NA | 0.127895 | 0.094281 | NA | NA | NA |
| 0.096235 | 0.099131 | NA | 0.110211 | 0.111852 | 0.097979 | 0.100941 | 0.101409 |
| 0.114979 | 0.112795 | 0.125606 | 0.108253 | 0.119884 | 0.106105 | 0.10891 | 0.108433 |
| 0.114979 | 0.112795 | 0.125606 | 0.108253 | 0.119884 | 0.106105 | 0.10891 | 0.108433 |

| 0.134359 | 0.11791 | 0.145845 | 0.111691 | 0.123459 | | 0.109276 | 0.113331 | 0.113088 |
| --- | --- | --- | --- | --- | --- | --- | --- | --- |
| 0.107334 | 0.101214 | NA | NA | NA |  | NA | NA | NA |
| 0.124572 | 0.119993 | 0.141209 | 0.10984 | 0.119141 | | 0.114036 | 0.113358 | 0.113939 |
| 0.099274 | 0.100651 | 0.115534 | 0.11397 | 0.120332 | | 0.096907 | 0.102783 | 0.101848 |
| 0.114158 | 0.112221 | NA | 0.111749 | 0.11882 | | 0.116344 | 0.115013 | 0.116202 |
| NA | NA | NA | NA | NA |  | 0.123103 | 0.125253 | 0.121716 |
| NA | NA | NA | NA | NA |  | NA | NA | NA |
| NA | NA | NA | NA | NA |  | NA | NA | NA |
| NA | 0.091385 | NA | 0.096584 | 0.101239 | | NA | 0.094364 | 0.093886 |
| NA | 0.09081 | NA | NA |  | 0.0966 | 0.090306 | 0.093412 | 0.092441 |
| NA | 0.095827 | NA | 0.10234 | 0.108103 | | 0.094647 | 0.099071 | 0.098881 |
| NA | NA | NA | 0.128015 | 0.095904 | | NA | NA | NA |
| NA | NA | NA | 0.091075 | NA |  | NA | NA | NA |
| NA | NA | NA | NA | NA |  | NA | NA | NA |
| NA | NA | NA | NA | NA |  | NA | NA | NA |
| NA | NA | NA | NA | NA |  | NA | NA | NA |
| NA | NA | NA | 0.127643 | 0.093669 | | NA | NA | NA |
| NA | NA | NA | 0.120553 | 0.092746 | | NA | NA | NA |
| NA | NA | NA | 0.122345 | 0.094336 | | NA | NA | NA |
| NA | NA | NA | 0.09043 | NA |  | NA | NA | NA |
| NA | NA | NA | 0.096474 | NA |  | NA | NA | NA |
| NA | NA | NA | 0.126297 | 0.098478 | | NA | NA | NA |
| NA | NA | NA | 0.127919 | 0.09294 | | NA | NA | NA |
| NA | NA | NA | 0.133786 | 0.096843 | | NA | NA | NA |
| NA | NA | NA | NA | NA |  | NA | NA | NA |
| NA | NA | NA | 0.094214 | NA |  | NA | NA | NA |
| NA | NA | NA | 0.091953 | NA |  | NA | NA | NA |
| NA | NA | NA | 0.100789 | NA |  | NA | NA | NA |
| NA | NA | NA | 0.127144 | 0.09291 | | NA | NA | NA |
| NA | NA | NA | 0.127205 | 0.092989 | | NA | NA | NA |
| NA | NA | NA | 0.108134 | 0.101016 | | NA | NA | NA |
| NA | NA | NA | 0.128564 | 0.095346 | | NA | NA | NA |
| NA | NA | NA | 0.096524 | NA |  | NA | NA | NA |
| NA | NA | NA | 0.096212 | NA |  | NA | NA | NA |
| NA | NA | NA | 0.09303 | NA |  | NA | NA | NA |
| NA | NA | NA | 0.100268 | NA |  | NA | NA | NA |
| NA | NA | NA | NA | NA |  | NA | NA | NA |
| NA | NA | NA | 0.094263 | NA |  | NA | NA | NA |
| NA | NA | NA | 0.099177 | NA |  | NA | NA | NA |

| NA |  | NA | NA | 0.132744 | 0.101715 | NA | NA | NA |  |
| --- | --- | --- | --- | --- | --- | --- | --- | --- | --- |
| NA |  | NA | NA | 0.090807 | NA | NA | NA | NA |  |
| NA |  | NA | NA | 0.127777 | 0.093624 | NA | NA | NA |  |
| NA |  | NA | NA | NA | NA | NA | NA | NA |  |
| NA |  | NA | NA | NA | NA | NA | NA | NA |  |
| NA |  | NA | NA | 0.097558 | NA | NA | NA | NA |  |
| NA |  | NA | NA | 0.092184 | NA | NA | NA | NA |  |
| NA |  | NA | NA | 0.098167 | NA | NA | NA | NA |  |
| NA |  | NA | NA | 0.128884 | 0.09684 | NA | NA | NA |  |
| NA |  | NA | NA | 0.097096 | NA | NA | NA | NA |  |
| 0.115323 | | 0.121497 | NA | 0.091689 | NA | 0.137825 | 0.127794 | 0.130075 | |
| NA |  | 0.096329 | NA | 0.118114 | NA | 0.095235 | 0.096794 | 0.096714 | |
| NA |  | NA | NA | NA | NA | NA | 0.094404 | NA |  |
| 0.103357 | | 0.111695 | NA | 0.093612 | NA | 0.112901 | 0.109223 | 0.109858 | |
| 0.143069 | | 0.143894 | NA | 0.101296 | NA | 0.129108 | 0.13388 | 0.135608 | |
| 0.108093 | | 0.110992 | NA | 0.09698 | NA | 0.103142 | 0.104664 | 0.10613 | |
| 0.101524 | | 0.096904 | NA | NA | NA | 0.094562 | 0.09828 |  | 0.097 |
| NA |  | 0.095879 | NA | 0.088727 | NA | 0.094787 | 0.094545 | 0.095018 | |
| 0.114108 | | 0.113701 | NA | 0.094392 | NA | 0.110826 | 0.113014 | 0.113855 | |
| 0.102118 | | 0.108545 | NA | NA | NA | 0.102285 | 0.104575 | 0.103573 | |
| NA |  | 0.098515 | NA | 0.100047 | 0.108792 | 0.097845 | 0.102775 | 0.10282 | |
| NA |  | 0.09918 | NA | 0.098915 | 0.104693 | 0.099598 | 0.105411 | 0.106032 | |
| NA |  | 0.103166 | NA | 0.099584 | 0.109779 | 0.104677 | 0.110625 | 0.110994 | |
| NA |  | NA | NA | NA | 0.098302 | NA | 0.097708 | 0.097813 | |
| 0.101189 | | 0.110635 | NA | NA | 0.094695 | 0.099137 | NA | NA |  |
| NA |  | 0.106069 | NA | 0.105301 | 0.109761 | 0.10361 | 0.101902 | 0.101691 | |
|  | 0.1073 | 0.111492 | 0.123536 | NA | 0.099614 | 0.100188 | 0.098459 | 0.098051 | |
| 0.115247 | | 0.109443 | 0.131313 | 0.105762 | 0.109874 | 0.104443 | 0.100621 | 0.101337 | |
| 0.115247 | | 0.109443 | 0.131313 | 0.105762 | 0.109874 | 0.104443 | 0.100621 | 0.101337 | |
| 0.111489 | | 0.108836 | 0.135816 | NA | 0.106453 | 0.099293 | NA | NA |  |
| 0.112881 | | 0.113771 | 0.114818 | 0.098033 | 0.113205 | 0.106363 | 0.089223 | 0.09011 | |
| 0.105105 | | 0.111082 | NA | NA | 0.097591 | 0.098923 | NA | NA |  |
| 0.105105 | | 0.111082 | NA | NA | 0.097591 | 0.098923 | NA | NA |  |
| 0.105105 | | 0.111082 | NA | NA | 0.097591 | 0.098923 | NA | NA |  |
| 0.109123 | | 0.114089 | 0.114786 | 0.096337 | 0.109842 | 0.107128 | 0.090465 | 0.091578 | |
| 0.122295 | | 0.114176 | 0.146331 | 0.113249 | 0.133277 | 0.11262 | 0.103296 | 0.104939 | |
|  | 0.1051 | 0.106859 | 0.115534 | NA | NA | 0.094613 | NA | NA |  |
| NA |  | NA | NA | 0.135088 | 0.099832 | NA | NA | NA |  |
| 0.097385 | | 0.093614 | NA | 0.105377 | 0.098277 | NA | NA | NA |  |

| NA | NA | NA | 0.134709 | 0.097616 | NA | NA | NA |
| --- | --- | --- | --- | --- | --- | --- | --- |
| NA | NA | NA | 0.122743 | 0.091373 | NA | NA | NA |
| NA | NA | NA | 0.125517 | 0.08834 | NA | NA | NA |
| NA | NA | NA | 0.122179 | NA | NA | NA | NA |
| 0.116266 | 0.112024 | NA | 0.115163 | 0.133816 | 0.109682 | 0.116028 | 0.116742 |
| 0.108797 | 0.109575 | NA | 0.110116 | 0.118631 | 0.107731 | 0.113222 | 0.114059 |
| 0.09806 | 0.106465 | NA | 0.104005 | 0.113586 | 0.102578 | 0.10642 | 0.106173 |
| 0.119792 | 0.11522 | NA | 0.120971 | 0.129209 | 0.113795 | 0.119263 | 0.120564 |
| 0.100277 | 0.102523 | NA | 0.096278 | 0.103215 | 0.101826 | 0.109178 | 0.109505 |
| 0.103928 | 0.105042 | NA | 0.104317 | 0.108306 | 0.104558 | 0.111403 | 0.111905 |
| 0.117061 | 0.12106 | NA | 0.102229 | 0.106643 | 0.112778 | 0.114009 | 0.114402 |
| 0.161243 | 0.116044 | 0.212515 | 0.123149 | 0.149606 | 0.113934 | 0.114857 | 0.115886 |
| 0.176516 | 0.121735 | 0.189346 | 0.136011 | 0.155201 | 0.123031 | 0.128791 | 0.129827 |
| NA | 0.103558 | NA | 0.102538 | 0.108958 | 0.096967 | 0.101495 | 0.10177 |
| NA | NA | NA | 0.141541 | 0.105885 | NA | NA | NA |
| NA | NA | NA | 0.128718 | 0.095744 | NA | NA | NA |
| 0.110978 | 0.11495 | NA | NA | NA | 0.108004 | 0.108929 | 0.108599 |
| NA | NA | NA | 0.128314 | 0.092273 | NA | NA | NA |
| 0.111513 | 0.110708 | NA | 0.113135 | 0.120436 | 0.107443 | 0.11222 | 0.112517 |
| NA | NA | NA | 0.128924 | 0.101949 | NA | NA | NA |
| NA | NA | NA | 0.128924 | 0.101949 | NA | NA | NA |
| 0.124098 | 0.115553 | 0.143472 | 0.116398 | 0.124363 | 0.11231 | 0.112998 | 0.113623 |
| 0.116982 | 0.124153 | NA | 0.105838 | 0.109655 | 0.116283 | 0.117428 | 0.118156 |
| 0.116982 | 0.124153 | NA | 0.105838 | 0.109655 | 0.116283 | 0.117428 | 0.118156 |
| 0.116982 | 0.124153 | NA | 0.105838 | 0.109655 | 0.116283 | 0.117428 | 0.118156 |
| 0.116982 | 0.124153 | NA | 0.105838 | 0.109655 | 0.116283 | 0.117428 | 0.118156 |
| 0.116507 | 0.117804 | NA | 0.11285 | 0.116475 | 0.118193 | 0.117435 | 0.118394 |
| 0.116507 | 0.117804 | NA | 0.11285 | 0.116475 | 0.118193 | 0.117435 | 0.118394 |
| 0.116507 | 0.117804 | NA | 0.11285 | 0.116475 | 0.118193 | 0.117435 | 0.118394 |
| 0.116507 | 0.117804 | NA | 0.11285 | 0.116475 | 0.118193 | 0.117435 | 0.118394 |
| 0.108599 | 0.110825 | NA | 0.116484 | 0.104553 | 0.111263 | 0.111252 | 0.112447 |
| 0.108599 | 0.110825 | NA | 0.116484 | 0.104553 | 0.111263 | 0.111252 | 0.112447 |
| 0.109633 | 0.109632 | NA | 0.110216 | 0.102727 | 0.110125 | 0.109408 | 0.108878 |
| 0.109633 | 0.109632 | NA | 0.110216 | 0.102727 | 0.110125 | 0.109408 | 0.108878 |
| 0.133281 | 0.131139 | NA | 0.112051 | 0.119023 | 0.120917 | 0.122411 | 0.123182 |
| 0.114314 | 0.112608 | NA | 0.112206 | 0.129715 | 0.108787 | 0.114993 | 0.115429 |
| 0.114314 | 0.112608 | NA | 0.112206 | 0.129715 | 0.108787 | 0.114993 | 0.115429 |
| 0.110011 | 0.11061 | NA | 0.111462 | 0.117935 | 0.110343 | 0.113962 | 0.114814 |
| 0.110011 | 0.11061 | NA | 0.111462 | 0.117935 | 0.110343 | 0.113962 | 0.114814 |

0.1151 0.112749 NA	0.109219 0.123113	0.10879 0.112478	0.11323

0.119098 0.118168 0.132782 0.120861 0.137182 0.113475 0.119042 0.119618

0.107273 0.111924 NA	0.111992 0.122908 0.109028 0.111733 0.112457

0.095873 0.102475 NA	0.098681 0.100107 0.103082 0.103494	0.1044

0.098164 0.106985 NA	0.101825 0.104706 0.110528 0.117469 0.119119

NA	0.097502 NA	0.096315 0.107043 0.096491 0.101109 0.101142

0.102964 0.110181 NA	0.104465 0.110132 0.111175 0.119256 0.120205

0.103111 0.108126 NA	0.166917	0.17474 0.113735 0.121961 0.122945

0.105659 0.109515 NA	0.170883 0.179643 0.115298 0.124015 0.124605

NA	NA	NA	0.120779 0.113195 NA	0.098853 0.098394

0.105107 0.109804 NA	0.172842 0.179836 0.114991 0.123528	0.12435

0.098595 0.101782 NA	0.148369 0.138093 0.105542 0.111122	0.1115

NA	0.095795 NA	NA	NA	NA	NA	NA

0.100905 0.103375 NA	0.096003 0.099398 0.105056 0.104635 0.105629

NA	NA	NA	NA	NA	NA	NA	NA NA	NA	NA	NA	0.096517 NA	NA	NA NA	NA	NA	NA	NA	NA	NA	NA

NA	0.098081 NA	0.096176 0.103029 0.095301 0.094472 0.094947

NA	NA	NA	NA	NA	NA	NA	NA NA	NA	NA	NA	NA	NA	NA	NA NA	NA	NA	NA	NA	NA	NA	NA NA	NA	NA	NA	NA	NA	NA	NA NA	NA	NA	NA	0.100128 NA	NA	NA

NA	0.100403 NA	NA	NA	0.096256 0.093849 0.093972

0.104472	0.10769 NA	NA	0.097267 0.106713	0.10092 0.101556

NA	NA	NA	0.128233 0.094847 NA	NA	NA NA	NA	NA	0.127924 0.094503 NA	NA	NA NA	NA	NA	0.124646 0.098849 NA	NA	NA

0.123364 0.129344 NA	0.111469 0.114541 0.119486 0.120041 0.120658

0.113642 0.110779 0.116019 0.109303 0.124236 0.115606 0.101832 0.102855

0.113642 0.110779 0.116019 0.109303 0.124236 0.115606 0.101832 0.102855

NA	NA	NA	NA	NA	NA	NA	NA

NA	0.0945 NA	NA	0.099685 NA	0.095542 0.095434

NA	NA	NA	NA	NA	NA	NA	NA NA	0.098139 NA	NA	NA	0.094967 NA	NA

0.146974 0.126159 0.135744 0.102274 0.108257 0.131577 0.137396	0.13746

NA	0.09321 NA	NA	NA	0.089956 0.092089 0.091662

NA	NA	NA	NA	NA	NA	NA	NA

NA	0.094707 NA	NA	NA	0.092305 0.094039 0.093194

| NA | 0.091582 | NA | NA | NA |  | 0.091913 | 0.090105 | | 0.089183 | |
| --- | --- | --- | --- | --- | --- | --- | --- | --- | --- | --- |
| NA | NA | NA | NA | NA |  | NA | NA |  | NA |  |
| NA | NA | NA | NA | NA |  | NA | NA |  | NA |  |
| NA | NA | NA | NA | NA |  | NA | NA |  | NA |  |
| NA | NA | NA | NA | NA |  | NA | NA |  | NA |  |
| 0.107052 | 0.108543 | NA | 0.110358 | 0.117099 | | 0.108271 | 0.113485 | | 0.11445 | |
| NA | NA | NA | NA | NA |  | NA | NA |  | NA |  |
| NA | NA | NA | NA | NA |  | NA | NA |  | NA |  |
| NA | NA | NA | NA | NA |  | NA | NA |  | NA |  |
| NA | NA | NA | 0.122261 | NA |  | NA | NA |  | NA |  |
| 0.109875 | 0.108346 | NA | 0.116165 | 0.116112 | | 0.106296 | 0.111035 | | 0.110208 | |
| NA | 0.099852 | NA | 0.101984 | 0.103839 | | 0.098292 | 0.101458 | | 0.101037 | |
| 0.101555 | 0.109186 | NA | 0.101867 | 0.108602 | | 0.108048 | 0.113646 | | 0.11349 | |
| NA | NA | NA | 0.128696 | 0.092074 | | NA | NA |  | NA |  |
| 0.098535 | 0.096968 | NA | 0.105857 | 0.109265 | | 0.09474 | 0.099606 | | 0.098764 | |
| NA | 0.09752 | NA | 0.112214 | 0.09987 | | 0.09659 | 0.097827 | | 0.098545 | |
| NA | 0.098104 | NA | 0.109506 | 0.110639 | | 0.096475 | 0.098632 | | 0.098874 | |
| NA | 0.096933 | NA | 0.105729 | 0.115153 | | 0.096867 | 0.101537 | | 0.100105 | |
| NA | NA | NA | 0.137005 | 0.098117 | | NA | NA |  | NA |  |
| 0.105413 | 0.106177 | 0.137525 | 0.107074 | 0.123252 | | 0.103904 | 0.103984 | | 0.104843 | |
| 0.114033 | 0.111647 | 0.147039 | 0.111647 | 0.124489 | | 0.109602 | 0.106244 | | 0.107207 | |
| NA | 0.098287 | NA | NA | 0.09421 | | 0.099639 | 0.097556 | | 0.096893 | |
| NA | 0.097238 | NA | NA | 0.09632 | | 0.097194 | 0.093747 | | 0.09354 | |
| 0.148289 | 0.123795 | NA | 0.12315 | 0.132891 | | 0.127147 | 0.129728 | | 0.130054 | |
| 0.102757 | 0.108212 | NA | 0.107556 | 0.115841 | | 0.107298 | 0.108558 | | 0.109075 | |
| NA | 0.097856 | NA | NA | NA |  | 0.095468 | 0.094188 | |  | 0.0939 |
| 0.101289 | 0.105058 | NA | 0.097601 | 0.101195 | | 0.106354 | 0.106528 | | 0.107575 | |
| NA | 0.098118 | 0.120757 | 0.100249 | 0.103269 | | 0.099113 | 0.09967 | | 0.099969 | |
| 0.107562 | 0.108923 | NA | 0.118491 |  | 0.1218 | 0.112543 | 0.116108 | | 0.115568 | |
| 0.098552 | 0.100316 | NA | 0.118675 | 0.127582 | | 0.099822 | 0.103612 | | 0.102992 | |
| 0.107534 | 0.109919 | NA | 0.153638 | 0.164979 | | 0.114382 | 0.118955 | | 0.118744 | |
| 0.105351 | 0.105924 | NA | 0.131333 | 0.139351 | | 0.104369 | 0.108376 | | 0.108638 | |
| 0.114679 | 0.112073 | NA | 0.138543 | 0.139756 | | 0.11093 |  | 0.1173 | 0.11852 | |
| 0.102048 | 0.106386 | NA | 0.120351 | 0.129483 | | 0.105472 | 0.106931 | | 0.106767 | |
| 0.10305 | 0.105778 | NA | 0.141276 | 0.150228 | | 0.106012 | 0.107661 | | 0.107637 | |
| NA | 0.098938 | NA | 0.138118 | 0.149568 | | 0.099466 | 0.101086 | | 0.10086 | |
| NA | NA | NA | 0.095844 | NA |  | NA | NA |  | NA |  |
| NA | NA | NA | 0.095844 | NA |  | NA | NA |  | NA |  |
| NA | NA | NA | 0.095844 | NA |  | NA | NA |  | NA |  |

| 0.137348 | 0.158291 | NA | NA |  | NA | 0.131648 | 0.129166 | 0.131055 |
| --- | --- | --- | --- | --- | --- | --- | --- | --- |
| 0.146329 | 0.165006 | NA | NA |  | NA | 0.137963 | 0.134917 | 0.13729 |
| 0.142519 | 0.148188 | NA | NA |  | 0.097999 | 0.124084 | 0.120374 | 0.122514 |
| NA | 0.096916 | NA | NA |  | 0.094442 | 0.095608 | 0.093848 | 0.093995 |
| NA | NA | NA | NA |  | NA | NA | NA | NA |
| NA | NA | NA | NA |  | NA | NA | NA | NA |
| NA | NA | NA | NA |  | NA | NA | NA | NA |
| NA | 0.09383 | NA | NA |  | NA | 0.096456 | 0.098084 | 0.097951 |
| 0.100655 | 0.104923 | NA | 0.108425 | | 0.120108 | 0.104842 | 0.105237 | 0.10533 |
| 0.101428 | 0.107548 | NA | 0.113068 | | 0.120291 | 0.102357 | 0.104894 | 0.105195 |
| 0.101326 | 0.104665 | NA |  | 0.1163 | 0.120062 | 0.102266 | 0.106598 | 0.106539 |
| 0.101326 | 0.104665 | NA |  | 0.1163 | 0.120062 | 0.102266 | 0.106598 | 0.106539 |
| 0.106452 | 0.107026 | NA | 0.112049 | | 0.117348 | 0.104229 | 0.108183 | 0.107797 |
| NA | 0.099133 | NA | NA |  | 0.101828 | 0.098027 | 0.105171 | 0.105269 |
| NA | 0.099133 | NA | NA |  | 0.101828 | 0.098027 | 0.105171 | 0.105269 |
| NA | NA | NA | 0.117172 | | 0.122175 | 0.090612 | 0.093622 | 0.092933 |
| NA | NA | NA | 0.119838 | | 0.122905 | NA | NA | NA |
| NA | NA | NA | 0.125063 | | 0.131981 | NA | NA | NA |
| 0.110317 | 0.111947 | 0.114129 | 0.094186 | | 0.109418 | 0.104963 | NA | NA |
| 0.161066 | 0.147232 | NA | 0.104218 | | 0.10524 | 0.154765 | 0.159399 | 0.160268 |
| 0.156717 | 0.166028 | NA | NA |  | NA | 0.209431 | 0.198076 | 0.202419 |
| 0.115071 | 0.125734 | NA | 0.089976 | | 0.091579 | 0.14887 | 0.131388 | 0.132666 |
| 0.143498 | 0.13888 | NA | 0.097356 | | 0.101134 | 0.163736 | 0.154729 | 0.156596 |
| 0.149772 | 0.155647 | NA | 0.092613 | | NA | 0.20078 | 0.191273 | 0.194109 |
| 0.138799 | 0.144366 | NA | 0.092343 | | NA | 0.181876 | 0.169234 | 0.171696 |
| 0.094807 | 0.093376 | NA | 0.100045 | | 0.094996 | 0.103023 | 0.107231 | 0.106918 |
| 0.10118 | 0.108448 | NA | 0.090263 | | NA | 0.110001 | 0.109085 | 0.109478 |
| 0.09927 | 0.098819 | NA | 0.106938 | | 0.104289 | 0.104423 | 0.108106 | 0.108154 |
| NA | 0.100398 | NA | 0.099852 | | 0.09867 | 0.099858 | 0.102049 | 0.102053 |
| 0.119273 | 0.11454 | 0.145975 | 0.116049 | | 0.131357 | 0.111023 | 0.115124 | 0.115141 |
| 0.115472 | 0.11486 | NA | 0.118236 | | 0.12616 | 0.114083 | 0.119424 | 0.119824 |
| 0.101999 | 0.099458 | NA | NA |  | NA | NA | NA | NA |
| 0.091957 | NA | NA | 0.101137 | | 0.093719 | NA | NA | NA |
| NA | NA | NA | 0.097968 | | NA | NA | NA | NA |
| NA | NA | NA | 0.095138 | | NA | NA | NA | NA |
| 0.10637 | 0.103913 | 0.142592 | 0.102927 | | 0.116136 | 0.100619 | 0.103539 | 0.103885 |
| 0.109292 | 0.11112 | 0.138537 | 0.113503 | | 0.125171 | 0.109094 | 0.110909 | 0.111495 |
| 0.114589 | 0.110219 | 0.173379 | 0.113536 | | 0.131972 | 0.105372 | 0.109957 | 0.110455 |
| NA | NA | 0.146659 | NA |  | 0.098603 | NA | NA | NA |

| 0.112069 | 0.110591 | | 0.144315 | 0.111959 | 0.123662 | | 0.107801 | 0.109877 | 0.110652 |
| --- | --- | --- | --- | --- | --- | --- | --- | --- | --- |
| 0.117478 | 0.117126 | | 0.134672 | 0.119144 | 0.136757 | | 0.113061 | 0.118524 | 0.119264 |
| 0.11341 | 0.115974 | | NA | 0.098609 | 0.101517 | | 0.110683 | 0.11215 | 0.11262 |
| 0.111404 | 0.115807 | | NA | 0.095842 | 0.099233 | | 0.10882 | 0.111741 | 0.111723 |
| 0.10673 | 0.110149 | | NA | NA | NA |  | 0.104799 | 0.108373 | 0.108347 |
| 0.103128 | 0.110193 | | NA | 0.102228 | 0.111534 | | 0.102795 | 0.107275 | 0.109277 |
| NA | 0.097373 | | NA | 0.127099 | 0.132336 | | 0.099712 | 0.106963 | 0.106067 |
| 0.109947 | 0.115936 | | NA | 0.097449 | 0.100664 | | 0.110227 | 0.113236 | 0.113323 |
| 0.105652 | 0.112114 | | NA | NA | NA |  | 0.105112 | 0.108556 | 0.108283 |
| NA | NA |  | NA | 0.137289 | 0.099603 | | NA | NA | NA |
| NA | NA |  | NA | 0.138981 |  | 0.1021 | NA | NA | NA |
| NA | NA |  | NA | 0.134534 | 0.096761 | | NA | NA | NA |
| 0.157249 | 0.129525 | | 0.178066 | 0.124391 | 0.13947 | | 0.125839 | 0.126764 | 0.128331 |
| 0.122417 | 0.116289 | | 0.143135 | 0.115279 | 0.128237 | | 0.11103 | 0.113264 | 0.112994 |
| NA | 0.097381 | | NA | NA | NA |  | 0.093952 | 0.098594 | 0.098207 |
| 0.118214 | 0.11139 | | 0.122103 | 0.106674 | 0.117177 | | 0.107686 | 0.107484 | 0.108164 |
| NA | 0.096908 | | NA | NA | NA |  | NA | 0.097674 | 0.097006 |
| 0.112287 | 0.111988 | | 0.128953 | NA | 0.098994 | | 0.098154 | NA | 0.094166 |
| 0.106566 | 0.103999 | | 0.138758 | 0.106891 | 0.118683 | | 0.101153 | 0.103432 | 0.103571 |
| NA | NA |  | NA | 0.122563 | NA |  | NA | NA | NA |
| 0.129307 |  | 0.1211 | 0.140291 | 0.129945 | 0.144846 | | 0.118055 | 0.124598 | 0.12485 |
| NA | NA |  | NA | 0.105689 | 0.100992 | | 0.097953 | 0.099544 | 0.099785 |
| 0.100214 | NA |  | 0.136963 | NA | 0.108718 | | NA | NA | NA |
| NA | NA |  | 0.134685 | 0.096508 | 0.109498 | | NA | NA | NA |
| 0.11454 | 0.113955 | | 0.150685 | 0.116631 | 0.127139 | | 0.106813 | 0.10616 | 0.105945 |
| 0.111981 | 0.114893 | | 0.140291 | 0.110873 | 0.122903 | | 0.110885 | 0.100985 | 0.102675 |
| 0.114636 | 0.114385 | | 0.151844 | 0.121166 | 0.131682 | | 0.109548 | 0.108143 | 0.107719 |
| 0.111686 | 0.110776 | | 0.139932 | 0.113797 | 0.122872 | | 0.106062 | 0.109244 | 0.108838 |
| 0.101766 | 0.107715 | | NA | 0.097033 | 0.111171 | | 0.10714 | 0.09664 | 0.098755 |
| 0.120006 | 0.113225 | | NA | 0.114354 | 0.120855 | | 0.112716 | 0.114373 | 0.114796 |
| 0.119523 | 0.119145 | | 0.122288 | 0.104776 | 0.121758 | | 0.117867 | 0.106066 | 0.107194 |
| 0.128027 | 0.11934 | | 0.160138 | 0.125172 | 0.137254 | | 0.116146 | 0.12235 | 0.123202 |
| 0.105376 | 0.108227 | | NA | 0.107664 | 0.110649 | | 0.106981 | 0.108387 | 0.108686 |
| NA | 0.108074 | | NA | 0.111045 | 0.108879 | | 0.106033 | 0.106669 | 0.107067 |
| 0.124136 | 0.105644 | | 0.150253 | 0.100838 | 0.113247 | | 0.104914 | 0.101989 | 0.101589 |
| 0.104528 | 0.103213 | | NA | 0.099238 | 0.097549 | | 0.112571 | 0.110628 | 0.11145 |
| 0.111862 | 0.112781 | | NA | 0.099268 | 0.093315 | | 0.125693 | 0.125138 | 0.125909 |
| 0.111721 | 0.11102 | | 0.113459 | 0.109252 | 0.122706 | | 0.108861 | 0.107436 | 0.107838 |
| 0.131296 | 0.111151 | | 0.168162 | 0.125875 | 0.125823 | | 0.108459 | 0.113935 | 0.114545 |

| 0.115038 | 0.114278 | 0.135729 | | 0.11488 | | 0.126564 | | 0.11381 | 0.117094 | 0.117401 | |
| --- | --- | --- | --- | --- | --- | --- | --- | --- | --- | --- | --- |
| 0.112851 | 0.11375 | 0.123061 | | 0.113152 | | 0.121795 | | 0.115114 | 0.112522 | 0.11234 | |
| 0.110971 | 0.111745 | NA |  | 0.109126 | | 0.115927 | | 0.11319 | 0.115413 | 0.115995 | |
| 0.108526 | 0.113557 | NA |  | 0.105454 | | 0.11663 | | 0.104953 | 0.104122 | 0.104019 | |
| 0.142267 | 0.119053 | NA |  | 0.123968 | | 0.111822 | | 0.137066 | 0.136334 | 0.135894 | |
| NA | NA | NA |  | 0.108841 | | NA |  | NA | NA | NA |  |
| 0.112539 | 0.110486 | NA |  | 0.116704 | | 0.123387 | | 0.111573 | 0.115539 | 0.116196 | |
| NA | 0.103419 | NA |  | 0.110467 | | 0.120455 | | 0.101357 | 0.10687 | 0.106875 | |
| 0.113268 | 0.112687 | 0.148148 | | 0.115744 | | 0.127927 | | 0.109926 | 0.112457 | 0.112387 | |
| 0.109071 | 0.110782 | NA |  | 0.108961 | | 0.113608 | | 0.107227 | 0.106819 | 0.106881 | |
| 0.108498 | 0.109974 | 0.123876 | | 0.108253 | | 0.125357 | | 0.109933 | 0.105674 | 0.106196 | |
| NA | NA | NA |  | 0.098786 | | NA |  | NA | NA | NA |  |
| 0.108104 | 0.107677 | NA |  | 0.108157 | | 0.108815 | | 0.10833 | 0.112435 | 0.111853 | |
| 0.118098 | 0.109748 | 0.143931 | | 0.097941 | | 0.114062 | | 0.104553 | 0.095564 | 0.096505 | |
| 0.124135 | 0.118626 | 0.143932 | | 0.100308 | | 0.114631 | | 0.110275 | 0.100611 | 0.102155 | |
| 0.127957 | 0.119314 | 0.154586 | |  | 0.1322 | 0.147114 | | 0.115534 | 0.12307 | 0.123928 | |
| 0.101899 | 0.101581 |  | 0.1317 | 0.106374 | | 0.113035 | | 0.09632 | 0.098596 | 0.098811 | |
| 0.119957 | 0.113887 | 0.137427 | | 0.114881 | | 0.128967 | | 0.104963 | 0.111203 | 0.112214 | |
| NA | NA | NA |  | 0.102967 | | NA |  | NA | NA | NA |  |
| 0.100162 | 0.105365 | NA |  | 0.100533 | | 0.105529 | | 0.105793 | 0.105606 | 0.105433 | |
| 0.112135 | 0.120664 | NA |  | 0.112207 | | 0.124608 | | 0.113757 | 0.104703 | 0.105229 | |
| NA | 0.098224 | NA |  | NA |  | NA |  | 0.110762 | 0.099174 | 0.099066 | |
| 0.100715 | 0.104459 | 0.129486 | | 0.113588 | | 0.120587 | | 0.101956 | 0.107633 | 0.107638 | |
| 0.10916 | 0.108782 | NA |  | 0.093015 | | 0.099204 | | 0.118999 | 0.110133 | 0.110531 | |
| NA | 0.108333 | NA |  | 0.112529 | |  | 0.1243 | 0.105754 | 0.107552 | 0.107604 | |
| 0.105854 | 0.106504 | NA |  | 0.099624 | | 0.09579 | | 0.114174 | 0.112126 | 0.112231 | |
| 0.101357 | 0.105644 | NA |  | 0.117037 | | 0.123127 | | 0.109824 | 0.109815 | 0.110164 | |
| 0.109131 | 0.11033 | 0.12719 | | 0.103051 | | 0.117483 | | 0.108738 | 0.093726 | 0.095209 | |
| 0.102932 | 0.103245 | NA |  | 0.106149 | | 0.114087 | | 0.101304 | 0.105257 | 0.105276 | |
| NA | 0.102232 | NA |  | 0.109287 | | 0.104562 | | 0.101423 | 0.100966 | 0.101324 | |
| 0.10761 | 0.109045 | NA |  | 0.095779 | | 0.101688 | | 0.117975 | 0.108492 | 0.109277 | |
| 0.138571 | 0.118236 | 0.177066 | | 0.122389 | | 0.13683 | | 0.113558 | 0.112579 | 0.112885 | |
| 0.103668 | 0.105068 | 0.122696 | | 0.106338 | | 0.11739 | | 0.105154 | 0.101886 |  | 0.1029 |
| 0.111383 | 0.115069 | NA |  | NA |  | NA |  | 0.107132 | 0.105555 | 0.106796 | |
| NA | 0.099739 | NA |  | 0.097307 | | 0.10943 | | 0.095776 | 0.096764 | 0.09544 | |
| 0.104243 | 0.105477 | NA |  | 0.108254 | | 0.116007 | | 0.10115 | 0.104758 | 0.104398 | |
| 0.108498 | 0.111082 | NA |  | 0.10835 | | 0.110337 | | 0.109819 | 0.110743 | 0.111376 | |
| 0.109419 | 0.113386 | 0.132554 | | 0.105093 | | 0.122299 | | 0.114842 | 0.101121 | 0.102427 | |

normal_hu osteoclast plasma_ce podocyte.. preadipoc precursor_ precursor_ regulatory

0.103733 0.111303 0.106162 0.116056 0.110272 0.113879	0.11877 0.113732

NA	NA	NA	NA	NA	NA	NA	NA

0.112914 0.113061 0.119425 0.118011 0.111803 0.113497 0.114275 0.117906

0.114652 0.104104 0.118304 0.110781 0.108716 0.108649 0.121503 0.112569

0.109216 0.099007 0.138297 0.113051 0.096062 0.102629 0.106285 0.112513

0.118067	0.11673 0.120151 0.120119	0.1217 0.131122 0.146993 0.123211

0.112246 0.113491 0.132351	0.11948 0.109049 0.112775 0.114869 0.118755

0.101168 0.100766 0.123951 0.110365 0.104542	0.10474 0.109408	0.11115

0.101168 0.100766 0.123951 0.110365 0.104542	0.10474 0.109408	0.11115

0.109709 0.120311 0.101679 0.120852 0.136574 0.099512 NA	0.102

0.10807 0.112738	0.11247 0.115042 0.123788 0.119782	0.12856 0.111303

0.109628 0.101794 0.114792 0.108671 0.103271 0.111796 0.119914 0.108478

0.101593 0.120003 0.111194 0.145314 0.115408 0.101065 0.100707 0.124864

0.110513	0.11908 0.113317 0.152082 0.122492 0.105082 0.106863 0.126396

0.093336 NA	NA	NA	NA	NA	NA	NA NA	NA	NA	0.092795 NA	NA	NA	NA

0.096639 0.102725 0.096464	0.12122 0.102865 0.093744 NA	0.112523

0.096639 0.102725 0.096464	0.12122 0.102865 0.093744 NA	0.112523

0.097086 0.169796	0.10172	0.09846 0.098761 0.152138 0.132342 0.133462

0.118421 0.109258 0.133475 0.118302 0.104838 0.113656	0.11413 0.121812

NA	NA	NA	0.093181 NA	NA	NA	NA

0.095867 0.137331 0.111069 0.107165 0.099327 0.143836 0.146011 0.142655

0.118123 NA	NA	NA	NA	NA	NA	NA

0.13148 NA	NA	0.100661 NA	NA	NA	NA

0.094395 0.112835 NA	NA	0.096763 NA	NA	NA NA	0.131703 NA	NA	0.096021 NA	NA	NA NA	0.132915 NA	NA	0.097426 NA	NA	NA NA	0.12399 NA	NA	NA	NA	NA	NA

0.114446 0.110732 0.125408 0.117143 0.112824 0.115227	0.11344 0.119554

0.114446 0.110732 0.125408 0.117143 0.112824 0.115227	0.11344 0.119554

0.130916 0.126729 0.153308 0.135313	0.12317 0.129591 0.134514 0.140208

0.133928 0.131195 0.153456 0.136387 0.125085 0.132565 0.133656 0.140881

0.103643 0.117356 0.096747 0.113497 0.124637 0.097989 NA	0.098718

NA	0.122064 NA	NA	0.100282 NA	NA	NA NA	NA	NA	NA	NA	NA	NA	NA NA	NA	NA	NA	NA	NA	NA	NA NA	NA	NA	NA	NA	NA	NA	NA

0.095901 0.103756 0.094066 0.102238 0.104839	0.10592 0.118044 0.104028

| 0.093658 | | 0.095173 | 0.117942 | 0.106414 | 0.122274 | 0.139074 | 0.140005 | 0.095567 |
| --- | --- | --- | --- | --- | --- | --- | --- | --- |
| 0.093658 | | 0.095173 | 0.117942 | 0.106414 | 0.122274 | 0.139074 | 0.140005 | 0.095567 |
| 0.093658 | | 0.095173 | 0.117942 | 0.106414 | 0.122274 | 0.139074 | 0.140005 | 0.095567 |
| 0.10207 | | 0.103491 | 0.097119 | 0.117246 | 0.113451 | 0.105981 | 0.098758 | 0.11638 |
| 0.104014 | | 0.10181 | 0.098528 | 0.112839 | 0.110966 | 0.105664 | 0.100346 | 0.112656 |
| 0.108498 | | 0.176115 | 0.111428 | 0.107465 | 0.101947 | 0.171864 | 0.166404 | 0.149137 |
| 0.126848 | | 0.192438 | 0.115783 | 0.124716 | 0.111969 | 0.181272 | 0.183604 | 0.165452 |
| 0.124844 | | 0.21198 | 0.123537 | 0.126354 | 0.114851 | 0.20052 | 0.204059 | 0.181115 |
| 0.116652 | | 0.192612 | 0.114477 | 0.121193 | 0.113371 | 0.185277 | 0.183171 | 0.164376 |
| 0.132782 | | 0.182537 | 0.117764 | 0.127115 | 0.113303 | 0.172448 | 0.177994 | 0.160817 |
| 0.124924 | | 0.191242 | 0.121408 | 0.125467 | 0.112247 | 0.180701 | 0.190347 | 0.167773 |
| 0.127078 | | 0.210874 | 0.118203 | 0.120085 | 0.108347 | 0.193503 | 0.190978 | 0.168598 |
| 0.120331 | | 0.178007 | 0.118804 | 0.130306 | 0.120031 | 0.174306 | 0.18809 | 0.165661 |
| 0.118256 | | 0.187919 | 0.120762 | 0.126081 | 0.116759 | 0.182185 | 0.195086 | 0.176815 |
| 0.122507 | | 0.197377 | 0.123712 | 0.130355 | 0.119253 | 0.19263 | 0.206949 | 0.181841 |
| NA |  | NA | NA | NA | NA | NA | NA | NA |
| 0.108394 | | 0.100301 | 0.132543 | 0.111142 | 0.103318 | 0.104524 | 0.100231 | 0.112947 |
| 0.11735 | | 0.200337 | 0.121527 | 0.128839 | 0.118574 | 0.193137 | 0.206537 | 0.184591 |
| 0.117654 | | 0.207449 | 0.126924 | 0.129842 | 0.118935 | 0.202126 | 0.217535 | 0.194127 |
| 0.108853 | | 0.155098 | 0.114713 | 0.123601 | 0.118072 | 0.149589 | 0.161461 | 0.145561 |
| 0.117659 | | 0.221001 | 0.126683 | 0.123477 | 0.111379 | 0.216673 | 0.223226 | 0.195852 |
|  | 0.1057 | NA | 0.102142 | 0.103468 | NA | NA | NA | NA |
| NA |  | NA | NA | NA | NA | NA | NA | NA |
| 0.117737 | | 0.212142 | 0.126892 | 0.127447 | 0.11506 | 0.20657 | 0.218422 | 0.195481 |
|  | 0.1078 | 0.148911 | 0.114224 | 0.119514 | 0.118668 | 0.144685 | 0.149434 | 0.137852 |
| 0.110583 | | 0.181217 | 0.109301 | 0.115127 | 0.108275 | 0.169885 | 0.166787 | 0.154073 |
| 0.11994 | | 0.208918 | 0.125022 | 0.130502 | 0.118809 | 0.20295 | 0.217117 | 0.192298 |
| 0.114872 | | 0.197583 | 0.120551 | 0.125577 | 0.115597 | 0.189823 | 0.19996 | 0.18241 |
| 0.112177 | | 0.154045 | 0.110992 | 0.121261 | 0.111729 | 0.148417 | 0.159851 | 0.14872 |
| 0.112177 | | 0.154045 | 0.110992 | 0.121261 | 0.111729 | 0.148417 | 0.159851 | 0.14872 |
| 0.118269 | | 0.216987 | 0.124578 | 0.12746 | 0.115671 | 0.210261 | 0.220215 | 0.195501 |
| 0.108129 | | 0.168487 | 0.115812 | 0.119544 | 0.112776 | 0.16513 | 0.176381 | 0.158469 |
| 0.10247 | | 0.170269 | 0.109163 | 0.105252 | 0.103162 | 0.15832 | 0.155494 | 0.139363 |
| 0.113711 | | 0.169977 | 0.119344 | 0.117839 | 0.108109 | 0.164303 | 0.165636 | 0.157881 |
| NA |  | NA | NA | 0.096841 | NA | NA | NA | NA |
| 0.10976 | | 0.104165 | 0.116527 | 0.11215 | 0.104841 | 0.113089 | 0.121241 | 0.110926 |
| 0.117116 | | 0.215151 | 0.122316 | 0.123272 | 0.112397 | 0.204396 | 0.207684 | 0.192122 |
| 0.119055 | | 0.220935 | 0.127256 | 0.128518 | 0.115806 | 0.215355 | 0.22622 | 0.199358 |
| 0.110221 | | 0.156231 | 0.118462 | 0.125024 | 0.121224 | 0.150941 | 0.163085 | 0.151374 |

| 0.108115 | 0.121028 | 0.109739 | 0.160787 | 0.124466 | 0.101511 | NA | 0.128777 |
| --- | --- | --- | --- | --- | --- | --- | --- |
| 0.105366 | 0.120466 | 0.104928 | 0.148488 | 0.120533 | 0.102378 | NA | 0.123127 |
| 0.108225 | 0.124225 | 0.112405 | 0.156447 | 0.123996 | 0.107304 | 0.102496 | 0.130617 |
| 0.107004 | 0.111607 | 0.113547 | 0.149944 | 0.115669 | NA | NA | 0.121696 |
| 0.135011 | NA | NA | 0.104041 | NA | NA | NA | NA |
| 0.120204 | NA | NA | 0.09945 | NA | NA | NA | NA |
| 0.125205 | NA | NA | 0.106449 | NA | NA | NA | NA |
| 0.118144 | NA | NA | 0.098987 | NA | NA | NA | NA |
| 0.130457 | NA | NA | 0.108108 | 0.098529 | NA | NA | NA |
| 0.127191 | NA | NA | 0.10709 | 0.09926 | NA | NA | NA |
| 0.109153 | NA | NA | 0.09661 | NA | NA | NA | NA |
| 0.109153 | NA | NA | 0.09661 | NA | NA | NA | NA |
| 0.109153 | NA | NA | 0.09661 | NA | NA | NA | NA |
| 0.124205 | NA | NA | 0.102373 | NA | NA | NA | NA |
| 0.127794 | NA | NA | 0.107656 | 0.099177 | NA | NA | NA |
| 0.126339 | 0.091037 | NA | 0.108614 | 0.101865 | NA | NA | NA |
| 0.113729 | NA | NA | 0.102661 | 0.094353 | NA | NA | NA |
| 0.115402 | NA | NA | 0.102308 | 0.096561 | NA | NA | NA |
| 0.123522 | 0.094421 | NA | 0.108467 | 0.103886 | 0.094367 | NA | 0.093655 |
| 0.130184 | 0.090165 | NA | 0.10771 | 0.099776 | NA | NA | NA |
| 0.106103 | NA | NA | 0.094392 | NA | NA | NA | NA |
| 0.121643 | 0.202567 | 0.123627 | 0.130349 | 0.120003 | 0.196989 | 0.208723 | 0.185077 |
| 0.105121 | 0.098844 | NA | NA | 0.101359 | 0.094848 | NA | 0.098477 |
| 0.105121 | 0.098844 | NA | NA | 0.101359 | 0.094848 | NA | 0.098477 |
| 0.095602 | NA | NA | NA | NA | NA | NA | NA |
| 0.095602 | NA | NA | NA | NA | NA | NA | NA |
| 0.105415 | 0.090811 | NA | NA | 0.094508 | NA | NA | NA |
| 0.115167 | 0.098347 | NA | NA | 0.098301 | 0.092015 | NA | 0.098348 |
| 0.09979 | NA | NA | NA | NA | NA | NA | NA |
| 0.101514 | NA | NA | NA | NA | NA | NA | NA |
| 0.101514 | NA | NA | NA | NA | NA | NA | NA |
| 0.101514 | NA | NA | NA | NA | NA | NA | NA |
| NA | NA | NA | NA | NA | NA | NA | NA |
| NA | NA | NA | NA | NA | NA | NA | NA |
| NA | NA | NA | 0.093974 | NA | NA | NA | NA |
| NA | NA | NA | 0.094204 | NA | NA | NA | NA |
| 0.101804 | 0.097919 | 0.104062 | 0.10545 | 0.113053 | 0.098381 | 0.104119 | 0.099635 |
| 0.101474 | 0.098739 | 0.125485 | 0.107077 | 0.104346 | 0.10492 | 0.114659 | 0.109902 |
| 0.101474 | 0.098739 | 0.125485 | 0.107077 | 0.104346 | 0.10492 | 0.114659 | 0.109902 |

| 0.113852 | 0.102628 | 0.13839 | 0.117089 | 0.103723 | 0.108114 | 0.122582 | 0.118176 |
| --- | --- | --- | --- | --- | --- | --- | --- |
| 0.103146 | NA | 0.137086 | 0.099775 | NA | NA | NA | NA |
| 0.108985 | 0.109585 | 0.122758 | 0.118873 | 0.102958 | 0.115719 | 0.12325 | 0.116399 |
| 0.096066 | NA | 0.114847 | 0.099836 | NA | 0.096142 | 0.103168 | 0.102945 |
| 0.103741 | 0.113417 | 0.109318 | 0.11218 | 0.109059 | 0.110577 | 0.113965 | 0.112545 |
| NA | 0.118606 | NA | NA | 0.095083 | NA | NA | NA |
| 0.097757 | NA | NA | 0.104575 | NA | NA | NA | NA |
| 0.099415 | NA | NA | 0.10967 | NA | NA | NA | NA |
| 0.132849 | 0.090071 | 0.101319 | 0.104534 | 0.10405 | NA | NA | 0.096271 |
| 0.133663 | 0.09039 | 0.099638 | 0.106374 | 0.102114 | 0.091219 | NA | 0.095468 |
| 0.129591 | 0.093575 | 0.104374 | 0.107388 | 0.107502 | 0.095453 | 0.102313 | 0.098454 |
| NA | NA | NA | 0.094973 | NA | NA | NA | NA |
| 0.127639 | NA | NA | 0.100747 | NA | NA | NA | NA |
| 0.117796 | NA | NA | NA | NA | NA | NA | NA |
| 0.122986 | NA | NA | NA | NA | NA | NA | NA |
| 0.109912 | NA | NA | NA | NA | NA | NA | NA |
| NA | NA | NA | 0.096162 | NA | NA | NA | NA |
| NA | NA | NA | 0.100712 | 0.093742 | NA | NA | NA |
| NA | NA | NA | 0.100143 | NA | NA | NA | NA |
| 0.103048 | 0.092337 | NA | 0.118265 | 0.101569 | NA | NA | NA |
| 0.118417 | NA | NA | 0.094883 | NA | NA | NA | NA |
| 0.09309 | NA | NA | 0.100623 | 0.093621 | NA | NA | NA |
| NA | NA | NA | 0.10129 | NA | NA | NA | NA |
| NA | NA | NA | 0.10242 | 0.089816 | NA | NA | NA |
| 0.129581 | NA | NA | 0.104594 | NA | NA | NA | NA |
| 0.114056 | 0.093061 | NA | 0.12123 | 0.101113 | NA | NA | NA |
| 0.124505 | NA | NA | 0.098682 | NA | NA | NA | NA |
| 0.122623 | NA | NA | 0.101538 | NA | NA | NA | NA |
| NA | NA | NA | 0.095574 | NA | NA | NA | NA |
| NA | NA | NA | 0.095561 | NA | NA | NA | NA |
| 0.09525 | 0.096157 | NA | 0.117126 | 0.108397 | NA | NA | 0.092101 |
| NA | NA | NA | 0.094335 | NA | NA | NA | NA |
| NA | NA | NA | 0.108791 | 0.09402 | NA | NA | NA |
| NA | NA | NA | 0.101969 | NA | NA | NA | NA |
| 0.128365 | NA | NA | 0.103698 | NA | NA | NA | NA |
| 0.129189 | NA | NA | 0.109882 | 0.09387 | NA | NA | NA |
| 0.128681 | NA | NA | 0.100511 | NA | NA | NA | NA |
| 0.136275 | NA | NA | 0.108933 | NA | NA | NA | NA |
| 0.120438 | NA | NA | 0.099917 | NA | NA | NA | NA |

| NA | NA | NA | 0.104169 | | 0.092021 | NA |  | NA | NA |
| --- | --- | --- | --- | --- | --- | --- | --- | --- | --- |
| 0.104743 | 0.094584 | NA | 0.119553 | | 0.10276 | NA |  | NA | NA |
| NA | NA | NA | 0.093564 | | NA | NA |  | NA | NA |
| 0.116454 | NA | NA | NA |  | NA | NA |  | NA | NA |
| 0.118752 | NA | NA | NA |  | NA | NA |  | NA | NA |
| 0.132839 | 0.09573 | NA | 0.11106 | | 0.094237 | NA |  | NA | NA |
| 0.123804 | NA | NA | 0.09608 | | NA | NA |  | NA | NA |
| 0.123843 | NA | NA | 0.101683 | | NA | NA |  | NA | NA |
| NA | NA | NA | 0.095033 | | NA | NA |  | NA | NA |
| 0.133087 | NA | NA | 0.107513 | | 0.093677 | NA |  | NA | NA |
| 0.095067 | 0.143532 | 0.095729 | 0.09932 | | 0.09887 | 0.133116 | | 0.122494 | 0.119043 |
| 0.118621 | 0.097246 | 0.114877 | 0.126265 | | 0.093747 | 0.095726 | | NA | 0.096926 |
| NA | 0.10534 | NA | NA |  | NA |  | 0.1037 | NA | 0.101497 |
| 0.093454 | 0.129081 | 0.097056 | 0.096576 | | 0.095817 | 0.123508 | | 0.106848 | 0.120302 |
| 0.12117 | 0.163004 | 0.137102 | 0.129128 | | 0.126247 | 0.158841 | | 0.152655 | 0.174444 |
| 0.097448 | 0.115014 | 0.108092 | 0.103584 | | 0.102845 | 0.111928 | | 0.107822 | 0.120549 |
| NA | 0.098182 | 0.118383 | 0.095742 | | NA | 0.099062 | | NA | 0.105794 |
| NA | 0.107798 | NA | NA |  | 0.094934 | 0.10285 | | NA | 0.104852 |
| 0.098911 | 0.13299 | 0.107744 | 0.102111 | | 0.103818 | 0.125337 | | 0.118863 | 0.129018 |
| 0.096311 | 0.121351 | 0.103141 | 0.100189 | | 0.09806 | 0.118078 | | NA | 0.12637 |
| 0.109779 | 0.118625 | 0.108517 | 0.162685 | | 0.124942 | 0.099147 | | NA | 0.127107 |
| 0.104433 | 0.115916 | 0.109567 | 0.154313 | | 0.119965 | 0.099889 | | NA | 0.122248 |
| 0.107601 | 0.122037 | 0.108517 |  | 0.1531 | 0.121428 | 0.102166 | | 0.09852 | 0.128518 |
| 0.106271 | 0.109028 | 0.109203 | 0.145915 | | 0.11407 | NA |  | NA | 0.121778 |
| 0.129756 | 0.097405 | 0.097163 | 0.095257 | | 0.103564 | 0.096741 | | NA | 0.098663 |
| 0.093417 | 0.10099 | 0.106328 | 0.107016 | | 0.10071 | 0.104486 | | 0.102437 | 0.102298 |
| 0.134624 | 0.098924 | 0.112879 | 0.096472 | | 0.103611 | 0.09858 | | 0.099331 | 0.102745 |
| 0.125871 | 0.103623 | 0.111861 | 0.102858 | | 0.105024 | 0.102936 | | 0.10439 | 0.110037 |
| 0.125871 | 0.103623 | 0.111861 | 0.102858 | | 0.105024 | 0.102936 | | 0.10439 | 0.110037 |
| 0.119877 | 0.09509 | 0.101164 | 0.097951 | | 0.099894 | 0.097476 | | NA | 0.100473 |
| 0.112882 | 0.10113 | 0.096214 |  | 0.1051 | 0.105328 | 0.105324 | | 0.091109 | 0.103604 |
| 0.131888 | 0.097026 | 0.100523 | 0.095715 | | 0.103503 | 0.097113 | | NA | 0.099707 |
| 0.131888 | 0.097026 | 0.100523 | 0.095715 | | 0.103503 | 0.097113 | | NA | 0.099707 |
| 0.131888 | 0.097026 | 0.100523 | 0.095715 | | 0.103503 | 0.097113 | | NA | 0.099707 |
| 0.119351 | 0.103916 | 0.091469 | 0.102513 | | 0.108294 | 0.104684 | | 0.091016 | 0.100153 |
| 0.09624 | 0.105822 | 0.102482 | 0.110078 | | 0.108545 | 0.110467 | | 0.116672 | 0.105647 |
| 0.133565 | NA | 0.10371 | 0.093154 | | 0.098498 | NA |  | NA | 0.097004 |
| NA | NA | NA | 0.096872 | | NA | NA |  | NA | NA |
| 0.103023 | 0.09551 | NA | 0.092201 | | NA | 0.100809 | | NA | 0.105955 |

| 0.092577 | NA | NA |  | 0.097446 | NA | NA | NA |  | NA |
| --- | --- | --- | --- | --- | --- | --- | --- | --- | --- |
| NA | NA | NA |  | 0.094096 | NA | NA | NA |  | NA |
| NA | NA | NA |  | 0.093427 | NA | NA | NA |  | NA |
| NA | NA | NA |  | 0.096538 | NA | NA | NA |  | NA |
| 0.107758 | 0.108034 | 0.118558 | | 0.113628 | 0.117751 | 0.122675 | 0.136896 | | 0.114334 |
| 0.103868 | 0.110725 | 0.110012 | | 0.115257 | 0.116454 | 0.10505 | 0.119286 | | 0.116142 |
| 0.099659 | 0.100802 | 0.113757 | | 0.110118 | 0.107869 | 0.102634 | 0.109583 | | 0.10649 |
| 0.106184 | 0.115426 | 0.110067 | | 0.117447 | 0.118876 | 0.112961 | 0.127953 | | 0.118573 |
| NA | 0.102903 | 0.109671 | | 0.108416 | 0.102202 | 0.098276 | 0.107082 | | 0.106516 |
| 0.096046 | 0.106996 | 0.108359 | | 0.108818 | 0.106666 | 0.101588 | 0.109785 | | 0.108692 |
| 0.112905 | 0.111184 | 0.129008 | | 0.115826 | 0.107828 | 0.114545 |  | 0.1152 | 0.121045 |
| 0.108345 | 0.109947 | 0.10592 | | 0.125583 | 0.125625 | 0.106861 | 0.131893 | | 0.110458 |
| 0.114634 | 0.118935 | 0.10867 | | 0.14521 | 0.136803 | 0.116873 | 0.139311 | | 0.115791 |
| 0.106445 | 0.098964 | 0.10923 | | 0.107465 | 0.097595 | 0.102078 | 0.104906 | | 0.10606 |
| 0.093853 | NA | NA |  | 0.102493 | 0.093085 | NA | NA |  | NA |
| NA | NA | NA |  | 0.097219 | NA | NA | NA |  | NA |
| 0.118577 | 0.105436 | 0.133605 | | 0.111666 | 0.096932 | 0.109499 | 0.10854 | | 0.118405 |
| NA | NA | NA |  | 0.094704 | NA | NA | NA |  | NA |
| 0.116514 | 0.107116 | 0.112971 | | 0.114592 | 0.111079 | 0.117703 | 0.125284 | | 0.114193 |
| 0.096849 | NA | NA |  | 0.093687 | NA | NA | NA |  | NA |
| 0.096849 | NA | NA |  | 0.093687 | NA | NA | NA |  | NA |
| 0.113648 | 0.11009 | 0.113331 | | 0.114947 | 0.110101 | 0.111288 | 0.121582 | | 0.116447 |
| 0.113631 | 0.114318 | 0.131724 | | 0.118344 | 0.112536 | 0.117468 | 0.11776 | | 0.118017 |
| 0.113631 | 0.114318 | 0.131724 | | 0.118344 | 0.112536 | 0.117468 | 0.11776 | | 0.118017 |
| 0.113631 | 0.114318 | 0.131724 | | 0.118344 | 0.112536 | 0.117468 | 0.11776 | | 0.118017 |
| 0.113631 | 0.114318 | 0.131724 | | 0.118344 | 0.112536 | 0.117468 | 0.11776 | | 0.118017 |
| 0.106928 | 0.117722 | 0.104509 | | 0.114247 | 0.110643 | 0.117568 | 0.120801 | | 0.114762 |
| 0.106928 | 0.117722 | 0.104509 | | 0.114247 | 0.110643 | 0.117568 | 0.120801 | | 0.114762 |
| 0.106928 | 0.117722 | 0.104509 | | 0.114247 | 0.110643 | 0.117568 | 0.120801 | | 0.114762 |
| 0.106928 | 0.117722 | 0.104509 | | 0.114247 | 0.110643 | 0.117568 | 0.120801 | | 0.114762 |
| 0.120759 | 0.115248 | 0.105732 | | 0.124141 | 0.114708 | 0.112115 | 0.110226 | | 0.113621 |
| 0.120759 | 0.115248 | 0.105732 | | 0.124141 | 0.114708 | 0.112115 | 0.110226 | | 0.113621 |
| 0.114783 | 0.112344 | 0.101866 | | 0.114546 | 0.108493 | 0.109752 | 0.10389 | | 0.110718 |
| 0.114783 | 0.112344 | 0.101866 | | 0.114546 | 0.108493 | 0.109752 | 0.10389 | | 0.110718 |
| 0.117481 | 0.118822 |  | 0.1398 | 0.124416 | 0.118272 | 0.122227 | 0.125869 | | 0.131516 |
| 0.116819 | 0.107327 | 0.120079 | | 0.1158 | 0.114288 | 0.125151 | 0.137409 | | 0.116876 |
| 0.116819 | 0.107327 | 0.120079 | | 0.1158 | 0.114288 | 0.125151 | 0.137409 | | 0.116876 |
| 0.116495 | 0.110508 | 0.109968 | | 0.113978 | 0.11014 | 0.120124 | 0.127636 | | 0.114224 |
| 0.116495 | 0.110508 | 0.109968 | | 0.113978 | 0.11014 | 0.120124 | 0.127636 | | 0.114224 |

| 0.116821 | 0.105832 | 0.120098 | 0.112623 | 0.105168 | 0.121977 | 0.133972 | 0.114953 |
| --- | --- | --- | --- | --- | --- | --- | --- |
| 0.119279 | 0.111708 | 0.121464 | 0.120034 | 0.116636 | 0.128565 | 0.142774 | 0.120668 |
| 0.113918 | 0.106635 | 0.118752 | 0.114231 | 0.112934 | 0.117434 | 0.126427 | 0.112479 |
| 0.099847 | 0.101837 | 0.104597 | 0.100332 | 0.103547 | 0.107936 | 0.108001 | 0.100907 |
| 0.102567 | 0.122677 | 0.106092 | 0.133994 | 0.113962 | 0.105825 | 0.106592 | 0.121414 |
| 0.110538 | 0.116444 | 0.109366 | 0.160047 | 0.12366 | 0.096613 | NA | 0.12595 |
| 0.102773 | 0.120747 | 0.111604 | 0.131215 | 0.112982 | 0.106265 | 0.111018 | 0.120733 |
| 0.128592 | 0.11056 | 0.112695 | 0.140402 | 0.140066 | 0.101471 | 0.105903 | 0.108506 |
| 0.130453 | 0.112106 | 0.113874 | 0.144743 | 0.144736 | 0.102322 | 0.108883 | 0.108868 |
| 0.10319 | NA | 0.104475 | 0.101958 | 0.116058 | NA | NA | NA |
| 0.131999 | 0.112586 | 0.113633 | 0.145882 | 0.147678 | 0.102233 | 0.107387 | 0.108572 |
| 0.115926 | 0.10629 | 0.104196 | 0.117939 | 0.127423 | 0.100408 | 0.102419 | 0.104542 |
| NA | NA | 0.117102 | NA | NA | 0.097079 | 0.099557 | 0.10256 |
| 0.094558 | 0.106828 | 0.111538 | 0.107141 | 0.103496 | 0.106293 | 0.111851 | 0.109451 |
| NA | NA | 0.117994 | NA | NA | NA | NA | 0.098098 |
| NA | NA | 0.09594 | NA | NA | NA | NA | NA |
| NA | NA | NA | NA | NA | NA | NA | NA |
| 0.091743 | 0.096269 | 0.09504 | 0.095709 | 0.094409 | 0.097251 | NA | 0.095717 |
| NA | NA | 0.093765 | NA | NA | NA | NA | NA |
| NA | NA | 0.095527 | NA | NA | NA | NA | NA |
| NA | NA | 0.096379 | NA | NA | NA | NA | NA |
| NA | NA | 0.101052 | NA | NA | NA | NA | NA |
| NA | NA | 0.096542 | NA | NA | NA | NA | 0.094494 |
| 0.092478 | 0.099243 | 0.093416 | NA | NA | 0.098266 | NA | 0.09877 |
| 0.092105 | 0.107905 | 0.091957 | 0.093528 | NA | 0.10484 | 0.096567 | 0.101995 |
| NA | NA | NA | 0.094606 | NA | NA | NA | NA |
| NA | NA | NA | 0.096625 | NA | NA | NA | NA |
| NA | NA | NA | 0.09253 | NA | NA | NA | NA |
| 0.117975 | 0.117951 | 0.135615 | 0.122797 | 0.11633 | 0.121676 | 0.120199 | 0.128224 |
| 0.097913 | 0.110253 | 0.096017 | 0.114315 | 0.11433 | 0.112882 | 0.10325 | 0.105951 |
| 0.097913 | 0.110253 | 0.096017 | 0.114315 | 0.11433 | 0.112882 | 0.10325 | 0.105951 |
| 0.16757 | NA | NA | 0.120014 | NA | NA | NA | NA |
| 0.100653 | NA | 0.109019 | 0.111859 | 0.094051 | NA | NA | 0.095119 |
| 0.105377 | NA | 0.104933 | 0.106492 | 0.097097 | NA | NA | NA |
| 0.114755 | 0.093178 | 0.09949 | 0.105797 | 0.095827 | 0.093236 | NA | 0.096822 |
| 0.104474 | 0.128621 | 0.113059 | 0.113448 | 0.108294 | 0.127862 | 0.142972 | 0.125492 |
| 0.122563 | 0.090404 | 0.10216 | 0.107154 | 0.10073 | 0.090552 | NA | 0.096256 |
| 0.099506 | NA | 0.108299 | 0.098334 | NA | NA | NA | NA |
| 0.118303 | NA | 0.104692 | 0.109949 | 0.099599 | NA | NA | 0.097023 |

| 0.116026 | 0.093394 | 0.096874 | 0.105496 | 0.097403 | | 0.092707 | NA | 0.096219 |
| --- | --- | --- | --- | --- | --- | --- | --- | --- |
| 0.106551 | NA | NA | 0.103689 | 0.095896 | | NA | NA | NA |
| 0.107434 | NA | NA | 0.104392 | 0.095376 | | NA | NA | NA |
| 0.111531 | NA | 0.098905 | 0.106691 | 0.092924 | | NA | NA | 0.096174 |
| 0.106746 | NA | 0.104457 | 0.103143 | 0.095467 | | NA | NA | NA |
| 0.103569 | 0.111576 | 0.106791 | 0.11715 | 0.117997 | | 0.103956 | 0.117468 | 0.116464 |
| NA | NA | NA | NA | NA |  | NA | NA | NA |
| NA | NA | NA | NA | NA |  | NA | NA | NA |
| NA | NA | NA | NA | NA |  | NA | NA | NA |
| NA | NA | NA | NA | NA |  | NA | NA | NA |
| 0.099531 | 0.105925 | 0.127839 | 0.173677 | 0.10492 | | 0.154823 | 0.143816 | 0.110406 |
| 0.096964 | 0.097963 | 0.116499 | 0.138639 | 0.100156 | | 0.118492 | 0.111451 | 0.102137 |
| 0.104335 | 0.113174 | 0.110591 | 0.130754 | 0.108253 | | 0.111794 | 0.113281 | 0.112584 |
| NA | NA | NA | NA | NA |  | NA | NA | NA |
| NA | NA | 0.120619 | 0.135469 | NA |  | 0.123089 | 0.122645 | 0.097583 |
| 0.099343 | 0.100389 | 0.103708 | 0.132669 | 0.097549 | | 0.119856 | 0.108621 | 0.099655 |
| 0.101448 | 0.099365 | 0.106622 | 0.122762 | 0.104296 | | 0.104788 | 0.101638 | 0.103331 |
| NA | NA | 0.117133 | 0.119696 | 0.096073 | | 0.107665 | 0.11292 | 0.096323 |
| NA | NA | NA | 0.096882 | NA |  | NA | NA | NA |
| NA | 0.097297 | 0.116016 | 0.096486 | 0.098418 | | 0.106252 | 0.119809 | 0.103538 |
| 0.096452 | 0.104413 | 0.110536 | 0.104127 | 0.10261 | | 0.109827 | 0.122873 | 0.107803 |
| 0.110158 | 0.09732 | 0.097624 | 0.103679 | 0.09783 | | 0.096371 | 0.096637 | 0.096669 |
| 0.117672 | 0.096478 | 0.097155 | 0.107601 | 0.099446 | | 0.094668 | NA | 0.096317 |
| 0.110384 | 0.122878 | 0.091304 | 0.11685 | 0.120817 | | 0.112881 | 0.134155 | 0.115477 |
| 0.102688 | 0.106545 | 0.108402 | 0.107484 | 0.105205 | | 0.111178 | 0.112071 | 0.110382 |
| 0.101625 | 0.093529 | 0.104774 | 0.108261 | 0.100412 | | NA | NA | 0.099866 |
| 0.095663 | 0.108836 | 0.111934 | 0.109893 | 0.103209 | | 0.10857 | 0.111506 | 0.114096 |
| 0.093979 | 0.099301 | 0.108231 | 0.104705 | 0.103639 | | 0.100388 | 0.100678 | 0.103951 |
| 0.105634 | 0.112108 | 0.110036 | 0.112002 | 0.107577 | | 0.109495 | 0.115498 | 0.112098 |
| 0.097217 | 0.096554 | 0.109356 | 0.104374 | 0.099844 | | 0.100392 | 0.104825 | 0.102887 |
| 0.117622 | 0.115057 | 0.111236 | 0.126508 | 0.117959 | | 0.110322 | 0.120487 | 0.115668 |
| 0.104006 | 0.103095 | 0.110034 | 0.111434 | 0.103879 | | 0.104811 | 0.108636 | 0.107629 |
| 0.114083 | 0.113779 | 0.112006 | 0.121534 | 0.11686 | | 0.110861 | 0.11913 | 0.119012 |
| 0.100113 | 0.102723 | 0.109901 | 0.109779 | 0.103859 | | 0.105166 | 0.110371 | 0.106651 |
| 0.107017 | 0.106113 | 0.10519 | 0.114871 | 0.107559 | | 0.105803 | 0.105186 | 0.109281 |
| 0.101491 | 0.098856 | 0.103364 | 0.108722 |  | 0.0993 | 0.099431 | NA | 0.102613 |
| 0.093933 | NA | 0.100014 | NA | 0.09556 | | NA | NA | NA |
| 0.093933 | NA | 0.100014 | NA | 0.09556 | | NA | NA | NA |
| 0.093933 | NA | 0.100014 | NA | 0.09556 | | NA | NA | NA |

| 0.093166 | 0.137374 | 0.112296 | 0.099345 | NA | 0.145717 | 0.139248 | 0.141795 |
| --- | --- | --- | --- | --- | --- | --- | --- |
| 0.09463 | 0.143725 | 0.110346 | 0.10132 | 0.094789 | 0.151172 | 0.145485 | 0.148707 |
| NA | 0.12798 | 0.102708 | 0.099239 | NA | 0.134628 | 0.13414 | 0.130217 |
| 0.118284 | 0.095247 | 0.099061 | 0.108692 | 0.097706 | 0.093887 | NA | 0.097434 |
| 0.109097 | NA | 0.108686 | 0.107043 | 0.096702 | NA | NA | NA |
| 0.145759 | NA | 0.096784 | 0.117122 | 0.103984 | NA | NA | NA |
| 0.137075 | NA | 0.097461 | 0.115459 | 0.102721 | NA | NA | NA |
| 0.095011 | 0.099286 | 0.096653 | 0.114848 | 0.101322 | 0.095705 | NA | 0.099924 |
| 0.100515 | 0.10259 | 0.109699 | 0.105274 | 0.10423 | 0.104189 | 0.108291 | 0.104205 |
| 0.105568 | 0.100907 | 0.110073 | 0.111018 | 0.10551 | 0.103701 | 0.109438 | 0.108034 |
| 0.105081 | 0.101567 | 0.11047 | 0.111183 | 0.111755 | 0.101677 | 0.111713 | 0.107195 |
| 0.105081 | 0.101567 | 0.11047 | 0.111183 | 0.111755 | 0.101677 | 0.111713 | 0.107195 |
| 0.105516 | 0.10415 | 0.109132 | 0.110039 | 0.112588 | 0.104438 | 0.112961 | 0.108702 |
| 0.101377 | 0.112044 | 0.112317 | 0.143481 | 0.113366 | 0.098408 | NA | 0.122405 |
| 0.101377 | 0.112044 | 0.112317 | 0.143481 | 0.113366 | 0.098408 | NA | 0.122405 |
| 0.101678 | 0.093094 | NA | 0.090751 | NA | 0.092198 | NA | 0.095493 |
| 0.095351 | 0.09131 | NA | NA | NA | NA | NA | NA |
| 0.102133 | 0.091214 | NA | NA | NA | NA | NA | 0.091035 |
| 0.113595 | 0.099097 | 0.094114 | 0.099229 | 0.103086 | 0.100873 | 0.086038 | 0.099378 |
| 0.115585 | 0.15929 | 0.115735 | 0.119734 | 0.110371 | 0.152677 | 0.162988 | 0.15232 |
| 0.103058 | 0.231847 | 0.117829 | 0.09729 | 0.095583 | 0.205185 | 0.187374 | 0.15866 |
| 0.090988 | 0.159748 | 0.089685 | 0.090022 | 0.090153 | 0.144604 | 0.119098 | 0.116989 |
| 0.100689 | 0.170419 | 0.103648 | 0.104636 | 0.104202 | 0.159672 | 0.155263 | 0.135092 |
| 0.105189 | 0.222017 | 0.112444 | 0.098427 | 0.097555 | 0.191483 | 0.179919 | 0.15013 |
| 0.102779 | 0.194646 | 0.104401 | 0.096856 | 0.095115 | 0.172136 | 0.156903 | 0.139678 |
| 0.098103 | 0.108778 | 0.092643 | 0.106465 | 0.109738 | 0.104172 | 0.104095 | 0.102684 |
| 0.093384 | 0.131262 | 0.097658 | 0.097576 | 0.097413 | 0.123698 | 0.10646 | 0.120637 |
| 0.105039 | 0.107371 | 0.097093 | 0.111855 | 0.111937 | 0.102208 | 0.10433 | 0.107753 |
| 0.10392 | 0.098996 | 0.108409 | 0.099834 | 0.099931 | 0.103499 | 0.100711 | 0.104105 |
| 0.114292 | 0.108127 | 0.120369 | 0.116317 | 0.111326 | 0.123419 | 0.135701 | 0.120567 |
| 0.116038 | 0.117507 | 0.111397 | 0.120217 | 0.123905 | 0.126148 | 0.135173 | 0.117687 |
| 0.114069 | NA | 0.120682 | 0.098091 | NA | 0.10481 | 0.109863 | 0.10644 |
| 0.102433 | NA | NA | 0.091611 | NA | 0.09582 | NA | 0.101433 |
| 0.096873 | NA | NA | 0.122796 | 0.108615 | NA | NA | NA |
| 0.094758 | NA | NA | 0.122809 | 0.104887 | NA | NA | NA |
| 0.093749 | NA | 0.116811 | 0.104913 | 0.101113 | 0.102272 | 0.116446 | 0.100766 |
| 0.101501 | 0.103784 | 0.120211 | 0.117624 | 0.111947 | 0.113618 | 0.126798 | 0.109129 |
| 0.09658 | 0.100475 | 0.117934 | 0.109132 | 0.108158 | 0.110875 | 0.130099 | 0.10452 |
| NA | NA | 0.112042 | NA | NA | NA | NA | NA |

| 0.1014 | 0.102002 | 0.119039 | 0.116083 | 0.110229 | 0.11105 | 0.125524 | 0.108437 |
| --- | --- | --- | --- | --- | --- | --- | --- |
| 0.112501 | 0.111283 | 0.119095 | 0.121273 | 0.120683 | 0.123696 | 0.13731 | 0.119404 |
| 0.112831 | 0.110198 | 0.129895 | 0.115662 | 0.102442 | 0.110689 | 0.111354 | 0.118426 |
| 0.110759 | 0.10938 | 0.129691 | 0.113831 | 0.101315 | 0.10837 | 0.109198 | 0.116679 |
| 0.107383 | 0.104793 | 0.123248 | 0.108025 | 0.098189 | 0.102308 | 0.10568 | 0.11203 |
| 0.108758 | 0.104489 | 0.111046 | 0.11415 | 0.100008 | 0.107645 | 0.118354 | 0.109743 |
| 0.10575 NA | | 0.109541 | 0.11102 | 0.106708 NA NA | | | 0.097374 |
| 0.112984 0.109046 | | 0.130659 | 0.114252 | 0.101824 0.109256 0.112501 | | | 0.116936 |
| 0.109712 0.103205 | | 0.129808 | 0.109253 | 0.096402 0.102788 0.108138 | | | 0.112322 |

NA	NA	NA	0.099535 NA	NA	NA	NA NA	NA	NA	0.101012 NA	NA	NA	NA NA	NA	NA	NA	NA	NA	NA	NA

0.114672 0.122026	0.09982 0.122179 0.124212 0.117298 0.138604 0.119786

0.107476 0.108031 0.121932 0.116494 0.110495 0.110154 0.120848	0.11628

0.097687 NA	0.108062 0.094627 0.102381 0.105157 0.105775 0.100789

0.100275 0.103416 0.119943 0.109525 0.111299 0.114486 0.121804 0.110329

0.096971 NA	0.10667 NA	0.101925 0.104111 0.106214 0.099734

NA	NA	0.10938 NA	NA	0.098961 0.101037	0.10438

0.099956 0.097163 0.111193 0.102228 0.099007 0.100842 0.112924 0.103465

NA	NA	NA	0.094052 NA	NA	NA	NA

0.110861	0.11564 0.116276	0.12541 0.125698 0.114858 0.133906 0.119604

0.102604 0.097324 0.105451 0.104522 0.101091 0.095883 NA	0.101615

0.094829 NA	0.118707 NA	NA	NA	0.101685 NA

0.098179 NA	0.12186 NA	0.094628 NA	0.100759 NA

N

| 0.097765 | 0.100621 | 0.113785 | 0.106019 | 0.100112 | 0.105888 | 0.118911 | 0.107287 |
| --- | --- | --- | --- | --- | --- | --- | --- |
| 0.096634 | 0.10485 | 0.098284 | 0.106232 | 0.103323 | 0.106234 | 0.10916 | 0.103192 |
| 0.096514 | 0.10513 | 0.106195 | 0.108767 | 0.103907 | 0.108933 | 0.121539 | 0.106639 |
| A | 0.101343 | 0.1112 | 0.106726 | 0.100694 | 0.107502 | 0.124382 | 0.10573 |
| 0.095593 | 0.101959 | 0.098914 | 0.099545 | 0.098704 | 0.102081 | 0.099276 | 0.101277 |
| 0.107497 | 0.110182 | 0.111015 | 0.114056 | 0.114083 | 0.110897 | 0.117412 | 0.112283 |
| 0.105638 | 0.113434 | 0.106517 | 0.11005 | 0.111128 | 0.111288 | 0.105078 | 0.112039 |
| 0.108161 | 0.115411 | 0.119317 | 0.122397 | 0.118155 | 0.115712 | 0.130663 | 0.12296 |
| 0.099735 | 0.105441 | 0.106809 | 0.107215 | 0.106823 | 0.105908 | 0.106666 | 0.109579 |
| 0.104842 | 0.107504 | 0.111772 | 0.110697 | 0.10611 | 0.109248 | 0.104478 | 0.111233 |
| 0.09535 | 0.098926 | 0.092374 | 0.099554 | 0.103231 | 0.100909 | 0.106809 | 0.099055 |
| 0.096076 | 0.115325 | 0.091213 | 0.101305 | 0.104743 | 0.111863 | 0.108159 | 0.106785 |
| 0.098492 | 0.130378 | 0.094956 | 0.104352 | 0.105474 | 0.122855 | 0.119304 | 0.115998 |
| 0.102224 | 0.105889 | 0.110242 | 0.110903 | 0.109523 | 0.106247 | 0.111416 | 0.108895 |
| 0.108198 | 0.110336 | 0.103576 | 0.117693 | 0.108475 | 0.103671 | 0.12638 | 0.11461 |

| 0.100646 | 0.112362 | 0.111317 | 0.113987 | 0.113867 | 0.113209 | 0.120962 | 0.114241 |
| --- | --- | --- | --- | --- | --- | --- | --- |
| 0.100182 | 0.111948 | 0.107343 | 0.108349 | 0.107493 | 0.115826 | 0.120418 | 0.109613 |
| 0.115832 | 0.113757 | 0.114835 | 0.120394 | 0.120309 | 0.113328 | 0.119725 | 0.117916 |
| 0.096683 | 0.101913 | 0.110344 | 0.105839 | 0.098872 | 0.104953 | 0.110093 | 0.107539 |
| 0.122853 | 0.136418 | 0.100456 | 0.128627 | 0.128315 | 0.117415 | 0.124566 | 0.114278 |
| 0.101575 | NA | 0.107616 | 0.105649 | 0.09445 | NA | NA | 0.09568 |
| 0.105112 | 0.111858 | 0.108631 | 0.11339 | 0.112866 | 0.113332 | 0.125391 | 0.113669 |
| 0.099692 | 0.099716 | 0.109546 | 0.108117 | 0.109332 | 0.106883 | NA | 0.104965 |
| 0.103697 | 0.105161 | 0.114564 | 0.112976 | 0.11114 | 0.105522 | 0.114216 | 0.111887 |
| 0.099432 | 0.107518 | 0.102903 | 0.105909 | 0.102999 | 0.108157 | 0.108491 | 0.107118 |
| 0.098927 | 0.106358 | 0.105285 | 0.108405 | 0.105582 | 0.105296 | 0.107297 | 0.107192 |
| 0.108329 | NA | NA | 0.096105 | NA | NA | NA | NA |
| 0.095979 | 0.108295 | 0.107787 | 0.100054 | 0.099976 | 0.10939 | 0.111202 | 0.109778 |
| 0.099866 | 0.098936 | 0.099111 | 0.099216 | 0.099437 | 0.102219 | 0.103112 | 0.101677 |
| 0.102543 | 0.103951 | 0.106035 | 0.102276 | 0.099729 | 0.108618 | 0.107949 | 0.106204 |
| 0.108251 | 0.114811 | 0.117385 | 0.123584 | 0.123515 | 0.11916 | 0.14287 | 0.122618 |
| 0.100547 | NA | 0.108062 | 0.10057 | 0.102868 | 0.098315 | 0.106187 | 0.101591 |
| 0.103168 | 0.103059 | 0.113529 | 0.109715 | 0.110794 | 0.108005 | 0.128183 | 0.114255 |
| 0.091543 | NA | 0.105201 | 0.094739 | NA | NA | NA | NA |
| 0.096247 | 0.105518 | 0.104212 | 0.100282 | 0.099007 | 0.105916 | 0.100643 | 0.106516 |
| 0.104026 | 0.111369 | 0.103725 | 0.111801 | 0.107789 | 0.111713 | 0.105341 | 0.113726 |
| NA | 0.113321 | NA | NA | NA | 0.104778 | NA | 0.098457 |
| 0.101565 | 0.100999 | 0.114659 | 0.106565 | 0.107549 | 0.102925 | 0.111838 | 0.10508 |
| 0.091418 | 0.116556 | NA | 0.09535 | 0.099633 | 0.114752 | 0.104604 | 0.100482 |
| 0.10353 | 0.100889 | 0.112309 | 0.109616 | 0.104006 | 0.10402 | NA | 0.106716 |
| 0.098674 | 0.117714 | 0.097754 | 0.09987 | 0.100938 | 0.11195 | 0.105262 | 0.110502 |
| 0.105036 | 0.108497 | 0.104779 | 0.11205 | 0.110635 | 0.10375 | 0.10782 | 0.106555 |
| 0.101428 | 0.103276 | 0.094431 | 0.103163 | 0.102371 | 0.105746 | 0.099807 | 0.100008 |
| 0.100932 | 0.101396 | 0.105161 | 0.106611 | 0.105412 | 0.10251 | 0.108048 | 0.105442 |
| 0.099024 | 0.102556 | 0.103571 | 0.104279 | 0.099678 | 0.101439 | 0.10015 | 0.104016 |
| 0.095768 | 0.118483 | 0.095751 | 0.099328 | 0.10322 | 0.115286 | 0.108055 | 0.103856 |
| 0.111737 | 0.108847 | 0.112104 | 0.117379 | 0.115073 | 0.110976 | 0.125255 | 0.11204 |
| 0.097535 | 0.101188 | 0.107813 | 0.102716 | 0.10642 | 0.105481 | 0.110745 | 0.100323 |
| 0.098357 | 0.108837 | 0.096797 | 0.10058 | 0.094098 | 0.11064 | 0.11109 | 0.110796 |
| 0.100225 | NA | 0.09988 | NA | 0.095275 | NA | NA | NA |
| 0.102143 | 0.101713 | 0.10962 | 0.109692 | 0.107482 | 0.102419 | 0.107013 | 0.107871 |
| 0.102054 | 0.110825 | 0.10473 | 0.103205 | 0.101554 | 0.107175 | 0.110404 | 0.110934 |
| 0.096299 | 0.109564 | 0.096475 | 0.105232 | 0.107089 | 0.109623 | 0.102503 | 0.104206 |

segmente sigmoid_c t_cell..cee thyroid..ce venous_bl Motif	geneid	average ex

0.113487 0.102775 0.111047 0.113224 0.108141 AHR_1.mo AHR	10.61403

NA	NA	NA	NA	NA	ALX3_1.m ALX3	0.250098

0.113127 0.113707 0.113729 0.119702 0.120086 ARNT_1.m ARNT	2.901621

0.120241 0.101166 0.107182	0.12165 0.114428 ARNT_2.m ARNT	2.901621

0.108671 NA	0.103956 0.123212 0.125629 ARNTL_1. ARNTL	2.455715

0.127615 0.126415 0.115529 0.113116 0.112776 ASCL2_1. ASCL2	0.481194

0.116886 0.108502 0.112686 0.113066 0.125568 ATF3_1.m ATF3	9.129839

0.104832 NA	0.10378 0.110381 0.116396 ATF6_1.m ATF6	6.25221

0.104832 NA	0.10378 0.110381 0.116396 ATF6_1.m CREBZF	6.8113

0.122165 0.113347 0.101329 0.099437 0.102107 ATF7_1.m ATF7	1.466524

0.117282 0.120212 0.107873	0.10292 0.106527 ATOH1_1. ATOH1	0.597205

0.10787 0.109827	0.10321 0.098773 0.107789 ATOH7_1. ATOH7	0.027864

0.115563 0.106556	0.1106 0.103014 0.102329 BACH1_1. BACH1	8.719565

0.113864 0.112066 0.112557 0.107372 0.105768 BACH2_1. BACH2	3.691286

NA	0.097689 NA	NA	0.099172 BARHL2_1BARHL2	0.009287

NA	0.094394 NA	NA	NA	BARX1_2. BARX1	0.026636

NA	0.105949 0.108156 0.096391	0.09847 BATF_1.m BATF	3.482843

NA	0.105949 0.108156 0.096391	0.09847 BATF_1.m BATF2	0.376024

0.131437 0.121203 0.145464 0.094409	0.12373 BCL11A_1 BCL11A	1.353909

0.115282 0.112048 0.114649	0.1197 0.128814 BHLHE40_BHLHE40	11.27597

NA	0.095156 NA	NA	NA	BSX_1.mo BSX	0.000413

0.133006 0.108156 0.153822 0.099848 0.117551 CBFB_1.m CBFB	8.639489

NA	0.128445 NA	NA	NA	CDX1_1.m CDX1	6.33423

NA	0.146349 NA	NA	NA	CDX2_1.m CDX2	1.342978

0.117414 0.098611 0.092747 NA	NA	CEBPA_1. CEBPA	3.564659

0.146178 NA	NA	NA	NA	CEBPB_1. CEBPB	11.41955

0.147522 NA	NA	NA	NA	CEBPD_1. CEBPD	6.440049

0.137615 NA	NA	NA	NA	CEBPE_1. CEBPE	0.196569

0.112475 0.117889 0.116103 0.117723 0.125868 CLOCK_1. CLOCK	1.35989

0.112475 0.117889 0.116103 0.117723 0.125868 CLOCK_1. SOHLH1	0.00548

0.133167 0.130436 0.131888 0.135169 0.147392 CREB3L2_ CREB3L2	2.45618

0.134362	0.13322 0.133163	0.13537	0.15077 CREB3L3_ CREB3L3	1.504123

0.115214 0.109585 0.101105 NA	0.098594 CREB5_1. CREB5	1.161423

0.11812 0.102499 NA	NA	NA	DBP_1.mo DBP	0.951032

NA	NA	NA	NA	NA	DLX1_1.m DLX1	0.940891

NA	NA	NA	NA	NA	DLX2_1.m DLX2	0.029089

NA	NA	NA	NA	NA	DLX6_1.m DLX6	0.039721

0.108312 NA	0.101495 0.102916 NA	E2F1_1.m E2F1	1.348975

| 0.100005 | 0.093853 NA | 0.102597 | 0.105954 EBF1_1.m EBF1 | 0.744094 |
| --- | --- | --- | --- | --- |
| 0.100005 | 0.093853 NA | 0.102597 | 0.105954 EBF1_1.m EBF2 | 0.056299 |
| 0.100005 | 0.093853 NA | 0.102597 | 0.105954 EBF1_1.m EBF4 | 1.13221 |

0.096908 NA	0.113861	0.10438 0.108765 EGR2_1.m EGR2	4.92017

0.099053 NA	0.107391	0.10282 0.105396 EGR4_1.m EGR4	0.055799

0.161683 0.129795 0.161421	0.10124 0.123374 EHF_1.mo EHF	1.937504

0.181253 0.158807 0.177986 0.110799 0.128183 EHF_2.mo EHF	1.937504

0.200349 0.161785 0.196021 0.113455 0.136606 ELF1_1.m ELF1	17.15837

0.172054 0.148322 0.177613 0.105215 0.131643 ELF2_1.m ELF2	3.619342

0.174807 0.161384 0.169559 0.114533 0.127672 ELF3_1.m ELF3	5.23303

0.185686	0.15452 0.177704 0.114267	0.12949 ELF4_1.m ELF4	4.955086

0.19123 0.165245 0.186579 0.105946	0.13426 ELF5_1.m ELF5	0.063411

0.181332	0.14638 0.173807 0.120194 0.123279 ELK1_1.m ELK1	2.523555

0.186946 0.150839 0.186208 0.112606 0.128754 ELK3_1.m ELK3	6.052051

0.196974 0.156137 0.193967 0.116825 0.131407 ELK4_1.m ELK4	4.222079

NA	0.096072 NA	NA	NA	EN1_2.mo EN1	0.021821

0.105482 0.100535 0.106942 0.108023 0.122437 ENO1_1.m ENO1	53.1855

0.193811 0.153517 0.198588 0.112533 0.132546 ERF_1.mo ERF	3.044918

0.201602 0.157114 0.208287 0.114129 0.137603 ERG_1.mo ERG	0.473923

0.156726	0.12631 0.147255 0.116989 0.117605 ERG_2.mo ERG	0.473923

0.209707 0.162712 0.214894 0.108703 0.142045 ERG_3.mo ERG	0.473923

NA	0.098201 NA	NA	0.094855 ESRRG_3. ESRRG	0.138447

NA	0.094806 NA	NA	NA	ESX1_1.m ESX1	0.002003

0.205399 0.161638 0.212435 0.109265 0.139065 ETS1_1.m ETS1	34.5177

0.147265 0.127236 0.143278 0.112513 0.115623 ETS1_2.m ETS1	34.5177

0.168789 0.135492 0.165768 0.104752 0.120257 ETS2_1.m ETS2	11.82639

0.204953 0.157188 0.206489 0.115515 0.134785 ETV1_1.m ETV1	1.281508

0.188325 0.152492 0.199385	0.10958 0.133871 ETV2_1.m ETV2	0.20043

0.154427 0.131576 0.153349 0.113363 0.114494 ETV3_1.m ETV3	3.409202

0.154427 0.131576 0.153349 0.113363 0.114494 ETV3_1.m ETV3L	0.225297

0.207189 0.159252 0.211585 0.111668 0.138277 ETV4_1.m ETV4	0.660554

0.168102	0.13052 0.163907 0.106127 0.119186 ETV5_1.m ETV5	1.380658

0.16417 0.119046 0.147019 0.102685 0.120178 ETV6_1.m ETV6	5.168063

0.160721 0.140981	0.16523 0.107061 0.128385 ETV7_1.m ETV7	0.322632

NA	NA	NA	NA	NA	EVX2_1.m EVX2	0.002047

0.110364 0.112217 0.105651 0.100654 0.109414 FERD3L_1 FERD3L	0.000705

0.200207 0.161101 0.211694 0.106526	0.13928 FEV_1.mo FEV	0.041193

0.212697 0.163194 0.217685 0.111689 0.140769 FLI1_1.mo FLI1	6.202505

0.158569 0.129577 0.152736	0.11725 0.120473 FLI1_2.mo FLI1	6.202505

| 0.106499 | 0.114456 | 0.115291 | 0.098988 | 0.103915 FOS_1.mo FOS | | 102.9197 |
| --- | --- | --- | --- | --- | --- | --- |
| 0.105577 | 0.115982 | 0.116015 | 0.099669 | 0.104428 FOSB_1.m FOSB | | 23.89371 |
| 0.113503 | 0.115279 | 0.118138 | 0.106194 | 0.107243 FOSL1_1. FOSL1 | | 1.499884 |
| 0.101224 | 0.107213 | 0.106954 | NA | 0.101514 FOSL2_1. FOSL2 | | 10.92123 |
| NA | 0.143816 NA | | 0.115538 NA | | FOXA1_1. FOXA1 | 0.196486 |
| NA | 0.127749 NA | | 0.108468 NA | | FOXA2_1. FOXA2 | 0.197973 |
| NA | 0.135494 NA | | 0.124492 NA | | FOXC1_1. FOXC1 | 0.403768 |
| NA | 0.128488 NA | | 0.10636 NA | | FOXD1_1. FOXD1 | 0.098075 |
| NA | 0.146774 NA | | 0.107171 NA | | FOXD2_2. FOXD2 | 0.248824 |
| NA | 0.144465 NA | | 0.1057 NA | | FOXD4_1. FOXD4 | 0.043985 |
| NA | 0.12267 NA | | 0.105139 NA | | FOXF1_1. FOXF1 | 0.242067 |
| NA | 0.12267 NA | | 0.105139 NA | | FOXF1_1. FOXN1 | 0.012039 |
| NA | 0.12267 NA | | 0.105139 NA | | FOXF1_1. FOXN4 | 0.016733 |
| NA | 0.138717 NA | | 0.106447 NA | | FOXF2_1. FOXF2 | 0.161942 |
| NA | 0.144778 0.091383 | | 0.1069 NA | | FOXG1_1. FOXG1 | 2.496727 |
| NA | 0.144339 0.095014 | | 0.110374 NA | | FOXK1_1. FOXK1 | 2.550975 |
| NA | 0.12897 NA | | 0.112285 NA | | FOXO3_1. FOXO3 | 6.132707 |
| NA | 0.129239 NA | | 0.113146 NA | | FOXP1_1. FOXP1 | 6.563558 |

NA	0.142213 0.097043 0.110241 0.089845 FOXP2_1. FOXP2	0.134166

NA	0.146563 0.093052 0.108153 NA	FOXP3_1. FOXP3	0.257873

NA	0.120608 NA	0.098758 NA	FOXQ1_1. FOXQ1	0.482164

0.199584 0.154454 0.195954 0.117701 0.132119 GABPA_1. GABPA	5.447024

0.092145 0.108687 0.103908 NA	0.09459 GATA1_1. GATA1	4.270272

0.092145 0.108687 0.103908 NA	0.09459 GATA1_1. GATAD2B 2.744726

NA	0.096829 0.096316 NA	NA	GATA2_1. GATA2	1.004915

NA	0.096829 0.096316 NA	NA	GATA2_1. ZGLP1	0.332022

NA	0.110544 0.096124 NA	NA	GATA3_2. GATA3	1.734603

0.092979 0.121334 0.104331 NA	NA	GATA4_1. GATA4	0.454987

| NA | 0.102882 NA | | NA | NA | GATA5_1. | GATA5 | 0.056141 |
| --- | --- | --- | --- | --- | --- | --- | --- |
| NA | 0.104084 NA | | NA | NA | GATA6_1. | GATA6 | 1.124258 |
| NA | 0.104084 NA | | NA | NA | GATA6_1. | MTA2 | 9.732748 |
| NA | 0.104084 NA | | NA | NA | GATA6_1. | RERE | 2.849697 |
| NA | NA NA | | NA | NA | GBX1_1.m | GBX1 | 0.016024 |
| NA | 0.093618 NA | | NA | NA | GBX2_1.m | GBX2 | 0.041387 |
| NA | NA NA | | NA | NA | GSX1_1.m | GSX1 | 0.355979 |
| NA | NA NA | | NA | NA | GSX2_1.m | GSX2 | 0.384309 |
| 0.101977 | 0.104691 | 0.096616 | 0.105351 | 0.098322 HAND1_1. HAND1 | | | 0.030481 |
| 0.113215 | 0.095346 | 0.100163 | 0.114783 | 0.110335 HES1_1.m HES1 | | | 5.953764 |
| 0.113215 | 0.095346 | 0.100163 | 0.114783 | 0.110335 HES1_1.m HES6 | | | 3.89245 |

0.119003 0.101926 0.104888	0.12323 0.120409 HES4_1.m HES4	1.33165

0.100964 NA	NA	0.115695 0.114904 HES7_1.m HES7	0.036308

0.116021 0.104224 0.111574 0.120391 0.117085 HEYL_1.m HEYL	0.195637

0.105819 NA	NA	0.102423 0.100074 HIC1_1.moHIC1	0.612449

0.115628 0.103866 0.108536 0.108131 0.106454 HIVEP2_1. HIVEP2	5.716578

0.121818 0.096001 NA	NA	NA	HLF_1.mo HLF	1.869067

NA	0.10366 NA	NA	NA	HNF1A_1. HNF1A	0.461921

NA	0.103779 NA	NA	NA	HNF1B_1. HNF1B	0.254001

0.094329 0.128023 NA	0.101986 0.095022 HNF4A_1. HNF4A	3.22015

0.092903 0.128646 NA	0.098295 0.096241 HNF4A_2. HNF4A	3.22015

0.099865 0.126671 0.092686 0.104263 0.097491 HNF4G_1. HNF4G	1.277264

NA	NA	NA	NA	NA	HOXA1_1. HOXA1	0.138011

NA	0.139652 NA	NA	NA	HOXA10_1HOXA10	0.441861

NA	0.126763 NA	NA	NA	HOXA11_1HOXA11	0.137845

NA	0.129548 NA	NA	NA	HOXA13_1HOXA13	0.288091

NA	0.114952 NA	NA	NA	HOXA13_2HOXA13	0.288091

NA	NA	NA	NA	NA	HOXA2_1. HOXA2	0.13228

NA	0.09534 NA	NA	NA	HOXA5_2. HOXA5	0.665632

NA	NA	NA	NA	NA	HOXA6_1. HOXA6	0.214317

NA	0.121767 0.092185 NA	NA	HOXA6_10HOXA6	0.214317

NA	0.126092 NA	NA	NA	HOXA6_4. HOXA6	0.214317

NA	0.097401 NA	NA	NA	HOXA6_7. HOXA6	0.214317

NA	0.093508 NA	NA	NA	HOXA6_8. HOXA6	0.214317

NA	NA	NA	NA	NA	HOXA6_9. HOXA6	0.214317

NA	0.144403 NA	NA	NA	HOXA9_1. HOXA9	0.858129

NA	0.13349 0.093582 NA	NA	HOXA9_2. HOXA9	0.858129

NA	0.133188 NA	NA	NA	HOXB13_1HOXB13	0.614015

NA	0.130738 NA	NA	NA	HOXB13_2HOXB13	0.614015

NA	NA	NA	NA	NA	HOXB2_1. HOXB2	0.629625

NA	NA	NA	NA	NA	HOXB3_1. HOXB3	0.324204

NA	0.104173 0.093585 NA	NA	HOXB4_1. HOXB4	0.239336

NA	NA	NA	NA	NA	HOXB5_1. HOXB5	0.284203

NA	0.101229 NA	NA	NA	HOXB7_1. HOXB7	1.382744

NA	0.094748 NA	NA	NA	HOXB8_1. HOXB8	0.153745

NA	0.141942 NA	NA	NA	HOXC10_1HOXC10	0.174896

NA	0.140915 NA	NA	NA	HOXC11_1HOXC11	0.017421

NA	0.140753 NA	NA	NA	HOXC11_2HOXC11	0.017421

NA	0.14681 NA	NA	NA	HOXC12_2HOXC12	0.011721

NA	0.128547 NA	NA	NA	HOXC13_2HOXC13	0.008466

NA	NA	NA	NA	NA	HOXC5_1. HOXC5	0.042607

NA	0.123999 0.094042 NA	NA	HOXC9_1. HOXC9	0.133631

NA	NA	NA	NA	NA	HOXD1_1. HOXD1	0.26398

NA	0.125998 NA	NA	NA	HOXD11_ HOXD11	0.088419

NA	0.129625 NA	NA	NA	HOXD12_ HOXD12	0.01026

NA	0.1449 0.098403 NA	NA	HOXD12_ HOXD12	0.01026

NA	0.133786 NA	NA	NA	HOXD13_ HOXD13	0.106701

NA	0.133334 NA	NA	NA	HOXD13_ HOXD13	0.106701

NA	NA	NA	NA	NA	HOXD3_1. HOXD3	0.204829

NA	0.144705 0.095789 NA	NA	HOXD9_2. HOXD9	0.150324

0.123801 0.110845 0.128684 0.091617 0.110149 IKZF3_1.mIKZF3	7.546775

0.095296 0.121573 0.116474 0.123136 0.110633 IKZF5_1.mIKZF5	3.351358

NA	0.097502	0.1133 0.091361	0.09949 IRF1_1.mo IRF1	14.28949

0.105999 0.109741 0.132879 0.098234 0.112622 IRF3_1.mo IRF3	6.215972

0.128103 0.159373 0.185612 0.123753 0.144643 IRF4_2.mo IRF4	4.446254

0.104332 0.107596 0.123292	0.10275 0.111457 IRF5_1.mo IRF5	2.860249

0.10017 NA	0.101744 0.100434 0.109867 IRF6_1.Inf IRF6	2.014436

NA	0.103578 0.113637 0.092538 0.103838 IRF7_1.mo IRF7	4.29338

0.111372 0.118437 0.135509 0.100679 0.118576 IRF8_1.mo IRF8	8.990926

0.101271 0.120275 0.137743 0.102315 0.114326 IRF9_1.mo IRF9	8.277572

0.101739 0.118518 0.115107 0.098797 0.103429 JDP2_1.m JDP2	1.213055

0.105613 0.110188 0.110348 0.100053 0.102552 JUN_1.mo JUN	54.59067

0.109998 0.115182 0.116106 0.099482 0.104164 JUNB_1.m JUNB	123.2367

0.096293 0.108513 0.108142 NA	0.100562 JUND_1.m JUND	57.71767

NA	0.114073 0.098941 NA	0.100607 KLF1_1.m KLF1	0.917087

0.099846 0.094813 0.102737 0.100946 0.101561 KLF12_1.mKLF12	4.036184

0.102081 0.116043 0.099004 0.097371 0.105717 KLF12_2.mKLF12	4.036184

0.102678 0.112492 0.104549 0.101705 0.111614 KLF13_1.mKLF11	3.749098

0.102678 0.112492 0.104549 0.101705 0.111614 KLF13_1.mKLF13	10.98144

NA	0.098106 0.097649 NA	0.10314 KLF14_1.mKLF14	0.019144

0.088905 0.100232 0.103217 NA	0.10382 KLF16_1.mKLF16	2.186776

NA	0.112147 0.098157 NA	0.101515 KLF4_1.m KLF17	0.016255

NA	0.112147 0.098157 NA	0.101515 KLF4_1.m KLF2	26.99103

NA	0.112147 0.098157 NA	0.101515 KLF4_1.m KLF4	11.46665

0.090879 0.104737 0.101639 0.094177 0.100639 KLF5_1.m KLF5	7.1987

0.105779 NA	0.101765 0.100967 0.101053 KLF6_1.m KLF6	32.46888

NA	0.109247 0.095078 NA	0.101394 KLF7_1.m KLF7	2.706802

NA	0.094814 NA	NA	NA	LBX1_1.m LBX1	0.011622

NA	0.106583 0.113345 0.095004 0.094478 LEF1_1.m LEF1	12.31133

9

0.101655 0.110231 0.102648 0.098519	0.10287 MEIS2_1. MEIS2	0.190007

| NA | 0.0 | 4888 NA | | NA | NA | LHX2_1.m LHX2 | 1.756814 |
| --- | --- | --- | --- | --- | --- | --- | --- |
| NA | NA | NA | | NA | NA | LHX6_1.m LHX6 | 0.048099 |
| NA | NA | NA | | NA | NA | LMX1A_1. LMX1A | 0.011598 |
| NA | NA | NA | | NA | NA | LMX1B_1. LMX1B | 0.130513 |
| 0.121211 0.111064 0.106483 0.108571 0.10718 LYL1_1.In LYL1 1.744865 | | | | | | | |
| 0.115273 | 0.10769 | | 0.108318 | 0.109164 | 0.103483 MAF_1.mo MAF | | 2.59284 |
| 0.108571 | 0.09753 | | 0.098498 | 0.107362 | 0.101806 MAFA_1.mMAFA | | 0.079265 |
| 0.121261 | 0.113264 | | 0.11359 | 0.112727 | 0.105825 MAFB_1.mMAFB | | 3.265332 |
| 0.113571 | NA | | 0.096631 | 0.103148 | 0.095344 MAFF_2.mMAFF | | 6.932717 |
| 0.115088 | 0.096732 | | 0.101536 | 0.107309 | 0.096777 MAFG_1.mMAFG | | 2.589326 |
| 0.114872 | 0.111912 | | 0.114045 | 0.111256 | 0.124558 MAX_2.moMAX | | 6.827517 |
| 0.122402 | 0.098948 | | 0.101455 | 0.117857 NA | | MBD2_1.mMBD2 | 6.630145 |
| 0.134454 | 0.114032 | | 0.112391 | 0.135054 NA | | MECP2_1. MECP2 | 2.081471 |

NA	0.098543 NA	NA	NA	MEOX1_1. MEOX1	0.244084

NA	NA	NA	NA	NA	MEOX2_1. MEOX2	0.334494

0.110266 0.108917 0.111476 0.110792 0.129398 MLXIPL_1 MLXIPL	0.200437

NA	NA	NA	NA	NA	MNX1_1.mMNX1	0.034428

0.11349 0.123817 0.109716 0.111304 0.109339 MSC_1.moMSC	1.076751

| NA | 0.101181 | NA | NA | NA MSX1_1.mMSX1 | 1.18465 |
| --- | --- | --- | --- | --- | --- |
| NA | 0.101181 | NA | NA | NA MSX1_1.mNKX1-2 | 0.055957 |
| 0.117286 | 0.115043 | 0.113454 | 0.118484 | 0.108708 MTF1_1.m MTF1 | 1.574401 |
| 0.118004 | 0.112417 | 0.114931 | 0.112346 | 0.128253 MXI1_1.m MXD1 | 8.725089 |
| 0.118004 | 0.112417 | 0.114931 | 0.112346 | 0.128253 MXI1_1.m MXD3 | 3.972446 |
| 0.118004 | 0.112417 | 0.114931 | 0.112346 | 0.128253 MXI1_1.m MXD4 | 3.215709 |
| 0.118004 | 0.112417 | 0.114931 | 0.112346 | 0.128253 MXI1_1.m MXI1 | 6.653613 |
| 0.116812 | 0.115276 | 0.116114 | 0.112426 | 0.10688 MYB_1.moMYB | 3.758304 |
| 0.116812 | 0.115276 | 0.116114 | 0.112426 | 0.10688 MYB_1.moRCOR2 | 1.071596 |
| 0.116812 | 0.115276 | 0.116114 | 0.112426 | 0.10688 MYB_1.moRCOR3 | 3.501184 |
| 0.116812 | 0.115276 | 0.116114 | 0.112426 | 0.10688 MYB_1.moSNAPC4 | 1.185122 |
| 0.10872 | 0.130987 | 0.118063 | 0.116512 | 0.111165 MYBL1_1. DNAJC2 | 2.665174 |
| 0.10872 | 0.130987 | 0.118063 | 0.116512 | 0.111165 MYBL1_1. MYBL1 | 2.180452 |
| 0.106312 | 0.123292 | 0.112874 | 0.110192 | 0.107365 MYBL1_2. DNAJC2 | 2.665174 |
| 0.106312 | 0.123292 | 0.112874 | 0.110192 | 0.107365 MYBL1_2. MYBL1 | 2.180452 |
| 0.124075 | 0.118211 | 0.124311 | 0.117495 | 0.135631 MYCN_1.mMYCL | 0.526898 |
| 0.117967 | 0.123336 | 0.108797 | 0.106626 | 0.111708 MYF5_1.m ASCL1 | 1.797811 |
| 0.117967 | 0.123336 | 0.108797 | 0.106626 | 0.111708 MYF5_1.m ASCL5 | 0.033189 |
| 0.114226 | 0.124924 | 0.11231 | 0.112826 | 0.10886 MYF6_1.m ASCL3 | 0.012304 |
| 0.114226 | 0.124924 | 0.11231 | 0.112826 | 0.10886 MYF6_1.m MYF6 | 1.676987 |

0.116129 0.119491 0.108375 0.105928 0.111315 MYOD1_1 MYOD1	0.087118

0.123461 0.123259 0.113591	0.11206 0.113555 MYOG_1. MYOG	0.367647

0.113254 0.115869	0.10519 0.103172 0.109978 NEUROD1 NEUROD1 0.066005

0.103407 0.105239 0.099488 NA	0.101823 NEUROD4 NEUROD4 0.068027

0.117677 0.110489 0.113604 0.106981 0.102963 NFE2_1.m NFE2	2.598176

0.100723 0.118122 0.113406 0.099393 0.102735 NFE2_2.m NFE2	2.598176

0.120877 0.106799 0.110662 0.107523 0.102845 NFE2L2_1 NFE2L2	7.469351

0.125729 0.147532 0.101345 0.162099 0.101447 NFIA_1.moNFIA	2.355015

0.127738 0.149508 0.101574 0.165069 0.102026 NFIB_1.m NFIB	1.698401

0.100558 0.104176 NA	0.114506 0.094057 NFIC_1.m NFIC	2.6715

0.126547 0.151832 0.101574 0.166838 0.101863 NFIX_1.moNFIX	3.995795

0.111559 0.124915 0.099821 0.133029	0.09855 NFIX_2.moNFIX	3.995795

NA	NA	NA	NA	0.10927 NFKB1_1. NFKB1	8.082149

0.103863 0.095957 0.104194 0.094634 0.109603 NFKB1_2. NFKB1	8.082149

NA	NA	NA	NA	0.107754 NFKB2_1. NFKB2	6.526764

NA	NA	NA	0.130762 NA	NKX2_1_1 NKX2-1	0.939153

NA	NA	NA	0.124661 NA	NKX2_2_1 NKX2-2	0.359503

NA	0.096605 0.096173 0.146871 0.096188 NKX2_3_1 NKX2-3	0.199471

NA	NA	NA	0.150842 NA	NKX2_4_2 NKX2-4	0.008688

NA	NA	NA	0.126638 NA	NKX2_5_1 NKX2-5	0.003991

NA	NA	NA	0.126889 NA	NKX2_5_3 NKX2-5	0.003991

NA	NA	NA	0.144731 NA	NKX2_6_1 NKX2-6	0.001654

NA	NA	NA	0.147256	0.09427 NKX2_8_1 NKX2-8	0.042752

NA	0.097141 0.101549	0.1275 0.096295 NKX3_1_2 NKX3-1	0.17122

0.099253 0.098648 0.106002 0.123716 0.098177 NKX3_2_1 NKX3-2	0.007983

NA	NA	NA	NA	NA	NKX6_2_1 NKX6-2	0.474414

NA	NA	NA	NA	NA	NKX6_3_1 NKX6-3	0.098861

NA	0.094754 NA	NA	NA	NOBOX_1. NOBOX	0.001604

0.118769	0.11908 0.122642 0.117609	0.13421 NPAS2_1. NPAS2	0.448158

0.100916 0.095961 0.106776 0.106036 0.106149 NR0B1_1. NR0B1	0.022118

0.100916 0.095961 0.106776 0.106036 0.106149 NR0B1_1. NR0B2	0.189404

NA	0.16717 NA	0.103965 NA	NR1H2_1. NR1H2	7.465455

0.095968 NA	NA	0.100392 0.099243 NR1H4_1. NR1H4	1.108643

NA	0.099989 NA	0.094942	0.09614 NR2C1_1. NR2C1	2.076182

NA	0.11017 NA	0.095483 0.097927 NR2C2_1. NR2C2	2.743007

0.141727	0.11184 0.125123 0.108352	0.10515 NR2C2_2. NR2C2	2.743007

0.092454 0.118993 NA	0.096396 0.097684 NR2F1_1. NR2F1	1.852534

NA	NA	NA	NA	0.093602 NR2F1_2. NR2F1	1.852534

0.093414 0.115261 NA	0.097319 0.098935 NR2F2_2. NR2F2	1.293198

NA	0.113189 NA	0.097232 0.097026 NR2F6_3. NR2F6	2.752082

NA	0.10678 NA	0.091207 NA	NR4A1_1. NR4A1	5.962652

NA	0.108892 NA	0.092321 0.091617 NR4A2_3. NR4A2	7.684468

NA	0.107337 NA	0.093844 0.094297 NR4A3_1. NR4A3	2.108374

0.09438 0.101352 NA	0.095982 0.094732 NR5A1_1. NR5A1	0.011618

0.114774 0.109815 0.110387 0.109122 0.102947 NRL_1.mo NRL	0.255957

NA	NA	NA	NA	NA	ONECUT1 ONECUT1 0.006722

NA	NA	NA	NA	NA	ONECUT1 SATB1	6.054599

NA	NA	NA	NA	NA	ONECUT1 SATB2	0.392513

NA	NA	NA	NA	NA	OTP_1.mo OTP	0.001086

0.114193 0.103209 0.104482 0.204597 0.120622 PAX1_1.m PAX1	0.057813

0.102385 0.095922 0.095686 0.153748 0.108198 PAX2_1.m PAX2	0.083629

0.114385 0.108032 0.106481 0.118435 0.106447 PAX2_3.m PAX2	0.083629

NA	NA	NA	NA	NA	PAX4_5.m PAX4	0.001872

0.103289 NA	NA	0.163428 0.106866 PAX5_2.m PAX5	4.414078

0.095681 0.101984 0.100183 0.154874 0.105684 PAX6_1.m PAX6	0.177948

0.099129 0.101907 0.097865	0.12825 0.103378 PAX8_1.m PAX8	3.387947

0.105868 NA	NA	0.137105 0.100462 PAX8_2.m PAX8	3.387947

NA	NA	NA	NA	NA	PDX1_2.m PDX1	0.087691

0.106985 NA	NA	NA	0.103297 PLAGL1_1 PLAGL1	0.832122

0.1085 NA	0.101648 0.097407 0.104809 PLAGL2_2 PLAGL2	1.804292

0.096152 0.106388 0.095313 0.096256 0.096788 PPARD_1. PPARD	4.818098

0.093185 0.115767 0.092746	0.09649 0.096724 PPARG_1. PPARG	2.696744

0.135019 0.102001 0.112149 0.126572 NA	PRDM15_ PRDM15	0.777298

| 0.109147 | 0.103229 | 0.106401 | 0.108859 | 0.105815 PTF1A_1. PTF1A | 0.024978 |
| --- | --- | --- | --- | --- | --- |

0.09461 0.098303 NA	0.094874 0.099649 RARA_1.m RARA	4.082062

| 0.106002 | 0.098077 | 0.108337 | 0.096156 | 0.111307 RELA_1.m RELA |
| --- | --- | --- | --- | --- |

5.487663

0.099003 NA	0.098228 0.097045 0.105022 RELB_1.m RELB	3.929958

0.116938 0.109926	0.10828 0.109551 0.106431 RFX1_1.m RFX1	0.856113

0.104734 0.099399 0.099702 0.105964 0.099828 RFX1_2.m RFX1	0.856113

0.11859 0.125622 0.110064 0.126949 0.109969 RFX2_3.m RFX2	0.442999

0.109822 0.107659 0.106782 0.106859 0.103422 RFX3_1.m RFX3	1.095085

0.117604 0.120157 0.115009 0.122078 0.107892 RFX4_2.m RFX4	0.90913

0.108092 0.102999 0.103031 0.104753 0.102128 RFX5_1.m RFX5	5.630639

0.106741 0.114919 0.110728 0.112083 0.103339 RFX5_2.m RFX5	5.630639

0.101531 0.108916 0.103711 0.107436 0.099645 RFX6_4.m RFX6	0.017003

NA	NA	NA	NA	NA	RHOXF1_1RHOXF1	0.096169

NA	NA	NA	NA	NA	RHOXF1_1RHOXF2	0.030711

NA	NA	NA	NA	NA	RHOXF1_1RHOXF2B 0.015795

| 0.128199 | 0.105923 | 0.155913 NA | | 0.117394 RUNX1_1. RUNX1 | | 1.60074 |
| --- | --- | --- | --- | --- | --- | --- |
| 0.134481 | 0.107825 | 0.163423 NA | | 0.118614 RUNX2_1. RUNX2 | | 2.605004 |
| 0.121212 | 0.101033 | 0.141807 NA | | 0.108998 RUNX3_1. RUNX3 | | 6.534423 |
| 0.093037 0.114663 NA 0.096762 0.097459 RXRA_3.m RXRA 2.37302 | | | | | | |
| NA | 0.101499 | NA | NA | 0.095302 RXRA_4.m RXRA | | 2.37302 |
| NA | 0.141922 | NA | 0.09822 | 0.095763 RXRB_1.m RXRB | | 3.36201 |
| NA | 0.134086 | NA | 0.097274 | 0.096666 RXRG_1.m RXRG | | 0.367997 |
| 0.09617 | 0.100393 | 0.099767 | 0.097495 | 0.099012 SIX1_1.mo SIX1 | | 0.236202 |
| 0.107125 | 0.09732 | 0.099214 | 0.104981 | 0.101024 SMAD1_1. SMAD9 | | 0.708703 |
| 0.106545 | 0.105181 | 0.102906 | 0.108336 | 0.102165 SMAD2_2. SMAD2 | | 3.471561 |
| 0.108104 | 0.107155 | 0.101418 | 0.109003 | 0.100876 SMAD3_2. SMAD3 | | 3.454065 |
| 0.108104 | 0.107155 | 0.101418 | 0.109003 | 0.100876 SMAD3_2. SMAD7 | | 2.705763 |
| 0.109258 | 0.108071 | 0.104688 | 0.106354 | 0.102662 SMAD4_1. SMAD6 | | 0.710363 |
| 0.10562 | 0.104211 | 0.109412 | 0.099466 | 0.102985 SMARCC1 SMARCC1 | | 6.702631 |
| 0.10562 | 0.104211 | 0.109412 | 0.099466 | 0.102985 SMARCC1 TADA2A | | 1.595376 |
| NA | 0.104853 | 0.097589 | 0.096819 NA | | SOX10_5. SOX10 | 0.799382 |
| NA | 0.09695 | 0.094843 | 0.097881 NA | | SOX3_1.m SOX3 | 0.150503 |
| NA | 0.10368 | 0.09432 | 0.095491 NA | | SOX6_1.m SOX6 | 0.397481 |
| NA 0.096544 0.099654 NA 0.10119 SP3_1.mo SP3 7.519624 | | | | | | |
| 0.158995 | 0.13976 | 0.158794 | 0.105467 | 0.11812 SPDEF_1. SPDEF | | 0.641713 |
| 0.190727 | 0.144697 | 0.181425 | 0.095431 | 0.144277 SPI1_1.mo SPI1 | | 12.10549 |
| 0.125437 | 0.107013 | 0.133769 | 0.089318 | 0.112363 SPI1_2.mo SPI1 | | 12.10549 |
| 0.150351 | 0.122382 | 0.147704 | 0.10219 | 0.118359 SPIB_1.moSPIB | | 3.262822 |
| 0.184116 | 0.141509 | 0.169998 | 0.094567 | 0.13566 SPIB_2.moSPIB | | 3.262822 |
| 0.16253 | 0.1312 | 0.158048 | 0.093656 | 0.125949 SPIC_1.moSPIC | | 0.017859 |
| 0.104425 | 0.107156 | 0.103181 | 0.09895 | 0.096942 STAT1_1. STAT1 | | 16.22525 |
| 0.105168 | 0.114453 | 0.132199 | 0.097906 | 0.112258 STAT2_1. STAT2 | | 7.309842 |
| 0.107462 | 0.112665 | 0.107561 | 0.104262 | 0.097688 STAT3_1. STAT3 | | 8.555032 |
| 0.101931 | 0.104872 | 0.100744 | 0.096667 | 0.104976 TAL1_1.m TAL1 | | 1.030632 |
| 0.117822 | 0.11565 | 0.111052 | 0.10987 | 0.112684 TCF12_1. TCF12 | | 7.928887 |
| 0.119927 | 0.133613 | 0.115611 | 0.111712 | 0.11004 TCF21_1. TCF21 | | 0.14149 |
| NA | 0.103292 | 0.096924 | NA | 0.109334 TCF3_1.m TCF3 | | 4.68618 |
| NA | 0.106719 | 0.107911 | 0.093096 | 0.090756 TCF7_1.m TCF7 | | 7.841648 |
| NA 0.096931 NA 0.114603 NA TEAD1_1. TEAD2 1.414405 | | | | | | |

NA	NA	NA	0.113258 NA	TEAD3_2. TEAD3	0.713064

0.109387 NA	NA	0.100966 0.098062 TFAP2A_1 TFAP2A	0.20299

0.115249	0.09799 0.099447 0.107743 0.105216 TFAP2A_2 TFAP2A	0.20299

0.115272 NA	NA	0.102551 0.102021 TFAP2A_3 TFAP2A	0.20299

NA	NA	NA	NA	NA	TFAP2D_1 TFAP2D	0.002057

0.114638 NA	0.097265 0.105721 0.102525 TFAP2E_1 TFAP2E	0.436942

0.121652 0.116674 0.110409 0.113483 0.109431 TFAP4_2. TFAP4	0.579232

0.113148	0.10878 0.111292 0.111737 0.123989 TFE3_1.m TFE3	4.236608

0.112496 0.105572	0.10969 0.109711 0.121284 TFEB_1.m TFEB	2.083665

0.109967 0.100918 0.104723 0.105239 0.114083 TFEC_1.m TFEC	1.918274

0.108516 0.115365 0.106448 0.098787 0.105274 TGIF2LX_ TGIF2LX	0.001095

0.111498	0.11241 NA	0.129061	0.09587 TLX1_2.m TLX1	0.061207

0.114657 0.106777 0.108539 0.110781 0.121098 USF1_1.m USF1	10.64016

0.110678 0.101157 0.102812 0.105493 0.117926 USF2_1.m USF2	8.614929

NA	NA	NA	NA	NA	VAX1_1.m VAX1	0.013566

NA	NA	NA	NA	NA	VAX2_1.m VAX2	0.240713

NA	NA	NA	NA	NA	VSX1_1.m VSX1	0.006399

0.133105 0.106461 0.115329 0.127097 0.093528 ZBTB1_1.I ZBTB1	9.00999

0.115448	0.10503 0.109502 0.117634 0.113522 ZBTB14_1 ZBTB14	2.724519

0.09772 0.102193 0.097379 NA	0.101366 ZBTB18_2 ZBTB18	7.287842

0.109546 NA	0.104476 0.115922 0.110853 ZBTB33_1 ZBTB33	4.750713

0.096553	0.10227 0.096726 NA	0.100905 ZBTB42_1 ZBTB42	0.675859

0.096389 NA	0.101821 NA	0.104881 ZBTB7A_2 ZBTB7A	2.860968

0.106505 NA	NA	0.10118 0.100564 ZFAT_2.m ZFAT	0.737812

NA	NA	NA	NA	NA	ZFHX2_1. ZFHX2	0.092875

0.127224 0.112981 0.112005 0.123235 0.108656 ZFP57_1.mZFP57	0.171536

0.099852 0.101514 0.096464 0.101258 0.099442 ZFP64_1.mZFP64	0.566698

0.098335 NA	NA	NA	0.094492 ZFX_1.mo ZFX	4.876437

0.097571 NA	NA	NA	0.098159 ZFY_1.mo ZFY	1.543006

0.108918 NA	0.100748 0.096963 0.104209 ZIC4_1.mo ZIC4	0.019551

0.101037 NA	0.102945 0.097468 0.100327 ZIC5_1.mo ZIC5	0.252151

0.109636 NA	0.103406 0.100699 0.102532 ZIC5_2.mo ZIC5	0.252151

0.113391 NA	0.098255 0.103989 0.100301 ZIC5_3.mo ZIC5	0.252151

0.096774 NA	0.098605 0.096378 0.100747 ZNF100_1 ZNF100	1.153388

0.116779 0.104672	0.10712	0.11724	0.1068 ZNF142_3 ZNF142	1.427406

0.10589 0.097286 0.110721 0.103427 0.110043 ZNF19_1. ZNF19	0.203274

0.12497 0.113608 0.116047 0.122984 0.109669 ZNF2_1.m ZNF2	0.586233

0.107991	0.10136	0.10569 0.101897 0.103455 ZNF208_1 ZNF208	0.249059

0.105318 0.104483 0.108128 0.107271 0.108647 ZNF208_2 ZNF208	0.249059

0.106945 NA	0.095564 0.106638 0.093452 ZNF292_1 ZNF292	6.657

0.108382 0.105677 0.111841 0.095139 0.101346 ZNF296_1 ZNF296	0.548868

0.122364 0.114558 0.123523 0.091076	0.1054 ZNF296_2 ZNF296	0.548868

0.110341 0.098712 0.103785 0.109291 0.104899 ZNF329_1 ZNF329	1.458582

0.11851 0.103919 0.108084	0.12216 0.095921 ZNF335_1 ZNF335	2.801287

0.117738 0.100979 0.109329 0.113821 0.103778 ZNF343_1 ZNF343	0.855262

0.111718	0.10233 0.108423	0.10728 0.104826 ZNF350_1 ZNF350	1.897095

0.115368 0.119649 0.113324 0.111889	0.11274 ZNF433_2 ZNF433	0.573033

0.105549 NA	0.103526 0.104433 0.103474 ZNF441_1 ZNF441	1.737943

0.135813 0.122017 0.122584 0.129656 0.104993 ZNF445_2 ZNF445	1.094173

NA	0.101714	0.09928 0.101553	0.09931 ZNF445_3 ZNF445	1.094173

0.117055 0.111672 0.109599 0.111918 0.104034 ZNF516_2 ZNF516	2.081164

0.11025 0.103572 0.098918 0.107055 0.099991 ZNF536_2 ZNF536	0.09247

0.11581 0.101888 0.102598	0.1103 0.105076 ZNF547_1 ZNF547	0.499899

0.107913 0.100798 0.108093	0.10348 0.100504 ZNF548_1 ZNF548	3.502894

0.107623 0.097121 0.102534 0.109721 0.100833 ZNF550_1 ZNF550	1.381006

NA	0.116665 NA	NA	NA	ZNF560_3 ZNF560	0.105033

0.111778 0.098448 0.107705 0.099988 0.101811 ZNF566_1 ZNF566	0.65478

0.098125 NA	0.099373 0.095703 0.100744 ZNF579_1 ZNF579	0.700497

0.103715 NA	0.103968 0.104666 0.107328 ZNF579_3 ZNF579	0.700497

0.127897 0.115494 0.113808 0.121038 0.107383 ZNF594_2 ZNF594	0.737013

0.099845 NA	0.095641 0.112213 0.099741 ZNF594_3 ZNF594	0.737013

0.116329 0.102302	0.10522 0.108956 0.102322 ZNF594_4 ZNF594	0.737013

NA	NA	0.09631 0.095916 0.098392 ZNF597_2 ZNF597	0.794163

0.105463 NA	0.104039 0.102715 0.102441 ZNF606_1 ZNF606	0.652742

0.103906 0.102007 0.112615 0.111869 0.108156 ZNF676_1 ZNF676	0.120049

0.096019 NA	0.106897 NA	0.096794 ZNF681_1 ZNF681	0.62747

0.109201 0.098198 0.097411 0.105568 0.102345 ZNF699_1 ZNF699	0.9745

0.10784 NA	0.101814 0.095404 0.095454 ZNF705D_ ZNF705D	0.007241

0.110644 0.101234	0.09971 0.108135 0.100585 ZNF716_1 ZNF716	0.063332

0.110224 0.104935 0.114242 0.100728	0.10521 ZNF721_2 ZNF721	3.328157

0.109458 0.106537	0.10321 0.105769 0.101484 ZNF732_1 ZNF732	0.446547

0.093759 0.094631	0.10024 0.095048 0.100358 ZNF746_1 ZNF746	1.574863

0.105824 0.105099 0.102493 0.100927 0.100169 ZNF763_2 ZNF763	1.289435

0.100309 0.101445 0.102889 0.105718 0.101144 ZNF768_1 ZNF768	2.664671

0.106231	0.09908 0.108933 0.096906 0.102956 ZNF770_2 ZNF770	5.337993

0.117469 0.101268 0.104826 0.116232 0.104057 ZNF777_2 ZNF777	1.496846

0.103409 NA	0.095956 0.094197 0.102459 ZNF781_1 ZNF781	0.312442

0.104315 0.107035 0.116208 0.108993 0.117303 ZNF80_2. ZNF80	0.187056

NA	NA	NA	0.100947 NA	ZNF827_1 ZNF827	1.009661

| 0.106517 0.100128 | 0.101532 | 0.113025 | 0.101422 ZNF836_2 ZNF836 | 1.112337 |
| --- | --- | --- | --- | --- |
| 0.111572 0.102986 | 0.109684 | 0.102633 | 0.103886 ZNF91_3. ZNF91 | 4.451705 |
| 0.100311 NA | 0.105999 | 0.10941 | 0.102417 ZSCAN25_ZSCAN25 | 2.014644 |

es_cell..ni Motif	geneid	TF family	TF group

1.28673 AHR_1.mo AHR	bHLH	BasicDomainsGroup

0 ALX3_1.m ALX3	Homeobox Helix-turn-helix

0 ARNT_1.m ARNT	bHLH	BasicDomainsGroup

0 ARNT_2.m ARNT	bHLH	BasicDomainsGroup

0.593921 ARNTL_1. ARNTL	bHLH	BasicDomainsGroup

0 ASCL2_1. ASCL2	bHLH	BasicDomainsGroup

1.97091 ATF3_1.m ATF3	TF_bZIP	BasicDomainsGroup

3.57604 ATF6_1.m ATF6	TF_bZIP	BasicDomainsGroup

0 ATF6_1.m CREBZF	TF_bZIP	BasicDomainsGroup

0 ATF7_1.m ATF7	TF_bZIP	BasicDomainsGroup

0.214117 ATOH1_1. ATOH1	bHLH	BasicDomainsGroup

0 ATOH7_1. ATOH7	bHLH	BasicDomainsGroup

4.17508 BACH1_1. BACH1	TF_bZIP	BasicDomainsGroup

0 BACH2_1. BACH2	TF_bZIP	BasicDomainsGroup

0 BARHL2_1BARHL2	Homeobox Helix-turn-helix

0 BARX1_2. BARX1	Homeobox Helix-turn-helix

0 BATF_1.m BATF	TF_bZIP	BasicDomainsGroup

0 BATF_1.m BATF2	TF_bZIP	BasicDomainsGroup

0 BCL11A_1 BCL11A	zf-C2H2	Zinc-CoordinatingGroup

0.787163 BHLHE40_BHLHE40 bHLH	BasicDomainsGroup

0 BSX_1.mo BSX	Homeobox Helix-turn-helix

5.20103 CBFB_1.m CBFB	CBF	Beta-ScaffoldFactors

0.186346 CDX1_1.m CDX1	Homeobox Helix-turn-helix

0 CDX2_1.m CDX2	Homeobox Helix-turn-helix

0 CEBPA_1. CEBPA	TF_bZIP	BasicDomainsGroup

4.51701 CEBPB_1. CEBPB	TF_bZIP	BasicDomainsGroup

0 CEBPD_1. CEBPD	TF_bZIP	BasicDomainsGroup

0 CEBPE_1. CEBPE	TF_bZIP	BasicDomainsGroup

0 CLOCK_1. CLOCK	bHLH	BasicDomainsGroup

0 CLOCK_1. SOHLH1	bHLH	BasicDomainsGroup

0 CREB3L2_ CREB3L2 TF_bZIP	BasicDomainsGroup

0.081619 CREB3L3_ CREB3L3 TF_bZIP	BasicDomainsGroup

0.289593 CREB5_1. CREB5	TF_bZIP	BasicDomainsGroup

0 DBP_1.mo DBP	TF_bZIP	BasicDomainsGroup

0.083267 DLX1_1.m DLX1	Homeobox Helix-turn-helix

0 DLX2_1.m DLX2	Homeobox Helix-turn-helix

0.018687 DLX6_1.m DLX6	Homeobox Helix-turn-helix

0 E2F1_1.m E2F1	E2F	Helix-turn-helix

| 0 EBF1_1.m EBF1 | COE | Helix-turn-helix |
| --- | --- | --- |
| 0 EBF1_1.m EBF2 | COE | Helix-turn-helix |
| 0.257358 EBF1_1.m EBF4 | COE | Helix-turn-helix |
| 0 EGR2_1.m EGR2 | zf-C2H2 | Zinc-CoordinatingGroup |
| 0 EGR4_1.m EGR4 | zf-C2H2 | Zinc-CoordinatingGroup |
| 0.006189 EHF_1.mo EHF | ETS | Helix-turn-helix |
| 0.006189 EHF_2.mo EHF | ETS | Helix-turn-helix |
| 0 ELF1_1.m ELF1 | ETS | Helix-turn-helix |
| 0 ELF2_1.m ELF2 | ETS | Helix-turn-helix |
| 0.775968 ELF3_1.m ELF3 | ETS | Helix-turn-helix |
| 0 ELF4_1.m ELF4 | ETS | Helix-turn-helix |
| 0 ELF5_1.m ELF5 | ETS | Helix-turn-helix |
| 0 ELK1_1.m ELK1 | ETS | Helix-turn-helix |
| 4.74678 ELK3_1.m ELK3 | ETS | Helix-turn-helix |
| 0 ELK4_1.m ELK4 | ETS | Helix-turn-helix |
| 0 EN1_2.mo EN1 Homeobox Helix-turn-helix | | |
| 0 ENO1_1.m ENO1 | NA | NA |
| 0 ERF_1.mo ERF | ETS | Helix-turn-helix |
| 0 ERG_1.mo ERG | ETS | Helix-turn-helix |
| 0 ERG_2.mo ERG | ETS | Helix-turn-helix |
| 0 ERG_3.mo ERG | ETS | Helix-turn-helix |
| 0 ESRRG_3. ESRRG | ESR-like | Zinc-CoordinatingGroup |

0 FERD3L_1 FERD3L	bHLH	BasicDomainsGroup

| 0 ESX1_1.m ESX1 | Hom | eobox Helix-turn-helix |
| --- | --- | --- |
| 0 ETS1_1.m ETS1 | ETS | Helix-turn-helix |
| 0 ETS1_2.m ETS1 | ETS | Helix-turn-helix |
| 6.34133 ETS2_1.m ETS2 | ETS | Helix-turn-helix |
| 0 ETV1_1.m ETV1 | ETS | Helix-turn-helix |
| 0.19877 ETV2_1.m ETV2 | ETS | Helix-turn-helix |
| 0 ETV3_1.m ETV3 | ETS | Helix-turn-helix |
| 0 ETV3_1.m ETV3L ETS Helix-turn-helix | | |
| 0 ETV4_1.m ETV4 | ETS | Helix-turn-helix |
| 0 ETV5_1.m ETV5 | ETS | Helix-turn-helix |
| 3.8455 ETV6_1.m ETV6 | ETS | Helix-turn-helix |
| 0 ETV7_1.m ETV7 | ETS | Helix-turn-helix |
| 0 EVX2_1.m EVX2 | Hom | eobox Helix-turn-helix |

| 0 FEV_1.mo FEV | ETS | Helix-turn-helix |
| --- | --- | --- |
| 0.043796 FLI1_1.mo FLI1 | ETS | Helix-turn-helix |
| 0.043796 FLI1_2.mo FLI1 | ETS | Helix-turn-helix |

| 2.04408 FOS_1.mo FOS | TF_bZIP | BasicDomainsGroup |
| --- | --- | --- |
| 0.175345 FOSB_1.m FOSB | TF_bZIP | BasicDomainsGroup |
| 0 FOSL1_1. FOSL1 | TF_bZIP | BasicDomainsGroup |
| 0.540295 FOSL2_1. FOSL2 | TF_bZIP | BasicDomainsGroup |

0 FOXA1_1. FOXA1	Fork_head Helix-turn-helix

0 FOXA2_1. FOXA2	Fork_head Helix-turn-helix

0.243567 FOXC1_1. FOXC1	Fork_head Helix-turn-helix

0 FOXD1_1. FOXD1	Fork_head Helix-turn-helix

0.018341 FOXD2_2. FOXD2	Fork_head Helix-turn-helix

0 FOXD4_1. FOXD4	Fork_head Helix-turn-helix

0.025399 FOXF1_1. FOXF1	Fork_head Helix-turn-helix

0.00165 FOXF1_1. FOXN1	Fork_head Helix-turn-helix

0 FOXF1_1. FOXN4	Fork_head Helix-turn-helix

0.017265 FOXF2_1. FOXF2	Fork_head Helix-turn-helix

0.078511 FOXG1_1. FOXG1	Fork_head Helix-turn-helix

0.990825 FOXK1_1. FOXK1	Fork_head Helix-turn-helix

1.90919 FOXO3_1. FOXO3	Fork_head Helix-turn-helix

0 FOXP1_1. FOXP1	Fork_head Helix-turn-helix

0.033925 FOXP2_1. FOXP2	Fork_head Helix-turn-helix

0 FOXP3_1. FOXP3	Fork_head Helix-turn-helix

0.094998 FOXQ1_1. FOXQ1	Fork_head Helix-turn-helix

3.92125 GABPA_1. GABPA	ETS	Helix-turn-helix

0.012908 GATA1_1. GATA1	zf-GATA	Zinc-CoordinatingGroup

0 GATA1_1. GATAD2B zf-GATA	Zinc-CoordinatingGroup

0 GATA2_1. GATA2	zf-GATA	Zinc-CoordinatingGroup

0 GATA2_1. ZGLP1	zf-GATA	Zinc-CoordinatingGroup

0.123397 GATA3_2. GATA3	zf-GATA	Zinc-CoordinatingGroup

0.185314 GATA4_1. GATA4	zf-GATA	Zinc-CoordinatingGroup

0 GATA5_1. GATA5	zf-GATA	Zinc-CoordinatingGroup

0.247141 GATA6_1. GATA6	zf-GATA	Zinc-CoordinatingGroup

0 GATA6_1. MTA2	zf-GATA	Zinc-CoordinatingGroup

0 GATA6_1. RERE	zf-GATA	Zinc-CoordinatingGroup

0 GBX1_1.m GBX1	Homeobox Helix-turn-helix

0 GBX2_1.m GBX2	Homeobox Helix-turn-helix

0.058401 GSX1_1.m GSX1	Homeobox Helix-turn-helix

0.009437 GSX2_1.m GSX2	Homeobox Helix-turn-helix

0 HAND1_1. HAND1	bHLH	BasicDomainsGroup

4.80524 HES1_1.m HES1	bHLH	BasicDomainsGroup

0 HES1_1.m HES6	bHLH	BasicDomainsGroup

| 0 HES4_1.m HES4 | bHLH | BasicDomainsGroup |
| --- | --- | --- |
| 0 HES7_1.m HES7 | bHLH | BasicDomainsGroup |
| 0 HEYL_1.m HEYL | bHLH | BasicDomainsGroup |
| 0.020724 HIC1_1.moHIC1 | ZBTB | Zinc-CoordinatingGroup |
| 0 HIVEP2_1. HIVEP2 | zf-C2H2 | Zinc-CoordinatingGroup |
| 0.046627 HLF_1.mo HLF | TF_bZIP | BasicDomainsGroup |

0.236573 HNF1A_1. HNF1A	Homeobox Helix-turn-helix

0 HNF1B_1. HNF1B	Homeobox Helix-turn-helix

0.029085 HNF4A_1. HNF4A	RXR-like	Zinc-CoordinatingGroup

0.029085 HNF4A_2. HNF4A	RXR-like	Zinc-CoordinatingGroup

0.181429 HNF4G_1. HNF4G	RXR-like	Zinc-CoordinatingGroup

0 HOXA1_1. HOXA1	Homeobox Helix-turn-helix

0 HOXA10_1HOXA10	Homeobox Helix-turn-helix

0 HOXA11_1HOXA11	Homeobox Helix-turn-helix

0 HOXA13_1HOXA13	Homeobox Helix-turn-helix

0 HOXA13_2HOXA13	Homeobox Helix-turn-helix

0 HOXA2_1. HOXA2	Homeobox Helix-turn-helix

0 HOXA5_2. HOXA5	Homeobox Helix-turn-helix

0 HOXA6_1. HOXA6	Homeobox Helix-turn-helix

0 HOXA6_10HOXA6	Homeobox Helix-turn-helix

0 HOXA6_4. HOXA6	Homeobox Helix-turn-helix

0 HOXA6_7. HOXA6	Homeobox Helix-turn-helix

0 HOXA6_8. HOXA6	Homeobox Helix-turn-helix

0 HOXA6_9. HOXA6	Homeobox Helix-turn-helix

0 HOXA9_1. HOXA9	Homeobox Helix-turn-helix

0 HOXA9_2. HOXA9	Homeobox Helix-turn-helix

0 HOXB13_1HOXB13	Homeobox Helix-turn-helix

0 HOXB13_2HOXB13	Homeobox Helix-turn-helix

0 HOXB2_1. HOXB2	Homeobox Helix-turn-helix

0 HOXB3_1. HOXB3	Homeobox Helix-turn-helix

0 HOXB4_1. HOXB4	Homeobox Helix-turn-helix

0 HOXB5_1. HOXB5	Homeobox Helix-turn-helix

0 HOXB7_1. HOXB7	Homeobox Helix-turn-helix

0 HOXB8_1. HOXB8	Homeobox Helix-turn-helix

0.053301 HOXC10_1HOXC10	Homeobox Helix-turn-helix

0.002246 HOXC11_1HOXC11	Homeobox Helix-turn-helix

0.002246 HOXC11_2HOXC11	Homeobox Helix-turn-helix

0.000757 HOXC12_2HOXC12	Homeobox Helix-turn-helix

0.008199 HOXC13_2HOXC13	Homeobox Helix-turn-helix

0.039055 HOXC5_1. HOXC5	Homeobox Helix-turn-helix

0.041879 HOXC9_1. HOXC9	Homeobox Helix-turn-helix

0.038921 HOXD1_1. HOXD1	Homeobox Helix-turn-helix

0.009748 HOXD11_ HOXD11	Homeobox Helix-turn-helix

0.000414 HOXD12_ HOXD12	Homeobox Helix-turn-helix

0.000414 HOXD12_ HOXD12	Homeobox Helix-turn-helix

0.015992 HOXD13_ HOXD13	Homeobox Helix-turn-helix

0.015992 HOXD13_ HOXD13	Homeobox Helix-turn-helix

0.073244 HOXD3_1. HOXD3	Homeobox Helix-turn-helix

0.0374 HOXD9_2. HOXD9	Homeobox Helix-turn-helix

0 IKZF3_1.mIKZF3	zf-C2H2	Zinc-CoordinatingGroup

0 IKZF5_1.mIKZF5	zf-C2H2	Zinc-CoordinatingGroup

0 IRF1_1.mo IRF1	IRF	Helix-turn-helix

0 IRF3_1.mo IRF3	IRF	Helix-turn-helix

0.311254 IRF4_2.mo IRF4	IRF	Helix-turn-helix

0.160841 IRF5_1.mo IRF5	IRF	Helix-turn-helix

0 IRF6_1.Inf IRF6	IRF	Helix-turn-helix

0 IRF7_1.mo IRF7	IRF	Helix-turn-helix

0.063062 IRF8_1.mo IRF8	IRF	Helix-turn-helix

1.10448 IRF9_1.mo IRF9	IRF	Helix-turn-helix

1.11926 JDP2_1.m JDP2	TF_bZIP	BasicDomainsGroup

0 JUN_1.mo JUN	TF_bZIP	BasicDomainsGroup

1.86719 JUNB_1.m JUNB	TF_bZIP	BasicDomainsGroup

0 JUND_1.m JUND	TF_bZIP	BasicDomainsGroup

0 KLF1_1.m KLF1	zf-C2H2	Zinc-CoordinatingGroup

0 KLF12_1.mKLF12	zf-C2H2	Zinc-CoordinatingGroup

0 KLF12_2.mKLF12	zf-C2H2	Zinc-CoordinatingGroup

2.23157 KLF13_1.mKLF11	zf-C2H2	Zinc-CoordinatingGroup

3.79191 KLF13_1.mKLF13	zf-C2H2	Zinc-CoordinatingGroup

0 KLF14_1.mKLF14	zf-C2H2	Zinc-CoordinatingGroup

0 KLF16_1.mKLF16	zf-C2H2	Zinc-CoordinatingGroup

0.00354 KLF4_1.m KLF17	zf-C2H2	Zinc-CoordinatingGroup

0.081014 KLF4_1.m KLF2	zf-C2H2	Zinc-CoordinatingGroup

0 KLF4_1.m KLF4	zf-C2H2	Zinc-CoordinatingGroup

0.564651 KLF5_1.m KLF5	zf-C2H2	Zinc-CoordinatingGroup

0 KLF6_1.m KLF6	zf-C2H2	Zinc-CoordinatingGroup

0 KLF7_1.m KLF7	zf-C2H2	Zinc-CoordinatingGroup

0 LBX1_1.m LBX1	Homeobox Helix-turn-helix

0 LEF1_1.m LEF1	HMG	OtherAlpha-HelixGroup

0.095584 LHX2_1.m LHX2	Homeobox Helix-turn-helix

0 LHX6_1.m LHX6	Homeobox Helix-turn-helix

0 LMX1A_1. LMX1A	Homeobox Helix-turn-helix

0.120805 LMX1B_1. LMX1B	Homeobox Helix-turn-helix

0 LYL1_1.In LYL1	bHLH	BasicDomainsGroup

0 MAF_1.mo MAF	TF_bZIP	BasicDomainsGroup

0 MAFA_1.mMAFA	TF_bZIP	BasicDomainsGroup

0 MAFB_1.mMAFB	TF_bZIP	BasicDomainsGroup

0.984885 MAFF_2.mMAFF	TF_bZIP	BasicDomainsGroup

0 MAFG_1.mMAFG	TF_bZIP	BasicDomainsGroup

0 MAX_2.moMAX	bHLH	BasicDomainsGroup

0 MBD2_1.mMBD2	MBD	UnclassifiedStructure

0 MECP2_1. MECP2	MBD	UnclassifiedStructure

0 MEIS2_1. MEIS2	Homeobox Helix-turn-helix

0 MEOX1_1. MEOX1	Homeobox Helix-turn-helix

0 MEOX2_1. MEOX2	Homeobox Helix-turn-helix

0 MLXIPL_1 MLXIPL	bHLH	BasicDomainsGroup

0 MNX1_1.mMNX1	Homeobox Helix-turn-helix

0 MSC_1.moMSC	bHLH	BasicDomainsGroup

0.520448 MSX1_1.mMSX1	Homeobox Helix-turn-helix

0 MSX1_1.mNKX1-2	Homeobox Helix-turn-helix

0 MTF1_1.m MTF1	zf-C2H2	Zinc-CoordinatingGroup

0.808387 MXI1_1.m MXD1	bHLH	BasicDomainsGroup

0 MXI1_1.m MXD3	bHLH	BasicDomainsGroup

0 MXI1_1.m MXD4	bHLH	BasicDomainsGroup

1.73143 MXI1_1.m MXI1	bHLH	BasicDomainsGroup

1.08115 MYB_1.moMYB	MYB	Helix-turn-helix

0 MYB_1.moRCOR2	MYB	Helix-turn-helix

2.33251 MYB_1.moRCOR3	MYB	Helix-turn-helix

0 MYB_1.moSNAPC4	MYB	Helix-turn-helix

0 MYBL1_1. DNAJC2	MYB	Helix-turn-helix

0 MYBL1_1. MYBL1	MYB	Helix-turn-helix

0 MYBL1_2. DNAJC2	MYB	Helix-turn-helix

0 MYBL1_2. MYBL1	MYB	Helix-turn-helix

0 MYCN_1.mMYCL	bHLH	BasicDomainsGroup

0.165604 MYF5_1.m ASCL1	bHLH	BasicDomainsGroup

0 MYF5_1.m ASCL5	bHLH	BasicDomainsGroup

0 MYF6_1.m ASCL3	bHLH	BasicDomainsGroup

0.054988 MYF6_1.m MYF6	bHLH	BasicDomainsGroup

0.006465 MYOD1_1 MYOD1	bHLH	BasicDomainsGroup

0 MYOG_1. MYOG	bHLH	BasicDomainsGroup

0 NEUROD1 NEUROD1 bHLH	BasicDomainsGroup

0.046889 NEUROD4 NEUROD4 bHLH	BasicDomainsGroup

0 NFE2_1.m NFE2	TF_bZIP	BasicDomainsGroup

0 NFE2_2.m NFE2	TF_bZIP	BasicDomainsGroup

0 NFE2L2_1 NFE2L2	TF_bZIP	BasicDomainsGroup

0.213143 NFIA_1.moNFIA	CTF/NFI	UnclassifiedStructure

0 NFIB_1.m NFIB	CTF/NFI	UnclassifiedStructure

0.539222 NFIC_1.m NFIC	CTF/NFI	UnclassifiedStructure

0.042693 NFIX_1.moNFIX	CTF/NFI	UnclassifiedStructure

0.042693 NFIX_2.moNFIX	CTF/NFI	UnclassifiedStructure

0.717566 NFKB1_1. NFKB1	RHD	Beta-ScaffoldFactors

0.717566 NFKB1_2. NFKB1	RHD	Beta-ScaffoldFactors

2.25456 NFKB2_1. NFKB2	RHD	Beta-ScaffoldFactors

0 NKX2_1_1 NKX2-1	Homeobox Helix-turn-helix

0 NKX2_2_1 NKX2-2	Homeobox Helix-turn-helix

0.001359 NKX2_3_1 NKX2-3	Homeobox Helix-turn-helix

0 NKX2_4_2 NKX2-4	Homeobox Helix-turn-helix

0 NKX2_5_1 NKX2-5	Homeobox Helix-turn-helix

0 NKX2_5_3 NKX2-5	Homeobox Helix-turn-helix

0 NKX2_6_1 NKX2-6	Homeobox Helix-turn-helix

0 NKX2_8_1 NKX2-8	Homeobox Helix-turn-helix

0 NKX3_1_2 NKX3-1	Homeobox Helix-turn-helix

0 NKX3_2_1 NKX3-2	Homeobox Helix-turn-helix

0 NKX6_2_1 NKX6-2	Homeobox Helix-turn-helix

0 NKX6_3_1 NKX6-3	Homeobox Helix-turn-helix

0 NOBOX_1. NOBOX	Homeobox Helix-turn-helix

0.097449 NPAS2_1. NPAS2	bHLH	BasicDomainsGroup

0 NR0B1_1. NR0B1	MiscellaneZinc-CoordinatingGroup

0 NR0B1_1. NR0B2	MiscellaneZinc-CoordinatingGroup

4.36856 NR1H2_1. NR1H2	THR-like	Zinc-CoordinatingGroup

0.00019 NR1H4_1. NR1H4	THR-like	Zinc-CoordinatingGroup

0 NR2C1_1. NR2C1	RXR-like	Zinc-CoordinatingGroup

2.5095 NR2C2_1. NR2C2	RXR-like	Zinc-CoordinatingGroup

2.5095 NR2C2_2. NR2C2	RXR-like	Zinc-CoordinatingGroup

1.71335 NR2F1_1. NR2F1	RXR-like	Zinc-CoordinatingGroup

1.71335 NR2F1_2. NR2F1	RXR-like	Zinc-CoordinatingGroup

0.366164 NR2F2_2. NR2F2	RXR-like	Zinc-CoordinatingGroup

0 NR2F6_3. NR2F6	RXR-like	Zinc-CoordinatingGroup

1.50198 NR4A1_1. NR4A1	NGFIB-lik Zinc-CoordinatingGroup

0 NR4A2_3. NR4A2	NGFIB-lik Zinc-CoordinatingGroup

0.037467 NR4A3_1. NR4A3	NGFIB-lik Zinc-CoordinatingGroup

0 NR5A1_1. NR5A1	SF-like	Zinc-CoordinatingGroup

0 NRL_1.mo NRL	TF_bZIP	BasicDomainsGroup

0 ONECUT1 ONECUT1 CUT	Helix-turn-helix

0 ONECUT1 SATB1	CUT	Helix-turn-helix

0 ONECUT1 SATB2	CUT	Helix-turn-helix

0 OTP_1.mo OTP	Homeobox Helix-turn-helix

0.000945 PAX1_1.m PAX1	PAX	Helix-turn-helix

0.049473 PAX2_1.m PAX2	PAX	Helix-turn-helix

0.049473 PAX2_3.m PAX2	PAX	Helix-turn-helix

0 PAX4_5.m PAX4	PAX	Helix-turn-helix

0 PAX5_2.m PAX5	PAX	Helix-turn-helix

0 PAX6_1.m PAX6	PAX	Helix-turn-helix

0 PAX8_1.m PAX8	PAX	Helix-turn-helix

0 PAX8_2.m PAX8	PAX	Helix-turn-helix

0.002187 PDX1_2.m PDX1	Homeobox Helix-turn-helix

0 PLAGL1_1 PLAGL1	zf-C2H2	Zinc-CoordinatingGroup

0 PLAGL2_2 PLAGL2	zf-C2H2	Zinc-CoordinatingGroup

3.96421 PPARD_1. PPARD	THR-like	Zinc-CoordinatingGroup

0.019715 PPARG_1. PPARG	THR-like	Zinc-CoordinatingGroup

0 PRDM15_ PRDM15	zf-C2H2	Zinc-CoordinatingGroup

0.000967 PTF1A_1. PTF1A	bHLH	BasicDomainsGroup

2.31156 RARA_1.m RARA	THR-like	Zinc-CoordinatingGroup

0 RELA_1.m RELA	RHD	Beta-ScaffoldFactors

1.62286 RELB_1.m RELB	RHD	Beta-ScaffoldFactors

0 RFX1_1.m RFX1	RFX	BasicDomainsGroup

0 RFX1_2.m RFX1	RFX	BasicDomainsGroup

0 RFX2_3.m RFX2	RFX	BasicDomainsGroup

0 RFX3_1.m RFX3	RFX	BasicDomainsGroup

0.323205 RFX4_2.m RFX4	RFX	BasicDomainsGroup

0 RFX5_1.m RFX5	RFX	BasicDomainsGroup

0 RFX5_2.m RFX5	RFX	BasicDomainsGroup

0.008809 RFX6_4.m RFX6	RFX	BasicDomainsGroup

0 RHOXF1_1RHOXF1	Homeobox Helix-turn-helix

0.00479 RHOXF1_1RHOXF2	Homeobox Helix-turn-helix

0 RHOXF1_1RHOXF2B Homeobox Helix-turn-helix

| 0 RUNX1_1. RUNX1 | Runt | Beta-ScaffoldFactors |
| --- | --- | --- |
| 0.113622 RUNX2_1. RUNX2 | Runt | Beta-ScaffoldFactors |
| 0 RUNX3_1. RUNX3 | Runt | Beta-ScaffoldFactors |
| 2.22573 RXRA_3.m RXRA | RXR-like | Zinc-CoordinatingGroup |
| 2.22573 RXRA_4.m RXRA | RXR-like | Zinc-CoordinatingGroup |
| 0 RXRB_1.m RXRB | RXR-like | Zinc-CoordinatingGroup |
| 0 RXRG_1.m RXRG | RXR-like | Zinc-CoordinatingGroup |

0 SIX1_1.mo SIX1	Homeobox Helix-turn-helix

0 SMAD1_1. SMAD9	MH1	UnclassifiedStructure

0 SMAD2_2. SMAD2	MH1	UnclassifiedStructure

1.63248 SMAD3_2. SMAD3	MH1	UnclassifiedStructure

0 SMAD3_2. SMAD7	MH1	UnclassifiedStructure

0.675159 SMAD4_1. SMAD6	MH1	UnclassifiedStructure

0 SMARCC1 SMARCC1 MYB	Helix-turn-helix

1.18837 SMARCC1 TADA2A	MYB	Helix-turn-helix

0 SOX10_5. SOX10	HMG	OtherAlpha-HelixGroup

0 SOX3_1.m SOX3	HMG	OtherAlpha-HelixGroup

0 SOX6_1.m SOX6	HMG	OtherAlpha-HelixGroup

0 SP3_1.mo SP3	zf-C2H2	Zinc-CoordinatingGroup

0 SPDEF_1. SPDEF	ETS	Helix-turn-helix

0 SPI1_1.mo SPI1	ETS	Helix-turn-helix

0 SPI1_2.mo SPI1	ETS	Helix-turn-helix

1.64038 SPIB_1.moSPIB	ETS	Helix-turn-helix

1.64038 SPIB_2.moSPIB	ETS	Helix-turn-helix

0.000414 SPIC_1.moSPIC	ETS	Helix-turn-helix

0 STAT1_1. STAT1	STAT	Beta-ScaffoldFactors

0 STAT2_1. STAT2	STAT	Beta-ScaffoldFactors

0 STAT3_1. STAT3	STAT	Beta-ScaffoldFactors

0 TAL1_1.m TAL1	bHLH	BasicDomainsGroup

7.50889 TCF12_1. TCF12	bHLH	BasicDomainsGroup

0.02867 TCF21_1. TCF21	bHLH	BasicDomainsGroup

0 TCF3_1.m TCF3	bHLH	BasicDomainsGroup

0.943527 TCF7_1.m TCF7	HMG	OtherAlpha-HelixGroup

0 TEAD1_1. TEAD2	TEA	Helix-turn-helix

0 TEAD3_2. TEAD3	TEA	Helix-turn-helix

0 TFAP2A_1 TFAP2A	AP-2	BasicDomainsGroup

0 TFAP2A_2 TFAP2A	AP-2	BasicDomainsGroup

0 TFAP2A_3 TFAP2A	AP-2	BasicDomainsGroup

0.000746 TFAP2D_1 TFAP2D	AP-2	BasicDomainsGroup

| 0.163721 TFAP2E_1 TFAP2E | AP-2 | BasicDomainsGroup |
| --- | --- | --- |
| 0 TFAP4_2. TFAP4 | bHLH | BasicDomainsGroup |
| 0 TFE3_1.m TFE3 | bHLH | BasicDomainsGroup |
| 0 TFEB_1.m TFEB | bHLH | BasicDomainsGroup |
| 0 TFEC_1.m TFEC | bHLH | BasicDomainsGroup |

0.000875 TGIF2LX_ TGIF2LX	Homeobox Helix-turn-helix

0.001273 TLX1_2.m TLX1	Homeobox Helix-turn-helix

0 USF1_1.m USF1	bHLH	BasicDomainsGroup

6.30736 USF2_1.m USF2	bHLH	BasicDomainsGroup

0 VAX1_1.m VAX1	Homeobox Helix-turn-helix

0.009852 VAX2_1.m VAX2	Homeobox Helix-turn-helix

0 VSX1_1.m VSX1	Homeobox Helix-turn-helix

4.04733 ZBTB1_1.I ZBTB1	ZBTB	Zinc-CoordinatingGroup

0 ZBTB14_1 ZBTB14	ZBTB	Zinc-CoordinatingGroup

3.62723 ZBTB18_2 ZBTB18	ZBTB	Zinc-CoordinatingGroup

4.6711 ZBTB33_1 ZBTB33	ZBTB	Zinc-CoordinatingGroup

0.605583 ZBTB42_1 ZBTB42	ZBTB	Zinc-CoordinatingGroup

0 ZBTB7A_2 ZBTB7A	ZBTB	Zinc-CoordinatingGroup

0 ZFAT_2.m ZFAT	zf-C2H2	Zinc-CoordinatingGroup

0 ZFHX2_1. ZFHX2	Homeobox Helix-turn-helix

| 0 ZFP57_1.mZFP57 | zf-C2H2 | Zinc-CoordinatingGroup |
| --- | --- | --- |
| 0 ZFP64_1.mZFP64 | zf-C2H2 | Zinc-CoordinatingGroup |
| 2.43414 ZFX_1.mo ZFX | zf-C2H2 | Zinc-CoordinatingGroup |
| 1.00493 ZFY_1.mo ZFY | zf-C2H2 | Zinc-CoordinatingGroup |
| 0 ZIC4_1.mo ZIC4 | zf-C2H2 | Zinc-CoordinatingGroup |
| 0 ZIC5_1.mo ZIC5 | zf-C2H2 | Zinc-CoordinatingGroup |
| 0 ZIC5_2.mo ZIC5 | zf-C2H2 | Zinc-CoordinatingGroup |
| 0 ZIC5_3.mo ZIC5 | zf-C2H2 | Zinc-CoordinatingGroup |
| 0 ZNF100_1 ZNF100 | zf-C2H2 | Zinc-CoordinatingGroup |
| 0 ZNF142_3 ZNF142 | zf-C2H2 | Zinc-CoordinatingGroup |
| 0 ZNF19_1. ZNF19 | zf-C2H2 | Zinc-CoordinatingGroup |
| 0.583185 ZNF2_1.m ZNF2 | zf-C2H2 | Zinc-CoordinatingGroup |
| 0 ZNF208_1 ZNF208 | zf-C2H2 | Zinc-CoordinatingGroup |
| 0 ZNF208_2 ZNF208 | zf-C2H2 | Zinc-CoordinatingGroup |
| 5.9619 ZNF292_1 ZNF292 | zf-C2H2 | Zinc-CoordinatingGroup |
| 0 ZNF296_1 ZNF296 | zf-C2H2 | Zinc-CoordinatingGroup |
| 0 ZNF296_2 ZNF296 | zf-C2H2 | Zinc-CoordinatingGroup |
| 0 ZNF329_1 ZNF329 | zf-C2H2 | Zinc-CoordinatingGroup |
| 0 ZNF335_1 ZNF335 | zf-C2H2 | Zinc-CoordinatingGroup |

0 ZNF343_1 ZNF343	zf-C2H2	Zinc-CoordinatingGroup

0 ZNF350_1 ZNF350	zf-C2H2	Zinc-CoordinatingGroup

0 ZNF433_2 ZNF433	zf-C2H2	Zinc-CoordinatingGroup

0.15551 ZNF441_1 ZNF441	zf-C2H2	Zinc-CoordinatingGroup

0 ZNF445_2 ZNF445	zf-C2H2	Zinc-CoordinatingGroup

0 ZNF445_3 ZNF445	zf-C2H2	Zinc-CoordinatingGroup

0 ZNF516_2 ZNF516	zf-C2H2	Zinc-CoordinatingGroup

0.026761 ZNF536_2 ZNF536	zf-C2H2	Zinc-CoordinatingGroup

0.387874 ZNF547_1 ZNF547	zf-C2H2	Zinc-CoordinatingGroup

1.41035 ZNF548_1 ZNF548	zf-C2H2	Zinc-CoordinatingGroup

0 ZNF550_1 ZNF550	zf-C2H2	Zinc-CoordinatingGroup

0 ZNF560_3 ZNF560	zf-C2H2	Zinc-CoordinatingGroup

0 ZNF566_1 ZNF566	zf-C2H2	Zinc-CoordinatingGroup

0 ZNF579_1 ZNF579	zf-C2H2	Zinc-CoordinatingGroup

0 ZNF579_3 ZNF579	zf-C2H2	Zinc-CoordinatingGroup

0 ZNF594_2 ZNF594	zf-C2H2	Zinc-CoordinatingGroup

0 ZNF594_3 ZNF594	zf-C2H2	Zinc-CoordinatingGroup

0 ZNF594_4 ZNF594	zf-C2H2	Zinc-CoordinatingGroup

0 ZNF597_2 ZNF597	zf-C2H2	Zinc-CoordinatingGroup

0 ZNF606_1 ZNF606	zf-C2H2	Zinc-CoordinatingGroup

0 ZNF676_1 ZNF676	zf-C2H2	Zinc-CoordinatingGroup

0 ZNF681_1 ZNF681	zf-C2H2	Zinc-CoordinatingGroup

0 ZNF699_1 ZNF699	zf-C2H2	Zinc-CoordinatingGroup

0.001601 ZNF705D_ ZNF705D zf-C2H2	Zinc-CoordinatingGroup

0.001454 ZNF716_1 ZNF716	zf-C2H2	Zinc-CoordinatingGroup

0 ZNF721_2 ZNF721	zf-C2H2	Zinc-CoordinatingGroup

0 ZNF732_1 ZNF732	zf-C2H2	Zinc-CoordinatingGroup

0 ZNF746_1 ZNF746	zf-C2H2	Zinc-CoordinatingGroup

0.66682 ZNF763_2 ZNF763	zf-C2H2	Zinc-CoordinatingGroup

0 ZNF768_1 ZNF768	zf-C2H2	Zinc-CoordinatingGroup

0 ZNF770_2 ZNF770	zf-C2H2	Zinc-CoordinatingGroup

0 ZNF777_2 ZNF777	zf-C2H2	Zinc-CoordinatingGroup

0 ZNF781_1 ZNF781	zf-C2H2	Zinc-CoordinatingGroup

0 ZNF80_2. ZNF80	zf-C2H2	Zinc-CoordinatingGroup

0 ZNF827_1 ZNF827	zf-C2H2	Zinc-CoordinatingGroup

0 ZNF836_2 ZNF836	zf-C2H2	Zinc-CoordinatingGroup

0 ZNF91_3. ZNF91	zf-C2H2	Zinc-CoordinatingGroup

1.75987 ZSCAN25_ZSCAN25 zf-C2H2	Zinc-CoordinatingGroup
